# How the Structure of Per- and Polyfluoroalkyl Substances (PFAS) Influences Their Binding Potency to the Peroxisome Proliferator-Activated and Thyroid Hormone Receptors—An In Silico Screening Study

**DOI:** 10.3390/molecules28020479

**Published:** 2023-01-04

**Authors:** Dominika Kowalska, Anita Sosnowska, Natalia Bulawska, Maciej Stępnik, Harrie Besselink, Peter Behnisch, Tomasz Puzyn

**Affiliations:** 1 QSAR Lab Ltd., Trzy Lipy 3, 80-172 Gdansk, Poland; 2 BioDetection Systems B.V., Science Park 406, 1098 XH Amsterdam, The Netherlands; 3 Faculty of Chemistry, University of Gdansk, Wita Stwosza 63, 80-308 Gdansk, Poland

**Keywords:** perfluoroalkyl compounds, PFAS, peroxisome proliferator-activated receptor, thyroid receptor, QSAR, MLR, in silico, binding probability, molecular docking, virtual screening

## Abstract

In this study, we investigated PFAS (per- and polyfluoroalkyl substances) binding potencies to nuclear hormone receptors (NHRs): peroxisome proliferator-activated receptors (PPARs) α, β, and γ and thyroid hormone receptors (TRs) α and β. We have simulated the docking scores of 43 perfluoroalkyl compounds and based on these data developed QSAR (Quantitative Structure-Activity Relationship) models for predicting the binding probability to five receptors. In the next step, we implemented the developed QSAR models for the screening approach of a large group of compounds (4464) from the NORMAN Database. The in silico analyses indicated that the probability of PFAS binding to the receptors depends on the chain length, the number of fluorine atoms, and the number of branches in the molecule. According to the findings, the considered PFAS group bind to the PPARα, β, and γ only with low or moderate probability, while in the case of TR α and β it is similar except that those chemicals with longer chains show a moderately high probability of binding.

## 1. Introduction

Per-and polyfluoroalkyl compounds (PFAS) are widely used in many consumer products and industrial applications, such as flame retardants [1], adhesives [2], varnishes [3], coatings [3], and food packaging materials [4]. PFAS are recognized by their hydrophobic carbon backbone, which is fully or partially saturated with fluorine atoms, and by possessing a hydrophilic functional group. PFAS, because of the C-F bonds, are very stable [2] and highly persistent in the environment, which can have a huge impact on humans and living organisms [5,6]. Continuous usage of PFAS leads to their accumulation in the environment and simultaneously increases the likelihood of harmful effects [2]. Many various PFAS have been detected so far in the air, water, soil, and dust [7,8,9]. Moreover, there are also studies indicating their presence in the human liver, blood, serum, and breast milk [10,11,12,13]. PFAS are known for causing different toxic effects including developmental, reproductive, carcinogenic, and immunological effects [14,15,16]. All these reports prompted scientists and regulatory bodies to take a closer look at the PFAS properties and behavior in the environment as well as the adverse effects they may pose. The results of the previous scientific research led to the introduction of the two most frequently used PFAS, i.e., PFOS and PFOA, into the Stockholm International Convention in 2009 and 2019, respectively [17,18]. All subsequent research and activities aimed at limiting/banning the use of PFAS compounds, but also at proposing new strategies to protect the environment and human health from PFAS and PM (persistent and mobile) chemicals. These new strategies for monitoring the PFAS are the subject of the three European Horizon 2020 founded projects (PROMISCES (101036449), SCENARIOS (101036756), ZeroPM (101037509)).

Recently, studies indicated that some perfluoroalkyl substances may have been endocrine disrupting chemicals (EDCs) by interfering with hormone action [19,20]. These substances may act as agonists or antagonists of nuclear hormone receptors (NHRs). One of the groups of NHRs with which PFAS compounds may interact is peroxisome proliferator-activated receptors (PPAR) alpha, beta, and gamma [6,21]. They are members of the nuclear receptor superfamily which plays a significant role in the regulation of lipid, glucose metabolism, and cellular growth [22]. Three PPAR isoforms (α, β/δ, and γ) have a similar sequence but they are encoded by distinct genes and are expressed in different tissues. Depending on the isoform, PPARs have specific roles during development and in adult life [23]. PPARα is mainly expressed in the liver, heart, intestine, and kidney [24]. PPARα mediates its functions by affecting fatty acid transport, esterification, and oxidation [23,25]. PPARβ has a proven role in reverse cholesterol transport, wound healing, and cell proliferation and apoptosis [23]. It has been shown to be ubiquitously expressed in both humans and rodents and participates in fatty acid oxidation, but also has a role in the regulation of blood glucose levels [23,25]. PPARγ is abundantly expressed in white adipose tissue, placenta, liver, heart, and skeletal muscles [26,27]. Several studies have shown activation of the PPAR isoforms by perfluoroalkyl acids in Cos-1 and 3T3-L1 cells using various binding and reporter constructs in cell-based assays [28,29].

The other, very important group of the NHR receptors that may be affected by EDCs are alpha and beta thyroid hormone receptors (TRs). Thyroid hormones are synthesized from tyrosine; hence, they are the amino acid derivatives [30]. The amino acids sequences of TRα and TRβ are identical in 85%, and both show high homology across species [31]. Thyroid hormones play an important role in numerous physiological processes, such as bone remodeling, metabolism regulation, cardiac and neurophysiological functions [32,33,34]. PFAS may compete with thyroid hormones for the binding to the transporting proteins such as transthyretin or albumin, which may eventually increase the level of bioavailable hormone [35,36]. In people exposed to PFAS, both a decrease and an increase in thyroid hormones have been observed, and both are harmful to human health [37].

Recently, the development of computational methods has made it possible to virtually screen chemical compounds for various purposes [38,39]. Application of *machine learning techniques* can aid in assessing the potential toxicity of chemicals significantly reducing the time and cost associated with conducting biological experiments. One of the methods used in this study is molecular docking-a computational modeling technique that enables the prediction of the interactions between two molecules such as drugs, enzymes, or proteins [40]. Molecular docking can be successfully applied for the study of potential ligand (PFAS)-protein binding [41]. Second computational method widely used in virtual screening is QSAR (Quantitative Structure-Activity Relationship) modeling. QSAR modeling is based on the fact that the molecular structure of chemicals contains information about biological properties; hence, it can be used to predict such properties of chemicals without a need to perform experiments [42,43]. According to the QSAR models, it is possible to interpolate the activity of a group of chemicals, provided that the molecular descriptors were calculated for a whole dataset, and there is only part of a group with unknown activity [44,45].

The main purpose of this study is to indicate if there is a relationship between the chemical structure of PFAS (e.g., carbon chain length) and their binding to PPARs and TRs. Combining molecular docking with the QSAR model allows for studying the endocrine disruption with sufficient predictive power by analyzing the influence of PFAS structural features on docking scores. We have determined docking scores for 43 commonly used PFAS using the open-source Endocrine Disruptome tool [41]. Next, we have developed the QSAR models for predicting the binding scores of PFAS to receptors (PPARα, PPARβ, PPARγ, TRα, and TRβ), and identified the structural features mainly affecting the binding probability. In the further step, we have implemented the developed models for predicting the binding scores for 4464 different PFAS and thus for the virtual screening of influences of the PFAS structure on the binding probability to the NHRs. Due to very little experimental data on the nuclear receptor affinity of PFAS, the use of in silico studies is essential to learn more about the subject.

## 2. Results and Discussion

**Figure 1:**
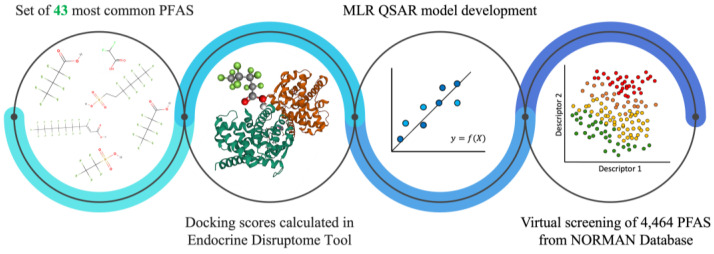
Workflow of research.

In the presented work we raised the important topic regarding the effects of the presence of PFAS in the environment and their impact on humans. We have applied an integrated computational approach, consisting of several steps allowing for virtual screening of PFAS in terms of their ability to bind to the NHRs. Firstly, using free-available software [41] we have calculated the binding scores of 43 PFAS to human nuclear hormone receptors (PPARα, PPARβ, PPARγ, TRα, and TRβ). Then using obtained values, we developed and validated five QSAR models in accordance with the standards and recommendations of the Organization for Economic Co-operation and Development (OECD) [46,47,48]. Finally, we have performed a virtual screening of the binding potential of 4464 PFAS from the NORMAN Database to NHRs. The stages of our work are shown in Figure 1.

### 2.1. Predicting PFAS Binding Probability to PPAR (α, β, and γ) and TR (α and β) with QSAR

Based on PFAS binding probability predicted by Endocrine Disruptome Tool we have developed five independent QSAR models (for PPARα, PPARβ, PPARγ, TRα, and TRβ). All developed models fulfill the OECD guidance recommendations dedicated to the principles for validation of the QSAR/QSPR models [46,47,48]. For the properly prepared QMRFs please refer to Supplementary Materials S2. Prepared QSAR models are scientifically valid and ready to be reproduced for the prediction of novel PFAS compounds [49]. In Table 1 we have summarized the equations and statistics obtained by developed models for the PFAS binding to the PPARs and TRs. All statistical parameters are described in Section 4.4 of this paper (Methodology: QSAR model calibration and validation).

#### 2.1.1. PPAR α, β, and γ

**Figure 2:**
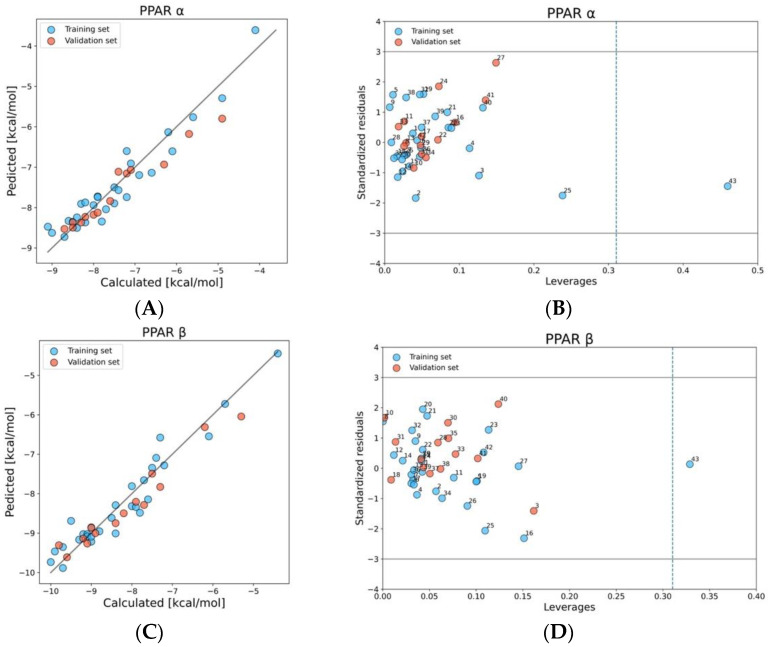

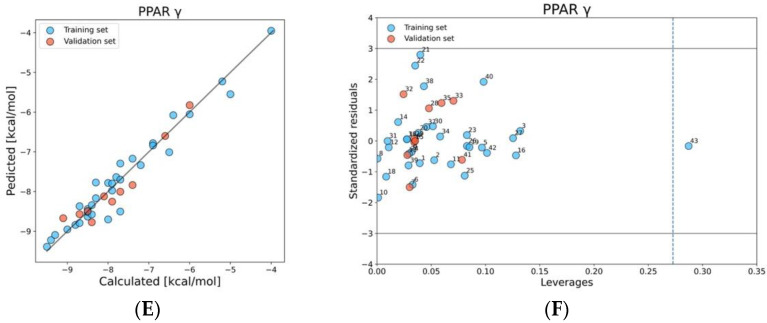
Plot of calculated versus predicted values of binding scores PPARα, β, and γ (**A**,**C**,**E**). Williams plot: dash line indicates the critical leverage value, solid lines represent ±3 standard deviation units (**B**,**D**,**F**).

The best QSAR model for predicting the PFAS-PPAR*α* binding affinity utilized two molecular descriptors: radial centric information index (ICR) and path/walk 2-Randic shape index (PW2), between which the correlation coefficient was small (r = 0.31). ICR descriptor gives information about centricity in the molecules [50], whereas the PW2 is defined as (P2/W2), the quotient of the path length of 2 (P2) and walk length of 2 (W2) [51]. The results indicated a high correlation between calculated docking scores (using Endocrine Disruptome Tool) [41] and predicted by the developed QSAR model values (Figure 2A). The values of R^2^, Q^2^_LOO_, and Q^2^_F1,F2,F3_ are close to 1, which confirms that the predictions were accurate, the model is stable, and that the model has good predictive abilities. The model is characterized by relatively low values of the root mean square errors (Table 1) of prediction in the training and validation sets (respectively RMSE_C_, RMSE_CV_, and RMSE_EXT_). More details about statistical results can be found in Supplementary Materials S1. To verify the reliability of predictions (they should be located within the optimum prediction space) we have checked the applicability domain of the developed model using the Williams plot (Figure 2B). This method allows to graphically present the standardized residuals (differences between observed and predicted values) versus the leverage value (indicates deviations of the structure of the compound from those used for the QSAR development). All in this work studied compounds were in the range of residuals differing by ±3 standard deviations from the mean value (h* = 0.310). One compound on the Williams plot, trifluoroacetic acid-TFA (43), has a higher leverage value than h*, but its activity has been predicted correctly [52]. It is worth mentioning that TFA is one of ten carboxylic acids used to build and validate the model; however, it is the shortest one, which may be the reason of being for outlier. To prove that the model is not a “correlation-by-chance”, Y^2^_SCR_ has been calculated (Table 1). Regarding the mechanistic interpretation of the model, PFAS with the lowest binding scores, 10:2 FTUCA (2) and 4:2 diPAPs (3) exceeding −8.9 kcal/mol, also have a high branching descriptor value (ICR) [53], which confirms that binding affinity is higher when it comes to long-chain perfluoroalkyl compounds (with more than eight carbon atoms in a molecule) [54].

We have estimated the binding probability of PFAS to PPARβ based on two molecular descriptors 2D (r = 0.26): radial centric information index (ICR) and the percentage of halogen atoms (X%). X% is a constitutional indices descriptor, which increases with the number of CF_2_ groups in a molecule. The main reason for choosing a model based on this structure properties was the high similarity between calculated and predicted values (Figure 2C) and very good statistics (Table 1). The Williams plot (Figure 2D) indicates that all compounds were located within the optimum prediction space of the model; however, one point-TFA (43) has a leverage value higher than h* = 0.310. However, attendance of this compound in the training set stabilizes the model. Nevertheless, the predictions for compounds with h > h* are treated as the results of extrapolation, so they are less reliable [55]. Scrambling test (Y^2^_SCR_) confirms that the presented model is statistically significant. Only five compounds (10:2FTUCA, 4:2diPAPs, PFDA, PFNS, and PFUdA) from the entire dataset show a moderate probability of binding to PPARβ (values are lower than −9.6). Compounds with the highest binding scores are characterized by X% and ICR on a high level (X% not lower than 40.91, and ICR higher than 2.51).

To estimate PFAS binding probability to PPARγ we have developed a QSAR model based on the same descriptors as for PPARβ (X% and ICR), with a correlation at r = 0.26. Figure 2E presents a significant correlation between the observed vs. predicted values of binding scores for PPAR*γ*. Model is characterized by satisfactory goodness-of-fit, robustness, and predictive capabilities (Table 1), which proves the accuracy of predictions. Values of errors (RMSE_C_, RMSEc_V_, RMSE_EXT_) are also acceptable (Table 1). A small Y^2^_SCR_ value confirms that the model is not a “correlation by chance”. Considering the applicability domain (Figure 2F), it can be seen that TFA (43) structurally diverges from the rest of the compounds (h > h* = 0.273) but is situated in the range of residuals differing by ±3 standard deviations from the mean value. The lowest binding score was observed for PFUdA (42), which had the highest value of percentage of halogen atoms (X%) and a relatively high value of ICR.

#### 2.1.2. TR α and β

**Figure 3:**
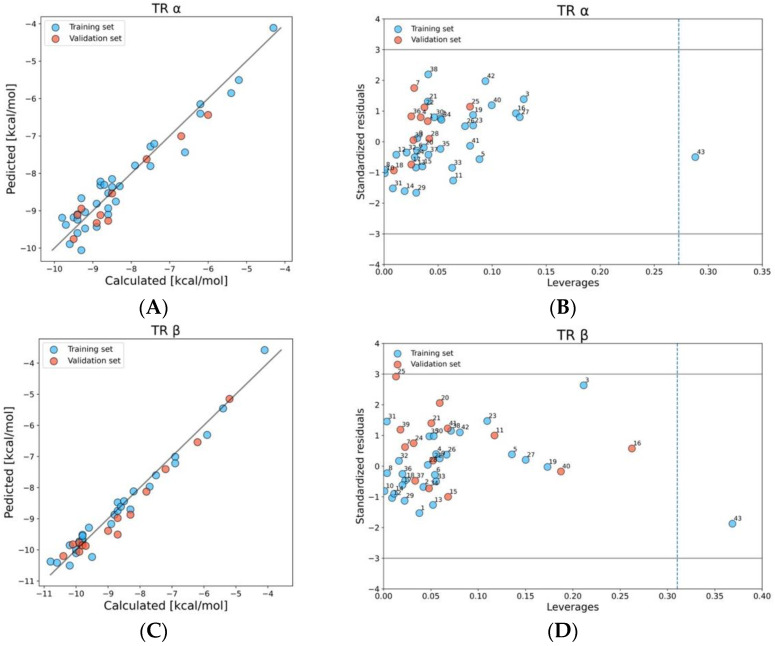
Plot of calculated versus predicted values of binding scores TRα, and β (**A**,**C**). Williams plot: dash line indicates the critical leverage value, solid lines represent ±3 standard deviation units (**B**,**D**).

We developed the QSAR model for predicting PFAS-TRα binding probability based on two molecular descriptors: percentage of halogen atoms in a molecule (X%) and radial centric information index (ICR). The correlation coefficient of the descriptors was low (r = 0.16). A good correlation between observed (calculated using Endocrine Disruptome Tool) [41] and predicted values can be seen on a scatter plot (Figure 3A). Additionally, the model is characterized by high accuracy of prediction and low values of the root mean square errors (Table 1). Compounds that were found between ±3 standard deviations from the mean value on Wiliams plot and do not exceed the value of h* = 0.273 are inside the structural space of the model (Figure 3B). In the case of TRα, only TFA (43) has been classified as an outlier due to the higher leverage value than h*. To prove that the model is not a “correlation-by-chance”, Y^2^_SCR_ has been calculated (Table 1). 8:2 FTUCA with a binding energy of −9.8 kcal/mol was identified as the compound with the highest probability of binding to the TRα ICR value of more than 2.5 and an X% of 53.33. Other compounds with a high probability of binding (binding score of less than −9.3 kcal/mol) had an X% value of no lower than 45 and an ICR of more than 2.24.

The best QSAR model for predicting PFAS binding probability to TRβ is based on two relatively low correlated (r = 0.47) molecular descriptors: the percentage of halogen atoms in a molecule (X%) and the total path count (TPC). TPC is the walk and path count descriptor, which describes the total number of paths, and reflects the size of the molecule as well as its complexity [56]. The Figure 3C confirms the good correlation between calculated and predicted data. We have selected the appropriate model based on robustness and ability of prediction confirmed by R^2^, Q^2^_LOO_, and Q^2^_F1,F2,F3_ values which are close to 0.95 (Table 1). The analysis of the applicability domain using the Williams plot (Figure 3D) shows that the same chemical as in the case of TRα-TFA (43) has a higher average hat value than h* = 0.273; however, it does not have standard residuals greater than 3 standard deviation units (±3σ). Thus, it can be stated that TFA (43) is slightly structurally different from the rest in the set, and has a positive effect on the extension of the model applicability domain. In the training set, two compounds, 10:2 FTOH (1) and 10:2 FTUCA (2), had the highest probability of binding to TRβ (binding score lower than −10.6 kcal/mol) with having one of the highest TPC values in the set (6.39), and an X% value exceeding 50.

### 2.2. Screening of the Binding Probability of 4464 PFAS

After developing the models, we implemented them into an external dataset from the NORMAN Database System [57] to predict the binding probability based on selected structural features of PFAS. We have estimated the endocrine action against five receptors for 4464 PFAS and have assessed if the predictions are in the applicability domain (please refer to Supplementary Materials S1). The number of compounds that fall within the applicability domain of developed models was verified using leverage value (it should be lower than h*). Compounds were also outliers when they did not fall within the range of values predicted by the model. Next, we analyzed the relationships between the particular receptor binding strength and the chemical structure of PFAS. Additionally, we have selected 59 PFAS which were from five different structural groups (carboxylic acids-group C, sulfonic acids-group S, phosphonic acids-group P, fluorotelomer alcohols-group F, and dicarboxylic acids-group Y) to illustrate how the structure of PFAS influences the value of the docking score between PFAS and receptors. Within the groups, each subsequent chemical differs from the previous one by an additional CF_2_ group. The increasing number of CF_2_ groups affects the change in the values of descriptors, while also influencing binding scores.

#### 2.2.1. PPAR α, β, and γ

**Figure 4:**
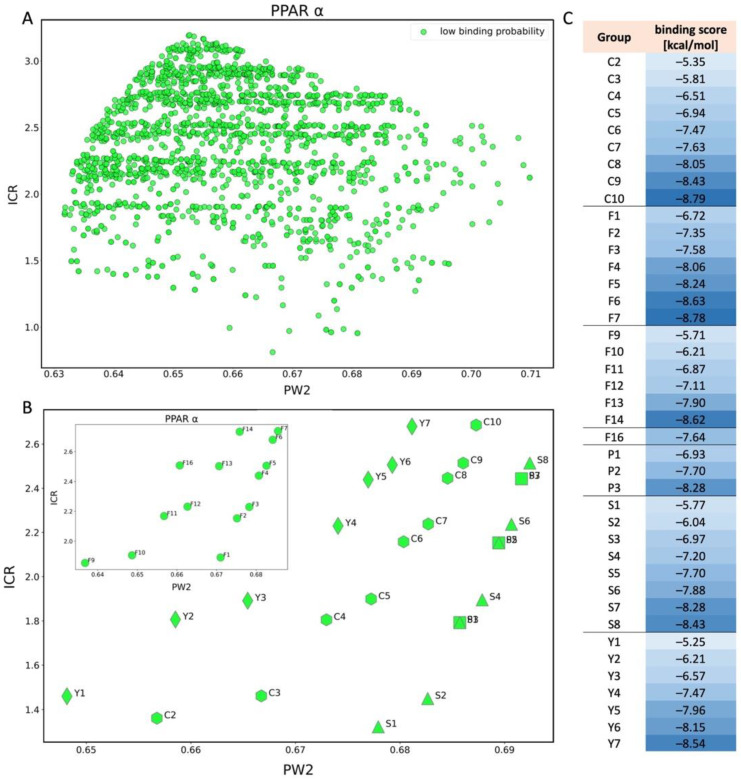
(**A**) Scatter plot of binding scores obtained for PPARα in PW2 and ICR space; (**B**) scatter plot for PPARα showing the division of PFAS into five different structural groups in space of PW2 and ICR. C-carboxylic acids (COOH-(CF2)n-CF3), S-sulfonic acids (SO3H-(CF2)n-CF3), P-phosphonic acids (PO3H2-(CF2)n-CF3, F-Fluorotelomer alcohol (F1-F7(OH-(CH2)-(CF2)n-CF3), F8-F14 (OH-(CH2)2-(CF2)n-CF3), F16 (OH-(CH2)3-(CF2)n-CF3), F17 (OH-(CH2)4-(CF2)n-CF3)), Y-dicarboxylic acids (COOH-(CF2)n-COOH). Colors: green-low binding probability; (**C**) Table showing binding score values for each compound shown in chart B.

**Figure 5:**
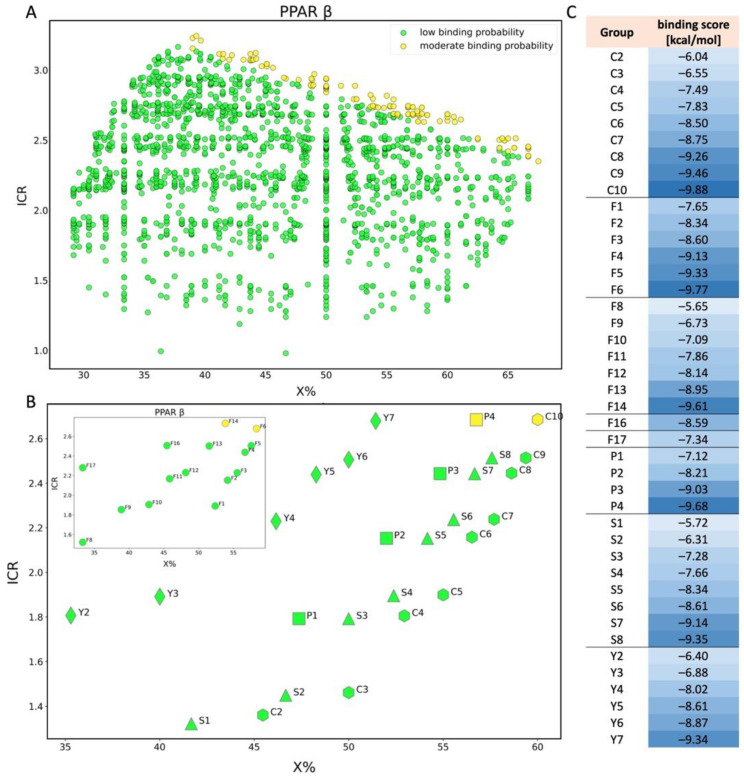
Scatter plot of binding scores obtained for PPARβ in X% and ICR space (**A**); Scatter plot for PPAR β showing the division of PFAS into 5 different structural groups in space of X% and ICR. C-carboxylic acids (COOH-(CF2)n-CF3), S-sulfonic acids (SO3H-(CF2)n-CF3), P-phosphonic acids (PO3H2-(CF2)n-CF3, F-Fluorotelomer alcohol (F1-F7(OH-(CH2)-(CF2)n-CF3), F8-F14 (OH-(CH2)2-(CF2)n-CF3), F16 (OH-(CH2)3-(CF2)n-CF3), F17 (OH-(CH2)4-(CF2)n-CF3)), Y-dicarboxylic acids (COOH-(CF2)n-COOH). Colors: green-low binding probability, yellow-moderate binding probability (**B**); table showing binding score values for each compound shown in chart B (**C**).

With all predictions for PFAS selected for the virtual screening, 1799, 2234, and 2631 compounds were outside the applicability domain of the developed models for PPARα, PPARβ, and PPARγ, respectively. Information about all compounds outside the structural AD is attached in Supplementary Materials S1. Based on docking scores obtained in models, we have assigned each compound to classes by color: “red” indicates a high probability of binding, “orange” indicates a moderately high binding probability, “yellow” corresponds to a moderate probability of binding, and “green” corresponds to a low probability of binding (more details in Supplementary Materials S1). To visualize the relationship between the chemical structure of the PFAS and their binding potency to PPAR receptors we have prepared the scatter plots, where the x and y axes represent the descriptors used in the QSAR models (Figure 4, Figure 5 and Figure 7).

Analyzing the results obtained for PPARα (Figure 4A), all predictions for PFAS falling into the applicability domain (2665 chemicals) created only one class of low binding probability (green) to PPARα (more details are in Supplementary Materials S1). The docking scores for compounds within AD estimated for this endpoint range from −8.78 to −4.49, and the h* = 0.310. For compounds with higher values of PW2 (close to 0.7) and ICR (close to 3), the docking scores decrease and have values of about −8.7. PW2 is a descriptor that gives information related to the shape of compounds and ICR informs about two-dimensional centricity in the molecules. The results obtained during the analysis suggest that all compounds similar to the training set of the QSAR model (based on PW2 and ICR) will have a low binding probability to PPARα. The above results indicate that PFAS are unlikely to bind to the PPARα receptor [22]. However, the compounds in the NORMAN database include many specific elements and groups (e.g., Ag, Ba, Cd, Ce, Cs, Si, I, Br, etc.), so it cannot be stated unequivocally that these compounds will not bind to PPARα.

In the case of the PPARα, we can state that increasing the length of the carbon chain increases the PW2 and ICR values (Figure 4A). Within all groups (C, S, P, F, and Y), a tendency can be seen for the probability of binding to increase with increasing descriptors values (Figure 4C). However, all compounds remain in the class with a low probability of binding to the receptor (green), because of the similarity of compounds to the training set (Figure 4B). The conclusion is consistent with the equation of the model, where the regression coefficient of PW2 and ICR is negative.

Several experimental studies can be found in the literature describing the effect of PFAS on PPARα, however, these studies vary in terms of the technique used to perform the tests, the type of cells tested, and the conditions under which the experiments were conducted. For example, Takacs and Abbott [23] who used transiently transfected Cos-1 cells (from African green monkey kidney) to evaluate the potential of PFOA and PFOS to activate human PPARα, found that PFOA was able to significantly increase the PPARα activity. The same authors reported also in vitro studies [24], which show that, e.g., perfluorodecanoic acid-PFDA and perfluorononanoic acid-PFNA bind more strongly to the PPARα than perfluorohexanoic acid-PFHxA, indicating that response depends on chain length and functional group. It is in line with our studies, where PFDA and PFNA have a lower value of docking scores (−8.5 and −8.0, respectively) than PFHxA (−7.1), which indicates that even if the PFDA and PFDA remain in a group with a low binding probability to PPARα, they bind strongly than PFHxA.

**Figure 6:**
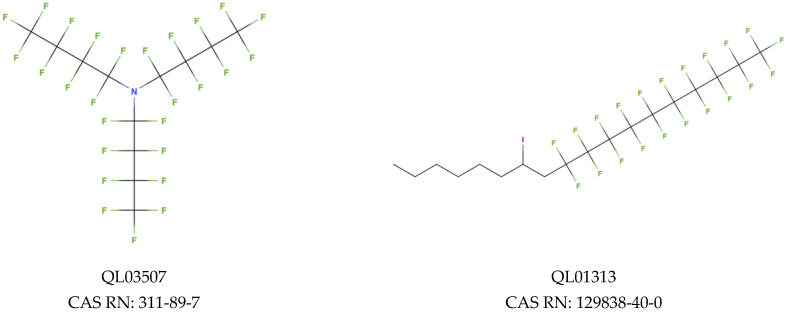

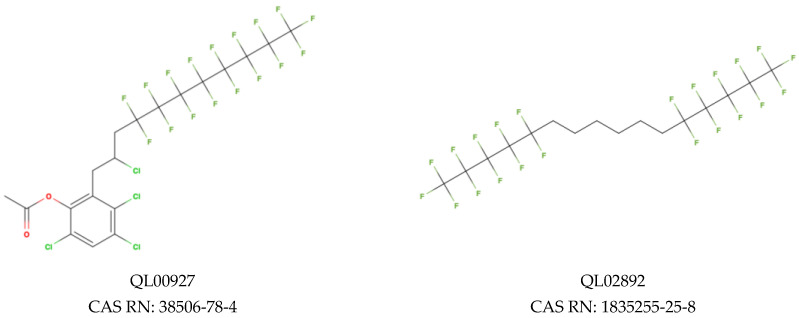
Chemical structure of PFAS selected from NORMAN Database.

Regarding the screening for PPARβ, we have classified the predictions into two groups: low binding probability (green-2108 PFAS) and moderate binding probability (yellow-122 PFAS). In Figure 5A we have presented the correlation between ICR (corresponds to a number of branching), X% (percentage of halogen atoms), and PFAS binding potency to PPARβ. The docking scores estimated for this endpoint range from −9.88 to −9.60 for the moderate binding probability class (yellow) and from −9.59 to −4.67 for the low binding probability class (green). An analysis of the descriptor values shows that compounds with more halogen atoms (high X% value), and more branches in the main chain have lower binding scores (they bind more strongly to the receptor) [58]. Worth considering is that QL01313 (Figure 6), despite the relatively low content of halogen atoms (X% = 40%), was in the group of compounds with a moderate probability of binding due to the high value of branching (ICR = 3.24). However, in the same group, there is also less branched compound (ICR = 2.35): QL03507 (Figure 6), which consists of 12 atoms of carbon and contains a lot of fluorine atoms in its structure (X% = 67.5%). Both mentioned PFAS were classified into a group with a moderate probability of binding (Figure 5A).

In the case of PPARβ, for all chemical groups (perfluorinated carboxylic acids, fluorotelomer alcohols, phosphonic acids, and dicarboxylic acids), an increase in binding probability is observed as the molecule is extended by another -CF_2_ group (Figure 5C). Perfluoroalkyl carboxylic acid (C10) and phosphonic acid (P4) with X% close to 60% and ICR higher than 2.6 belong to the class of compounds with a moderate probability of binding (yellow). It can be seen in Figure 5B that long-chain PFAS bind stronger to PPARβ than to PPARα, because the same compounds (C10, P4, F6, and F14) that belong to the group with moderate binding probability to PPARβ (yellow) have a lower binding probability to PPARα (green). Two perfluorotelomer alcohols (F14 and F6) differ by one -CH_3_ moiety and are classified as PFAS with a moderate binding probability to PPARβ because both consist of 10 carbon atoms fully saturated with fluorine. Furthermore, it can be concluded that all considered perfluoroalkyl dicarboxylic acids (Y, which contain less than 10 carbon atoms saturated with fluorine) are classified as PFAS with low binding probability to PPARβ. None of the analyzed perfluoroalkyl sulfonic acids shows a significant probability of binding.

The impact of PFAS on the beta isoform of PPAR has also already been considered in the literature. Takacs and Abbott [23] in their study on transiently transfected Cos-1 cells indicated that neither PFOA nor PFOS affected PPARβ. Moreover, Heuvel et al. [29] in 3T3-L1 mouse fibroblasts assay found that both PFOA and PFOS could not activate human PPARβ. Contradictory conclusions were reached by Li et al. [58] who investigated that the binding potency and transcriptional activity of PFOA, PFOS, and other PFAS toward human PPARβ (PDB: 3OZ0) increase with increasing carbon chain length. The authors found that differences in the cell type and reporter system may lead to different conclusions. Results obtained in the mentioned studies are in line with our results, where 10:2FTUCA, 4:2diPAPs, PFDA, PFNS, and PFUdA belong to the group with moderate binding probability, and the other PFAS with shorter chains have a low binding probability of binding to PPARβ.

**Figure 7:**
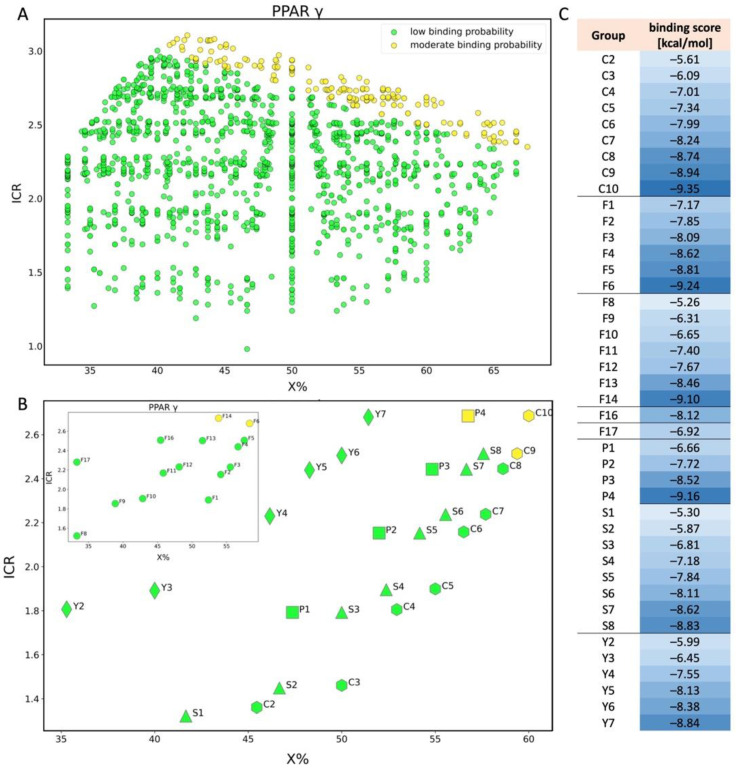
Scatter plot of binding scores obtained for PPARγ in X% and ICR space (**A**); scatter plot for PPAR γ showing the division of PFAS into 5 different structural groups in space of X% and ICR. C-carboxylic acids (COOH-(CF2)n-CF3), S-sulfonic acids (SO3H-(CF2)n-CF3), P-phosphonic acids (PO3H2-(CF2)n-CF3, F-Fluorotelomer alcohol (F1-F7 (OH-(CH2)-(CF2)n-CF3), F8–F14 (OH-(CH2)2-(CF2)n-CF3), F16 (OH-(CH2)3-(CF2)n-CF3), F17 (OH-(CH2)4-(CF2)n-CF3)), Y-dicarboxylic acids (COOH-(CF2)n-COOH). Colors: green-low binding probability, yellow-moderate binding probability (**B**); table showing binding score values for each compound shown in chart B (**C**).

Results obtained for the PPARγ receptor indicate that the predictions of binding are classified into two groups: low binding probability (green-1633 PFAS) and moderate binding probability (yellow-200 PFAS). Figure 7A presents the scatter plot in the space of ICR (radial centric information index) and X% (percentage of halogen atoms). As in previous cases, assignment to groups is based on molecular descriptors values (X% and ICR), and when they are large it indicates a long-chain molecule, which can determine a higher probability of binding to the receptor [24]. More compounds from NORMAN Database show moderate binding probability (are in the yellow group) compared to PPARβ. For example, QL00927 and QL02892 (Figure 6) belong to the group of compounds with moderate binding probability to PPARγ, while there is a low probability of binding to PPARβ for the same PFAS (for more details please refer to Supplementary Materials S1). It is due to the differences between the threshold values set in the tool based on statistical analysis for PPARβ and PPARγ (Table 1).

In the case of the PPARγ, we can state that increasing length of the carbon chain increases the PW2 and ICR values (Figure 7B). Considering the different functional groups of PFAS (C, S, P, Y, F), the tendency can be seen that as the value of descriptors increases, the probability of binding to PPARγ increases (Figure 7C). Both perfluoroalkyl carboxylic acid (C10) and phosphonic acid (P4) belong to the class of compounds with a moderate probability of binding (yellow) to PPARβ. Interestingly, the C9 compound with the lower ICR value (2.5) shows a moderate binding probability, indicating that it is more likely to bind to the gamma isoform rather than to beta. Two perfluorotelomer alcohols (F14 and F6) are classified as PFAS with a moderate binding probability to PPARγ (F14 has one -CH_3_ group more, but both consist of ten carbon atoms fully saturated with fluorine). Despite dicarboxylic acid Y2 containing three carbon atoms fully saturated with fluorine and Y7 containing nine, all perfluoroalkyl dicarboxylic acids (Y2-Y7) are classified as PFAS with low binding probability to PPARγ, for which their binding score decreases when number of C-F bonds in the main chain increases (Figure 7C). None of the analyzed perfluoroalkyl sulfonic acids shows a significant probability of binding to PPARγ isoform.

Researchers have also paid attention to the dysfunction of PPARγ after binding to PFAS. Zhang et al. [59] reported that 11 perfluorinated carboxylic acids and three perfluorinated sulfonic acids were able to activate human PPARγ, which activity increased with increasing carbon chain length of PFAS. However, results obtained by Behr et al. [22] indicate that PFBA, PFHxA, PFBS, PFOA, and PFOS do not affect PPARγ. Our study confirms the effect of chemical chain length on the probability of binding to the receptor; however, our analysis also shows that the compounds studied by Behr et al. have a low probability of binding to the PPARγ (green). The differences probably result from using a different cell line and the transfection procedure in the case of Zhang’s studies. Zhang et al. also specified that molecular docking has been performed on the specific 3D structure of PPARγ (PDB entry:3U9Q), while Behr et al. did not include that information. In the case of our study, PDB entry:3ET3 has been taken into consideration, which also affects the differences in results.

#### 2.2.2. TR α and β

**Figure 8:**
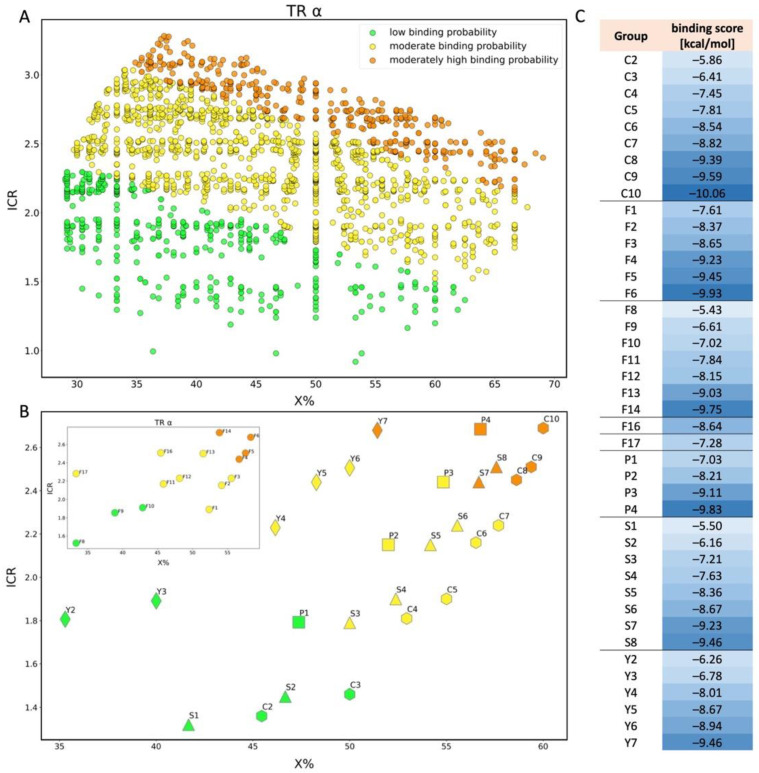
Scatter plot of binding scores obtained for TRα in X% and ICR space (**A**); scatter plot for TRα showing the division of PFAS into 5 different structural groups in space of ICR and X%. C-carboxylic acids (COOH-(CF2)n-CF3), S-sulfonic acids (SO3H-(CF2)n-CF3), P-phosphonic acids (PO3H2-(CF2)n-CF3, F-Fluorotelomer alcohol (F1-F7(OH-(CH2)-(CF2)n-CF3), F8-F14 (OH-(CH2)2-(CF2)n-CF3), F16 (OH-(CH2)3-(CF2)n-CF3), F17 (OH-(CH2)4-(CF2)n-CF3)), Y-dicarboxylic acids (COOH-(CF2)n-COOH). Colors: green-low binding probability, yellow-moderate binding probability, orange-moderately high binding probability (**B**); table showing binding score values for each compound shown in chart B (**C**).

**Figure 9:**
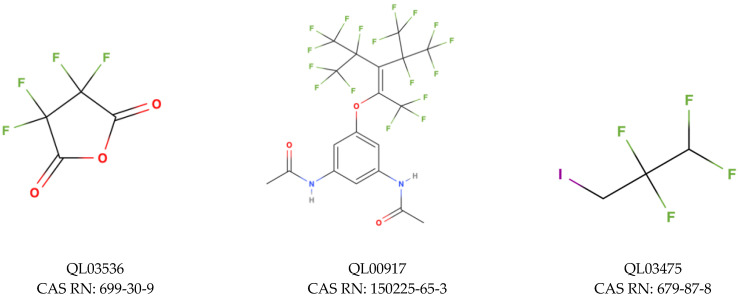

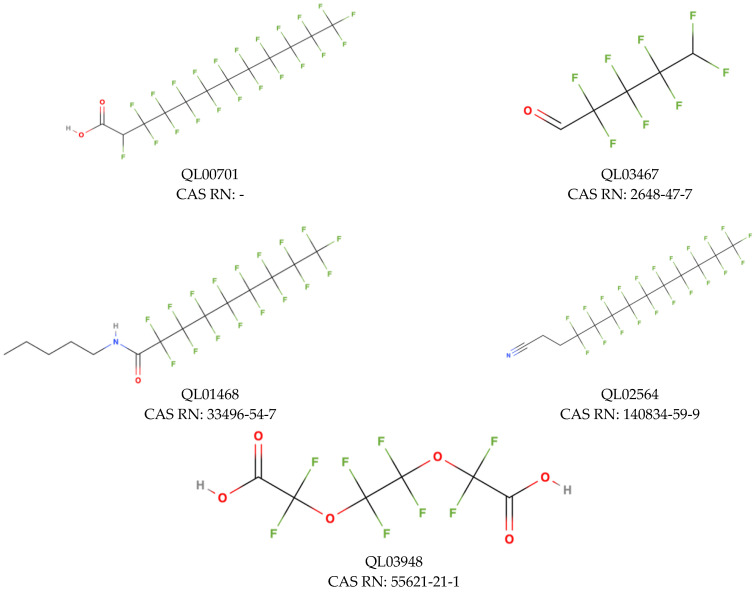
Chemical structure of PFAS selected from NORMAN Database.

For TRα, the minimum and maximum values of the estimated endpoint were −10.06 and −4.36 kcal/mol respectively, and the hat value obtained was h* = 0.273. Compounds that were found between the minimum and maximum value of predicted binding energy for the training set and do not exceed the value of h* are classified as a compound in the structural space of the model. We have classified 2104 compounds as outliers (52.9% coverage). Information about all compounds outside the structural AD is attached in Supplementary Materials S1. Compounds falling into the applicability domain of the TRα model (Figure 8A) represent three classes of binding: low binding probability (504 chemicals), moderate binding probability (1400 chemicals), and moderately high binding probability (456 chemicals). Based on standardized coefficients used in the QSAR model we can indicate that ICR is a descriptor that has a greater influence on the outcome of the estimated value of binding probability to the receptor than X%. In the first class (green–low binding probability) the range of values for the X% is from 29.03 to 62.5, and for the ICR it is from 0.92 to 2.29. Compound QL03536 (Figure 9), with the highest value of energy (−4.36 kcal/mol), has a low value of ICR (0.99) and X% value that equals 36.36, and is a cyclic structure with four halogen atoms in the structure. On the other hand, the lowest value of energy (−7.19 kcal/mol) has a molecule QL00917 (Figure 9) with a high ICR value (2.27) resulting from a large number of branching, and the X% equals only 32.69. In the second class (yellow–moderate binding probability) the range of X% values are from 29.73 to 67.74, and of ICR is from 1.52 to 3.04. Compound QL03467 (Figure 9) is the carboxylic acid with almost all carbon atoms fully substituted by fluorine, and it has the highest value of energy (−7.20 kcal/mol), one of the lowest values of ICR (1.79), and X% equals 50.00. The lowest value of energy (−9.19 kcal/mol) has molecule QL01468, with a high value of ICR due to the main chain of 15 atoms and X% equals 37.78. In the class with moderately high binding probability, the compound with the lowest energy value (−10.06 kcal/mol) is QL00701 (Figure 9). It has a relatively high value of X% (57.89) because almost all atoms are completely substituted with fluorine and the value of ICR is equal to 2.75 which is close to the mean value of this descriptor in the group.

Analyzing PFAS from different groups (Figure 8B) we can observe that carboxylic acid with five carbon atoms (C4) has a moderate binding probability, whereas acids composed of nine or more carbon atoms (C8-C10) are classified as a moderately high binding probability. In the case of the Y group, dicarboxylic acid with six carbon atoms in structure (Y3) is classified as a compound with a low probability of binding. Y4, which is first in the moderate binding probability class, has eight carbon atoms in the structure, while acid with moderately high binding probability (Y7) has 11 carbon atoms in the structure. All presented above examples confirm the higher probability of binding to a receptor in the case of long PFAS than for shorter compounds. Based on Figure 8C, it can be observed that extending the inner chain (+CF_2_) of groups causes a decrease in the value of the binding score, although the compounds are sometimes assigned to the same class.

Many studies have focused on the detrimental effects of PFAS on the thyroid system [60], but few have looked at the docking of these compounds to thyroid α and β receptors. Our study consisted of examining the probability of PFAS binding to thyroid α and β sites, respectively: 3ILZ (structure of TRα bound to selective thyromimetic GC-1 in P212121 space group [61] and 3IMY (structure of TRβ bound to selective thyromimetic GC-1 [62]. The binding of a ligand (GC-1) to sites selected for PFAS docking induces positive effects, such as lowering serum LDL cholesterol and triglycerides, so PFAS binding may interfere with this process [63].

The in vitro studies reported in the literature prove that short-chain PFAS such as perfluoropentanoic acid–PFPeA, perfluorobutanoic acid–PFBA, and perfluorobutanesulfonic acid-PFBS have a lesser effect on TR dysfunction (reduced cell viability of rat-thyroid cells [64]) compared to long-chain (more than eight carbon atoms) PFAS. In our set, the energy of binding for those three studied short-chain PFAS does not exceed −7.4 kcal/mol, which confirms the above statement. Another in vitro study documented the accumulation of PFOA and PFOS in thyroid cells [65], which, according to our study, have low binding energy values (−8.9 and −9.4 kcal/mol, respectively), meaning they are more likely to bind to the receptor. These long-chain compounds were considered less safe for human health and the environment.

**Figure 10:**
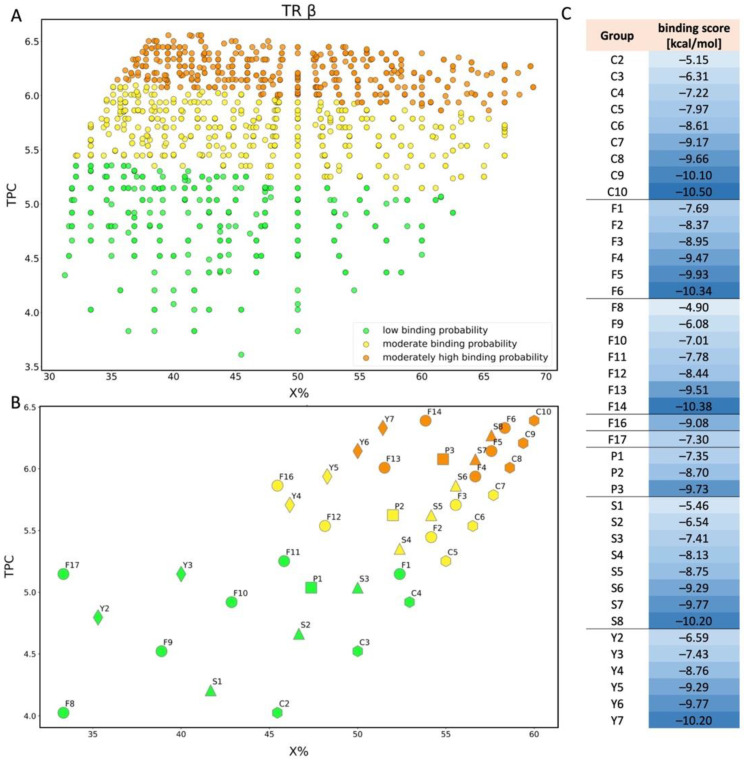
Scatter plot of binding scores obtained for TRβ in X% and TPC space (**A**); scatter plot for TRβ showing the division of PFAS into 5 different structural groups in space of TPC and X%. C-carboxylic acids (COOH-(CF2)n-CF3), S-sulfonic acids (SO3H-(CF2)n-CF3), P-phosphonic acids (PO3H2-(CF2)n-CF3, F–Fluorotelomer alcohol (F1–F7 (OH-(CH2)-(CF2)n-CF3), F8-F14 (OH-(CH2)2-(CF2)n-CF3), F16 (OH-(CH2)3-(CF2)n-CF3), F17 (OH-(CH2)4-(CF2)n-CF3)), Y-dicarboxylic acids (COOH-(CF2)n-COOH). Colors: green-low binding probability, yellow-moderate binding probability, orange-moderately high binding probability (**B**); table showing binding score values for each compound shown in chart B (**C**).

Analyzing the screening approach for the TRβ, the minimum and maximum values of the estimated endpoint were −10.5 and −4.27 kcal/mol respectively, and the hat value obtained was h* = 0.310. The 2694 compounds were classified as outliers (39.7% coverage). Considering the results obtained for TRβ, similarly to TRα, we have classified compounds into three classes (Figure 10A): low binding probability (477 chemicals), moderate binding probability (731 chemicals), and moderately high binding probability (562 chemicals). Here also the descriptor based on the size of the molecule (TPC) has a greater influence on the binding energy value, regarding the QSAR model. The low binding probability class consists of compounds with a range from 31.25 to 62.50 for X% and a range from 3.61 to 5.36 for TPC. A molecule QL03475 (Figure 9), with the highest value of energy (−4.27 kcal/mol) has a TPC equal to 3.61, and a quite high value of X% (45.45). In contrast, a molecule QL03948 (Figure 9) with the lowest value of energy (−7.80 kcal/mol) has TPC equal to 5.35 due to the eight atoms in the main chain and even lower X% (36.36) because only eight fluorine atoms are present in the molecule. The group of compounds with moderate binding probability includes molecules with X% in the range from 32.14 to 66.67 and TPC in the range from 5.15 to 6.09. In the third class (moderately high binding probability) compound QL02564 (Figure 9) has the lowest value of binding energy (−10.5 kcal/mol) with a TPC value equal to 6.45 due to the 13 atoms in the main chain and a relatively high value of X% (53.85) because of 10 fully fluorinated carbons.

In the case of TRβ intra-group classification (Figure 10B), sulfonic acid with five carbon atoms in the structure (S4) is classified as a group with moderate binding probability, and eight or more carbon atoms in a molecule (S7-8) it is classified as a moderately high binding probability. For all other groups (perfluorinated carboxylic acids, fluorotelomer alcohols, phosphonic acids, and dicarboxylic acids), a decrease in the value of the binding score parameter can also be observed as the molecule is extended by another -CF_2_ group (Figure 10C).

For the TRβ receptor, there are no studies available that describe the binding probability of PFAS to the docking site we studied (3IMY). Young et al. [66] in their work presented a classification of selected PFAS as active (PFOS, FOSA, PFNA, PFDA) and as inactive (PFHxA, PFOA, PFHpA, and PFUnDA) antagonists against thyroid hormone receptor β. Our docking results classified PFOS, FOSA, PFNA, and PFDA compounds to second class with moderately high binding probability (binding score is not higher than –9.6 kcal/mol). Behnisch et al. [67] have presented research based on the TTR-TRβ-CALUX^®^ assay in which the activity of 13 PFAS was demonstrated, which indicates a potential for disturbing the functioning of thyroid hormones. Potency factors were expressed as PC_80_-based and IC_50_-based using PFOA as a reference compound. Based on the PC_80_ values, short-chain compounds such as PFBS or PFBA and the longest compound tested (PFDcA) showed lower activity than the reference compound. Higher activity than PFOA was reported for PFHpA, PFHxS, and PFOS. It is not possible to directly compare the docking results we obtained with the study conducted in the above paper, due to the completely different nature of the experiment. However, based on the binding score values we calculated for the PFAS group studied here, PFOS can be identified as the compound with the highest probability of binding to the receptor.

## 3. Discussion—The Biological Significance of the In Silico Predictions

In the presented work we raised the important topic regarding the effects of the presence of PFAS in the environment and their impact on humans. Due to the constantly emerging PFAS in the environment, it is crucial to evaluate various perfluorinated compounds in terms of disrupting the human endocrine system. This study documents that the chemical structure of PFAS (particularly the carbon chain length, the number of fluorine atoms in a structure, and the number of branches) may have an influence on binding to the selected human nuclear hormone receptors. Therefore, the use of molecular docking in conjunction with the QSAR model is needed.

### 3.1. Peroxisome Proliferator-Activated Receptors

It is generally recognized that all PPAR isoforms bind to DNA as heterodimers with retinoid X-receptor (RXR), therefore PPAR/RXR heterodimers can be activated by RXR ligands as well [68]. However, our simulations were based solely on PFAS interactions with particular PPAR isoforms, more specifically with the PPAR ligand binding domain (LBD). In general, our analysis indicates that activation of PPARα, β, or γ increases with an increasing chain length of carbons saturated with fluorine. This suggests that the hydrophobic interactions between the carbon chains of PFAS and amino acid residues in the binding pocket contribute significantly to the stabilization of the PFAS/protein complex [59]. The analysis shows that the considered PFAS group bind to the PPARα, β, and γ only with low or moderate probability. The fact that all the analyzed PFAS showed only low binding strength to PPARα (in contrast to PPARβ and PPARγ for which some PFAS showed moderate binding probability) could be explained by slight differences in amino acid sequence and spatial conformation in the LBD. The molecular analysis of all three isoforms leads to rather surprising conclusions. As was proved by Xu et al [69]. PPARα isoform is composed of a helical sandwich and a four-stranded β-sheet, similar to PPARβ and PPARγ. However, the PPARα and PPARγ LBD are significantly larger and more resemble each other in terms of size and shape in comparison to the PPARβ pocket which has a narrowing of the pocket adjacent to the activation function-2 (AF-2) surface. One can assume that such a situation should favor easier binding of larger variety of PFAS molecules, especially those of bigger sizes, to PPARα and PPARγ in comparison to PPARβ. It remains to be investigated why we observed comparatively lower binding of PFAS to PPARα. The one difference in the case of the PPARα pocket is that it is considered more lipophilic and less solvent exposed than the LBD of PPARβ and PPARγ [69]. Possibly, the more hydrophobic nature of the PPARα pocket may predispose it to bind to the more lipophilic PFAS.

Our in silico predictions may be in contrast to the biological results obtained by other researchers. Generally, PFAS were shown to activate mouse and human PPARα in the in vitro assays using cells transfected with PPARα reporter constructs, with higher sensitivity of the mouse assays [22,23,24]. In these studies, the PFAS with a carboxylic acid group showed a higher potential to activate PPARα than the PFAS with a sulfonic acid group [22]. PFAS potential towards activating PPARβ and PPARγ also differed depending on the carbon chain length and the terminal functional group. Interestingly, in the study by Li et al. [58] the three perfluorinated sulfonic acids used exhibited a stronger binding potency to PPARβ than their carboxylic counterparts, a phenomenon which is opposite to the results generally observed for PPARα. These observations indicate rather complex and yet not well understood interactions of PFAS with PPARs. It is known that the in vitro assays have their limitations and do not necessarily reflect complex nuclear receptor interactions in vivo, e.g., activation of other receptors by ligands, the influence of co-activators, or endogenous ligands. In our opinion, while assessing the activating effects of PFAS on PPARs (but also on other NHRs) the possibility of their binding to alternate sites not only to the canonical LBD should be considered [70].

### 3.2. Thyroid Hormone Receptors

In the case of TRα and TRβ, we observed that both the shape and size of the PFAS molecule or the percentage of fluorine atoms have a considerable influence on binding strength to these receptors. The PFAS compounds with longer chains showed a moderately high probability of binding. Our results are to some extent corroborated by the study of Mortensen et al., who predicted that in the gull TRα model several PFAS, including two perfluoroalkyl carboxylic acids (PFCA), could bind to the LBD [71]. In that study, long-chained PFCA were the strongest binders, possibly due to their enhanced hydrophobicity and the highly hydrophobic LBD of TRα. Based on the data some PFASs that are not aromatic and have a different structure from the thyroid hormones, resembling more fatty acids and acyl-CoA esters, can be predicted to be relatively good TR ligands. PFOS was predicted in our study as a good binder to TRα and TRβ, which was supported by molecular docking and biological testing by Ren et al. [72] and Xiu et al. [73] According to Xiu et al., PFOS fits into the LBD of TRα and TRβ with the negative charged end group bound near the entrance of the LBD, and the hydrophobic part towards the interior of the LBD [73].

In our study perfluoroacids with C8–C10 were classified as having a moderately high binding potential to both TRs, which was fully supported by the results obtained by Ren et al. [72] who reported that acids with C8–C18 effectively bound to TRα-LBD. Moreover, a very interesting correlation between our predictions and the biological relative potency of binding of perfluoroalkyl sulfonates to TRα-LBD as reported in the study by Ren et al. [72] can be observed. In the case of TRα, we observed an increased binding probability for sulfonic acids with ≥5 carbon atoms in the structure (S4–S8). A very similar increasing trend was observed in the TRα-LBD binding assay by Ren et al. [72] for perfluorobutane sulfonate (PFBS, RP = 0.0003 relative to T3), perfluorohexane sulfonate (PFHxS, RP = 0.0015 relative to T3), perfluorooctane sulfonate (PFOS, RP = 0.01875 relative to T3).

## 4. Materials and Methods

### 4.1. Dataset

Dataset for the development QSAR models.

The commonly used 43 PFAS encompassing perfluorinated carboxylic acids (PFCAs), fluorotelomer alcohols (FTOHs), perfluorinated sulfonic acids (PFSAs), and other related PFAS, were selected [19]. Names, acronyms, CAS numbers, and SMILES of PFAS examined in this study are listed in Supplementary Materials S1.

Dataset for virtual screening of PFAS.

The external dataset including more than 5000 per- and polyfluoroalkyl compounds from NORMAN Database System was downloaded [57]. The chemicals without SMILES were removed from the PFAS dataset. The resultant dataset contains a total of 4464 PFAS. For all details regarding this dataset please refer to Supplementary Materials S1.

### 4.2. Docking Score Calculations

Docking scores of 43 PFAS (using SMILES code) to five human nuclear hormone receptors (PPARα, PPARβ, PPARγ, TRα, TRβ) were calculated using the Endocrine Disruptome-open-source prediction tool [41] based on the technique of inverse docking. The 3D crystal structures integrated with Endocrine Disruptome are 3KDU (PPARα), 3GZ9 (PPARβ), 3ET3 (PPARγ), 3ILZ (TRα), 3IMY (TRβ). All modeled compounds have a molecular weight lower than 600 g/mol, which is in line with the accuracy of the predictions of this tool. Docking simulations are provided by Docking Interface for Target Systems (DOTS) [41] platform, while the docking calculations were run with AutoDock Vina.

Obtained results were color-coded into four probability binding classes according to the thresholds set in the tool [41], where “green” corresponds to a low probability of binding, “yellow” corresponds to a moderate probability of binding, “orange” indicates a moderately high binding probability and “red” indicates a high probability of binding (Table 2). For docking results please refer to Supplementary Materials S1.

### 4.3. Molecular Descriptors

The molecular descriptors are chemical structures translated into numbers, which represent information about a chemical compound based on its chemical constitution elements. Based on the descriptors, it is possible to determine the correlation of chemical structure with biological activity [74]. For all selected 43 PFAS a total number of 186 1D and 2D descriptors were calculated in alvaDesc software (version 2.0.10, Alvascience, Lecco (Italy)) using SMILES notation [75]. These descriptors belong to constitutional indices, topological indices, walk and path counts, and molecular properties. The number of descriptors has been firstly reduced by constant values and by descriptors with no data for at least one compound. For the modeling, we applied the multiple linear regression method (MLR) combined with a genetic algorithm (GA). The GA was used to select the optimal combination of the previously calculated structural descriptors, to be utilized in the final model (Supplementary Materials S2). 

### 4.4. QSAR Model Calibration and Validation

In this work five models to predict PFAS binding probability to PPARα, PPARβ, PPARγ, TRα, and TRβ were developed. After collecting docking scores from the Endocrine Disruptome tool [41] the dataset was split into training (T) and validation (V) sets by following the “1:Z” algorithm [76]. In this case, every *Z*^th^ compound in a group of chemicals, sorted by calculated values of docking score in ascending order, was assigned to the validation set, and the remaining compounds created the training set. Here, for PPARα, PPARβ, and TRβ, Z = 3, while for TRα and PPARγ Z = 4, which means 14 and 10 compounds were assigned to the validation set, respectively. The training set is used to build the model and the validation set is necessary to assess the predictability of the QSAR model [77]. Before training the models for estimating binding energy to NHRs, the independent variables (descriptors) and dependent variables (docking scores) were preprocessed by standard scaling [78]. For scientific validity of the QSAR models [49] all of them were developed in accordance with the standards and recommendations of the Organization for Economic Co-operation and Development (OECD) [46,47,48]. The analyses were carried out using packages available in Python 3.8.8 [79]. For defining the relationship between the structure of PFAS and binding probability (endpoint) to NHRs the multiple linear regression (MLR) method [80,81] was applied. Structural descriptors, which have an influence on the probability of binding to the receptor, and which were readily interpretable, were selected. Only a combination of molecular descriptors, which correlation does not exceed 0.6, were considered. One of the most important stages in the calibration of the QSAR model is to verify the goodness-of-fit by calculating the appropriate parameters. In this work, the squared regression coefficient (R^2^), which measures the variation of observed data with the fitted one (preferably if R^2^ is equal to 1) and the root mean square error of calibration (RMSE_C_), based on the prediction for the training set, were estimated. To demonstrate the robustness and predictive ability the validation of obtained models was divided into two main steps: (i) internal validation (by using the training set) and (ii) external validation (by using the validation set). In the case of internal validation, the leave-one-out cross-validation method was used. This involves modifying the original training set of n compounds by removing one compound and predicting its activity using the model developed with n-1 compounds. Several parameters were estimated for verifying the robustness of the developed models: cross-validation coefficient (Q^2^_LOO_), the root mean square error of cross-validation (RMSE_CV_), mean absolute error (MAE_CV_), concordance correlation coefficient (CCC_CV_) [42]. The external validation of the developed models was performed using the validation set defined in the first step of the modeling procedure. To indicate if the developed models predict correctly, the external coefficients Q^2^ (F1, F2, and F3), the root mean square error (RMSE_EXT_), and the mean absolute error (MAE_EXT_) were calculated. In addition, a parameter CCC_EXT_ (concordance correlation coefficient), which measures precision and accuracy, detecting the distance of the observations from the fitting line was obtained [82]. All calculated parameters can be found in Supplementary Materials S1. A detailed description of the applied metrics can be found in the literature [83]. To ensure that developed models are not correlated by chance, the Y-randomization has been performed and the R^2^Y_SCR_ was evaluated. The applicability domain (AD), defined as the scope and limitations of a model, was verified using the Williams plot [48,81] and Insubria graph [55] (according to h*). Williams plot is a graphical representation of standardized residuals (y-axis) versus leverages (hat values; x-axis) for each compound of the dataset [84]. The QMRF documents for all five developed models are implemented in Supplementary Materials S2.

### 4.5. Screening of the Binding Potential to NHRs for a Large Set of PFAS

After the development and validation of models, they have been applied for predicting binding energies to peroxisome proliferator-activated (α, β, and γ) and thyroid hormone (α and β) receptors for the 4464 PFAS implemented in the NORMAN Database System, for which data are not available so far. All compounds with their Names, CAS numbers, and SMILES are attached in Supplementary Materials S1. The predictions were verified if they are in the AD using the Williams plot and Insubria Graph (involving leverage value h*). Results obtained for all compounds were color coded into four classes according to the threshold set in the tool: [41] “green”—low probability of binding; “yellow”—moderately probability of binding; “orange”—moderately high binding probability; and “red”—a high probability of binding (Table 2). Screening of a new comprehensive dataset was conducted to determine the dependence of receptor binding strength on a chemical structure.

## 5. Conclusions

As a result of intensive industrial development, new diverse PFAS are constantly emerging in the environment. Therefore, it is important to implement in silico methods to assess their impact on the human body. A combination of molecular docking and QSAR models makes it possible to determine which structural features of a chemical compound influence endocrine system disruption. In this work, we have presented an analysis of the applicability of the developed models, which shows that they can be successfully used to predict the probability of PFAS binding to nuclear human receptors. Furthermore, it should be borne in mind that compounds with very high structural variability appear in the environment, so they should be approached with caution.

When analyzing the classification of PFAS into functional groups, the trend was observed indicating an increase in binding strength to the selected NHRs as the descriptor values increase. The analysis of available literature data on biological significance of the observed predictions generally correlated well with the measured binding potential of PFAS to the NHRs in biological assays. The differences observed may result from some limitations of the in vitro assays, mainly the possibility of activating NHRs through the binding of PFAS to alternative binding sites in NHRs. In our opinion compounds from the NORMAN Database include various specific elements and functional groups, therefore the developed models based on selected molecular descriptors are rather suitable for linear perfluorinated compounds. To gain additional knowledge on PFAS binding to human nuclear receptors, further scientific studies are needed. However, our results suggest that PPARα,β,γ and TRα,β mediated pathways should be taken into consideration when studying the toxicity mechanisms of PFAS.

## Figures and Tables

**Table 1:** Statistical parameters of to PPARα, PPARβ, PPARγ, TRα, and TRβ models.

Model	PPAR α	PPAR β	PPAR γ	TR α	TR β
**Equation**	PPARα BS = −7.499 (±0.067) − 0.947 (±0.070) × ICR − 0.394 (±0.070) × PW2	PPARβ BS = −8.248 (±0.069) − 1.059 (±0.071) × ICR − 0.409 (±0.071) × X%	PPARγ BS = −7.727 (±0.052) − 1.099 (±0.053) × ICR − 0.398 (±0.053) × X%	TRα BS = −8.230 (±0.070) − 0.454 (±0.071) × X% − 1.189 (±0.071) × ICR	TRβ BS = −8.724 (±0.054) − 0.137 (±0.061) × X% − 1.509 (±0.061) × TPC
**n**	29	29	33	33	29
**k**	14	14	10	10	14
**R^2^**	0.917	0.924	0.949	0.924	0.970
**RMSE_C_**	0.341	0.352	0.287	0.384	0.276
**MAE_C_**	0.288	0.272	0.201	0.327	0.221
**Q^2^_LOO_**	0.878	0.903	0.940	0.908	0.955
**RMSE_CV_**	0.350	0.398	0.310	0.422	0.340
**MAE_CV_**	0.244	0.304	0.219	0.360	0.256
**Q^2^_F1_**	0.897	0.917	0.907	0.899	0.948
**Q^2^_F2_**	0.897	0.916	0.906	0.899	0.947
**Q^2^_F3_**	0.912	0.922	0.952	0.934	0.954
**RMSE_EXT_**	0.352	0.359	0.279	0.359	0.344
**MAE_EXT_**	0.257	0.279	0.223	0.312	0.280
**CCC_EXT_**	0.937	0.952	0.953	0.946	0.973
**Y^2^_SCR_**	0.070	0.068	0.062	0.061	0.070

**Table 2:** Threshold values of binding free energies for PPARα, PPARβ, PPARγ, TRα, TRβ.

**Nuclear Hormone Receptor**	**High probability**	**Threshold 1**	**Moderately high probability**	**Threshold 2**	**Moderate probability**	**Threshold 3**	**Low probability**
PPARα		−10		−9.4		−8.9	
PPARβ		−10.5		−10.1		−9.6	
PPARγ		−10.3		−9.6		−8.9	
TRα		−10.2		−9.2		−7.2	
TRβ		−10.5		−9.4		−7.8	

## Data Availability

Not applicable.

## Supplementary Materials

The following are available online at https://www.mdpi.com/article/10.3390/molecules28020479/s1, QMRF prepared for developed models.

## Sample Availability

Samples of the compounds are not available from the authors.
