# Supplementary material for: How the Structure of Per- and Polyfluoroalkyl Substances (PFAS) Influences Their Binding Potency to the Peroxisome Proliferator-Activated and Thyroid Hormone Receptors—An In Silico Screening Study
Source: Molecules. 2023 Jan 4;28(2):479. doi: 10.3390/molecules28020479 (PMC9866891; doi:10.3390/molecules28020479)
Supplement: Supplementary file 1 [file molecules-28-00479-s001.zip › Supplementary Materials 1.1 - Introduction.pdf]

Supplementary Materials 1

How the structure of Per- and Polyfluoroalkyl Substances (PFAS) influences their binding potency to the peroxisome proliferator-activated and thyroid hormone receptors – an in silico screening study.

Authors: Dominika Jurkiewicz[1], Anita Sosnowska[1*], Natalia Buławska[1], Maciej Stępnik[1], Harrie Besselink[2], Peter Behnisch[2], Tomasz Puzyn[1,3*]

1. QSAR Lab, ul. Trzy Lipy 3, Gdańsk, Poland
2. BioDetection Systems B.V., Science Park 406, 1098XH, Amsterdam, the Netherlands
3. University of Gdansk, Faculty of Chemistry, Wita Stwosza 63, 80-308 Gdansk, Poland

* Corresponding authors.

E-mail address: t.puzyn@qsarlab.com, a.sosnowska@qsarlab.com
