# Supplementary material for: How the Structure of Per- and Polyfluoroalkyl Substances (PFAS) Influences Their Binding Potency to the Peroxisome Proliferator-Activated and Thyroid Hormone Receptors—An In Silico Screening Study
Source: Molecules. 2023 Jan 4;28(2):479. doi: 10.3390/molecules28020479 (PMC9866891; doi:10.3390/molecules28020479)
Supplement: Supplementary file 1 [file molecules-28-00479-s001.zip › Supplementary Materials 1.3- PPAR_α.pdf]

Model information	
**Type of model**	MLR
**Data split**	3:1
**Descriptors**	ICR,PW2
**Descriptors correla**	0.31
**Preprocessing**	Standard scaler - Standardize features by removing the mean and scaling to unit variance.
**Model equation**	PPARαBS=-7,499(±0,067) - 0,947(±0,07) x ICR - 0,394(±0,07) x PW2

Model statistics	
R^2	0.917
$RMSE_C$	0.341
MAE_C	0.288
Q^2_{LOO}	0.878
R^2-Q^2_{LOO}	0.039
$RMSE_{CV}$	0,350
MAE_{CV}	0.244
CCC_{CV}	0.932
Q^2_{F1}	0.897
Q^2_{F2}	0.897
Q^2_{F3}	0.912
$RMSE_{EXT}$	0.352
MAE_{EXT}	0.257
CCC_{EXT}	0.937
R^2Y_{SCR}	0,070

**MIN binding score**	-8.79
**MAX binding score**	-3.59
**HAT kryt.**	0.31

ID	NAME	CAS	SMILES	binding score (calc.)	binding score (pred.)	PW2	ICR	split (t/v)	AD (IN/OUT)
1	10:2FTOH	865-86-1	C(CO)C(C(C(C(C(C(C(C(C(C(F)(F)F)(F)F)(F)F)(F)F)(F)F)(F)F)(F)F)(F)F)(F)F)(F)F	-8.4	-8.62	0.68	2.73	T	IN
2	10:2FTUCA	70887-94-4	C(=C(C(C(C(C(C(C(C(C(C(F)(F)F)(F)F)(F)F)(F)F)(F)F)(F)F)(F)F)(F)F)(F)F)(F)F)F)C(=O)O	-9.1	-8.39	0.67	2.74	T	IN
3	4:2diPAPs	135098-69-0	C(COP(=O)(O)OCCC(C(C(C(F)(F)F)(F)F)(F)F)(F)F)C(C(C(C(F)(F)F)(F)F)(F)F)(F)F	-9	-8.58	0.66	2.96	T	IN
4	4:2FTOH	2043-47-2	C(CO)C(C(C(C(F)(F)F)(F)F)(F)F)(F)F	-6.2	-6.21	0.65	1.91	T	IN
5	4:2FTS	757124-72-4	C(CS(=O)(=O)O)C(C(C(C(F)(F)F)(F)F)(F)F)(F)F	-6.6	-7.24	0.67	2.17	T	IN
6	5:3FTCA	914637-49-3	C(CC(C(C(C(C(F)(F)F)(F)F)(F)F)(F)F)(F)F)C(=O)O	-7.1	-7.13	0.66	2.24	V	IN
7	6:2 Cl-PFESA	756426-58-1	C(C(C(C(F)(F)Cl)(F)F)(F)F)(C(C(OC(C(F)(F)S(=O)(=O)O)(F)F)(F)F)(F)F)(F)F	-8.5	-8.43	0.69	2.51	T	IN
8	6:2FTOH	647-42-7	C(CO)C(C(C(C(C(C(F)(F)F)(F)F)(F)F)(F)F)(F)F)(F)F	-7.2	-7.11	0.66	2.23	V	IN
9	6:2FTS	27619-97-2	C(CS(=O)(=O)O)C(C(C(C(C(C(F)(F)F)(F)F)(F)F)(F)F)(F)F)(F)F	-7.5	-7.78	0.67	2.44	T	IN
10	6:2FTUCA	70887-88-6	C(=C(C(C(C(C(C(F)(F)F)(F)F)(F)F)(F)F)(F)F)F)C(=O)O	-7.4	-7.13	0.66	2.24	V	IN
11	6:2PAPs	57678-01-0	C(COP(=O)(O)O)C(C(C(C(C(C(F)(F)F)(F)F)(F)F)(F)F)(F)F)(F)F	-7.6	-7.92	0.67	2.51	V	IN
12	8:2FTOH	678-39-7	C(CO)C(C(C(C(C(C(C(C(F)(F)F)(F)F)(F)F)(F)F)(F)F)(F)F)(F)F)(F)F	-8.3	-7.90	0.67	2.50	T	IN
13	8:2FTS	39108-34-4	C(CS(=O)(=O)O)C(C(C(C(C(C(C(C(F)(F)F)(F)F)(F)F)(F)F)(F)F)(F)F)(F)F)(F)F	-8.5	-8.52	0.68	2.68	V	IN
14	8:2FTUCA	70887-84-2	C(=C(C(C(C(C(C(C(C(F)(F)F)(F)F)(F)F)(F)F)(F)F)(F)F)(F)F)F)C(=O)O	-8.2	-7.92	0.67	2.51	T	IN
15	EtFOSA	4151-50-2	CCNS(=O)(=O)C(C(C(C(C(C(C(C(F)(F)F)(F)F)(F)F)(F)F)(F)F)(F)F)(F)F)(F)F	-8.6	-8.27	0.67	2.68	T	IN
16	EtFOSE	1691-99-2	CCN(CCO)S(=O)(=O)C(C(C(C(C(C(C(C(F)(F)F)(F)F)(F)F)(F)F)(F)F)(F)F)(F)F)(F)F	-7.9	-8.14	0.66	2.74	V	IN
17	FOSA	754-91-6	C(C(C(C(C(F)(F)S(=O)(=O)N)(F)F)(F)F)(F)F)(C(C(C(F)(F)F)(F)F)(F)F)(F)F	-8.3	-8.28	0.69	2.44	V	IN
18	MeFOSA	31506-32-8	CNS(=O)(=O)C(C(C(C(C(C(C(C(F)(F)F)(F)F)(F)F)(F)F)(F)F)(F)F)(F)F)(F)F	-8.4	-8.17	0.68	2.51	T	IN
19	MeFOSE	24448-09-7	CN(CCO)S(=O)(=O)C(C(C(C(C(C(C(C(F)(F)F)(F)F)(F)F)(F)F)(F)F)(F)F)(F)F)(F)F	-7.8	-8.39	0.67	2.74	T	IN
20	Mhopflca n=6	-	C(C(=O)O)OCC(C(C(C(C(C(F)(F)F)(F)F)(F)F)(F)F)(F)F)(F)F	-7.4	-7.69	0.66	2.52	T	IN
21	Mhopflca n=7	-	C(C(=O)O)OCC(C(C(C(C(C(C(F)(F)F)(F)F)(F)F)(F)F)(F)F)(F)F)(F)F	-7.7	-8.06	0.66	2.70	T	IN
22	Mhopflca n=8	-	C(C(=O)O)OCC(C(C(C(C(C(C(C(F)(F)F)(F)F)(F)F)(F)F)(F)F)(F)F)(F)F)(F)F	-8.2	-8.39	0.67	2.74	V	IN
23	PFBA	375-22-4	C(=O)(C(C(C(F)(F)F)(F)F)(F)F)O	-5.6	-5.81	0.67	1.46	T	IN
24	PFBS	375-73-5	C(C(C(F)(F)S(=O)(=O)O)(F)F)(C(F)(F)F)(F)F	-6.3	-6.97	0.69	1.79	V	IN
25	PFCHS	2106-55-0	C1(C(C(C(C(C1(F)F)(F)F)(F)S(=O)(=O)O)(F)F)(F)F)(F)F	-7.2	-6.58	0.70	1.47	T	IN
26	PFDA	335-76-2	C(=O)(C(C(C(C(C(C(C(C(C(F)(F)F)(F)F)(F)F)(F)F)(F)F)(F)F)(F)F)(F)F)(F)F)O	-8.5	-8.43	0.69	2.51	T	IN
27	PFEtS	354-88-1	C(C(F)(F)S(=O)(=O)O)(F)(F)F	-4.9	-5.77	0.68	1.32	V	IN
28	PFHpA	375-85-9	C(=O)(C(C(C(C(C(C(F)(F)F)(F)F)(F)F)(F)F)(F)F)(F)F)O	-7.5	-7.47	0.68	2.16	T	IN
29	PFHpS	375-92-8	C(C(C(C(F)(F)F)(F)F)(F)F)(C(C(C(F)(F)S(=O)(=O)O)(F)F)(F)F)(F)F	-8	-7.88	0.69	2.24	T	IN
30	PFHxA	307-24-4	C(=O)(C(C(C(C(C(F)(F)F)(F)F)(F)F)(F)F)(F)F)O	-7.1	-6.94	0.68	1.90	T	IN
31	PFHxPA	40143-76-8	C(C(C(C(F)(F)P(=O)(O)O)(F)F)(F)F)(C(C(F)(F)F)(F)F)(F)F	-7.9	-7.70	0.69	2.15	T	IN
32	PFHxS	355-46-4	C(C(C(C(F)(F)S(=O)(=O)O)(F)F)(F)F)(C(C(F)(F)F)(F)F)(F)F	-7.2	-7.70	0.69	2.15	T	IN
33	PFNA	375-95-1	C(=O)(C(C(C(C(C(C(C(C(F)(F)F)(F)F)(F)F)(F)F)(F)F)(F)F)(F)F)(F)F)O	-8	-8.05	0.68	2.45	V	IN
34	PFNS	68259-12-1	C(C(C(C(C(F)(F)F)(F)F)(F)F)(F)F)(C(C(C(C(F)(F)S(=O)(=O)O)(F)F)(F)F)(F)F)(F)F	-8.7	-8.43	0.69	2.51	V	IN
35	PFOA	335-67-1	C(=O)(C(C(C(C(C(C(C(F)(F)F)(F)F)(F)F)(F)F)(F)F)(F)F)(F)F)O	-7.9	-7.63	0.68	2.24	T	IN
36	PFOPA	40143-78-0	C(C(C(C(C(F)(F)P(=O)(O)O)(F)F)(F)F)(F)F)(C(C(C(F)(F)F)(F)F)(F)F)(F)F	-8.5	-8.28	0.69	2.44	V	IN
37	PFOS	1763-23-1	C(C(C(C(C(F)(F)S(=O)(=O)O)(F)F)(F)F)(F)F)(C(C(C(F)(F)F)(F)F)(F)F)(F)F	-8.2	-8.28	0.69	2.44	T	IN
38	PFPeA	2706-90-3	C(=O)(C(C(C(C(F)(F)F)(F)F)(F)F)(F)F)O	-6.1	-6.51	0.67	1.81	T	IN
39	PFPeS	2706-91-4	C(C(C(F)(F)F)(F)F)(C(C(F)(F)S(=O)(=O)O)(F)F)(F)F	-6.9	-7.20	0.69	1.90	T	IN
40	PFPrA	422-64-0	C(=O)(C(C(F)(F)F)(F)F)O	-4.9	-5.35	0.66	1.36	T	IN
41	PFPrS	110676-15-8	C(C(F)(F)F)(C(F)(F)S(=O)(=O)O)(F)F	-5.7	-6.04	0.68	1.45	V	IN
42	PFUdA	2058-94-8	C(=O)(C(C(C(C(C(C(C(C(C(C(F)(F)F)(F)F)(F)F)(F)F)(F)F)(F)F)(F)F)(F)F)(F)F)(F)F)O	-8.7	-8.79	0.69	2.69	T	IN
43	TFA	76-05-1	C(=O)(C(F)(F)F)O	-4.1	-3.59	0.63	0.86	T	OUT

QL00001	Europium, tris[6,6,7,7,8,8,8-heptafluoro-2,2-di(methyl-d3)-3,5-octanedion-1,1,1-d3-ato-ßO3,ßO5]-|Praseodymium, tris[6,6,7,7,8,8,8-heptafluoro-2,2-di(methyl-d3)-3,5-octanedion-1,1,1-d3-ato-O,O']- (9CI)	36393-27-8|42942-19-8	[2H]C([2H])(C(C([2H])([2H])[2H])(C([2H])([2H])[2H])C(/C=C(C(F)(C(F)(C(F)(F)F)F)F)O)=O)[2H]		-6.95	0.66	2.15	P	IN
QL00002	Silver perfluorooctanoate	335-93-3	[Ag+].[O-]C(=O)C(F)(F)C(F)(F)C(F)(F)C(F)(F)C(F)(F)C(F)(F)C(F)(F)F		-7.29	0.66	2.39	P	IN
QL00003	Silver heptafluorobutyrate	3794-64-7	[Ag+].[O-]C(=O)C(F)(F)C(F)(F)C(F)(F)F		-4.97	0.62	1.73	P	OUT
QL00004	Silver, (6,6,7,7,8,8,8-heptafluoro-2,2-dimethyl-3,5-octanedionato-βO3,βO5)-	76121-99-8	[Ag+].CC(C)(C)C([O-])=CC(=O)C(F)(F)C(F)(F)C(F)(F)F		-6.39	0.63	2.33	P	OUT
QL00005	Aluminum perfluoro-6-methylheptanoate	15715-47-6	[Al+3].[O-]C(=O)C(F)(F)C(F)(F)C(F)(F)C(F)(F)C(F)(C(F)(F)F)C(F)(F)F.[O-]C(=O)C(F)(F)C(F)(F)C(F)(F)C(F)(F)C(F)(C(F)(F)F)C(F)(F)F.[O-]C(=O)C(F)(F)C(F)(F)C(F)(F)C(F)(F)C(F)(C(F)(F)F)C(F)(F)F		-7.31	0.67	2.20	P	IN
QL00006	1-Octanesulfonic acid, 3,3,4,4,5,5,6,6,7,7,8,8,8-tridecafluoro-, barium salt (21)	1807944-82-6	[Ba++].[O-]S(=O)(=O)CCC(F)(F)C(F)(F)C(F)(F)C(F)(F)C(F)(F)C(F)(F)F.[O-]S(=O)(=O)CCC(F)(F)C(F)(F)C(F)(F)C(F)(F)C(F)(F)C(F)(F)F		-7.71	0.66	2.53	P	IN
QL00007	Barium bis(6,6,7,7,8,8,8-heptafluoro-2,2-dimethyl-5-oxooct-3-en-3-olate)	36885-31-1	[Ba++].CC(C)(C)C([O-])=CC(=O)C(F)(F)C(F)(F)C(F)(F)F.CC(C)(C)C([O-])=CC(=O)C(F)(F)C(F)(F)C(F)(F)F		-6.71	0.64	2.27	P	IN
QL00008	5-(Perfluoropropyl)-2,2-dimethyl-3,5-pentanedione barium(2+) (21)	118360-70-6	[Ba++].CC(C)(C)C(=O)CC(=O)C(F)(F)C(F)(F)C(F)(F)F.CC(C)(C)C(=O)CC(=O)C(F)(F)C(F)(F)C(F)(F)F		-6.71	0.64	2.27	P	IN
QL00009	Perfluorooctane sulfonamido ammonium bromide	73149-44-7	[Br-].C[N+](C)(C)CCCNS(=O)(=O)C(F)(F)C(F)(F)C(F)(F)C(F)(F)C(F)(F)C(F)(F)C(F)(F)C(F)(F)F		-8.46	0.65	3.03	P	IN
QL00010	2-(2,4-Di-tert-pentylphenoxy)-3'-hydroxy-4'-[(5H-perfluorovaleryl)amino]hexananilide	72494-14-5	[C@H](CCCC)(C(=O)NC1=CC=C(C(=C1)O)NC(=O)C(C(C(C(F)F)(F)F)(F)F)(F)F)OC2=CC=C(C=C2C(CC)(C)C)C(CC)(C)C		-8.04	0.61	3.37	P	OUT
QL00011	Perfluoroundecanoic acid calcium salt (21)	97163-17-2	[Ca++].[O-]C(=O)C(F)(F)C(F)(F)C(F)(F)C(F)(F)C(F)(F)C(F)(F)C(F)(F)C(F)(F)C(F)(F)C(F)(F)F.[O-]C(=O)C(F)(F)C(F)(F)C(F)(F)C(F)(F)C(F)(F)C(F)(F)C(F)(F)C(F)(F)C(F)(F)C(F)(F)F		-8.59	0.68	2.76	P	IN
QL00012	Calcium (3E)-6,6,7,7,8,8,8-heptafluoro-2,2-dimethyl-5-oxooct-3-en-3-olate (3Z)-6,6,7,7,8,8,8-heptafluoro-2,2-dimethyl-5-oxooct-3-en-3-olate (1/1/1)	124053-49-2	[Ca++].CC(C)(C)C([O-])=C/C(=O)C(F)(F)C(F)(F)C(F)(F)F.CC(C)(C)C([O-])=CC(=O)C(F)(F)C(F)(F)C(F)(F)F		-6.71	0.64	2.27	P	IN
QL00013	Cadmium bis(1,1,2,2,3,3,4,4,4-nonafluorobutan-1-ide)	93367-04-5	[Cd++].F[C-](F)C(F)(F)C(F)(F)C(F)(F)F.F[C-](F)C(F)(F)C(F)(F)C(F)(F)F		-5.42	0.64	1.63	P	IN
QL00014	Cerium, tris(6,6,7,7,8,8,8-heptafluoro-2,2-dimethyl-3,5-octanedionato-βO3,βO5)-	172424-98-5	[Ce+3].CC(C)(C)C([O-])=CC(=O)C(F)(F)C(F)(F)C(F)(F)F.CC(C)(C)C([O-])=CC(=O)C(F)(F)C(F)(F)C(F)(F)F.CC(C)(C)C([O-])=CC(=O)C(F)(F)C(F)(F)C(F)(F)F		-6.81	0.65	2.24	P	IN
QL00015	Palladium, dichlorobis[tris[4-(2-(perfluorooctyl)ethyl)phenyl]phosphine-βP]-	326475-46-1	[Cl-].[Cl-].[Pd++].FC(F)(F)C(F)(F)C(F)(F)C(F)(F)C(F)(F)C(F)(F)C(F)(F)C(F)(F)CCC1=CC=C(C=C1)P(C1=CC=C(CCC(F)(F)C(F)(F)C(F)(F)C(F)(F)C(F)(F)C(F)(F)C(F)(F)C(F)(F)F)C=C1)C1=CC=C(CCC(F)(F)C(F)(F)C(F)(F)C(F)(F)C(F)(F)C(F)(F)C(F)(F)C(F)(F)F)C=C1.FC(F)(F)C(F)(F)C(F)(F)C(F)(F)C(F)(F)C(F)(F)C(F)(F)C(F)(F)CCC1=CC=C(C=C1)P(C1=CC=C(CCC(F)(F)C(F)(F)C(F)(F)C(F)(F)C(F)(F)C(F)(F)C(F)(F)C(F)(F)F)C=C1)C1=CC=C(CCC(F)(F)C(F)(F)C(F)(F)C(F)(F)C(F)(F)C(F)(F)C(F)(F)C(F)(F)F)C=C1		-10.21	0.66	3.87	P	OUT
QL00016	Palladium, dichlorobis[tris[4-(heptadecafluorooctyl)phenyl]phosphine-βP]- (9CI)	326475-44-9	[Cl-].[Cl-].[Pd++].FC(F)C(F)(F)C(F)(F)C(F)(F)C(F)(F)C(F)(F)C(F)(F)C1=CC=C(C=C1)P(C1=CC=C(C=C1)C(F)(F)C(F)(F)C(F)(F)C(F)(F)C(F)(F)C(F)(F)C(F)(F)C(F)(F)F)C1=CC=C(C=C1)C(F)(F)C(F)(F)C(F)(F)C(F)(F)C(F)(F)C(F)(F)C(F)(F)C(F)(F)F.FC(F)(F)C(F)(F)C(F)(F)C(F)(F)CC(F)(F)C(F)(F)C(F)(F)C1=CC=C(C=C1)P(C1=CC=C(C=C1)C(F)(F)C(F)(F)C(F)(F)C(F)(F)C(F)(F)C(F)(F)C(F)(F)C(F)(F)F)C1=CC=C(C=C1)C(F)(F)C(F)(F)C(F)(F)C(F)(F)C(F)(F)C(F)(F)C(F)(F)C(F)(F)F		-10.02	0.66	3.71	P	OUT
QL00017	62 Fluorotelomer thiohydroxy ammonium chloride	88992-45-4	[Cl-].C[N+](C)(C)CC(O)CSCCC(F)(F)C(F)(F)C(F)(F)C(F)(F)C(F)(F)C(F)(F)F		-7.77	0.63	2.98	P	OUT
QL00018	N,N,N-trimethyl-3-[(perfluorooctanoyl)amino]-1-propanaminium chloride	53517-98-9	[Cl-].C[N+](C)(C)CCCNC(=O)C(F)(F)C(F)(F)C(F)(F)C(F)(F)C(F)(F)C(F)(F)C(F)(F)F		-8.11	0.64	3.01	P	OUT

QL00019	[3-[[(heptadecafluorooctyl)sulphonyl]amino]propyl]trimethylammonium chloride	38006-74-5	[Cl-].C[N+](C)(C)CCCNS(=O)(=O)C(F)(F)C(F)(F)C(F)(F)C(F)(F)C(F)(F)C(F)(F)C(F)(F)C(F)(F)F		-8.46	0.65	3.03	P	IN
QL00020	Trimethyl-3-[[(pentadecafluoroheptyl)sulphonyl]amino]propylammonium chloride	68555-81-7	[Cl-].C[N+](C)(C)CCCNS(=O)(=O)C(F)(F)C(F)(F)C(F)(F)C(F)(F)C(F)(F)C(F)(F)C(F)(F)F		-8.28	0.65	3.00	P	IN
QL00021	Trimethyl-3-[[(tridecafluorohexyl)sulphonyl]amino]propylammonium chloride	52166-82-2	[Cl-].C[N+](C)(C)CCCNS(=O)(=O)C(F)(F)C(F)(F)C(F)(F)C(F)(F)C(F)(F)C(F)(F)F		-7.86	0.64	2.86	P	IN
QL00022	Trimethyl-3-[[(undecafluoropentyl)sulphonyl]amino]propylammonium chloride	68957-55-1	[Cl-].C[N+](C)(C)CCCNS(=O)(=O)C(F)(F)C(F)(F)C(F)(F)C(F)(F)C(F)(F)F		-7.64	0.64	2.82	P	OUT
QL00023	Trimethyl-3-[[(nonafluorobutyl)sulphonyl]amino]propylammonium chloride	53518-00-6	[Cl-].C[N+](C)(C)CCCNS(=O)(=O)C(F)(F)C(F)(F)C(F)(F)C(F)(F)F		-7.12	0.63	2.66	P	OUT
QL00024	Chromium(3+) perfluorooctanoate	68141-02-6	[Cr+3].[O-]C(=O)C(F)(F)C(F)(F)C(F)(F)C(F)(F)C(F)(F)C(F)(F)C(F)(F)F.[O-]C(=O)C(F)(F)C(F)(F)C(F)(F)C(F)(F)C(F)(F)C(F)(F)C(F)(F)F.[O-]C(=O)C(F)(F)C(F)(F)C(F)(F)C(F)(F)C(F)(F)C(F)(F)C(F)(F)F		-7.62	0.67	2.31	P	IN
QL00025	Caesium perfluoroheptanoate	171198-24-6	[Cs+].[O-]C(=O)C(F)(F)C(F)(F)C(F)(F)C(F)(F)C(F)(F)C(F)(F)F		-7.01	0.65	2.32	P	IN
QL00026	1,1,1,2,2,3,3-Heptafluoro-6-hydroxy-7,7-dimethyloct-5-en-4-one--copper (2/1)	38926-19-1	[Cu].CC(C)(C)C(O)=CC(=O)C(F)(F)C(F)(F)C(F)(F)F.CC(C)(C)C(O)=CC(=O)C(F)(F)C(F)(F)C(F)(F)F		-6.71	0.64	2.27	P	IN
QL00027	Copper(2+) bis(6,6,7,7,8,8,8-heptafluoro-2,2-dimethyl-5-oxooct-3-en-3-olate)	80289-21-0	[Cu++].CC(C)(C)C([O-])=CC(=O)C(F)(F)C(F)(F)C(F)(F)F.CC(C)(C)C([O-])=CC(=O)C(F)(F)C(F)(F)C(F)(F)F		-6.71	0.64	2.27	P	IN
QL00028	Dysprosium, tris(6,6,7,7,8,8,8-heptafluoro-2,2-dimethyl-3,5-octanedionato-βO3,βO5)-	18323-98-3	[Dy+3].CC(C)(C)C([O-])=CC(=O)C(F)(F)C(F)(F)C(F)(F)F.CC(C)(C)C([O-])=CC(=O)C(F)(F)C(F)(F)C(F)(F)F.CC(C)(C)C([O-])=CC(=O)C(F)(F)C(F)(F)C(F)(F)F		-6.81	0.65	2.24	P	IN
QL00029	Er(hfc)3	123359-45-5	[Er].CC1(C)[C@H]2CC[C@]1(C)C(=O)C2=C(/O)C(F)(F)C(F)(F)C(F)(F)F.CC1(C)[C@H]2CC[C@]1(C)C(=O)C2=C(/O)C(F)(F)C(F)(F)C(F)(F)F.CC1(C)[C@H]2CC[C@]1(C)C(=O)C2=C(O)C(F)(F)C(F)(F)C(F)(F)F		-7.01	0.66	2.18	P	IN
QL00030	Erbium, tris(6,6,7,7,8,8,8-heptafluoro-2,2-dimethyl-3,5-octanedionato-βO3,βO5)-	17978-75-5	[Er+3].CC(C)(C)C([O-])=CC(=O)C(F)(F)C(F)(F)C(F)(F)F.CC(C)(C)C([O-])=CC(=O)C(F)(F)C(F)(F)C(F)(F)F.CC(C)(C)C([O-])=CC(=O)C(F)(F)C(F)(F)C(F)(F)F		-6.81	0.65	2.24	P	IN
QL00031	Eu(fod)3-d30	56689-47-5	[Eu].[2H]C(=C(O)C(C([2H])([2H])[2H])(C([2H])([2H])[2H])C([2H])([2H])[2H])C(=O)C(F)(F)C(F)(F)C(F)(F)F.[2H]C(=C(O)C(C([2H])([2H])[2H])(C([2H])([2H])[2H])C([2H])([2H])[2H])C(=O)C(F)(F)C(F)(F)C(F)(F)F.[2H]C(=C(O)C(C([2H])([2H])[2H])(C([2H])([2H])[2H])C([2H])([2H])[2H])C(=O)C(F)(F)C(F)(F)C(F)(F)F		-6.81	0.65	2.24	P	IN
QL00032	Europium, tris[6,6,7,7,8,8,8-heptafluoro-2,2-di(methyl-d3)-3,5-octanedion-1,1,1-d3-ato-βO3,βO5]-	36393-27-8	[Eu+3].[2H]C([2H])([2H])C(C(=O)C=C(/[O-])C(F)(F)C(F)(F)C(F)(F)F)(C([2H])([2H])[2H])C([2H])([2H])[2H].[2H]C([2H])([2H])C(C(=O)C=C(/[O-])C(F)(F)C(F)(F)C(F)(F)F)(C([2H])([2H])[2H])C([2H])([2H])[2H].[2H]C([2H])([2H])C(C(=O)C=C(/[O-])C(F)(F)C(F)(F)C(F)(F)F)(C([2H])([2H])[2H])C([2H])([2H])[2H]		-6.81	0.65	2.24	P	IN
QL00033	Europium, tris(6,6,7,7,8,8,8-heptafluoro-2,2-dimethyl-3,5-octanedionato-βO3,βO5)-	17631-68-4	[Eu+3].CC(C)(C)C([O-])=CC(=O)C(F)(F)C(F)(F)C(F)(F)F.CC(C)(C)C([O-])=CC(=O)C(F)(F)C(F)(F)C(F)(F)F.CC(C)(C)C([O-])=CC(=O)C(F)(F)C(F)(F)C(F)(F)F		-6.81	0.65	2.24	P	IN
QL00034	Europium, tris[3-[2,2,3,3,4,4,4-heptafluoro-1-(oxo-βO)butyl]-1,7,7-trimethylbicyclo[2.2.1]heptan-2-onato-βO]-	34788-82-4	[Eu+3].CC1(C)C2CCC1(C)C(=O)[C-]2C(=O)C(F)(F)C(F)(F)C(F)(F)F.CC1(C)C2CCC1(C)C(=O)[C-]2C(=O)C(F)(F)C(F)(F)C(F)(F)F.CC1(C)C2CCC1(C)C(=O)[C-]2C(=O)C(F)(F)C(F)(F)C(F)(F)F		-7.01	0.66	2.18	P	IN
QL00035	Tris(6,6,7,7,8,8,8-heptafluoro-2,2-dimethyl-3,5-octanedionato)gadolinium(III)	17631-67-3	[Gd+3].CC(C)(C)C(=O)[CH-]C(=O)C(F)(F)C(F)(F)C(F)(F)F.CC(C)(C)C(=O)[CH-]C(=O)C(F)(F)C(F)(F)C(F)(F)F.CC(C)(C)C(=O)[CH-]C(=O)C(F)(F)C(F)(F)C(F)(F)F		-6.81	0.65	2.24	P	IN
QL00036	(-)-1-Perfluoropropylethanol	85571-83-1	[H][C@](C)(O)C(F)(F)C(F)(F)C(F)(F)F		-5.76	0.67	1.46	P	IN
QL00037	(2S)-2-Pyrrolidinyl-bis(4-(perfluorooctyl)phenyl)methanol	862589-19-3	[H][C@]1(CCCN1)C(O)(C1=CC=C(C=C1)C(F)(F)C(F)(F)C(F)(F)C(F)(F)C(F)(F)C(F)(F)C(F)(F)C(F)(F)F)C1=CC=C(C=C1)C(F)(F)C(F)(F)C(F)(F)C(F)(F)C(F)(F)C(F)(F)C(F)(F)C(F)(F)F		-10.17	0.67	3.69	P	OUT

QL00038	(2S)-2-Pyrrolidinyl-bis(4-(perfluorohexyl)phenyl)methanol	1075687-38-5	[H][C@]1(CCCN1)C(O)(C1=CC=C(C=C1)C(F)(F)C(F)(F)C(F)(F)C(F)(F)C(F)(F)C(F)(F)F)C1=CC=C(C=C1)C(F)(F)C(F)(F)C(F)(F)C(F)(F)C(F)(F)C(F)(F)F		-9.52	0.66	3.45	P	OUT
QL00039	2-C-(Heptafluoropropyl)-α-D-ribofuranose	467220-81-1	[H][C@]1(CO)O[C@]([H])(O)[C@](O)([C@]1([H])O)C(F)(F)C(F)(F)C(F)(F)F		-6.67	0.65	2.14	P	IN
QL00040	(17β)-3-[(Trimethylsilyl)oxy]estra-1,3,5(10)-trien-17-ol perfluorobutanoate	120623-45-2	[H][C@@]1(CC[C@@]2([H])[C@]3([H])CCC4=CC(O[Si](C)(C)C)=CC=C4[C@@]3([H])CC[C@]12C)OC(=O)C(F)(F)C(F)(F)C(F)(F)F		-8.25	0.64	3.13	P	OUT
QL00041	2-C-(Nonafluorobutyl)-β-L-ribofuranose	467220-84-4	[H][C@@]1(CO)O[C@]([H])(O)[C@@](O)([C@@]1([H])O)C(F)(F)C(F)(F)C(F)(F)C(F)(F)F		-6.99	0.66	2.21	P	IN
QL00042	(1R,4R)-3-(Heptafluorobutyryl)-camphor	51800-99-8	[H][C@@]12CC[C@@](C)(C(=O)C1C(=O)C(F)(F)C(F)(F)C(F)(F)F)C2(C)C		-7.12	0.67	2.10	P	IN
QL00043	Ammonium perfluorooctanoate	3825-26-1	[H][N]([H])([H])([H])OC(=O)C(F)(F)C(F)(F)C(F)(F)C(F)(F)C(F)(F)C(F)(F)C(F)(F)F		-7.83	0.67	2.45	P	IN
QL00044	Ammonium perfluorohexanoate	21615-47-4	[H][N]([H])([H])([H])OC(=O)C(F)(F)C(F)(F)C(F)(F)C(F)(F)C(F)(F)F		-7.06	0.66	2.17	P	IN
QL00045	WG-III	4089-61-6	[H][N+]([H])([H])[H].[H]N([H])S(=O)(=O)C(F)(F)C(F)(F)OC(F)(C(F)(F)F)C(F)(F)OC(F)(C([O-])=O)C(F)(F)F		-7.44	0.65	2.53	P	IN
QL00046	Ammonium bis(N-ethyl-2-perfluorooctylsulfonaminoethyl)phosphate	30381-98-7	[H][N+]([H])([H])[H].CCN(CCOP([O-])(=O)OCCN(CC)S(=O)(=O)C(F)(F)C(F)(F)C(F)(F)C(F)(F)C(F)(F)C(F)(F)C(F)(F)C(F)(F)F)S(=O)(=O)C(F)(F)C(F)(F)C(F)(F)C(F)(F)C(F)(F)C(F)(F)C(F)(F)C(F)(F)F		-10.15	0.66	3.81	P	OUT
QL00047	Ammonium perfluoro-9-(methyl)decanoate	3658-63-7	[H][NH+]([H])[H].[O-]C(=O)C(F)(F)C(F)(F)C(F)(F)C(F)(F)C(F)(F)C(F)(F)C(F)(F)C(F)(C(F)(F)F)C(F)(F)F		-7.98	0.67	2.61	P	IN
QL00048	Ammonium perfluoroundecanoate	4234-23-5	[H][NH+]([H])[H].[O-]C(=O)C(F)(F)C(F)(F)C(F)(F)C(F)(F)C(F)(F)C(F)(F)C(F)(F)C(F)(F)C(F)(F)C(F)(F)F		-8.40	0.67	2.80	P	IN
QL00049	Ammonium perfluoroheptanoate	6130-43-4	[H][NH+]([H])[H].[O-]C(=O)C(F)(F)C(F)(F)C(F)(F)C(F)(F)C(F)(F)C(F)(F)F		-7.01	0.65	2.32	P	IN
QL00050	Glycine, N-[(heptadecafluorooctyl)sulfonyl]-N-methyl-, chromium complex	97298-47-0	[H][O-]1[Cr++]([Cl-])([Cl-])[O-]C(CN(C)S(=O)(=O)C(F)(F)C(F)(F)C(F)(F)C(F)(F)C(F)(F)C(F)(F)C(F)(F)C(F)(F)F)=[O]/[Cr++]([Cl-])([Cl-])[O-]([H])[Cr++]1([Cl-])[Cl-]		-9.09	0.67	3.08	P	OUT
QL00051	Diaquatetrachloro[mu-[N-ethyl-N-[(perfluorooctyl)sulfonyl]glycinato-O1O1']]-mu-hydroxybis[2-methylpropanol]dichromium	68891-96-3	[H][O]([H])[Cr+3]1([Cl-])([Cl-])([O-]C(CN(CC)S(=O)(=O)C(F)(F)C(F)(F)C(F)(F)C(F)(F)C(F)(F)C(F)(F)C(F)(F)C(F)(F)F)=[O][Cr+3]([Cl-])([Cl-])([O]([H])[H])([O]([H])CC(C)C)[O-]1[H])[O]([H])CC(C)C		-9.15	0.67	3.16	P	OUT
QL00052	Diaquatetrachloro(mu-(N-ethyl-N-((perfluorobutyl)sulfonyl)glycinato-O1O1'))-mu-hydroxybis(2-propanol)dichromium	68900-97-0	[H][O]([H])[Cr+3]1([Cl-])([Cl-])([OH]C(C)C)[O]=C(CN(CC)S(=O)(=O)C(F)(F)C(F)(F)C(F)(F)C(F)(F)F)O[Cr+3]([Cl-])([Cl-])([O]([H])[H])([O]([H])C(C)C)[O]1[H]		-7.98	0.67	2.62	P	IN
QL00053	Diaquatetrachloro(mu-(N-ethyl-N-((pentadecafluoroheptyl)sulfonyl)glycinato-O1O1'))-mu-hydroxybis(2-propanol) chromium	68891-97-4	[H][O]([H])[Cr+3]1([Cl-])([Cl-])(OC(CN(CC)S(=O)(=O)C(F)(F)C(F)(F)C(F)(F)C(F)(F)C(F)(F)C(F)(F)C(F)(F)F)=[O][Cr+3]([Cl-])([Cl-])([O]([H])[H])([O]([H])C(C)C)[O-]1[H])[O]([H])C(C)C		-8.88	0.67	2.99	P	OUT
QL00054	Diaquatetrachloro(mu-(N-ethyl-N-((perfluorohexyl)sulfonyl)glycinato-O1O1'))-mu-hydroxybis(2-propanol)dichromium	68891-98-5	[H][O]([H])[Cr+3]1([Cl-])([Cl-])(OC(CN(CC)S(=O)(=O)C(F)(F)C(F)(F)C(F)(F)C(F)(F)C(F)(F)C(F)(F)F)=[O][Cr+3]([Cl-])([Cl-])([O]([H])[H])([O]([H])C(C)C)[O]1[H])[O]([H])C(C)C		-8.57	0.67	2.86	P	IN
QL00055	Diaquatetrachloro(mu-(N-ethyl-N-((perfluoropentyl)sulfonyl)glycinato-O1O1'))-mu-hydroxybis(2-propanol)dichromium	68891-99-6	[H][O]([H])[Cr+3]1([Cl-])([Cl-])(OC(CN(CC)S(=O)(=O)C(F)(F)C(F)(F)C(F)(F)C(F)(F)C(F)(F)F)=[O][Cr+3]([Cl-])([Cl-])([O]([H])[H])([O]([H])C(C)C)[O-]1[H])[O]([H])C(C)C		-8.36	0.67	2.78	P	IN
QL00056	Bis(heneicosafluorodecyl)phosphinic acid	52299-27-1	[H]C([H])(C([H])([H])P(O)(=O)C([H])([H])C([H])([H])C(F)(F)C(F)(F)C(F)(F)C(F)(F)C(F)(F)C(F)(F)C(F)(F)C(F)(F)F)C(F)(F)C(F)(F)C(F)(F)C(F)(F)C(F)(F)C(F)(F)C(F)(F)C(F)(F)F		-10.15	0.69	3.45	P	OUT
QL00057	Tris(2-(perfluorododecyl)ethyl)phosphate	393098-42-5	[H]C([H])(OP(=O)(OC([H])([H])C([H])([H])C(F)(F)C(F)(F)C(F)(F)C(F)(F)C(F)(F)C(F)(F)C(F)(F)C(F)(F)C(F)(F)C(F)(F)C(F)(F)C(F)(F)F)OC([H])([H])C([H])([H])C(F)(F)C(F)(F)C(F)(F)C(F)(F)C(F)(F)C(F)(F)C(F)(F)C(F)(F)C(F)(F)C(F)(F)C(F)(F)C(F)(F)F)C([H])([H])C(F)(F)C(F)(F)C(F)(F)C(F)(F)C(F)(F)C(F)(F)C(F)(F)C(F)(F)C(F)(F)C(F)(F)C(F)(F)C(F)(F)F		-11.15	0.69	3.94	P	OUT
QL00058	2-Chloro-2-propenoic acid 3,3,4,4,5,5,6,6,6-nonafluorohexyl ester	701909-41-3	[H]C([H])=C(Cl)C(=O)OCCC(F)(F)C(F)(F)C(F)(F)C(F)(F)F		-7.06	0.65	2.43	P	IN

QL00059	1,4-bis(Perfluorooctyl) fumarate	934166-50-4	[H]C(=C([H])C(=O)OC(F)(F)C(F)(F)C(F)(F)C(F)(F)C(F)(F)C(F)(F)C(F)(F)C(F)(F)F)C(=O)OC(F)(F)C(F)(F)C(F)(F)C(F)(F)C(F)(F)C(F)(F)C(F)(F)C(F)(F)F		-10.01	0.68	3.48	P	OUT
QL00060	1,4-bis(1H,1H,2H,2H-Perfluorooctyl) fumarate	24120-19-2	[H]C(=C([H])C(=O)OCCC(F)(F)C(F)(F)C(F)(F)C(F)(F)C(F)(F)C(F)(F)F)C(=O)OCCC(F)(F)C(F)(F)C(F)(F)C(F)(F)C(F)(F)C(F)(F)F		-9.43	0.66	3.42	P	OUT
QL00061	1,4-bis(Perfluorooctyl) maleate	234096-30-1	[H]C(=C(/[H])C(=O)OC(F)(F)C(F)(F)C(F)(F)C(F)(F)C(F)(F)C(F)(F)C(F)(F)C(F)(F)F)C(=O)OC(F)(F)C(F)(F)C(F)(F)C(F)(F)C(F)(F)C(F)(F)C(F)(F)C(F)(F)F		-10.01	0.68	3.48	P	OUT
QL00062	mono(Perfluorobutyl) maleate	229957-05-5	[H]C(=C(/[H])C(=O)OC(F)(F)C(F)(F)C(F)(F)C(F)(F)F)C([O-])=O		-6.92	0.64	2.43	P	IN
QL00063	[N(E),S(S)]-N-(2-Fluorophenyl)methyleneperfluorobutanesulfinamide	1612778-40-1	[H]C(=N/[S@@](=O)C(F)(F)C(F)(F)C(F)(F)C(F)(F)F)C1=C(F)C=CC=C1		-7.01	0.64	2.51	P	IN
QL00064	[N(E),S(S)]-N-(3-Fluorophenyl)methyleneperfluorobutanesulfinamide	1612778-41-2	[H]C(=N/[S@@](=O)C(F)(F)C(F)(F)C(F)(F)C(F)(F)F)C1=CC(F)=CC=C1		-7.03	0.64	2.52	P	IN
QL00065	[N(E),S(S)]-N-(4-methylphenyl)methyleneperfluorobutanesulfinamide	1612778-39-8	[H]C(=N/[S@@](=O)C(F)(F)C(F)(F)C(F)(F)C(F)(F)F)C1=CC=C(C)C=C1		-7.37	0.64	2.67	P	OUT
QL00066	[N(E),S(S)]-N-(4-nitrophenyl)methyleneperfluorobutanesulfinamide	1612778-37-6	[H]C(=N/[S@@](=O)C(F)(F)C(F)(F)C(F)(F)C(F)(F)F)C1=CC=C(C=C1)[N+]([O-])=O		-7.44	0.64	2.73	P	OUT
QL00067	[N(E),S(S)]-N-(4-chlorophenyl)methyleneperfluorobutanesulfinamide	1612778-35-4	[H]C(=N/[S@@](=O)C(F)(F)C(F)(F)C(F)(F)C(F)(F)F)C1=CC=C(Cl)C=C1		-7.37	0.64	2.67	P	OUT
QL00068	[N(E),S(S)]-N-phenylmethyleneperfluorobutanesulfinamide	1612778-34-3	[H]C(=N/[S@@](=O)C(F)(F)C(F)(F)C(F)(F)C(F)(F)F)C1=CC=CC=C1		-6.93	0.63	2.52	P	OUT
QL00069	Ethyl (2E)-2-[(S)-perfluorobutylsulfinylimino]acetate	1612778-42-3	[H]C(=N/S(=O)C(F)(F)C(F)(F)C(F)(F)C(F)(F)F)C(=O)OCC		-6.86	0.63	2.53	P	OUT
QL00070	(3β,5α)-Cholestan-3-ol 2,3,3,4,4,5,5,6,6,7,7,8,8,8-tetradecafluorooctanoate	123613-18-3	[H]C(C)(CCCC(C)C)[C@@]1([H])CC[C@@]2([H])[C@]3([H])CC[C@@]4([H])C[C@]([H])(CC[C@]4(C)[C@@]3([H])CC[C@]12C)OC(=O)C([H])(F)C(F)(F)C(F)(F)C(F)(F)C(F)(F)C(F)(F)C(F)(F)F		-9.36	0.64	3.67	P	OUT
QL00071	(3β,7α)-25,26,26,26,27,27,27-Heptafluorocholest-5-ene-3,7-diol	240129-40-2	[H]C(C)(CCCC(F)(C(F)(F)F)C(F)(F)F)[C@@]1([H])CC[C@@]2([H])[C@]3([H])[C@]([H])(O)C=C4C[C@@]([H])(O)CC[C@]4(C)[C@@]3([H])CC[C@]12C		-7.99	0.64	3.04	P	OUT
QL00073	(3β,7β)-25,26,26,26,27,27,27-Heptafluorocholest-5-ene-3,7-diol	240129-42-4	[H]C(C)(CCCC(F)(C(F)(F)F)C(F)(F)F)[C@@]1([H])CC[C@@]2([H])[C@]3([H])[C@@]([H])(O)C=C4C[C@@]([H])(O)CC[C@]4(C)[C@@]3([H])CC[C@]12C		-7.99	0.64	3.04	P	OUT
QL00074	(3β)-25,26,26,26,27,27,27-Heptafluoro-3-hydroxycholest-5-en-7-one	215094-37-4	[H]C(C)(CCCC(F)(C(F)(F)F)C(F)(F)F)[C@@]1([H])CC[C@@]2([H])[C@]3([H])C(=O)C=C4C[C@@]([H])(O)CC[C@]4(C)[C@@]3([H])CC[C@]12C		-7.99	0.64	3.04	P	OUT
QL00075	(3β,5α,6α)-5,6-Epoxy-25,26,26,26,27,27,27-heptafluorocholestan-3-ol	240129-21-9	[H]C(C)(CCCC(F)(C(F)(F)F)C(F)(F)F)[C@@]1([H])CC[C@@]2([H])[C@]3([H])C[C@]4([H])O[C@@]44C[C@@]([H])(O)CC[C@]4(C)[C@@]3([H])CC[C@]12C		-8.15	0.64	3.03	P	OUT
QL00077	(3β,5β,6β)-5,6-Epoxy-25,26,26,26,27,27,27-heptafluorocholestan-3-ol	240129-24-2	[H]C(C)(CCCC(F)(C(F)(F)F)C(F)(F)F)[C@@]1([H])CC[C@@]2([H])[C@]3([H])C[C@@]4([H])O[C@]44C[C@@]([H])(O)CC[C@]4(C)[C@@]3([H])CC[C@]12C		-8.15	0.64	3.03	P	OUT
QL00078	(3β)-25,26,26,26,27,27,27-Heptafluoro-cholest-5-en-3-ol	153463-21-9	[H]C(C)(CCCC(F)(C(F)(F)F)C(F)(F)F)[C@@]1([H])CC[C@@]2([H])[C@]3([H])CC=C4C[C@@]([H])(O)CC[C@]4(C)[C@@]3([H])CC[C@]12C		-7.94	0.63	3.03	P	OUT
QL00079	(Z)-(Perfluorodecyl)ethyl 9-octadecenoate	125768-41-4	[H]C(CCCCCCCC)=C(/[H])CCCCCCCC(=O)OCCC(F)(F)C(F)(F)C(F)(F)C(F)(F)C(F)(F)C(F)(F)C(F)(F)C(F)(F)C(F)(F)C(F)(F)F		-9.27	0.62	3.94	P	OUT
QL00080	(Z)-(Perfluorooctyl)ethyl 9-octadecenoate	167289-73-8	[H]C(CCCCCCCC)=C(/[H])CCCCCCCC(=O)OCCC(F)(F)C(F)(F)C(F)(F)C(F)(F)C(F)(F)C(F)(F)C(F)(F)C(F)(F)F		-8.77	0.61	3.84	P	OUT
QL00081	(Z)-(Perfluorohexyl)ethyl 9-octadecenoate	220237-47-8	[H]C(CCCCCCCC)=C(/[H])CCCCCCCC(=O)OCCC(F)(F)C(F)(F)C(F)(F)C(F)(F)C(F)(F)C(F)(F)F		-8.19	0.59	3.73	P	OUT
QL00082	(αR,7α)-3-Hydroxy-α-((perfluorobutyl)propyl)-17-oxo-estra-1,3,5(10)-triene-7-decanoic acid	615563-01-4	[H]C(CCCCCCCC[C@]1([H])CC2=CC(O)=CC=C2[C@@]2([H])CC[C@]3(C)C(=O)CC[C@@]3([H])[C@]12[H])(CCCC(F)(F)C(F)(F)C(F)(F)C(F)(F)F)C(O)=O		-7.94	0.61	3.31	P	OUT
QL00083	H-substitued ,ether-substituted perfluoroalkyl (linear)sulfonic acids, n=8		[H]C(F)(F)C(F)(F)C(F)(F)C(F)(F)C(F)(F)C(F)(F)C(F)(F)C(F)(F)OC(F)(F)C(F)(F)S(=O)(O)=O		-8.72	0.68	2.74	P	IN
QL00084	H-substitued ,ether-substituted perfluoroalkyl (linear)sulfonic acids, n=6		[H]C(F)(F)C(F)(F)C(F)(F)C(F)(F)C(F)(F)C(F)(F)OC(F)(F)C(F)(F)S(=O)(O)=O		-8.17	0.68	2.51	P	IN
QL00085	H-substitued ,ether-substituted perfluoroalkyl (linear)sulfonic acids, n=5		[H]C(F)(F)C(F)(F)C(F)(F)C(F)(F)C(F)(F)OC(F)(F)C(F)(F)S(=O)(O)=O		-7.99	0.68	2.45	P	IN
QL00086	H-substituted perfluoroalkyl (linear) sulfonic acids_i, n=3		[H]C(F)(F)C(F)(F)C(F)(F)S(=O)(O)=O		-5.76	0.67	1.46	P	IN

QL00087	multiple H-substituted perfluoroalkyl (linear) carboxylic acids, n=1		[H]C(F)(F)CC(F)(F)C(O)=O		-4.21	0.61	1.52	P	OUT
QL00088	multiple H-substituted perfluoroalkyl (linear) carboxylic acids, n=2		[H]C(F)(F)CC(F)(F)CC(F)(F)C(O)=O		-5.36	0.62	1.95	P	OUT
QL00089	multiple H-substituted perfluoroalkyl (linear) carboxylic acids, n=3		[H]C(F)(F)CC(F)(F)CC(F)(F)CC(F)(F)C(O)=O		-6.18	0.62	2.28	P	OUT
QL00090	multiple H-substituted perfluoroalkyl (linear) carboxylic acids, n=4		[H]C(F)(F)CC(F)(F)CC(F)(F)CC(F)(F)CC(F)(F)C(O)=O		-6.82	0.63	2.55	P	OUT
QL00091	multiple H-substituted perfluoroalkyl (linear) carboxylic acids, n=5		[H]C(F)(F)CC(F)(F)CC(F)(F)CC(F)(F)CC(F)(F)CC(F)(F)C(O)=O		-7.34	0.63	2.78	P	OUT
QL00092	multiple H-substituted perfluoroalkyl (linear) carboxylic acids, n=6		[H]C(F)(F)CC(F)(F)CC(F)(F)CC(F)(F)CC(F)(F)CC(F)(F)CC(F)(F)C(O)=O		-7.78	0.63	2.97	P	OUT
QL00093	multiple H-substituted perfluoroalkyl (linear) carboxylic acids, n=7		[H]C(F)(F)CC(F)(F)CC(F)(F)CC(F)(F)CC(F)(F)CC(F)(F)CC(F)(F)CC(F)(F)C(O)=O		-8.16	0.63	3.15	P	OUT
QL00094	multiple H-substituted perfluoroalkyl (linear) carboxylic acids, n=8		[H]C(F)(F)CC(F)(F)CC(F)(F)CC(F)(F)CC(F)(F)CC(F)(F)CC(F)(F)CC(F)(F)CC(F)(F)C(O)=O		-8.50	0.64	3.30	P	OUT
QL00095	multiple H-substituted perfluoroalkyl (linear) carboxylic acids, n=9		[H]C(F)(F)CC(F)(F)CC(F)(F)CC(F)(F)CC(F)(F)CC(F)(F)CC(F)(F)CC(F)(F)CC(F)(F)CC(F)(F)C(O)=O		-8.80	0.64	3.44	P	OUT
QL00096	(S)-(Perfluoropropyl)phenylmethanol	59153-45-6	[H]C(O)(C1=CC=CC=C1)C(F)(F)C(F)(F)C(F)(F)F		-6.23	0.63	2.17	P	IN
QL00097	D-Galactose-6-[5-[(3(-perfluorooctyl)-1-nonylpropyl)oxy]pentylhydrogen phosphate]	172916-27-7	[H]C(O)(COP([O-])(=O)OCCCCCOC([H])(CCCCCCCCC)CCC(F)(F)C(F)(F)C(F)(F)C(F)(F)C(F)(F)C(F)(F)C(F)(F)C(F)(F)F)C([H])(O)C([H])(O)C([H])(O)C=O		-8.64	0.61	3.69	P	OUT
QL00098	1H-Perfluoro-3,3-bis(trifluoromethyl)hexyne	261503-44-0	[H]C#CC(C(F)(F)F)(C(F)(F)F)C(F)(F)C(F)(F)C(F)(F)F		-6.28	0.67	1.69	P	IN
QL00099	Bicyclo[2.2.1]hept-2-ene, 5,5,6-trifluoro-6-(heptafluoropropoxy)-	154717-36-9	[H]C12CC([H])(C=C1)C(F)(OC(F)(F)C(F)(F)C(F)(F)F)C2(F)F		-6.95	0.67	2.03	P	IN
QL00100	3,3,4-Trifluoro-4-(heptafluoropropoxy)tricyclo[4.2.1.0~2,5~]non-7-ene	262617-19-6	[H]C12CC([H])(C=C1)C1C2C(F)(F)C1(F)OC(F)(F)C(F)(F)C(F)(F)F		-7.24	0.67	2.21	P	IN
QL00101	5-(Nonafluorobutyl)bicyclo[2.2.1]hept-2-ene	118777-97-2	[H]C12CC(C([H])(C1)C=C2)C(F)(F)C(F)(F)C(F)(F)C(F)(F)F		-6.96	0.66	2.17	P	IN
QL00102	Sulfonamides, C4-8-alkane, perfluoro, N-ethyl-N-(hydroxyethyl)-, polymer with 1,1'-methylenebis[4-isocyanatobenzene] and polymethylenepolyphenylene isocyanate, 2-ethylhexyl esters, Me Et ketone oxime-blocked	178535-22-3	[H]c1cc(C([H])([H])c2ccc(N([H])C(=O)OCCN(CC)S(=O)(=O)C(F)(F)C(F)(F)C(F)(F)C(F)(F)C(F)(F)C(F)(F)C(F)(F)C(F)(F)F)cc2)ccc1N([H])C(=O)ON=C(C)CC.[H]N(C(=O)OCC([H])(CC)CCCC)c1ccc(Cc2ccc(N([H])C(=O)ON=C(C)CC)cc2)cc1		-9.33	0.60	4.22	P	OUT
QL00103	(Perfluoro-n-octyl)ethane	77117-48-7	[H]CCC(F)(F)C(F)(F)C(F)(F)C(F)(F)C(F)(F)C(F)(F)C(F)(F)C(F)(F)F		-8.06	0.68	2.44	P	IN
QL00104	4'-[2-(2,4-Di-tert-pentylphenoxy)butyramido]perfluoro-2'-hydroxybutyranilide	346-10-1	[H]N(C(=O)C(CC)OC1=C(C=C(C=C1)C(C)(C)CC)C(C)(C)CC)C1=CC(O)=C(C=C1)N([H])C(=O)C(F)(F)C(F)(F)C(F)(F)F		-8.19	0.62	3.35	P	OUT
QL00105	N-(3,4-Dichlorophenyl)-2,2,3,3,4,4,4-heptafluorobutanamide	106376-37-8	[H]N(C(=O)C(F)(F)C(F)(F)C(F)(F)F)C1=CC(Cl)=C(Cl)C=C1		-6.92	0.64	2.43	P	IN
QL00106	N-(3,5-Dichlorophenyl)-2,2,3,3,4,4,4-heptafluorobutanamide	106376-38-9	[H]N(C(=O)C(F)(F)C(F)(F)C(F)(F)F)C1=CC(Cl)=CC(Cl)=C1		-6.43	0.64	2.23	P	IN
QL00107	2-Propenoic acid, 2-methyl-, 2-[[[[5-[[[2-[ethyl[(heptadecafluorooctyl)sulfonyl]amino]ethoxy]carbonyl]amino]-2-methylphenyl]amino]carbonyl]oxy]propyl ester	68298-72-6	[H]N(C(=O)OCCN(CC)S(=O)(=O)C(F)(F)C(F)(F)C(F)(F)C(F)(F)C(F)(F)C(F)(F)C(F)(F)C(F)(F)F)C1=CC(N([H])C(=O)OC(C)COC(=O)C(C)=C)=C(C)C=C1		-9.34	0.63	3.74	P	OUT
QL00108	2-Propenoic acid, 2-methyl-, 2-[[[[5-[[[2-[ethyl[(pentadecafluoroheptyl)sulfonyl]amino]ethoxy]carbonyl]amino]-2-methylphenyl]amino]carbonyl]oxy]propyl ester	68298-73-7	[H]N(C(=O)OCCN(CC)S(=O)(=O)C(F)(F)C(F)(F)C(F)(F)C(F)(F)C(F)(F)C(F)(F)C(F)(F)F)C1=CC(N([H])C(=O)OC(C)COC(=O)C(C)=C)=C(C)C=C1		-9.18	0.63	3.71	P	OUT
QL00109	2-Propenoic acid, 2-methyl-, 2-[[[[5-[[[2-[ethyl[(tridecafluorohexyl)sulfonyl]amino]ethoxy]carbonyl]amino]-2-methylphenyl]amino]carbonyl]oxy]propyl ester	68298-74-8	[H]N(C(=O)OCCN(CC)S(=O)(=O)C(F)(F)C(F)(F)C(F)(F)C(F)(F)C(F)(F)C(F)(F)F)C1=CC(N([H])C(=O)OC(C)COC(=O)C(C)=C)=C(C)C=C1		-8.93	0.63	3.65	P	OUT
QL00110	2-Propenoic acid, 2-methyl-, 2-[[[[5-[[[2-[ethyl[(undecafluoropentyl)sulfonyl]amino]ethoxy]carbonyl]amino]-2-methylphenyl]amino]carbonyl]oxy]propyl ester	68298-75-9	[H]N(C(=O)OCCN(CC)S(=O)(=O)C(F)(F)C(F)(F)C(F)(F)C(F)(F)C(F)(F)F)C1=CC(N([H])C(=O)OC(C)COC(=O)C(C)=C)=C(C)C=C1		-8.74	0.62	3.61	P	OUT
QL00111	2-Propenoic acid, 2-methyl-, 2-[[[[5-[[[2-[ethyl[(nonafluorobutyl)sulfonyl]amino]ethoxy]carbonyl]amino]-2-methylphenyl]amino]carbonyl]oxy]propyl ester	68298-76-0	[H]N(C(=O)OCCN(CC)S(=O)(=O)C(F)(F)C(F)(F)C(F)(F)C(F)(F)F)C1=CC(N([H])C(=O)OC(C)COC(=O)C(C)=C)=C(C)C=C1		-8.43	0.62	3.53	P	OUT
QL00112	(2-Carboxylatoethyl)dimethyl(((perfluorododecyl)-2-hydroxypropyl)amino)propylammonium	93776-12-6	[H]N(CCC[N+]([H])(CC)CCC([O-])=O)CC(O)CC(F)(F)C(F)(F)C(F)(F)C(F)(F)C(F)(F)C(F)(F)C(F)(F)C(F)(F)C(F)(F)C(F)(F)C(F)(F)C(F)(F)F		-9.57	0.65	3.64	P	OUT

QL00113	Bis(1,1,2,2,3,3,4,4,4-nonafluoro-1-butanesulfonyl)imide	39847-39-7	[H]N(S(=O)(=O)C(F)(F)C(F)(F)C(F)(F)C(F)(F)F)S(=O)(=O)C(F)(F)C(F)(F)C(F)(F)C(F)(F)F		-8.64	0.69	2.63	P	IN
QL00114	Heptafluorobutanoic acid anhydride, compd. with dihexyl phosphonate (21)	110086-60-7	[H]P(=O)(OCCCCCC)OCCCCCC.FC(F)(F)C(F)(F)C(F)(F)C(=O)OC(=O)C(F)(F)C(F)(F)C(F)(F)F.FC(F)(F)C(F)(F)C(F)(F)C(=O)OC(=O)C(F)(F)C(F)(F)C(F)(F)F		-7.43	0.63	2.84	P	OUT
QL00115	Holmium, tris(6,6,7,7,8,8,8-heptafluoro-2,2-dimethyl-3,5-octanedionato-βO3,βO5)-	18323-97-2	[Ho+3].CC(C)(C)C([O-])=CC(=O)C(F)(F)C(F)(F)C(F)(F)F.CC(C)(C)C([O-])=CC(=O)C(F)(F)C(F)(F)C(F)(F)F.CC(C)(C)C([O-])=CC(=O)C(F)(F)C(F)(F)C(F)(F)F		-6.81	0.65	2.24	P	IN
QL00116	3-((Perfluorooctylsulphonyl)amino)-N,N,N-trimethylpropanaminium iodide	68310-75-8	[I-].[H]N([H])[H].[H]N(CCC[N+](C)(C)C)S(=O)(=O)C(F)(F)C(F)(F)C(F)(F)C(F)(F)C(F)(F)C(F)(F)C(F)(F)C(F)(F)F		-8.07	0.64	3.08	P	OUT
QL00117	Trimethyl-3-((perfluorohexyl)ethylsulfonyl)aminopropylammonium iodide	94088-80-9	[I-].[H]N(CCC[N+](C)(C)C)S(=O)(=O)CCC(F)(F)C(F)(F)C(F)(F)C(F)(F)C(F)(F)C(F)(F)F		-7.99	0.64	3.01	P	OUT
QL00118	[4,4,5,5,6,6,7,7,8,8,9,9,10,11,11,11-hexadecafluoro-2-hydroxy-10-(trifluoromethyl)undecyl]trimethylammonium iodide	80909-29-1	[I-].C[N+](C)(C)CC(O)CC(F)(F)C(F)(F)C(F)(F)C(F)(F)C(F)(F)C(F)(F)C(F)(C(F)(F)F)C(F)(F)F		-8.07	0.66	2.80	P	IN
QL00119	((Perfluorooctyl)-2-hydroxypropyl)trimethylammonium iodide	92071-84-6	[I-].C[N+](C)(C)CC(O)CC(F)(F)C(F)(F)C(F)(F)C(F)(F)C(F)(F)C(F)(F)C(F)(F)C(F)(F)F		-8.11	0.65	2.85	P	IN
QL00120	1-(3-(4-((Heptadecafluorononyl)oxy)-benzamido)propyl)-N,N,N-trimethylammonium iodide	59493-72-0	[I-].C[N+](C)(C)CCCNC(=O)C1=CC=C(OC(F)(F)C(F)(F)C(F)(F)C(F)(F)C(F)(F)C(F)(F)C(F)(F)C(F)(F)C(F)(F)F)C=C1		-9.00	0.64	3.49	P	OUT
QL00121	3-(4-{[1,1,1,4,5,5,5-Heptafluoro-3-(1,1,1,2,3,3,3-heptafluoropropan-2-yl)-4-(trifluoromethyl)pent-2-en-2-yl]oxy}benzamido)-N,N,N-trimethylpropan-1-aminium iodide	170778-67-3	[I-].C[N+](C)(C)CCCNC(=O)c1ccc(OC(=C(C(F)(C(F)(F)F)C(F)(F)F)C(F)(C(F)(F)F)C(F)(F)F)C(F)(F)F)cc1		-7.39	0.63	2.87	P	OUT
QL00122	N,N,N-Trimethyl-3-[(1,1,2,2,3,3,4,4,5,5,6,6,7,7,8-pentadecafluoro-8-oxooctyl)amino]propan-1-aminium iodide	335-90-0	[I-].C[N+](C)(C)CCCNC(F)(F)C(F)(F)C(F)(F)C(F)(F)C(F)(F)C(F)(F)C(F)(F)C(F)=O		-8.10	0.64	3.01	P	OUT
QL00123	Perfluorooctanesulfonamido ammonium iodide	1652-63-7	[I-].C[N+](C)(C)CCCNS(=O)(=O)C(F)(F)C(F)(F)C(F)(F)C(F)(F)C(F)(F)C(F)(F)C(F)(F)C(F)(F)F		-8.46	0.65	3.03	P	IN
QL00124	Trimethyl-3-[[(pentadecafluoroheptyl)sulphonyl]amino]propylammonium iodide	67584-58-1	[I-].C[N+](C)(C)CCCNS(=O)(=O)C(F)(F)C(F)(F)C(F)(F)C(F)(F)C(F)(F)C(F)(F)C(F)(F)F		-8.28	0.65	3.00	P	IN
QL00125	Trimethyl-3-[[(tridecafluorohexyl)sulphonyl]amino]propylammonium iodide	68957-58-4	[I-].C[N+](C)(C)CCCNS(=O)(=O)C(F)(F)C(F)(F)C(F)(F)C(F)(F)C(F)(F)C(F)(F)F		-7.86	0.64	2.86	P	IN
QL00126	Trimethyl-3-[[(undecafluoropentyl)sulphonyl]amino]propylammonium iodide	68957-57-3	[I-].C[N+](C)(C)CCCNS(=O)(=O)C(F)(F)C(F)(F)C(F)(F)C(F)(F)C(F)(F)F		-7.64	0.64	2.82	P	OUT
QL00127	Trimethyl-3-[[(nonafluorobutyl)sulphonyl]amino]propylammonium iodide	67939-95-1	[I-].C[N+](C)(C)CCCNS(=O)(=O)C(F)(F)C(F)(F)C(F)(F)C(F)(F)F		-7.12	0.63	2.66	P	OUT
QL00128	Trimethyl{3-{4-[perfluoro-4-methyl-3-(propan-2-yl)pent-2-en-2-oxy]phenylsulfonylamino}propyl}ammonium iodide	158658-62-9	[I-].C[N+](C)(C)CCCNS(=O)(=O)C1=CC=C(OC(=C(C(F)(C(F)(F)F)C(F)(F)F)C(F)(C(F)(F)F)C(F)(F)F)C(F)(F)F)C=C1		-7.59	0.63	2.89	P	OUT
QL00129	3-[[[4-[(Heptadecafluorononenyl)oxy]phenyl]sulfonyl]amino]-N,N,N-trimethyl-1-propanaminium, iodide	59493-70-8	[I-].C[N+](C)(C)CCCNS(=O)(=O)C1=CC=C(OC(F)=C(F)C(F)(F)C(F)(F)C(F)(F)C(F)(F)C(F)(F)C(F)(F)C(F)(F)F)C=C1		-8.85	0.64	3.48	P	OUT
QL00130	2-[[(Heptadecafluorooctyl)sulfonyl]amino]-N,N,N-trimethylethanaminium iodide	57765-32-9	[I-].C[N+](C)(C)CCNS(=O)(=O)C(F)(F)C(F)(F)C(F)(F)C(F)(F)C(F)(F)C(F)(F)C(F)(F)C(F)(F)F		-8.49	0.66	2.99	P	IN
QL00131	Bis(2-hydroxyethyl)methyl(1(-perfluoro-7-methyloctyl))-2-hydroxyprop-3-ylammonium iodide	94159-78-1	[I-].C[N+](CCO)(CCO)CC(O)CC(F)(F)C(F)(F)C(F)(F)C(F)(F)C(F)(F)C(F)(F)C(F)(C(F)(F)F)C(F)(F)F		-7.88	0.63	3.01	P	OUT
QL00132	Bis(2-hydroxyethyl)methyl(1-(perfluoro-9-methyldecyl))-2-hydroxyprop-3-ylammonium iodide	94159-77-0	[I-].C[N+](CCO)(CCO)CC(O)CC(F)(F)C(F)(F)C(F)(F)C(F)(F)C(F)(F)C(F)(F)C(F)(F)C(F)(F)C(F)(C(F)(F)F)C(F)(F)F		-8.44	0.64	3.18	P	OUT
QL00133	Bis(2-hydroxyethyl)methyl(1-(perfluoro-11-methyldodecyl))-2-hydroxyprop-3-ylammonium iodide	94159-76-9	[I-].C[N+](CCO)(CCO)CC(O)CC(F)(F)C(F)(F)C(F)(F)C(F)(F)C(F)(F)C(F)(F)C(F)(F)C(F)(F)C(F)(F)C(F)(F)C(F)(C(F)(F)F)C(F)(F)F		-8.91	0.65	3.33	P	OUT

QL00134	Bis(2-hydroxyethyl)methyl(3-(perfluorododecyl)-2-hydroxypropyl)ammonium iodide	93776-16-0	[I-].C[N+](CCO)(CCO)CC(O)CC(F)(F)C(F)(F)C(F)(F)C(F)(F)C(F)(F)C(F)(F)C(F)(F)C(F)(F)C(F)(F)C(F)(F)C(F)(F)C(F)(F)F		-8.91	0.65	3.35	P	OUT
QL00135	Bis(2-hydroxyethyl)methyl(3-(perfluorodecyl)-2-hydroxypropyl)ammonium iodide	93776-17-1	[I-].C[N+](CCO)(CCO)CC(O)CC(F)(F)C(F)(F)C(F)(F)C(F)(F)C(F)(F)C(F)(F)C(F)(F)C(F)(F)C(F)(F)C(F)(F)F		-8.43	0.64	3.20	P	OUT
QL00136	Bis(2-hydroxyethyl)methyl(3-(perfluorooctyl)-2-hydroxypropyl)ammonium iodide	93776-18-2	[I-].C[N+](CCO)(CCO)CC(O)CC(F)(F)C(F)(F)C(F)(F)C(F)(F)C(F)(F)C(F)(F)C(F)(F)C(F)(F)F		-7.85	0.63	3.04	P	OUT
QL00137	(3,3,4,4,5,5,6,6,7,7,8,8,9,9,10,10,10-heptadecafluorodecyl)bis(2-hydroxyethyl)methylammonium iodide	31841-41-5	[I-].C[N+](CCO)(CCO)CCC(F)(F)C(F)(F)C(F)(F)C(F)(F)C(F)(F)C(F)(F)C(F)(F)C(F)(F)F		-7.84	0.63	3.00	P	OUT
QL00138	2-[[(Heptadecafluorooctyl)sulfonyl]amino]-N,N,N-trimethyl-1-propanaminium iodide	75033-26-0	[I-].CC(C[N+](C)(C)C)NS(=O)(=O)C(F)(F)C(F)(F)C(F)(F)C(F)(F)C(F)(F)C(F)(F)C(F)(F)C(F)(F)F		-8.50	0.66	2.99	P	IN
QL00139	N,N-Diethyl-1,1,1,2,2,3,3,5,6,6,8,9,9,11-tetradecafluoro-N-methyl-12-oxo-5,8,11-tris(trifluoromethyl)-4,7,10-trioxa-13-azahexadecan-16-aminium iodide	84166-37-0	[I-].CC[N+](C)(CC)CCCNC(=O)C(F)(OC(F)(F)C(F)(OC(F)(F)C(F)(OC(F)(F)C(F)(F)C(F)(F)F)C(F)(F)F)C(F)(F)F)C(F)(F)F		-8.68	0.64	3.31	P	OUT
QL00140	Diethyl(3-(((heptadecafluoroisooctyl)sulphonyl)amino)propyl)methylammonium iodide	93894-72-5	[I-].CC[N+](C)(CC)CCCNS(=O)(=O)C(F)(F)C(F)(F)C(F)(F)C(F)(F)C(F)(F)C(F)(C(F)(F)F)C(F)(F)F		-8.08	0.64	3.00	P	OUT
QL00141	Diethyl(3-(((heptadecafluorooctyl)sulphonyl)amino)propyl)methylammonium iodide	93803-06-6	[I-].CC[N+](C)(CC)CCCNS(=O)(=O)C(F)(F)C(F)(F)C(F)(F)C(F)(F)C(F)(F)C(F)(F)C(F)(F)C(F)(F)F		-8.45	0.64	3.17	P	OUT
QL00142	4-[(Perfluorooctyl)sulfonyl]-1,1-dioctylpiperazinium iodide	113584-52-4	[I-].CCCCCCCC[N+]1(CCCCCCCC)CCN(CC1)S(=O)(=O)C(F)(F)C(F)(F)C(F)(F)C(F)(F)C(F)(F)C(F)(F)C(F)(F)C(F)(F)F		-8.19	0.61	3.51	P	OUT
QL00143	1-(3,3,4,4,5,5,6,6,7,7,8,8,9,9,10,10,10-heptadecafluorodecyl)pyridinium iodide	25935-14-2	[I-].FC(F)(F)C(F)(F)C(F)(F)C(F)(F)C(F)(F)C(F)(F)C(F)(F)C(F)(F)CC[n+]1ccccc1		-8.04	0.64	3.01	P	OUT
QL00144	Triphenyl(3,3,4,4,5,5,6,6,7,7,8,8,8-tridecafluorooctyl)phosphanium iodide	94190-73-5	[I-].FC(F)(F)C(F)(F)C(F)(F)C(F)(F)C(F)(F)C(F)(F)CC[P+](c1ccccc1)(c1ccccc1)c1ccccc1		-6.77	0.61	2.77	P	OUT
QL00145	(3,3,4,4,5,5,6,6,6-Nonafluorohexyl)(triphenyl)phosphanium iodide	94190-72-4	[I-].FC(F)(F)C(F)(F)C(F)(F)C(F)(F)CC[P+](C1=CC=CC=C1)(C1=CC=CC=C1)C1=CC=CC=C1		-5.81	0.59	2.50	P	OUT
QL00146	Diphenyliodanium nonafluorobutane-1-sulfonate	194999-82-1	[I+](C1=CC=CC=C1)C1=CC=CC=C1.[O-]S(=O)(=O)C(F)(F)C(F)(F)C(F)(F)C(F)(F)F		-6.19	0.62	2.28	P	OUT
QL00147	Potassium perfluoro-p-ethylcyclohexanesulfonate	335-24-0	[K].O=[S](=O)(O)C1(F)C(F)(F)C(F)(F)C(F)(C(F)(F)C1(F)F)C(F)(F)C(F)(F)F		-7.60	0.67	2.36	P	IN
QL00148	Pentapotassium 6,9,12-tris(carboxylatomethyl)-3-(1,1,2,2,3,3,4,4,5,5,6,6,7,7,8,8,8-heptadecafluorooctane-1-sulfonyl)-3,6,9,12,15-pentaazaheptadecane-1,17-dioate	155286-99-0	[K+].[K+].[K+].[K+].[K+].[O-]C(=O)CNCCN(CCN(CCN(CCN(CC([O-])=O)S(=O)(=O)C(F)(F)C(F)(F)C(F)(F)C(F)(F)C(F)(F)C(F)(F)C(F)(F)C(F)(F)F)CC([O-])=O)CC([O-])=O)CC([O-])=O		-7.70	0.57	3.75	P	OUT
QL00149	Potassium 2,3,3,3-tetrafluoro-2-(trifluoromethyl)propanoate	95091-35-3	[K+].[O-]C(=O)C(F)(C(F)(F)F)C(F)(F)F		-4.45	0.61	1.52	P	OUT
QL00150	Potassium 2,2,3,3,4,4,5,5,6,7,7,7-dodecafluoro-6-(trifluoromethyl)heptanoate	29457-73-6	[K+].[O-]C(=O)C(F)(F)C(F)(F)C(F)(F)C(F)(F)C(F)(C(F)(F)F)C(F)(F)F		-6.99	0.65	2.28	P	IN
QL00151	Potassium henicosafluoroundecanoate	30377-53-8	[K+].[O-]C(=O)C(F)(F)C(F)(F)C(F)(F)C(F)(F)C(F)(F)C(F)(F)C(F)(F)C(F)(F)C(F)(F)C(F)(F)F		-8.40	0.67	2.80	P	IN
QL00152	Potassium 2,2,3,3,4,4,5,5,6,6,7,7,8,8,9,9,10,10,11,11-icosafluoroundecanoate	307-71-1	[K+].[O-]C(=O)C(F)(F)C(F)(F)C(F)(F)C(F)(F)C(F)(F)C(F)(F)C(F)(F)C(F)(F)C(F)(F)C(F)F		-8.21	0.66	2.79	P	IN
QL00153	Potassium heptadecafluorononanoate	21049-38-7	[K+].[O-]C(=O)C(F)(F)C(F)(F)C(F)(F)C(F)(F)C(F)(F)C(F)(F)C(F)(F)C(F)(F)F		-7.79	0.66	2.58	P	IN
QL00154	Potassium perfluorooctanoate	2395-00-8	[K+].[O-]C(=O)C(F)(F)C(F)(F)C(F)(F)C(F)(F)C(F)(F)C(F)(F)C(F)(F)F		-7.29	0.66	2.39	P	IN
QL00155	Potassium tridecafluoroheptanoate	21049-36-5	[K+].[O-]C(=O)C(F)(F)C(F)(F)C(F)(F)C(F)(F)C(F)(F)C(F)(F)F		-7.01	0.65	2.32	P	IN
QL00156	Potassium undecafluorohexanoate	3109-94-2	[K+].[O-]C(=O)C(F)(F)C(F)(F)C(F)(F)C(F)(F)C(F)(F)F		-6.35	0.64	2.09	P	IN
QL00157	Potassium nonafluoropentanoate	336-23-2	[K+].[O-]C(=O)C(F)(F)C(F)(F)C(F)(F)C(F)(F)F		-5.94	0.63	2.02	P	IN
QL00158	Potassium heptafluorobutanoate	2966-54-3	[K+].[O-]C(=O)C(F)(F)C(F)(F)C(F)(F)F		-4.97	0.62	1.73	P	OUT
QL00159	Potassium 2,3,3,3-tetrafluoro-2-(heptafluoropropoxy)propionate	67118-55-2	[K+].[O-]C(=O)C(F)(OC(F)(F)C(F)(F)C(F)(F)F)C(F)(F)F		-6.08	0.64	2.03	P	IN

QL00160	Perfluoro-2-(3-methoxypropoxy)propanoic acid potassium salt	496805-64-2	[K+].[O-]C(=O)C(F)(OC(F)(F)C(F)(F)C(F)(F)OC(F)(F)F)C(F)(F)F		-6.66	0.64	2.34	P	IN
QL00161	Potassium 2,3,3,3-tetrafluoro-2-[1,1,2,3,3,3-hexafluoro-2-(heptafluoropropoxy)propoxy]propionate	67118-57-4	[K+].[O-]C(=O)C(F)(OC(F)(F)C(F)(OC(F)(F)C(F)(F)C(F)(F)F)C(F)(F)F)C(F)(F)F		-7.44	0.65	2.53	P	IN
QL00162	Potassium 2,3,4,5-tetrachloro-6-[[[3-[[(heptadecafluorooctyl)sulphonyl]oxy]phenyl]amino]carbonyl]benzoate	57589-85-2	[K+].[O-]C(=O)c1c(Cl)c(Cl)c(Cl)c(Cl)c1C(=O)Nc2cccc(OS(=O)(=O)C(F)(F)C(F)(F)C(F)(F)C(F)(F)C(F)(F)C(F)(F)C(F)(F)C(F)(F)F)c2		-8.83	0.64	3.40	P	OUT
QL00163	Potassium 2,3,4,5-tetrachloro-6-[[[3-[[(pentadecafluoroheptyl)sulphonyl]oxy]phenyl]amino]carbonyl]benzoate	68541-01-5	[K+].[O-]C(=O)c1c(Cl)c(Cl)c(Cl)c(Cl)c1C(=O)Nc2cccc(OS(=O)(=O)C(F)(F)C(F)(F)C(F)(F)C(F)(F)C(F)(F)C(F)(F)C(F)(F)F)c2		-8.56	0.64	3.31	P	OUT
QL00164	Potassium 2,3,4,5-tetrachloro-6-[[[3-[[(tridecafluorohexyl)sulphonyl]oxy]phenyl]amino]carbonyl]benzoate	68815-72-5	[K+].[O-]C(=O)c1c(Cl)c(Cl)c(Cl)c(Cl)c1C(=O)Nc2cccc(OS(=O)(=O)C(F)(F)C(F)(F)C(F)(F)C(F)(F)C(F)(F)C(F)(F)F)c2		-8.35	0.63	3.26	P	OUT
QL00165	Potassium 2,3,4,5-tetrachloro-6-[[[3-[[(undecafluoropentyl)sulphonyl]oxy]phenyl]amino]carbonyl]benzoate	68541-02-6	[K+].[O-]C(=O)c1c(Cl)c(Cl)c(Cl)c(Cl)c1C(=O)Nc2cccc(OS(=O)(=O)C(F)(F)C(F)(F)C(F)(F)C(F)(F)C(F)(F)F)c2		-8.02	0.63	3.16	P	OUT
QL00166	Potassium 2,3,4,5-tetrachloro-6-[[[3-[[(nonafluorobutyl)sulphonyl]oxy]phenyl]amino]carbonyl]benzoate	68568-54-7	[K+].[O-]C(=O)c1c(Cl)c(Cl)c(Cl)c(Cl)c1C(=O)Nc2cccc(OS(=O)(=O)C(F)(F)C(F)(F)C(F)(F)C(F)(F)F)c2		-7.72	0.62	3.08	P	OUT
QL00167	Potassium 4,4,5,5,6,6,7,7,8,8,9,9,10,10,11,11,11-heptadecafluoroundecanoate	83310-58-1	[K+].[O-]C(=O)CCC(F)(F)C(F)(F)C(F)(F)C(F)(F)C(F)(F)C(F)(F)C(F)(F)C(F)(F)F		-7.91	0.65	2.80	P	IN
QL00168	Potassium [(1,1,2,2,3,3,4,4,5,5,6,6,7,7,8,8,8-heptadecafluorooctane-1-sulfonyl)amino]acetate	75260-69-4	[K+].[O-]C(=O)CNS(=O)(=O)C(F)(F)C(F)(F)C(F)(F)C(F)(F)C(F)(F)C(F)(F)C(F)(F)C(F)(F)F		-8.12	0.65	2.85	P	IN
QL00169	Potassium 1,1,2,3,3,3-hexafluoro-2-(trifluoromethyl)propanesulphonate	93762-10-8	[K+].[O-]S(=O)(=O)C(F)(F)C(F)(C(F)(F)F)C(F)(F)F		-5.41	0.64	1.61	P	IN
QL00170	Potassium 1,1,2,2,3,3,4,4,5,5,6,6,7,7,8,8,9,9,10,10,11,11,12,12,12-pentacosafluorododecane-1-sulphonate	85187-17-3	[K+].[O-]S(=O)(=O)C(F)(F)C(F)(F)C(F)(F)C(F)(F)C(F)(F)C(F)(F)C(F)(F)C(F)(F)C(F)(F)C(F)(F)C(F)(F)C(F)(F)F		-9.04	0.68	2.99	P	OUT
QL00171	Potassium henicosafluorodecanesulphonate	2806-16-8	[K+].[O-]S(=O)(=O)C(F)(F)C(F)(F)C(F)(F)C(F)(F)C(F)(F)C(F)(F)C(F)(F)C(F)(F)C(F)(F)C(F)(F)F		-8.56	0.67	2.79	P	IN
QL00172	Perfluorononanesulfonate potassium	29359-39-5	[K+].[O-]S(=O)(=O)C(F)(F)C(F)(F)C(F)(F)C(F)(F)C(F)(F)C(F)(F)C(F)(F)C(F)(F)C(F)(F)F		-8.19	0.67	2.63	P	IN
QL00173	Potassium heptadecafluorooctane-1-sulphonate	2795-39-3	[K+].[O-]S(=O)(=O)C(F)(F)C(F)(F)C(F)(F)C(F)(F)C(F)(F)C(F)(F)C(F)(F)C(F)(F)F		-7.99	0.67	2.57	P	IN
QL00174	Potassium 1,1,2,2,3,3,4,4,5,5,6,6,7,7,7-pentadecafluoroheptane-1-sulphonate	60270-55-5	[K+].[O-]S(=O)(=O)C(F)(F)C(F)(F)C(F)(F)C(F)(F)C(F)(F)C(F)(F)C(F)(F)F		-7.52	0.67	2.38	P	IN
QL00175	PFHS-K	3871-99-6	[K+].[O-]S(=O)(=O)C(F)(F)C(F)(F)C(F)(F)C(F)(F)C(F)(F)C(F)(F)F		-7.26	0.66	2.31	P	IN
QL00176	Potassium perfluoropentanesulfonate	3872-25-1	[K+].[O-]S(=O)(=O)C(F)(F)C(F)(F)C(F)(F)C(F)(F)C(F)(F)F		-6.66	0.66	2.08	P	IN
QL00177	Potassium 1,1,2,2,3,3,4,4,4-nonafluorobutane-1-sulphonate	29420-49-3	[K+].[O-]S(=O)(=O)C(F)(F)C(F)(F)C(F)(F)C(F)(F)F		-6.29	0.65	2.00	P	IN
QL00178	Potassium 1,1,2,2,3,3,4,4-octafluorobutane-1-sulphonate	70259-85-7	[K+].[O-]S(=O)(=O)C(F)(F)C(F)(F)C(F)(F)C(F)F		-5.94	0.63	2.02	P	IN
QL00179	Potassium 1,1,2,2-tetrafluoro-2-{[1,1,1,2,3,3-hexafluoro-3-(pentafluoroethoxy)propan-2-yl]oxy}ethane-1-sulfonate	70755-50-9	[K+].[O-]S(=O)(=O)C(F)(F)C(F)(F)OC(F)(C(F)(F)F)C(F)(F)OC(F)(F)C(F)(F)F		-7.61	0.66	2.56	P	IN
QL00180	Potassium 9-chlorohexadecafluoro-3-oxanonane-1-sulfonate	73606-19-6	[K+].[O-]S(=O)(=O)C(F)(F)C(F)(F)OC(F)(F)C(F)(F)C(F)(F)C(F)(F)C(F)(F)C(F)(F)Cl		-7.99	0.66	2.63	P	IN
QL00181	Perfluoro-2-(hexyloxy)ethane-1-sulfonic acid potassium salt	68136-88-9	[K+].[O-]S(=O)(=O)C(F)(F)C(F)(F)OC(F)(F)C(F)(F)C(F)(F)C(F)(F)C(F)(F)C(F)(F)F		-7.99	0.66	2.63	P	IN
QL00182	Potassium 1,1,2,2-tetrafluoro-2-(pentafluoroethoxy)ethane-1-sulfonate	117205-07-9	[K+].[O-]S(=O)(=O)C(F)(F)C(F)(F)OC(F)(F)C(F)(F)F		-6.32	0.64	2.09	P	IN
QL00183	Cyclohexanesulfonic acid, decafluoro(trifluoromethyl)-, potassium salt (11)	68156-07-0	[K+].[O-]S(=O)(=O)C1(C(F)(F)F)C(F)(F)C(F)(F)C(F)(F)C(F)(F)C1(F)F		-6.16	0.66	1.70	P	IN
QL00184	Potassium 1,2,2,3,3,4,5,5,6,6-decafluoro-4-(pentafluoroethyl)cyclohexanesulphonate	335-24-0	[K+].[O-]S(=O)(=O)C1(F)C(F)(F)C(F)(F)C(F)(C(F)(F)C(F)(F)F)C(F)(F)C1(F)F		-7.60	0.67	2.36	P	IN
QL00185	Potassium undecafluorocyclohexanesulphonate	3107-18-4	[K+].[O-]S(=O)(=O)C1(F)C(F)(F)C(F)(F)C(F)(F)C(F)(F)C1(F)F		-6.12	0.66	1.67	P	IN

QL00186	Potassium 3,3,4,4,5,5,6,6,7,7,8,8,8-tridecafluorooctanesulphonate	59587-38-1	[K+].[O-]S(=O)(=O)CCC(F)(F)C(F)(F)C(F)(F)C(F)(F)C(F)(F)C(F)(F)F		-7.46	0.65	2.58	P	IN
QL00187	Potassium 5,5,6,6,7,7,8,8,8-nonafluorooctane-1-sulfonate	1604816-60-5	[K+].[O-]S(=O)(=O)CCCCC(F)(F)C(F)(F)C(F)(F)C(F)(F)F		-6.68	0.62	2.57	P	OUT
QL00188	Potassium 3,3,4,4,5,5,6,6,7,7,8,8,9,9,10,10,11,11,12,12,12-henicosafluorododecyl sulfate	1262446-14-9	[K+].[O-]S(=O)(=O)OCCC(F)(F)C(F)(F)C(F)(F)C(F)(F)C(F)(F)C(F)(F)C(F)(F)C(F)(F)C(F)(F)C(F)(F)F		-8.66	0.66	3.03	P	IN
QL00189	Potassium 3,3,4,4,5,5,6,6,7,7,8,8,9,9,10,10,10-heptadecafluorodecyl sulfate	1262446-13-8	[K+].[O-]S(=O)(=O)OCCC(F)(F)C(F)(F)C(F)(F)C(F)(F)C(F)(F)C(F)(F)C(F)(F)C(F)(F)F		-8.11	0.65	2.85	P	IN
QL00190	3-[(Perfluorooctane-1-sulfonyl)amino]-N,N-dimethylpropan-1-amine N-oxide potassium	178094-69-4	[K+].C[N+](C)([O-])CCC[N-]S(=O)(=O)C(F)(F)C(F)(F)C(F)(F)C(F)(F)C(F)(F)C(F)(F)C(F)(F)C(F)(F)F		-8.46	0.65	3.03	P	IN
QL00191	3-[(Perfluoroheptane-1-sulfonyl)amino]-N,N-dimethylpropan-1-amine N-oxide potassium salt	178094-70-7	[K+].C[N+](C)([O-])CCC[N-]S(=O)(=O)C(F)(F)C(F)(F)C(F)(F)C(F)(F)C(F)(F)C(F)(F)C(F)(F)F		-8.28	0.65	3.00	P	IN
QL00192	3-[(Perfluorohexane-1-sulfonyl)amino]-N,N-dimethylpropan-1-amine N-oxide potassium salt	178094-71-8	[K+].C[N+](C)([O-])CCC[N-]S(=O)(=O)C(F)(F)C(F)(F)C(F)(F)C(F)(F)C(F)(F)C(F)(F)F		-7.86	0.64	2.86	P	IN
QL00193	3-[(Perfluoropentane-1-sulfonyl)amino]-N,N-dimethylpropan-1-amine N-oxide potassium salt	178094-72-9	[K+].C[N+](C)([O-])CCC[N-]S(=O)(=O)C(F)(F)C(F)(F)C(F)(F)C(F)(F)C(F)(F)F		-7.64	0.64	2.82	P	OUT
QL00194	3-[(Perfluorobutane-1-sulfonyl)amino]-N,N-dimethylpropan-1-amine N-oxide potassium salt	178094-73-0	[K+].C[N+](C)([O-])CCC[N-]S(=O)(=O)C(F)(F)C(F)(F)C(F)(F)C(F)(F)F		-7.12	0.63	2.66	P	OUT
QL00195	Cyclohexanesulfonic acid, decafluoro(pentafluoroethyl)-, potassium salt (TSCA, DSL, AICS)	67584-42-3	[K+].C1(C(C(C(C(C1(F)F)(F)F)(F)F)(F)F)(F)F)(S(=O)(=O)[O-])C(C(F)(F)F)(F)F		-6.87	0.67	2.01	P	IN
QL00196	Potassium N-propyl-N-((tridecafluorohexyl)sulphonyl)glycinate	85665-66-3	[K+].CCCN(CC([O-])=O)S(=O)(=O)C(F)(F)C(F)(F)C(F)(F)C(F)(F)C(F)(F)C(F)(F)F		-7.07	0.63	2.64	P	OUT
QL00197	Potassium N-[(heptadecafluorooctyl)sulphonyl]-N-propylglycinate	55910-10-6	[K+].CCCN(CC(=O)[O-])S(=O)(=O)C(F)(F)C(F)(F)C(F)(F)C(F)(F)C(F)(F)C(F)(F)C(F)(F)C(F)(F)F		-7.79	0.64	2.85	P	OUT
QL00198	Potassium N-ethyl-N-[(heptadecafluorooctyl)sulphonyl]glycinate	2991-51-7	[K+].CCN(CC(=O)[O-])S(=O)(=O)C(F)(F)C(F)(F)C(F)(F)C(F)(F)C(F)(F)C(F)(F)C(F)(F)C(F)(F)F		-7.96	0.65	2.85	P	IN
QL00199	Potassium N-ethyl-N-[(pentadecafluoroheptyl)sulphonyl]glycinate	67584-62-7	[K+].CCN(CC(=O)[O-])S(=O)(=O)C(F)(F)C(F)(F)C(F)(F)C(F)(F)C(F)(F)C(F)(F)C(F)(F)F		-7.73	0.64	2.81	P	IN
QL00200	Potassium N-ethyl-N-[(tridecafluorohexyl)sulphonyl]glycinate	67584-53-6	[K+].CCN(CC(=O)[O-])S(=O)(=O)C(F)(F)C(F)(F)C(F)(F)C(F)(F)C(F)(F)C(F)(F)F		-7.26	0.64	2.65	P	OUT
QL00201	Potassium N-ethyl-N-[(undecafluoropentyl)sulphonyl]glycinate	67584-52-5	[K+].CCN(CC(=O)[O-])S(=O)(=O)C(F)(F)C(F)(F)C(F)(F)C(F)(F)C(F)(F)F		-6.97	0.63	2.59	P	OUT
QL00202	Potassium N-ethyl-N-[(nonafluorobutyl)sulphonyl]glycinate	67584-51-4	[K+].CCN(CC(=O)[O-])S(=O)(=O)C(F)(F)C(F)(F)C(F)(F)C(F)(F)F		-6.35	0.62	2.40	P	OUT
QL00203	1-[2-[Ethyl[(perfluorooctyl)sulfonyl]amino]ethyl] butanedioate potassium	92340-26-6	[K+].CCN(CCOC(=O)CCC([O-])=O)S(=O)(=O)C(F)(F)C(F)(F)C(F)(F)C(F)(F)C(F)(F)C(F)(F)C(F)(F)C(F)(F)F		-8.51	0.63	3.33	P	OUT
QL00204	Potassium N-[(heptadecafluorooctyl)sulphonyl]-N-methylglycinate	70281-93-5	[K+].CN(CC(=O)[O-])S(=O)(=O)C(F)(F)C(F)(F)C(F)(F)C(F)(F)C(F)(F)C(F)(F)C(F)(F)C(F)(F)F		-8.16	0.65	2.85	P	IN
QL00205	Potassium N-((perfluoro-7-methyloctyl)-2-hydroxypropyl)-N-methylaminoacetate	94159-89-4	[K+].CN(CC(O)CC(F)(F)C(F)(F)C(F)(F)C(F)(F)C(F)(F)C(F)(F)C(F)(C(F)(F)F)C(F)(F)F)CC([O-])=O		-8.13	0.64	3.01	P	OUT
QL00206	Potassium N-((perfluorooctyl)-2-hydroxypropyl)-N-methylglycinate	94159-87-2	[K+].CN(CC(O)CC(F)(F)C(F)(F)C(F)(F)C(F)(F)C(F)(F)C(F)(F)C(F)(F)C(F)(F)F)CC([O-])=O		-8.14	0.64	3.05	P	OUT
QL00207	Potassium bis(perfluorohexyl)difluoroborate	937038-66-9	[K+].F[B-](F)(C(F)(F)C(F)(F)C(F)(F)C(F)(F)C(F)(F)C(F)(F)F)C(F)(F)C(F)(F)C(F)(F)C(F)(F)C(F)(F)C(F)(F)F		-9.04	0.68	2.99	P	OUT
QL00208	Potassium N,N-Bis(perfluorobutanesulfonyl)amide	129135-87-1	[K+].FC(F)(F)C(F)(F)C(F)(F)C(F)(F)S(=O)(=O)[N-]S(=O)(=O)C(F)(F)C(F)(F)C(F)(F)C(F)(F)F		-8.31	0.67	2.75	P	IN
QL00209	Potassium 4,4,5,5,6,6-hexafluoro-1,1,3,3-tetraoxo-1lambda~6~,3lambda~6~,2-dithiazinan-2-ide	588668-97-7	[K+].FC1(F)C(F)(F)S(=O)(=O)[N-]S(=O)(=O)C1(F)F		-5.42	0.65	1.52	P	IN
QL00210	Potassium decafluoro(pentafluoroethyl)cyclohexanesulphonate	67584-42-3	[K+].O=S(=O)([O-])C1(F)C(F)(F)C(F)(F)C(F)(F)C(F)(F)C1(F)C(F)(F)C(F)(F)F		-6.90	0.67	2.01	P	IN
QL00211	Lanthanum, tris(6,6,7,7,8,8,8-heptafluoro-2,2-dimethyl-3,5-octanedionato-βO3,βO5)-	19106-89-9	[La+3].CC(C)(C)C([O-])=CC(=O)C(F)(F)C(F)(F)C(F)(F)F.CC(C)(C)C([O-])=CC(=O)C(F)(F)C(F)(F)C(F)(F)F.CC(C)(C)C([O-])=CC(=O)C(F)(F)C(F)(F)C(F)(F)F		-6.81	0.65	2.24	P	IN

QL00212	Bis((perfluorobutyl)ethyl) 2-[(carboxymethyl)thio]butanedioate lithium salt	503534-33-6	[Li+].[H]C([H])(OC(=O)CC(SCC([O-])=O)C(=O)OC([H])([H])C([H])([H])C(F)(F)C(F)(F)C(F)(F)C(F)(F)F)C([H])([H])C(F)(F)C(F)(F)C(F)(F)C(F)(F)F		-8.23	0.63	3.30	P	OUT
QL00213	Isooctanesulfonic acid, heptadecafluoro-, lithium salt	93894-67-8	[Li+].[O-][S](=O)(=O)C(F)(F)C(F)(F)C(F)(F)C(F)(F)C(F)(F)C(F)(C(F)(F)F)C(F)(F)F		-7.46	0.67	2.34	P	IN
QL00214	3-[(3,3,4,4,5,5,6,6,7,7,8,8,8-Tridecafluorooctyl)thio]propanoic acid lithium salt	433333-62-1	[Li+].[O-]C(=O)CCSCCC(F)(F)C(F)(F)C(F)(F)C(F)(F)C(F)(F)C(F)(F)F		-7.34	0.62	2.89	P	OUT
QL00215	Lithium 1,1,1,3,3,4,4-heptafluoro-4-[(1,1,1,2,3,3,3-heptafluoropropan-2-yl)oxy]-2-(trifluoromethyl)butan-2-olate	78740-39-3	[Li+].[O-]C(C(F)(F)F)(C(F)(F)F)C(F)(F)C(F)(F)OC(F)(C(F)(F)F)C(F)(F)F		-6.83	0.66	2.15	P	IN
QL00216	Lithium heptadecafluorooctanesulphonate	29457-72-5	[Li+].[O-]S(=O)(=O)C(F)(F)C(F)(F)C(F)(F)C(F)(F)C(F)(F)C(F)(F)C(F)(F)C(F)(F)F		-7.99	0.67	2.57	P	IN
QL00217	Lithium perfluoroheptanesulfonate	117806-54-9	[Li+].[O-]S(=O)(=O)C(F)(F)C(F)(F)C(F)(F)C(F)(F)C(F)(F)C(F)(F)C(F)(F)F		-7.52	0.67	2.38	P	IN
QL00218	Lithium perfluorohexanesulfonate	55120-77-9	[Li+].[O-]S(=O)(=O)C(F)(F)C(F)(F)C(F)(F)C(F)(F)C(F)(F)C(F)(F)F		-7.26	0.66	2.31	P	IN
QL00219	Lithium nonafluorobutane-1-sulfonate	131651-65-5	[Li+].[O-]S(=O)(=O)C(F)(F)C(F)(F)C(F)(F)C(F)(F)F		-6.29	0.65	2.00	P	IN
QL00220	Lithium 5,5,6,6,7,7,8,8,8-nonafluorooctane-1-sulfonate	1604816-75-2	[Li+].[O-]S(=O)(=O)CCCCC(F)(F)C(F)(F)C(F)(F)C(F)(F)F		-6.68	0.62	2.57	P	OUT
QL00221	3-[(3,3,4,4,5,5,6,6,7,7,8,8,9,9,10,10,10-Heptadecafluorodecyl)thio]-2-methylpropanoic acid lithium salt	476304-39-9	[Li+].CC(CSCCC(F)(F)C(F)(F)C(F)(F)C(F)(F)C(F)(F)C(F)(F)C(F)(F)C(F)(F)F)C([O-])=O		-8.17	0.64	3.05	P	OUT
QL00222	N,N-bis(1,1,2,2,3,3,4,4,4-nonafluorobutyl)sulfonylamine lithium salt	119229-99-1	[Li+].FC(F)(F)C(F)(F)C(F)(F)C(F)(F)S(=O)(=O)[N-]S(=O)(=O)C(F)(F)C(F)(F)C(F)(F)C(F)(F)F		-8.31	0.67	2.75	P	IN
QL00223	Lithium bis[(pentafluoroethyl)sulfonyl]azanide	132843-44-8	[Li+].FC(F)(F)C(F)(F)S(=O)(=O)[N-]S(=O)(=O)C(F)(F)C(F)(F)F		-6.86	0.65	2.25	P	IN
QL00224	Lithium 4,4,5,5,6,6-hexafluoro-1,1,3,3-tetraoxo-1lambda~6~,3lambda~6~,2-dithiazinan-2-ide	189217-62-7	[Li+].FC1(F)C(F)(F)S(=O)(=O)[N-]S(=O)(=O)C1(F)F		-5.42	0.65	1.52	P	IN
QL00225	Magnesium heptadecafluoroisooctanesulphonate	93894-73-6	[Mg++].[O-]S(=O)(=O)C(F)(F)C(F)(F)C(F)(F)C(F)(F)C(F)(F)C(F)(C(F)(F)F)C(F)(F)F.[O-]S(=O)(=O)C(F)(F)C(F)(F)C(F)(F)C(F)(F)C(F)(F)C(F)(C(F)(F)F)C(F)(F)F		-7.67	0.68	2.29	P	IN
QL00226	Magnesium nonafluorobutanesulfonate	507453-86-3	[Mg++].[O-]S(=O)(=O)C(F)(F)C(F)(F)C(F)(F)C(F)(F)F.[O-]S(=O)(=O)C(F)(F)C(F)(F)C(F)(F)C(F)(F)F		-6.66	0.67	1.93	P	IN
QL00227	Magnesium bis[heptadecafluorooctanesulphonate]	91036-71-4	[Mg+2].[O-]S(=O)(=O)C(F)(F)C(F)(F)C(F)(F)C(F)(F)C(F)(F)C(F)(F)C(F)(F)C(F)(F)F.[O-]S(=O)(=O)C(F)(F)C(F)(F)C(F)(F)C(F)(F)C(F)(F)C(F)(F)C(F)(F)C(F)(F)F		-8.21	0.68	2.53	P	IN
QL00228	Sodium hydrogen ((perfluorohexyl)ethyl)phosphonate	1189052-95-6	[Na+].[H]C([H])(CP(O)([O-])=O)C(F)(F)C(F)(F)C(F)(F)C(F)(F)C(F)(F)C(F)(F)F		-7.46	0.65	2.58	P	IN
QL00229	Sodium 3-(methyl(3-(((tridecafluorohexyl)sulphonyl)amino)propyl)amino)propanesulphonate	80621-17-6	[Na+].[H]N(CCCN(C)CCCS([O-])(=O)=O)S(=O)(=O)C(F)(F)C(F)(F)C(F)(F)C(F)(F)C(F)(F)C(F)(F)F		-8.18	0.63	3.19	P	OUT
QL00230	N-(Carboxymethyl)-N-[3-[[(perfluorooctyl)sulfonyl]amino]propyl]-N,N-bis(2-hydroxy-3-sulfopropyl)ammonium chloride, trisodium	109669-84-3	[Na+].[Na+].[Na+].[Cl-].OC(C[N+](CCC[N-]S(=O)(=O)C(F)(F)C(F)(F)C(F)(F)C(F)(F)C(F)(F)C(F)(F)C(F)(F)C(F)(F)F)(CC(O)CS([O-])(=O)=O)CC(O)=O)CS([O-])(=O)=O		-7.71	0.60	3.35	P	OUT
QL00231	Disodium 2,2,3,3,4,4,5,5,6,6,7,7-dodecafluoroheptyl phosphate	3107-32-2	[Na+].[Na+].OP(O)(=O)OCC(F)(F)C(F)(F)C(F)(F)C(F)(F)C(F)(F)C(F)F		-6.66	0.61	2.66	P	OUT
QL00232	Sodium 2,3,3,4,4,5,5,6,6,6-decafluoro-2-(pentafluoroethyl)hexanoate	1195164-59-0	[Na+].[O-]C(=O)C(F)(C(F)(F)C(F)(F)F)C(F)(F)C(F)(F)C(F)(F)C(F)(F)F		-7.01	0.65	2.28	P	IN
QL00233	Sodium 2,2,3,3,4,4,5,5,6,7,7,7-dodecafluoro-6-(trifluoromethyl)heptanoate	18017-22-6	[Na+].[O-]C(=O)C(F)(F)C(F)(F)C(F)(F)C(F)(F)C(F)(C(F)(F)F)C(F)(F)F		-6.99	0.65	2.28	P	IN
QL00234	Sodium henicosafluoroundecanoate	60871-96-7	[Na+].[O-]C(=O)C(F)(F)C(F)(F)C(F)(F)C(F)(F)C(F)(F)C(F)(F)C(F)(F)C(F)(F)C(F)(F)C(F)(F)F		-8.40	0.67	2.80	P	IN
QL00235	Sodium perfluorodecanoate	3830-45-3	[Na+].[O-]C(=O)C(F)(F)C(F)(F)C(F)(F)C(F)(F)C(F)(F)C(F)(F)C(F)(F)C(F)(F)C(F)(F)F		-8.00	0.66	2.64	P	IN
QL00236	Sodium heptadecafluorononanoate	21049-39-8	[Na+].[O-]C(=O)C(F)(F)C(F)(F)C(F)(F)C(F)(F)C(F)(F)C(F)(F)C(F)(F)C(F)(F)F		-7.79	0.66	2.58	P	IN

QL00237	Sodium pentadecafluorooctanoate	335-95-5	[Na+].[O-]C(=O)C(F)(F)C(F)(F)C(F)(F)C(F)(F)C(F)(F)C(F)(F)C(F)(F)F		-7.29	0.66	2.39	P	IN
QL00238	Sodium 2,2,3,3,4,4,5,5,6,6,7,7-dodecafluoroheptanoate	2264-25-7	[Na+].[O-]C(=O)C(F)(F)C(F)(F)C(F)(F)C(F)(F)C(F)(F)C(F)F		-6.71	0.64	2.32	P	IN
QL00239	Sodium undecafluorohexanoate	2923-26-4	[Na+].[O-]C(=O)C(F)(F)C(F)(F)C(F)(F)C(F)(F)C(F)(F)F		-6.35	0.64	2.09	P	IN
QL00240	Sodium heptafluorobutyrate	2218-54-4	[Na+].[O-]C(=O)C(F)(F)C(F)(F)C(F)(F)F		-4.97	0.62	1.73	P	OUT
QL00241	Sodium 2,2,3,3-tetrafluoro-3-(heptafluoropropoxy)propanoate	79630-80-1	[Na+].[O-]C(=O)C(F)(F)C(F)(F)OC(F)(F)C(F)(F)C(F)(F)F		-6.57	0.64	2.27	P	IN
QL00242	Sodium 2,3,3,3-tetrafluoro-2-(heptafluoropropoxy)propanoate	67963-75-1	[Na+].[O-]C(=O)C(F)(OC(F)(F)C(F)(F)C(F)(F)F)C(F)(F)F		-6.08	0.64	2.03	P	IN
QL00243	Sodium bis(heptadecafluorooctyl)phosphinate	500776-69-2	[Na+].[O-]P(=O)(C(F)(F)C(F)(F)C(F)(F)C(F)(F)C(F)(F)C(F)(F)C(F)(F)C(F)(F)F)C(F)(F)C(F)(F)C(F)(F)C(F)(F)C(F)(F)C(F)(F)C(F)(F)C(F)(F)F		-9.81	0.68	3.31	P	OUT
QL00244	Sodium bis(tridecafluorohexyl)phosphinate	70609-44-8	[Na+].[O-]P(=O)(C(F)(F)C(F)(F)C(F)(F)C(F)(F)C(F)(F)C(F)(F)F)C(F)(F)C(F)(F)C(F)(F)C(F)(F)C(F)(F)C(F)(F)F		-9.04	0.68	2.99	P	OUT
QL00245	1,4-Bis(1H,1H,9H-perfluorononyl) sulfobutanedioate sodium	63367-17-9	[Na+].[O-]S(=O)(=O)C(CC(=O)OCC(F)(F)C(F)(F)C(F)(F)C(F)(F)C(F)(F)C(F)(F)C(F)(F)C(F)F)C(=O)OCC(F)(F)C(F)(F)C(F)(F)C(F)(F)C(F)(F)C(F)(F)C(F)(F)C(F)F		-9.80	0.66	3.64	P	OUT
QL00246	Bis(2,2,3,3,4,4,5,5,6,6,7,7-Dodecafluoroheptyl)sulfosuccinate sodium salt	60131-27-3	[Na+].[O-]S(=O)(=O)C(CC(=O)OCC(F)(F)C(F)(F)C(F)(F)C(F)(F)C(F)(F)C(F)F)C(=O)OCC(F)(F)C(F)(F)C(F)(F)C(F)(F)C(F)(F)C(F)F		-9.04	0.65	3.40	P	OUT
QL00247	Bis(1H,1H,6H-perfluorohexyl)-2-sulfobutanedioate sodium salt	98151-22-5	[Na+].[O-]S(=O)(=O)C(CC(=O)OCC(F)(F)C(F)(F)C(F)(F)C(F)(F)C(F)F)C(=O)OCC(F)(F)C(F)(F)C(F)(F)C(F)(F)C(F)F		-8.58	0.64	3.26	P	OUT
QL00248	Sodium 1,4-bis(3,3,4,4,5,5,6,6,7,7,8,8,8-tridecafluorooctyl) sulphonatosuccinate	54950-05-9	[Na+].[O-]S(=O)(=O)C(CC(=O)OCCC(F)(F)C(F)(F)C(F)(F)C(F)(F)C(F)(F)C(F)(F)F)C(=O)OCCC(F)(F)C(F)(F)C(F)(F)C(F)(F)C(F)(F)C(F)(F)F		-9.34	0.65	3.50	P	OUT
QL00249	Sodium perfluorodecanesulfonate	2806-15-7	[Na+].[O-]S(=O)(=O)C(F)(F)C(F)(F)C(F)(F)C(F)(F)C(F)(F)C(F)(F)C(F)(F)C(F)(F)C(F)(F)C(F)(F)F		-8.56	0.67	2.79	P	IN
QL00250	Sodium Perfluorononanesulfonate	98789-57-2	[Na+].[O-]S(=O)(=O)C(F)(F)C(F)(F)C(F)(F)C(F)(F)C(F)(F)C(F)(F)C(F)(F)C(F)(F)C(F)(F)F		-8.19	0.67	2.63	P	IN
QL00251	Sodium perfluorooctanesulfonate	4021-47-0	[Na+].[O-]S(=O)(=O)C(F)(F)C(F)(F)C(F)(F)C(F)(F)C(F)(F)C(F)(F)C(F)(F)C(F)(F)F		-7.99	0.67	2.57	P	IN
QL00252	Sodium perfluorohexanesulfonate	82382-12-5	[Na+].[O-]S(=O)(=O)C(F)(F)C(F)(F)C(F)(F)C(F)(F)C(F)(F)C(F)(F)F		-7.26	0.66	2.31	P	IN
QL00253	Sodium nonafluorobutane-1-sulfonate	60453-92-1	[Na+].[O-]S(=O)(=O)C(F)(F)C(F)(F)C(F)(F)C(F)(F)F		-6.29	0.65	2.00	P	IN
QL00254	Sodium 1,1,2,2-tetrafluoro-2-(1,1,2,2,2-pentafluoroethoxy)ethanesulfonate	113507-87-2	[Na+].[O-]S(=O)(=O)C(F)(F)C(F)(F)OC(F)(F)C(F)(F)F		-6.32	0.64	2.09	P	IN
QL00255	Sodium 2â€[(perfluorooctyl)oxy]benzeneâ€?â€sulfonate	41674-07-1	[Na+].[O-]S(=O)(=O)C1=C(OC(F)(F)C(F)(F)C(F)(F)C(F)(F)C(F)(F)C(F)(F)C(F)(F)C(F)(F)F)C=CC=C1		-7.97	0.65	2.84	P	IN
QL00256	4-Heptadecafluorooctanesulfonamidobenzenesulfonic acid sodium salt	172398-80-0	[Na+].[O-]S(=O)(=O)C1=CC=C(NS(=O)(=O)C(F)(F)C(F)(F)C(F)(F)C(F)(F)C(F)(F)C(F)(F)C(F)(F)C(F)(F)F)C=C1		-8.78	0.66	3.17	P	IN
QL00257	Sodium 4-(perfluoro(2-methylpent-2-en-3-yl)oxy)benzene-1-sulfonate	85284-17-9	[Na+].[O-]S(=O)(=O)C1=CC=C(OC(=C(C(F)(F)F)C(F)(F)F)C(F)(F)C(F)(F)F)C=C1		-6.29	0.62	2.35	P	OUT
QL00258	Sodium 4-(perfluoro(3,4-dimethylhex-3-en-2-yloxy))benzene-1-sulfonate	94042-94-1	[Na+].[O-]S(=O)(=O)C1=CC=C(OC(F)(C(=C(C(F)(F)F)C(F)(F)C(F)(F)F)C(F)(F)F)C(F)(F)F)C=C1		-7.36	0.63	2.73	P	OUT
QL00259	Sodium 4-{[(4E)-4,6-dimethylhept-4-en-3-yl]oxy}benzene-1-sulfonate	85284-15-7	[Na+].[O-]S(=O)(=O)C1=CC=C(OC(F)(C(=C(F)C(F)(C(F)(F)F)C(F)(F)F)C(F)(F)F)C(F)(F)C(F)(F)F)C=C1		-7.38	0.64	2.66	P	IN
QL00260	Sodium 4-perfluorononyloxybenzenesulphonate	59536-17-3	[Na+].[O-]S(=O)(=O)C1=CC=C(OC(F)(F)C(F)(F)C(F)(F)C(F)(F)C(F)(F)C(F)(F)C(F)(F)C(F)(F)C(F)(F)F)C=C1		-8.78	0.66	3.17	P	IN
QL00261	Sodium 4-(perfluoro-2-(butan-2-yl)-3-ethyl-3-methylpent-1-en-1-yloxy)benzenesulfonate	94042-95-2	[Na+].[O-]S(=O)(=O)C1=CC=C(OC(F)=C(C(F)(C(F)(F)F)C(F)(F)C(F)(F)F)C(C(F)(F)F)(C(F)(F)C(F)(F)F)C(F)(F)C(F)(F)F)C=C1		-7.27	0.65	2.48	P	IN
QL00262	Sodium 4-perfluorohexenyloxybenzenesulfonate	59493-84-4	[Na+].[O-]S(=O)(=O)C1=CC=C(OC(F)=C(F)C(F)(F)C(F)(F)C(F)(F)C(F)(F)F)C=C1		-7.39	0.63	2.86	P	OUT

QL00263	Sodium 1-(2,2,3,3,4,4,5,5,6,6,7,7-dodecafluoroheptyl) 2-sulphonatobenzoate	29811-19-6	[Na+].[O-]S(=O)(=O)C1=CC=CC=C1C(=O)OCC(F)(F)C(F)(F)C(F)(F)C(F)(F)C(F)(F)C(F)F		-7.40	0.63	2.86	P	OUT
QL00264	Sodium [[[(heptadecafluorooctyl)sulphonyl]amino]methyl]benzenesulphonate	51032-47-4	[Na+].[O-]S(=O)(=O)c1ccccc1CNS(=O)(=O)C(F)(F)C(F)(F)C(F)(F)C(F)(F)C(F)(F)C(F)(F)C(F)(F)C(F)(F)F		-8.36	0.65	3.02	P	IN
QL00265	Sodium [[[(tridecafluorohexyl)sulphonyl]amino]methyl]benzenesulphonate	68299-21-8	[Na+].[O-]S(=O)(=O)c1ccccc1CNS(=O)(=O)C(F)(F)C(F)(F)C(F)(F)C(F)(F)C(F)(F)C(F)(F)F		-7.77	0.64	2.84	P	OUT
QL00266	Sodium [[[(undecafluoropentyl)sulphonyl]amino]methyl]benzenesulphonate	68299-20-7	[Na+].[O-]S(=O)(=O)c1ccccc1CNS(=O)(=O)C(F)(F)C(F)(F)C(F)(F)C(F)(F)C(F)(F)F		-7.52	0.64	2.79	P	OUT
QL00267	Sodium 3,3,4,4,5,5,6,6,7,7,8,8,8-tridecafluorooctane-1-sulfonate	27619-94-9	[Na+].[O-]S(=O)(=O)CCC(F)(F)C(F)(F)C(F)(F)C(F)(F)C(F)(F)C(F)(F)F		-7.46	0.65	2.58	P	IN
QL00268	Sodium 5,5,6,6,7,7,8,8,8-nonafluorooctane-1-sulfonate	65345-91-7	[Na+].[O-]S(=O)(=O)CCCCC(F)(F)C(F)(F)C(F)(F)C(F)(F)F		-6.68	0.62	2.57	P	OUT
QL00269	1,3-Bis[(6H-perfluorohexyl)methoxy]-2-propanol hydrogen sulfate sodium	61119-62-8	[Na+].[O-]S(=O)(=O)OC(COCC(F)(F)C(F)(F)C(F)(F)C(F)(F)C(F)(F)C(F)F)COCC(F)(F)C(F)(F)C(F)(F)C(F)(F)C(F)(F)C(F)F		-8.93	0.64	3.40	P	OUT
QL00270	Sodium 2-(((perfluorohexyl)ethyloxy)carbonylamino)ethylsulfanylsulfonate	82199-07-3	[Na+].[O-]S(=O)(=O)SCCNC(=O)OCCC(F)(F)C(F)(F)C(F)(F)C(F)(F)C(F)(F)C(F)(F)F		-7.88	0.63	3.13	P	OUT
QL00271	Sodium heptadecafluorooctanesulphinate	68555-67-9	[Na+].[O-]S(=O)C(F)(F)C(F)(F)C(F)(F)C(F)(F)C(F)(F)C(F)(F)C(F)(F)C(F)(F)F		-7.79	0.66	2.58	P	IN
QL00272	Sodium 1,1,2,2,3,3,4,4,5,5,6,6,7,7,7-pentadecafluoroheptane-1-sulphinate	68555-66-8	[Na+].[O-]S(=O)C(F)(F)C(F)(F)C(F)(F)C(F)(F)C(F)(F)C(F)(F)C(F)(F)F		-7.29	0.66	2.39	P	IN
QL00273	Sodium undecafluoropentane-1-sulfinate	1997344-07-6	[Na+].[O-]S(=O)C(F)(F)C(F)(F)C(F)(F)C(F)(F)C(F)(F)F		-6.35	0.64	2.09	P	IN
QL00274	Sodium 1,1,2,2,3,3,4,4,4-nonafluoro1-butanesulfinate	102061-82-5	[Na+].[O-]S(=O)C(F)(F)C(F)(F)C(F)(F)C(F)(F)F		-5.94	0.63	2.02	P	IN
QL00275	Lodyne S 103A	62880-93-7	[Na+].CC(C)(CS([O-])(=O)=O)NC(=O)CCSCCC(F)(F)C(F)(F)C(F)(F)C(F)(F)C(F)(F)C(F)(F)F		-8.04	0.63	3.15	P	OUT
QL00276	1-(Perfluorooctyl)nonanol sulfate sodium	799267-73-5	[Na+].CCCCCCCCC(OS([O-])(=O)=O)C(F)(F)C(F)(F)C(F)(F)C(F)(F)C(F)(F)C(F)(F)C(F)(F)C(F)(F)F		-8.05	0.63	3.20	P	OUT
QL00277	(1H,1H,7H-perfluoroheptyl)-2-sulfohexadecanoate sodium salt	75032-94-9	[Na+].CCCCCCCCCCCCCCC(C(=O)OCC(F)(F)C(F)(F)C(F)(F)C(F)(F)C(F)(F)C(F)F)S([O-])(=O)=O		-7.95	0.59	3.65	P	OUT
QL00278	N-Ethyl-N-(perfluoro-1-oxoheptyl)-glycine sodium salt	116537-74-7	[Na+].CCN(CC([O-])=O)C(=O)C(F)(F)C(F)(F)C(F)(F)C(F)(F)C(F)(F)C(F)(F)F		-7.06	0.63	2.65	P	OUT
QL00279	Sodium N-ethyl-N-[(heptadecafluorooctyl)sulphonyl]glycinate	3871-50-9	[Na+].CCN(CC(=O)[O-])S(=O)(=O)C(F)(F)C(F)(F)C(F)(F)C(F)(F)C(F)(F)C(F)(F)C(F)(F)C(F)(F)F		-7.96	0.65	2.85	P	IN
QL00280	Sodium N-ethyl-N-[(pentadecafluoroheptyl)sulphonyl]glycinate	68555-71-5	[Na+].CCN(CC(=O)[O-])S(=O)(=O)C(F)(F)C(F)(F)C(F)(F)C(F)(F)C(F)(F)C(F)(F)C(F)(F)F		-7.73	0.64	2.81	P	IN
QL00281	Sodium N-ethyl-N-[(tridecafluorohexyl)sulphonyl]glycinate	68555-70-4	[Na+].CCN(CC(=O)[O-])S(=O)(=O)C(F)(F)C(F)(F)C(F)(F)C(F)(F)C(F)(F)C(F)(F)F		-7.26	0.64	2.65	P	OUT
QL00282	Sodium N-ethyl-N-[(undecafluoropentyl)sulphonyl]glycinate	68555-69-1	[Na+].CCN(CC(=O)[O-])S(=O)(=O)C(F)(F)C(F)(F)C(F)(F)C(F)(F)C(F)(F)F		-6.97	0.63	2.59	P	OUT
QL00283	Sodium N-ethyl-N-[(nonafluorobutyl)sulphonyl]glycinate	68555-68-0	[Na+].CCN(CC(=O)[O-])S(=O)(=O)C(F)(F)C(F)(F)C(F)(F)C(F)(F)F		-6.35	0.62	2.40	P	OUT
QL00284	3-[Ethyl(perfluoro-1-oxooctyl)amino]-1-propanesulfonic acid sodium salt	89685-61-0	[Na+].CCN(CCCS([O-])(=O)=O)C(=O)C(F)(F)C(F)(F)C(F)(F)C(F)(F)C(F)(F)C(F)(F)C(F)(F)F		-7.98	0.64	3.01	P	OUT
QL00285	1-Propanesulfonic acid, 3-[ethyl[(heptadecafluorooctyl)sulfonyl]amino]-, sodium salt	75032-81-4	[Na+].CCN(CCCS([O-])(=O)=O)S(=O)(=O)C(F)(F)C(F)(F)C(F)(F)C(F)(F)C(F)(F)C(F)(F)C(F)(F)C(F)(F)F		-8.33	0.65	3.04	P	IN
QL00286	Sodium 3-[[3-(dimethylamino)propyl][(perfluorooctyl)sulfonyl]amino]-2-hydroxypropanesulfonateSodium 3-[[3-(dimethylamino)propyl][(heptadecafluorooctyl)sulphonyl]amino]-2-hydroxypropanesulphonate	94133-90-1	[Na+].CN(C)CCCN(CC(O)CS(=O)(=O)[O-])S(=O)(=O)C(F)(F)C(F)(F)C(F)(F)C(F)(F)C(F)(F)C(F)(F)C(F)(F)C(F)(F)F		-8.12	0.64	3.02	P	OUT
QL00287	Sodium 3-[[3-(dimethylamino)propyl][(tridecafluorohexyl)sulphonyl]amino]-2-hydroxypropanesulphonate	73772-32-4	[Na+].CN(C)CCCN(CC(O)CS(=O)(=O)[O-])S(=O)(=O)C(F)(F)C(F)(F)C(F)(F)C(F)(F)C(F)(F)C(F)(F)F		-7.50	0.63	2.83	P	OUT

QL00288	Sodium {[2-(dimethylamino)ethyl](1,1,2,2,3,3,4,4,5,5,6,6,7,7,8,8,8-heptadecafluorooctane-1-sulfonyl)amino}acetate	96426-90-3	[Na+].CN(C)CCN(CC([O-])=O)S(=O)(=O)C(F)(F)C(F)(F)C(F)(F)C(F)(F)C(F)(F)C(F)(F)C(F)(F)C(F)(F)F		-8.12	0.64	2.99	P	OUT
QL00289	N-(Perfluoro-1-oxononyl)-N-methylglycine sodium salt	51588-53-5	[Na+].CN(CC([O-])=O)C(=O)C(F)(F)C(F)(F)C(F)(F)C(F)(F)C(F)(F)C(F)(F)C(F)(F)C(F)(F)F		-7.98	0.65	2.86	P	IN
QL00290	N-[[4-[(Heptadecafluorononenyl)oxy]phenyl]sulfonyl]-N-methylglycine sodium salt	85228-95-1	[Na+].CN(CC([O-])=O)S(=O)(=O)C1=CC=C(OC(F)=C(F)C(F)(F)C(F)(F)C(F)(F)C(F)(F)C(F)(F)C(F)(F)C(F)(F)F)C=C1		-8.63	0.64	3.35	P	OUT
QL00291	Sodium 3-(methyl{3-[(tridecafluorohexyl)amino]propyl}amino)propane-1-sulfonate	94492-37-2	[Na+].CN(CCCNC(F)(F)C(F)(F)C(F)(F)C(F)(F)C(F)(F)C(F)(F)F)CCCS([O-])(=O)=O		-7.88	0.63	3.13	P	OUT
QL00292	Sodium 3-((3-(((heptadecafluorooctyl)sulphonyl)amino)propyl)methylamino)propanesulphonate	85665-65-2	[Na+].CN(CCCNS(=O)(=O)C(F)(F)C(F)(F)C(F)(F)C(F)(F)C(F)(F)C(F)(F)C(F)(F)C(F)(F)F)CCCS([O-])(=O)=O		-8.78	0.64	3.37	P	OUT
QL00293	Sodium 4-[[4,4,5,5,5-pentafluoro-3-(pentafluoroethyl)-1,2,3-tris(trifluoromethyl)-1-pentenyl]oxy]benzenesulphonate	52584-45-9	[Na+].FC(F)(F)C(F)(F)C(C(=C(Oc1ccc(cc1)S([O-])(=O)=O)C(F)(F)F)C(F)(F)F)(C(F)(F)C(F)(F)F)C(F)(F)F		-7.39	0.64	2.65	P	IN
QL00294	Perfluoro-N-[(trifluoromethyl)sulfonyl]butanesulfonamide sodium salt	176719-69-0	[Na+].FC(F)(F)C(F)(F)C(F)(F)C(F)(F)S(=O)(=O)[N-]S(=O)(=O)C(F)(F)F		-7.28	0.66	2.38	P	IN
QL00295	Sodium (2S)-3-carboxy-2-({2-hydroxy-3-[(3,3,4,4,5,5,6,6,7,7,8,8,8-tridecafluorooctyl)sulfanyl]propyl}amino)propanoate	93128-61-1	[Na+].OC(CN[C@@H](CC(O)=O)C([O-])=O)CSCCC(F)(F)C(F)(F)C(F)(F)C(F)(F)C(F)(F)C(F)(F)F		-7.68	0.62	3.16	P	OUT
QL00296	[[4-[(Heptadecafluorononenyl)oxy]phenyl]methyl]phosphonic acid, monosodium salt	63661-51-8	[Na+].OP([O-])(=O)CC1=CC=C(OC(F)=C(F)C(F)(F)C(F)(F)C(F)(F)C(F)(F)C(F)(F)C(F)(F)C(F)(F)F)C=C1		-8.43	0.64	3.22	P	OUT
QL00297	Sodium hydrogen (3,3,4,4,5,5,6,6,7,7,8,8,9,9,10,10,10-heptadecafluorodecyl)phosphonate	92678-93-8	[Na+].OP([O-])(=O)CCC(F)(F)C(F)(F)C(F)(F)C(F)(F)C(F)(F)C(F)(F)C(F)(F)C(F)(F)F		-8.14	0.66	2.80	P	IN
QL00298	Neodymium, tris(6,6,7,7,8,8,8-heptafluoro-2,2-dimethyl-3,5-octanedionato-βO3,βO5)-	17978-76-6	[Nd+3].CC(C)(C)C([O-])=CC(=O)C(F)(F)C(F)(F)C(F)(F)F.CC(C)(C)C([O-])=CC(=O)C(F)(F)C(F)(F)C(F)(F)F.CC(C)(C)C([O-])=CC(=O)C(F)(F)C(F)(F)C(F)(F)F		-6.81	0.65	2.24	P	IN
QL00299	Cyclohexanaminium perfluorononanoate	328531-06-2	[NH3+]C1CCCCC1.[O-]C(=O)C(F)(F)C(F)(F)C(F)(F)C(F)(F)C(F)(F)C(F)(F)C(F)(F)C(F)(F)F		-8.16	0.66	2.85	P	IN
QL00300	72 Fluorotelomer diammonium phosphate	63439-39-4	[NH4+].[NH4+].[O-]P([O-])(=O)OCCC(F)(F)C(F)(F)C(F)(F)C(F)(F)C(F)(F)C(F)(F)C(F)(F)F		-7.42	0.63	2.86	P	OUT
QL00301	Diammonium 3,3,4,4,5,5,6,6,7,7,8,8,9,10,10,10-hexadecafluoro-9-(trifluoromethyl)decyl phosphate	93857-49-9	[NH4+].[NH4+].[O-]P(=O)([O-])OCCC(F)(F)C(F)(F)C(F)(F)C(F)(F)C(F)(F)C(F)(F)C(F)(C(F)(F)F)C(F)(F)F		-7.66	0.64	2.84	P	OUT
QL00302	Diammonium 3,3,4,4,5,5,6,6,7,7,8,8,9,9,10,10,11,12,12,12-icosafluoro-11-(trifluoromethyl)dodecyl phosphate	93857-50-2	[NH4+].[NH4+].[O-]P(=O)([O-])OCCC(F)(F)C(F)(F)C(F)(F)C(F)(F)C(F)(F)C(F)(F)C(F)(F)C(F)(F)C(F)(C(F)(F)F)C(F)(F)F		-8.26	0.65	3.03	P	OUT
QL00303	Diammonium 3,3,4,4,5,5,6,6,7,7,8,8,9,9,10,10,11,11,12,12,13,14,14,14-tetracosafluoro-13-(trifluoromethyl)tetradecyl phosphate	93857-51-3	[NH4+].[NH4+].[O-]P(=O)([O-])OCCC(F)(F)C(F)(F)C(F)(F)C(F)(F)C(F)(F)C(F)(F)C(F)(F)C(F)(F)C(F)(F)C(F)(F)C(F)(C(F)(F)F)C(F)(F)F		-8.77	0.65	3.19	P	IN
QL00304	Diammonium 3,3,4,4,5,5,6,6,7,7,8,8,9,9,10,10,11,11,12,12,13,13,14,14,15,16,16,16-octacosafluoro-15-(trifluoromethyl)hexadecyl phosphate	93857-52-4	[NH4+].[NH4+].[O-]P(=O)([O-])OCCC(F)(F)C(F)(F)C(F)(F)C(F)(F)C(F)(F)C(F)(F)C(F)(F)C(F)(F)C(F)(F)C(F)(F)C(F)(F)C(F)(F)C(F)(C(F)(F)F)C(F)(F)F		-9.20	0.66	3.34	P	OUT
QL00305	Diammonium 3,3,4,4,5,5,6,6,7,7,8,8,9,9,10,10,11,11,12,12,13,13,14,14,15,15,16,16,17,18,18,18-dotriacontafluoro-17-(trifluoromethyl)octadecyl phosphate	93857-43-3	[NH4+].[NH4+].[O-]P(=O)([O-])OCCC(F)(F)C(F)(F)C(F)(F)C(F)(F)C(F)(F)C(F)(F)C(F)(F)C(F)(F)C(F)(F)C(F)(F)C(F)(F)C(F)(F)C(F)(F)C(F)(F)C(F)(C(F)(F)F)C(F)(F)F		-9.58	0.66	3.48	P	OUT
QL00306	Diammonium 3,3,4,4,5,5,6,6,7,7,8,8,9,9,10,10,11,11,12,12,13,13,14,14,15,15,16,16,17,17,18,18,18-tritriacontafluorooctadecyl phosphate	93857-48-8	[NH4+].[NH4+].[O-]P(=O)([O-])OCCC(F)(F)C(F)(F)C(F)(F)C(F)(F)C(F)(F)C(F)(F)C(F)(F)C(F)(F)C(F)(F)C(F)(F)C(F)(F)C(F)(F)C(F)(F)C(F)(F)C(F)(F)C(F)(F)F		-9.61	0.66	3.50	P	OUT

QL00307	Diammonium 3,3,4,4,5,5,6,6,7,7,8,8,9,9,10,10,11,11,12,12,13,13,14,14,15,15,16,16,16-nonacosafluorohexadecyl phosphate	93857-47-7	[NH4+].[NH4+].[O-]P(=O)([O-])OCCC(F)(F)C(F)(F)C(F)(F)C(F)(F)C(F)(F)C(F)(F)C(F)(F)C(F)(F)C(F)(F)C(F)(F)C(F)(F)C(F)(F)C(F)(F)C(F)(F)F		-9.23	0.66	3.37	P	OUT
QL00308	Diammonium 3,3,4,4,5,5,6,6,7,7,8,8,9,9,10,10,11,11,12,12,13,13,14,14,14-pentacosafluorotetradecyl phosphate	93857-46-6	[NH4+].[NH4+].[O-]P(=O)([O-])OCCC(F)(F)C(F)(F)C(F)(F)C(F)(F)C(F)(F)C(F)(F)C(F)(F)C(F)(F)C(F)(F)C(F)(F)C(F)(F)C(F)(F)F		-8.80	0.65	3.23	P	OUT
QL00309	Diammonium 3,3,4,4,5,5,6,6,7,7,8,8,9,9,10,10,11,11,12,12,12-henicosafluorododecyl phosphate	93857-45-5	[NH4+].[NH4+].[O-]P(=O)([O-])OCCC(F)(F)C(F)(F)C(F)(F)C(F)(F)C(F)(F)C(F)(F)C(F)(F)C(F)(F)C(F)(F)C(F)(F)F		-8.29	0.64	3.07	P	OUT
QL00310	Diammonium 3,3,4,4,5,5,6,6,7,7,8,8,9,9,10,10,10-heptadecafluorodecyl phosphate	93857-44-4	[NH4+].[NH4+].[O-]P(=O)([O-])OCCC(F)(F)C(F)(F)C(F)(F)C(F)(F)C(F)(F)C(F)(F)C(F)(F)C(F)(F)F		-7.67	0.63	2.90	P	OUT
QL00311	N-ethyl-N-[2-(phosphonooxy)ethyl]perfluorooctanesulfonamide diammonium salt	67969-69-1	[NH4+].[NH4+].CCN(CCOP([O-])([O-])=O)S(=O)(=O)C(F)(F)C(F)(F)C(F)(F)C(F)(F)C(F)(F)C(F)(F)C(F)(F)C(F)(F)F		-7.97	0.63	3.08	P	OUT
QL00312	Diammonium 2-[ethyl[(pentadecafluoroheptyl)sulphonyl]amino]ethyl phosphate	67939-98-4	[NH4+].[NH4+].CCN(CCOP(=O)([O-])[O-])S(=O)(=O)C(F)(F)C(F)(F)C(F)(F)C(F)(F)C(F)(F)C(F)(F)C(F)(F)F		-7.74	0.63	3.04	P	OUT
QL00313	Diammonium 4,4,5,5,6,6,7,7,8,8,9,9,10,11,11,11-hexadecafluoro-2-hydroxy-10-(trifluoromethyl)undecyl phosphate	63295-18-1	[NH4+].[NH4+].OC(COP(=O)([O-])[O-])CC(F)(F)C(F)(F)C(F)(F)C(F)(F)C(F)(F)C(F)(F)C(F)(C(F)(F)F)C(F)(F)F		-7.90	0.64	2.99	P	OUT
QL00314	Diammonium 4,4,5,5,6,6,7,7,8,8,9,9,10,10,11,11,12,13,13,13-icosafluoro-2-hydroxy-12-(trifluoromethyl)tridecyl phosphate	94200-50-7	[NH4+].[NH4+].OC(COP(=O)([O-])[O-])CC(F)(F)C(F)(F)C(F)(F)C(F)(F)C(F)(F)C(F)(F)C(F)(F)C(F)(F)C(F)(C(F)(F)F)C(F)(F)F		-8.46	0.64	3.15	P	OUT
QL00315	Diammonium 4,4,5,5,6,6,7,7,8,8,9,9,10,10,11,11,12,12,13,13,14,15,15,15-tetracosafluoro-2-hydroxy-14-(trifluoromethyl)pentadecyl phosphate	94200-51-8	[NH4+].[NH4+].OC(COP(=O)([O-])[O-])CC(F)(F)C(F)(F)C(F)(F)C(F)(F)C(F)(F)C(F)(F)C(F)(F)C(F)(F)C(F)(F)C(F)(F)C(F)(C(F)(F)F)C(F)(F)F		-8.94	0.65	3.31	P	OUT
QL00316	Diammonium 4,4,5,5,6,6,7,7,8,8,9,9,10,10,11,11,12,12,13,13,14,14,15,15,16,17,17,17-octacosafluoro-2-hydroxy-16-(trifluoromethyl)heptadecyl phosphate	94200-52-9	[NH4+].[NH4+].OC(COP(=O)([O-])[O-])CC(F)(F)C(F)(F)C(F)(F)C(F)(F)C(F)(F)C(F)(F)C(F)(F)C(F)(F)C(F)(F)C(F)(F)C(F)(F)C(F)(F)C(F)(C(F)(F)F)C(F)(F)F		-9.35	0.66	3.44	P	OUT
QL00317	Diammonium 4,4,5,5,6,6,7,7,8,8,9,9,10,10,11,11,12,12,13,13,14,14,15,15,16,16,17,17,18,19,19,19-dotriacontafluoro-2-hydroxy-18-(trifluoromethyl)nonadecyl phosphate	94200-53-0	[NH4+].[NH4+].OC(COP(=O)([O-])[O-])CC(F)(F)C(F)(F)C(F)(F)C(F)(F)C(F)(F)C(F)(F)C(F)(F)C(F)(F)C(F)(F)C(F)(F)C(F)(F)C(F)(F)C(F)(F)C(F)(F)C(F)(C(F)(F)F)C(F)(F)F		-9.71	0.66	3.57	P	OUT
QL00318	Diammonium 4,4,5,5,6,6,7,7,8,8,9,9,10,10,11,11,12,12,13,13,14,14,15,15,16,16,17,17,18,18,19,19,19-tritriacontafluoro-2-hydroxynonadecyl phosphate	94200-49-4	[NH4+].[NH4+].OC(COP(=O)([O-])[O-])CC(F)(F)C(F)(F)C(F)(F)C(F)(F)C(F)(F)C(F)(F)C(F)(F)C(F)(F)C(F)(F)C(F)(F)C(F)(F)C(F)(F)C(F)(F)C(F)(F)C(F)(F)C(F)(F)F		-9.74	0.66	3.60	P	OUT
QL00319	Diammonium 4,4,5,5,6,6,7,7,8,8,9,9,10,10,11,11,12,12,13,13,14,14,15,15,16,16,17,17,17-nonacosafluoro-2-hydroxyheptadecyl phosphate	94200-48-3	[NH4+].[NH4+].OC(COP(=O)([O-])[O-])CC(F)(F)C(F)(F)C(F)(F)C(F)(F)C(F)(F)C(F)(F)C(F)(F)C(F)(F)C(F)(F)C(F)(F)C(F)(F)C(F)(F)C(F)(F)C(F)(F)F		-9.38	0.66	3.47	P	OUT
QL00320	Diammonium 4,4,5,5,6,6,7,7,8,8,9,9,10,10,11,11,12,12,13,13,14,14,15,15,15-pentacosafluoro-2-hydroxypentadecyl phosphate	94200-47-2	[NH4+].[NH4+].OC(COP(=O)([O-])[O-])CC(F)(F)C(F)(F)C(F)(F)C(F)(F)C(F)(F)C(F)(F)C(F)(F)C(F)(F)C(F)(F)C(F)(F)C(F)(F)C(F)(F)F		-8.97	0.65	3.34	P	OUT
QL00321	Diammonium 4,4,5,5,6,6,7,7,8,8,9,9,10,10,11,11,12,12,13,13,13-henicosafluoro-2-hydroxytridecyl phosphate	94200-46-1	[NH4+].[NH4+].OC(COP(=O)([O-])[O-])CC(F)(F)C(F)(F)C(F)(F)C(F)(F)C(F)(F)C(F)(F)C(F)(F)C(F)(F)C(F)(F)C(F)(F)F		-8.49	0.64	3.20	P	OUT
QL00322	Diammonium 4,4,5,5,6,6,7,7,8,8,9,9,10,10,11,11,11-heptadecafluoro-2-hydroxyundecyl phosphate	94200-45-0	[NH4+].[NH4+].OC(COP(=O)([O-])[O-])CC(F)(F)C(F)(F)C(F)(F)C(F)(F)C(F)(F)C(F)(F)C(F)(F)C(F)(F)F		-7.92	0.63	3.04	P	OUT
QL00323	Ammonium 2,3,3,4,4,5,5,6,6,6-decafluoro-2-(pentafluoroethyl)hexanoate	13058-06-5	[NH4+].[O-]C(=O)C(F)(C(F)(F)C(F)(F)F)C(F)(F)C(F)(F)C(F)(F)C(F)(F)F		-7.01	0.65	2.28	P	IN
QL00324	Ammonium 2,2,3,3,4,4,5,5,6,7,7,7-dodecafluoro-6-(trifluoromethyl)heptanoate	19742-57-5	[NH4+].[O-]C(=O)C(F)(F)C(F)(F)C(F)(F)C(F)(F)C(F)(C(F)(F)F)C(F)(F)F		-6.99	0.65	2.28	P	IN
QL00325	Ammonium perfluoro(7-methyloctanoate)	3658-62-6	[NH4+].[O-]C(=O)C(F)(F)C(F)(F)C(F)(F)C(F)(F)C(F)(F)C(F)(C(F)(F)F)C(F)(F)F		-7.28	0.66	2.36	P	IN

QL00326	Ammonium tricosafluorododecanoate	3793-74-6	[NH4+].[O-]C(=O)C(F)(F)C(F)(F)C(F)(F)C(F)(F)C(F)(F)C(F)(F)C(F)(F)C(F)(F)C(F)(F)C(F)(F)C(F)(F)F		-8.57	0.67	2.85	P	IN
QL00327	Ammonium 2,2,3,3,4,4,5,5,6,6,7,7,8,8,9,9,10,10,11,11-icosafluoroundecanoate	1161876	[NH4+].[O-]C(=O)C(F)(F)C(F)(F)C(F)(F)C(F)(F)C(F)(F)C(F)(F)C(F)(F)C(F)(F)C(F)(F)C(F)F		-8.21	0.66	2.79	P	IN
QL00328	Ammonium nonadecafluorodecanoate	3108-42-7	[NH4+].[O-]C(=O)C(F)(F)C(F)(F)C(F)(F)C(F)(F)C(F)(F)C(F)(F)C(F)(F)C(F)(F)C(F)(F)F		-8.00	0.66	2.64	P	IN
QL00329	Ammonium perfluorononanoate	4149-60-4	[NH4+].[O-]C(=O)C(F)(F)C(F)(F)C(F)(F)C(F)(F)C(F)(F)C(F)(F)C(F)(F)C(F)(F)F		-7.79	0.66	2.58	P	IN
QL00330	Ammonium 2,2,3,3,4,4,5,5,6,6,7,7,8,8,9,9-hexadecafluorononan-1-oate	1868-86-6	[NH4+].[O-]C(=O)C(F)(F)C(F)(F)C(F)(F)C(F)(F)C(F)(F)C(F)(F)C(F)(F)C(F)F		-7.56	0.65	2.57	P	IN
QL00331	Ammonium 8-chlorotetradecafluorooctanoate	16557-94-1	[NH4+].[O-]C(=O)C(F)(F)C(F)(F)C(F)(F)C(F)(F)C(F)(F)C(F)(F)C(F)(F)Cl		-7.29	0.66	2.39	P	IN
QL00332	Ammonium 2,2,3,3,4,4,5,5,6,6,7,7-dodecafluoroheptanoate	376-34-1	[NH4+].[O-]C(=O)C(F)(F)C(F)(F)C(F)(F)C(F)(F)C(F)(F)C(F)F		-6.71	0.64	2.32	P	IN
QL00333	Ammonium perfluoropentanoate	68259-11-0	[NH4+].[O-]C(=O)C(F)(F)C(F)(F)C(F)(F)C(F)(F)F		-5.94	0.63	2.02	P	IN
QL00334	Ammonium 2,2,3,3,4,4,5,5-octafluorovalerate	22715-45-3	[NH4+].[O-]C(=O)C(F)(F)C(F)(F)C(F)(F)C(F)F		-5.52	0.62	2.03	P	OUT
QL00335	Ammonium 4,8-dioxa-3H-perfluorononanoate	958445-44-8	[NH4+].[O-]C(=O)C(F)(F)C(F)OC(F)(F)C(F)(F)C(F)(F)OC(F)(F)F		-6.97	0.63	2.62	P	OUT
QL00336	Acetic acid, 2,2-difluoro-2-[[2,2,4,5-tetrafluoro-5-(trifluoromethoxy)-1,3-dioxolan-4-yl]oxy]-, ammonium salt (11)	1190931-27-1	[NH4+].[O-]C(=O)C(F)(F)OC1(F)OC(F)(F)OC1(F)OC(F)(F)F		-6.21	0.62	2.34	P	OUT
QL00337	Ammonium 2,3,3,3-tetrafluoro-2-[(undecafluoropentyl)oxy]propanoate	510774-81-9	[NH4+].[O-]C(=O)C(F)(OC(F)(F)C(F)(F)C(F)(F)C(F)(F)C(F)(F)F)C(F)(F)F		-7.08	0.65	2.35	P	IN
QL00338	Ammonium 2,3,3,3-tetrafluoro-2-(nonafluorobutoxy)propanoate	96513-97-2	[NH4+].[O-]C(=O)C(F)(OC(F)(F)C(F)(F)C(F)(F)C(F)(F)F)C(F)(F)F		-6.76	0.65	2.27	P	IN
QL00339	Ammonium perfluoro-2-methyl-3-oxahexanoate	62037-80-3	[NH4+].[O-]C(=O)C(F)(OC(F)(F)C(F)(F)C(F)(F)F)C(F)(F)F		-6.08	0.64	2.03	P	IN
QL00340	Ammonium 2,3,3,3-tetrafluoro-2-[1,1,2,3,3,3-hexafluoro-2-(trifluoromethoxy)propoxy]propanoate	510774-77-3	[NH4+].[O-]C(=O)C(F)(OC(F)(F)C(F)(OC(F)(F)F)C(F)(F)F)C(F)(F)F		-6.45	0.64	2.26	P	IN
QL00341	Ammonium 2-{[(nonafluorobutyl)sulfonyl]amino}ethanolate	484024-67-1	[NH4+].[O-]CCNS(=O)(=O)C(F)(F)C(F)(F)C(F)(F)C(F)(F)F		-6.29	0.62	2.42	P	OUT
QL00342	Ammonium bis[3,3,4,4,5,5,6,6,7,7,8,8,9,10,10,10-hexadecafluoro9-(trifluoromethyl)decyl] phosphate	93776-24-0	[NH4+].[O-]P(=O)(OCCC(F)(F)C(F)(F)C(F)(F)C(F)(F)C(F)(F)C(F)(F)C(F)(C(F)(F)F)C(F)(F)F)OCCC(F)(F)C(F)(F)C(F)(F)C(F)(F)C(F)(F)C(F)(F)C(F)(C(F)(F)F)C(F)(F)F		-9.86	0.67	3.54	P	OUT
QL00343	Ammonium bis[3,3,4,4,5,5,6,6,7,7,8,8,9,9,10,10,11,12,12,12-icosafluoro-11-(trifluoromethyl)dodecyl] phosphate	93776-25-1	[NH4+].[O-]P(=O)(OCCC(F)(F)C(F)(F)C(F)(F)C(F)(F)C(F)(F)C(F)(F)C(F)(F)C(F)(F)C(F)(C(F)(F)F)C(F)(F)F)OCCC(F)(F)C(F)(F)C(F)(F)C(F)(F)C(F)(F)C(F)(F)C(F)(F)C(F)(C(F)(F)F)C(F)(F)F		-10.44	0.67	3.76	P	OUT
QL00344	Ammonium bis(3,3,4,4,5,5,6,6,7,7,8,8,9,9,10,10,11,11,12,12,13,14,14,14-tetracosafluoro-13-(trifluoromethyl)tetradecyl) phosphate	93776-26-2	[NH4+].[O-]P(=O)(OCCC(F)(F)C(F)(F)C(F)(F)C(F)(F)C(F)(F)C(F)(F)C(F)(F)C(F)(F)C(F)(F)C(F)(F)C(F)(C(F)(F)F)C(F)(F)F)OCCC(F)(F)C(F)(F)C(F)(F)C(F)(F)C(F)(F)C(F)(F)C(F)(F)C(F)(F)C(F)(F)C(F)(F)C(F)(C(F)(F)F)C(F)(F)F		-10.93	0.68	3.96	P	OUT
QL00345	Ammonium bis[3,3,4,4,5,5,6,6,7,7,8,8,9,9,10,10,11,11,12,12,13,13,14,14,15,16,16,16-octacosafluoro-15-(trifluoromethyl)hexadecyl] phosphate	93776-27-3	[NH4+].[O-]P(=O)(OCCC(F)(F)C(F)(F)C(F)(F)C(F)(F)C(F)(F)C(F)(F)C(F)(F)C(F)(F)C(F)(F)C(F)(F)C(F)(F)C(F)(F)C(F)(C(F)(F)F)C(F)(F)F)OCCC(F)(F)C(F)(F)C(F)(F)C(F)(F)C(F)(F)C(F)(F)C(F)(F)C(F)(F)C(F)(F)C(F)(F)C(F)(F)C(F)(F)C(F)(C(F)(F)F)C(F)(F)F		-11.35	0.68	4.14	P	OUT
QL00346	Ammonium bis[3,3,4,4,5,5,6,6,7,7,8,8,9,9,10,10,11,11,12,12,13,13,14,14,15,15,16,16,17,18,18,18-dotriacontafluoro-17-(trifluoromethyl)octadecyl] phosphate	93776-28-4	[NH4+].[O-]P(=O)(OCCC(F)(F)C(F)(F)C(F)(F)C(F)(F)C(F)(F)C(F)(F)C(F)(F)C(F)(F)C(F)(F)C(F)(F)C(F)(F)C(F)(F)C(F)(F)C(F)(F)C(F)(C(F)(F)F)C(F)(F)F)OCCC(F)(F)C(F)(F)C(F)(F)C(F)(F)C(F)(F)C(F)(F)C(F)(F)C(F)(F)C(F)(F)C(F)(F)C(F)(F)C(F)(F)C(F)(F)C(F)(F)C(F)(C(F)(F)F)C(F)(F)F		-11.71	0.68	4.29	P	OUT
QL00347	Ammonium bis[3,3,4,4,5,5,6,6,7,7,8,8,9,9,10,10,11,11,12,12,13,13,14,14,15,15,16,16,17,17,18,18,18-tritriacontafluorooctadecyl] phosphate	93776-23-9	[NH4+].[O-]P(=O)(OCCC(F)(F)C(F)(F)C(F)(F)C(F)(F)C(F)(F)C(F)(F)C(F)(F)C(F)(F)C(F)(F)C(F)(F)C(F)(F)C(F)(F)C(F)(F)C(F)(F)C(F)(F)C(F)(F)F)OCCC(F)(F)C(F)(F)C(F)(F)C(F)(F)C(F)(F)C(F)(F)C(F)(F)C(F)(F)C(F)(F)C(F)(F)C(F)(F)C(F)(F)C(F)(F)C(F)(F)C(F)(F)F		-11.79	0.68	4.33	P	OUT

QL00348	Ammonium bis[3,3,4,4,5,5,6,6,7,7,8,8,9,9,10,10,11,11,12,12,13,13,14,14,15,15,16,16,16-nonacosafluorohexadecyl] phosphate	93777-13-0	[NH4+].[O-]P(=O)(OCCC(F)(F)C(F)(F)C(F)(F)C(F)(F)C(F)(F)C(F)(F)C(F)(F)C(F)(F)C(F)(F)C(F)(F)C(F)(F)C(F)(F)C(F)(F)C(F)(F)F)OCCC(F)(F)C(F)(F)C(F)(F)C(F)(F)C(F)(F)C(F)(F)C(F)(F)C(F)(F)C(F)(F)C(F)(F)C(F)(F)C(F)(F)C(F)(F)C(F)(F)F		-11.43	0.68	4.18	P	OUT
QL00349	Ammonium bis(3,3,4,4,5,5,6,6,7,7,8,8,9,9,10,10,11,11,12,12,13,13,14,14,14-pentacosafluorotetradecyl) phosphate	93776-22-8	[NH4+].[O-]P(=O)(OCCC(F)(F)C(F)(F)C(F)(F)C(F)(F)C(F)(F)C(F)(F)C(F)(F)C(F)(F)C(F)(F)C(F)(F)C(F)(F)C(F)(F)F)OCCC(F)(F)C(F)(F)C(F)(F)C(F)(F)C(F)(F)C(F)(F)C(F)(F)C(F)(F)C(F)(F)C(F)(F)C(F)(F)C(F)(F)F		-11.03	0.68	4.01	P	OUT
QL00350	Ammonium bis[3,3,4,4,5,5,6,6,7,7,8,8,9,9,10,10,11,11,12,12,12-henicosafluorododecyl] phosphate	93776-21-7	[NH4+].[O-]P(=O)(OCCC(F)(F)C(F)(F)C(F)(F)C(F)(F)C(F)(F)C(F)(F)C(F)(F)C(F)(F)C(F)(F)F)OCCC(F)(F)C(F)(F)C(F)(F)C(F)(F)C(F)(F)C(F)(F)C(F)(F)C(F)(F)C(F)(F)F		-10.55	0.67	3.82	P	OUT
QL00351	Ammonium bis(3,3,4,4,5,5,6,6,7,7,8,8,9,9,10,10,10-heptadecafluorodecyl) phosphate	93776-20-6	[NH4+].[O-]P(=O)(OCCC(F)(F)C(F)(F)C(F)(F)C(F)(F)C(F)(F)C(F)(F)C(F)(F)F)OCCC(F)(F)C(F)(F)C(F)(F)C(F)(F)C(F)(F)C(F)(F)C(F)(F)F		-9.98	0.67	3.61	P	OUT
QL00352	Ammonium perfluorodecanesulfonate	67906-42-7	[NH4+].[O-]S(=O)(=O)C(F)(F)C(F)(F)C(F)(F)C(F)(F)C(F)(F)C(F)(F)C(F)(F)C(F)(F)C(F)(F)C(F)(F)F		-8.56	0.67	2.79	P	IN
QL00353	Ammonium nonadecafluorononanesulphonate	17202-41-4	[NH4+].[O-]S(=O)(=O)C(F)(F)C(F)(F)C(F)(F)C(F)(F)C(F)(F)C(F)(F)C(F)(F)C(F)(F)C(F)(F)F		-8.19	0.67	2.63	P	IN
QL00354	Ammonium heptadecafluorooctanesulphonate	29081-56-9	[NH4+].[O-]S(=O)(=O)C(F)(F)C(F)(F)C(F)(F)C(F)(F)C(F)(F)C(F)(F)C(F)(F)C(F)(F)F		-7.99	0.67	2.57	P	IN
QL00355	Ammonium 1,1,2,2,3,3,4,4,5,5,6,6,7,7,7-pentadecafluoroheptane-1-sulphonate	68259-07-4	[NH4+].[O-]S(=O)(=O)C(F)(F)C(F)(F)C(F)(F)C(F)(F)C(F)(F)C(F)(F)C(F)(F)F		-7.52	0.67	2.38	P	IN
QL00356	Ammonium perfluorohexanesulfonate	68259-08-5	[NH4+].[O-]S(=O)(=O)C(F)(F)C(F)(F)C(F)(F)C(F)(F)C(F)(F)C(F)(F)F		-7.26	0.66	2.31	P	IN
QL00357	Ammonium 1,1,2,2,3,3,4,4,5,5,5-undecafluoropentane-1-sulphonate	68259-09-6	[NH4+].[O-]S(=O)(=O)C(F)(F)C(F)(F)C(F)(F)C(F)(F)C(F)(F)F		-6.66	0.66	2.08	P	IN
QL00358	Ammonium 1,1,2,2,3,3,4,4,4-nonafluorobutane-1-sulphonate	68259-10-9	[NH4+].[O-]S(=O)(=O)C(F)(F)C(F)(F)C(F)(F)C(F)(F)F		-6.29	0.65	2.00	P	IN
QL00359	Ammonium 1,1,2,2-tetrafluoro-2-{[1,1,1,2,3,3-hexafluoro-3-(pentafluoroethoxy)propan-2-yl]oxy}ethane-1-sulfonate	93644-65-6	[NH4+].[O-]S(=O)(=O)C(F)(F)C(F)(F)OC(F)(C(F)(F)F)C(F)(F)OC(F)(F)C(F)(F)F		-7.61	0.66	2.56	P	IN
QL00360	82 Fluorotelomer sulfonate ammonium salt	149724-40-3	[NH4+].[O-]S(=O)(=O)CCC(F)(F)C(F)(F)C(F)(F)C(F)(F)C(F)(F)C(F)(F)C(F)(F)C(F)(F)F		-8.14	0.66	2.80	P	IN
QL00361	62 Fluorotelomer sulphonate ammonium	59587-39-2	[NH4+].[O-]S(=O)(=O)CCC(F)(F)C(F)(F)C(F)(F)C(F)(F)C(F)(F)C(F)(F)F		-7.46	0.65	2.58	P	IN
QL00362	4-(Nonafluorobutoxy)butanesulfonic acid ammonium salt	484023-69-0	[NH4+].[O-]S(=O)(=O)CCCCOC(F)(F)C(F)(F)C(F)(F)C(F)(F)F		-6.65	0.62	2.63	P	OUT
QL00363	1H,1H,2H,2H-Perfluorotetradecanol sulfate ammonium salt	63225-59-2	[NH4+].[O-]S(=O)(=O)OCCC(F)(F)C(F)(F)C(F)(F)C(F)(F)C(F)(F)C(F)(F)C(F)(F)C(F)(F)C(F)(F)C(F)(F)C(F)(F)C(F)(F)F		-9.12	0.67	3.19	P	OUT
QL00364	1H,1H,2H,2H-Perfluorododecanol sulfate ammonium salt	63225-58-1	[NH4+].[O-]S(=O)(=O)OCCC(F)(F)C(F)(F)C(F)(F)C(F)(F)C(F)(F)C(F)(F)C(F)(F)C(F)(F)C(F)(F)C(F)(F)F		-8.66	0.66	3.03	P	IN
QL00365	1H,1H,2H,2H-Perfluorodecanol sulfate ammonium salt	63225-57-0	[NH4+].[O-]S(=O)(=O)OCCC(F)(F)C(F)(F)C(F)(F)C(F)(F)C(F)(F)C(F)(F)C(F)(F)C(F)(F)F		-8.11	0.65	2.85	P	IN
QL00366	1H,1H,2H,2H-Perfluorooctanol sulfate ammonium salt	63225-56-9	[NH4+].[O-]S(=O)(=O)OCCC(F)(F)C(F)(F)C(F)(F)C(F)(F)C(F)(F)C(F)(F)F		-7.42	0.64	2.64	P	IN
QL00367	Ammonium N-ethyl-N-[(heptadecafluorooctyl)sulphonyl]glycinate	2991-52-8	[NH4+].CCN(CC(=O)[O-])S(=O)(=O)C(F)(F)C(F)(F)C(F)(F)C(F)(F)C(F)(F)C(F)(F)C(F)(F)C(F)(F)F		-7.96	0.65	2.85	P	IN
QL00368	2-Hydroxy-3-[ethyl[(nonafluorobutyl)sulfonyl]amino]propanesulfonic acid monoammonium salt	484023-83-8	[NH4+].CCN(CC(O)CS([O-])(=O)=O)S(=O)(=O)C(F)(F)C(F)(F)C(F)(F)C(F)(F)F		-7.00	0.63	2.65	P	OUT
QL00369	Ammonium bis[2-[ethyl[(pentadecafluoroheptyl)sulphonyl]amino]ethyl] phosphate	67939-97-3	[NH4+].CCN(CCOP(=O)([O-])OCCN(CC)S(=O)(=O)C(F)(F)C(F)(F)C(F)(F)C(F)(F)C(F)(F)C(F)(F)C(F)(F)F)S(=O)(=O)C(F)(F)C(F)(F)C(F)(F)C(F)(F)C(F)(F)C(F)(F)C(F)(F)F		-9.85	0.66	3.70	P	OUT

QL00370	Ammonium N,N-bis(perfluorobutanesulfonyl)amide	129318-48-5	[NH4+].FC(F)(F)C(F)(F)C(F)(F)C(F)(F)S(=O)(=O)[N-]S(=O)(=O)C(F)(F)C(F)(F)C(F)(F)C(F)(F)F		-8.31	0.67	2.75	P	IN
QL00371	2-Hydroxy-3-[(2-hydroxyethyl)[(nonafluorobutyl)sulfonyl]amino]propanesulfonic acid monoammonium salt	484023-82-7	[NH4+].OCCN(CC(O)CS([O-])(=O)=O)S(=O)(=O)C(F)(F)C(F)(F)C(F)(F)C(F)(F)F		-6.80	0.62	2.64	P	OUT
QL00372	3,3,4,4,5,5,6,6,6-nonafluoro-1-nitrohex-1-ene	306935-66-0	[O-][N+](=O)C=CC(F)(F)C(F)(F)C(F)(F)C(F)(F)F		-6.77	0.65	2.19	P	IN
QL00373	1H,2H,2H-perfluorooctanal (2,4-dinitrophenyl)hydrazone	128917-19-1	[O-][N+](=O)C1=CC(=C(NN=CCC(F)(F)C(F)(F)C(F)(F)C(F)(F)C(F)(F)C(F)(F)F)C=C1)[N+]([O-])=O		-8.09	0.64	3.01	P	OUT
QL00374	1-(1,1,1,2,3,3,3-Heptafluoropropan-2-yl)-3,5-dinitrobenzene	24813-57-8	[O-][N+](=O)C1=CC(=CC(=C1)[N+]([O-])=O)C(F)(C(F)(F)F)C(F)(F)F		-5.44	0.64	1.72	P	IN
QL00375	Butanamide, N-(2-bromo-4-nitrophenyl)-2,2,3,3,4,4,4-heptafluoro-	111189-67-4	[O-][N+](=O)C1=CC(Br)=C(NC(=O)C(F)(F)C(F)(F)C(F)(F)F)C=C1		-7.02	0.64	2.52	P	OUT
QL00376	3-Nitrophenyl heptafluorobutanoate	52512-15-9	[O-][N+](=O)C1=CC(OC(=O)C(F)(F)C(F)(F)C(F)(F)F)=CC=C1		-6.69	0.63	2.42	P	OUT
QL00377	N-(4-Nitrophenyl)perfluorobutanamide	3869-06-5	[O-][N+](=O)C1=CC=C(NC(=O)C(F)(F)C(F)(F)C(F)(F)F)C=C1		-6.95	0.63	2.53	P	OUT
QL00378	4-Nitrophenyl heptafluorobutanoate	1799-92-4	[O-][N+](=O)C1=CC=C(OC(=O)C(F)(F)C(F)(F)C(F)(F)F)C=C1		-6.95	0.63	2.53	P	OUT
QL00379	6-Nitro-2-(pentafluoroethyl)-1H-benzimidazole	4229-01-0	[O-][N+](=O)C1=CC2=C(C=C1)N=C(N2)C(F)(F)C(F)(F)F		-6.43	0.63	2.29	P	OUT
QL00380	1,2,4-Trifluoro-3,5-bis(1,1,1,2,3,3,3-heptafluoropropan-2-yl)-6-nitrobenzene	20017-51-0	[O-][N+](=O)c1c(F)c(F)c(c(F)c1C(F)(C(F)(F)F)C(F)(F)F)C(F)(C(F)(F)F)C(F)(F)F		-6.53	0.66	1.97	P	IN
QL00381	1,2,4,5-Tetrafluoro-3-(1,1,1,2,3,3,3-heptafluoropropan-2-yl)-6-nitrobenzene	20017-50-9	[O-][N+](=O)c1c(F)c(F)c(c(F)c1F)C(F)(C(F)(F)F)C(F)(F)F		-6.58	0.64	2.19	P	IN
QL00382	1,4-Dichloro-2-(1,1,2,3,3,3-hexafluoropropoxy)-5-nitrobenzene	130841-23-5	[O-][N+](=O)c1cc(Cl)c(OC(F)(F)C(F)C(F)(F)F)cc1Cl		-6.59	0.63	2.45	P	OUT
QL00383	2,2,3,3,4,4,5,5,5-Nonafluoropentyl 2,5,7-trinitro-9-oxo-9H-fluorene-4-carboxylate	253605-71-9	[O-][N+](=O)c1cc2C(=O)c3cc(cc(c3-c2c(c1)C(=O)OCC(F)(F)C(F)(F)C(F)(F)C(F)(F)F)[N+]([O-])=O)[N+]([O-])=O		-7.68	0.63	2.89	P	OUT
QL00384	2,2,3,3,4,4,4-Heptafluoro-1-(4-nitrophenyl)butan-1-one	144459-62-1	[O-][N+](=O)c1ccc(cc1)C(=O)C(F)(F)C(F)(F)C(F)(F)F		-6.95	0.64	2.47	P	IN
QL00385	1-(1,1,1,2,3,3,3-Heptafluoropropan-2-yl)-4-nitrobenzene	2396-14-7	[O-][N+](=O)c1ccc(cc1)C(F)(C(F)(F)F)C(F)(F)F		-6.20	0.64	2.10	P	IN
QL00386	1-Nitro-4-(nonafluorobutyl)benzene	196932-95-3	[O-][N+](=O)c1ccc(cc1)C(F)(F)C(F)(F)C(F)(F)C(F)(F)F		-7.21	0.65	2.46	P	IN
QL00387	1-(Heptafluoropropyl)-4-nitrobenzene	1300746-85-3	[O-][N+](=O)c1ccc(cc1)C(F)(F)C(F)(F)C(F)(F)F		-6.66	0.64	2.29	P	IN
QL00388	2,2,3,3,4,4,5,5,6,6,7,7,8,8,9,9,9-Heptadecafluoro-N-(4-nitrophenyl)nonanamide	164796-46-7	[O-][N+](=O)c1ccc(NC(=O)C(F)(F)C(F)(F)C(F)(F)C(F)(F)C(F)(F)C(F)(F)C(F)(F)C(F)(F)F)cc1		-8.78	0.66	3.09	P	IN
QL00389	2,2,3,3,4,4,5,5,6,6,7,7,7-Tridecafluoro-N-(4-nitrophenyl)heptanamide	164796-44-5	[O-][N+](=O)c1ccc(NC(=O)C(F)(F)C(F)(F)C(F)(F)C(F)(F)C(F)(F)C(F)(F)F)cc1		-8.21	0.65	2.90	P	IN
QL00390	2,2,3,3,4,4,5,5,5-Nonafluoro-N-(4-nitrophenyl)pentanamide	150333-62-3	[O-][N+](=O)c1ccc(NC(=O)C(F)(F)C(F)(F)C(F)(F)C(F)(F)F)cc1		-7.45	0.64	2.68	P	IN
QL00391	N-(2,4-Dinitrophenyl)-2,3,3,3-tetrafluoro-2-(heptafluoropropoxy)propanamide	105936-30-9	[O-][N+](=O)c1ccc(NC(=O)C(F)(OC(F)(F)C(F)(F)C(F)(F)F)C(F)(F)F)c(c1)[N+]([O-])=O		-7.55	0.64	2.71	P	IN
QL00392	1-Nitro-4-[(2,2,3,3,4,4,5,5,6,6,6-undecafluorohexyl)oxy]benzene	183997-46-8	[O-][N+](=O)c1ccc(OCC(F)(F)C(F)(F)C(F)(F)C(F)(F)C(F)(F)F)cc1		-7.67	0.65	2.72	P	IN
QL00393	1-(2,2,3,3,4,4,4-Heptafluorobutoxy)-4-nitrobenzene	109230-68-4	[O-][N+](=O)c1ccc(OCC(F)(F)C(F)(F)C(F)(F)F)cc1		-6.87	0.63	2.53	P	OUT
QL00394	2-(Heptafluoropropyl)-6-nitro-1H-benzimidazole	6826-38-6	[O-][N+](=O)c1ccc2nc(Nc2c1)C(F)(F)C(F)(F)C(F)(F)F		-7.01	0.64	2.46	P	IN
QL00395	1-Nitro-3-(undecafluoropentyl)benzene	1463530-16-6	[O-][N+](=O)c1cccc(c1)C(F)(F)C(F)(F)C(F)(F)C(F)(F)C(F)(F)F		-7.36	0.65	2.47	P	IN
QL00396	1-Nitro-3-(nonafluorobutyl)benzene	2398-76-7	[O-][N+](=O)c1cccc(c1)C(F)(F)C(F)(F)C(F)(F)C(F)(F)F		-6.77	0.65	2.26	P	IN
QL00397	1,1,1,2,2,3,3-heptafluoro-5-nitro-4-(nitromethyl)pentane	355-91-9	[O-][N+](=O)CC(C[N+]([O-])=O)C(F)(F)C(F)(F)C(F)(F)F		-5.57	0.63	1.89	P	OUT
QL00398	3,3,4,4,5,5,6,6,6-Nonafluorohexyl nitrate	34839-44-6	[O-][N+](=O)OCCC(F)(F)C(F)(F)C(F)(F)C(F)(F)F		-6.70	0.64	2.27	P	IN
QL00399	Perfluorooctanoate ion(1-)	45285-51-6	[O-]C(=O)C(F)(F)C(F)(F)C(F)(F)C(F)(F)C(F)(F)C(F)(F)C(F)(F)F		-7.72	0.68	2.24	P	IN
QL00400	Perfluoroheptanoate	120885-29-2	[O-]C(=O)C(F)(F)C(F)(F)C(F)(F)C(F)(F)C(F)(F)C(F)(F)F		-7.50	0.68	2.16	P	IN
QL00401	1-Propanaminium, 3-[[(heptadecafluorooctyl)sulfonyl]amino]-N,N,N-trimethyl-, sulfate (21)	70225-26-2	[O-]S([O-])(=O)=O.C[N+](C)(C)CCCNS(=O)(=O)C(F)(F)C(F)(F)C(F)(F)C(F)(F)C(F)(F)C(F)(F)C(F)(F)C(F)(F)F.C[N+](C)(C)CCCNS(=O)(=O)C(F)(F)C(F)(F)C(F)(F)C(F)(F)C(F)(F)C(F)(F)C(F)(F)C(F)(F)F		-9.03	0.67	3.14	P	OUT
QL00402	Perfluorooctanesulfonate	45298-90-6	[O-]S(=O)(=O)C(F)(F)C(F)(F)C(F)(F)C(F)(F)C(F)(F)C(F)(F)C(F)(F)C(F)(F)F		-8.37	0.69	2.44	P	IN
QL00403	Perfluorohexanesulfonate	108427-53-8	[O-]S(=O)(=O)C(F)(F)C(F)(F)C(F)(F)C(F)(F)C(F)(F)C(F)(F)F		-7.74	0.69	2.15	P	IN

QL00404	(4-Cyclohexylphenyl)(diphenyl)sulfanium nonafluorobutane-1-sulfonate	425670-64-0	[O-]S(=O)(=O)C(F)(F)C(F)(F)C(F)(F)C(F)(F)F.C1CCC(CC1)c1ccc(cc1)[S+](c1ccccc1)c1ccccc1		-7.51	0.61	3.14	P	OUT
QL00405	Bis(4-tert-butylphenyl)iodanium nonafluorobutane-1-sulfonate	194999-85-4	[O-]S(=O)(=O)C(F)(F)C(F)(F)C(F)(F)C(F)(F)F.CC(C)(C)C1=CC=C([I+]C2=CC=C(C=C2)C(C)(C)C)C=C1		-8.44	0.65	3.13	P	OUT
QL00406	[4-(2-tert-Butoxy-2-oxoethoxy)phenyl](diphenyl)sulfanium nonafluorobutane-1-sulfonate	857285-80-4	[O-]S(=O)(=O)C(F)(F)C(F)(F)C(F)(F)C(F)(F)F.CC(C)(C)OC(=O)COC1=CC=C(C=C1)[S+](C1=CC=CC=C1)C1=CC=CC=C1		-8.23	0.62	3.42	P	OUT
QL00407	1-Butanesulfonic acid, 1,1,2,2,3,3,4,4,4-nonafluoro-, ion(1-), 1-(4-butoxy-1-naphthalenyl)tetrahydrothiophenium	209482-18-8	[O-]S(=O)(=O)C(F)(F)C(F)(F)C(F)(F)C(F)(F)F.CCCCOC1=CC=C([S]2CCCC2)C2=CC=CC=C12		-7.28	0.61	2.99	P	OUT
QL00408	(1,1,1,2,3,3,3-Heptafluoropropan-2-yl)(phenyl)iodanium trifluoromethanesulfonate	82959-18-0	[O-]S(=O)(=O)C(F)(F)F.FC(F)(F)C(F)([I+]C1=CC=CC=C1)C(F)(F)F		-6.92	0.64	2.42	P	IN
QL00409	(Heptafluoropropyl)(phenyl)iodanium trifluoromethanesulfonate	77758-79-3	[O-]S(=O)(=O)C(F)(F)F.FC(F)(F)C(F)(F)C(F)(F)[I+]C1=CC=CC=C1		-7.34	0.64	2.61	P	IN
QL00410	Phenyl(tridecafluorohexyl)iodanium trifluoromethanesulfonate	77758-84-0	[O-]S(=O)(=O)C(F)(F)F.FC(F)(F)C(F)(F)C(F)(F)C(F)(F)C(F)(F)C(F)(F)[I+]C1=CC=CC=C1		-8.33	0.66	2.90	P	IN
QL00411	(Heptadecafluorooctyl)(phenyl)iodanium trifluoromethanesulfonate	77758-89-5	[O-]S(=O)(=O)C(F)(F)F.FC(F)(F)C(F)(F)C(F)(F)C(F)(F)C(F)(F)C(F)(F)C(F)(F)C(F)(F)[I+]C1=CC=CC=C1		-8.84	0.66	3.08	P	OUT
QL00412	Pyridinium, 1-(2,2,3,3,4,4,5,5,6,6,7,7,8,8,8-pentadecafluorooctyl)-, trifluoromethanesulfonate	25061-59-0	[O-]S(=O)(=O)C(F)(F)F.FC(F)(F)C(F)(F)C(F)(F)C(F)(F)C(F)(F)C(F)(F)C(F)(F)C[N+]1=CC=CC=C1		-8.69	0.66	3.04	P	IN
QL00413	62 Fluorotelomer sulfonate	425670-75-3	[O-]S(=O)(=O)CCC(F)(F)C(F)(F)C(F)(F)C(F)(F)C(F)(F)C(F)(F)F		-7.90	0.67	2.44	P	IN
QL00414	3-((Perfluorooctylsulfonyl)-2-hydroxy-3-sulfopropylamino)-N-(2-hydroxyethyl)-N,N-dimethyl-1-propanaminium monosodium hydroxide	94133-91-2	[OH-].[Na+].C[N+](C)(CCO)CCCN(CC(O)CS([O-])(=O)=O)S(=O)(=O)C(F)(F)C(F)(F)C(F)(F)C(F)(F)C(F)(F)C(F)(F)C(F)(F)C(F)(F)F		-8.05	0.63	3.20	P	OUT
QL00415	[3-[[(heptadecafluorooctyl)sulphonyl]amino]propyl]trimethylammonium hydroxide	85665-67-4	[OH-].C[N+](C)(C)CCCNS(=O)(=O)C(F)(F)C(F)(F)C(F)(F)C(F)(F)C(F)(F)C(F)(F)C(F)(F)C(F)(F)F		-8.46	0.65	3.03	P	IN
QL00416	(2-carboxyethyl)-3-[[4,4,5,5,6,6,7,7,8,8,9,9,10,11,11,11-hexadecafluoro-2-hydroxy-10-(trifluoromethyl)undecyl]amino]propyldimethylammonium hydroxide	73353-25-0	[OH-].C[N+](C)(CCCNCC(O)CC(F)(F)C(F)(F)C(F)(F)C(F)(F)C(F)(F)C(F)(F)C(F)(C(F)(F)F)C(F)(F)F)CCC(=O)O		-8.65	0.63	3.40	P	OUT
QL00417	2-Carboxyethylbis(2-hydroxyethyl)-3-((perfluoro-1-oxooctyl)amino)propylammonium hydroxide	39186-68-0	[OH-].OCC[N+](CCO)(CCCNC(=O)C(F)(F)C(F)(F)C(F)(F)C(F)(F)C(F)(F)C(F)(F)C(F)(F)F)CCC(O)=O		-7.63	0.61	3.19	P	OUT
QL00418	Lead, bis(6,6,7,7,8,8,8-heptafluoro-2,2-dimethyl-3,5-octanedionato-βO,βO')-, (T-4)- (9CI)	21600-78-2	[Pb++].CC(C)(C)C([O-])=CC(=O)C(F)(F)C(F)(F)C(F)(F)F.CC(C)(C)C([O-])=CC(=O)C(F)(F)C(F)(F)C(F)(F)F		-6.71	0.64	2.27	P	IN
QL00419	Praseodymium, tris[6,6,7,7,8,8,8-heptafluoro-2,2-di(methyl-d3)-3,5-octanedion-1,1,1-d3-ato-O,O']- (9CI)	42942-19-8	[Pr+3].[2H]C([2H])([2H])C(C(=O)C=C(/[O-])C(F)(F)C(F)(F)C(F)(F)F)(C([2H])([2H])[2H])C([2H])([2H])[2H].[2H]C([2H])([2H])C(C(=O)C=C(/[O-])C(F)(F)C(F)(F)C(F)(F)F)(C([2H])([2H])[2H])C([2H])([2H])[2H].[2H]C([2H])([2H])C(C(=O)C=C(/[O-])C(F)(F)C(F)(F)C(F)(F)F)(C([2H])([2H])[2H])C([2H])([2H])[2H]		-6.81	0.65	2.24	P	IN
QL00420	Praseodymium, tris(6,6,7,7,8,8,8-heptafluoro-2,2-dimethyl-3,5-octanedionato-βO3,βO5)-	17978-77-7	[Pr+3].CC(C)(C)C([O-])=CC(=O)C(F)(F)C(F)(F)C(F)(F)F.CC(C)(C)C([O-])=CC(=O)C(F)(F)C(F)(F)C(F)(F)F.CC(C)(C)C([O-])=CC(=O)C(F)(F)C(F)(F)C(F)(F)F		-6.81	0.65	2.24	P	IN
QL00421	Praseodymium, tris[3-[2,2,3,3,4,4,4-heptafluoro-1-(oxo-βO)butyl]-1,7,7-trimethylbicyclo[2.2.1]heptan-2-onato-βO]-	38832-94-9	[Pr+3].CC1(C)C2CCC1(C)C(=O)[C-]2C(=O)C(F)(F)C(F)(F)C(F)(F)F.CC1(C)C2CCC1(C)C(=O)[C-]2C(=O)C(F)(F)C(F)(F)C(F)(F)F.CC1(C)C2CCC1(C)C(=O)[C-]2C(=O)C(F)(F)C(F)(F)C(F)(F)F		-7.01	0.66	2.18	P	IN
QL00422	Rhodium(II) perfluorobutyrate dimer	73755-28-9	[Rh++].[Rh++].[O-]C(=O)C(F)(F)C(F)(F)C(F)(F)F.[O-]C(=O)C(F)(F)C(F)(F)C(F)(F)F.[O-]C(=O)C(F)(F)C(F)(F)C(F)(F)F.[O-]C(=O)C(F)(F)C(F)(F)C(F)(F)F		-5.42	0.64	1.63	P	IN
QL00423	5-(Perfluoropropyl)-2,2-dimethyl-3,5-pentanedione strontium(2+) (21)	125009-58-7	[Sr++].CC(C)(C)C(=O)CC(=O)C(F)(F)C(F)(F)C(F)(F)F.CC(C)(C)C(=O)CC(=O)C(F)(F)C(F)(F)C(F)(F)F		-6.71	0.64	2.27	P	IN

QL00424	Yttrium, tris(6,6,7,7,8,8,8-heptafluoro-2,2-dimethyl-3,5-octanedionato-βO,βO')- (9CI)	19186-73-3	[Y+3].CC(C)(C)C([O-])=CC(=O)C(F)(F)C(F)(F)C(F)(F)F.CC(C)(C)C([O-])=CC(=O)C(F)(F)C(F)(F)C(F)(F)F.CC(C)(C)C([O-])=CC(=O)C(F)(F)C(F)(F)C(F)(F)F		-6.81	0.65	2.24	P	IN
QL00425	Ytterbium, tris(6,6,7,7,8,8,8-heptafluoro-2,2-dimethyl-3,5-octanedionato-βO3,βO5)-	18323-96-1	[Yb+3].CC(C)(C)C([O-])=CC(=O)C(F)(F)C(F)(F)C(F)(F)F.CC(C)(C)C([O-])=CC(=O)C(F)(F)C(F)(F)C(F)(F)F.CC(C)(C)C([O-])=CC(=O)C(F)(F)C(F)(F)C(F)(F)F		-6.81	0.65	2.24	P	IN
QL00426	Ytterbium, tris[3-[2,2,3,3,4,4,4-heptafluoro-1-(oxo-βO)butyl]-1,7,7-trimethylbicyclo[2.2.1]heptan-2-onato-βO]-	80464-74-0	[Yb+3].CC1(C)C2CCC1(C)C(=O)[C-]2C(=O)C(F)(F)C(F)(F)C(F)(F)F.CC1(C)C2CCC1(C)C(=O)[C-]2C(=O)C(F)(F)C(F)(F)C(F)(F)F.CC1(C)C2CCC1(C)C(=O)[C-]2C(=O)C(F)(F)C(F)(F)C(F)(F)F		-7.01	0.66	2.18	P	IN
QL00427	Perfluorohexanesulfinate zinc salt	86525-30-6	[Zn++].[O-]S(=O)C(F)(F)C(F)(F)C(F)(F)C(F)(F)C(F)(F)C(F)(F)F.[O-]S(=O)C(F)(F)C(F)(F)C(F)(F)C(F)(F)C(F)(F)C(F)(F)F		-7.29	0.67	2.26	P	IN
QL00428	3-(Perfluoro-7-(methyl)octyl)-2-hydroxypropyltrimethylammonium iodide	80909-29-1	C([N+](C)(C)C)C(O)CC(F)(F)C(F)(F)C(F)(F)C(F)(F)C(F)(F)C(F)(F)C(F)(C(F)(F)F)C(F)(F)F.[I-]		-8.07	0.66	2.80	P	IN
QL00429	N,N-Dimethyl-N-(2-hydroxyethyl)-3-((2-hydroxy-3-sulfopropyl)((perfluorohexyl)sulfonyl)amino)propanaminium hydroxide sodium	81190-38-7	C([N+](CCO)(C)C)CCN([S](=O)(=O)C(F)(F)C(F)(F)C(F)(F)C(F)(F)C(F)(F)C(F)(F)F)CC(O)C[S]([O-])(=O)=O.[K+].[OH-]		-7.44	0.62	3.04	P	OUT
QL00431	(Perfluorododecyl)ethylsulfonyl chloride	68758-57-6	C([S](Cl)(=O)=O)CC(F)(F)C(F)(F)C(F)(F)C(F)(F)C(F)(F)C(F)(F)C(F)(F)C(F)(F)C(F)(F)C(F)(F)C(F)(F)C(F)(F)F		-9.41	0.69	3.06	P	OUT
QL00432	(Perfluorodecanyl)ethylsulfonyl chloride	27619-91-6	C([S](Cl)(=O)=O)CC(F)(F)C(F)(F)C(F)(F)C(F)(F)C(F)(F)C(F)(F)C(F)(F)C(F)(F)C(F)(F)C(F)(F)F		-8.99	0.68	2.88	P	OUT
QL00433	1-Undecanesulfonyl chloride, 3,3,4,4,5,5,6,6,7,7,8,8,9,9,10,10,11,11,11-nonadecafluoro-	65702-24-1	C([S](Cl)(=O)=O)CC(F)(F)C(F)(F)C(F)(F)C(F)(F)C(F)(F)C(F)(F)C(F)(F)C(F)(F)C(F)(F)F		-8.65	0.68	2.73	P	IN
QL00434	1-Nonanesulfonyl chloride, 3,3,4,4,5,5,6,6,7,7,8,8,9,9,9-pentadecafluoro-	27607-61-0	C([S](Cl)(=O)=O)CC(F)(F)C(F)(F)C(F)(F)C(F)(F)C(F)(F)C(F)(F)C(F)(F)F		-8.09	0.68	2.50	P	IN
QL00435	1-Heptanesulfonyl chloride, 3,3,4,4,5,5,6,6,7,7,7-undecafluoro-	65702-23-0	C([S](Cl)(=O)=O)CC(F)(F)C(F)(F)C(F)(F)C(F)(F)C(F)(F)F		-7.39	0.67	2.23	P	IN
QL00436	1-Hexanesulfonyl chloride, 3,3,4,4,5,5,6,6,6-nonafluoro-	27619-88-1	C([S](Cl)(=O)=O)CC(F)(F)C(F)(F)C(F)(F)C(F)(F)F		-7.14	0.67	2.17	P	IN
QL00437	102 Fluorotelomer unsaturated aldehyde	864551-40-6	C(=C(/C(C(C(C(C(C(C(C(F)(F)F)(F)F)(F)F)(F)F)(F)F)(F)F)(F)F)(F)F)F)C=O		-8.34	0.67	2.74	P	IN
QL00438	Chlorotrifluoroethylene	79-38-9	C(=C(F)Cl)(F)F		-2.25	0.58	0.92	P	OUT
QL00439	1,1,2,3,3,3-Hexafluoro-1-propene	116-15-4	C(=C(F)F)(C(F)(F)F)F		-4.35	0.62	1.35	P	OUT
QL00440	Tetrafluoroethylene	116-14-3	C(=C(F)F)(F)F		-2.25	0.58	0.92	P	OUT
QL00441	Heptafluoropropyl trifluorovinyl ether	1623-05-8	C(=C(F)F)(OC(C(C(F)(F)F)(F)F)(F)F)F		-6.19	0.65	1.92	P	IN
QL00442	Pentafluoroethyl trifluorovinyl ether	10493-43-3	C(=C(F)F)(OC(C(F)(F)F)(F)F)F		-5.55	0.64	1.76	P	IN
QL00443	Trifluoro(trifluoromethoxy)ethylene	1187-93-5	C(=C(F)F)(OC(F)(F)F)F		-4.15	0.61	1.49	P	OUT
QL00444	2-Perfluorooctyl ethanoic acid	27854-31-5	C(C(=O)O)C(C(C(C(C(C(C(C(F)(F)F)(F)F)(F)F)(F)F)(F)F)(F)F)(F)F)(F)F		-8.04	0.68	2.51	P	IN
QL00445	2-Perfluorohexyl ethanoic acid	53826-12-3	C(C(=O)O)C(C(C(C(C(C(F)(F)F)(F)F)(F)F)(F)F)(F)F)(F)F		-7.33	0.67	2.24	P	IN
QL00446	N-[(1,1,2,2,3,3,4,4,5,5,6,6,7,7,8,8,8-Heptadecafluorooctyl)sulfonyl]glycine (FOSAA)	2806-24-8	C(C(=O)O)NS(=O)(=O)C(C(C(C(C(C(C(C(F)(F)F)(F)F)(F)F)(F)F)(F)F)(F)F)(F)F)(F)F		-8.45	0.67	2.74	P	IN
QL00447	7H-Perfluoroheptanoyl chloride	41405-35-0	C(C(C(C(C(C(C(=O)Cl)(F)F)(F)F)(F)F)(F)F)(F)F)(F)F		-7.22	0.67	2.15	P	IN
QL00448	(1H,1H-Perfluoro-16-methylheptadecyl)oxirane	54009-79-9	C(C(C(C(C(C(C(C(C(C(C(C(C(C(C(C(F)(F)F)(C(F)(F)F)F)(F)F)(F)F)(F)F)(F)F)(F)F)(F)F)(F)F)(F)F)(F)F)(F)F)(F)F)(F)F)(F)F)(F)F)C1OC1		-9.86	0.69	3.26	P	OUT
QL00449	1,1,2,2-Tetrahydroperfluoro-1-octadecanol	65104-67-8	C(C(C(C(C(C(C(C(C(C(C(C(C(C(C(C(F)(F)F)(F)F)(F)F)(F)F)(F)F)(F)F)(F)F)(F)F)(F)F)(F)F)(F)F)(F)F)(F)F)(F)F)(F)F)(F)F)CO		-9.78	0.68	3.27	P	OUT
QL00450	3-(Perfluoro(13-methyltetradecyl))-2-hydroxypropyl dihydrogen phosphate	63295-29-4	C(C(C(C(C(C(C(C(C(C(C(C(C(C(F)(F)F)(C(F)(F)F)F)(F)F)(F)F)(F)F)(F)F)(F)F)(F)F)(F)F)(F)F)(F)F)(F)F)(F)F)(F)F)C(CO[P](=O)(O)O)O		-9.81	0.68	3.34	P	OUT
QL00451	2-(Perfluorotetradecyl)ethan-1-ol	60699-51-6	C(C(C(C(C(C(C(C(C(C(C(C(C(C(F)(F)F)(F)F)(F)F)(F)F)(F)F)(F)F)(F)F)(F)F)(F)F)(F)F)(F)F)(F)F)(F)F)(F)F)CO		-9.41	0.68	3.11	P	OUT
QL00452	(Perfluorotetradecyl)ethyl 2-methyl-2-propenoate	4980-53-4	C(C(C(C(C(C(C(C(C(C(C(C(C(C(F)(F)F)(F)F)(F)F)(F)F)(F)F)(F)F)(F)F)(F)F)(F)F)(F)F)(F)F)(F)F)(F)F)(F)F)COC(=O)C(=C)C		-9.80	0.68	3.37	P	OUT
QL00453	1,1,2,2-Tetrahydroperfluorohexadecyl acrylate	34362-49-7	C(C(C(C(C(C(C(C(C(C(C(C(C(C(F)(F)F)(F)F)(F)F)(F)F)(F)F)(F)F)(F)F)(F)F)(F)F)(F)F)(F)F)(F)F)(F)F)(F)F)COC(=O)C=C		-9.71	0.67	3.37	P	OUT
QL00454	Diammonium 3-(perfluoro-15-methylhexadecyl)-2-hydroxypropyl dihydrogen phosphate	94200-53-0	C(C(C(C(C(C(C(C(C(C(C(C(C(C(CC(COP(=O)([O-])[O-])O)(F)F)(F)F)(F)F)(F)F)(F)F)(F)F)(F)F)(F)F)(F)F)(F)F)(F)F)(F)F)(F)F)(C(C(F)(F)F)(C(F)(F)F)F)(F)F.[NH4+].[NH4+]		-9.71	0.66	3.57	P	OUT
QL00455	3-(Perfluoro(11-methyldodecyl))-2-hydroxypropyl dihydrogen phosphate	63295-28-3	C(C(C(C(C(C(C(C(C(C(C(C(F)(F)F)(C(F)(F)F)F)(F)F)(F)F)(F)F)(F)F)(F)F)(F)F)(F)F)(F)F)(F)F)(F)F)C(CO[P](=O)(O)O)O		-9.45	0.68	3.19	P	OUT

QL00456	(1H,1H-Perfluoro-12-methyltridecyl)oxirane	54009-78-8	C(C(C(C(C(C(C(C(C(C(C(C(C(F)(F)F)(C(F)(F)F)F)(F)F)(F)F)(F)F)(F)F)(F)F)(F)F)(F)F)(F)F)(F)F)(F)F)C1OC1		-9.11	0.68	2.92	P	OUT
QL00457	1-Iodo-1H,1H,2H,2H-perfluorotetradecane	30046-31-2	C(C(C(C(C(C(C(C(C(C(C(C(C(F)(F)F)(F)F)(F)F)(F)F)(F)F)(F)F)(F)F)(F)F)(F)F)(F)F)(F)F)(F)F)CI		-8.99	0.68	2.93	P	OUT
QL00458	2-(Perfluorododecyl)ethanol	39239-77-5	C(C(C(C(C(C(C(C(C(C(C(C(C(F)(F)F)(F)F)(F)F)(F)F)(F)F)(F)F)(F)F)(F)F)(F)F)(F)F)(F)F)(F)F)CO		-8.99	0.68	2.93	P	OUT
QL00459	Bis(2-(perfluorododecyl)ethyl) hydrogen phosphate	57677-99-3	C(C(C(C(C(C(C(C(C(C(C(C(C(F)(F)F)(F)F)(F)F)(F)F)(F)F)(F)F)(F)F)(F)F)(F)F)(F)F)(F)F)(F)F)CO[P](=O)(OCCC(C(C(C(C(C(C(C(C(C(C(C(F)(F)F)(F)F)(F)<br>F)(F)F)(F)F)(F)F)(F)F)(F)F)(F)F)(F)F)(F)F)(F)F)O		-11.16	0.69	3.96	P	OUT
QL00460	(Perfluorododecyl)ethyl methacrylate	6014-75-1	C(C(C(C(C(C(C(C(C(C(C(C(C(F)(F)F)(F)F)(F)F)(F)F)(F)F)(F)F)(F)F)(F)F)(F)F)(F)F)(F)F)(F)F)COC(=O)C(=C)C		-9.43	0.67	3.23	P	OUT
QL00461	1,1,2,2-Tetrahydroperfluorotetradecyl acrylate	34395-24-9	C(C(C(C(C(C(C(C(C(C(C(C(C(F)(F)F)(F)F)(F)F)(F)F)(F)F)(F)F)(F)F)(F)F)(F)F)(F)F)(F)F)(F)F)COC(=O)C=C		-9.33	0.67	3.23	P	OUT
QL00462	3-(Perfluoro(9-methyldecyl))-2-hydroxypropyl dihydrogen phosphate	63295-27-2	C(C(C(C(C(C(C(C(C(C(C(F)(F)F)(C(F)(F)F)F)(F)F)(F)F)(F)F)(F)F)(F)F)(F)F)(F)F)(F)F)C(CO[P](=O)(O)O)O		-9.04	0.67	3.03	P	OUT
QL00463	((Perfluoro-9-methyldecyl)methyl)oxirane	47795-34-6	C(C(C(C(C(C(C(C(C(C(C(F)(F)F)(C(F)(F)F)F)(F)F)(F)F)(F)F)(F)F)(F)F)(F)F)(F)F)(F)F)C1CO1		-8.65	0.68	2.72	P	IN
QL00464	(Perfluoro-9-methyldecyl)ethyl methacrylate	74256-14-7	C(C(C(C(C(C(C(C(C(C(C(F)(F)F)(C(F)(F)F)F)(F)F)(F)F)(F)F)(F)F)(F)F)(F)F)(F)F)(F)F)COC(C(C)=C)=O		-8.97	0.67	3.04	P	OUT
QL00465	(Perfluoro-9-methyldecyl)ethyl acrylate	52956-81-7	C(C(C(C(C(C(C(C(C(C(C(F)(F)F)(C(F)(F)F)F)(F)F)(F)F)(F)F)(F)F)(F)F)(F)F)(F)F)(F)F)COC(C=C)=O		-8.88	0.67	3.06	P	OUT
QL00466	(Perfluorodecyl)ethylene	30389-25-4	C(C(C(C(C(C(C(C(C(C(C(F)(F)F)(F)F)(F)F)(F)F)(F)F)(F)F)(F)F)(F)F)(F)F)(F)F)=C		-8.63	0.68	2.68	P	IN
QL00467	102 Fluorotelomer iodide	2043-54-1	C(C(C(C(C(C(C(C(C(C(C(F)(F)F)(F)F)(F)F)(F)F)(F)F)(F)F)(F)F)(F)F)(F)F)(F)F)CI		-8.50	0.68	2.73	P	IN
QL00468	122 Fluorotelomer phosphate diester	1895-26-7	C(C(C(C(C(C(C(C(C(C(C(F)(F)F)(F)F)(F)F)(F)F)(F)F)(F)F)(F)F)(F)F)(F)F)(F)F)CO[P](=O)(OCCC(C(C(C(C(C(C(C(C(C(F)(F)F)(F)F)(F)F)(F)F)(F)F)(F)F)(F)F)(F)<br>F)(F)F)(F)F)O		-10.71	0.68	3.77	P	OUT
QL00469	3-(Perfluoro-7-methyloctyl)-2-hydroxypropyl acrylate	24407-09-8	C(C(C(C(C(C(C(C(C(F)(F)F)(C(F)(F)F)F)(F)F)(F)F)(F)F)(F)F)(F)F)(F)F)C(COC(C=C)=O)O		-8.39	0.66	2.93	P	IN
QL00470	(2,2,3,3,4,4,5,5,6,6,7,7,8,9,9,9-Hexadecafluoro-8-(trifluoromethyl)nonyl)oxirane	41925-33-1	C(C(C(C(C(C(C(C(C(F)(F)F)(C(F)(F)F)F)(F)F)(F)F)(F)F)(F)F)(F)F)(F)F)C1CO1		-8.09	0.68	2.48	P	IN
QL00471	3,3,4,4,5,5,6,6,7,7,8,8,9,10,10,10-Hexadecafluoro-9-(trifluoromethyl)decyl methacrylate	15166-00-4	C(C(C(C(C(C(C(C(C(F)(F)F)(C(F)(F)F)F)(F)F)(F)F)(F)F)(F)F)(F)F)(F)F)COC(C(C)=C)=O		-8.47	0.67	2.86	P	IN
QL00472	3,3,4,4,5,5,6,6,7,7,8,8,9,10,10,10-Hexadecafluoro-9-(trifluoromethyl)decyl acrylate	15577-26-1	C(C(C(C(C(C(C(C(C(F)(F)F)(C(F)(F)F)F)(F)F)(F)F)(F)F)(F)F)(F)F)(F)F)COC(C=C)=O		-8.38	0.66	2.88	P	IN
QL00473	(2-(Perfluorooctyl)-1-ethyl)bis(2-hydroxyethyl)methylammonium iodide	31841-41-5	C(C(C(C(C(C(C(C(C(F)(F)F)(F)F)(F)F)(F)F)(F)F)(F)F)(F)F)(F)F)C[N+](C)(CCO)CCO.[I-]		-7.84	0.63	3.00	P	OUT
QL00474	1-Decanesulfonyl chloride, 3,3,4,4,5,5,6,6,7,7,8,8,9,9,10,10,10-heptadecafluoro-	27619-90-5	C(C(C(C(C(C(C(C(C(F)(F)F)(F)F)(F)F)(F)F)(F)F)(F)F)(F)F)(F)F)C[S](=O)(=O)Cl		-8.49	0.68	2.68	P	IN
QL00475	3-(Perfluorooctyl)-1,2-propenoxide	38565-53-6	C(C(C(C(C(C(C(C(C(F)(F)F)(F)F)(F)F)(F)F)(F)F)(F)F)(F)F)(F)F)C1CO1		-8.15	0.68	2.51	P	IN
QL00476	82 Fluorotelomer iodide	2043-53-0	C(C(C(C(C(C(C(C(C(F)(F)F)(F)F)(F)F)(F)F)(F)F)(F)F)(F)F)(F)F)CI		-7.91	0.67	2.50	P	IN
QL00478	82 Fluorotelomer acrylate	27905-45-9	C(C(C(C(C(C(C(C(C(F)(F)F)(F)F)(F)F)(F)F)(F)F)(F)F)(F)F)(F)F)COC(C=C)=O		-8.39	0.66	2.90	P	IN
QL00479	1H,1H,9H-Hexadecafluoro-1-nonanol	376-18-1	C(C(C(C(C(C(C(C(C(F)F)(F)F)(F)F)(F)F)(F)F)(F)F)(F)F)(F)F)O		-7.80	0.67	2.42	P	IN
QL00480	1H,1H,9H-perfluorononyl methacrylate	1841-46-9	C(C(C(C(C(C(C(C(C(F)F)(F)F)(F)F)(F)F)(F)F)(F)F)(F)F)(F)F)OC(C(C)=C)=O		-8.16	0.66	2.74	P	IN
QL00481	1H,1H,9H-Perfluorononyl acrylate	4180-26-1	C(C(C(C(C(C(C(C(C(F)F)(F)F)(F)F)(F)F)(F)F)(F)F)(F)F)(F)F)OC(C=C)=O		-7.98	0.66	2.73	P	IN
QL00482	71 Fluorotelomer alcohol	307-30-2	C(C(C(C(C(C(C(C(F)(F)F)(F)F)(F)F)(F)F)(F)F)(F)F)(F)F)O		-7.58	0.68	2.23	P	IN
QL00483	1H,1H-Perfluorooctyl methacrylate	3934-23-4	C(C(C(C(C(C(C(C(F)(F)F)(F)F)(F)F)(F)F)(F)F)(F)F)(F)F)OC(=O)C(C)=C		-8.19	0.67	2.69	P	IN
QL00484	1H,1H-Perfluorooctyl acrylate	307-98-2	C(C(C(C(C(C(C(C(F)(F)F)(F)F)(F)F)(F)F)(F)F)(F)F)(F)F)OC(C=C)=O		-8.02	0.66	2.70	P	IN
QL00485	3-(Perfluorohexyl)-1,2-epoxypropane	38565-52-5	C(C(C(C(C(C(C(F)(F)F)(F)F)(F)F)(F)F)(F)F)(F)F)C1CO1		-7.47	0.67	2.24	P	IN
QL00486	1H,1H,2H,2H-Perfluorooctyl iodide	2043-57-4	C(C(C(C(C(C(C(F)(F)F)(F)F)(F)F)(F)F)(F)F)(F)F)CI		-7.16	0.66	2.23	P	IN
QL00488	62 Fluorotelomer methacrylate	2144-53-8	C(C(C(C(C(C(C(F)(F)F)(F)F)(F)F)(F)F)(F)F)(F)F)COC(C(C)=C)=O		-7.94	0.66	2.71	P	IN
QL00489	61 Fluorotelomer alcohol	375-82-6	C(C(C(C(C(C(C(F)(F)F)(F)F)(F)F)(F)F)(F)F)(F)F)O		-7.35	0.68	2.15	P	IN
QL00490	Dodecafluoroheptanol	335-99-9	C(C(C(C(C(C(C(F)F)(F)F)(F)F)(F)F)(F)F)(F)F)O		-7.01	0.66	2.13	P	IN
QL00491	(Perfluorohexyl)ethylene	25291-17-2	C(C(C(C(C(C(F)(F)C(F)(F)F)(F)F)(F)F)(F)F)(F)F)=C		-7.35	0.68	2.15	P	IN
QL00492	51 Fluorotelomer alcohol	423-46-1	C(C(C(C(C(C(F)(F)F)(F)F)(F)F)(F)F)(F)F)O		-6.72	0.67	1.89	P	IN

QL00493	8-10-phosphinic		C(C(C(C(C(C(F)(F)P(=O)(C(C(C(C(C(C(C(C(C(F)(F)F)(F)F)(F)F)(F)F)(F)F)(F)F)(F)F)(F)F)O)(F)F)(F)F)(F)F)(F)F)(C(C(C(C(F)(F)F)(F)F)(F)F)(F)F)(F)F		-10.32	0.70	3.38	P	OUT
QL00494	PFDPA		C(C(C(C(C(C(F)(F)P(=O)(O)O)(F)F)(F)F)(F)F)(F)F)(C(C(C(C(F)(F)F)(F)F)(F)F)(F)F)(F)F		-8.88	0.69	2.69	P	OUT
QL00495	Perfluorononanesulfonyl fluoride	68259-06-3	C(C(C(C(C(F)(F)F)(F)F)(F)F)(F)F)(C(C(C(C(F)(F)S(=O)(=O)F)(F)F)(F)F)(F)F)(F)F		-8.53	0.69	2.51	P	IN
QL00496	1,1,1,2,2,3,3,4,4-Nonafluoro-6-iodohexane	2043-55-2	C(C(C(C(C(F)(F)F)(F)F)(F)F)(F)F)CI		-6.13	0.65	1.91	P	IN
QL00497	1H,1H,2H,2H-Perfluorohexyl methacrylate	1799-84-4	C(C(C(C(C(F)(F)F)(F)F)(F)F)(F)F)COC(C(C)=C)=O		-7.06	0.65	2.43	P	IN
QL00498	2-(Perfluorobutyl)ethyl acrylate	52591-27-2	C(C(C(C(C(F)(F)F)(F)F)(F)F)(F)F)COC(C=C)=O		-6.82	0.64	2.44	P	OUT
QL00499	Bis(heptadecafluorooctyl)phosphinic acid		C(C(C(C(C(F)(F)P(=O)(C(C(C(C(C(C(C(C(F)(F)F)(F)F)(F)F)(F)F)(F)F)(F)F)(F)F)(F)F)O)(F)F)(F)F)(F)F)(C(C(C(F)(F)F)(F)F)(F)F)(F)F		-10.02	0.70	3.24	P	OUT
QL00500	6-8-phosphinic		C(C(C(C(C(F)(F)P(=O)(C(C(C(C(C(C(F)(F)F)(F)F)(F)F)(F)F)(F)F)(F)F)O)(F)F)(F)F)(F)F)(C(C(C(F)(F)F)(F)F)(F)F)(F)F		-9.69	0.69	3.07	P	OUT
QL00503	1H,1H,5H-Perfluoropentanol	355-80-6	C(C(C(C(C(F)F)(F)F)(F)F)(F)F)O		-5.92	0.65	1.79	P	IN
QL00504	Perfluorobutanesulfonic anhydride	36913-91-4	C(C(C(C(F)(F)[S](=O)(=O)O[S](C(C(C(C(F)(F)F)(F)F)(F)F)(F)F)(=O)=O)(F)F)(F)F)(F)(F)F		-8.64	0.69	2.63	P	IN
QL00505	Bis(tridecafluorohexyl)phosphinic acid		C(C(C(C(F)(F)P(=O)(C(C(C(C(C(C(F)(F)F)(F)F)(F)F)(F)F)(F)F)(F)F)O)(F)F)(F)F)(C(C(F)(F)F)(F)F)(F)F		-9.32	0.69	2.89	P	OUT
QL00506	1H,1H,2H-Perfluoro-1-decene	21652-58-4	C(C(C(F)(F)C(F)(F)C(F)(C(F)(F)C(C(F)(F)C(F)(F)F)(F)F)F)(F)F)=C		-8.06	0.68	2.44	P	IN
QL00507	Perfluoroisobutyl methyl ether	163702-08-7	C(C(C(F)(F)F)(C(F)(F)F)F)OC(F)F		-5.36	0.64	1.64	P	IN
QL00508	Bis(nonafluorobutyl)phosphinic acid	52299-25-9	C(C(C(F)(F)P(=O)(C(C(C(C(F)(F)F)(F)F)(F)F)(F)F)O)(F)F)(C(F)(F)F)(F)F		-8.37	0.69	2.44	P	IN
QL00509	C4-phosphonic		C(C(C(F)(F)P(=O)(O)O)(F)F)(C(F)(F)F)(F)F		-6.93	0.69	1.79	P	IN
QL00511	1H,1H,11H-Eicosafluoro-1-undecanol	307-70-0	C(C(F)(C(C(F)(C(C(F)(C(C(F)(C(C(F)(C(F)F)F)(F)F)F)(F)F)F)(F)F)F)(F)F)F)O		-8.42	0.68	2.66	P	IN
QL00512	3-(Perfluoro(11-methyl)dodecyl)-2-hydroxypropyl propenoate	16083-87-7	C(C(F)(F)C(C(C(C(C(C(C(C(C(C(F)(F)F)(F)C(F)(F)F)(F)F)(F)F)(F)F)(F)F)(F)F)(F)F)(F)F)(F)F)(F)F)C(COC(C=C)=O)O		-9.32	0.67	3.26	P	OUT
QL00513	(Perfluoro-7-methyloctyl)-2-hydroxypropyl dihydrogen phosphate	54009-73-3	C(C(F)(F)C(F)(C(F)(F)C(F)(F)C(F)(F)C(F)(F)C(F)(C(F)(F)F)C(F)(F)F)F)C(CO[P](=O)(O)O)O		-8.56	0.67	2.84	P	IN
QL00514	4-((Perfluoro-7-methyloctyl)methyl)-2,2-dimethyl-1,3-dioxolane	94159-90-7	C(C(F)(F)C(F)(F)C(F)(F)C(F)(F)C(F)(F)C(F)(F)C(F)(C(F)(F)F)C(F)(F)F)C1OC(OC1)(C)C		-8.47	0.68	2.72	P	IN
QL00515	82 Fluorotelomer 9-(trifluoromethyl)-dihydrogen phosphate	78974-42-2	C(C(F)(F)C(F)(F)C(F)(F)C(F)(F)C(F)(F)C(F)(F)C(F)(C(F)(F)F)C(F)(F)F)CO[P](O)(O)=O		-8.35	0.67	2.69	P	IN
QL00516	Bis(2-(perfluoro-7-methyloctyl)ethyl) hydrogen phosphate	78974-41-1	C(C(F)(F)C(F)(F)C(F)(F)C(F)(F)C(F)(F)C(F)(F)C(F)(C(F)(F)F)C(F)(F)F)CO[P](OCCC(F)(F)C(F)(F)C(F)(F)C(F)(F)C(F)(F)C(F)(F)C(F)(C(F)(F)F)C(F)(F)F)(O)=O		-10.02	0.68	3.47	P	OUT
QL00517	32-(Perfluoro-9-methyldecyl)-2,5,8,11,14,17,20,23,26,29-decaoxodotriacontan-31-ol	93776-10-4	C(C(F)(F)C(F)(F)C(F)(F)C(F)(F)C(F)(F)C(F)(F)C(F)(F)C(F)(F)C(F)(C(F)(F)F)C(F)(F)F)C(O)CC(=O)CCC(=O)CCC(=O)CCC(=O)CCC(=O)CCC(=O)CCC(=O)CCC(=O)CCC(=O)CCC(=O)C		-10.18	0.62	4.34	P	OUT
QL00518	(2-Carboxylatoethyl)-3-((perfluoro-9-methyldecyl)-2-hydroxypropyl)aminopropyldimethylammonium	93777-12-9	C(C(F)(F)C(F)(F)C(F)(F)C(F)(F)C(F)(F)C(F)(F)C(F)(F)C(F)(F)C(F)(C(F)(F)F)C(F)(F)F)C(O)CNCCC[N+](CCC([O-])=O)(C)C		-9.34	0.65	3.46	P	OUT
QL00519	(Perfluoro-9-methyldecyl)ethyl dihydrogen phosphate	94200-56-3	C(C(F)(F)C(F)(F)C(F)(F)C(F)(F)C(F)(F)C(F)(F)C(F)(F)C(F)(F)C(F)(C(F)(F)F)C(F)(F)F)CO[P](O)(O)=O		-8.86	0.68	2.89	P	OUT
QL00520	Bis((perfluoro-9-methyldecyl)ethyl) phosphate	93857-55-7	C(C(F)(F)C(F)(F)C(F)(F)C(F)(F)C(F)(F)C(F)(F)C(F)(F)C(F)(F)C(F)(C(F)(F)F)C(F)(F)F)CO[P](OCCC(F)(F)C(F)(F)C(F)(F)C(F)(F)C(F)(F)C(F)(F)C(F)(F)C(F)(F)C(F)(C(F)(F)F)C(F)(F)F)([O-])=O		-10.58	0.68	3.71	P	OUT
QL00521	((Perfluoro-11-methyldodecyl)ethyl) dihydrogen phosphate	94200-57-4	C(C(F)(F)C(F)(F)C(F)(F)C(F)(F)C(F)(F)C(F)(F)C(F)(F)C(F)(F)C(F)(F)C(F)(F)C(F)(C(F)(F)F)C(F)(F)F)CO[P](O)(O)=O		-9.30	0.68	3.07	P	OUT
QL00522	Bis((perfluoro-11-methyldodecyl)ethyl) hydrogen phosphate	93857-56-8	C(C(F)(F)C(F)(F)C(F)(F)C(F)(F)C(F)(F)C(F)(F)C(F)(F)C(F)(F)C(F)(F)C(F)(F)C(F)(C(F)(F)F)C(F)(F)F)CO[P](OCCC(F)(F)C(F)(F)C(F)(F)C(F)(F)C(F)(F)C(F)(F)C(F)(F)C(F)(F)C(F)(F)C(F)(F)C(F)(C(F)(F)F)C(F)(F)F)(O)=O		-11.05	0.68	3.92	P	OUT
QL00523	(Perfluoro-11-methyldodecyl)ethyl methacrylate	74256-15-8	C(C(F)(F)C(F)(F)C(F)(F)C(F)(F)C(F)(F)C(F)(F)C(F)(F)C(F)(F)C(F)(F)C(F)(F)C(F)(C(F)(F)F)C(F)(F)F)COC(=O)C(C)=C		-9.39	0.67	3.21	P	OUT
QL00524	3-(Perfluoro(13-methyl)tetradecyl)-2-hydroxypropyl acrylate	16083-78-6	C(C(F)(F)C(F)(F)C(F)(F)C(F)(F)C(F)(F)C(F)(F)C(F)(F)C(F)(F)C(F)(F)C(F)(F)C(F)(F)C(F)(F)C(F)(C(F)(F)F)C(F)(F)F)C(O)COC(=O)C=C		-9.70	0.67	3.40	P	OUT
QL00525	(Perfluoro-13-methyltetradecyl)ethyl dihydrogen phosphate	93857-42-2	C(C(F)(F)C(F)(F)C(F)(F)C(F)(F)C(F)(F)C(F)(F)C(F)(F)C(F)(F)C(F)(F)C(F)(F)C(F)(F)C(F)(F)C(F)(C(F)(F)F)C(F)(F)F)CO[P](O)(O)=O		-9.67	0.68	3.24	P	OUT

QL00526	Bis((perfluoro-13-methyltetradecyl)ethyl) hydrogen phosphate	93776-29-5	C(C(F)(F)C(F)(F)C(F)(F)C(F)(F)C(F)(F)C(F)(F)C(F)(F)C(F)(F)C(F)(F)C(F)(F)C(F)(F)C(F)(F)C(F)(C(F)(F)F)C(F)(F)F)CO[P](OCCC(F)(F)C(F)(F)C(F)(F)C(F)(F)C(F)(F)C(F)(F)C(F)(F)C(F)(F)C(F)(F)C(F)(F)C(F)(F)C(F)(F)C(F)(C(F)(F)F)C(F)(F)F)(O)=O		-11.45	0.69	4.10	P	OUT
QL00527	((Perfluoro-13-methyltetradecyl)ethyl) methacrylate	94158-64-2	C(C(F)(F)C(F)(F)C(F)(F)C(F)(F)C(F)(F)C(F)(F)C(F)(F)C(F)(F)C(F)(F)C(F)(F)C(F)(F)C(F)(F)C(F)(C(F)(F)F)C(F)(F)F)COC(=O)C(C)=C		-9.75	0.68	3.35	P	OUT
QL00528	((Perfluoro-13-methyltetradecyl)ethyl) acrylate	91615-22-4	C(C(F)(F)C(F)(F)C(F)(F)C(F)(F)C(F)(F)C(F)(F)C(F)(F)C(F)(F)C(F)(F)C(F)(F)C(F)(F)C(F)(F)C(F)(C(F)(F)F)C(F)(F)F)COC(=O)C=C		-9.69	0.67	3.36	P	OUT
QL00529	(Perfluoro-15-methylhexadecyl)ethyl dihydrogen phosphate	94231-59-1	C(C(F)(F)C(F)(F)C(F)(F)C(F)(F)C(F)(F)C(F)(F)C(F)(F)C(F)(F)C(F)(F)C(F)(F)C(F)(F)C(F)(F)C(F)(F)C(F)(F)C(F)(C(F)(F)F)C(F)(F)F)CO[P](O)(O)=O		-10.01	0.68	3.38	P	OUT
QL00530	(Perfluoro-15-methylhexadecyl)ethyl methacrylate	94158-65-3	C(C(F)(F)C(F)(F)C(F)(F)C(F)(F)C(F)(F)C(F)(F)C(F)(F)C(F)(F)C(F)(F)C(F)(F)C(F)(F)C(F)(F)C(F)(F)C(F)(F)C(F)(C(F)(F)F)C(F)(F)F)COC(=O)C(C)=C		-10.08	0.68	3.49	P	OUT
QL00531	(Perfluoro-15-methylhexadecyl)ethyl acrylate	94158-63-1	C(C(F)(F)C(F)(F)C(F)(F)C(F)(F)C(F)(F)C(F)(F)C(F)(F)C(F)(F)C(F)(F)C(F)(F)C(F)(F)C(F)(F)C(F)(F)C(F)(F)C(F)(C(F)(F)F)C(F)(F)F)COC(=O)C=C		-10.02	0.68	3.50	P	OUT
QL00532	182 Fluorotelomer iodide	65104-63-4	C(C(F)(F)C(F)(F)C(F)(F)C(F)(F)C(F)(F)C(F)(F)C(F)(F)C(F)(F)C(F)(F)C(F)(F)C(F)(F)C(F)(F)C(F)(F)C(F)(F)C(F)(F)C(F)(F)C(C(F)(F)F)(F)F)CI		-10.10	0.69	3.41	P	OUT
QL00533	182 Fluorotelomer alcohol	65104-65-6	C(C(F)(F)C(F)(F)C(F)(F)C(F)(F)C(F)(F)C(F)(F)C(F)(F)C(F)(F)C(F)(F)C(F)(F)C(F)(F)C(F)(F)C(F)(F)C(F)(F)C(F)(F)C(F)(F)C(C(F)(F)F)(F)F)CO		-10.10	0.69	3.41	P	OUT
QL00534	3-(Perfluorohexadecyl)-2-hydroxypropyl dihydrogen phosphate	94200-44-9	C(C(F)(F)C(F)(F)C(F)(F)C(F)(F)C(F)(F)C(F)(F)C(F)(F)C(F)(F)C(F)(F)C(F)(F)C(F)(F)C(F)(F)C(F)(F)C(F)(F)C(F)(F)C(F)(F)F)C(O)CO[P](O)(O)=O		-10.18	0.68	3.50	P	OUT
QL00535	3-(Perfluorotetradecyl)-2-hydroxypropyl dihydrogen phosphate	94200-43-8	C(C(F)(F)C(F)(F)C(F)(F)C(F)(F)C(F)(F)C(F)(F)C(F)(F)C(F)(F)C(F)(F)C(F)(F)C(F)(F)C(F)(F)C(F)(F)C(F)(F)F)C(O)CO[P](O)(O)=O		-9.87	0.68	3.37	P	OUT
QL00536	142 Fluorotelomer dihydrogen phosphate	94200-54-1	C(C(F)(F)C(F)(F)C(F)(F)C(F)(F)C(F)(F)C(F)(F)C(F)(F)C(F)(F)C(F)(F)C(F)(F)C(F)(F)C(F)(F)C(F)(F)C(F)(F)F)CO[P](=O)(O)O		-9.74	0.68	3.26	P	OUT
QL00537	Bis((perfluorotetradecyl)ethyl) hydrogen phosphate	93857-53-5	C(C(F)(F)C(F)(F)C(F)(F)C(F)(F)C(F)(F)C(F)(F)C(F)(F)C(F)(F)C(F)(F)C(F)(F)C(F)(F)C(F)(F)C(F)(F)C(F)(F)F)CO[P](OCCC(F)(F)C(F)(F)C(F)(F)C(F)(F)C(F)(F)C(F)(F)C(F)(F)C(F)(F)C(F)(F)C(F)(F)C(F)(F)C(F)(F)C(F)(F)C(F)(F)F)(O)=O		-11.55	0.69	4.14	P	OUT
QL00538	3-(Perfluorododecyl)-2-hydroxypropyl dihydrogen phosphate	94200-42-7	C(C(F)(F)C(F)(F)C(F)(F)C(F)(F)C(F)(F)C(F)(F)C(F)(F)C(F)(F)C(F)(F)C(F)(F)C(F)(F)C(F)(F)F)C(O)CO[P](O)(O)=O		-9.52	0.68	3.23	P	OUT
QL00539	3,3,4,4,5,5,6,6,7,7,8,8,9,9,10,10,11,11,12,12,12-Henicosafluorododecanoyl chloride	64018-26-4	C(C(F)(F)C(F)(F)C(F)(F)C(F)(F)C(F)(F)C(F)(F)C(F)(F)C(F)(F)C(F)(F)C(F)(F)F)C(Cl)=O		-8.61	0.68	2.74	P	IN
QL00540	(2-Carboxylatoethyl)-3-((perfluorodecyl)-2-hydroxypropyl)aminopropyldimethylammonium	93776-13-7	C(C(F)(F)C(F)(F)C(F)(F)C(F)(F)C(F)(F)C(F)(F)C(F)(F)C(F)(F)C(F)(F)C(F)(F)F)C(O)CNCCC[N+](CCC([O-])=O)(C)C		-9.38	0.65	3.50	P	OUT
QL00541	3-(Perfluorodecyl)-2-hydroxypropyl dihydrogen phosphate	94158-70-0	C(C(F)(F)C(F)(F)C(F)(F)C(F)(F)C(F)(F)C(F)(F)C(F)(F)C(F)(F)C(F)(F)C(F)(F)F)C(O)CO[P](O)(O)=O		-9.12	0.67	3.07	P	OUT
QL00542	N-(Carboxymethyl)-N,N-dimethyl-3-(methyl((perfluorohexyl)ethylsulfonyl)amino)-1-propanaminium	66008-71-7	C(C(F)(F)C(F)(F)C(F)(F)C(F)(F)C(F)(F)C(F)(F)F)C[S](=O)(=O)N(CCC[N+](CC([O-])=O)(C)C)C		-8.60	0.65	3.12	P	IN
QL00543	N-(2-Carboxyethyl)-N,N-dimethyl-3-[methyl[(perfluorohexyl)ethylsulfonyl]amino]-1-propanaminium	66008-72-8	C(C(F)(F)C(F)(F)C(F)(F)C(F)(F)C(F)(F)C(F)(F)F)C[S](=O)(=O)N(CCC[N+](CCC([O-])=O)(C)C)C		-8.71	0.65	3.21	P	OUT
QL00544	62 Fluorotelomer sulfonamide betaine	34455-29-3	C(C(F)(F)C(F)(F)C(F)(F)C(F)(F)C(F)(F)C(F)(F)F)C[S](=O)(=O)NCCC[N+](CC([O-])=O)(C)C		-8.47	0.65	3.10	P	OUT
QL00545	N-(2-Carboxyethyl)-N,N-dimethyl-3-[[(perfluorohexyl)ethylsulfonyl]amino]propanaminium	61798-69-4	C(C(F)(F)C(F)(F)C(F)(F)C(F)(F)C(F)(F)C(F)(F)F)C[S](=O)(=O)NCCC[N+](CCC([O-])=O)(C)C		-8.59	0.65	3.19	P	OUT
QL00546	1,4-Bis((perfluorohexyl)ethyl) sulphonatosuccinate sodium	54950-05-9	C(C(F)(F)C(F)(F)C(F)(F)C(F)(F)C(F)(F)C(F)(F)F)COC(=O)C([S]([O-])(=O)=O)CC(OCCC(F)(F)C(F)(F)C(F)(F)C(F)(F)C(F)(F)C(F)(F)F)=O.[K+]		-9.34	0.65	3.50	P	OUT
QL00547	Nonafluoro-tert-butanol	2378-02-1	C(C(F)(F)F)(C(F)(F)F)(C(F)(F)F)O		-5.53	0.68	1.20	P	IN
QL00548	Pentanoic acid, 2,2,3,3,4,4,5,5-octafluoro-, octyl ester	72828-80-9	C(C)CCC(=O)OCC(C(C(C(CCC)(F)F)(F)F)(F)F)(F)F		-6.58	0.61	2.71	P	OUT
QL00549	102 Fluorotelomer aldehyde	864551-38-2	C(C=O)C(C(C(C(C(C(C(C(C(F)(F)F)(F)F)(F)F)(F)F)(F)F)(F)F)(F)F)(F)F)(F)F		-8.50	0.68	2.73	P	IN
QL00550	Undecafluorocyclohexanemethanol dihydrogen phosphate	32582-74-4	C(C1(C(C(C(C(C1(F)F)(F)F)(F)F)(F)F)(F)F)F)OP(=O)(O)O		-7.50	0.68	2.14	P	IN
QL00551	Undecafluorocyclohexanemethanol hydrogen phosphate	32582-75-5	C(C1(C(C(C(C(C1(F)F)(F)F)(F)F)(F)F)(F)F)F)OP(=O)(O)OCC2(C(C(C(C(C2(F)F)(F)F)(F)F)(F)F)(F)F)F		-8.68	0.69	2.63	P	IN

QL00552	p,p'-Bis(undecafluorocyclohexanemethanol) pyrophosphate	48240-25-1	C(C1(C(C(C(C(C1(F)F)(F)F)(F)F)(F)F)(F)F)F)OP(=O)(O)OP(=O)(O)OCC2(C(C(C(C(C2(F)F)(F)F)(F)F)(F)F)(F)F)F		-8.90	0.69	2.80	P	OUT
QL00553	(1H,1H-Perfluoro-14-methylpentadecyl)oxirane	54009-77-7	C(C1OC1)C(F)(F)C(F)(F)C(F)(F)C(F)(F)C(F)(F)C(F)(F)C(F)(F)C(F)(C(F)(F)C(F)(F)C(F)(F)C(F)(F)C(F)(C(F)(F)F)C(F)(F)F)F		-9.51	0.69	3.10	P	OUT
QL00554	Propanedioic acid, 2-(3,3,4,4,5,5,6,6,7,7,8,8,8-tridecafluorooctyl)-, 1,3-dimethyl ester	220075-01-4	C(CC(C(=O)OC)C(=O)OC)C(C(C(C(C(C(F)(F)F)(F)F)(F)F)(F)F)(F)F)(F)F		-7.50	0.64	2.68	P	IN
QL00555	1,3-Bis[4-(ethenyloxy)butyl] 2-(perfluorohexyl)ethylpropanedioate	226409-30-9	C(CC(C(=O)OCCCCOC=C)C(=O)OCCCCOC=C)C(C(C(C(C(C(F)(F)F)(F)F)(F)F)(F)F)(F)F)(F)F		-7.37	0.60	3.23	P	OUT
QL00556	63 Fluorotelomer carboxylic acid	27854-30-4	C(CC(C(C(C(C(C(F)(F)F)(F)F)(F)F)(F)F)(F)F)(F)F)C(=O)O		-7.62	0.66	2.45	P	IN
QL00558	1-Propanesulfonic acid, 3-[[3-(dimethylamino)propyl][(heptadecafluorooctyl)sulfonyl]amino]-	72785-08-1	C(CCN([S](=O)(=O)C(C(C(C(C(C(C(C(F)(F)F)(F)F)(F)F)(F)F)(F)F)(F)F)(F)F)(F)F)CCCN(C)C)[S](=O)(=O)O		-8.34	0.66	2.93	P	IN
QL00559	N,N'-[Phosphinicobis(oxy-2,1-ethanediyl)]bis[N-ethyl-perfluorohexanesulfonamide]	67939-92-8	C(CO[P](=O)(O)OCCN([S](C(C(C(C(C(C(F)(F)F)(F)F)(F)F)(F)F)(F)F)(F)F)(=O)=O)CC)N([S](C(C(C(C(C(C(F)(F)F)(F)F)(F)F)(F)F)(F)F)(F)F)(=O)=O)CC		-9.68	0.66	3.53	P	OUT
QL00560	N,N'-[Phosphinicobis(oxy-2,1-ethanediyl)]bis[N-ethyl-perfluoropentanesulfonamide]	67939-87-1	C(CO[P](=O)(O)OCCN([S](C(C(C(C(C(F)(F)F)(F)F)(F)F)(F)F)(F)F)(=O)=O)CC)N([S](C(C(C(C(C(F)(F)F)(F)F)(F)F)(F)F)(F)F)(=O)=O)CC		-9.31	0.66	3.40	P	OUT
QL00561	N,N',N''-(Phosphoryltris(oxyethylene))tris(N-ethyl-perfluoroheptane-1-sulphonamide)	67939-94-0	C(CO[P](OCCN([S](C(C(C(C(C(C(C(F)(F)F)(F)F)(F)F)(F)F)(F)F)(F)F)(F)F)(=O)=O)CC)(OCCN([S](C(C(C(C(C(C(C(F)(F)F)(F)F)(F)F)(F)F)(F)F)(F)F)(F)F)(=O)=O)CC)=O)N([S](C(C(C(C(C(C(C(F)(F)F)(F)F)(F)F)(F)F)(F)F)(F)F)(F)F)(=O)=O)CC		-9.98	0.67	3.62	P	OUT
QL00562	162 Fluorotelomer dihydrogen phosphate	94200-55-2	C(COP(=O)(O)O)C(C(C(C(C(C(C(C(C(C(C(C(C(C(C(C(F)(F)F)(F)F)(F)F)(F)F)(F)F)(F)F)(F)F)(F)F)(F)F)(F)F)(F)F)(F)F)(F)F)(F)F)(F)F)(F)F		-10.07	0.69	3.40	P	OUT
QL00563	Poly(difluoromethylene), .alpha.,.alpha.'-[phosphinicobis(oxy-2,1-ethanediyl)]bis[.omega.-fluoro-|Ethanol, 2,2'-iminobis-, compd. with .alpha.,.alpha.'-[phosphinicobis(oxy-2,1-ethanediyl)]bis[.omega.-fluoropoly(difluoromethylene)] (11)	65530-62-3|65530-64-5	C(COP(=O)(O)OCCC(F)(F)F)C(F)(F)F		-5.99	0.61	2.35	P	OUT
QL00564	Poly(difluoromethylene), alpha-fluoro-omega-[2-sulphoethyl)-|Poly(difluoromethylene), .alpha.-fluoro-.omega.-(2-sulfoethyl)-, ammonium salt	80010-37-3|80010-38-4	C(CS(=O)(=O)O)C(F)(F)F		-4.39	0.62	1.37	P	OUT
QL00565	N-ethyl-N-[2-(phosphonooxy)ethyl]perfluorooctanesulfonamide	3820-83-5	C(F)(F)(F)C(F)(F)C(F)(F)C(F)(F)C(F)(F)C(F)(F)C(F)(F)C(F)(F)[S](=O)(=O)N(CC)CCO[P](=O)(O[H])O[H]		-8.61	0.67	2.94	P	IN
QL00566	Bis(2-{ethyl[(perfluorooctyl)sulfonyl]amino}ethyl) hydrogen phosphate	2965-52-8	C(F)(F)(F)C(F)(F)C(F)(F)C(F)(F)C(F)(F)C(F)(F)C(F)(F)C(F)(F)[S](=O)(=O)N(CC)CCO[P](=O)(OCCN(CC)[S](C(C(C(C(C(C(C(C(F)(F)F)(F)F)(F)F)(F)F)(F)F)(F)F)(F)F)(F)F)(=O)=O)O[H]		-10.31	0.67	3.75	P	OUT
QL00567	1-Octanesulfonamide, N,N',N''-[phosphinylidynetris(oxy-2,1-ethanediyl)]tris[N-ethyl-1,1,2,2,3,3,4,4,5,5,6,6,7,7,8,8,8-heptadecafluoro-	2250-98-8	C(F)(F)(F)C(F)(F)C(F)(F)C(F)(F)C(F)(F)C(F)(F)C(F)(F)C(F)(F)[S](=O)(=O)N(CC)CCO[P](=O)(OCCN(CC)[S](C(C(C(C(C(C(C(C(F)(F)F)(F)F)(F)F)(F)F)(F)F)(F)F)(F)F)(F)F)(=O)=O)OCCN(CC)[S](=O)(=O)C(C(C(C(C(C(C(C(F)(F)F)(F)F)(F)F)(F)F)(F)F)(F)F)(F)F)(F)F		-10.28	0.67	3.73	P	OUT
QL00568	1,1,2,2,3,3,4,4,5,5-Decafluoro-1-((trifluorovinyl)oxy)pentane	73928-40-2	C(F)(F)=C(F)OC(F)(F)C(F)(F)C(F)(F)C(F)(F)C(F)F		-6.90	0.65	2.24	P	IN
QL00569	(Perfluorohexadecyl)ethyl iodide	65150-94-9	C(I)CC(F)(F)C(F)(F)C(F)(F)C(F)(F)C(F)(F)C(F)(F)C(F)(F)C(F)(F)C(F)(F)C(F)(F)C(F)(F)C(F)(F)C(F)(F)C(F)(F)C(F)(F)C(F)(F)F		-9.78	0.68	3.27	P	OUT
QL00570	2-(Perfluorotetradecyl)-1-iodoethane	65510-55-6	C(I)CC(F)(F)C(F)(F)C(F)(F)C(F)(F)C(F)(F)C(F)(F)C(F)(F)C(F)(F)C(F)(F)C(F)(F)C(F)(F)C(F)(F)C(F)(F)C(F)(F)F		-9.41	0.68	3.11	P	OUT
QL00571	1,1,1,2,2,3,3,4,4,5,5,6,6,7,7,8,8,9,9-nonadecafluoro-11-iodoundecane	65510-56-7	C(I)CC(F)(F)C(F)(F)C(F)(F)C(F)(F)C(F)(F)C(F)(F)C(F)(F)C(F)(F)C(F)(F)F		-8.33	0.67	2.68	P	IN
QL00572	1-Iodo-1H,1H,2H,2H-perfluorononane	2043-52-9	C(I)CC(F)(F)C(F)(F)C(F)(F)C(F)(F)C(F)(F)C(F)(F)C(F)(F)F		-7.69	0.67	2.44	P	IN
QL00573	1-Iodo-1H,1H,2H,2H-perfluoroheptane	1682-31-1	C(I)CC(F)(F)C(F)(F)C(F)(F)C(F)(F)C(F)(F)F		-6.87	0.66	2.17	P	IN
QL00574	N-[3-Dimethylaminopropyl]-N-2-[2-(2-hydroxyethoxy)ethoxy]ethylperfluorohexanesulfonamide	73772-34-6	C(N([S](=O)(=O)C(C(C(C(C(C(F)(F)F)(F)F)(F)F)(F)F)(F)F)(F)F)CCOCCOCCO)CCN(C)C		-7.72	0.62	3.11	P	OUT
QL00575	1,1,2,2,3,3,4,4,4-Nonafluoro-N,N-bis(2-hydroxyethyl)butane-1-sulphonamide	34455-00-0	C(N([S](=O)(=O)C(C(C(C(F)(F)F)(F)F)(F)F)(F)F)CCO)CO		-6.33	0.63	2.25	P	OUT
QL00576	N-(4-Hydroxybutyl)-N-methylperfluorooctanesulfonamide	68239-73-6	C(N([S](=O)(=O)C(F)(F)C(F)(F)C(F)(F)C(F)(F)C(F)(F)C(F)(F)C(F)(F)C(F)(F)F)C)CCCO		-8.47	0.66	2.94	P	IN
QL00577	4-{[(Perfluorooctyl)sulfonyl](methyl)amino}butyl 2-methyl-2-propenoate	61577-14-8	C(N([S](=O)(=O)C(F)(F)C(F)(F)C(F)(F)C(F)(F)C(F)(F)C(F)(F)C(F)(F)C(F)(F)F)C)CCCOC(=O)C(=C)C		-8.99	0.66	3.25	P	OUT
QL00578	4-(Methyl((perfluorooctyl)sulfonyl)amino)butyl 2-propenoate	58920-31-3	C(N([S](=O)(=O)C(F)(F)C(F)(F)C(F)(F)C(F)(F)C(F)(F)C(F)(F)C(F)(F)C(F)(F)F)C)CCCOC(=O)C=C		-8.87	0.65	3.25	P	OUT

QL00579	2-(((Heptadecafluorooctyl)sulfonyl)methylamino)ethyl methacrylate	14650-24-9	C(N([S](=O)(=O)C(F)(F)C(F)(F)C(F)(F)C(F)(F)C(F)(F)C(F)(F)C(F)(F)C(F)(F)F)C)COC(=O)C(=C)C		-8.89	0.67	3.08	P	OUT
QL00580	Glycine, N-ethyl-N-[(heptadecafluorooctyl)sulfonyl]-, ethyl ester	1869-77-8	C(N([S](=O)(=O)C(F)(F)C(F)(F)C(F)(F)C(F)(F)C(F)(F)C(F)(F)C(F)(F)C(F)(F)F)CC(=O)OCC)C		-8.33	0.65	2.94	P	IN
QL00581	N-Allyl-N-ethyl-perfluorooctanesulfonamide	24924-36-5	C(N([S](=O)(=O)C(F)(F)C(F)(F)C(F)(F)C(F)(F)C(F)(F)C(F)(F)C(F)(F)C(F)(F)F)CC=C)C		-8.12	0.66	2.74	P	IN
QL00582	Potassium N-((heptadecafluorooctyl)sulphonyl)-N-propylglycinate	55910-10-6	C(N([S](=O)(=O)C(F)(F)C(F)(F)C(F)(F)C(F)(F)C(F)(F)C(F)(F)C(F)(F)C(F)(F)F)CCC)C([O-])=O.[K+]		-7.79	0.64	2.85	P	OUT
QL00583	N,N,N-Trimethyl-3-(carboxymethyl(perfluorooctylsulfonyl)amino)-1-propanaminium	68318-36-5	C(N([S](=O)(=O)C(F)(F)C(F)(F)C(F)(F)C(F)(F)C(F)(F)C(F)(F)C(F)(F)C(F)(F)F)CCC[N+](C)(C)C)C([O-])=O		-8.56	0.66	2.94	P	IN
QL00584	Phosphonic acid, [3-[ethyl[(heptadecafluorooctyl)sulfonyl]amino]propyl]-	71463-78-0	C(N([S](=O)(=O)C(F)(F)C(F)(F)C(F)(F)C(F)(F)C(F)(F)C(F)(F)C(F)(F)C(F)(F)F)CCC[P](=O)(O)O)C		-8.61	0.67	2.94	P	IN
QL00585	Phosphonic acid, [3-[ethyl[(heptadecafluorooctyl)sulfonyl]amino]propyl]-, diethyl ester	71463-80-4	C(N([S](=O)(=O)C(F)(F)C(F)(F)C(F)(F)C(F)(F)C(F)(F)C(F)(F)C(F)(F)C(F)(F)F)CCC[P](=O)(OCC)OCC)C		-8.35	0.64	3.12	P	OUT
QL00586	N-Butyl-N-(2-hydroxyethyl)perfluoro-1-octanesulfonamide	2263-09-4	C(N([S](=O)(=O)C(F)(F)C(F)(F)C(F)(F)C(F)(F)C(F)(F)C(F)(F)C(F)(F)C(F)(F)F)CCCC)CO		-8.13	0.65	2.90	P	IN
QL00587	Perfluorooctylsulfonylpropylaminoethyl acrylate	2357-60-0	C(N([S](=O)(=O)C(F)(F)C(F)(F)C(F)(F)C(F)(F)C(F)(F)C(F)(F)C(F)(F)C(F)(F)F)CCOC(=O)C=C)CC		-8.41	0.65	3.08	P	OUT
QL00588	N-(4-Hydroxybutyl)-N-methyl-perfluoroheptanesulfonamide	68298-89-5	C(N([S](=O)(=O)C(F)(F)C(F)(F)C(F)(F)C(F)(F)C(F)(F)C(F)(F)C(F)(F)F)C)CCCO		-8.30	0.66	2.90	P	IN
QL00589	4-[Methyl[(pentadecafluoroheptyl)sulfonyl]amino]butyl methacrylate	67906-38-1	C(N([S](=O)(=O)C(F)(F)C(F)(F)C(F)(F)C(F)(F)C(F)(F)C(F)(F)C(F)(F)F)C)CCCOC(=O)C(=C)C		-8.66	0.65	3.13	P	IN
QL00590	2-Propenoic acid, 4-[methyl[(pentadecafluoroheptyl)sulfonyl]amino]butyl ester	68227-97-4	C(N([S](=O)(=O)C(F)(F)C(F)(F)C(F)(F)C(F)(F)C(F)(F)C(F)(F)C(F)(F)F)C)CCCOC(=O)C=C		-8.53	0.65	3.14	P	OUT
QL00591	N-(2-Hydroxyethyl)-N-methyl-perfluoroheptanesulfonamide	68555-76-0	C(N([S](=O)(=O)C(F)(F)C(F)(F)C(F)(F)C(F)(F)C(F)(F)C(F)(F)C(F)(F)F)C)CO		-8.16	0.67	2.69	P	IN
QL00592	2-Propenoic acid, 2-methyl-, 2-[methyl[(pentadecafluoroheptyl)sulfonyl]amino]ethyl ester	67939-96-2	C(N([S](=O)(=O)C(F)(F)C(F)(F)C(F)(F)C(F)(F)C(F)(F)C(F)(F)C(F)(F)F)C)COC(=O)C(=C)C		-8.54	0.66	2.95	P	IN
QL00593	2-Propenoic acid, 2-[methyl[(pentadecafluoroheptyl)sulfonyl]amino]ethyl ester	68084-62-8	C(N([S](=O)(=O)C(F)(F)C(F)(F)C(F)(F)C(F)(F)C(F)(F)C(F)(F)C(F)(F)F)C)COC(=O)C=C		-8.40	0.66	2.95	P	IN
QL00594	Glycine, N-ethyl-N-[(pentadecafluoroheptyl)sulfonyl]-	68957-63-1	C(N([S](=O)(=O)C(F)(F)C(F)(F)C(F)(F)C(F)(F)C(F)(F)C(F)(F)C(F)(F)F)CC(=O)O)C		-8.06	0.66	2.69	P	IN
QL00595	Glycine, N-ethyl-N-[(pentadecafluoroheptyl)sulfonyl]-, ethyl ester	68957-54-0	C(N([S](=O)(=O)C(F)(F)C(F)(F)C(F)(F)C(F)(F)C(F)(F)C(F)(F)C(F)(F)F)CC(=O)OCC)C		-8.14	0.65	2.90	P	IN
QL00596	Bis(2-{ethyl[(perfluoroheptyl)sulfonyl]amino}ethyl) hydrogen phosphate	67939-93-9	C(N([S](=O)(=O)C(F)(F)C(F)(F)C(F)(F)C(F)(F)C(F)(F)C(F)(F)C(F)(F)F)CC)CO[P](OCCN([S](=O)(=O)C(F)(F)C(F)(F)C(F)(F)C(F)(F)C(F)(F)C(F)(F)C(F)(F<br>)F)CC)(O)=O		-10.01	0.67	3.64	P	OUT
QL00597	N-Ethyl-N-2-propenylperfluoroheptanesulfonamide	67906-41-6	C(N([S](=O)(=O)C(F)(F)C(F)(F)C(F)(F)C(F)(F)C(F)(F)C(F)(F)C(F)(F)F)CC=C)C		-7.92	0.66	2.69	P	IN
QL00598	Phosphonic acid, [3-[ethyl[(pentadecafluoroheptyl)sulfonyl]amino]propyl]-	71463-79-1	C(N([S](=O)(=O)C(F)(F)C(F)(F)C(F)(F)C(F)(F)C(F)(F)C(F)(F)C(F)(F)F)CCC[P](=O)(O)O)C		-8.44	0.66	2.89	P	IN
QL00599	Phosphonic acid, [3-[ethyl[(pentadecafluoroheptyl)sulfonyl]amino]propyl]-, diethyl ester	71463-81-5	C(N([S](=O)(=O)C(F)(F)C(F)(F)C(F)(F)C(F)(F)C(F)(F)C(F)(F)C(F)(F)F)CCC[P](=O)(OCC)OCC)C		-8.16	0.64	3.08	P	OUT
QL00600	N-Butyl-N-(2-hydroxyethyl)perfluoroheptanesulfonamide	68310-02-1	C(N([S](=O)(=O)C(F)(F)C(F)(F)C(F)(F)C(F)(F)C(F)(F)C(F)(F)C(F)(F)F)CCCC)CO		-7.71	0.65	2.75	P	IN
QL00601	N-Ethyl--N-[2-(phosphonooxy)ethyl]perfluoroheptanesulfonamide	67923-61-9	C(N([S](=O)(=O)C(F)(F)C(F)(F)C(F)(F)C(F)(F)C(F)(F)C(F)(F)C(F)(F)F)CCO[P](=O)(O)O)C		-8.44	0.66	2.89	P	IN
QL00602	2-Propenoic acid, 2-methyl-, 2-[ethyl[(pentadecafluoroheptyl)sulfonyl]amino]ethyl ester	67939-36-0	C(N([S](=O)(=O)C(F)(F)C(F)(F)C(F)(F)C(F)(F)C(F)(F)C(F)(F)C(F)(F)F)CCOC(=O)C(=C)C)C		-8.33	0.65	2.95	P	IN
QL00603	2-Propenoic acid, 2-[ethyl[(pentadecafluoroheptyl)sulfonyl]amino]ethyl ester	59071-10-2	C(N([S](=O)(=O)C(F)(F)C(F)(F)C(F)(F)C(F)(F)C(F)(F)C(F)(F)C(F)(F)F)CCOC(=O)C=C)C		-8.19	0.65	2.95	P	IN
QL00604	2-Propenoic acid, 2-[butyl[(pentadecafluoroheptyl)sulfonyl]amino]ethyl ester	68298-60-2	C(N([S](=O)(=O)C(F)(F)C(F)(F)C(F)(F)C(F)(F)C(F)(F)C(F)(F)C(F)(F)F)CCOC(=O)C=C)CCC		-7.93	0.64	2.95	P	OUT

QL00605	1-Hexanesulfonamide, 1,1,2,2,3,3,4,4,5,5,6,6,6-tridecafluoro-N-(4-hydroxybutyl)-N-methyl-	68239-74-7	C(N([S](=O)(=O)C(F)(F)C(F)(F)C(F)(F)C(F)(F)C(F)(F)C(F)(F)F)C)CCCO		-7.88	0.65	2.75	P	IN
QL00606	4-[Methyl[(tridecafluorohexyl)sulfonyl]amino]butyl methacrylate	67939-61-1	C(N([S](=O)(=O)C(F)(F)C(F)(F)C(F)(F)C(F)(F)C(F)(F)C(F)(F)F)C)CCCOC(=O)C(=C)C		-8.46	0.65	3.09	P	IN
QL00607	2-Propenoic acid, 4-[methyl[(tridecafluorohexyl)sulfonyl]amino]butyl ester	68227-98-5	C(N([S](=O)(=O)C(F)(F)C(F)(F)C(F)(F)C(F)(F)C(F)(F)C(F)(F)F)C)CCCOC(=O)C=C		-8.32	0.64	3.09	P	OUT
QL00608	N-(2-Hydroxyethyl)-N-methylperfluorohexanesulfonamide	68555-75-9	C(N([S](=O)(=O)C(F)(F)C(F)(F)C(F)(F)C(F)(F)C(F)(F)C(F)(F)F)C)CO		-7.71	0.66	2.51	P	IN
QL00609	2-Propenoic acid, 2-methyl-, 2-[methyl[(tridecafluorohexyl)sulfonyl]amino]ethyl ester	67584-61-6	C(N([S](=O)(=O)C(F)(F)C(F)(F)C(F)(F)C(F)(F)C(F)(F)C(F)(F)F)C)COC(=O)C(=C)C		-8.37	0.66	2.91	P	IN
QL00610	2-Propenoic acid, 2-[methyl[(tridecafluorohexyl)sulfonyl]amino]ethyl ester	67584-57-0	C(N([S](=O)(=O)C(F)(F)C(F)(F)C(F)(F)C(F)(F)C(F)(F)C(F)(F)F)C)COC(=O)C=C		-8.22	0.65	2.91	P	IN
QL00611	N-Ethyl-N-((tridecafluorohexyl)sulfonyl)glycine	68957-32-4	C(N([S](=O)(=O)C(F)(F)C(F)(F)C(F)(F)C(F)(F)C(F)(F)C(F)(F)F)CC(=O)O)C		-7.62	0.66	2.52	P	IN
QL00612	Glycine, N-ethyl-N-[(tridecafluorohexyl)sulfonyl]-, ethyl ester	68957-53-9	C(N([S](=O)(=O)C(F)(F)C(F)(F)C(F)(F)C(F)(F)C(F)(F)C(F)(F)F)CC(=O)OCC)C		-7.71	0.65	2.75	P	IN
QL00613	3-(Carboxymethyl(perfluorohexylsulfonyl)amino)-N,N,N-trimethylpropanaminium	38850-52-1	C(N([S](=O)(=O)C(F)(F)C(F)(F)C(F)(F)C(F)(F)C(F)(F)C(F)(F)F)CCC[N+](C)(C)C)C([O-])=O		-7.99	0.66	2.74	P	IN
QL00614	(2-Hydroxyethyl)dimethyl(3-((3-sulfopropyl)((tridecafluorohexyl)sulfonyl)amino)propyl)ammonium	38850-58-7	C(N([S](=O)(=O)C(F)(F)C(F)(F)C(F)(F)C(F)(F)C(F)(F)C(F)(F)F)CCC[S]([O-])(=O)=O)CC[N+](CCO)(C)C		-8.00	0.65	2.90	P	IN
QL00615	1-Hexanesulfonamide, N-ethyl-1,1,2,2,3,3,4,4,5,5,6,6,6-tridecafluoro-N-(2-hydroxyethyl)-	34455-03-3	C(N([S](=O)(=O)C(F)(F)C(F)(F)C(F)(F)C(F)(F)C(F)(F)C(F)(F)F)CCO)C		-7.45	0.65	2.51	P	IN
QL00616	N-Ethyl-N-[2-(phosphonooxy)ethyl]perfluorohexanesulfonamide	67969-65-7	C(N([S](=O)(=O)C(F)(F)C(F)(F)C(F)(F)C(F)(F)C(F)(F)C(F)(F)F)CCO[P](=O)(O)O)C		-8.05	0.66	2.74	P	IN
QL00617	2-Propenoic acid, 2-methyl-, 2-[ethyl[(tridecafluorohexyl)sulfonyl]amino]ethyl ester	67906-70-1	C(N([S](=O)(=O)C(F)(F)C(F)(F)C(F)(F)C(F)(F)C(F)(F)C(F)(F)F)CCOC(=O)C(=C)C)C		-8.14	0.65	2.91	P	IN
QL00618	2-Propenoic acid, 2-[ethyl[(tridecafluorohexyl)sulfonyl]amino]ethyl ester	1893-52-3	C(N([S](=O)(=O)C(F)(F)C(F)(F)C(F)(F)C(F)(F)C(F)(F)C(F)(F)F)CCOC(=O)C=C)C		-7.99	0.64	2.91	P	IN
QL00619	1-Pentanesulfonamide, 1,1,2,2,3,3,4,4,5,5,5-undecafluoro-N-(4-hydroxybutyl)-N-methyl-	68239-72-5	C(N([S](=O)(=O)C(F)(F)C(F)(F)C(F)(F)C(F)(F)C(F)(F)F)C)CCCO		-7.66	0.65	2.72	P	IN
QL00620	4-[Methyl[(undecafluoropentyl)sulfonyl]amino]butyl methacrylate	67906-40-5	C(N([S](=O)(=O)C(F)(F)C(F)(F)C(F)(F)C(F)(F)C(F)(F)F)C)CCCOC(=O)C(=C)C		-8.07	0.64	2.96	P	OUT
QL00621	2-Propenoic acid, 4-[methyl[(undecafluoropentyl)sulfonyl]amino]butyl ester	68227-99-6	C(N([S](=O)(=O)C(F)(F)C(F)(F)C(F)(F)C(F)(F)C(F)(F)F)C)CCCOC(=O)C=C		-7.90	0.64	2.96	P	OUT
QL00622	2-Propenoic acid, 2-methyl-, 2-[methyl[(undecafluoropentyl)sulfonyl]amino]ethyl ester	67584-60-5	C(N([S](=O)(=O)C(F)(F)C(F)(F)C(F)(F)C(F)(F)C(F)(F)F)C)COC(=O)C(=C)C		-7.96	0.65	2.76	P	IN
QL00623	Glycine, N-ethyl-N-[(undecafluoropentyl)sulfonyl]-	68957-31-3	C(N([S](=O)(=O)C(F)(F)C(F)(F)C(F)(F)C(F)(F)C(F)(F)F)CC(=O)O)C		-7.36	0.65	2.46	P	IN
QL00624	Glycine, N-ethyl-N-[(undecafluoropentyl)sulfonyl]-, ethyl ester	68555-79-3	C(N([S](=O)(=O)C(F)(F)C(F)(F)C(F)(F)C(F)(F)C(F)(F)F)CC(=O)OCC)C		-7.47	0.64	2.71	P	IN
QL00625	N-Ethyl-N-(2-(phosphonooxy)ethyl)perfluoropentanesulfonamide	67939-90-6	C(N([S](=O)(=O)C(F)(F)C(F)(F)C(F)(F)C(F)(F)C(F)(F)F)CCO[P](=O)(O)O)C		-7.83	0.65	2.69	P	IN
QL00626	2-Propenoic acid, 2-methyl-, 2-[ethyl[(undecafluoropentyl)sulfonyl]amino]ethyl ester	67906-73-4	C(N([S](=O)(=O)C(F)(F)C(F)(F)C(F)(F)C(F)(F)C(F)(F)F)CCOC(=O)C(=C)C)C		-7.71	0.65	2.76	P	IN
QL00627	2-Propenoic acid, 2-[ethyl[(undecafluoropentyl)sulfonyl]amino]ethyl ester	68298-06-6	C(N([S](=O)(=O)C(F)(F)C(F)(F)C(F)(F)C(F)(F)C(F)(F)F)CCOC(=O)C=C)C		-7.54	0.64	2.76	P	OUT
QL00628	1-Butanesulfonamide, 1,1,2,2,3,3,4,4,4-nonafluoro-N-(4-hydroxybutyl)-N-methyl-	812-94-2	C(N([S](=O)(=O)C(F)(F)C(F)(F)C(F)(F)C(F)(F)F)C)CCCO		-7.12	0.64	2.54	P	IN
QL00629	4-[Methyl[(nonafluorobutyl)sulfonyl]amino]butyl methacrylate	67906-39-2	C(N([S](=O)(=O)C(F)(F)C(F)(F)C(F)(F)C(F)(F)F)C)CCCOC(=O)C(=C)C		-7.75	0.64	2.88	P	OUT

QL00630	2-Propenoic acid, 4-[methyl[(nonafluorobutyl)sulfonyl]amino]butyl ester	1492-87-1	C(N([S](=O)(=O)C(F)(F)C(F)(F)C(F)(F)C(F)(F)F)C)CCCOC(=O)C=C		-7.57	0.63	2.89	P	OUT
QL00631	2-Propenoic acid, 2-methyl-, 2-[methyl[(nonafluorobutyl)sulfonyl]amino]ethyl ester	67584-59-2	C(N([S](=O)(=O)C(F)(F)C(F)(F)C(F)(F)C(F)(F)F)C)COC(=O)C(=C)C		-7.70	0.65	2.70	P	IN
QL00632	N-Ethyl-N-[(nonafluorobutyl)sulfonyl]glycine	68957-33-5	C(N([S](=O)(=O)C(F)(F)C(F)(F)C(F)(F)C(F)(F)F)CC(=O)O)C		-6.79	0.65	2.25	P	IN
QL00633	Glycine, N-ethyl-N-[(nonafluorobutyl)sulfonyl]-, ethyl ester	67584-63-8	C(N([S](=O)(=O)C(F)(F)C(F)(F)C(F)(F)C(F)(F)F)CC(=O)OCC)C		-6.93	0.63	2.53	P	OUT
QL00634	N,N'-Phosphinicobis(oxy-2,1-ethanediyl)bis(N-ethyl-perfluoro-1-butanesulfonamide)	67939-91-7	C(N([S](=O)(=O)C(F)(F)C(F)(F)C(F)(F)C(F)(F)F)CC)CO[P](=O)(O)OCCN([S](=O)(=O)C(F)(F)C(F)(F)C(F)(F)C(F)(F)F)CC		-8.89	0.65	3.26	P	OUT
QL00635	1-Butanesulfonamide, N-ethyl-1,1,2,2,3,3,4,4,4-nonafluoro-N-(2-hydroxyethyl)-	34449-89-3	C(N([S](=O)(=O)C(F)(F)C(F)(F)C(F)(F)C(F)(F)F)CCO)C		-6.57	0.64	2.24	P	IN
QL00636	1-Butanesulfonamide, N-ethyl-1,1,2,2,3,3,4,4,4-nonafluoro-N-[2-(phosphonooxy)ethyl]-	67939-89-3	C(N([S](=O)(=O)C(F)(F)C(F)(F)C(F)(F)C(F)(F)F)CCO[P](=O)(O)O)C		-7.34	0.65	2.52	P	IN
QL00637	2-Propenoic acid, 2-methyl-, 2-[ethyl[(nonafluorobutyl)sulfonyl]amino]ethyl ester	67939-33-7	C(N([S](=O)(=O)C(F)(F)C(F)(F)C(F)(F)C(F)(F)F)CCOC(=O)C(=C)C)C		-7.42	0.64	2.70	P	OUT
QL00638	2-Propenoic acid, 2-[ethyl[(nonafluorobutyl)sulfonyl]amino]ethyl ester	17329-79-2	C(N([S](=O)(=O)C(F)(F)C(F)(F)C(F)(F)C(F)(F)F)CCOC(=O)C=C)C		-7.23	0.63	2.70	P	OUT
QL00639	2-[N-(1,1-Dihydroperfluorotridecylsulfonyl)-N-methylamino]ethyl acrylate	66008-67-1	C(N([S](=O)(=O)CC(F)(F)C(F)(F)C(F)(F)C(F)(F)C(F)(F)C(F)(F)C(F)(F)C(F)(F)C(F)(F)C(F)(F)C(F)(F)C(F)(F)F)C)COC(=O)C=C		-9.59	0.67	3.41	P	OUT
QL00640	2-[N-(1,1-Dihydroperfluoroundecylsulfonyl)-N-methylamino]ethyl acrylate	66008-68-2	C(N([S](=O)(=O)CC(F)(F)C(F)(F)C(F)(F)C(F)(F)C(F)(F)C(F)(F)C(F)(F)C(F)(F)C(F)(F)C(F)(F)F)C)COC(=O)C=C		-9.20	0.66	3.27	P	OUT
QL00641	2-(N-((Perfluorooctyl)methylsulfonyl)-N-methylamino)ethyl acrylate	66008-69-3	C(N([S](=O)(=O)CC(F)(F)C(F)(F)C(F)(F)C(F)(F)C(F)(F)C(F)(F)C(F)(F)C(F)(F)F)C)COC(=O)C=C		-8.74	0.66	3.12	P	IN
QL00642	2-(Methyl((perfluorohexyl)methylsulfonyl)amino)ethyl 2-propenoate	66008-70-6	C(N([S](=O)(=O)CC(F)(F)C(F)(F)C(F)(F)C(F)(F)C(F)(F)C(F)(F)F)C)COC(=O)C=C		-8.20	0.65	2.96	P	IN
QL00643	2-[Methyl[(perfluorododecyl)ethylsulfonyl]amino]ethyl 2-propenoate	72276-06-3	C(N([S](=O)(=O)CCC(F)(F)C(F)(F)C(F)(F)C(F)(F)C(F)(F)C(F)(F)C(F)(F)C(F)(F)C(F)(F)C(F)(F)C(F)(F)C(F)(F)F)C)COC(=O)C=C		-9.74	0.67	3.51	P	OUT
QL00644	(((Perfluorodecyl)ethylsulphonyl)methylamino)ethyl acrylate	72276-05-2	C(N([S](=O)(=O)CCC(F)(F)C(F)(F)C(F)(F)C(F)(F)C(F)(F)C(F)(F)C(F)(F)C(F)(F)C(F)(F)C(F)(F)F)C)COC(=O)C=C		-9.37	0.66	3.39	P	OUT
QL00645	2-(1-(Perfluorooctyl)-2-ethylsulfonylmethylamino)ethyl 2-propenoate	48077-95-8	C(N([S](=O)(=O)CCC(F)(F)C(F)(F)C(F)(F)C(F)(F)C(F)(F)C(F)(F)C(F)(F)C(F)(F)F)C)COC(=O)C=C		-8.87	0.66	3.22	P	OUT
QL00646	2-[Methyl[(perfluorohexyl)ethylsulfonyl]amino]ethyl 2-propenoate	49859-70-3	C(N([S](=O)(=O)CCC(F)(F)C(F)(F)C(F)(F)C(F)(F)C(F)(F)C(F)(F)F)C)COC(=O)C=C		-8.36	0.65	3.07	P	OUT
QL00649	Heptadecafluoro-N-(2-hydroxyethyl)-N-propyloctanesulphonamide	4236-15-1	C(N([S](C(C(C(C(C(C(C(C(F)(F)F)(F)F)(F)F)(F)F)(F)F)(F)F)(F)F)(F)F)(=O)=O)CCC)CO		-7.96	0.66	2.75	P	IN
QL00650	3-((3-(Dimethylamino)propyl)((tridecafluorohexyl)sulfonyl)amino)-1-propanesulfonic acid	38850-60-1	C(N([S](C(C(C(C(C(C(F)(F)F)(F)F)(F)F)(F)F)(F)F)(F)F)(=O)=O)CCCN(C)C)CC[S](=O)(=O)O		-7.75	0.65	2.73	P	IN
QL00651	1-((3-(Dimethylamino)propyl)amino)-(3-(perfluoro-9-methyldecyl))propanol	94159-83-8	C(N(C)C)CCNC(O)CCC(F)(F)C(F)(F)C(F)(F)C(F)(F)C(F)(F)C(F)(F)C(F)(F)C(F)(F)C(F)(C(F)(F)F)C(F)(F)F		-9.01	0.66	3.24	P	OUT
QL00652	1-Octanesulfonamide, 1,1,2,2,3,3,4,4,5,5,6,6,7,7,8,8,8-heptadecafluoro-N-2-propenyl-	423-86-9	C(N[S](=O)(=O)C(F)(F)C(F)(F)C(F)(F)C(F)(F)C(F)(F)C(F)(F)C(F)(F)C(F)(F)F)C=C		-8.29	0.67	2.73	P	IN
QL00653	1-Propanaminium, 3-[[(heptadecafluorooctyl)sulfonyl]amino]-N,N,N-trimethyl-, sulfate (21) (TSCA, NDSL, ENCS)	70225-26-2	C(N[S](=O)(=O)C(F)(F)C(F)(F)C(F)(F)C(F)(F)C(F)(F)C(F)(F)C(F)(F)C(F)(F)F)CC[N+](C)(C)C.C(N[S](=O)(=O)C(F)(F)C(F)(F)C(F)(F)C(F)(F)C(F)(F)C(F)(F)C(F)(F)C(F)(F)F)CC[N+](C)(C)C.O=[S]([O-])([O-])=O		-9.03	0.67	3.14	P	OUT
QL00654	Perfluorooctane sulfonamido amine	13417-01-1	C(N[S](=O)(=O)C(F)(F)C(F)(F)C(F)(F)C(F)(F)C(F)(F)C(F)(F)C(F)(F)C(F)(F)F)CCN(C)C		-8.57	0.66	2.94	P	IN
QL00655	1-Heptanesulfonamide, N-ethyl-1,1,2,2,3,3,4,4,5,5,6,6,7,7,7-pentadecafluoro-	68957-62-0	C(N[S](=O)(=O)C(F)(F)C(F)(F)C(F)(F)C(F)(F)C(F)(F)C(F)(F)C(F)(F)F)C		-7.91	0.67	2.50	P	IN
QL00656	1-Heptanesulfonamide, 1,1,2,2,3,3,4,4,5,5,6,6,7,7,7-pentadecafluoro-N-2-propenyl-	67584-49-0	C(N[S](=O)(=O)C(F)(F)C(F)(F)C(F)(F)C(F)(F)C(F)(F)C(F)(F)C(F)(F)F)C=C		-8.10	0.66	2.68	P	IN
QL00657	Perfluoroheptane sulfonamido amine	67584-54-7	C(N[S](=O)(=O)C(F)(F)C(F)(F)C(F)(F)C(F)(F)C(F)(F)C(F)(F)C(F)(F)F)CCN(C)C		-8.40	0.66	2.90	P	IN
QL00658	1-Hexanesulfonamide, 1,1,2,2,3,3,4,4,5,5,6,6,6-tridecafluoro-N-2-propenyl-	67584-48-9	C(N[S](=O)(=O)C(F)(F)C(F)(F)C(F)(F)C(F)(F)C(F)(F)C(F)(F)F)C=C		-7.64	0.66	2.51	P	IN
QL00659	Perfluorohexane sulfonamido amine	50598-28-2	C(N[S](=O)(=O)C(F)(F)C(F)(F)C(F)(F)C(F)(F)C(F)(F)C(F)(F)F)CCN(C)C		-7.99	0.66	2.75	P	IN

QL00660	1-Pentanesulfonamide, 1,1,2,2,3,3,4,4,5,5,5-undecafluoro-N-2-propenyl-	335-97-7	C(N[S](=O)(=O)C(F)(F)C(F)(F)C(F)(F)C(F)(F)C(F)(F)F)C=C		-7.41	0.66	2.46	P	IN
QL00661	Perfluoropentane sulfonamido amine	68555-78-2	C(N[S](=O)(=O)C(F)(F)C(F)(F)C(F)(F)C(F)(F)C(F)(F)F)CCN(C)C		-7.79	0.65	2.72	P	IN
QL00662	N-Allylperfluorobutanesulfonamide	40630-65-7	C(N[S](=O)(=O)C(F)(F)C(F)(F)C(F)(F)C(F)(F)F)C=C		-6.81	0.65	2.25	P	IN
QL00663	3-((Perfluoroheptyl)sulfonylamino)-N,N,N-trimethyl-1-propanaminium iodide	67584-58-1	C(N[S](C(C(C(C(C(C(C(F)(F)F)(F)F)(F)F)(F)F)(F)F)(F)F)(F)F)(=O)=O)CC[N+](C)(C)C.[I-]		-8.28	0.65	3.00	P	IN
QL00664	3-((Perfluorohexylsulfonyl)amino)-N,N,N-trimethyl-1-propanaminium iodide	68957-58-4	C(N[S](C(C(C(C(C(C(F)(F)F)(F)F)(F)F)(F)F)(F)F)(F)F)(=O)=O)CC[N+](C)(C)C.[I-]		-7.86	0.64	2.86	P	IN
QL00665	3-((Perfluoropentylsulfonyl)amino)-N,N,N-trimethylpropanaminium chloride	68957-55-1	C(N[S](C(C(C(C(C(F)(F)F)(F)F)(F)F)(F)F)(F)F)(=O)=O)CC[N+](C)(C)C.[Cl-]		-7.64	0.64	2.82	P	OUT
QL00666	Trimethyl-3-((perfluorobutylsulphonyl)amino)propylammonium chloride	53518-00-6	C(N[S](C(C(C(C(F)(F)F)(F)F)(F)F)(F)F)(=O)=O)CC[N+](C)(C)C.[Cl-]		-7.12	0.63	2.66	P	OUT
QL00667	S-(3-(Ethoxycarbonylaminomethyl)-3-(perfluorohexyl)propyl)sulfurothioate sodium	67846-66-6	C(NC(OCC)=O)C(C(F)(F)C(F)(F)C(F)(F)C(F)(F)C(F)(F)C(F)(F)F)CCS[S]([O-])(=O)=O.[K+]		-7.16	0.62	2.84	P	OUT
QL00668	perfluoroalkylamido betaine-related, n=14_pos		C(NCCC[N+](CC(O)=O)(C)C)(=O)C(F)(F)C(F)(F)C(F)(F)C(F)(F)C(F)(F)C(F)(F)C(F)(F)C(F)(F)C(F)(F)C(F)(F)C(F)(F)C(F)(F)C(F)(F)C(F)(F)F		-9.93	0.67	3.53	P	OUT
QL00669	perfluoroalkylamido betaine-related, n=13_pos		C(NCCC[N+](CC(O)=O)(C)C)(=O)C(F)(F)C(F)(F)C(F)(F)C(F)(F)C(F)(F)C(F)(F)C(F)(F)C(F)(F)C(F)(F)C(F)(F)C(F)(F)C(F)(F)C(F)(F)F		-9.83	0.67	3.51	P	OUT
QL00670	perfluoroalkylamido betaine-related, n=12		C(NCCC[N+](CC(O)=O)(C)C)(=O)C(F)(F)C(F)(F)C(F)(F)C(F)(F)C(F)(F)C(F)(F)C(F)(F)C(F)(F)C(F)(F)C(F)(F)C(F)(F)C(F)(F)F		-9.59	0.67	3.41	P	OUT
QL00671	perfluoroalkylamido betaine-related, n=11_pos		C(NCCC[N+](CC(O)=O)(C)C)(=O)C(F)(F)C(F)(F)C(F)(F)C(F)(F)C(F)(F)C(F)(F)C(F)(F)C(F)(F)C(F)(F)C(F)(F)C(F)(F)F		-9.48	0.67	3.38	P	OUT
QL00672	perfluoroalkylamido betaine-related, n=10		C(NCCC[N+](CC(O)=O)(C)C)(=O)C(F)(F)C(F)(F)C(F)(F)C(F)(F)C(F)(F)C(F)(F)C(F)(F)C(F)(F)C(F)(F)C(F)(F)F		-9.20	0.66	3.27	P	OUT
QL00673	perfluoroalkylamido betaine-related, n=9_pos		C(NCCC[N+](CC(O)=O)(C)C)(=O)C(F)(F)C(F)(F)C(F)(F)C(F)(F)C(F)(F)C(F)(F)C(F)(F)C(F)(F)C(F)(F)F		-9.07	0.66	3.24	P	OUT
QL00674	perfluoroalkylamido betaine-related, n=8		C(NCCC[N+](CC(O)=O)(C)C)(=O)C(F)(F)C(F)(F)C(F)(F)C(F)(F)C(F)(F)C(F)(F)C(F)(F)C(F)(F)F		-8.75	0.66	3.12	P	IN
QL00675	perfluoroalkylamido betaine-related, n=7		C(NCCC[N+](CC(O)=O)(C)C)(=O)C(F)(F)C(F)(F)C(F)(F)C(F)(F)C(F)(F)C(F)(F)C(F)(F)F		-8.55	0.65	3.08	P	IN
QL00676	perfluoroalkylamido betaine-related, n=6		C(NCCC[N+](CC(O)=O)(C)C)(=O)C(F)(F)C(F)(F)C(F)(F)C(F)(F)C(F)(F)C(F)(F)F		-8.17	0.65	2.94	P	IN
QL00677	Perfluoroalkylamido betaine-related_i, n=12		C(NCCC[N+](CC(OC(C)C)=O)(C)C)(=O)C(F)(F)C(F)(F)C(F)(F)C(F)(F)C(F)(F)C(F)(F)C(F)(F)C(F)(F)C(F)(F)C(F)(F)C(F)(F)C(F)(F)F		-9.71	0.66	3.54	P	OUT
QL00678	Perfluoroalkylamido betaine-related_i, n=10		C(NCCC[N+](CC(OC(C)C)=O)(C)C)(=O)C(F)(F)C(F)(F)C(F)(F)C(F)(F)C(F)(F)C(F)(F)C(F)(F)C(F)(F)C(F)(F)C(F)(F)F		-9.34	0.66	3.42	P	OUT
QL00679	Perfluoroalkylamido betaine-related_i, n=8		C(NCCC[N+](CC(OC(C)C)=O)(C)C)(=O)C(F)(F)C(F)(F)C(F)(F)C(F)(F)C(F)(F)C(F)(F)C(F)(F)C(F)(F)F		-8.88	0.65	3.27	P	OUT
QL00680	(2-Carboxyethyl)-3-(((perfluoro-7-methyloctyl)2-hydroxypropyl)amino)propyldimethylammonium hydroxide	73353-25-0	C(NCCC[N+](CCC(O)=O)(C)C)C(O)CC(F)(F)C(F)(F)C(F)(F)C(F)(F)C(F)(F)C(F)(F)C(F)(C(F)(F)F)C(F)(F)F.[OH-]		-8.65	0.63	3.40	P	OUT
QL00681	perfluoroalkylamido ammonium, n=14		C(NCCCN(C)C)(=O)C(F)(F)C(F)(F)C(F)(F)C(F)(F)C(F)(F)C(F)(F)C(F)(F)C(F)(F)C(F)(F)C(F)(F)C(F)(F)C(F)(F)C(F)(F)C(F)(F)F		-9.72	0.67	3.41	P	OUT
QL00682	perfluoroalkylamido ammonium, n=12		C(NCCCN(C)C)(=O)C(F)(F)C(F)(F)C(F)(F)C(F)(F)C(F)(F)C(F)(F)C(F)(F)C(F)(F)C(F)(F)C(F)(F)C(F)(F)C(F)(F)F		-9.35	0.67	3.27	P	OUT
QL00683	perfluoroalkylamido ammonium, n=11		C(NCCCN(C)C)(=O)C(F)(F)C(F)(F)C(F)(F)C(F)(F)C(F)(F)C(F)(F)C(F)(F)C(F)(F)C(F)(F)C(F)(F)C(F)(F)F		-9.22	0.67	3.23	P	OUT
QL00684	perfluoroalkylamido ammonium, n=10		C(NCCCN(C)C)(=O)C(F)(F)C(F)(F)C(F)(F)C(F)(F)C(F)(F)C(F)(F)C(F)(F)C(F)(F)C(F)(F)C(F)(F)F		-8.92	0.66	3.12	P	OUT
QL00685	perfluoroalkylamido ammonium, n=9		C(NCCCN(C)C)(=O)C(F)(F)C(F)(F)C(F)(F)C(F)(F)C(F)(F)C(F)(F)C(F)(F)C(F)(F)C(F)(F)F		-8.77	0.66	3.08	P	IN
QL00686	perfluoroalkylamido ammonium, n=8		C(NCCCN(C)C)(=O)C(F)(F)C(F)(F)C(F)(F)C(F)(F)C(F)(F)C(F)(F)C(F)(F)C(F)(F)F		-8.42	0.66	2.95	P	IN
QL00687	perfluoroalkylamido ammonium, n=7		C(NCCCN(C)C)(=O)C(F)(F)C(F)(F)C(F)(F)C(F)(F)C(F)(F)C(F)(F)C(F)(F)F		-8.24	0.65	2.91	P	IN
QL00688	perfluoroalkylamido ammonium, n=6		C(NCCCN(C)C)(=O)C(F)(F)C(F)(F)C(F)(F)C(F)(F)C(F)(F)C(F)(F)F		-7.81	0.65	2.76	P	IN
QL00689	perfluoroalkylamido ammonium, n=5		C(NCCCN(C)C)(=O)C(F)(F)C(F)(F)C(F)(F)C(F)(F)C(F)(F)F		-7.53	0.64	2.71	P	IN
QL00690	perfluoroalkylamido ammonium, n=4		C(NCCCN(C)C)(=O)C(F)(F)C(F)(F)C(F)(F)C(F)(F)F		-6.97	0.63	2.53	P	OUT
QL00691	perfluoroalkylamido ammonium, n=3		C(NCCCN(C)C)(=O)C(F)(F)C(F)(F)C(F)(F)F		-6.50	0.62	2.44	P	OUT
QL00692	1-((3-(Dimethylamino)propyl)amino)-3-(perfluoro-7-methyloctyl)propan-2-ol	73353-26-1	C(NCCCN(C)C)C(O)CC(F)(F)C(F)(F)C(F)(F)C(F)(F)C(F)(F)C(F)(F)C(F)(C(F)(F)F)C(F)(F)F		-8.50	0.65	3.09	P	IN

QL00693	1-((3-(Dimethylamino)propyl)amino)-(3-(perfluoro-11-methyldodecyl))propan-2-ol	94159-82-7	C(NCCCN(C)C)C(O)CC(F)(F)C(F)(F)C(F)(F)C(F)(F)C(F)(F)C(F)(F)C(F)(F)C(F)(F)C(F)(F)C(F)(F)C(F)(C(F)(F)F)C(F)(F)F		-9.40	0.66	3.39	P	OUT
QL00694	1-((3-(Dimethylamino)propyl)amino)-(3-(perfluorododecyl))propan-2-ol	94159-79-2	C(NCCCN(C)C)C(O)CC(F)(F)C(F)(F)C(F)(F)C(F)(F)C(F)(F)C(F)(F)C(F)(F)C(F)(F)C(F)(F)C(F)(F)C(F)(F)C(F)(F)F		-9.43	0.66	3.41	P	OUT
QL00695	1-((3-(Dimethylamino)propyl)amino)-(3-(perfluorodecyl))propan-2-ol	94159-80-5	C(NCCCN(C)C)C(O)CC(F)(F)C(F)(F)C(F)(F)C(F)(F)C(F)(F)C(F)(F)C(F)(F)C(F)(F)C(F)(F)C(F)(F)F		-9.02	0.66	3.28	P	OUT
QL00696	H-substituted Perfluoroalkyl (linear) carboxylic acids_i, n=15		C(O)(=O)C(F)C(F)(F)C(F)(F)C(F)(F)C(F)(F)C(F)(F)C(F)(F)C(F)(F)C(F)(F)C(F)(F)C(F)(F)C(F)(F)C(F)(F)C(F)(F)C(F)(F)C(F)(F)F		-9.80	0.69	3.24	P	OUT
QL00697	H-substituted Perfluoroalkyl (linear) carboxylic acids_i, n=14		C(O)(=O)C(F)C(F)(F)C(F)(F)C(F)(F)C(F)(F)C(F)(F)C(F)(F)C(F)(F)C(F)(F)C(F)(F)C(F)(F)C(F)(F)C(F)(F)C(F)(F)C(F)(F)F		-9.56	0.69	3.12	P	OUT
QL00698	H-substituted Perfluoroalkyl (linear) carboxylic acids_i, n=13		C(O)(=O)C(F)C(F)(F)C(F)(F)C(F)(F)C(F)(F)C(F)(F)C(F)(F)C(F)(F)C(F)(F)C(F)(F)C(F)(F)C(F)(F)C(F)(F)C(F)(F)F		-9.44	0.69	3.07	P	OUT
QL00699	H-substituted Perfluoroalkyl (linear) carboxylic acids_i, n=12		C(O)(=O)C(F)C(F)(F)C(F)(F)C(F)(F)C(F)(F)C(F)(F)C(F)(F)C(F)(F)C(F)(F)C(F)(F)C(F)(F)C(F)(F)C(F)(F)F		-9.16	0.68	2.95	P	OUT
QL00700	H-substituted Perfluoroalkyl (linear) carboxylic acids_i, n=11		C(O)(=O)C(F)C(F)(F)C(F)(F)C(F)(F)C(F)(F)C(F)(F)C(F)(F)C(F)(F)C(F)(F)C(F)(F)C(F)(F)C(F)(F)F		-9.03	0.68	2.89	P	OUT
QL00701	H-substituted Perfluoroalkyl (linear) carboxylic acids_i, n=10		C(O)(=O)C(F)C(F)(F)C(F)(F)C(F)(F)C(F)(F)C(F)(F)C(F)(F)C(F)(F)C(F)(F)C(F)(F)C(F)(F)F		-8.70	0.68	2.75	P	IN
QL00702	H-substituted Perfluoroalkyl (linear) carboxylic acids_i, n=9		C(O)(=O)C(F)C(F)(F)C(F)(F)C(F)(F)C(F)(F)C(F)(F)C(F)(F)C(F)(F)C(F)(F)C(F)(F)F		-8.54	0.68	2.69	P	IN
QL00703	H-substituted Perfluoroalkyl (linear) carboxylic acids_i, n=8		C(O)(=O)C(F)C(F)(F)C(F)(F)C(F)(F)C(F)(F)C(F)(F)C(F)(F)C(F)(F)C(F)(F)F		-8.15	0.68	2.52	P	IN
QL00704	H-substituted Perfluoroalkyl (linear) carboxylic acids_i, n=7		C(O)(=O)C(F)C(F)(F)C(F)(F)C(F)(F)C(F)(F)C(F)(F)C(F)(F)C(F)(F)F		-7.96	0.68	2.45	P	IN
QL00705	H-substituted Perfluoroalkyl (linear) carboxylic acids_i, n=6		C(O)(=O)C(F)C(F)(F)C(F)(F)C(F)(F)C(F)(F)C(F)(F)C(F)(F)F		-7.47	0.67	2.24	P	IN
QL00706	H-substituted Perfluoroalkyl (linear) carboxylic acids_i, n=5		C(O)(=O)C(F)C(F)(F)C(F)(F)C(F)(F)C(F)(F)C(F)(F)F		-7.22	0.67	2.17	P	IN
QL00707	H-substituted Perfluoroalkyl (linear) carboxylic acids_i, n=4		C(O)(=O)C(F)C(F)(F)C(F)(F)C(F)(F)C(F)(F)F		-6.57	0.66	1.91	P	IN
QL00708	H-substituted Perfluoroalkyl (linear) carboxylic acids_i, n=3		C(O)(=O)C(F)C(F)(F)C(F)(F)C(F)(F)F		-6.21	0.66	1.83	P	IN
QL00709	H-substituted Perfluoroalkyl (linear) carboxylic acids_i, n=2		C(O)(=O)C(F)C(F)(F)C(F)(F)F		-5.25	0.65	1.48	P	IN
QL00710	4,4,5,5,6,6,7,7,8,8,9,9,10,11,11,11-Hexadecafluoro-10-(trifluoromethyl)undecane-1,2-diol	67824-44-6	C(O)C(O)CC(F)(F)C(F)(F)C(F)(F)C(F)(F)C(F)(F)C(F)(F)C(F)(C(F)(F)F)C(F)(F)F		-8.20	0.67	2.67	P	IN
QL00711	Heptadecafluoro-N,N-bis(2-hydroxyethyl)octanesulphonamide	40630-61-3	C(O)CN([S](=O)(=O)C(F)(F)C(F)(F)C(F)(F)C(F)(F)C(F)(F)C(F)(F)C(F)(F)C(F)(F)F)CCO		-7.96	0.66	2.75	P	IN
QL00712	Tridecafluoro-N-(2-hydroxyethyl)-N-propylhexanesulphonamide	85665-64-1	C(O)CN([S](=O)(=O)C(F)(F)C(F)(F)C(F)(F)C(F)(F)C(F)(F)C(F)(F)F)CCC		-7.25	0.65	2.52	P	IN
QL00713	1,1,2,2,3,3,4,4-Octafluoro-N-(2-hydroxyethyl)-N-methylbutane-1-sulphonamide	93894-53-2	C(O)CN([S](=O)(=O)C(F)(F)C(F)(F)C(F)(F)C(F)F)C		-6.55	0.64	2.23	P	IN
QL00714	1,1,2,2,3,3,4,4-Octafluoro-N,N-bis(2-hydroxyethyl)butane-1-sulphonamide	93894-54-3	C(O)CN([S](=O)(=O)C(F)(F)C(F)(F)C(F)(F)C(F)F)CCO		-6.00	0.62	2.24	P	OUT
QL00715	122 Fluorotelomer dihydrogen phosphate	57678-07-6	C(O[P](=O)(O)O)CC(C(C(C(C(C(C(C(C(C(F)(F)C(F)(F)F)(F)F)(F)F)(F)F)(F)F)(F)F)(F)F)(F)F)(F)F)(F)F)(F)F		-9.37	0.68	3.11	P	OUT
QL00716	102 Fluorotelomer dihydrogen phosphate	57678-05-4	C(O[P](=O)(O)O)CC(C(C(C(C(C(C(C(C(F)(F)F)(F)F)(F)F)(F)F)(F)F)(F)F)(F)F)(F)F)(F)F)(F)F		-8.95	0.68	2.93	P	OUT
QL00717	Heptadecafluoro-N-(2-(phosphonooxy)ethyl)-N-propyloctanesulphonamide	64264-44-4	C(O[P](=O)(O)O)CN([S](=O)(=O)C(F)(F)C(F)(F)C(F)(F)C(F)(F)C(F)(F)C(F)(F)C(F)(F)C(F)(F)F)CCC		-8.45	0.66	2.94	P	IN
QL00718	Bis((perfluoro-15-methylhexadecyl)ethyl) hydrogen phosphate	93776-19-3	C(O[P](OCCC(F)(F)C(F)(F)C(F)(F)C(F)(F)C(F)(F)C(F)(F)C(F)(F)C(F)(F)C(F)(F)C(F)(F)C(F)(F)C(F)(F)C(F)(F)C(F)(F)C(F)(C(F)(F)F)C(F)(F)F)(=O)O)CC(F)(F)C(F)(F)C(F)(F)C(F)(F)C(F)(F)C(F)(F)C(F)(F)C(F)(F)C(F)(F)C(F)(F)C(F)(F)C(F)(F)C(F)(F)C(F)(F)C(F)(C(F)(F)F)C(F)(F)F		-11.81	0.69	4.26	P	OUT
QL00719	Bis((perfluorohexadecyl)ethyl) hydrogen phosphate	93857-54-6	C(O[P](OCCC(F)(F)C(F)(F)C(F)(F)C(F)(F)C(F)(F)C(F)(F)C(F)(F)C(F)(F)C(F)(F)C(F)(F)C(F)(F)C(F)(F)C(F)(F)C(F)(F)C(F)(F)C(F)(F)F)(=O)O)CC(F)(F)C(F)(F)C(F)(F)C(F)(F)C(F)(F)C(F)(F)C(F)(F)C(F)(F)C(F)(F)C(F)(F)C(F)(F)C(F)(F)C(F)(F)C(F)(F)C(F)(F)C(F)(F)F		-11.90	0.69	4.29	P	OUT
QL00720	1,H,1H,2H,2H-perfluoroeicosyl methacrylate	65104-66-7	C(OC(C(=C)C)=O)CC(C(C(C(C(C(C(C(C(C(C(C(C(C(C(C(C(F)(F)F)(F)F)(F)F)(F)F)(F)F)(F)F)(F)F)(F)F)(F)F)(F)F)(F)F)(F)F)(F)F)(F)F)(F)F)(F)F)(F)F)(F)F		-10.40	0.68	3.62	P	OUT
QL00721	(Perfluorohexadecyl)ethyl 2-methyl-2-propenoate	59778-97-1	C(OC(C(=C)C)=O)CC(C(C(C(C(C(C(C(C(C(C(C(C(C(C(F)(F)F)(F)F)(F)F)(F)F)(F)F)(F)F)(F)F)(F)F)(F)F)(F)F)(F)F)(F)F)(F)F)(F)F)(F)F)(F)F		-10.12	0.68	3.50	P	OUT

QL00722	Diethyl perfluoroadipate	376-50-1	C(OC(C(C(C(C(C(OCC)=O)(F)F)(F)F)(F)F)(F)F)=O)C		-6.35	0.62	2.41	P	OUT
QL00723	2-(((Heptadecafluorooctyl)sulphonyl)propylamino)ethyl methacrylate	13285-40-0	C(OC(C(C)=C)=O)CN(CCC)[S](=O)(=O)C(F)(F)C(F)(F)C(F)(F)C(F)(F)C(F)(F)C(F)(F)C(C(F)(F)F)(F)F		-8.54	0.65	3.08	P	IN
QL00724	(Perfluorooctadecyl)ethyl 2-propenoate	65104-64-5	C(OC(C=C)=O)CC(C(C(C(C(C(C(C(C(C(C(C(C(C(C(C(C(C(F)(F)F)(F)F)(F)F)(F)F)(F)F)(F)F)(F)F)(F)F)(F)F)(F)F)(F)F)(F)F)(F)F)(F)F)(F)F)(F)F)(F)F)(F)F		-10.33	0.68	3.62	P	OUT
QL00725	1,1,2,2-Tetrahydroperfluorooctadecyl acrylate	65150-93-8	C(OC(C=C)=O)CC(C(C(C(C(C(C(C(C(C(C(C(C(C(C(C(F)(F)F)(F)F)(F)F)(F)F)(F)F)(F)F)(F)F)(F)F)(F)F)(F)F)(F)F)(F)F)(F)F)(F)F)(F)F)(F)F		-10.04	0.68	3.50	P	OUT
QL00726	2-(Perfluoro-11-methyldodecyl)ethyl propenoate	52956-82-8	C(OC(C=C)=O)CC(C(C(C(C(C(C(C(C(C(C(C(F)(F)F)(F)C(F)(F)F)(F)F)(F)F)(F)F)(F)F)(F)F)(F)F)(F)F)(F)F)(F)F)(F)F		-9.32	0.67	3.22	P	OUT
QL00727	2-[Methyl[(perfluorohexadecyl)methylsulfonyl]amino]ethyl 2-propenoate	68758-56-5	C(OC(C=C)=O)CN([S](=O)(=O)CC(C(C(C(C(C(C(C(C(C(C(C(C(C(C(C(F)(F)F)(F)F)(F)F)(F)F)(F)F)(F)F)(F)F)(F)F)(F)F)(F)F)(F)F)(F)F)(F)F)(F)F)(F)F)(F)F)C		-10.24	0.67	3.65	P	OUT
QL00728	2-(N-(1,1-dihydroperfluoropentadecylsulfonyl)-N-methylamino)ethyl acrylate	68758-55-4	C(OC(C=C)=O)CN([S](=O)(=O)CC(C(C(C(C(C(C(C(C(C(C(C(C(F)(F)F)(F)F)(F)F)(F)F)(F)F)(F)F)(F)F)(F)F)(F)F)(F)F)(F)F)(F)F)(F)F)(F)F)C		-9.93	0.67	3.53	P	OUT
QL00729	2-[Methyl[(perfluorohexadecyl)ethylsulfonyl]amino]ethyl 2-propenoate	72276-08-5	C(OC(C=C)=O)CN([S](=O)(=O)CCC(C(C(C(C(C(C(C(C(C(C(C(C(C(C(C(F)(F)F)(F)F)(F)F)(F)F)(F)F)(F)F)(F)F)(F)F)(F)F)(F)F)(F)F)(F)F)(F)F)(F)F)(F)F)(F)F)C		-10.36	0.67	3.74	P	OUT
QL00730	2-[Methyl[(perfluorotetradecyl)ethylsulfonyl]amino]ethyl 2-propenoate	72276-07-4	C(OC(C=C)=O)CN([S](=O)(=O)CCC(C(C(C(C(C(C(C(C(C(C(C(C(F)(F)F)(F)F)(F)F)(F)F)(F)F)(F)F)(F)F)(F)F)(F)F)(F)F)(F)F)(F)F)(F)F)(F)F)C		-10.07	0.67	3.63	P	OUT
QL00731	Ethyl perfluorobutyl ether	163702-05-4	C(OC(F)(F)C(F)(F)C(F)(F)C(F)(F)F)C		-6.13	0.65	1.91	P	IN
QL00732	1,1,2,2,3,3,4,4,5,5,6,6,7,7,8,8,9,9,10,10-Icosafluoro-11-(vinyloxy)undecane	94231-58-0	C(OC=C)C(F)(F)C(F)(F)C(F)(F)C(F)(F)C(F)(F)C(F)(F)C(F)(F)C(F)(F)C(F)(F)C(F)F		-8.46	0.66	2.87	P	IN
QL00733	1,1,2,2,3,3,4,4,5,5,6,6,7,7,8,8-Hexadecafluoro-9-(vinyloxy)nonane	71726-31-3	C(OC=C)C(F)(F)C(F)(F)C(F)(F)C(F)(F)C(F)(F)C(F)(F)C(F)(F)C(F)F		-7.86	0.66	2.67	P	IN
QL00734	1,1,2,2,3,3,4,4,5,5,6,6-Dodecafluoro-7-(vinyloxy)heptane	78971-81-0	C(OC=C)C(F)(F)C(F)(F)C(F)(F)C(F)(F)C(F)(F)C(F)F		-7.09	0.64	2.45	P	IN
QL00735	1,1,2,2,3,3,4,4-Octafluoro-5-(vinyloxy)pentane	66396-73-4	C(OC=C)C(F)(F)C(F)(F)C(F)(F)C(F)F		-6.04	0.62	2.20	P	OUT
QL00736	1-(Perfluoro-7-methyloctyl)-4,7,10-trioxatetradecan-2-ol	94158-62-0	C(OCCOCCOCCCC)C(O)CC(F)(F)C(F)(F)C(F)(F)C(F)(F)C(F)(F)C(F)(F)C(F)(C(F)(F)F)C(F)(F)F		-8.62	0.63	3.46	P	OUT
QL00737	17-Oxoestra-1,3,5(10)-trien-3-yl heptafluorobutanoate	2192-60-1	C[C@]12CC[C@H]3[C@@H](CCc4cc(OC(=O)C(F)(F)C(F)(F)C(F)(F)F)ccc34)[C@@H]1CCC2=O		-7.91	0.64	2.96	P	OUT
QL00738	Fulvestrant sulfone	98008-06-1	C[C@]12CC[C@H]3[C@H]([C@@H]1CC[C@@H]2O)[C@H](CCCCCCCCCS(=O)(=O)CCCC(F)(F)C(F)(F)F)CC1=C3C=CC(O)=C1		-7.57	0.61	3.24	P	OUT
QL00739	S-Deoxo fulvestrant	153004-31-0	C[C@]12CC[C@H]3[C@H]([C@@H]1CC[C@@H]2O)[C@H](CCCCCCCCCSCCCC(F)(F)C(F)(F)F)CC1=C3C=CC(O)=C1		-7.20	0.59	3.21	P	OUT
QL00740	N-(2,2,3,3,4,4,5,5,6,6,7,7,8,8,9,9,9-Heptadecafluorononanoyl)-L-alanine	330548-64-6	C[C@H](NC(=O)C(F)(F)C(F)(F)C(F)(F)C(F)(F)C(F)(F)C(F)(F)C(F)(F)C(F)(F)F)C(O)=O		-8.39	0.67	2.75	P	IN
QL00741	(2S)-3,3,4,4,5,5,5-Heptafluoropentan-2-ol	1313024-90-6	C[C@H](O)C(F)(F)C(F)(F)C(F)(F)F		-5.76	0.67	1.46	P	IN
QL00742	Sulfonamides, C4-8-alkane, perfluoro, N-[3-(dimethylamino)propyl], reaction products with acrylic acid	192662-29-6	C[N](C)(CCCNS(=O)(=O)C(F)(F)C(F)(F)C(F)(F)C(F)(F)F)CCC(O)=O		-7.80	0.64	2.84	P	OUT
QL00743	3-(((Perfluorooctyl)sulfonyl)(3-sulfopropyl)amino)-N-(2-hydroxyethyl)-N,N-dimethylpropanaminium	68298-11-3	C[N](C)(CCO)CCCN(CCCS(O)(=O)=O)S(=O)(=O)C(F)(F)C(F)(F)C(F)(F)C(F)(F)C(F)(F)C(F)(F)C(F)(F)C(F)(F)F		-8.55	0.65	3.08	P	IN
QL00744	Sulfonamides, C4-8-alkane, perfluoro, N-[3-(dimethyloxidoamino)propyl], potassium salts	179005-06-2	C[N](C)(O)CCCNS(=O)(=O)C(F)(F)C(F)(F)C(F)(F)C(F)(F)F		-7.56	0.66	2.52	P	IN
QL00745	N-Methylperfluorooctanesulfonamidoethyl acrylate	25268-77-3	C[N@@](CCOC(C=C)=O)S(C(C(C(C(C(F)(F)C(C(C(F)(F)F)(F)F)(F)F)(F)F)(F)F)(F)F)(F)F)(F)F)(=O)=O		-8.76	0.66	3.08	P	IN
QL00746	3-[2,2,3,3,4,4-Hexafluoro-4-(heptafluoropropoxy)butanamido]-N,N-dimethylpropan-1-amine N-oxide	87112-48-9	C[N+](C)([O-])CCCNC(=O)C(F)(F)C(F)(F)C(F)(F)OC(F)(F)C(F)(F)C(F)(F)F		-8.18	0.65	2.87	P	IN
QL00747	Perfluorooctanoate N,N,N-trimethylmethanaminium	32609-65-7	C[N+](C)(C)C.[O-]C(=O)C(F)(F)C(F)(F)C(F)(F)C(F)(F)C(F)(F)C(F)(F)C(F)(F)F		-8.12	0.67	2.64	P	IN
QL00748	n2 fluorotelomer thioamido hydroxy ammonium, n=4		C[N+](C)(C)CC(O)CS(=O)CCC(F)(F)C(F)(F)C(F)(F)C(F)(F)C(F)(F)C(F)(F)C(F)(F)C(F)(F)F		-8.81	0.66	3.08	P	OUT
QL00749	n2 fluorotelomer thioamido hydroxy ammonium, n=3		C[N+](C)(C)CC(O)CS(=O)CCC(F)(F)C(F)(F)C(F)(F)C(F)(F)C(F)(F)C(F)(F)F		-8.19	0.66	2.87	P	IN
QL00750	n2 fluorotelomer thio hydroxy ammonium, n=5		C[N+](C)(C)CC(O)CSCCC(F)(F)C(F)(F)C(F)(F)C(F)(F)C(F)(F)C(F)(F)C(F)(F)C(F)(F)C(F)(F)C(F)(F)F		-9.21	0.67	3.23	P	OUT
QL00751	n2 fluorotelomer thio hydroxy ammonium, n=4		C[N+](C)(C)CC(O)CSCCC(F)(F)C(F)(F)C(F)(F)C(F)(F)C(F)(F)C(F)(F)C(F)(F)C(F)(F)F		-8.76	0.66	3.08	P	IN

QL00752	n2 fluorotelomer thio hydroxy ammonium, n=3		C[N+](C)(C)CC(O)CSCCC(F)(F)C(F)(F)C(F)(F)C(F)(F)C(F)(F)C(F)(F)F		-8.13	0.65	2.87	P	IN
QL00753	n2 fluorotelomer thio hydroxy ammonium, n=2		C[N+](C)(C)CC(O)CSCCC(F)(F)C(F)(F)C(F)(F)C(F)(F)F		-7.31	0.64	2.62	P	IN
QL00754	1-Propanaminium, 3-[[4-[(heptadecafluorononyl)oxy]benzoyl]amino]-N,N,N-trimethyl- (9CI)	59493-71-9	C[N+](C)(C)CCCNC(=O)C1=CC=C(OC(F)(F)C(F)(F)C(F)(F)C(F)(F)C(F)(F)C(F)(F)C(F)(F)CC(F)(F)F)C=C1		-8.96	0.65	3.39	P	OUT
QL00755	Bis[trimethyl-3-[[(pentadecafluoroheptyl)sulphonyl]amino]propylammonium] sulphate	70225-20-6	C[N+](C)(C)CCCNS(=O)(=O)C(F)(F)C(F)(F)C(F)(F)C(F)(F)C(F)(F)C(F)(F)C(F)(F)F.C[N+](C)(C)CCCNS(=O)(=O)C(F)(F)C(F)(F)C(F)(F)C(F)(F)C(F)(F)C(F)(F)C(F)(F)C(F)(F)F.[O-]S(=O)(=O)[O-]		-8.90	0.66	3.11	P	OUT
QL00756	Bis[trimethyl-3-[[(tridecafluorohexyl)sulphonyl]amino]propylammonium] sulphate	70248-52-1	C[N+](C)(C)CCCNS(=O)(=O)C(F)(F)C(F)(F)C(F)(F)C(F)(F)C(F)(F)C(F)(F)F.C[N+](C)(C)CCCNS(=O)(=O)C(F)(F)C(F)(F)C(F)(F)C(F)(F)C(F)(F)C(F)(F)F.[O-]S(=O)(=O)[O-]		-8.55	0.66	2.98	P	IN
QL00757	Bis[trimethyl-3-[[(undecafluoropentyl)sulphonyl]amino]propylammonium] sulphate	70225-24-0	C[N+](C)(C)CCCNS(=O)(=O)C(F)(F)C(F)(F)C(F)(F)C(F)(F)C(F)(F)F.C[N+](C)(C)CCCNS(=O)(=O)C(F)(F)C(F)(F)C(F)(F)C(F)(F)C(F)(F)F.[O-]S(=O)(=O)[O-]		-8.39	0.66	2.95	P	IN
QL00758	Bis[trimethyl-3-[[(nonafluorobutyl)sulphonyl]amino]propylammonium] sulphate	70225-22-8	C[N+](C)(C)CCCNS(=O)(=O)C(F)(F)C(F)(F)C(F)(F)C(F)(F)F.C[N+](C)(C)CCCNS(=O)(=O)C(F)(F)C(F)(F)C(F)(F)C(F)(F)F.[O-]S(=O)(=O)[O-]		-7.96	0.65	2.80	P	IN
QL00759	Perfluorononane sulfonamido ammonium iodide	69091-20-9	C[N+](C)(C)CCCNS(=O)C(F)(F)C(F)(F)C(F)(F)C(F)(F)C(F)(F)C(F)(F)C(F)(F)C(F)(F)C(F)(F)F		-8.95	0.67	3.07	P	OUT
QL00760	73 Fluorotelomer betaine	171184-15-9	C[N+](C)(CCCC(F)(F)C(F)(F)C(F)(F)C(F)(F)C(F)(F)C(F)(F)C(F)(F)F)CC([O-])=O		-8.42	0.66	2.90	P	IN
QL00761	N-(2-Carboxyethyl)-3-[(perfluoro-1-oxononyl)amino]-N,N-dimethyl-1-propanaminium	119131-05-4	C[N+](C)(CCCN=C(/O)C(F)(F)C(F)(F)C(F)(F)C(F)(F)C(F)(F)C(F)(F)C(F)(F)C(F)(F)F)CCC(O)=O		-8.90	0.65	3.24	P	OUT
QL00762	Perfluorodecane amide betaine	70674-74-7	C[N+](C)(CCCNC(=O)C(F)(F)C(F)(F)C(F)(F)C(F)(F)C(F)(F)C(F)(F)C(F)(F)C(F)(F)C(F)(F)F)CC([O-])=O		-9.07	0.66	3.24	P	OUT
QL00763	N-(2-Carboxyethyl)-N,N-dimethyl-3-[(perfluoro-1-oxodecyl)amino]propanaminium	113933-98-5	C[N+](C)(CCCNC(=O)C(F)(F)C(F)(F)C(F)(F)C(F)(F)C(F)(F)C(F)(F)C(F)(F)C(F)(F)C(F)(F)F)CCC([O-])=O		-9.07	0.66	3.28	P	OUT
QL00764	Perfluorononane amide betaine	77958-18-0	C[N+](C)(CCCNC(=O)C(F)(F)C(F)(F)C(F)(F)C(F)(F)C(F)(F)C(F)(F)C(F)(F)C(F)(F)F)CC([O-])=O		-8.75	0.66	3.12	P	IN
QL00765	Perfluorooctaneamide betaine	90179-39-8	C[N+](C)(CCCNC(=O)C(F)(F)C(F)(F)C(F)(F)C(F)(F)C(F)(F)C(F)(F)C(F)(F)F)CC([O-])=O		-8.55	0.65	3.08	P	IN
QL00766	(2-Carboxyethyl)dimethyl[3-(perfluorooctanamido)propyl]ammonium	5158-52-1	C[N+](C)(CCCNC(=O)C(F)(F)C(F)(F)C(F)(F)C(F)(F)C(F)(F)C(F)(F)C(F)(F)F)CCC(O)=O		-8.56	0.65	3.12	P	IN
QL00767	Perfluoroheptane amide betaine	130114-31-7	C[N+](C)(CCCNC(=O)C(F)(F)C(F)(F)C(F)(F)C(F)(F)C(F)(F)C(F)(F)F)CC([O-])=O		-8.17	0.65	2.94	P	IN
QL00768	(2-Carboxyethyl)-3-((perfluorooctyl)-2-hydroxypropyl)aminopropyldimethylammonium hydroxide	93776-14-8	C[N+](C)(CCCNCC(O)CC(F)(F)C(F)(F)C(F)(F)C(F)(F)C(F)(F)C(F)(F)C(F)(F)C(F)(F)F)CCC([O-])=O		-8.92	0.65	3.36	P	OUT
QL00769	Perfluorooctanesulfonamido betaine	75046-16-1	C[N+](C)(CCCNS(=O)(=O)C(F)(F)C(F)(F)C(F)(F)C(F)(F)C(F)(F)C(F)(F)C(F)(F)C(F)(F)F)CC([O-])=O		-8.89	0.66	3.12	P	OUT
QL00770	102 Fluorotelomer sulfonamide betaine	34455-35-1	C[N+](C)(CCCNS(=O)(=O)CCC(F)(F)C(F)(F)C(F)(F)C(F)(F)C(F)(F)C(F)(F)C(F)(F)C(F)(F)C(F)(F)C(F)(F)F)CC([O-])=O		-9.45	0.66	3.41	P	OUT
QL00771	82 Fuorotelomer sulfonamide betaine	34455-21-5	C[N+](C)(CCCNS(=O)(=O)CCC(F)(F)C(F)(F)C(F)(F)C(F)(F)C(F)(F)C(F)(F)C(F)(F)C(F)(F)F)CC([O-])=O		-8.96	0.66	3.24	P	OUT
QL00772	n2 fluorotelomer betaine, n=5		C[N+](CC(O)=O)(C)CCC(F)(F)C(F)(F)C(F)(F)C(F)(F)C(F)(F)C(F)(F)C(F)(F)C(F)(F)C(F)(F)C(F)(F)F		-9.10	0.67	3.07	P	OUT
QL00773	n2 fluorotelomer betaine, n=4		C[N+](CC(O)=O)(C)CCC(F)(F)C(F)(F)C(F)(F)C(F)(F)C(F)(F)C(F)(F)C(F)(F)C(F)(F)F		-8.62	0.67	2.89	P	IN
QL00774	n2 fluorotelomer betaine, n=3		C[N+](CC(O)=O)(C)CCC(F)(F)C(F)(F)C(F)(F)C(F)(F)C(F)(F)C(F)(F)F		-8.05	0.66	2.70	P	IN
QL00775	n12 fluorotelomer betaine-related, n=7		C[N+](CC(O)=O)(C)CCC(F)C(F)(F)C(F)(F)C(F)(F)C(F)(F)C(F)(F)C(F)(F)C(F)(F)C(F)(F)C(F)(F)C(F)(F)C(F)(F)C(F)(F)C(F)(F)C(F)(F)C(F)(F)F		-10.08	0.68	3.50	P	OUT
QL00776	n12 fluorotelomer betaine-related, n=6		C[N+](CC(O)=O)(C)CCC(F)C(F)(F)C(F)(F)C(F)(F)C(F)(F)C(F)(F)C(F)(F)C(F)(F)C(F)(F)C(F)(F)C(F)(F)C(F)(F)C(F)(F)C(F)(F)F		-9.75	0.68	3.37	P	OUT
QL00777	n12 fluorotelomer betaine-related, n=5		C[N+](CC(O)=O)(C)CCC(F)C(F)(F)C(F)(F)C(F)(F)C(F)(F)C(F)(F)C(F)(F)C(F)(F)C(F)(F)C(F)(F)C(F)(F)C(F)(F)F		-9.39	0.67	3.23	P	OUT
QL00778	n12 fluorotelomer betaine-related, n=4		C[N+](CC(O)=O)(C)CCC(F)C(F)(F)C(F)(F)C(F)(F)C(F)(F)C(F)(F)C(F)(F)C(F)(F)C(F)(F)C(F)(F)F		-8.97	0.67	3.07	P	OUT
QL00779	n12 fluorotelomer betaine-related, n=3		C[N+](CC(O)=O)(C)CCC(F)C(F)(F)C(F)(F)C(F)(F)C(F)(F)C(F)(F)C(F)(F)C(F)(F)F		-8.47	0.66	2.90	P	IN
QL00780	n12 fluorotelomer betaine-related, n=2		C[N+](CC(O)=O)(C)CCC(F)C(F)(F)C(F)(F)C(F)(F)C(F)(F)C(F)(F)F		-7.82	0.65	2.69	P	IN
QL00781	n3 fluorotelomer betaine-related, n=6		C[N+](CC(O)=O)(C)CCCC(F)(F)C(F)(F)C(F)(F)C(F)(F)C(F)(F)C(F)(F)C(F)(F)C(F)(F)C(F)(F)C(F)(F)C(F)(F)C(F)(F)C(F)(F)F		-9.72	0.67	3.37	P	OUT

QL00782	n3 fluorotelomer betaine-related, n=5		C[N+](CC(O)=O)(C)CCCC(F)(F)C(F)(F)C(F)(F)C(F)(F)C(F)(F)C(F)(F)C(F)(F)C(F)(F)C(F)(F)C(F)(F)C(F)(F)F		-9.35	0.67	3.23	P	OUT
QL00783	n3 fluorotelomer betaine-related, n=4		C[N+](CC(O)=O)(C)CCCC(F)(F)C(F)(F)C(F)(F)C(F)(F)C(F)(F)C(F)(F)C(F)(F)C(F)(F)C(F)(F)F		-8.92	0.67	3.07	P	OUT
QL00784	n3 fluorotelomer betaine-related, n=3		C[N+](CC(O)=O)(C)CCCC(F)(F)C(F)(F)C(F)(F)C(F)(F)C(F)(F)C(F)(F)C(F)(F)F		-8.42	0.66	2.90	P	IN
QL00785	n3 fluorotelomer betaine-related, n=2		C[N+](CC(O)=O)(C)CCCC(F)(F)C(F)(F)C(F)(F)C(F)(F)C(F)(F)F		-7.69	0.65	2.66	P	IN
QL00786	n4 fluorotelomer betaine-related, n=4		C[N+](CC(O)=O)(C)CCCCC(F)(F)C(F)(F)C(F)(F)C(F)(F)C(F)(F)C(F)(F)C(F)(F)C(F)(F)F		-8.74	0.66	3.08	P	IN
QL00787	n4 fluorotelomer betaine-related, n=3		C[N+](CC(O)=O)(C)CCCCC(F)(F)C(F)(F)C(F)(F)C(F)(F)C(F)(F)C(F)(F)F		-8.10	0.65	2.87	P	IN
QL00788	n4 fluorotelomer betaine-related, n=2		C[N+](CC(O)=O)(C)CCCCC(F)(F)C(F)(F)C(F)(F)C(F)(F)F		-7.28	0.64	2.62	P	IN
QL00789	(2-Carboxylatoethyl)-3-((perfluoro-11-methyldodecyl)-2-hydroxypropyl)aminopropyldimethylammonium	93776-15-9	C[N+](CCCNCC(O)CC(F)(F)C(F)(F)C(F)(F)C(F)(F)C(F)(F)C(F)(F)C(F)(F)C(F)(F)C(F)(F)C(F)(F)C(C(F)(F)F)(F)C(F)(F)F)(CCC(O)=O)C		-9.76	0.66	3.61	P	OUT
QL00790	Methanaminium perfluorononanoate	77032-23-6	C[NH3+].[O-]C(=O)C(F)(F)C(F)(F)C(F)(F)C(F)(F)C(F)(F)C(F)(F)C(F)(F)C(F)(F)F		-7.29	0.64	2.64	P	IN
QL00791	Methanaminium perfluoro-2-(2-propoxypropoxy)propanoate	328065-29-8	C[NH3+].[O-]C(=O)C(F)(OC(F)(F)C(F)(OC(F)(F)C(F)(F)C(F)(F)F)C(F)(F)F)C(F)(F)F		-7.00	0.63	2.59	P	OUT
QL00792	1-Butanesulfonic acid, 1,1,2,2,3,3,4,4,4-nonafluoro-, salt with sulfonium, dimethylphenyl- (1:1)	220133-51-7	C[S](C)C1=CC=CC=C1.[O-]S(=O)(=O)C(F)(F)C(F)(F)C(F)(F)C(F)(F)F		-5.99	0.64	1.94	P	IN
QL00793	Silane, (heptadecafluorooctyl)trimethyl-	52112-19-3	C[Si](C)(C)C(F)(F)C(F)(F)C(F)(F)C(F)(F)C(F)(F)C(F)(F)C(F)(F)C(F)(F)F		-8.37	0.69	2.44	P	IN
QL00794	Silane, trimethyl(tridecafluorohexyl)-	135841-49-5	C[Si](C)(C)C(F)(F)C(F)(F)C(F)(F)C(F)(F)C(F)(F)C(F)(F)F		-7.74	0.69	2.15	P	IN
QL00795	Trimethyl(nonafluorobutyl)silane	204316-01-8	C[Si](C)(C)C(F)(F)C(F)(F)C(F)(F)C(F)(F)F		-6.93	0.69	1.79	P	IN
QL00796	(Heptafluoropropyl)trimethylsilane	3834-42-2	C[Si](C)(C)C(F)(F)C(F)(F)C(F)(F)F		-6.18	0.68	1.45	P	IN
QL00797	(3,3,4,4,5,5,5-Heptafluoro-1-iodopentyl)(trimethyl)silane	89608-32-2	C[Si](C)(C)C(I)CC(F)(F)C(F)(F)C(F)(F)F		-6.60	0.67	1.90	P	IN
QL00798	1,1,1,3-Tetramethyl-3,3-bis(1,1,2,2,3,3,6,6,6-nonafluorohexyl)disiloxane	122179-35-5	C[Si](C)(C)O[Si](C)(C(F)(F)C(F)(F)C(F)(F)CCC(F)(F)F)C(F)(F)C(F)(F)C(F)(F)CCC(F)(F)F		-8.19	0.66	2.74	P	IN
QL00799	1,1,1,3,5,5,5-Heptamethyl-3-(3,3,4,4,5,5,6,6,7,7,8,8,8-tridecafluorooctyl)trisiloxane	136772-44-6	C[Si](C)(C)O[Si](C)(CCC(F)(F)C(F)(F)C(F)(F)C(F)(F)C(F)(F)C(F)(F)F)O[Si](C)(C)C		-8.04	0.67	2.63	P	IN
QL00800	Trimethylsilyloxy-1,1,1,5,5,5-hexamethyl-3-((perfluorohexyl)ethyl)trisiloxane	94237-07-7	C[Si](C)(C)O[Si](CCC(F)(F)C(F)(F)C(F)(F)C(F)(F)C(F)(F)C(F)(F)F)(O[Si](C)(C)C)O[Si](C)(C)C		-7.79	0.66	2.56	P	IN
QL00801	(2S)-2-{Bis[4-(heptadecafluorooctyl)phenyl][(trimethylsilyl)oxy]methyl}pyrrolidine	906077-31-4	C[Si](C)(C)OC([C@@H]1CCCN1)(c1ccc(cc1)C(F)(F)C(F)(F)C(F)(F)C(F)(F)C(F)(F)C(F)(F)C(F)(F)C(F)(F)F)c1ccc(cc1)C(F)(F)C(F)(F)C(F)(F)C(F)(F)C(F)(F)C(F)(F)C(F)(F)C(F)(F)F		-10.06	0.67	3.67	P	OUT
QL00802	Trimethylsilyl heptafluorobutanoate	24929-99-5	C[Si](C)(C)OC(=O)C(F)(F)C(F)(F)C(F)(F)F		-6.47	0.66	1.90	P	IN
QL00803	Trimethylsilyl 2,3,3,3-tetrafluoro-2-(heptafluoropropoxy)propanoate	152718-74-6	C[Si](C)(C)OC(=O)C(F)(OC(F)(F)C(F)(F)C(F)(F)F)C(F)(F)F		-7.14	0.66	2.19	P	IN
QL00804	Trimethylsilyl perfluorobutanesulfonate	68734-62-3	C[Si](C)(C)OS(=O)(=O)C(F)(F)C(F)(F)C(F)(F)C(F)(F)F		-7.35	0.68	2.15	P	IN
QL00805	Trimethylsilyl 1,1,2,2-tetrafluoro-2-(pentafluoroethoxy)ethane-1-sulfonate	136049-37-1	C[Si](C)(C)OS(=O)(=O)C(F)(F)C(F)(F)OC(F)(F)C(F)(F)F		-7.34	0.67	2.23	P	IN
QL00806	1,3-Bis(3,3,4,4,5,5,6,6,7,7,8,8,9,9,10,10,10-heptadecafluorodecyl)-1,1,3,3-tetramethyldisiloxane	129498-18-6	C[Si](C)(CCC(F)(F)C(F)(F)C(F)(F)C(F)(F)C(F)(F)C(F)(F)C(F)(F)C(F)(F)F)O[Si](C)(C)CCC(F)(F)C(F)(F)C(F)(F)C(F)(F)C(F)(F)C(F)(F)C(F)(F)C(F)(F)F		-10.27	0.68	3.54	P	OUT
QL00807	1,1,3,3-Tetramethyl-1,3-bis((perfluorohexyl)ethyl)disiloxane	71363-70-7	C[Si](C)(CCC(F)(F)C(F)(F)C(F)(F)C(F)(F)C(F)(F)C(F)(F)F)O[Si](C)(C)CCC(F)(F)C(F)(F)C(F)(F)C(F)(F)C(F)(F)C(F)(F)F		-9.63	0.68	3.28	P	OUT
QL00808	3-Chloro-1,1,3,5,5-pentamethyl-1,5-bis(3,3,4,4,5,5,6,6,7,7,8,8,8-tridecafluorooctyl)trisiloxane	521069-01-2	C[Si](C)(CCC(F)(F)C(F)(F)C(F)(F)C(F)(F)C(F)(F)C(F)(F)F)O[Si](C)(Cl)O[Si](C)(C)CCC(F)(F)C(F)(F)C(F)(F)C(F)(F)C(F)(F)C(F)(F)F		-9.84	0.68	3.42	P	OUT
QL00809	Tricyclo[7.3.3.15,11]heptasiloxane-3,7-diol, 1,3,5,7,9,11,14-heptacyclopentyl-14-[[dimethyl(3,3,4,4,5,5,6,6,7,7,8,8,8-tridecafluorooctyl)silyl]oxy]-	352538-84-2	C[Si](C)(CCC(F)(F)C(F)(F)C(F)(F)C(F)(F)C(F)(F)C(F)(F)F)O[Si]1(O[Si]2(O[Si](O)(O[Si]3(O[Si](O)(O[Si](O1)(O[Si](O3)(O2)C1CCCC1)C1CCCC1)C1CCCC1)C1CCCC1)C1CCCC1)C1CCCC1)C1CCCC1		-8.07	0.63	3.22	P	OUT
QL00810	2-(Perfluorooctyl)ethyldimethylchlorosilane	74612-30-9	C[Si](C)(Cl)CCC(F)(F)C(F)(F)C(F)(F)C(F)(F)C(F)(F)C(F)(F)C(F)(F)C(F)(F)F		-8.49	0.68	2.68	P	IN
QL00811	1H,1H,2H,2H-Perfluorooctyldimethylchlorosilane	102488-47-1	C[Si](C)(Cl)CCC(F)(F)C(F)(F)C(F)(F)C(F)(F)C(F)(F)C(F)(F)F		-7.90	0.67	2.44	P	IN
QL00812	Dimethyl(3,3,4,4,5,5,6,6,6-nonafluorohexyl)chlorosilane	119386-82-2	C[Si](C)(Cl)CCC(F)(F)C(F)(F)C(F)(F)C(F)(F)F		-7.14	0.67	2.17	P	IN
QL00813	1H,1H,2H,2H-Perfluorodecylmethyldichlorosilane	3102-79-2	C[Si](Cl)(Cl)CCC(F)(F)C(F)(F)C(F)(F)C(F)(F)C(F)(F)C(F)(F)C(F)(F)C(F)(F)F		-8.49	0.68	2.68	P	IN

QL00814	(Heptadecafluorooctyl)(dimethyl)silane	198283-43-1	C[SiH](C)C(F)(F)C(F)(F)C(F)(F)C(F)(F)C(F)(F)C(F)(F)C(F)(F)C(F)(F)F		-8.18	0.68	2.45	P	IN
QL00815	1,1,3,5,5-Pentamethyl-1,5-bis(3,3,4,4,5,5,6,6,7,7,8,8,8-tridecafluorooctyl)trisiloxane	521069-00-1	C[SiH](O[Si](C)(C)CCC(F)(F)C(F)(F)C(F)(F)C(F)(F)C(F)(F)C(F)(F)F)O[Si](C)(C)CCC(F)(F)C(F)(F)C(F)(F)C(F)(F)C(F)(F)C(F)(F)F		-9.73	0.67	3.41	P	OUT
QL00816	1,1,3,5,5-Pentamethyl-1,5-bis(3,3,4,4,5,5,6,6,6-nonafluorohexyl)trisiloxane	147986-73-0	C[SiH](O[Si](C)(C)CCC(F)(F)C(F)(F)C(F)(F)C(F)(F)F)O[Si](C)(C)CCC(F)(F)C(F)(F)C(F)(F)C(F)(F)F		-8.94	0.66	3.11	P	OUT
QL00817	(Perfluorododecyl)ethylene	67103-05-3	C=CC(C(C(C(C(C(C(C(C(C(C(C(F)(F)F)(F)F)(F)F)(F)F)(F)F)(F)F)(F)F)(F)F)(F)F)(F)F)(F)F)(F)F		-9.10	0.69	2.89	P	OUT
QL00818	Sodium 2-(N-ethylperfluorooctanesulfonamido)acetate	3871-50-9	C=O.C(N([S](=O)(=O)C(F)(F)C(F)(F)C(F)(F)C(F)(F)C(F)(F)C(F)(F)C(F)(F)C(F)(F)F)CC)C([O-])=O.C(N[S](=O)C(F)(F)C(F)(F)C(F)(F)C(F)(F)C(F)(F)C(F)(F)C(F)(F)C(F)(F)F)C([O-])=O.[K+].[K+].C(C)C		-7.57	0.62	3.09	P	OUT
QL00819	Benzene, 1-(1-(difluoro((1,2,2-trifluoroethenyl)oxy)methyl)-1,2,2,2-tetrafluoroethoxy)-2,3,4,5,6-pentafluoro-	24520-19-2	C1(=C(F)C(=C(C(=C1F)F)OC(F)(C(F)(F)OC(F)=C(F)F)C(F)(F)F)F)F		-6.91	0.64	2.46	P	IN
QL00820	Furan, 2,2,3,3,4,4-hexafluorotetrahydro-5-(2,2,2-trifluoro-1,1-bis(trifluoromethyl)ethyl)-	71302-72-2	C1(C(C(C(F)(O1)F)(F)F)(F)F)C(C(F)(F)F)(C(F)(F)F)C(F)(F)F		-6.35	0.68	1.56	P	IN
QL00821	N-((Perfluorooctyl)-1-ethyl)pyridinium 4-methylbenzenesulfonate	61798-68-3	C1=C([S]([O-])(=O)=O)C=CC(=C1)C.C2=[N+](CCC(F)(F)C(F)(F)C(F)(F)C(F)(F)C(F)(F)C(F)(F)C(F)(F)C(F)(F)F)C=CC=C2		-9.12	0.65	3.47	P	OUT
QL00822	2-((Perfluorooctyl)sulfonylaminomethyl)benzenesulfonate sodium	51032-47-4	C1=C(CN[S](=O)(=O)C(F)(F)C(F)(F)C(F)(F)C(F)(F)C(F)(F)C(F)(F)C(F)(F)C(F)(F)F)C(=CC=C1)[S]([O-])(=O)=O.[Na+]		-8.36	0.65	3.02	P	IN
QL00823	Sodium 2-((pentadecafluoroheptylsulphonylamino)methyl)benzenesulphonate	68299-29-6	C1=C(CN[S](=O)(=O)C(F)(F)C(F)(F)C(F)(F)C(F)(F)C(F)(F)C(F)(F)C(F)(F)F)C(=CC=C1)[S]([O-])(=O)=O.[Na+]		-8.17	0.65	2.98	P	IN
QL00824	Sodium 2-((tridecafluorohexylsulphonylamino)methyl)benzenesulphonate	68299-21-8	C1=C(CN[S](=O)(=O)C(F)(F)C(F)(F)C(F)(F)C(F)(F)C(F)(F)C(F)(F)F)C(=CC=C1)[S]([O-])(=O)=O.[Na+]		-7.77	0.64	2.84	P	OUT
QL00825	Sodium 2-((undecafluoropentylsulphonylamino)methyl)benzenesulphonate	68299-20-7	C1=C(CN[S](=O)(=O)C(F)(F)C(F)(F)C(F)(F)C(F)(F)C(F)(F)F)C(=CC=C1)[S]([O-])(=O)=O.[Na+]		-7.52	0.64	2.79	P	OUT
QL00826	Sodium 2-((nonafluorobutylsulfonylamino)methyl)benzenesulfonate	68299-19-4	C1=C(CN[S](=O)(=O)C(F)(F)C(F)(F)C(F)(F)C(F)(F)F)C(=CC=C1)[S]([O-])(=O)=O.[Na+]		-7.02	0.63	2.62	P	OUT
QL00827	Bis[2-[ethyl[(perfluorooctyl)sulfonyl]amino]ethyl] (4-methyl-1,3-phenylene)-bis(carbamate)	21055-88-9	C1=C(NC(OCCN([S](=O)(=O)C(F)(F)C(F)(F)C(F)(F)C(F)(F)C(F)(F)C(F)(F)C(F)(F)C(F)(F)F)CC)=O)C(=CC=C1NC(OCCN([S](=O)(=O)C(F)(F)C(F)(F)C(F)(F)C(F)(F)C(F)(F)C(F)(F)C(F)(F)C(F)(F)F)CC)=O)C		-10.46	0.66	4.02	P	OUT
QL00828	2,3,4,5-Tetrachloro-6-((3-(perfluorooctyl)sulfonyloxy)phenylaminocarbonyl)benzoic acid, potassium salt	57589-85-2	C1=C(O[S](=O)(=O)C(F)(F)C(F)(F)C(F)(F)C(F)(F)C(F)(F)C(F)(F)C(F)(F)C(F)(F)F)C=CC=C1NC(=O)C2=C(C(=C(Cl)C(=C2Cl)Cl)Cl)C([O-])=O.[K+]		-8.83	0.64	3.40	P	OUT
QL00829	Potassium 6-(((3-(perfluorobutylsulphonyloxy)phenyl)amino)carbonyl)perchlorobenzoate	68568-54-7	C1=C(O[S](=O)(=O)C(F)(F)C(F)(F)C(F)(F)C(F)(F)F)C=CC=C1NC(=O)C2=C(C(=C(Cl)C(=C2Cl)Cl)Cl)C([O-])=O.[K+]		-7.72	0.62	3.08	P	OUT
QL00830	Sodium 4-{perfluoro[4-ethyl-3,4-dimethylhex-2-en-2-yl]oxy}benzene-1-sulfonate	52584-45-9	C1=C(OC(=C(/C(C(F)(F)C(F)(F)F)(C(F)(F)C(F)(F)F)C(F)(F)F)C(F)(F)F)C(F)(F)F)C=CC(=C1)[S]([O-])(=O)=O.[K+]		-7.39	0.64	2.65	P	IN
QL00831	1-Phenoxy(3-(perfluoro-7-methyloctyl)propan-2-ol)	94159-92-9	C1=C(OCC(O)CC(F)(F)C(F)(F)C(F)(F)C(F)(F)C(F)(F)C(F)(F)C(F)(C(F)(F)F)C(F)(F)F)C=CC=C1		-8.54	0.65	3.07	P	IN
QL00832	N-Methyl-4-[[4,4,5,5,5-pentafluoro-3-(pentafluoroethyl)-1,2,3-tris(trifluoromethyl)-1-pentenyl]oxy]-N-[2-(phosphonooxy)ethyl]benzenesulfonamide	69013-34-9	C1=CC(=CC=C1[S](=O)(=O)N(CCO[P](=O)(O)O)C)OC(=C(C(F)(F)F)C(C(F)(F)F)(C(F)(F)C(F)(F)F)C(F)(F)C(F)(F)F)C(F)(F)F		-8.26	0.65	2.98	P	IN
QL00833	4-(Perfluoro(4-ethyl-3,4-dimethylhex-2-en-2-yl)oxy)benzene-1-sulfonyl chloride	51947-19-4	C1=CC(=CC=C1[S](Cl)(=O)=O)OC(C(F)(F)F)=C(C(F)(F)F)C(C(F)(F)F)(C(F)(F)C(F)(F)F)C(F)(F)C(F)(F)F		-7.63	0.66	2.55	P	IN
QL00834	1-Octanesulfonamide, 1,1,2,2,3,3,4,4,5,5,6,6,7,7,8,8,8-heptadecafluoro-N-(phenylmethyl)-	50598-29-3	C1=CC=C(C=C1)CN[S](=O)(=O)C(F)(F)C(F)(F)C(F)(F)C(F)(F)C(F)(F)C(F)(F)C(F)(F)C(F)(F)F		-8.54	0.66	2.94	P	IN
QL00835	1-Heptanesulfonamide, 1,1,2,2,3,3,4,4,5,5,6,6,7,7,7-pentadecafluoro-N-(phenylmethyl)-	68298-10-2	C1=CC=C(C=C1)CN[S](=O)(=O)C(F)(F)C(F)(F)C(F)(F)C(F)(F)C(F)(F)C(F)(F)C(F)(F)F		-8.36	0.66	2.90	P	IN
QL00836	1-Hexanesulfonamide, 1,1,2,2,3,3,4,4,5,5,6,6,6-tridecafluoro-N-(phenylmethyl)-	68298-09-9	C1=CC=C(C=C1)CN[S](=O)(=O)C(F)(F)C(F)(F)C(F)(F)C(F)(F)C(F)(F)C(F)(F)F		-7.95	0.65	2.75	P	IN
QL00837	1-Pentanesulfonamide, 1,1,2,2,3,3,4,4,5,5,5-undecafluoro-N-(phenylmethyl)-	68298-08-8	C1=CC=C(C=C1)CN[S](=O)(=O)C(F)(F)C(F)(F)C(F)(F)C(F)(F)C(F)(F)F		-7.74	0.65	2.71	P	IN

QL00838	1-Butanesulfonamide, 1,1,2,2,3,3,4,4,4-nonafluoro-N-(phenylmethyl)-	68298-07-7	C1=CC=C(C=C1)CN[S](=O)(=O)C(F)(F)C(F)(F)C(F)(F)C(F)(F)F		-7.22	0.64	2.53	P	IN
QL00839	1-(Carboxymethyl)-1-(2-hydroxyethyl)-4-(perfluoro-1-oxodecyl)piperazinium	71356-38-2	C1C[N+](CCN1C(=O)C(C(C(C(C(C(C(C(C(F)(F)F)(F)F)(F)F)(F)F)(F)F)(F)F)(F)F)(F)F)(F)F)(CCO)CC(=O)[O-]		-8.67	0.65	3.12	P	IN
QL00840	Piperidinium perfluorononanoate	95682-66-9	C1CC[NH2+]CC1.[O-]C(=O)C(F)(F)C(F)(F)C(F)(F)C(F)(F)C(F)(F)C(F)(F)C(F)(F)C(F)(F)F		-7.75	0.65	2.69	P	IN
QL00841	Piperidinium perfluorooctanesulfonate	71463-74-6	C1CC[NH2+]CC1.[O-]S(=O)(=O)C(F)(F)C(F)(F)C(F)(F)C(F)(F)C(F)(F)C(F)(F)C(F)(F)C(F)(F)F		-7.94	0.66	2.69	P	IN
QL00842	3-Pentanol, 1,1,1,2,4,5,5,5-octafluoro-2,4-bis(trifluoromethyl)-, compd. with tetrahydrofuran (11)	6884-92-0	C1CCOC1.OC(C(F)(C(F)(F)F)C(F)(F)F)C(F)(C(F)(F)F)C(F)(F)F		-6.15	0.64	1.98	P	IN
QL00843	Undecafluorohexanoic acid--piperazine (1/1)	423-47-2	C1CNCCN1.OC(=O)C(F)(F)C(F)(F)C(F)(F)C(F)(F)C(F)(F)F		-6.40	0.63	2.24	P	IN
QL00844	Heptafluorobutanoic acid--piperazine (1/1)	375-04-2	C1CNCCN1.OC(=O)C(F)(F)C(F)(F)C(F)(F)F		-4.50	0.61	1.56	P	OUT
QL00845	Difluoro(trifluoromethoxy)acetic acid--piperazine (1/1)	2266-82-2	C1CNCCN1.OC(=O)C(F)(F)OC(F)(F)F		-3.66	0.59	1.52	P	OUT
QL00846	2,3,4,5-Tetrachloro-6-[3-(perfluoroheptyl)sulfonyloxyphenylamino]carbonylbenzoate potassium	68541-01-5	C2=C(NC(=O)C1=C(Cl)C(=C(Cl)C(=C1C([O-])=O)Cl)Cl)C=CC=C2O[S](=O)(=O)C(F)(F)C(F)(F)C(F)(F)C(F)(F)C(F)(F)C(F)(F)C(F)(F)F.[K+]		-8.56	0.64	3.31	P	OUT
QL00847	Potassium 6-(3-(perfluorohexyl)sulfonyloxyphenylaminocarbonyl)perchlorobenzoate	68815-72-5	C2=C(NC(=O)C1=C(Cl)C(=C(Cl)C(=C1C([O-])=O)Cl)Cl)C=CC=C2O[S](=O)(=O)C(F)(F)C(F)(F)C(F)(F)C(F)(F)C(F)(F)C(F)(F)F.[K+]		-8.35	0.63	3.26	P	OUT
QL00848	2,3,4,5-Tetrachloro-6-[3-[(perfluoropentyl)sulfonyloxy]phenylaminocarbonyl]benzoate potassium salt	68541-02-6	C2=C(NC(=O)C1=C(Cl)C(=C(Cl)C(=C1C([O-])=O)Cl)Cl)C=CC=C2O[S](=O)(=O)C(F)(F)C(F)(F)C(F)(F)C(F)(F)C(F)(F)F.[K+]		-8.02	0.63	3.16	P	OUT
QL00849	1,1,1,3,3,4,4,4-Octafluoro-2-(trifluoromethyl)butan-2-yl 2-methylprop-2-enoate	101061-05-6	CC(=C)C(=O)OC(C(F)(F)F)(C(F)(F)F)C(F)(F)C(F)(F)F		-6.07	0.66	1.75	P	IN
QL00850	1,1,1,3,3,3-Hexafluoro-2-(trifluoromethyl)propan-2-yl 2-methylprop-2-enoate	17526-97-5	CC(=C)C(=O)OC(C(F)(F)F)(C(F)(F)F)C(F)(F)F		-5.45	0.65	1.53	P	IN
QL00851	1,1,1,2,3,3,3-Heptafluoropropan-2-yl 2-methylprop-2-enoate	7459-59-8	CC(=C)C(=O)OC(F)(C(F)(F)F)C(F)(F)F		-5.60	0.65	1.65	P	IN
QL00852	Pentadecafluoroheptyl 2-methylprop-2-enoate	96724-20-8	CC(=C)C(=O)OC(F)(F)C(F)(F)C(F)(F)C(F)(F)C(F)(F)C(F)(F)C(F)(F)F		-7.94	0.67	2.51	P	IN
QL00853	Undecafluoropentyl 2-methylprop-2-enoate	89004-77-3	CC(=C)C(=O)OC(F)(F)C(F)(F)C(F)(F)C(F)(F)C(F)(F)F		-7.20	0.66	2.24	P	IN
QL00854	2,2,3,3,4,4,5,5,6,6,7,7,8,8,9,9,10,10,11,11-Icosafluoroundecyl methacrylate	41123-44-8	CC(=C)C(=O)OC(F)C(F)(F)C(F)(F)C(F)(F)C(F)(F)C(F)(F)C(F)(F)C(F)(F)C(F)(F)C(F)(F)CF		-8.62	0.67	2.93	P	IN
QL00855	2,2,3,3,4,4,5,5,6,6,7,7,8,8,9,9,10,10,11,11,12,12,13,13,14,14,15,15,16,16,17,17,18,18,19,19,20,20,21,21,21-Hentetracontafluorohenicosyl 2-methylprop-2-enoate	132405-54-0	CC(=C)C(=O)OCC(F)(F)C(F)(F)C(F)(F)C(F)(F)C(F)(F)C(F)(F)C(F)(F)C(F)(F)C(F)(F)C(F)(F)C(F)(F)C(F)(F)C(F)(F)C(F)(F)C(F)(F)C(F)(F)C(F)(F)C(F)(F)C(F)(F)C(F)(F)F		-10.59	0.69	3.66	P	OUT
QL00856	2,2,3,3,4,4,5,5,6,6,7,7,8,8,9,9,10,10,11,11,11-Henicosafluoroundecyl methacrylate	48077-86-7	CC(=C)C(=O)OCC(F)(F)C(F)(F)C(F)(F)C(F)(F)C(F)(F)C(F)(F)C(F)(F)C(F)(F)C(F)(F)C(F)(F)F		-8.88	0.67	2.94	P	OUT
QL00857	2,2,3,3,4,4,5,5,6,6,7,7,8,8,9,9,10,10,10-nonadecafluorodecyl 2-methylprop-2-enoate	23069-32-1	CC(=C)C(=O)OCC(F)(F)C(F)(F)C(F)(F)C(F)(F)C(F)(F)C(F)(F)C(F)(F)C(F)(F)C(F)(F)F		-8.73	0.67	2.89	P	IN
QL00858	2,2,3,3,4,4,5,5,6,6,7,7,8,8,9,9,9-Heptadecafluorononyl methacrylate	48077-33-4	CC(=C)C(=O)OCC(F)(F)C(F)(F)C(F)(F)C(F)(F)C(F)(F)C(F)(F)C(F)(F)C(F)(F)F		-8.36	0.67	2.74	P	IN
QL00859	(Perfluorohexyl)methyl methacrylate	48076-44-4	CC(=C)C(=O)OCC(F)(F)C(F)(F)C(F)(F)C(F)(F)C(F)(F)C(F)(F)F		-7.74	0.66	2.52	P	IN
QL00860	(6H-Perfluorohexyl)methyl methacrylate	2261-99-6	CC(=C)C(=O)OCC(F)(F)C(F)(F)C(F)(F)C(F)(F)C(F)(F)C(F)F		-7.49	0.65	2.52	P	IN
QL00861	2,2,3,3,4,4,5,5,6,6,6-Undecafluorohexyl 2-methylprop-2-enoate	13173-36-9	CC(=C)C(=O)OCC(F)(F)C(F)(F)C(F)(F)C(F)(F)C(F)(F)F		-7.51	0.66	2.47	P	IN
QL00862	2,2,3,3,4,4,5,5,5-Nonafluoropentyl methacrylate	59006-65-4	CC(=C)C(=O)OCC(F)(F)C(F)(F)C(F)(F)C(F)(F)F		-6.93	0.65	2.26	P	IN
QL00863	1H,1H,5H-Perfluoropentyl methacrylate	355-93-1	CC(=C)C(=O)OCC(F)(F)C(F)(F)C(F)(F)C(F)F		-6.60	0.64	2.27	P	IN
QL00864	1H,1H-Perfluorobutyl methacrylate	13695-31-3	CC(=C)C(=O)OCC(F)(F)C(F)(F)C(F)(F)F		-6.45	0.64	2.13	P	IN
QL00865	1H,1H-Perfluoropropyl methacrylate	45115-53-5	CC(=C)C(=O)OCC(F)(F)C(F)(F)F		-5.59	0.63	1.86	P	IN
QL00866	1H,1H,3H-Perfluorobutyl 2-methylacrylate	36405-47-7	CC(=C)C(=O)OCC(F)(F)C(F)C(F)(F)F		-5.98	0.63	2.15	P	OUT
QL00867	4,4,5,5,6,7,7,7-Octafluoro-2-hydroxy-6-(trifluoromethyl)heptyl 2-methylprop-2-enoate	16083-79-7	CC(=C)C(=O)OCC(O)CC(F)(F)C(F)(F)C(F)(C(F)(F)F)C(F)(F)F		-7.12	0.65	2.46	P	IN
QL00868	3-(Perfluoro-5-methylhexyl)-2-hydroxypropyl methacrylate	16083-81-1	CC(=C)C(=O)OCC(O)CC(F)(F)C(F)(F)C(F)(F)C(F)(F)C(F)(C(F)(F)F)C(F)(F)F		-7.90	0.66	2.71	P	IN
QL00869	(Perfluoro-7-methyloctyl)-2-hydroxypropyl 2-methylprop-2-enoate	88752-37-8	CC(=C)C(=O)OCC(O)CC(F)(F)C(F)(F)C(F)(F)C(F)(F)C(F)(F)C(F)(F)C(F)(C(F)(F)F)C(F)(F)F		-8.49	0.66	2.91	P	IN
QL00870	4,4,5,5,6,6,7,7,8,8,9,9,10,10,11,11,11-Heptadecafluoro-2-hydroxyundecyl 2-methylprop-2-enoate	93706-76-4	CC(=C)C(=O)OCC(O)CC(F)(F)C(F)(F)C(F)(F)C(F)(F)C(F)(F)C(F)(F)C(F)(F)C(F)(F)F		-8.53	0.66	2.95	P	IN

QL00871	4,4,5,5,6,6,7,7,8,8,9,9,9-Tridecafluoro-2-hydroxynonyl 2-methylprop-2-enoate	86994-47-0	CC(=C)C(=O)OCC(O)CC(F)(F)C(F)(F)C(F)(F)C(F)(F)C(F)(F)C(F)(F)F		-7.95	0.65	2.76	P	IN
QL00872	4,4,5,5,6,6,7,7,7-Nonafluoro-2-hydroxyheptyl 2-methylprop-2-enoate	36915-03-4	CC(=C)C(=O)OCC(O)CC(F)(F)C(F)(F)C(F)(F)C(F)(F)F		-7.15	0.64	2.52	P	IN
QL00873	(Perfluorocyclohexyl)methyl methacrylate	25965-83-7	CC(=C)C(=O)OCC1(F)C(F)(F)C(F)(F)C(F)(F)C(F)(F)C1(F)F		-7.26	0.67	2.20	P	IN
QL00874	2-(Perfluoro-3-methylbutyl)ethylÂ methacrylate	65195-44-0	CC(=C)C(=O)OCCC(F)(F)C(F)(F)C(F)(C(F)(F)F)C(F)(F)F		-7.00	0.65	2.35	P	IN
QL00875	3,3,4,4,5,5,6,6,7,7-Decafluorooctyl 2-methylprop-2-enoate	61915-92-2	CC(=C)C(=O)OCCC(F)(F)C(F)(F)C(F)(F)C(F)(F)C(C)(F)F		-7.45	0.65	2.53	P	IN
QL00876	(Perfluoro-5-methylhexyl)ethyl 2-methylprop-2-enoate	50836-66-3	CC(=C)C(=O)OCCC(F)(F)C(F)(F)C(F)(F)C(F)(F)C(F)(C(F)(F)F)C(F)(F)F		-7.88	0.66	2.65	P	IN
QL00877	102 Fluorotelomer methacrylate	2144-54-9	CC(=C)C(=O)OCCC(F)(F)C(F)(F)C(F)(F)C(F)(F)C(F)(F)C(F)(F)C(F)(F)C(F)(F)C(F)(F)C(F)(F)F		-9.02	0.67	3.07	P	OUT
QL00878	82 Fluorotelomer methacrylate	1996-88-9	CC(=C)C(=O)OCCC(F)(F)C(F)(F)C(F)(F)C(F)(F)C(F)(F)C(F)(F)C(F)(F)C(F)(F)F		-8.53	0.67	2.90	P	IN
QL00879	3,3,4,4,5,5,5-Heptafluoropentyl 2-methylprop-2-enoate	2145-81-5	CC(=C)C(=O)OCCC(F)(F)C(F)(F)C(F)(F)F		-6.38	0.64	2.21	P	IN
QL00880	Poly(difluoromethylene), alpha-fluoro-omega-[2-[(2-methyl-1-oxo-2-propenyl)oxy]ethyl]-|2-Propenoic acid, esters, 2-methyl-, dodecyl ester, polymer with .alpha.-fluoro-.omega.-[2-[(2-methyl-1-oxo-2-propen-1-yl)oxy]ethyl]poly(difluoromethylene)|2-Propenoic acid, 2-methyl-, 2-ethylhexyl ester, polymer with a-fluoro-<U+03C9>-[2-[(2-methyl-1-oxo-2-propen-1-yl)oxy]ethyl]poly(difluoromethylene), 2-hydroxyethyl 2-methyl-2-propenoate and N-(hydroxymethyl)-2-propenamide|POLYFLGSID_897592	65530-66-7|65605-58-5|68239-43-0|65636-35-3	CC(=C)C(=O)OCCC(F)(F)F		-4.57	0.59	1.89	P	OUT
QL00881	4,4,5,5,6,6,7,7,8,8,9,9,10,10,11,11,11-Heptadecafluoroundecyl 2-methylprop-2-enoate	150625-20-0	CC(=C)C(=O)OCCCC(F)(F)C(F)(F)C(F)(F)C(F)(F)C(F)(F)C(F)(F)C(F)(F)C(F)(F)F		-8.49	0.66	2.95	P	IN
QL00882	4,4,5,5,6,6,7,7,8,8,9,9,9-Tridecafluorononyl 2-methylprop-2-enoate	1228350-17-1	CC(=C)C(=O)OCCCC(F)(F)C(F)(F)C(F)(F)C(F)(F)C(F)(F)C(F)(F)F		-7.89	0.65	2.76	P	IN
QL00883	4,4,5,5,6,6,7,7,7-Nonafluoroheptyl 2-methylprop-2-enoate	243842-15-1	CC(=C)C(=O)OCCCC(F)(F)C(F)(F)C(F)(F)C(F)(F)F		-7.00	0.64	2.49	P	IN
QL00884	2-[(2-Methylprop-2-enoyl)oxy]ethyl heptafluorobutanoate	146615-73-8	CC(=C)C(=O)OCCOC(=O)C(F)(F)C(F)(F)C(F)(F)F		-6.69	0.63	2.49	P	OUT
QL00885	S-((Perfluoro-5-methylhexyl)ethyl)-2-methylpropenethioate	30769-91-6	CC(=C)C(=O)SCCC(F)(F)C(F)(F)C(F)(F)C(F)(F)C(F)(C(F)(F)F)C(F)(F)F		-7.88	0.66	2.65	P	IN
QL00886	S-((Perfluoro-7-(methyl)octyl)ethyl) thiomethacrylate	28506-33-4	CC(=C)C(=O)SCCC(F)(F)C(F)(F)C(F)(F)C(F)(F)C(F)(F)C(F)(F)C(F)(C(F)(F)F)C(F)(F)F		-8.47	0.67	2.86	P	IN
QL00887	S-((Perfluoro-9-methyldecyl)ethyl) 2-methyl-2-propenethioate	30769-88-1	CC(=C)C(=O)SCCC(F)(F)C(F)(F)C(F)(F)C(F)(F)C(F)(F)C(F)(F)C(F)(F)C(F)(F)C(F)(C(F)(F)F)C(F)(F)F		-8.97	0.67	3.04	P	OUT
QL00888	3-[(1,1,1,2,3,3,3-Heptafluoropropan-2-yl)oxy]-2-methylprop-1-ene	38471-83-9	CC(=C)COC(F)(C(F)(F)F)C(F)(F)F		-5.36	0.64	1.64	P	IN
QL00889	3-Acetyl-5,5,6,6,7,7,8,8,8-nonafluorooctane-2,4-dione	120110-91-0	CC(=O)C(C(C)=O)C(=O)C(F)(F)C(F)(F)C(F)(F)C(F)(F)F		-6.72	0.65	2.15	P	IN
QL00890	3,3,4,4,5,5,6,6,7,7,8,8,9,9,9-Pentadecafluorononan-2-one	754-85-8	CC(=O)C(F)(F)C(F)(F)C(F)(F)C(F)(F)C(F)(F)C(F)(F)C(F)(F)F		-7.72	0.68	2.24	P	IN
QL00891	3,3,4,4,5,5,6,6,7,7,8,8,8-Tridecafluorooctan-2-one	80793-21-1	CC(=O)C(F)(F)C(F)(F)C(F)(F)C(F)(F)C(F)(F)C(F)(F)F		-7.50	0.68	2.16	P	IN
QL00892	Methyl perfluoropentyl ketone	2708-07-8	CC(=O)C(F)(F)C(F)(F)C(F)(F)C(F)(F)C(F)(F)F		-6.91	0.68	1.90	P	IN
QL00893	Methyl perfluorobutyl ketone	678-18-2	CC(=O)C(F)(F)C(F)(F)C(F)(F)C(F)(F)F		-6.61	0.67	1.81	P	IN
QL00894	Methyl heptafluoropropylketone	355-17-9	CC(=O)C(F)(F)C(F)(F)C(F)(F)F		-5.76	0.67	1.46	P	IN
QL00895	Methyl perfluoroethyl ketone	374-41-4	CC(=O)C(F)(F)C(F)(F)F		-5.29	0.66	1.36	P	IN
QL00896	3,4,5,5,6,6,7,7,8,8,9,9,10,10,10-Pentadecafluorodec-3-en-2-one	92604-91-6	CC(=O)C(F)=C(F)C(F)(F)C(F)(F)C(F)(F)C(F)(F)C(F)(F)C(F)(F)F		-7.78	0.67	2.46	P	IN
QL00897	(3Z)-4,5,5,6,6,7,7,8,8,9,9,10,10,11,11,11-Hexadecafluoroundec-3-en-2-one	136909-85-8	CC(=O)C=C(/F)C(F)(F)C(F)(F)C(F)(F)C(F)(F)C(F)(F)C(F)(F)C(F)(F)F		-7.87	0.67	2.51	P	IN
QL00898	4-Amino-5,5,6,6,7,7,7-heptafluorohept-3-en-2-one	70168-24-0	CC(=O)C=C(N)C(F)(F)C(F)(F)C(F)(F)F		-6.07	0.65	1.92	P	IN
QL00899	1-[4-(Heptafluoropropyl)-1H-pyrrol-3-yl]ethan-1-one	121103-97-7	CC(=O)C1=CNC=C1C(F)(F)C(F)(F)C(F)(F)F		-5.96	0.64	1.91	P	IN
QL00900	1-[4-(1,1,1,2,3,3,3-Heptafluoropropan-2-yl)phenyl]ethan-1-one	353273-22-0	CC(=O)c1ccc(cc1)C(F)(C(F)(F)F)C(F)(F)F		-6.20	0.64	2.10	P	IN
QL00901	5,5,6,6,7,7,8,8,9,9,10,10,11,11,12,12,12-Heptadecafluorododecane-2,4-dione	203201-14-3	CC(=O)CC(=O)C(F)(F)C(F)(F)C(F)(F)C(F)(F)C(F)(F)C(F)(F)C(F)(F)C(F)(F)F		-8.30	0.67	2.69	P	IN

QL00902	(Perfluoroheptanoyl)acetone	82822-26-2	CC(=O)CC(=O)C(F)(F)C(F)(F)C(F)(F)C(F)(F)C(F)(F)C(F)(F)F		-7.66	0.66	2.45	P	IN
QL00903	5,5,6,6,7,7,8,8,9,9,9-Undecafluorononane-2,4-dione	75824-01-0	CC(=O)CC(=O)C(F)(F)C(F)(F)C(F)(F)C(F)(F)C(F)(F)F		-7.11	0.66	2.24	P	IN
QL00904	(Perfluoro-1-butyl)butane-1,3-dione	355-84-0	CC(=O)CC(=O)C(F)(F)C(F)(F)C(F)(F)C(F)(F)F		-6.82	0.65	2.18	P	IN
QL00905	5,5,6,6,7,7,7-heptafluoroheptane-2,4-dione	356-30-9	CC(=O)CC(=O)C(F)(F)C(F)(F)C(F)(F)F		-6.07	0.65	1.92	P	IN
QL00906	5,6,6,6-Tetrafluoro-5-(heptafluoropropoxy)hexane-2,4-dione	261760-03-6	CC(=O)CC(=O)C(F)(OC(F)(F)C(F)(F)C(F)(F)F)C(F)(F)F		-6.85	0.65	2.19	P	IN
QL00907	4,4,5,5,6,6,7,7,8,8,9,9,9-tridecafluorononan-2-one	77893-60-8	CC(=O)CC(F)(F)C(F)(F)C(F)(F)C(F)(F)C(F)(F)C(F)(F)F		-7.33	0.67	2.24	P	IN
QL00908	4,4,5,5,6,6,7,7,7-Nonafluoroheptan-2-one	86358-19-2	CC(=O)CC(F)(F)C(F)(F)C(F)(F)C(F)(F)F		-6.37	0.66	1.90	P	IN
QL00909	3-(Perfluoropropyl)-2-propanone	136909-72-3	CC(=O)CC(F)(F)C(F)(F)C(F)(F)F		-5.99	0.65	1.84	P	IN
QL00910	5,5,6,6,7,7,8,8,9,9,10,10,11,11,12,12,12-Heptadecafluorododecan-2-one	82486-22-4	CC(=O)CCC(F)(F)C(F)(F)C(F)(F)C(F)(F)C(F)(F)C(F)(F)C(F)(F)C(F)(F)F		-8.27	0.67	2.69	P	IN
QL00911	4-(Perfluorobutyl)-2-butanone	140834-64-6	CC(=O)CCC(F)(F)C(F)(F)C(F)(F)C(F)(F)F		-6.77	0.65	2.19	P	IN
QL00912	N-[(4-Bromophenyl)(perfluorobutyl)-λ4-sulfanylidene]acetamide	1178899-27-8	CC(=O)N=S(/C1=CC=C(Br)C=C1)C(F)(F)C(F)(F)C(F)(F)C(F)(F)F		-6.75	0.64	2.41	P	IN
QL00913	N-[(Perfluorobutyl)phenyl-lambda4-sulfanylidene]acetamide	1178899-18-7	CC(=O)N=S(/C1=CC=CC=C1)C(F)(F)C(F)(F)C(F)(F)C(F)(F)F		-6.24	0.63	2.22	P	OUT
QL00914	Pyrifluquinazon	337458-27-2	CC(=O)N1C2=C(CN(C1=O)NCC3=CN=CC=C3)C=C(C=C2)C(C(F)(F)F)(C(F)(F)F)F		-6.88	0.62	2.73	P	OUT
QL00915	1-[3-Methylidene-4-(2,2,3,3,4,4,5,5,5-nonafluoropentyl)pyrrolidin-1-yl]ethan-1-one	31164-13-3	CC(=O)N1CC(CC(F)(F)C(F)(F)C(F)(F)C(F)(F)F)C(=C)C1		-7.17	0.65	2.40	P	IN
QL00916	1-Acetyl-3,5-bis(perfluorohexyl)pyrazole	231953-34-7	CC(=O)N1N=C(C=C1C(F)(F)C(F)(F)C(F)(F)C(F)(F)C(F)(F)C(F)(F)F)C(F)(F)C(F)(F)C(F)(F)C(F)(F)C(F)(F)C(F)(F)F		-9.20	0.68	3.04	P	OUT
QL00917	N,N'-(5-{[1,1,1,4,5,5,5-Heptafluoro-3-(1,1,1,2,3,3,3-heptafluoropropan-2-yl)-4-(trifluoromethyl)pent-2-en-2-yl]oxy}-1,3-phenylene)diacetamide	150225-65-3	CC(=O)Nc1cc(NC(C)=O)cc(OC(=C(C(F)(C(F)(F)F)C(F)(F)F)C(F)(C(F)(F)F)C(F)(F)F)C(F)(F)F)c1		-6.57	0.64	2.27	P	IN
QL00918	N-[4-(Heptafluoropropyl)phenyl]acetamide	199530-73-9	CC(=O)Nc1ccc(cc1)C(F)(F)C(F)(F)C(F)(F)F		-6.80	0.63	2.50	P	OUT
QL00919	4-Acetamidophenyl nonafluorobutane-1-sulfonate	41605-57-6	CC(=O)Nc1ccc(OS(=O)(=O)C(F)(F)C(F)(F)C(F)(F)C(F)(F)F)cc1		-7.62	0.64	2.76	P	IN
QL00920	4-Acetamido-3-chlorophenyl nonafluorobutane-1-sulfonate	1428370-26-6	CC(=O)Nc1ccc(OS(=O)(=O)C(F)(F)C(F)(F)C(F)(F)C(F)(F)F)cc1Cl		-7.67	0.64	2.75	P	IN
QL00921	N-[8-(Heptafluoropropyl)-9H-purin-6-yl]acetamide	147916-78-7	CC(=O)Nc1ncnc2Nc(nc12)C(F)(F)C(F)(F)C(F)(F)F		-6.69	0.63	2.44	P	OUT
QL00922	N-[5-(Heptafluoropropyl)-1,3,4-thiadiazol-2-yl]acetamide	356795-59-0	CC(=O)Nc1nnc(s1)C(F)(F)C(F)(F)C(F)(F)F		-6.53	0.64	2.29	P	IN
QL00923	N-{4-[(1,1,2,2,3,3,4,4,4-Nonafluorobutane-1-sulfonyl)amino]benzene-1-sulfonyl}acetamide	320599-24-4	CC(=O)NS(=O)(=O)c1ccc(NS(=O)(=O)C(F)(F)C(F)(F)C(F)(F)C(F)(F)F)cc1		-8.04	0.65	2.89	P	IN
QL00924	Iodine, bis(acetato-βO)[4-(3,3,4,4,5,5,6,6,7,7,8,8,9,9,10,10,10-heptadecafluorodecyl)phenyl]- (9CI)	882186-02-9	CC(=O)O[I](OC(C)=O)C1=CC=C(CCC(F)(F)C(F)(F)C(F)(F)C(F)(F)C(F)(F)C(F)(F)C(F)(F)C(F)(F)F)C=C1		-8.56	0.64	3.20	P	OUT
QL00925	(1H,1H,2H,2H-perfluorooctyl)triacetoxysilane	1034681-41-8	CC(=O)O[Si](CCC(F)(F)C(F)(F)C(F)(F)C(F)(F)C(F)(F)C(F)(F)F)(OC(C)=O)OC(C)=O		-7.35	0.64	2.63	P	IN
QL00926	4,4,5,5,6,6,6-Heptafluorohex-1-en-2-yl acetate	136909-80-3	CC(=O)OC(=C)CC(F)(F)C(F)(F)C(F)(F)F		-6.23	0.64	2.13	P	IN
QL00927	3,4,6-Trichloro-2-(2-chloro-4,4,5,5,6,6,7,7,8,8,9,9,10,10,11,11,11-heptadecafluoroundecyl)phenyl acetate	38506-78-4	CC(=O)Oc1c(Cl)cc(Cl)c(Cl)c1CC(Cl)CC(F)(F)C(F)(F)C(F)(F)C(F)(F)C(F)(F)C(F)(F)C(F)(F)C(F)(F)F		-8.53	0.65	3.06	P	IN
QL00928	5-Chloro-2-(2-chloro-4,4,5,5,6,6,7,7,8,8,9,9,10,10,11,11,11-heptadecafluoroundecyl)phenyl acetate	38506-72-8	CC(=O)Oc1cc(Cl)ccc1CC(Cl)CC(F)(F)C(F)(F)C(F)(F)C(F)(F)C(F)(F)C(F)(F)C(F)(F)C(F)(F)F		-8.50	0.65	3.08	P	IN
QL00929	4-Chloro-2-(2-chloro-4,4,5,5,6,6,7,7,8,8,9,9,10,10,11,11,11-heptadecafluoroundecyl)phenyl acetate	38506-76-2	CC(=O)Oc1ccc(Cl)cc1CC(Cl)CC(F)(F)C(F)(F)C(F)(F)C(F)(F)C(F)(F)C(F)(F)C(F)(F)C(F)(F)F		-8.50	0.65	3.08	P	IN
QL00930	2,2,3,3,4,4,4-Heptafluorobutyl acetate	356-06-9	CC(=O)OCC(F)(F)C(F)(F)C(F)(F)F		-6.00	0.64	1.93	P	IN
QL00931	3-(Perfluoropropyl)-2-iodopropanol acetate	3108-05-2	CC(=O)OCC(I)CC(F)(F)C(F)(F)C(F)(F)F		-6.32	0.63	2.26	P	OUT
QL00932	[3,4,6-triacetyloxy-5-(1,1,2,2,3,3,4,4,4-nonafluorobutylsulfonyloxy)oxan-2-yl]methyl Acetate	480438-48-0	CC(=O)OCC1OC(OC(C)=O)C(OS(=O)(=O)C(F)(F)C(F)(F)C(F)(F)C(F)(F)F)C(OC(C)=O)C1OC(C)=O		-7.23	0.62	2.83	P	OUT
QL00933	3,3,4,4,5,5,6,6,7,7,8,8,9,9,10,10,10-Heptadecafluorodecyl acetate	37858-04-1	CC(=O)OCCC(F)(F)C(F)(F)C(F)(F)C(F)(F)C(F)(F)C(F)(F)C(F)(F)C(F)(F)F		-8.23	0.67	2.74	P	IN
QL00934	3,3,4,4,5,5,6,6,7,7,8,8,8-Tridecafluorooctyl acetate	37858-03-0	CC(=O)OCCC(F)(F)C(F)(F)C(F)(F)C(F)(F)C(F)(F)C(F)(F)F		-7.57	0.66	2.52	P	IN
QL00935	3,3,4,4,5,5,6,6,6-Nonafluorohexyl acetate	80705-15-3	CC(=O)OCCC(F)(F)C(F)(F)C(F)(F)C(F)(F)F		-6.70	0.64	2.27	P	IN
QL00936	3,3,4,4,5,5,5-Heptafluoropentyl acetate	425-68-3	CC(=O)OCCC(F)(F)C(F)(F)C(F)(F)F		-6.18	0.63	2.15	P	IN
QL00937	(Perfluorooctyl)propyl acetate	150225-00-6	CC(=O)OCCCC(F)(F)C(F)(F)C(F)(F)C(F)(F)C(F)(F)C(F)(F)C(F)(F)C(F)(F)F		-8.40	0.66	2.90	P	IN

QL00938	4,4,5,5,6,6,6-Heptafluorohexyl acetate	25600-64-0	CC(=O)OCCCC(F)(F)C(F)(F)C(F)(F)F		-6.12	0.63	2.23	P	OUT
QL00939	S-[3,4,4,4-Tetrafluoro-3-(trifluoromethyl)butyl] ethanethioate	1097631-01-0	CC(=O)SCCC(F)(C(F)(F)F)C(F)(F)F		-5.34	0.63	1.75	P	OUT
QL00940	S-(3,3,4,4,5,5,6,6,7,7,8,8,9,9,10,10,10-Heptadecafluorodecyl) ethanethioate	125640-21-3	CC(=O)SCCC(F)(F)C(F)(F)C(F)(F)C(F)(F)C(F)(F)C(F)(F)C(F)(F)C(F)(F)F		-8.23	0.67	2.74	P	IN
QL00941	S-(3,3,4,4,5,5,6,6,7,7,8,8,8-Tridecafluorooctyl) ethanethioate	213681-67-5	CC(=O)SCCC(F)(F)C(F)(F)C(F)(F)C(F)(F)C(F)(F)C(F)(F)F		-7.57	0.66	2.52	P	IN
QL00942	2-Bromo-3-(bromomethyl)-4,4,5,5,6,6,6-heptafluorohexan-3-ol	93339-87-8	CC(Br)C(O)(CBr)C(F)(F)C(F)(F)C(F)(F)F		-6.13	0.66	1.79	P	IN
QL00943	2-Methyl-2-trifluoromethylperfluoropentane	100645-99-6	CC(C(F)(F)F)(C(F)(F)F)C(F)(F)C(F)(F)C(F)(F)F		-6.74	0.68	1.72	P	IN
QL00944	3,3,4,4,5,5,6,6,7,7,8,8,9,9,10,10,10-Heptadecafluoro-2-methyldecanoic acid	136022-86-1	CC(C(O)=O)C(F)(F)C(F)(F)C(F)(F)C(F)(F)C(F)(F)C(F)(F)C(F)(F)C(F)(F)F		-8.15	0.68	2.52	P	IN
QL00945	1,1,1,2,2,3,3,4,4,5,5,6,6-Tridecafluoro-7-methyl-7-nitrooctane	84108-41-8	CC(C)([N+]([O-])=O)C(F)(F)C(F)(F)C(F)(F)C(F)(F)C(F)(F)C(F)(F)F		-7.72	0.68	2.24	P	IN
QL00946	Calcium (3E)-6,6,7,7,8,8,8-heptafluoro-2,2-dimethyl-5-oxooct-3-en-3-olate (3Z)-6,6,7,7,8,8,8-heptafluoro-2,2-dimethyl-5-oxooct-3-en-3-olate (1/1/1)|1,1,1,2,2,3,3-heptafluoro-6-hydroxy-7,7-dimethyloct-5-en-4-one|Silver, (6,6,7,7,8,8,8-heptafluoro-2,2-dimethyl-3,5-octanedionato-ßO3,ßO5)-|1,1,1,2,2,3,3-Heptafluoro-6-hydroxy-7,7-dimethyloct-5-en-4-one--copper (2/1)|Lead, bis(6,6,7,7,8,8,8-heptafluoro-2,2-dimethyl-3,5-octanedionato-ßO,ßO')-, (T-4)- (9CI)|Europium, tris(6,6,7,7,8,8,8-heptafluoro-2,2-dimethyl-3,5-octanedionato-ßO3,ßO5)-|Erbium, tris(6,6,7,7,8,8,8-heptafluoro-2,2-dimethyl-3,5-octanedionato-ßO3,ßO5)-|Neodymium, tris(6,6,7,7,8,8,8-heptafluoro-2,2-dimethyl-3,5-octanedionato-ßO3,ßO5)-|Praseodymium, tris(6,6,7,7,8,8,8-heptafluoro-2,2-dimethyl-3,5-	124053-49-2|62773-05-1|76121-99-8|38926-19-1|21600-78-2|17631-68-4|17978-75-5|17978-76-6|17978-77-7|18323-96-1|18323-97-2|18323-98-3|172424-98-5|19106-89-9|19186-73-	CC(C)(/C(O)=C/C(C(F)(C(F)(C(F)(F)F)F)F)=O)C		-6.95	0.66	2.15	P	IN
QL00947	N-{1-[3,5-Bis({[tert-butyl(dimethyl)silyl]oxy}methyl)phenyl]-2,2,3,3,4,4,5,5,6,6,7,7,7-tridecafluoroheptylidene}hydroxylamine	1335496-61-1	CC(C)(C)[Si](C)(C)OCc1cc(CO[Si](C)(C)C(C)(C)C)cc(c1)C(=NO)C(F)(F)C(F)(F)C(F)(F)C(F)(F)C(F)(F)C(F)(F)F		-8.31	0.66	2.89	P	IN
QL00948	1-[3,5-Bis({[tert-butyl(dimethyl)silyl]oxy}methyl)phenyl]-2,2,3,3,4,4,5,5,6,6,7,7,7-tridecafluoroheptan-1-one	1335496-60-0	CC(C)(C)[Si](C)(C)OCc1cc(CO[Si](C)(C)C(C)(C)C)cc(c1)C(=O)C(F)(F)C(F)(F)C(F)(F)C(F)(F)C(F)(F)C(F)(F)F		-8.47	0.66	2.88	P	IN
QL00949	6,6,7,7,8,8,8-Heptafluoro-2,2-dimethyl-3,4,5-octanetrione 4-(4-nitrophenyl)hydrazone	80284-51-1	CC(C)(C)C(=O)C(=NNC1=CC=C(C=C1)[N+]([O-])=O)C(=O)C(F)(F)C(F)(F)C(F)(F)F		-7.26	0.63	2.68	P	OUT
QL00950	6,6,7,7,8,8,9,9,9-Nonafluoro-2,2-dimethylnon-4-en-3-one	105602-69-5	CC(C)(C)C(=O)C=CC(F)(F)C(F)(F)C(F)(F)C(F)(F)F		-7.25	0.67	2.24	P	IN
QL00951	(Heptafluorobutanoyl)pivaloylmethane	17587-22-3	CC(C)(C)C(=O)CC(=O)C(F)(F)C(F)(F)C(F)(F)F		-6.95	0.66	2.15	P	IN
QL00952	4H,4H-Perfluoro-6,6-dimethylheptane-3,5-dione	2145-68-8	CC(C)(C)C(=O)CC(=O)C(F)(F)C(F)(F)F		-6.23	0.65	1.88	P	IN
QL00953	1,1-Difluoro-5,5-dimethyl-1-(trifluoromethoxy)hexane-2,4-dione	61894-06-2	CC(C)(C)C(=O)CC(=O)C(F)(F)OC(F)(F)F		-6.32	0.64	2.15	P	IN
QL00954	1,1,1,2,2,3,3,4,4-Nonafluoro-6-iodo-7,7-dimethyloctane	1464148-69-3	CC(C)(C)C(I)CC(F)(F)C(F)(F)C(F)(F)C(F)(F)F		-7.25	0.67	2.17	P	IN
QL00955	6-Amino-1,1,1,2,2,3,3-heptafluoro-7,7-dimethyloct-5-en-4-one	188838-46-2	CC(C)(C)C(N)=CC(=O)C(F)(F)C(F)(F)C(F)(F)F		-6.95	0.66	2.15	P	IN
QL00956	1,1,1,2,2,3,3-heptafluoro-6-hydroxy-7,7-dimethyloct-5-en-4-one	62773-05-1	CC(C)(C)C(O)=CC(=O)C(F)(F)C(F)(F)C(F)(F)F		-6.95	0.66	2.15	P	IN
QL00957	1,4-Bis(1,1-dimethylethyl)-2,3,5,6-tetrafluorobenzene	54111-17-0	CC(C)(C)C1=C(F)C(F)=C(C(F)=C1F)C(C)(C)C		-5.87	0.64	1.89	P	IN
QL00958	Phenol, 2,2'-[(1R,2R)-1,2-cyclohexanediylbis[(E)-nitrilomethylidyne]]bis[6-(1,1-dimethylethyl)-4-(heptadecafluorooctyl)-	417715-98-1	CC(C)(C)C1=CC(=CC(C=NC2CCCC[C@H]2N=CC2=C(O)C(=CC(=C2)C(F)(F)C(F)(F)C(F)(F)C(F)(F)C(F)(F)C(F)(F)C(F)(F)C(F)(F)F)C(C)(C)C)=C1O)C(F)(F)C(F)(F)C(F)(F)C(F)(F)C(F)(F)C(F)(F)C(F)(F)C(F)(F)F		-10.24	0.66	3.79	P	OUT
QL00960	(1Z,4Z,9Z,12E)-2,10-Di-tert-butyl-4,12-bis(heptafluoropropyl)-1,5,9,13-tetraazacyclohexadeca-1,4,9,12-tetraene	925457-51-8	CC(C)(C)C1=NCCCN=C(CC(=NCCCN=C(C1)C(F)(F)C(F)(F)C(F)(F)F)C(C)(C)C)C(F)(F)C(F)(F)C(F)(F)F		-8.08	0.64	3.05	P	OUT
QL00961	5-tert-Butyl-7-(heptafluoropropyl)-2-phenylpyrazolo[1,5-a]pyrimidine	346637-99-8	CC(C)(C)c1cc(n2nc(cc2n1)-c1ccccc1)C(F)(F)C(F)(F)C(F)(F)F		-6.93	0.64	2.50	P	OUT
QL00962	5-(4-tert-Butylphenyl)-N-(2,2,3,3,4,4,4-heptafluorobutyl)-1,2-oxazole-3-carboxamide	922421-40-7	CC(C)(C)c1ccc(cc1)-c1cc(no1)C(=O)NCC(F)(F)C(F)(F)C(F)(F)F		-7.79	0.63	2.95	P	OUT
QL00963	1,1,1,2,2,3,3,4-Octafluoro-7,7-dimethyloct-4-ene	90292-33-4	CC(C)(C)CC=C(F)C(F)(F)C(F)(F)C(F)(F)F		-6.80	0.65	2.16	P	IN
QL00964	N-tert-Butyl-2,2,3,3,4,4,4-heptafluorobutanamide	99089-22-2	CC(C)(C)NC(=O)C(F)(F)C(F)(F)C(F)(F)F		-6.47	0.66	1.90	P	IN
QL00965	tert-Butyl heptafluorobutanoate	425-24-1	CC(C)(C)OC(=O)C(F)(F)C(F)(F)C(F)(F)F		-6.47	0.66	1.90	P	IN

QL00966	tert-Butyl (4,4,5,5,6,6,7,7,7-nonafluoro-2-iodoheptyl)carbamate	1301739-79-6	CC(C)(C)OC(=O)NCC(I)CC(F)(F)C(F)(F)C(F)(F)C(F)(F)F		-7.45	0.64	2.65	P	IN
QL00967	tert-Butyl heptafluorobutaneperoxoate	91481-65-1	CC(C)(C)OOC(=O)C(F)(F)C(F)(F)C(F)(F)F		-6.80	0.65	2.16	P	IN
QL00968	1,1,1,2,2,3,3,4-Octafluoro-7-methyl-7-nitrooct-4-ene	90292-31-2	CC(C)(CC=C(F)C(F)(F)C(F)(F)C(F)(F)F)[N+]([O-])=O		-6.94	0.66	2.23	P	IN
QL00969	(2-Perfluorooctylethyl)isopropoxycarbonyloxyiminophenylacetonitrile	350716-42-6	CC(C)(CCC(F)(F)C(F)(F)C(F)(F)C(F)(F)C(F)(F)C(F)(F)C(F)(F)C(F)(F)F)OC(=O)ON=C(C#N)C1=CC=CC=C1		-8.81	0.64	3.36	P	OUT
QL00970	sodium 2-methyl-2-{3-[(3,3,4,4,4-pentafluorobutyl)sulfanyl]propanamido}propane-1-sulfonate		CC(C)(CS(O)(=O)=O)NC(=O)CCSCCC(F)(F)C(F)(F)F		-6.87	0.62	2.69	P	OUT
QL00971	N-(2,2,3,3,4,4,4-Heptafluorobutanoyl)-2-methylalanine	880353-20-8	CC(C)(NC(=O)C(F)(F)C(F)(F)C(F)(F)F)C(O)=O		-6.94	0.66	2.15	P	IN
QL00972	3,3,4,4,5,5,6,6,7,7,8,8,9,9,9-Pentadecafluoro-2-methylnonan-2-ol	92914-88-0	CC(C)(O)C(F)(F)C(F)(F)C(F)(F)C(F)(F)C(F)(F)C(F)(F)C(F)(F)F		-7.94	0.69	2.24	P	IN
QL00973	2-Perfluoropropyl-2-propanol	355-22-6	CC(C)(O)C(F)(F)C(F)(F)C(F)(F)F		-6.18	0.68	1.45	P	IN
QL00974	5,5,6,6,7,7,8,8,8-Nonafluoro-2-methyloct-3-yn-2-ol	82721-68-4	CC(C)(O)C#CC(F)(F)C(F)(F)C(F)(F)C(F)(F)F		-7.14	0.67	2.17	P	IN
QL00975	5,5,6,6,7,7,8,8,9,9,10,10,11,11,12,12,12-Heptadecafluoro-2-methyldodecan-2-ol	141183-94-0	CC(C)(O)CCC(F)(F)C(F)(F)C(F)(F)C(F)(F)C(F)(F)C(F)(F)C(F)(F)C(F)(F)F		-8.49	0.68	2.68	P	IN
QL00976	5,5,6,6,7,7,8,8,9,9,10,10,10-Tridecafluoro-2-methyldecan-2-ol	159142-65-1	CC(C)(O)CCC(F)(F)C(F)(F)C(F)(F)C(F)(F)C(F)(F)C(F)(F)F		-7.90	0.67	2.44	P	IN
QL00977	5,5,6,6,7,7,8,8,8-Nonafluoro-2-methyloctan-2-ol	269394-08-3	CC(C)(O)CCC(F)(F)C(F)(F)C(F)(F)C(F)(F)F		-7.14	0.67	2.17	P	IN
QL00978	(4S,5R)-4-(Propan-2-yl)-5-(3,3,4,4,5,5,6,6,7,7,8,8,8-tridecafluorooctyl)-1,3-oxazolidin-2-one	871210-22-9	CC(C)[C@@H]1NC(=O)O[C@@H]1CCC(F)(F)C(F)(F)C(F)(F)C(F)(F)C(F)(F)C(F)(F)F		-7.86	0.66	2.69	P	IN
QL00979	N-[(2S)-2-Amino-3-methylbutyl]-1,1,2,2,3,3,4,4,4-nonafluorobutane-1-sulfonamide	1417316-15-4	CC(C)[C@H](N)CNS(=O)(=O)C(F)(F)C(F)(F)C(F)(F)C(F)(F)F		-7.51	0.66	2.47	P	IN
QL00980	N,N-Dimethyl-N-(propan-2-yl)propan-2-aminium nonafluorobutane-1-sulfonate	374571-81-0	CC(C)[N+](C)(C)C(C)C.[O-]S(=O)(=O)C(F)(F)C(F)(F)C(F)(F)C(F)(F)F		-7.07	0.67	2.14	P	IN
QL00981	(3,3,4,4,5,5,6,6,7,7,8,8,9,9,10,10,11,11,12,12,12-Henicosafluorododecyl)di(propan-2-yl)silane	356056-16-1	CC(C)[SiH](CCC(F)(F)C(F)(F)C(F)(F)C(F)(F)C(F)(F)C(F)(F)C(F)(F)C(F)(F)C(F)(F)C(F)(F)F)C(C)C		-8.75	0.67	2.93	P	IN
QL00982	((Perfluorooctyl)ethyl)di(propan-2-yl)silane	356056-15-0	CC(C)[SiH](CCC(F)(F)C(F)(F)C(F)(F)C(F)(F)C(F)(F)C(F)(F)C(F)(F)C(F)(F)F)C(C)C		-8.21	0.67	2.73	P	IN
QL00983	Di(propan-2-yl)(3,3,4,4,5,5,6,6,7,7,8,8,8-tridecafluorooctyl)silane	356056-14-9	CC(C)[SiH](CCC(F)(F)C(F)(F)C(F)(F)C(F)(F)C(F)(F)C(F)(F)F)C(C)C		-7.56	0.66	2.49	P	IN
QL00984	(3,3,4,4,5,5,6,6,6-Nonafluorohexyl)di(propan-2-yl)silane	356056-13-8	CC(C)[SiH](CCC(F)(F)C(F)(F)C(F)(F)C(F)(F)F)C(C)C		-6.71	0.65	2.22	P	IN
QL00985	6-(1-Methylethylidene)-5-(triphenylphosphoranylidene)-(perfluoro)-nonan-4-one	139225-92-6	CC(C)=C(C(C(=O)C(F)(F)C(F)(F)C(F)(F)F)=P(C1=CC=CC=C1)(C1=CC=CC=C1)C1=CC=CC=C1)C(F)(F)C(F)(F)C(F)(F)F		-6.20	0.62	2.28	P	OUT
QL00986	7,7,8,8,9,9,9-Heptafluoro-2-methylnon-2-ene-4,6-dione	559-99-9	CC(C)=CC(=O)CC(=O)C(F)(F)C(F)(F)C(F)(F)F		-6.42	0.63	2.25	P	IN
QL00987	5,6,6,7,7,8,8,8-Octafluoro-2-methylocta-2,4-diene	90292-28-7	CC(C)=CC=C(F)C(F)(F)C(F)(F)C(F)(F)F		-6.40	0.64	2.18	P	IN
QL00988	5,5,6,6,7,7,8,8,8-Nonafluoro-2-methyloct-2-ene	97294-02-5	CC(C)=CCC(F)(F)C(F)(F)C(F)(F)C(F)(F)F		-6.77	0.65	2.19	P	IN
QL00989	2-Methylprop-1-en-1-yl nonafluorobutane-1-sulfonate	84224-48-6	CC(C)=COS(=O)(=O)C(F)(F)C(F)(F)C(F)(F)C(F)(F)F		-7.07	0.66	2.24	P	IN
QL00990	1,1,1,2-Tetrafluoro-4-methyl-2-(trifluoromethyl)pentan-3-one	62656-72-8	CC(C)C(=O)C(F)(C(F)(F)F)C(F)(F)F		-5.27	0.65	1.40	P	IN
QL00991	3,3,4,4,5,5,6,6,7,7,8,8,8-tridecafluorooctyl 2-methylpropanoate	242812-05-1	CC(C)C(=O)OCCC(F)(F)C(F)(F)C(F)(F)C(F)(F)C(F)(F)C(F)(F)F		-7.94	0.66	2.71	P	IN
QL00992	3,3,4,4,5,5,6,6,6-Nonafluorohexyl 2,2-difluoro-3-hydroxy-4-methylpentanoate	1370345-78-0	CC(C)C(O)C(F)(F)C(=O)OCCC(F)(F)C(F)(F)C(F)(F)C(F)(F)F		-7.56	0.65	2.61	P	IN
QL00993	1-(1,3-Dimethylbutoxy)-2-(perfluorohexyl)ethane	210896-25-6	CC(C)CC(C)OCCC(F)(F)C(F)(F)C(F)(F)C(F)(F)C(F)(F)C(F)(F)F		-7.81	0.65	2.76	P	IN
QL00994	3-Methylbutyl heptadecafluorononanoate	117374-32-0	CC(C)CCOC(=O)C(F)(F)C(F)(F)C(F)(F)C(F)(F)C(F)(F)C(F)(F)C(F)(F)C(F)(F)F		-8.46	0.66	2.90	P	IN
QL00995	3-Methylbutyl 2,3,3,3-tetrafluoro-2-(heptafluoropropoxy)propanoate	183873-56-5	CC(C)CCOC(=O)C(F)(OC(F)(F)C(F)(F)C(F)(F)F)C(F)(F)F		-7.20	0.64	2.53	P	IN
QL00996	Diisopentyl 2-[(2,2,3,3,4,4,4-heptafluorobutanoyl)amino]succinate	75743-06-5	CC(C)CCOC(=O)CC(NC(=O)C(F)(F)C(F)(F)C(F)(F)F)C(=O)OCCC(C)C		-6.55	0.61	2.72	P	OUT
QL00997	2,2,3,3,4,4,4-Heptafluoro-N,N-bis(2-methylpropyl)butanamide	336-30-1	CC(C)CN(CC(C)C)C(=O)C(F)(F)C(F)(F)C(F)(F)F		-6.01	0.63	2.12	P	OUT
QL00998	2,2,3,3,4,4,4-Heptafluoro-N-(2-methylpropyl)butanamide	75668-25-6	CC(C)CNC(=O)C(F)(F)C(F)(F)C(F)(F)F		-6.40	0.64	2.18	P	IN

QL00999	Tris(2-methylpropyl) 3-(2,2,3,3,4,4,4-heptafluorobutanamido)propane-1,1,3-tricarboxylate	95654-14-1	CC(C)COC(=O)C(CC(C(=O)OCC(C)C)C(=O)OCC(C)C)NC(=O)C(F)(F)C(F)(F)C(F)(F)F		-6.54	0.61	2.70	P	OUT
QL01000	2-Methylpropyl 2,3,3,3-tetrafluoro-2-(heptafluoropropoxy)propanoate	183873-54-3	CC(C)COC(=O)C(F)(OC(F)(F)C(F)(F)C(F)(F)F)C(F)(F)F		-7.22	0.65	2.45	P	IN
QL01001	2-Methylpropyl 2,3,3,3-tetrafluoro-2-[1,1,2,3,3,3-hexafluoro-2-(heptafluoropropoxy)propoxy]propanoate	340036-47-7	CC(C)COC(=O)C(F)(OC(F)(F)C(F)(OC(F)(F)C(F)(F)C(F)(F)F)C(F)(F)F)C(F)(F)F		-7.94	0.66	2.69	P	IN
QL01002	4-(1,1,1,2,3,3,3-Heptafluoropropan-2-yl)-2-methyl-3-(2-methylpropane-1-sulfonyl)benzoic acid	1355554-54-9	CC(C)CS(=O)(=O)c1c(C)c(ccc1C(F)(C(F)(F)F)C(F)(F)F)C(O)=O		-6.05	0.64	2.05	P	IN
QL01003	4-(1,1,1,2,3,3,3-Heptafluoropropan-2-yl)-2-methyl-3-(2-methylpropane-1-sulfinyl)benzoic acid	1355554-32-3	CC(C)CS(=O)c1c(C)c(ccc1C(F)(C(F)(F)F)C(F)(F)F)C(O)=O		-5.83	0.63	2.05	P	OUT
QL01004	4-(1,1,1,2,3,3,3-Heptafluoropropan-2-yl)-2-methyl-3-[(2-methylpropyl)sulfanyl]benzoic acid	1355554-12-9	CC(C)CSc1c(C)c(ccc1C(F)(C(F)(F)F)C(F)(F)F)C(O)=O		-5.77	0.63	2.04	P	OUT
QL01005	2-Cyanoethyl 4,4,5,5,6,6,7,7,8,8,9,9,10,10,11,11,11-heptadecafluoroundecyl N,N-dipropan-2-ylphosphoramidoite	1036029-24-9	CC(C)N(C(C)C)P(OCCCC(F)(F)C(F)(F)C(F)(F)C(F)(F)C(F)(F)C(F)(F)C(F)(F)C(F)(F)F)OCCC#N		-8.25	0.64	3.18	P	OUT
QL01006	1-[Di(propan-2-yl)amino]-3,3,4,4,5,5,6,6,7,7,8,8,8-tridecafluorooctan-2-ol	125672-20-0	CC(C)N(CC(O)C(F)(F)C(F)(F)C(F)(F)C(F)(F)C(F)(F)C(F)(F)F)C(C)C		-7.60	0.66	2.50	P	IN
QL01007	2,2,3,3,4,4,5,5,6,6,7,7,8,8,9,9,9-Heptadecafluoro-N-(propan-2-yl)nonanamide	6104-22-9	CC(C)NC(=O)C(F)(F)C(F)(F)C(F)(F)C(F)(F)C(F)(F)C(F)(F)C(F)(F)C(F)(F)F		-8.30	0.67	2.69	P	IN
QL01008	1H,1H,2H,2H-Perfluorodecyl(tri-iso-propoxy)silane	246234-80-0	CC(C)O[Si](CCC(F)(F)C(F)(F)C(F)(F)C(F)(F)C(F)(F)C(F)(F)C(F)(F)C(F)(F)F)(OC(C)C)OC(C)C		-8.01	0.65	2.85	P	IN
QL01009	1-Methylethyl perfluorobutanimidoate	4192-05-6	CC(C)OC(=N)C(F)(F)C(F)(F)C(F)(F)F		-6.07	0.65	1.92	P	IN
QL01010	Propan-2-yl heptadecafluorononanoate	70910-41-7	CC(C)OC(=O)C(F)(F)C(F)(F)C(F)(F)C(F)(F)C(F)(F)C(F)(F)C(F)(F)C(F)(F)F		-8.30	0.67	2.69	P	IN
QL01011	Propan-2-yl 2,3,3,3-tetrafluoro-2-(heptafluoropropoxy)propanoate	147224-17-7	CC(C)OC(=O)C(F)(OC(F)(F)C(F)(F)C(F)(F)F)C(F)(F)F		-6.85	0.65	2.19	P	IN
QL01012	Propan-2-yl 2,3,3,3-tetrafluoro-2-[1,1,2,3,3,3-hexafluoro-2-(heptafluoropropoxy)propoxy]propanoate	145510-88-9	CC(C)OC(=O)C(F)(OC(F)(F)C(F)(OC(F)(F)C(F)(F)C(F)(F)F)C(F)(F)F)C(F)(F)F		-7.95	0.66	2.64	P	IN
QL01013	Dipropan-2-yl (3,3,4,4,5,5,6,6,7,7,8,8,9,9,10,10,10-heptadecafluorodecyl)phosphonate	90183-56-5	CC(C)OP(=O)(CCC(F)(F)C(F)(F)C(F)(F)C(F)(F)C(F)(F)C(F)(F)C(F)(F)C(F)(F)F)OC(C)C		-8.32	0.66	2.88	P	IN
QL01014	4-(1,1,1,2,3,3,3-Heptafluoropropan-2-yl)-2-methyl-3-(propane-2-sulfonyl)benzoic acid	1355554-50-5	CC(C)S(=O)(=O)c1c(C)c(ccc1C(F)(C(F)(F)F)C(F)(F)F)C(O)=O		-6.45	0.65	2.12	P	IN
QL01015	4-(1,1,1,2,3,3,3-Heptafluoropropan-2-yl)-2-methyl-3-(propane-2-sulfinyl)benzoic acid	1355554-27-6	CC(C)S(=O)c1c(C)c(ccc1C(F)(C(F)(F)F)C(F)(F)F)C(O)=O		-6.18	0.64	2.12	P	IN
QL01016	4-(1,1,1,2,3,3,3-Heptafluoropropan-2-yl)-2-methyl-3-[(propan-2-yl)sulfanyl]benzoic acid	1355554-05-0	CC(C)Sc1c(C)c(ccc1C(F)(C(F)(F)F)C(F)(F)F)C(O)=O		-6.03	0.63	2.12	P	OUT
QL01017	4,4,5,5,6,6,7,7,8,8,9,9,9-Tridecafluoro-2-methylnonanenitrile	1274722-59-6	CC(CC(F)(F)C(F)(F)C(F)(F)C(F)(F)C(F)(F)C(F)(F)F)C#N		-7.54	0.66	2.45	P	IN
QL01018	4,4,5,5,6,6,6-Heptafluorohexan-2-yl acetate	136909-75-6	CC(CC(F)(F)C(F)(F)C(F)(F)F)OC(C)=O		-6.23	0.64	2.13	P	IN
QL01019	1,1,1,7,7,7-Hexafluoro-5-methylhept-4-en-3-one	502457-69-4	CC(CC(F)(F)F)=CC(=O)CC(F)(F)F		-5.36	0.61	2.07	P	OUT
QL01020	2,2,3,3,4,4,4-Heptafluoro-N-(1-phenylpropan-2-yl)butanamide	90582-01-7	CC(CC1=CC=CC=C1)NC(=O)C(F)(F)C(F)(F)C(F)(F)F		-6.58	0.62	2.52	P	OUT
QL01021	4,4-Bis[(3,3,4,4,5,5,6,6,6-nonafluorohexyl)sulfanyl]pentanoic acid	92886-04-9	CC(CCC(O)=O)(SCCC(F)(F)C(F)(F)C(F)(F)C(F)(F)F)SCCC(F)(F)C(F)(F)C(F)(F)C(F)(F)F		-8.46	0.65	3.04	P	IN
QL01022	Pentanoic acid, 4,4-bis[(.gamma.-.omega.-perfluoro-C8-20-alkyl)thio] derivs., compds. with diethanolamine	71608-61-2	CC(CCN(CCO)CCO)(SCCC(F)(F)C(F)(F)C(F)(F)C(F)(F)C(F)(F)C(F)(F)F)SCCC(F)(F)C(F)(F)C(F)(F)C(F)(F)C(F)(F)C(F)(F)F		-8.87	0.65	3.32	P	OUT
QL01023	2,2,3,3,4,4,4-Heptafluoro-N-(2-phenylpropyl)butanamide	70414-03-8	CC(CNC(=O)C(F)(F)C(F)(F)C(F)(F)F)c1ccccc1		-6.59	0.62	2.49	P	OUT
QL01024	N,N'-(Propane-1,2-diyl)bis(2,2,3,3,4,4,4-heptafluorobutanamide)	133911-24-7	CC(CNC(=O)C(F)(F)C(F)(F)C(F)(F)F)NC(=O)C(F)(F)C(F)(F)C(F)(F)F		-7.89	0.66	2.68	P	IN
QL01025	3,3,4,4,5,5,6,6,7,7,8,8,9,9,10,10,10-Heptadecafluoro-2-methyldecan-1-ol	136022-91-8	CC(CO)C(F)(F)C(F)(F)C(F)(F)C(F)(F)C(F)(F)C(F)(F)C(F)(F)C(F)(F)F		-8.04	0.67	2.51	P	IN
QL01026	1,1'-(Oxybis(propyleneoxy))bis(3-(perfluoro-7-methyloctyl)propan-2-ol)	93776-05-7	CC(COCC(O)CC(F)(F)C(F)(F)C(F)(F)C(F)(F)C(F)(F)C(F)(C(F)(F)F)C(F)(F)F)OC(C)COCC(O)CC(F)(F)C(F)(F)C(F)(F)C(F)(F)C(F)(F)C(F)(C(F)(F)F)C(F)(F)F		-10.14	0.66	3.76	P	OUT
QL01027	1,1'-(Oxybis((1-methylethylene)oxy))bis(3-(perfluoro-11-methyldodecyl)propan-2-ol)	93776-03-5	CC(COCC(O)CC(F)(F)C(F)(F)C(F)(F)C(F)(F)C(F)(F)C(F)(F)C(F)(F)C(F)(F)C(F)(F)C(F)(C(F)(F)F)C(F)(F)F)OC(C)COCC(O)CC(F)(F)C(F)(F)C(F)(F)C(F)(F)C(F)(F)C(F)(F)C(F)(F)C(F)(F)C(F)(F)C(F)(F)C(F)(C(F)(F)F)C(F)(F)F		-11.12	0.67	4.13	P	OUT

QL01028	1,1'-(Oxybis((1-methylethylene)oxy))bis(3-(perfluorododecyl)propan-2-ol)	93776-00-2	CC(COCC(O)CC(F)(F)C(F)(F)C(F)(F)C(F)(F)C(F)(F)C(F)(F)C(F)(F)C(F)(F)C(F)(F)C(F)(F)C(F)(F)C(F)(F)F)OC(C)COCC(O)CC(F)(F)C(F)(F)C(F)(F)C(F)(F)C(F)(F)C(F)(F)C(F)(F)C(F)(F)C(F)(F)C(F)(F)C(F)(F)C(F)(F)F	-11.21	0.67	4.18	P	OUT
QL01029	1,1'-(Oxybis(propyleneoxy))bis(3-(perfluorooctyl)propan-2-ol)	93776-02-4	CC(COCC(O)CC(F)(F)C(F)(F)C(F)(F)C(F)(F)C(F)(F)C(F)(F)C(F)(F)C(F)(F)F)OC(C)COCC(O)CC(F)(F)C(F)(F)C(F)(F)C(F)(F)C(F)(F)C(F)(F)C(F)(F)C(F)(F)F	-10.24	0.66	3.83	P	OUT
QL01030	Sulfonic acids, C6-8-alkane, perfluoro, compds. with polyethylene-polypropylene glycol bis(2-aminopropyl) ether	306974-45-8	CC(COCCOC(C)COCC(C)NS(=O)(=O)C(F)(F)C(F)(F)C(F)(F)C(F)(F)F)NS(=O)(=O)C(F)(F)C(F)(F)C(F)(F)C(F)(F)F	-9.16	0.65	3.50	P	OUT
QL01031	2-Propenoic acid, 3,3,4,4,5,5,6,6,7,7,8,8,9,9,10,10,11,11-octadecafluorododecyl ester	57678-90-7	CC(F)(F)C(F)(F)C(F)(F)C(F)(F)C(F)(F)C(F)(F)C(F)(F)C(F)(F)C(F)(F)CCOC(=O)C=C	-8.56	0.66	2.94	P	IN
QL01032	1,1,1,2,2,3,3,4,4-Nonafluoropentane	158118-01-5	CC(F)(F)C(F)(F)C(F)(F)C(F)(F)F	-6.18	0.68	1.45	P	IN
QL01033	Disulfides, bis(.gamma.-.omega.-perfluoro-C6-20-alkyl)	118400-71-8	CC(F)(F)C(F)(F)C(F)(F)CCSSCCC(F)(F)C(F)(F)C(C)(F)F	-7.53	0.65	2.60	P	IN
QL01034	1,1,1,2,2,3,3-heptafluorobutane	662-00-0	CC(F)(F)C(F)(F)C(F)(F)F	-5.80	0.68	1.32	P	IN
QL01035	6-(1,1-Difluoroethoxy)-1,1,1,2,2,3,3,4,4,5,5-undecafluorohexane	197963-86-3	CC(F)(F)OCC(F)(F)C(F)(F)C(F)(F)C(F)(F)C(F)(F)F	-7.39	0.67	2.23	P	IN
QL01036	4-(1,1-Difluoroethoxy)-1,1,1,2,2,3,3-heptafluorobutane	1365808-20-3	CC(F)(F)OCC(F)(F)C(F)(F)C(F)(F)F	-6.45	0.66	1.91	P	IN
QL01037	1,1,1,2,2,3,3,4,4,5,5,6,6,7,7,8,8,9,9,10,10-Henicosafluoro-12-iodotridecane	38550-36-6	CC(I)CC(F)(F)C(F)(F)C(F)(F)C(F)(F)C(F)(F)C(F)(F)C(F)(F)C(F)(F)C(F)(F)C(F)(F)F	-8.61	0.68	2.74	P	IN
QL01038	1,1,1,2,2,3,3,4,4,5,5,6,6,7,7,8,8-Heptadecafluoro-10-iodoundecane	38550-35-5	CC(I)CC(F)(F)C(F)(F)C(F)(F)C(F)(F)C(F)(F)C(F)(F)C(F)(F)C(F)(F)F	-8.04	0.68	2.51	P	IN
QL01039	1-(Perfluoroheptyl)-2-iodopropane	25291-12-7	CC(I)CC(F)(F)C(F)(F)C(F)(F)C(F)(F)C(F)(F)C(F)(F)C(F)(F)F	-7.84	0.67	2.45	P	IN
QL01040	1-(Perfluorohexyl)-2-iodopropane	38550-34-4	CC(I)CC(F)(F)C(F)(F)C(F)(F)C(F)(F)C(F)(F)C(F)(F)F	-7.33	0.67	2.24	P	IN
QL01041	1,1,1,2,2,3,3,4,4-Nonafluoro-6-iodoheptane	89889-25-8	CC(I)CC(F)(F)C(F)(F)C(F)(F)C(F)(F)F	-6.37	0.66	1.90	P	IN
QL01042	3,3,4,4,5,5,5-Heptafluoropentan-2-amine	1446355-42-5	CC(N)C(F)(F)C(F)(F)C(F)(F)F	-5.76	0.67	1.46	P	IN
QL01043	2-Amino-4,4,5,5,6,6,6-heptafluorohexan-3-ol	355-88-4	CC(N)C(O)C(F)(F)C(F)(F)C(F)(F)F	-6.21	0.66	1.83	P	IN
QL01044	2,2,3,3,4,4,4-Heptafluoro-N-(1-phenylethyl)butanamide	51241-61-3	CC(NC(=O)C(F)(F)C(F)(F)C(F)(F)F)C1=CC=CC=C1	-6.58	0.63	2.43	P	OUT
QL01045	2,3,3,3-Tetrafluoro-2-(heptafluoropropoxy)-N-(1-phenylethyl)propanamide	98873-61-1	CC(NC(=O)C(F)(OC(F)(F)C(F)(F)C(F)(F)F)C(F)(F)F)c1ccccc1	-7.40	0.64	2.68	P	OUT
QL01046	N'-[(2E)-5,5,6,6,7,7,7-Heptafluoro-4-oxohept-2-en-2-yl]-4-nitrobenzohydrazide	203201-05-2	CC(NNC(=O)c1ccc(cc1)[N+]([O-])=O)=C/C(=O)C(F)(F)C(F)(F)C(F)(F)F	-7.45	0.62	2.94	P	OUT
QL01047	N'-[(2Z)-5,5,6,6,7,7,8,8,9,9,10,10,11,11,12,12,12-Heptadecafluoro-4-oxododec-2-en-2-yl]benzohydrazide	203200-82-2	CC(NNC(=O)c1ccccc1)=CC(=O)C(F)(F)C(F)(F)C(F)(F)C(F)(F)C(F)(F)C(F)(F)C(F)(F)C(F)(F)F	-8.75	0.65	3.24	P	OUT
QL01048	N'-[(2Z)-5,5,6,6,7,7,7-Heptafluoro-4-oxohept-2-en-2-yl]benzohydrazide	203200-76-4	CC(NNC(=O)c1ccccc1)=CC(=O)C(F)(F)C(F)(F)C(F)(F)F	-6.89	0.62	2.72	P	OUT
QL01049	N-[3-(Dimethylamino)propyl]-perfluoro-1-hexanesulfonamide acetate	73772-33-5	CC(O)=O.CN(C)CCCNS(=O)(=O)C(F)(F)C(F)(F)C(F)(F)C(F)(F)C(F)(F)C(F)(F)F	-8.05	0.64	3.05	P	OUT
QL01050	Acetic acid--4-(heptafluoropropyl)phenol (1/1)	61324-27-4	CC(O)=O.OC1=CC=C(C=C1)C(F)(F)C(F)(F)C(F)(F)F	-6.63	0.62	2.62	P	OUT
QL01051	3,3,4,4,5,5,6,6,7,7,8,8,8-Tridecafluorooctan-2-ol	64773-44-0	CC(O)C(F)(F)C(F)(F)C(F)(F)C(F)(F)C(F)(F)C(F)(F)F	-7.50	0.68	2.16	P	IN
QL01052	1H,1H,1H,2H-Perfluoro-2-heptanol	914637-05-1	CC(O)C(F)(F)C(F)(F)C(F)(F)C(F)(F)C(F)(F)F	-6.91	0.68	1.90	P	IN
QL01053	1-Perfluoropropylethanol	375-14-4	CC(O)C(F)(F)C(F)(F)C(F)(F)F	-5.76	0.67	1.46	P	IN
QL01054	1-Pentafluoroethylethanol	374-40-3	CC(O)C(F)(F)C(F)(F)F	-5.29	0.66	1.36	P	IN
QL01055	3,3,4,5,5,6,6,7,7,7-Decafluoroheptan-2-ol	7618-17-9	CC(O)C(F)(F)C(F)C(F)(F)C(F)(F)C(F)(F)F	-6.63	0.67	1.91	P	IN
QL01056	3,3,4,4-Tetrafluoro-2-butanol	17425-25-1	CC(O)C(F)(F)C(F)F	-4.61	0.63	1.39	P	OUT
QL01057	4,4,5,5,6,6,7,7,8,9,9,9-Dodecafluoro-2-(2-hydroxypropyl)-8-(trifluoromethyl)non-2-enoate	16083-75-3	CC(O)CC(=CC(F)(F)C(F)(F)C(F)(F)C(F)(F)C(F)(C(F)(F)F)C(F)(F)F)C([O-])=O	-7.49	0.66	2.48	P	IN
QL01058	4(perfluorooctyl)-2-butanol	149868-74-6	CC(O)CCC(F)(F)C(F)(F)C(F)(F)C(F)(F)C(F)(F)C(F)(F)C(F)(F)C(F)(F)F	-8.27	0.67	2.69	P	IN
QL01059	2,2,3,3,4,4,5,5,6,6,7,7,8,8,8-Pentadecafluoro-N-(2-hydroxypropyl)octanamide	1407514-44-6	CC(O)CNC(=O)C(F)(F)C(F)(F)C(F)(F)C(F)(F)C(F)(F)C(F)(F)C(F)(F)F	-8.10	0.66	2.69	P	IN
QL01060	3,3-Dimethylbutan-2-yl 2,3,3,3-tetrafluoro-2-[1,1,2,3,3,3-hexafluoro-2-(heptafluoropropoxy)propoxy]propanoate	107368-63-8	CC(OC(=O)C(F)(OC(F)(F)C(F)(OC(F)(F)C(F)(F)C(F)(F)F)C(F)(F)F)C(F)(F)F)C(C)(C)C	-8.25	0.67	2.69	P	IN
QL01061	1-(2,2,3,3,4,4,4-Heptafluorobutoxy)ethyl acetate	378-08-5	CC(OCC(F)(F)C(F)(F)C(F)(F)F)OC(C)=O	-6.26	0.63	2.21	P	OUT
QL01062	1,1'-(Oxybis((2-methylethylene)oxy))bis(3-(perfluoro-9-methyldecyl)propan-2-ol)	93776-04-6	CC(OCC(O)CC(F)(F)C(F)(F)C(F)(F)C(F)(F)C(F)(F)C(F)(F)C(F)(F)C(F)(F)C(C(F)(F)F)(F)C(F)(F)F)COCC(OCC(O)CC(F)(F)C(F)(F)C(F)(F)C(F)(F)C(F)(F)C(F)(F)C(F)(F)C(F)(F)C(C(F)(F)F)(F)C(F)(F)F)C	-10.68	0.67	3.96	P	OUT

QL01063	1,1'-[Oxybis[(1-methyl-2,1-ethanediyl)oxy]]bis[3-(perfluorodecyl)propan-2-ol]	93776-01-3	CC(OCC(O)CC(F)(F)C(F)(F)C(F)(F)C(F)(F)C(F)(F)C(F)(F)C(F)(F)C(F)(F)C(F)(F)C(F)(F)F)COCC(OCC(O)CC(F)(F)C(F)(F)C(F)(F)C(F)(F)C(F)(F)C(F)(F)C(F)(F)C(F)(F)C(F)(F)C(F)(F)F)C		-10.78	0.67	4.01	P	OUT
QL01064	1,1,1-Trifluoropropan-2-yl nonafluorobutane-1-sulfonate	118334-94-4	CC(OS(=O)(=O)C(F)(F)C(F)(F)C(F)(F)C(F)(F)F)C(F)(F)F		-7.49	0.67	2.24	P	IN
QL01065	2,2,3,3,4,4,5,5,6,6,7,7,8,8,8-Pentadecafluorooctyl 4'-[(2S)-2-methylbutyl][1,1'-biphenyl]-4-carboxylate	118826-52-1	CC[C@H](C)Cc1ccc(cc1)-c1ccc(cc1)C(=O)OCC(F)(F)C(F)(F)C(F)(F)C(F)(F)C(F)(F)C(F)(F)C(F)(F)F		-8.88	0.64	3.48	P	OUT
QL01066	N,N,N,-Triethylethanaminium 1,1,2,2,3,3,4,4,4-nonafluorobutane-1-sulfonate	25628-08-4	CC[N+](CC)(CC)C(C)OS(=O)(=O)C(F)(F)C(F)(F)C(F)(F)C(F)(F)F		-7.11	0.65	2.45	P	IN
QL01067	N,N,N-Triethylethanaminium pentadecafluorooctanoate	98241-25-9	CC[N+](CC)(CC)CC.[O-]C(=O)C(F)(F)C(F)(F)C(F)(F)C(F)(F)C(F)(F)C(F)(F)C(F)(F)F		-7.91	0.65	2.85	P	IN
QL01068	Tetraethylammonium perfluoroisooctanesulfonate	93894-70-3	CC[N+](CC)(CC)CC.[O-]S(=O)(=O)C(F)(F)C(F)(F)C(F)(F)C(F)(F)C(F)(F)C(F)(C(F)(F)F)C(F)(F)F		-8.05	0.65	2.80	P	IN
QL01069	tetraethylammonium heptadecafluorooctanesulphonate	56773-42-3	CC[N+](CC)(CC)CC.[O-]S(=O)(=O)C(F)(F)C(F)(F)C(F)(F)C(F)(F)C(F)(F)C(F)(F)C(F)(F)C(F)(F)F		-8.47	0.66	2.98	P	IN
QL01070	1-Ethyl-3-methylpyridin-1-ium nonafluorobutane-1-sulfonate	1015420-87-7	CC[n+]1cccc(C)c1.[O-]S(=O)(=O)C(F)(F)C(F)(F)C(F)(F)C(F)(F)F		-5.83	0.64	1.90	P	IN
QL01071	N,N-Diethylethanaminium heptadecafluorononanoate	327176-80-7	CC[NH+](CC)CC.[O-]C(=O)C(F)(F)C(F)(F)C(F)(F)C(F)(F)C(F)(F)C(F)(F)C(F)(F)C(F)(F)F		-8.18	0.65	2.97	P	IN
QL01072	Triethylaminium perfluoro-2-propoxypropanoate	165951-18-8	CC[NH+](CC)CC.[O-]C(=O)C(F)(OC(F)(F)C(F)(F)C(F)(F)F)C(F)(F)F		-6.44	0.63	2.39	P	OUT
QL01073	Triethylaminium perfluoro-2-(2-propoxypropoxy)propanoate	165951-19-9	CC[NH+](CC)CC.[O-]C(=O)C(F)(OC(F)(F)C(F)(OC(F)(F)C(F)(F)C(F)(F)F)C(F)(F)F)C(F)(F)F		-7.90	0.64	2.92	P	OUT
QL01074	N,N-diethylethanaminium ((perfluorooctyl)ethyl)phosphonate (11)	92678-96-1	CC[NH+](CC)CC.OP([O-])(=O)CCC(F)(F)C(F)(F)C(F)(F)C(F)(F)C(F)(F)C(F)(F)C(F)(F)C(F)(F)F		-8.49	0.65	3.14	P	OUT
QL01075	Perfluoro-11-methyldodecanoic acid compd. with ethanamine	68015-87-2	CC[NH3+].[O-]C(=O)C(F)(F)C(F)(F)C(F)(F)C(F)(F)C(F)(F)C(F)(F)C(F)(F)C(F)(F)C(F)(F)C(F)(C(F)(F)F)C(F)(F)F		-8.60	0.66	2.97	P	IN
QL01076	Perfluoro-13-methyltetradecanoic acid compd. with ethanamine	68052-68-6	CC[NH3+].[O-]C(=O)C(F)(F)C(F)(F)C(F)(F)C(F)(F)C(F)(F)C(F)(F)C(F)(F)C(F)(F)C(F)(F)C(F)(F)C(F)(F)C(F)(C(F)(F)F)C(F)(F)F		-9.07	0.67	3.14	P	OUT
QL01077	(2S)-2-{Bis[4-(heptadecafluorooctyl)phenyl][(triethylsilyl)oxy]methyl}pyrrolidine	914391-37-0	CC[Si](CC)(CC)OC([C@@H]1CCCN1)(c1ccc(cc1)C(F)(F)C(F)(F)C(F)(F)C(F)(F)C(F)(F)C(F)(F)C(F)(F)C(F)(F)F)c1ccc(cc1)C(F)(F)C(F)(F)C(F)(F)C(F)(F)C(F)(F)C(F)(F)C(F)(F)C(F)(F)F		-9.89	0.66	3.70	P	OUT
QL01078	[(4-{3-[(3,3,4,4,5,5,6,6,7,7,8,8,8-Tridecafluorooctyl)sulfanyl]propyl}-1,2-phenylene)bis(oxy)]bis(triethylsilane)	1637311-39-7	CC[Si](CC)(CC)Oc1ccc(CCCSCCC(F)(F)C(F)(F)C(F)(F)C(F)(F)C(F)(F)C(F)(F)F)cc1O[Si](CC)(CC)CC		-7.78	0.61	3.23	P	OUT
QL01079	1,1,1,2-Tetrafluoro-2-(trifluoromethyl)hex-4-en-3-one	184711-88-4	CC=CC(=O)C(F)(C(F)(F)F)C(F)(F)F		-5.30	0.63	1.75	P	OUT
QL01080	4,4,5,5,6,6,7,7,8,8,9,9,10,10,11,11,11-Heptadecafluoroundec-2-ene	67103-04-2	CC=CC(F)(F)C(F)(F)C(F)(F)C(F)(F)C(F)(F)C(F)(F)C(F)(F)C(F)(F)F		-7.91	0.67	2.50	P	IN
QL01081	(2E)-4,4,5,5,6,6,7,7,8,8,9,9,10,10,10-Pentadecafluorodec-2-ene	89889-22-5	CC=CC(F)(F)C(F)(F)C(F)(F)C(F)(F)C(F)(F)C(F)(F)C(F)(F)F		-7.69	0.67	2.44	P	IN
QL01083	4,4,5,5,6,6,7,7,8,8,9,9,9-Tridecafluoro-2-nonene	63967-46-4	CC=CC(F)(F)C(F)(F)C(F)(F)C(F)(F)C(F)(F)C(F)(F)F		-7.16	0.66	2.23	P	IN
QL01085	1-Propenylperfluoropropane	355-95-3	CC=CC(F)(F)C(F)(F)C(F)(F)F		-5.71	0.64	1.85	P	IN
QL01086	1,1,1,2,2,3,3,4,4-Nonafluoro-4-(prop-1-ene-1-sulfonyl)butane	66406-80-2	CC=CS(=O)(=O)C(F)(F)C(F)(F)C(F)(F)C(F)(F)F		-6.87	0.66	2.17	P	IN
QL01087	Bis-(1H,1H,7H-perfluoroheptyl) (1S,3R)-camphorate	2355-57-9	CC1(C)[C@@H](CC[C@]1(C)C(=O)OCC(F)(F)C(F)(F)C(F)(F)C(F)(F)C(F)(F)C(F)F)C(=O)OCC(F)(F)C(F)(F)C(F)(F)C(F)(F)C(F)(F)C(F)F		-9.62	0.67	3.43	P	OUT
QL01088	Bicyclo[2.2.1]heptan-2-one, 3-(2,2,3,3,4,4,4-heptafluoro-1-hydroxybutylidene)-1,7,7-trimethyl-, (1R,4S)-	63180-06-3	CC1(C)[C@@H]2CC[C@@]1(C)C(=O)C2=C(O)C(F)(F)C(F)(F)C(F)(F)F		-7.12	0.67	2.10	P	IN
QL01089	(1S,4S)-3-(Heptafluorobutyryl)camphor	115224-00-5	CC1(C)[C@H]2CC[C@]1(C)C(=O)C2C(=O)C(F)(F)C(F)(F)C(F)(F)F		-7.12	0.67	2.10	P	IN
QL01090	N'-(3,3-Dimethyl-3,4-dihydroisoquinolin-1-yl)-2,2,3,3,4,4,4-heptafluorobutanehydrazide	304435-60-7	CC1(C)Cc2ccccc2C(NNC(=O)C(F)(F)C(F)(F)C(F)(F)F)=N1		-6.74	0.63	2.41	P	OUT
QL01091	4,4,5,5-Tetramethyl-2-[5-(tridecafluorohexyl)thiophen-2-yl]-1,3,2-dioxaborolane	1338083-27-4	CC1(C)OB(OC1(C)C)c1ccc(s1)C(F)(F)C(F)(F)C(F)(F)C(F)(F)C(F)(F)C(F)(F)F		-8.42	0.67	2.74	P	IN

QL01092	4-(2,2,3,3,4,4,5,5,6,6,7,7,8,8,9,9,9-Heptadecafluorononyl)-2,2-dimethyl-1,3-dioxolane	94159-88-3	CC1(C)OCC(CC(F)(F)C(F)(F)C(F)(F)C(F)(F)C(F)(F)C(F)(F)C(F)(F)C(F)(F)F)O1		-8.53	0.68	2.75	P	IN
QL01093	1-Methyl-N-(1,1,2,2,3,3,4,4,4-nonafluorobutane-1-sulfonyl)cyclopentane-1-carboxamide	1440968-23-9	CC1(CCCC1)C(=O)NS(=O)(=O)C(F)(F)C(F)(F)C(F)(F)C(F)(F)F		-7.54	0.66	2.45	P	IN
QL01094	3-[(2,2,3,3,4,4,4-Heptafluorobutoxy)methyl]-3-methyloxetane	156294-55-2	CC1(COCC(F)(F)C(F)(F)C(F)(F)F)COC1		-6.62	0.65	2.20	P	IN
QL01095	Oxetane, 3-methyl-3-[[(3,3,4,4,5,5,6,6,6-nonafluorohexyl)oxy]methyl]-	475678-78-5	CC1(COCCC(F)(F)C(F)(F)C(F)(F)C(F)(F)F)COC1		-7.20	0.65	2.48	P	IN
QL01096	4-(2,2,3,3,4,4,4-Heptafluorobutoxy)-1,2-dimethylpyridin-1-ium	140160-19-6	CC1=[N+](C)C=CC(OCC(F)(F)C(F)(F)C(F)(F)F)=C1		-6.83	0.64	2.42	P	IN
QL01097	3-(Heptafluoropropyl)-5-methyl-4-nitro-1H-pyrazole	82633-69-0	CC1=C(C(=NN1)C(F)(F)C(F)(F)C(F)(F)F)[N+]([O-])=O		-6.04	0.65	1.90	P	IN
QL01098	5-(Heptafluoropropyl)-6-methylpyrimidine-2,4(1H,3H)-dione	145663-05-4	CC1=C(C(=O)NC(=O)N1)C(F)(F)C(F)(F)C(F)(F)F		-6.47	0.65	2.12	P	IN
QL01099	3,3'-(Perfluoro-1-cyclopentene-1,2-diyl)bis(2,5-dimethylthiophene)	190394-25-3	CC1=CC(=C(C)S1)C1=C(C2=C(C)SC(C)=C2)C(F)(F)C(F)(F)C1(F)F		-6.21	0.64	2.07	P	IN
QL01100	3-(Perfluoro-1-hexyl)-5-(methyl)pyrazole	129257-23-4	CC1=CC(=NN1)C(F)(F)C(F)(F)C(F)(F)C(F)(F)C(F)(F)C(F)(F)F		-7.73	0.67	2.45	P	IN
QL01101	3-(Perfluoropentyl)-5-methylpyrazole	82633-43-0	CC1=CC(=NN1)C(F)(F)C(F)(F)C(F)(F)C(F)(F)C(F)(F)F		-7.20	0.66	2.24	P	IN
QL01102	3-(Heptafluoropropyl)-5-methyl-1H-pyrazole	75823-64-2	CC1=CC(=NN1)C(F)(F)C(F)(F)C(F)(F)F		-6.19	0.65	1.92	P	IN
QL01103	Diphenyl(2,4,6-trimethylphenyl)sulfonium perfluoro-1-octanesulfonate	258341-99-0	CC1=CC(C)=C([S+](C2=CC=CC=C2)C2=CC=CC=C2)C(C)=C1.[O-]S(=O)(=O)C(F)(F)C(F)(F)C(F)(F)C(F)(F)C(F)(F)C(F)(F)C(F)(F)C(F)(F)F		-6.98	0.64	2.45	P	IN
QL01104	2,2,3,3,4,4,5,5,5-Nonafluoro-1-(4-methylphenyl)pentan-1-one	647-60-9	CC1=CC=C(C=C1)C(=O)C(F)(F)C(F)(F)C(F)(F)C(F)(F)F		-7.21	0.65	2.46	P	IN
QL01105	2,2,3,3,4,4,4-Heptafluoro-1-(4-methylphenyl)butan-1-one	559-92-2	CC1=CC=C(C=C1)C(=O)C(F)(F)C(F)(F)C(F)(F)F		-6.61	0.64	2.27	P	IN
QL01106	4-(Heptafluoroisopropyl)toluene	2396-26-1	CC1=CC=C(C=C1)C(F)(C(F)(F)F)C(F)(F)F		-5.86	0.64	1.90	P	IN
QL01108	2,2,3,3,4,4,5,5,6,6,7,7,8,8,8-Pentadecafluorooctyl 4-methylbenzene-1-sulfonate	24962-65-0	CC1=CC=C(C=C1)S(=O)(=O)OCC(F)(F)C(F)(F)C(F)(F)C(F)(F)C(F)(F)C(F)(F)C(F)(F)F		-8.56	0.66	2.95	P	IN
QL01109	1H,1H,7H-Perfluoroheptyl 4-methylbenzenesulfonate	424-16-8	CC1=CC=C(C=C1)S(=O)(=O)OCC(F)(F)C(F)(F)C(F)(F)C(F)(F)C(F)(F)C(F)F		-8.07	0.65	2.86	P	IN
QL01110	1h,1h-perfluorohexyl p-toluenesulfonate	355-77-1	CC1=CC=C(C=C1)S(=O)(=O)OCC(F)(F)C(F)(F)C(F)(F)C(F)(F)C(F)(F)F		-7.87	0.65	2.71	P	IN
QL01111	1h,1h-nonafluoropentyl p-toluenesulfonate	883499-79-4	CC1=CC=C(C=C1)S(=O)(=O)OCC(F)(F)C(F)(F)C(F)(F)C(F)(F)F		-7.66	0.65	2.68	P	IN
QL01112	2,2,3,3,4,4,4-Heptafluorobutyl 4-methylbenzene-1-sulfonate	312-66-3	CC1=CC=C(C=C1)S(=O)(=O)OCC(F)(F)C(F)(F)C(F)(F)F		-7.19	0.64	2.53	P	IN
QL01113	2,2,3,3,3-Pentafluoropropyl 4-methylbenzene-1-sulfonate	565-42-4	CC1=CC=C(C=C1)S(=O)(=O)OCC(F)(F)C(F)(F)F		-6.84	0.63	2.48	P	OUT
QL01114	1,3-Diethyl-2-methyl-5-[(perfluorohexyl)sulfonyl]-1H-benzimidazolium 4-methylbenzenesulfonate	76848-68-5	CC1=CC=C(C=C1)S(O)(=O)=O.CCN1C(C)[NH+](CC)C2=C1C=CC(=C2)S(=O)(=O)C(F)(F)C(F)(F)C(F)(F)C(F)(F)C(F)(F)C(F)(F)F		-8.96	0.64	3.46	P	OUT
QL01115	1-Methyl-2-(nonafluorobutyl)benzene	93697-09-7	CC1=CC=CC=C1C(F)(F)C(F)(F)C(F)(F)C(F)(F)F		-6.64	0.65	2.17	P	IN
QL01116	(2-methylphenyl) 1,1,2,2,3,3,4,4,4-nonafluorobutane-1-sulfonate	42096-33-3	CC1=CC=CC=C1OS(=O)(=O)C(F)(F)C(F)(F)C(F)(F)C(F)(F)F		-7.28	0.65	2.45	P	IN
QL01117	6-Methyl-2-(undecafluoropentyl)-1,3-benzoxazole	93416-26-3	CC1=CC2=C(C=C1)N=C(O2)C(F)(F)C(F)(F)C(F)(F)C(F)(F)C(F)(F)F		-7.57	0.66	2.53	P	IN
QL01118	(4-Bromophenyl){5-[difluoro(trifluoromethoxy)methyl]-5-hydroxy-3-methyl-4,5-dihydro-1H-pyrazol-1-yl}methanone	722470-55-5	CC1=NN(C(=O)c2ccc(Br)cc2)C(O)(C1)C(F)(F)OC(F)(F)F		-6.50	0.62	2.46	P	OUT
QL01119	Orthobutyric acid, heptafluoro-, cyclic ester with 2-(hydroxymethyl)-2-methyl-1,3-propanediol (11)	31185-67-8	CC12COC(OC1)(OC2)C(F)(F)C(F)(F)C(F)(F)F		-7.13	0.67	2.17	P	IN
QL01120	4-(1,1,1,2,3,3,3-Heptafluoropropan-2-yl)-2-methyl-3-(2-methylpropane-2-sulfonyl)benzoic acid	1355554-59-4	Cc1c(ccc(c1S(=O)(=O)C(C)(C)C)C(F)(C(F)(F)F)C(F)(F)F)C(O)=O		-6.70	0.65	2.11	P	IN
QL01121	4-(1,1,1,2,3,3,3-Heptafluoropropan-2-yl)-2-methyl-3-(2-methylpropane-2-sulfinyl)benzoic acid	1355554-39-0	Cc1c(ccc(c1S(=O)C(C)(C)C)C(F)(C(F)(F)F)C(F)(F)F)C(O)=O		-6.46	0.65	2.12	P	IN
QL01122	4-(1,1,1,2,3,3,3-Heptafluoropropan-2-yl)-3-(methanesulfonyl)-2-methylbenzoic acid	1355554-41-4	Cc1c(ccc(c1S(C)(=O)=O)C(F)(C(F)(F)F)C(F)(F)F)C(O)=O		-6.63	0.65	2.19	P	IN
QL01123	4-(1,1,1,2,3,3,3-Heptafluoropropan-2-yl)-3-(methanesulfinyl)-2-methylbenzoic acid	1355554-19-6	Cc1c(ccc(c1S(C)=O)C(F)(C(F)(F)F)C(F)(F)F)C(O)=O		-6.36	0.64	2.19	P	IN
QL01124	3-(tert-Butylsulfanyl)-4-(1,1,1,2,3,3,3-heptafluoropropan-2-yl)-2-methylbenzoic acid	1355554-16-3	Cc1c(ccc(c1SC(C)(C)C)C(F)(C(F)(F)F)C(F)(F)F)C(O)=O		-6.33	0.64	2.11	P	IN
QL01125	3-Methyl-2-(nonafluorobutyl)-1H-indole	1622133-36-1	Cc1c(Nc2ccccc12)C(F)(F)C(F)(F)C(F)(F)C(F)(F)F		-6.78	0.65	2.25	P	IN

QL01126	2-(Heptafluoropropyl)-3-methyl-1H-indole	1030606-42-8	Cc1c(Nc2ccccc12)C(F)(F)C(F)(F)C(F)(F)F		-6.37	0.64	2.15	P	IN
QL01127	5-Methyl-6-[(3,3,4,4,5,5,6,6,7,7,8,8,8-tridecafluorooctyl)sulfanyl]hexahydro-4,7-methano-2-benzofuran-1,3-dione	94413-32-8	CC1C2CC(C1SCCC(F)(F)C(F)(F)C(F)(F)C(F)(F)C(F)(F)C(F)(F)F)C1C2C(=O)OC1=O		-8.26	0.66	2.89	P	IN
QL01128	6-[(4,6-Dimethylpyrimidin-2-yl)sulfanyl]-3-(heptafluoropropyl)[1,2,4]triazolo[4,3-b]pyridazine	351346-36-6	Cc1cc(C)nc(Sc2ccc3nnc(n3n2)C(F)(F)C(F)(F)C(F)(F)F)n1		-7.07	0.63	2.68	P	OUT
QL01129	2-Bromo-4-(1,1,1,2,3,3,3-heptafluoropropan-2-yl)-6-methylaniline	1770825-71-2	Cc1cc(cc(Br)c1N)C(F)(C(F)(F)F)C(F)(F)F		-6.09	0.65	1.90	P	IN
QL01130	2-Bromo-5-(1,1,1,2,3,3,3-heptafluoropropan-2-yl)-1,3-dimethylbenzene	353272-55-6	Cc1cc(cc(C)c1Br)C(F)(C(F)(F)F)C(F)(F)F		-6.09	0.65	1.90	P	IN
QL01131	4-(1,1,1,2,3,3,3-Heptafluoropropan-2-yl)-2,6-dimethylbenzaldehyde	1771743-29-3	Cc1cc(cc(C)c1C=O)C(F)(C(F)(F)F)C(F)(F)F		-6.32	0.63	2.20	P	IN
QL01132	4-(1,1,1,2,3,3,3-Heptafluoropropan-2-yl)-2,6-dimethylaniline	273735-43-6	Cc1cc(cc(C)c1N)C(F)(C(F)(F)F)C(F)(F)F		-6.09	0.65	1.90	P	IN
QL01133	3-Benzamido-2,6-difluoro-N-[4-(1,1,1,2,3,3,3-heptafluoropropan-2-yl)-2,6-dimethylphenyl]benzamide	862133-36-6	Cc1cc(cc(C)c1NC(=O)c1c(F)ccc(NC(=O)c2ccccc2)c1F)C(F)(C(F)(F)F)C(F)(F)F		-7.60	0.62	3.09	P	OUT
QL01134	5-Benzamido-2,4-dibromo-N-[4-(1,1,1,2,3,3,3-heptafluoropropan-2-yl)-2,6-dimethylphenyl]benzamide	862133-37-7	Cc1cc(cc(C)c1NC(=O)c1cc(NC(=O)c2ccccc2)c(Br)cc1Br)C(F)(C(F)(F)F)C(F)(F)F		-7.60	0.62	3.08	P	OUT
QL01135	2,4-Difluoro-N-(2-fluoro-5-{[4-(1,1,1,2,3,3,3-heptafluoropropan-2-yl)-2,6-dimethylphenyl]carbamoyl}phenyl)benzamide	862133-29-7	Cc1cc(cc(C)c1NC(=O)c1ccc(F)c(NC(=O)c2ccc(F)cc2F)c1)C(F)(C(F)(F)F)C(F)(F)F		-7.93	0.62	3.20	P	OUT
QL01136	2,3,4,5,6-Pentafluoro-N-(3-{[4-(1,1,1,2,3,3,3-heptafluoropropan-2-yl)-2,6-dimethylphenyl]carbamoyl}phenyl)benzamide	862130-96-9	Cc1cc(cc(C)c1NC(=O)c1cccc(NC(=O)c2c(F)c(F)c(F)c(F)c2F)c1)C(F)(C(F)(F)F)C(F)(F)F		-7.99	0.63	3.18	P	OUT
QL01137	2,3,6-Trifluoro-N-(3-{[4-(1,1,1,2,3,3,3-heptafluoropropan-2-yl)-2,6-dimethylphenyl]carbamoyl}phenyl)benzamide	862130-95-8	Cc1cc(cc(C)c1NC(=O)c1cccc(NC(=O)c2c(F)ccc(F)c2F)c1)C(F)(C(F)(F)F)C(F)(F)F		-7.60	0.62	3.04	P	OUT
QL01138	2,5-Difluoro-N-(3-{[4-(1,1,1,2,3,3,3-heptafluoropropan-2-yl)-2,6-dimethylphenyl]carbamoyl}phenyl)benzamide	862130-67-4	Cc1cc(cc(C)c1NC(=O)c1cccc(NC(=O)c2cc(F)ccc2F)c1)C(F)(C(F)(F)F)C(F)(F)F		-7.56	0.62	3.05	P	OUT
QL01139	4-Chloro-N-(3-{[4-(1,1,1,2,3,3,3-heptafluoropropan-2-yl)-2,6-dimethylphenyl]carbamoyl}phenyl)-2-nitrobenzamide	862130-91-4	Cc1cc(cc(C)c1NC(=O)c1cccc(NC(=O)c2ccc(Cl)cc2[N+]([O-])=O)c1)C(F)(C(F)(F)F)C(F)(F)F		-7.78	0.62	3.18	P	OUT
QL01140	3-(4-Chloro-2-nitrobenzamido)-2-fluoro-N-[4-(1,1,1,2,3,3,3-heptafluoropropan-2-yl)-2,6-dimethylphenyl]benzamide	862133-18-4	Cc1cc(cc(C)c1NC(=O)c1cccc(NC(=O)c2ccc(Cl)cc2[N+]([O-])=O)c1F)C(F)(C(F)(F)F)C(F)(F)F		-7.83	0.62	3.18	P	OUT
QL01141	2,4-Dichloro-N-(3-{[4-(1,1,1,2,3,3,3-heptafluoropropan-2-yl)-2,6-dimethylphenyl]carbamoyl}phenyl)benzamide	862130-69-6	Cc1cc(cc(C)c1NC(=O)c1cccc(NC(=O)c2ccc(Cl)cc2Cl)c1)C(F)(C(F)(F)F)C(F)(F)F		-7.83	0.62	3.19	P	OUT
QL01142	4-Chloro-2-fluoro-N-(3-{[4-(1,1,1,2,3,3,3-heptafluoropropan-2-yl)-2,6-dimethylphenyl]carbamoyl}phenyl)benzamide	862130-90-3	Cc1cc(cc(C)c1NC(=O)c1cccc(NC(=O)c2ccc(Cl)cc2F)c1)C(F)(C(F)(F)F)C(F)(F)F		-7.83	0.62	3.19	P	OUT
QL01143	3-(4-Chloro-2-fluorobenzamido)-2-fluoro-N-[4-(1,1,1,2,3,3,3-heptafluoropropan-2-yl)-2,6-dimethylphenyl]benzamide	862133-16-2	Cc1cc(cc(C)c1NC(=O)c1cccc(NC(=O)c2ccc(Cl)cc2F)c1F)C(F)(C(F)(F)F)C(F)(F)F		-7.88	0.62	3.19	P	OUT
QL01144	2-Fluoro-3-(4-fluoro-2-nitrobenzamido)-N-[4-(1,1,1,2,3,3,3-heptafluoropropan-2-yl)-2,6-dimethylphenyl]benzamide	862133-14-0	Cc1cc(cc(C)c1NC(=O)c1cccc(NC(=O)c2ccc(F)cc2[N+]([O-])=O)c1F)C(F)(C(F)(F)F)C(F)(F)F		-7.83	0.62	3.18	P	OUT
QL01145	2-Chloro-4-fluoro-N-(3-{[4-(1,1,1,2,3,3,3-heptafluoropropan-2-yl)-2,6-dimethylphenyl]carbamoyl}phenyl)benzamide	862130-87-8	Cc1cc(cc(C)c1NC(=O)c1cccc(NC(=O)c2ccc(F)cc2Cl)c1)C(F)(C(F)(F)F)C(F)(F)F		-7.83	0.62	3.19	P	OUT
QL01146	3-(2-Chloro-4-fluorobenzamido)-2-fluoro-N-[4-(1,1,1,2,3,3,3-heptafluoropropan-2-yl)-2,6-dimethylphenyl]benzamide	862133-15-1	Cc1cc(cc(C)c1NC(=O)c1cccc(NC(=O)c2ccc(F)cc2Cl)c1F)C(F)(C(F)(F)F)C(F)(F)F		-7.88	0.62	3.19	P	OUT
QL01147	2,4-Difluoro-N-(3-{[4-(1,1,1,2,3,3,3-heptafluoropropan-2-yl)-2,6-dimethylphenyl]carbamoyl}phenyl)benzamide	862130-66-3	Cc1cc(cc(C)c1NC(=O)c1cccc(NC(=O)c2ccc(F)cc2F)c1)C(F)(C(F)(F)F)C(F)(F)F		-7.83	0.62	3.19	P	OUT
QL01148	3-methyl-4-(2,2,3,3,4,4,5,5,5-nonafluoropentyl)cyclopentane-1,1-dicarboxylic Acid	20116-32-9	CC1CC(CC1CC(F)(F)C(F)(F)C(F)(F)C(F)(F)F)(C(O)=O)C(O)=O		-7.23	0.65	2.38	P	IN
QL01149	4-(1,1,1,2,3,3,3-Heptafluoropropan-2-yl)-2-methyl-1-nitrobenzene	238098-38-9	Cc1cc(ccc1[N+]([O-])=O)C(F)(C(F)(F)F)C(F)(F)F		-6.35	0.64	2.15	P	IN

QL01150	1-Bromo-4-(1,1,1,2,3,3,3-heptafluoropropan-2-yl)-2-methylbenzene	353272-53-4	Cc1cc(ccc1Br)C(F)(C(F)(F)F)C(F)(F)F		-6.01	0.64	1.90	P	IN
QL01151	2-[4-(1,1,1,2,3,3,3-Heptafluoropropan-2-yl)-2-methylphenyl]-4-iodo-1H-isoindole-1,3(2H)-dione	346575-66-4	Cc1cc(ccc1N1C(=O)c2cccc(I)c2C1=O)C(F)(C(F)(F)F)C(F)(F)F		-6.81	0.63	2.47	P	OUT
QL01152	Flubendiamide	272451-65-7	Cc1cc(ccc1NC(=O)c1cccc(I)c1C(O)=NC(C)(C)CS(C)(=O)=O)C(F)(C(F)(F)F)C(F)(F)F		-7.64	0.63	2.95	P	OUT
QL01153	N-(2-Methylsulfinyl-1,1-dimethyl-ethyl)-N'-{2-methyl-4-[perfluoroisopropyl]phenyl}phthalamide	371771-07-2	Cc1cc(ccc1NC(=O)c1ccccc1C(=O)NC(C)(C)CS(C)=O)C(F)(C(F)(F)F)C(F)(F)F		-7.44	0.62	2.99	P	OUT
QL01154	4-(Heptafluoropropyl)-7,8-dimethyl-1,3-dihydro-2H-1,5-benzodiazepin-2-one	505063-74-1	Cc1cc2NC(=O)CC(=Nc2cc1C)C(F)(F)C(F)(F)C(F)(F)F		-6.97	0.64	2.47	P	IN
QL01155	2-(1,1,1,2,3,3,3-Heptafluoropropan-2-yl)-5-methylcyclohexan-1-ol	52450-55-2	CC1CCC(C(O)C1)C(F)(C(F)(F)F)C(F)(F)F		-5.97	0.64	1.93	P	IN
QL01156	1,4-Dimethyl-2-(tridecafluorohexyl)benzene	123524-58-3	Cc1ccc(C)c(c1)C(F)(F)C(F)(F)C(F)(F)C(F)(F)C(F)(F)C(F)(F)F		-7.59	0.66	2.45	P	IN
QL01157	2,2,3,3,4,4,5,5,6,6,7,7,7-Tridecafluoro-N'-[(4-methylphenyl)methylidene]heptanehydrazide	445258-02-6	Cc1ccc(C=NNC(=O)C(F)(F)C(F)(F)C(F)(F)C(F)(F)C(F)(F)C(F)(F)F)cc1		-8.13	0.65	2.96	P	IN
QL01158	1-(Heptadecafluorooctyl)-4-methylbenzene	82396-51-8	Cc1ccc(cc1)C(F)(F)C(F)(F)C(F)(F)C(F)(F)C(F)(F)C(F)(F)C(F)(F)C(F)(F)F		-8.35	0.67	2.74	P	IN
QL01159	1-Methyl-4-(tridecafluorohexyl)benzene	128720-26-3	Cc1ccc(cc1)C(F)(F)C(F)(F)C(F)(F)C(F)(F)C(F)(F)C(F)(F)F		-7.72	0.66	2.52	P	IN
QL01160	1-(3,3,4,4,5,5,6,6,7,7,8,8,9,9,10,10,10-heptadecafluorodecyl)pyridinium toluene-p-sulphonate	61798-68-3	Cc1ccc(cc1)S(=O)(=O)[O-].FC(F)(F)C(F)(F)C(F)(F)C(F)(F)C(F)(F)C(F)(F)C(F)(F)C(F)(F)CC[n+]2ccccc2		-9.12	0.65	3.47	P	OUT
QL01161	2,2,3,3,4,4,5,5,6,6,7,7,7-Tridecafluoroheptyl 4-methylbenzene-1-sulfonate	883500-11-6	Cc1ccc(cc1)S(=O)(=O)OCC(F)(F)C(F)(F)C(F)(F)C(F)(F)C(F)(F)C(F)(F)F		-8.30	0.66	2.87	P	IN
QL01162	3,3,4,4,5,5,6,6,7,7,8,8,9,9,10,10,10-Heptadecafluorodecyl 4-methylbenzene-1-sulfonate	113823-56-6	Cc1ccc(cc1)S(=O)(=O)OCCC(F)(F)C(F)(F)C(F)(F)C(F)(F)C(F)(F)C(F)(F)C(F)(F)C(F)(F)F		-8.87	0.66	3.12	P	OUT
QL01163	3,3,4,4,5,5,6,6,6-Nonafluorohexyl 4-methylbenzene-1-sulfonate	188492-56-0	Cc1ccc(cc1)S(=O)(=O)OCCC(F)(F)C(F)(F)C(F)(F)C(F)(F)F		-7.61	0.64	2.73	P	IN
QL01164	(Heptadecafluorooctyl)phosphonic acid--4-methylaniline (1/1)	1263361-03-0	Cc1ccc(N)cc1.OP(O)(=O)C(F)(F)C(F)(F)C(F)(F)C(F)(F)C(F)(F)C(F)(F)C(F)(F)C(F)(F)F		-8.55	0.66	2.92	P	IN
QL01165	(Tridecafluorohexyl)phosphonic acid--4-methylaniline (1/1)	1263361-02-9	Cc1ccc(N)cc1.OP(O)(=O)C(F)(F)C(F)(F)C(F)(F)C(F)(F)C(F)(F)C(F)(F)F		-7.63	0.66	2.54	P	IN
QL01166	N-(2,4-Dimethylphenyl)-2,2,3,3,4,4,4-heptafluorobutanamide	90791-28-9	Cc1ccc(NC(=O)C(F)(F)C(F)(F)C(F)(F)F)c(C)c1		-6.85	0.64	2.42	P	IN
QL01167	N-(3-Chloro-4-methylphenyl)-2,2,3,3,4,4,4-heptafluorobutanamide	111216-49-0	Cc1ccc(NC(=O)C(F)(F)C(F)(F)C(F)(F)F)cc1Cl		-6.92	0.64	2.43	P	IN
QL01168	4-Methylphenyl nonafluorobutane-1-sulfonate	93131-73-8	Cc1ccc(OS(=O)(=O)C(F)(F)C(F)(F)C(F)(F)C(F)(F)F)cc1		-7.47	0.65	2.53	P	IN
QL01169	N-(2,6-Dimethylphenyl)-2,2,3,3,4,4,4-heptafluorobutanamide	101948-89-4	Cc1cccc(C)c1NC(=O)C(F)(F)C(F)(F)C(F)(F)F		-6.42	0.64	2.20	P	IN
QL01170	1-(1,1,1,2,3,3,3-Heptafluoropropan-2-yl)-2-methylbenzene	51114-10-4	Cc1ccccc1C(F)(C(F)(F)F)C(F)(F)F		-5.45	0.64	1.74	P	IN
QL01171	2-Methyl-N-(2-methyl-5-(((1,3-bis((perfluoro-9-methyldecyl)ethylthio)-2-propoxy)carbonyl)amino)phenyl)-1-aziridinecarboxamide	72779-04-5	CC1CN1C(=O)NC1=C(C)C=CC(NC(=O)OC(CSCCC(F)(F)C(F)(F)C(F)(F)C(F)(F)C(F)(F)C(F)(F)C(F)(F)C(F)(F)C(F)(C(F)(F)F)C(F)(F)F)CSCCC(F)(F)C(F)(F)C(F)(F)C(F)(F)C(F)(F)C(F)(F)C(F)(F)C(F)(F)C(F)(C(F)(F)F)C(F)(F)F)=C1		-10.27	0.66	3.84	P	OUT
QL01172	1,3-bis((perfluoro-5-methylhexyl)ethylthio)prop-2-yl (4-methyl-3-(((2-methyl-1-aziridinyl)carbonyl)amino)phenyl)carbamate	72779-05-6	CC1CN1C(=O)NC1=CC(NC(=O)OC(CSCCC(F)(F)C(F)(F)C(F)(F)C(F)(F)C(F)(C(F)(F)F)C(F)(F)F)CSCCC(F)(F)C(F)(F)C(F)(F)C(F)(F)C(F)(C(F)(F)F)C(F)(F)F)=CC=C1C		-8.96	0.65	3.36	P	OUT
QL01173	1,3-Bis(2-(perfluoro-7-methyloctyl)-1-ethylsulfanyl)propan-2-yl {4-methyl-3-[(2-methylaziridine-1-carbonyl)amino]phenyl}carbamate	53122-42-2	CC1CN1C(=O)NC1=CC(NC(=O)OC(CSCCC(F)(F)C(F)(F)C(F)(F)C(F)(F)C(F)(F)C(F)(F)C(F)(C(F)(F)F)C(F)(F)F)CSCCC(F)(F)C(F)(F)C(F)(F)C(F)(F)C(F)(F)C(F)(F)C(F)(C(F)(F)F)C(F)(F)F)=CC=C1C		-9.69	0.66	3.62	P	OUT
QL01174	1-(Heptafluoropropyl)-4-methylisoquinoline	70414-16-3	Cc1cnc(c2ccccc12)C(F)(F)C(F)(F)C(F)(F)F		-6.19	0.63	2.14	P	IN
QL01175	3-(Heptafluoropropyl)-5-methyl-1H-pyrrole-2-carbaldehyde	123825-55-8	Cc1Nc(C=O)c(c1)C(F)(F)C(F)(F)C(F)(F)F		-5.83	0.64	1.91	P	IN
QL01176	5-(Heptafluoropropyl)-1-methylisoquinoline	28439-77-2	Cc1nccc2c(cccc12)C(F)(F)C(F)(F)C(F)(F)F		-6.19	0.63	2.14	P	IN
QL01177	4-Bromo-3-(heptafluoropropyl)-5-methyl-1H-pyrazole	82633-48-5	Cc1Nnc(c1Br)C(F)(F)C(F)(F)C(F)(F)F		-6.26	0.65	1.90	P	IN
QL01178	1-Methyl-4H-[1,2,4]triazolo[4,3-a][1]benzazepin-6-yl nonafluorobutane-1-sulfonate	1588522-06-8	Cc1nnc2CC=C(OS(=O)(=O)C(F)(F)C(F)(F)C(F)(F)C(F)(F)F)c3ccccc3-n12		-7.34	0.64	2.63	P	IN
QL01179	5,5,6,6,7,7,7-Heptafluoro-4-methylheptan-3-one	61282-89-1	CCC(=O)C(C)C(F)(F)C(F)(F)C(F)(F)F		-6.02	0.64	1.92	P	IN
QL01180	Ethyl perfluoropentanyl ketone	383177-55-7	CCC(=O)C(F)(F)C(F)(F)C(F)(F)C(F)(F)C(F)(F)F		-7.06	0.66	2.17	P	IN
QL01181	1-[4-(1,1,2,2,3,3,4,4,5,5,6,6,7,7,8,8,8-Heptadecafluorooctane-1-sulfonyl)piperazin-1-yl]propan-1-one	113584-34-2	CCC(=O)N1CCN(CC1)S(=O)(=O)C(F)(F)C(F)(F)C(F)(F)C(F)(F)C(F)(F)C(F)(F)C(F)(F)C(F)(F)F		-8.83	0.66	3.08	P	OUT

QL01182	1,1,1,2,2,3,3-Heptafluoro-4,4-bis(trifluoromethyl)hexane	100646-00-2	CCC(C(F)(F)F)(C(F)(F)F)C(F)(F)C(F)(F)C(F)(F)F		-6.28	0.67	1.69	P	IN
QL01183	2-Ethyl-3,3,4,4,5,5,6,6,7,7,8,8,8-tridecafluorooctanoic acid	136022-87-2	CCC(C(O)=O)C(F)(F)C(F)(F)C(F)(F)C(F)(F)C(F)(F)C(F)(F)F		-7.13	0.66	2.24	P	IN
QL01184	1,1,1,2,2,3,3-Heptafluoro-5-nitroheptan-4-ol	355-90-8	CCC(C(O)C(F)(F)C(F)(F)C(F)(F)F)[N+]([O-])=O		-5.84	0.64	1.91	P	IN
QL01185	Hexanamide, 6-[2,4-bis(1,1-dimethylpropyl)phenoxy]-N-[3-hydroxy-4-[(octafluoro-1-oxopentyl)amino]phenyl]-(9CI)	97331-50-5	CCC(C)(C)C1=CC(=C(OCCCCCC(=O)NC2=CC(O)=C(NC(=O)C(F)(F)C(F)(F)C(F)C(F)(F)F)C=C2)C=C1)C(C)(C)CC		-8.42	0.61	3.59	P	OUT
QL01186	4,4,5,5,6,6,6-Heptafluoro-3-methylhexan-3-ol	423-43-8	CCC(C)(O)C(F)(F)C(F)(F)C(F)(F)F		-6.40	0.67	1.81	P	IN
QL01187	6,6,7,7,8,8,9,9,9-Nonafluoro-3-methylnon-4-yn-3-ol	82721-69-5	CCC(C)(O)C#CC(F)(F)C(F)(F)C(F)(F)C(F)(F)F		-7.04	0.66	2.25	P	IN
QL01188	Butan-2-one O,O',O''-((3,3,4,4,5,5,6,6,7,7,8,8,8-tridecafluorooctyl)silylidyne)oxime	94158-20-0	CCC(C)=NO[Si](CCC(F)(F)C(F)(F)C(F)(F)C(F)(F)C(F)(F)C(F)(F)F)(ON=C(/C)CC)ON=C(/C)CC		-7.19	0.62	2.81	P	OUT
QL01189	N-(Butan-2-yl)-2,2,3,3,4,4,4-heptafluorobutanamide	75668-26-7	CCC(C)NC(=O)C(F)(F)C(F)(F)C(F)(F)F		-6.32	0.64	2.18	P	IN
QL01190	1-[(Butan-2-yl)amino]-3,3,4,4,5,5,6,6,7,7,8,8,9,9,10,10,11,11,11-nonadecafluoroundecan-2-ol	94817-79-5	CCC(C)NCC(O)C(F)(F)C(F)(F)C(F)(F)C(F)(F)C(F)(F)C(F)(F)C(F)(F)C(F)(F)C(F)(F)F		-8.61	0.66	2.94	P	IN
QL01191	Butan-2-yl N-(2,2,3,3,4,4,4-heptafluorobutanoyl)-D-alaninate	93080-42-3	CCC(C)OC(=O)[C@@H](C)NC(=O)C(F)(F)C(F)(F)C(F)(F)F		-6.78	0.63	2.53	P	OUT
QL01192	Butan-2-yl 2,3,3,3-tetrafluoro-2-(heptafluoropropoxy)propanoate	340034-57-3	CCC(C)OC(=O)C(F)(OC(F)(F)C(F)(F)C(F)(F)F)C(F)(F)F		-7.16	0.65	2.45	P	IN
QL01193	Butan-2-yl 2,3,3,3-tetrafluoro-2-[1,1,2,3,3,3-hexafluoro-2-(heptafluoropropoxy)propoxy]propanoate	330154-39-7	CCC(C)OC(=O)C(F)(OC(F)(F)C(F)(OC(F)(F)C(F)(F)C(F)(F)F)C(F)(F)F)C(F)(F)F		-7.89	0.66	2.69	P	IN
QL01194	3-(Butane-2-sulfonyl)-4-(1,1,1,2,3,3,3-heptafluoropropan-2-yl)-2-methylbenzoic acid	1355554-57-2	CCC(C)S(=O)(=O)c1c(C)c(ccc1C(F)(C(F)(F)F)C(F)(F)F)C(O)=O		-6.10	0.64	2.07	P	IN
QL01195	3-(Butane-2-sulfonyl)-2-ethyl-4-(1,1,1,2,3,3,3-heptafluoropropan-2-yl)benzoic acid	1355555-25-7	CCC(C)S(=O)(=O)c1c(CC)c(ccc1C(F)(C(F)(F)F)C(F)(F)F)C(O)=O		-5.84	0.63	2.06	P	OUT
QL01196	3-(Butane-2-sulfinyl)-4-(1,1,1,2,3,3,3-heptafluoropropan-2-yl)-2-methylbenzoic acid	1355554-34-5	CCC(C)S(=O)c1c(C)c(ccc1C(F)(C(F)(F)F)C(F)(F)F)C(O)=O		-5.86	0.63	2.07	P	OUT
QL01197	3-(Butane-2-sulfinyl)-2-ethyl-4-(1,1,1,2,3,3,3-heptafluoropropan-2-yl)benzoic acid	1355555-01-9	CCC(C)S(=O)c1c(CC)c(ccc1C(F)(C(F)(F)F)C(F)(F)F)C(O)=O		-5.60	0.62	2.06	P	OUT
QL01198	3-[(Butan-2-yl)sulfanyl]-4-(1,1,1,2,3,3,3-heptafluoropropan-2-yl)-2-methylbenzoic acid	1355554-14-1	CCC(C)Sc1c(C)c(ccc1C(F)(C(F)(F)F)C(F)(F)F)C(O)=O		-5.76	0.62	2.06	P	OUT
QL01199	3-[(Butan-2-yl)sulfanyl]-2-ethyl-4-(1,1,1,2,3,3,3-heptafluoropropan-2-yl)benzoic acid	1355554-79-8	CCC(C)Sc1c(CC)c(ccc1C(F)(C(F)(F)F)C(F)(F)F)C(O)=O		-5.48	0.61	2.05	P	OUT
QL01200	2-Ethyl-3,3,4,4,5,5,6,6,7,7,8,8,8-tridecafluorooctanal (2,4-dinitrophenyl)hydrazone	136022-83-8	CCC(C=NNC1=C(C=C(C=C1)[N+]([O-])=O)[N+]([O-])=O)C(F)(F)C(F)(F)C(F)(F)C(F)(F)C(F)(F)C(F)(F)F		-8.01	0.64	3.03	P	OUT
QL01201	2-Ethyl-3,4,4,5,5,6,6,7,7,8,8,8-dodecafluorooct-2-enal	61282-83-5	CCC(C=O)=C(F)C(F)(F)C(F)(F)C(F)(F)C(F)(F)C(F)(F)F		-6.78	0.65	2.25	P	IN
QL01202	1,1,1,2,2,3,3-heptafluoro-5-nitroheptane	356-66-1	CCC(CC(F)(F)C(F)(F)C(F)(F)F)[N+]([O-])=O		-5.76	0.64	1.90	P	IN
QL01203	7-Ethyl-1,1,1,2,2,3,3-heptafluorononane-4,6-dione	80284-42-0	CCC(CC)C(=O)CC(=O)C(F)(F)C(F)(F)C(F)(F)F		-6.11	0.62	2.24	P	OUT
QL01204	1,1,1,2,2,3,3,4,4,5,5,6,6,7,7,8,8,9,9,10,10,11,11,12,12-Pentacosafluorotetradecane	89109-68-2	CCC(F)(F)C(F)(F)C(F)(F)C(F)(F)C(F)(F)C(F)(F)C(F)(F)C(F)(F)C(F)(F)C(F)(F)C(F)(F)C(F)(F)F		-9.10	0.69	2.89	P	OUT
QL01205	1,1,1,2,2,3,3,4,4,5,5,6,6,7,7,8,8,9,9,10,10-Henicosafluorododecane	154478-87-2	CCC(F)(F)C(F)(F)C(F)(F)C(F)(F)C(F)(F)C(F)(F)C(F)(F)C(F)(F)C(F)(F)C(F)(F)F		-8.63	0.68	2.68	P	IN
QL01206	1-(Perfluorohexyl)ethane	80793-17-5	CCC(F)(F)C(F)(F)C(F)(F)C(F)(F)C(F)(F)C(F)(F)F		-7.35	0.68	2.15	P	IN
QL01207	1,1,1,2,2,3,3,4,4,5,5-Undecafluoroheptane	503189-10-4	CCC(F)(F)C(F)(F)C(F)(F)C(F)(F)C(F)(F)F		-6.72	0.67	1.89	P	IN
QL01208	1,1,1,2,2,3,3,4,4-Nonafluorohexane	38436-17-8	CCC(F)(F)C(F)(F)C(F)(F)C(F)(F)F		-6.40	0.67	1.81	P	IN
QL01209	1,1,1,2,2,3,3-Heptafluoropentane	30423-63-3	CCC(F)(F)C(F)(F)C(F)(F)F		-5.48	0.66	1.46	P	IN
QL01210	1,1,1,2,2,3,3,4,4,5,5,6,6,7,7,8,8-Heptadecafluoro-10-iodododecane	99325-00-5	CCC(I)CC(F)(F)C(F)(F)C(F)(F)C(F)(F)C(F)(F)C(F)(F)C(F)(F)C(F)(F)F		-8.21	0.67	2.69	P	IN
QL01211	5,5,6,6,7,7,7-Heptafluoroheptan-3-amine	679-00-5	CCC(N)CC(F)(F)C(F)(F)C(F)(F)F		-5.87	0.64	1.93	P	IN
QL01212	4-{2-[2,4-Bis(2-methylbutan-2-yl)phenoxy]butanamido}-2-hydroxy-N-(nonafluorobutyl)benzamide	94705-65-4	CCC(Oc1ccc(cc1C(C)(C)CC)C(C)(C)CC)C(=O)Nc1ccc(C(=O)NC(F)(F)C(F)(F)C(F)(F)C(F)(F)F)c(O)c1		-8.40	0.63	3.39	P	OUT
QL01213	Tetrapropylammonium perfluorooctanoate	277749-00-5	CCC[N+](CCC)(CCC)CCC.[O-]C(=O)C(F)(F)C(F)(F)C(F)(F)C(F)(F)C(F)(F)C(F)(F)C(F)(F)F		-6.77	0.62	2.63	P	OUT
QL01214	5,5,6,6,7,7,7-Heptafluorohept-3-ene	355-98-6	CCC=CC(F)(F)C(F)(F)C(F)(F)F		-5.63	0.63	1.95	P	OUT
QL01215	Orthobutyric acid, heptafluoro-, cyclic ester with 2-ethyl-2-(hydroxymethyl)-1,3-propanediol (11)	31185-68-9	CCC12COC(OC1)(OC2)C(F)(F)C(F)(F)C(F)(F)F		-7.09	0.65	2.32	P	IN
QL01216	2-Ethyl-4-(1,1,1,2,3,3,3-heptafluoropropan-2-yl)-3-(2-methylpropane-2-sulfonyl)benzoic acid	1355555-26-8	CCc1c(ccc(c1S(=O)(=O)C(C)(C)C)C(F)(C(F)(F)F)C(F)(F)F)C(O)=O		-6.42	0.64	2.11	P	IN

QL01217	2-Ethyl-4-(1,1,1,2,3,3,3-heptafluoropropan-2-yl)-3-(propane-2-sulfonyl)benzoic acid	1355555-15-5	CCc1c(ccc(c1S(=O)(=O)C(C)C)C(F)(C(F)(F)F)C(F)(F)F)C(O)=O		-6.18	0.64	2.11	P	IN
QL01218	2-Ethyl-4-(1,1,1,2,3,3,3-heptafluoropropan-2-yl)-3-(2-methylpropane-1-sulfonyl)benzoic acid	1355555-21-3	CCc1c(ccc(c1S(=O)(=O)CC(C)C)C(F)(C(F)(F)F)C(F)(F)F)C(O)=O		-5.81	0.63	2.05	P	OUT
QL01219	3-(Ethanesulfonyl)-2-ethyl-4-(1,1,1,2,3,3,3-heptafluoropropan-2-yl)benzoic acid	1355555-11-1	CCc1c(ccc(c1S(=O)(=O)CC)C(F)(C(F)(F)F)C(F)(F)F)C(O)=O		-6.00	0.63	2.12	P	OUT
QL01220	2-Ethyl-4-(1,1,1,2,3,3,3-heptafluoropropan-2-yl)-3-(2-methylpropane-2-sulfinyl)benzoic acid	1355555-05-3	CCc1c(ccc(c1S(=O)C(C)(C)C)C(F)(C(F)(F)F)C(F)(F)F)C(O)=O		-6.17	0.64	2.11	P	IN
QL01221	2-Ethyl-4-(1,1,1,2,3,3,3-heptafluoropropan-2-yl)-3-(propane-2-sulfinyl)benzoic acid	1355554-94-7	CCc1c(ccc(c1S(=O)C(C)C)C(F)(C(F)(F)F)C(F)(F)F)C(O)=O		-5.91	0.63	2.12	P	OUT
QL01222	2-Ethyl-4-(1,1,1,2,3,3,3-heptafluoropropan-2-yl)-3-(2-methylpropane-1-sulfinyl)benzoic acid	1355554-99-2	CCc1c(ccc(c1S(=O)CC(C)C)C(F)(C(F)(F)F)C(F)(F)F)C(O)=O		-5.59	0.62	2.05	P	OUT
QL01223	3-(Ethanesulfinyl)-2-ethyl-4-(1,1,1,2,3,3,3-heptafluoropropan-2-yl)benzoic acid	1355554-87-8	CCc1c(ccc(c1S(=O)CC)C(F)(C(F)(F)F)C(F)(F)F)C(O)=O		-5.72	0.62	2.12	P	OUT
QL01224	2-Ethyl-4-(1,1,1,2,3,3,3-heptafluoropropan-2-yl)-3-(methanesulfonyl)benzoic acid	1355555-09-7	CCc1c(ccc(c1S(C)(=O)=O)C(F)(C(F)(F)F)C(F)(F)F)C(O)=O		-6.24	0.64	2.13	P	IN
QL01225	2-Ethyl-4-(1,1,1,2,3,3,3-heptafluoropropan-2-yl)-3-(methanesulfinyl)benzoic acid	1355554-84-5	CCc1c(ccc(c1S(C)=O)C(F)(C(F)(F)F)C(F)(F)F)C(O)=O		-5.96	0.63	2.13	P	OUT
QL01226	2-Ethyl-4-(1,1,1,2,3,3,3-heptafluoropropan-2-yl)-3-[(propan-2-yl)sulfanyl]benzoic acid	1355554-70-9	CCc1c(ccc(c1SC(C)C)C(F)(C(F)(F)F)C(F)(F)F)C(O)=O		-5.75	0.62	2.11	P	OUT
QL01227	2-Ethyl-4-(1,1,1,2,3,3,3-heptafluoropropan-2-yl)-3-[(2-methylpropyl)sulfanyl]benzoic acid	1355554-74-3	CCc1c(ccc(c1SCC(C)C)C(F)(C(F)(F)F)C(F)(F)F)C(O)=O		-5.51	0.62	2.04	P	OUT
QL01228	3-(tert-Butylsulfanyl)-2-ethyl-4-(1,1,1,2,3,3,3-heptafluoropropan-2-yl)benzoic acid	1355554-81-2	CCc1c(SC(C)(C)C)c(ccc1C(O)=O)C(F)(C(F)(F)F)C(F)(F)F		-6.04	0.63	2.10	P	OUT
QL01229	2-Ethyl-4-(1,1,1,2,3,3,3-heptafluoropropan-2-yl)-3-(methylsulfanyl)benzoic acid	1355554-62-9	CCc1c(SC)c(ccc1C(O)=O)C(F)(C(F)(F)F)C(F)(F)F		-5.76	0.62	2.12	P	OUT
QL01230	2-Chloro-6-ethyl-4-(1,1,1,2,3,3,3-heptafluoropropan-2-yl)aniline	1630969-89-9	CCc1cc(cc(Cl)c1N)C(F)(C(F)(F)F)C(F)(F)F		-5.65	0.64	1.85	P	IN
QL01231	2-Ethyl-4-(1,1,1,2,3,3,3-heptafluoropropan-2-yl)aniline	273735-34-5	CCc1cc(ccc1N)C(F)(C(F)(F)F)C(F)(F)F		-5.55	0.63	1.85	P	IN
QL01232	N'-[(4-Ethylphenyl)methylidene]-2,2,3,3,4,4,5,5,6,6,7,7,7-tridecafluoroheptanehydrazide	445258-05-9	CCc1ccc(C=NNC(=O)C(F)(F)C(F)(F)C(F)(F)C(F)(F)C(F)(F)C(F)(F)F)cc1		-8.15	0.64	3.06	P	OUT
QL01233	N-(2-Ethylphenyl)-2,2,3,3,4,4,4-heptafluorobutanamide	51814-65-4	CCc1ccccc1NC(=O)C(F)(F)C(F)(F)C(F)(F)F		-6.05	0.62	2.23	P	OUT
QL01234	1,1,1,2,2,3,3-Heptafluoroheptan-4-one	425-22-9	CCCC(=O)C(F)(F)C(F)(F)C(F)(F)F		-5.78	0.63	1.93	P	IN
QL01235	2,2,3,3,4,4,4-Heptafluorobutyl butanoate	2926-44-5	CCCC(=O)OCC(F)(F)C(F)(F)C(F)(F)F		-5.98	0.62	2.23	P	OUT
QL01236	1,1,1,2,2,3,3,4,4,5,5,6,6,7,7,8,8,9,9,10,10-Henicosafluorotridecane	1835251-22-3	CCCC(F)(F)C(F)(F)C(F)(F)C(F)(F)C(F)(F)C(F)(F)C(F)(F)C(F)(F)C(F)(F)C(F)(F)F		-8.50	0.68	2.73	P	IN
QL01237	1,1,1,2,2,3,3,4,4,5,5,6,6,7,7,8,8-Heptadecafluoroundecane	1835250-28-6	CCCC(F)(F)C(F)(F)C(F)(F)C(F)(F)C(F)(F)C(F)(F)C(F)(F)C(F)(F)F		-7.91	0.67	2.50	P	IN
QL01238	1,1,1,2,2,3,3,4,4,5,5,6,6-Tridecafluorononane	NOCAS_871521	CCCC(F)(F)C(F)(F)C(F)(F)C(F)(F)C(F)(F)C(F)(F)F		-7.16	0.66	2.23	P	IN
QL01239	1,1,1,2,2,3,3,4,4,5,5-Undecafluorooctane	1645298-12-9	CCCC(F)(F)C(F)(F)C(F)(F)C(F)(F)C(F)(F)F		-6.87	0.66	2.17	P	IN
QL01240	1,1,1,2,2,3,3,4,4-Nonafluoroheptane	154381-51-8	CCCC(F)(F)C(F)(F)C(F)(F)C(F)(F)F		-6.13	0.65	1.91	P	IN
QL01241	1,2,4,5-Benzenetetracarboxylic acid, mixed 3-chloro-2-hydroxypropyl and .gamma.-.omega.-perfluoro-C8-10-alkyl esters	68954-01-8	CCCC(F)(F)C(F)(F)C(F)(F)CCCOC(CCl)COC(=O)c1cc(C(O)=O)c(cc1C(=O)OCC(O)CCl)C(=O)OCC(O)CCl		-7.53	0.59	3.49	P	OUT
QL01242	1,1,1,2,2,3,3-Heptafluorohexane	678-98-8	CCCC(F)(F)C(F)(F)C(F)(F)F		-5.71	0.64	1.85	P	IN
QL01243	1,1,1,2,2,3,3,4,4,5,5,6,6,7,7-Pentadecafluoro-9-iodododecane	25431-48-5	CCCC(I)CC(F)(F)C(F)(F)C(F)(F)C(F)(F)C(F)(F)C(F)(F)C(F)(F)F		-7.91	0.66	2.70	P	IN
QL01244	1,1,1,2,2,3,3-heptafluoroheptan-4-ol	425-21-8	CCCC(O)C(F)(F)C(F)(F)C(F)(F)F		-5.78	0.63	1.93	P	IN
QL01245	N,N-Dibutyl-N-methylbutan-1-aminium heptadecafluorooctane-1-sulfonate	124472-68-0	CCCC[N+](C)(CCCC)CCCC.[O-]S(=O)(=O)C(F)(F)C(F)(F)C(F)(F)C(F)(F)C(F)(F)C(F)(F)C(F)(F)C(F)(F)F		-7.17	0.63	2.65	P	OUT
QL01246	N,N-Dibutyl-N-methylbutan-1-aminium nonafluorobutane-1-sulfonate	124472-66-8	CCCC[N+](C)(CCCC)CCCC.[O-]S(=O)(=O)C(F)(F)C(F)(F)C(F)(F)C(F)(F)F		-5.74	0.61	2.28	P	OUT
QL01247	Tetrabutylammonium perfluorooctanesulfonate	111873-33-7	CCCC[N+](CCCC)(CCCC)CCCC.[O-]S(=O)(=O)C(F)(F)C(F)(F)C(F)(F)C(F)(F)C(F)(F)C(F)(F)C(F)(F)C(F)(F)F		-6.79	0.62	2.63	P	OUT
QL01248	N,N,N-Tributylbutan-1-aminium nonafluorobutane-1-sulfonate	108427-52-7	CCCC[N+](CCCC)(CCCC)CCCC.[O-]S(=O)(=O)C(F)(F)C(F)(F)C(F)(F)C(F)(F)F		-5.41	0.59	2.32	P	OUT
QL01249	Perfluoro(4-[(4-ethyl-3,4-dimethylhex-2-en-2-yl)oxy])benzene-1-sulfonate tributylammonium	70729-63-4	CCCC[NH+](CCCC)CCCC.[O-]S(=O)(=O)C1=CC=C(OC(=C(C(F)(F)F)C(C(F)(F)F)(C(F)(F)C(F)(F)F)C(F)(F)C(F)(F)F)C(F)(F)F)C=C1		-7.33	0.62	2.99	P	OUT

QL01250	Tetrabutylphosphonium perfluorobutanesulfonate	220689-12-3	CCCC[PH+](CCCC)(CCCC)CCCC.[O-]S(=O)(=O)C(F)(F)C(F)(F)C(F)(F)C(F)(F)F		-5.41	0.59	2.32	P	OUT
QL01251	1-(Butylselanyl)-2,2,3,3,4,4,4-heptafluorobutan-1-ol	1092379-04-8	CCCC[Se]C(O)C(F)(F)C(F)(F)C(F)(F)F		-6.02	0.62	2.28	P	OUT
QL01252	1,1,1,2,2,3,3-Heptafluorooct-4-ene	2707-72-4	CCCC=CC(F)(F)C(F)(F)C(F)(F)F		-5.83	0.62	2.17	P	OUT
QL01253	2-Ethylhexyl 2,3,3,3-tetrafluoro-2-(heptafluoropropoxy)propanoate	340034-61-9	CCCCC(CC)COC(=O)C(F)(OC(F)(F)C(F)(F)C(F)(F)F)C(F)(F)F		-7.05	0.62	2.74	P	OUT
QL01254	2-Butyl-5,6,6,7,7,8,8,8-octafluorooct-4-enoic acid	90292-29-8	CCCCC(CC=C(F)C(F)(F)C(F)(F)C(F)(F)F)C(O)=O		-6.31	0.61	2.48	P	OUT
QL01255	1,1,1,2,2,3,3,4,4,5,5,6,6,7,7,8,8,9,9,10,10,11,11,12,12-Pentacosafluorohexadecane	89109-69-3	CCCCC(F)(F)C(F)(F)C(F)(F)C(F)(F)C(F)(F)C(F)(F)C(F)(F)C(F)(F)C(F)(F)C(F)(F)C(F)(F)C(F)(F)F		-9.13	0.68	3.06	P	OUT
QL01256	1,1,1,2,2,3,3,4,4,5,5,6,6,7,7,8,8,9,9,10,10-Henicosafluorotetradecane	1244062-17-6	CCCCC(F)(F)C(F)(F)C(F)(F)C(F)(F)C(F)(F)C(F)(F)C(F)(F)C(F)(F)C(F)(F)C(F)(F)F		-8.66	0.67	2.89	P	IN
QL01257	1,1,1,2,2,3,3,4,4,5,5,6,6,7,7,8,8-Heptadecafluorododecane	182130-12-7	CCCCC(F)(F)C(F)(F)C(F)(F)C(F)(F)C(F)(F)C(F)(F)C(F)(F)C(F)(F)F		-8.10	0.66	2.68	P	IN
QL01258	1,1,1,2,2,3,3,4,4,5,5,6,6-Tridecafluorodecane	154478-86-1	CCCCC(F)(F)C(F)(F)C(F)(F)C(F)(F)C(F)(F)C(F)(F)F		-7.41	0.66	2.46	P	IN
QL01259	1,1,1,2,2,3,3,4,4-Nonafluorooctane	253342-14-2	CCCCC(F)(F)C(F)(F)C(F)(F)C(F)(F)F		-6.48	0.64	2.21	P	IN
QL01260	1,1,1,2,2,3,3-Heptafluoroheptane	152076-35-2	CCCCC(F)(F)C(F)(F)C(F)(F)F		-5.63	0.63	1.95	P	OUT
QL01261	(5Z)-1,1,1,2,2,3,3,4,4-Nonafluoro-6-iododec-5-ene	107831-93-6	CCCCC(I)=CC(F)(F)C(F)(F)C(F)(F)C(F)(F)F		-6.66	0.63	2.44	P	OUT
QL01262	1,1,1,2,2,3,3-Heptafluoro-6-iodo-4,4-bis(trifluoromethyl)decane	123629-50-5	CCCCC(I)CC(C(F)(F)F)(C(F)(F)F)C(F)(F)C(F)(F)C(F)(F)F		-6.66	0.64	2.28	P	IN
QL01263	1,1,1,2-Tetrafluoro-4-iodo-2-(trifluoromethyl)octane	161094-94-6	CCCCC(I)CC(F)(C(F)(F)F)C(F)(F)F		-5.52	0.62	2.06	P	OUT
QL01264	1,1,1,2,2,3,3,4,4,5,5,6,6,7,7,8,8,9,9,10,10-Henicosafluoro-12-iodohexadecane	129838-38-6	CCCCC(I)CC(F)(F)C(F)(F)C(F)(F)C(F)(F)C(F)(F)C(F)(F)C(F)(F)C(F)(F)C(F)(F)C(F)(F)F		-8.81	0.66	3.07	P	OUT
QL01265	1,1,1,2,2,3,3,4,4,5,5,6,6,7,7,8,8-Heptadecafluoro-10-iodotetradecane	92835-74-0	CCCCC(I)CC(F)(F)C(F)(F)C(F)(F)C(F)(F)C(F)(F)C(F)(F)C(F)(F)C(F)(F)F		-8.29	0.66	2.90	P	IN
QL01266	1,1,1,2,2,3,3,4,4,5,5,6,6-Tridecafluoro-8-iodododecane	120695-82-1	CCCCC(I)CC(F)(F)C(F)(F)C(F)(F)C(F)(F)C(F)(F)C(F)(F)F		-7.64	0.65	2.72	P	IN
QL01267	1,1,1,2,2,3,3,4,4-Nonafluoro-6-iododecane	40735-32-8	CCCCC(I)CC(F)(F)C(F)(F)C(F)(F)C(F)(F)F		-6.66	0.63	2.44	P	OUT
QL01268	1,1,1,2,2,3,3-heptafluoro-5-iodononane	755-48-6	CCCCC(I)CC(F)(F)C(F)(F)C(F)(F)F		-6.00	0.62	2.28	P	OUT
QL01269	Hexanamide, 2-[2,4-bis(1,1-dimethylpropyl)phenoxy]-N-[3-hydroxy-4-[(octafluoro-1-oxopentyl)amino]phenyl]-(9CI)	83003-52-5	CCCCC(OC1=C(C=C(C=C1)C(C)(C)CC)C(C)(C)CC)C(=O)NC1=CC(O)=C(NC(=O)C(F)(F)C(F)(F)C(F)C(F)(F)F)C=C1		-8.06	0.61	3.39	P	OUT
QL01270	Hexanamide, 2-[2,4-bis(1,1-dimethylpropyl)phenoxy]-N-[4-[(2,2,3,3,4,4,4-heptafluoro-1-oxobutyl)amino]-3-hydroxyphenyl]-	2923-93-5	CCCCC(Oc1ccc(cc1C(C)(C)CC)C(C)(C)CC)C(=O)Nc1ccc(NC(=O)C(F)(F)C(F)(F)C(F)(F)F)c(O)c1		-8.01	0.61	3.35	P	OUT
QL01271	N,N,N-Tripropylpentan-1-aminium heptadecafluorooctane-1-sulfonate	56773-56-9	CCCCC[N+](CCC)(CCC)CCC.[O-]S(=O)(=O)C(F)(F)C(F)(F)C(F)(F)C(F)(F)C(F)(F)C(F)(F)C(F)(F)C(F)(F)F		-6.86	0.63	2.59	P	OUT
QL01272	N,N,N-Tripropylpentan-1-aminium nonafluorobutane-1-sulfonate	56773-55-8	CCCCC[N+](CCC)(CCC)CCC.[O-]S(=O)(=O)C(F)(F)C(F)(F)C(F)(F)C(F)(F)F		-5.62	0.60	2.35	P	OUT
QL01273	(5E)-7,7,8,8,9,9,10,10,11,11,12,12,13,13,14,14,14-Heptadecafluorotetradec-5-ene	210491-04-6	CCCCC=CC(F)(F)C(F)(F)C(F)(F)C(F)(F)C(F)(F)C(F)(F)C(F)(F)C(F)(F)F		-8.24	0.65	2.91	P	IN
QL01274	7,7,8,8,9,9,10,10,11,11,12,12,13,13,13-Pentadecafluorotridec-5-ene	52717-05-2	CCCCC=CC(F)(F)C(F)(F)C(F)(F)C(F)(F)C(F)(F)C(F)(F)C(F)(F)F		-7.81	0.65	2.75	P	IN
QL01275	(5E)-1,1,1,2,2,3,3,4,4-Nonafluorodec-5-ene	131294-67-2	CCCCC=CC(F)(F)C(F)(F)C(F)(F)C(F)(F)F		-6.57	0.63	2.46	P	OUT
QL01276	1,1,1,2,2,3,3-heptafluoronon-4-ene	57325-40-3	CCCCC=CC(F)(F)C(F)(F)C(F)(F)F		-5.77	0.61	2.25	P	OUT
QL01277	1-[4-(1,1,2,2,3,3,4,4,5,5,6,6,7,7,8,8,8-Heptadecafluorooctane-1-sulfonyl)piperazin-1-yl]hexan-1-one	113584-36-4	CCCCCC(=O)N1CCN(CC1)S(=O)(=O)C(F)(F)C(F)(F)C(F)(F)C(F)(F)C(F)(F)C(F)(F)C(F)(F)C(F)(F)F		-8.86	0.65	3.29	P	OUT
QL01278	Tetratriacontafluoro-10,13,16,19-tetraoxaoctacosadiene	84029-54-9	CCCCCC(C(C(C(OC(C(OC(C(OC(C(OC(C(C(C(C(/C(=C(/C(=C(F)F)F)F)/F)(F)F)(F)F)(F)F)(F)F)(F)F)(F)F)(F)F)(F)F)(F)F)(F)F)(F)F)(F)F)(F)F)(F)F)F		-10.08	0.66	3.81	P	OUT
QL01279	2,2,3,3,4,4,4-heptafluoro-N-heptan-2-ylbutanamide	61578-74-3	CCCCCC(C)NC(=O)C(F)(F)C(F)(F)C(F)(F)F		-6.38	0.61	2.55	P	OUT
QL01280	1,1,1,2,2,3,3,4,4,5,5,6,6,7,7,8,8,9,9,10,10-Henicosafluoropentadecane	250738-42-2	CCCCCC(F)(F)C(F)(F)C(F)(F)C(F)(F)C(F)(F)C(F)(F)C(F)(F)C(F)(F)C(F)(F)C(F)(F)F		-8.62	0.67	2.93	P	IN
QL01281	1,1,1,2,2,3,3,4,4,5,5,6,6,7,7,8,8-Heptadecafluorotridecane	1835250-47-9	CCCCCC(F)(F)C(F)(F)C(F)(F)C(F)(F)C(F)(F)C(F)(F)C(F)(F)C(F)(F)F		-8.06	0.66	2.74	P	IN
QL01282	1,1,1,2,2,3,3,4,4,5,5,6,6-Tridecafluoroundecane	1287702-48-0	CCCCCC(F)(F)C(F)(F)C(F)(F)C(F)(F)C(F)(F)C(F)(F)F		-7.35	0.65	2.52	P	IN
QL01283	1,1,1,2,2,3,3,4,4,5,5-Undecafluorodecane	1287702-46-8	CCCCCC(F)(F)C(F)(F)C(F)(F)C(F)(F)C(F)(F)F		-7.08	0.64	2.49	P	IN
QL01284	1,1,1,2,2,3,3,4,4-Nonafluorononane	1190430-21-7	CCCCCC(F)(F)C(F)(F)C(F)(F)C(F)(F)F		-6.41	0.63	2.28	P	OUT

QL01285	1,1,1,2,2,3,3-Heptafluorooctane	2708-54-5	CCCCCC(F)(F)C(F)(F)C(F)(F)F		-5.83	0.62	2.17	P	OUT
QL01286	1,1,1,2-Tetrafluoro-4-iodo-2-(trifluoromethyl)nonane	756-92-3	CCCCCC(I)CC(F)(C(F)(F)F)C(F)(F)F		-5.51	0.61	2.15	P	OUT
QL01287	1,1,1,2,2,3,3,4,4,5,5,6,6,7,7,8,8,9,9,10,10-Henicosafluoro-12-iodoheptadecane	129838-39-7	CCCCCC(I)CC(F)(F)C(F)(F)C(F)(F)C(F)(F)C(F)(F)C(F)(F)C(F)(F)C(F)(F)C(F)(F)C(F)(F)F		-8.78	0.66	3.12	P	IN
QL01288	1,1,1,2,2,3,3,4,4,5,5,6,6,7,7-Pentadecafluoro-9-iodotetradecane	38787-66-5	CCCCCC(I)CC(F)(F)C(F)(F)C(F)(F)C(F)(F)C(F)(F)C(F)(F)C(F)(F)F		-8.06	0.65	2.92	P	IN
QL01289	1,1,1,2,2,3,3,4,4-Nonafluoro-6-iodoundecane	40735-33-9	CCCCCC(I)CC(F)(F)C(F)(F)C(F)(F)C(F)(F)F		-6.69	0.62	2.55	P	OUT
QL01290	1,1,1,2,2,3,3-Heptafluoro-5-iododecane	755-49-7	CCCCCC(I)CC(F)(F)C(F)(F)C(F)(F)F		-6.18	0.61	2.46	P	OUT
QL01291	(2S)-2-(Nonafluorobutyl)octan-1-ol	1180131-16-1	CCCCCC[C@@H](CO)C(F)(F)C(F)(F)C(F)(F)C(F)(F)F		-6.39	0.61	2.53	P	OUT
QL01292	1,1,1,2,2,3,3,4,4-Nonafluoro-5-methylundec-5-ene	113999-55-6	CCCCCC=C(C)C(F)(F)C(F)(F)C(F)(F)C(F)(F)F		-6.71	0.62	2.55	P	OUT
QL01293	1,1,1,2,2,3,3,4,4,5,5,6-Dodecafluorododec-6-ene	92610-11-2	CCCCCC=C(F)C(F)(F)C(F)(F)C(F)(F)C(F)(F)C(F)(F)F		-7.31	0.63	2.72	P	OUT
QL01294	1,1,1,2,2,3,3,4-Octafluorodec-4-ene	90292-32-3	CCCCCC=C(F)C(F)(F)C(F)(F)C(F)(F)F		-6.20	0.61	2.46	P	OUT
QL01295	(6E)-8,8,9,9,10,10,11,11,12,12,13,13,14,14,15,15,15-Heptadecafluoropentadec-6-ene	350608-65-0	CCCCCC=CC(F)(F)C(F)(F)C(F)(F)C(F)(F)C(F)(F)C(F)(F)C(F)(F)C(F)(F)F		-8.20	0.65	2.95	P	IN
QL01296	8,8,9,9,10,10,11,11,12,12,13,13,14,14,14-Pentadecafluorotetradec-6-ene	38787-60-9	CCCCCC=CC(F)(F)C(F)(F)C(F)(F)C(F)(F)C(F)(F)C(F)(F)C(F)(F)F		-8.01	0.64	2.93	P	OUT
QL01297	(6E)-8,8,9,9,10,10,11,11,12,12,13,13,13-Tridecafluorotridec-6-ene	350608-60-5	CCCCCC=CC(F)(F)C(F)(F)C(F)(F)C(F)(F)C(F)(F)C(F)(F)F		-7.54	0.64	2.78	P	OUT
QL01298	1,1,1,2,2,3,3,4,4-Nonafluoroundec-5-ene	383145-46-8	CCCCCC=CC(F)(F)C(F)(F)C(F)(F)C(F)(F)F		-6.52	0.62	2.52	P	OUT
QL01299	(4E)-1,1,1,2,2,3,3-Heptafluorodec-4-ene	57325-42-5	CCCCCC=CC(F)(F)C(F)(F)C(F)(F)F		-5.97	0.60	2.44	P	OUT
QL01300	5-Pentyl-2-[4-(tridecafluorohexyl)phenyl]pyridine	139030-54-9	CCCCCc1ccc(nc1)-c1ccc(cc1)C(F)(F)C(F)(F)C(F)(F)C(F)(F)C(F)(F)C(F)(F)F		-8.33	0.63	3.31	P	OUT
QL01301	Octan-2-yl heptafluorobutanoate	560-01-0	CCCCCCC(C)OC(=O)C(F)(F)C(F)(F)C(F)(F)F		-6.53	0.61	2.70	P	OUT
QL01302	1-bromo-1,1,2,2-tetrafluorooctane	231630-92-5	CCCCCCC(F)(F)C(F)(F)Br		-5.04	0.59	2.16	P	OUT
QL01303	1,1,1,2,2,3,3,4,4,5,5,6,6,7,7,8,8,9,9,10,10,11,11,12,12-Pentacosafluorooctadecane	89109-70-6	CCCCCCC(F)(F)C(F)(F)C(F)(F)C(F)(F)C(F)(F)C(F)(F)C(F)(F)C(F)(F)C(F)(F)C(F)(F)C(F)(F)C(F)(F)F		-9.22	0.67	3.23	P	OUT
QL01304	1,1,1,2,2,3,3,4,4,5,5,6,6,7,7,8,8,9,9,10,10-Henicosafluorohexadecane	116177-54-9	CCCCCCC(F)(F)C(F)(F)C(F)(F)C(F)(F)C(F)(F)C(F)(F)C(F)(F)C(F)(F)C(F)(F)C(F)(F)F		-8.78	0.66	3.07	P	IN
QL01305	1,1,1,2,2,3,3,4,4,5,5,6,6,7,7,8,8-Heptadecafluorotetradecane	182130-14-9	CCCCCCC(F)(F)C(F)(F)C(F)(F)C(F)(F)C(F)(F)C(F)(F)C(F)(F)C(F)(F)F		-8.24	0.65	2.91	P	IN
QL01306	1,1,1,2,2,3,3,4,4,5,5,6,6,7,7-Pentadecafluorotridecane	169331-74-2	CCCCCCC(F)(F)C(F)(F)C(F)(F)C(F)(F)C(F)(F)C(F)(F)C(F)(F)F		-7.81	0.65	2.75	P	IN
QL01307	1,1,1,2,2,3,3,4,4,5,5,6,6-Tridecafluorododecane	69125-80-0	CCCCCCC(F)(F)C(F)(F)C(F)(F)C(F)(F)C(F)(F)C(F)(F)F		-7.58	0.64	2.72	P	IN
QL01308	1,1,1,2,2,3,3,4,4,5,5-Undecafluoroundecane	1287702-50-4	CCCCCCC(F)(F)C(F)(F)C(F)(F)C(F)(F)C(F)(F)F		-7.03	0.64	2.55	P	OUT
QL01309	1,1,1,2,2,3,3,4,4-Nonafluorodecane	1190430-19-3	CCCCCCC(F)(F)C(F)(F)C(F)(F)C(F)(F)F		-6.57	0.63	2.46	P	OUT
QL01310	1,1,1,2,2,3,3-Heptafluorononane	755-89-5	CCCCCCC(F)(F)C(F)(F)C(F)(F)F		-5.77	0.61	2.25	P	OUT
QL01311	(5E)-1,1,1,2,2,3,3,4,4-Nonafluoro-6-iodododec-5-ene	109574-88-1	CCCCCCC(I)=C/C(F)(F)C(F)(F)C(F)(F)C(F)(F)F		-6.84	0.62	2.70	P	OUT
QL01312	1,1,1,2,2,3,3,4,4,5,5,6,6,7,7,8,8,9,9,10,10,11,11,12,12-Pentacosafluoro-14-iodoicosane	93454-69-4	CCCCCCC(I)CC(F)(F)C(F)(F)C(F)(F)C(F)(F)C(F)(F)C(F)(F)C(F)(F)C(F)(F)C(F)(F)C(F)(F)C(F)(F)C(F)(F)F		-9.35	0.66	3.38	P	OUT
QL01313	1,1,1,2,2,3,3,4,4,5,5,6,6,7,7,8,8,9,9,10,10-Henicosafluoro-12-iodooctadecane	129838-40-0	CCCCCCC(I)CC(F)(F)C(F)(F)C(F)(F)C(F)(F)C(F)(F)C(F)(F)C(F)(F)C(F)(F)C(F)(F)C(F)(F)F		-8.92	0.65	3.24	P	OUT
QL01314	1,1,1,2,2,3,3,4,4,5,5,6,6,7,7,8,8-Heptadecafluoro-10-iodohexadecane	96791-81-0	CCCCCCC(I)CC(F)(F)C(F)(F)C(F)(F)C(F)(F)C(F)(F)C(F)(F)C(F)(F)C(F)(F)F		-8.42	0.65	3.10	P	OUT
QL01315	1,1,1,2,2,3,3,4,4,5,5,6,6,7,7-Pentadecafluoro-9-iodopentadecane	918-32-1	CCCCCCC(I)CC(F)(F)C(F)(F)C(F)(F)C(F)(F)C(F)(F)C(F)(F)C(F)(F)F		-8.02	0.64	2.97	P	OUT
QL01316	1,1,1,2,2,3,3,4,4,5,5,6,6-Tridecafluoro-8-iodotetradecane	109574-84-7	CCCCCCC(I)CC(F)(F)C(F)(F)C(F)(F)C(F)(F)C(F)(F)C(F)(F)F		-7.70	0.64	2.89	P	OUT
QL01317	Dodecane, 1,1,1,2,2,3,3,4,4-nonafluoro-6-iodo-	109603-73-8	CCCCCCC(I)CC(F)(F)C(F)(F)C(F)(F)C(F)(F)F		-6.84	0.62	2.70	P	OUT
QL01318	1,1,1,2,2,3,3-Heptafluoro-5-iodoundecane	23885-16-7	CCCCCCC(I)CC(F)(F)C(F)(F)C(F)(F)F		-6.14	0.60	2.52	P	OUT
QL01319	(7E)-9,9,10,10,11,11,12,12,13,13,14,14,15,15,16,16,16-Heptadecafluorohexadec-7-ene	84201-60-5	CCCCCCC=CC(F)(F)C(F)(F)C(F)(F)C(F)(F)C(F)(F)C(F)(F)C(F)(F)C(F)(F)F		-8.37	0.64	3.11	P	OUT
QL01320	1,1,1,2,2,3,3,4,4,5,5,6,6-Tridecafluorotetradec-7-ene	120464-26-8	CCCCCCC=CC(F)(F)C(F)(F)C(F)(F)C(F)(F)C(F)(F)C(F)(F)F		-7.64	0.63	2.90	P	OUT
QL01321	(5E)-1,1,1,2,2,3,3,4,4-Nonafluorododec-5-ene	113999-54-5	CCCCCCC=CC(F)(F)C(F)(F)C(F)(F)C(F)(F)F		-6.67	0.61	2.68	P	OUT
QL01322	1,1,1,2,2,3,3,4,4,5,5,6,6,7,7,8,8,9,9,10,10-Henicosafluoroheptadecane	200817-54-5	CCCCCCCC(F)(F)C(F)(F)C(F)(F)C(F)(F)C(F)(F)C(F)(F)C(F)(F)C(F)(F)C(F)(F)C(F)(F)F		-8.74	0.66	3.12	P	IN
QL01323	1,1,1,2,2,3,3,4,4,5,5,6,6,7,7,8,8-Heptadecafluoropentadecane	182130-15-0	CCCCCCCC(F)(F)C(F)(F)C(F)(F)C(F)(F)C(F)(F)C(F)(F)C(F)(F)C(F)(F)F		-8.20	0.65	2.95	P	IN

QL01324	1,1,1,2,2,3,3,4,4,5,5,6,6,7,7-Pentadecafluorotetradecane	38787-57-4	CCCCCCCC(F)(F)C(F)(F)C(F)(F)C(F)(F)C(F)(F)C(F)(F)C(F)(F)F		-8.01	0.64	2.93	P	OUT
QL01325	1,1,1,2,2,3,3,4,4,5,5,6,6-Tridecafluorotridecane	1835249-87-0	CCCCCCCC(F)(F)C(F)(F)C(F)(F)C(F)(F)C(F)(F)C(F)(F)F		-7.54	0.64	2.78	P	OUT
QL01326	1,1,1,2,2,3,3,4,4-Nonafluoroundecane	1190430-22-8	CCCCCCCC(F)(F)C(F)(F)C(F)(F)C(F)(F)F		-6.52	0.62	2.52	P	OUT
QL01327	1,1,1,2,2,3,3,4,4,5,5,6,6,7,7,8,8,9,9,10,10-Henicosafluoro-12-iodononadecane	129838-41-1	CCCCCCCC(I)CC(F)(F)C(F)(F)C(F)(F)C(F)(F)C(F)(F)C(F)(F)C(F)(F)C(F)(F)C(F)(F)C(F)(F)F		-8.89	0.65	3.28	P	OUT
QL01328	1,1,1,2,2,3,3,4,4-Nonafluorododecan-5-ol	131294-52-5	CCCCCCCC(O)C(F)(F)C(F)(F)C(F)(F)C(F)(F)F		-6.85	0.62	2.70	P	OUT
QL01329	N,N,N-Trimethyloctan-1-aminium pentadecafluorooctanoate	927835-01-6	CCCCCCCC[N+](C)(C)C.[O-]C(=O)C(F)(F)C(F)(F)C(F)(F)C(F)(F)C(F)(F)C(F)(F)C(F)(F)F		-6.50	0.64	2.28	P	IN
QL01330	1,1,1,2,2,3,3,4,4,5,5,6,6-Tridecafluoropentadec-7-ene	1980786-99-9	CCCCCCCC=CC(F)(F)C(F)(F)C(F)(F)C(F)(F)C(F)(F)C(F)(F)F		-7.60	0.63	2.95	P	OUT
QL01331	1,1,1,2,2,3,3,4,4,5,5,6,6,7,7,8,8,9,9,10,10,11,11,12,12-Pentacosafluoroicosane	90499-31-3	CCCCCCCCC(F)(F)C(F)(F)C(F)(F)C(F)(F)C(F)(F)C(F)(F)C(F)(F)C(F)(F)C(F)(F)C(F)(F)C(F)(F)C(F)(F)F		-9.32	0.66	3.38	P	OUT
QL01332	1,1,1,2,2,3,3,4,4,5,5,6,6,7,7,8,8,9,9,10,10-Henicosafluorooctadecane	93454-70-7	CCCCCCCCC(F)(F)C(F)(F)C(F)(F)C(F)(F)C(F)(F)C(F)(F)C(F)(F)C(F)(F)C(F)(F)C(F)(F)F		-8.89	0.65	3.25	P	OUT
QL01333	1,1,1,2,2,3,3,4,4-Nonafluorododecane	69125-79-7	CCCCCCCCC(F)(F)C(F)(F)C(F)(F)C(F)(F)F		-6.67	0.61	2.68	P	OUT
QL01334	1,1,1,2,2,3,3-Heptafluoroundecane	1190430-20-6	CCCCCCCCC(F)(F)C(F)(F)C(F)(F)F		-5.93	0.60	2.50	P	OUT
QL01335	1,1,1,2,2,3,3,4,4,5,5,6,6,7,7,8,8,9,9,10,10-Henicosafluoro-12-iodoicosane	121633-31-6	CCCCCCCCC(I)CC(F)(F)C(F)(F)C(F)(F)C(F)(F)C(F)(F)C(F)(F)C(F)(F)C(F)(F)C(F)(F)C(F)(F)F		-9.03	0.65	3.40	P	OUT
QL01336	1,1,1,2,2,3,3,4,4,5,5,6,6,7,7,8,8-Heptadecafluoro-10-iodooctadecane	176979-17-2	CCCCCCCCC(I)CC(F)(F)C(F)(F)C(F)(F)C(F)(F)C(F)(F)C(F)(F)C(F)(F)C(F)(F)F		-8.46	0.64	3.24	P	OUT
QL01337	1,1,1,2,2,3,3,4,4,5,5,6,6-Tridecafluoro-8-iodohexadecane	207122-00-7	CCCCCCCCC(I)CC(F)(F)C(F)(F)C(F)(F)C(F)(F)C(F)(F)C(F)(F)F		-7.82	0.63	3.08	P	OUT
QL01338	(Z)-Octadec-9-enyl (5-(((2-(((perfluorooctyl)sulphonyl)methylamino)ethoxy)carbonyl)amino)-o-tolyl)carbamate	94313-84-5	CCCCCCCCC=C/CCCCCCCCOC(=O)NC1=CC(NC(=O)OCCN(C)S(=O)(=O)C(F)(F)C(F)(F)C(F)(F)C(F)(F)C(F)(F)C(F)(F)C(F)(F)C(F)(F)F)=CC=C1C		-9.58	0.61	4.27	P	OUT
QL01339	(9E)-1,1,1,2,2,3,3,4,4,5,5,6,6,7,7,8,8-Heptadecafluorooctadec-9-ene	113999-61-4	CCCCCCCCC=CC(F)(F)C(F)(F)C(F)(F)C(F)(F)C(F)(F)C(F)(F)C(F)(F)C(F)(F)F		-8.42	0.64	3.24	P	OUT
QL01340	1,1,1,2,2,3,3,4,4,5,5,6,6,7,7-Pentadecafluoroheptadec-8-ene	52717-06-3	CCCCCCCCC=CC(F)(F)C(F)(F)C(F)(F)C(F)(F)C(F)(F)C(F)(F)C(F)(F)F		-8.02	0.63	3.13	P	OUT
QL01341	1,1,1,2,2,3,3,4,4,5,5,6,6-tridecafluorohexadec-7-ene	120464-27-9	CCCCCCCCC=CC(F)(F)C(F)(F)C(F)(F)C(F)(F)C(F)(F)C(F)(F)F		-7.71	0.62	3.06	P	OUT
QL01343	(5E)-1,1,1,2,2,3,3,4,4-Nonafluorotetradec-5-ene	104504-30-5	CCCCCCCCC=CC(F)(F)C(F)(F)C(F)(F)C(F)(F)F		-6.78	0.60	2.87	P	OUT
QL01344	Carbamic acid, [5-[[[2-[[(heptadecafluoroisooctyl)sulfonyl]methylamino]ethoxy]carbonyl]amino]-2-methylphenyl]-, 9-octadecenyl ester	93894-74-7	CCCCCCCCC=CCCCCCCCCOC(=O)NC1=CC(NC(=O)OCCN(C)S(=O)(=O)C(F)(F)C(F)(F)C(F)(F)C(F)(F)C(F)(F)C(F)(C(F)(F)F)C(F)(F)F)=CC=C1C		-9.44	0.60	4.22	P	OUT
QL01345	1,1,1,2,2,3,3,4,4,5,5,6,6,7,7,8,8,9,9,10,10,11,11,12,12,13,13-Heptacosafluorodocosane	1309378-06-0	CCCCCCCCCC(F)(F)C(F)(F)C(F)(F)C(F)(F)C(F)(F)C(F)(F)C(F)(F)C(F)(F)C(F)(F)C(F)(F)C(F)(F)C(F)(F)C(F)(F)F		-9.55	0.66	3.51	P	OUT
QL01346	1,1,1,2,2,3,3,4,4,5,5,6,6,7,7,8,8,9,9,10,10,11,11,12,12-Pentacosafluorohenicosane	677324-30-0	CCCCCCCCCC(F)(F)C(F)(F)C(F)(F)C(F)(F)C(F)(F)C(F)(F)C(F)(F)C(F)(F)C(F)(F)C(F)(F)C(F)(F)C(F)(F)F		-9.29	0.66	3.41	P	OUT
QL01347	1,1,1,2,2,3,3,4,4,5,5,6,6,7,7,8,8,9,9,10,10-Henicosafluorononadecane	125635-85-0	CCCCCCCCCC(F)(F)C(F)(F)C(F)(F)C(F)(F)C(F)(F)C(F)(F)C(F)(F)C(F)(F)C(F)(F)C(F)(F)F		-8.85	0.65	3.28	P	OUT
QL01348	1,1,1,2,2,3,3,4,4,5,5,6,6,7,7,8,8,9,9-Nonadecafluorooctadecane	1287702-47-9	CCCCCCCCCC(F)(F)C(F)(F)C(F)(F)C(F)(F)C(F)(F)C(F)(F)C(F)(F)C(F)(F)C(F)(F)F		-8.70	0.65	3.26	P	OUT
QL01349	1,1,1,2,2,3,3,4,4,5,5,6,6,7,7,8,8-Heptadecafluoroheptadecane	931415-52-0	CCCCCCCCCC(F)(F)C(F)(F)C(F)(F)C(F)(F)C(F)(F)C(F)(F)C(F)(F)C(F)(F)F		-8.34	0.64	3.15	P	OUT
QL01350	1,1,1,2,2,3,3,4,4,5,5,6,6-Tridecafluoropentadecane	113659-13-5	CCCCCCCCCC(F)(F)C(F)(F)C(F)(F)C(F)(F)C(F)(F)C(F)(F)F		-7.60	0.63	2.95	P	OUT
QL01351	1,1,1,2,2,3,3,4,4-Nonafluorotridecane	234433-63-7	CCCCCCCCCC(F)(F)C(F)(F)C(F)(F)C(F)(F)F		-6.63	0.61	2.73	P	OUT
QL01352	1,1,1,2,2,3,3-Heptafluorododecane	69125-78-6	CCCCCCCCCC(F)(F)C(F)(F)C(F)(F)F		-6.11	0.59	2.67	P	OUT
QL01353	1,1,1,2,2,3,3,4,4,5,5,6,6,7,7,8,8,9,9,10,10-Henicosafluoro-12-iodohenicosane	129838-42-2	CCCCCCCCCC(I)CC(F)(F)C(F)(F)C(F)(F)C(F)(F)C(F)(F)C(F)(F)C(F)(F)C(F)(F)C(F)(F)C(F)(F)F		-9.00	0.64	3.44	P	OUT
QL01354	1,1,1,2,2,3,3,4,4,5,5,6,6-Tridecafluoro-8-iodoheptadecane	207122-01-8	CCCCCCCCCC(I)CC(F)(F)C(F)(F)C(F)(F)C(F)(F)C(F)(F)C(F)(F)F		-7.79	0.62	3.12	P	OUT
QL01355	N-Decyl-N,N-dimethyl-1-decanaminium perfluorooctanesulfonate	251099-16-8	CCCCCCCCCC[N+](C)(C)CCCCCCCCCC.[O-]S(=O)(=O)C(F)(F)C(F)(F)C(F)(F)C(F)(F)C(F)(F)C(F)(F)C(F)(F)C(F)(F)F		-8.81	0.61	3.77	P	OUT
QL01356	N,N,N-Triethyldecan-1-aminium heptadecafluorooctane-1-sulfonate	773895-92-4	CCCCCCCCCC[N+](CC)(CC)CC.[O-]S(=O)(=O)C(F)(F)C(F)(F)C(F)(F)C(F)(F)C(F)(F)C(F)(F)C(F)(F)C(F)(F)F		-7.37	0.63	2.84	P	OUT
QL01357	1,1,1,2,2,3,3,4,4,5,5,6,6,7,7,8,8,9,9,10,10,11,11,12,12-Pentacosafluorodocosane	93454-72-9	CCCCCCCCCCC(F)(F)C(F)(F)C(F)(F)C(F)(F)C(F)(F)C(F)(F)C(F)(F)C(F)(F)C(F)(F)C(F)(F)C(F)(F)C(F)(F)F		-9.41	0.65	3.52	P	OUT

QL01358	1,1,1,2,2,3,3,4,4,5,5,6,6,7,7,8,8,9,9,10,10-Henicosafluoroicosane	90499-29-9	CCCCCCCCCCC(F)(F)C(F)(F)C(F)(F)C(F)(F)C(F)(F)C(F)(F)C(F)(F)C(F)(F)C(F)(F)C(F)(F)F		-8.99	0.65	3.41	P	OUT
QL01359	1-(Perfluoro-n-octyl)decane	138472-76-1	CCCCCCCCCCC(F)(F)C(F)(F)C(F)(F)C(F)(F)C(F)(F)C(F)(F)C(F)(F)C(F)(F)F		-8.42	0.64	3.24	P	OUT
QL01360	1,1,1,2,2,3,3,4,4,5,5,6,6--Tridecafluorohexadecane	147492-59-9	CCCCCCCCCCC(F)(F)C(F)(F)C(F)(F)C(F)(F)C(F)(F)C(F)(F)F		-7.71	0.62	3.06	P	OUT
QL01361	1,1,1,2,2,3,3,4,4-Nonafluorotetradecane	214196-02-8	CCCCCCCCCCC(F)(F)C(F)(F)C(F)(F)C(F)(F)F		-6.78	0.60	2.87	P	OUT
QL01362	1,1,1,2,2,3,3,4,4,5,5,6,6,7,7,8,8,9,9,10,10-Henicosafluoro-12-iodododocosane	129838-43-3	CCCCCCCCCCC(I)CC(F)(F)C(F)(F)C(F)(F)C(F)(F)C(F)(F)C(F)(F)C(F)(F)C(F)(F)C(F)(F)C(F)(F)F		-9.06	0.64	3.51	P	OUT
QL01363	1,1,1,2,2,3,3,4,4,5,5,6,6,7,7,8,8-Heptadecafluoro-10-iodoicosane	1257261-93-0	CCCCCCCCCCC(I)CC(F)(F)C(F)(F)C(F)(F)C(F)(F)C(F)(F)C(F)(F)C(F)(F)C(F)(F)F		-8.55	0.63	3.39	P	OUT
QL01364	1,1,1,2,2,3,3,4,4,5,5,6,6,7,7-Pentadecafluoro-9-iodononadecane	25431-52-1	CCCCCCCCCCC(I)CC(F)(F)C(F)(F)C(F)(F)C(F)(F)C(F)(F)C(F)(F)C(F)(F)F		-8.18	0.63	3.28	P	OUT
QL01365	1,1,1,2,2,3,3,4,4,5,5,6,6-Tridecafluoro-8-iodooctadecane	125081-35-8	CCCCCCCCCCC(I)CC(F)(F)C(F)(F)C(F)(F)C(F)(F)C(F)(F)C(F)(F)F		-7.89	0.62	3.23	P	OUT
QL01366	1,1,1,2,2,3,3,4,4-Nonafluoro-6-iodohexadecane	1789721-11-4	CCCCCCCCCCC(I)CC(F)(F)C(F)(F)C(F)(F)C(F)(F)F		-7.04	0.60	3.06	P	OUT
QL01367	(9E)-1,1,1,2,2,3,3,4,4,5,5,6,6,7,7,8,8-Heptadecafluoroicos-9-ene	124921-25-1	CCCCCCCCCCC=CC(F)(F)C(F)(F)C(F)(F)C(F)(F)C(F)(F)C(F)(F)C(F)(F)C(F)(F)F		-8.46	0.63	3.37	P	OUT
QL01368	(7E)-1,1,1,2,2,3,3,4,4,5,5,6,6-Tridecafluorooctadec-7-ene	125081-49-4	CCCCCCCCCCC=CC(F)(F)C(F)(F)C(F)(F)C(F)(F)C(F)(F)C(F)(F)F		-7.78	0.62	3.21	P	OUT
QL01369	1,1,1,2,2,3,3,4,4-Nonafluorohexadec-5-ene	131851-20-2	CCCCCCCCCCC=CC(F)(F)C(F)(F)C(F)(F)C(F)(F)F		-6.89	0.60	3.04	P	OUT
QL01370	1,1,1,2,2,3,3,4,4,5,5,6,6,7,7,8,8,9,9,10,10,11,11,12,12-Pentacosafluorotricosane	677324-31-1	CCCCCCCCCCCC(F)(F)C(F)(F)C(F)(F)C(F)(F)C(F)(F)C(F)(F)C(F)(F)C(F)(F)C(F)(F)C(F)(F)C(F)(F)C(F)(F)F		-9.38	0.65	3.55	P	OUT
QL01371	1,1,1,2,2,3,3,4,4,5,5,6,6,7,7,8,8,9,9,10,10-Henicosafluorohenicosane	125635-86-1	CCCCCCCCCCCC(F)(F)C(F)(F)C(F)(F)C(F)(F)C(F)(F)C(F)(F)C(F)(F)C(F)(F)C(F)(F)C(F)(F)F		-8.96	0.64	3.44	P	OUT
QL01372	1,1,1,2,2,3,3,4,4,5,5,6,6,7,7,8,8-Heptadecafluorononadecane	931415-53-1	CCCCCCCCCCCC(F)(F)C(F)(F)C(F)(F)C(F)(F)C(F)(F)C(F)(F)C(F)(F)C(F)(F)F		-8.39	0.63	3.28	P	OUT
QL01373	Tetradecanoic acid, 1,1'-(methyl(3,3,4,4,5,5,6,6,6-nonafluorohexyl)silylene) ester	94094-26-5	CCCCCCCCCCCCC(C(O)=O)[Si](C)(CCC(F)(F)C(F)(F)C(F)(F)C(F)(F)F)C(CCCCCCCCCCCC)C(O)=O		-7.47	0.58	3.62	P	OUT
QL01374	1,1,1,2,2,3,3,4,4,5,5,6,6,7,7,8,8,9,9,10,10,11,11,12,12,13,13-Heptacosafluoropentacosane	677324-22-0	CCCCCCCCCCCCC(F)(F)C(F)(F)C(F)(F)C(F)(F)C(F)(F)C(F)(F)C(F)(F)C(F)(F)C(F)(F)C(F)(F)C(F)(F)C(F)(F)C(F)(F)F		-9.61	0.65	3.67	P	OUT
QL01375	1,1,1,2,2,3,3,4,4,5,5,6,6,7,7,8,8,9,9,10,10,11,11,12,12-Pentacosafluorotetracosane	89109-71-7	CCCCCCCCCCCCC(F)(F)C(F)(F)C(F)(F)C(F)(F)C(F)(F)C(F)(F)C(F)(F)C(F)(F)C(F)(F)C(F)(F)C(F)(F)C(F)(F)F		-9.50	0.65	3.66	P	OUT
QL01376	1,1,1,2,2,3,3,4,4,5,5,6,6,7,7,8,8,9,9,10,10,11,11-Tricosafluorotricosane	677324-21-9	CCCCCCCCCCCCC(F)(F)C(F)(F)C(F)(F)C(F)(F)C(F)(F)C(F)(F)C(F)(F)C(F)(F)C(F)(F)C(F)(F)C(F)(F)F		-9.23	0.64	3.57	P	OUT
QL01377	1,1,1,2,2,3,3,4,4,5,5,6,6,7,7,8,8,9,9,10,10-Henicosafluorodocosane	93454-71-8	CCCCCCCCCCCCC(F)(F)C(F)(F)C(F)(F)C(F)(F)C(F)(F)C(F)(F)C(F)(F)C(F)(F)C(F)(F)C(F)(F)F		-9.03	0.64	3.52	P	OUT
QL01378	1,1,1,2,2,3,3,4,4,5,5,6,6,7,7,8,8,9,9-Nonadecafluorohenicosane	677324-20-8	CCCCCCCCCCCCC(F)(F)C(F)(F)C(F)(F)C(F)(F)C(F)(F)C(F)(F)C(F)(F)C(F)(F)C(F)(F)F		-8.71	0.63	3.42	P	OUT
QL01379	1-(Perfluoro-n-octyl)dodecane	106873-67-0	CCCCCCCCCCCCC(F)(F)C(F)(F)C(F)(F)C(F)(F)C(F)(F)C(F)(F)C(F)(F)C(F)(F)F		-8.46	0.63	3.37	P	OUT
QL01380	1,1,1,2,2,3,3,4,4,5,5,6,6,7,7-Pentadecafluorononadecane	109856-51-1	CCCCCCCCCCCCC(F)(F)C(F)(F)C(F)(F)C(F)(F)C(F)(F)C(F)(F)C(F)(F)F		-8.08	0.62	3.26	P	OUT
QL01381	1-(Perfluoro-n-hexyl)dodecane	90499-30-2	CCCCCCCCCCCCC(F)(F)C(F)(F)C(F)(F)C(F)(F)C(F)(F)C(F)(F)F		-7.78	0.62	3.21	P	OUT
QL01382	1,1,1,2,2,3,3,4,4-Nonafluorohexadecane	106873-68-1	CCCCCCCCCCCCC(F)(F)C(F)(F)C(F)(F)C(F)(F)F		-6.89	0.60	3.04	P	OUT
QL01383	1,1,1,2,2,3,3-Heptafluoropentadecane	106873-69-2	CCCCCCCCCCCCC(F)(F)C(F)(F)C(F)(F)F		-6.24	0.58	2.91	P	OUT
QL01384	1,1,1,2,2,3,3,4,4,5,5,6,6,7,7,8,8,9,9,10,10-Henicosafluoro-12-iodotetracosane	1257261-92-9	CCCCCCCCCCCCC(I)CC(F)(F)C(F)(F)C(F)(F)C(F)(F)C(F)(F)C(F)(F)C(F)(F)C(F)(F)C(F)(F)C(F)(F)F		-9.14	0.63	3.64	P	OUT
QL01385	1,1,1,2,2,3,3,4,4,5,5,6,6,7,7,8,8-Heptadecafluoro-10-iodododocosane	176979-18-3	CCCCCCCCCCCCC(I)CC(F)(F)C(F)(F)C(F)(F)C(F)(F)C(F)(F)C(F)(F)C(F)(F)C(F)(F)F		-8.60	0.62	3.50	P	OUT
QL01386	2-(o-Cyanophenoxy)-3'-hydroxy-4'-(perfluorobutyryl)aminotetradecananilide	117844-18-5	CCCCCCCCCCCCC(OC1=CC=CC=C1C#N)C(O)=N/C1=CC(O)=C(NC(=O)C(F)(F)C(F)(F)C(F)(F)F)C=C1		-7.60	0.58	3.59	P	OUT
QL01387	1,1,1,2,2,3,3,4,4,5,5,6,6,7,7,8,8-Heptadecafluorodocos-9-ene	118642-84-5	CCCCCCCCCCCCC=CC(F)(F)C(F)(F)C(F)(F)C(F)(F)C(F)(F)C(F)(F)C(F)(F)C(F)(F)F		-8.51	0.62	3.49	P	OUT
QL01388	1,1,1,2,2,3,3,4,4,5,5,6,6-Tridecafluoroicos-7-ene	160460-04-8	CCCCCCCCCCCCC=CC(F)(F)C(F)(F)C(F)(F)C(F)(F)C(F)(F)C(F)(F)F		-7.85	0.61	3.35	P	OUT
QL01389	1,1,1,2,2,3,3,4,4-Nonafluorooctadec-5-ene	160460-03-7	CCCCCCCCCCCCC=CC(F)(F)C(F)(F)C(F)(F)C(F)(F)F		-7.00	0.59	3.20	P	OUT
QL01390	1,1,1,2,2,3,3,4,4,5,5,6,6,7,7,8,8,9,9,10,10,11,11,12,12-Pentacosafluoropentacosane	116177-47-0	CCCCCCCCCCCCCC(F)(F)C(F)(F)C(F)(F)C(F)(F)C(F)(F)C(F)(F)C(F)(F)C(F)(F)C(F)(F)C(F)(F)C(F)(F)C(F)(F)F		-9.47	0.64	3.68	P	OUT
QL01391	1,1,1,2,2,3,3,4,4,5,5,6,6,7,7,8,8,9,9,10,10-Henicosafluorotricosane	200817-55-6	CCCCCCCCCCCCCC(F)(F)C(F)(F)C(F)(F)C(F)(F)C(F)(F)C(F)(F)C(F)(F)C(F)(F)C(F)(F)C(F)(F)F		-9.00	0.64	3.55	P	OUT
QL01392	1,1,1,2,2,3,3,4,4,5,5,6,6,7,7,8,8-Heptadecafluorohenicosane	871311-23-8	CCCCCCCCCCCCCC(F)(F)C(F)(F)C(F)(F)C(F)(F)C(F)(F)C(F)(F)C(F)(F)C(F)(F)F		-8.44	0.63	3.41	P	OUT
QL01394	1,1,1,2,2,3,3,4,4,5,5,6,6,7,7,8,8,9,9,10,10,11,11,12,12-Pentacosafluorohexacosane	93454-73-0	CCCCCCCCCCCCCCC(F)(F)C(F)(F)C(F)(F)C(F)(F)C(F)(F)C(F)(F)C(F)(F)C(F)(F)C(F)(F)C(F)(F)C(F)(F)C(F)(F)F		-9.53	0.64	3.75	P	OUT

QL01395	1,1,1,2,2,3,3,4,4,5,5,6,6,7,7,8,8,9,9,10,10-Henicosafluorotetracosane	200817-56-7	CCCCCCCCCCCCCCC(F)(F)C(F)(F)C(F)(F)C(F)(F)C(F)(F)C(F)(F)C(F)(F)C(F)(F)C(F)(F)C(F)(F)F		-9.06	0.63	3.63	P	OUT
QL01396	1-(Perfluoro-n-octyl)tetradecane	133310-72-2	CCCCCCCCCCCCCCC(F)(F)C(F)(F)C(F)(F)C(F)(F)C(F)(F)C(F)(F)C(F)(F)C(F)(F)F		-8.51	0.62	3.49	P	OUT
QL01397	1,1,1,2,2,3,3,4,4,5,5,6,6-Tridecafluoroicosane	154628-00-9	CCCCCCCCCCCCCCC(F)(F)C(F)(F)C(F)(F)C(F)(F)C(F)(F)C(F)(F)F		-7.85	0.61	3.35	P	OUT
QL01398	1,1,1,2,2,3,3,4,4,5,5-Undecafluorononadecane	112344-48-6	CCCCCCCCCCCCCCC(F)(F)C(F)(F)C(F)(F)C(F)(F)C(F)(F)F		-7.38	0.60	3.24	P	OUT
QL01399	1,1,1,2,2,3,3,4,4-Nonafluorooctadecane	133310-68-6	CCCCCCCCCCCCCCC(F)(F)C(F)(F)C(F)(F)C(F)(F)F		-7.00	0.59	3.20	P	OUT
QL01400	1,1,1,2,2,3,3,4,4,5,5,6,6,7,7,8,8,9,9,10,10-Henicosafluoro-12-iodohexacosane	1257261-91-8	CCCCCCCCCCCCCCC(I)CC(F)(F)C(F)(F)C(F)(F)C(F)(F)C(F)(F)C(F)(F)C(F)(F)C(F)(F)C(F)(F)C(F)(F)F		-9.17	0.63	3.74	P	OUT
QL01401	1,1,1,2,2,3,3,4,4,5,5,6,6,7,7,8,8-Heptadecafluoro-10-iodotetracosane	1257261-94-1	CCCCCCCCCCCCCCC(I)CC(F)(F)C(F)(F)C(F)(F)C(F)(F)C(F)(F)C(F)(F)C(F)(F)C(F)(F)F		-8.65	0.62	3.61	P	OUT
QL01402	1,1,1,2,2,3,3,4,4,5,5,6,6,7,7-Pentadecafluoro-9-iodotricosane	57325-43-6	CCCCCCCCCCCCCCC(I)CC(F)(F)C(F)(F)C(F)(F)C(F)(F)C(F)(F)C(F)(F)C(F)(F)F		-8.30	0.61	3.52	P	OUT
QL01403	1,1,1,2,2,3,3,4,4,5,5,6,6,7,7,8,8,9,9,10,10,11,11,12,12-Pentacosafluorooctacos-13-ene	1151525-71-1	CCCCCCCCCCCCCCC=CC(F)(F)C(F)(F)C(F)(F)C(F)(F)C(F)(F)C(F)(F)C(F)(F)C(F)(F)C(F)(F)C(F)(F)C(F)(F)C(F)(F)F		-9.56	0.64	3.84	P	OUT
QL01404	1,1,1,2,2,3,3,4,4,5,5,6,6,7,7,8,8,9,9,10,10-Henicosafluorohexacos-11-ene	1244062-18-7	CCCCCCCCCCCCCCC=CC(F)(F)C(F)(F)C(F)(F)C(F)(F)C(F)(F)C(F)(F)C(F)(F)C(F)(F)C(F)(F)C(F)(F)F		-9.10	0.63	3.72	P	OUT
QL01405	1,1,1,2,2,3,3,4,4,5,5,6,6,7,7,8,8-Heptadecafluorotetracos-9-ene	1244062-16-5	CCCCCCCCCCCCCCC=CC(F)(F)C(F)(F)C(F)(F)C(F)(F)C(F)(F)C(F)(F)C(F)(F)C(F)(F)F		-8.57	0.62	3.60	P	OUT
QL01406	1,1,1,2,2,3,3,4,4,5,5,6,6-Tridecafluorodocos-7-ene	1244062-15-4	CCCCCCCCCCCCCCC=CC(F)(F)C(F)(F)C(F)(F)C(F)(F)C(F)(F)C(F)(F)F		-7.92	0.60	3.47	P	OUT
QL01407	2,2,3,3,4,4,5,5,6,6,7,7,8,8,8-pentadecafluorooctyl hexadecanoate	111918-97-9	CCCCCCCCCCCCCCCC(=O)OCC(F)(F)C(F)(F)C(F)(F)C(F)(F)C(F)(F)C(F)(F)C(F)(F)F		-8.40	0.61	3.63	P	OUT
QL01408	1,1,1,2,2,3,3,4,4,5,5,6,6,7,7,8,8,9,9,10,10,11,11,12,12-Pentacosafluoroheptacosane	116177-48-1	CCCCCCCCCCCCCCCC(F)(F)C(F)(F)C(F)(F)C(F)(F)C(F)(F)C(F)(F)C(F)(F)C(F)(F)C(F)(F)C(F)(F)C(F)(F)C(F)(F)F		-9.50	0.64	3.78	P	OUT
QL01409	1,1,1,2,2,3,3,4,4,5,5,6,6,7,7,8,8,9,9,10,10-Henicosafluoropentacosane	250738-43-3	CCCCCCCCCCCCCCCC(F)(F)C(F)(F)C(F)(F)C(F)(F)C(F)(F)C(F)(F)C(F)(F)C(F)(F)C(F)(F)C(F)(F)F		-9.04	0.63	3.65	P	OUT
QL01410	1,1,1,2,2,3,3,4,4,5,5,6,6,7,7,8,8,9,9-Nonadecafluorotetracosane	113659-16-8	CCCCCCCCCCCCCCCC(F)(F)C(F)(F)C(F)(F)C(F)(F)C(F)(F)C(F)(F)C(F)(F)C(F)(F)C(F)(F)F		-8.82	0.63	3.61	P	OUT
QL01411	1,1,1,2,2,3,3,4,4,5,5,6,6,7,7,8,8-Heptadecafluorotricosane	871311-24-9	CCCCCCCCCCCCCCCC(F)(F)C(F)(F)C(F)(F)C(F)(F)C(F)(F)C(F)(F)C(F)(F)C(F)(F)F		-8.49	0.62	3.52	P	OUT
QL01412	1-(Perfluoro-n-octyl)hexadecane	117146-18-6	CCCCCCCCCCCCCCCCC(C(C(C(C(C(C(C(F)(F)F)(F)F)(F)F)(F)F)(F)F)(F)F)(F)F)(F)F		-8.57	0.62	3.60	P	OUT
QL01413	1,1,1,2,2,3,3,4,4,5,5,6,6,7,7,8,8,9,9,10,10,11,11,12,12,13,13,14,14-Nonacosafluorotriacontane	1228093-93-3	CCCCCCCCCCCCCCCCC(F)(F)C(F)(F)C(F)(F)C(F)(F)C(F)(F)C(F)(F)C(F)(F)C(F)(F)C(F)(F)C(F)(F)C(F)(F)C(F)(F)C(F)(F)C(F)(F)F		-9.95	0.64	3.95	P	OUT
QL01414	1,1,1,2,2,3,3,4,4,5,5,6,6,7,7,8,8,9,9,10,10,11,11,12,12-Pentacosafluorooctacosane	93454-74-1	CCCCCCCCCCCCCCCCC(F)(F)C(F)(F)C(F)(F)C(F)(F)C(F)(F)C(F)(F)C(F)(F)C(F)(F)C(F)(F)C(F)(F)C(F)(F)C(F)(F)F		-9.56	0.64	3.84	P	OUT
QL01415	1,1,1,2,2,3,3,4,4,5,5,6,6,7,7,8,8,9,9,10,10,11,11-Tricosafluoroheptacosane	1980054-34-9	CCCCCCCCCCCCCCCCC(F)(F)C(F)(F)C(F)(F)C(F)(F)C(F)(F)C(F)(F)C(F)(F)C(F)(F)C(F)(F)C(F)(F)C(F)(F)F		-9.30	0.63	3.76	P	OUT
QL01416	1,1,1,2,2,3,3,4,4,5,5,6,6,7,7,8,8,9,9,10,10-Henicosafluorohexacosane	167641-85-2	CCCCCCCCCCCCCCCCC(F)(F)C(F)(F)C(F)(F)C(F)(F)C(F)(F)C(F)(F)C(F)(F)C(F)(F)C(F)(F)C(F)(F)F		-9.10	0.63	3.72	P	OUT
QL01417	1,1,1,2,2,3,3,4,4,5,5,6,6,7,7-Pentadecafluorotricosane	57325-44-7	CCCCCCCCCCCCCCCCC(F)(F)C(F)(F)C(F)(F)C(F)(F)C(F)(F)C(F)(F)C(F)(F)F		-8.21	0.61	3.51	P	OUT
QL01418	1-(Perfluorohexyl)hexadecane	133310-71-1	CCCCCCCCCCCCCCCCC(F)(F)C(F)(F)C(F)(F)C(F)(F)C(F)(F)C(F)(F)F		-7.92	0.60	3.47	P	OUT
QL01419	1,1,1,2,2,3,3,4,4-Nonafluoroicosane	133310-69-7	CCCCCCCCCCCCCCCCC(F)(F)C(F)(F)C(F)(F)C(F)(F)F		-7.11	0.58	3.34	P	OUT
QL01420	1,1,1,2,2,3,3,4,4,5,5,6,6,7,7,8,8,9,9,10,10-Henicosafluoro-12-iodooctacosane	1257261-90-7	CCCCCCCCCCCCCCCCC(I)CC(F)(F)C(F)(F)C(F)(F)C(F)(F)C(F)(F)C(F)(F)C(F)(F)C(F)(F)C(F)(F)C(F)(F)F		-9.21	0.62	3.83	P	OUT
QL01421	1,1,1,2,2,3,3,4,4,5,5,6,6,7,7,8,8-Heptadecafluoro-10-iodohexacosane	176979-19-4	CCCCCCCCCCCCCCCCC(I)CC(F)(F)C(F)(F)C(F)(F)C(F)(F)C(F)(F)C(F)(F)C(F)(F)C(F)(F)F		-8.70	0.61	3.72	P	OUT
QL01422	1,1,1,2,2,3,3,4,4,5,5,6,6,7,7-Pentadecafluoro-9-iodopentacosane	2837-34-5	CCCCCCCCCCCCCCCCC(I)CC(F)(F)C(F)(F)C(F)(F)C(F)(F)C(F)(F)C(F)(F)C(F)(F)F		-8.37	0.61	3.63	P	OUT
QL01423	1,1,1,2,2,3,3,4,4,5,5,6,6,7,7,8,8-Heptadecafluorohexacos-9-ene	907206-01-3	CCCCCCCCCCCCCCCCC=CC(F)(F)C(F)(F)C(F)(F)C(F)(F)C(F)(F)C(F)(F)C(F)(F)C(F)(F)F		-8.62	0.61	3.71	P	OUT
QL01424	1,1,1,2,2,3,3,4,4,5,5,6,6,7,7,8,8,9,9,10,10,11,11,12,12-Pentacosafluorononacosane	677324-32-2	CCCCCCCCCCCCCCCCCC(F)(F)C(F)(F)C(F)(F)C(F)(F)C(F)(F)C(F)(F)C(F)(F)C(F)(F)C(F)(F)C(F)(F)C(F)(F)C(F)(F)F		-9.54	0.63	3.87	P	OUT
QL01425	1,1,1,2,2,3,3,4,4,5,5,6,6,7,7,8,8,9,9,10,10-Henicosafluoroheptacosane	250738-44-4	CCCCCCCCCCCCCCCCCC(F)(F)C(F)(F)C(F)(F)C(F)(F)C(F)(F)C(F)(F)C(F)(F)C(F)(F)C(F)(F)C(F)(F)F		-9.08	0.62	3.75	P	OUT
QL01426	1,1,1,2,2,3,3,4,4,5,5,6,6,7,7,8,8-Heptadecafluoropentacosane	871311-25-0	CCCCCCCCCCCCCCCCCC(F)(F)C(F)(F)C(F)(F)C(F)(F)C(F)(F)C(F)(F)C(F)(F)C(F)(F)F		-8.55	0.61	3.63	P	OUT
QL01427	1,1,1,2,2,3,3,4,4,5,5,6,6-Tridecafluorotricosane	863126-43-6	CCCCCCCCCCCCCCCCCC(F)(F)C(F)(F)C(F)(F)C(F)(F)C(F)(F)C(F)(F)F		-7.90	0.60	3.50	P	OUT
QL01428	1,1,1,2,2,3,3,4,4,5,5,6,6,7,7,8,8,9,9,10,10,11,11,12,12-Pentacosafluorotriacontane	93454-75-2	CCCCCCCCCCCCCCCCCCC(F)(F)C(F)(F)C(F)(F)C(F)(F)C(F)(F)C(F)(F)C(F)(F)C(F)(F)C(F)(F)C(F)(F)C(F)(F)C(F)(F)F		-9.59	0.63	3.93	P	OUT
QL01429	1,1,1,2,2,3,3,4,4,5,5,6,6,7,7,8,8,9,9,10,10-Henicosafluorooctacosane	250738-45-5	CCCCCCCCCCCCCCCCCCC(F)(F)C(F)(F)C(F)(F)C(F)(F)C(F)(F)C(F)(F)C(F)(F)C(F)(F)C(F)(F)C(F)(F)F		-9.14	0.62	3.82	P	OUT

QL01430	1,1,1,2,2,3,3,4,4,5,5,6,6,7,7,8,8,9,9-Nonadecafluoroheptacosane	117948-74-0	CCCCCCCCCCCCCCCCCCC(F)(F)C(F)(F)C(F)(F)C(F)(F)C(F)(F)C(F)(F)C(F)(F)C(F)(F)C(F)(F)F		-8.85	0.62	3.74	P	OUT
QL01431	1,1,1,2,2,3,3,4,4,5,5,6,6,7,7,8,8-Heptadecafluorohexacosane	133310-73-3	CCCCCCCCCCCCCCCCCCC(F)(F)C(F)(F)C(F)(F)C(F)(F)C(F)(F)C(F)(F)C(F)(F)C(F)(F)F		-8.62	0.61	3.71	P	OUT
QL01432	1,1,1,2,2,3,3,4,4,5,5,6,6-Tridecafluorotetracosane	154628-01-0	CCCCCCCCCCCCCCCCCCC(F)(F)C(F)(F)C(F)(F)C(F)(F)C(F)(F)C(F)(F)F		-7.99	0.60	3.59	P	OUT
QL01433	1,1,1,2,2,3,3,4,4-Nonafluorodocosane	133310-70-0	CCCCCCCCCCCCCCCCCCC(F)(F)C(F)(F)C(F)(F)C(F)(F)F		-7.21	0.58	3.47	P	OUT
QL01434	1,1,1,2,2,3,3,4,4,5,5,6,6,7,7,8,8,9,9,10,10-Henicosafluoro-12-iodotriacontane	1257261-89-4	CCCCCCCCCCCCCCCCCC(I)CC(F)(F)C(F)(F)C(F)(F)C(F)(F)C(F)(F)C(F)(F)C(F)(F)C(F)(F)C(F)(F)C(F)(F)F		-9.25	0.62	3.92	P	OUT
QL01435	1,1,1,2,2,3,3,4,4,5,5,6,6,7,7,8,8-Heptadecafluoro-10-iodooctacosane	1257261-95-2	CCCCCCCCCCCCCCCCCC(I)CC(F)(F)C(F)(F)C(F)(F)C(F)(F)C(F)(F)C(F)(F)C(F)(F)C(F)(F)F		-8.76	0.61	3.81	P	OUT
QL01436	1,1,1,2,2,3,3,4,4,5,5,6,6,7,7,8,8,9,9,10,10,11,11,12,12-Pentacosafluorohentriacontane	264229-79-0	CCCCCCCCCCCCCCCCCCCC(F)(F)C(F)(F)C(F)(F)C(F)(F)C(F)(F)C(F)(F)C(F)(F)C(F)(F)C(F)(F)C(F)(F)C(F)(F)C(F)(F)F		-9.57	0.63	3.95	P	OUT
QL01437	1,1,1,2,2,3,3,4,4,5,5,6,6,7,7,8,8,9,9,10,10-Henicosafluorononacosane	250738-46-6	CCCCCCCCCCCCCCCCCCCC(F)(F)C(F)(F)C(F)(F)C(F)(F)C(F)(F)C(F)(F)C(F)(F)C(F)(F)C(F)(F)C(F)(F)F		-9.12	0.62	3.84	P	OUT
QL01438	1,1,1,2,2,3,3,4,4,5,5,6,6,7,7,8,8-Heptadecafluoroheptacosane	871311-26-1	CCCCCCCCCCCCCCCCCCCC(F)(F)C(F)(F)C(F)(F)C(F)(F)C(F)(F)C(F)(F)C(F)(F)C(F)(F)F		-8.60	0.61	3.73	P	OUT
QL01439	1,1,1,2,2,3,3,4,4,5,5,6,6-Tridecafluoropentacosane	863126-44-7	CCCCCCCCCCCCCCCCCCCC(F)(F)C(F)(F)C(F)(F)C(F)(F)C(F)(F)C(F)(F)F		-7.98	0.59	3.62	P	OUT
QL01440	1,1,1,2,2,3,3,4,4,5,5,6,6,7,7,8,8,9,9,10,10,11,11,12,12-Pentacosafluorodotriacontane	89109-72-8	CCCCCCCCCCCCCCCCCCCCC(F)(F)C(F)(F)C(F)(F)C(F)(F)C(F)(F)C(F)(F)C(F)(F)C(F)(F)C(F)(F)C(F)(F)C(F)(F)C(F)(F)F		-9.62	0.63	4.01	P	OUT
QL01441	1,1,1,2,2,3,3,4,4,5,5,6,6,7,7,8,8,9,9,10,10-Henicosafluorotriacontane	766529-12-8	CCCCCCCCCCCCCCCCCCCCC(F)(F)C(F)(F)C(F)(F)C(F)(F)C(F)(F)C(F)(F)C(F)(F)C(F)(F)C(F)(F)C(F)(F)F		-9.18	0.62	3.91	P	OUT
QL01442	1,1,1,2,2,3,3,4,4,5,5,6,6,7,7,8,8,9,9-Nonadecafluorononacosane	117948-75-1	CCCCCCCCCCCCCCCCCCCCC(F)(F)C(F)(F)C(F)(F)C(F)(F)C(F)(F)C(F)(F)C(F)(F)C(F)(F)C(F)(F)F		-8.90	0.61	3.83	P	OUT
QL01443	1,1,1,2,2,3,3,4,4,5,5,6,6,7,7,8,8-Heptadecafluorooctacosane	137338-39-7	CCCCCCCCCCCCCCCCCCCCC(F)(F)C(F)(F)C(F)(F)C(F)(F)C(F)(F)C(F)(F)C(F)(F)C(F)(F)F		-8.68	0.61	3.80	P	OUT
QL01444	1,1,1,2,2,3,3,4,4,5,5,6,6-Tridecafluorohexacosane	825651-73-8	CCCCCCCCCCCCCCCCCCCCC(F)(F)C(F)(F)C(F)(F)C(F)(F)C(F)(F)C(F)(F)F		-8.07	0.59	3.70	P	OUT
QL01445	1,1,1,2,2,3,3,4,4-Nonafluorotetracosane	808758-67-0	CCCCCCCCCCCCCCCCCCCCC(F)(F)C(F)(F)C(F)(F)C(F)(F)F		-7.32	0.57	3.59	P	OUT
QL01446	1,1,1,2,2,3,3,4,4,5,5,6,6,7,7,8,8-Heptadecafluorotriacontane	137338-40-0	CCCCCCCCCCCCCCCCCCCCCCC(F)(F)C(F)(F)C(F)(F)C(F)(F)C(F)(F)C(F)(F)C(F)(F)C(F)(F)F		-8.73	0.60	3.90	P	OUT
QL01447	1,1,1,2,2,3,3,4,4,5,5,6,6,7,7,8,8-Heptadecafluorodotriacontane	137338-41-1	CCCCCCCCCCCCCCCCCCCCCCCCC(F)(F)C(F)(F)C(F)(F)C(F)(F)C(F)(F)C(F)(F)C(F)(F)C(F)(F)F		-8.79	0.60	3.98	P	OUT
QL01448	N-methyl-N-[(3-octadecyl-2-oxo-4-oxazolidinyl)methyl]perfluorooctanesulfonamide	348144-85-4	CCCCCCCCCCCCCCCCCCN1C(CN(C)S(=O)(=O)C(F)(F)C(F)(F)C(F)(F)C(F)(F)C(F)(F)C(F)(F)C(F)(F)C(F)(F)F)COC1=O		-9.20	0.62	3.94	P	OUT
QL01449	N-Methyl-N-[(3-octadecyl-2-oxo-5-oxazolidinyl)methyl]perfluoro-1-octanesulfonamide	138704-55-9	CCCCCCCCCCCCCCCCCCN1CC(CN(C)S(=O)(=O)C(F)(F)C(F)(F)C(F)(F)C(F)(F)C(F)(F)C(F)(F)C(F)(F)C(F)(F)F)OC1=O		-9.33	0.62	4.01	P	OUT
QL01450	4-(4-(2-((Perfluorooctylsulfonyl)propylamino)ethylaminocarbonyl)phenylmethyl)-N-octadecylbenzamide	73019-19-9	CCCCCCCCCCCCCCCCCCNC(=O)c1ccc(cc1)Cc2ccc(cc2)C(=O)NCCN(CCC)S(=O)(=O)C(C(C(C(C(C(C(C(F)(F)F)(F)F)(F)F)(F)F)(F)F)(F)F)(F)F)(F)F		-9.64	0.60	4.39	P	OUT
QL01451	Octadecyl 2,2,3,3,4,4,4-heptafluorobutanoate	400-57-7	CCCCCCCCCCCCCCCCCCOC(=O)C(F)(F)C(F)(F)C(F)(F)F		-6.96	0.56	3.51	P	OUT
QL01452	(2R)-2-(Acetyloxy)-3-(hexadecyloxy)propyl heptafluorobutanoate	119206-62-1	CCCCCCCCCCCCCCCCOC[C@H](COC(=O)C(F)(F)C(F)(F)C(F)(F)F)OC(C)=O		-7.25	0.57	3.63	P	OUT
QL01453	N-[5-(2-Chloro-1,1,2-trifluoroethoxy)-2-hydroxy-4-{2-[3-(pentadecyloxy)phenoxy]butanamido}phenyl]-2,2,3,3,4,4,4-heptafluorobutanamide	92589-42-9	CCCCCCCCCCCCCCCOc1cccc(OC(CC)C(=O)Nc2cc(O)c(NC(=O)C(F)(F)C(F)(F)C(F)(F)F)cc2OC(F)(F)C(F)Cl)c1		-8.54	0.59	4.02	P	OUT
QL01454	Tetradecyl heptafluorobutanoate	7365-36-8	CCCCCCCCCCCCCCOC(=O)C(F)(F)C(F)(F)C(F)(F)F		-6.71	0.57	3.25	P	OUT
QL01455	N-Dodecyl-2,2,3,3,4,4,5,5,6,6,7,7,8,8,9,9,9-heptadecafluorononanamide	34111-24-5	CCCCCCCCCCCCNC(=O)C(F)(F)C(F)(F)C(F)(F)C(F)(F)C(F)(F)C(F)(F)C(F)(F)C(F)(F)F		-8.61	0.63	3.50	P	OUT
QL01456	N-Dodecyl-2,2,3,3,4,4,5,5,6,6,7,7,8,8,8-pentadecafluorooctanamide	37696-86-9	CCCCCCCCCCCCNC(=O)C(F)(F)C(F)(F)C(F)(F)C(F)(F)C(F)(F)C(F)(F)C(F)(F)F		-8.25	0.62	3.41	P	OUT
QL01457	N-Dodecyl-2,2,3,3,4,4,4-heptafluorobutanamide	2284-51-7	CCCCCCCCCCCCNC(=O)C(F)(F)C(F)(F)C(F)(F)F		-6.57	0.58	3.09	P	OUT
QL01458	2,2,3,3,4,4,4-Heptafluoro-N-undecylbutanamide	120253-76-1	CCCCCCCCCCCNC(=O)C(F)(F)C(F)(F)C(F)(F)F		-6.59	0.58	3.05	P	OUT
QL01459	N-Decyl-2,2,3,3,4,4,4-heptafluorobutanamide	120219-44-5	CCCCCCCCCCNC(=O)C(F)(F)C(F)(F)C(F)(F)F		-6.44	0.59	2.93	P	OUT
QL01460	1-Heptafluorobutyryloxydecane	2994-16-3	CCCCCCCCCCOC(=O)C(F)(F)C(F)(F)C(F)(F)F		-6.44	0.59	2.93	P	OUT
QL01461	1-Methyl-3-octyl-1H-imidazolium perfluorobutanesulfonate	905972-83-0	CCCCCCCCN1C[NH+](C)C=C1.[O-]S(=O)(=O)C(F)(F)C(F)(F)C(F)(F)C(F)(F)F		-7.16	0.61	2.92	P	OUT
QL01462	2,2,3,3,4,4,5,5,6,6,7,7,8,8,9,9,9-Heptadecafluoro-N-octylnonanamide	33496-55-8	CCCCCCCCNC(=O)C(F)(F)C(F)(F)C(F)(F)C(F)(F)C(F)(F)C(F)(F)C(F)(F)C(F)(F)F		-8.52	0.64	3.26	P	OUT
QL01463	3-Hexyl-1-methyl-1H-Imidazolium perfluorobutanesulfonate	1001557-05-6	CCCCCCN1C[NH+](C)C=C1.[O-]S(=O)(=O)C(F)(F)C(F)(F)C(F)(F)C(F)(F)F		-6.61	0.62	2.53	P	OUT
QL01464	2,2,3,3,4,4,4-Heptafluoro-N-hexylbutanamide	75668-28-9	CCCCCCNC(=O)C(F)(F)C(F)(F)C(F)(F)F		-6.16	0.61	2.52	P	OUT
QL01465	Hexyl heptafluorobutanoate	336-65-2	CCCCCCOC(=O)C(F)(F)C(F)(F)C(F)(F)F		-6.16	0.61	2.52	P	OUT

QL01466	Hexyl 2,3,3,3-tetrafluoro-2-(heptafluoropropoxy)propanoate	183873-57-6	CCCCCCOC(=O)C(F)(OC(F)(F)C(F)(F)C(F)(F)F)C(F)(F)F		-7.14	0.62	2.76	P	OUT
QL01467	1-(3,3,4,4,5,5,6,6,7,7,8,8,9,9,10,10,11,11,12,12,12-Henicosafluorododecane-1-sulfonyl)-4-(hexyloxy)benzene	1161917-33-4	CCCCCCOc1ccc(cc1)S(=O)(=O)CCC(F)(F)C(F)(F)C(F)(F)C(F)(F)C(F)(F)C(F)(F)C(F)(F)C(F)(F)C(F)(F)C(F)(F)F		-9.46	0.65	3.63	P	OUT
QL01468	2,2,3,3,4,4,5,5,6,6,7,7,8,8,9,9,9-Heptadecafluoro-N-pentylnonanamide	33496-54-7	CCCCCNC(=O)C(F)(F)C(F)(F)C(F)(F)C(F)(F)C(F)(F)C(F)(F)C(F)(F)C(F)(F)F		-8.26	0.65	2.95	P	IN
QL01469	2,2,3,3,4,4,4-Heptafluoro-N-pentylbutanamide	75668-27-8	CCCCCNC(=O)C(F)(F)C(F)(F)C(F)(F)F		-6.20	0.61	2.46	P	OUT
QL01470	Pentyl 2,3,3,3-tetrafluoro-2-(heptafluoropropoxy)propanoate	183873-55-4	CCCCCOC(=O)C(F)(OC(F)(F)C(F)(F)C(F)(F)F)C(F)(F)F		-7.17	0.63	2.71	P	OUT
QL01471	Pentyl 4,4,5,5,6,6,7,7,7-nonafluoroheptanoate	94345-13-8	CCCCCOC(=O)CCC(F)(F)C(F)(F)C(F)(F)C(F)(F)F		-6.86	0.62	2.75	P	OUT
QL01472	1,1,1,2,2,3,3,4,4,5,5,6,6-Tridecafluoro-8-(pentyloxy)octane	1193009-93-6	CCCCCOCCC(F)(F)C(F)(F)C(F)(F)C(F)(F)C(F)(F)C(F)(F)F		-7.64	0.63	2.90	P	OUT
QL01473	1,1,1,2,2,3,3,4,4-Nonafluoro-6-(pentyloxy)hexane	1193009-96-9	CCCCCOCCC(F)(F)C(F)(F)C(F)(F)C(F)(F)F		-6.67	0.61	2.68	P	OUT
QL01474	N,N-Dibutyl-2,2,3,3,4,4,4-heptafluorobutanamide	120219-46-7	CCCCN(CCCC)C(=O)C(F)(F)C(F)(F)C(F)(F)F		-5.43	0.60	2.26	P	OUT
QL01475	N,N-Dibutyl-2,3,3,3-tetrafluoro-2-[1,1,2,3,3,3-hexafluoro-2-(heptafluoropropoxy)propoxy]propanamide	178167-73-2	CCCCN(CCCC)C(=O)C(F)(OC(F)(F)C(F)(OC(F)(F)C(F)(F)C(F)(F)F)C(F)(F)F)C(F)(F)F		-7.55	0.63	2.83	P	OUT
QL01476	2-(N-Butylperfluorooctanesulfonamido)ethyl acrylate	383-07-3	CCCCN(CCOC(=O)C=C)[S](=O)(=O)C(F)(F)C(F)(F)C(F)(F)C(F)(F)C(F)(F)C(F)(F)C(F)(F)C(F)(F)F		-8.31	0.64	3.08	P	OUT
QL01477	3-Butyl-1-methyl-1H-imidazolium N,N-bis(perfluorobutanesulfonyl)amide	872345-66-9	CCCCN1C[NH+](C)C=C1.FC(F)(F)C(F)(F)C(F)(F)C(F)(F)S(=O)(=O)[N-]S(=O)(=O)C(F)(F)C(F)(F)C(F)(F)C(F)(F)F		-8.57	0.65	3.09	P	IN
QL01478	N-Butyl-2,2,3,3,4,4,5,5,6,6,7,7,8,8,9,9,9-heptadecafluorononanamide	73602-42-3	CCCCNC(=O)C(F)(F)C(F)(F)C(F)(F)C(F)(F)C(F)(F)C(F)(F)C(F)(F)C(F)(F)F		-8.30	0.66	2.90	P	IN
QL01479	N-Butyl-2,2,3,3,4,4,5,5,6,6,7,7,8,8,8-pentadecafluorooctanamide	37696-83-6	CCCCNC(=O)C(F)(F)C(F)(F)C(F)(F)C(F)(F)C(F)(F)C(F)(F)C(F)(F)F		-7.88	0.65	2.75	P	IN
QL01480	N-Butyl-2,2,3,3,4,4,5,5,6,6,7,7,7-tridecafluoroheptanamide	570420-18-7	CCCCNC(=O)C(F)(F)C(F)(F)C(F)(F)C(F)(F)C(F)(F)C(F)(F)F		-7.66	0.65	2.72	P	IN
QL01481	N-Butyl-2,2,3,3,4,4,5,5,5-nonafluoropentanamide	73602-44-5	CCCCNC(=O)C(F)(F)C(F)(F)C(F)(F)C(F)(F)F		-6.76	0.63	2.48	P	OUT
QL01482	N-Butyl-2,2,3,3,4,4,4-heptafluorobutanamide	377-67-3	CCCCNC(=O)C(F)(F)C(F)(F)C(F)(F)F		-6.02	0.62	2.28	P	OUT
QL01483	N-Butyl-2,3,3,3-tetrafluoro-2-[1,1,2,3,3,3-hexafluoro-2-(heptafluoropropoxy)propoxy]propanamide	178167-72-1	CCCCNC(=O)C(F)(OC(F)(F)C(F)(OC(F)(F)C(F)(F)C(F)(F)F)C(F)(F)F)C(F)(F)F		-7.91	0.65	2.82	P	IN
QL01484	Ethyl 3-(butylamino)-4,4,5,5,6,6,6-heptafluorohexanoate	2802-65-5	CCCCNC(CC(=O)OCC)C(F)(F)C(F)(F)C(F)(F)F		-5.57	0.59	2.39	P	OUT
QL01485	N-Butylperfluorooctane-1-sulfonamide	31506-34-0	CCCCNS(=O)(=O)C(F)(F)C(F)(F)C(F)(F)C(F)(F)C(F)(F)C(F)(F)C(F)(F)C(F)(F)F		-8.46	0.66	2.89	P	IN
QL01486	Butyl heptadecafluorononanoate	117374-31-9	CCCCOC(=O)C(F)(F)C(F)(F)C(F)(F)C(F)(F)C(F)(F)C(F)(F)C(F)(F)C(F)(F)F		-8.30	0.66	2.90	P	IN
QL01487	Butyl pentadecafluorooctanoate	307-96-0	CCCCOC(=O)C(F)(F)C(F)(F)C(F)(F)C(F)(F)C(F)(F)C(F)(F)C(F)(F)F		-7.88	0.65	2.75	P	IN
QL01488	Butyl heptafluorobutanoate	1559-07-5	CCCCOC(=O)C(F)(F)C(F)(F)C(F)(F)F		-6.02	0.62	2.28	P	OUT
QL01489	Butyl 2,3,3,3-tetrafluoro-2-(heptafluoropropoxy)propanoate	147224-18-8	CCCCOC(=O)C(F)(OC(F)(F)C(F)(F)C(F)(F)F)C(F)(F)F		-6.97	0.63	2.53	P	OUT
QL01490	Butyl 2,3,3,3-tetrafluoro-2-[1,1,2,3,3,3-hexafluoro-2-(heptafluoropropoxy)propoxy]propanoate	145510-89-0	CCCCOC(=O)C(F)(OC(F)(F)C(F)(OC(F)(F)C(F)(F)C(F)(F)F)C(F)(F)F)C(F)(F)F		-7.91	0.65	2.82	P	IN
QL01492	N-(Perfluoro-1-oxobutyl)aspartic acid 1-butyl ester	88434-83-7	CCCCOC(O)[C@H](CC(O)=O)NC(=O)C(F)(F)C(F)(F)C(F)(F)F		-6.54	0.61	2.71	P	OUT
QL01493	1-(2-(2-Butoxyethoxy)ethoxy)3-(perfluorooctyl)propan-2-ol	94158-61-9	CCCCOCCOCCOCC(O)CC(F)(F)C(F)(F)C(F)(F)C(F)(F)C(F)(F)C(F)(F)C(F)(F)C(F)(F)F		-8.60	0.62	3.50	P	OUT
QL01494	Phosphinic acid, dibutyl-, 4-(2,2,3,3,4,4,4-heptafluorobutyl)phenyl ester	7526-47-8	CCCCP(=O)(CCCC)OC1=CC=C(CC(F)(F)C(F)(F)C(F)(F)F)C=C1		-6.71	0.60	2.89	P	OUT
QL01495	3-(Butane-1-sulfonyl)-4-(1,1,1,2,3,3,3-heptafluoropropan-2-yl)-2-methylbenzoic acid	1355554-53-8	CCCCS(=O)(=O)c1c(C)c(ccc1C(F)(C(F)(F)F)C(F)(F)F)C(O)=O		-6.03	0.62	2.19	P	OUT
QL01496	3-(Butane-1-sulfonyl)-2-ethyl-4-(1,1,1,2,3,3,3-heptafluoropropan-2-yl)benzoic acid	1355555-19-9	CCCCS(=O)(=O)c1c(CC)c(ccc1C(F)(C(F)(F)F)C(F)(F)F)C(O)=O		-5.79	0.62	2.19	P	OUT
QL01497	3-(Butane-1-sulfinyl)-4-(1,1,1,2,3,3,3-heptafluoropropan-2-yl)-2-methylbenzoic acid	1355554-30-1	CCCCS(=O)c1c(C)c(ccc1C(F)(C(F)(F)F)C(F)(F)F)C(O)=O		-5.75	0.62	2.18	P	OUT
QL01498	3-(Butane-1-sulfinyl)-2-ethyl-4-(1,1,1,2,3,3,3-heptafluoropropan-2-yl)benzoic acid	1355554-97-0	CCCCS(=O)c1c(CC)c(ccc1C(F)(C(F)(F)F)C(F)(F)F)C(O)=O		-5.51	0.61	2.17	P	OUT
QL01499	3-(Butylsulfanyl)-4-(1,1,1,2,3,3,3-heptafluoropropan-2-yl)-2-methylbenzoic acid	1355554-07-2	CCCCSc1c(C)c(ccc1C(F)(C(F)(F)F)C(F)(F)F)C(O)=O		-5.59	0.61	2.15	P	OUT
QL01500	3-(Butylsulfanyl)-2-ethyl-4-(1,1,1,2,3,3,3-heptafluoropropan-2-yl)benzoic acid	1355554-72-1	CCCCSc1c(CC)c(ccc1C(F)(C(F)(F)F)C(F)(F)F)C(O)=O		-5.34	0.60	2.14	P	OUT

QL01501	1-(Butylsulfanyl)-3,4,4,5,5,6,6,6-octafluorohex-2-ene	90292-35-6	CCCCSCC=C(F)C(F)(F)C(F)(F)C(F)(F)F		-6.16	0.61	2.52	P	OUT
QL01502	N-[(Heptadecafluorooctyl)sulfonyl]-N-propylglycine	66551-19-7	CCCN(CC(O)=O)S(=O)(=O)C(F)(F)C(F)(F)C(F)(F)C(F)(F)C(F)(F)C(F)(F)C(F)(F)C(F)(F)F.CCCN(CC(O)=O)S(=O)(=O)C(F)(F)C(F)(F)C(F)(F)C(F)(F)C(F)(F)C(F)(F)C(F)(F)C(F)(F)F		-8.08	0.66	2.75	P	IN
QL01503	N-(2,3-Dihydroxypropyl)-1,1,2,2,3,3,4,4,5,5,6,6,7,7,8,8,8-heptadecafluoro-N-propyloctane-1-sulfonamide	2262-49-9	CCCN(CC(O)CO)S(=O)(=O)C(F)(F)C(F)(F)C(F)(F)C(F)(F)C(F)(F)C(F)(F)C(F)(F)C(F)(F)F		-8.23	0.65	2.89	P	IN
QL01504	1,1,2,2,3,3,4,4,5,5,6,6,7,7,8,8,8-Heptadecafluoro-N-[(oxiran-2-yl)methyl]-N-propyloctane-1-sulfonamide	77620-64-5	CCCN(CC1CO1)S(=O)(=O)C(F)(F)C(F)(F)C(F)(F)C(F)(F)C(F)(F)C(F)(F)C(F)(F)C(F)(F)F		-8.17	0.66	2.75	P	IN
QL01505	Poly(oxy-1,2-ethanediyl), .alpha.-[2-[[(perfluorooctyl)sulfonyl]propylamino]ethyl]-.omega.-hydroxy-	52550-45-5	CCCN(CCOCCO)S(=O)(=O)C(F)(F)C(F)(F)C(F)(F)C(F)(F)C(F)(F)C(F)(F)C(F)(F)C(F)(F)F		-8.27	0.64	3.08	P	OUT
QL01506	2,2,3,3,4,4,4-Heptafluoro-N-propylbutanamide	75668-24-5	CCCNC(=O)C(F)(F)C(F)(F)C(F)(F)F		-6.07	0.63	2.20	P	OUT
QL01507	2,3,3,3-Tetrafluoro-2-(heptafluoropropoxy)propanoic acid--N-propylpropan-1-amine (1/1)	165951-17-7	CCCNCCC.OC(=O)C(F)(OC(F)(F)C(F)(F)C(F)(F)F)C(F)(F)F		-5.55	0.61	2.11	P	OUT
QL01508	4,5,5,5-Tetrafluoro-4-{1,1,2,3,3,3-hexafluoro-2-[1,1,2,3,3,3-hexafluoro-2-(heptafluoropropoxy)propoxy]propoxy}-N-propylpentan-1-amine	928655-42-9	CCCNCCCC(F)(OC(F)(F)C(F)(OC(F)(F)C(F)(OC(F)(F)C(F)(F)C(F)(F)F)C(F)(F)F)C(F)(F)F)C(F)(F)F		-8.75	0.65	3.21	P	OUT
QL01509	(3,3,4,4,5,5,6,6,7,7,8,8,9,9,10,10,10-Heptadecafluorodecyl)(tripropoxy)silane	521084-64-0	CCCO[Si](CCC(F)(F)C(F)(F)C(F)(F)C(F)(F)C(F)(F)C(F)(F)C(F)(F)C(F)(F)F)(OCCC)OCCC		-7.57	0.63	2.93	P	OUT
QL01510	N-(Perfluoro-1-oxobutyl)aspartic acid 1-propyl ester	88436-09-3	CCCOC(=O)[C@H](CC(O)=O)NC(=O)C(F)(F)C(F)(F)C(F)(F)F		-6.27	0.61	2.51	P	OUT
QL01511	Propyl heptadecafluorononanoate	99156-01-1	CCCOC(=O)C(F)(F)C(F)(F)C(F)(F)C(F)(F)C(F)(F)C(F)(F)C(F)(F)C(F)(F)F		-8.12	0.66	2.74	P	IN
QL01512	Heptanoic acid, tridecafluoro-, propyl ester	134638-94-1	CCCOC(=O)C(F)(F)C(F)(F)C(F)(F)C(F)(F)C(F)(F)C(F)(F)F		-7.43	0.65	2.52	P	IN
QL01513	Propyl 2,3,3,3-tetrafluoro-2-(heptafluoropropoxy)propanoate	147224-16-6	CCCOC(=O)C(F)(OC(F)(F)C(F)(F)C(F)(F)F)C(F)(F)F		-6.99	0.64	2.46	P	IN
QL01514	Propyl 2,3,3,3-tetrafluoro-2-[1,1,2,3,3,3-hexafluoro-2-(heptafluoropropoxy)propoxy]propanoate	145510-87-8	CCCOC(=O)C(F)(OC(F)(F)C(F)(OC(F)(F)C(F)(F)C(F)(F)F)C(F)(F)F)C(F)(F)F		-7.76	0.65	2.68	P	IN
QL01515	1,1,1,2,2,3,3,4,4-Nonafluoro-4-propoxybutane	72372-80-6	CCCOC(F)(F)C(F)(F)C(F)(F)C(F)(F)F		-6.48	0.64	2.21	P	IN
QL01516	1,1,1,2,2,3,3,4,4,5,5,6,6-Tridecafluoro-8-propoxyoctane	1193010-01-3	CCCOCCC(F)(F)C(F)(F)C(F)(F)C(F)(F)C(F)(F)C(F)(F)F		-7.58	0.64	2.72	P	IN
QL01517	1,1,1,2,2,3,3,4,4-Nonafluoro-6-propoxyhexane	1193009-98-1	CCCOCCC(F)(F)C(F)(F)C(F)(F)C(F)(F)F		-6.57	0.63	2.46	P	OUT
QL01518	4-(1,1,1,2,3,3,3-Heptafluoropropan-2-yl)-2-methyl-3-(propane-1-sulfonyl)benzoic acid	1355554-46-9	CCCS(=O)(=O)c1c(C)c(ccc1C(F)(C(F)(F)F)C(F)(F)F)C(O)=O		-5.92	0.63	2.07	P	OUT
QL01519	2-Ethyl-4-(1,1,1,2,3,3,3-heptafluoropropan-2-yl)-3-(propane-1-sulfonyl)benzoic acid	1355555-13-3	CCCS(=O)(=O)c1c(CC)c(ccc1C(F)(C(F)(F)F)C(F)(F)F)C(O)=O		-5.67	0.62	2.07	P	OUT
QL01520	4-(1,1,1,2,3,3,3-Heptafluoropropan-2-yl)-2-methyl-3-(propane-1-sulfinyl)benzoic acid	1355554-25-4	CCCS(=O)c1c(C)c(ccc1C(F)(C(F)(F)F)C(F)(F)F)C(O)=O		-5.67	0.62	2.07	P	OUT
QL01521	2-Ethyl-4-(1,1,1,2,3,3,3-heptafluoropropan-2-yl)-3-(propane-1-sulfinyl)benzoic acid	1355554-90-3	CCCS(=O)c1c(CC)c(ccc1C(F)(C(F)(F)F)C(F)(F)F)C(O)=O		-5.42	0.61	2.07	P	OUT
QL01522	4-(1,1,1,2,3,3,3-Heptafluoropropan-2-yl)-2-methyl-3-(propylsulfanyl)benzoic acid	1355554-03-8	CCCSc1c(C)c(ccc1C(F)(C(F)(F)F)C(F)(F)F)C(O)=O		-5.57	0.62	2.07	P	OUT
QL01523	2-Ethyl-4-(1,1,1,2,3,3,3-heptafluoropropan-2-yl)-3-(propylsulfanyl)benzoic acid	1355554-68-5	CCCSc1c(CC)c(ccc1C(F)(C(F)(F)F)C(F)(F)F)C(O)=O		-5.30	0.61	2.06	P	OUT
QL01524	Hexanoic acid, 2,2,3,3,4,4,5,6,6,6-decafluoro-5-(trifluoromethyl)-, compd. with ethanamine (11)	68015-84-9	CCN.OC(=O)C(F)(F)C(F)(F)C(F)(F)C(F)(C(F)(F)F)C(F)(F)F		-6.51	0.64	2.28	P	IN
QL01525	Perfluoro-7-(trifluoromethyl)octanoic acid ethanamine	68015-86-1	CCN.OC(=O)C(F)(F)C(F)(F)C(F)(F)C(F)(F)C(F)(F)C(F)(C(F)(F)F)C(F)(F)F		-7.38	0.65	2.55	P	IN
QL01526	Ethanaminium perfluoro-9-methyldecanoate	68015-85-0	CCN.OC(=O)C(F)(F)C(F)(F)C(F)(F)C(F)(F)C(F)(F)C(F)(F)C(F)(F)C(F)(C(F)(F)F)C(F)(F)F		-8.05	0.66	2.78	P	IN
QL01527	(Ethyl{3-[(1,1,2,2,3,3,4,4,5,5,6,6,6-tridecafluorohexane-1-sulfonyl)amino]propyl}amino)oxidanide	30295-56-8	CCN([O-])CCCNS(=O)(=O)C(F)(F)C(F)(F)C(F)(F)C(F)(F)C(F)(F)C(F)(F)F		-8.17	0.65	2.92	P	IN
QL01528	N-Ethyl-1,1,2,2,3,3,4,4,5,5,6,6,6-tridecafluoro-N-methylhexane-1-sulfonamide	1427176-17-7	CCN(C)S(=O)(=O)C(F)(F)C(F)(F)C(F)(F)C(F)(F)C(F)(F)C(F)(F)F		-7.83	0.67	2.45	P	IN
QL01529	N-Ethyl-1,1,2,2,3,3,4,4,4-nonafluoro-N-methylbutane-1-sulfonamide	1427176-16-6	CCN(C)S(=O)(=O)C(F)(F)C(F)(F)C(F)(F)C(F)(F)F		-7.06	0.66	2.17	P	IN
QL01530	N-Ethyl-N-[(heptadecafluorooctyl)sulfonyl]glycine methyl ester	87988-69-0	CCN(CC(=O)OC)S(=O)(=O)C(F)(F)C(F)(F)C(F)(F)C(F)(F)C(F)(F)C(F)(F)C(F)(F)C(F)(F)F		-8.41	0.66	2.90	P	IN

QL01531	N,N-Diethyl-2,2,3,3,4,4,5,5,6,6,7,7,8,8,9,9,9-heptadecafluorononanamide	90403-88-6	CCN(CC)C(=O)C(F)(F)C(F)(F)C(F)(F)C(F)(F)C(F)(F)C(F)(F)C(F)(F)C(F)(F)F		-8.05	0.66	2.69	P	IN
QL01532	Heptanamide, N,N-diethyl-2,2,3,3,4,4,5,5,6,6,7,7,7-tridecafluoro-	61881-15-0	CCN(CC)C(=O)C(F)(F)C(F)(F)C(F)(F)C(F)(F)C(F)(F)C(F)(F)F		-7.35	0.65	2.46	P	IN
QL01533	N,N-Diethyl-2,2,3,3,4,4,5,5,5-nonafluoropentanamide	158018-94-1	CCN(CC)C(=O)C(F)(F)C(F)(F)C(F)(F)C(F)(F)F		-6.43	0.64	2.18	P	IN
QL01534	N,N-Diethyl-2,2,3,3,4,4,4-heptafluorobutanamide	377-58-2	CCN(CC)C(=O)C(F)(F)C(F)(F)C(F)(F)F		-5.63	0.63	1.92	P	OUT
QL01535	N,N-Diethyl-2,3,3,3-tetrafluoro-2-(heptafluoropropoxy)propanamide	176702-71-9	CCN(CC)C(=O)C(F)(OC(F)(F)C(F)(F)C(F)(F)F)C(F)(F)F		-6.54	0.64	2.20	P	IN
QL01536	N,N-Diethyl-2,3,3,3-tetrafluoro-2-[1,1,2,3,3,3-hexafluoro-2-(heptafluoropropoxy)propoxy]propanamide	176702-74-2	CCN(CC)C(=O)C(F)(OC(F)(F)C(F)(OC(F)(F)C(F)(F)C(F)(F)F)C(F)(F)F)C(F)(F)F		-7.74	0.65	2.65	P	IN
QL01537	N,N-Diethyl-2H-perfluoropropanamine	309-88-6	CCN(CC)C(F)(F)C(F)C(F)(F)F		-4.93	0.61	1.84	P	OUT
QL01538	Heptadecafluorooctane-1-sulphonamide, compound with triethylamine (11)	76752-82-4	CCN(CC)CC.NS(=O)(=O)C(F)(F)C(F)(F)C(F)(F)C(F)(F)C(F)(F)C(F)(F)C(F)(F)C(F)(F)F		-8.35	0.65	2.96	P	IN
QL01539	N,N-Diethyl-1,1,2,2,3,3,4,4,5,5,6,6,6-tridecafluorohexane-1-sulfonamide	1270179-93-5	CCN(CC)S(=O)(=O)C(F)(F)C(F)(F)C(F)(F)C(F)(F)C(F)(F)C(F)(F)F		-7.55	0.66	2.45	P	IN
QL01540	N-ethyl-N-[3-(trichlorosilyl)propyl]perfluorooctanesulfonamide	67939-42-8	CCN(CCC[Si](Cl)(Cl)Cl)S(=O)(=O)C(F)(F)C(F)(F)C(F)(F)C(F)(F)C(F)(F)C(F)(F)C(F)(F)C(F)(F)F		-8.61	0.67	2.94	P	IN
QL01541	N-Ethyl-N-[3-(trimethoxysilyl)propyl]perfluorooctanesulfonamide	61660-12-6	CCN(CCC[Si](OC)(OC)OC)S(=O)(=O)C(F)(F)C(F)(F)C(F)(F)C(F)(F)C(F)(F)C(F)(F)C(F)(F)C(F)(F)F		-8.43	0.65	3.07	P	IN
QL01542	N-Ethyl-1,1,2,2,3,3,4,4,5,5,6,6,7,7,8,8,8-heptadecafluoro-N-(11-{2-[2-(2-methoxyethoxy)ethoxy]ethyl}-2,5,8-trioxa-11-azatetradecan-14-yl)octane-1-sulfonamide	117413-27-1	CCN(CCCN(CCOCCOCCOC)CCOCCOCCOC)S(=O)(=O)C(F)(F)C(F)(F)C(F)(F)C(F)(F)C(F)(F)C(F)(F)C(F)(F)C(F)(F)F		-8.16	0.60	3.59	P	OUT
QL01543	N-Ethyl--N-(2-hydroxyethyl)perfluoroheptanesulfonamide	68555-73-7	CCN(CCO)S(=O)(=O)C(F)(F)C(F)(F)C(F)(F)C(F)(F)C(F)(F)C(F)(F)C(F)(F)F		-7.92	0.66	2.69	P	IN
QL01544	1-Pentanesulfonamide, N-ethyl-1,1,2,2,3,3,4,4,5,5,5-undecafluoro-N-(2-hydroxyethyl)-	68555-72-6	CCN(CCO)S(=O)(=O)C(F)(F)C(F)(F)C(F)(F)C(F)(F)C(F)(F)F		-7.18	0.65	2.45	P	IN
QL01545	2-[Ethyl[(heptadecafluorooctyl)sulfonyl]amino]ethyl methacrylate	376-14-7	CCN(CCOC(=O)C(C)=C)[S](=O)(=O)C(F)(F)C(F)(F)C(F)(F)C(F)(F)C(F)(F)C(F)(F)C(F)(F)C(F)(F)F		-8.69	0.66	3.08	P	IN
QL01546	2-[Ethyl[(heptadecafluorooctyl)sulfonyl]amino]ethyl acrylate	423-82-5	CCN(CCOC(=O)C=C)[S](=O)(=O)C(F)(F)C(F)(F)C(F)(F)C(F)(F)C(F)(F)C(F)(F)C(F)(F)C(F)(F)F		-8.56	0.65	3.08	P	IN
QL01547	Sulfonamides, C4-8-alkane, perfluoro, N-ethyl-N-(hydroxyethyl), reaction products with 1,1'-methylenebis[4-isocyanatobenzene]	68608-14-0	CCN(CCOC(=O)Nc1ccc(Cc2ccc(NC(=O)OCCN(CC)S(=O)(=O)C(F)(F)C(F)(F)C(F)(F)C(F)(F)F)cc2)cc1)S(=O)(=O)C(F)(F)C(F)(F)C(F)(F)C(F)(F)F		-9.47	0.63	3.88	P	OUT
QL01548	Sulfonamides, C4-8-alkane, perfluoro, N-ethyl-N-(hydroxyethyl), reaction products with TDI	68608-13-9	CCN(CCOC(O)Nc1ccc(C)c(NC(=O)OCCN(CC)S(=O)(=O)C(F)(F)C(F)(F)C(F)(F)C(F)(F)F)c1)S(=O)(=O)C(F)(F)C(F)(F)C(F)(F)C(F)(F)F		-9.18	0.64	3.63	P	OUT
QL01549	[Ethyl(1,1,2,2,3,3,4,4,5,5,6,6,7,7,8,8,8-heptadecafluorooctane-1-sulfonyl)amino]methanesulfonic acid	94196-26-6	CCN(CS(O)(=O)=O)S(=O)(=O)C(F)(F)C(F)(F)C(F)(F)C(F)(F)C(F)(F)C(F)(F)C(F)(F)C(F)(F)F		-8.45	0.67	2.75	P	IN
QL01550	N-Ethyl-2,2,3,3,4,4,5,5,5-nonafluoropentanamide	158018-93-0	CCNC(=O)C(F)(F)C(F)(F)C(F)(F)C(F)(F)F		-6.59	0.64	2.19	P	IN
QL01551	N-Ethyl-2,2,3,3,4,4,4-heptafluorobutanamide	70473-76-6	CCNC(=O)C(F)(F)C(F)(F)C(F)(F)F		-5.78	0.63	1.93	P	IN
QL01552	Heptadecafluorononanoic acid--N-ethylethanamine (1/1)	77032-27-0	CCNCC.OC(=O)C(F)(F)C(F)(F)C(F)(F)C(F)(F)C(F)(F)C(F)(F)C(F)(F)C(F)(F)F		-8.06	0.65	2.92	P	IN
QL01553	N-Ethyl-2,2,3,3,4,4,4-heptafluorobutan-1-amine	355-99-7	CCNCC(F)(F)C(F)(F)C(F)(F)F		-5.63	0.63	1.95	P	OUT
QL01554	Diethoxy(2-(perfluorooctyl)-1-ethyl)methylsilane	152992-46-6	CCO[Si](C)(CCC(F)(F)C(F)(F)C(F)(F)C(F)(F)C(F)(F)C(F)(F)C(F)(F)C(F)(F)F)OCC		-8.14	0.65	2.89	P	IN
QL01555	1H,1H,2H,2H-Perfluorotetradecyltriethoxysilane	885275-56-9	CCO[Si](CCC(F)(F)C(F)(F)C(F)(F)C(F)(F)C(F)(F)C(F)(F)C(F)(F)C(F)(F)C(F)(F)C(F)(F)C(F)(F)C(F)(F)F)(OCC)OCC		-8.84	0.65	3.23	P	OUT
QL01556	1H,1H,2H,2H-Perfluorododecyltriethoxysilane	146090-84-8	CCO[Si](CCC(F)(F)C(F)(F)C(F)(F)C(F)(F)C(F)(F)C(F)(F)C(F)(F)C(F)(F)C(F)(F)C(F)(F)F)(OCC)OCC		-8.35	0.65	3.06	P	OUT
QL01557	1H,1H,2H,2H-Perfluorodecyltriethoxysilane	101947-16-4	CCO[Si](CCC(F)(F)C(F)(F)C(F)(F)C(F)(F)C(F)(F)C(F)(F)C(F)(F)C(F)(F)F)(OCC)OCC		-7.76	0.64	2.88	P	OUT
QL01558	Triethoxy[5,5,6,6,7,7,7-heptafluoro-4,4-bis(trifluoromethyl)heptyl]silane	130676-81-2	CCO[Si](CCCC(C(F)(F)F)(C(F)(F)F)C(F)(F)C(F)(F)C(F)(F)F)(OCC)OCC		-6.09	0.62	2.31	P	OUT
QL01559	N-(3-Triethoxysilylpropyl)perfluorooctanoamide	37043-12-2	CCO[Si](CCCNC(=O)C(F)(F)C(F)(F)C(F)(F)C(F)(F)C(F)(F)C(F)(F)C(F)(F)F)(OCC)OCC		-7.73	0.62	3.06	P	OUT
QL01560	N-[3-(trimethoxysilyl)propyl]perfluorohexanamide	154380-34-4	CCO[Si](CCCNC(=O)C(F)(F)C(F)(F)C(F)(F)C(F)(F)C(F)(F)F)(OC)OC		-7.32	0.62	2.85	P	OUT
QL01561	1,1,2,2,3,3,4,4,5,5,6,6,7,7,8,8,8-Heptadecafluoro-N-[3-(triethoxysilyl)propyl]octane-1-sulfonamide	106790-26-5	CCO[Si](CCCNS(=O)(=O)C(F)(F)C(F)(F)C(F)(F)C(F)(F)C(F)(F)C(F)(F)C(F)(F)C(F)(F)F)(OCC)OCC		-8.13	0.64	3.11	P	OUT

QL01562	3-(Heptafluoroisopropoxy)propyltriethoxysilane	149838-19-7	CCO[Si](CCCOC(F)(C(F)(F)F)C(F)(F)F)(OCC)OCC		-5.10	0.59	2.25	P	OUT
QL01563	Triethoxy(henicosafluorodecyl)silane	254095-33-5	CCO[Si](OCC)(OCC)C(F)(F)C(F)(F)C(F)(F)C(F)(F)C(F)(F)C(F)(F)C(F)(F)C(F)(F)C(F)(F)C(F)(F)F		-8.12	0.65	2.89	P	IN
QL01564	Silane, triethoxy(heptadecafluorooctyl)-	96305-13-4	CCO[Si](OCC)(OCC)C(F)(F)C(F)(F)C(F)(F)C(F)(F)C(F)(F)C(F)(F)C(F)(F)C(F)(F)F		-7.48	0.64	2.69	P	IN
QL01565	Triethoxy(1,1,2,2,3,3,6,6,6-nonafluorohexyl)silane	102390-98-7	CCO[Si](OCC)(OCC)C(F)(F)C(F)(F)C(F)(F)CCC(F)(F)F		-5.94	0.60	2.44	P	OUT
QL01566	N-(Perfluorobutanoyl)glutamic acid 1-ethyl ester	88435-87-4	CCOC(=O)[C@@H](CCC(O)=O)NC(=O)C(F)(F)C(F)(F)C(F)(F)F		-6.08	0.61	2.39	P	OUT
QL01567	Ethyl 2-diazo-4,4,5,5,6,6,6-heptafluoro-3-oxohexanoate	15959-98-5	CCOC(=O)C(=[N+]=[N-])C(=O)C(F)(F)C(F)(F)C(F)(F)F		-5.94	0.62	2.25	P	OUT
QL01568	Ethyl 3,3,4,4,5,5,6,6,6-nonafluoro-2-methylidenehexanoate	117374-41-1	CCOC(=O)C(=C)C(F)(F)C(F)(F)C(F)(F)C(F)(F)F		-6.63	0.64	2.26	P	IN
QL01569	Ethyl 2-(1,1,2,2,3,3,4,4,4-nonafluorobutane-1-sulfinyl)prop-2-enoate	1606997-40-3	CCOC(=O)C(=C)S(=O)C(F)(F)C(F)(F)C(F)(F)C(F)(F)F		-6.96	0.64	2.46	P	IN
QL01570	Ethyl 3-[4-(dimethylamino)phenyl]-2-(1,1,2,2,3,3,4,4,4-nonafluorobutane-1-sulfonyl)prop-2-enoate	75988-12-4	CCOC(=O)C(=Cc1ccc(cc1)N(C)C)S(=O)(=O)C(F)(F)C(F)(F)C(F)(F)C(F)(F)F		-7.21	0.63	2.71	P	OUT
QL01571	Ethyl 3,4,4,4-tetrafluoro-2-oxo-3-(trifluoromethyl)butanoate	74405-85-9	CCOC(=O)C(=O)C(F)(C(F)(F)F)C(F)(F)F		-5.48	0.63	1.87	P	OUT
QL01572	Ethyl 3,3,4,4,5,5,6,6,7,7,8,8,8-tridecafluoro-2-oxooctanoate	117516-21-9	CCOC(=O)C(=O)C(F)(F)C(F)(F)C(F)(F)C(F)(F)C(F)(F)C(F)(F)F		-7.50	0.65	2.52	P	IN
QL01573	Ethyl 2-acetyl-4,4,5,5,6,6,6-heptafluoro-3-oxohexanoate	115479-06-6	CCOC(=O)C(C(C)=O)C(=O)C(F)(F)C(F)(F)C(F)(F)F		-6.10	0.63	2.22	P	OUT
QL01574	Ethyl 2-acetyl-3,3,4,4,5,5,5-heptafluoropentanoate	189237-57-8	CCOC(=O)C(C(C)=O)C(F)(F)C(F)(F)C(F)(F)F		-5.89	0.62	2.12	P	OUT
QL01575	N-(Perfluoro-1-oxobutyl)aspartic acid 1-ethyl ester	88435-83-0	CCOC(=O)C(CC(O)=O)NC(=O)C(F)(F)C(F)(F)C(F)(F)F		-6.18	0.62	2.39	P	OUT
QL01576	Ethyl 2-[(diphenylmethylidene)amino]-5,5,6,6,7,7,8,8,8-nonafluorooctanoate	125162-61-0	CCOC(=O)C(CCC(F)(F)C(F)(F)C(F)(F)C(F)(F)F)N=C(c1ccccc1)c1ccccc1		-6.44	0.60	2.69	P	OUT
QL01577	Ethyl 2,3,3,3-tetrafluoro-2-(trifluoromethyl)propanoate	1526-49-4	CCOC(=O)C(F)(C(F)(F)F)C(F)(F)F		-5.30	0.63	1.75	P	OUT
QL01578	Diethyl perfluoroglutarate	424-40-8	CCOC(=O)C(F)(F)C(F)(F)C(F)(F)C(=O)OCC		-5.94	0.61	2.38	P	OUT
QL01579	Ethyl perfluorododecanoate	158607-41-1	CCOC(=O)C(F)(F)C(F)(F)C(F)(F)C(F)(F)C(F)(F)C(F)(F)C(F)(F)C(F)(F)C(F)(F)C(F)(F)C(F)(F)F		-8.86	0.67	2.94	P	OUT
QL01580	Ethyl perfluorononan-1-oate	30377-52-7	CCOC(=O)C(F)(F)C(F)(F)C(F)(F)C(F)(F)C(F)(F)C(F)(F)C(F)(F)C(F)(F)F		-8.16	0.67	2.69	P	IN
QL01581	Ethyl perfluorooctanoate	3108-24-5	CCOC(=O)C(F)(F)C(F)(F)C(F)(F)C(F)(F)C(F)(F)C(F)(F)C(F)(F)F		-7.71	0.66	2.51	P	IN
QL01582	Ethyl perfluoroheptanoate	41430-70-0	CCOC(=O)C(F)(F)C(F)(F)C(F)(F)C(F)(F)C(F)(F)C(F)(F)F		-7.48	0.66	2.45	P	IN
QL01583	Ethyl nonafluoropentanoate	424-36-2	CCOC(=O)C(F)(F)C(F)(F)C(F)(F)C(F)(F)F		-6.59	0.64	2.19	P	IN
QL01584	Ethyl heptafluorobutyrate	356-27-4	CCOC(=O)C(F)(F)C(F)(F)C(F)(F)F		-5.78	0.63	1.93	P	IN
QL01585	Ethyl perfluoropropionate	426-65-3	CCOC(=O)C(F)(F)C(F)(F)F		-5.17	0.62	1.86	P	OUT
QL01586	Ethyl 2,3,3,3-tetrafluoro-2-(heptafluoropropoxy)propanoate	79851-29-9	CCOC(=O)C(F)(OC(F)(F)C(F)(F)C(F)(F)F)C(F)(F)F		-6.63	0.65	2.19	P	IN
QL01587	Ethyl 2,3,3,3-tetrafluoro-2-[1,1,2,3,3,3-hexafluoro-2-(heptafluoropropoxy)propoxy]propanoate	76145-88-5	CCOC(=O)C(F)(OC(F)(F)C(F)(OC(F)(F)C(F)(F)C(F)(F)F)C(F)(F)F)C(F)(F)F		-7.81	0.66	2.64	P	IN
QL01588	Ethyl 2,4,4,5,7,7,8,10,10,11,13,13,14,16,16,17,19,19,20,20,21,21,21-tricosafluoro-2,5,8,11,14,17-hexakis(trifluoromethyl)-3,6,9,12,15,18-hexaoxahenicosan-1-oate	121368-60-3	CCOC(=O)C(F)(OC(F)(F)C(F)(OC(F)(F)C(F)(OC(F)(F)C(F)(OC(F)(F)C(F)(OC(F)(F)C(F)(OC(F)(F)C(F)(F)C(F)(F)F)C(F)(F)F)C(F)(F)F)C(F)(F)F)C(F)(F)F)C(F)(F)F)C(F)(F)F		-9.98	0.67	3.56	P	OUT
QL01589	Ethyl 4,4,5,5,6,6,7,7,8,8,9,9,9-tridecafluoro-2-iodononanoate	165281-74-3	CCOC(=O)C(I)CC(F)(F)C(F)(F)C(F)(F)C(F)(F)C(F)(F)C(F)(F)F		-7.75	0.65	2.71	P	IN
QL01590	Ethyl 4,4,5,5,6,6,6-heptafluoronorleucinate	63664-52-8	CCOC(=O)C(N)CC(F)(F)C(F)(F)C(F)(F)F		-6.19	0.63	2.26	P	OUT
QL01591	Ethyl 3,3,4,4,5,5,6,6,7,7,8,8,9,9,10,10,10-heptadecafluoro-2,2-dihydroxydecanoate	1401315-53-4	CCOC(=O)C(O)(O)C(F)(F)C(F)(F)C(F)(F)C(F)(F)C(F)(F)C(F)(F)C(F)(F)C(F)(F)F		-8.34	0.67	2.74	P	IN
QL01592	Ethyl 3-amino-4,4,5,5,6,6,6-heptafluorohex-2-enoate	87613-26-1	CCOC(=O)C=C(N)C(F)(F)C(F)(F)C(F)(F)F		-6.16	0.62	2.26	P	OUT
QL01593	Ethyl (2E)-4,4,5,5,6,6,6-heptafluorohex-2-enoate	37759-79-8	CCOC(=O)C=CC(F)(F)C(F)(F)C(F)(F)F		-5.98	0.62	2.23	P	OUT
QL01594	Ethyl (2E)-3-(1,1,2,2,3,3,4,4,4-nonafluorobutane-1-sulfinyl)prop-2-enoate	1606997-39-0	CCOC(=O)C=CS(=O)C(F)(F)C(F)(F)C(F)(F)C(F)(F)F		-6.86	0.63	2.53	P	OUT
QL01595	Ethyl 6-(nonafluorobutyl)-4-phenyl-2-sulfanylidene-1,2,3,4-tetrahydropyrimidine-5-carboxylate	281189-83-1	CCOC(=O)C1=C(NC(=S)NC1c1ccccc1)C(F)(F)C(F)(F)C(F)(F)C(F)(F)F		-6.42	0.62	2.48	P	OUT
QL01596	Diethyl 2,6-dihydroxy-2,6-bis(nonafluorobutyl)-4-phenyloxane-3,5-dicarboxylate	359875-73-3	CCOC(=O)C1C(C(C(=O)OCC)C(O)(OC1(O)C(F)(F)C(F)(F)C(F)(F)C(F)(F)F)C(F)(F)C(F)(F)C(F)(F)C(F)(F)F)c1ccccc1		-7.06	0.64	2.52	P	IN

QL01597	Ethyl 2-(pentadecafluoroheptyl)imidazo[1,2-a]pyridine-3-carboxylate	135339-05-8	CCOC(=O)c1c(nc2ccccn12)C(F)(F)C(F)(F)C(F)(F)C(F)(F)C(F)(F)C(F)(F)C(F)(F)F		-7.66	0.65	2.73	P	IN
QL01598	Ethyl 3-methyl-5-(nonafluorobutyl)-1H-pyrazole-4-carboxylate	119403-58-6	CCOC(=O)c1c(Nnc1C)C(F)(F)C(F)(F)C(F)(F)C(F)(F)F		-6.77	0.63	2.43	P	OUT
QL01599	Ethyl 6-(heptafluoropropyl)pyridine-3-carboxylate	1574401-92-5	CCOC(=O)c1ccc(nc1)C(F)(F)C(F)(F)C(F)(F)F		-6.53	0.62	2.51	P	OUT
QL01600	Ethyl 4-[(nonafluorobutyl)sulfanyl]benzoate	1262679-06-0	CCOC(=O)c1ccc(SC(F)(F)C(F)(F)C(F)(F)C(F)(F)F)cc1		-7.20	0.63	2.76	P	OUT
QL01601	Ethyl 2-(heptafluoropropyl)benzoate	1310492-25-1	CCOC(=O)c1ccccc1C(F)(F)C(F)(F)C(F)(F)F		-5.83	0.62	2.22	P	OUT
QL01602	Ethyl 5-(2,2,3,3,4,4,4-heptafluorobutanamido)-1-phenyl-1H-pyrazole-4-carboxylate	545359-89-5	CCOC(=O)c1cnn(c1NC(=O)C(F)(F)C(F)(F)C(F)(F)F)-c1ccccc1		-6.18	0.61	2.48	P	OUT
QL01603	Ethyl 4,4,5,5,6,6,7,7,7-nonafluoro-3-oxoheptanoate	82941-37-5	CCOC(=O)CC(=O)C(F)(F)C(F)(F)C(F)(F)C(F)(F)F		-6.87	0.64	2.47	P	OUT
QL01604	Ethyl heptafluorobutyrylacetate	336-62-9	CCOC(=O)CC(=O)C(F)(F)C(F)(F)C(F)(F)F		-6.16	0.62	2.26	P	OUT
QL01605	Ethyl pentafluoropropionyl acetate	663-35-4	CCOC(=O)CC(=O)C(F)(F)C(F)(F)F		-5.57	0.61	2.17	P	OUT
QL01606	Ethyl 7,7,8,8,9,9,10,10,10-nonafluoro-3-oxodecanoate	1444474-83-2	CCOC(=O)CC(=O)CCCC(F)(F)C(F)(F)C(F)(F)C(F)(F)F		-6.97	0.62	2.74	P	OUT
QL01607	Ethyl 4,4,5,5,6,6,6-heptafluoro-3,3-bis(trifluoromethyl)hexanoate	161261-13-8	CCOC(=O)CC(C(F)(F)F)(C(F)(F)F)C(F)(F)C(F)(F)C(F)(F)F		-6.42	0.65	2.08	P	IN
QL01608	Ethyl 4,4,5,5,6,6,6-heptafluoro-3-(nitromethyl)hexanoate	376-88-5	CCOC(=O)CC(C[N+]([O-])=O)C(F)(F)C(F)(F)C(F)(F)F		-5.76	0.61	2.21	P	OUT
QL01609	Octanoic acid, 3,3,4,4,5,5,6,6,7,7,8,8,8-tridecafluoro-, ethyl ester	153296-01-6	CCOC(=O)CC(F)(F)C(F)(F)C(F)(F)C(F)(F)C(F)(F)C(F)(F)F		-7.42	0.65	2.52	P	IN
QL01610	Ethyl 3,3,4,4,5,5,6,6,6-nonafluorohexanoate	172287-15-9	CCOC(=O)CC(F)(F)C(F)(F)C(F)(F)C(F)(F)F		-6.51	0.64	2.27	P	IN
QL01611	Ethyl 3,3,4,4,5,5,5-heptafluoropentanoate	773136-96-2	CCOC(=O)CC(F)(F)C(F)(F)C(F)(F)F		-5.95	0.62	2.15	P	OUT
QL01612	Ethyl 4,4,5,5,6,6,6-heptafluoro-3-hydroxyhexanoate	2707-76-8	CCOC(=O)CC(O)C(F)(F)C(F)(F)C(F)(F)F		-6.16	0.62	2.26	P	OUT
QL01613	Ethyl 4,4,5,5,6,6,6-heptafluoro-3-sulfanylhexanoate	377-64-0	CCOC(=O)CC(S)C(F)(F)C(F)(F)C(F)(F)F		-6.16	0.62	2.26	P	OUT
QL01614	Ethyl 3-(2-ethoxy-2-oxoethyl)-5-(2,2,3,3,4,4,5,5,6,6,7,7,8,8,9,9,9-heptadecafluorononyl)-2-oxooxolane-3-carboxylate	674786-75-5	CCOC(=O)CC1(CC(CC(F)(F)C(F)(F)C(F)(F)C(F)(F)C(F)(F)C(F)(F)C(F)(F)C(F)(F)F)OC1=O)C(=O)OCC		-8.31	0.64	3.12	P	OUT
QL01615	Ethyl [5-(heptafluoropropyl)-1-methyl-1H-pyrazol-3-yl]acetate	1428569-19-0	CCOC(=O)Cc1cc(n(C)n1)C(F)(F)C(F)(F)C(F)(F)F		-6.65	0.62	2.56	P	OUT
QL01616	Ethyl [3-(heptafluoropropyl)-1-methyl-1H-pyrazol-5-yl]acetate	1428569-18-9	CCOC(=O)Cc1cc(nn1C)C(F)(F)C(F)(F)C(F)(F)F		-6.65	0.62	2.53	P	OUT
QL01617	Ethyl [5-(heptafluoropropyl)-1H-pyrazol-3-yl]acetate	1428569-15-6	CCOC(=O)Cc1nNc(c1)C(F)(F)C(F)(F)C(F)(F)F		-6.53	0.62	2.53	P	OUT
QL01618	Ethyl 3-(perfluorohexyl)propanoate	60023-26-9	CCOC(=O)CCC(F)(F)C(F)(F)C(F)(F)C(F)(F)C(F)(F)C(F)(F)F		-7.70	0.65	2.72	P	IN
QL01619	Ethyl 4,4,5,5,6,6,7,7,7-nonafluoroheptanoate	136905-37-8	CCOC(=O)CCC(F)(F)C(F)(F)C(F)(F)C(F)(F)F		-6.73	0.63	2.44	P	OUT
QL01620	Ethyl 4,4,5,5,6,6,6-heptafluorohexanoate	424-66-8	CCOC(=O)CCC(F)(F)C(F)(F)C(F)(F)F		-5.98	0.62	2.23	P	OUT
QL01621	Ethyl 4,4-difluoro-5-[(1,1,2,2,3,3,4,4,4-nonafluorobutane-1-sulfonyl)oxy]pentanoate	1361253-41-9	CCOC(=O)CCC(F)(F)COS(=O)(=O)C(F)(F)C(F)(F)C(F)(F)C(F)(F)F		-7.82	0.64	2.89	P	OUT
QL01622	Ethyl N-(2,2,3,3,4,4,4-heptafluorobutanoyl)glycinate	88435-34-1	CCOC(=O)CNC(=O)C(F)(F)C(F)(F)C(F)(F)F		-6.38	0.62	2.44	P	OUT
QL01623	Ethyl 3-[(2-ethoxy-2-oxoethyl)amino]-4,4,5,5,6,6,7,7,7-nonafluoroheptanoate	132114-64-8	CCOC(=O)CNC(CC(=O)OCC)C(F)(F)C(F)(F)C(F)(F)C(F)(F)F		-6.16	0.61	2.49	P	OUT
QL01624	Ethyl [(2,2,3,3,4,4,4-heptafluorobutanimidoyl)oxy]acetate	4314-33-4	CCOC(=O)COC(=N)C(F)(F)C(F)(F)C(F)(F)F		-6.38	0.62	2.44	P	OUT
QL01625	Ethyl (1,1,2,2,3,3,4,4,4-nonafluorobutane-1-sulfonyl)acetate	161123-65-5	CCOC(=O)CS(=O)(=O)C(F)(F)C(F)(F)C(F)(F)C(F)(F)F		-7.15	0.65	2.48	P	IN
QL01626	3-Ethoxyperfluoro(2-methylhexane)	297730-93-9	CCOC(C(C(F)(F)F)(C(F)(F)F)F)(C(C(C(F)(F)F)(F)F)(F)F)F		-6.37	0.66	1.83	P	IN
QL01627	10,10-Diethoxy-1,1,1,2,2,3,3,4,4,5,5,6,6,7,7,8,8-heptadecafluorodecane	133377-48-7	CCOC(CC(F)(F)C(F)(F)C(F)(F)C(F)(F)C(F)(F)C(F)(F)C(F)(F)C(F)(F)F)OCC		-7.74	0.65	2.75	P	IN
QL01628	2-(1-Ethoxy-1,2,2,3,3,4,4,4-octafluorobutyl)aniline	217474-47-0	CCOC(F)(c1ccccc1N)C(F)(F)C(F)(F)C(F)(F)F		-5.94	0.63	2.13	P	OUT
QL01629	2-Trfluoromethylpropyl ethyl ether	163702-06-5	CCOC(F)(F)C(F)(C(F)(F)F)C(F)(F)F		-5.74	0.64	1.77	P	IN
QL01630	1-Ethoxy-1,1,2,2,3,3,4,4,5,5,5-undecafluoropentane	181214-75-5	CCOC(F)(F)C(F)(F)C(F)(F)C(F)(F)C(F)(F)F		-6.87	0.66	2.17	P	IN
QL01631	1-Ethoxy-1,1,2,3,3,3-hexafluoro-2-(trifluoromethyl)propane--1-ethoxy-1,1,2,2,3,3,4,4,4-nonafluorobutane (1/1)	813458-04-7	CCOC(F)(F)C(F)(F)C(F)(F)C(F)(F)F.CCOC(F)(F)C(F)(C(F)(F)F)C(F)(F)F		-6.33	0.65	2.04	P	IN
QL01632	Diethoxymethyl(1H,1H,2H,2H-perfluorohexyl)silane	83038-83-9	CCOC(OCC)[SiH2]CCC(F)(F)C(F)(F)C(F)(F)C(F)(F)F		-6.19	0.61	2.49	P	OUT

QL01634	1-Ethoxy-4,4,5,5,6,6,6-heptafluorohex-1-en-3-one	184783-37-7	CCOC=CC(=O)C(F)(F)C(F)(F)C(F)(F)F		-6.02	0.62	2.28	P	OUT
QL01635	1-Ethoxy-3,3,4,4,5,5,6,6,6-nonafluorohexan-2-ol	89807-88-5	CCOCC(O)C(F)(F)C(F)(F)C(F)(F)C(F)(F)F		-6.53	0.64	2.27	P	IN
QL01636	1-Ethoxy-2-(1,1,2,2-tetrafluoroethoxy)ethane	85567-23-3	CCOCCOC(F)(F)C(F)F		-4.34	0.56	2.19	P	OUT
QL01637	Diethyl (3,3,4,4,5,5,6,6,7,7,8,8,9,9,10,10,11,11,12,12,12-henicosafluorododecyl)phosphonate	854095-13-9	CCOP(=O)(CCC(F)(F)C(F)(F)C(F)(F)C(F)(F)C(F)(F)C(F)(F)C(F)(F)C(F)(F)C(F)(F)C(F)(F)F)OCC		-8.68	0.66	3.07	P	IN
QL01638	diethyl 3,3,4,4,5,5,6,6,7,7,8,8,9,9,10,10,10-heptadecafluorodecylphosphonate	90146-96-6	CCOP(=O)(CCC(F)(F)C(F)(F)C(F)(F)C(F)(F)C(F)(F)C(F)(F)C(F)(F)C(F)(F)F)OCC		-8.14	0.65	2.89	P	IN
QL01639	(3,3,4,4,5,5,6,6,7,7,8,8,8-Tridecafluorooctyl)phosphonic acid diethyl ester	350608-55-8	CCOP(=O)(CCC(F)(F)C(F)(F)C(F)(F)C(F)(F)C(F)(F)C(F)(F)F)OCC		-7.47	0.64	2.69	P	IN
QL01640	Diethyl (3,3,4,4,5,5,6,6,6-nonafluorohexyl)phosphonate	627909-24-4	CCOP(=O)(CCC(F)(F)C(F)(F)C(F)(F)C(F)(F)F)OCC		-6.48	0.63	2.40	P	OUT
QL01641	Phosphonous acid, ethyl-, diethyl ester, compd. with 1,1,1,2,2,3,3-heptafluoro-3-iodopropane (11)	72220-37-2	CCOP(CC)OCC.FC(F)(F)C(F)(F)C(F)(F)I		-4.69	0.59	1.98	P	OUT
QL01642	Phosphonous acid, ethyl-, diethyl ester, compd. with 1,1,1,2,3,3,3-heptafluoro-2-iodopropane (11)	72220-43-0	CCOP(CC)OCC.FC(F)(F)C(F)(I)C(F)(F)F		-4.69	0.59	1.98	P	OUT
QL01643	Ethyl hydrogen (3,3,4,4,5,5,6,6,7,7,8,8,8-tridecafluorooctyl)phosphonate	1189052-97-8	CCOP(O)(=O)CCC(F)(F)C(F)(F)C(F)(F)C(F)(F)C(F)(F)C(F)(F)F		-7.92	0.66	2.70	P	IN
QL01644	Ethyl 5,5,6,6,7,7,8,8,8-nonafluorooctane-1-sulfonate	1604816-68-3	CCOS(=O)(=O)CCCCC(F)(F)C(F)(F)C(F)(F)C(F)(F)F		-7.11	0.63	2.63	P	OUT
QL01645	Silver, (6,6,7,7,8,8,8-heptafluoro-2,2-dimethyl-3,5-octanedionato-βO3,βO5)(triethylphosphine)-	165461-74-5	CCP(CC)(CC)[Ag]1OC(CC(O1)C(F)(F)C(F)(F)C(F)(F)F)C(C)(C)C		-6.30	0.64	2.18	P	IN
QL01646	3-(Ethanesulfonyl)-4-(1,1,1,2,3,3,3-heptafluoropropan-2-yl)-2-methylbenzoic acid	1355554-44-7	CCS(=O)(=O)c1c(C)c(ccc1C(F)(C(F)(F)F)C(F)(F)F)C(O)=O		-6.26	0.64	2.11	P	IN
QL01647	3-(Ethanesulfinyl)-4-(1,1,1,2,3,3,3-heptafluoropropan-2-yl)-2-methylbenzoic acid	1355554-21-0	CCS(=O)c1c(C)c(ccc1C(F)(C(F)(F)F)C(F)(F)F)C(O)=O		-5.99	0.63	2.12	P	OUT
QL01648	S-Ethyl 3,3,4,4,5,5,5-heptafluoro-2-(2-oxopropyl)pentanethioate	1392881-17-2	CCSC(=O)C(CC(C)=O)C(F)(F)C(F)(F)C(F)(F)F		-5.72	0.62	2.13	P	OUT
QL01649	4,4-Bis(ethylsulfanyl)-1,1,1,2,2,3,3-heptafluorobutane	145327-73-7	CCSC(SCC)C(F)(F)C(F)(F)C(F)(F)F		-5.03	0.61	1.92	P	OUT
QL01650	3-(Ethylsulfanyl)-4-(1,1,1,2,3,3,3-heptafluoropropan-2-yl)-2-methylbenzoic acid	1355553-99-9	CCSc1c(C)c(ccc1C(F)(C(F)(F)F)C(F)(F)F)C(O)=O		-5.81	0.62	2.12	P	OUT
QL01651	2-Ethyl-3-(ethylsulfanyl)-4-(1,1,1,2,3,3,3-heptafluoropropan-2-yl)benzoic acid	1355554-66-3	CCSc1c(CC)c(ccc1C(F)(C(F)(F)F)C(F)(F)F)C(O)=O		-5.54	0.61	2.12	P	OUT
QL01652	2-Ethyl-3,3,4,4,5,5,5-heptafluoropentan-1-amine--hydrogen chloride (1/1)	308-38-3	Cl.CCC(CN)C(F)(F)C(F)(F)C(F)(F)F		-4.83	0.59	2.05	P	OUT
QL01653	5-Amino-1,1,1,2,2,3,3-heptafluoroheptan-4-ol--hydrogen chloride (1/1)	308-37-2	Cl.CCC(N)C(O)C(F)(F)C(F)(F)C(F)(F)F		-5.39	0.61	2.13	P	OUT
QL01654	5,5,6,6,7,7,7-Heptafluoroheptan-3-amine--hydrogen chloride (1/1)	2263-70-9	Cl.CCC(N)CC(F)(F)C(F)(F)C(F)(F)F		-5.20	0.60	2.15	P	OUT
QL01655	N-[3-(dimethylamino)propyl]heptadecafluorooctanesulphonamide monohydrochloride	67939-88-2	Cl.CN(C)CCCNS(=O)(=O)C(F)(F)C(F)(F)C(F)(F)C(F)(F)C(F)(F)C(F)(F)C(F)(F)C(F)(F)F		-8.26	0.65	3.04	P	OUT
QL01656	N-[3-(dimethylamino)propyl]-1,1,2,2,3,3,4,4,5,5,6,6,7,7,7-pentadecafluoroheptane-1-sulphonamide monohydrochloride	67940-02-7	Cl.CN(C)CCCNS(=O)(=O)C(F)(F)C(F)(F)C(F)(F)C(F)(F)C(F)(F)C(F)(F)C(F)(F)F		-8.06	0.64	3.01	P	OUT
QL01657	N-[3-(dimethylamino)propyl]tridecafluorohexanesulphonamide monohydrochloride	68957-61-9	Cl.CN(C)CCCNS(=O)(=O)C(F)(F)C(F)(F)C(F)(F)C(F)(F)C(F)(F)C(F)(F)F		-7.62	0.63	2.87	P	OUT
QL01658	N-[3-(dimethylamino)propyl]-1,1,2,2,3,3,4,4,5,5,5-undecafluoropentane-1-sulphonamide monohydrochloride	68957-60-8	Cl.CN(C)CCCNS(=O)(=O)C(F)(F)C(F)(F)C(F)(F)C(F)(F)C(F)(F)F		-7.37	0.63	2.84	P	OUT
QL01659	N-[3-(dimethylamino)propyl]-1,1,2,2,3,3,4,4,4-nonafluorobutane-1-sulphonamide monohydrochloride	68957-59-5	Cl.CN(C)CCCNS(=O)(=O)C(F)(F)C(F)(F)C(F)(F)C(F)(F)F		-6.81	0.62	2.68	P	OUT
QL01660	N-[4-(Nonafluorobutoxy)phenyl]pyridine-3-carboxamide--hydrogen chloride (1/1)	62315-22-4	Cl.FC(F)(F)C(F)(F)C(F)(F)C(F)(F)Oc1ccc(NC(=O)c2cccnc2)cc1		-7.05	0.60	3.01	P	OUT
QL01661	N-[4-(Nonafluorobutoxy)phenyl]pyridine-4-carboxamide--hydrogen chloride (1/1)	62315-29-1	Cl.FC(F)(F)C(F)(F)C(F)(F)C(F)(F)Oc1ccc(NC(=O)c2ccncc2)cc1		-7.05	0.60	3.01	P	OUT
QL01662	3,3,4,4-Tetrafluoropyrrolidine Hydrochloride	1810-13-5	Cl.FC1(F)CNCC1(F)F		-2.87	0.58	1.16	P	OUT
QL01663	2,2,3,3,4,4,4-Heptafluorobutanimidamide--hydrogen chloride (1/1)	2358-53-4	Cl.NC(=N)C(F)(F)C(F)(F)C(F)(F)F		-4.97	0.62	1.73	P	OUT

QL01664	2,2,3,3,4,4,5,5,6,6,7,7,8,8,8-Pentadecafluorooctan-1-amine--hydrogen chloride (1/1)	5678-75-1	Cl.NCC(F)(F)C(F)(F)C(F)(F)C(F)(F)C(F)(F)C(F)(F)C(F)(F)F		-7.14	0.65	2.38	P	IN
QL01665	2,2,3,3,4,4,4-Heptafluorobutan-1-amine--hydrogen chloride (1/1)	2794-75-4	Cl.NCC(F)(F)C(F)(F)C(F)(F)F		-4.63	0.61	1.74	P	OUT
QL01666	O-(4,4,5,5,6,6,7,7,8,8,9,9,10,10,11,11,11-Heptadecafluoroundecyl)hydroxylamine--hydrogen chloride (1/1)	1228693-32-0	Cl.NOCCCC(F)(F)C(F)(F)C(F)(F)C(F)(F)C(F)(F)C(F)(F)C(F)(F)C(F)(F)F		-7.70	0.64	2.86	P	OUT
QL01667	2,2,3,3,4,4,5,5,6,6,7,7,7-Tridecafluoro-1-[4-(2-hydroxyethyl)piperazin-1-yl]heptan-1-one--hydrogen chloride (1/1)	95851-62-0	Cl.OCCN1CCN(CC1)C(=O)C(F)(F)C(F)(F)C(F)(F)C(F)(F)C(F)(F)C(F)(F)F		-7.62	0.62	3.02	P	OUT
QL01668	Bis[tris(3-(perfluorooctyl)phenyl)phosphine]palladium(II) dichloride	326475-45-0	Cl[Pd]Cl.FC(F)(F)C(F)(F)C(F)(F)C(F)(F)C(F)(F)C(F)(F)C(F)(F)C(F)(F)C1=CC(=CC=C1)P(C1=CC=CC(=C1)C(F)(F)C(F)(F)C(F)(F)C(F)(F)C(F)(F)C(F)(F)C(F)(F)C(F)(F)F)C1=CC=CC(=C1)C(F)(F)C(F)(F)C(F)(F)C(F)(F)C(F)(F)C(F)(F)C(F)(F)C(F)(F)F.FC(F)(F)C(F)(F)C(F)(F)C(F)(F)C(F)(F)C(F)(F)C(F)(F)C(F)(F)C1=CC(=CC=C1)P(C1=CC=CC(=C1)C(F)(F)C(F)(F)C(F)(F)C(F)(F)C(F)(F)C(F)(F)C(F)(F)C(F)(F)F)C1=CC=CC(=C1)C(F)(F)C(F)(F)C(F)(F)C(F)(F)C(F)(F)C(F)(F)C(F)(F)C(F)(F)F		-10.11	0.67	3.66	P	OUT
QL01669	Dichloropalladium--tris[3-(3,3,4,4,5,5,6,6,7,7,8,8,9,9,10,10,10-heptadecafluorodecyl)phenyl]phosphane (1/2)	343343-17-9	Cl[Pd]Cl.FC(F)(F)C(F)(F)C(F)(F)C(F)(F)C(F)(F)C(F)(F)C(F)(F)C(F)(F)CCC1=CC(=CC=C1)P(C1=CC=CC(CCC(F)(F)C(F)(F)C(F)(F)C(F)(F)C(F)(F)C(F)(F)C(F)(F)C(F)(F)F)=C1)C1=CC=CC(CCC(F)(F)C(F)(F)C(F)(F)C(F)(F)C(F)(F)C(F)(F)C(F)(F)C(F)(F)F)=C1.FC(F)(F)C(F)(F)C(F)(F)C(F)(F)C(F)(F)C(F)(F)C(F)(F)C(F)(F)CCC1=CC(=CC=C1)P(C1=CC=CC(CCC(F)(F)C(F)(F)C(F)(F)C(F)(F)C(F)(F)C(F)(F)C(F)(F)C(F)(F)F)=C1)C1=CC=CC(CCC(F)(F)C(F)(F)C(F)(F)C(F)(F)C(F)(F)C(F)(F)C(F)(F)C(F)(F)F)=C1		-10.23	0.66	3.82	P	OUT
QL01670	3,3,4,4,5,5,6,6,7,7,8,8,8-tridecafluorooctyl 2-chloroacrylate	96383-55-0	ClC(C(=O)OCCC(C(C(C(C(C(F)(F)F)(F)F)(F)F)(F)F)(F)F)(F)F)=C		-7.94	0.66	2.71	P	IN
QL01671	2-((8-chloro-1,1,2,2,3,3,4,4,5,5,6,6,7,7,8,8-hexadecafluorooctyl)oxy)-1,1,2,2-tetrafluoroethanesulfonic acid	763051-92-9	ClC(F)(C(F)(C(F)(C(F)(C(F)(C(F)(C(F)(C(F)(OC(F)(C(F)(S(=O)(O)=O)F)F)F)F)F)F)F)F)F)F		-8.88	0.69	2.74	P	OUT
QL01672	1,1,1,2,2,3,3-Heptafluoro-3-methoxypropane--(E)-1,2-dichloroethene (1/1)	218462-19-2	ClC=CCl.COC(F)(F)C(F)(F)C(F)(F)F		-4.70	0.60	1.91	P	OUT
QL01673	4-Chlorobenzenediazonium perfluorobutanoate perfluorobutanoic acid (111)	630-31-9	ClC1=CC=C(C=C1)[N+]#N.OC(=O)C(F)(F)C(F)(F)C(F)(F)F.[O-]C(=O)C(F)(F)C(F)(F)C(F)(F)F		-5.47	0.64	1.73	P	IN
QL01674	Perfluorooctanesulfonamido amine oxide	30295-51-3	CN([O-])C(CCN[S](=O)(=O)C(F)(F)C(F)(F)C(F)(F)C(F)(F)C(F)(F)C(F)(F)C(F)(F)C(F)(F)F)N(C)[O-]		-8.55	0.66	2.92	P	IN
QL01675	N-Methyl-N-trimethylsilylheptafluorobutyramide	53296-64-3	CN(C(=O)C(F)(F)C(F)(F)C(F)(F)F)[Si](C)(C)C		-6.63	0.67	1.91	P	IN
QL01676	N-Methyl-bis-heptafluorobutyramide	73980-71-9	CN(C(=O)C(F)(F)C(F)(F)C(F)(F)F)C(=O)C(F)(F)C(F)(F)C(F)(F)F		-7.80	0.67	2.42	P	IN
QL01677	4-Chloro-2-fluoro-N-(2-fluoro-3-{[4-(1,1,1,2,3,3,3-heptafluoropropan-2-yl)-2,6-dimethylphenyl]carbamoyl}phenyl)-N-methylbenzamide	862134-30-3	CN(C(=O)c1ccc(Cl)cc1F)c1cccc(C(=O)Nc2c(C)cc(cc2C)C(F)(C(F)(F)F)C(F)(F)F)c1F		-7.95	0.62	3.20	P	OUT
QL01678	4-Fluoro-N-(2-fluoro-3-{[4-(1,1,1,2,3,3,3-heptafluoropropan-2-yl)-2,6-dimethylphenyl]carbamoyl}phenyl)-N-methyl-2-nitrobenzamide	862134-28-9	CN(C(=O)c1ccc(F)cc1[N+]([O-])=O)c1cccc(C(=O)Nc2c(C)cc(cc2C)C(F)(C(F)(F)F)C(F)(F)F)c1F		-7.90	0.62	3.19	P	OUT
QL01679	2-Chloro-4-fluoro-N-(2-fluoro-3-{[4-(1,1,1,2,3,3,3-heptafluoropropan-2-yl)-2,6-dimethylphenyl]carbamoyl}phenyl)-N-methylbenzamide	862134-29-0	CN(C(=O)c1ccc(F)cc1Cl)c1cccc(C(=O)Nc2c(C)cc(cc2C)C(F)(C(F)(F)F)C(F)(F)F)c1F		-7.95	0.62	3.20	P	OUT
QL01680	2,4-Difluoro-N-(2-fluoro-3-{[4-(1,1,1,2,3,3,3-heptafluoropropan-2-yl)-2,6-dimethylphenyl]carbamoyl}phenyl)-N-methylbenzamide	862134-26-7	CN(C(=O)c1ccc(F)cc1F)c1cccc(C(=O)Nc2c(C)cc(cc2C)C(F)(C(F)(F)F)C(F)(F)F)c1F		-7.95	0.62	3.20	P	OUT
QL01681	1-(3,3,4,4,5,5,6,6,7,7,8,8,9,9,10,10,10-Heptadecafluorodecyl)-N,N,1,1-tetramethylsilanamine	1221157-06-7	CN(C)[Si](C)(C)CCC(F)(F)C(F)(F)C(F)(F)C(F)(F)C(F)(F)C(F)(F)C(F)(F)C(F)(F)F		-8.57	0.68	2.74	P	IN
QL01682	N,N,1,1-Tetramethyl-1-(3,3,4,4,5,5,6,6,6-nonafluorohexyl)silanamine	1221157-07-8	CN(C)[Si](C)(C)CCC(F)(F)C(F)(F)C(F)(F)C(F)(F)F		-7.26	0.67	2.24	P	IN
QL01683	1-(3,3,4,4,5,5,6,6,7,7,8,8,9,9,10,10,10-Heptadecafluorodecyl)-N,N,N',N',1-pentamethylsilanediamine	186599-47-3	CN(C)[Si](C)(CCC(F)(F)C(F)(F)C(F)(F)C(F)(F)C(F)(F)C(F)(F)C(F)(F)C(F)(F)F)N(C)C		-8.43	0.67	2.72	P	IN

QL01684	N,N,N',N',1-Pentamethyl-1-(3,3,4,4,5,5,6,6,7,7,8,8,8-tridecafluorooctyl)silanediamine	879881-65-9	CN(C)[Si](C)(CCC(F)(F)C(F)(F)C(F)(F)C(F)(F)C(F)(F)C(F)(F)F)N(C)C		-7.82	0.67	2.49	P	IN
QL01685	1-(3,3,4,4,5,5,6,6,7,7,8,8,9,9,10,10,10-Heptadecafluorodecyl)-N,N,N',N',N'',N''-hexamethylsilanetriamine	186599-45-1	CN(C)[Si](CCC(F)(F)C(F)(F)C(F)(F)C(F)(F)C(F)(F)C(F)(F)C(F)(F)C(F)(F)F)(N(C)C)N(C)C		-8.23	0.67	2.69	P	IN
QL01686	N,N,N',N',N'',N''-Hexamethyl-1-(1,1,2,2,3,3,6,6,6-nonafluorohexyl)silanetriamine	186599-46-2	CN(C)[Si](N(C)C)(N(C)C)C(F)(F)C(F)(F)C(F)(F)CCC(F)(F)F		-6.59	0.65	2.16	P	IN
QL01687	N,N-dimethyl-2H-perfluoroethanamine	1550-50-1	CN(C)C(F)(F)C(F)F		-4.61	0.63	1.39	P	OUT
QL01688	Benzenamine, 4-(heptadecafluorooctyl)-N,N-dimethyl-	80791-09-9	CN(C)C1=CC=C(C=C1)C(F)(F)C(F)(F)C(F)(F)C(F)(F)C(F)(F)C(F)(F)C(F)(F)C(F)(F)F		-8.58	0.67	2.90	P	IN
QL01689	N'-{[4-(Dimethylamino)phenyl]methylidene}-2,2,3,3,4,4,5,5,6,6,7,7,7-tridecafluoroheptanehydrazide	328016-16-6	CN(C)c1ccc(C=NNC(=O)C(F)(F)C(F)(F)C(F)(F)C(F)(F)C(F)(F)C(F)(F)F)cc1		-8.28	0.64	3.06	P	OUT
QL01690	4-(1,1,1,2,3,3,3-Heptafluoropropan-2-yl)-N,N-dimethylaniline	2396-19-2	CN(C)c1ccc(cc1)C(F)(C(F)(F)F)C(F)(F)F		-6.20	0.64	2.10	P	IN
QL01691	N,N-Dimethyl-4-(tridecafluorohexyl)aniline	141822-33-5	CN(C)c1ccc(cc1)C(F)(F)C(F)(F)C(F)(F)C(F)(F)C(F)(F)C(F)(F)F		-8.00	0.66	2.71	P	IN
QL01692	4-(Dimethylamino)phenyl nonafluorobutane-1-sulfonate	1463426-91-6	CN(C)c1ccc(OS(=O)(=O)C(F)(F)C(F)(F)C(F)(F)C(F)(F)F)cc1		-7.66	0.65	2.67	P	IN
QL01693	1-(Dimethylamino)-4,4,5,5,6,6,7,7,7-nonafluoroheptan-2-ol	1023972-12-4	CN(C)CC(O)CC(F)(F)C(F)(F)C(F)(F)C(F)(F)F		-6.74	0.65	2.26	P	IN
QL01694	1-Propanaminium, 2-hydroxy-N,N,N-trimethyl-, 3-[(.gamma.-.omega.-perfluoro-C6-20-alkyl)thio] derivs., chlorides	70983-60-7	CN(C)CC(O)SC(F)(F)C(F)(F)C(F)(F)C(F)(F)C(F)(F)C(F)(F)F		-7.59	0.66	2.52	P	IN
QL01695	N,N-Dimethyl-3-((perfluorohexyl)ethylsulfonyl)aminopropanamine N-oxide	80475-32-7	CN(C)CCC[NH+]([O-])[S](=O)(=O)CCC(F)(F)C(F)(F)C(F)(F)C(F)(F)C(F)(F)C(F)(F)F		-8.20	0.65	2.91	P	IN
QL01696	N-[4-(Dimethylamino)butyl]-2,2,3,3,4,4,5,5,6,6,7,7,8,8,8-pentadecafluorooctanamide	118454-01-6	CN(C)CCCCNC(=O)C(F)(F)C(F)(F)C(F)(F)C(F)(F)C(F)(F)C(F)(F)C(F)(F)F		-8.20	0.65	2.96	P	IN
QL01697	1-Propanesulfonic acid, 3-[[3-(dimethylamino)propyl][(tridecafluorohexyl)sulfonyl]amino]-2-hydroxy-, monosodium salt	73772-32-4	CN(C)CCCN(CC(CS(=O)(=O)[O-])O)S(=O)(=O)C(C(C(C(C(C(F)(F)F)(F)F)(F)F)(F)F)(F)F)(F)F.[Na+]		-7.50	0.63	2.83	P	OUT
QL01698	1-((3-(Dimethylamino)propyl)amino)-(3-(perfluorooctyl))propan-2-ol	94159-81-6	CN(C)CCCNCC(O)CC(F)(F)C(F)(F)C(F)(F)C(F)(F)C(F)(F)C(F)(F)C(F)(F)C(F)(F)F		-8.53	0.65	3.14	P	OUT
QL01699	Perfluorobutane sulfonamido amine	68555-77-1	CN(C)CCCNS(=O)(=O)C(F)(F)C(F)(F)C(F)(F)C(F)(F)F		-7.27	0.64	2.54	P	IN
QL01700	62 Fluorotelomer sulfonamide amine	34455-22-6	CN(C)CCCNS(=O)(=O)CCC(F)(F)C(F)(F)C(F)(F)C(F)(F)C(F)(F)C(F)(F)F		-8.10	0.65	2.92	P	IN
QL01701	1,1,2,2,3,3,4,4,5,5,6,6,6-Tridecafluoro-N,N-dimethylhexane-1-sulfonamide	1270179-82-2	CN(C)S(=O)(=O)C(F)(F)C(F)(F)C(F)(F)C(F)(F)C(F)(F)C(F)(F)F		-7.72	0.68	2.24	P	IN
QL01702	N-{3-[(3,3,4,4,5,5,6,6,7,7,8,8,9,9,10,10,10-Heptadecafluorodecyl)sulfanyl]-2-hydroxypropyl}-N-methylglycine	93128-66-6	CN(CC(O)CSCCC(F)(F)C(F)(F)C(F)(F)C(F)(F)C(F)(F)C(F)(F)C(F)(F)C(F)(F)F)CC(O)=O		-8.68	0.65	3.23	P	OUT
QL01703	Isooctanesulfonamide, heptadecafluoro-N-(2-hydroxyethyl)-N-methyl-	93894-65-6	CN(CCO)[S](=O)(=O)C(F)(F)C(F)(F)C(F)(F)C(F)(F)C(F)(F)C(F)(C(F)(F)F)C(F)(F)F		-8.15	0.67	2.67	P	IN
QL01704	N-(2-hydroxyethyl)-N-methylperfluoro-2-methylpropanesulfonamide	93762-11-9	CN(CCO)S(=O)(=O)C(F)(F)C(F)(C(F)(F)F)C(F)(F)F		-6.60	0.65	2.14	P	IN
QL01705	1-Pentanesulfonamide, 1,1,2,2,3,3,4,4,5,5,5-undecafluoro-N-(2-hydroxyethyl)-N-methyl-	68555-74-8	CN(CCO)S(=O)(=O)C(F)(F)C(F)(F)C(F)(F)C(F)(F)C(F)(F)F		-7.48	0.66	2.45	P	IN
QL01706	1-Butanesulfonamide, 1,1,2,2,3,3,4,4,4-nonafluoro-N-(2-hydroxyethyl)-N-methyl-	34454-97-2	CN(CCO)S(=O)(=O)C(F)(F)C(F)(F)C(F)(F)C(F)(F)F		-6.91	0.65	2.24	P	IN
QL01707	3,3,4,4,5,5,6,6,7,7,8,8,8-Tridecafluoro-N-(2-hydroxyethyl)-N-methyl-1-octanesulfonamide	51619-73-9	CN(CCO)S(=O)(=O)CCC(F)(F)C(F)(F)C(F)(F)C(F)(F)C(F)(F)C(F)(F)F		-7.98	0.66	2.75	P	IN
QL01708	2-[(Heptadecafluorooctyl)(methyl)amino]ethyl prop-2-enoate	93491-54-4	CN(CCOC(=O)C=C)C(F)(F)C(F)(F)C(F)(F)C(F)(F)C(F)(F)C(F)(F)C(F)(F)C(F)(F)F		-8.40	0.66	2.95	P	IN
QL01709	2-Propenoic acid, 2-[methyl[(undecafluoropentyl)sulfonyl]amino]ethyl ester	67584-56-9	CN(CCOC(=O)C=C)S(=O)(=O)C(F)(F)C(F)(F)C(F)(F)C(F)(F)C(F)(F)F		-7.79	0.65	2.76	P	IN
QL01710	2-Propenoic acid, 2-[methyl[(nonafluorobutyl)sulfonyl]amino]ethyl ester	67584-55-8	CN(CCOC(=O)C=C)S(=O)(=O)C(F)(F)C(F)(F)C(F)(F)C(F)(F)F		-7.51	0.64	2.71	P	IN
QL01711	Bis[N-methyl(perfluorobutyl)sulfonamidoethyl]phosphoric acid	120945-47-3	CN(CCOP(O)(=O)OCCN(C)S(=O)(=O)C(F)(F)C(F)(F)C(F)(F)C(F)(F)F)S(=O)(=O)C(F)(F)C(F)(F)C(F)(F)C(F)(F)F		-9.26	0.66	3.28	P	OUT
QL01712	2-{Methyl[(nonafluorobutyl)sulfonyl]amino}ethyl phosphate	147545-41-3	CN(CCOP(O)(O)=O)S(=O)(=O)C(F)(F)C(F)(F)C(F)(F)C(F)(F)F		-7.63	0.66	2.52	P	IN

QL01713	2,2,3,3,4,4,4-Heptafluoro-N-hydroxy-N-methylbutanamide	193008-44-5	CN(O)C(=O)C(F)(F)C(F)(F)C(F)(F)F		-6.21	0.66	1.83	P	IN
QL01714	N,N-Dimethyl perfluorooctane sulfonamide	213181-78-3	CN(S(=O)(C(F)(F)C(F)(F)C(F)(F)C(F)(F)C(F)(F)C(F)(F)C(F)(F)C(F)(F)F)=O)C		-8.35	0.69	2.51	P	IN
QL01715	1,1,2,2,3,3,4,4,4-Nonafluoro-N-methyl-N-(trimethylstannyl)butane-1-sulfinamide	41006-33-1	CN(S(=O)C(F)(F)C(F)(F)C(F)(F)C(F)(F)F)[Sn](C)(C)C		-7.27	0.67	2.17	P	IN
QL01716	Heptadecafluoro-N-methylisooctanesulphonamide	93894-71-4	CN[S](=O)(=O)C(F)(F)C(F)(F)C(F)(F)C(F)(F)C(F)(F)C(F)(C(F)(F)F)C(F)(F)F		-8.03	0.68	2.43	P	IN
QL01717	1-Heptanesulfonamide, 1,1,2,2,3,3,4,4,5,5,6,6,7,7,7-pentadecafluoro-N-methyl-	68259-14-3	CN[S](=O)(=O)C(F)(F)C(F)(F)C(F)(F)C(F)(F)C(F)(F)C(F)(F)C(F)(F)F		-8.06	0.68	2.44	P	IN
QL01718	1-Hexanesulfonamide, 1,1,2,2,3,3,4,4,5,5,6,6,6-tridecafluoro-N-methyl-	68259-15-4	CN[S](=O)(=O)C(F)(F)C(F)(F)C(F)(F)C(F)(F)C(F)(F)C(F)(F)F		-7.58	0.68	2.23	P	IN
QL01719	1-Pentanesulfonamide, 1,1,2,2,3,3,4,4,5,5,5-undecafluoro-N-methyl-	68298-13-5	CN[S](=O)(=O)C(F)(F)C(F)(F)C(F)(F)C(F)(F)C(F)(F)F		-7.35	0.68	2.15	P	IN
QL01720	1-Butanesulfonamide, 1,1,2,2,3,3,4,4,4-nonafluoro-N-methyl-	68298-12-4	CN[S](=O)(=O)C(F)(F)C(F)(F)C(F)(F)C(F)(F)F		-6.72	0.67	1.89	P	IN
QL01721	1-Methyl-2-(nonafluorobutyl)-1H-indole	1608112-35-1	Cn1c(cc2ccccc12)C(F)(F)C(F)(F)C(F)(F)C(F)(F)F		-6.78	0.65	2.25	P	IN
QL01722	3,3,4,4,5,5,5-Heptafluoro-1-(3-methyl-1,3-benzothiazol-2(3H)-ylidene)pentan-2-one	41131-07-1	CN1C(Sc2ccccc12)=CC(=O)C(F)(F)C(F)(F)C(F)(F)F		-6.74	0.64	2.40	P	OUT
QL01723	1H-Pyrrole, 1-methyl-2-(tridecafluorohexyl)-	64435-67-2	CN1C=CC=C1C(F)(F)C(F)(F)C(F)(F)C(F)(F)C(F)(F)C(F)(F)F		-7.28	0.67	2.24	P	IN
QL01724	1-(1-Methyl-1H-indol-3-yl)thiolan-1-ium nonafluorobutane-1-sulfonate	867373-18-0	Cn1cc([S+]2CCCC2)c2ccccc12.[O-]S(=O)(=O)C(F)(F)C(F)(F)C(F)(F)C(F)(F)F		-5.98	0.64	2.01	P	IN
QL01725	2,2,3,3,4,4,5,5,6,6,7,7,7-Tridecafluoro-1-(4-methylpiperazin-1-yl)heptan-1-one	560092-29-7	CN1CCN(CC1)C(=O)C(F)(F)C(F)(F)C(F)(F)C(F)(F)C(F)(F)C(F)(F)F		-8.00	0.66	2.71	P	IN
QL01726	2,2,3,3,4,4,4-Heptafluoro-1-(4-methylpiperazin-1-yl)butan-1-one	499975-92-7	CN1CCN(CC1)C(=O)C(F)(F)C(F)(F)C(F)(F)F		-6.61	0.64	2.27	P	IN
QL01727	Heptadecafluorononanoic acid--N-methylmethanamine (1/1)	77032-24-7	CNC.OC(=O)C(F)(F)C(F)(F)C(F)(F)C(F)(F)C(F)(F)C(F)(F)C(F)(F)C(F)(F)F		-7.85	0.65	2.76	P	IN
QL01728	2,3,3,3-Tetrafluoro-2-[1,1,2,3,3,3-hexafluoro-2-(heptafluoropropoxy)propoxy]propanoic acid--N-methylmethanamine (1/1)	328065-54-9	CNC.OC(=O)C(F)(OC(F)(F)C(F)(OC(F)(F)C(F)(F)C(F)(F)F)C(F)(F)F)C(F)(F)F		-7.53	0.64	2.71	P	IN
QL01729	2,2,3,3,4,4,5,5,6,6,7,7,8,8,9,9,9-Heptadecafluoro-N-methylnonanamide	314053-71-9	CNC(=O)C(F)(F)C(F)(F)C(F)(F)C(F)(F)C(F)(F)C(F)(F)C(F)(F)C(F)(F)F		-8.04	0.67	2.51	P	IN
QL01730	N-Methylperfluoroheptanamide	89932-74-1	CNC(=O)C(F)(F)C(F)(F)C(F)(F)C(F)(F)C(F)(F)C(F)(F)F		-7.32	0.67	2.24	P	IN
QL01731	2,2,3,3,4,4,5,5,5-Nonafluoro-N-methylpentanamide	115585-00-7	CNC(=O)C(F)(F)C(F)(F)C(F)(F)C(F)(F)F		-6.37	0.66	1.90	P	IN
QL01732	2,2,3,3,4,4,4-Heptafluoro-N-methylbutanamide	4314-42-5	CNC(=O)C(F)(F)C(F)(F)C(F)(F)F		-5.98	0.65	1.84	P	IN
QL01733	3,3,4,4,5,5,6,6,6-Nonafluoro-2-hydroxy-N,N~2~-dimethylnorleucinamide	107070-94-0	CNC(=O)C(O)(NC)C(F)(F)C(F)(F)C(F)(F)C(F)(F)F		-6.68	0.65	2.15	P	IN
QL01734	4-(Heptadecafluorooctyl)-N-methylbenzamide	89932-69-4	CNC(=O)C1=CC=C(C=C1)C(F)(F)C(F)(F)C(F)(F)C(F)(F)C(F)(F)C(F)(F)C(F)(F)C(F)(F)F		-8.48	0.66	2.95	P	IN
QL01735	N-Methyl-4-(nonafluorobutyl)aniline	1426840-85-8	CNc1ccc(cc1)C(F)(F)C(F)(F)C(F)(F)C(F)(F)F		-7.03	0.64	2.47	P	IN
QL01736	5-(Heptafluoropropyl)-N,1-dimethyl-1H-1,2,4-triazol-3-amine	714975-28-7	CNc1nc(n(C)n1)C(F)(F)C(F)(F)C(F)(F)F		-6.35	0.64	2.16	P	IN
QL01737	5-(Heptafluoropropyl)-N-methyl-1,3,4-oxadiazol-2-amine	714975-29-8	CNc1nnc(o1)C(F)(F)C(F)(F)C(F)(F)F		-6.35	0.64	2.18	P	IN
QL01738	2,2,3,3,4,4,4-Heptafluoro-N-methylbutan-1-amine	755-42-0	CNCC(F)(F)C(F)(F)C(F)(F)F		-5.71	0.64	1.85	P	IN
QL01739	methyl-(7,7,8,8,9,9,10,10,10-nonafluorodecyl)amine	228569-98-0	CNCCCCCCC(F)(F)C(F)(F)C(F)(F)C(F)(F)F		-6.67	0.61	2.68	P	OUT
QL01740	N-Methyl-4-[3,4,4,4-tetrafluoro-3-(trifluoromethyl)butane-1-sulfonyl]butan-1-amine	1353009-38-7	CNCCCCS(=O)(=O)CCC(F)(C(F)(F)F)C(F)(F)F		-6.19	0.61	2.41	P	OUT
QL01741	Silane, trimethoxy(3,3,4,4,5,5,6,6,6-nonafluorohexyl)-		CO[Si](CCC(C(C(C(F)(F)F)(F)F)(F)F)(F)F)(OC)OC		-6.45	0.64	2.23	P	IN
QL01742	(3,3,4,4,5,5,6,6,7,7,8,8,9,9,10,10,11,11,12,12,12-Henicosafluorododecyl)(trimethoxy)silane	123445-18-1	CO[Si](CCC(F)(F)C(F)(F)C(F)(F)C(F)(F)C(F)(F)C(F)(F)C(F)(F)C(F)(F)C(F)(F)C(F)(F)F)(OC)OC		-8.60	0.67	2.93	P	IN
QL01743	1H,1H,2H,2H-Perfluorodecyltrimethoxysilane	83048-65-1	CO[Si](CCC(F)(F)C(F)(F)C(F)(F)C(F)(F)C(F)(F)C(F)(F)C(F)(F)C(F)(F)F)(OC)OC		-8.04	0.66	2.73	P	IN
QL01744	1,6-bis(trimethoxysilanylethyl)perfluorohexane	94403-04-0	CO[Si](CCC(F)(F)C(F)(F)C(F)(F)C(F)(F)C(F)(F)C(F)(F)CC[Si](OC)(OC)OC)(OC)OC		-7.46	0.63	2.85	P	OUT
QL01745	(3,3,4,4,5,5,5-Heptafluoropentyl)(trimethoxy)silane	109134-39-6	CO[Si](CCC(F)(F)C(F)(F)C(F)(F)F)(OC)OC		-5.89	0.63	2.09	P	OUT

QL01746	3,3,4,4,5,5,6,6,7,7,8,8,8-Tridecafluorooctyl [3-(trimethoxysilyl)propyl]carbamate	1421688-36-9	CO[Si](CCCNC(=O)OCCC(F)(F)C(F)(F)C(F)(F)C(F)(F)C(F)(F)C(F)(F)F)(OC)OC		-7.86	0.63	3.08	P	OUT
QL01747	3-(Heptafluoroisopropoxy)propyltrimethoxysilane	19116-61-1	CO[Si](CCCOC(F)(C(F)(F)F)C(F)(F)F)(OC)OC		-5.36	0.61	2.01	P	OUT
QL01748	[3-(Heptafluoropropoxy)propyl](trimethoxy)silane	903890-42-6	CO[Si](CCCOC(F)(F)C(F)(F)C(F)(F)F)(OC)OC		-6.06	0.61	2.34	P	OUT
QL01749	Methyl 4,4,5,5,6,6,6-heptafluoro-2-methylidenehexanoate	88021-90-3	COC(=O)C(=C)CC(F)(F)C(F)(F)C(F)(F)F		-6.25	0.64	2.13	P	IN
QL01750	Methyl 3,3,4,4,5,5,6,6,6-nonafluoro-2-oxohexanoate	74067-16-6	COC(=O)C(=O)C(F)(F)C(F)(F)C(F)(F)C(F)(F)F		-6.78	0.65	2.18	P	IN
QL01751	Methyl 5-cyano-α-((perfluoropropyl)ethyl)sulfinyl-α,β-dimethyl-3-thiophenepropanoate	1137678-56-8	COC(=O)C(C)(C(C)C1=CSC(=C1)C#N)S(=O)CCC(F)(F)C(F)(F)C(F)(F)F		-7.13	0.62	2.76	P	OUT
QL01752	Methyl 4,4,5,5,6,6,6-heptafluoro-2-methylhex-2-enoate	88021-94-7	COC(=O)C(C)=CC(F)(F)C(F)(F)C(F)(F)F		-6.25	0.64	2.13	P	IN
QL01753	Hexanoic acid, 4,4,5,5,6,6,6-heptafluoro-2-methyl-, methyl ester	88021-93-6	COC(=O)C(C)CC(F)(F)C(F)(F)C(F)(F)F		-6.25	0.64	2.13	P	IN
QL01754	Methyl 2-(heptafluoropropyl)-2-(trifluoromethyl)pent-4-en-1-	83846-71-3	COC(=O)C(CC=C)(C(F)(F)F)C(F)(F)C(F)(F)C(F)(F)F		-5.65	0.64	1.84	P	IN
QL01755	Methyl 2-chloro-3,3,4,4,5,5,6,6,6-nonafluorohexanoate	116356-70-8	COC(=O)C(Cl)C(F)(F)C(F)(F)C(F)(F)C(F)(F)F		-6.78	0.65	2.18	P	IN
QL01756	Methyl tetrafluoro-2-(trifluoromethyl)propionate	680-05-7	COC(=O)C(F)(C(F)(F)F)C(F)(F)F		-5.07	0.64	1.43	P	IN
QL01757	Methyl perfluorooctadecanoate	16753-33-6	COC(=O)C(F)(F)C(F)(F)C(F)(F)C(F)(F)C(F)(F)C(F)(F)C(F)(F)C(F)(F)C(F)(F)C(F)(F)C(F)(F)C(F)(F)C(F)(F)C(F)(F)C(F)(F)C(F)(F)C(F)(F)F		-10.07	0.69	3.38	P	OUT
QL01758	Methyl perfluorohexadecanoate	165457-57-8	COC(=O)C(F)(F)C(F)(F)C(F)(F)C(F)(F)C(F)(F)C(F)(F)C(F)(F)C(F)(F)C(F)(F)C(F)(F)C(F)(F)C(F)(F)C(F)(F)C(F)(F)C(F)(F)F		-9.74	0.69	3.23	P	OUT
QL01759	Methyl perfluoropentadecanoate	203303-00-8	COC(=O)C(F)(F)C(F)(F)C(F)(F)C(F)(F)C(F)(F)C(F)(F)C(F)(F)C(F)(F)C(F)(F)C(F)(F)C(F)(F)C(F)(F)C(F)(F)C(F)(F)F		-9.49	0.68	3.11	P	OUT
QL01760	Methyl perfluorotetradecanoate	203302-99-2	COC(=O)C(F)(F)C(F)(F)C(F)(F)C(F)(F)C(F)(F)C(F)(F)C(F)(F)C(F)(F)C(F)(F)C(F)(F)C(F)(F)C(F)(F)C(F)(F)F		-9.37	0.68	3.07	P	OUT
QL01761	Methyl pentacosafluorotridecanoate	185230-63-1	COC(=O)C(F)(F)C(F)(F)C(F)(F)C(F)(F)C(F)(F)C(F)(F)C(F)(F)C(F)(F)C(F)(F)C(F)(F)C(F)(F)C(F)(F)F		-9.08	0.68	2.94	P	OUT
QL01762	Methyl Perfluorododecanoate	56554-52-0	COC(=O)C(F)(F)C(F)(F)C(F)(F)C(F)(F)C(F)(F)C(F)(F)C(F)(F)C(F)(F)C(F)(F)C(F)(F)C(F)(F)F		-8.94	0.68	2.89	P	OUT
QL01763	Methyl perfluoroundecanoate	203302-98-1	COC(=O)C(F)(F)C(F)(F)C(F)(F)C(F)(F)C(F)(F)C(F)(F)C(F)(F)C(F)(F)C(F)(F)C(F)(F)F		-8.61	0.68	2.74	P	IN
QL01764	Methyl Perfluorodecanoate	307-79-9	COC(=O)C(F)(F)C(F)(F)C(F)(F)C(F)(F)C(F)(F)C(F)(F)C(F)(F)C(F)(F)C(F)(F)F		-8.44	0.68	2.68	P	IN
QL01765	Methyl Perfluorononanoate	51502-45-5	COC(=O)C(F)(F)C(F)(F)C(F)(F)C(F)(F)C(F)(F)C(F)(F)C(F)(F)C(F)(F)F		-8.04	0.67	2.51	P	IN
QL01766	Methyl perfluorooctanoate	376-27-2	COC(=O)C(F)(F)C(F)(F)C(F)(F)C(F)(F)C(F)(F)C(F)(F)C(F)(F)F		-7.83	0.67	2.45	P	IN
QL01767	Methyl perfluoroheptanoate	14312-89-1	COC(=O)C(F)(F)C(F)(F)C(F)(F)C(F)(F)C(F)(F)C(F)(F)F		-7.32	0.67	2.24	P	IN
QL01768	Methyl perfluorohexanoate	424-18-0	COC(=O)C(F)(F)C(F)(F)C(F)(F)C(F)(F)C(F)(F)F		-7.06	0.66	2.17	P	IN
QL01769	Methyl nonafluorovalerate	13038-26-1	COC(=O)C(F)(F)C(F)(F)C(F)(F)C(F)(F)F		-6.37	0.66	1.90	P	IN
QL01770	Methyl heptafluorobutyrate	356-24-1	COC(=O)C(F)(F)C(F)(F)C(F)(F)F		-5.98	0.65	1.84	P	IN
QL01771	Methyl 4-trifluoroethenoxy-2,2,3,3,4,4-hexafluorobutanoate	19190-61-5	COC(=O)C(F)(F)C(F)(F)C(F)(F)OC(F)=C(F)F		-6.32	0.63	2.23	P	OUT
QL01772	Methyl 3-chloroperfluoropropanoate	127589-63-3	COC(=O)C(F)(F)C(F)(F)Cl		-4.92	0.63	1.49	P	IN
QL01773	Methyl pentafluoropropionate	378-75-6	COC(=O)C(F)(F)C(F)(F)F		-4.92	0.63	1.49	P	IN
QL01774	Methyl 2,2,3,3-tetrafluoro-3-[(1,1,1,2,3,3,3-heptafluoropropan-2-yl)oxy]propanoate	51502-43-3	COC(=O)C(F)(F)C(F)(F)OC(F)(C(F)(F)F)C(F)(F)F		-6.81	0.66	2.14	P	IN
QL01775	Methyl perfluoro(8-(fluoroformyl)-5-methyl-4,7-dioxanonanoate)	69116-73-0	COC(=O)C(F)(F)C(F)(F)OC(F)(C(F)(F)F)C(F)(F)OC(F)(C(F)=O)C(F)(F)F		-7.44	0.66	2.45	P	IN
QL01776	Methyl perfluoro(3-(1-ethenyloxypropan-2-yloxy)propanoate)	63863-43-4	COC(=O)C(F)(F)C(F)(F)OC(F)(C(F)(F)F)C(F)(F)OC(F)=C(F)F		-6.99	0.64	2.42	P	IN
QL01777	Perfluoromethoxypropionic acid methyl ester	356-69-4	COC(=O)C(F)(F)C(F)(F)OC(F)(F)F		-5.67	0.63	1.89	P	IN
QL01778	Methyl perfluoro-3-(3-methoxypropoxy)-3H-propanoate	958445-54-0	COC(=O)C(F)(F)C(F)OC(F)(F)C(F)(F)C(F)(F)OC(F)(F)F		-7.22	0.65	2.51	P	IN
QL01779	Methyl difluoro[1,1,2,2-tetrafluoro-2-(nonafluorobutoxy)ethoxy]acetate	129301-40-2	COC(=O)C(F)(F)OC(F)(F)C(F)(F)OC(F)(F)C(F)(F)C(F)(F)C(F)(F)F		-8.06	0.66	2.70	P	IN
QL01780	Methyl perfluoro-3,6,9-trioxatridecanoate	330562-42-0	COC(=O)C(F)(F)OC(F)(F)C(F)(F)OC(F)(F)C(F)(F)OC(F)(F)C(F)(F)C(F)(F)C(F)(F)F		-8.62	0.67	2.94	P	IN
QL01781	Methyl difluoro{1,1,2,2-tetrafluoro-2-[1,1,2,2-tetrafluoro-2-(trifluoromethoxy)ethoxy]ethoxy}acetate	169289-58-1	COC(=O)C(F)(F)OC(F)(F)C(F)(F)OC(F)(F)C(F)(F)OC(F)(F)F		-7.67	0.65	2.69	P	IN
QL01782	Methyl perfluoro-3,6-dioxaheptanoate	39187-41-2	COC(=O)C(F)(F)OC(F)(F)C(F)(F)OC(F)(F)F		-6.48	0.64	2.23	P	IN

QL01783	Methyl 2,3,3,3-tetrafluoro-2-[(undecafluoropentyl)oxy]propanoate	220369-95-9	COC(=O)C(F)(OC(F)(F)C(F)(F)C(F)(F)C(F)(F)C(F)(F)F)C(F)(F)F		-7.60	0.67	2.40	P	IN
QL01784	Methyl perfluoro(2-propoxypropanoate)	13140-34-6	COC(=O)C(F)(OC(F)(F)C(F)(F)C(F)(F)F)C(F)(F)F		-6.61	0.66	2.03	P	IN
QL01785	Methyl 2,3,3,3-tetrafluoro-2-(pentafluoroethoxy)propanoate	117516-09-3	COC(=O)C(F)(OC(F)(F)C(F)(F)F)C(F)(F)F		-6.03	0.65	1.84	P	IN
QL01786	Methyl 2,3,3,3-tetrafluoro-2-[1,1,2,3,3,3-hexafluoro-2-(heptafluoropropoxy)propoxy]propanoate	26131-32-8	COC(=O)C(F)(OC(F)(F)C(F)(OC(F)(F)C(F)(F)C(F)(F)F)C(F)(F)F)C(F)(F)F		-7.71	0.66	2.48	P	IN
QL01787	Methylperfluoro-2,5,8-trimethyl-3,6,9-trioxadodecanoic acid	39187-47-8	COC(=O)C(F)(OC(F)(F)C(F)(OC(F)(F)C(F)(OC(F)(F)C(F)(F)C(F)(F)F)C(F)(F)F)C(F)(F)F)C(F)(F)F		-8.43	0.67	2.79	P	IN
QL01788	Perfluorotetramethyltetraoxapentadecanoicacidme	133609-46-8	COC(=O)C(F)(OC(F)(F)C(F)(OC(F)(F)C(F)(OC(F)(F)C(F)(OC(F)(F)C(F)(F)C(F)(F)F)C(F)(F)F)C(F)(F)F)C(F)(F)F)C(F)(F)F		-9.05	0.67	3.08	P	OUT
QL01789	Methyl 2,4,4,5,7,7,8,10,10,11,13,13,14,16,16,17,17,18,18,18-icosafluoro-2,5,8,11,14-pentakis(trifluoromethyl)-3,6,9,12,15-pentaoxaoctadecan-1-oate	1005033-64-6	COC(=O)C(F)(OC(F)(F)C(F)(OC(F)(F)C(F)(OC(F)(F)C(F)(OC(F)(F)C(F)(OC(F)(F)C(F)(F)C(F)(F)F)C(F)(F)F)C(F)(F)F)C(F)(F)F)C(F)(F)F)C(F)(F)F		-9.52	0.67	3.29	P	OUT
QL01790	Methyl 2,3,4,4,5,5,6,6,6-nonafluorohex-2-enoate	88022-46-2	COC(=O)C(F)=C(F)C(F)(F)C(F)(F)C(F)(F)F		-6.43	0.64	2.17	P	IN
QL01791	Methyl 3-amino-4,4,5,5,6,6,6-heptafluorohex-2-enoate	106203-17-2	COC(=O)C=C(N)C(F)(F)C(F)(F)C(F)(F)F		-6.32	0.64	2.18	P	IN
QL01792	Methyl 4,4,5,5,6,6,6-heptafluorohex-2-enoate	813-06-9	COC(=O)C=CC(F)(F)C(F)(F)C(F)(F)F		-6.15	0.63	2.15	P	OUT
QL01793	Methyl 3-(heptadecafluorooctyl)benzoate	80791-11-3	COC(=O)C1=CC(=CC=C1)C(F)(F)C(F)(F)C(F)(F)C(F)(F)C(F)(F)C(F)(F)C(F)(F)C(F)(F)F		-8.37	0.66	2.90	P	IN
QL01794	Methyl 4-(heptadecafluorooctyl)benzoate	80791-12-4	COC(=O)C1=CC=C(C=C1)C(F)(F)C(F)(F)C(F)(F)C(F)(F)C(F)(F)C(F)(F)C(F)(F)C(F)(F)F		-8.48	0.66	2.95	P	IN
QL01795	Methyl 4-(nonafluorobutyl)benzoate	88951-00-2	COC(=O)C1=CC=C(C=C1)C(F)(F)C(F)(F)C(F)(F)C(F)(F)F		-7.15	0.64	2.56	P	IN
QL01796	Methyl 4-(heptafluoropropyl)benzoate	64299-47-4	COC(=O)C1=CC=C(C=C1)C(F)(F)C(F)(F)C(F)(F)F		-6.79	0.63	2.50	P	OUT
QL01797	Methyl 5-(heptafluoropropyl)furan-2-carboxylate	104939-25-5	COC(=O)C1=CC=C(O1)C(F)(F)C(F)(F)C(F)(F)F		-6.52	0.63	2.29	P	IN
QL01798	Methyl 2-(heptafluoropropyl)-6-methoxybenzoate	109296-89-1	COC(=O)c1c(OC)cccc1C(F)(F)C(F)(F)C(F)(F)F		-5.72	0.62	2.12	P	OUT
QL01799	Methyl 2-methoxy-6-[(1,1,2,2,3,3,4,4,4-nonafluorobutane-1-sulfonyl)oxy]benzoate	475630-76-3	COC(=O)c1c(OC)cccc1OS(=O)(=O)C(F)(F)C(F)(F)C(F)(F)C(F)(F)F		-6.88	0.63	2.50	P	OUT
QL01800	Methyl 3,4,5-tris[1,1,2-trifluoro-2-(heptafluoropropoxy)ethoxy]benzoate	1233312-86-1	COC(=O)c1cc(OC(F)(F)C(F)OC(F)(F)C(F)(F)C(F)(F)F)c(OC(F)(F)C(F)OC(F)(F)C(F)(F)C(F)(F)F)c(OC(F)(F)C(F)OC(F)(F)C(F)(F)C(F)(F)F)c1		-8.78	0.66	3.17	P	IN
QL01801	Methyl 4-(1,1,1,2,3,3,3-heptafluoropropan-2-yl)benzoate	51114-06-8	COC(=O)c1ccc(cc1)C(F)(C(F)(F)F)C(F)(F)F		-6.03	0.63	2.16	P	OUT
QL01802	Methyl 4-(pentadecafluoroheptyl)cyclohexane-1-carboxylate	88951-17-1	COC(=O)C1CCC(CC1)C(F)(F)C(F)(F)C(F)(F)C(F)(F)C(F)(F)C(F)(F)C(F)(F)F		-8.31	0.66	2.91	P	IN
QL01803	Methyl 4-(tridecafluorohexyl)cyclohexane-1-carboxylate	88951-16-0	COC(=O)C1CCC(CC1)C(F)(F)C(F)(F)C(F)(F)C(F)(F)C(F)(F)C(F)(F)F		-7.89	0.65	2.76	P	IN
QL01804	Methyl 4-(undecafluoropentyl)cyclohexane-1-carboxylate	88951-15-9	COC(=O)C1CCC(CC1)C(F)(F)C(F)(F)C(F)(F)C(F)(F)C(F)(F)F		-7.62	0.65	2.70	P	IN
QL01805	Methyl 4-(nonafluorobutyl)cyclohexane-1-carboxylate	88951-14-8	COC(=O)C1CCC(CC1)C(F)(F)C(F)(F)C(F)(F)C(F)(F)F		-7.15	0.64	2.56	P	IN
QL01806	Methyl 2,4-dichloro-3-[(1,1,2,2,3,3,4,4,4-nonafluorobutane-1-sulfonyl)oxy]benzoate	1451750-40-5	COC(=O)c1ccc(Cl)c(OS(=O)(=O)C(F)(F)C(F)(F)C(F)(F)C(F)(F)F)c1Cl		-7.47	0.65	2.63	P	IN
QL01807	Methyl 4-{[1,1,1,4,5,5,5-heptafluoro-3-(1,1,1,2,3,3,3-heptafluoropropan-2-yl)-4-(trifluoromethyl)pent-2-en-2-yl]oxy}benzoate	66432-83-5	COC(=O)c1ccc(OC(=C(C(F)(C(F)(F)F)C(F)(F)F)C(F)(C(F)(F)F)C(F)(F)F)C(F)(F)F)cc1		-7.08	0.64	2.46	P	IN
QL01808	Methyl 4,4,5,5,6,6,7,7,7-nonafluoro-3-oxoheptanoate	89129-70-4	COC(=O)CC(=O)C(F)(F)C(F)(F)C(F)(F)C(F)(F)F		-6.74	0.65	2.26	P	IN
QL01809	Methyl 4,4,5,5,6,6,6-heptafluoro-3-oxohexanoate	89129-68-0	COC(=O)CC(=O)C(F)(F)C(F)(F)C(F)(F)F		-6.32	0.64	2.18	P	IN
QL01810	Methyl 4,6,6-trichloro-7,7,7-trifluoro-3,3-dimethylheptanoate	72714-62-6	COC(=O)CC(C)(C)C(Cl)CC(Cl)(Cl)C(F)(F)F		-6.22	0.63	2.21	P	OUT
QL01811	Methyl 4,4,5,5,6,6,6-heptafluoro-3,3-dihydroxyhexanoate	89148-14-1	COC(=O)CC(O)(O)C(F)(F)C(F)(F)C(F)(F)F		-6.67	0.65	2.19	P	IN
QL01812	Methyl 5,5,6,6,7,7,8,8,9,9,9-undecafluoro-4-oxononanoate	1456734-51-2	COC(=O)CCC(=O)C(F)(F)C(F)(F)C(F)(F)C(F)(F)C(F)(F)F		-7.33	0.65	2.53	P	IN
QL01813	Methyl 4,4,5,5,6,6,7,7,8,8,9,9,10,10,11,11,11-heptadecafluoroundecanoate	132424-37-4	COC(=O)CCC(F)(F)C(F)(F)C(F)(F)C(F)(F)C(F)(F)C(F)(F)C(F)(F)C(F)(F)F		-8.21	0.66	2.74	P	IN
QL01814	Methyl 3-(perfluorohexyl)propanoate	110260-75-8	COC(=O)CCC(F)(F)C(F)(F)C(F)(F)C(F)(F)C(F)(F)C(F)(F)F		-7.55	0.66	2.52	P	IN
QL01815	Methyl 2H,2H,3H,3H-perfluoroheptanoate	132424-36-3	COC(=O)CCC(F)(F)C(F)(F)C(F)(F)C(F)(F)F		-6.68	0.64	2.27	P	IN
QL01816	Methyl 4,4,5,5,6,6,6-heptafluorohexanoate	2070-60-2	COC(=O)CCC(F)(F)C(F)(F)C(F)(F)F		-6.15	0.63	2.15	P	OUT
QL01817	Methyl N-(2,2,3,3,4,4,4-heptafluorobutanoyl)glycinate	81084-29-9	COC(=O)CNC(=O)C(F)(F)C(F)(F)C(F)(F)F		-6.31	0.63	2.26	P	OUT

QL01818	Dimethyl 2-(perfluoropropyl)-p-benzenedicarbamate	25642-22-2	COC(=O)NC1=CC(=C(NC(=O)OC)C=C1)C(F)(F)C(F)(F)C(F)(F)F		-6.25	0.61	2.48	P	OUT
QL01819	2,2,3,3,4,4,4-Heptafluorobutyl methyl carbonate	277332-95-3	COC(=O)OCC(F)(F)C(F)(F)C(F)(F)F		-6.15	0.63	2.15	P	OUT
QL01820	1,1,1,2,2,3,3,4,4,5,5,6,6-Tridecafluoro-8,8-dimethoxyoctane	142502-76-9	COC(CC(F)(F)C(F)(F)C(F)(F)C(F)(F)C(F)(F)C(F)(F)F)OC		-7.29	0.65	2.45	P	IN
QL01821	3-Methoxyperfluoro(2-methylpentane)	132182-92-4	COC(F)(C(F)(F)C(F)(F)F)C(F)(C(F)(F)F)C(F)(F)F		-6.35	0.67	1.72	P	IN
QL01822	Heptafluoroisopropyl methyl ether	22052-84-2	COC(F)(C(F)(F)F)C(F)(F)F		-5.05	0.65	1.28	P	IN
QL01823	1,1,3,3,4,4,5,5,6,6,6-Undecafluoro-1-methoxyhexan-2-one	106693-00-9	COC(F)(F)C(=O)C(F)(F)C(F)(F)C(F)(F)C(F)(F)F		-7.09	0.66	2.17	P	IN
QL01824	1,1,1,3,3-Pentafluoro-3-methoxy-2-(trifluoromethyl)propane	382-26-3	COC(F)(F)C(C(F)(F)F)C(F)(F)F		-4.94	0.64	1.43	P	IN
QL01825	1,1,1,2,2,3,3,4,4,5,5,6,6-Tridecafluoro-6-methoxyhexane	226909-72-4	COC(F)(F)C(F)(F)C(F)(F)C(F)(F)C(F)(F)C(F)(F)F		-7.35	0.68	2.15	P	IN
QL01826	1,1,1,2,2,3,3,4,4,5,5-Undecafluoro-5-methoxypentane	181214-74-4	COC(F)(F)C(F)(F)C(F)(F)C(F)(F)C(F)(F)F		-6.72	0.67	1.89	P	IN
QL01827	Perfluorobutyl methyl ether	163702-07-6	COC(F)(F)C(F)(F)C(F)(F)C(F)(F)F		-6.40	0.67	1.81	P	IN
QL01828	Heptafluoro-1-methoxypropane	375-03-1	COC(F)(F)C(F)(F)C(F)(F)F		-5.48	0.66	1.46	P	IN
QL01829	(1E)-1,2,3,3,4,4,5,5,6,6,6-Undecafluoro-1-methoxyhex-1-ene	62949-64-8	COC(F)=C(/F)C(F)(F)C(F)(F)C(F)(F)C(F)(F)F		-6.78	0.65	2.18	P	IN
QL01830	2,2,3,3,4,4,4-Heptafluoro-1-methoxybutan-1-ol	377-52-6	COC(O)C(F)(F)C(F)(F)C(F)(F)F		-5.98	0.65	1.84	P	IN
QL01831	2-Methoxyperfluoro(2,5-di(propan-2-yl)oxolane)	957209-18-6	COC1(OC(F)(C(F)(C(F)(F)F)C(F)(F)F)C(F)(F)C1(F)F)C(F)(C(F)(F)F)C(F)(F)F		-7.21	0.68	2.04	P	IN
QL01832	2-(Heptafluoropropyl)-1-methoxy-4-methylbenzene	87461-66-3	COC1=C(C=C(C)C=C1)C(F)(F)C(F)(F)C(F)(F)F		-5.66	0.63	1.90	P	IN
QL01833	1-Chloro-3,3,4,4,5,5-hexafluoro-2-methoxycyclopentene	336-34-5	COC1=C(Cl)C(F)(F)C(F)(F)C1(F)F		-5.57	0.65	1.53	P	IN
QL01834	N-[2-(3,4-Dimethoxyphenyl)ethyl]-2,2,3,3,4,4,4-heptafluorobutanamide	13230-87-0	COC1=C(OC)C=C(CCNC(=O)C(F)(F)C(F)(F)C(F)(F)F)C=C1		-6.71	0.61	2.72	P	OUT
QL01835	(1Z)-2,2,3,3,4,4,4-Heptafluoro-N-(4-methoxy-2-methylphenyl)butanimidoyl chloride	923569-77-1	COC1=CC(C)=C(C=C1)N=C(/Cl)C(F)(F)C(F)(F)C(F)(F)F		-6.83	0.63	2.53	P	OUT
QL01836	(1Z)-N-[2-(Bromomethyl)-4-methoxyphenyl]-2,2,3,3,4,4,4-heptafluorobutanimidoyl chloride	923569-78-2	COC1=CC(CBr)=C(C=C1)N=C(/Cl)C(F)(F)C(F)(F)C(F)(F)F		-6.54	0.62	2.52	P	OUT
QL01837	2,5-Dimethoxyphenyl heptafluorobutanoate	87461-68-5	COC1=CC(OC(=O)C(F)(F)C(F)(F)C(F)(F)F)=C(OC)C=C1		-6.28	0.62	2.39	P	OUT
QL01838	1,1-Di-(4-methoxyphenyl)-1-[4-(1H,1H,2H,2H-perfluorodecyl)phenyl]methyl chloride	865758-36-7	COC1=CC=C(C=C1)C(Cl)(C1=CC=C(CCC(F)(F)C(F)(F)C(F)(F)C(F)(F)C(F)(F)C(F)(F)C(F)(F)C(F)(F)F)C=C1)C1=CC=C(OC)C=C1		-8.68	0.64	3.38	P	OUT
QL01839	1-(Chloro[4-(2-(perfluorooctyl)ethyl)phenyl]phenylmethyl)-4-methoxybenzene	865758-37-8	COC1=CC=C(C=C1)C(Cl)(C1=CC=CC=C1)C1=CC=C(CCC(F)(F)C(F)(F)C(F)(F)C(F)(F)C(F)(F)C(F)(F)C(F)(F)C(F)(F)F)C=C1		-8.71	0.64	3.37	P	OUT
QL01840	[4-(3,3,4,4,5,5,6,6,7,7,8,8,9,9,10,10,10-Heptadecafluorodecyl)phenyl]bis(4-methoxyphenyl)methanol	865758-47-0	COC1=CC=C(C=C1)C(O)(C1=CC=C(CCC(F)(F)C(F)(F)C(F)(F)C(F)(F)C(F)(F)C(F)(F)C(F)(F)C(F)(F)F)C=C1)C1=CC=C(OC)C=C1		-8.68	0.64	3.38	P	OUT
QL01841	Thymidine, 5'-O-[[4-(1H,1H,2H,2H-perfluorodecyl)phenyl]bis(4-methoxyphenyl)methyl]-, 3'-[2-cyanoethyl N,N-bis(1-methylethyl)phosphoramidite]	902456-09-1	COC1=CC=C(C=C1)C(OC[C@H]1O[C@H](C[C@@H]1OP(OCCC#N)N(C(C)C)C(C)C)N1C=C(C)C(=O)NC1=O)(C1=CC=C(CCC(F)(F)C(F)(F)C(F)(F)C(F)(F)C(F)(F)C(F)(F)C(F)(F)C(F)(F)F)C=C1)C1=CC=C(OC)C=C1		-8.71	0.62	3.67	P	OUT
QL01842	Cytidine, N-benzoyl-2'-deoxy-5'-O-[[4-(1H,1H,2H,2H-perfluorodecyl)phenyl]bis(4-methoxyphenyl)methyl]-, 3'-[2-cyanoethyl N,N-bis(1-methylethyl)phosphoramidite]	1401422-19-2	COC1=CC=C(C=C1)C(OC[C@H]1O[C@H](C[C@@H]1OP(OCCC#N)N(C(C)C)C(C)C)N1C=CC(NC(=O)C2=CC=CC=C2)=NC1=O)(C1=CC=C(CCC(F)(F)C(F)(F)C(F)(F)C(F)(F)C(F)(F)C(F)(F)C(F)(F)C(F)(F)F)C=C1)C1=CC=C(OC)C=C1		-9.00	0.61	3.91	P	OUT
QL01843	Guanosine, 2'-deoxy-5'-O-[[4-(1H,1H,2H,2H-perfluorodecyl)phenyl]bis(4-methoxyphenyl)methyl]-N-(2-methyl-1-oxopropyl)-, 3'-[2-cyanoethyl N,N-bis(1-methylethyl)phosphoramidite]	1401422-21-6	COC1=CC=C(C=C1)C(OC[C@H]1OC(C[C@@H]1OP(OCCC#N)N(C(C)C)C(C)C)N1C=NC2=C1N=C(NC(=O)C(C)C)NC2=O)(C1=CC=C(CCC(F)(F)C(F)(F)C(F)(F)C(F)(F)C(F)(F)C(F)(F)C(F)(F)C(F)(F)F)C=C1)C1=CC=C(OC)C=C1		-8.91	0.62	3.80	P	OUT
QL01844	Guanosine, 2'-deoxy-5'-O-[[4-(1H,1H,2H,2H-perfluorodecyl)phenyl](4-methoxyphenyl)phenylmethyl]-N-(2-methyl-1-oxopropyl)-, 3'-[2-cyanoethyl N,N-bis(1-methylethyl)phosphoramidite]	1401422-22-7	COC1=CC=C(C=C1)C(OC[C@H]1OC(C[C@@H]1OP(OCCC#N)N(C(C)C)C(C)C)N1C=NC2=C1N=C(NC(=O)C(C)C)NC2=O)(C1=CC=CC=C1)C1=CC=C(CCC(F)(F)C(F)(F)C(F)(F)C(F)(F)C(F)(F)C(F)(F)C(F)(F)C(F)(F)F)C=C1		-8.92	0.62	3.80	P	OUT
QL01845	Butanoic acid, heptafluoro-, (4-methoxyphenyl)methyl ester	87461-67-4	COC1=CC=C(COC(=O)C(F)(F)C(F)(F)C(F)(F)F)C=C1		-6.93	0.62	2.70	P	OUT
QL01846	2-(Heptafluoropropyl)-5-methoxy-1H-indole	923569-79-3	COC1=CC2=C(NC(=C2)C(F)(F)C(F)(F)C(F)(F)F)C=C1		-6.82	0.63	2.47	P	OUT
QL01847	4,9-Dimethoxy-7-(nonafluorobutyl)-5H-furo[3,2-g][1]benzopyran-5-one	376380-66-4	COc1c2C(=O)C=C(Oc2c(OC)c2occc12)C(F)(F)C(F)(F)C(F)(F)C(F)(F)F		-6.83	0.63	2.51	P	OUT
QL01848	7-(Heptafluoropropyl)-4,9-dimethoxy-5H-furo[3,2-g][1]benzopyran-5-one	376383-49-2	COc1c2C(=O)C=C(Oc2c(OC)c2occc12)C(F)(F)C(F)(F)C(F)(F)F		-6.45	0.62	2.43	P	OUT

QL01849	7-Hydroxy-4,9-dimethoxy-7-(nonafluorobutyl)-6,7-dihydro-5H-furo[3,2-g][1]benzopyran-5-one	376382-61-5	COc1c2C(=O)CC(O)(Oc2c(OC)c2occc12)C(F)(F)C(F)(F)C(F)(F)C(F)(F)F		-7.03	0.64	2.53	P	OUT
QL01850	7-(Heptafluoropropyl)-7-hydroxy-4,9-dimethoxy-6,7-dihydro-5H-furo[3,2-g][1]benzopyran-5-one	376380-76-6	COc1c2C(=O)CC(O)(Oc2c(OC)c2occc12)C(F)(F)C(F)(F)C(F)(F)F		-6.66	0.63	2.43	P	OUT
QL01851	4-[(4,4,5,5,6,6,7,7,8,8,9,9,10,10,11,11,11-Heptadecafluoroundecyl)oxy]-5-methoxy-2-nitrobenzaldehyde	853928-88-8	COc1cc(C=O)c(cc1OCCCC(F)(F)C(F)(F)C(F)(F)C(F)(F)C(F)(F)C(F)(F)C(F)(F)C(F)(F)F)[N+]([O-])=O		-8.47	0.64	3.20	P	OUT
QL01852	4-(1,1,1,2,3,3,3-Heptafluoropropan-2-yl)-2-methoxyaniline	273735-38-9	COc1cc(ccc1N)C(F)(C(F)(F)F)C(F)(F)F		-5.55	0.63	1.85	P	IN
QL01853	{4-[(4,4,5,5,6,6,7,7,8,8,9,9,10,10,11,11,11-Heptadecafluoroundecyl)oxy]-5-methoxy-2-nitrophenyl}methanol	853929-03-0	COc1cc(CO)c(cc1OCCCC(F)(F)C(F)(F)C(F)(F)C(F)(F)C(F)(F)C(F)(F)C(F)(F)C(F)(F)F)[N+]([O-])=O		-8.47	0.64	3.20	P	OUT
QL01854	2-(Heptafluoropropyl)-1,3,5-trimethoxybenzene	1422444-58-3	COc1cc(OC)c(c(OC)c1)C(F)(F)C(F)(F)C(F)(F)F		-5.92	0.61	2.28	P	OUT
QL01855	N'-(6,7-Dimethoxy-3,3-dimethyl-3,4-dihydroisoquinolin-1-yl)-2,2,3,3,4,4,4-heptafluorobutanehydrazide	304435-61-8	COc1cc2CC(C)(C)N=C(NNC(=O)C(F)(F)C(F)(F)C(F)(F)F)c2cc1OC		-7.10	0.63	2.72	P	OUT
QL01856	4-Bromo-1-methoxy-2-(nonafluorobutyl)benzene	610778-27-3	COc1ccc(Br)cc1C(F)(F)C(F)(F)C(F)(F)C(F)(F)F		-6.42	0.64	2.16	P	IN
QL01857	4,4,5,5,6,6,6-Heptafluoro-1-(4-methoxyphenyl)hexane-1,3-dione	515163-04-9	COc1ccc(cc1)C(=O)CC(=O)C(F)(F)C(F)(F)C(F)(F)F		-7.08	0.63	2.72	P	OUT
QL01858	1-Methoxy-4-(nonafluorobutyl)benzene	153910-98-6	COc1ccc(cc1)C(F)(F)C(F)(F)C(F)(F)C(F)(F)F		-7.03	0.64	2.47	P	IN
QL01859	1-(Heptafluoropropyl)-4-methoxybenzene	97745-82-9	COc1ccc(cc1)C(F)(F)C(F)(F)C(F)(F)F		-6.44	0.63	2.31	P	OUT
QL01860	4-(4-Methoxyphenyl)-2-methyl-5-(1,1,2,2,3,3,4,4,5,5,6,6,6-tridecafluorohexane-1-sulfonyl)-1,2-oxazolidine	93416-31-0	COc1ccc(cc1)C1CN(C)OC1S(=O)(=O)C(F)(F)C(F)(F)C(F)(F)C(F)(F)C(F)(F)C(F)(F)F		-8.19	0.65	2.94	P	IN
QL01861	2,2,3,3,4,4,5,5,6,6,7,7,8,8,8-Pentadecafluoro-N-(4-methoxyphenyl)octanamide	103188-04-1	COc1ccc(NC(=O)C(F)(F)C(F)(F)C(F)(F)C(F)(F)C(F)(F)C(F)(F)C(F)(F)F)cc1		-8.31	0.65	2.96	P	IN
QL01862	2,2,3,3,4,4,4-Heptafluoro-N-(4-methoxy-2-nitrophenyl)butanamide	1799-52-6	COc1ccc(NC(=O)C(F)(F)C(F)(F)C(F)(F)F)c(c1)[N+]([O-])=O		-6.70	0.63	2.51	P	OUT
QL01863	2,2,3,3,4,4,4-Heptafluoro-N-(4-methoxyphenyl)butanamide	793-36-2	COc1ccc(NC(=O)C(F)(F)C(F)(F)C(F)(F)F)cc1		-6.76	0.63	2.55	P	OUT
QL01864	2-(Heptafluoropropyl)-1,4-dimethoxybenzene	80790-98-3	COc1ccc(OC)c(c1)C(F)(F)C(F)(F)C(F)(F)F		-5.84	0.62	2.13	P	OUT
QL01865	2-Chloro-4-methoxyphenyl nonafluorobutane-1-sulfonate	1131890-97-5	COc1ccc(OS(=O)(=O)C(F)(F)C(F)(F)C(F)(F)C(F)(F)F)c(Cl)c1		-7.53	0.65	2.66	P	IN
QL01866	6-Methoxyquinolin-4-yl nonafluorobutane-1-sulfonate	1161941-02-1	COc1ccc2nccc(OS(=O)(=O)C(F)(F)C(F)(F)C(F)(F)C(F)(F)F)c2c1		-7.31	0.64	2.64	P	OUT
QL01867	N'-[(2E)-5,5,6,6,7,7,7-Heptafluoro-4-oxohept-2-en-2-yl]-3-methoxybenzohydrazide	1164461-08-8	COc1cccc(c1)C(=O)NNC(C)=CC(=O)C(F)(F)C(F)(F)C(F)(F)F		-7.11	0.62	2.88	P	OUT
QL01868	1-Methoxy-3-(nonafluorobutyl)benzene	18739-65-6	COc1cccc(c1)C(F)(F)C(F)(F)C(F)(F)C(F)(F)F		-6.57	0.64	2.26	P	IN
QL01869	2,2,3,3,4,4,5,5,6,6,7,7,7-Tridecafluoro-N-(3-methoxyphenyl)heptanamide	904565-42-0	COc1cccc(NC(=O)C(F)(F)C(F)(F)C(F)(F)C(F)(F)C(F)(F)C(F)(F)F)c1		-7.75	0.65	2.76	P	IN
QL01870	2,2,3,3,4,4,4-Heptafluoro-N-(3-methoxyphenyl)butanamide	550302-16-4	COc1cccc(NC(=O)C(F)(F)C(F)(F)C(F)(F)F)c1		-6.48	0.62	2.43	P	OUT
QL01871	1-Methoxy-2-(nonafluorobutyl)benzene	153910-97-5	COc1ccccc1C(F)(F)C(F)(F)C(F)(F)C(F)(F)F		-6.32	0.64	2.17	P	IN
QL01872	2,3,5-Trifluoro-4-(1,1,1,2,3,3,3-heptafluoropropan-2-yl)-6-methoxypyridine	20017-48-5	COc1nc(F)c(F)c(c1F)C(F)(C(F)(F)F)C(F)(F)F		-5.72	0.64	1.87	P	IN
QL01873	5-(Heptafluoropropyl)-3-methoxy-1-methyl-1H-1,2,4-triazole	714975-34-5	COc1nc(n(C)n1)C(F)(F)C(F)(F)C(F)(F)F		-6.35	0.64	2.16	P	IN
QL01874	2,4-Dimethoxy-5-(nonafluorobutyl)pyrimidine	71623-69-3	COc1ncc(c(OC)n1)C(F)(F)C(F)(F)C(F)(F)C(F)(F)F		-6.73	0.63	2.44	P	OUT
QL01875	Methyl 2H,2H-perfluorobutyl ether	376-98-7	COCC(F)(F)C(F)(F)C(F)(F)F		-5.71	0.64	1.85	P	IN
QL01876	3,4,4,5,5,6,6,6-Octafluoro-1-methoxyhex-2-ene	90292-38-9	COCC=C(F)C(F)(F)C(F)(F)C(F)(F)F		-6.07	0.63	2.20	P	OUT
QL01877	1,1,2,2,3,3,4,4,5,5,6,6,6-Tridecafluoro-N,N-bis(2-methoxyethyl)hexane-1-sulfonamide	1427176-20-2	COCCN(CCOC)S(=O)(=O)C(F)(F)C(F)(F)C(F)(F)C(F)(F)C(F)(F)C(F)(F)F		-7.34	0.64	2.70	P	OUT
QL01878	1,1,2,2,3,3,4,4,4-Nonafluoro-N,N-bis(2-methoxyethyl)butane-1-sulfonamide	1427176-19-9	COCCN(CCOC)S(=O)(=O)C(F)(F)C(F)(F)C(F)(F)C(F)(F)F		-6.47	0.62	2.47	P	OUT
QL01879	2,2,3,3,4,4,4-Heptafluoro-N-(2-methoxyethyl)butanamide	70473-74-4	COCCNC(=O)C(F)(F)C(F)(F)C(F)(F)F		-6.02	0.62	2.28	P	OUT
QL01880	2-Methoxyethyl 2,3,3,3-tetrafluoro-2-(heptafluoropropoxy)propanoate	340034-67-5	COCCOC(=O)C(F)(OC(F)(F)C(F)(F)C(F)(F)F)C(F)(F)F		-6.97	0.63	2.53	P	OUT
QL01881	Heptaethylene glycol perfluoro-2-isopropyl-1,3-dimethylbut-1-enyl methyl ether	88987-13-7	COCCOCCOCCOCCOCCOCCOCCOC(=C(C(F)(C(F)(F)F)C(F)(F)F)C(F)(C(F)(F)F)C(F)(F)F)C(F)(F)F		-7.49	0.59	3.41	P	OUT
QL01882	32-(Perfluorodecyl)-2,5,8,11,14,17,20,23,26,29-decaoxadotriacontan-31-ol	93776-07-9	COCCOCCOCCOCCOCCOCCOCCOCCOCCOCC(CC(C(C(C(C(C(C(C(C(C(F)(F)F)(F)F)(F)F)(F)F)(F)F)(F)F)(F)F)(F)F)(F)F)(F)F)O		-9.51	0.60	4.36	P	OUT
QL01883	32-(Perfluoro-7-methyloctyl)-2,5,8,11,14,17,20,23,26,29-decaoxadotriacontan-31-ol	93776-11-5	COCCOCCOCCOCCOCCOCCOCCOCCOCCOCC(O)CC(F)(F)C(F)(F)C(F)(F)C(F)(F)C(F)(F)C(F)(F)C(C(F)(F)F)(F)C(F)(F)F		-9.12	0.59	4.25	P	OUT

QL01884	32-(Perfluoro-11-methyldodecyl)-2,5,8,11,14,17,20,23,26,29-decaoxadotriacontan-31-ol	93776-09-1	COCCOCCOCCOCCOCCOCCOCCOCCOCCOCC(O)CC(F)(F)C(F)(F)C(F)(F)C(F)(F)C(F)(F)C(F)(F)C(F)(F)C(F)(F)C(F)(F)C(F)(F)C(C(F)(F)F)(F)C(F)(F)F		-9.91	0.61	4.40	P	OUT
QL01885	33,33,34,34,35,35,36,36,37,37,38,38,39,39,40,40,41,41,42,42,43,43,44,44,44-pentacosafluoro-2,5,8,11,14,17,20,23,26-decaoxatetratetracontan-31-ol (English, Spanish) (EINECS)	93776-06-8	COCCOCCOCCOCCOCCOCCOCCOCCOCCOCC(O)CC(F)(F)C(F)(F)C(F)(F)C(F)(F)C(F)(F)C(F)(F)C(F)(F)C(F)(F)C(F)(F)C(F)(F)C(F)(F)C(F)(F)F		-9.89	0.61	4.43	P	OUT
QL01886	32-(Perfluorooctyl)-2,5,8,11,14,17,20,23,26,29-decaoxadotriacontan-31-ol	93776-08-0	COCCOCCOCCOCCOCCOCCOCCOCCOCCOCC(O)CC(F)(F)C(F)(F)C(F)(F)C(F)(F)C(F)(F)C(F)(F)C(F)(F)C(F)(F)F		-9.08	0.59	4.29	P	OUT
QL01887	Dimethyl (3,3,4,4,5,5,6,6,7,7,8,8,9,9,10,10,11,11,12,12,12-henicosafluorododecyl)phosphonate	61726-45-2	COP(=O)(CCC(F)(F)C(F)(F)C(F)(F)C(F)(F)C(F)(F)C(F)(F)C(F)(F)C(F)(F)C(F)(F)C(F)(F)F)OC		-8.78	0.67	2.94	P	IN
QL01888	Dimethyl (3,3,4,4,5,5,6,6,7,7,8,8,9,9,10,10,10-heptadecafluorodecyl)phosphonate	61726-43-0	COP(=O)(CCC(F)(F)C(F)(F)C(F)(F)C(F)(F)C(F)(F)C(F)(F)C(F)(F)C(F)(F)F)OC		-8.25	0.67	2.74	P	IN
QL01889	Dimethyl (3,3,4,4,5,5,6,6,7,7,8,8,8-tridecafluorooctyl)phosphonatato	61726-44-1	COP(=O)(CCC(F)(F)C(F)(F)C(F)(F)C(F)(F)C(F)(F)C(F)(F)F)OC		-7.60	0.66	2.51	P	IN
QL01890	1-Cyano-2,2,3,3,4,4,4-heptafluorobutyl dimethyl phosphate	56376-19-3	COP(=O)(OC)OC(C#N)C(F)(F)C(F)(F)C(F)(F)F		-6.03	0.63	2.15	P	OUT
QL01891	Methyl nonaflate	6401-03-2	COS(=O)(=O)C(F)(F)C(F)(F)C(F)(F)C(F)(F)F		-6.72	0.67	1.89	P	IN
QL01892	1,1,1,2,2,3,3,4,4-Nonafluoro-4-(methanesulfonyl)butane	40630-22-6	CS(=O)(=O)C(F)(F)C(F)(F)C(F)(F)C(F)(F)F		-6.93	0.69	1.79	P	IN
QL01893	2,2,3,3,4,4,5,5,6,6,6-Undecafluorohexyl methanesulfonate	188534-35-2	CS(=O)(=O)OCC(F)(F)C(F)(F)C(F)(F)C(F)(F)C(F)(F)F		-7.39	0.67	2.23	P	IN
QL01894	2,2,3,3,4,4,4-Heptafluorobutyl methanesulfonate	755-88-4	CS(=O)(=O)OCC(F)(F)C(F)(F)C(F)(F)F		-6.45	0.66	1.91	P	IN
QL01895	3,3,4,4,5,5,6,6,7,7,8,8,8-Tridecafluorooctyl methanesulfonate	156575-40-5	CS(=O)(=O)OCCC(F)(F)C(F)(F)C(F)(F)C(F)(F)C(F)(F)C(F)(F)F		-7.84	0.67	2.51	P	IN
QL01896	1,1,1,2,2,3,3,4,4-Nonafluoro-6-(methanesulfinyl)hexane	357604-65-0	CS(=O)CCC(F)(F)C(F)(F)C(F)(F)C(F)(F)F		-6.77	0.65	2.19	P	IN
QL01897	4-(1,1,1,2,3,3,3-Heptafluoropropan-2-yl)-2-methyl-3-(methylsulfanyl)benzoic acid	1355553-97-7	CSc1c(C)c(ccc1C(F)(C(F)(F)F)C(F)(F)F)C(O)=O		-6.17	0.63	2.19	P	OUT
QL01898	Phenyl(tridecafluorohexyl)iodanium tetrafluoridoborate(1-)	74061-32-8	F[B-](F)(F)F.FC(F)(F)C(F)(F)C(F)(F)C(F)(F)C(F)(F)C(F)(F)[I+]c1ccccc1		-7.91	0.64	2.87	P	IN
QL01899	Dichloro[fluoro(heptadecafluorooctyl)-lambda~3~-chloranyl](pentafluorophenyl)silane	753025-21-7	F[Cl](C(F)(F)C(F)(F)C(F)(F)C(F)(F)C(F)(F)C(F)(F)C(F)(F)C(F)(F)F)[Si](Cl)(Cl)C1=C(F)C(F)=C(F)C(F)=C1F		-8.79	0.67	2.93	P	OUT
QL01900	1-(Difluoroiodo)perfluoro-1-octyne	1424379-19-0	F[I](F)C#CC(F)(F)C(F)(F)C(F)(F)C(F)(F)C(F)(F)C(F)(F)F		-7.62	0.66	2.45	P	IN
QL01901	3-Methyl-1-((perfluorohexyl)ethyl)-1H-imidazol-3-ium hexafluorophosphate	313475-50-2	F[P-](F)(F)(F)(F)F.C[N+]1=CN(CCC(F)(F)C(F)(F)C(F)(F)C(F)(F)C(F)(F)C(F)(F)F)C=C1		-8.88	0.67	3.01	P	OUT
QL01902	3-Butyl-1-((perfluorohexyl)eth-2-yl)-1H-imidazol-3-ium hexafluorophosphate	313475-52-4	F[P-](F)(F)(F)(F)F.CCCC[N+]1=CN(CCC(F)(F)C(F)(F)C(F)(F)C(F)(F)C(F)(F)C(F)(F)F)C=C1		-8.75	0.65	3.19	P	IN
QL01903	1-Methyl-3-octylimidazolium hexafluorophosphate	304680-36-2	F[P-](F)(F)(F)(F)F.CCCCCCCCN1C=C[N+](C)=C1		-6.28	0.59	2.82	P	OUT
QL01906	double bond-perfluoroalkyl (linear) carboxylic acids_n_1, n=10		F/C((C(F)(F)C(F)(F)C(F)(F)C(F)(F)C(F)(F)C(F)(F)C(F)(F)C(F)(F)C(F)(F)C(F)(F)C(O)=O))=C(C(F)(F)F)/F		-9.05	0.68	2.95	P	OUT
QL01907	double bond-perfluoroalkyl (linear) carboxylic acids_n_1, n=9		F/C((C(F)(F)C(F)(F)C(F)(F)C(F)(F)C(F)(F)C(F)(F)C(F)(F)C(F)(F)C(F)(F)C(O)=O))=C(C(F)(F)F)/F		-8.91	0.68	2.90	P	OUT
QL01908	double bond-perfluoroalkyl (linear) carboxylic acids_n_1, n=8		F/C((C(F)(F)C(F)(F)C(F)(F)C(F)(F)C(F)(F)C(F)(F)C(F)(F)C(F)(F)C(O)=O))=C(C(F)(F)F)/F		-8.57	0.68	2.75	P	IN
QL01909	double bond-perfluoroalkyl (linear) carboxylic acids_n_1, n=7		F/C((C(F)(F)C(F)(F)C(F)(F)C(F)(F)C(F)(F)C(F)(F)C(F)(F)C(O)=O))=C(C(F)(F)F)/F		-8.41	0.68	2.69	P	IN
QL01910	double bond-perfluoroalkyl (linear) carboxylic acids_n_1, n=6		F/C((C(F)(F)C(F)(F)C(F)(F)C(F)(F)C(F)(F)C(F)(F)C(O)=O))=C(C(F)(F)F)/F		-8.00	0.67	2.52	P	IN
QL01911	double bond-perfluoroalkyl (linear) carboxylic acids_5_n, n=6		F/C(C(F)(C(F)(F)C(F)(F)C(F)(F)C(F)(F)C(O)=O)F)=C(C(F)(F)C(F)(F)C(F)(F)C(F)(F)C(F)(F)C(F)(F)F)/F		-9.07	0.68	2.94	P	OUT
QL01912	double bond-perfluoroalkyl (linear) carboxylic acids_5_n, n=5		F/C(C(F)(C(F)(F)C(F)(F)C(F)(F)C(F)(F)C(O)=O)F)=C(C(F)(F)C(F)(F)C(F)(F)C(F)(F)C(F)(F)F)/F		-8.92	0.68	2.88	P	OUT
QL01913	double bond-perfluoroalkyl (linear) carboxylic acids_5_n, n=4		F/C(C(F)(C(F)(F)C(F)(F)C(F)(F)C(F)(F)C(O)=O)F)=C(C(F)(F)C(F)(F)C(F)(F)C(F)(F)F)/F		-8.61	0.68	2.75	P	IN
QL01914	double bond-perfluoroalkyl (linear) carboxylic acids_5_n, n=3		F/C(C(F)(C(F)(F)C(F)(F)C(F)(F)C(F)(F)C(O)=O)F)=C(C(F)(F)C(F)(F)C(F)(F)F)/F		-8.45	0.68	2.69	P	IN
QL01915	double bond-perfluoroalkyl (linear) carboxylic acids_5_n, n=2		F/C(C(F)(C(F)(F)C(F)(F)C(F)(F)C(F)(F)C(O)=O)F)=C(C(F)(F)C(F)(F)F)/F		-8.05	0.67	2.52	P	IN
QL01916	double bond-perfluoroalkyl (linear) carboxylic acids_5_n, n=1		F/C(C(F)(C(F)(F)C(F)(F)C(F)(F)C(F)(F)C(O)=O)F)=C(C(F)(F)F)/F		-7.79	0.67	2.46	P	IN

QL01917	double bond-, H-, ether-substituted perfluoroalkyl (linear) carboyxlic acids, n=10		F/C(C(F)(F)C(F)(F)C(F)(F)C(F)(F)C(F)(F)C(F)(F)C(F)(F)C(F)(F)C(F)(F)C(F)(F)OCC(O)=O)=CC(F)(F)F		-8.96	0.67	3.06	P	OUT
QL01918	double bond perfluoroalkyl (linear) sulfonic aicds, n=10		F/C(C(F)(F)C(F)(F)C(F)(F)C(F)(F)C(F)(F)C(F)(F)C(F)(F)C(F)(F)C(F)(F)C(F)(F)S(=O)(O)=O)=C(F)C(F)(F)F		-9.19	0.69	2.95	P	OUT
QL01919	double bond-, H-, ether-substituted perfluoroalkyl (linear) carboyxlic acids, n=9		F/C(C(F)(F)C(F)(F)C(F)(F)C(F)(F)C(F)(F)C(F)(F)C(F)(F)C(F)(F)C(F)(F)OCC(O)=O)=CC(F)(F)F		-8.81	0.67	3.02	P	OUT
QL01920	double bond perfluoroalkyl (linear) sulfonic aicds, n=9		F/C(C(F)(F)C(F)(F)C(F)(F)C(F)(F)C(F)(F)C(F)(F)C(F)(F)C(F)(F)C(F)(F)S(=O)(O)=O)=C(F)C(F)(F)F		-9.06	0.68	2.89	P	OUT
QL01921	double bond-, H-, ether-substituted perfluoroalkyl (linear) carboyxlic acids, n=8		F/C(C(F)(F)C(F)(F)C(F)(F)C(F)(F)C(F)(F)C(F)(F)C(F)(F)C(F)(F)OCC(O)=O)=CC(F)(F)F		-8.47	0.66	2.88	P	IN
QL01922	double bond perfluoroalkyl (linear) sulfonic aicds, n=8		F/C(C(F)(F)C(F)(F)C(F)(F)C(F)(F)C(F)(F)C(F)(F)C(F)(F)C(F)(F)S(=O)(O)=O)=C(F)C(F)(F)F		-8.73	0.68	2.75	P	IN
QL01923	double bond-, H-, ether-substituted perfluoroalkyl (linear) carboyxlic acids, n=7		F/C(C(F)(F)C(F)(F)C(F)(F)C(F)(F)C(F)(F)C(F)(F)C(F)(F)OCC(O)=O)=CC(F)(F)F		-8.29	0.66	2.84	P	IN
QL01924	double bond perfluoroalkyl (linear) sulfonic aicds, n=7		F/C(C(F)(F)C(F)(F)C(F)(F)C(F)(F)C(F)(F)C(F)(F)C(F)(F)S(=O)(O)=O)=C(F)C(F)(F)F		-8.58	0.68	2.69	P	IN
QL01925	double bond-, H-, ether-substituted perfluoroalkyl (linear) carboyxlic acids, n=6		F/C(C(F)(F)C(F)(F)C(F)(F)C(F)(F)C(F)(F)C(F)(F)OCC(O)=O)=CC(F)(F)F		-7.87	0.66	2.68	P	IN
QL01926	double bond perfluoroalkyl (linear) sulfonic aicds, n=6		F/C(C(F)(F)C(F)(F)C(F)(F)C(F)(F)C(F)(F)C(F)(F)S(=O)(O)=O)=C(F)C(F)(F)F		-8.19	0.68	2.52	P	IN
QL01927	double bond-, H-, ether-substituted perfluoroalkyl (linear) carboyxlic acids, n=5		F/C(C(F)(F)C(F)(F)C(F)(F)C(F)(F)C(F)(F)OCC(O)=O)=CC(F)(F)F		-7.65	0.65	2.63	P	IN
QL01928	double bond perfluoroalkyl (linear) sulfonic aicds, n=5		F/C(C(F)(F)C(F)(F)C(F)(F)C(F)(F)C(F)(F)S(=O)(O)=O)=C(F)C(F)(F)F		-8.00	0.68	2.45	P	IN
QL01929	double bond-, H-, ether-substituted perfluoroalkyl (linear) carboyxlic acids, n=4		F/C(C(F)(F)C(F)(F)C(F)(F)C(F)(F)OCC(O)=O)=CC(F)(F)F		-7.12	0.65	2.44	P	IN
QL01930	double bond perfluoroalkyl (linear) sulfonic aicds, n=4		F/C(C(F)(F)C(F)(F)C(F)(F)C(F)(F)S(=O)(O)=O)=C(F)C(F)(F)F		-7.52	0.68	2.24	P	IN
QL01931	multiple double bond-perfluoroalkyl (linear) carboxylic acids, n=3		F/C(C(F)(F)C(F)(F)C(F)(F)C(O)=O)=C(F)C(F)=C(F)F		-6.72	0.65	2.26	P	IN
QL01932	double bond-, H-, ether-substituted perfluoroalkyl (linear) carboyxlic acids, n=3		F/C(C(F)(F)C(F)(F)C(F)(F)OCC(O)=O)=CC(F)(F)F		-6.82	0.64	2.40	P	IN
QL01933	double bond perfluoroalkyl (linear) sulfonic aicds, n=3		F/C(C(F)(F)C(F)(F)C(F)(F)S(=O)(O)=O)=C(F)C(F)(F)F		-7.27	0.67	2.17	P	IN
QL01934	multiple double bond-perfluoroalkyl (linear) carboxylic acids, n=2		F/C(C(F)(F)C(F)(F)C(O)=O)=C(F)C(F)=C(F)F		-6.30	0.64	2.17	P	IN
QL01935	double bond-, H-, ether-substituted perfluoroalkyl (linear) carboyxlic acids, n=2		F/C(C(F)(F)C(F)(F)OCC(O)=O)=CC(F)(F)F		-6.10	0.63	2.18	P	OUT
QL01936	double bond perfluoroalkyl (linear) sulfonic aicds, n=2		F/C(C(F)(F)C(F)(F)S(=O)(O)=O)=C(F)C(F)(F)F		-6.63	0.67	1.91	P	IN
QL01937	multiple double bond-perfluoroalkyl (linear) carboxylic acids, n=1		F/C(C(F)(F)C(O)=O)=C(F)C(F)=C(F)F		-5.46	0.62	1.93	P	OUT
QL01938	double bond-, H-, ether-substituted perfluoroalkyl (linear) carboyxlic acids, n=1		F/C(C(F)(F)OCC(O)=O)=CC(F)(F)F		-5.60	0.61	2.15	P	OUT
QL01939	double bond perfluoroalkyl (linear) sulfonic aicds, n=1		F/C(C(F)(F)S(=O)(O)=O)=C(F)C(F)(F)F		-6.21	0.66	1.78	P	IN
QL01940	Pentafluorosulfide Perfluoroalkyl (linear) carboxylic acids, n=8		FC([S](F)(F)(F)(F)F)(C(F)(F)C(F)(F)C(F)(F)C(F)(F)C(F)(F)C(F)(F)C(F)(F)C(O)=O)F		-8.77	0.70	2.51	P	IN
QL01941	Pentafluorosulfide Perfluoroalkyl (linear) carboxylic acids, n=7		FC([S](F)(F)(F)(F)F)(C(F)(F)C(F)(F)C(F)(F)C(F)(F)C(F)(F)C(F)(F)C(O)=O)F		-8.63	0.70	2.43	P	IN
QL01942	Pentafluorosulfide Perfluoroalkyl (linear) carboxylic acids, n=6		FC([S](F)(F)(F)(F)F)(C(F)(F)C(F)(F)C(F)(F)C(F)(F)C(F)(F)C(O)=O)F		-8.24	0.70	2.23	P	IN
QL01943	double bond-, H-substituted perfluoroalkyl sulfonic acids, n=6		FC(/C(F)=C(F)/C(F)(F)F)([H])C(F)(F)C(F)(F)C(F)(F)C(F)(F)C(F)(F)C(F)(F)S(=O)(O)=O		-8.43	0.68	2.69	P	IN
QL01944	double bond-, H-substituted perfluoroalkyl sulfonic acids, n=5		FC(/C(F)=C(F)/C(F)(F)F)([H])C(F)(F)C(F)(F)C(F)(F)C(F)(F)C(F)(F)S(=O)(O)=O		-8.02	0.67	2.52	P	IN
QL01945	double bond-, H-substituted perfluoroalkyl sulfonic acids, n=4		FC(/C(F)=C(F)/C(F)(F)F)([H])C(F)(F)C(F)(F)C(F)(F)C(F)(F)S(=O)(O)=O		-7.82	0.67	2.46	P	IN
QL01946	double bond-, H-substituted perfluoroalkyl sulfonic acids, n=3		FC(/C(F)=C(F)/C(F)(F)F)([H])C(F)(F)C(F)(F)C(F)(F)S(=O)(O)=O		-7.31	0.67	2.25	P	IN
QL01947	double bond-, H-substituted perfluoroalkyl sulfonic acids, n=2		FC(/C(F)=C(F)/C(F)(F)F)([H])C(F)(F)C(F)(F)S(=O)(O)=O		-6.97	0.66	2.15	P	IN
QL01948	double bond-, H-substituted perfluoroalkyl sulfonic acids, n=1		FC(/C(F)=C(F)/C(F)(F)F)([H])C(F)(F)S(=O)(O)=O		-6.31	0.66	1.90	P	IN
QL01949	(2Z)-1,1,1,2,4,4,5,5,5-Nonafluoro-3-(1,1,1,2,3,3,3-heptafluoropropan-2-yl)pent-2-ene	58621-71-9	FC(=C(/C(F)(F)C(F)(F)F)C(F)(C(F)(F)F)C(F)(F)F)C(F)(F)F		-6.15	0.67	1.59	P	IN

QL01950	(4Z)-1,1,1,2,4,5,6,7,7,7-Decafluoro-2,6-bis(trifluoromethyl)hept-4-en-3-one	134330-95-3	FC(=C(/F)C(F)(C(F)(F)F)C(F)(F)F)C(=O)C(F)(C(F)(F)F)C(F)(F)F		-6.77	0.67	1.96	P	IN
QL01951	(4Z)-1,1,1,2,2,4,5,6,7,7,7-Undecafluoro-6-(trifluoromethyl)hept-4-en-3-one	134330-92-0	FC(=C(/F)C(F)(C(F)(F)F)C(F)(F)F)C(=O)C(F)(F)C(F)(F)F		-6.92	0.67	2.05	P	IN
QL01952	2-Pentene, 1,1,1,2,3,4,5,5,5-nonafluoro-4-(trifluoromethyl)-, (Z)-	3709-70-4	FC(=C(/F)C(F)(C(F)(F)F)C(F)(F)F)C(F)(F)F		-6.09	0.66	1.68	P	IN
QL01953	Perfluoro(3-isopropyl-4-methyl-2-pentene)	30320-27-5	FC(=C(C(F)(C(F)(F)F)C(F)(F)F)C(F)(C(F)(F)F)C(F)(F)F)C(F)(F)F		-6.03	0.67	1.52	P	IN
QL01954	(2E)-1,1,1,2,4,4,5,5,5-Nonafluoro-3-(1,1,1,2,3,3,3-heptafluoropropan-2-yl)pent-2-ene	58621-72-0	FC(=C(C(F)(F)C(F)(F)F)C(F)(C(F)(F)F)C(F)(F)F)C(F)(F)F		-6.15	0.67	1.59	P	IN
QL01955	Hexafluoropropene Trimer	6792-31-0	FC(=C(C(F)(F)F)C(F)(C(F)(F)F)C(F)(F)F)C(F)(C(F)(F)F)C(F)(F)F		-6.45	0.67	1.73	P	IN
QL01956	Perfluoro(2-methylpent-2-ene)	1584-03-8	FC(=C(C(F)(F)F)C(F)(F)F)C(F)(F)C(F)(F)F		-6.06	0.66	1.68	P	IN
QL01957	5,6,6,7,7,7-Hexafluoro-4-(trifluoromethyl)-1,4-heptadiene	84100-12-9	FC(=C(CC=C)C(F)(F)F)C(F)(F)C(F)(F)F		-5.55	0.63	1.87	P	OUT
QL01958	(E)-1,1,1,2,3,4,4,4-octafluorobut-2-ene	1516-64-9	FC(=C(F)/C(F)(F)F)C(F)(F)F		-5.18	0.65	1.46	P	IN
QL01959	Perfluoro-2,2,9,9-tetramethyldeca-3,7-diene	96288-56-1	FC(=C(F)C(C(F)(F)F)(C(F)(F)F)C(F)(F)F)C(F)(F)C(F)(F)C(F)=C(F)C(C(F)(F)F)(C(F)(F)F)C(F)(F)F		-7.35	0.67	2.22	P	IN
QL01960	1,1,1,2,4,5,6,7,7,7-Decafluoro-2,6-bis(trifluoromethyl)hept-4-en-3-one	235772-91-5	FC(=C(F)C(F)(C(F)(F)F)C(F)(F)F)C(=O)C(F)(C(F)(F)F)C(F)(F)F		-6.77	0.67	1.96	P	IN
QL01963	1,1,1,2,2,4,5,6,7,7,7-Undecafluoro-6-(trifluoromethyl)hept-4-en-3-one	143582-62-1	FC(=C(F)C(F)(C(F)(F)F)C(F)(F)F)C(=O)C(F)(F)C(F)(F)F		-6.92	0.67	2.05	P	IN
QL01964	4-(Trifluoromethyl)perfluoro-2-pentene	2070-70-4	FC(=C(F)C(F)(C(F)(F)F)C(F)(F)F)C(F)(F)F		-6.09	0.66	1.68	P	IN
QL01967	1,1,2,3,4,5,5,5-Octafluoro-1-iodo-4-(trifluoromethyl)-2-pentene	96288-51-6	FC(=C(F)C(F)(C(F)(F)F)C(F)(F)F)C(F)(F)I		-6.09	0.66	1.68	P	IN
QL01968	1,1,1,2,3,4,4,5,5,6,6,7,7,8,8,8-Hexadecafluorooct-2-ene	65500-50-7	FC(=C(F)C(F)(F)C(F)(F)C(F)(F)C(F)(F)C(F)(F)F)C(F)(F)F		-7.52	0.68	2.24	P	IN
QL01970	1,1,1,2,3,4,4,5,5,6,6,7,7,7-Tetradecafluorohept-2-ene	1582-32-7	FC(=C(F)C(F)(F)C(F)(F)C(F)(F)C(F)(F)F)C(F)(F)F		-7.27	0.67	2.17	P	IN
QL01973	1,1,1,2,2,3,4,5,5,6,6,7,7,7-Tetradecafluorohept-3-ene	71039-88-8	FC(=C(F)C(F)(F)C(F)(F)C(F)(F)F)C(F)(F)C(F)(F)F		-7.28	0.67	2.14	P	IN
QL01975	Perfluoro-2-hexene	1584-00-5	FC(=C(F)C(F)(F)C(F)(F)C(F)(F)F)C(F)(F)F		-6.63	0.67	1.91	P	IN
QL01976	Perfluoro-2-pentene	72804-49-0	FC(=C(F)C(F)(F)C(F)(F)F)C(F)(F)F		-6.21	0.66	1.78	P	IN
QL01977	Perfluorobut-2-ene	360-89-4	FC(=C(F)C(F)(F)F)C(F)(F)F		-5.18	0.65	1.46	P	IN
QL01978	2,3,3,3-Tetrafluoro-2-(heptafluoropropoxy)propyl 2-fluoroprop-2-enoate	96250-49-6	FC(=C)C(=O)OCC(F)(OC(F)(F)C(F)(F)C(F)(F)F)C(F)(F)F		-7.36	0.65	2.47	P	IN
QL01979	3,3,4,4,5,5,6,6,7,7,8,8,9,9,10,10,10-Heptadecafluorodecyl 2-fluoroprop-2-enoate	96250-52-1	FC(=C)C(=O)OCCC(F)(F)C(F)(F)C(F)(F)C(F)(F)C(F)(F)C(F)(F)C(F)(F)C(F)(F)F		-8.53	0.67	2.90	P	IN
QL01980	1,1,1,2,2,3,5,5,6,6,7,7,7-Tridecafluorohept-3-ene	648-39-5	FC(=CC(F)(F)C(F)(F)C(F)(F)F)C(F)(F)C(F)(F)F		-7.17	0.67	2.09	P	IN
QL01981	4,5,5,6,6,7,7,8,8,9,9,9-Dodecafluoronona-1,3-diene	74338-55-9	FC(=CC=C)C(F)(F)C(F)(F)C(F)(F)C(F)(F)C(F)(F)F		-6.91	0.65	2.24	P	IN
QL01982	3,4,4,5,5,6,6,7,7,8,8,9,9,10,10,11,11,12,12,12-Icosafluorododec-2-enal	864551-40-6	FC(=CC=O)C(F)(F)C(F)(F)C(F)(F)C(F)(F)C(F)(F)C(F)(F)C(F)(F)C(F)(F)C(F)(F)F		-8.34	0.67	2.74	P	IN
QL01983	3,4,4,5,5,6,6,7,7,8,8,8-Dodecafluorooct-2-enal	69534-12-9	FC(=CC=O)C(F)(F)C(F)(F)C(F)(F)C(F)(F)C(F)(F)F		-6.91	0.65	2.24	P	IN
QL01984	1,1,1,2,2,3,3,4,4,7,8,8,9,9,10,10,11,11,12,12,13,13,14,14,14-Pentacosafluorotetradec-6-ene	53638-14-5	FC(=CCC(F)(F)C(F)(F)C(F)(F)C(F)(F)F)C(F)(F)C(F)(F)C(F)(F)C(F)(F)C(F)(F)C(F)(F)C(F)(F)F		-9.08	0.68	2.94	P	OUT
QL01985	1,1,1,2,2,3,3,4,7,7,8,8,8-Tridecafluorooct-4-ene	343627-68-9	FC(=CCC(F)(F)C(F)(F)F)C(F)(F)C(F)(F)C(F)(F)F		-7.25	0.67	2.21	P	IN
QL01986	2,2,3,3,4,4,4-Heptafluoro-N-(nonafluorobutyl)butanimidoyl fluoride	103573-07-5	FC(=NC(F)(F)C(F)(F)C(F)(F)C(F)(F)F)C(F)(F)C(F)(F)C(F)(F)F		-7.92	0.68	2.39	P	IN
QL01987	Hentriacontafluorohexadecanoyl fluoride	210972-74-0	FC(=O)C(F)(F)C(F)(F)C(F)(F)C(F)(F)C(F)(F)C(F)(F)C(F)(F)C(F)(F)C(F)(F)C(F)(F)C(F)(F)C(F)(F)C(F)(F)C(F)(F)C(F)(F)F		-9.68	0.69	3.12	P	OUT
QL01988	ketone perfluoroalkyl (linear) carboxylic acids_i, n=12		FC(=O)C(F)(F)C(F)(F)C(F)(F)C(F)(F)C(F)(F)C(F)(F)C(F)(F)C(F)(F)C(F)(F)C(F)(F)C(F)(F)C(F)(F)S(=O)(O)=O		-9.31	0.69	2.95	P	OUT
QL01989	ketone perfluoroalkyl (linear) carboxylic acids_i, n=11		FC(=O)C(F)(F)C(F)(F)C(F)(F)C(F)(F)C(F)(F)C(F)(F)C(F)(F)C(F)(F)C(F)(F)C(F)(F)C(F)(F)S(=O)(O)=O		-9.18	0.69	2.89	P	OUT
QL01990	ketone perfluoroalkyl (linear) carboxylic acids_i, n=10		FC(=O)C(F)(F)C(F)(F)C(F)(F)C(F)(F)C(F)(F)C(F)(F)C(F)(F)C(F)(F)C(F)(F)C(F)(F)S(=O)(O)=O		-8.87	0.69	2.75	P	OUT
QL01991	ketone perfluoroalkyl (linear) carboxylic acids_i, n=9		FC(=O)C(F)(F)C(F)(F)C(F)(F)C(F)(F)C(F)(F)C(F)(F)C(F)(F)C(F)(F)C(F)(F)S(=O)(O)=O		-8.72	0.69	2.69	P	IN
QL01992	Heptadecafluorononanoyl fluoride	558-95-2	FC(=O)C(F)(F)C(F)(F)C(F)(F)C(F)(F)C(F)(F)C(F)(F)C(F)(F)C(F)(F)F		-8.18	0.68	2.45	P	IN
QL01993	ketone perfluoroalkyl (linear) carboxylic acids_i, n=8		FC(=O)C(F)(F)C(F)(F)C(F)(F)C(F)(F)C(F)(F)C(F)(F)C(F)(F)C(F)(F)S(=O)(O)=O		-8.35	0.69	2.51	P	IN
QL01994	ketone perfluoroalkyl (linear) carboxylic acids_i, n=7		FC(=O)C(F)(F)C(F)(F)C(F)(F)C(F)(F)C(F)(F)C(F)(F)C(F)(F)S(=O)(O)=O		-8.18	0.68	2.45	P	IN
QL01995	ketone perfluoroalkyl (linear) carboxylic acids_i, n=6		FC(=O)C(F)(F)C(F)(F)C(F)(F)C(F)(F)C(F)(F)C(F)(F)S(=O)(O)=O		-7.72	0.68	2.24	P	IN

QL01996	ketone perfluoroalkyl (linear) carboxylic acids_i, n=5		FC(=O)C(F)(F)C(F)(F)C(F)(F)C(F)(F)C(F)(F)S(=O)(O)=O	-7.50	0.68	2.16	P	IN
QL01997	Perfluoropentanoyl fluoride	375-62-2	FC(=O)C(F)(F)C(F)(F)C(F)(F)C(F)(F)F	-6.61	0.67	1.81	P	IN
QL01998	ketone perfluoroalkyl (linear) carboxylic acids_i, n=4		FC(=O)C(F)(F)C(F)(F)C(F)(F)C(F)(F)S(=O)(O)=O	-6.91	0.68	1.90	P	IN
QL01999	ketone perfluoroalkyl (linear) carboxylic acids_i, n=3		FC(=O)C(F)(F)C(F)(F)C(F)(F)S(=O)(O)=O	-6.61	0.67	1.81	P	IN
QL02000	Pentafluoropropionyl fluoride	422-61-7	FC(=O)C(F)(F)C(F)(F)F	-5.29	0.66	1.36	P	IN
QL02001	Perfluoromethoxypropionyl fluoride	425-38-7	FC(=O)C(F)(F)C(F)(F)OC(F)(F)F	-6.01	0.65	1.84	P	IN
QL02002	Difluoro[1,1,2,2-tetrafluoro-2-(nonafluorobutoxy)ethoxy]acetyl fluoride	172897-75-5	FC(=O)C(F)(F)OC(F)(F)C(F)(F)OC(F)(F)C(F)(F)C(F)(F)C(F)(F)F	-7.95	0.67	2.51	P	IN
QL02003	Perfluoro(2-[(1-propoxypropan-2-yl)oxy]propanoyl) fluoride	112820-37-8	FC(=O)C(F)(OC(F)(C(F)(F)F)C(F)(F)OC(F)(F)C(F)(F)C(F)(F)F)C(F)(F)F	-7.79	0.67	2.40	P	IN
QL02004	Perfluoro(2-methyl-3-oxahexanoyl) fluoride	2062-98-8	FC(=O)C(F)(OC(F)(F)C(F)(F)C(F)(F)F)C(F)(F)F	-6.59	0.67	1.85	P	IN
QL02005	Propanoyl fluoride, 2,3,3,3-tetrafluoro-2-[1,1,2,2,3,3-hexafluoro-3-(trifluoromethoxy)propoxy]-	58194-00-6	FC(=O)C(F)(OC(F)(F)C(F)(F)C(F)(F)OC(F)(F)F)C(F)(F)F	-7.08	0.66	2.19	P	IN
QL02006	2,3,3,3-Tetrafluoro-2-(perfluoroethoxy)propanoyl fluoride	1682-78-6	FC(=O)C(F)(OC(F)(F)C(F)(F)F)C(F)(F)F	-6.05	0.66	1.65	P	IN
QL02007	Perfluoro(2,7-dimethyl-3,6-dioxasuberoyl) fluoride	1644-10-6	FC(=O)C(F)(OC(F)(F)C(F)(F)OC(F)(C(F)=O)C(F)(F)F)C(F)(F)F	-6.85	0.66	2.08	P	IN
QL02008	Perfluoro(3-chloro-2-[2-(fluorosulfonyl)ethoxy]propanoyl) fluoride	81126-57-0	FC(=O)C(F)(OC(F)(F)C(F)(F)S(F)(=O)=O)C(F)(F)Cl	-6.59	0.67	1.85	P	IN
QL02009	2,3,3,3-Tetrafluoro-2-(1,1,2,2-tetrafluoro-2-(fluorosulphonyl)ethoxy)propionyl fluoride	4089-57-0	FC(=O)C(F)(OC(F)(F)C(F)(F)S(F)(=O)=O)C(F)(F)F	-6.59	0.67	1.85	P	IN
QL02010	Hexafluoropropene oxide trimer	2641-34-1	FC(=O)C(F)(OC(F)(F)C(F)(OC(F)(F)C(F)(F)C(F)(F)F)C(F)(F)F)C(F)(F)F	-7.79	0.67	2.40	P	IN
QL02011	8-Fluorosulfonylperfluoro(2,5-dimethyl-3,6-dioxaoctanoyl) fluoride	4089-58-1	FC(=O)C(F)(OC(F)(F)C(F)(OC(F)(F)C(F)(F)S(F)(=O)=O)C(F)(F)F)C(F)(F)F	-7.79	0.67	2.40	P	IN
QL02012	Perfluoro-2,5,8-trimethyl-3,6,9-trioxadodecanoyl fluoride	27639-98-1	FC(=O)C(F)(OC(F)(F)C(F)(OC(F)(F)C(F)(OC(F)(F)C(F)(F)C(F)(F)F)C(F)(F)F)C(F)(F)F)C(F)(F)F	-8.36	0.68	2.67	P	IN
QL02013	Perfluoro-2,5,8,11-tetramethyl-3,6,9,12-tetraoxapentadecanoyl fluoride	34761-47-2	FC(=O)C(F)(OC(F)(F)C(F)(OC(F)(F)C(F)(OC(F)(F)C(F)(OC(F)(F)C(F)(F)C(F)(F)F)C(F)(F)F)C(F)(F)F)C(F)(F)F)C(F)(F)F	-9.06	0.68	3.01	P	OUT
QL02014	HFPO hexamer acid fluoride	13252-15-8	FC(=O)C(F)(OC(F)(F)C(F)(OC(F)(F)C(F)(OC(F)(F)C(F)(OC(F)(F)C(F)(OC(F)(F)C(F)(F)C(F)(F)F)C(F)(F)F)C(F)(F)F)C(F)(F)F)C(F)(F)F)C(F)(F)F	-9.44	0.68	3.19	P	OUT
QL02015	3,6,9,12,15,18-Hexaoxaheneicosanoyl fluoride, 2,4,4,5,7,7,8,10,10,11,13,13,14,16,16,17,19,19,20,20,21,21,21-tricosafluoro-2,5,8,11,14,17-hexakis(trifluoromethyl)-	13140-24-4	FC(=O)C(F)(OC(F)(F)C(F)(OC(F)(F)C(F)(OC(F)(F)C(F)(OC(F)(F)C(F)(OC(F)(F)C(F)(OC(F)(F)C(F)(F)C(F)(F)F)C(F)(F)F)C(F)(F)F)C(F)(F)F)C(F)(F)F)C(F)(F)F)C(F)(F)F	-9.94	0.68	3.43	P	OUT
QL02016	3,6,9,12,15,18,21-Heptaoxatetracosanoyl fluoride, 2,4,4,5,7,7,8,10,10,11,13,13,14,16,16,17,19,19,20,22,22,23,23,24,24,24-hexacosafluoro-2,5,8,11,14,17,20-heptakis(trifluoromethyl)-	13140-25-5	FC(=O)C(F)(OC(F)(F)C(F)(OC(F)(F)C(F)(OC(F)(F)C(F)(OC(F)(F)C(F)(OC(F)(F)C(F)(OC(F)(F)C(F)(OC(F)(F)C(F)(F)C(F)(F)F)C(F)(F)F)C(F)(F)F)C(F)(F)F)C(F)(F)F)C(F)(F)F)C(F)(F)F)C(F)(F)F	-10.22	0.68	3.58	P	OUT
QL02017	3,6,9,12,15,18,21,24-Octaoxaheptacosanoyl fluoride, 2,4,4,5,7,7,8,10,10,11,13,13,14,16,16,17,19,19,20,22,22,23,25,25,26,26,27,27,27-nonacosafluoro-2,5,8,11,14,17,20,23-octakis(trifluoromethyl)-	13140-26-6	FC(=O)C(F)(OC(F)(F)C(F)(OC(F)(F)C(F)(OC(F)(F)C(F)(OC(F)(F)C(F)(OC(F)(F)C(F)(OC(F)(F)C(F)(OC(F)(F)C(F)(OC(F)(F)C(F)(F)C(F)(F)F)C(F)(F)F)C(F)(F)F)C(F)(F)F)C(F)(F)F)C(F)(F)F)C(F)(F)F)C(F)(F)F)C(F)(F)F	-10.60	0.68	3.77	P	OUT
QL02018	2,4,4,5,7,7,8,10,10,11,13,13,14,16,16,17,19,19,20,22,22,23,25,25,26,28,28,29,29,30,30,30-Dotriacontafluoro-2,5,8,11,14,17,20,23,26-nonakis(trifluoromethyl)-3,6,9,12,15,18,21,24,27-nonaoxatriacontan-1-oyl fluoride	65150-88-1	FC(=O)C(F)(OC(F)(F)C(F)(OC(F)(F)C(F)(OC(F)(F)C(F)(OC(F)(F)C(F)(OC(F)(F)C(F)(OC(F)(F)C(F)(OC(F)(F)C(F)(OC(F)(F)C(F)(OC(F)(F)C(F)(F)C(F)(F)F)C(F)(F)F)C(F)(F)F)C(F)(F)F)C(F)(F)F)C(F)(F)F)C(F)(F)F)C(F)(F)F)C(F)(F)F)C(F)(F)F	-10.83	0.68	3.88	P	OUT
QL02019	Perfluoro(2-(2-phenoxypropoxy)propanoyl) fluoride	25711-77-7	FC(=O)C(F)(OC(F)(F)C(F)(OC1=C(F)C(F)=C(F)C(F)=C1F)C(F)(F)F)C(F)(F)F	-7.35	0.65	2.48	P	IN
QL02020	2,3,3,3-Tetrafluoro-2-(trifluoromethoxy)propionyl fluoride	2927-83-5	FC(=O)C(F)(OC(F)(F)F)C(F)(F)F	-5.07	0.65	1.38	P	IN
QL02021	(E)-Perfluoro-4-methylpent-2-enoyl fluoride	103229-77-2	FC(=O)C(F)=C(/F)C(F)(C(F)(F)F)C(F)(F)F	-5.77	0.65	1.71	P	IN
QL02022	Perfluoro-4-methylpent-2-enoyl fluoride	88022-48-4	FC(=O)C(F)=C(F)C(F)(C(F)(F)F)C(F)(F)F	-5.77	0.65	1.71	P	IN
QL02023	Cyclopentanecarbonyl fluoride, 1,2,2,3,3,4,5,5-octafluoro-4-(trifluoromethyl)-	1646599-70-3	FC(=O)C1(F)C(F)(F)C(F)(F)C(F)(C(F)(F)F)C1(F)F	-6.82	0.68	1.77	P	IN
QL02024	Perfluorocyclohexanecarbonyl fluoride	6588-63-2	FC(=O)C1(F)C(F)(F)C(F)(F)C(F)(F)C(F)(F)C1(F)F	-6.29	0.69	1.44	P	IN
QL02025	Tribromofluoromethane	353-54-8	FC(Br)(Br)Br	-2.48	0.60	0.72	P	OUT
QL02026	ammonium difluoro[1,1,2,2-tetrafluoro-2-(pentafluoroethoxy)ethoxy]acetate	908020-52-0	FC(C(=O)[O-])(OC(C(OC(C(F)(F)F)(F)F)(F)F)(F)F)F.[NH4+]	-6.57	0.63	2.34	P	OUT

QL02027	Bis(2,2,3,3,4,4,5,5,6,6,7,7-dodecafluoroheptanoyl) peroxide	32687-76-6	FC(C(C(C(C(C(C(=O)OOC(=O)C(C(C(C(C(C(F)F)(F)F)(F)F)(F)F)(F)F)(F)F)(F)F)(F)F)(F)F)(F)F)(F)F)F		-9.02	0.67	3.07	P	OUT
QL02028	Perfluoroeicosyl iodide	29809-34-5	FC(C(C(C(C(C(C(C(C(C(C(C(C(C(C(C(C(C(C(C(F)(F)F)(F)F)(F)F)(F)F)(F)F)(F)F)(F)F)(F)F)(F)F)(F)F)(F)F)(F)F)(F)F)(F)F)(F)F)(F)F)(F)F)(F)F)(I)F		-10.40	0.70	3.42	P	OUT
QL02029	Perfluorooctadecyl iodide	29809-35-6	FC(C(C(C(C(C(C(C(C(C(C(C(C(C(C(C(C(C(F)(F)F)(F)F)(F)F)(F)F)(F)F)(F)F)(F)F)(F)F)(F)F)(F)F)(F)F)(F)F)(F)F)(F)F)(F)F)(F)F)(I)F		-10.12	0.70	3.28	P	OUT
QL02030	Perfluoro-1-iodo-9-methyldecane	677-93-0	FC(C(C(C(C(C(C(C(C(F)(F)I)(F)F)(F)F)(F)F)(F)F)(F)F)(F)F)(F)F)(C(F)(F)F)C(F)(F)F		-8.43	0.69	2.48	P	IN
QL02031	Perfluoro(7-methyl)octanesulfonate potassium salt	40365-28-4	FC(C(C(C(C(C(C(C(F)(F)F)(C(F)(F)F)F)(F)F)(F)F)(F)F)(F)F)(F)F)([S](=O)(=O)[O-])F.[K+]		-7.91	0.67	2.53	P	IN
QL02032	N3-(2-(((heptadecafluorooctyl)sulfonyl)methylamino)ethyl)-N1-(2-(((heptadecafluorooctyl)sulfonyl)propylamino)ethyl)-4-methyl-1,3-benzenedicarboxamide	73019-20-2	FC(C(C(C(C(C(C(C(F)(F)F)(F)F)(F)F)(F)F)(F)F)(F)F)(F)F)(F)S(=O)(=O)N(CCNC(=O)C=1C=C(C=CC1C)C(=O)NCCN(CCC)S(=O)(=O)C(C(C(C(C(C(C(C(F)(F)F)(F)F)(F)F)(F)F)(F)F)(F)F)(F)F)(F)F)C		-10.43	0.66	3.95	P	OUT
QL02033	Pentacosafluoro-1-iodododecane	307-60-8	FC(C(C(C(C(C(C(F)(F)I)(F)F)(F)F)(F)F)(F)F)(F)F)(F)F)(C(C(C(C(C(F)(F)F)(F)F)(F)F)(F)F)(F)F)F		-9.02	0.69	2.75	P	OUT
QL02034	Perflubrodec	307-43-7	FC(C(C(C(C(C(F)(F)Br)(F)F)(F)F)(F)F)(F)F)(F)F)(C(C(C(C(F)(F)F)(F)F)(F)F)(F)F)F		-8.53	0.69	2.51	P	IN
QL02035	Perfluorodecyl iodide	423-62-1	FC(C(C(C(C(C(F)(F)I)(F)F)(F)F)(F)F)(F)F)(F)F)(C(C(C(C(F)(F)F)(F)F)(F)F)(F)F)F		-8.53	0.69	2.51	P	IN
QL02036	1-Chloro-perfluorohexanesulfonic acid		FC(C(C(C(C(C(S(=O)(=O)O)(Cl)F)(F)F)(F)F)(F)F)(F)F)(F)F		-7.74	0.69	2.15	P	IN
QL02037	Perfluorooctyl iodide	507-63-1	FC(C(C(C(C(F)(F)I)(F)F)(F)F)(F)F)(C(C(C(F)(F)F)(F)F)(F)F)F		-7.94	0.69	2.24	P	IN
QL02038	Fluorocarbon FC 70	338-84-1	FC(C(C(C(C(N(C(F)(F)C(F)(F)C(F)(F)C(F)(F)C(F)(F)F)C(C(C(C(C(F)(F)F)(F)F)(F)F)(F)F)(F)F)(F)F)(F)F)(F)F)(F)F)(F)F		-8.67	0.69	2.61	P	IN
QL02039	1-Chloro-perfluorooctanesulfonic acid		FC(C(C(C(C(S(=O)(=O)O)(Cl)F)(F)F)(F)F)(F)F)(F)F)(C(C(C(F)(F)F)(F)F)(F)F)F		-8.37	0.69	2.44	P	IN
QL02040	Perflexane	355-42-0	FC(C(C(C(F)(F)F)(F)F)(F)F)(C(C(F)(F)F)(F)F)F		-7.19	0.69	1.90	P	IN
QL02041	Perfluorohexyl iodide	355-43-1	FC(C(C(C(F)(F)I)(F)F)(F)F)(C(C(F)(F)F)(F)F)F		-7.19	0.69	1.90	P	IN
QL02042	Perfluoroisononyl iodide	865-77-0	FC(C(C(F)(F)F)(C(F)(F)F)F)(C(C(C(C(C(F)(F)I)(F)F)(F)F)(F)F)(F)F)F		-7.82	0.69	2.20	P	IN
QL02043	Iodononafluoro-t-butane	4459-18-1	FC(C(C(F)(F)F)(C(F)(F)F)I)(F)F		-5.53	0.68	1.20	P	IN
QL02044	1,1,2,2,3,3,4,4,5,5,6,6,7,7,7-pentadecafluoro-N-(2-hydroxyethyl)heptane-1-sulfonamide	167398-54-1	FC(C(C(F)(F)F)(F)F)(F)C(F)(F)C(F)(F)C(F)(F)C(F)(F)S(=O)(NCCO)=O		-8.10	0.66	2.68	P	IN
QL02045	Nonafluoro-1-iodobutane	423-39-2	FC(C(C(F)(F)I)(F)F)(C(F)(F)F)F		-6.18	0.68	1.45	P	IN
QL02046	Perfluoro-1,4-diiodobutane	375-50-8	FC(C(C(F)(F)I)(F)F)(C(F)(F)I)F		-6.18	0.68	1.45	P	IN
QL02047	Perfluoro-4-methylpent-2-enoyl chloride	207671-78-1	FC(C(Cl)=O)=C(F)C(F)(C(F)(F)F)C(F)(F)F		-5.77	0.65	1.71	P	IN
QL02048	122 Fluorotelomer unsaturated carboxylic acid	70887-95-5	FC(C(F)(C(F)(C(C(F)CC(O)=O)(F)F)F)F)(C(F)(F)C(F)(F)C(F)(C(F)(C(F)(C(F)(C(F)(F)F)F)F)F)F)F		-8.97	0.68	2.94	P	OUT
QL02052	Perfluoroheptadecanoic acid	57475-95-3	FC(C(F)(C(O)=O)F)(C(F)(C(F)(C(F)(C(F)(C(F)(C(F)(C(F)(C(F)(F)C(F)(F)C(F)(F)C(F)(F)C(F)(C(F)(F)C(F)(F)F)F)F)F)F)F)F)F)F)F		-9.92	0.69	3.24	P	OUT
QL02053	Perfluoropentadecanoic acid (PFPeDA)	141074-63-7	FC(C(F)(C(O)=O)F)(C(F)(C(F)(C(F)(C(F)(C(F)(C(F)(C(F)(C(F)(F)C(F)(F)C(F)(F)C(F)(F)C(F)(F)F)F)F)F)F)F)F)F)F		-9.58	0.69	3.07	P	OUT
QL02054	3,3,4,4,5,5,6,6,7,7,8,8,9,9,10,10,11,11,12,12,12-henicosafluorododecyl (3,3,4,4,5,5,6,6,7,7,8,8,9,9,10,10,11,11,12,12,13,13,14,14,14-pentacosafluorotetradecyl) hydrogen phosphate	1158182-61-6	FC(C(F)(F)C(F)(C(F)(F)C(F)(F)F)F)(C(F)(C(F)(C(F)(C(F)(C(F)(F)CCOP(O)(OCCC(F)(C(F)(F)C(F)(F)C(F)(F)C(F)(F)C(F)(F)C(F)(F)C(F)(C(F)(F)C(F)(F)C(F)(F)C(F)(F)F)F)F)=O)F)F)F)F		-10.98	0.68	3.89	P	OUT
QL02055	(Perfluorohexadecyl)hexadecene	1244062-14-3	FC(C(F)(F)C(F)(F)C(F)(F)C(F)(F)C(F)(F)/C=C/CCCCCCCCCCCCCC[H])(F)C(F)(F)C(F)(F)C(F)(F)C(F)(F)C(F)(F)C(F)(F)C(F)(F)C(F)(F)C(F)(F)C(F)(F)F		-10.30	0.65	4.05	P	OUT
QL02056	(Perfluorooctyl)hexadecene	1244062-16-5	FC(C(F)(F)C(F)(F)C(F)(F)C(F)(F)C(F)(F)/C=C/CCCCCCCCCCCCCC[H])(F)C(F)(F)C(F)(F)F		-8.57	0.62	3.60	P	OUT
QL02057	Thiocyanic acid, 3,3,4,4,5,5,6,6,7,7,8,8,8-tridecafluorooctyl ester	26650-09-9	FC(C(F)(F)C(F)(F)C(F)(F)C(F)(F)C(F)(F)F)(F)CCSC#N		-7.35	0.65	2.52	P	IN
QL02058	3,3-Dichloro-1,1,1-trifluoroacetone hydrate	1049731-87-4	FC(C(F)(F)C(F)(F)C(F)(F)C(O)O)(F)C(F)(F)C(F)(C(F)(F)C(F)(C(F)(F)C(F)(F)F)F)F		-8.72	0.69	2.69	P	IN
QL02059	Perfluorononanal hydrate	191528-99-1	FC(C(F)(F)C(F)(F)C(F)(F)C(O)O)(F)C(F)(F)C(F)(C(F)(F)C(F)(F)F)F		-8.18	0.68	2.45	P	IN
QL02060	2,2,3,3,4,4,5,5,6,6,7,7,7-Tridecafluoro-1,1-heptanediol	64739-16-8	FC(C(F)(F)C(F)(F)C(F)(F)C(O)O)(F)C(F)(F)C(F)(F)F		-7.50	0.68	2.16	P	IN
QL02061	3,3,4,4,5,5,6,6,7,7,8,8,9,9,10,10,11,11,12,12,12-Henicosafluorododecyl 3,3,4,4,5,5,6,6,7,7,8,8,9,9,10,10,10-heptadecafluorodecyl hydrogen phosphate	1158182-60-5	FC(C(F)(F)C(F)(F)F)(C(F)(C(F)(C(F)(C(F)(C(F)(F)CCOP(O)(OCCC(F)(C(F)(F)C(F)(F)C(F)(F)C(F)(F)C(F)(F)C(F)(F)C(F)(C(F)(F)C(F)(F)F)F)F)=O)F)F)F)F		-10.50	0.68	3.68	P	OUT

QL02062	double bond perfluoroalkyl (linear) sulfonic aicds, n=5_i		FC(C(F)(F)C(F)(F)F)(F)/C(F)=C(F)/C(F)(F)C(F)(F)C(F)(F)S(O)(=O)=O		-8.01	0.68	2.43	P	IN
QL02063	1,1,1,2,2,3,3,4,5,5,6,6,6-Tridecafluorohexane	1998-67-0	FC(C(F)(F)C(F)(F)F)C(F)(F)C(F)(F)C(F)(F)F		-6.96	0.68	1.90	P	IN
QL02064	4-[bis(4-phenylphenyl)-1,3,5-triazin-2-yl]benzene-1,3-diol		FC(C(F)(F)F)(C(F)(F)F)C1=CC(=C(N)C=C1)C		-6.01	0.64	1.90	P	IN
QL02065	1,1,2,2,3,3,4,4,5,5,6,6,6-tridecafluoro-N-(2-hydroxyethyl)hexane-1-sulfonamide	106443-63-4	FC(C(F)(F)F)(F)C(F)(F)C(F)(F)C(F)(F)C(F)(F)S(=O)(NCCO)=O		-7.64	0.66	2.51	P	IN
QL02066	1H,1H,3H-Perfluorobutyl acrylate	54052-90-3	FC(C(F)(F)F)C(F)(F)COC(=O)C=C		-5.65	0.61	2.17	P	OUT
QL02067	2,2,3,4,4,4-Hexafluorobutyl nonafluorobutane-1-sulfonate	1205020-79-6	FC(C(F)(F)F)C(F)(F)COS(=O)(=O)C(F)(F)C(F)(F)C(F)(F)C(F)(F)F		-7.97	0.67	2.51	P	IN
QL02068	Lufenuron	103055-07-8	FC(C(F)(F)F)C(F)(F)Oc1cc(Cl)c(NC(=O)NC(=O)c2c(F)cccc2F)cc1Cl		-7.39	0.61	3.04	P	OUT
QL02069	1,1,1,2,2,3,3-Heptafluoro-4-(1,1,2,3,3,3-hexafluoropropoxy)butane	1184-97-0	FC(C(F)(F)F)C(F)(F)OCC(F)(F)C(F)(F)C(F)(F)F		-7.09	0.66	2.17	P	IN
QL02070	2H-Hexafluoropropyl allyl ether	59158-81-5	FC(C(F)(F)F)C(F)(F)OCC=C		-4.98	0.60	1.95	P	OUT
QL02071	1,2,2,2-Tetrafluoroethanesulfonyl fluoride	2127-74-4	FC(C(F)(F)F)S(F)(=O)=O		-4.98	0.65	1.30	P	IN
QL02072	double bond-, H-substituted perfluoroalkyl sulfonic acids, n=4_iii		FC(C(F)(F)OC(F)(F)C(F)(F)C(F)(F)/C(F)=C(F)/C([H])(F)F)(F)S(=O)(O)=O		-7.78	0.66	2.52	P	IN
QL02073	double bond-, H-substituted perfluoroalkyl sulfonic acids, n=4_ii		FC(C(F)(F)OC(F)(F)C(F)(F)C(F)(F)C(F)(F)/C([H])=C(F)F)(F)S(=O)(O)=O		-7.84	0.67	2.51	P	IN
QL02074	double bond-, H-substituted perfluoroalkyl sulfonic acids, n=4_i		FC(C(F)(F)OC(F)(F)C(F)(F)C(F)(F)C(F)(F)/C(F)=C([H])F)(F)S(=O)(O)=O		-7.83	0.67	2.51	P	IN
QL02075	diH-substituted, O-subsitituted perfluoroalkyl (linear) sulfonic acids, c8		FC(C(F)(F)OC(F)(F)C(F)(F)C(F)(F)C(F)(F)C([H])(F)C([H])(F)F)(F)S(=O)(O)=O		-7.96	0.67	2.51	P	IN
QL02076	1,1,1,2,3,4,5,5,5-Nonafluoro-2-(trifluoromethyl)pentane	85720-78-1	FC(C(F)C(F)(C(F)(F)F)C(F)(F)F)C(F)(F)F		-6.09	0.66	1.68	P	IN
QL02077	Perfluoroalkyl (linear) dicarboxylic acids, n=15		FC(C(O)=O)(C(F)(F)C(F)(F)C(F)(F)C(F)(F)C(F)(F)C(F)(F)C(F)(F)C(F)(F)C(F)(F)C(F)(F)C(F)(F)C(F)(F)C(F)(F)C(F)(F)C(O)=O)F		-9.80	0.69	3.23	P	OUT
QL02078	Perfluoroalkyl (linear) dicarboxylic acids, n=14		FC(C(O)=O)(C(F)(F)C(F)(F)C(F)(F)C(F)(F)C(F)(F)C(F)(F)C(F)(F)C(F)(F)C(F)(F)C(F)(F)C(F)(F)C(F)(F)C(F)(F)C(O)=O)F		-9.56	0.69	3.11	P	OUT
QL02079	Perfluoroalkyl (linear) dicarboxylic acids, n=13		FC(C(O)=O)(C(F)(F)C(F)(F)C(F)(F)C(F)(F)C(F)(F)C(F)(F)C(F)(F)C(F)(F)C(F)(F)C(F)(F)C(F)(F)C(F)(F)C(O)=O)F		-9.44	0.69	3.07	P	OUT
QL02080	Perfluoroalkyl (linear) dicarboxylic acids, n=12		FC(C(O)=O)(C(F)(F)C(F)(F)C(F)(F)C(F)(F)C(F)(F)C(F)(F)C(F)(F)C(F)(F)C(F)(F)C(F)(F)C(F)(F)C(O)=O)F		-9.16	0.69	2.94	P	OUT
QL02081	Perfluoroalkyl (linear) dicarboxylic acids, n=11		FC(C(O)=O)(C(F)(F)C(F)(F)C(F)(F)C(F)(F)C(F)(F)C(F)(F)C(F)(F)C(F)(F)C(F)(F)C(F)(F)C(O)=O)F		-9.03	0.68	2.89	P	OUT
QL02082	Perfluoroalkyl (linear) dicarboxylic acids, n=9		FC(C(O)=O)(C(F)(F)C(F)(F)C(F)(F)C(F)(F)C(F)(F)C(F)(F)C(F)(F)C(F)(F)C(O)=O)F		-8.54	0.68	2.68	P	IN
QL02083	1,4-Dibromooctafluorobutane	335-48-8	FC(F)(Br)C(F)(F)C(F)(F)C(F)(F)Br		-6.18	0.68	1.45	P	IN
QL02084	1,6-Dibromododecafluorohexane	918-22-9	FC(F)(Br)C(F)(F)C(F)(F)C(F)(F)C(F)(F)C(F)(F)Br		-7.19	0.69	1.90	P	IN
QL02085	2-Vinyl(1-bromoperfluoroethane)	18599-22-9	FC(F)(Br)C(F)(F)C=C		-4.95	0.64	1.39	P	IN
QL02086	1,4-Dibromo-1,1,2,2-tetrafluorobutane	18599-20-7	FC(F)(Br)C(F)(F)CCBr		-4.54	0.62	1.52	P	OUT
QL02087	Hexafluoroglutaryl chloride	678-77-3	FC(F)(C(Cl)=O)C(F)(F)C(F)(F)C(Cl)=O		-6.21	0.66	1.81	P	IN
QL02088	Perfluoroadipoyl chloride	336-06-1	FC(F)(C(Cl)=O)C(F)(F)C(F)(F)C(F)(F)C(Cl)=O		-6.57	0.67	1.89	P	IN
QL02089	62/82 Fluorotelomer phosphate diester	943913-15-3	FC(F)(C(F)(C(F)(C(F)(C(F)(C(F)(F)CCOP(O)(OCCC(F)(C(F)(F)C(F)(F)C(F)(F)C(F)(F)C(F)(F)C(F)(F)C(F)(F)F)F)=O)F)F)F)F		-9.91	0.68	3.44	P	OUT
QL02090	82 Fluorotelomer unsaturated aldehyde	58544-13-1	FC(F)(C(F)(C(F)(C(F)(F)C(F)(F)F)F)F)C(F)(F)C(F)(F)/C(F)=C/C=O		-7.71	0.66	2.51	P	IN
QL02091	3,3,4,4,5,5,6,6,6-nonafluorohexyl (3,3,4,4,5,5,6,6,7,7,8,8,8-tridecafluorooctyl) hydrogen phosphate	1158182-59-2	FC(F)(C(F)(C(F)(C(F)(F)CCOP(O)(OCCC(F)(C(F)(F)C(F)(F)C(F)(F)C(F)(F)C(F)(F)F)F)=O)F)F)F		-9.17	0.67	3.16	P	OUT
QL02093	62 Fluorotelomer unsaturated aldehyde	69534-12-9	FC(F)(C(F)(C(F)(F)F)F)C(F)(F)C(F)(F)/C(F)=C/C=O		-6.91	0.65	2.24	P	IN
QL02094	122 Fluorotelomer unsaturated aldehyde		FC(F)(C(F)(C(F)(F)F)F)C(F)(F)C(F)(F)C(F)(F)C(F)(F)C(F)(F)C(F)(F)C(F)(F)C(F)(F)/C(F)=C/C=O		-8.86	0.67	2.94	P	OUT
QL02097	H-substituted Perfluoroalkyl (linear) dicarboxylic acids, n=12		FC(F)(C(F)(F)C(F)(F)C(F)(F)C(F)(F)C(F)(F)C(F)(F)C(F)(F)C(F)(F)C(F)(F)C(F)(F)C(F)(F)C(O)=O)C(F)C(O)=O		-9.31	0.68	3.07	P	OUT
QL02098	H-substituted Perfluoroalkyl (linear) dicarboxylic acids, n=11		FC(F)(C(F)(F)C(F)(F)C(F)(F)C(F)(F)C(F)(F)C(F)(F)C(F)(F)C(F)(F)C(F)(F)C(F)(F)C(O)=O)C(F)C(O)=O		-9.01	0.68	2.94	P	OUT
QL02099	H-substituted Perfluoroalkyl (linear) dicarboxylic acids, n=10		FC(F)(C(F)(F)C(F)(F)C(F)(F)C(F)(F)C(F)(F)C(F)(F)C(F)(F)C(F)(F)C(F)(F)C(O)=O)C(F)C(O)=O		-8.87	0.68	2.89	P	OUT
QL02100	H-substituted Perfluoroalkyl (linear) dicarboxylic acids, n=9		FC(F)(C(F)(F)C(F)(F)C(F)(F)C(F)(F)C(F)(F)C(F)(F)C(F)(F)C(F)(F)C(O)=O)C(F)C(O)=O		-8.53	0.68	2.74	P	IN
QL02101	H-substituted Perfluoroalkyl (linear) dicarboxylic acids, n=8		FC(F)(C(F)(F)C(F)(F)C(F)(F)C(F)(F)C(F)(F)C(F)(F)C(F)(F)C(O)=O)C(F)C(O)=O		-8.35	0.67	2.68	P	IN
QL02102	2-(N-Methylperfluorooctanesulfonamido)acetic acid (MeFOSAA)	2355-31-9	FC(F)(C(F)(F)C(F)(F)C(F)(F)C(F)(F)C(F)(F)C(F)(F)C(F)(F)F)S(=O)(=O)N(C)CC(O)=O		-8.47	0.67	2.74	P	IN

QL02103	2-(N-Ethylperfluorooctanesulfonamido)acetic acid (EtFOSAA)	2991-50-6	FC(F)(C(F)(F)C(F)(F)C(F)(F)C(F)(F)C(F)(F)C(F)(F)C(F)(F)F)S(=O)(=O)N(CC(O)=O)CC		-8.26	0.67	2.75	P	IN
QL02105	1,1,2,2,3,3,4,4,5,5,6,6,7,7,7-pentadecafluoroheptane-1-sulfonamide	82765-77-3	FC(F)(C(F)(F)C(F)(F)C(F)(F)C(F)(F)C(F)(F)F)C(F)(F)S(=O)(N)=O		-7.94	0.69	2.24	P	IN
QL02106	n2 fluorotelomer thioamido ammonium, n=3		FC(F)(C(F)(F)C(F)(F)C(F)(F)C(F)(F)C(F)(F)F)CCS(CC(NCCC[N+](C)(C)C)=O)=O		-8.30	0.64	3.09	P	OUT
QL02107	Perfluorooctanesulfonamido ethanol	10116-92-4	FC(F)(C(F)(F)C(F)(F)C(F)(F)C(F)(F)F)C(F)(F)C(F)(F)C(F)(F)S(=O)(NCCO)=O		-8.29	0.67	2.73	P	IN
QL02108	Perfluorohexanesulfonamide	41997-13-1	FC(F)(C(F)(F)C(F)(F)C(F)(F)C(F)(F)F)C(F)(F)S(=O)(N)=O		-7.74	0.69	2.15	P	IN
QL02109	N-ethyl-1,1,2,2,3,3,4,4,5,5,6,6,6-tridecafluorohexane-1-sulfonamide	87988-56-5	FC(F)(C(F)(F)C(F)(F)C(F)(F)C(F)(F)F)C(F)(F)S(=O)(NCC)=O		-7.69	0.67	2.44	P	IN
QL02110	1,1,1,2,2,3,3,4,4,5,5-undecafluoro-5-(propylsulfonyl)pentane	40630-67-9	FC(F)(C(F)(F)C(F)(F)C(F)(F)F)C(F)(F)S(=O)(CCC)=O		-7.16	0.66	2.23	P	IN
QL02111	1,1,2,2,3,3,4,4,5,5,5-undecafluoropentane-1-sulfonamide	82765-76-2	FC(F)(C(F)(F)C(F)(F)C(F)(F)F)C(F)(F)S(=O)(N)=O		-7.19	0.69	1.90	P	IN
QL02112	N-ethyl-1,1,2,2,3,3,4,4,5,5,5-undecafluoropentane-1-sulfonamide	162682-16-8	FC(F)(C(F)(F)C(F)(F)C(F)(F)F)C(F)(F)S(=O)(NCC)=O		-7.16	0.66	2.23	P	IN
QL02113	3-(Perfluorobutyl)propanoic acid	80705-13-1	FC(F)(C(F)(F)C(F)(F)C(F)(F)F)CCC(=O)O		-6.77	0.65	2.19	P	IN
QL02114	1-(Perfluorohexyl)octane	133331-77-8	FC(F)(C(F)(F)C(F)(F)F)C(F)(F)C(F)(F)C(F)(F)CCCCCCCC		-7.64	0.63	2.90	P	OUT
QL02115	Perfluorobutylsulphonamide	30334-69-1	FC(F)(C(F)(F)C(F)(F)F)C(F)(F)S(=O)(N)=O		-6.93	0.69	1.79	P	IN
QL02116	di Cl-substituted perfluoroalkyl (linear) sulfonic acids, C8		FC(F)(C(F)(F)C(S(=O)(O)=O)(F)F)C(Cl)(F)C(C(F)(F)C(Cl)(F)C(F)(F)F)(F)F		-8.37	0.69	2.44	P	IN
QL02117	Perfluoro-1,2-bis(perfluorocyclohexyl)ethane	306-99-0	FC(F)(C(F)(F)C1(F)C(F)(F)C(F)(F)C(F)(F)C(F)(F)C1(F)F)C1(F)C(F)(F)C(F)(F)C(F)(F)C(F)(F)C1(F)F		-8.55	0.70	2.39	P	IN
QL02118	82 Fluorotelomer aldehyde	135984-68-8	FC(F)(C(F)(F)CC=O)C(F)(F)C(F)(F)C(F)(F)C(F)(F)C(F)(F)C(F)(F)F		-7.91	0.67	2.50	P	IN
QL02119	42 Fluorotelomer aldehyde	135984-67-7	FC(F)(C(F)(F)CC=O)C(F)(F)C(F)(F)F		-6.13	0.65	1.91	P	IN
QL02121	double bond-, di-ether substituted perfluoroalkyl (linear) sulfonic acids, C7		FC(F)(C(S(=O)(O)=O)(F)F)OC(F)(C(F)(F)F)C(F)(F)O/C(F)=C(F)F		-7.74	0.66	2.44	P	IN
QL02122	H-substituted, di-ether-substituted perfluoroalkyl (linear) sulfonic acids, C7		FC(F)(C(S(=O)(O)=O)(F)F)OC(F)(C(F)(F)F)C(F)(F)OC(F)(F)C(F)([H])F		-7.76	0.67	2.43	P	IN
QL02123	1,8-Divinylperfluorooctane	35192-44-0	FC(F)(C=C)C(F)(F)C(F)(F)C(F)(F)C(F)(F)C(F)(F)C(F)(F)C(F)(F)C=C		-7.82	0.67	2.45	P	IN
QL02124	1,6-Divinylperfluorohexane	1800-91-5	FC(F)(C=C)C(F)(F)C(F)(F)C(F)(F)C(F)(F)C(F)(F)C=C		-7.05	0.66	2.16	P	IN
QL02125	1,4-Divinylperfluorobutane	678-65-9	FC(F)(C=C)C(F)(F)C(F)(F)C(F)(F)C=C		-5.99	0.65	1.81	P	IN
QL02126	Perfluoro(4a-(cyclohexylmethyl)decahydronaphthalene)	125061-94-1	FC(F)(C1(F)C(F)(F)C(F)(F)C(F)(F)C(F)(F)C1(F)F)C1(F)C(F)(F)C(F)(F)C(F)(F)C2(F)C(F)(F)C(F)(F)C(F)(F)C(F)(F)C12F		-8.53	0.70	2.36	P	IN
QL02127	Perfluoroperhydrobenzyl tetralin	116265-66-8	FC(F)(C1(F)C(F)(F)C(F)(F)C(F)(F)C(F)(F)C1(F)F)C1(F)C(F)(F)C(F)(F)C2(F)C(F)(F)C(F)(F)C(F)(F)C(F)(F)C2(F)C1(F)F		-8.97	0.70	2.58	P	OUT
QL02128	[1,1,1,2,2,3,3,4,4,14,14,15,15,16,16,17,17,17-Octadecafluoro-10-(4,4,5,5,6,6,7,7,7-nonafluoroheptyl)heptadec-9-en-8-yl]-lambda~2~-stannanyl	217805-03-3	FC(F)(CCCC([SnH])C=C(CCCC(F)(F)C(F)(F)C(F)(F)C(F)(F)F)CCCC(F)(F)C(F)(F)C(F)(F)C(F)(F)F)C(F)(F)C(F)(F)C(F)(F)F |^1:7|		-8.58	0.66	3.04	P	IN
QL02129	Decane, 3,3,4,4,5,5,6,6,7,7,8,8-dodecafluoro-1,10-diiodo-	1813-83-8	FC(F)(CCI)C(F)(F)C(F)(F)C(F)(F)C(F)(F)C(F)(F)CCI		-6.90	0.64	2.40	P	IN
QL02130	82 Fluorotelomer phosphate diester	678-41-1	FC(F)(CCOP(=O)(OCCC(F)(F)C(F)(F)C(F)(F)C(F)(F)C(F)(F)C(F)(F)C(F)(F)C(F)(F)F)O)C(F)(F)C(F)(F)C(F)(F)C(F)(F)C(F)(F)C(F)(F)C(F)(F)F		-10.17	0.68	3.54	P	OUT
QL02131	62 Fluorotelomer phosphate diester	4004-51-7	FC(F)(CCOP(=O)(OCCC(F)(F)C(F)(F)C(F)(F)C(F)(F)C(F)(F)C(F)(F)F)O)C(F)(F)C(F)(F)C(F)(F)C(F)(F)C(F)(F)F		-9.50	0.67	3.28	P	OUT
QL02133	Bis(3,5,6-trichloro-2,2,3,4,4,5,6,6-octafluoro-1-oxohexyl)peroxide	28285-49-6	FC(F)(Cl)C(F)(Cl)C(F)(F)C(F)(Cl)C(F)(F)C(=O)OOC(=O)C(F)(F)C(F)(Cl)C(F)(F)C(F)(Cl)C(F)(F)Cl		-8.86	0.68	2.89	P	OUT
QL02134	3,5,6-Trichloro-2,2,3,4,4,5,6,6-octafluorohexanoyl chloride	1422-98-6	FC(F)(Cl)C(F)(Cl)C(F)(F)C(F)(Cl)C(F)(F)C(Cl)=O		-6.91	0.68	1.90	P	IN
QL02135	4,5-Dichloroperfluoro-3-oxapentanesulfonyl fluoride	144728-59-6	FC(F)(Cl)C(F)(Cl)OC(F)(F)C(F)(F)S(F)(=O)=O		-6.92	0.68	1.89	P	IN
QL02136	1,1,3-trichloro-1,2,2,3,3-pentafluoropropane	1652-81-9	FC(F)(Cl)C(F)(F)C(F)(Cl)Cl		-5.80	0.68	1.32	P	IN
QL02137	1,8-Dichloroperfluorooctane	647-25-6	FC(F)(Cl)C(F)(F)C(F)(F)C(F)(F)C(F)(F)C(F)(F)C(F)(F)C(F)(F)Cl		-7.94	0.69	2.24	P	IN
QL02138	1-Chloro-8-iodoperfluorooctane	16486-98-9	FC(F)(Cl)C(F)(F)C(F)(F)C(F)(F)C(F)(F)C(F)(F)C(F)(F)C(F)(F)I		-7.94	0.69	2.24	P	IN
QL02139	11-chloroperfluoro-3-oxaundecanesulfonyl fluoride	73606-15-2	FC(F)(Cl)C(F)(F)C(F)(F)C(F)(F)C(F)(F)C(F)(F)C(F)(F)C(F)(F)OC(F)(F)C(F)(F)S(F)(=O)=O		-8.88	0.69	2.74	P	OUT
QL02140	1-Chloro-6-iodoperfluorohexane	16486-97-8	FC(F)(Cl)C(F)(F)C(F)(F)C(F)(F)C(F)(F)C(F)(F)I		-7.19	0.69	1.90	P	IN
QL02142	1,4-Dichloro-1,1,2,2,3,3,4,4-octafluorobutane	355-24-8	FC(F)(Cl)C(F)(F)C(F)(F)C(F)(F)Cl		-6.18	0.68	1.45	P	IN
QL02143	1,3-Dichloro-1,1,2,2,3,3-hexafluoropropane	662-01-1	FC(F)(Cl)C(F)(F)C(F)(F)Cl		-5.80	0.68	1.32	P	IN
QL02144	1H,1H,6H,6H-Perfluorohexane-1,6-diol diacrylate	2264-01-9	FC(F)(COC(=O)C=C)C(F)(F)C(F)(F)C(F)(F)COC(=O)C=C		-6.74	0.62	2.63	P	OUT
QL02145	1H,1H,5H,5H-Perfluoro-1,5-pentanediol diacrylate	678-95-5	FC(F)(COC(=O)C=C)C(F)(F)C(F)(F)COC(=O)C=C		-6.38	0.61	2.61	P	OUT

QL02146	1,5-bis(2,3-epoxypropoxy)-2,2,3,3,4,4-hexafluoropentane	4798-39-4	FC(F)(COCC1OC1)C(F)(F)C(F)(F)COCC2OC2		-6.73	0.62	2.61	P	OUT
QL02147	cis-Perfluoro-2,4-dimethylcyclobutane	1583-97-7	FC(F)(F)[C@]1(F)C(F)(F)[C@](F)(C(F)(F)F)C1(F)F		-7.28	0.69	1.89	P	IN
QL02148	(2R,4R)-1,1,2,3,3,4-Hexafluoro-2,4-bis(trifluoromethyl)cyclobutane	1583-98-8	FC(F)(F)[C@]1(F)C(F)(F)[C@@](F)(C(F)(F)F)C1(F)F		-7.28	0.69	1.89	P	IN
QL02149	double bond-, ether-substituted perfluoroalkyl (linear) carboxylic acids, n=4		FC(F)(F)/C(F)=C(F)/C(F)(F)C(F)(F)C(F)(F)C(F)(F)OC(F)(F)C(O)=O		-7.72	0.66	2.50	P	IN
QL02150	double bond-, ether-substituted perfluoroalkyl (linear) carboxylic acids, n=3		FC(F)(F)/C(F)=C(F)/C(F)(F)C(F)(F)C(F)(F)OC(F)(F)C(O)=O		-7.48	0.66	2.44	P	IN
QL02151	double bond-, ether-substituted perfluoroalkyl (linear) carboxylic acids, n=2		FC(F)(F)/C(F)=C(F)/C(F)(F)C(F)(F)OC(F)(F)C(O)=O		-6.91	0.65	2.23	P	IN
QL02152	double bond-, ether-substituted perfluoroalkyl (linear) carboxylic acids, n=1		FC(F)(F)/C(F)=C(F)/C(F)(F)OC(F)(F)C(O)=O		-6.55	0.64	2.17	P	IN
QL02153	double bond-, ether-substituted perfluoroalkyl (linear) carboxylic acids, n=0		FC(F)(F)/C(F)=C(OC(F)(F)C(O)=O)F		-5.75	0.63	1.91	P	IN
QL02154	H-substituted perfluoroalkyl (linear) sulfonic acids_ii, n=8		FC(F)(F)C([H])(F)C(F)(F)C(F)(F)C(F)(F)C(F)(F)C(F)(F)C(F)(F)C(F)(F)C(F)(F)S(=O)(O)=O		-8.71	0.69	2.69	P	IN
QL02155	H-substituted perfluoroalkyl (linear) sulfonic acids_ii, n=7		FC(F)(F)C([H])(F)C(F)(F)C(F)(F)C(F)(F)C(F)(F)C(F)(F)C(F)(F)C(F)(F)S(=O)(O)=O		-8.34	0.69	2.51	P	IN
QL02156	H-substituted perfluoroalkyl (linear) sulfonic acids_ii, n=6		FC(F)(F)C([H])(F)C(F)(F)C(F)(F)C(F)(F)C(F)(F)C(F)(F)C(F)(F)S(=O)(O)=O		-8.16	0.68	2.45	P	IN
QL02157	H-substituted perfluoroalkyl (linear) sulfonic acids_ii, n=5		FC(F)(F)C([H])(F)C(F)(F)C(F)(F)C(F)(F)C(F)(F)C(F)(F)S(=O)(O)=O		-7.70	0.68	2.24	P	IN
QL02158	H-substituted perfluoroalkyl (linear) sulfonic acids_ii, n=4		FC(F)(F)C([H])(F)C(F)(F)C(F)(F)C(F)(F)C(F)(F)S(=O)(O)=O		-7.48	0.68	2.16	P	IN
QL02159	H-substituted perfluoroalkyl (linear) sulfonic acids_ii, n=3		FC(F)(F)C([H])(F)C(F)(F)C(F)(F)C(F)(F)S(=O)(O)=O		-6.88	0.68	1.90	P	IN
QL02160	H-substituted perfluoroalkyl (linear) sulfonic acids_ii, n=2		FC(F)(F)C([H])(F)C(F)(F)C(F)(F)S(=O)(O)=O		-6.58	0.67	1.81	P	IN
QL02161	H-substituted perfluoroalkyl (linear) sulfonic acids_ii, n=1		FC(F)(F)C([H])(F)C(F)(F)S(=O)(O)=O		-5.73	0.67	1.46	P	IN
QL02162	Perfluoro-3-ethyl-2,4-dimethyl-2-pentene	30320-26-4	FC(F)(F)C(=C(C(F)(F)C(F)(F)F)C(F)(C(F)(F)F)C(F)(F)F)C(F)(F)F		-6.01	0.67	1.52	P	IN
QL02163	1,1-Bis(trifluoromethyl)ethene	382-10-5	FC(F)(F)C(=C)C(F)(F)F		-4.98	0.65	1.30	P	IN
QL02164	5-Fluoro-5,7,7-tris(trifluoromethyl)-6-(2,2,2-trifluoro-1-(trifluoromethyl)ethylidene)-1,4-dioxepane	94333-56-9	FC(F)(F)C(=C1C(F)(OCCOC1(C(F)(F)F)C(F)(F)F)C(F)(F)F)C(F)(F)F		-5.49	0.66	1.47	P	IN
QL02165	3-Trifluoromethylperfluoro-2-pentanone	93830-92-3	FC(F)(F)C(=O)C(F)(C(F)(F)F)C(F)(F)C(F)(F)F		-6.50	0.67	1.75	P	IN
QL02166	1,1,1,3,4,4,4-Heptafluoro-3-(trifluoromethyl)butan-2-one	756-12-7	FC(F)(F)C(=O)C(F)(C(F)(F)F)C(F)(F)F		-5.62	0.67	1.37	P	IN
QL02167	1,1,1,3,3,4,5,5,5-Nonafluoro-4-(trifluoromethyl)pentan-2-one	73041-05-1	FC(F)(F)C(=O)C(F)(F)C(F)(C(F)(F)F)C(F)(F)F		-6.41	0.67	1.71	P	IN
QL02168	ketone perfluoroalkyl (linear) carboxylic acids, n=8		FC(F)(F)C(=O)C(F)(F)C(F)(F)C(F)(F)C(F)(F)C(F)(F)C(F)(F)C(F)(F)C(F)(F)S(=O)(O)=O		-8.71	0.69	2.69	P	IN
QL02169	ketone perfluoroalkyl (linear) carboxylic acids, n=7		FC(F)(F)C(=O)C(F)(F)C(F)(F)C(F)(F)C(F)(F)C(F)(F)C(F)(F)C(F)(F)S(=O)(O)=O		-8.34	0.69	2.51	P	IN
QL02170	ketone perfluoroalkyl (linear) carboxylic acids, n=5		FC(F)(F)C(=O)C(F)(F)C(F)(F)C(F)(F)C(F)(F)C(F)(F)S(=O)(O)=O		-7.70	0.68	2.24	P	IN
QL02171	ketone perfluoroalkyl (linear) carboxylic acids, n=4		FC(F)(F)C(=O)C(F)(F)C(F)(F)C(F)(F)C(F)(F)S(=O)(O)=O		-7.48	0.68	2.16	P	IN
QL02172	ketone perfluoroalkyl (linear) carboxylic acids, n=3		FC(F)(F)C(=O)C(F)(F)C(F)(F)C(F)(F)S(=O)(O)=O		-6.88	0.68	1.90	P	IN
QL02173	2,2,2-Trifluoro-1-[4-(tridecafluorohexyl)phenyl]ethan-1-one	171183-94-1	FC(F)(F)C(=O)c1ccc(cc1)C(F)(F)C(F)(F)C(F)(F)C(F)(F)C(F)(F)C(F)(F)F		-8.23	0.66	2.75	P	IN
QL02174	1,1,1,5,5,6,6,7,7,8,8,9,9,10,10,10-hexadecafluorodecane-2,4-dione	147874-76-8	FC(F)(F)C(=O)CC(=O)C(F)(F)C(F)(F)C(F)(F)C(F)(F)C(F)(F)C(F)(F)F		-8.01	0.67	2.51	P	IN
QL02175	1,1,1,5,5,6,6,7,7,8,8,8-dodecafluorooctane-2,4-dione	261503-40-6	FC(F)(F)C(=O)CC(=O)C(F)(F)C(F)(F)C(F)(F)C(F)(F)F		-7.28	0.67	2.24	P	IN
QL02176	3H,3H-Perfluoroheptane-2,4-dione	20583-66-8	FC(F)(F)C(=O)CC(=O)C(F)(F)C(F)(F)C(F)(F)F		-6.95	0.66	2.15	P	IN
QL02177	3H,3H-Perfluoro-2,4-hexanedione	20825-07-4	FC(F)(F)C(=O)CC(=O)C(F)(F)C(F)(F)F		-6.23	0.65	1.88	P	IN
QL02178	3-(Perfluorohexyl)-1-(trifluoroacetyl)-5-(trifluoromethyl)-1H-pyrazole	261778-38-5	FC(F)(F)C(=O)N1N=C(C=C1C(F)(F)F)C(F)(F)C(F)(F)C(F)(F)C(F)(F)C(F)(F)C(F)(F)F		-8.26	0.67	2.67	P	IN
QL02179	2,2,3,3,4,4,4-Heptafluorobutyl trifluoroacetate	1365808-09-8	FC(F)(F)C(=O)OCC(F)(F)C(F)(F)C(F)(F)F		-6.82	0.66	2.11	P	IN
QL02180	2,2,3,3,4,4,4-Heptafluoro-N-[(trifluoroacetyl)oxy]butanamide	88326-80-1	FC(F)(F)C(=O)ONC(=O)C(F)(F)C(F)(F)C(F)(F)F		-6.93	0.65	2.23	P	IN
QL02181	1,1,1,3,3,5,5,5-Octafluoro-2,2,4,4-tetrakis(trifluoromethyl)pentane	62375-54-6	FC(F)(F)C(C(F)(F)F)(C(F)(F)F)C(F)(F)C(C(F)(F)F)(C(F)(F)F)C(F)(F)F		-6.08	0.68	1.46	P	IN

QL02182	Perfluoro-2,2,5,5-tetramethylhexane	71076-47-6	FC(F)(F)C(C(F)(F)F)(C(F)(F)F)C(F)(F)C(F)(F)C(C(F)(F)F)(C(F)(F)F)C(F)(F)F		-6.45	0.68	1.62	P	IN
QL02183	2,2-bis(Trifluoromethyl)perfluoropropane	374-51-6	FC(F)(F)C(C(F)(F)F)(C(F)(F)F)C(F)(F)F		-5.24	0.67	1.09	P	IN
QL02184	Perfluoro tert-butylcyclohexane	84808-64-0	FC(F)(F)C(C(F)(F)F)(C(F)(F)F)C1(F)C(F)(F)C(F)(F)C(F)(F)C(F)(F)C1(F)F		-7.25	0.69	1.88	P	IN
QL02185	Perfluorodecahydro-1-t-butylnaphthalene	118914-94-6	FC(F)(F)C(C(F)(F)F)(C(F)(F)F)C1(F)C(F)(F)C(F)(F)C(F)(F)C2(F)C(F)(F)C(F)(F)C(F)(F)C(F)(F)C12F		-7.62	0.70	2.01	P	IN
QL02186	Perfluorodecahydro-2-t-butylnaphthalene	119107-97-0	FC(F)(F)C(C(F)(F)F)(C(F)(F)F)C1(F)C(F)(F)C(F)(F)C2(F)C(F)(F)C(F)(F)C(F)(F)C(F)(F)C2(F)C1(F)F		-8.04	0.70	2.23	P	IN
QL02187	2H-Perfluoro(2-methylpentane)	30320-28-6	FC(F)(F)C(C(F)(F)F)C(F)(F)C(F)(F)C(F)(F)F		-6.38	0.67	1.71	P	IN
QL02188	(1,1,1,5,5,6,6,7,7,7-Decafluorohept-3-yn-2-ylidene)cyclopentane	139226-05-4	FC(F)(F)C(C#CC(F)(F)C(F)(F)C(F)(F)F)=C1CCCC1		-6.32	0.64	2.13	P	IN
QL02189	Hexafluoro-2,2,3,3-tetrachlorobutane	375-34-8	FC(F)(F)C(Cl)(Cl)C(Cl)(Cl)C(F)(F)F		-6.18	0.68	1.45	P	IN
QL02190	1,2-Dibromohexafluoropropane	661-95-0	FC(F)(F)C(F)(Br)C(F)(F)Br		-5.80	0.68	1.32	P	IN
QL02191	Bis(perfluoroisopropyl)ketone	813-44-5	FC(F)(F)C(F)(C(=O)C(F)(C(F)(F)F)C(F)(F)F)C(F)(F)F		-6.27	0.68	1.58	P	IN
QL02192	1,1,1,2,4,4,5,5-Octafluoro-5-iodo-2-(trifluoromethyl)pentan-3-one	93822-96-9	FC(F)(F)C(F)(C(=O)C(F)(F)C(F)(F)I)C(F)(F)F		-6.44	0.68	1.68	P	IN
QL02193	1-Propanone, 2,3,3,3-tetrafluoro-1-phenyl-2-(trifluoromethyl)-	56112-35-7	FC(F)(F)C(F)(C(=O)C1=CC=CC=C1)C(F)(F)F		-5.56	0.63	1.88	P	OUT
QL02194	1-(4-Chlorophenyl)-2,3,3,3-tetrafluoro-2-(trifluoromethyl)propan-1-one	56041-73-7	FC(F)(F)C(F)(C(=O)c1ccc(Cl)cc1)C(F)(F)F		-6.24	0.64	2.12	P	IN
QL02195	Benzyl 2,3,3,3-tetrafluoro-2-(trifluoromethyl)propanoate	38392-11-9	FC(F)(F)C(F)(C(=O)OCc1ccccc1)C(F)(F)F		-5.86	0.62	2.16	P	OUT
QL02196	Bis[2,3,3,3-tetrafluoro-1-oxo-2-(trifluoromethyl)propyl] peroxide	111632-55-4	FC(F)(F)C(F)(C(=O)OOC(=O)C(F)(C(F)(F)F)C(F)(F)F)C(F)(F)F		-6.67	0.66	1.99	P	IN
QL02197	2-bromo-3,4,4,4-tetrafluoro-3-(trifluoromethyl)but-1-ene	234443-24-4	FC(F)(F)C(F)(C(Br)=C)C(F)(F)F		-5.20	0.66	1.24	P	IN
QL02198	Perfluoro-2,3-dimethylbutane	354-96-1	FC(F)(F)C(F)(C(F)(F)F)C(F)(C(F)(F)F)C(F)(F)F		-5.80	0.68	1.30	P	IN
QL02199	Heptane, 1,1,1,2,3,3,4,5,5,6,7,7,7-tridecafluoro-2,4,6-tris(trifluoromethyl)-	84808-63-9	FC(F)(F)C(F)(C(F)(F)F)C(F)(F)C(F)(C(F)(F)F)C(F)(F)C(F)(C(F)(F)F)C(F)(F)F		-7.38	0.68	2.03	P	IN
QL02200	Perfluoro-2,7-dimethyloctane	3021-63-4	FC(F)(F)C(F)(C(F)(F)F)C(F)(F)C(F)(F)C(F)(F)C(F)(F)C(F)(C(F)(F)F)C(F)(F)F		-7.66	0.69	2.14	P	IN
QL02201	Perfluoro-2,9-dimethyldecane	103188-55-2	FC(F)(F)C(F)(C(F)(F)F)C(F)(F)C(F)(F)C(F)(F)C(F)(F)C(F)(F)C(F)(F)C(F)(C(F)(F)F)C(F)(F)F		-8.28	0.69	2.43	P	IN
QL02202	1,1,1,2,3,3,4,4,5,5,6,6,7,7,8,8,9,9,10,10,11,12,12,12-tetracosafluoro-2,11-bis(trifluoromethyl)dodecane	118694-32-9	FC(F)(F)C(F)(C(F)(F)F)C(F)(F)C(F)(F)C(F)(F)C(F)(F)C(F)(F)C(F)(F)C(F)(F)C(F)(F)C(F)(C(F)(F)F)C(F)(F)F		-8.80	0.69	2.67	P	OUT
QL02203	1,1,1,2,3,3,4,4,5,5,6,6,7,7,8,8,9,9,10,10-Icosafluoro-12-iodo-2-(trifluoromethyl)dodecane	242142-80-9	FC(F)(F)C(F)(C(F)(F)F)C(F)(F)C(F)(F)C(F)(F)C(F)(F)C(F)(F)C(F)(F)C(F)(F)C(F)(F)CCI		-8.49	0.68	2.73	P	IN
QL02204	2-(Perfluoro-7-methyloctyl)ethyl iodide	40678-31-7	FC(F)(F)C(F)(C(F)(F)F)C(F)(F)C(F)(F)C(F)(F)C(F)(F)C(F)(F)C(F)(F)CCI		-7.90	0.67	2.50	P	IN
QL02205	2-[2,2,3,3,4,4,5,5,6,7,7,7-dodecafluoro-6-(trifluoromethyl)heptyl]oxirane	24564-77-0	FC(F)(F)C(F)(C(F)(F)F)C(F)(F)C(F)(F)C(F)(F)C(F)(F)CC1CO1		-7.40	0.67	2.20	P	IN
QL02206	1,1,1,2,3,3,4,4,5,5,6,6-dodecafluoro-8-iodo-2-(trifluoromethyl)octane	18017-20-4	FC(F)(F)C(F)(C(F)(F)F)C(F)(F)C(F)(F)C(F)(F)C(F)(F)CCI		-7.17	0.66	2.22	P	IN
QL02207	3,3,4,4,5,5,6,6,7,8,8,8-Dodecafluoro-7-(trifluoromethyl)octyl prop-2-enoate	50836-65-2	FC(F)(F)C(F)(C(F)(F)F)C(F)(F)C(F)(F)C(F)(F)C(F)(F)CCOC(=O)C=C		-7.76	0.65	2.68	P	IN
QL02208	Perfluoroisoheptyl Iodide	3486-08-6	FC(F)(F)C(F)(C(F)(F)F)C(F)(F)C(F)(F)C(F)(F)C(F)(F)I		-7.05	0.69	1.85	P	IN
QL02209	3,3,4,4,5,6,6,6-Octafluoro-5-(trifluoromethyl)hex-1-ene	139060-76-7	FC(F)(F)C(F)(C(F)(F)F)C(F)(F)C(F)(F)C=C		-6.37	0.67	1.77	P	IN
QL02210	3-(Perfluoro-3-methylbutyl)-1,2-propenoxide	54009-81-3	FC(F)(F)C(F)(C(F)(F)F)C(F)(F)C(F)(F)CC1CO1		-6.49	0.67	1.85	P	IN
QL02211	[3,3,4,4,5,6,6,6-octafluoro-5-(trifluoromethyl)hexyl] Prop-2-enoate	86217-01-8	FC(F)(F)C(F)(C(F)(F)F)C(F)(F)C(F)(F)CCOC(=O)C=C		-6.84	0.64	2.38	P	IN
QL02212	Perfluoro-3-methylbutyl iodide	1514-90-5	FC(F)(F)C(F)(C(F)(F)F)C(F)(F)C(F)(F)I		-6.00	0.68	1.38	P	IN
QL02213	Perfluorodecahydro-1-isobutylnaphthalene	118945-65-6	FC(F)(F)C(F)(C(F)(F)F)C(F)(F)C1(F)C(F)(F)C(F)(F)C(F)(F)C2(F)C(F)(F)C(F)(F)C(F)(F)C(F)(F)C12F		-7.97	0.70	2.15	P	IN
QL02214	Perfluorodecahydro-2-iso-butylnaphthalene	119141-87-6	FC(F)(F)C(F)(C(F)(F)F)C(F)(F)C1(F)C(F)(F)C(F)(F)C2(F)C(F)(F)C(F)(F)C(F)(F)C(F)(F)C2(F)C1(F)F		-8.51	0.70	2.42	P	IN
QL02215	Perflisobutane	354-92-7	FC(F)(F)C(F)(C(F)(F)F)C(F)(F)F		-5.53	0.68	1.20	P	IN
QL02216	Propane, 2-(difluoroiodomethyl)-1,1,1,2,3,3,3-heptafluoro-	1542-18-3	FC(F)(F)C(F)(C(F)(F)F)C(F)(F)I		-5.53	0.68	1.20	P	IN
QL02217	1,1,2,3,3,3-Hexafluoro-2-(trifluoromethyl)propane-1-sulphonic anhydride	93894-52-1	FC(F)(F)C(F)(C(F)(F)F)C(F)(F)S(=O)(=O)OS(=O)(=O)C(F)(F)C(F)(C(F)(F)F)C(F)(F)F		-7.80	0.68	2.27	P	IN
QL02218	Undecafluoro(1,1,1,2,3,3,3-heptafluoropropan-2-yl)cyclohexane	423-02-9	FC(F)(F)C(F)(C(F)(F)F)C1(F)C(F)(F)C(F)(F)C(F)(F)C(F)(F)C1(F)F		-7.40	0.69	1.91	P	IN
QL02219	3,4,4,4-tetrafluoro-3-(trifluoromethyl)but-1-yne	24690-53-7	FC(F)(F)C(F)(C#C)C(F)(F)F		-5.05	0.65	1.28	P	IN

QL02220	2,3,3,3-Tetrafluoro-2-(trifluoromethyl)propanenitrile	42532-60-5	FC(F)(F)C(F)(C#N)C(F)(F)F		-5.05	0.65	1.28	P	IN
QL02221	2-Vinylperfluoropropane	88562-41-8	FC(F)(F)C(F)(C=C)C(F)(F)F		-5.05	0.65	1.28	P	IN
QL02223	3,4,4,4-Tetrafluoro-1-iodo-3-(trifluoromethyl)but-1-ene	24716-85-6	FC(F)(F)C(F)(C=CI)C(F)(F)F		-4.78	0.63	1.42	P	IN
QL02224	1,4-Bis(heptafluoroisopropyl)benzene	51114-12-6	FC(F)(F)C(F)(C1=CC=C(C=C1)C(F)(C(F)(F)F)C(F)(F)F)C(F)(F)F		-6.65	0.66	1.99	P	IN
QL02225	AGN-PC-0NHL39	40161-59-9	FC(F)(F)C(F)(C1=CC=C(I)C=C1)C(F)(F)F		-5.86	0.64	1.90	P	IN
QL02226	6-(1,1,1,2,3,3,3-Heptafluoropropan-2-yl)-3-{[(pyridin-3-yl)methyl]amino}-3,4-dihydroquinazolin-2(1H)-one	337457-78-0	FC(F)(F)C(F)(C1=CC2=C(NC(=O)N(C2)NCC2=CN=CC=C2)C=C1)C(F)(F)F		-6.84	0.62	2.72	P	OUT
QL02227	3-Chloro-5-(1,1,1,2,3,3,3-heptafluoropropan-2-yl)pyridin-2(1H)-one	1771743-43-1	FC(F)(F)C(F)(C1=CNC(=O)C(Cl)=C1)C(F)(F)F		-6.01	0.64	1.90	P	IN
QL02228	5-(1,1,1,2,3,3,3-Heptafluoropropan-2-yl)pyrimidine-2,4(1H,3H)-dione	80384-59-4	FC(F)(F)C(F)(C1=CNC(=O)NC1=O)C(F)(F)F		-5.97	0.64	1.93	P	IN
QL02229	2-(1,1,1,2,3,3,3-Heptafluoropropan-2-yl)pyridine-4-carbonitrile	929211-36-9	FC(F)(F)C(F)(c1cc(ccn1)C#N)C(F)(F)F		-5.45	0.63	1.88	P	OUT
QL02230	2-Bromo-1,3-dichloro-5-(1,1,1,2,3,3,3-heptafluoropropan-2-yl)benzene	353272-59-0	FC(F)(F)C(F)(c1cc(Cl)c(Br)c(Cl)c1)C(F)(F)F		-6.09	0.65	1.90	P	IN
QL02231	1-Bromo-4-(1,1,1,2,3,3,3-heptafluoropropan-2-yl)benzene	2396-23-8	FC(F)(F)C(F)(c1ccc(Br)cc1)C(F)(F)F		-5.86	0.64	1.90	P	IN
QL02232	4-(1,1,1,2,3,3,3-Heptafluoropropan-2-yl)benzaldehyde	875306-42-6	FC(F)(F)C(F)(c1ccc(C=O)cc1)C(F)(F)F		-6.03	0.63	2.15	P	OUT
QL02233	1-(Chloromethyl)-4-(1,1,1,2,3,3,3-heptafluoropropan-2-yl)benzene	75462-58-7	FC(F)(F)C(F)(c1ccc(CCl)cc1)C(F)(F)F		-6.03	0.63	2.15	P	OUT
QL02234	2-Chloro-5-(1,1,1,2,3,3,3-heptafluoropropan-2-yl)pyridine	1807450-20-9	FC(F)(F)C(F)(c1ccc(Cl)nc1)C(F)(F)F		-5.86	0.64	1.90	P	IN
QL02235	2-(1,1,1,2,3,3,3-Heptafluoropropan-2-yl)naphthalene	51114-14-8	FC(F)(F)C(F)(c1ccc2ccccc2c1)C(F)(F)F		-6.05	0.63	2.12	P	OUT
QL02236	5-(1,1,1,2,3,3,3-Heptafluoropropan-2-yl)-1,3-dihydro-2H-benzimidazol-2-one	1433216-51-3	FC(F)(F)C(F)(c1ccc2NC(=O)Nc2c1)C(F)(F)F		-6.33	0.64	2.12	P	IN
QL02237	6-(1,1,1,2,3,3,3-Heptafluoropropan-2-yl)-1,3-benzoxazol-2(3H)-one	1248393-88-5	FC(F)(F)C(F)(c1ccc2NC(=O)Oc2c1)C(F)(F)F		-6.33	0.64	2.12	P	IN
QL02238	6-(1,1,1,2,3,3,3-Heptafluoropropan-2-yl)-1,3-benzothiazol-2(3H)-one	1433216-60-4	FC(F)(F)C(F)(c1ccc2NC(=O)Sc2c1)C(F)(F)F		-6.33	0.64	2.12	P	IN
QL02239	5-(1,1,1,2,3,3,3-Heptafluoropropan-2-yl)-2,3-dihydro-1H-indole	273735-78-7	FC(F)(F)C(F)(c1ccc2NCCc2c1)C(F)(F)F		-5.76	0.64	1.87	P	IN
QL02240	5-(1,1,1,2,3,3,3-Heptafluoropropan-2-yl)-1H-indazole	1433216-39-7	FC(F)(F)C(F)(c1ccc2Nncc2c1)C(F)(F)F		-5.76	0.64	1.87	P	IN
QL02241	6-(1,1,1,2,3,3,3-Heptafluoropropan-2-yl)-1H-benzotriazole	1433216-34-2	FC(F)(F)C(F)(c1ccc2nnNc2c1)C(F)(F)F		-5.76	0.64	1.87	P	IN
QL02242	1-Bromo-3-(1,1,1,2,3,3,3-heptafluoropropan-2-yl)benzene	2396-22-7	FC(F)(F)C(F)(c1cccc(Br)c1)C(F)(F)F		-5.44	0.64	1.71	P	IN
QL02243	(1,1,1,2,3,3,3-Heptafluoropropan-2-yl)benzene	378-34-7	FC(F)(F)C(F)(c1ccccc1)C(F)(F)F		-5.35	0.63	1.75	P	OUT
QL02244	1-(1,1,1,2,3,3,3-Heptafluoropropan-2-yl)-2-iodocyclohexane	4316-00-1	FC(F)(F)C(F)(C1CCCCC1I)C(F)(F)F		-5.45	0.64	1.74	P	IN
QL02245	2-Chloro-3-(1,1,1,2,3,3,3-heptafluoropropan-2-yl)pyridine	1816284-43-1	FC(F)(F)C(F)(c1cccnc1Cl)C(F)(F)F		-5.45	0.64	1.74	P	IN
QL02246	2-Chloro-4-(1,1,1,2,3,3,3-heptafluoropropan-2-yl)pyridine	1816286-71-1	FC(F)(F)C(F)(c1ccnc(Cl)c1)C(F)(F)F		-5.44	0.64	1.71	P	IN
QL02247	3-Bromo-5-(1,1,1,2,3,3,3-heptafluoropropan-2-yl)pyridine	1816285-38-7	FC(F)(F)C(F)(c1cncc(Br)c1)C(F)(F)F		-5.44	0.64	1.71	P	IN
QL02248	2,4,6-Tris(1,1,1,2,3,3,3-heptafluoropropan-2-yl)-1,3,5-triazine	10271-37-1	FC(F)(F)C(F)(c1nc(nc(n1)C(F)(C(F)(F)F)C(F)(F)F)C(F)(C(F)(F)F)C(F)(F)F)C(F)(F)F		-6.44	0.67	1.73	P	IN
QL02249	2-(1,1,1,2,3,3,3-Heptafluoropropan-2-yl)-1H-imidazole	81790-00-3	FC(F)(F)C(F)(c1Nccn1)C(F)(F)F		-4.90	0.64	1.40	P	IN
QL02250	5-(1,1,1,2,3,3,3-Heptafluoropropan-2-yl)-1H-imidazole	81790-01-4	FC(F)(F)C(F)(c1Ncnc1)C(F)(F)F		-4.90	0.64	1.40	P	IN
QL02251	3,4,4,4-Tetrafluoro-1-phenyl-3-(trifluoromethyl)butan-1-one	82959-21-5	FC(F)(F)C(F)(CC(=O)c1ccccc1)C(F)(F)F		-5.90	0.63	2.10	P	OUT
QL02252	3,4,4,4-Tetrafluoro-3-(trifluoromethyl)butanal	131118-41-7	FC(F)(F)C(F)(CC=O)C(F)(F)F		-4.78	0.63	1.42	P	IN
QL02253	[2,3,3,3-Tetrafluoro-2-(trifluoromethyl)propyl]oxirane	74328-57-7	FC(F)(F)C(F)(CC1CO1)C(F)(F)F		-5.25	0.65	1.38	P	IN
QL02254	4-Bromo-1,1,1,2-tetrafluoro-2-(trifluoromethyl)butane	1274903-97-7	FC(F)(F)C(F)(CCBr)C(F)(F)F		-4.78	0.63	1.42	P	IN
QL02255	2,4-Bis[3,4,4,4-tetrafluoro-3-(trifluoromethyl)butyl]-1lambda~6~,3lambda~6~-dithietane-1,1,3,3-tetrone	1346175-94-7	FC(F)(F)C(F)(CCC1S(=O)(=O)C(CCC(F)(C(F)(F)F)C(F)(F)F)S1(=O)=O)C(F)(F)F		-7.74	0.67	2.44	P	IN

QL02256	1,1,1,2-Tetrafluoro-6-iodo-2-(trifluoromethyl)hexane	99324-97-7	FC(F)(F)C(F)(CCCCI)C(F)(F)F		-5.03	0.62	1.80	P	OUT
QL02257	2-(2-Iodoethyl)perfluoropropane	99324-96-6	FC(F)(F)C(F)(CCI)C(F)(F)F		-4.78	0.63	1.42	P	IN
QL02258	4-(Ethenyloxy)-1,1,1,2-tetrafluoro-2-(trifluoromethyl)butane	90999-86-3	FC(F)(F)C(F)(CCOC=C)C(F)(F)F		-5.03	0.62	1.80	P	OUT
QL02259	2,3-Dichloro-1,1,1,2,3,4,5,5,5-nonafluoro-4-(trifluoromethyl)pentane	85720-79-2	FC(F)(F)C(F)(Cl)C(F)(Cl)C(F)(C(F)(F)F)C(F)(F)F		-6.74	0.68	1.72	P	IN
QL02260	2,3-Dichlorooctafluorobutane	NOCAS_870512	FC(F)(F)C(F)(Cl)C(F)(Cl)C(F)(F)F		-6.18	0.68	1.45	P	IN
QL02261	1,2-Dichlorohexafluoropropane	661-97-2	FC(F)(F)C(F)(Cl)C(F)(F)Cl		-5.80	0.68	1.32	P	IN
QL02262	2λ5,4λ5,6λ5-1,3,5,2,4,6-Triazatriphosphorine, 2,2,4,4,6,6-hexakis[2,3,3,3-tetrafluoro-2-[1,1,2,3,3,3-hexafluoro-2-(1,1,2,2,3,3,3-heptafluoropropoxy)propoxy]propoxy]-	1383437-42-0	FC(F)(F)C(F)(COP1(OCC(F)(OC(F)(F)C(F)(OC(F)(F)C(F)(F)C(F)(F)F)C(F)(F)F)C(F)(F)F)=NP(OCC(F)(OC(F)(F)C(F)(OC(F)(F)C(F)(F)C(F)(F)F)C(F)(F)F)C(F)(F)F)(OCC(F)(OC(F)(F)C(F)(OC(F)(F)C(F)(F)C(F)(F)F)C(F)(F)F)C(F)(F)F)=NP(OCC(F)(OC(F)(F)C(F)(OC(F)(F)C(F)(F)C(F)(F)F)C(F)(F)F)C(F)(F)F)(OCC(F)(OC(F)(F)C(F)(OC(F)(F)C(F)(F)C(F)(F)F)C(F)(F)F)C(F)(F)F)=N1)OC(F)(F)C(F)(OC(F)(F)C(F)(F)C(F)(F)F)C(F)(F)F		-9.84	0.68	3.41	P	OUT
QL02263	Perfluoro-2-methyl-3-(propan-2-yl)pentan-3-yl	93683-27-3	FC(F)(F)C(F)(F)[C](C(F)(C(F)(F)F)C(F)(F)F)C(F)(C(F)(F)F)C(F)(F)F		-6.25	0.68	1.53	P	IN
QL02264	Perfluoro-2-methyl-3-pentanone	756-13-8	FC(F)(F)C(F)(F)C(=O)C(F)(C(F)(F)F)C(F)(F)F		-6.44	0.68	1.68	P	IN
QL02265	1,1,1,2,2,4,4,5,5,5-Decafluoro-3-pentanone	684-32-2	FC(F)(F)C(F)(F)C(=O)C(F)(F)C(F)(F)F		-6.59	0.68	1.76	P	IN
QL02266	1-(2,2,3,3,3-Pentafluoro-1-oxopropyl)-1H-imidazole	71735-32-5	FC(F)(F)C(F)(F)C(=O)N1C=CN=C1		-5.39	0.63	1.81	P	OUT
QL02267	Pentafluoropropanoic anhydride	NOCAS_870515	FC(F)(F)C(F)(F)C(=O)OC(=O)C(F)(F)C(F)(F)F		-6.91	0.66	2.09	P	IN
QL02268	Perfluoro(3-ethyl-2,3-dimethylpentane)	50285-19-3	FC(F)(F)C(F)(F)C(C(F)(F)F)(C(F)(F)C(F)(F)F)C(F)(C(F)(F)F)C(F)(F)F		-6.49	0.68	1.62	P	IN
QL02269	3,3-Bis(trifluoromethyl)perfluoropentane	36591-89-6	FC(F)(F)C(F)(F)C(C(F)(F)F)(C(F)(F)F)C(F)(F)C(F)(F)F		-6.49	0.68	1.63	P	IN
QL02270	3,3-Dichloro-1,1,1,2,2-pentafluoropropane	422-56-0	FC(F)(F)C(F)(F)C(Cl)Cl		-5.29	0.66	1.36	P	IN
QL02271	Perfluoro dimethylethylpentane	50285-18-2	FC(F)(F)C(F)(F)C(F)(C(F)(C(F)(F)F)C(F)(F)F)C(F)(C(F)(F)F)C(F)(F)F		-6.45	0.68	1.58	P	IN
QL02272	Perfluoro-2-methyl-3-ethylpentane	354-97-2	FC(F)(F)C(F)(F)C(F)(C(F)(F)C(F)(F)F)C(F)(C(F)(F)F)C(F)(F)F		-6.61	0.68	1.64	P	IN
QL02273	Perfluoro-3,4-dimethylhexane	1735-48-4	FC(F)(F)C(F)(F)C(F)(C(F)(F)F)C(F)(C(F)(F)F)C(F)(F)C(F)(F)F		-6.87	0.68	1.78	P	IN
QL02274	Perfluoro-2,3-dimethylpentane	335-14-8	FC(F)(F)C(F)(F)C(F)(C(F)(F)F)C(F)(C(F)(F)F)C(F)(F)F		-6.66	0.68	1.69	P	IN
QL02275	1,1,1,2,2,3,4,4,5,5,5-Undecafluoro-3-(trifluoromethyl)pentane	865-71-4	FC(F)(F)C(F)(F)C(F)(C(F)(F)F)C(F)(F)C(F)(F)F		-6.74	0.68	1.72	P	IN
QL02276	Perfluoro-2-methylbutane	594-91-2	FC(F)(F)C(F)(F)C(F)(C(F)(F)F)C(F)(F)F		-6.00	0.68	1.38	P	IN
QL02277	Perfluorodecahydro-1-sec-butylnaphthalene	118945-64-5	FC(F)(F)C(F)(F)C(F)(C(F)(F)F)C1(F)C(F)(F)C(F)(F)C(F)(F)C2(F)C(F)(F)C(F)(F)C(F)(F)C(F)(F)C12F		-7.85	0.70	2.09	P	IN
QL02278	Perfluorodecahydro-2-sec-butylnaphthalene	119141-86-5	FC(F)(F)C(F)(F)C(F)(C(F)(F)F)C1(F)C(F)(F)C(F)(F)C2(F)C(F)(F)C(F)(F)C(F)(F)C(F)(F)C2(F)C1(F)F		-8.42	0.70	2.38	P	IN
QL02279	(1S,2R)-1-(Heptafluoropropyl)-2-iodocyclohexane	7589-44-8	FC(F)(F)C(F)(F)C(F)(F)[C@@H]1CCCC[C@H]1I		-5.87	0.64	1.90	P	IN
QL02280	CIS-1-IODO-2-(HEPTAFLUOROPROPYL)CYCLOHEXANE	7589-43-7	FC(F)(F)C(F)(F)C(F)(F)[C@H]1CCCC[C@H]1I		-5.87	0.64	1.90	P	IN
QL02281	42 Fluorotelomer unsaturated aldehyde	864551-39-3	FC(F)(F)C(F)(F)C(F)(F)/C(F)=C/C=O		-5.78	0.63	1.93	P	IN
QL02282	1-Bromoheptafluoropropane	422-85-5	FC(F)(F)C(F)(F)C(F)(F)Br		-5.80	0.68	1.32	P	IN
QL02283	Bis[1,1,1,3,3,4,4,5,5,5-decafluoro-2-(trifluoromethyl)pentan-2-yl]mercury	120674-92-2	FC(F)(F)C(F)(F)C(F)(F)C([Hg]C(C(F)(F)F)(C(F)(F)F)C(F)(F)C(F)(F)C(F)(F)F)(C(F)(F)F)C(F)(F)F		-7.50	0.68	2.12	P	IN
QL02284	N-Chloro-2,2,3,3,4,4,4-heptafluorobutanimidamide	13050-20-9	FC(F)(F)C(F)(F)C(F)(F)C(=N)NCl		-5.98	0.65	1.84	P	IN
QL02285	N'-(Benzenesulfonyl)-2,2,3,3,4,4,4-heptafluorobutanimidohydrazide	4454-57-3	FC(F)(F)C(F)(F)C(F)(F)C(=N)NNS(=O)(=O)c1ccccc1		-6.88	0.63	2.49	P	OUT
QL02286	2-Diazo-4,4,5,5,6,6,6-heptafluoro-1-phenylhexane-1,3-dione	872186-76-0	FC(F)(F)C(F)(F)C(F)(F)C(=O)C(=[N+]=[N-])C(=O)c1ccccc1		-6.42	0.62	2.45	P	OUT
QL02287	1,2-Pentanedione, 3,3,4,4,5,5,5-heptafluoro-1-phenyl-	69481-44-3	FC(F)(F)C(F)(F)C(F)(F)C(=O)C(=O)C1=CC=CC=C1		-6.36	0.63	2.26	P	OUT
QL02288	1-(1-Piperidinyl)-2-(triphenylphosphoranylidene)perfluorohexane-1,3-dione	148192-46-5	FC(F)(F)C(F)(F)C(F)(F)C(=O)C(C(=O)N1CCCCC1)=P(C1=CC=CC=C1)(C1=CC=CC=C1)C1=CC=CC=C1		-5.58	0.60	2.32	P	OUT
QL02289	1,1,1,2,4,4,5,5,6,6,6-Undecafluoro-2-(trifluoromethyl)-3-hexanone	813-45-6	FC(F)(F)C(F)(F)C(F)(F)C(=O)C(F)(C(F)(F)F)C(F)(F)F		-6.84	0.68	1.84	P	IN
QL02290	4,4,5,5,6,6,6-Heptafluoro-1-phenylhex-1-yn-3-one	67036-08-2	FC(F)(F)C(F)(F)C(F)(F)C(=O)C#CC1=CC=CC=C1		-6.46	0.62	2.42	P	OUT
QL02291	1-Anilino-4,4,5,5,6,6,6-heptafluorohex-1-en-3-one	34648-00-5	FC(F)(F)C(F)(F)C(F)(F)C(=O)C=CNC1=CC=CC=C1		-6.42	0.62	2.49	P	OUT

QL02292	1-(3,5-Dibromophenyl)-2,2,3,3,4,4,4-heptafluorobutan-1-one	79851-20-0	FC(F)(F)C(F)(F)C(F)(F)C(=O)C1=CC(Br)=CC(Br)=C1		-6.41	0.64	2.15	P	IN
QL02293	2,2,3,3,4,4,4-heptafluoro-1-phenylbutan-1-one	559-91-1	FC(F)(F)C(F)(F)C(F)(F)C(=O)C1=CC=CC=C1		-6.23	0.63	2.17	P	IN
QL02294	2-(2,2,3,3,4,4,4-Heptafluorobutanoyl)cyclopentan-1-one	141478-83-3	FC(F)(F)C(F)(F)C(F)(F)C(=O)C1CCCC1=O		-6.05	0.65	1.91	P	IN
QL02295	2-(2,2,3,3,4,4,4-Heptafluorobutanoyl)cyclohexan-1-one	37172-87-5	FC(F)(F)C(F)(F)C(F)(F)C(=O)C1CCCCC1=O		-6.31	0.64	2.15	P	IN
QL02296	5H,5H-Perfluoro-4,6-nonanedione	113116-18-0	FC(F)(F)C(F)(F)C(F)(F)C(=O)CC(=O)C(F)(F)C(F)(F)C(F)(F)F		-7.72	0.67	2.38	P	IN
QL02297	4,4''-Bis((perfluoropropyl)-1,3-dioxopropyl)-o-terphenyl-4'-sulfonyl chloride	200862-70-0	FC(F)(F)C(F)(F)C(F)(F)C(=O)CC(=O)C1=CC=C(C=C1)C1=C(C=C(C=C1)S(Cl)(=O)=O)C1=CC=C(C=C1)C(=O)CC(=O)C(F)(F)C(F)(F)C(F)(F)F		-8.95	0.64	3.48	P	OUT
QL02298	4,4,5,5,6,6,6-Heptafluoro-1-phenylhexane-1,3-dione	53580-21-5	FC(F)(F)C(F)(F)C(F)(F)C(=O)CC(=O)C1=CC=CC=C1		-6.58	0.63	2.43	P	OUT
QL02299	4,4,5,5,6,6,6-Heptafluoro-1-(furan-2-yl)hexane-1,3-dione	595-26-6	FC(F)(F)C(F)(F)C(F)(F)C(=O)CC(=O)C1=CC=CO1		-6.39	0.63	2.24	P	IN
QL02300	(Perfluorobutyryl)-2-thenoylmethane	559-94-4	FC(F)(F)C(F)(F)C(F)(F)C(=O)CC(=O)C1=CC=CS1		-6.39	0.63	2.24	P	IN
QL02301	3,3,4,4,5,5,5-Heptafluoro-1-phenylpentan-2-one	559-96-6	FC(F)(F)C(F)(F)C(F)(F)C(=O)CC1=CC=CC=C1		-6.25	0.63	2.25	P	OUT
QL02302	3,3,4,4,5,5,5-heptafluoro-1-pyridin-2-ylpentan-2-one	336-60-7	FC(F)(F)C(F)(F)C(F)(F)C(=O)CC1=CC=CC=N1		-6.25	0.63	2.25	P	OUT
QL02303	N,N-Dicyclohexyl-2,2,3,3,4,4,4-heptafluorobutanamide	386-84-5	FC(F)(F)C(F)(F)C(F)(F)C(=O)N(C1CCCCC1)C1CCCCC1		-5.75	0.61	2.21	P	OUT
QL02304	N,N-Dichloro-2,2,3,3,4,4,4-heptafluorobutanamide	86451-48-1	FC(F)(F)C(F)(F)C(F)(F)C(=O)N(Cl)Cl		-6.21	0.66	1.83	P	IN
QL02305	1-(Heptafluorobutyryl)imidazole	32477-35-3	FC(F)(F)C(F)(F)C(F)(F)C(=O)N1C=CN=C1		-5.95	0.64	1.92	P	IN
QL02306	N-Heptafluorobutyrylproline	56236-41-0	FC(F)(F)C(F)(F)C(F)(F)C(=O)N1CCC[C@H]1C(Cl)=O		-6.38	0.64	2.15	P	IN
QL02307	1-(2,3-Dihydro-1H-indol-1-yl)-2,2,3,3,4,4,4-heptafluoro-1-butanone	543708-46-9	FC(F)(F)C(F)(F)C(F)(F)C(=O)N1CCC2=CC=CC=C12		-6.44	0.64	2.24	P	IN
QL02308	2,2,3,3,4,4,4-heptafluoro-1-pyrrolidin-1-ylbutan-1-one	424-54-4	FC(F)(F)C(F)(F)C(F)(F)C(=O)N1CCCC1		-5.95	0.64	1.92	P	IN
QL02309	2,2,3,3,4,4,4-Heptafluoro-1-(piperidin-1-yl)butan-1-one	425-19-4	FC(F)(F)C(F)(F)C(F)(F)C(=O)N1CCCCC1		-6.23	0.63	2.17	P	IN
QL02310	1,1'-(Piperazine-1,4-diyl)bis(heptafluorobutan-1-one)	120219-50-3	FC(F)(F)C(F)(F)C(F)(F)C(=O)N1CCN(CC1)C(=O)C(F)(F)C(F)(F)C(F)(F)F		-7.97	0.66	2.69	P	IN
QL02311	1-{4-[(2H-1,3-Benzodioxol-5-yl)methyl]piperazin-1-yl}-2,2,3,3,4,4,4-heptafluorobutan-1-one	546063-97-2	FC(F)(F)C(F)(F)C(F)(F)C(=O)N1CCN(Cc2ccc3OCOc3c2)CC1		-7.39	0.62	2.89	P	OUT
QL02312	2,2,3,3,4,4,4-Heptafluoro-1-(piperazin-1-yl)butan-1-one	89245-13-6	FC(F)(F)C(F)(F)C(F)(F)C(=O)N1CCNCC1		-6.23	0.63	2.17	P	IN
QL02313	2,2,3,3,4,4,4-Heptafluoro-1-(morpholin-4-yl)butan-1-one	425-20-7	FC(F)(F)C(F)(F)C(F)(F)C(=O)N1CCOCC1		-6.23	0.63	2.17	P	IN
QL02314	N,N'-1,3-Phenylenebis(perfluorobutanamide)	524726-48-5	FC(F)(F)C(F)(F)C(F)(F)C(=O)NC1=CC(NC(=O)C(F)(F)C(F)(F)C(F)(F)F)=CC=C1		-8.03	0.65	2.83	P	IN
QL02315	Butanamide, N,N'-(1,1'-biphenyl)-4,4'-diylbis(2,2,3,3,4,4,4-heptafluoro-	75042-07-8	FC(F)(F)C(F)(F)C(F)(F)C(=O)NC1=CC=C(C=C1)C1=CC=C(NC(=O)C(F)(F)C(F)(F)C(F)(F)F)C=C1		-8.46	0.64	3.18	P	OUT
QL02316	N,N'-(Methylenedi-1,4-phenylene)bis(perfluorobutanamide)	124232-75-3	FC(F)(F)C(F)(F)C(F)(F)C(=O)NC1=CC=C(CC2=CC=C(NC(=O)C(F)(F)C(F)(F)C(F)(F)F)C=C2)C=C1		-8.56	0.64	3.27	P	OUT
QL02317	N,N'-1,4-Phenylenebis(perfluorobutanamide)	524726-49-6	FC(F)(F)C(F)(F)C(F)(F)C(=O)NC1=CC=C(NC(=O)C(F)(F)C(F)(F)C(F)(F)F)C=C1		-8.10	0.65	2.86	P	IN
QL02318	AI 3-18011	336-61-8	FC(F)(F)C(F)(F)C(F)(F)C(=O)NC1=CC=CC=C1		-6.25	0.63	2.25	P	OUT
QL02319	Thiobenzyl N-heptafluorobutyrylanthranilate	82422-25-1	FC(F)(F)C(F)(F)C(F)(F)C(=O)NC1=CC=CC=C1C(=O)SCC1=CC=CC=C1		-7.00	0.61	2.89	P	OUT
QL02320	N-2-Pyridinylperfluorobutanamide	283172-09-8	FC(F)(F)C(F)(F)C(F)(F)C(=O)NC1=CC=CC=N1		-6.25	0.63	2.25	P	OUT
QL02321	2,2,3,3,4,4,4-Heptafluoro-N-(2-oxo-2,3-dihydropyrimidin-4-yl)butanamide	94720-20-4	FC(F)(F)C(F)(F)C(F)(F)C(=O)NC1=CC=NC(=O)N1		-6.38	0.63	2.24	P	OUT
QL02322	(2-Naphthalenyl)perfluorobutanamide	582-00-3	FC(F)(F)C(F)(F)C(F)(F)C(=O)NC1=CC2=CC=CC=C2C=C1		-6.78	0.63	2.52	P	OUT
QL02323	N-(2,6-Dichlorophenyl)-2,2,3,3,4,4,4-heptafluorobutanamide	121806-69-7	FC(F)(F)C(F)(F)C(F)(F)C(=O)Nc1c(Cl)cccc1Cl		-6.42	0.64	2.20	P	IN
QL02324	2,2,3,3,4,4,4-Heptafluoro-N-(2,4,5-trichlorophenyl)butanamide	121806-70-0	FC(F)(F)C(F)(F)C(F)(F)C(=O)Nc1cc(Cl)c(Cl)cc1Cl		-6.96	0.64	2.41	P	IN
QL02325	N-(2,5-Dichlorophenyl)-2,2,3,3,4,4,4-heptafluorobutanamide	121806-67-5	FC(F)(F)C(F)(F)C(F)(F)C(=O)Nc1cc(Cl)ccc1Cl		-6.48	0.64	2.23	P	IN
QL02326	N-(4-Bromophenyl)-2,2,3,3,4,4,4-heptafluorobutanamide	308-67-8	FC(F)(F)C(F)(F)C(F)(F)C(=O)Nc1ccc(Br)cc1		-6.81	0.64	2.44	P	OUT
QL02327	N-(4-Chlorophenyl)-2,2,3,3,4,4,4-heptafluorobutanamide	79497-98-6	FC(F)(F)C(F)(F)C(F)(F)C(=O)Nc1ccc(Cl)cc1		-6.81	0.64	2.44	P	OUT
QL02328	N-(2,4-Dichlorophenyl)-2,2,3,3,4,4,4-heptafluorobutanamide	121806-66-4	FC(F)(F)C(F)(F)C(F)(F)C(=O)Nc1ccc(Cl)cc1Cl		-6.85	0.64	2.42	P	IN

QL02329	N-(3-Chlorophenyl)-2,2,3,3,4,4,4-heptafluorobutanamide	106376-36-7	FC(F)(F)C(F)(F)C(F)(F)C(=O)Nc1cccc(Cl)c1		-6.38	0.63	2.24	P	OUT
QL02330	N-(2,3-Dichlorophenyl)-2,2,3,3,4,4,4-heptafluorobutanamide	121806-68-6	FC(F)(F)C(F)(F)C(F)(F)C(=O)Nc1cccc(Cl)c1Cl		-6.49	0.64	2.23	P	IN
QL02331	N-(9,10-Dioxo-9,10-dihydroanthracen-1-yl)-2,2,3,3,4,4,4-heptafluorobutanamide	162441-56-7	FC(F)(F)C(F)(F)C(F)(F)C(=O)Nc1cccc2C(=O)c3ccccc3C(=O)c12		-7.08	0.63	2.67	P	OUT
QL02332	N-Cyclohexyl-2,2,3,3,4,4,4-heptafluorobutanamide	56663-38-8	FC(F)(F)C(F)(F)C(F)(F)C(=O)NC1CCCCC1		-6.25	0.63	2.25	P	OUT
QL02333	N-(2-Chlorophenyl)-2,2,3,3,4,4,4-heptafluorobutanamide	121806-65-3	FC(F)(F)C(F)(F)C(F)(F)C(=O)Nc1ccccc1Cl		-6.36	0.63	2.23	P	IN
QL02334	2,2,3,3,4,4,4-Heptafluoro-N-(2-iodophenyl)butanamide	378215-44-2	FC(F)(F)C(F)(F)C(F)(F)C(=O)Nc1ccccc1I		-6.36	0.63	2.23	P	IN
QL02335	(1R-trans)-N,N'-1,2-Cyclohexanediylbis(perfluorobutanamide)	133964-60-0	FC(F)(F)C(F)(F)C(F)(F)C(=O)NC1CCCCC1NC(=O)C(F)(F)C(F)(F)C(F)(F)F		-7.81	0.65	2.73	P	IN
QL02336	2,2,3,3,4,4,4-Heptafluoro-N-(1H-1,2,4-triazol-5-yl)butanamide	70501-47-2	FC(F)(F)C(F)(F)C(F)(F)C(=O)Nc1Nncn1		-6.29	0.64	2.17	P	IN
QL02337	Allylperfluorobutanamide	24429-61-6	FC(F)(F)C(F)(F)C(F)(F)C(=O)NCC=C		-6.07	0.63	2.20	P	OUT
QL02338	N-(Phenylmethyl)perfluorobutanamide	560-02-1	FC(F)(F)C(F)(F)C(F)(F)C(=O)NCC1=CC=CC=C1		-6.46	0.62	2.42	P	OUT
QL02339	2,2,3,3,4,4,4-Heptafluoro-N-(2-phenylethyl)butanamide	29723-29-3	FC(F)(F)C(F)(F)C(F)(F)C(=O)NCCC1=CC=CC=C1		-6.42	0.62	2.49	P	OUT
QL02340	2,2,3,3,4,4,4-Heptafluoro-N-(3-phenylpropyl)butanamide	77970-73-1	FC(F)(F)C(F)(F)C(F)(F)C(=O)NCCCc1ccccc1		-6.58	0.61	2.64	P	OUT
QL02341	N,N'-bis(Perfluorobutanoyl)1,6-hexanediamine	123665-83-8	FC(F)(F)C(F)(F)C(F)(F)C(=O)NCCCCCCNC(=O)C(F)(F)C(F)(F)C(F)(F)F		-7.89	0.64	2.98	P	OUT
QL02342	N,N'-bis(Perfluorobutanoyl)1,5-pentanediamine	1814-80-8	FC(F)(F)C(F)(F)C(F)(F)C(=O)NCCCCCNC(=O)C(F)(F)C(F)(F)C(F)(F)F		-7.92	0.64	2.94	P	OUT
QL02343	N,N'-bis(Perfluorobutanoyl)1,4-butanediamine	2967-59-1	FC(F)(F)C(F)(F)C(F)(F)C(=O)NCCCCNC(=O)C(F)(F)C(F)(F)C(F)(F)F		-7.82	0.64	2.82	P	IN
QL02344	n-(2-chloroethyl)-2,2,3,3,4,4,4-heptafluorobutanamide	4314-29-8	FC(F)(F)C(F)(F)C(F)(F)C(=O)NCCCl		-6.07	0.63	2.20	P	OUT
QL02345	N,N'-bis(Perfluorobutanoyl)1,3-propanediamine	149981-59-9	FC(F)(F)C(F)(F)C(F)(F)C(=O)NCCCNC(=O)C(F)(F)C(F)(F)C(F)(F)F		-7.86	0.65	2.77	P	IN
QL02346	ST50925188	86273-64-5	FC(F)(F)C(F)(F)C(F)(F)C(=O)NCCNC(=O)C(F)(F)C(F)(F)C(F)(F)F		-7.76	0.66	2.65	P	IN
QL02347	N'-Benzylidene-2,2,3,3,4,4,4-heptafluorobutanehydrazide	736-62-9	FC(F)(F)C(F)(F)C(F)(F)C(=O)NN=Cc1ccccc1		-6.42	0.62	2.49	P	OUT
QL02348	2,2,3,3,4,4,4-Heptafluoro-N'-[(pyridin-4-yl)methylidene]butanehydrazide	109373-81-1	FC(F)(F)C(F)(F)C(F)(F)C(=O)NN=Cc1ccncc1		-6.42	0.62	2.49	P	OUT
QL02349	2,2,3,3,4,4,4-heptafluoro-n'-(2,2,3,3,4,4,4-heptafluorobutanoyl)butanehydrazide	648-19-1	FC(F)(F)C(F)(F)C(F)(F)C(=O)NNC(=O)C(F)(F)C(F)(F)C(F)(F)F		-7.71	0.67	2.45	P	IN
QL02350	2,2,3,3,4,4,4-Heptafluoro-N'-phenylbutanehydrazide	77146-66-8	FC(F)(F)C(F)(F)C(F)(F)C(=O)NNc1ccccc1		-6.46	0.62	2.42	P	OUT
QL02351	4-Chlorophenyl (2,2,3,3,4,4,4-heptafluorobutanoyl)sulfamate	1443258-17-0	FC(F)(F)C(F)(F)C(F)(F)C(=O)NS(=O)(=O)Oc1ccc(Cl)cc1		-7.35	0.64	2.68	P	OUT
QL02352	Heptafluorobutanoic anhydride	NOCAS_861881	FC(F)(F)C(F)(F)C(F)(F)C(=O)OC(=O)C(F)(F)C(F)(F)C(F)(F)F		-7.72	0.67	2.38	P	IN
QL02353	1,1,1,3,3,3-Hexafluoro-2-(trifluoromethyl)propan-2-yl heptafluorobutanoate	922168-38-5	FC(F)(F)C(F)(F)C(F)(F)C(=O)OC(C(F)(F)F)(C(F)(F)F)C(F)(F)F		-6.95	0.67	1.96	P	IN
QL02354	Vinyl perfluorobutyrate	356-28-5	FC(F)(F)C(F)(F)C(F)(F)C(=O)OC=C		-5.78	0.63	1.93	P	IN
QL02355	Decane-1,10-diyl bis(heptafluorobutanoate)	426-64-2	FC(F)(F)C(F)(F)C(F)(F)C(=O)OCCCCCCCCCCOC(=O)C(F)(F)C(F)(F)C(F)(F)F		-8.03	0.62	3.27	P	OUT
QL02356	bis(heptafluorobutyryl)peroxide	336-64-1	FC(F)(F)C(F)(F)C(F)(F)C(=O)OOC(=O)C(F)(F)C(F)(F)C(F)(F)F		-7.71	0.67	2.45	P	IN
QL02357	2,2,3,3,4,4,4-Heptafluoro-1-(morpholin-4-yl)butane-1-thione	1206684-15-2	FC(F)(F)C(F)(F)C(F)(F)C(=S)N1CCOCC1		-6.23	0.63	2.17	P	IN
QL02358	2,2,3,3,4,4,4-Heptafluoro-N-phenylbutanethioamide	61881-12-7	FC(F)(F)C(F)(F)C(F)(F)C(=S)NC1=CC=CC=C1		-6.25	0.63	2.25	P	OUT
QL02359	2-bromo-1,1,1,3,3,4,4,5,5,5-decafluoro-2-(trifluoromethyl)pentane	22528-67-2	FC(F)(F)C(F)(F)C(F)(F)C(Br)(C(F)(F)F)C(F)(F)F		-6.74	0.68	1.72	P	IN
QL02360	2-bromo-3,3,4,4,5,5,5-heptafluoropent-1-ene	96916-53-9	FC(F)(F)C(F)(F)C(F)(F)C(Br)=C		-5.76	0.67	1.46	P	IN
QL02361	heptafluorobutanoyl bromide	375-13-3	FC(F)(F)C(F)(F)C(F)(F)C(Br)=O		-5.76	0.67	1.46	P	IN
QL02362	4,5-Dibromo-1,1,1,2,2,3,3-heptafluoropentane	377-46-8	FC(F)(F)C(F)(F)C(F)(F)C(Br)CBr		-5.98	0.65	1.84	P	IN
QL02363	1,1,1,2,2,3,3,5,6,6,6-Undecafluoro-4,4,5-tris(trifluoromethyl)hexane	86714-23-0	FC(F)(F)C(F)(F)C(F)(F)C(C(F)(F)F)(C(F)(F)F)C(F)(C(F)(F)F)C(F)(F)F		-6.83	0.68	1.79	P	IN
QL02364	1,1,1,2,2,3,3,5,5,5-Decafluoro-4,4-bis(trifluoromethyl)pentane	29971-79-7	FC(F)(F)C(F)(F)C(F)(F)C(C(F)(F)F)(C(F)(F)F)C(F)(F)F		-6.49	0.68	1.63	P	IN

QL02365	4,4,5,5,6,6,6-Heptafluoro-3,3-bis(trifluoromethyl)hex-1-ene	79272-26-7	FC(F)(F)C(F)(F)C(F)(F)C(C=C)(C(F)(F)F)C(F)(F)F		-6.28	0.67	1.69	P	IN
QL02366	1-bromo-4,4,5,5,6,6,6-heptafluoro-3,3-bis(trifluoromethyl)hex-1-ene	128454-94-4	FC(F)(F)C(F)(F)C(F)(F)C(C=CBr)(C(F)(F)F)C(F)(F)F		-6.13	0.66	1.79	P	IN
QL02367	6,6,7,7,8,8,8-Heptafluoro-5,5-bis(trifluoromethyl)octa-1,3-diene	1429188-77-1	FC(F)(F)C(F)(F)C(F)(F)C(C=CC=C)(C(F)(F)F)C(F)(F)F		-6.43	0.65	2.04	P	IN
QL02368	4,4,5,5,6,6,6-heptafluoro-1-iodo-3,3-bis(trifluoromethyl)hex-1-ene	126681-21-8	FC(F)(F)C(F)(F)C(F)(F)C(C=CI)(C(F)(F)F)C(F)(F)F		-6.13	0.66	1.79	P	IN
QL02369	(Perfluoropropyl)-2-(2-phenylhydrazinylidene)ethanal phenylhydrazone	57912-15-9	FC(F)(F)C(F)(F)C(F)(F)C(C=NNC1=CC=CC=C1)=NNC1=CC=CC=C1		-6.26	0.60	2.68	P	OUT
QL02370	2,2'-(2,2,3,3,4,4,4-Heptafluorobutane-1,1-diyl)di(1H-pyrrole)	164792-01-2	FC(F)(F)C(F)(F)C(F)(F)C(c1Nccc1)c1Nccc1		-5.35	0.62	1.87	P	OUT
QL02371	(6E)-1,1,1,2,2,3,3,10,10,11,11,12,12,12-Tetradecafluoro-4,4,9,9-tetrakis(trifluoromethyl)dodec-6-ene	1946796-71-9	FC(F)(F)C(F)(F)C(F)(F)C(CC=CCC(C(F)(F)F)(C(F)(F)F)C(F)(F)C(F)(F)C(F)(F)F)(C(F)(F)F)C(F)(F)F		-7.66	0.67	2.35	P	IN
QL02372	Bis{4-[3,3,4,4,5,5,5-heptafluoro-2,2-bis(trifluoromethyl)pentyl]phenyl}(phenyl)phosphane	322647-83-6	FC(F)(F)C(F)(F)C(F)(F)C(CC1=CC=C(C=C1)P(C1=CC=CC=C1)C1=CC=C(CC(C(F)(F)F)(C(F)(F)F)C(F)(F)C(F)(F)C(F)(F)F)C=C1)(C(F)(F)F)C(F)(F)F		-8.38	0.65	3.07	P	OUT
QL02373	1-Bromo-4-[3,3,4,4,5,5,5-heptafluoro-2,2-bis(trifluoromethyl)pentyl]benzene	149068-60-0	FC(F)(F)C(F)(F)C(F)(F)C(Cc1ccc(Br)cc1)(C(F)(F)F)C(F)(F)F		-6.92	0.65	2.25	P	IN
QL02374	Tris{4-[3,3,4,4,5,5,5-heptafluoro-2,2-bis(trifluoromethyl)pentyl]phenyl}phosphane	322647-82-5	FC(F)(F)C(F)(F)C(F)(F)C(Cc1ccc(cc1)P(c1ccc(CC(C(F)(F)F)(C(F)(F)F)C(F)(F)C(F)(F)C(F)(F)F)cc1)c1ccc(CC(C(F)(F)F)(C(F)(F)F)C(F)(F)C(F)(F)C(F)(F)F)cc1)(C(F)(F)F)C(F)(F)F		-8.49	0.66	2.96	P	IN
QL02375	2-[3,3,4,4,5,5,5-Heptafluoro-2,2-bis(trifluoromethyl)pentyl]oxirane	119285-89-1	FC(F)(F)C(F)(F)C(F)(F)C(CC1CO1)(C(F)(F)F)C(F)(F)F		-6.49	0.67	1.81	P	IN
QL02376	6-Bromo-1,1,1,2,2,3,3-heptafluoro-4,4-bis(trifluoromethyl)hexane	128454-91-1	FC(F)(F)C(F)(F)C(F)(F)C(CCBr)(C(F)(F)F)C(F)(F)F		-6.13	0.66	1.79	P	IN
QL02377	1,1,1,2,2,3,3-heptafluoro-6-iodo-4,4-bis(trifluoromethyl)hexane	115347-68-7	FC(F)(F)C(F)(F)C(F)(F)C(CCI)(C(F)(F)F)C(F)(F)F		-6.13	0.66	1.79	P	IN
QL02378	2-Chloro(perfluoro-2-methylpentane)	67437-97-2	FC(F)(F)C(F)(F)C(F)(F)C(Cl)(C(F)(F)F)C(F)(F)F		-6.74	0.68	1.72	P	IN
QL02379	Trichloroheptafluorobutane	28984-80-7	FC(F)(F)C(F)(F)C(F)(F)C(Cl)(Cl)Cl		-6.18	0.68	1.45	P	IN
QL02380	Heptafluorobutyryl Chloride	375-16-6	FC(F)(F)C(F)(F)C(F)(F)C(Cl)=O		-5.76	0.67	1.46	P	IN
QL02381	4,5-Dichloro-1,1,1,2,2,3,3-heptafluoropentane	577773-57-0	FC(F)(F)C(F)(F)C(F)(F)C(Cl)CCl		-5.98	0.65	1.84	P	IN
QL02382	1,1,1,2,4,5,5,6,6,7,7,7-dodecafluoro-2,4-bis(trifluoromethyl)heptan-3-one	95639-15-9	FC(F)(F)C(F)(F)C(F)(F)C(F)(C(=O)C(F)(C(F)(F)F)C(F)(F)F)C(F)(F)F		-7.40	0.68	2.09	P	IN
QL02383	Hexane, 1,1,1,2,2,3,3,4,5,5,6,6,6-tridecafluoro-4-(trifluoromethyl)-	558-69-0	FC(F)(F)C(F)(F)C(F)(F)C(F)(C(F)(F)F)C(F)(F)C(F)(F)F		-7.05	0.69	1.85	P	IN
QL02384	Perfluoroisohexane	355-04-4	FC(F)(F)C(F)(F)C(F)(F)C(F)(C(F)(F)F)C(F)(F)F		-6.74	0.68	1.72	P	IN
QL02385	Chromium pentacarbonyl[perfluoro-4-(isocyano-βC)butane]	106065-05-8	FC(F)(F)C(F)(F)C(F)(F)C(F)(F)[N]#[C-][Cr]([C-]#[O+])([C-]#[O+])([C-]#[O+])([C-]#[O+])[C-]#[O+]		-6.64	0.65	2.16	P	IN
QL02386	1-Bromoperfluorobutane	375-48-4	FC(F)(F)C(F)(F)C(F)(F)C(F)(F)Br		-6.18	0.68	1.45	P	IN
QL02387	1-(4-Bromophenyl)-2,2,3,3,4,4,5,5,5-nonafluoropentan-1-one	1228786-80-8	FC(F)(F)C(F)(F)C(F)(F)C(F)(F)C(=O)c1ccc(Br)cc1		-7.21	0.65	2.46	P	IN
QL02388	2-(2,2,3,3,4,4,5,5,5-Nonafluoropentanoyl)cyclopentan-1-one	347361-45-9	FC(F)(F)C(F)(F)C(F)(F)C(F)(F)C(=O)C1CCCC1=O		-6.77	0.65	2.17	P	IN
QL02389	1,1,1,2,2,3,3,4,4,8,8,9,9,10,10,11,11,11-Octadecafluoroundecane-5,7-dione	261503-82-6	FC(F)(F)C(F)(F)C(F)(F)C(F)(F)C(=O)CC(=O)C(F)(F)C(F)(F)C(F)(F)C(F)(F)F		-8.35	0.68	2.63	P	IN
QL02390	4,4,5,5,6,6,7,7,7-Nonafluoro-1-phenylheptane-1,3-dione	168920-97-6	FC(F)(F)C(F)(F)C(F)(F)C(F)(F)C(=O)CC(=O)c1ccccc1		-7.00	0.64	2.52	P	OUT
QL02391	3,5-Bis(perfluorohexyl)-1-(nonafluoropentanoyl)-1H-pyrazole	231630-88-9	FC(F)(F)C(F)(F)C(F)(F)C(F)(F)C(=O)N1N=C(C=C1C(F)(F)C(F)(F)C(F)(F)C(F)(F)C(F)(F)C(F)(F)F)C(F)(F)C(F)(F)C(F)(F)C(F)(F)C(F)(F)C(F)(F)F		-9.26	0.69	2.99	P	OUT
QL02392	2,2,3,3,4,4,5,5,5-Nonafluoro-N-phenylpentanamide	122590-97-0	FC(F)(F)C(F)(F)C(F)(F)C(F)(F)C(=O)Nc1ccccc1		-6.93	0.64	2.46	P	IN
QL02393	N,N'-bis(Perfluoropentanoyl)1,2-ethanediamine	119403-53-1	FC(F)(F)C(F)(F)C(F)(F)C(F)(F)C(=O)NCCNC(=O)C(F)(F)C(F)(F)C(F)(F)C(F)(F)F		-8.38	0.66	2.85	P	IN
QL02394	Sodium perfluorovalerate	2706-89-0	FC(F)(F)C(F)(F)C(F)(F)C(F)(F)C(=O)O[Na]		-6.37	0.66	1.90	P	IN
QL02395	Perfluoropentanoic anhydride	308-28-1	FC(F)(F)C(F)(F)C(F)(F)C(F)(F)C(=O)OC(=O)C(F)(F)C(F)(F)C(F)(F)C(F)(F)F		-8.35	0.68	2.63	P	IN
QL02396	Vinyl perfluorovalerate	424-37-3	FC(F)(F)C(F)(F)C(F)(F)C(F)(F)C(=O)OC=C		-6.59	0.64	2.19	P	IN
QL02397	Allyl perfluoropentanoate	84145-17-5	FC(F)(F)C(F)(F)C(F)(F)C(F)(F)C(=O)OCC=C		-6.53	0.64	2.27	P	IN
QL02398	3-(Trifluorosilyl)propyl nonafluoropentanoate	92916-37-5	FC(F)(F)C(F)(F)C(F)(F)C(F)(F)C(=O)OCCC[Si](F)(F)F		-7.28	0.65	2.51	P	IN
QL02399	1,1,2-Tribromo-1-(nonafluorobutyl)ethane	59665-24-6	FC(F)(F)C(F)(F)C(F)(F)C(F)(F)C(Br)(Br)CBr		-6.72	0.67	1.89	P	IN
QL02400	2-Bromo-3,3,4,4,5,5,6,6,6-nonafluorohex-1-ene	59665-23-5	FC(F)(F)C(F)(F)C(F)(F)C(F)(F)C(Br)=C		-6.61	0.67	1.81	P	IN
QL02401	Nonafluoropentanoyl bromide	159623-35-5	FC(F)(F)C(F)(F)C(F)(F)C(F)(F)C(Br)=O		-6.61	0.67	1.81	P	IN
QL02402	5,6-dibromo-1,1,1,2,2,3,3,4,4-nonafluorohexane	236736-19-9	FC(F)(F)C(F)(F)C(F)(F)C(F)(F)C(Br)CBr		-6.37	0.66	1.90	P	IN
QL02403	2-Chloro-3,3,4,4,5,5,6,6,6-nonafluorohex-1-ene	110927-72-5	FC(F)(F)C(F)(F)C(F)(F)C(F)(F)C(Cl)=C		-6.61	0.67	1.81	P	IN

QL02404	Nonafluoropentanoyl chloride	375-60-0	FC(F)(F)C(F)(F)C(F)(F)C(F)(F)C(Cl)=O		-6.61	0.67	1.81	P	IN
QL02405	5,6-Dichloro-1,1,1,2,2,3,3,4,4-nonafluorohexane	136483-77-7	FC(F)(F)C(F)(F)C(F)(F)C(F)(F)C(Cl)CCl		-6.37	0.66	1.90	P	IN
QL02406	Perfluoro-3-methylheptane	153273-33-7	FC(F)(F)C(F)(F)C(F)(F)C(F)(F)C(F)(C(F)(F)F)C(F)(F)C(F)(F)F		-7.59	0.69	2.10	P	IN
QL02407	2,2,3,3,4,4,5,5,6,6,6-Undecafluoro-N-(2-phenylethyl)hexanamide	70414-01-6	FC(F)(F)C(F)(F)C(F)(F)C(F)(F)C(F)(F)C(=O)NCCc1ccccc1		-7.45	0.64	2.76	P	OUT
QL02408	Perfluorohexanoic anhydride	308-13-4	FC(F)(F)C(F)(F)C(F)(F)C(F)(F)C(F)(F)C(=O)OC(=O)C(F)(F)C(F)(F)C(F)(F)C(F)(F)C(F)(F)F		-8.87	0.68	2.84	P	OUT
QL02409	Ethenyl undecafluorohexanoate	464-35-7	FC(F)(F)C(F)(F)C(F)(F)C(F)(F)C(F)(F)C(=O)OC=C		-6.91	0.65	2.24	P	IN
QL02410	Undecafluorohexanoyl chloride	335-53-5	FC(F)(F)C(F)(F)C(F)(F)C(F)(F)C(F)(F)C(Cl)=O		-6.91	0.68	1.90	P	IN
QL02411	1,2-Dibromoperfluoroheptane	678-32-0	FC(F)(F)C(F)(F)C(F)(F)C(F)(F)C(F)(F)C(F)(Br)C(F)(F)Br		-7.74	0.69	2.15	P	IN
QL02412	Perfluoro-2-methylheptane	153273-32-6	FC(F)(F)C(F)(F)C(F)(F)C(F)(F)C(F)(F)C(F)(C(F)(F)F)C(F)(F)F		-7.59	0.69	2.10	P	IN
QL02413	(Perfluorohexyl)hexadecene	1244062-15-4	FC(F)(F)C(F)(F)C(F)(F)C(F)(F)C(F)(F)C(F)(F)/C=C/CCCCCCCCCCCCCC[H]		-7.92	0.60	3.47	P	OUT
QL02414	Perfluorohexylbromide	335-56-8	FC(F)(F)C(F)(F)C(F)(F)C(F)(F)C(F)(F)C(F)(F)Br		-7.19	0.69	1.90	P	IN
QL02415	N-(2,2,3,3,4,4,5,5,6,6,7,7,7-Tridecafluoroheptanimidoyl)benzenecarboximidamide	123528-68-7	FC(F)(F)C(F)(F)C(F)(F)C(F)(F)C(F)(F)C(F)(F)C(=N)NC(=N)c1ccccc1		-7.82	0.65	2.76	P	IN
QL02416	N-[(2,2,3,3,4,4,5,5,6,6,7,7,7-Tridecafluoroheptanimidoyl)oxy]benzenecarboximidamide	146304-61-2	FC(F)(F)C(F)(F)C(F)(F)C(F)(F)C(F)(F)C(F)(F)C(=N)ONC(=N)c1ccccc1		-8.00	0.65	2.90	P	IN
QL02417	2-(2,2,3,3,4,4,5,5,6,6,7,7,7-Tridecafluoroheptanoyl)cyclohexan-1-one	141478-89-9	FC(F)(F)C(F)(F)C(F)(F)C(F)(F)C(F)(F)C(F)(F)C(=O)C1CCCCC1=O		-7.57	0.66	2.51	P	IN
QL02418	8H,8H-Perfluoropentadecane-7,9-dione	261503-74-6	FC(F)(F)C(F)(F)C(F)(F)C(F)(F)C(F)(F)C(F)(F)C(=O)CC(=O)C(F)(F)C(F)(F)C(F)(F)C(F)(F)C(F)(F)C(F)(F)F		-9.31	0.68	3.03	P	OUT
QL02419	4,4,5,5,6,6,7,7,8,8,9,9,9-Tridecafluoro-1-phenylnonane-1,3-dione	99338-16-6	FC(F)(F)C(F)(F)C(F)(F)C(F)(F)C(F)(F)C(F)(F)C(=O)CC(=O)C1=CC=CC=C1		-7.82	0.65	2.76	P	IN
QL02420	7,7,8,8,9,9,10,10,11,11,12,12,12-Tridecafluoro-1-(1H-naphtho[2,3-d]imidazol-2-yl)dodecan-6-one	240121-57-7	FC(F)(F)C(F)(F)C(F)(F)C(F)(F)C(F)(F)C(F)(F)C(=O)CCCCCc1Nc2cc3ccccc3cc2n1		-8.31	0.63	3.22	P	OUT
QL02421	1-(1H-Benzimidazol-2-yl)-7,7,8,8,9,9,10,10,11,11,12,12,12-tridecafluorododecan-6-one	237738-71-5	FC(F)(F)C(F)(F)C(F)(F)C(F)(F)C(F)(F)C(F)(F)C(=O)CCCCCc1Nc2ccccc2n1		-8.10	0.64	3.10	P	OUT
QL02422	2,2,3,3,4,4,5,5,6,6,7,7,7-Tridecafluoro-1-(morpholin-4-yl)heptan-1-one	144237-77-4	FC(F)(F)C(F)(F)C(F)(F)C(F)(F)C(F)(F)C(F)(F)C(=O)N1CCOCC1		-7.52	0.66	2.52	P	IN
QL02423	N-Cyclohexyl-2,2,3,3,4,4,5,5,6,6,7,7,7-tridecafluoroheptanamide	73602-45-6	FC(F)(F)C(F)(F)C(F)(F)C(F)(F)C(F)(F)C(F)(F)C(=O)NC1CCCCC1		-7.78	0.65	2.71	P	IN
QL02424	Sodium perfluoroheptanoate	20109-59-5	FC(F)(F)C(F)(F)C(F)(F)C(F)(F)C(F)(F)C(F)(F)C(=O)O[Na]		-7.32	0.67	2.24	P	IN
QL02425	Perfluoroheptanoic anhydride	78225-99-7	FC(F)(F)C(F)(F)C(F)(F)C(F)(F)C(F)(F)C(F)(F)C(=O)OC(=O)C(F)(F)C(F)(F)C(F)(F)C(F)(F)C(F)(F)C(F)(F)F		-9.31	0.68	3.03	P	OUT
QL02426	Ethenyl tridecafluoroheptanoate	240408-96-2	FC(F)(F)C(F)(F)C(F)(F)C(F)(F)C(F)(F)C(F)(F)C(=O)OC=C		-7.48	0.66	2.45	P	IN
QL02427	Prop-2-en-1-yl tridecafluoroheptanoate	53378-90-8	FC(F)(F)C(F)(F)C(F)(F)C(F)(F)C(F)(F)C(F)(F)C(=O)OCC=C		-7.43	0.65	2.52	P	IN
QL02428	7,7,8-Tribromo-1,1,1,2,2,3,3,4,4,5,5,6,6-tridecafluorooctane	59665-25-7	FC(F)(F)C(F)(F)C(F)(F)C(F)(F)C(F)(F)C(F)(F)C(Br)(Br)CBr		-7.58	0.68	2.23	P	IN
QL02429	2-Bromo-2-(Perfluorohexyl)ethene	51249-64-0	FC(F)(F)C(F)(F)C(F)(F)C(F)(F)C(F)(F)C(F)(F)C(Br)=C		-7.50	0.68	2.16	P	IN
QL02430	(1Z)-1,2-Dibromo-3,3,4,4,5,5,6,6,7,7,8,8,8-tridecafluorooct-1-ene	59665-28-0	FC(F)(F)C(F)(F)C(F)(F)C(F)(F)C(F)(F)C(F)(F)C(Br)=CBr		-7.32	0.67	2.24	P	IN
QL02431	tridecafluoroheptanoyl bromide	159623-34-4	FC(F)(F)C(F)(F)C(F)(F)C(F)(F)C(F)(F)C(F)(F)C(Br)=O		-7.50	0.68	2.16	P	IN
QL02432	7,8-Dibromo-1,1,1,2,2,3,3,4,4,5,5,6,6-tridecafluorooctane	51249-62-8	FC(F)(F)C(F)(F)C(F)(F)C(F)(F)C(F)(F)C(F)(F)C(Br)CBr		-7.32	0.67	2.24	P	IN
QL02433	Perfluoroheptanoyl chloride	52447-22-0	FC(F)(F)C(F)(F)C(F)(F)C(F)(F)C(F)(F)C(F)(F)C(Cl)=O		-7.50	0.68	2.16	P	IN
QL02434	7,8-Dichloro-1,1,1,2,2,3,3,4,4,5,5,6,6-tridecafluorooctane	319490-55-6	FC(F)(F)C(F)(F)C(F)(F)C(F)(F)C(F)(F)C(F)(F)C(Cl)CCl		-7.32	0.67	2.24	P	IN
QL02435	1-Bromopentadecafluoroheptane	375-88-2	FC(F)(F)C(F)(F)C(F)(F)C(F)(F)C(F)(F)C(F)(F)C(F)(F)Br		-7.74	0.69	2.15	P	IN
QL02436	9H,9H-Triacontafluoro-8,10-heptadecanedione	36554-97-9	FC(F)(F)C(F)(F)C(F)(F)C(F)(F)C(F)(F)C(F)(F)C(F)(F)C(=O)CC(=O)C(F)(F)C(F)(F)C(F)(F)C(F)(F)C(F)(F)C(F)(F)C(F)(F)F		-9.68	0.69	3.19	P	OUT
QL02437	(1R,2R)-N,N'-bis(Perfluorooctanoyl)-1,2-cyclohexanediamine	864149-62-2	FC(F)(F)C(F)(F)C(F)(F)C(F)(F)C(F)(F)C(F)(F)C(F)(F)C(=O)N[C@@H]1CCCC[C@H]1NC(=O)C(F)(F)C(F)(F)C(F)(F)C(F)(F)C(F)(F)C(F)(F)C(F)(F)F		-9.70	0.67	3.40	P	OUT
QL02438	3,4-Bis((perfluoro-1-oxooctyl)amino)benzenesulfonyl chloride	24216-05-5	FC(F)(F)C(F)(F)C(F)(F)C(F)(F)C(F)(F)C(F)(F)C(F)(F)C(=O)NC1=C(NC(=O)C(F)(F)C(F)(F)C(F)(F)C(F)(F)C(F)(F)C(F)(F)C(F)(F)F)C=C(C=C1)S(Cl)(=O)=O		-9.76	0.67	3.39	P	OUT
QL02439	N,N'-(Propane-1,3-diyl)bis(2,2,3,3,4,4,5,5,6,6,7,7,8,8,8-pentadecafluorooctanamide)	29680-98-6	FC(F)(F)C(F)(F)C(F)(F)C(F)(F)C(F)(F)C(F)(F)C(F)(F)C(=O)NCCCNC(=O)C(F)(F)C(F)(F)C(F)(F)C(F)(F)C(F)(F)C(F)(F)C(F)(F)F		-9.75	0.67	3.42	P	OUT
QL02440	Perfluorooctanoic anhydride	33496-48-9	FC(F)(F)C(F)(F)C(F)(F)C(F)(F)C(F)(F)C(F)(F)C(F)(F)C(=O)OC(=O)C(F)(F)C(F)(F)C(F)(F)C(F)(F)C(F)(F)C(F)(F)C(F)(F)F		-9.68	0.69	3.19	P	OUT
QL02441	Vinyl perfluorooctanoate	307-93-7	FC(F)(F)C(F)(F)C(F)(F)C(F)(F)C(F)(F)C(F)(F)C(F)(F)C(=O)OC=C		-7.71	0.66	2.51	P	IN

QL02442	Prop-2-en-1-yl pentadecafluorooctanoate	20120-44-9	FC(F)(F)C(F)(F)C(F)(F)C(F)(F)C(F)(F)C(F)(F)C(F)(F)C(=O)OCC=C		-7.92	0.66	2.70	P	IN
QL02443	4-Vinylbenzyl perfluorooctanoate	230295-06-4	FC(F)(F)C(F)(F)C(F)(F)C(F)(F)C(F)(F)C(F)(F)C(F)(F)C(=O)OCC1=CC=C(C=C)C=C1		-8.46	0.65	3.09	P	IN
QL02444	3,3,4,4,5,5,6,6,7,7,8,8,9,9,10,10,10-Heptadecafluorodecyl pentadecafluorooctanoate	886046-97-5	FC(F)(F)C(F)(F)C(F)(F)C(F)(F)C(F)(F)C(F)(F)C(F)(F)C(=O)OCCC(F)(F)C(F)(F)C(F)(F)C(F)(F)C(F)(F)C(F)(F)C(F)(F)C(F)(F)F		-9.83	0.68	3.31	P	OUT
QL02445	Pentadecafluorooctanoyl bromide	222037-87-8	FC(F)(F)C(F)(F)C(F)(F)C(F)(F)C(F)(F)C(F)(F)C(F)(F)C(Br)=O		-7.72	0.68	2.24	P	IN
QL02446	Pentadecafluorooctyl chloride	335-64-8	FC(F)(F)C(F)(F)C(F)(F)C(F)(F)C(F)(F)C(F)(F)C(F)(F)C(Cl)=O		-7.72	0.68	2.24	P	IN
QL02447	1,1,1,3,3,5-Hexafluoro-5-(heptadecafluorooctyl)-5-(pentafluoroethyl)trisiloxane	163921-85-5	FC(F)(F)C(F)(F)C(F)(F)C(F)(F)C(F)(F)C(F)(F)C(F)(F)C(F)(F)[Si](F)(O[Si](F)(F)O[Si](F)(F)F)C(F)(F)C(F)(F)F		-8.90	0.68	2.85	P	OUT
QL02448	1-Bromoheptadecafluorooctane	423-55-2	FC(F)(F)C(F)(F)C(F)(F)C(F)(F)C(F)(F)C(F)(F)C(F)(F)C(F)(F)Br		-7.94	0.69	2.24	P	IN
QL02449	4,4,5,5,6,6,7,7,8,8,9,9,10,10,11,11,11-heptadecafluoro-1-phenylundecane-1,3-dione	141522-69-2	FC(F)(F)C(F)(F)C(F)(F)C(F)(F)C(F)(F)C(F)(F)C(F)(F)C(F)(F)C(=O)CC(=O)C1=CC=CC=C1		-8.42	0.66	2.95	P	IN
QL02450	2,2,3,3,4,4,5,5,6,6,7,7,8,8,9,9,9-Heptadecafluoro-N,N-diphenylnonanamide	157584-47-9	FC(F)(F)C(F)(F)C(F)(F)C(F)(F)C(F)(F)C(F)(F)C(F)(F)C(F)(F)C(=O)N(c1ccccc1)c1ccccc1		-7.97	0.65	2.88	P	IN
QL02451	2,2,3,3,4,4,5,5,6,6,7,7,8,8,9,9,9-Heptadecafluoro-N-phenylnonanamide	313366-93-7	FC(F)(F)C(F)(F)C(F)(F)C(F)(F)C(F)(F)C(F)(F)C(F)(F)C(F)(F)C(=O)Nc1ccccc1		-8.39	0.66	2.90	P	IN
QL02452	N-Cyclohexyl-2,2,3,3,4,4,5,5,6,6,7,7,8,8,9,9,9-heptadecafluorononanamide	73602-46-7	FC(F)(F)C(F)(F)C(F)(F)C(F)(F)C(F)(F)C(F)(F)C(F)(F)C(F)(F)C(=O)NC1CCCCC1		-8.39	0.66	2.90	P	IN
QL02453	N-Allylperfluorononanamide	221466-77-9	FC(F)(F)C(F)(F)C(F)(F)C(F)(F)C(F)(F)C(F)(F)C(F)(F)C(F)(F)C(=O)NCC=C		-8.12	0.66	2.74	P	IN
QL02454	N,N'-bis(Perfluorononanoyl)1,12-dodecandiamine	204922-38-3	FC(F)(F)C(F)(F)C(F)(F)C(F)(F)C(F)(F)C(F)(F)C(F)(F)C(F)(F)C(=O)NCCCCCCCCCCCCNC(=O)C(F)(F)C(F)(F)C(F)(F)C(F)(F)C(F)(F)C(F)(F)C(F)(F)C(F)(F)F		-10.15	0.65	3.91	P	OUT
QL02455	N,N'-bis(Perfluorononanoyl)1,6-hexanediamine	174495-83-1	FC(F)(F)C(F)(F)C(F)(F)C(F)(F)C(F)(F)C(F)(F)C(F)(F)C(F)(F)C(=O)NCCCCCCNC(=O)C(F)(F)C(F)(F)C(F)(F)C(F)(F)C(F)(F)C(F)(F)C(F)(F)C(F)(F)F		-10.07	0.67	3.66	P	OUT
QL02456	N,N'-bis(Perfluorononanoyl)1,2-ethanediamine	97005-61-3	FC(F)(F)C(F)(F)C(F)(F)C(F)(F)C(F)(F)C(F)(F)C(F)(F)C(F)(F)C(=O)NCCNC(=O)C(F)(F)C(F)(F)C(F)(F)C(F)(F)C(F)(F)C(F)(F)C(F)(F)C(F)(F)F		-10.03	0.68	3.48	P	OUT
QL02457	Ethenyl heptadecafluorononanoate	54640-64-1	FC(F)(F)C(F)(F)C(F)(F)C(F)(F)C(F)(F)C(F)(F)C(F)(F)C(F)(F)C(=O)OC=C		-8.16	0.67	2.69	P	IN
QL02458	Prop-2-en-1-yl heptadecafluorononanoate	117374-33-1	FC(F)(F)C(F)(F)C(F)(F)C(F)(F)C(F)(F)C(F)(F)C(F)(F)C(F)(F)C(=O)OCC=C		-8.12	0.66	2.74	P	IN
QL02459	Benzyl heptadecafluorononanoate	117374-38-6	FC(F)(F)C(F)(F)C(F)(F)C(F)(F)C(F)(F)C(F)(F)C(F)(F)C(F)(F)C(=O)OCc1ccccc1		-8.39	0.66	2.95	P	IN
QL02460	9,9,10-Tribromo-1,1,1,2,2,3,3,4,4,5,5,6,6,7,7,8,8-heptadecafluorodecane	59665-26-8	FC(F)(F)C(F)(F)C(F)(F)C(F)(F)C(F)(F)C(F)(F)C(F)(F)C(F)(F)C(Br)(Br)CBr		-8.24	0.68	2.51	P	IN
QL02461	2-Bromo-3,3,4,4,5,5,6,6,7,7,8,8,9,9,10,10,10-heptadecafluorodec-1-ene	51249-65-1	FC(F)(F)C(F)(F)C(F)(F)C(F)(F)C(F)(F)C(F)(F)C(F)(F)C(F)(F)C(Br)=C		-8.18	0.68	2.45	P	IN
QL02462	Heptadecafluorononanoyl bromide	261503-42-8	FC(F)(F)C(F)(F)C(F)(F)C(F)(F)C(F)(F)C(F)(F)C(F)(F)C(F)(F)C(Br)=O		-8.18	0.68	2.45	P	IN
QL02463	9,10-Dibromo-1,1,1,2,2,3,3,4,4,5,5,6,6,7,7,8,8-heptadecafluorodecane	51249-63-9	FC(F)(F)C(F)(F)C(F)(F)C(F)(F)C(F)(F)C(F)(F)C(F)(F)C(F)(F)C(Br)CBr		-8.04	0.67	2.51	P	IN
QL02464	2-Chloro-3,3,4,4,5,5,6,6,7,7,8,8,9,9,10,10,10-heptadecafluorodec-1-ene	110927-73-6	FC(F)(F)C(F)(F)C(F)(F)C(F)(F)C(F)(F)C(F)(F)C(F)(F)C(F)(F)C(Cl)=C		-8.18	0.68	2.45	P	IN
QL02465	Perfluorononanoyl chloride	52447-23-1	FC(F)(F)C(F)(F)C(F)(F)C(F)(F)C(F)(F)C(F)(F)C(F)(F)C(F)(F)C(Cl)=O		-8.18	0.68	2.45	P	IN
QL02466	1-Bromoperfluorononane	558-96-3	FC(F)(F)C(F)(F)C(F)(F)C(F)(F)C(F)(F)C(F)(F)C(F)(F)C(F)(F)C(F)(F)Br		-8.37	0.69	2.44	P	IN
QL02467	Nonadecafluorodecanoic anhydride	942199-24-8	FC(F)(F)C(F)(F)C(F)(F)C(F)(F)C(F)(F)C(F)(F)C(F)(F)C(F)(F)C(F)(F)C(=O)OC(=O)C(F)(F)C(F)(F)C(F)(F)C(F)(F)C(F)(F)C(F)(F)C(F)(F)C(F)(F)C(F)(F)F		-10.31	0.69	3.48	P	OUT
QL02468	Ethenyl nonadecafluorodecanoate	423-88-1	FC(F)(F)C(F)(F)C(F)(F)C(F)(F)C(F)(F)C(F)(F)C(F)(F)C(F)(F)C(F)(F)C(=O)OC=C		-8.34	0.67	2.74	P	IN
QL02469	Nonadecafluorodecanoyl chloride	307-38-0	FC(F)(F)C(F)(F)C(F)(F)C(F)(F)C(F)(F)C(F)(F)C(F)(F)C(F)(F)C(F)(F)C(Cl)=O		-8.35	0.69	2.51	P	IN
QL02470	11,12-Dibromo-1,1,1,2,2,3,3,4,4,5,5,6,6,7,7,8,8,9,9,10,10-henicosafluorododecane	214358-54-0	FC(F)(F)C(F)(F)C(F)(F)C(F)(F)C(F)(F)C(F)(F)C(F)(F)C(F)(F)C(F)(F)C(F)(F)C(Br)CBr		-8.61	0.68	2.74	P	IN
QL02471	1-Bromo-1,1,2,2,3,3,4,4,5,5,6,6,7,7,8,8,9,9,10,10,11,11,12,12,12-pentacosafluorododecane	67193-90-2	FC(F)(F)C(F)(F)C(F)(F)C(F)(F)C(F)(F)C(F)(F)C(F)(F)C(F)(F)C(F)(F)C(F)(F)C(F)(F)C(F)(F)Br		-9.02	0.69	2.75	P	OUT
QL02472	Perfluorotetracosane	1766-41-2	FC(F)(F)F		-10.90	0.70	3.67	P	OUT

QL02473	Hexatetracontafluorodocosane	1127427-64-8	FC(F)(F)F		-10.66	0.70	3.55	P	OUT
QL02474	Dotetracontafluoroicosane	37589-57-4	FC(F)(F)C(F)(F)C(F)(F)C(F)(F)C(F)(F)C(F)(F)C(F)(F)C(F)(F)C(F)(F)C(F)(F)C(F)(F)C(F)(F)C(F)(F)C(F)(F)C(F)(F)C(F)(F)C(F)(F)C(F)(F)C(F)(F)F		-10.40	0.70	3.42	P	OUT
QL02475	Octatriacontafluorooctadecane	24768-65-8	FC(F)(F)C(F)(F)C(F)(F)C(F)(F)C(F)(F)C(F)(F)C(F)(F)C(F)(F)C(F)(F)C(F)(F)C(F)(F)C(F)(F)C(F)(F)C(F)(F)C(F)(F)C(F)(F)C(F)(F)F		-10.12	0.70	3.28	P	OUT
QL02476	((Perfluorohexadecyl)methyl)oxirane	94158-68-6	FC(F)(F)C(F)(F)C(F)(F)C(F)(F)C(F)(F)C(F)(F)C(F)(F)C(F)(F)C(F)(F)C(F)(F)C(F)(F)C(F)(F)C(F)(F)C(F)(F)C(F)(F)C(F)(F)CC1CO1		-9.91	0.69	3.27	P	OUT
QL02477	(Perfluorohexadecyl)hexadecane	137338-42-2	FC(F)(F)C(F)(F)C(F)(F)C(F)(F)C(F)(F)C(F)(F)C(F)(F)C(F)(F)C(F)(F)C(F)(F)C(F)(F)C(F)(F)C(F)(F)C(F)(F)C(F)(F)C(F)(F)CCCCCCCCCCCCCCCC		-10.30	0.65	4.05	P	OUT
QL02478	n2 fluorotelomer sulfonamido betaine-related, n=8_pos		FC(F)(F)C(F)(F)C(F)(F)C(F)(F)C(F)(F)C(F)(F)C(F)(F)C(F)(F)C(F)(F)C(F)(F)C(F)(F)C(F)(F)C(F)(F)C(F)(F)C(F)(F)CCS(NCCC[N+](CC(O)=O)(C)C)(=O)=O		-10.41	0.67	3.76	P	OUT
QL02479	Perfluorohexadecane	355-49-7	FC(F)(F)C(F)(F)C(F)(F)C(F)(F)C(F)(F)C(F)(F)C(F)(F)C(F)(F)C(F)(F)C(F)(F)C(F)(F)C(F)(F)C(F)(F)C(F)(F)C(F)(F)C(F)(F)F		-9.79	0.70	3.12	P	OUT
QL02480	Perfluoropentadecane	2264-03-1	FC(F)(F)C(F)(F)C(F)(F)C(F)(F)C(F)(F)C(F)(F)C(F)(F)C(F)(F)C(F)(F)C(F)(F)C(F)(F)C(F)(F)C(F)(F)C(F)(F)C(F)(F)F		-9.69	0.69	3.07	P	OUT
QL02481	Hentriacontafluoropentadecyl iodide	335-79-5	FC(F)(F)C(F)(F)C(F)(F)C(F)(F)C(F)(F)C(F)(F)C(F)(F)C(F)(F)C(F)(F)C(F)(F)C(F)(F)C(F)(F)C(F)(F)C(F)(F)C(F)(F)I		-9.69	0.69	3.07	P	OUT
QL02482	3,3,4,4,5,5,6,6,7,7,8,8,9,9,10,10,11,11,12,12,13,13,14,14,15,15,16,16,16-Nonacosafluorohexadec-1-ene	104564-28-5	FC(F)(F)C(F)(F)C(F)(F)C(F)(F)C(F)(F)C(F)(F)C(F)(F)C(F)(F)C(F)(F)C(F)(F)C(F)(F)C(F)(F)C(F)(F)C(F)(F)C=C		-9.50	0.69	3.07	P	OUT
QL02483	((Perfluorotetradecyl)methyl)oxirane	94158-67-5	FC(F)(F)C(F)(F)C(F)(F)C(F)(F)C(F)(F)C(F)(F)C(F)(F)C(F)(F)C(F)(F)C(F)(F)C(F)(F)C(F)(F)C(F)(F)C(F)(F)CC1CO1		-9.56	0.69	3.11	P	OUT
QL02484	n2 perfluoroalkyl acrylates, n=14		FC(F)(F)C(F)(F)C(F)(F)C(F)(F)C(F)(F)C(F)(F)C(F)(F)C(F)(F)C(F)(F)C(F)(F)C(F)(F)C(F)(F)C(F)(F)C(F)(F)CCOC(CC(C)=C)=O		-9.72	0.67	3.41	P	OUT
QL02485	multiple H-substituted, multiple ether-substituted perfluoroalkyl alcohols, n=7, m=5		FC(F)(F)C(F)(F)C(F)(F)C(F)(F)C(F)(F)C(F)(F)C(F)(F)C(F)(F)C(F)(F)C(F)(F)C(F)(F)C(F)(F)C(F)(F)C(F)(F)CCOCCOCCOCCOCCO[H]		-9.91	0.64	3.89	P	OUT
QL02486	multiple H-substituted, multiple ether-substituted perfluoroalkyl alcohols, n=7, m=6		FC(F)(F)C(F)(F)C(F)(F)C(F)(F)C(F)(F)C(F)(F)C(F)(F)C(F)(F)C(F)(F)C(F)(F)C(F)(F)C(F)(F)C(F)(F)C(F)(F)CCOCCOCCOCCOCCOCCO[H]		-9.98	0.64	4.03	P	OUT
QL02487	multiple H-substituted, multiple ether-substituted perfluoroalkyl alcohols, n=7, m=7		FC(F)(F)C(F)(F)C(F)(F)C(F)(F)C(F)(F)C(F)(F)C(F)(F)C(F)(F)C(F)(F)C(F)(F)C(F)(F)C(F)(F)C(F)(F)C(F)(F)CCOCCOCCOCCOCCOCCOCCO[H]		-9.99	0.63	4.13	P	OUT
QL02488	multiple H-substituted, multiple ether-substituted perfluoroalkyl alcohols, n=7, m=8		FC(F)(F)C(F)(F)C(F)(F)C(F)(F)C(F)(F)C(F)(F)C(F)(F)C(F)(F)C(F)(F)C(F)(F)C(F)(F)C(F)(F)C(F)(F)C(F)(F)CCOCCOCCOCCOCCOCCOCCOCCO[H]		-10.06	0.63	4.25	P	OUT
QL02489	multiple H-substituted, multiple ether-substituted perfluoroalkyl alcohols, n=7, m=9		FC(F)(F)C(F)(F)C(F)(F)C(F)(F)C(F)(F)C(F)(F)C(F)(F)C(F)(F)C(F)(F)C(F)(F)C(F)(F)C(F)(F)C(F)(F)C(F)(F)CCOCCOCCOCCOCCOCCOCCOCCOCCO[H]		-10.08	0.62	4.33	P	OUT
QL02490	multiple H-substituted, multiple ether-substituted perfluoroalkyl alcohols, n=7, m=10		FC(F)(F)C(F)(F)C(F)(F)C(F)(F)C(F)(F)C(F)(F)C(F)(F)C(F)(F)C(F)(F)C(F)(F)C(F)(F)C(F)(F)C(F)(F)C(F)(F)CCOCCOCCOCCOCCOCCOCCOCCOCCOCCO[H]		-10.15	0.61	4.44	P	OUT
QL02491	multiple H-substituted, multiple ether-substituted perfluoroalkyl alcohols, n=7, m=11		FC(F)(F)C(F)(F)C(F)(F)C(F)(F)C(F)(F)C(F)(F)C(F)(F)C(F)(F)C(F)(F)C(F)(F)C(F)(F)C(F)(F)C(F)(F)C(F)(F)CCOCCOCCOCCOCCOCCOCCOCCOCCOCCOCCO[H]		-10.17	0.61	4.51	P	OUT
QL02492	multiple H-substituted, multiple ether-substituted perfluoroalkyl alcohols, n=7, m=12		FC(F)(F)C(F)(F)C(F)(F)C(F)(F)C(F)(F)C(F)(F)C(F)(F)C(F)(F)C(F)(F)C(F)(F)C(F)(F)C(F)(F)C(F)(F)C(F)(F)CCOCCOCCOCCOCCOCCOCCOCCOCCOCCOCCOCCO[H]		-10.25	0.61	4.61	P	OUT
QL02493	multiple H-substituted, multiple ether-substituted perfluoroalkyl alcohols, n=7, m=13		FC(F)(F)C(F)(F)C(F)(F)C(F)(F)C(F)(F)C(F)(F)C(F)(F)C(F)(F)C(F)(F)C(F)(F)C(F)(F)C(F)(F)C(F)(F)C(F)(F)CCOCCOCCOCCOCCOCCOCCOCCOCCOCCOCCOCCOCCO[H]		-10.27	0.60	4.67	P	OUT
QL02494	multiple H-substituted, multiple ether-substituted perfluoroalkyl alcohols, n=7, m=14		FC(F)(F)C(F)(F)C(F)(F)C(F)(F)C(F)(F)C(F)(F)C(F)(F)C(F)(F)C(F)(F)C(F)(F)C(F)(F)C(F)(F)C(F)(F)C(F)(F)CCOCCOCCOCCOCCOCCOCCOCCOCCOCCOCCOCCOCCOCCO[H]		-10.34	0.60	4.76	P	OUT
QL02495	multiple H-substituted, multiple ether-substituted perfluoroalkyl alcohols, n=7, m=15		FC(F)(F)C(F)(F)C(F)(F)C(F)(F)C(F)(F)C(F)(F)C(F)(F)C(F)(F)C(F)(F)C(F)(F)C(F)(F)C(F)(F)C(F)(F)C(F)(F)CCOCCOCCOCCOCCOCCOCCOCCOCCOCCOCCOCCOCCOCCOCCO[H]		-10.37	0.60	4.82	P	OUT
QL02496	multiple H-substituted, multiple ether-substituted perfluoroalkyl alcohols, n=7, m=16		FC(F)(F)C(F)(F)C(F)(F)C(F)(F)C(F)(F)C(F)(F)C(F)(F)C(F)(F)C(F)(F)C(F)(F)C(F)(F)C(F)(F)C(F)(F)C(F)(F)CCOCCOCCOCCOCCOCCOCCOCCOCCOCCOCCOCCOCCOCCOCCOCCO[H]		-10.44	0.59	4.90	P	OUT

QL02497	multiple H-substituted, multiple ether-substituted perfluoroalkyl alcohols, n=7, m=17		FC(F)(F)C(F)(F)C(F)(F)C(F)(F)C(F)(F)C(F)(F)C(F)(F)C(F)(F)C(F)(F)C(F)(F)C(F)(F)C(F)(F)C(F)(F)C(F)(F)CCOCCOCCOCCOCCOCCOCCOCCOCCOCCOCCOCCOCCOCCOCCOCCOCCOCCO[H]		-10.47	0.59	4.96	P	OUT
QL02498	multiple H-substituted, multiple ether-substituted perfluoroalkyl alcohols, n=7, m=18		FC(F)(F)C(F)(F)C(F)(F)C(F)(F)C(F)(F)C(F)(F)C(F)(F)C(F)(F)C(F)(F)C(F)(F)C(F)(F)C(F)(F)C(F)(F)C(F)(F)CCOCCOCCOCCOCCOCCOCCOCCOCCOCCOCCOCCOCCOCCOCCOCCOCCOCCOCCO[H]		-10.54	0.59	5.03	P	OUT
QL02499	multiple H-substituted, multiple ether-substituted perfluoroalkyl alcohols, n=7, m=19		FC(F)(F)C(F)(F)C(F)(F)C(F)(F)C(F)(F)C(F)(F)C(F)(F)C(F)(F)C(F)(F)C(F)(F)C(F)(F)C(F)(F)C(F)(F)C(F)(F)CCOCCOCCOCCOCCOCCOCCOCCOCCOCCOCCOCCOCCOCCOCCOCCOCCOCCOCCOCCO[H]		-10.56	0.58	5.08	P	OUT
QL02500	multiple H-substituted, multiple ether-substituted perfluoroalkyl alcohols, n=7, m=20		FC(F)(F)C(F)(F)C(F)(F)C(F)(F)C(F)(F)C(F)(F)C(F)(F)C(F)(F)C(F)(F)C(F)(F)C(F)(F)C(F)(F)C(F)(F)C(F)(F)CCOCCOCCOCCOCCOCCOCCOCCOCCOCCOCCOCCOCCOCCOCCOCCOCCOCCOCCOCCOCCO[H]		-10.63	0.58	5.15	P	OUT
QL02501	Tris(2-(perfluorotetradecanyl)ethyl)phosphate	393098-44-7	FC(F)(F)C(F)(F)C(F)(F)C(F)(F)C(F)(F)C(F)(F)C(F)(F)C(F)(F)C(F)(F)C(F)(F)C(F)(F)C(F)(F)C(F)(F)C(F)(F)CCOP(=O)(OCCC(F)(F)C(F)(F)C(F)(F)C(F)(F)C(F)(F)C(F)(F)C(F)(F)C(F)(F)C(F)(F)C(F)(F)C(F)(F)C(F)(F)C(F)(F)C(F)(F)F)OCCC(F)(F)C(F)(F)C(F)(F)C(F)(F)C(F)(F)C(F)(F)C(F)(F)C(F)(F)C(F)(F)C(F)(F)C(F)(F)C(F)(F)C(F)(F)C(F)(F)F		-11.54	0.69	4.12	P	OUT
QL02502	n2 fluorotelomer sulfonic acids, n=14		FC(F)(F)C(F)(F)C(F)(F)C(F)(F)C(F)(F)C(F)(F)C(F)(F)C(F)(F)C(F)(F)C(F)(F)C(F)(F)C(F)(F)C(F)(F)C(F)(F)CCS(=O)(O)=O		-9.77	0.69	3.23	P	OUT
QL02503	n2 fluorotelomer sulfonamido betaine-related, n=7_pos		FC(F)(F)C(F)(F)C(F)(F)C(F)(F)C(F)(F)C(F)(F)C(F)(F)C(F)(F)C(F)(F)C(F)(F)C(F)(F)C(F)(F)C(F)(F)C(F)(F)CCS(NCCC[N+](CC(O)=O)(C)C)(=O)=O		-10.13	0.67	3.65	P	OUT
QL02504	n2 fluorotelomer thio alkyl amido betaine-related, n=7		FC(F)(F)C(F)(F)C(F)(F)C(F)(F)C(F)(F)C(F)(F)C(F)(F)C(F)(F)C(F)(F)C(F)(F)C(F)(F)C(F)(F)C(F)(F)C(F)(F)CCSCC(NCCC[N+](C)(C)CC(O)=O)=O		-10.10	0.66	3.77	P	OUT
QL02505	n2 fluorotelomer thio alkyl amido sulfonic acids, n=7		FC(F)(F)C(F)(F)C(F)(F)C(F)(F)C(F)(F)C(F)(F)C(F)(F)C(F)(F)C(F)(F)C(F)(F)C(F)(F)C(F)(F)C(F)(F)C(F)(F)CCSCCC(NC(C)(C)CS(O)(=O)=O)=O		-10.10	0.67	3.65	P	OUT
QL02506	Perfluorotetradecane	307-62-0	FC(F)(F)C(F)(F)C(F)(F)C(F)(F)C(F)(F)C(F)(F)C(F)(F)C(F)(F)C(F)(F)C(F)(F)C(F)(F)C(F)(F)C(F)(F)C(F)(F)F		-9.43	0.69	2.95	P	OUT
QL02507	3,3,4,4,5,5,6,6,7,7,8,8,9,9,10,10,11,11,12,12,13,13,14,14,15,15,15-Heptacosafluoropentadec-1-ene	1270180-04-5	FC(F)(F)C(F)(F)C(F)(F)C(F)(F)C(F)(F)C(F)(F)C(F)(F)C(F)(F)C(F)(F)C(F)(F)C(F)(F)C(F)(F)C(F)(F)C=C		-9.23	0.69	2.94	P	OUT
QL02508	1,1,1,2,2,3,3,4,4,5,5,6,6,7,7,8,8,9,9,10,10,11,11,12,12,13,13-Heptacosafluoro-15-iodopentadecane	146983-96-2	FC(F)(F)C(F)(F)C(F)(F)C(F)(F)C(F)(F)C(F)(F)C(F)(F)C(F)(F)C(F)(F)C(F)(F)C(F)(F)C(F)(F)C(F)(F)CCI		-9.29	0.68	3.06	P	OUT
QL02509	n2 perfluoroalkyl acrylates, n=13		FC(F)(F)C(F)(F)C(F)(F)C(F)(F)C(F)(F)C(F)(F)C(F)(F)C(F)(F)C(F)(F)C(F)(F)C(F)(F)C(F)(F)C(F)(F)CCOC(CC(C)=C)=O		-9.61	0.67	3.37	P	OUT
QL02510	Perfluorotridecane	376-03-4	FC(F)(F)C(F)(F)C(F)(F)C(F)(F)C(F)(F)C(F)(F)C(F)(F)C(F)(F)C(F)(F)C(F)(F)C(F)(F)C(F)(F)C(F)(F)F		-9.32	0.69	2.89	P	OUT
QL02511	Perfluorotridecyl iodide	376-04-5	FC(F)(F)C(F)(F)C(F)(F)C(F)(F)C(F)(F)C(F)(F)C(F)(F)C(F)(F)C(F)(F)C(F)(F)C(F)(F)C(F)(F)C(F)(F)I		-9.32	0.69	2.89	P	OUT
QL02512	1,1,1,2,2,3,3,4,4,5,5,6,6,7,7,8,8,9,9,10,10,11,11,12,12,19,19,20,20,21,21,22,22,23,23,24,24,25,25,26,26,27,27,28,28,29,29,30,30,30-Pentacontafluoro-14,17-diiodotriacontane	114221-75-9	FC(F)(F)C(F)(F)C(F)(F)C(F)(F)C(F)(F)C(F)(F)C(F)(F)C(F)(F)C(F)(F)C(F)(F)C(F)(F)C(F)(F)CC(I)CCC(I)CC(F)(F)C(F)(F)C(F)(F)C(F)(F)C(F)(F)C(F)(F)C(F)(F)C(F)(F)C(F)(F)C(F)(F)C(F)(F)C(F)(F)F		-11.05	0.68	3.92	P	OUT
QL02513	1,8-Bis(perfluorododecyl)-2,7-diiodooctane	1980044-62-9	FC(F)(F)C(F)(F)C(F)(F)C(F)(F)C(F)(F)C(F)(F)C(F)(F)C(F)(F)C(F)(F)C(F)(F)C(F)(F)C(F)(F)CC(I)CCCCC(I)CC(F)(F)C(F)(F)C(F)(F)C(F)(F)C(F)(F)C(F)(F)C(F)(F)C(F)(F)C(F)(F)C(F)(F)C(F)(F)C(F)(F)F		-11.07	0.68	3.99	P	OUT
QL02514	1,1,1,2,2,3,3,4,4,5,5,6,6,7,7,8,8,9,9,10,10,11,11,12,12,25,25,26,26,27,27,28,28,29,29,30,30,31,31,32,32,33,33,34,34,35,35,36,36,36-Pentacontafluoro-14,23-diiodohexatriacontane	1980039-13-1	FC(F)(F)C(F)(F)C(F)(F)C(F)(F)C(F)(F)C(F)(F)C(F)(F)C(F)(F)C(F)(F)C(F)(F)C(F)(F)C(F)(F)CC(I)CCCCCCCCC(I)CC(F)(F)C(F)(F)C(F)(F)C(F)(F)C(F)(F)C(F)(F)C(F)(F)C(F)(F)C(F)(F)C(F)(F)C(F)(F)C(F)(F)F		-11.10	0.67	4.12	P	OUT
QL02515	1,1,1,2,2,3,3,4,4,5,5,6,6,7,7,8,8,9,9,10,10,11,11,12,12,35,35,36,36,37,37,38,38,39,39,40,40,41,41,42,42,43,43,44,44,45,45,46,46,46-Pentacontafluoro-14,33-diiodohexatetracontane	114221-76-0	FC(F)(F)C(F)(F)C(F)(F)C(F)(F)C(F)(F)C(F)(F)C(F)(F)C(F)(F)C(F)(F)C(F)(F)C(F)(F)C(F)(F)CC(I)CCCCCCCCCCCCCCCCCCC(I)CC(F)(F)C(F)(F)C(F)(F)C(F)(F)C(F)(F)C(F)(F)C(F)(F)C(F)(F)C(F)(F)C(F)(F)C(F)(F)C(F)(F)F		-11.19	0.65	4.41	P	OUT
QL02516	1H,1H,2H,2H-Perfluorotetradecyltrichlorosilane	102488-50-6	FC(F)(F)C(F)(F)C(F)(F)C(F)(F)C(F)(F)C(F)(F)C(F)(F)C(F)(F)C(F)(F)C(F)(F)C(F)(F)C(F)(F)CC[Si](Cl)(Cl)Cl		-9.41	0.69	3.06	P	OUT
QL02517	((Perfluorododecyl)methyl)oxirane	94158-66-4	FC(F)(F)C(F)(F)C(F)(F)C(F)(F)C(F)(F)C(F)(F)C(F)(F)C(F)(F)C(F)(F)C(F)(F)C(F)(F)C(F)(F)CC1CO1		-9.16	0.69	2.94	P	OUT
QL02518	1,1,1,2,2,3,3,4,4,5,5,6,6,7,7,8,8,9,9,10,10,11,11,12,12,19,19,20,20,21,21,22,22,23,23,24,24,25,25,26,26,27,27,28,28,29,29,30,30,30-Pentacontafluorotriacontane	114221-77-1	FC(F)(F)C(F)(F)C(F)(F)C(F)(F)C(F)(F)C(F)(F)C(F)(F)C(F)(F)C(F)(F)C(F)(F)C(F)(F)C(F)(F)CCCCCCC(F)(F)C(F)(F)C(F)(F)C(F)(F)C(F)(F)C(F)(F)C(F)(F)C(F)(F)C(F)(F)C(F)(F)C(F)(F)C(F)(F)F		-10.99	0.68	3.90	P	OUT

QL02519	1,1,1,2,2,3,3,4,4,5,5,6,6,7,7,8,8,9,9,10,10,11,11,12,12,21,21,22,22,23,23,24,24,25,25,26,26,27,27,28,28,29,29,30,30,31,31,32,32,32-Pentacontafluorodotriacontane	100550-09-2	FC(F)(F)C(F)(F)C(F)(F)C(F)(F)C(F)(F)C(F)(F)C(F)(F)C(F)(F)C(F)(F)C(F)(F)C(F)(F)C(F)(F)CCCCCCCCC(F)(F)C(F)(F)C(F)(F)C(F)(F)C(F)(F)C(F)(F)C(F)(F)C(F)(F)C(F)(F)C(F)(F)C(F)(F)C(F)(F)F		-11.00	0.68	3.97	P	OUT
QL02520	1,1,1,2,2,3,3,4,4,5,5,6,6,7,7,8,8,9,9,10,10,11,11,12,12,23,23,24,24,25,25,26,26,27,27,28,28,29,29,30,30,31,31,32,32,33,33,34,34,34-Pentacontafluorotetratriacontane	100550-10-5	FC(F)(F)C(F)(F)C(F)(F)C(F)(F)C(F)(F)C(F)(F)C(F)(F)C(F)(F)C(F)(F)C(F)(F)C(F)(F)C(F)(F)CCCCCCCCCCC(F)(F)C(F)(F)C(F)(F)C(F)(F)C(F)(F)C(F)(F)C(F)(F)C(F)(F)C(F)(F)C(F)(F)C(F)(F)C(F)(F)F		-11.02	0.67	4.03	P	OUT
QL02521	1,1,1,2,2,3,3,4,4,5,5,6,6,7,7,8,8,9,9,10,10,11,11,12,12,25,25,26,26,27,27,28,28,29,29,30,30,31,31,32,32,33,33,34,34,35,35,36,36,36-Pentacontafluorohexatriacontane	100550-11-6	FC(F)(F)C(F)(F)C(F)(F)C(F)(F)C(F)(F)C(F)(F)C(F)(F)C(F)(F)C(F)(F)C(F)(F)C(F)(F)C(F)(F)CCCCCCCCCCCCC(F)(F)C(F)(F)C(F)(F)C(F)(F)C(F)(F)C(F)(F)C(F)(F)C(F)(F)C(F)(F)C(F)(F)C(F)(F)C(F)(F)F		-11.04	0.67	4.10	P	OUT
QL02522	1,1,1,2,2,3,3,4,4,5,5,6,6,7,7,8,8,9,9,10,10,11,11,12,12,33,33,34,34,35,35,36,36,37,37,38,38,39,39,40,40,41,41,42,42,43,43,44,44,44-Pentacontafluorotetratetracontane	114221-78-2	FC(F)(F)C(F)(F)C(F)(F)C(F)(F)C(F)(F)C(F)(F)C(F)(F)C(F)(F)C(F)(F)C(F)(F)C(F)(F)C(F)(F)CCCCCCCCCCCCCCCCCCCCC(F)(F)C(F)(F)C(F)(F)C(F)(F)C(F)(F)C(F)(F)C(F)(F)C(F)(F)C(F)(F)C(F)(F)C(F)(F)C(F)(F)F		-11.11	0.66	4.34	P	OUT
QL02523	1,1,1,2,2,3,3,4,4,5,5,6,6,7,7,8,8,9,9,10,10,11,11,12,12,35,35,36,36,37,37,38,38,39,39,40,40,41,41,42,42,43,43,44,44,45,45,46,46,46-Pentacontafluorohexatetracontane	114221-79-3	FC(F)(F)C(F)(F)C(F)(F)C(F)(F)C(F)(F)C(F)(F)C(F)(F)C(F)(F)C(F)(F)C(F)(F)C(F)(F)C(F)(F)CCCCCCCCCCCCCCCCCCCCCCC(F)(F)C(F)(F)C(F)(F)C(F)(F)C(F)(F)C(F)(F)C(F)(F)C(F)(F)C(F)(F)C(F)(F)C(F)(F)C(F)(F)F		-11.13	0.65	4.40	P	OUT
QL02524	n2 perfluoroalkyl acrylates, n=12		FC(F)(F)C(F)(F)C(F)(F)C(F)(F)C(F)(F)C(F)(F)C(F)(F)C(F)(F)C(F)(F)C(F)(F)C(F)(F)C(F)(F)CCOC(CC(C)=C)=O		-9.35	0.67	3.27	P	OUT
QL02525	multiple H-substituted, multiple ether-substituted perfluoroalkyl alcohols, n=6, m=5		FC(F)(F)C(F)(F)C(F)(F)C(F)(F)C(F)(F)C(F)(F)C(F)(F)C(F)(F)C(F)(F)C(F)(F)C(F)(F)C(F)(F)CCOCCOCCOCCOCCO[H]		-9.50	0.64	3.78	P	OUT
QL02526	multiple H-substituted, multiple ether-substituted perfluoroalkyl alcohols, n=6, m=6		FC(F)(F)C(F)(F)C(F)(F)C(F)(F)C(F)(F)C(F)(F)C(F)(F)C(F)(F)C(F)(F)C(F)(F)C(F)(F)C(F)(F)CCOCCOCCOCCOCCOCCO[H]		-9.59	0.63	3.93	P	OUT
QL02527	multiple H-substituted, multiple ether-substituted perfluoroalkyl alcohols, n=6, m=7		FC(F)(F)C(F)(F)C(F)(F)C(F)(F)C(F)(F)C(F)(F)C(F)(F)C(F)(F)C(F)(F)C(F)(F)C(F)(F)C(F)(F)CCOCCOCCOCCOCCOCCOCCO[H]		-9.61	0.62	4.03	P	OUT
QL02528	multiple H-substituted, multiple ether-substituted perfluoroalkyl alcohols, n=6, m=8		FC(F)(F)C(F)(F)C(F)(F)C(F)(F)C(F)(F)C(F)(F)C(F)(F)C(F)(F)C(F)(F)C(F)(F)C(F)(F)C(F)(F)CCOCCOCCOCCOCCOCCOCCOCCO[H]		-9.69	0.62	4.16	P	OUT
QL02529	multiple H-substituted, multiple ether-substituted perfluoroalkyl alcohols, n=6, m=9		FC(F)(F)C(F)(F)C(F)(F)C(F)(F)C(F)(F)C(F)(F)C(F)(F)C(F)(F)C(F)(F)C(F)(F)C(F)(F)C(F)(F)CCOCCOCCOCCOCCOCCOCCOCCOCCO[H]		-9.72	0.61	4.25	P	OUT
QL02530	multiple H-substituted, multiple ether-substituted perfluoroalkyl alcohols, n=6, m=10		FC(F)(F)C(F)(F)C(F)(F)C(F)(F)C(F)(F)C(F)(F)C(F)(F)C(F)(F)C(F)(F)C(F)(F)C(F)(F)C(F)(F)CCOCCOCCOCCOCCOCCOCCOCCOCCOCCO[H]		-9.80	0.61	4.36	P	OUT
QL02531	multiple H-substituted, multiple ether-substituted perfluoroalkyl alcohols, n=6, m=11		FC(F)(F)C(F)(F)C(F)(F)C(F)(F)C(F)(F)C(F)(F)C(F)(F)C(F)(F)C(F)(F)C(F)(F)C(F)(F)C(F)(F)CCOCCOCCOCCOCCOCCOCCOCCOCCOCCOCCO[H]		-9.83	0.60	4.44	P	OUT
QL02532	multiple H-substituted, multiple ether-substituted perfluoroalkyl alcohols, n=6, m=12		FC(F)(F)C(F)(F)C(F)(F)C(F)(F)C(F)(F)C(F)(F)C(F)(F)C(F)(F)C(F)(F)C(F)(F)C(F)(F)C(F)(F)CCOCCOCCOCCOCCOCCOCCOCCOCCOCCOCCOCCO[H]		-9.91	0.60	4.55	P	OUT
QL02533	multiple H-substituted, multiple ether-substituted perfluoroalkyl alcohols, n=6, m=13		FC(F)(F)C(F)(F)C(F)(F)C(F)(F)C(F)(F)C(F)(F)C(F)(F)C(F)(F)C(F)(F)C(F)(F)C(F)(F)C(F)(F)CCOCCOCCOCCOCCOCCOCCOCCOCCOCCOCCOCCOCCO[H]		-9.94	0.59	4.62	P	OUT
QL02534	multiple H-substituted, multiple ether-substituted perfluoroalkyl alcohols, n=6, m=14		FC(F)(F)C(F)(F)C(F)(F)C(F)(F)C(F)(F)C(F)(F)C(F)(F)C(F)(F)C(F)(F)C(F)(F)C(F)(F)C(F)(F)CCOCCOCCOCCOCCOCCOCCOCCOCCOCCOCCOCCOCCOCCO[H]		-10.03	0.59	4.71	P	OUT
QL02535	multiple H-substituted, multiple ether-substituted perfluoroalkyl alcohols, n=6, m=15		FC(F)(F)C(F)(F)C(F)(F)C(F)(F)C(F)(F)C(F)(F)C(F)(F)C(F)(F)C(F)(F)C(F)(F)C(F)(F)C(F)(F)CCOCCOCCOCCOCCOCCOCCOCCOCCOCCOCCOCCOCCOCCOCCO[H]		-10.06	0.59	4.77	P	OUT
QL02536	multiple H-substituted, multiple ether-substituted perfluoroalkyl alcohols, n=6, m=16		FC(F)(F)C(F)(F)C(F)(F)C(F)(F)C(F)(F)C(F)(F)C(F)(F)C(F)(F)C(F)(F)C(F)(F)C(F)(F)C(F)(F)CCOCCOCCOCCOCCOCCOCCOCCOCCOCCOCCOCCOCCOCCOCCOCCO[H]		-10.14	0.58	4.86	P	OUT
QL02537	multiple H-substituted, multiple ether-substituted perfluoroalkyl alcohols, n=6, m=17		FC(F)(F)C(F)(F)C(F)(F)C(F)(F)C(F)(F)C(F)(F)C(F)(F)C(F)(F)C(F)(F)C(F)(F)C(F)(F)C(F)(F)CCOCCOCCOCCOCCOCCOCCOCCOCCOCCOCCOCCOCCOCCOCCOCCOCCO[H]		-10.17	0.58	4.91	P	OUT
QL02538	multiple H-substituted, multiple ether-substituted perfluoroalkyl alcohols, n=6, m=18		FC(F)(F)C(F)(F)C(F)(F)C(F)(F)C(F)(F)C(F)(F)C(F)(F)C(F)(F)C(F)(F)C(F)(F)C(F)(F)C(F)(F)CCOCCOCCOCCOCCOCCOCCOCCOCCOCCOCCOCCOCCOCCOCCOCCOCCOCCO[H]		-10.25	0.58	4.99	P	OUT
QL02539	multiple H-substituted, multiple ether-substituted perfluoroalkyl alcohols, n=6, m=19		FC(F)(F)C(F)(F)C(F)(F)C(F)(F)C(F)(F)C(F)(F)C(F)(F)C(F)(F)C(F)(F)C(F)(F)C(F)(F)C(F)(F)CCOCCOCCOCCOCCOCCOCCOCCOCCOCCOCCOCCOCCOCCOCCOCCOCCOCCOCCO[H]		-10.28	0.58	5.04	P	OUT
QL02540	multiple H-substituted, multiple ether-substituted perfluoroalkyl alcohols, n=6, m=20		FC(F)(F)C(F)(F)C(F)(F)C(F)(F)C(F)(F)C(F)(F)C(F)(F)C(F)(F)C(F)(F)C(F)(F)C(F)(F)C(F)(F)CCOCCOCCOCCOCCOCCOCCOCCOCCOCCOCCOCCOCCOCCOCCOCCOCCOCCOCCOCCO[H]		-10.36	0.57	5.12	P	OUT

QL02541	n2 fluorotelomer sulfonic acids, n=12		FC(F)(F)C(F)(F)C(F)(F)C(F)(F)C(F)(F)C(F)(F)C(F)(F)C(F)(F)C(F)(F)C(F)(F)C(F)(F)CCS(=O)(O)=O		-9.41	0.69	3.06	P	OUT
QL02542	n2 fluorotelomer sulfonamido betaine-related, n=6_pos		FC(F)(F)C(F)(F)C(F)(F)C(F)(F)C(F)(F)C(F)(F)C(F)(F)C(F)(F)C(F)(F)C(F)(F)C(F)(F)CCS(NCCC[N+](CC(O)=O)(C)C)(=O)=O		-9.81	0.67	3.54	P	OUT
QL02543	n2 fluorotelomer sulfonamido amines, n=6		FC(F)(F)C(F)(F)C(F)(F)C(F)(F)C(F)(F)C(F)(F)C(F)(F)C(F)(F)C(F)(F)C(F)(F)C(F)(F)CCS(NCCCN(C)C)(=O)=O		-9.58	0.67	3.41	P	OUT
QL02544	n2 fluorotelomer thio alkyl amido betaine-related, n=6		FC(F)(F)C(F)(F)C(F)(F)C(F)(F)C(F)(F)C(F)(F)C(F)(F)C(F)(F)C(F)(F)C(F)(F)C(F)(F)CCSCC(NCCC[N+](C)(C)CC(O)=O)=O		-9.78	0.66	3.67	P	OUT
QL02545	n2 fluorotelomer thio alkyl amido ammonium, n=6		FC(F)(F)C(F)(F)C(F)(F)C(F)(F)C(F)(F)C(F)(F)C(F)(F)C(F)(F)C(F)(F)C(F)(F)C(F)(F)CCSCC(NCCCN(C)C)=O		-9.55	0.66	3.55	P	OUT
QL02546	n2 fluorotelomer thio alkyl amido sulfonic acids, n=6		FC(F)(F)C(F)(F)C(F)(F)C(F)(F)C(F)(F)C(F)(F)C(F)(F)C(F)(F)C(F)(F)C(F)(F)C(F)(F)CCSCCC(NC(C)(C)CS(O)(=O)=O)=O		-9.78	0.67	3.54	P	OUT
QL02547	Perfluorododecane	307-59-5	FC(F)(F)C(F)(F)C(F)(F)C(F)(F)C(F)(F)C(F)(F)C(F)(F)C(F)(F)C(F)(F)C(F)(F)C(F)(F)C(F)(F)F		-9.02	0.69	2.75	P	OUT
QL02548	3,3,4,4,5,5,6,6,7,7,8,8,9,9,10,10,11,11,12,12,13,13,13-Tricosafluorotridec-1-ene	1270180-02-3	FC(F)(F)C(F)(F)C(F)(F)C(F)(F)C(F)(F)C(F)(F)C(F)(F)C(F)(F)C(F)(F)C(F)(F)C(F)(F)C=C		-8.78	0.69	2.74	P	IN
QL02549	1,1,1,2,2,3,3,4,4,5,5,6,6,7,7,8,8,9,9,10,10,11,11-Tricosafluoro-13-iodotridecane	57216-88-3	FC(F)(F)C(F)(F)C(F)(F)C(F)(F)C(F)(F)C(F)(F)C(F)(F)C(F)(F)C(F)(F)C(F)(F)C(F)(F)CCI		-8.85	0.68	2.88	P	OUT
QL02550	n2 perfluoroalkyl acrylates, n=11		FC(F)(F)C(F)(F)C(F)(F)C(F)(F)C(F)(F)C(F)(F)C(F)(F)C(F)(F)C(F)(F)C(F)(F)C(F)(F)CCOC(CC(C)=C)=O		-9.23	0.67	3.23	P	OUT
QL02551	multiple H-substituted-ether-substituted-perfluoroalkyl (linear) carboxylic acids, n=11		FC(F)(F)C(F)(F)C(F)(F)C(F)(F)C(F)(F)C(F)(F)C(F)(F)C(F)(F)C(F)(F)C(F)(F)C(F)(F)COCC(O)=O		-9.09	0.67	3.07	P	OUT
QL02552	2,2,3,3,4,4,5,5,6,6,7,7,8,8,9,9,10,10,11,11,12,12,12-Tricosafluorododecyl nonafluorobutane-1-sulfonate	924894-11-1	FC(F)(F)C(F)(F)C(F)(F)C(F)(F)C(F)(F)C(F)(F)C(F)(F)C(F)(F)C(F)(F)C(F)(F)C(F)(F)COS(=O)(=O)C(F)(F)C(F)(F)C(F)(F)C(F)(F)F		-9.94	0.69	3.27	P	OUT
QL02553	Perfluoroundecane	86508-42-1	FC(F)(F)C(F)(F)C(F)(F)C(F)(F)C(F)(F)C(F)(F)C(F)(F)C(F)(F)C(F)(F)C(F)(F)C(F)(F)F		-8.88	0.69	2.69	P	OUT
QL02554	2,2,3,3,4,4,5,5,6,6,7,7,8,8,9,9,10,10,11,11,11-Tricosafluoro-1-iodoundecane	307-50-6	FC(F)(F)C(F)(F)C(F)(F)C(F)(F)C(F)(F)C(F)(F)C(F)(F)C(F)(F)C(F)(F)C(F)(F)C(F)(F)I		-8.88	0.69	2.69	P	OUT
QL02555	2-(Henicosafluorodecyl)-1H-imidazole	81769-51-9	FC(F)(F)C(F)(F)C(F)(F)C(F)(F)C(F)(F)C(F)(F)C(F)(F)C(F)(F)C(F)(F)C(F)(F)c1Nccn1		-8.55	0.68	2.75	P	IN
QL02556	5-(Henicosafluorodecyl)-1H-imidazole	81769-57-5	FC(F)(F)C(F)(F)C(F)(F)C(F)(F)C(F)(F)C(F)(F)C(F)(F)C(F)(F)C(F)(F)C(F)(F)c1Ncnc1		-8.55	0.68	2.75	P	IN
QL02557	1,1,1,2,2,3,3,4,4,5,5,6,6,7,7,8,8,9,9,10,10,17,17,18,18,19,19,20,20,21,21,22,22,23,23,24,24,25,25,26,26,26-Dotetracontafluoro-12,15-diiodohexacosane	1980086-18-7	FC(F)(F)C(F)(F)C(F)(F)C(F)(F)C(F)(F)C(F)(F)C(F)(F)C(F)(F)C(F)(F)C(F)(F)CC(I)CCC(I)CC(F)(F)C(F)(F)C(F)(F)C(F)(F)C(F)(F)C(F)(F)C(F)(F)C(F)(F)C(F)(F)C(F)(F)F		-10.58	0.68	3.71	P	OUT
QL02558	1,1,1,2,2,3,3,4,4,5,5,6,6,7,7,8,8,9,9,10,10,18,18,19,19,20,20,21,21,22,22,23,23,24,24,25,25,26,26,27,27,27-Dotetracontafluoro-12,16-diiodoheptacosane	784189-62-4	FC(F)(F)C(F)(F)C(F)(F)C(F)(F)C(F)(F)C(F)(F)C(F)(F)C(F)(F)C(F)(F)C(F)(F)CC(I)CCCC(I)CC(F)(F)C(F)(F)C(F)(F)C(F)(F)C(F)(F)C(F)(F)C(F)(F)C(F)(F)C(F)(F)C(F)(F)F		-10.62	0.68	3.77	P	OUT
QL02559	1,1,1,2,2,3,3,4,4,5,5,6,6,7,7,8,8,9,9,10,10,19,19,20,20,21,21,22,22,23,23,24,24,25,25,26,26,27,27,28,28,28-Dotetracontafluoro-12,17-diiodooctacosane	1980064-30-9	FC(F)(F)C(F)(F)C(F)(F)C(F)(F)C(F)(F)C(F)(F)C(F)(F)C(F)(F)C(F)(F)C(F)(F)CC(I)CCCCC(I)CC(F)(F)C(F)(F)C(F)(F)C(F)(F)C(F)(F)C(F)(F)C(F)(F)C(F)(F)C(F)(F)C(F)(F)F		-10.60	0.68	3.79	P	OUT
QL02560	1,12-Bis(perfluorodecyl)-2,11-diiodododecane	1980048-92-7	FC(F)(F)C(F)(F)C(F)(F)C(F)(F)C(F)(F)C(F)(F)C(F)(F)C(F)(F)C(F)(F)C(F)(F)CC(I)CCCCCCCCC(I)CC(F)(F)C(F)(F)C(F)(F)C(F)(F)C(F)(F)C(F)(F)C(F)(F)C(F)(F)C(F)(F)C(F)(F)F		-10.64	0.67	3.94	P	OUT
QL02561	1H,1H,2H,2H-Perfluorododecyltrichlorosilane	102488-49-3	FC(F)(F)C(F)(F)C(F)(F)C(F)(F)C(F)(F)C(F)(F)C(F)(F)C(F)(F)C(F)(F)C(F)(F)CC[Si](Cl)(Cl)Cl		-8.99	0.68	2.88	P	OUT
QL02562	((Perfluorodecyl)methyl)oxirane	38565-54-7	FC(F)(F)C(F)(F)C(F)(F)C(F)(F)C(F)(F)C(F)(F)C(F)(F)C(F)(F)C(F)(F)C(F)(F)CC1CO1		-8.70	0.68	2.74	P	IN
QL02563	12-Bromo-1,1,1,2,2,3,3,4,4,5,5,6,6,7,7,8,8,9,9,10,10-henicosafluorododecane	332136-76-2	FC(F)(F)C(F)(F)C(F)(F)C(F)(F)C(F)(F)C(F)(F)C(F)(F)C(F)(F)C(F)(F)C(F)(F)CCBr		-8.50	0.68	2.73	P	IN
QL02564	4,4,5,5,6,6,7,7,8,8,9,9,10,10,11,11,12,12,13,13,13-Henicosafluorotridecanenitrile	140834-59-9	FC(F)(F)C(F)(F)C(F)(F)C(F)(F)C(F)(F)C(F)(F)C(F)(F)C(F)(F)C(F)(F)C(F)(F)CCC#N		-8.66	0.67	2.89	P	IN
QL02565	1,1,1,2,2,3,3,4,4,5,5,6,6,7,7,8,8,9,9,10,10,17,17,18,18,19,19,20,20,21,21,22,22,23,23,24,24,25,25,26,26,26-Dotetracontafluorohexacosane	391894-95-4	FC(F)(F)C(F)(F)C(F)(F)C(F)(F)C(F)(F)C(F)(F)C(F)(F)C(F)(F)C(F)(F)C(F)(F)CCCCCC(F)(F)C(F)(F)C(F)(F)C(F)(F)C(F)(F)C(F)(F)C(F)(F)C(F)(F)C(F)(F)C(F)(F)F		-10.51	0.68	3.69	P	OUT
QL02566	1,1,1,2,2,3,3,4,4,5,5,6,6,7,7,8,8,9,9,10,10,19,19,20,20,21,21,22,22,23,23,24,24,25,25,26,26,27,27,28,28,28-Dotetracontafluorooctacosane	115249-34-8	FC(F)(F)C(F)(F)C(F)(F)C(F)(F)C(F)(F)C(F)(F)C(F)(F)C(F)(F)C(F)(F)C(F)(F)CCCCCCCCC(F)(F)C(F)(F)C(F)(F)C(F)(F)C(F)(F)C(F)(F)C(F)(F)C(F)(F)C(F)(F)C(F)(F)F		-10.52	0.68	3.77	P	OUT
QL02567	1,1,1,2,2,3,3,4,4,5,5,6,6,7,7,8,8,9,9,10,10,21,21,22,22,23,23,24,24,25,25,26,26,27,27,28,28,29,29,30,30,30-Dotetracontafluorotriacontane	135588-58-8	FC(F)(F)C(F)(F)C(F)(F)C(F)(F)C(F)(F)C(F)(F)C(F)(F)C(F)(F)C(F)(F)C(F)(F)CCCCCCCCCCC(F)(F)C(F)(F)C(F)(F)C(F)(F)C(F)(F)C(F)(F)C(F)(F)C(F)(F)C(F)(F)C(F)(F)F		-10.54	0.67	3.85	P	OUT

QL02568	1,12-Bis(perfluorodecyl)-n-dodecane	1980039-46-0	FC(F)(F)C(F)(F)C(F)(F)C(F)(F)C(F)(F)C(F)(F)C(F)(F)C(F)(F)C(F)(F)C(F)(F)CCCCCCCCCCCCC(F)(F)C(F)(F)C(F)(F)C(F)(F)C(F)(F)C(F)(F)C(F)(F)C(F)(F)C(F)(F)C(F)(F)F		-10.56	0.67	3.92	P	OUT
QL02569	1,1,1,2,2,3,3,4,4,5,5,6,6,7,7,8,8,9,9,10,10-Henicosafluoro-13-iodotridecane	541547-94-8	FC(F)(F)C(F)(F)C(F)(F)C(F)(F)C(F)(F)C(F)(F)C(F)(F)C(F)(F)C(F)(F)C(F)(F)CCCI		-8.66	0.67	2.89	P	IN
QL02570	12-Azido-1,1,1,2,2,3,3,4,4,5,5,6,6,7,7,8,8,9,9,10,10-henicosafluorododecane	145876-00-2	FC(F)(F)C(F)(F)C(F)(F)C(F)(F)C(F)(F)C(F)(F)C(F)(F)C(F)(F)C(F)(F)C(F)(F)CCN=[N+]=[N-]		-8.62	0.67	2.93	P	IN
QL02571	102 Fluorotelomer acrylate	17741-60-5	FC(F)(F)C(F)(F)C(F)(F)C(F)(F)C(F)(F)C(F)(F)C(F)(F)C(F)(F)C(F)(F)C(F)(F)CCOC(=O)C=C		-8.90	0.67	3.07	P	OUT
QL02572	2-(Perfluorodecyl)ethyl N-1-naphthalenylcarbamate	305849-27-8	FC(F)(F)C(F)(F)C(F)(F)C(F)(F)C(F)(F)C(F)(F)C(F)(F)C(F)(F)C(F)(F)C(F)(F)CCOC(=O)NC1=CC=CC2=CC=CC=C12		-9.11	0.65	3.38	P	OUT
QL02573	3,3,4,4,5,5,6,6,7,7,8,8,9,9,10,10,11,11,12,12,12-Henicosafluorododecyl phenylcarbamate	305849-13-2	FC(F)(F)C(F)(F)C(F)(F)C(F)(F)C(F)(F)C(F)(F)C(F)(F)C(F)(F)C(F)(F)C(F)(F)CCOC(=O)Nc1ccccc1		-9.00	0.66	3.28	P	OUT
QL02574	n2 perfluoroalkyl acrylates, n=10		FC(F)(F)C(F)(F)C(F)(F)C(F)(F)C(F)(F)C(F)(F)C(F)(F)C(F)(F)C(F)(F)C(F)(F)CCOC(CC(C)=C)=O		-8.93	0.66	3.12	P	OUT
QL02575	multiple H-substituted, multiple ether-substituted perfluoroalkyl alcohols, n=5, m=5		FC(F)(F)C(F)(F)C(F)(F)C(F)(F)C(F)(F)C(F)(F)C(F)(F)C(F)(F)C(F)(F)C(F)(F)CCOCCOCCOCCOCCO[H]		-9.04	0.63	3.65	P	OUT
QL02576	multiple H-substituted, multiple ether-substituted perfluoroalkyl alcohols, n=5, m=6		FC(F)(F)C(F)(F)C(F)(F)C(F)(F)C(F)(F)C(F)(F)C(F)(F)C(F)(F)C(F)(F)C(F)(F)CCOCCOCCOCCOCCOCCO[H]		-9.14	0.62	3.82	P	OUT
QL02577	multiple H-substituted, multiple ether-substituted perfluoroalkyl alcohols, n=5, m=7		FC(F)(F)C(F)(F)C(F)(F)C(F)(F)C(F)(F)C(F)(F)C(F)(F)C(F)(F)C(F)(F)C(F)(F)CCOCCOCCOCCOCCOCCOCCO[H]		-9.17	0.61	3.93	P	OUT
QL02578	multiple H-substituted, multiple ether-substituted perfluoroalkyl alcohols, n=5, m=8		FC(F)(F)C(F)(F)C(F)(F)C(F)(F)C(F)(F)C(F)(F)C(F)(F)C(F)(F)C(F)(F)C(F)(F)CCOCCOCCOCCOCCOCCOCCOCCO[H]		-9.27	0.61	4.07	P	OUT
QL02579	multiple H-substituted, multiple ether-substituted perfluoroalkyl alcohols, n=5, m=9		FC(F)(F)C(F)(F)C(F)(F)C(F)(F)C(F)(F)C(F)(F)C(F)(F)C(F)(F)C(F)(F)C(F)(F)CCOCCOCCOCCOCCOCCOCCOCCOCCO[H]		-9.31	0.60	4.17	P	OUT
QL02580	multiple H-substituted, multiple ether-substituted perfluoroalkyl alcohols, n=5, m=10		FC(F)(F)C(F)(F)C(F)(F)C(F)(F)C(F)(F)C(F)(F)C(F)(F)C(F)(F)C(F)(F)C(F)(F)CCOCCOCCOCCOCCOCCOCCOCCOCCOCCO[H]		-9.41	0.60	4.29	P	OUT
QL02581	multiple H-substituted, multiple ether-substituted perfluoroalkyl alcohols, n=5, m=11		FC(F)(F)C(F)(F)C(F)(F)C(F)(F)C(F)(F)C(F)(F)C(F)(F)C(F)(F)C(F)(F)C(F)(F)CCOCCOCCOCCOCCOCCOCCOCCOCCOCCOCCO[H]		-9.44	0.59	4.38	P	OUT
QL02582	multiple H-substituted, multiple ether-substituted perfluoroalkyl alcohols, n=5, m=12		FC(F)(F)C(F)(F)C(F)(F)C(F)(F)C(F)(F)C(F)(F)C(F)(F)C(F)(F)C(F)(F)C(F)(F)CCOCCOCCOCCOCCOCCOCCOCCOCCOCCOCCOCCO[H]		-9.54	0.59	4.48	P	OUT
QL02583	multiple H-substituted, multiple ether-substituted perfluoroalkyl alcohols, n=5, m=13		FC(F)(F)C(F)(F)C(F)(F)C(F)(F)C(F)(F)C(F)(F)C(F)(F)C(F)(F)C(F)(F)C(F)(F)CCOCCOCCOCCOCCOCCOCCOCCOCCOCCOCCOCCOCCO[H]		-9.58	0.59	4.56	P	OUT
QL02584	multiple H-substituted, multiple ether-substituted perfluoroalkyl alcohols, n=5, m=14		FC(F)(F)C(F)(F)C(F)(F)C(F)(F)C(F)(F)C(F)(F)C(F)(F)C(F)(F)C(F)(F)C(F)(F)CCOCCOCCOCCOCCOCCOCCOCCOCCOCCOCCOCCOCCOCCO[H]		-9.67	0.58	4.66	P	OUT
QL02585	multiple H-substituted, multiple ether-substituted perfluoroalkyl alcohols, n=5, m=15		FC(F)(F)C(F)(F)C(F)(F)C(F)(F)C(F)(F)C(F)(F)C(F)(F)C(F)(F)C(F)(F)C(F)(F)CCOCCOCCOCCOCCOCCOCCOCCOCCOCCOCCOCCOCCOCCOCCO[H]		-9.71	0.58	4.72	P	OUT
QL02586	multiple H-substituted, multiple ether-substituted perfluoroalkyl alcohols, n=5, m=16		FC(F)(F)C(F)(F)C(F)(F)C(F)(F)C(F)(F)C(F)(F)C(F)(F)C(F)(F)C(F)(F)C(F)(F)CCOCCOCCOCCOCCOCCOCCOCCOCCOCCOCCOCCOCCOCCOCCOCCO[H]		-9.81	0.58	4.81	P	OUT
QL02587	multiple H-substituted, multiple ether-substituted perfluoroalkyl alcohols, n=5, m=17		FC(F)(F)C(F)(F)C(F)(F)C(F)(F)C(F)(F)C(F)(F)C(F)(F)C(F)(F)C(F)(F)C(F)(F)CCOCCOCCOCCOCCOCCOCCOCCOCCOCCOCCOCCOCCOCCOCCOCCOCCO[H]		-9.85	0.57	4.87	P	OUT
QL02588	multiple H-substituted, multiple ether-substituted perfluoroalkyl alcohols, n=5, m=18		FC(F)(F)C(F)(F)C(F)(F)C(F)(F)C(F)(F)C(F)(F)C(F)(F)C(F)(F)C(F)(F)C(F)(F)CCOCCOCCOCCOCCOCCOCCOCCOCCOCCOCCOCCOCCOCCOCCOCCOCCOCCO[H]		-9.93	0.57	4.95	P	OUT
QL02589	multiple H-substituted, multiple ether-substituted perfluoroalkyl alcohols, n=5, m=19		FC(F)(F)C(F)(F)C(F)(F)C(F)(F)C(F)(F)C(F)(F)C(F)(F)C(F)(F)C(F)(F)C(F)(F)CCOCCOCCOCCOCCOCCOCCOCCOCCOCCOCCOCCOCCOCCOCCOCCOCCOCCOCCO[H]		-9.97	0.57	5.00	P	OUT
QL02590	multiple H-substituted, multiple ether-substituted perfluoroalkyl alcohols, n=5, m=20		FC(F)(F)C(F)(F)C(F)(F)C(F)(F)C(F)(F)C(F)(F)C(F)(F)C(F)(F)C(F)(F)C(F)(F)CCOCCOCCOCCOCCOCCOCCOCCOCCOCCOCCOCCOCCOCCOCCOCCOCCOCCOCCOCCO[H]		-10.06	0.57	5.08	P	OUT
QL02591	Tris(3,3,4,4,5,5,6,6,7,7,8,8,9,9,10,10,11,11,12,12,12-henicosafluorododecyl) phosphate	106554-16-9	FC(F)(F)C(F)(F)C(F)(F)C(F)(F)C(F)(F)C(F)(F)C(F)(F)C(F)(F)C(F)(F)C(F)(F)CCOP(=O)(OCCC(F)(F)C(F)(F)C(F)(F)C(F)(F)C(F)(F)C(F)(F)C(F)(F)C(F)(F)C(F)(F)C(F)(F)F)OCCC(F)(F)C(F)(F)C(F)(F)C(F)(F)C(F)(F)C(F)(F)C(F)(F)C(F)(F)C(F)(F)C(F)(F)F		-10.70	0.68	3.74	P	OUT
QL02592	1,1,2,2-Tetrahydroperfluorododecanethiol	34451-28-0	FC(F)(F)C(F)(F)C(F)(F)C(F)(F)C(F)(F)C(F)(F)C(F)(F)C(F)(F)C(F)(F)C(F)(F)CCS		-8.50	0.68	2.73	P	IN
QL02593	n2 fluorotelomer thioamido sulfonic acids, n=5		FC(F)(F)C(F)(F)C(F)(F)C(F)(F)C(F)(F)C(F)(F)C(F)(F)C(F)(F)C(F)(F)C(F)(F)CCS(CCC(NC(C)(C)CS(O)(=O)=O)=O)=O		-9.48	0.66	3.41	P	OUT

QL02594	n2 fluorotelomer sulfonamido betaine-related, n=5		FC(F)(F)C(F)(F)C(F)(F)C(F)(F)C(F)(F)C(F)(F)C(F)(F)C(F)(F)C(F)(F)C(F)(F)CCS(NCCC[N+](CC(O)=O)(C)C)(=O)=O		-9.45	0.66	3.41	P	OUT
QL02595	n2 fluorotelomer sulfonamido amines, n=5		FC(F)(F)C(F)(F)C(F)(F)C(F)(F)C(F)(F)C(F)(F)C(F)(F)C(F)(F)C(F)(F)C(F)(F)CCS(NCCCN(C)C)(=O)=O		-9.19	0.66	3.27	P	OUT
QL02596	n2 fluorotelomer thio alkyl amido carboxylic acids, n=5		FC(F)(F)C(F)(F)C(F)(F)C(F)(F)C(F)(F)C(F)(F)C(F)(F)C(F)(F)C(F)(F)C(F)(F)CCSC(C(O)=O)CC(NCCCN(C)C)=O		-9.20	0.65	3.49	P	OUT
QL02597	n2 fluorotelomer thio alkyl amido ammonium_i, n=5		FC(F)(F)C(F)(F)C(F)(F)C(F)(F)C(F)(F)C(F)(F)C(F)(F)C(F)(F)C(F)(F)C(F)(F)CCSCC(NCCC[N+](C)(C)C)=O		-9.31	0.66	3.42	P	OUT
QL02598	n2 fluorotelomer thio alkyl amido betaine-related, n=5		FC(F)(F)C(F)(F)C(F)(F)C(F)(F)C(F)(F)C(F)(F)C(F)(F)C(F)(F)C(F)(F)C(F)(F)CCSCC(NCCC[N+](C)(C)CC(O)=O)=O		-9.35	0.65	3.53	P	OUT
QL02599	n2 fluorotelomer thio alkyl amido ammonium, n=5		FC(F)(F)C(F)(F)C(F)(F)C(F)(F)C(F)(F)C(F)(F)C(F)(F)C(F)(F)C(F)(F)C(F)(F)CCSCC(NCCCN(C)C)=O		-9.15	0.65	3.43	P	OUT
QL02600	n2 fluorotelomer thio alkyl amido sulfonic acids, n=5		FC(F)(F)C(F)(F)C(F)(F)C(F)(F)C(F)(F)C(F)(F)C(F)(F)C(F)(F)C(F)(F)C(F)(F)CCSCCC(NC(C)(C)CS(O)(=O)=O)=O		-9.42	0.66	3.41	P	OUT
QL02601	2,2,3,3,4,4,5,5,6,6,7,7,8,8,9,9,10,10,11,11,11-Henicosafluoroundecyl acrylate	94231-57-9	FC(F)(F)C(F)(F)C(F)(F)C(F)(F)C(F)(F)C(F)(F)C(F)(F)C(F)(F)C(F)(F)C(F)(F)COC(=O)C=C		-8.75	0.67	2.94	P	IN
QL02602	multiple H-substituted-ether-substituted-perfluoroalkyl (linear) carboxylic acids, n=10		FC(F)(F)C(F)(F)C(F)(F)C(F)(F)C(F)(F)C(F)(F)C(F)(F)C(F)(F)C(F)(F)C(F)(F)COCC(O)=O		-8.77	0.67	2.94	P	IN
QL02603	Perfluorodecane	307-45-9	FC(F)(F)C(F)(F)C(F)(F)C(F)(F)C(F)(F)C(F)(F)C(F)(F)C(F)(F)C(F)(F)C(F)(F)F		-8.53	0.69	2.51	P	IN
QL02604	3,3,4,4,5,5,6,6,7,7,8,8,9,9,10,10,11,11,11-Nonadecafluoroundec-1-ene	57216-75-8	FC(F)(F)C(F)(F)C(F)(F)C(F)(F)C(F)(F)C(F)(F)C(F)(F)C(F)(F)C(F)(F)C=C		-8.24	0.68	2.51	P	IN
QL02605	4-(Nonadecafluorononyl)benzoyl azide	93238-01-8	FC(F)(F)C(F)(F)C(F)(F)C(F)(F)C(F)(F)C(F)(F)C(F)(F)C(F)(F)C(F)(F)C1=CC=C(C=C1)C(=O)N=[N+]=[N-]		-8.75	0.66	3.12	P	IN
QL02606	1-Isocyanato-4-(nonadecafluorononyl)benzene	93238-00-7	FC(F)(F)C(F)(F)C(F)(F)C(F)(F)C(F)(F)C(F)(F)C(F)(F)C(F)(F)C(F)(F)C1=CC=C(C=C1)N=C=O		-8.73	0.66	3.08	P	IN
QL02607	2,4,6-Tris(nonadecafluorononyl)-1,3,5-triazine	57101-59-4	FC(F)(F)C(F)(F)C(F)(F)C(F)(F)C(F)(F)C(F)(F)C(F)(F)C(F)(F)C(F)(F)C1=NC(=NC(=N1)C(F)(F)C(F)(F)C(F)(F)C(F)(F)C(F)(F)C(F)(F)C(F)(F)C(F)(F)C(F)(F)F)C(F)(F)C(F)(F)C(F)(F)C(F)(F)C(F)(F)C(F)(F)C(F)(F)C(F)(F)C(F)(F)F		-10.25	0.69	3.41	P	OUT
QL02608	n1 fluorotelomer carboxylic acids, n=9		FC(F)(F)C(F)(F)C(F)(F)C(F)(F)C(F)(F)C(F)(F)C(F)(F)C(F)(F)C(F)(F)CC(O)=O		-8.45	0.68	2.68	P	IN
QL02609	n2 perfluoroalkyl acrylates, n=9		FC(F)(F)C(F)(F)C(F)(F)C(F)(F)C(F)(F)C(F)(F)C(F)(F)C(F)(F)C(F)(F)CCOC(CC(C)=C)=O		-8.78	0.66	3.08	P	IN
QL02610	1H,1H-Perfluoro-n-decyl acrylate	335-83-1	FC(F)(F)C(F)(F)C(F)(F)C(F)(F)C(F)(F)C(F)(F)C(F)(F)C(F)(F)C(F)(F)COC(=O)C=C		-8.60	0.67	2.89	P	IN
QL02611	multiple H-substituted-ether-substituted-perfluoroalkyl (linear) carboxylic acids, n=9		FC(F)(F)C(F)(F)C(F)(F)C(F)(F)C(F)(F)C(F)(F)C(F)(F)C(F)(F)C(F)(F)COCC(O)=O		-8.61	0.67	2.89	P	IN
QL02612	Perfluorononane	375-96-2	FC(F)(F)C(F)(F)C(F)(F)C(F)(F)C(F)(F)C(F)(F)C(F)(F)C(F)(F)C(F)(F)F		-8.37	0.69	2.44	P	IN
QL02613	3,3,4,4,5,5,6,6,7,7,8,8,9,9,10,10,10-Heptadecafluorodec-1-yne	55009-88-6	FC(F)(F)C(F)(F)C(F)(F)C(F)(F)C(F)(F)C(F)(F)C(F)(F)C(F)(F)C#C		-8.06	0.68	2.44	P	IN
QL02614	Heptadecafluorononanenitrile	82416-71-5	FC(F)(F)C(F)(F)C(F)(F)C(F)(F)C(F)(F)C(F)(F)C(F)(F)C(F)(F)C#N		-8.06	0.68	2.44	P	IN
QL02615	(9E)-1,1,1,2,2,3,3,4,4,5,5,6,6,7,7,8,8,11,11,12,12,13,13,14,14,15,15,16,16,17,17,18,18,18-Tetratriacontafluorooctadec-9-ene	51249-70-8	FC(F)(F)C(F)(F)C(F)(F)C(F)(F)C(F)(F)C(F)(F)C(F)(F)C(F)(F)C=CC(F)(F)C(F)(F)C(F)(F)C(F)(F)C(F)(F)C(F)(F)C(F)(F)C(F)(F)F		-9.88	0.69	3.24	P	OUT
QL02616	Tris[3-(perfluorooctyl)phenyl]phosphine	325459-91-4	FC(F)(F)C(F)(F)C(F)(F)C(F)(F)C(F)(F)C(F)(F)C(F)(F)C(F)(F)C1=CC(=CC=C1)P(C1=CC=CC(=C1)C(F)(F)C(F)(F)C(F)(F)C(F)(F)C(F)(F)C(F)(F)C(F)(F)C(F)(F)F)C1=CC=CC(=C1)C(F)(F)C(F)(F)C(F)(F)C(F)(F)C(F)(F)C(F)(F)C(F)(F)C(F)(F)F		-10.11	0.67	3.58	P	OUT
QL02617	1-Bromo-3-(heptadecafluorooctyl)benzene	325459-90-3	FC(F)(F)C(F)(F)C(F)(F)C(F)(F)C(F)(F)C(F)(F)C(F)(F)C(F)(F)C1=CC(Br)=CC=C1		-8.22	0.67	2.69	P	IN
QL02618	1-Bromo-4-(heptadecafluorooctyl)benzene	206560-77-2	FC(F)(F)C(F)(F)C(F)(F)C(F)(F)C(F)(F)C(F)(F)C(F)(F)C(F)(F)C1=CC=C(Br)C=C1		-8.35	0.67	2.74	P	IN
QL02619	1-Ethenyl-4-(heptadecafluorooctyl)benzene	106209-21-6	FC(F)(F)C(F)(F)C(F)(F)C(F)(F)C(F)(F)C(F)(F)C(F)(F)C(F)(F)C1=CC=C(C=C)C=C1		-8.46	0.66	2.90	P	IN
QL02620	Tris[4-(heptadecafluorooctyl)phenyl]phosphine	284472-92-0	FC(F)(F)C(F)(F)C(F)(F)C(F)(F)C(F)(F)C(F)(F)C(F)(F)C(F)(F)C1=CC=C(C=C1)P(C1=CC=C(C=C1)C(F)(F)C(F)(F)C(F)(F)C(F)(F)C(F)(F)C(F)(F)C(F)(F)C(F)(F)F)C1=CC=C(C=C1)C(F)(F)C(F)(F)C(F)(F)C(F)(F)C(F)(F)C(F)(F)C(F)(F)C(F)(F)F		-10.26	0.68	3.64	P	OUT
QL02621	Benzene, (heptadecafluorooctyl)-	87405-83-2	FC(F)(F)C(F)(F)C(F)(F)C(F)(F)C(F)(F)C(F)(F)C(F)(F)C(F)(F)C1=CC=CC=C1		-8.15	0.67	2.69	P	IN
QL02622	4-(Heptadecafluorooctyl)-1H-pyrazole	92914-86-8	FC(F)(F)C(F)(F)C(F)(F)C(F)(F)C(F)(F)C(F)(F)C(F)(F)C(F)(F)C1=CNN=C1		-7.97	0.67	2.52	P	IN
QL02623	Thiophene, 3-(heptadecafluorooctyl)-	96721-82-3	FC(F)(F)C(F)(F)C(F)(F)C(F)(F)C(F)(F)C(F)(F)C(F)(F)C(F)(F)C1=CSC=C1		-7.97	0.67	2.52	P	IN

QL02624	2-(Heptadecafluorooctyl)thiophene	80791-16-8	FC(F)(F)C(F)(F)C(F)(F)C(F)(F)C(F)(F)C(F)(F)C(F)(F)C(F)(F)c1cccs1		-7.97	0.67	2.52	P	IN
QL02625	Oxirane, (heptadecafluorooctyl)-	52835-16-2	FC(F)(F)C(F)(F)C(F)(F)C(F)(F)C(F)(F)C(F)(F)C(F)(F)C(F)(F)C1CO1		-8.17	0.68	2.45	P	IN
QL02626	5-(Heptadecafluorooctyl)-1H-imidazole	81769-56-4	FC(F)(F)C(F)(F)C(F)(F)C(F)(F)C(F)(F)C(F)(F)C(F)(F)C(F)(F)c1Ncnc1		-7.97	0.67	2.52	P	IN
QL02627	3-(Heptadecafluorooctyl)-1,2,4-trioxolane	63967-43-1	FC(F)(F)C(F)(F)C(F)(F)C(F)(F)C(F)(F)C(F)(F)C(F)(F)C(F)(F)C1OCOO1		-7.97	0.67	2.52	P	IN
QL02628	3,3,4,4,5,5,6,6,7,7,8,8,9,9,10,10,10-Heptadecafluorodecanoyl chloride	64018-23-1	FC(F)(F)C(F)(F)C(F)(F)C(F)(F)C(F)(F)C(F)(F)C(F)(F)C(F)(F)CC(Cl)=O		-8.04	0.68	2.51	P	IN
QL02629	1-(3,3,4,4,5,5,6,6,7,7,8,8,9,9,10,10,10-Heptadecafluoro-1-iodododecyl)-2,8,9-trioxa-5-aza-1-silabicyclo[3.3.3]undecane	135587-17-6	FC(F)(F)C(F)(F)C(F)(F)C(F)(F)C(F)(F)C(F)(F)C(F)(F)C(F)(F)CC(I)[Si]12OCCN(CCO1)CCO2		-8.35	0.66	2.94	P	IN
QL02630	1,1,1,2,2,3,3,4,4,5,5,6,6,7,7,8,8,11,11,12,12,13,13,14,14,15,15,16,16,17,17,18,18,18-Tetratriacontafluoro-9-iodooctadecane	53638-12-3	FC(F)(F)C(F)(F)C(F)(F)C(F)(F)C(F)(F)C(F)(F)C(F)(F)C(F)(F)CC(I)C(F)(F)C(F)(F)C(F)(F)C(F)(F)C(F)(F)C(F)(F)C(F)(F)C(F)(F)F		-9.92	0.69	3.26	P	OUT
QL02631	1,1,1,2,2,3,3,4,4,5,5,6,6,7,7,8,8,14,14,15,15,16,16,17,17,18,18,19,19,20,20,21,21,21-Tetratriacontafluoro-10,12-diiodohenicosane	1254971-75-9	FC(F)(F)C(F)(F)C(F)(F)C(F)(F)C(F)(F)C(F)(F)C(F)(F)C(F)(F)CC(I)CC(I)CC(F)(F)C(F)(F)C(F)(F)C(F)(F)C(F)(F)C(F)(F)C(F)(F)C(F)(F)F		-10.02	0.68	3.45	P	OUT
QL02632	1,1,1,2,2,3,3,4,4,5,5,6,6,7,7,8,8,15,15,16,16,17,17,18,18,19,19,20,20,21,21,22,22,22-Tetratriacontafluoro-10,13-diiodododocosane	460744-98-3	FC(F)(F)C(F)(F)C(F)(F)C(F)(F)C(F)(F)C(F)(F)C(F)(F)C(F)(F)CC(I)CCC(I)CC(F)(F)C(F)(F)C(F)(F)C(F)(F)C(F)(F)C(F)(F)C(F)(F)C(F)(F)F		-10.01	0.68	3.48	P	OUT
QL02633	1,1,1,2,2,3,3,4,4,5,5,6,6,7,7,8,8,17,17,18,18,19,19,20,20,21,21,22,22,23,23,24,24,24-Tetratriacontafluoro-10,15-diiodotetracosane	959462-52-3	FC(F)(F)C(F)(F)C(F)(F)C(F)(F)C(F)(F)C(F)(F)C(F)(F)C(F)(F)CC(I)CCCCC(I)CC(F)(F)C(F)(F)C(F)(F)C(F)(F)C(F)(F)C(F)(F)C(F)(F)C(F)(F)F		-10.04	0.67	3.57	P	OUT
QL02634	Bis[2-iodo-3-(perfluorooctyl)propyl] adipate	238742-84-2	FC(F)(F)C(F)(F)C(F)(F)C(F)(F)C(F)(F)C(F)(F)C(F)(F)C(F)(F)CC(I)COC(=O)CCCCC(=O)OCC(I)CC(F)(F)C(F)(F)C(F)(F)C(F)(F)C(F)(F)C(F)(F)C(F)(F)C(F)(F)F		-10.24	0.66	3.85	P	OUT
QL02635	1 OH-substituted n2 fluorotelomer sulfonic acids, n=8		FC(F)(F)C(F)(F)C(F)(F)C(F)(F)C(F)(F)C(F)(F)C(F)(F)C(F)(F)CC(O)S(=O)(O)=O		-8.57	0.68	2.68	P	IN
QL02636	1H,1H,2H,2H-Perfluorodecyltrichlorosilane	78560-44-8	FC(F)(F)C(F)(F)C(F)(F)C(F)(F)C(F)(F)C(F)(F)C(F)(F)C(F)(F)CC[Si](Cl)(Cl)Cl		-8.49	0.68	2.68	P	IN
QL02637	1-Undecene, 4,4,5,5,6,6,7,7,8,8,9,9,10,10,11,11,11-heptadecafluoro-	61589-64-8	FC(F)(F)C(F)(F)C(F)(F)C(F)(F)C(F)(F)C(F)(F)C(F)(F)C(F)(F)CC=C		-7.91	0.67	2.50	P	IN
QL02638	3-(2,2,3,3,4,4,5,5,6,6,7,7,8,8,9,9,9-heptadecafluorononyl)-4-(iodomethyl)oxolane	131771-31-8	FC(F)(F)C(F)(F)C(F)(F)C(F)(F)C(F)(F)C(F)(F)C(F)(F)C(F)(F)CC1COCC1CI		-8.12	0.66	2.74	P	IN
QL02639	10-Bromo-1,1,1,2,2,3,3,4,4,5,5,6,6,7,7,8,8-heptadecafluorodecane	21652-57-3	FC(F)(F)C(F)(F)C(F)(F)C(F)(F)C(F)(F)C(F)(F)C(F)(F)C(F)(F)CCBr		-7.91	0.67	2.50	P	IN
QL02640	1-((Perfluorooctyl)propanoyloxy)pyrrolidine-2,5-dione	852527-45-8	FC(F)(F)C(F)(F)C(F)(F)C(F)(F)C(F)(F)C(F)(F)C(F)(F)C(F)(F)CCC(=O)ON1C(=O)CCC1=O		-8.46	0.66	2.93	P	IN
QL02641	(Perfluorooctyl)propanoyl chloride	89373-67-1	FC(F)(F)C(F)(F)C(F)(F)C(F)(F)C(F)(F)C(F)(F)C(F)(F)C(F)(F)CCC(Cl)=O		-8.27	0.67	2.69	P	IN
QL02642	1,1,1,2,2,3,3,4,4,5,5,6,6,7,7,8,8,11,11,12,12,13,13,14,14,15,15,16,16,17,17,18,18,18-Tetratriacontafluorooctadecane	181765-99-1	FC(F)(F)C(F)(F)C(F)(F)C(F)(F)C(F)(F)C(F)(F)C(F)(F)C(F)(F)CCC(F)(F)C(F)(F)C(F)(F)C(F)(F)C(F)(F)C(F)(F)C(F)(F)C(F)(F)F		-9.88	0.69	3.24	P	OUT
QL02643	4,4,5,5,6,6,7,7,8,8,9,9,10,10,11,11,11-Heptadecafluoroundecanenitrile	26649-26-3	FC(F)(F)C(F)(F)C(F)(F)C(F)(F)C(F)(F)C(F)(F)C(F)(F)C(F)(F)CCC#N		-8.10	0.66	2.68	P	IN
QL02644	4,4,5,5,6,6,7,7,8,8,9,9,10,10,11,11,11-Heptadecafluoroundecanal	42028-44-4	FC(F)(F)C(F)(F)C(F)(F)C(F)(F)C(F)(F)C(F)(F)C(F)(F)C(F)(F)CCC=O		-8.10	0.66	2.68	P	IN
QL02645	Tris[3-(3,3,4,4,5,5,6,6,7,7,8,8,9,9,10,10,10-heptadecafluorodecyl)phenyl]phosphane	342889-38-7	FC(F)(F)C(F)(F)C(F)(F)C(F)(F)C(F)(F)C(F)(F)C(F)(F)C(F)(F)CCC1=CC(=CC=C1)P(C1=CC=CC(CCC(F)(F)C(F)(F)C(F)(F)C(F)(F)C(F)(F)C(F)(F)C(F)(F)C(F)(F)F)=C1)C1=CC=CC(CCC(F)(F)C(F)(F)C(F)(F)C(F)(F)C(F)(F)C(F)(F)C(F)(F)C(F)(F)F)=C1		-10.23	0.67	3.75	P	OUT
QL02646	Tris[4-(3,3,4,4,5,5,6,6,7,7,8,8,9,9,10,10,10-heptadecafluorodecyl)phenyl]phosphane	325459-92-5	FC(F)(F)C(F)(F)C(F)(F)C(F)(F)C(F)(F)C(F)(F)C(F)(F)C(F)(F)CCC1=CC=C(C=C1)P(C1=CC=C(CCC(F)(F)C(F)(F)C(F)(F)C(F)(F)C(F)(F)C(F)(F)C(F)(F)C(F)(F)F)C=C1)C1=CC=C(CCC(F)(F)C(F)(F)C(F)(F)C(F)(F)C(F)(F)C(F)(F)C(F)(F)C(F)(F)F)C=C1		-10.38	0.67	3.81	P	OUT
QL02647	Bis[4-(1H,1H,2H,2H-perfluorodecyl)phenyl]phenylphosphine	892154-91-5	FC(F)(F)C(F)(F)C(F)(F)C(F)(F)C(F)(F)C(F)(F)C(F)(F)C(F)(F)CCC1=CC=C(C=C1)P(C1=CC=CC=C1)C1=CC=C(CCC(F)(F)C(F)(F)C(F)(F)C(F)(F)C(F)(F)C(F)(F)C(F)(F)C(F)(F)F)C=C1		-10.18	0.66	3.85	P	OUT
QL02648	[4-(3,3,4,4,5,5,6,6,7,7,8,8,9,9,10,10,10-Heptadecafluorodecyl)phenyl] diphenylphosphine	462996-04-9	FC(F)(F)C(F)(F)C(F)(F)C(F)(F)C(F)(F)C(F)(F)C(F)(F)C(F)(F)CCC1=CC=C(C=C1)P(C1=CC=CC=C1)C1=CC=CC=C1		-8.35	0.64	3.22	P	OUT
QL02649	1-[4-((Perfluorooctyl)ethyl)phenylmethoxycarbonyloxy]pyrrolidine-2,5-dione	556050-49-8	FC(F)(F)C(F)(F)C(F)(F)C(F)(F)C(F)(F)C(F)(F)C(F)(F)C(F)(F)CCC1=CC=C(COC(=O)ON2C(=O)CCC2=O)C=C1		-8.95	0.65	3.39	P	OUT

QL02650	1-Bromo-3-(3,3,4,4,5,5,6,6,7,7,8,8,9,9,10,10,10-heptadecafluorodecyl)benzene	340157-97-3	FC(F)(F)C(F)(F)C(F)(F)C(F)(F)C(F)(F)C(F)(F)C(F)(F)C(F)(F)CCc1cccc(Br)c1		-8.42	0.66	2.90	P	IN
QL02651	12-Bromo-1,1,1,2,2,3,3,4,4,5,5,6,6,7,7,8,8-heptadecafluorododecane	99324-99-9	FC(F)(F)C(F)(F)C(F)(F)C(F)(F)C(F)(F)C(F)(F)C(F)(F)C(F)(F)CCCCBr		-8.06	0.66	2.74	P	IN
QL02652	1,1,1,2,2,3,3,4,4,5,5,6,6,7,7,8,8,13,13,14,14,15,15,16,16,17,17,18,18,19,19,20,20,20-Tetratriacontafluoroicosane	133299-41-9	FC(F)(F)C(F)(F)C(F)(F)C(F)(F)C(F)(F)C(F)(F)C(F)(F)C(F)(F)CCCCC(F)(F)C(F)(F)C(F)(F)C(F)(F)C(F)(F)C(F)(F)C(F)(F)C(F)(F)F		-9.90	0.68	3.35	P	OUT
QL02653	1,1,1,2,2,3,3,4,4,5,5,6,6,7,7,8,8,15,15,16,16,17,17,18,18,19,19,20,20,21,21,22,22,22-Tetratriacontafluorodocosane	959462-55-6	FC(F)(F)C(F)(F)C(F)(F)C(F)(F)C(F)(F)C(F)(F)C(F)(F)C(F)(F)CCCCCCC(F)(F)C(F)(F)C(F)(F)C(F)(F)C(F)(F)C(F)(F)C(F)(F)C(F)(F)F		-9.92	0.68	3.45	P	OUT
QL02654	1,1,1,2,2,3,3,4,4,5,5,6,6,7,7,8,8,17,17,18,18,19,19,20,20,21,21,22,22,23,23,24,24,24-Tetratriacontafluorotetracosane	100550-08-1	FC(F)(F)C(F)(F)C(F)(F)C(F)(F)C(F)(F)C(F)(F)C(F)(F)C(F)(F)CCCCCCCCC(F)(F)C(F)(F)C(F)(F)C(F)(F)C(F)(F)C(F)(F)C(F)(F)C(F)(F)F		-9.95	0.67	3.55	P	OUT
QL02655	18-Bromo-1,1,1,2,2,3,3,4,4,5,5,6,6,7,7,8,8-heptadecafluorooctadecane	184007-06-5	FC(F)(F)C(F)(F)C(F)(F)C(F)(F)C(F)(F)C(F)(F)C(F)(F)C(F)(F)CCCCCCCCCCBr		-8.39	0.63	3.28	P	OUT
QL02656	19-Bromo-1,1,1,2,2,3,3,4,4,5,5,6,6,7,7,8,8-heptadecafluorononadecane	1597-70-2	FC(F)(F)C(F)(F)C(F)(F)C(F)(F)C(F)(F)C(F)(F)C(F)(F)C(F)(F)CCCCCCCCCCCBr		-8.46	0.63	3.37	P	OUT
QL02657	4'-[(12,12,13,13,14,14,15,15,16,16,17,17,18,18,19,19,19-Heptadecafluorononadecyl)oxy][1,1'-biphenyl]-4-carbonitrile	117750-26-2	FC(F)(F)C(F)(F)C(F)(F)C(F)(F)C(F)(F)C(F)(F)C(F)(F)C(F)(F)CCCCCCCCCCCOc1ccc(cc1)-c1ccc(cc1)C#N		-9.13	0.62	3.88	P	OUT
QL02658	(11-(F-Octyl)undecyl) dimorpholinophosphoramidate	134051-92-6	FC(F)(F)C(F)(F)C(F)(F)C(F)(F)C(F)(F)C(F)(F)C(F)(F)C(F)(F)CCCCCCCCCCCOP(=O)(N1CCOCC1)N1CCOCC1		-8.53	0.62	3.55	P	OUT
QL02659	1,1,1,2,2,3,3,4,4,5,5,6,6,7,7,8,8-Heptadecafluoro-12-iodododecane	38565-62-7	FC(F)(F)C(F)(F)C(F)(F)C(F)(F)C(F)(F)C(F)(F)C(F)(F)C(F)(F)CCCCI		-8.06	0.66	2.74	P	IN
QL02660	3-(Perfluorooctyl)propyl iodide	200112-75-0	FC(F)(F)C(F)(F)C(F)(F)C(F)(F)C(F)(F)C(F)(F)C(F)(F)C(F)(F)CCCI		-8.10	0.66	2.68	P	IN
QL02661	11-Azido-1,1,1,2,2,3,3,4,4,5,5,6,6,7,7,8,8-heptadecafluoroundecane	852527-61-8	FC(F)(F)C(F)(F)C(F)(F)C(F)(F)C(F)(F)C(F)(F)C(F)(F)C(F)(F)CCCN=[N+]=[N-]		-8.24	0.65	2.91	P	IN
QL02662	N-(4,4,5,5,6,6,7,7,8,8,9,9,10,10,11,11,11-Heptadecafluoroundecyl)maleimide	852527-40-3	FC(F)(F)C(F)(F)C(F)(F)C(F)(F)C(F)(F)C(F)(F)C(F)(F)C(F)(F)CCCN1C(=O)C=CC1=O		-8.43	0.66	2.88	P	IN
QL02663	1-(3-(Perfluorooctyl)propyl)-2H-3,1-benzoxazine-2,4(1H)-dione	544418-04-4	FC(F)(F)C(F)(F)C(F)(F)C(F)(F)C(F)(F)C(F)(F)C(F)(F)C(F)(F)CCCN1C(=O)OC(=O)C2=CC=CC=C12		-8.52	0.65	3.06	P	IN
QL02664	N-(4,4,5,5,6,6,7,7,8,8,9,9,10,10,11,11,11-Heptadecafluoroundecyl)iodoacetamide	852527-48-1	FC(F)(F)C(F)(F)C(F)(F)C(F)(F)C(F)(F)C(F)(F)C(F)(F)C(F)(F)CCCNC(=O)CI		-8.35	0.65	2.95	P	IN
QL02665	4,4,5,5,6,6,7,7,8,8,9,9,10,10,11,11,11-Heptadecafluoroundecyl prop-2-enoate	1652-60-4	FC(F)(F)C(F)(F)C(F)(F)C(F)(F)C(F)(F)C(F)(F)C(F)(F)C(F)(F)CCCOC(=O)C=C		-8.35	0.65	2.95	P	IN
QL02666	4-[3-(Perfluorooctyl)-1-propyloxy]benzaldehyde	494798-73-1	FC(F)(F)C(F)(F)C(F)(F)C(F)(F)C(F)(F)C(F)(F)C(F)(F)C(F)(F)CCCOC1=CC=C(C=O)C=C1		-8.68	0.65	3.23	P	OUT
QL02667	1,1,1,2,2,3,3,4,4,5,5,6,6,7,7,8,8-Heptadecafluoro-10-isocyanatodecane	142010-50-2	FC(F)(F)C(F)(F)C(F)(F)C(F)(F)C(F)(F)C(F)(F)C(F)(F)C(F)(F)CCN=C=O		-8.06	0.66	2.74	P	IN
QL02668	3,3,4,4,5,5,6,6,7,7,8,8,9,9,10,10,10-Heptadecafluorodecyl pyridine-3-carboxylate	331716-84-8	FC(F)(F)C(F)(F)C(F)(F)C(F)(F)C(F)(F)C(F)(F)C(F)(F)C(F)(F)CCOC(=O)c1cccnc1		-8.55	0.65	3.09	P	IN
QL02669	n2 perfluoroalkyl acrylates, n=8		FC(F)(F)C(F)(F)C(F)(F)C(F)(F)C(F)(F)C(F)(F)C(F)(F)C(F)(F)CCOC(CC(C)=C)=O		-8.42	0.66	2.95	P	IN
QL02670	multiple H-substituted, multiple ether-substituted perfluoroalkyl alcohols, n=4, m=5		FC(F)(F)C(F)(F)C(F)(F)C(F)(F)C(F)(F)C(F)(F)C(F)(F)C(F)(F)CCOCCOCCOCCOCCO[H]		-8.49	0.62	3.52	P	OUT
QL02671	multiple H-substituted, multiple ether-substituted perfluoroalkyl alcohols, n=4, m=6		FC(F)(F)C(F)(F)C(F)(F)C(F)(F)C(F)(F)C(F)(F)C(F)(F)C(F)(F)CCOCCOCCOCCOCCOCCO[H]		-8.62	0.61	3.71	P	OUT
QL02672	multiple H-substituted, multiple ether-substituted perfluoroalkyl alcohols, n=4, m=7		FC(F)(F)C(F)(F)C(F)(F)C(F)(F)C(F)(F)C(F)(F)C(F)(F)C(F)(F)CCOCCOCCOCCOCCOCCOCCO[H]		-8.66	0.60	3.83	P	OUT
QL02673	multiple H-substituted, multiple ether-substituted perfluoroalkyl alcohols, n=4, m=8		FC(F)(F)C(F)(F)C(F)(F)C(F)(F)C(F)(F)C(F)(F)C(F)(F)C(F)(F)CCOCCOCCOCCOCCOCCOCCOCCO[H]		-8.79	0.60	3.98	P	OUT
QL02674	multiple H-substituted, multiple ether-substituted perfluoroalkyl alcohols, n=4, m=9		FC(F)(F)C(F)(F)C(F)(F)C(F)(F)C(F)(F)C(F)(F)C(F)(F)C(F)(F)CCOCCOCCOCCOCCOCCOCCOCCOCCO[H]		-8.83	0.59	4.09	P	OUT
QL02675	multiple H-substituted, multiple ether-substituted perfluoroalkyl alcohols, n=4, m=10		FC(F)(F)C(F)(F)C(F)(F)C(F)(F)C(F)(F)C(F)(F)C(F)(F)C(F)(F)CCOCCOCCOCCOCCOCCOCCOCCOCCOCCO[H]		-8.96	0.59	4.22	P	OUT
QL02676	multiple H-substituted, multiple ether-substituted perfluoroalkyl alcohols, n=4, m=11		FC(F)(F)C(F)(F)C(F)(F)C(F)(F)C(F)(F)C(F)(F)C(F)(F)C(F)(F)CCOCCOCCOCCOCCOCCOCCOCCOCCOCCOCCO[H]		-9.01	0.58	4.31	P	OUT
QL02677	multiple H-substituted, multiple ether-substituted perfluoroalkyl alcohols, n=4, m=12		FC(F)(F)C(F)(F)C(F)(F)C(F)(F)C(F)(F)C(F)(F)C(F)(F)C(F)(F)CCOCCOCCOCCOCCOCCOCCOCCOCCOCCOCCOCCO[H]		-9.12	0.58	4.42	P	OUT
QL02678	multiple H-substituted, multiple ether-substituted perfluoroalkyl alcohols, n=4, m=13		FC(F)(F)C(F)(F)C(F)(F)C(F)(F)C(F)(F)C(F)(F)C(F)(F)C(F)(F)CCOCCOCCOCCOCCOCCOCCOCCOCCOCCOCCOCCOCCO[H]		-9.17	0.57	4.50	P	OUT
QL02679	multiple H-substituted, multiple ether-substituted perfluoroalkyl alcohols, n=4, m=14		FC(F)(F)C(F)(F)C(F)(F)C(F)(F)C(F)(F)C(F)(F)C(F)(F)C(F)(F)CCOCCOCCOCCOCCOCCOCCOCCOCCOCCOCCOCCOCCOCCO[H]		-9.28	0.57	4.60	P	OUT

QL02680	multiple H-substituted, multiple ether-substituted perfluoroalkyl alcohols, n=4, m=15		FC(F)(F)C(F)(F)C(F)(F)C(F)(F)C(F)(F)C(F)(F)C(F)(F)C(F)(F)CCOCCOCCOCCOCCOCCOCCOCCOCCOCCOCCOCCOCCOCCOCCO[H]		-9.33	0.57	4.67	P	OUT
QL02681	multiple H-substituted, multiple ether-substituted perfluoroalkyl alcohols, n=4, m=16		FC(F)(F)C(F)(F)C(F)(F)C(F)(F)C(F)(F)C(F)(F)C(F)(F)C(F)(F)CCOCCOCCOCCOCCOCCOCCOCCOCCOCCOCCOCCOCCOCCOCCOCCO[H]		-9.44	0.57	4.77	P	OUT
QL02682	multiple H-substituted, multiple ether-substituted perfluoroalkyl alcohols, n=4, m=17		FC(F)(F)C(F)(F)C(F)(F)C(F)(F)C(F)(F)C(F)(F)C(F)(F)C(F)(F)CCOCCOCCOCCOCCOCCOCCOCCOCCOCCOCCOCCOCCOCCOCCOCCOCCO[H]		-9.49	0.56	4.83	P	OUT
QL02683	multiple H-substituted, multiple ether-substituted perfluoroalkyl alcohols, n=4, m=18		FC(F)(F)C(F)(F)C(F)(F)C(F)(F)C(F)(F)C(F)(F)C(F)(F)C(F)(F)CCOCCOCCOCCOCCOCCOCCOCCOCCOCCOCCOCCOCCOCCOCCOCCOCCOCCO[H]		-9.59	0.56	4.91	P	OUT
QL02684	multiple H-substituted, multiple ether-substituted perfluoroalkyl alcohols, n=4, m=19		FC(F)(F)C(F)(F)C(F)(F)C(F)(F)C(F)(F)C(F)(F)C(F)(F)C(F)(F)CCOCCOCCOCCOCCOCCOCCOCCOCCOCCOCCOCCOCCOCCOCCOCCOCCOCCOCCO[H]		-9.63	0.56	4.97	P	OUT
QL02685	multiple H-substituted, multiple ether-substituted perfluoroalkyl alcohols, n=4, m=20		FC(F)(F)C(F)(F)C(F)(F)C(F)(F)C(F)(F)C(F)(F)C(F)(F)C(F)(F)CCOCCOCCOCCOCCOCCOCCOCCOCCOCCOCCOCCOCCOCCOCCOCCOCCOCCOCCOC CO[H]		-9.73	0.56	5.04	P	OUT
QL02686	Tris(3,3,4,4,5,5,6,6,7,7,8,8,9,9,10,10,10-heptadecafluorodecyl) phosphate	149790-22-7	FC(F)(F)C(F)(F)C(F)(F)C(F)(F)C(F)(F)C(F)(F)C(F)(F)C(F)(F)CCOP(=O)(OCCC(F)(F)C(F)(F)C(F)(F)C(F)(F)C(F)(F)C(F)(F)C(F)(F)C(F)(F)F)OCCC(F)(F)C(F)(F)C(F)(F)C(F)(F)C(F)(F)C(F)(F)C(F)(F)C(F)(F)F		-10.15	0.68	3.51	P	OUT
QL02687	2-(Perfluorooctyl)ethanthiol	34143-74-3	FC(F)(F)C(F)(F)C(F)(F)C(F)(F)C(F)(F)C(F)(F)C(F)(F)C(F)(F)CCS		-7.91	0.67	2.50	P	IN
QL02688	10-(Ethenesulfonyl)-1,1,1,2,2,3,3,4,4,5,5,6,6,7,7,8,8-heptadecafluorodecane	93345-48-3	FC(F)(F)C(F)(F)C(F)(F)C(F)(F)C(F)(F)C(F)(F)C(F)(F)C(F)(F)CCS(=O)(=O)C=C		-8.43	0.67	2.73	P	IN
QL02689	n2 fluorotelomer sulfonyl propano-amido alkyl sulfonic acids, n=4		FC(F)(F)C(F)(F)C(F)(F)C(F)(F)C(F)(F)C(F)(F)C(F)(F)C(F)(F)CCS(CCC(NC(C)(C)CS(O)(=O)=O)=O)(=O)=O		-9.14	0.66	3.24	P	OUT
QL02690	n2 fluorotelomer thioamido sulfonic acids, n=4		FC(F)(F)C(F)(F)C(F)(F)C(F)(F)C(F)(F)C(F)(F)C(F)(F)C(F)(F)CCS(CCC(NC(C)(C)CS(O)(=O)=O)=O)=O		-9.00	0.66	3.24	P	OUT
QL02691	n2 fluorotelomer sulfonyl propanoic acids, n=4		FC(F)(F)C(F)(F)C(F)(F)C(F)(F)C(F)(F)C(F)(F)C(F)(F)C(F)(F)CCS(CCC(O)=O)(=O)=O		-8.62	0.67	2.94	P	IN
QL02692	n2 fluorotelomer sulfonamido betaine-related, n=4		FC(F)(F)C(F)(F)C(F)(F)C(F)(F)C(F)(F)C(F)(F)C(F)(F)C(F)(F)CCS(NCCC[N+](CC(O)=O)(C)C)(=O)=O		-8.96	0.66	3.24	P	OUT
QL02693	n2 fluorotelomer sulfonamido amines, n=4		FC(F)(F)C(F)(F)C(F)(F)C(F)(F)C(F)(F)C(F)(F)C(F)(F)C(F)(F)CCS(NCCCN(C)C)(=O)=O		-8.74	0.66	3.13	P	IN
QL02694	n2 fluorotelomer thio alkyl amido carboxylic acids, n=4		FC(F)(F)C(F)(F)C(F)(F)C(F)(F)C(F)(F)C(F)(F)C(F)(F)C(F)(F)CCSC(C(O)=O)CC(NCCCN(C)C)=O		-8.69	0.64	3.34	P	OUT
QL02695	Thiocyanic acid, 3,3,4,4,5,5,6,6,7,7,8,8,9,9,10,10,10-heptadecafluorodecyl ester	26650-10-2	FC(F)(F)C(F)(F)C(F)(F)C(F)(F)C(F)(F)C(F)(F)C(F)(F)C(F)(F)CCSC#N		-8.06	0.66	2.74	P	IN
QL02696	n2 fluorotelomer thio alkyl amido ammonium_i, n=4		FC(F)(F)C(F)(F)C(F)(F)C(F)(F)C(F)(F)C(F)(F)C(F)(F)C(F)(F)CCSCC(NCCC[N+](C)(C)C)=O		-8.80	0.65	3.26	P	OUT
QL02697	n2 fluorotelomer thio alkyl amido betaine-related, n=4		FC(F)(F)C(F)(F)C(F)(F)C(F)(F)C(F)(F)C(F)(F)C(F)(F)C(F)(F)CCSCC(NCCC[N+](C)(C)CC(O)=O)=O		-8.85	0.64	3.37	P	OUT
QL02698	n2 fluorotelomer thio alkyl amido ammonium, n=4		FC(F)(F)C(F)(F)C(F)(F)C(F)(F)C(F)(F)C(F)(F)C(F)(F)C(F)(F)CCSCC(NCCCN(C)C)=O		-8.62	0.64	3.27	P	OUT
QL02699	1,1,1,2,2,3,3,4,4,5,5,6,6,7,7,8,8-Heptadecafluoro-10-[(3,3,4,4,5,5,6,6,7,7,8,8,9,9,10,10,10-heptadecafluorodecyl)sulfanyl]decane	96497-21-1	FC(F)(F)C(F)(F)C(F)(F)C(F)(F)C(F)(F)C(F)(F)C(F)(F)C(F)(F)CCSCCC(F)(F)C(F)(F)C(F)(F)C(F)(F)C(F)(F)C(F)(F)C(F)(F)C(F)(F)F		-9.95	0.68	3.42	P	OUT
QL02700	n2 fluorotelomer thio alkyl amido sulfonic acids, n=4		FC(F)(F)C(F)(F)C(F)(F)C(F)(F)C(F)(F)C(F)(F)C(F)(F)C(F)(F)CCSCCC(NC(C)(C)CS(O)(=O)=O)=O		-8.93	0.66	3.24	P	OUT
QL02701	Bis(3,3,4,4,5,5,6,6,7,7,8,8,9,9,10,10,10-heptadecafluorodecyl) disulphide	42977-21-9	FC(F)(F)C(F)(F)C(F)(F)C(F)(F)C(F)(F)C(F)(F)C(F)(F)C(F)(F)CCSSCCC(F)(F)C(F)(F)C(F)(F)C(F)(F)C(F)(F)C(F)(F)C(F)(F)C(F)(F)F		-9.92	0.68	3.45	P	OUT
QL02702	1-Chloroheptadecafluorooctane	307-33-5	FC(F)(F)C(F)(F)C(F)(F)C(F)(F)C(F)(F)C(F)(F)C(F)(F)C(F)(F)Cl		-7.94	0.69	2.24	P	IN
QL02703	2,2,3,3,4,4,5,5,6,6,7,7,8,8,9,9,9-Heptadecafluorononyl acrylate	307-87-9	FC(F)(F)C(F)(F)C(F)(F)C(F)(F)C(F)(F)C(F)(F)C(F)(F)C(F)(F)COC(=O)C=C		-8.21	0.66	2.74	P	IN
QL02704	multiple H-substituted-ether-substituted-perfluoroalkyl (linear) carboxylic acids, n=8		FC(F)(F)C(F)(F)C(F)(F)C(F)(F)C(F)(F)C(F)(F)C(F)(F)C(F)(F)COCC(O)=O		-8.23	0.67	2.74	P	IN
QL02705	Perfluorooctane	307-34-6	FC(F)(F)C(F)(F)C(F)(F)C(F)(F)C(F)(F)C(F)(F)C(F)(F)C(F)(F)F		-7.94	0.69	2.24	P	IN
QL02706	Heptadecafluorooctyl prop-2-enoate	15498-45-0	FC(F)(F)C(F)(F)C(F)(F)C(F)(F)C(F)(F)C(F)(F)C(F)(F)C(F)(F)OC(=O)C=C		-8.21	0.67	2.69	P	IN
QL02707	Bis(heptadecafluorooctyl) 2-methylidenebutanedioate	243977-25-5	FC(F)(F)C(F)(F)C(F)(F)C(F)(F)C(F)(F)C(F)(F)C(F)(F)C(F)(F)OC(=O)CC(=C)C(=O)OC(F)(F)C(F)(F)C(F)(F)C(F)(F)C(F)(F)C(F)(F)C(F)(F)C(F)(F)F		-10.08	0.68	3.50	P	OUT
QL02708	2-[[3-[(Perfluorooctyl)sulfonyl]propyl]amino]ethyl-2-propenoate	110570-90-6	FC(F)(F)C(F)(F)C(F)(F)C(F)(F)C(F)(F)C(F)(F)C(F)(F)C(F)(F)S(=O)(=O)CCCNCCOC(=O)C=C		-8.79	0.65	3.30	P	OUT

QL02709	1-(1,1,2,2,3,3,4,4,5,5,6,6,7,7,8,8,8-Heptadecafluorooctane-1-sulfonyl)piperazine	113584-32-0	FC(F)(F)C(F)(F)C(F)(F)C(F)(F)C(F)(F)C(F)(F)C(F)(F)C(F)(F)S(=O)(=O)N1CCNCC1		-8.34	0.67	2.74	P	IN
QL02710	4-(1,1,2,2,3,3,4,4,5,5,6,6,7,7,8,8,8-Heptadecafluorooctane-1-sulfonyl)morpholine	312-19-6	FC(F)(F)C(F)(F)C(F)(F)C(F)(F)C(F)(F)C(F)(F)C(F)(F)C(F)(F)S(=O)(=O)N1CCOCC1		-8.34	0.67	2.74	P	IN
QL02711	N-(1,1,2,2,3,3,4,4,5,5,6,6,7,7,8,8,8-Heptadecafluorooctane-1-sulfonyl)benzamide	87988-75-8	FC(F)(F)C(F)(F)C(F)(F)C(F)(F)C(F)(F)C(F)(F)C(F)(F)C(F)(F)S(=O)(=O)NC(=O)C1=CC=CC=C1		-8.56	0.66	2.94	P	IN
QL02712	1,1,2,2,3,3,4,4,5,5,6,6,7,7,8,8,8-Heptadecafluoro-N-(2-oxocyclooctyl)octane-1-sulfonamide	240801-56-3	FC(F)(F)C(F)(F)C(F)(F)C(F)(F)C(F)(F)C(F)(F)C(F)(F)C(F)(F)S(=O)(=O)NC1CCCCCCC1=O		-8.43	0.66	2.94	P	IN
QL02713	2-[(Perfluorooctane-1-sulfonyl)amino]ethyl prop-2-enoate	60194-47-0	FC(F)(F)C(F)(F)C(F)(F)C(F)(F)C(F)(F)C(F)(F)C(F)(F)C(F)(F)S(=O)(=O)NCCOC(=O)C=C		-8.71	0.66	3.08	P	IN
QL02714	Bis(Perfluorooctylsulfonyl)amine	39847-41-1	FC(F)(F)C(F)(F)C(F)(F)C(F)(F)C(F)(F)C(F)(F)C(F)(F)C(F)(F)S(=O)(=O)NS(=O)(=O)C(F)(F)C(F)(F)C(F)(F)C(F)(F)C(F)(F)C(F)(F)C(F)(F)C(F)(F)F		-10.18	0.69	3.35	P	OUT
QL02715	Perfluoro-1-octanesulfonyl chloride	423-60-9	FC(F)(F)C(F)(F)C(F)(F)C(F)(F)C(F)(F)C(F)(F)C(F)(F)C(F)(F)S(Cl)(=O)=O		-8.37	0.69	2.44	P	IN
QL02716	Perfluorooctanenitrile	647-12-1	FC(F)(F)C(F)(F)C(F)(F)C(F)(F)C(F)(F)C(F)(F)C(F)(F)C#N		-7.58	0.68	2.23	P	IN
QL02717	(Perfluoroheptyl)ethene	25431-45-2	FC(F)(F)C(F)(F)C(F)(F)C(F)(F)C(F)(F)C(F)(F)C(F)(F)C=C		-7.58	0.68	2.23	P	IN
QL02718	(8E)-1,1,1,2,2,3,3,4,4,5,5,6,6,7,7,10,10,11,11,12,12,13,13,14,14,15,15,16,16,16-Triacontafluorohexadec-8-ene	35709-15-0	FC(F)(F)C(F)(F)C(F)(F)C(F)(F)C(F)(F)C(F)(F)C(F)(F)C=CC(F)(F)C(F)(F)C(F)(F)C(F)(F)C(F)(F)C(F)(F)C(F)(F)F		-9.53	0.69	3.08	P	OUT
QL02719	AC1LBTUH	29723-33-9	FC(F)(F)C(F)(F)C(F)(F)C(F)(F)C(F)(F)C(F)(F)C(F)(F)C=NCCC1=CC=CC=C1		-8.10	0.65	2.96	P	OUT
QL02720	p-(Pentadecafluoroheptyl)benzenesulphonyl chloride	25444-35-3	FC(F)(F)C(F)(F)C(F)(F)C(F)(F)C(F)(F)C(F)(F)C(F)(F)C1=CC=C(C=C1)S(Cl)(=O)=O		-8.38	0.67	2.74	P	IN
QL02721	2,4,6-Tris(pentadecafluoroheptyl)-1,3,5-triazine	21674-38-4	FC(F)(F)C(F)(F)C(F)(F)C(F)(F)C(F)(F)C(F)(F)C(F)(F)C1=NC(=NC(=N1)C(F)(F)C(F)(F)C(F)(F)C(F)(F)C(F)(F)C(F)(F)C(F)(F)F)C(F)(F)C(F)(F)C(F)(F)C(F)(F)C(F)(F)C(F)(F)C(F)(F)F		-9.60	0.69	3.11	P	OUT
QL02722	2,2-Diazido-2,2-dihydro-4,6-bis(pentadecafluoroheptyl)1,3,5,2-triazaphosphorine	95215-99-9	FC(F)(F)C(F)(F)C(F)(F)C(F)(F)C(F)(F)C(F)(F)C(F)(F)C1=NP(N=[N+]=[N-])(N=[N+]=[N-])=NC(=N1)C(F)(F)C(F)(F)C(F)(F)C(F)(F)C(F)(F)C(F)(F)C(F)(F)F		-9.16	0.67	3.20	P	OUT
QL02723	5-(Pentadecafluoroheptyl)-1H-imidazole	81769-55-3	FC(F)(F)C(F)(F)C(F)(F)C(F)(F)C(F)(F)C(F)(F)C(F)(F)c1Ncnc1		-7.75	0.67	2.45	P	IN
QL02724	1,1,1,2,2,3,3,4,4,5,5,6,6,7,7,14,14,15,15,16,16,17,17,18,18,19,19,20,20,20-Triacontafluoro-9,12-diiodoicosane	25474-72-0	FC(F)(F)C(F)(F)C(F)(F)C(F)(F)C(F)(F)C(F)(F)C(F)(F)CC(I)CCC(I)CC(F)(F)C(F)(F)C(F)(F)C(F)(F)C(F)(F)C(F)(F)C(F)(F)F		-9.68	0.68	3.34	P	OUT
QL02725	1 OH-substituted n2 fluorotelomer sulfonic acids, n=7		FC(F)(F)C(F)(F)C(F)(F)C(F)(F)C(F)(F)C(F)(F)C(F)(F)CC(O)S(=O)(O)=O		-8.17	0.68	2.51	P	IN
QL02726	2-(2,2,3,3,4,4,5,5,6,6,7,7,8,8,8-Pentadecafluorooctyl)oxirane	20084-49-5	FC(F)(F)C(F)(F)C(F)(F)C(F)(F)C(F)(F)C(F)(F)C(F)(F)CC1CO1		-7.96	0.68	2.45	P	IN
QL02727	2-Propenoic acid, 3,3,4,4,5,5,6,6,7,7,8,8,9,9,9-pentadecafluorononyl ester	1799-55-9	FC(F)(F)C(F)(F)C(F)(F)C(F)(F)C(F)(F)C(F)(F)C(F)(F)CCOC(=O)C=C		-7.98	0.66	2.75	P	IN
QL02728	n2 perfluoroalkyl acrylates, n=7		FC(F)(F)C(F)(F)C(F)(F)C(F)(F)C(F)(F)C(F)(F)C(F)(F)CCOC(CC(C)=C)=O		-8.24	0.65	2.91	P	IN
QL02729	1-Iodo-1H,1H-perfluorooctane	10258-49-8	FC(F)(F)C(F)(F)C(F)(F)C(F)(F)C(F)(F)C(F)(F)C(F)(F)CI		-7.58	0.68	2.23	P	IN
QL02730	N-(2,2,3,3,4,4,5,5,6,6,7,7,8,8,8-Pentadecafluorooctyl)prop-2-enamide	424-01-1	FC(F)(F)C(F)(F)C(F)(F)C(F)(F)C(F)(F)C(F)(F)C(F)(F)CNC(=O)C=C		-8.02	0.66	2.70	P	IN
QL02731	Bis(1H,1H-perfluorooct-1-yl)amine	3145-68-4	FC(F)(F)C(F)(F)C(F)(F)C(F)(F)C(F)(F)C(F)(F)C(F)(F)CNCC(F)(F)C(F)(F)C(F)(F)C(F)(F)C(F)(F)C(F)(F)C(F)(F)F		-9.59	0.68	3.16	P	OUT
QL02732	Bis(2,2,3,3,4,4,5,5,6,6,7,7,8,8,8-pentadecafluorooctyl) (2E)-but-2-enedioate	24120-18-1	FC(F)(F)C(F)(F)C(F)(F)C(F)(F)C(F)(F)C(F)(F)C(F)(F)COC(=O)C=CC(=O)OCC(F)(F)C(F)(F)C(F)(F)C(F)(F)C(F)(F)C(F)(F)C(F)(F)F		-9.76	0.67	3.45	P	OUT
QL02733	Bis(2,2,3,3,4,4,5,5,6,6,7,7,8,8,8-pentadecafluorooctyl) 3,4,5,6-tetrachlorobenzene-1,2-dicarboxylate	2212-77-3	FC(F)(F)C(F)(F)C(F)(F)C(F)(F)C(F)(F)C(F)(F)C(F)(F)COC(=O)c1c(Cl)c(Cl)c(Cl)c(Cl)c1C(=O)OCC(F)(F)C(F)(F)C(F)(F)C(F)(F)C(F)(F)C(F)(F)C(F)(F)F		-9.81	0.67	3.49	P	OUT
QL02734	multiple H-substituted-ether-substituted-perfluoroalkyl (linear) carboxylic acids, n=7		FC(F)(F)C(F)(F)C(F)(F)C(F)(F)C(F)(F)C(F)(F)C(F)(F)COCC(O)=O		-8.04	0.66	2.70	P	IN
QL02735	Allyl 1H,1H-perfluorooctyl ether	812-72-6	FC(F)(F)C(F)(F)C(F)(F)C(F)(F)C(F)(F)C(F)(F)C(F)(F)COCC=C		-7.85	0.65	2.70	P	IN
QL02736	AC1MCPYN	186043-52-7	FC(F)(F)C(F)(F)C(F)(F)C(F)(F)C(F)(F)C(F)(F)C(F)(F)COP1(OCC(F)(F)C(F)(F)C(F)(F)C(F)(F)C(F)(F)C(F)(F)C(F)(F)F)=NP(OCC(F)(F)C(F)(F)C(F)(F)C(F)(F)C(F)(F)C(F)(F)C(F)(F)F)(OCC(F)(F)C(F)(F)C(F)(F)C(F)(F)C(F)(F)C(F)(F)C(F)(F)F)=NP(OCC(F)(F)C(F)(F)C(F)(F)C(F)(F)C(F)(F)C(F)(F)C(F)(F)F)(OCC(F)(F)C(F)(F)C(F)(F)C(F)(F)C(F)(F)C(F)(F)C(F)(F)F)=N1		-9.87	0.69	3.30	P	OUT
QL02737	2,2,3,3,4,4,5,5,6,6,7,7,8,8,8-Pentadecafluorooctyl nonafluorobutane-1-sulfonate	187039-77-6	FC(F)(F)C(F)(F)C(F)(F)C(F)(F)C(F)(F)C(F)(F)C(F)(F)COS(=O)(=O)C(F)(F)C(F)(F)C(F)(F)C(F)(F)F		-9.21	0.69	2.93	P	OUT
QL02738	2,2,3,3,4,4,5,5,6,6,7,7,8,8,8-Pentadecafluorooctyl trifluoromethanesulfonate	17352-09-9	FC(F)(F)C(F)(F)C(F)(F)C(F)(F)C(F)(F)C(F)(F)C(F)(F)COS(=O)(=O)C(F)(F)F		-8.61	0.68	2.68	P	IN

QL02739	n1 fluorotelomer sulfonic acids, n=7		FC(F)(F)C(F)(F)C(F)(F)C(F)(F)C(F)(F)C(F)(F)C(F)(F)CS(=O)(O)=O		-8.06	0.68	2.44	P	IN
QL02740	Perfluoroheptane	335-57-9	FC(F)(F)C(F)(F)C(F)(F)C(F)(F)C(F)(F)C(F)(F)C(F)(F)F		-7.74	0.69	2.15	P	IN
QL02741	1-Iodopentadecafluoroheptane	335-58-0	FC(F)(F)C(F)(F)C(F)(F)C(F)(F)C(F)(F)C(F)(F)C(F)(F)I		-7.74	0.69	2.15	P	IN
QL02742	1H-Perfluorooct-1-yne	55756-24-6	FC(F)(F)C(F)(F)C(F)(F)C(F)(F)C(F)(F)C(F)(F)C#C		-7.35	0.68	2.15	P	IN
QL02743	Perfluoro-1-(tetrafluoroiodo)-1-octyne	1424379-21-4	FC(F)(F)C(F)(F)C(F)(F)C(F)(F)C(F)(F)C(F)(F)C#C[I](F)(F)(F)F		-8.17	0.69	2.43	P	IN
QL02744	3,3,4,4,5,5,6,6,7,7,8,8,8-Tridecafluoro-1-iodooct-1-yne	58292-95-8	FC(F)(F)C(F)(F)C(F)(F)C(F)(F)C(F)(F)C(F)(F)C#CI		-7.16	0.66	2.23	P	IN
QL02745	Perfluoroheptanenitrile	62038-08-8	FC(F)(F)C(F)(F)C(F)(F)C(F)(F)C(F)(F)C(F)(F)C#N		-7.35	0.68	2.15	P	IN
QL02746	(7E)-1,1,1,2,2,3,3,4,4,5,5,6,6,9,9,10,10,11,11,12,12,13,13,14,14,15,15,16,16,16-Triacontafluorohexadec-7-ene	51249-68-4	FC(F)(F)C(F)(F)C(F)(F)C(F)(F)C(F)(F)C(F)(F)C=CC(F)(F)C(F)(F)C(F)(F)C(F)(F)C(F)(F)C(F)(F)C(F)(F)C(F)(F)F		-9.60	0.69	3.11	P	OUT
QL02748	1,1,1,2,2,3,3,4,4,5,5,6,6,9,9,10,10,11,11,12,12,13,13,14,14,14-Hexacosafluorotetradec-7-ene	56523-43-4	FC(F)(F)C(F)(F)C(F)(F)C(F)(F)C(F)(F)C(F)(F)C=CC(F)(F)C(F)(F)C(F)(F)C(F)(F)C(F)(F)C(F)(F)F		-9.13	0.69	2.90	P	OUT
QL02749	(3E)-5,5,6,6,7,7,8,8,9,9,10,10,10-Tridecafluorodeca-1,3-diene	148626-04-4	FC(F)(F)C(F)(F)C(F)(F)C(F)(F)C(F)(F)C(F)(F)C=CC=C		-7.41	0.66	2.46	P	IN
QL02750	3,3,4,4,5,5,6,6,7,7,8,8,8-Tridecafluoro-1-iodooct-1-ene	150223-14-6	FC(F)(F)C(F)(F)C(F)(F)C(F)(F)C(F)(F)C(F)(F)C=CI		-7.16	0.66	2.23	P	IN
QL02752	Oxirane, trifluoro(tridecafluorohexyl)-	87375-53-9	FC(F)(F)C(F)(F)C(F)(F)C(F)(F)C(F)(F)C(F)(F)C1(F)OC1(F)F		-8.00	0.69	2.24	P	IN
QL02753	1-Bromo-4-(tridecafluorohexyl)benzene	149068-56-4	FC(F)(F)C(F)(F)C(F)(F)C(F)(F)C(F)(F)C(F)(F)C1=CC=C(Br)C=C1		-7.72	0.66	2.52	P	IN
QL02754	1-[4-(Tridecafluorohexyl)phenyl]pyridin-4(1H)-one	821789-63-3	FC(F)(F)C(F)(F)C(F)(F)C(F)(F)C(F)(F)C(F)(F)C1=CC=C(C=C1)N1C=CC(=O)C=C1		-8.24	0.65	2.98	P	IN
QL02755	Tris[4-(tridecafluorohexyl)phenyl]phosphine	193197-68-1	FC(F)(F)C(F)(F)C(F)(F)C(F)(F)C(F)(F)C(F)(F)C1=CC=C(C=C1)P(C1=CC=C(C=C1)C(F)(F)C(F)(F)C(F)(F)C(F)(F)C(F)(F)C(F)(F)F)C1=CC=C(C=C1)C(F)(F)C(F)(F)C(F)(F)C(F)(F)C(F)(F)C(F)(F)F		-9.62	0.67	3.40	P	OUT
QL02756	1,1'-Oxybis[4-(tridecafluorohexyl)benzene]	151707-03-8	FC(F)(F)C(F)(F)C(F)(F)C(F)(F)C(F)(F)C(F)(F)C1=CC=C(OC2=CC=C(C=C2)C(F)(F)C(F)(F)C(F)(F)C(F)(F)C(F)(F)C(F)(F)F)C=C1		-9.58	0.67	3.42	P	OUT
QL02757	(Tridecafluorohexyl)benzene	65440-93-9	FC(F)(F)C(F)(F)C(F)(F)C(F)(F)C(F)(F)C(F)(F)C1=CC=CC=C1		-7.48	0.66	2.45	P	IN
QL02758	Thiophene, 2-(tridecafluorohexyl)-	128032-22-4	FC(F)(F)C(F)(F)C(F)(F)C(F)(F)C(F)(F)C(F)(F)C1=CC=CS1		-7.24	0.66	2.24	P	IN
QL02759	3-(Tridecafluorohexyl)thiophene	128032-23-5	FC(F)(F)C(F)(F)C(F)(F)C(F)(F)C(F)(F)C(F)(F)C1=CSC=C1		-7.24	0.66	2.24	P	IN
QL02760	2-(Tridecafluorohexyl)-1H-benzimidazole	119403-54-2	FC(F)(F)C(F)(F)C(F)(F)C(F)(F)C(F)(F)C(F)(F)C1=NC2=CC=CC=C2N1		-7.61	0.66	2.52	P	IN
QL02761	1-Benzoyl-3,5-bis(perfluorohexyl)pyrazole	230295-10-0	FC(F)(F)C(F)(F)C(F)(F)C(F)(F)C(F)(F)C(F)(F)C1=NN(C(=O)C2=CC=CC=C2)C(=C1)C(F)(F)C(F)(F)C(F)(F)C(F)(F)C(F)(F)C(F)(F)F		-8.82	0.67	3.02	P	OUT
QL02762	3-(Perfluorohexyl)-5-phenyl-1H-pyrazole	510768-12-4	FC(F)(F)C(F)(F)C(F)(F)C(F)(F)C(F)(F)C(F)(F)C1=NNC(=C1)C1=CC=CC=C1		-7.81	0.65	2.76	P	IN
QL02763	1-Iodo-4-(tridecafluorohexyl)benzene	162083-76-3	FC(F)(F)C(F)(F)C(F)(F)C(F)(F)C(F)(F)C(F)(F)c1ccc(I)cc1		-7.72	0.66	2.52	P	IN
QL02764	5-Bromo-5'-(tridecafluorohexyl)-2,2'-bithiophene	327630-35-3	FC(F)(F)C(F)(F)C(F)(F)C(F)(F)C(F)(F)C(F)(F)c1ccc(s1)-c1ccc(Br)s1		-8.03	0.66	2.76	P	IN
QL02765	1~5~,4~5~-Bis(tridecafluorohexyl)-1~2~,2~2~2~5~,3~2~3~5~,4~2~-quaterthiophene	446043-85-2	FC(F)(F)C(F)(F)C(F)(F)C(F)(F)C(F)(F)C(F)(F)c1ccc(s1)-c1ccc(s1)-c1ccc(s1)-c1ccc(s1)C(F)(F)C(F)(F)C(F)(F)C(F)(F)C(F)(F)C(F)(F)F		-9.82	0.66	3.60	P	OUT
QL02766	5-(Tridecafluorohexyl)-2,2'-bithiophene	327630-34-2	FC(F)(F)C(F)(F)C(F)(F)C(F)(F)C(F)(F)C(F)(F)c1ccc(s1)-c1cccs1		-7.85	0.65	2.70	P	IN
QL02767	1-Bromo-3-(tridecafluorohexyl)benzene	157829-77-1	FC(F)(F)C(F)(F)C(F)(F)C(F)(F)C(F)(F)C(F)(F)c1cccc(Br)c1		-7.56	0.66	2.46	P	IN
QL02768	1,3-Bis(tridecafluorohexyl)benzene	124389-29-3	FC(F)(F)C(F)(F)C(F)(F)C(F)(F)C(F)(F)C(F)(F)c1cccc(c1)C(F)(F)C(F)(F)C(F)(F)C(F)(F)C(F)(F)C(F)(F)F		-9.22	0.68	3.05	P	OUT
QL02769	1,1'-[1,3-Phenylenebis(oxy)]bis[3-(tridecafluorohexyl)benzene]	151707-04-9	FC(F)(F)C(F)(F)C(F)(F)C(F)(F)C(F)(F)C(F)(F)c1cccc(Oc2cccc(Oc3cccc(c3)C(F)(F)C(F)(F)C(F)(F)C(F)(F)C(F)(F)C(F)(F)F)c2)c1		-9.58	0.65	3.59	P	OUT
QL02770	N-Phenyl-N'-[2-(tridecafluorohexyl)phenyl]urea	288161-40-0	FC(F)(F)C(F)(F)C(F)(F)C(F)(F)C(F)(F)C(F)(F)c1ccccc1NC(=O)Nc1ccccc1		-7.76	0.63	2.95	P	OUT
QL02771	2-Iodo-3-(tridecafluorohexyl)thiophene	1453469-53-8	FC(F)(F)C(F)(F)C(F)(F)C(F)(F)C(F)(F)C(F)(F)c1ccsc1I		-7.28	0.67	2.24	P	IN
QL02772	2-Iodo-4-(tridecafluorohexyl)thiophene	1453469-60-7	FC(F)(F)C(F)(F)C(F)(F)C(F)(F)C(F)(F)C(F)(F)c1csc(I)c1		-7.73	0.67	2.45	P	IN
QL02773	3,3'-(1,4-Phenylene)bis[5-(tridecafluorohexyl)-1,2,4-oxadiazole]	146304-71-4	FC(F)(F)C(F)(F)C(F)(F)C(F)(F)C(F)(F)C(F)(F)c1nc(no1)-c1ccc(cc1)-c1noc(n1)C(F)(F)C(F)(F)C(F)(F)C(F)(F)C(F)(F)C(F)(F)F		-9.67	0.67	3.48	P	OUT
QL02774	3-Phenyl-5-(tridecafluorohexyl)-1,2,4-oxadiazole	146304-76-9	FC(F)(F)C(F)(F)C(F)(F)C(F)(F)C(F)(F)C(F)(F)c1nc(no1)-c1ccccc1		-7.81	0.65	2.76	P	IN
QL02775	1,1,1,2,2,3,3,4,4,5,5,6,6,8,8-Pentadecafluoro-10-iododecane	49676-71-3	FC(F)(F)C(F)(F)C(F)(F)C(F)(F)C(F)(F)C(F)(F)CC(F)(F)CCI		-7.63	0.66	2.51	P	IN
QL02776	1,1,1,2,2,3,3,4,4,5,5,6,6,8,8-Pentadecafluoro-8-iodooctane	53826-16-7	FC(F)(F)C(F)(F)C(F)(F)C(F)(F)C(F)(F)C(F)(F)CC(F)(F)I		-7.58	0.68	2.23	P	IN

QL02777	1-(3,3,4,4,5,5,6,6,7,7,8,8,8-Tridecafluoro-1-iodooctyl)-2,8,9-trioxa-5-aza-1-silabicyclo[3.3.3]undecane	135587-15-4	FC(F)(F)C(F)(F)C(F)(F)C(F)(F)C(F)(F)C(F)(F)CC(I)[Si]12OCCN(CCO1)CCO2		-7.74	0.65	2.74	P	IN
QL02778	1,1,1,2,2,3,3,4,4,5,5,6,6,7,7,8,8,11,11,12,12,13,13,14,14,15,15,16,16,16-Triacontafluoro-9-iodohexadecane	53713-87-4	FC(F)(F)C(F)(F)C(F)(F)C(F)(F)C(F)(F)C(F)(F)CC(I)C(F)(F)C(F)(F)C(F)(F)C(F)(F)C(F)(F)C(F)(F)C(F)(F)F		-9.60	0.69	3.11	P	OUT
QL02779	1,1,1,2,2,3,3,4,4,5,5,6,6,9,9,10,10,11,11,12,12,13,13,14,14,14-Hexacosafluoro-7-iodotetradecane	53638-11-2	FC(F)(F)C(F)(F)C(F)(F)C(F)(F)C(F)(F)C(F)(F)CC(I)C(F)(F)C(F)(F)C(F)(F)C(F)(F)C(F)(F)C(F)(F)F		-9.18	0.69	2.92	P	OUT
QL02780	1,1,1,2,2,3,3,4,4,5,5,6,6,12,12,13,13,14,14,15,15,16,16,17,17,17-Hexacosafluoro-8,10-diiodoheptadecane	1254971-76-0	FC(F)(F)C(F)(F)C(F)(F)C(F)(F)C(F)(F)C(F)(F)CC(I)CC(I)CC(F)(F)C(F)(F)C(F)(F)C(F)(F)C(F)(F)C(F)(F)F		-9.31	0.68	3.16	P	OUT
QL02781	1,1,1,2,2,3,3,4,4,5,5,6,6,13,13,14,14,15,15,16,16,17,17,18,18,18-Hexacosafluoro-8,11-diiodooctadecane	959462-50-1	FC(F)(F)C(F)(F)C(F)(F)C(F)(F)C(F)(F)C(F)(F)CC(I)CCC(I)CC(F)(F)C(F)(F)C(F)(F)C(F)(F)C(F)(F)C(F)(F)F		-9.31	0.67	3.20	P	OUT
QL02782	1,1,1,2,2,3,3,4,4,5,5,6,6,15,15,16,16,17,17,18,18,19,19,20,20,20-Hexacosafluoro-8,13-diiodoicosane	959462-51-2	FC(F)(F)C(F)(F)C(F)(F)C(F)(F)C(F)(F)C(F)(F)CC(I)CCCCC(I)CC(F)(F)C(F)(F)C(F)(F)C(F)(F)C(F)(F)C(F)(F)F		-9.34	0.66	3.31	P	OUT
QL02783	1 OH-substituted n2 fluorotelomer sulfonic acids, n=6		FC(F)(F)C(F)(F)C(F)(F)C(F)(F)C(F)(F)C(F)(F)CC(O)S(=O)(O)=O		-7.98	0.68	2.45	P	IN
QL02784	CTK5F5734	857637-92-4	FC(F)(F)C(F)(F)C(F)(F)C(F)(F)C(F)(F)C(F)(F)CC[C@H]1OC(=O)N[C@H]1CC1=CC=CC=C1		-7.86	0.64	2.91	P	OUT
QL02785	Trichloro(3,3,4,4,5,5,6,6,7,7,8,8,8-tridecafluorooctyl)silane	78560-45-9	FC(F)(F)C(F)(F)C(F)(F)C(F)(F)C(F)(F)C(F)(F)CC[Si](Cl)(Cl)Cl		-7.90	0.67	2.44	P	IN
QL02786	1,1-dichloro-3,3,3-trimethyl-1-(3,3,4,4,5,5,6,6,7,7,8,8,8-tridecafluorooctyl)disiloxane	94237-17-9	FC(F)(F)C(F)(F)C(F)(F)C(F)(F)C(F)(F)C(F)(F)CC[Si](Cl)(Cl)O[Si](C)(C)C		-8.26	0.67	2.68	P	IN
QL02787	3-chloro-1,1,1,5,5,5-hexamethyl-3-(3,3,4,4,5,5,6,6,7,7,8,8,8-tridecafluorooctyl)trisiloxane	94237-06-6	FC(F)(F)C(F)(F)C(F)(F)C(F)(F)C(F)(F)C(F)(F)CC[Si](Cl)(O[Si](C)(C)C)O[Si](C)(C)C		-8.04	0.67	2.63	P	IN
QL02788	Methoxydimethyl(3,3,4,4,5,5,6,6,7,7,8,8,8-tridecafluorooctyl)silane	94237-08-8	FC(F)(F)C(F)(F)C(F)(F)C(F)(F)C(F)(F)C(F)(F)CC[Si](OC)(C)C		-7.82	0.67	2.51	P	IN
QL02789	Trimethoxy(3,3,4,4,5,5,6,6,7,7,8,8,8-tridecafluorooctyl)silane	85857-16-5	FC(F)(F)C(F)(F)C(F)(F)C(F)(F)C(F)(F)C(F)(F)CC[Si](OC)(OC)OC		-7.35	0.65	2.50	P	IN
QL02790	Triethoxy(3,3,4,4,5,5,6,6,7,7,8,8,8-tridecafluorooctyl)silane	51851-37-7	FC(F)(F)C(F)(F)C(F)(F)C(F)(F)C(F)(F)C(F)(F)CC[Si](OCC)(OCC)OCC		-7.03	0.63	2.68	P	OUT
QL02791	Dichloromethyl(3,3,4,4,5,5,6,6,7,7,8,8,8-tridecafluorooctyl)silane	73609-36-6	FC(F)(F)C(F)(F)C(F)(F)C(F)(F)C(F)(F)C(F)(F)CC[SiH2]C(Cl)Cl		-7.57	0.66	2.52	P	IN
QL02792	Dimethoxymethyl(3,3,4,4,5,5,6,6,7,7,8,8,8-tridecafluorooctyl)silane	85857-17-6	FC(F)(F)C(F)(F)C(F)(F)C(F)(F)C(F)(F)C(F)(F)CC[SiH2]C(OC)OC		-7.54	0.64	2.71	P	IN
QL02793	(3,3,4,4,5,5,6,6,7,7,8,8,8-Tridecafluorooctyl)silane	469904-32-3	FC(F)(F)C(F)(F)C(F)(F)C(F)(F)C(F)(F)C(F)(F)CC[SiH3]		-7.16	0.66	2.23	P	IN
QL02794	Bis((perfluorohexyl)ethyl)stannanone	324063-66-3	FC(F)(F)C(F)(F)C(F)(F)C(F)(F)C(F)(F)C(F)(F)CC[Sn](=O)CCC(F)(F)C(F)(F)C(F)(F)C(F)(F)C(F)(F)C(F)(F)F		-9.32	0.68	3.15	P	OUT
QL02795	Tris(3,3,4,4,5,5,6,6,7,7,8,8,8-tridecafluorooctyl)tin bromide	175354-31-1	FC(F)(F)C(F)(F)C(F)(F)C(F)(F)C(F)(F)C(F)(F)CC[Sn](Br)(CCC(F)(F)C(F)(F)C(F)(F)C(F)(F)C(F)(F)C(F)(F)F)CCC(F)(F)C(F)(F)C(F)(F)C(F)(F)C(F)(F)C(F)(F)F		-9.46	0.68	3.12	P	OUT
QL02796	Phenyltris((perfluorohexyl)ethyl)stannane	175354-30-0	FC(F)(F)C(F)(F)C(F)(F)C(F)(F)C(F)(F)C(F)(F)CC[Sn](CCC(F)(F)C(F)(F)C(F)(F)C(F)(F)C(F)(F)C(F)(F)F)(CCC(F)(F)C(F)(F)C(F)(F)C(F)(F)C(F)(F)C(F)(F)F)C1=CC=CC=C1		-9.28	0.67	3.17	P	OUT
QL02797	Allyltris(3,3,4,4,5,5,6,6,7,7,8,8,8-tridecafluorooctyl)stannane	192212-66-1	FC(F)(F)C(F)(F)C(F)(F)C(F)(F)C(F)(F)C(F)(F)CC[Sn](CCC(F)(F)C(F)(F)C(F)(F)C(F)(F)C(F)(F)C(F)(F)F)(CCC(F)(F)C(F)(F)C(F)(F)C(F)(F)C(F)(F)C(F)(F)F)CC=C		-9.30	0.68	3.15	P	OUT
QL02798	Tris(3,3,4,4,5,5,6,6,7,7,8,8,8-tridecafluorooctyl)tin azide	201740-73-0	FC(F)(F)C(F)(F)C(F)(F)C(F)(F)C(F)(F)C(F)(F)CC[Sn](CCC(F)(F)C(F)(F)C(F)(F)C(F)(F)C(F)(F)C(F)(F)F)(CCC(F)(F)C(F)(F)C(F)(F)C(F)(F)C(F)(F)C(F)(F)F)N=[N+]=[N-]		-9.30	0.68	3.15	P	OUT
QL02799	Tris((perfluorohexyl)ethyl)stannane	175354-32-2	FC(F)(F)C(F)(F)C(F)(F)C(F)(F)C(F)(F)C(F)(F)CC[SnH](CCC(F)(F)C(F)(F)C(F)(F)C(F)(F)C(F)(F)C(F)(F)F)CCC(F)(F)C(F)(F)C(F)(F)C(F)(F)C(F)(F)C(F)(F)F		-9.35	0.68	3.10	P	OUT
QL02800	4-(2,2,3,3,4,4,5,5,6,6,7,7,7-Tridecafluoroheptyl)-1,3-dioxolan-2-one	114810-52-5	FC(F)(F)C(F)(F)C(F)(F)C(F)(F)C(F)(F)C(F)(F)CC1COC(=O)O1		-7.72	0.66	2.52	P	IN
QL02801	1-bromo-1h,1h,2h,2h-perfluorooctane	161583-34-2	FC(F)(F)C(F)(F)C(F)(F)C(F)(F)C(F)(F)C(F)(F)CCBr		-7.16	0.66	2.23	P	IN
QL02802	1-(4,4,5,5,6,6,7,7,8,8,9,9,9-Tridecafluoro-1-oxononyl)-2,5-pyrrolidinedione	932710-51-5	FC(F)(F)C(F)(F)C(F)(F)C(F)(F)C(F)(F)C(F)(F)CCC(=O)N1C(=O)CCC1=O		-7.88	0.66	2.68	P	IN
QL02803	1-[(4,4,5,5,6,6,7,7,8,8,9,9,9-Tridecafluorononanoyl)oxy]pyrrolidine-2,5-dione	1262770-54-6	FC(F)(F)C(F)(F)C(F)(F)C(F)(F)C(F)(F)C(F)(F)CCC(=O)ON1C(=O)CCC1=O		-7.87	0.65	2.73	P	IN
QL02804	Trichloro[5,5,6,6,7,7,8,8,9,9,10,10,10-tridecafluoro-2-(tridecafluorohexyl)decyl]silane	1621953-16-9	FC(F)(F)C(F)(F)C(F)(F)C(F)(F)C(F)(F)C(F)(F)CCC(C[Si](Cl)(Cl)Cl)C(F)(F)C(F)(F)C(F)(F)C(F)(F)C(F)(F)C(F)(F)F		-9.09	0.68	2.98	P	OUT

QL02805	1,1,1,2,2,3,3,4,4,5,5,6,6,9,9,10,10,11,11,12,12,13,13,14,14,14-Hexacosafluorotetradecane	53749-64-7	FC(F)(F)C(F)(F)C(F)(F)C(F)(F)C(F)(F)C(F)(F)CCC(F)(F)C(F)(F)C(F)(F)C(F)(F)C(F)(F)C(F)(F)F		-9.13	0.69	2.90	P	OUT
QL02806	Tris[4-(3,3,4,4,5,5,6,6,7,7,8,8,8-tridecafluorooctyl)phenyl]phosphine	219985-31-6	FC(F)(F)C(F)(F)C(F)(F)C(F)(F)C(F)(F)C(F)(F)CCC1=CC=C(C=C1)P(C1=CC=C(CCC(F)(F)C(F)(F)C(F)(F)C(F)(F)C(F)(F)C(F)(F)F)C=C1)C1=CC=C(CCC(F)(F)C(F)(F)C(F)(F)C(F)(F)C(F)(F)C(F)(F)F)C=C1		-9.76	0.66	3.60	P	OUT
QL02807	Phenylbis[4-(3,3,4,4,5,5,6,6,7,7,8,8,8-tridecafluorooctyl)phenyl]phosphane	290827-94-0	FC(F)(F)C(F)(F)C(F)(F)C(F)(F)C(F)(F)C(F)(F)CCC1=CC=C(C=C1)P(C1=CC=CC=C1)C1=CC=C(CCC(F)(F)C(F)(F)C(F)(F)C(F)(F)C(F)(F)C(F)(F)F)C=C1		-9.53	0.65	3.64	P	OUT
QL02808	N-[4-(3,3,4,4,5,5,6,6,7,7,8,8,8-Tridecafluorooctyl)benzyloxycarbonyloxy] succinimide	556050-48-7	FC(F)(F)C(F)(F)C(F)(F)C(F)(F)C(F)(F)C(F)(F)CCC1=CC=C(COC(=O)ON2C(=O)CCC2=O)C=C1		-8.39	0.64	3.22	P	OUT
QL02809	[2,7-Bis((perfluorohexyl)ethyl)-9H-fluoren-9-yl]methyl carbonochloridate	932710-57-1	FC(F)(F)C(F)(F)C(F)(F)C(F)(F)C(F)(F)C(F)(F)CCC1=CC2=C(C=C1)C1=C(C=C(CCC(F)(F)C(F)(F)C(F)(F)C(F)(F)C(F)(F)C(F)(F)F)C=C1)C2COC(Cl)=O		-9.59	0.66	3.58	P	OUT
QL02810	1-Bromo-4-(3,3,4,4,5,5,6,6,7,7,8,8,8-tridecafluorooctyl)benzene	195324-87-9	FC(F)(F)C(F)(F)C(F)(F)C(F)(F)C(F)(F)C(F)(F)CCc1ccc(Br)cc1		-7.96	0.65	2.76	P	IN
QL02811	10-Bromo-1,1,1,2,2,3,3,4,4,5,5,6,6-tridecafluorodecane	181997-60-4	FC(F)(F)C(F)(F)C(F)(F)C(F)(F)C(F)(F)C(F)(F)CCCCBr		-7.35	0.65	2.52	P	IN
QL02812	1,1,1,2,2,3,3,4,4,5,5,6,6,13,13,14,14,15,15,16,16,17,17,18,18,18-Hexacosafluorooctadecane	959462-53-4	FC(F)(F)C(F)(F)C(F)(F)C(F)(F)C(F)(F)C(F)(F)CCCCCCC(F)(F)C(F)(F)C(F)(F)C(F)(F)C(F)(F)C(F)(F)F		-9.19	0.67	3.16	P	OUT
QL02813	12,12,13,13,14,14,15,15,16,16,17,17,17-Tridecafluoroheptadecanoyl chloride	118624-66-1	FC(F)(F)C(F)(F)C(F)(F)C(F)(F)C(F)(F)C(F)(F)CCCCCCCCCCC(Cl)=O		-7.95	0.62	3.20	P	OUT
QL02814	1,1,1,2,2,3,3,4,4,5,5,6,6,17,17,18,18,19,19,20,20,21,21,22,22,22-Hexacosafluorodocosane	1835255-18-9	FC(F)(F)C(F)(F)C(F)(F)C(F)(F)C(F)(F)C(F)(F)CCCCCCCCCCC(F)(F)C(F)(F)C(F)(F)C(F)(F)C(F)(F)C(F)(F)F		-9.25	0.66	3.40	P	OUT
QL02815	17-Bromo-1,1,1,2,2,3,3,4,4,5,5,6,6-tridecafluoroheptadecane	155401-47-1	FC(F)(F)C(F)(F)C(F)(F)C(F)(F)C(F)(F)C(F)(F)CCCCCCCCCCCBr		-7.78	0.62	3.21	P	OUT
QL02816	(11-(F-Hexyl)undecyl) dimorpholinophosphoramidate	134051-91-5	FC(F)(F)C(F)(F)C(F)(F)C(F)(F)C(F)(F)C(F)(F)CCCCCCCCCCCOP(=O)(N1CCOCC1)N1CCOCC1		-7.93	0.61	3.39	P	OUT
QL02817	1,1,1,2,2,3,3,4,4,5,5,6,6-Tridecafluoro-10-iodododecane	38565-61-6	FC(F)(F)C(F)(F)C(F)(F)C(F)(F)C(F)(F)C(F)(F)CCCCI		-7.35	0.65	2.52	P	IN
QL02818	5,5,6,6,7,7,8,8,9,9,10,10,10-Tridecafluorodecyl prop-2-enoate	451456-30-7	FC(F)(F)C(F)(F)C(F)(F)C(F)(F)C(F)(F)C(F)(F)CCCCOC(=O)C=C		-7.82	0.64	2.89	P	OUT
QL02819	1,1,1,2,2,3,3,4,4,5,5,6,6-Tridecafluoro-9-iodononane	89889-20-3	FC(F)(F)C(F)(F)C(F)(F)C(F)(F)C(F)(F)C(F)(F)CCCI		-7.41	0.66	2.46	P	IN
QL02820	3,3,4,4,5,5,6,6,7,7,8,8,8-Tridecafluorooctyl chloride	64018-25-3	FC(F)(F)C(F)(F)C(F)(F)C(F)(F)C(F)(F)C(F)(F)CCCl		-7.16	0.66	2.23	P	IN
QL02821	4,4,5,5,6,6,7,7,8,8,9,9,9-Tridecafluorononyl azide	852527-60-7	FC(F)(F)C(F)(F)C(F)(F)C(F)(F)C(F)(F)C(F)(F)CCCN=[N+]=[N-]		-7.58	0.64	2.72	P	IN
QL02822	1-(4,4,5,5,6,6,7,7,8,8,9,9,9-Tridecafluorononyl)-1H-pyrrole-2,5-dione	852527-41-4	FC(F)(F)C(F)(F)C(F)(F)C(F)(F)C(F)(F)C(F)(F)CCCN1C(=O)C=CC1=O		-7.83	0.66	2.68	P	IN
QL02823	2-Iodo-N-(4,4,5,5,6,6,7,7,8,8,9,9,9-tridecafluorononyl)acetamide	852527-50-5	FC(F)(F)C(F)(F)C(F)(F)C(F)(F)C(F)(F)C(F)(F)CCCNC(=O)CI		-7.72	0.64	2.77	P	IN
QL02824	4,4,5,5,6,6,7,7,8,8,9,9,9-Tridecafluorononyl prop-2-enoate	216389-85-4	FC(F)(F)C(F)(F)C(F)(F)C(F)(F)C(F)(F)C(F)(F)CCCOC(=O)C=C		-7.72	0.64	2.77	P	IN
QL02825	Bis(4,4,5,5,6,6,7,7,8,8,9,9,9-tridecafluorononyl) diazene-1,2-dicarboxylate	462996-01-6	FC(F)(F)C(F)(F)C(F)(F)C(F)(F)C(F)(F)C(F)(F)CCCOC(=O)N=NC(=O)OCCCC(F)(F)C(F)(F)C(F)(F)C(F)(F)C(F)(F)C(F)(F)F		-9.46	0.65	3.53	P	OUT
QL02826	2-Chloro-4,6-bis[(4,4,5,5,6,6,7,7,8,8,9,9,9-tridecafluorononyl)oxy]-1,3,5-triazine	916770-15-5	FC(F)(F)C(F)(F)C(F)(F)C(F)(F)C(F)(F)C(F)(F)CCCOC1=NC(OCCCC(F)(F)C(F)(F)C(F)(F)C(F)(F)C(F)(F)C(F)(F)F)=NC(Cl)=N1		-9.47	0.66	3.51	P	OUT
QL02827	8-azido-1,1,1,2,2,3,3,4,4,5,5,6,6-tridecafluorooctane	62571-55-5	FC(F)(F)C(F)(F)C(F)(F)C(F)(F)C(F)(F)C(F)(F)CCN=[N+]=[N-]		-7.35	0.65	2.52	P	IN
QL02828	1,1,1,2,2,3,3,4,4,5,5,6,6-Tridecafluoro-8-isocyanatooctane	142010-49-9	FC(F)(F)C(F)(F)C(F)(F)C(F)(F)C(F)(F)C(F)(F)CCN=C=O		-7.35	0.65	2.52	P	IN
QL02829	62 Fluorotelomer acrylate	17527-29-6	FC(F)(F)C(F)(F)C(F)(F)C(F)(F)C(F)(F)C(F)(F)CCOC(=O)C=C		-7.77	0.65	2.72	P	IN
QL02830	Bis(3,3,4,4,5,5,6,6,7,7,8,8,8-tridecafluorooctyl) but-2-enedioate	55003-96-8	FC(F)(F)C(F)(F)C(F)(F)C(F)(F)C(F)(F)C(F)(F)CCOC(=O)C=CC(=O)OCCC(F)(F)C(F)(F)C(F)(F)C(F)(F)C(F)(F)C(F)(F)F		-9.43	0.66	3.42	P	OUT
QL02831	Bis(3,3,4,4,5,5,6,6,7,7,8,8,8-tridecafluorooctyl) 2-methylidenebutanedioate	49676-56-4	FC(F)(F)C(F)(F)C(F)(F)C(F)(F)C(F)(F)C(F)(F)CCOC(=O)CC(=C)C(=O)OCCC(F)(F)C(F)(F)C(F)(F)C(F)(F)C(F)(F)C(F)(F)F		-9.50	0.66	3.44	P	OUT
QL02832	Bis((perfluorohexyl)ethyl) diazene-1,2-dicarboxylate	452912-11-7	FC(F)(F)C(F)(F)C(F)(F)C(F)(F)C(F)(F)C(F)(F)CCOC(=O)N=NC(=O)OCCC(F)(F)C(F)(F)C(F)(F)C(F)(F)C(F)(F)C(F)(F)F		-9.43	0.66	3.42	P	OUT
QL02833	Ethenyl 3,3,4,4,5,5,6,6,7,7,8,8,8-tridecafluorooctyl carbonate	96383-57-2	FC(F)(F)C(F)(F)C(F)(F)C(F)(F)C(F)(F)C(F)(F)CCOC(=O)OC=C		-7.65	0.64	2.77	P	IN
QL02834	n2 perfluoroalkyl acrylates, n=6		FC(F)(F)C(F)(F)C(F)(F)C(F)(F)C(F)(F)C(F)(F)CCOC(CC(C)=C)=O		-7.81	0.65	2.76	P	IN
QL02835	1,1,1,2,2,3,3,4,4,5,5,6,6-Tridecafluoro-8-[(prop-2-yn-1-yl)oxy]octane	1334419-24-7	FC(F)(F)C(F)(F)C(F)(F)C(F)(F)C(F)(F)C(F)(F)CCOCC#C		-7.58	0.64	2.72	P	IN
QL02836	1,1,1,2,2,3,3,4,4,5,5,6,6-Tridecafluoro-8-[(prop-2-en-1-yl)oxy]octane	103628-86-0	FC(F)(F)C(F)(F)C(F)(F)C(F)(F)C(F)(F)C(F)(F)CCOCC=C		-7.58	0.64	2.72	P	IN

QL02837	2-{[(3,3,4,4,5,5,6,6,7,7,8,8,8-Tridecafluorooctyl)oxy]methyl}oxirane	122193-68-4	FC(F)(F)C(F)(F)C(F)(F)C(F)(F)C(F)(F)C(F)(F)CCOCC1CO1		-7.91	0.66	2.72	P	IN
QL02838	5-{[(3,3,4,4,5,5,6,6,7,7,8,8,8-Tridecafluorooctyl)oxy]methyl}-1H-1,2,3-triazole	1447923-23-0	FC(F)(F)C(F)(F)C(F)(F)C(F)(F)C(F)(F)C(F)(F)CCOCc1Nnnc1		-7.70	0.64	2.76	P	IN
QL02839	multiple H-substituted, multiple ether-substituted perfluoroalkyl alcohols, n=3, m=5		FC(F)(F)C(F)(F)C(F)(F)C(F)(F)C(F)(F)C(F)(F)CCOCCOCCOCCOCCO[H]		-7.83	0.61	3.38	P	OUT
QL02840	multiple H-substituted, multiple ether-substituted perfluoroalkyl alcohols, n=3, m=6		FC(F)(F)C(F)(F)C(F)(F)C(F)(F)C(F)(F)C(F)(F)CCOCCOCCOCCOCCOCCO[H]		-7.99	0.60	3.59	P	OUT
QL02841	multiple H-substituted, multiple ether-substituted perfluoroalkyl alcohols, n=3, m=7		FC(F)(F)C(F)(F)C(F)(F)C(F)(F)C(F)(F)C(F)(F)CCOCCOCCOCCOCCOCCOCCO[H]		-8.06	0.59	3.72	P	OUT
QL02842	multiple H-substituted, multiple ether-substituted perfluoroalkyl alcohols, n=3, m=8		FC(F)(F)C(F)(F)C(F)(F)C(F)(F)C(F)(F)C(F)(F)CCOCCOCCOCCOCCOCCOCCOCCO[H]		-8.22	0.58	3.89	P	OUT
QL02843	multiple H-substituted, multiple ether-substituted perfluoroalkyl alcohols, n=3, m=9		FC(F)(F)C(F)(F)C(F)(F)C(F)(F)C(F)(F)C(F)(F)CCOCCOCCOCCOCCOCCOCCOCCOCCO[H]		-8.28	0.58	4.00	P	OUT
QL02844	multiple H-substituted, multiple ether-substituted perfluoroalkyl alcohols, n=3, m=10		FC(F)(F)C(F)(F)C(F)(F)C(F)(F)C(F)(F)C(F)(F)CCOCCOCCOCCOCCOCCOCCOCCOCCOCCO[H]		-8.43	0.57	4.15	P	OUT
QL02845	multiple H-substituted, multiple ether-substituted perfluoroalkyl alcohols, n=3, m=11		FC(F)(F)C(F)(F)C(F)(F)C(F)(F)C(F)(F)C(F)(F)CCOCCOCCOCCOCCOCCOCCOCCOCCOCCOCCO[H]		-8.50	0.57	4.24	P	OUT
QL02846	multiple H-substituted, multiple ether-substituted perfluoroalkyl alcohols, n=3, m=12		FC(F)(F)C(F)(F)C(F)(F)C(F)(F)C(F)(F)C(F)(F)CCOCCOCCOCCOCCOCCOCCOCCOCCOCCOCCOCCO[H]		-8.64	0.57	4.36	P	OUT
QL02847	multiple H-substituted, multiple ether-substituted perfluoroalkyl alcohols, n=3, m=13		FC(F)(F)C(F)(F)C(F)(F)C(F)(F)C(F)(F)C(F)(F)CCOCCOCCOCCOCCOCCOCCOCCOCCOCCOCCOCCOCCO[H]		-8.70	0.56	4.44	P	OUT
QL02848	multiple H-substituted, multiple ether-substituted perfluoroalkyl alcohols, n=3, m=14		FC(F)(F)C(F)(F)C(F)(F)C(F)(F)C(F)(F)C(F)(F)CCOCCOCCOCCOCCOCCOCCOCCOCCOCCOCCOCCOCCOCCO[H]		-8.84	0.56	4.55	P	OUT
QL02849	multiple H-substituted, multiple ether-substituted perfluoroalkyl alcohols, n=3, m=15		FC(F)(F)C(F)(F)C(F)(F)C(F)(F)C(F)(F)C(F)(F)CCOCCOCCOCCOCCOCCOCCOCCOCCOCCOCCOCCOCCOCCOCCO[H]		-8.90	0.56	4.63	P	OUT
QL02850	multiple H-substituted, multiple ether-substituted perfluoroalkyl alcohols, n=3, m=16		FC(F)(F)C(F)(F)C(F)(F)C(F)(F)C(F)(F)C(F)(F)CCOCCOCCOCCOCCOCCOCCOCCOCCOCCOCCOCCOCCOCCOCCOCCO[H]		-9.02	0.55	4.72	P	OUT
QL02851	multiple H-substituted, multiple ether-substituted perfluoroalkyl alcohols, n=3, m=17		FC(F)(F)C(F)(F)C(F)(F)C(F)(F)C(F)(F)C(F)(F)CCOCCOCCOCCOCCOCCOCCOCCOCCOCCOCCOCCOCCOCCOCCOCCOCCO[H]		-9.08	0.55	4.79	P	OUT
QL02852	multiple H-substituted, multiple ether-substituted perfluoroalkyl alcohols, n=3, m=18		FC(F)(F)C(F)(F)C(F)(F)C(F)(F)C(F)(F)C(F)(F)CCOCCOCCOCCOCCOCCOCCOCCOCCOCCOCCOCCOCCOCCOCCOCCOCCOCCO[H]		-9.20	0.55	4.87	P	OUT
QL02853	multiple H-substituted, multiple ether-substituted perfluoroalkyl alcohols, n=3, m=19		FC(F)(F)C(F)(F)C(F)(F)C(F)(F)C(F)(F)C(F)(F)CCOCCOCCOCCOCCOCCOCCOCCOCCOCCOCCOCCOCCOCCOCCOCCOCCOCCOCCO[H]		-9.26	0.55	4.93	P	OUT
QL02854	multiple H-substituted, multiple ether-substituted perfluoroalkyl alcohols, n=3, m=20		FC(F)(F)C(F)(F)C(F)(F)C(F)(F)C(F)(F)C(F)(F)CCOCCOCCOCCOCCOCCOCCOCCOCCOCCOCCOCCOCCOCCOCCOCCOCCOCCOCCOCCO[H]		-9.37	0.55	5.01	P	OUT
QL02855	Tris(3,3,4,4,5,5,6,6,7,7,8,8,8-tridecafluorooctyl) phosphate	165325-62-2	FC(F)(F)C(F)(F)C(F)(F)C(F)(F)C(F)(F)C(F)(F)CCOP(=O)(OCCC(F)(F)C(F)(F)C(F)(F)C(F)(F)C(F)(F)C(F)(F)F)OCCC(F)(F)C(F)(F)C(F)(F)C(F)(F)C(F)(F)C(F)(F)F		-9.48	0.68	3.24	P	OUT
QL02856	2-(Perfluorohexyl)ethanethiol	34451-26-8	FC(F)(F)C(F)(F)C(F)(F)C(F)(F)C(F)(F)C(F)(F)CCS		-7.16	0.66	2.23	P	IN
QL02857	8-(2-Chloroethanesulfonyl)-1,1,1,2,2,3,3,4,4,5,5,6,6-tridecafluorooctane	93393-77-2	FC(F)(F)C(F)(F)C(F)(F)C(F)(F)C(F)(F)C(F)(F)CCS(=O)(=O)CCCl		-7.92	0.66	2.70	P	IN
QL02858	n2 fluorotelomer sulfonyl propano-amido alkyl sulfonic acids, n=3		FC(F)(F)C(F)(F)C(F)(F)C(F)(F)C(F)(F)C(F)(F)CCS(CCC(NC(C)(C)CS(O)(=O)=O)=O)(=O)=O		-8.68	0.66	3.08	P	IN
QL02859	n2 fluorotelomer thioamido sulfonic acids, n=3		FC(F)(F)C(F)(F)C(F)(F)C(F)(F)C(F)(F)C(F)(F)CCS(CCC(NC(C)(C)CS(O)(=O)=O)=O)=O		-8.48	0.65	3.08	P	IN
QL02860	n2 fluorotelomer sulfonyl propanoic acids, n=3		FC(F)(F)C(F)(F)C(F)(F)C(F)(F)C(F)(F)C(F)(F)CCS(CCC(O)=O)(=O)=O		-8.06	0.66	2.75	P	IN
QL02861	1-Octanesulfonyl chloride, 3,3,4,4,5,5,6,6,7,7,8,8,8-tridecafluoro-	27619-89-2	FC(F)(F)C(F)(F)C(F)(F)C(F)(F)C(F)(F)C(F)(F)CCS(Cl)(=O)=O		-7.90	0.67	2.44	P	IN
QL02862	n2 fluorotelomer sulfonamido betaine-related, n=3		FC(F)(F)C(F)(F)C(F)(F)C(F)(F)C(F)(F)C(F)(F)CCS(NCCC[N+](CC(O)=O)(C)C)(=O)=O		-8.47	0.65	3.10	P	OUT
QL02863	n2 fluorotelomer thio alkyl amido carboxylic acids, n=3		FC(F)(F)C(F)(F)C(F)(F)C(F)(F)C(F)(F)C(F)(F)CCSC(C(O)=O)CC(NCCCN(C)C)=O		-8.19	0.63	3.23	P	OUT
QL02864	n2 fluorotelomer thio alkyl amido ammonium_i, n=3		FC(F)(F)C(F)(F)C(F)(F)C(F)(F)C(F)(F)C(F)(F)CCSCC(NCCC[N+](C)(C)C)=O		-8.18	0.64	3.07	P	OUT
QL02865	n2 fluorotelomer thio alkyl amido betaine-related, n=3		FC(F)(F)C(F)(F)C(F)(F)C(F)(F)C(F)(F)C(F)(F)CCSCC(NCCC[N+](C)(C)CC(O)=O)=O		-8.31	0.63	3.23	P	OUT
QL02866	n2 fluorotelomer thio alkyl amido ammonium, n=3		FC(F)(F)C(F)(F)C(F)(F)C(F)(F)C(F)(F)C(F)(F)CCSCC(NCCCN(C)C)=O		-7.96	0.63	3.09	P	OUT
QL02867	n2 fluorotelomer thio alkyl amido sulfonic acids, n=3		FC(F)(F)C(F)(F)C(F)(F)C(F)(F)C(F)(F)C(F)(F)CCSCCC(NC(C)(C)CS(O)(=O)=O)=O		-8.35	0.65	3.05	P	OUT

QL02868	4-[(3,3,4,4,5,5,6,6,7,7,8,8,8-Tridecafluorooctyl)sulfanyl]butane-1-thiol	36097-07-1	FC(F)(F)C(F)(F)C(F)(F)C(F)(F)C(F)(F)C(F)(F)CCSCCCCS		-7.64	0.63	2.90	P	OUT
QL02869	Bis[3-(3,3,4,4,5,5,6,6,7,7,8,8,8-tridecafluorooctylsulfanyl)propyl] But-2-enedioate	56927-83-4	FC(F)(F)C(F)(F)C(F)(F)C(F)(F)C(F)(F)C(F)(F)CCSCCCOC(=O)C=CC(=O)OCCCSCCC(F)(F)C(F)(F)C(F)(F)C(F)(F)C(F)(F)C(F)(F)F		-9.57	0.64	3.81	P	OUT
QL02870	1H,1H-Tridecafluoro-1-iodoheptane	212563-43-4	FC(F)(F)C(F)(F)C(F)(F)C(F)(F)C(F)(F)C(F)(F)CI		-7.35	0.68	2.15	P	IN
QL02871	Perfluorohexylchloride	355-41-9	FC(F)(F)C(F)(F)C(F)(F)C(F)(F)C(F)(F)C(F)(F)Cl		-7.19	0.69	1.90	P	IN
QL02872	1,1-Dihydroperfluoroheptyl acrylate	559-11-5	FC(F)(F)C(F)(F)C(F)(F)C(F)(F)C(F)(F)C(F)(F)COC(=O)C=C		-7.55	0.66	2.52	P	IN
QL02873	multiple H-substituted-ether-substituted-perfluoroalkyl (linear) carboxylic acids, n=6		FC(F)(F)C(F)(F)C(F)(F)C(F)(F)C(F)(F)C(F)(F)COCC(O)=O		-7.57	0.66	2.52	P	IN
QL02874	Perfluorotrihexylamine	432-08-6	FC(F)(F)C(F)(F)C(F)(F)C(F)(F)C(F)(F)C(F)(F)N(C(F)(F)C(F)(F)C(F)(F)C(F)(F)C(F)(F)C(F)(F)F)C(F)(F)C(F)(F)C(F)(F)C(F)(F)C(F)(F)C(F)(F)F		-9.13	0.69	2.82	P	OUT
QL02875	1-Chloro-4-(1,1,2,2,3,3,4,4,5,5,6,6,6-tridecafluorohexane-1-sulfonyl)benzene	76848-59-4	FC(F)(F)C(F)(F)C(F)(F)C(F)(F)C(F)(F)C(F)(F)S(=O)(=O)c1ccc(Cl)cc1		-8.17	0.67	2.69	P	IN
QL02876	1-(Perfluorohexyl)sulfonylaminopyridinium	254889-10-6	FC(F)(F)C(F)(F)C(F)(F)C(F)(F)C(F)(F)C(F)(F)S(=O)(=O)N[N+]1=CC=CC=C1		-7.95	0.66	2.69	P	IN
QL02877	Hexane, 1-(ethenylsulfinyl)-1,1,2,2,3,3,4,4,5,5,6,6,6-tridecafluoro-	680187-85-3	FC(F)(F)C(F)(F)C(F)(F)C(F)(F)C(F)(F)C(F)(F)S(=O)C=C		-7.32	0.67	2.24	P	IN
QL02878	Hexane, 1-(pentafluorothio)-tridecafluoro-	1423-20-7	FC(F)(F)C(F)(F)C(F)(F)C(F)(F)C(F)(F)C(F)(F)S(F)(F)(F)(F)F		-8.24	0.71	2.12	P	IN
QL02879	Perfluoroalkyl (linear) sulfinate, n=6		FC(F)(F)C(F)(F)C(F)(F)C(F)(F)C(F)(F)C(F)(F)S(O)=O		-7.50	0.68	2.16	P	IN
QL02880	[(Tridecafluorohexyl)sulfanyl]benzene	69127-72-6	FC(F)(F)C(F)(F)C(F)(F)C(F)(F)C(F)(F)C(F)(F)SC1=CC=CC=C1		-7.47	0.65	2.52	P	IN
QL02881	Tridecafluorohexanesulphenyl chloride	89987-98-4	FC(F)(F)C(F)(F)C(F)(F)C(F)(F)C(F)(F)C(F)(F)SCl		-7.35	0.68	2.15	P	IN
QL02882	Bis(tridecafluorohexyl) disulphide	93962-52-8	FC(F)(F)C(F)(F)C(F)(F)C(F)(F)C(F)(F)C(F)(F)SSC(F)(F)C(F)(F)C(F)(F)C(F)(F)C(F)(F)C(F)(F)F		-9.13	0.69	2.90	P	OUT
QL02883	Pentadecafluoro-2-iodoheptane	24394-25-0	FC(F)(F)C(F)(F)C(F)(F)C(F)(F)C(F)(F)C(F)(I)C(F)(F)F		-7.74	0.69	2.15	P	IN
QL02884	Undecafluorohexanenitrile	23790-49-0	FC(F)(F)C(F)(F)C(F)(F)C(F)(F)C(F)(F)C#N		-6.72	0.67	1.89	P	IN
QL02885	3,3,4,4,5,5,6,6,7,7,7-Undecafluorohept-1-ene	84100-13-0	FC(F)(F)C(F)(F)C(F)(F)C(F)(F)C(F)(F)C=C		-6.72	0.67	1.89	P	IN
QL02886	2,2,3,3,4,4,5,5,6,6,6-Undecafluorohexanal	754-79-0	FC(F)(F)C(F)(F)C(F)(F)C(F)(F)C(F)(F)C=O		-6.72	0.67	1.89	P	IN
QL02887	5-(Undecafluoropentyl)-1H-imidazole	113934-18-2	FC(F)(F)C(F)(F)C(F)(F)C(F)(F)C(F)(F)C1=CN=CN1		-6.96	0.66	2.17	P	IN
QL02888	2,4,6-Tris(undecafluoropentyl)-1,3,5-triazine	23790-50-3	FC(F)(F)C(F)(F)C(F)(F)C(F)(F)C(F)(F)c1nc(nc(n1)C(F)(F)C(F)(F)C(F)(F)C(F)(F)C(F)(F)F)C(F)(F)C(F)(F)C(F)(F)C(F)(F)C(F)(F)F		-8.76	0.68	2.74	P	IN
QL02889	1 OH-substituted n2 fluorotelomer sulfonic acids, n=5		FC(F)(F)C(F)(F)C(F)(F)C(F)(F)C(F)(F)CC(O)S(=O)(O)=O		-7.49	0.67	2.24	P	IN
QL02890	1,1,1,2,2,3,3,4,4,5,5,8,8,9,9,10,10,11,11,12,12,12-Docosafluorododecane	1835255-38-3	FC(F)(F)C(F)(F)C(F)(F)C(F)(F)C(F)(F)CCC(F)(F)C(F)(F)C(F)(F)C(F)(F)C(F)(F)F		-8.67	0.69	2.69	P	IN
QL02891	4,4,5,5,6,6,7,7,8,8,8-Undecafluorooctanal	195047-10-0	FC(F)(F)C(F)(F)C(F)(F)C(F)(F)C(F)(F)CCC=O		-6.81	0.65	2.25	P	IN
QL02892	1,1,1,2,2,3,3,4,4,5,5,12,12,13,13,14,14,15,15,16,16,16-Docosafluorohexadecane	1835255-25-8	FC(F)(F)C(F)(F)C(F)(F)C(F)(F)C(F)(F)CCCCCCC(F)(F)C(F)(F)C(F)(F)C(F)(F)C(F)(F)F		-8.73	0.67	3.00	P	IN
QL02893	1,1,1,2,2,3,3,4,4,5,5,14,14,15,15,16,16,17,17,18,18,18-Docosafluorooctadecane	1835255-24-7	FC(F)(F)C(F)(F)C(F)(F)C(F)(F)C(F)(F)CCCCCCCCC(F)(F)C(F)(F)C(F)(F)C(F)(F)C(F)(F)F		-8.77	0.66	3.13	P	IN
QL02894	1,1,1,2,2,3,3,4,4,5,5,16,16,17,17,18,18,19,19,20,20,20-Docosafluoroicosane	1835252-07-7	FC(F)(F)C(F)(F)C(F)(F)C(F)(F)C(F)(F)CCCCCCCCCCC(F)(F)C(F)(F)C(F)(F)C(F)(F)C(F)(F)F		-8.81	0.65	3.26	P	OUT
QL02895	1,1,1,2,2,3,3,4,4,5,5,19,19,20,20,21,21,22,22,23,23,23-Docosafluorotricosane	1980076-48-9	FC(F)(F)C(F)(F)C(F)(F)C(F)(F)C(F)(F)CCCCCCCCCCCCCC(F)(F)C(F)(F)C(F)(F)C(F)(F)C(F)(F)F		-8.93	0.64	3.46	P	OUT
QL02896	n2 perfluoroalkyl acrylates, n=5		FC(F)(F)C(F)(F)C(F)(F)C(F)(F)C(F)(F)CCOC(CC(C)=C)=O		-7.47	0.64	2.68	P	IN
QL02897	1-Iodo-1H,1H-perfluorohexane	335-50-2	FC(F)(F)C(F)(F)C(F)(F)C(F)(F)C(F)(F)CI		-6.72	0.67	1.89	P	IN
QL02898	2,2,3,3,4,4,5,5,6,6,6-Undecafluorohexyl prop-2-enoate	376-56-7	FC(F)(F)C(F)(F)C(F)(F)C(F)(F)C(F)(F)COC(=O)C=C		-7.30	0.65	2.48	P	IN
QL02899	6-(Ethenyloxy)-1,1,1,2,2,3,3,4,4,5,5-undecafluorohexane	595-00-6	FC(F)(F)C(F)(F)C(F)(F)C(F)(F)C(F)(F)COC=C		-6.81	0.65	2.25	P	IN
QL02900	1,1,1,2,2,3,3,4,4,5,5-Undecafluoro-6-[(prop-2-en-1-yl)oxy]hexane	1516879-68-7	FC(F)(F)C(F)(F)C(F)(F)C(F)(F)C(F)(F)COCC=C		-7.08	0.64	2.49	P	IN
QL02901	2-{[(2,2,3,3,4,4,5,5,6,6,6-Undecafluorohexyl)oxy]methyl}oxirane	140369-64-8	FC(F)(F)C(F)(F)C(F)(F)C(F)(F)C(F)(F)COCC1CO1		-7.47	0.66	2.48	P	IN
QL02902	2,2,3,3,4,4,5,5,6,6,6-Undecafluorohexyl nonafluorobutane-1-sulfonate	924894-08-6	FC(F)(F)C(F)(F)C(F)(F)C(F)(F)C(F)(F)COS(=O)(=O)C(F)(F)C(F)(F)C(F)(F)C(F)(F)F		-8.67	0.69	2.69	P	IN
QL02903	2,2,3,3,4,4,5,5,6,6,6-Undecafluorohexyl trifluoromethanesulfonate	168632-11-9	FC(F)(F)C(F)(F)C(F)(F)C(F)(F)C(F)(F)COS(=O)(=O)C(F)(F)F		-8.04	0.68	2.44	P	IN
QL02904	n1 fluorotelomer sulfonic acids, n=5		FC(F)(F)C(F)(F)C(F)(F)C(F)(F)C(F)(F)CS(=O)(O)=O		-7.35	0.68	2.15	P	IN
QL02905	Perflenapent	678-26-2	FC(F)(F)C(F)(F)C(F)(F)C(F)(F)C(F)(F)F		-6.93	0.69	1.79	P	IN
QL02906	2,2,3,3,4,4,5,5-Octafluoro-1-(undecafluoropentyl)pyrrolidine	94718-24-8	FC(F)(F)C(F)(F)C(F)(F)C(F)(F)C(F)(F)N1C(F)(F)C(F)(F)C(F)(F)C1(F)F		-7.91	0.69	2.19	P	IN
QL02907	ether-substituted perfluoroalkyl (linear) caborxylic acids_i, n=5		FC(F)(F)C(F)(F)C(F)(F)C(F)(F)C(F)(F)OC(F)(C(F)(F)F)C(O)=O		-7.48	0.68	2.20	P	IN
QL02908	1,1,1,2,2,3,3,4,4,5,5-Undecafluoro-5-[1,1,2,3,3,3-hexafluoro-2-(trifluoromethyl)propoxy]pentane	169477-62-7	FC(F)(F)C(F)(F)C(F)(F)C(F)(F)C(F)(F)OC(F)(F)C(F)(C(F)(F)F)C(F)(F)F		-8.06	0.68	2.39	P	IN

QL02909	1,1,2,2,3,3,4,4,5,5,5-undecafluoro-N-(2-hydroxyethyl)pentane-1-sulfonamide		FC(F)(F)C(F)(F)C(F)(F)C(F)(F)C(F)(F)S(=O)(NCCO)=O	-7.41	0.66	2.46	P	IN
QL02910	Perfluoroalkyl (linear) sulfinate, n=5		FC(F)(F)C(F)(F)C(F)(F)C(F)(F)C(F)(F)S(O)=O	-6.91	0.68	1.90	P	IN
QL02911	1,1,1,2,2,3,3,4,4,5,6,6,6-Tridecafluoro-5-iodohexane	355-44-2	FC(F)(F)C(F)(F)C(F)(F)C(F)(F)C(F)(I)C(F)(F)F	-7.19	0.69	1.90	P	IN
QL02912	2,2,3,3,4,4,5,5,5-Nonafluoropentane-1,1-diol	355-30-6	FC(F)(F)C(F)(F)C(F)(F)C(F)(F)C(O)O	-6.61	0.67	1.81	P	IN
QL02913	(3,3,4,4,5,5,6,6,6-Nonafluorohex-1-yn-1-yl)benzene	66249-23-8	FC(F)(F)C(F)(F)C(F)(F)C(F)(F)C#Cc1ccccc1	-6.80	0.64	2.43	P	OUT
QL02914	2,2,3,3,4,4,5,5,5-nonafluoropentanenitrile	22325-71-9	FC(F)(F)C(F)(F)C(F)(F)C(F)(F)C#N	-6.40	0.67	1.81	P	IN
QL02915	1H,1H,2H-Perfluoro-1-hexene	19430-93-4	FC(F)(F)C(F)(F)C(F)(F)C(F)(F)C=C	-6.40	0.67	1.81	P	IN
QL02916	(3,3,4,4,5,5,6,6,6-Nonafluoro-1-iodohex-1-en-1-yl)benzene	109574-90-5	FC(F)(F)C(F)(F)C(F)(F)C(F)(F)C=C(I)C1=CC=CC=C1	-6.84	0.64	2.42	P	IN
QL02917	[(1Z)-3,3,4,4,5,5,6,6,6-Nonafluorohex-1-en-1-yl]benzene	95452-53-2	FC(F)(F)C(F)(F)C(F)(F)C(F)(F)C=C/c1ccccc1	-6.80	0.64	2.43	P	OUT
QL02918	4,4,5,5,6,6,7,7,7-Nonafluorohepta-1,2-diene	132673-98-4	FC(F)(F)C(F)(F)C(F)(F)C(F)(F)C=C=C	-6.13	0.65	1.91	P	IN
QL02919	(5E)-1,1,1,2,2,3,3,4,4,7,7,8,8,9,9,10,10,11,11,12,12,13,13,14,14,14-Hexacosafluorotetradec-5-ene	51249-69-5	FC(F)(F)C(F)(F)C(F)(F)C(F)(F)C=CC(F)(F)C(F)(F)C(F)(F)C(F)(F)C(F)(F)C(F)(F)C(F)(F)C(F)(F)F	-9.21	0.69	2.93	P	OUT
QL02920	(5E)-1,1,1,2,2,3,3,4,4,7,7,8,8,9,9,10,10,11,11,12,12,12-Docosafluorododec-5-ene	51249-66-2	FC(F)(F)C(F)(F)C(F)(F)C(F)(F)C=CC(F)(F)C(F)(F)C(F)(F)C(F)(F)C(F)(F)C(F)(F)F	-8.75	0.69	2.73	P	IN
QL02921	1,1,1,2,2,3,3,4,4,7,7,8,8,9,9,10,10,11,11,11-Icosafluoroundec-5-ene	100508-28-9	FC(F)(F)C(F)(F)C(F)(F)C(F)(F)C=CC(F)(F)C(F)(F)C(F)(F)C(F)(F)C(F)(F)F	-8.51	0.68	2.63	P	IN
QL02922	1,1,1,2,2,3,3,4,4,7,7,8,8,9,9,10,10,10-Octadecafluorodec-5-ene	84551-43-9	FC(F)(F)C(F)(F)C(F)(F)C(F)(F)C=CC(F)(F)C(F)(F)C(F)(F)C(F)(F)F	-8.11	0.68	2.45	P	IN
QL02923	Perfluorovaleraldehyde	375-53-1	FC(F)(F)C(F)(F)C(F)(F)C(F)(F)C=O	-6.40	0.67	1.81	P	IN
QL02924	Perfluorobutylcyclohexane	374-60-7	FC(F)(F)C(F)(F)C(F)(F)C(F)(F)C1(F)C(F)(F)C(F)(F)C(F)(F)C(F)(F)C1(F)F	-8.02	0.70	2.19	P	IN
QL02925	Perfluorodecahydro-1-(perfluorobutyl)naphthalene	118914-93-5	FC(F)(F)C(F)(F)C(F)(F)C(F)(F)C1(F)C(F)(F)C(F)(F)C(F)(F)C2(F)C(F)(F)C(F)(F)C(F)(F)C(F)(F)C12F	-8.40	0.70	2.34	P	IN
QL02926	Perfluorodecahydro-2-n-butylnaphthalene	119107-96-9	FC(F)(F)C(F)(F)C(F)(F)C(F)(F)C1(F)C(F)(F)C(F)(F)C2(F)C(F)(F)C(F)(F)C(F)(F)C(F)(F)C2(F)C1(F)F	-8.74	0.70	2.51	P	IN
QL02927	Furan, 2,2,3,3,4,5,5-heptafluorotetrahydro-4-(nonafluorobutyl)-	646-85-5	FC(F)(F)C(F)(F)C(F)(F)C(F)(F)C1(F)C(F)(F)OC(F)(F)C1(F)F	-7.67	0.69	2.10	P	IN
QL02928	3,4,4-Trifluoro-3-(nonafluorobutyl)-1,2lambda~6~-oxathietane-2,2-dione	85211-95-6	FC(F)(F)C(F)(F)C(F)(F)C(F)(F)C1(F)C(F)(F)OS1(=O)=O	-7.19	0.69	1.87	P	IN
QL02929	2,2,3-Trifluoro-3-(nonafluorobutyl)oxirane	72804-48-9	FC(F)(F)C(F)(F)C(F)(F)C(F)(F)C1(F)OC1(F)F	-7.27	0.69	1.90	P	IN
QL02930	2-(Nonafluorobutyl)-4H-1-benzopyran-4-one	304877-96-1	FC(F)(F)C(F)(F)C(F)(F)C(F)(F)C1=CC(=O)c2ccccc2O1	-7.05	0.64	2.44	P	IN
QL02931	6-(Nonafluorobutyl)-2-phenylpyrimidin-4(3H)-one	180748-44-1	FC(F)(F)C(F)(F)C(F)(F)C(F)(F)C1=CC(=O)NC(=N1)c1ccccc1	-6.91	0.63	2.52	P	OUT
QL02932	6-(Nonafluorobutyl)-2-sulfanylidene-2,3-dihydropyrimidin-4(1H)-one	1426894-99-6	FC(F)(F)C(F)(F)C(F)(F)C(F)(F)C1=CC(=O)NC(=S)N1	-6.74	0.65	2.17	P	IN
QL02933	9-(Nonafluorobutyl)-2,3,6,7-tetrahydro-1H,5H,11H-pyrano[2,3-f]pyrido[3,2,1-ij]quinolin-11-one	355822-08-1	FC(F)(F)C(F)(F)C(F)(F)C(F)(F)C1=CC(=O)Oc2c3CCCN4CCCc(cc12)c34	-6.99	0.64	2.51	P	IN
QL02934	Benzene, (nonafluorobutyl)-	2398-75-6	FC(F)(F)C(F)(F)C(F)(F)C(F)(F)C1=CC=CC=C1	-6.58	0.65	2.18	P	IN
QL02935	2-(Nonafluorobutyl)-1H-indene	1608112-37-3	FC(F)(F)C(F)(F)C(F)(F)C(F)(F)C1=Cc2ccccc2C1	-6.78	0.65	2.26	P	IN
QL02936	SCHEMBL1270621	58671-28-6	FC(F)(F)C(F)(F)C(F)(F)C(F)(F)C1=CNC(=O)NC1=O	-6.90	0.65	2.24	P	IN
QL02937	4-(Nonafluorobutyl)-1,3-dihydro-2H-1,5-benzodiazepin-2-one	96146-12-2	FC(F)(F)C(F)(F)C(F)(F)C(F)(F)C1=Nc2ccccc2NC(=O)C1	-6.86	0.64	2.42	P	IN
QL02938	2-(Nonafluorobutyl)-1-benzofuran	1608112-36-2	FC(F)(F)C(F)(F)C(F)(F)C(F)(F)c1cc2ccccc2o1	-6.78	0.65	2.26	P	IN
QL02939	2-(Nonafluorobutyl)-1-benzothiophene	1608112-38-4	FC(F)(F)C(F)(F)C(F)(F)C(F)(F)c1cc2ccccc2s1	-6.78	0.65	2.26	P	IN
QL02940	4-(Nonafluorobutyl)benzene-1,2-dicarbonitrile	1207299-71-5	FC(F)(F)C(F)(F)C(F)(F)C(F)(F)c1ccc(C#N)c(c1)C#N	-6.79	0.63	2.45	P	OUT
QL02941	1-Ethenyl-4-(nonafluorobutyl)benzene	159241-92-6	FC(F)(F)C(F)(F)C(F)(F)C(F)(F)c1ccc(C=C)cc1	-7.03	0.64	2.47	P	IN
QL02942	4-(Nonafluorobutyl)benzonitrile	137052-16-5	FC(F)(F)C(F)(F)C(F)(F)C(F)(F)c1ccc(cc1)C#N	-7.03	0.64	2.47	P	IN
QL02943	7-(Nonafluorobutyl)-1-benzothiophene	1608112-40-8	FC(F)(F)C(F)(F)C(F)(F)C(F)(F)c1cccc2ccsc12	-6.54	0.65	2.16	P	IN
QL02944	Tetrahydro(perfluorobutyl)furan	26446-59-3	FC(F)(F)C(F)(F)C(F)(F)C(F)(F)C1CCCO1	-6.26	0.65	1.91	P	IN
QL02945	2-(Nonafluorobutyl)thiophene	96721-81-2	FC(F)(F)C(F)(F)C(F)(F)C(F)(F)c1cccs1	-6.26	0.65	1.91	P	IN
QL02946	3-(Nonafluorobutyl)-1H-indole	1608112-33-9	FC(F)(F)C(F)(F)C(F)(F)C(F)(F)c1cNc2ccccc12	-6.71	0.65	2.24	P	IN
QL02947	Nonafluorobutylepoxide	89807-87-4	FC(F)(F)C(F)(F)C(F)(F)C(F)(F)C1CO1	-6.59	0.67	1.81	P	IN
QL02948	4-(Nonafluorobutyl)-1,3-dioxolan-2-one	1469980-27-5	FC(F)(F)C(F)(F)C(F)(F)C(F)(F)C1COC(=O)O1	-6.92	0.66	2.18	P	IN

QL02949	3-(Nonafluorobutyl)-1-benzothiophene	1608112-39-5	FC(F)(F)C(F)(F)C(F)(F)C(F)(F)c1csc2ccccc12		-6.71	0.65	2.24	P	IN
QL02950	2-(Nonafluorobutyl)-1H-indole	1608112-34-0	FC(F)(F)C(F)(F)C(F)(F)C(F)(F)c1Nc2ccccc2c1		-6.78	0.65	2.26	P	IN
QL02951	3-(Nonafluorobutyl)-5-phenyl-1H-pyrazole	204130-95-0	FC(F)(F)C(F)(F)C(F)(F)C(F)(F)c1nNc(c1):c1ccccc1		-7.06	0.64	2.55	P	OUT
QL02952	11-(Nonafluorobutyl)-9,10-dihydro-9,10-(epithiomethano)anthracene	267648-90-8	FC(F)(F)C(F)(F)C(F)(F)C(F)(F)C1SC2c3ccccc3C1c1ccccc21		-6.77	0.64	2.40	P	IN
QL02953	3,3,4,4,5,5,6,6,6-Nonafluorohexanoyl chloride	64018-24-2	FC(F)(F)C(F)(F)C(F)(F)C(F)(F)CC(Cl)=O		-6.37	0.66	1.90	P	IN
QL02954	Thiols, C10-20, .gamma.-.omega.-perfluoro (Perfluoroalkyl(C8-18)ethyl mercaptan)	68140-21-6	FC(F)(F)C(F)(F)C(F)(F)C(F)(F)CC(F)(F)C(F)(F)C(F)(F)CCS		-7.70	0.66	2.51	P	IN
QL02955	1,1,1,2,2,3,3,4,4,6,6,8,8-Tridecafluoro-10-iodododecane	272451-64-6	FC(F)(F)C(F)(F)C(F)(F)C(F)(F)CC(F)(F)CC(F)(F)CCI		-7.31	0.65	2.52	P	IN
QL02956	1,1,1,2,2,3,3,4,4,6,6,8,8-Tridecafluoro-8-iodooctane	99325-17-4	FC(F)(F)C(F)(F)C(F)(F)C(F)(F)CC(F)(F)CC(F)(F)I		-7.26	0.67	2.23	P	IN
QL02957	1,1,1,2,2,3,3,4,4,6,6-Undecafluoro-8-iodooctane	53626-95-2	FC(F)(F)C(F)(F)C(F)(F)C(F)(F)CC(F)(F)CCI		-6.79	0.65	2.25	P	IN
QL02958	4-(Perfluorobutyl)-3,3-difluorobutanesulfonyl chloride	1225375-54-1	FC(F)(F)C(F)(F)C(F)(F)C(F)(F)CC(F)(F)CCS(Cl)(=O)=O		-7.48	0.66	2.39	P	IN
QL02959	1-(3,3,4,4,5,5,6,6,6-Nonafluoro-1-iodohexyl)-2,8,9-trioxa-5-aza-1-silabicyclo[3.3.3]undecane	135587-13-2	FC(F)(F)C(F)(F)C(F)(F)C(F)(F)CC(I)[Si]12OCCN(CCO1)CCO2		-6.93	0.64	2.49	P	IN
QL02960	1,1,1,2,2,3,3,4,4,5,5,6,6,7,7,8,8,11,11,12,12,13,13,14,14,14-Hexacosafluoro-9-iodotetradecane	53638-13-4	FC(F)(F)C(F)(F)C(F)(F)C(F)(F)CC(I)C(F)(F)C(F)(F)C(F)(F)C(F)(F)C(F)(F)C(F)(F)C(F)(F)C(F)(F)F		-9.21	0.69	2.94	P	OUT
QL02961	1-Iodo-1,2-bis(perfluoro-n-butyl)ethane	53638-10-1	FC(F)(F)C(F)(F)C(F)(F)C(F)(F)CC(I)C(F)(F)C(F)(F)C(F)(F)C(F)(F)F		-8.18	0.68	2.49	P	IN
QL02962	(3,3,4,4,5,5,6,6,6-Nonafluoro-1-iodohexyl)cyclohexane	1464148-71-7	FC(F)(F)C(F)(F)C(F)(F)C(F)(F)CC(I)C1CCCCC1		-6.84	0.64	2.42	P	IN
QL02963	1,1,1,2,2,3,3,4,4,10,10,11,11,12,12,13,13,13-Octadecafluoro-6,8-diiodotridecane	40735-21-5	FC(F)(F)C(F)(F)C(F)(F)C(F)(F)CC(I)CC(I)CC(F)(F)C(F)(F)C(F)(F)C(F)(F)F		-8.37	0.67	2.80	P	IN
QL02964	1,1,1,2,2,3,3,4,4,11,11,12,12,13,13,14,14,14-Octadecafluoro-6,9-diiodotetradecane	40735-23-7	FC(F)(F)C(F)(F)C(F)(F)C(F)(F)CC(I)CCC(I)CC(F)(F)C(F)(F)C(F)(F)C(F)(F)F		-8.36	0.66	2.85	P	IN
QL02965	1,1,1,2,2,3,3,4,4,12,12,13,13,14,14,15,15,15-Octadecafluoro-6,10-diiodopentadecane	40735-25-9	FC(F)(F)C(F)(F)C(F)(F)C(F)(F)CC(I)CCCC(I)CC(F)(F)C(F)(F)C(F)(F)C(F)(F)F		-8.44	0.66	2.96	P	IN
QL02966	1,1,1,2,2,3,3,4,4,13,13,14,14,15,15,16,16,16-Octadecafluoro-6,11-diiodohexadecane	13105-46-9	FC(F)(F)C(F)(F)C(F)(F)C(F)(F)CC(I)CCCCC(I)CC(F)(F)C(F)(F)C(F)(F)C(F)(F)F		-8.41	0.65	3.00	P	IN
QL02967	1 OH-substituted n2 fluorotelomer sulfonic acids, n=4		FC(F)(F)C(F)(F)C(F)(F)C(F)(F)CC(O)S(=O)(O)=O		-7.25	0.67	2.17	P	IN
QL02968	Dichloromethyl(3,3,4,4,5,5,6,6,6-nonafluorohexyl)silane	38436-16-7	FC(F)(F)C(F)(F)C(F)(F)C(F)(F)CC[SiH2]C(Cl)Cl		-6.70	0.64	2.27	P	IN
QL02969	Bromotris(3,3,4,4,5,5,6,6,6-nonafluorohexyl)stannane	240497-37-4	FC(F)(F)C(F)(F)C(F)(F)C(F)(F)CC[Sn](Br)(CCC(F)(F)C(F)(F)C(F)(F)C(F)(F)F)CCC(F)(F)C(F)(F)C(F)(F)C(F)(F)F		-8.57	0.68	2.75	P	IN
QL02970	Tris(3,3,4,4,5,5,6,6,6-nonafluorohexyl)(phenyl)stannane	240497-36-3	FC(F)(F)C(F)(F)C(F)(F)C(F)(F)CC[Sn](CCC(F)(F)C(F)(F)C(F)(F)C(F)(F)F)(CCC(F)(F)C(F)(F)C(F)(F)C(F)(F)F)c1ccccc1		-8.33	0.66	2.82	P	IN
QL02971	Tris(3,3,4,4,5,5,6,6,6-nonafluorohexyl)(prop-2-en-1-yl)stannane	215186-99-5	FC(F)(F)C(F)(F)C(F)(F)C(F)(F)CC[Sn](CCC(F)(F)C(F)(F)C(F)(F)C(F)(F)F)(CCC(F)(F)C(F)(F)C(F)(F)C(F)(F)F)CC=C		-8.35	0.67	2.80	P	IN
QL02972	Tris(3,3,4,4,5,5,6,6,6-nonafluorohexyl)stannane	240497-26-1	FC(F)(F)C(F)(F)C(F)(F)C(F)(F)CC[SnH](CCC(F)(F)C(F)(F)C(F)(F)C(F)(F)F)CCC(F)(F)C(F)(F)C(F)(F)C(F)(F)F		-8.41	0.67	2.73	P	IN
QL02973	4,4,5,5,6,6,7,7,7-Nonafluorohept-1-ene	129846-67-9	FC(F)(F)C(F)(F)C(F)(F)C(F)(F)CC=C		-6.13	0.65	1.91	P	IN
QL02974	[(1Z)-4,4,5,5,6,6,7,7,7-Nonafluorohept-1-en-1-yl]benzene	97041-10-6	FC(F)(F)C(F)(F)C(F)(F)C(F)(F)CC=C/c1ccccc1		-6.75	0.63	2.49	P	OUT
QL02975	2-(2,2,3,3,4,4,5,5,5-Nonafluoropentyl)oxirane	81190-28-5	FC(F)(F)C(F)(F)C(F)(F)C(F)(F)CC1CO1		-6.56	0.66	1.90	P	IN
QL02976	4-(2,2,3,3,4,4,5,5,5-Nonafluoropentyl)-1,3-dioxolan-2-one	912364-40-0	FC(F)(F)C(F)(F)C(F)(F)C(F)(F)CC1COC(=O)O1		-6.90	0.65	2.26	P	IN
QL02977	1H,1H,2H,2H-Perfluorohexyl bromide	38436-14-5	FC(F)(F)C(F)(F)C(F)(F)C(F)(F)CCBr		-6.13	0.65	1.91	P	IN
QL02978	1-[(4,4,5,5,6,6,7,7,7-Nonafluoroheptanoyl)oxy]pyrrolidine-2,5-dione	852527-43-6	FC(F)(F)C(F)(F)C(F)(F)C(F)(F)CCC(=O)ON1C(=O)CCC1=O		-7.00	0.64	2.45	P	IN
QL02979	1,1,1,2,2,3,3,4,4,5,5,6,6,7,7,8,8,11,11,12,12,13,13,14,14,14-Hexacosafluorotetradecane	53638-09-8	FC(F)(F)C(F)(F)C(F)(F)C(F)(F)CCC(F)(F)C(F)(F)C(F)(F)C(F)(F)C(F)(F)C(F)(F)C(F)(F)C(F)(F)F		-9.21	0.69	2.93	P	OUT
QL02980	Decane, 1,1,1,2,2,3,3,4,4,7,7,8,8,9,9,10,10,10-octadecafluoro-	142083-52-1	FC(F)(F)C(F)(F)C(F)(F)C(F)(F)CCC(F)(F)C(F)(F)C(F)(F)C(F)(F)F		-8.11	0.68	2.45	P	IN
QL02981	1,1,1,2,2,3,3,4,4-Nonafluoro-7,7-diisocyanatoheptane	306976-22-7	FC(F)(F)C(F)(F)C(F)(F)C(F)(F)CCC(N=C=O)N=C=O		-6.19	0.61	2.42	P	OUT
QL02982	4,4,5,5,6,6,7,7,7-Nonafluoroheptanenitrile	26649-23-0	FC(F)(F)C(F)(F)C(F)(F)C(F)(F)CCC#N		-6.48	0.64	2.21	P	IN
QL02983	1-[[[[4-(2-(Perfluorobutyl)-1-ethyl)phenyl]methoxy]carbonyl]oxy]-2,5-pyrrolidinedione	825635-46-9	FC(F)(F)C(F)(F)C(F)(F)C(F)(F)CCC1=CC=C(COC(=O)ON2C(=O)CCC2=O)C=C1		-7.67	0.63	3.02	P	OUT
QL02984	8-Bromo-1,1,1,2,2,3,3,4,4-nonafluorooctane	38436-18-9	FC(F)(F)C(F)(F)C(F)(F)C(F)(F)CCCCBr		-6.41	0.63	2.28	P	OUT
QL02985	10-Bromo-1,1,1,2,2,3,3,4,4-nonafluorodecane	457603-91-7	FC(F)(F)C(F)(F)C(F)(F)C(F)(F)CCCCCCBr		-6.52	0.62	2.52	P	OUT

QL02986	1,1,1,2,2,3,3,4,4,11,11,12,12,13,13,14,14,14-Octadecafluorotetradecane	1835252-03-3	FC(F)(F)C(F)(F)C(F)(F)C(F)(F)CCCCCCC(F)(F)C(F)(F)C(F)(F)C(F)(F)F		-8.19	0.66	2.81	P	IN
QL02987	1,1,1,2,2,3,3,4,4,13,13,14,14,15,15,16,16,16-Octadecafluorohexadecane	1835252-00-0	FC(F)(F)C(F)(F)C(F)(F)C(F)(F)CCCCCCCCC(F)(F)C(F)(F)C(F)(F)C(F)(F)F		-8.24	0.65	2.97	P	IN
QL02988	12,12,13,13,14,14,15,15,15-Nonafluoropentadecanoyl chloride	118624-68-3	FC(F)(F)C(F)(F)C(F)(F)C(F)(F)CCCCCCCCCCC(Cl)=O		-7.12	0.60	3.02	P	OUT
QL02989	1,1,1,2,2,3,3,4,4,15,15,16,16,17,17,18,18,18-Octadecafluorooctadecane	1835251-99-4	FC(F)(F)C(F)(F)C(F)(F)C(F)(F)CCCCCCCCCCC(F)(F)C(F)(F)C(F)(F)C(F)(F)F		-8.30	0.64	3.11	P	OUT
QL02990	15-Bromo-1,1,1,2,2,3,3,4,4-nonafluoropentadecane	213207-95-5	FC(F)(F)C(F)(F)C(F)(F)C(F)(F)CCCCCCCCCCCBr		-6.89	0.60	3.04	P	OUT
QL02991	1,1,1,2,2,3,3,4,4-Nonafluoro-8-iodooctane	99324-98-8	FC(F)(F)C(F)(F)C(F)(F)C(F)(F)CCCCI		-6.41	0.63	2.28	P	OUT
QL02992	4,4,5,5,6,6,7,7,7-Nonafluoroheptyl iodide	183547-74-2	FC(F)(F)C(F)(F)C(F)(F)C(F)(F)CCCI		-6.48	0.64	2.21	P	IN
QL02993	4,4,5,5,6,6,7,7,7-Nonafluoroheptyl prop-2-enoate	443145-46-8	FC(F)(F)C(F)(F)C(F)(F)C(F)(F)CCCOC(=O)C=C		-6.77	0.63	2.51	P	OUT
QL02994	4,4,5,5,6,6,7,7,7-Nonafluoroheptyl carbonochloridate	821806-42-2	FC(F)(F)C(F)(F)C(F)(F)C(F)(F)CCCOC(Cl)=O		-6.85	0.64	2.44	P	IN
QL02995	(3,3,4,4,5,5,6,6,6-Nonafluorohexyl)carbonimidoyl	142030-27-1	FC(F)(F)C(F)(F)C(F)(F)C(F)(F)CCN=C(Cl)Cl		-6.70	0.64	2.27	P	IN
QL02996	Bis(3,3,4,4,5,5,6,6,6-nonafluorohexyl) butanedioate	508194-70-5	FC(F)(F)C(F)(F)C(F)(F)C(F)(F)CCOC(=O)CCC(=O)OCCC(F)(F)C(F)(F)C(F)(F)C(F)(F)F		-8.53	0.65	3.14	P	OUT
QL02997	bis((Perfluorobutyl)ethyl) carbonate	1132677-78-1	FC(F)(F)C(F)(F)C(F)(F)C(F)(F)CCOC(=O)OCCC(F)(F)C(F)(F)C(F)(F)C(F)(F)F		-8.42	0.66	2.95	P	IN
QL02998	3,3,4,4,5,5,6,6,6-Nonafluorohexyl carbonochloridate	40678-17-9	FC(F)(F)C(F)(F)C(F)(F)C(F)(F)CCOC(Cl)=O		-6.70	0.64	2.27	P	IN
QL02999	6-(Ethenyloxy)-1,1,1,2,2,3,3,4,4-nonafluorohexane	63391-84-4	FC(F)(F)C(F)(F)C(F)(F)C(F)(F)CCOC=C		-6.41	0.63	2.28	P	OUT
QL03000	3,3,4,4,5,5,6,6,6-Nonafluorohexyl formate	78689-20-0	FC(F)(F)C(F)(F)C(F)(F)C(F)(F)CCOC=O		-6.41	0.63	2.28	P	OUT
QL03001	1,1,1,2,2,3,3,4,4-Nonafluoro-6-[(prop-2-en-1-yl)oxy]hexane	188298-09-1	FC(F)(F)C(F)(F)C(F)(F)C(F)(F)CCOCC=C		-6.57	0.63	2.46	P	OUT
QL03002	2-{[(3,3,4,4,5,5,6,6,6-Nonafluorohexyl)oxy]methyl}oxirane	384828-82-4	FC(F)(F)C(F)(F)C(F)(F)C(F)(F)CCOCC1CO1		-7.01	0.64	2.44	P	IN
QL03003	3-[(3,3,4,4,5,5,6,6,6-Nonafluorohexyl)oxy]propanenitrile	56300-32-4	FC(F)(F)C(F)(F)C(F)(F)C(F)(F)CCOCCC#N		-6.52	0.62	2.52	P	OUT
QL03004	Tris(1H,1H,2H,2H-perfluorohexyl) phosphate	1887067-55-1	FC(F)(F)C(F)(F)C(F)(F)C(F)(F)CCOP(=O)(OCCC(F)(F)C(F)(F)C(F)(F)C(F)(F)F)OCCC(F)(F)C(F)(F)C(F)(F)C(F)(F)F		-8.60	0.67	2.92	P	IN
QL03005	3,3,4,4,5,5,6,6,6-Nonafluorohexyl trifluoromethanesulfonate	171182-86-8	FC(F)(F)C(F)(F)C(F)(F)C(F)(F)CCOS(=O)(=O)C(F)(F)F		-7.65	0.67	2.39	P	IN
QL03006	3,3,4,4,5,5,6,6,6-Nonafluoro-1-hexanethiol	68140-20-5	FC(F)(F)C(F)(F)C(F)(F)C(F)(F)CCS		-6.13	0.65	1.91	P	IN
QL03007	n2 fluorotelomer sulfonyl propano-amido alkyl sulfonic acids, n=2		FC(F)(F)C(F)(F)C(F)(F)C(F)(F)CCS(CCC(NC(C)(C)CS(O)(=O)=O)=O)(=O)=O		-8.09	0.65	2.90	P	IN
QL03008	n2 fluorotelomer thioamido sulfonic acids, n=2		FC(F)(F)C(F)(F)C(F)(F)C(F)(F)CCS(CCC(NC(C)(C)CS(O)(=O)=O)=O)=O		-7.84	0.64	2.89	P	OUT
QL03009	n2 fluorotelomer sulfonyl propanoic acids, n=2		FC(F)(F)C(F)(F)C(F)(F)C(F)(F)CCS(CCC(O)=O)(=O)=O		-7.22	0.65	2.47	P	IN
QL03010	n2 fluorotelomer sulfonamido betaine-related, n=2		FC(F)(F)C(F)(F)C(F)(F)C(F)(F)CCS(NCCC[N+](CC(O)=O)(C)C)(=O)=O		-7.77	0.64	2.89	P	OUT
QL03011	3,3,4,4,5,5,6,6,6-Nonafluorohexyl thiocyanate	26650-08-8	FC(F)(F)C(F)(F)C(F)(F)C(F)(F)CCSC#N		-6.41	0.63	2.28	P	OUT
QL03012	n2 fluorotelomer thio alkyl amido betaine-related, n=2		FC(F)(F)C(F)(F)C(F)(F)C(F)(F)CCSCC(NCCC[N+](C)(C)CC(O)=O)=O		-7.58	0.62	3.04	P	OUT
QL03013	n2 fluorotelomer thio alkyl amido ammonium, n=2		FC(F)(F)C(F)(F)C(F)(F)C(F)(F)CCSCC(NCCCN(C)C)=O		-7.19	0.62	2.92	P	OUT
QL03014	n2 fluorotelomer thio alkyl amido sulfonic acids, n=2		FC(F)(F)C(F)(F)C(F)(F)C(F)(F)CCSCCC(NC(C)(C)CS(O)(=O)=O)=O		-7.68	0.64	2.87	P	OUT
QL03015	1-Iodo-1H,1H-nonafluoropentane	2253-14-7	FC(F)(F)C(F)(F)C(F)(F)C(F)(F)CI		-6.40	0.67	1.81	P	IN
QL03016	1-Chlorononafluorobutane	558-89-4	FC(F)(F)C(F)(F)C(F)(F)C(F)(F)Cl		-6.18	0.68	1.45	P	IN
QL03017	2,2,3,3,4,4,5,5,5-Nonafluoropentyl acrylate	308-26-9	FC(F)(F)C(F)(F)C(F)(F)C(F)(F)COC(=O)C=C		-6.68	0.64	2.27	P	IN
QL03018	1,1,1,2,2,3,3,4,4-Nonafluoro-5-[(prop-2-en-1-yl)oxy]pentane	646063-05-0	FC(F)(F)C(F)(F)C(F)(F)C(F)(F)COCC=C		-6.41	0.63	2.28	P	OUT
QL03019	5-[2-(Ethenyloxy)ethoxy]-1,1,1,2,2,3,3,4,4-nonafluoropentane	572911-07-0	FC(F)(F)C(F)(F)C(F)(F)C(F)(F)COCCOC=C		-6.52	0.62	2.52	P	OUT
QL03020	Perfluorobutane	355-25-9	FC(F)(F)C(F)(F)C(F)(F)C(F)(F)F		-6.18	0.68	1.45	P	IN
QL03021	Perfluoro-2-butyl-3-propyloxaziridine	143813-70-1	FC(F)(F)C(F)(F)C(F)(F)C(F)(F)N1OC1(F)C(F)(F)C(F)(F)C(F)(F)F		-8.24	0.69	2.43	P	IN
QL03022	Nonafluorobutyl prop-2-enoate	2708-03-4	FC(F)(F)C(F)(F)C(F)(F)C(F)(F)OC(=O)C=C		-6.67	0.65	2.19	P	IN
QL03023	ether-substituted perfluoroalkyl (linear) caborxylic acids_i, n=4		FC(F)(F)C(F)(F)C(F)(F)C(F)(F)OC(F)(C(F)(F)F)C(O)=O		-7.22	0.67	2.11	P	IN
QL03024	Ether, bis(nonafluorobutyl)	308-48-5	FC(F)(F)C(F)(F)C(F)(F)C(F)(F)OC(F)(F)C(F)(F)C(F)(F)C(F)(F)F		-8.08	0.69	2.38	P	IN
QL03025	1-Bromoperfluoro-2,5,8-trioxadodecane	330562-47-5	FC(F)(F)C(F)(F)C(F)(F)C(F)(F)OC(F)(F)C(F)(F)OC(F)(F)C(F)(F)OC(F)(F)Br		-8.37	0.67	2.69	P	IN

QL03026	4-Pyridinecarboxamide, N-[4-(nonafluorobutoxy)phenyl]-	62315-14-4	FC(F)(F)C(F)(F)C(F)(F)C(F)(F)OC1=CC=C(NC(=O)C2=CC=NC=C2)C=C1		-7.40	0.62	2.90	P	OUT
QL03027	3-Pyridinecarboxamide, N-[4-(nonafluorobutoxy)phenyl]-	62315-07-5	FC(F)(F)C(F)(F)C(F)(F)C(F)(F)OC1=CC=C(NC(=O)C2=CN=CC=C2)C=C1		-7.40	0.62	2.90	P	OUT
QL03028	Tris(nonafluorobutyl)(oxo)-lambda~5~-phosphane	58431-34-8	FC(F)(F)C(F)(F)C(F)(F)C(F)(F)P(=O)(C(F)(F)C(F)(F)C(F)(F)C(F)(F)F)C(F)(F)C(F)(F)C(F)(F)C(F)(F)F		-8.26	0.69	2.38	P	IN
QL03029	Phosphorane, difluorotris(nonafluorobutyl)-	91543-34-9	FC(F)(F)C(F)(F)C(F)(F)C(F)(F)P(F)(F)(C(F)(F)C(F)(F)C(F)(F)C(F)(F)F)C(F)(F)C(F)(F)C(F)(F)C(F)(F)F		-8.44	0.70	2.40	P	IN
QL03030	[S-(1,1,2,2,3,3,4,4,4-Nonafluorobutane-1-)sulfonimidoyl]benzene	1335014-68-0	FC(F)(F)C(F)(F)C(F)(F)C(F)(F)S(=N)(=O)c1ccccc1		-6.91	0.65	2.24	P	IN
QL03031	N-[(4-Bromophenyl)(perfluorobutyl)oxido-λ4-sulfanylidene]-1,1,1-trifluoromethanesulfonamide	1421133-01-8	FC(F)(F)C(F)(F)C(F)(F)C(F)(F)S(=O)(=NS(=O)(=O)C(F)(F)F)C1=CC=C(Br)C=C1		-7.46	0.66	2.40	P	IN
QL03032	bis(Perfluorobutylsulfonyl)methylenephenyliodine	681477-42-9	FC(F)(F)C(F)(F)C(F)(F)C(F)(F)S(=O)(=O)C(=[I]C1=CC=CC=C1)S(=O)(=O)C(F)(F)C(F)(F)C(F)(F)C(F)(F)F		-8.03	0.67	2.63	P	IN
QL03033	1-[Bis(1,1,2,2,3,3,4,4,4-nonafluorobutane-1-sulfonyl)methanesulfonyl]-1,1,2,2,3,3,4,4,4-nonafluorobutane	182505-69-7	FC(F)(F)C(F)(F)C(F)(F)C(F)(F)S(=O)(=O)C(S(=O)(=O)C(F)(F)C(F)(F)C(F)(F)C(F)(F)F)S(=O)(=O)C(F)(F)C(F)(F)C(F)(F)C(F)(F)F		-8.67	0.69	2.61	P	IN
QL03034	[(1,1,2,2,3,3,4,4,4-Nonafluorobutane-1-sulfonyl)ethynyl]benzene	63369-94-8	FC(F)(F)C(F)(F)C(F)(F)C(F)(F)S(=O)(=O)C#CC1=CC=CC=C1		-7.22	0.64	2.53	P	IN
QL03035	2-(1,1,2,2,3,3,4,4,4-Nonafluorobutane-1-sulfonyl)-1-phenylethan-1-one	58510-75-1	FC(F)(F)C(F)(F)C(F)(F)C(F)(F)S(=O)(=O)CC(=O)C1=CC=CC=C1		-7.26	0.65	2.53	P	IN
QL03036	[(1,1,2,2,3,3,4,4,4-Nonafluorobutane-1-sulfonyl)methyl]benzene	879081-10-4	FC(F)(F)C(F)(F)C(F)(F)C(F)(F)S(=O)(=O)Cc1ccccc1		-7.21	0.65	2.47	P	IN
QL03037	1,1,1,2,2,3,3,4,4-Nonafluoro-4-[(1,1,2,2,3,3,4,4,4-nonafluorobutane-1-sulfonyl)methanesulfonyl]butane	29214-37-7	FC(F)(F)C(F)(F)C(F)(F)C(F)(F)S(=O)(=O)CS(=O)(=O)C(F)(F)C(F)(F)C(F)(F)C(F)(F)F		-8.64	0.69	2.63	P	IN
QL03038	N-[(4-Bromophenyl)oxido(trifluoromethyl)sulfanylidene]perfluorobutanesulfonamide	1421133-00-7	FC(F)(F)C(F)(F)C(F)(F)C(F)(F)S(=O)(=O)N=S(=O)(C1=CC=C(Br)C=C1)C(F)(F)F		-7.86	0.66	2.60	P	IN
QL03039	1-(1,1,2,2,3,3,4,4,4-Nonafluorobutane-1-sulfonyl)-1H-benzotriazole	681443-29-8	FC(F)(F)C(F)(F)C(F)(F)C(F)(F)S(=O)(=O)n1nnc2ccccc12		-7.30	0.65	2.45	P	IN
QL03040	N-(1,1,2,2,3,3,4,4,4-Nonafluorobutane-1-sulfonyl)cyclopent-2-ene-1-carboxamide	1440968-24-0	FC(F)(F)C(F)(F)C(F)(F)C(F)(F)S(=O)(=O)NC(=O)C1CCC=C1		-7.30	0.65	2.47	P	IN
QL03041	N-(1,1,2,2,3,3,4,4,4-Nonafluorobutane-1-sulfonyl)cyclopentanecarboxamide	1440968-21-7	FC(F)(F)C(F)(F)C(F)(F)C(F)(F)S(=O)(=O)NC(=O)C1CCCC1		-7.30	0.65	2.47	P	IN
QL03042	N-(1,1,2,2,3,3,4,4,4-Nonafluorobutane-1-sulfonyl)cyclohexanecarboxamide	1440968-37-5	FC(F)(F)C(F)(F)C(F)(F)C(F)(F)S(=O)(=O)NC(=O)C1CCCCC1		-7.26	0.65	2.53	P	IN
QL03043	N,N'-(3,4-Dioxocyclobut-1-ene-1,2-diyl)bis(1,1,2,2,3,3,4,4,4-nonafluorobutane-1-sulfonamide)	1234571-87-9	FC(F)(F)C(F)(F)C(F)(F)C(F)(F)S(=O)(=O)NC1=C(NS(=O)(=O)C(F)(F)C(F)(F)C(F)(F)C(F)(F)F)C(=O)C1=O		-8.95	0.68	2.93	P	OUT
QL03044	N-Cyclooctyl-1,1,2,2,3,3,4,4,4-nonafluorobutane-1-sulfonamide	1456900-48-3	FC(F)(F)C(F)(F)C(F)(F)C(F)(F)S(=O)(=O)NC1CCCCCCC1		-7.00	0.64	2.53	P	OUT
QL03045	1,1,2,2,3,3,4,4,4-nonafluoro-N-[(trifluoromethyl)sulfonyl]-1-Butanesulfonamide	39847-37-5	FC(F)(F)C(F)(F)C(F)(F)C(F)(F)S(=O)(=O)NS(=O)(=O)C(F)(F)F		-7.73	0.68	2.23	P	IN
QL03046	Vinyl nonafluoro-1-butanesulfonate	42409-05-2	FC(F)(F)C(F)(F)C(F)(F)C(F)(F)S(=O)(=O)OC=C		-6.87	0.66	2.17	P	IN
QL03047	Phenyl nonafluorobutane-1-sulfonate	25628-11-9	FC(F)(F)C(F)(F)C(F)(F)C(F)(F)S(=O)(=O)OC1=CC=CC=C1		-7.21	0.65	2.47	P	IN
QL03048	Cyclopent-1-en-1-yl nonafluorobutane-1-sulfonate	36839-94-8	FC(F)(F)C(F)(F)C(F)(F)C(F)(F)S(=O)(=O)OC1=CCCC1		-6.95	0.65	2.24	P	IN
QL03049	Cyclohex-1-en-1-yl nonafluorobutane-1-sulfonate	36839-95-9	FC(F)(F)C(F)(F)C(F)(F)C(F)(F)S(=O)(=O)OC1=CCCCC1		-7.21	0.65	2.47	P	IN
QL03050	Cyclohept-1-en-1-yl nonafluorobutane-1-sulfonate	42108-79-2	FC(F)(F)C(F)(F)C(F)(F)C(F)(F)S(=O)(=O)OC1=CCCCCC1		-7.03	0.64	2.47	P	IN
QL03051	4-Cyanophenyl nonafluorobutane-1-sulfonate	626201-15-8	FC(F)(F)C(F)(F)C(F)(F)C(F)(F)S(=O)(=O)Oc1ccc(cc1)C#N		-7.50	0.64	2.68	P	IN
QL03052	2,4-Dichlorophenyl nonafluorobutane-1-sulfonate	32848-21-8	FC(F)(F)C(F)(F)C(F)(F)C(F)(F)S(=O)(=O)Oc1ccc(Cl)cc1Cl		-7.50	0.65	2.52	P	IN
QL03053	Quinolin-8-yl nonafluorobutane-1-sulfonate	41605-60-1	FC(F)(F)C(F)(F)C(F)(F)C(F)(F)S(=O)(=O)Oc1cccc2cccnc12		-7.16	0.64	2.51	P	IN
QL03054	2-Cyanophenyl nonafluorobutane-1-sulfonate	873838-37-0	FC(F)(F)C(F)(F)C(F)(F)C(F)(F)S(=O)(=O)Oc1ccccc1C#N		-7.01	0.64	2.45	P	IN
QL03055	1,2-Ethanediyl bis(perfluorobutanesulfonate)	92982-03-1	FC(F)(F)C(F)(F)C(F)(F)C(F)(F)S(=O)(=O)OCCOS(=O)(=O)C(F)(F)C(F)(F)C(F)(F)C(F)(F)F		-8.70	0.67	2.85	P	IN
QL03056	2-[(1,1,2,2,3,3,4,4,4-Nonafluorobutane-1-sulfonyl)oxy]-3a,4,7,7a-tetrahydro-1H-4,7-methanoisoindole-1,3(2H)-dione	307531-76-6	FC(F)(F)C(F)(F)C(F)(F)C(F)(F)S(=O)(=O)ON1C(=O)C2C3CC(C=C3)C2C1=O		-7.61	0.66	2.50	P	IN
QL03057	1,1,2,2,3,3,4,4,4-nonafluoro-N-(2-hydroxyethyl)-1-Butanesulfonamide	34454-99-4	FC(F)(F)C(F)(F)C(F)(F)C(F)(F)S(=O)(NCCO)=O		-6.81	0.65	2.25	P	IN
QL03058	[1-(1,1,2,2,3,3,4,4,4-Nonafluorobutane-1-sulfinyl)ethenyl]benzene	1606997-41-4	FC(F)(F)C(F)(F)C(F)(F)C(F)(F)S(=O)C(=C)c1ccccc1		-6.99	0.64	2.45	P	IN
QL03059	Perfluoro-1-butanesulfonyl chloride	2991-84-6	FC(F)(F)C(F)(F)C(F)(F)C(F)(F)S(Cl)(=O)=O		-6.93	0.69	1.79	P	IN

QL03060	Perfluorobutanesulfinyl chloride	41006-41-1	FC(F)(F)C(F)(F)C(F)(F)C(F)(F)S(Cl)=O		-6.61	0.67	1.81	P	IN
QL03061	Perfluorobutane sulfonamidoacetic acid	347872-22-4	FC(F)(F)C(F)(F)C(F)(F)C(F)(F)S(NCC(O)=O)(=O)=O		-7.07	0.66	2.24	P	IN
QL03062	Perfluoroalkyl (linear) sulfinate, n=4	34642-43-8	FC(F)(F)C(F)(F)C(F)(F)C(F)(F)S(O)=O		-6.61	0.67	1.81	P	IN
QL03063	[(Nonafluorobutyl)disulfanyl]benzene	1606997-42-5	FC(F)(F)C(F)(F)C(F)(F)C(F)(F)SSc1ccccc1		-6.80	0.64	2.43	P	OUT
QL03064	1,1,1,2,2,3,3,4,5,5,5-Undecafluoro-4-iodopentane	68158-13-4	FC(F)(F)C(F)(F)C(F)(F)C(F)(I)C(F)(F)F		-6.93	0.69	1.79	P	IN
QL03065	1,1,1,2,2,3,3,5,5,5-Decafluoro-4-iodo-4-(trifluoromethyl)pentane	102780-88-1	FC(F)(F)C(F)(F)C(F)(F)C(I)(C(F)(F)F)C(F)(F)F		-6.74	0.68	1.72	P	IN
QL03066	1-ethoxy-2,2,3,3,4,4,4-heptafluorobutan-1-ol	356-26-3	FC(F)(F)C(F)(F)C(F)(F)C(O)OCC		-5.78	0.63	1.93	P	IN
QL03067	2,2,3,3,4,4,4-Heptafluoro-1-phenylbutyl diphenyl phosphate	98010-21-0	FC(F)(F)C(F)(F)C(F)(F)C(OP(=O)(Oc1ccccc1)Oc1ccccc1)c1ccccc1		-5.88	0.60	2.42	P	OUT
QL03068	3,3,4,4,5,5,5-heptafluoropent-1-yne	80337-25-3	FC(F)(F)C(F)(F)C(F)(F)C#C		-5.48	0.66	1.46	P	IN
QL03069	4,4,5,5,6,6,6-Heptafluoro-1-phenylhex-2-yn-1-one	89965-72-0	FC(F)(F)C(F)(F)C(F)(F)C#CC(=O)C1=CC=CC=C1		-6.47	0.62	2.42	P	OUT
QL03070	(3,3,4,4,5,5,5-Heptafluoropent-1-yn-1-yl)benzene	81674-08-0	FC(F)(F)C(F)(F)C(F)(F)C#Cc1ccccc1		-6.08	0.62	2.21	P	OUT
QL03071	Heptafluorobutyronitrile	375-00-8	FC(F)(F)C(F)(F)C(F)(F)C#N		-5.48	0.66	1.46	P	IN
QL03072	3,3,4,4,5,5,5-heptafluoropent-1-ene	355-08-8	FC(F)(F)C(F)(F)C(F)(F)C=C		-5.48	0.66	1.46	P	IN
QL03073	3-(2,2,3,3,4,4,4-Heptafluorobutylidene)oxolan-2-one	89608-45-7	FC(F)(F)C(F)(F)C(F)(F)C=C1CCOC1=O		-5.97	0.64	1.90	P	IN
QL03074	4,4,5,5,6,6,6-Heptafluoro-1-phenylhex-2-en-1-one	110960-47-9	FC(F)(F)C(F)(F)C(F)(F)C=CC(=O)c1ccccc1		-6.47	0.62	2.42	P	OUT
QL03075	1,1,1,2,2,3,3,6,6,7,7,8,8,9,9,10,10,11,11,11-Icosafluoroundec-4-ene	100603-63-2	FC(F)(F)C(F)(F)C(F)(F)C=CC(F)(F)C(F)(F)C(F)(F)C(F)(F)C(F)(F)C(F)(F)F		-8.61	0.68	2.68	P	IN
QL03076	1,1,1,2,2,3,3,6,6,7,7,8,8,9,9,10,10,10-Octadecafluorodec-4-ene	935477-16-0	FC(F)(F)C(F)(F)C(F)(F)C=CC(F)(F)C(F)(F)C(F)(F)C(F)(F)C(F)(F)F		-8.22	0.68	2.50	P	IN
QL03077	1,1,1,2,2,3,3,6,6,7,7,8,8,9,9,9-Hexadecafluoronon-4-ene	935477-07-9	FC(F)(F)C(F)(F)C(F)(F)C=CC(F)(F)C(F)(F)C(F)(F)C(F)(F)F		-7.92	0.68	2.38	P	IN
QL03078	1,1,1,2,2,3,3,6,6,7,7,8,8,8-Tetradecafluorooct-4-ene	3910-82-5	FC(F)(F)C(F)(F)C(F)(F)C=CC(F)(F)C(F)(F)C(F)(F)F		-7.41	0.68	2.16	P	IN
QL03080	3,3,4,4,5,5,5-Heptafluoro-1-iodo-1-pentene	376-97-6	FC(F)(F)C(F)(F)C(F)(F)C=CI		-5.71	0.64	1.85	P	IN
QL03082	(1E)-2,2,3,3,4,4,4-Heptafluoro-N-phenylbutan-1-imine	62765-24-6	FC(F)(F)C(F)(F)C(F)(F)C=NC1=CC=CC=C1		-6.08	0.62	2.21	P	OUT
QL03083	(1E)-2,2,3,3,4,4,4-Heptafluoro-N-(2-phenylethyl)butan-1-imine	29723-32-8	FC(F)(F)C(F)(F)C(F)(F)C=NCCC1=CC=CC=C1		-6.22	0.61	2.46	P	OUT
QL03084	Perfluorobutyraldehyde	375-02-0	FC(F)(F)C(F)(F)C(F)(F)C=O		-5.48	0.66	1.46	P	IN
QL03085	Undecafluoro(heptafluoropropyl)cyclohexane	374-59-4	FC(F)(F)C(F)(F)C(F)(F)C1(F)C(F)(F)C(F)(F)C(F)(F)C(F)(F)C1(F)F		-7.81	0.70	2.08	P	IN
QL03086	2,2,3,3,4,4,5,6,6-Nonafluoro-5-(heptafluoropropyl)oxane	801-26-3	FC(F)(F)C(F)(F)C(F)(F)C1(F)C(F)(F)OC(F)(F)C(F)(F)C1(F)F		-7.67	0.69	2.10	P	IN
QL03087	3,4,4-Trifluoro-3-(heptafluoropropyl)-1,2lambda~6~-oxathietane-2,2-dione	156465-44-0	FC(F)(F)C(F)(F)C(F)(F)C1(F)C(F)(F)OS1(=O)=O		-6.91	0.69	1.75	P	IN
QL03088	2,2,3,3,4,4,5,5,6-Nonafluoro-6-(heptafluoropropyl)tetrahydro-2H-pyran	335-35-3	FC(F)(F)C(F)(F)C(F)(F)C1(F)OC(F)(F)C(F)(F)C(F)(F)C1(F)F		-7.75	0.69	2.11	P	IN
QL03089	2,2,3,3,4,4,5-Heptafluoro-5-(heptafluoropropyl)oxolane	423-22-3	FC(F)(F)C(F)(F)C(F)(F)C1(F)OC(F)(F)C(F)(F)C1(F)F		-7.26	0.69	1.88	P	IN
QL03090	2,2,3-Trifluoro-3-(heptafluoropropyl)oxirane	74942-19-1	FC(F)(F)C(F)(F)C(F)(F)C1(F)OC1(F)F		-7.03	0.69	1.81	P	IN
QL03091	1,4-Bis(chloromethyl)-2-(heptafluoropropyl)benzene	139263-35-7	FC(F)(F)C(F)(F)C(F)(F)C1=C(CCl)C=CC(CCl)=C1		-5.84	0.62	2.13	P	OUT
QL03092	5-(Heptafluoropropyl)-3-phenyl-1H-pyrazole	54864-78-7	FC(F)(F)C(F)(F)C(F)(F)C1=CC(=NN1)C1=CC=CC=C1		-6.56	0.63	2.43	P	OUT
QL03093	6-(Heptafluoropropyl)-2-sulfanylidene-2,3-dihydropyrimidin-4(1H)-one	88191-88-2	FC(F)(F)C(F)(F)C(F)(F)C1=CC(=O)NC(=S)N1		-6.01	0.64	1.91	P	IN
QL03094	1-Bromo-3-(heptafluoropropyl)benzene	53104-06-6	FC(F)(F)C(F)(F)C(F)(F)C1=CC(Br)=CC=C1		-5.95	0.64	1.92	P	IN
QL03095	2-{[4-(Heptafluoropropyl)phenyl]methyl}oxirane	144006-68-8	FC(F)(F)C(F)(F)C(F)(F)C1=CC=C(CC2CO2)C=C1		-6.96	0.64	2.50	P	OUT
QL03096	N-[6-(Heptafluoropropyl)-2-oxo-2H-pyran-3-yl]benzamide	860454-18-8	FC(F)(F)C(F)(F)C(F)(F)C1=CC=C(NC(=O)c2ccccc2)C(=O)O1		-7.05	0.62	2.75	P	OUT
QL03097	Quinoline, 4-(heptafluoropropyl)-	181059-73-4	FC(F)(F)C(F)(F)C(F)(F)C1=CC=NC2=CC=CC=C12		-6.06	0.63	2.13	P	OUT
QL03098	AC1LC6EI	60007-37-6	FC(F)(F)C(F)(F)C(F)(F)C1=CNC(=O)NC1=O		-6.51	0.64	2.16	P	IN
QL03099	5-(Heptafluoropropyl)-2-sulfanylidene-2,3-dihydropyrimidin-4(1H)-one	145663-11-2	FC(F)(F)C(F)(F)C(F)(F)C1=CNC(=S)NC1=O		-6.51	0.64	2.16	P	IN
QL03100	(4S)-4-Benzyl-2-(heptafluoropropyl)-4,5-dihydro-1,3-oxazole	1366341-52-7	FC(F)(F)C(F)(F)C(F)(F)C1=N[C@@H](Cc2ccccc2)CO1		-6.61	0.62	2.52	P	OUT
QL03101	2,4,6-Tris(heptafluoropropyl)-1,3,5-triazine	915-76-4	FC(F)(F)C(F)(F)C(F)(F)C1=NC(=NC(=N1)C(F)(F)C(F)(F)C(F)(F)F)C(F)(F)C(F)(F)C(F)(F)F		-7.55	0.68	2.23	P	IN
QL03102	MLS002694827	4314-49-2	FC(F)(F)C(F)(F)C(F)(F)C1=NC(=O)ON1		-6.19	0.65	1.92	P	IN
QL03103	Benzimidazole, 5(or 6)-chloro-2-(heptafluoropropyl)-	6866-58-6	FC(F)(F)C(F)(F)C(F)(F)C1=NC2=C(N1)C=C(Cl)C=C2		-6.73	0.64	2.29	P	IN

QL03104	2-(Heptafluoropropyl)-1H-benzimidazole	559-37-5	FC(F)(F)C(F)(F)C(F)(F)C1=NC2=CC=CC=C2N1		-6.37	0.64	2.16	P	IN
QL03105	2-(Perfluoropropyl)benzoxazole	2008-06-2	FC(F)(F)C(F)(F)C(F)(F)C1=NC2=CC=CC=C2O1		-6.37	0.64	2.16	P	IN
QL03106	4-(Heptafluoropropyl)-1,3-dihydro-2H-1,5-benzodiazepin-2-one	96146-10-0	FC(F)(F)C(F)(F)C(F)(F)C1=Nc2ccccc2NC(=O)C1		-6.29	0.63	2.24	P	OUT
QL03107	2-(heptafluoropropyl)-4,5-dihydro-1h-imidazole	4472-70-2	FC(F)(F)C(F)(F)C(F)(F)C1=NCCN1		-5.85	0.64	1.83	P	IN
QL03108	2-(Heptafluoropropyl)-1-phenyl-4,5-dihydro-1H-imidazole	1069085-53-5	FC(F)(F)C(F)(F)C(F)(F)C1=NCCN1c1ccccc1		-6.13	0.62	2.26	P	OUT
QL03109	1-Benzyl-2-(heptafluoropropyl)-4,5-dihydro-1H-imidazole	1069085-51-3	FC(F)(F)C(F)(F)C(F)(F)C1=NCCN1Cc1ccccc1		-6.43	0.62	2.46	P	OUT
QL03110	2-Perfluoropropyl-5-phenyl-1,3,4-oxadiazole	5756-67-2	FC(F)(F)C(F)(F)C(F)(F)C1=NN=C(O1)C1=CC=CC=C1		-6.56	0.63	2.43	P	OUT
QL03111	5-(heptafluoropropyl)-1-phenyl-1h-tetrazole	2712-85-8	FC(F)(F)C(F)(F)C(F)(F)C1=NN=NN1C1=CC=CC=C1		-6.13	0.62	2.26	P	OUT
QL03112	4-Bromo-2-(heptafluoropropyl)pyridine	1816283-37-0	FC(F)(F)C(F)(F)C(F)(F)c1cc(Br)ccn1		-5.95	0.64	1.92	P	IN
QL03113	4-Chloro-2-(heptafluoropropyl)pyridine	1816283-32-5	FC(F)(F)C(F)(F)C(F)(F)c1cc(Cl)ccn1		-5.95	0.64	1.92	P	IN
QL03114	1-Bromo-4-(heptafluoropropyl)benzene	117874-91-6	FC(F)(F)C(F)(F)C(F)(F)c1ccc(Br)cc1		-6.51	0.64	2.18	P	IN
QL03115	5-Bromo-2-(heptafluoropropyl)pyridine	1816282-96-8	FC(F)(F)C(F)(F)C(F)(F)c1ccc(Br)cn1		-6.51	0.64	2.18	P	IN
QL03116	1-(Heptafluoropropyl)-4-iodobenzene	1417885-93-8	FC(F)(F)C(F)(F)C(F)(F)c1ccc(I)cc1		-6.51	0.64	2.18	P	IN
QL03117	N-[5-(Heptafluoropropyl)[1,1'-biphenyl]-2-yl]benzamide	748785-00-4	FC(F)(F)C(F)(F)C(F)(F)c1ccc(NC(=O)c2ccccc2)c(c1)-c1ccccc1		-6.76	0.61	2.78	P	OUT
QL03118	2-(Heptafluoropropyl)quinoline	57702-79-1	FC(F)(F)C(F)(F)C(F)(F)c1ccc2ccccc2n1		-6.46	0.63	2.29	P	OUT
QL03119	5-(Heptafluoropropyl)isoquinoline-1-carbaldehyde	31181-32-5	FC(F)(F)C(F)(F)C(F)(F)c1cccc2c(C=O)nccc12		-6.11	0.62	2.24	P	OUT
QL03120	(Heptafluoropropyl)benzene	378-98-3	FC(F)(F)C(F)(F)C(F)(F)c1ccccc1		-5.79	0.63	1.92	P	IN
QL03121	2-(Heptafluoropropyl)cyclohexan-1-one	61282-91-5	FC(F)(F)C(F)(F)C(F)(F)C1CCCCC1=O		-5.87	0.64	1.90	P	IN
QL03122	1-(heptafluoropropyl)-2-iodocyclohexane	2728-75-8	FC(F)(F)C(F)(F)C(F)(F)C1CCCCC1I		-5.87	0.64	1.90	P	IN
QL03123	3-(Heptafluoropropyl)oxolane	143837-48-3	FC(F)(F)C(F)(F)C(F)(F)C1CCOC1		-5.85	0.64	1.83	P	IN
QL03124	5-(Heptafluoropropyl)dihydropyrimidine-2,4(1H,3H)-dione	90715-84-7	FC(F)(F)C(F)(F)C(F)(F)C1CNC(=O)NC1=O		-6.51	0.64	2.16	P	IN
QL03125	5-(Heptafluoropropyl)pyrimidine	1354703-21-1	FC(F)(F)C(F)(F)C(F)(F)c1cncnc1		-5.79	0.63	1.92	P	IN
QL03126	2-(Heptafluoropropyl)oxirane	51035-20-2	FC(F)(F)C(F)(F)C(F)(F)C1CO1		-5.74	0.67	1.46	P	IN
QL03127	5-(Heptafluoropropyl)-1H-pyrrole-2-carbaldehyde	123825-52-5	FC(F)(F)C(F)(F)C(F)(F)c1Nc(C=O)cc1		-6.35	0.64	2.18	P	IN
QL03128	4,6-Dichloro-2-(heptafluoropropyl)pyrimidine	1023815-51-1	FC(F)(F)C(F)(F)C(F)(F)c1nc(Cl)cc(Cl)n1		-6.01	0.64	1.91	P	IN
QL03129	2-(Heptafluoropropyl)-1H-indole	123825-50-3	FC(F)(F)C(F)(F)C(F)(F)c1Nc2ccccc2c1		-6.37	0.64	2.16	P	IN
QL03130	2-(Heptafluoropropyl)-3-phenylquinoxaline	69492-70-2	FC(F)(F)C(F)(F)C(F)(F)c1nc2ccccc2nc1-c1ccccc1		-5.89	0.62	2.21	P	OUT
QL03131	4-Chloro-2-(heptafluoropropyl)pyrimidine	937676-00-1	FC(F)(F)C(F)(F)C(F)(F)c1nccc(Cl)n1		-5.95	0.64	1.92	P	IN
QL03132	2-(Heptafluoropropyl)-1H-pyrrole	118425-75-5	FC(F)(F)C(F)(F)C(F)(F)c1Nccc1		-5.85	0.64	1.83	P	IN
QL03133	1-(Heptafluoropropyl)isoquinoline	70414-12-9	FC(F)(F)C(F)(F)C(F)(F)c1nccc2ccccc12		-6.06	0.63	2.13	P	OUT
QL03134	2-(Heptafluoropropyl)pyrimidine	133391-64-7	FC(F)(F)C(F)(F)C(F)(F)c1ncccn1		-5.79	0.63	1.92	P	IN
QL03135	Imidazolidine, 2-heptafluoropropyl-	31185-53-2	FC(F)(F)C(F)(F)C(F)(F)C1NCCN1		-5.85	0.64	1.83	P	IN
QL03136	2-(Heptafluoropropyl)-1H-imidazole	81769-47-3	FC(F)(F)C(F)(F)C(F)(F)c1Nccn1		-5.85	0.64	1.83	P	IN
QL03138	4-Bromo-3-(heptafluoropropyl)-5-phenyl-1H-pyrazole	82633-52-1	FC(F)(F)C(F)(F)C(F)(F)c1nNc(c1Br):c1ccccc1		-6.60	0.63	2.42	P	OUT
QL03139	5-(Heptafluoropropyl)-3-phenyl-1H-1,2,4-triazole	2195-67-7	FC(F)(F)C(F)(F)C(F)(F)c1Nnc(n1)-c1ccccc1		-6.56	0.63	2.43	P	OUT
QL03140	3-(Heptafluoropropyl)-6-(morpholin-4-yl)[1,2,4]triazolo[4,3-b]pyridazine	296886-61-8	FC(F)(F)C(F)(F)C(F)(F)c1nnc2ccc(nn12)N1CCOCC1		-6.69	0.62	2.53	P	OUT
QL03141	3-(Heptafluoropropyl)-1H-pyrazole	158148-63-1	FC(F)(F)C(F)(F)C(F)(F)c1nNcc1		-5.85	0.64	1.83	P	IN
QL03142	2(5H)-Furanone, 5-(heptafluoropropyl)-	143469-51-6	FC(F)(F)C(F)(F)C(F)(F)C1OC(=O)C=C1		-6.19	0.65	1.92	P	IN
QL03143	2-(Heptafluoropropyl)-1,3-dioxolane	109799-66-8	FC(F)(F)C(F)(F)C(F)(F)C1OCCO1		-5.85	0.64	1.83	P	IN
QL03144	Allyl heptafluorobutyrate	17165-55-8	FC(F)(F)C(F)(F)C(F)(F)CC(=O)OC=C		-5.95	0.62	2.15	P	OUT
QL03145	1,1,1,2,2,3,3,10,10,11,11,12,12,12-Tetradecafluoro-5,8-diiodododecane	2357-30-4	FC(F)(F)C(F)(F)C(F)(F)CC(I)CCC(I)CC(F)(F)C(F)(F)C(F)(F)F		-7.74	0.65	2.65	P	IN
QL03146	1,1,1,2,2,3,3,11,11,12,12,13,13,13-Tetradecafluoro-5,9-diiodotridecane	1644-06-0	FC(F)(F)C(F)(F)C(F)(F)CC(I)CCCC(I)CC(F)(F)C(F)(F)C(F)(F)F		-7.83	0.65	2.77	P	IN
QL03147	Trichloro(3,3,4,4,5,5,5-heptafluoropentyl)silane	866-24-0	FC(F)(F)C(F)(F)C(F)(F)CC[Si](Cl)(Cl)Cl		-6.45	0.66	1.91	P	IN
QL03148	3,3,4,4,5,5,5-heptafluoropentanal	126015-32-5	FC(F)(F)C(F)(F)C(F)(F)CC=O		-5.71	0.64	1.85	P	IN
QL03149	(2,2,3,3,4,4,4-Heptafluorobutyl)benzene	81664-99-5	FC(F)(F)C(F)(F)C(F)(F)Cc1ccccc1		-6.04	0.63	2.13	P	OUT
QL03150	1H,1H-Heptafluorobutyl epoxide	1765-92-0	FC(F)(F)C(F)(F)C(F)(F)CC1CO1		-6.22	0.66	1.84	P	IN
QL03151	2-(2,2,3,3,4,4,4-Heptafluorobutyl)-1,3-dioxolane	175607-87-1	FC(F)(F)C(F)(F)C(F)(F)CC1OCCO1		-5.85	0.64	1.92	P	IN
QL03152	5-Bromo-1,1,1,2,2,3,3-heptafluoropentane	355-94-2	FC(F)(F)C(F)(F)C(F)(F)CCBr		-5.71	0.64	1.85	P	IN
QL03153	1,1,1,2,2,3,3,4,4,5,5,6,6,9,9,10,10,11,11,11-Icosafluoroundecane	777931-73-4	FC(F)(F)C(F)(F)C(F)(F)CCC(F)(F)C(F)(F)C(F)(F)C(F)(F)C(F)(F)C(F)(F)F		-8.61	0.68	2.68	P	IN

QL03154	1,1,1,2,2,3,3,6,6,7,7,8,8,8-Tetradecafluorooctane	1835251-92-7	FC(F)(F)C(F)(F)C(F)(F)CCC(F)(F)C(F)(F)C(F)(F)F		-7.41	0.68	2.16	P	IN
QL03155	7-Bromo-1,1,1,2,2,3,3-heptafluoroheptane	1391033-22-9	FC(F)(F)C(F)(F)C(F)(F)CCCCBr		-5.83	0.62	2.17	P	OUT
QL03156	1,1,1,2,2,3,3,10,10,11,11,12,12,12-Tetradecafluorododecane	69125-82-2	FC(F)(F)C(F)(F)C(F)(F)CCCCCCC(F)(F)C(F)(F)C(F)(F)F		-7.53	0.65	2.60	P	IN
QL03157	1,1,1,2,2,3,3,12,12,13,13,14,14,14-Tetradecafluorotetradecane	1835251-91-6	FC(F)(F)C(F)(F)C(F)(F)CCCCCCCCC(F)(F)C(F)(F)C(F)(F)F		-7.59	0.64	2.79	P	OUT
QL03158	1,1,1,2,2,3,3,14,14,15,15,16,16,16-Tetradecafluorohexadecane	168169-26-4	FC(F)(F)C(F)(F)C(F)(F)CCCCCCCCCCC(F)(F)C(F)(F)C(F)(F)F		-7.67	0.63	2.96	P	OUT
QL03159	1,1,1,2,2,3,3-Heptafluoro-7-iodoheptane	2967-60-4	FC(F)(F)C(F)(F)C(F)(F)CCCCI		-5.83	0.62	2.17	P	OUT
QL03160	1,1,1,2,2,3,3-Heptafluoro-5-iodopentane	68188-12-5	FC(F)(F)C(F)(F)C(F)(F)CCI		-5.71	0.64	1.85	P	IN
QL03161	1,1,1,2,2,3,3-Heptafluoro-4-chlorobutane	374-97-0	FC(F)(F)C(F)(F)C(F)(F)CCl		-5.48	0.66	1.46	P	IN
QL03162	Heptafluorobutyl iodide	374-98-1	FC(F)(F)C(F)(F)C(F)(F)CI		-5.48	0.66	1.46	P	IN
QL03163	1-chloro-1,1,2,2,3,3,3-heptafluoropropane	422-86-6	FC(F)(F)C(F)(F)C(F)(F)Cl		-5.80	0.68	1.32	P	IN
QL03164	N-Nitrosobis(2,2,3,3,4,4,4-heptafluorobutyl)amine	83329-16-2	FC(F)(F)C(F)(F)C(F)(F)CN(CC(F)(F)C(F)(F)C(F)(F)F)N=O		-7.40	0.66	2.40	P	IN
QL03165	Isocyanic acid, 2,2,3,3,4,4,4-heptafluorobutyl ester	377-00-4	FC(F)(F)C(F)(F)C(F)(F)CN=C=O		-5.63	0.63	1.95	P	OUT
QL03166	N-(2,2,3,3,4,4,4-Heptafluorobutyl)prop-2-enamide	1422-65-7	FC(F)(F)C(F)(F)C(F)(F)CNC(=O)C=C		-6.15	0.63	2.15	P	OUT
QL03167	3-Benzamido-N-(2,2,3,3,4,4,4-heptafluorobutyl)benzamide	917920-92-4	FC(F)(F)C(F)(F)C(F)(F)CNC(=O)C1=CC(NC(=O)C2=CC=CC=C2)=CC=C1		-7.13	0.61	2.93	P	OUT
QL03168	N-(2,2,3,3,4,4,4-Heptafluorobutyl)benzamide	304458-19-3	FC(F)(F)C(F)(F)C(F)(F)CNC(=O)c1ccccc1		-6.47	0.62	2.42	P	OUT
QL03169	2,2,3,3,4,4,4-Heptafluoro-N-(2,2,3,3,4,4,4-heptafluorobutyl)butan-1-amine	356-08-1	FC(F)(F)C(F)(F)C(F)(F)CNCC(F)(F)C(F)(F)C(F)(F)F		-7.52	0.67	2.33	P	IN
QL03170	Tris(1H,1H-heptafluorobutyl)phosphate	563-09-7	FC(F)(F)C(F)(F)C(F)(F)CO[P](=O)(OCC(F)(F)C(F)(F)C(F)(F)F)OCC(F)(F)C(F)(F)C(F)(F)F		-7.99	0.67	2.53	P	IN
QL03171	Chlorotris(2,2,3,3,4,4,4-heptafluorobutoxy)silane	757-72-2	FC(F)(F)C(F)(F)C(F)(F)CO[Si](Cl)(OCC(F)(F)C(F)(F)C(F)(F)F)OCC(F)(F)C(F)(F)C(F)(F)F		-7.99	0.67	2.53	P	IN
QL03172	tris((Perfluoropropyl)methyl) borate	755-53-3	FC(F)(F)C(F)(F)C(F)(F)COB(OCC(F)(F)C(F)(F)C(F)(F)F)OCC(F)(F)C(F)(F)C(F)(F)F		-7.79	0.67	2.51	P	IN
QL03173	2,2,3,3,4,4,4-Heptafluorobutyl 2-cyanoprop-2-enoate	130192-28-8	FC(F)(F)C(F)(F)C(F)(F)COC(=O)C(=C)C#N		-6.28	0.63	2.21	P	OUT
QL03174	2,2,3,3,4,4,4-Heptafluorobutyl 2-chloroprop-2-enoate	74359-14-1	FC(F)(F)C(F)(F)C(F)(F)COC(=O)C(Cl)=C		-6.45	0.64	2.13	P	IN
QL03175	2,2,3,3,4,4,4-Heptafluorobutyl heptafluorobutanoate	308-45-2	FC(F)(F)C(F)(F)C(F)(F)COC(=O)C(F)(F)C(F)(F)C(F)(F)F		-7.66	0.67	2.36	P	IN
QL03176	1H,1H-Perfluorobutyl acrylate	424-64-6	FC(F)(F)C(F)(F)C(F)(F)COC(=O)C=C		-6.15	0.63	2.15	P	OUT
QL03177	Bis(1H,1H-perfluorobutyl)fumarate	24120-17-0	FC(F)(F)C(F)(F)C(F)(F)COC(=O)C=CC(=O)OCC(F)(F)C(F)(F)C(F)(F)F		-7.89	0.65	2.82	P	IN
QL03178	2,2,3,3,4,4,4-Heptafluorobutyl 1,2,3-benzothiadiazole-7-carboxylate	1384278-50-5	FC(F)(F)C(F)(F)C(F)(F)COC(=O)c1cccc2nnsc12		-6.47	0.63	2.38	P	OUT
QL03179	2,2,3,3,4,4,4-Heptafluorobutyl but-3-enoate	17165-56-9	FC(F)(F)C(F)(F)C(F)(F)COC(=O)CC=C		-5.98	0.62	2.23	P	OUT
QL03180	1-Aziridinepropionic acid, 2,2,3,3,4,4,4-heptafluorobutyl ester	24160-20-1	FC(F)(F)C(F)(F)C(F)(F)COC(=O)CCN1CC1		-6.72	0.63	2.44	P	OUT
QL03181	Bis(2,2,3,3,4,4,4-heptafluorobutyl) carbonate	1026739-75-2	FC(F)(F)C(F)(F)C(F)(F)COC(=O)OCC(F)(F)C(F)(F)C(F)(F)F		-7.75	0.66	2.57	P	IN
QL03182	2,2,3,3,4,4,4-Heptafluorobutyl carbonochloridate	108156-21-4	FC(F)(F)C(F)(F)C(F)(F)COC(Cl)=O		-6.00	0.64	1.93	P	IN
QL03183	4-(Ethenyloxy)-1,1,1,2,2,3,3-heptafluorobutane	356-00-3	FC(F)(F)C(F)(F)C(F)(F)COC=C		-5.63	0.63	1.95	P	OUT
QL03184	1-Ethenyl-4-(2,2,3,3,4,4,4-heptafluorobutoxy)benzene	111144-99-1	FC(F)(F)C(F)(F)C(F)(F)COC1=CC=C(C=C)C=C1		-6.66	0.62	2.55	P	OUT
QL03185	2-{[4-(Perfluorobutoxy)pyridin-2-yl]methanesulfinyl}-1H-thieno[3,4-d]imidazole	NOCAS_869668	FC(F)(F)C(F)(F)C(F)(F)COC1=CC=NC(CS(=O)C2=NC3=CSC=C3N2)=C1		-7.39	0.62	2.94	P	OUT
QL03186	4-(2,2,3,3,4,4,4-Heptafluorobutoxy)benzaldehyde	943767-44-0	FC(F)(F)C(F)(F)C(F)(F)COc1ccc(C=O)cc1		-6.66	0.62	2.55	P	OUT
QL03187	4-(2,2,3,3,4,4,4-Heptafluorobutoxy)benzonitrile	96308-32-6	FC(F)(F)C(F)(F)C(F)(F)COc1ccc(cc1)C#N		-6.66	0.62	2.55	P	OUT
QL03188	1,1,1,2,2,3,3-Heptafluoro-4-(2,2,3,3,4,4,4-heptafluorobutoxy)butane	647-91-6	FC(F)(F)C(F)(F)C(F)(F)COCC(F)(F)C(F)(F)C(F)(F)F		-7.52	0.67	2.33	P	IN
QL03189	Allyl 1H,1H-heptafluorobutyl ether	648-42-0	FC(F)(F)C(F)(F)C(F)(F)COCC=C		-5.83	0.62	2.17	P	OUT
QL03190	NSC221106	59727-25-2	FC(F)(F)C(F)(F)C(F)(F)COCC1=CNC(=O)NC1=O		-6.72	0.63	2.47	P	OUT
QL03191	2-[(2,2,3,3,4,4,4-Heptafluorobutoxy)methyl]oxirane	648-43-1	FC(F)(F)C(F)(F)C(F)(F)COCC1CO1		-6.38	0.64	2.15	P	IN
QL03192	4-[(2,2,3,3,4,4,4-Heptafluorobutoxy)methyl]-1,3-dioxolan-2-one	181044-06-4	FC(F)(F)C(F)(F)C(F)(F)COCC1COC(=O)O1		-6.59	0.63	2.39	P	OUT
QL03193	4-(2-Chloroethoxy)-1,1,1,2,2,3,3-heptafluorobutane	679-53-8	FC(F)(F)C(F)(F)C(F)(F)COCCCl		-5.83	0.62	2.17	P	OUT

QL03194	2,2,3,3,4,4,4-Heptafluorobutyl diphenylphosphinate	55776-72-2	FC(F)(F)C(F)(F)C(F)(F)COP(=O)(c1ccccc1)c1ccccc1		-6.17	0.62	2.36	P	OUT
QL03195	Bis(2,2,3,3,4,4,4-heptafluorobutyl) phenylphosphonate	31430-72-5	FC(F)(F)C(F)(F)C(F)(F)COP(=O)(OCC(F)(F)C(F)(F)C(F)(F)F)c1ccccc1		-7.66	0.65	2.67	P	IN
QL03196	bis((Perfluoropropyl)methyl) phosphorochloridoate	6780-81-0	FC(F)(F)C(F)(F)C(F)(F)COP(Cl)(=O)OCC(F)(F)C(F)(F)C(F)(F)F		-8.02	0.67	2.59	P	IN
QL03197	1,3,5,2,4,6-Triazatriphosphorine, 2,2,4,4,6,6-hexakis(2,2,3,3,4,4,4-heptafluorobutoxy)-2,2,4,4,6,6-hexahydro-	470-73-5	FC(F)(F)C(F)(F)C(F)(F)COP1(OCC(F)(F)C(F)(F)C(F)(F)F)=NP(OCC(F)(F)C(F)(F)C(F)(F)F)(OCC(F)(F)C(F)(F)C(F)(F)F)=NP(OCC(F)(F)C(F)(F)C(F)(F)F)(OCC(F)(F)C(F)(F)C(F)(F)F)=N1		-8.09	0.67	2.57	P	IN
QL03198	1H,1H-Perfluorobutyl perfluorobutanesulfonate	883499-32-9	FC(F)(F)C(F)(F)C(F)(F)COS(=O)(=O)C(F)(F)C(F)(F)C(F)(F)C(F)(F)F		-8.22	0.68	2.50	P	IN
QL03199	1H,1H-Heptafluorobutyl triflate	6401-01-0	FC(F)(F)C(F)(F)C(F)(F)COS(=O)(=O)C(F)(F)F		-7.17	0.67	2.09	P	IN
QL03200	{[(2,2,3,3,4,4,4-Heptafluorobutyl)sulfanyl]methyl}benzene	200337-06-0	FC(F)(F)C(F)(F)C(F)(F)CSCc1ccccc1		-6.25	0.62	2.39	P	OUT
QL03201	Perflutren	76-19-7	FC(F)(F)C(F)(F)C(F)(F)F		-5.80	0.68	1.32	P	IN
QL03202	Heptafluoropropyl iodide	754-34-7	FC(F)(F)C(F)(F)C(F)(F)I		-5.80	0.68	1.32	P	IN
QL03203	Perfluoro-N,N,N',N'-tetrakis(heptafluoropropyl)-1,6-hexanediamine	143356-32-5	FC(F)(F)C(F)(F)C(F)(F)N(C(F)(F)C(F)(F)C(F)(F)F)C(F)(F)C(F)(F)C(F)(F)C(F)(F)C(F)(F)C(F)(F)N(C(F)(F)C(F)(F)C(F)(F)F)C(F)(F)C(F)(F)C(F)(F)F		-9.03	0.69	2.81	P	OUT
QL03204	Perfluamine	338-83-0	FC(F)(F)C(F)(F)C(F)(F)N(C(F)(F)C(F)(F)C(F)(F)F)C(F)(F)C(F)(F)C(F)(F)F		-7.41	0.69	2.04	P	IN
QL03205	1,1,1,2,4,5,5,5-Octafluoro-2-(heptafluoropropoxy)-4-(trifluoromethyl)pentan-3-one	851535-00-7	FC(F)(F)C(F)(F)C(F)(F)OC(F)(C(=O)C(F)(C(F)(F)F)C(F)(F)F)C(F)(F)F		-7.46	0.68	2.17	P	IN
QL03206	1,5-Dideoxy-1,1,1,5,5,5-hexafluoro-2,4-di-C-fluoro-2,4-bis-O-(heptafluoropropyl)pent-3-ulose	67118-49-4	FC(F)(F)C(F)(F)C(F)(F)OC(F)(C(=O)C(F)(OC(F)(F)C(F)(F)C(F)(F)F)C(F)(F)F)C(F)(F)F		-8.42	0.68	2.65	P	IN
QL03207	1,1,1,2,6,7,7,7-Octafluoro-2,6-bis(heptafluoropropoxy)heptane-3,5-dione	87405-74-1	FC(F)(F)C(F)(F)C(F)(F)OC(F)(C(=O)CC(=O)C(F)(OC(F)(F)C(F)(F)C(F)(F)F)C(F)(F)F)C(F)(F)F		-8.61	0.67	2.82	P	IN
QL03208	2,3,3,3-Tetrafluoro-2-(heptafluoropropoxy)-1-(piperidin-1-yl)propan-1-one	176702-72-0	FC(F)(F)C(F)(F)C(F)(F)OC(F)(C(=O)N1CCCCC1)C(F)(F)F		-7.10	0.64	2.45	P	IN
QL03209	2,3,3,3-Tetrafluoro-2-(heptafluoropropoxy)-1-(morpholin-4-yl)propan-1-one	176702-73-1	FC(F)(F)C(F)(F)C(F)(F)OC(F)(C(=O)N1CCOCC1)C(F)(F)F		-7.10	0.64	2.45	P	IN
QL03210	N-Benzyl-2,3,3,3-tetrafluoro-2-(heptafluoropropoxy)propanamide	82970-41-0	FC(F)(F)C(F)(F)C(F)(F)OC(F)(C(=O)NCc1ccccc1)C(F)(F)F		-7.36	0.64	2.70	P	OUT
QL03211	Cyclohexyl 2,3,3,3-tetrafluoro-2-(heptafluoropropoxy)propanoate	78755-41-6	FC(F)(F)C(F)(F)C(F)(F)OC(F)(C(=O)OC1CCCCC1)C(F)(F)F		-7.14	0.64	2.53	P	IN
QL03212	Phenyl 2,3,3,3-tetrafluoro-2-(heptafluoropropoxy)propanoate	78755-31-4	FC(F)(F)C(F)(F)C(F)(F)OC(F)(C(=O)Oc1ccccc1)C(F)(F)F		-7.14	0.64	2.53	P	IN
QL03213	Prop-2-en-1-yl 2,3,3,3-tetrafluoro-2-(heptafluoropropoxy)propanoate	84123-65-9	FC(F)(F)C(F)(F)C(F)(F)OC(F)(C(=O)OCC=C)C(F)(F)F		-6.99	0.64	2.46	P	IN
QL03214	Benzyl 2,3,3,3-tetrafluoro-2-(heptafluoropropoxy)propanoate	147224-19-9	FC(F)(F)C(F)(F)C(F)(F)OC(F)(C(=O)OCc1ccccc1)C(F)(F)F		-7.36	0.64	2.70	P	OUT
QL03215	bis(2,3,3,3-tetrafluoro-2-(heptafluoropropoxy)-1-oxopropyl)peroxide	56347-79-6	FC(F)(F)C(F)(F)C(F)(F)OC(F)(C(=O)OOC(=O)C(F)(OC(F)(F)C(F)(F)C(F)(F)F)C(F)(F)F)C(F)(F)F		-8.60	0.67	2.87	P	IN
QL03216	2,3,3,3-Tetrafluoro-2-(heptafluoropropoxy)propanoyl chloride	72848-57-8	FC(F)(F)C(F)(F)C(F)(F)OC(F)(C(Cl)=O)C(F)(F)F		-6.59	0.67	1.85	P	IN
QL03217	Tris{4-[1,1,2,3,3,3-hexafluoro-2-(heptafluoropropoxy)propyl]phenyl}phosphane	62512-90-7	FC(F)(F)C(F)(F)C(F)(F)OC(F)(C(F)(F)F)C(F)(F)C1=CC=C(C=C1)P(C1=CC=C(C=C1)C(F)(F)C(F)(OC(F)(F)C(F)(F)C(F)(F)F)C(F)(F)F)C1=CC=C(C=C1)C(F)(F)C(F)(OC(F)(F)C(F)(F)C(F)(F)F)C(F)(F)F		-9.42	0.66	3.38	P	OUT
QL03218	1-{[1-(1,2-Dibromo-1,2,2-trifluoroethoxy)-1,1,2,3,3,3-hexafluoropropan-2-yl]oxy}-1,1,2,2,3,3,3-heptafluoropropane	115783-72-7	FC(F)(F)C(F)(F)C(F)(F)OC(F)(C(F)(F)F)C(F)(F)OC(F)(Br)C(F)(F)Br		-8.00	0.68	2.43	P	IN
QL03219	1-(4-Bromophenyl)-2,3,3,3-tetrafluoro-2-[1,1,2,3,3,3-hexafluoro-2-(heptafluoropropoxy)propoxy]propan-1-one	67727-74-6	FC(F)(F)C(F)(F)C(F)(F)OC(F)(C(F)(F)F)C(F)(F)OC(F)(C(=O)C1=CC=C(Br)C=C1)C(F)(F)F		-8.19	0.66	2.82	P	IN
QL03220	2,3,3,3-Tetrafluoro-2-[1,1,2,3,3,3-hexafluoro-2-(heptafluoropropoxy)propoxy]-1-(piperidin-1-yl)propan-1-one	176702-75-3	FC(F)(F)C(F)(F)C(F)(F)OC(F)(C(F)(F)F)C(F)(F)OC(F)(C(=O)N1CCCCC1)C(F)(F)F		-7.86	0.66	2.69	P	IN
QL03221	2,3,3,3-Tetrafluoro-2-[1,1,2,3,3,3-hexafluoro-2-(heptafluoropropoxy)propoxy]-1-(morpholin-4-yl)propan-1-one	176702-76-4	FC(F)(F)C(F)(F)C(F)(F)OC(F)(C(F)(F)F)C(F)(F)OC(F)(C(=O)N1CCOCC1)C(F)(F)F		-7.86	0.66	2.69	P	IN
QL03222	N-2-Pyridinylperfluoro-2-(2-propoxypropoxy)propanamide	31339-60-3	FC(F)(F)C(F)(F)C(F)(F)OC(F)(C(F)(F)F)C(F)(F)OC(F)(C(=O)NC1=CC=CC=N1)C(F)(F)F		-8.03	0.65	2.83	P	IN
QL03223	N-2-Pyrimidinylperfluoro-2-(2-propoxypropoxy)propanamide	340131-92-2	FC(F)(F)C(F)(F)C(F)(F)OC(F)(C(F)(F)F)C(F)(F)OC(F)(C(=O)NC1=NC=CC=N1)C(F)(F)F		-8.03	0.65	2.83	P	IN

QL03224	2,3,3,3-Tetrafluoro-2-[1,1,2,3,3,3-hexafluoro-2-(heptafluoropropoxy)propoxy]-N-phenylpropanamide	178167-74-3	FC(F)(F)C(F)(F)C(F)(F)OC(F)(C(F)(F)F)C(F)(F)OC(F)(C(=O)Nc1ccccc1)C(F)(F)F		-8.03	0.65	2.83	P	IN
QL03225	N-Allylperfluoro-2-(2-propoxypropoxy)propanamide	174080-50-3	FC(F)(F)C(F)(F)C(F)(F)OC(F)(C(F)(F)F)C(F)(F)OC(F)(C(=O)NCC=C)C(F)(F)F		-7.76	0.65	2.68	P	IN
QL03226	Cyclohexyl 2,3,3,3-tetrafluoro-2-[1,1,2,3,3,3-hexafluoro-2-(heptafluoropropoxy)propoxy]propanoate	145510-90-3	FC(F)(F)C(F)(F)C(F)(F)OC(F)(C(F)(F)F)C(F)(F)OC(F)(C(=O)OC1CCCCC1)C(F)(F)F		-8.03	0.65	2.83	P	IN
QL03227	Phenyl 2,3,3,3-tetrafluoro-2-[1,1,2,3,3,3-hexafluoro-2-(heptafluoropropoxy)propoxy]propanoate	312943-34-3	FC(F)(F)C(F)(F)C(F)(F)OC(F)(C(F)(F)F)C(F)(F)OC(F)(C(=O)Oc1ccccc1)C(F)(F)F		-8.03	0.65	2.83	P	IN
QL03228	Prop-2-en-1-yl 2,3,3,3-tetrafluoro-2-[1,1,2,3,3,3-hexafluoro-2-(heptafluoropropoxy)propoxy]propanoate	117837-58-8	FC(F)(F)C(F)(F)C(F)(F)OC(F)(C(F)(F)F)C(F)(F)OC(F)(C(=O)OCC=C)C(F)(F)F		-7.76	0.65	2.68	P	IN
QL03229	Benzyl 2,3,3,3-tetrafluoro-2-[1,1,2,3,3,3-hexafluoro-2-(heptafluoropropoxy)propoxy]propanoate	140229-81-8	FC(F)(F)C(F)(F)C(F)(F)OC(F)(C(F)(F)F)C(F)(F)OC(F)(C(=O)OCc1ccccc1)C(F)(F)F		-8.12	0.65	2.92	P	IN
QL03230	perfluoro(5,6,9,12-tetramethyl-4,7,10,13-tetraoxahexadecane)		FC(F)(F)C(F)(F)C(F)(F)OC(F)(C(F)(F)F)C(F)(F)OC(F)(C(F)(F)F)C(F)(F)OC(F)(C(F)(F)F)C(F)(OC(F)(F)C(F)(F)C(F)(F)F)C(F)(F)F		-9.37	0.68	3.09	P	OUT
QL03231	1,1,1,2,2,3,3,5,6,6,8,9,9,11,12,14,14,15,17,17,18,20,20,21,21,22,22,22-Octacosafluoro-5,8,11,12,15,18-hexakis(trifluoromethyl)-4,7,10,13,16,19-hexaoxadocosane	61098-01-9	FC(F)(F)C(F)(F)C(F)(F)OC(F)(C(F)(F)F)C(F)(F)OC(F)(C(F)(F)F)C(F)(F)OC(F)(C(F)(F)F)C(F)(OC(F)(F)C(F)(OC(F)(F)C(F)(OC(F)(F)C(F)(F)C(F)(F)F)C(F)(F)F)C(F)(F)F)C(F)(F)F		-10.23	0.68	3.53	P	OUT
QL03232	perfluoro(5,8,9,12-tetramethyl-4,7,10,13-tetraoxahexadecane)		FC(F)(F)C(F)(F)C(F)(F)OC(F)(C(F)(F)F)C(F)(F)OC(F)(C(F)(F)F)C(F)(OC(F)(F)C(F)(OC(F)(F)C(F)(F)C(F)(F)F)C(F)(F)F)C(F)(F)F		-9.39	0.68	3.11	P	OUT
QL03233	2-{1,2,2,2-Tetrafluoro-1-[1,1,2,3,3,3-hexafluoro-2-(heptafluoropropoxy)propoxy]ethyl}-1H-benzimidazole	76145-90-9	FC(F)(F)C(F)(F)C(F)(F)OC(F)(C(F)(F)F)C(F)(F)OC(F)(C1=NC2=CC=CC=C2N1)C(F)(F)F		-7.94	0.66	2.70	P	IN
QL03234	2,3,3,3-Tetrafluoro-2-[1,1,2,3,3,3-hexafluoro-2-(heptafluoropropoxy)propoxy]propyl prop-2-enoate	17559-01-2	FC(F)(F)C(F)(F)C(F)(F)OC(F)(C(F)(F)F)C(F)(F)OC(F)(COC(=O)C=C)C(F)(F)F		-7.92	0.66	2.71	P	IN
QL03235	Perfluoro-5,6-dimethyl-4,7-dioxadecane	2501-01-1	FC(F)(F)C(F)(F)C(F)(F)OC(F)(C(F)(F)F)C(F)(OC(F)(F)C(F)(F)C(F)(F)F)C(F)(F)F		-8.21	0.68	2.50	P	IN
QL03236	1,1,1,3,4,4,4-Heptafluoro-3-(heptafluoropropoxy)-2-(trifluoromethyl)butan-2-yl prop-2-enoate	1622868-66-9	FC(F)(F)C(F)(F)C(F)(F)OC(F)(C(F)(F)F)C(OC(=O)C=C)(C(F)(F)F)C(F)(F)F		-7.32	0.66	2.36	P	IN
QL03237	2,3,3,3-Tetrafluoro-2-(heptafluoropropoxy)propanenitrile	60308-66-9	FC(F)(F)C(F)(F)C(F)(F)OC(F)(C#N)C(F)(F)F		-6.48	0.66	1.87	P	IN
QL03238	3,4,4,4-Tetrafluoro-3-(heptafluoropropoxy)but-1-ene	131628-34-7	FC(F)(F)C(F)(F)C(F)(F)OC(F)(C=C)C(F)(F)F		-6.48	0.66	1.87	P	IN
QL03239	1,1,1,2-Tetrafluoro-2-(heptafluoropropoxy)-4-iodobutane	243128-38-3	FC(F)(F)C(F)(F)C(F)(F)OC(F)(CCI)C(F)(F)F		-6.50	0.65	2.06	P	IN
QL03240	1,2-Dichloro-1,2,2-trifluoroethyl heptafluoropropyl ether	106372-30-9	FC(F)(F)C(F)(F)C(F)(F)OC(F)(Cl)C(F)(F)Cl		-6.92	0.68	1.89	P	IN
QL03241	3-[2,3,3,3-Tetrafluoro-2-(heptafluoropropoxy)propoxy]prop-1-ene	119511-16-9	FC(F)(F)C(F)(F)C(F)(F)OC(F)(COCC=C)C(F)(F)F		-6.93	0.64	2.48	P	IN
QL03242	(R*,S*)-1,1,2,3,4,4-Hexafluoro-1,2,3,4-tetrakis(heptafluoropropoxy)butane	54572-17-7	FC(F)(F)C(F)(F)C(F)(F)OC(F)(F)[C@@](F)(OC(F)(F)C(F)(F)C(F)(F)F)[C@@](F)(OC(F)(F)C(F)(F)C(F)(F)F)C(F)(F)OC(F)(F)C(F)(F)C(F)(F)F		-8.56	0.68	2.66	P	IN
QL03243	3,3,4,4-Tetrafluoro-4-(heptafluoropropoxy)but-1-ene	1152098-94-6	FC(F)(F)C(F)(F)C(F)(F)OC(F)(F)C(F)(F)C=C		-6.93	0.66	2.09	P	IN
QL03244	1,1,2,3,4,4-Hexafluoro-1,2,3,4-tetrakis(heptafluoropropoxy)butane	54208-66-1	FC(F)(F)C(F)(F)C(F)(F)OC(F)(F)C(F)(OC(F)(F)C(F)(F)C(F)(F)F)C(F)(OC(F)(F)C(F)(F)C(F)(F)F)C(F)(F)OC(F)(F)C(F)(F)C(F)(F)F		-8.56	0.68	2.66	P	IN
QL03245	1,1,1,2,2,3,3-Heptafluoro-3-(1,2,2,2-tetrafluoro-1-iodoethoxy)propane	107432-46-2	FC(F)(F)C(F)(F)C(F)(F)OC(F)(I)C(F)(F)F		-6.92	0.68	1.89	P	IN
QL03246	1,1,2,2,3,3,3-heptafluoro-1-(vinyloxy)propane	6996-01-6	FC(F)(F)C(F)(F)C(F)(F)OC=C		-5.71	0.64	1.85	P	IN
QL03247	Perfluoro-1-ethyl-3-propoxycyclohexane	400626-82-6	FC(F)(F)C(F)(F)C(F)(F)OC1(F)C(F)(F)C(F)(F)C(F)(F)C(F)(C(F)(F)C(F)(F)F)C1(F)F		-8.31	0.69	2.41	P	IN
QL03248	Perfluoro(1,3-dipropoxycyclohexane)	400626-83-7	FC(F)(F)C(F)(F)C(F)(F)OC1(F)C(F)(F)C(F)(F)C(F)(F)C(F)(OC(F)(F)C(F)(F)C(F)(F)F)C1(F)F		-8.79	0.69	2.68	P	OUT
QL03249	2-Iodoperfluorobutane	375-51-9	FC(F)(F)C(F)(F)C(F)(I)C(F)(F)F		-6.18	0.68	1.45	P	IN
QL03250	1,1,1,2,2,5,5,6,6,7,7,8,8,9,9,10,10,10-Octadecafluorodec-3-ene	86563-87-3	FC(F)(F)C(F)(F)C=CC(F)(F)C(F)(F)C(F)(F)C(F)(F)C(F)(F)C(F)(F)F		-8.22	0.68	2.50	P	IN
QL03251	1,1,1,2,2,5,5,6,6,7,7,8,8,9,9,9-Hexadecafluoronon-3-ene	935477-04-6	FC(F)(F)C(F)(F)C=CC(F)(F)C(F)(F)C(F)(F)C(F)(F)C(F)(F)F		-8.04	0.68	2.44	P	IN
QL03252	1,1,1,2,2,5,5,6,6,7,7,8,8,8-Tetradecafluorooct-3-ene	84808-65-1	FC(F)(F)C(F)(F)C=CC(F)(F)C(F)(F)C(F)(F)C(F)(F)F		-7.55	0.68	2.23	P	IN
QL03253	1,1,1,2,2,5,5,6,6,7,7,7-Dodecafluorohept-3-ene	887111-61-7	FC(F)(F)C(F)(F)C=CC(F)(F)C(F)(F)C(F)(F)F		-7.17	0.67	2.09	P	IN

QL03254	Perfluoro-5-(pentafluoroethyl)oxolane	NOCAS_870516	FC(F)(F)C(F)(F)C1(F)OC(F)(F)C(F)(F)C1(F)F		-6.99	0.69	1.76	P	IN
QL03255	2-(1,1,1,2,3,3,3-Heptafluoropropan-2-yl)-2-(pentafluoroethyl)-3,3-bis(trifluoromethyl)oxirane	103697-21-8	FC(F)(F)C(F)(F)C1(OC1(C(F)(F)F)C(F)(F)F)C(F)(C(F)(F)F)C(F)(F)F		-6.42	0.68	1.57	P	IN
QL03256	2,4,6-Tris(pentafluoroethyl)-1,3,5-triazine	858-46-8	FC(F)(F)C(F)(F)C1=NC(=NC(=N1)C(F)(F)C(F)(F)F)C(F)(F)C(F)(F)F		-6.68	0.67	1.89	P	IN
QL03257	1,1,1,2,2,3,3,4,4,5,5,6,6,7,7,8,8,11,11,12,12,12-Docosafluoro-9-iodododecane	641617-25-6	FC(F)(F)C(F)(F)CC(I)C(F)(F)C(F)(F)C(F)(F)C(F)(F)C(F)(F)C(F)(F)C(F)(F)C(F)(F)F		-8.76	0.68	2.74	P	IN
QL03258	1,1,1,2,2,3,3,4,4,7,7,8,8,8-Tetradecafluoro-5-iodooctane	935553-89-2	FC(F)(F)C(F)(F)CC(I)C(F)(F)C(F)(F)C(F)(F)C(F)(F)F		-7.55	0.68	2.24	P	IN
QL03259	Bis(3-(perfluoroethyl)-1-(9-bromononyl)propyl) thioether	148757-89-5	FC(F)(F)C(F)(F)CCC(CCCCCCCCCBr)SC(CCCCCCCCCBr)CCC(F)(F)C(F)(F)F		-6.75	0.57	3.31	P	OUT
QL03260	1,1,1,2,2,3,3,4,4,7,7,8,8,8-Tetradecafluorooctane	343627-66-7	FC(F)(F)C(F)(F)CCC(F)(F)C(F)(F)C(F)(F)C(F)(F)F		-7.55	0.68	2.23	P	IN
QL03261	22 Fluorotelomer iodide	40723-80-6	FC(F)(F)C(F)(F)CCI		-4.54	0.62	1.52	P	OUT
QL03262	Thiols, C4-10, .gamma.-.omega.-perfluoro- (Perfluoroalkyl (C2-C8)ethyl mercaptan)	68140-18-1	FC(F)(F)C(F)(F)CCS		-4.54	0.62	1.52	P	OUT
QL03263	(Perfluoroethyl)methyl iodide	354-69-8	FC(F)(F)C(F)(F)CI		-4.95	0.64	1.39	P	IN
QL03264	1H,1H-Perfluoropropyl acrylate	356-86-5	FC(F)(F)C(F)(F)COC(=O)C=C		-5.20	0.62	1.88	P	OUT
QL03265	2,2,3,3,3-Pentafluoropropyl nonafluorobutane-1-sulfonate	893556-35-9	FC(F)(F)C(F)(F)COS(=O)(=O)C(F)(F)C(F)(F)C(F)(F)C(F)(F)F		-8.04	0.68	2.44	P	IN
QL03266	Perfluoroethane	76-16-4	FC(F)(F)C(F)(F)F		-4.57	0.67	0.81	P	OUT
QL03267	Pentafluoroiodoethane	354-64-3	FC(F)(F)C(F)(F)I		-4.57	0.67	0.81	P	OUT
QL03268	Perfluorotriethylamine	359-70-6	FC(F)(F)C(F)(F)N(C(F)(F)C(F)(F)F)C(F)(F)C(F)(F)F		-6.49	0.68	1.65	P	IN
QL03269	Morpholine, 2,2,3,3,5,5,6,6-octafluoro-4-(pentafluoroethyl)-	55716-11-5	FC(F)(F)C(F)(F)N1C(F)(F)C(F)(F)OC(F)(F)C1(F)F		-6.61	0.68	1.66	P	IN
QL03270	1,1,1,2,2,3,3,4,5,5,5-Undecafluoro-4-(pentafluoroethoxy)pentane	92978-07-9	FC(F)(F)C(F)(F)OC(F)(C(F)(F)F)C(F)(F)C(F)(F)C(F)(F)F		-7.52	0.68	2.14	P	IN
QL03271	Hexane, 1,1,2,2,3,3,4,4,5,5,6,6,6-tridecafluoro-1-(pentafluoroethoxy)-	133080-89-4	FC(F)(F)C(F)(F)OC(F)(F)C(F)(F)C(F)(F)C(F)(F)C(F)(F)C(F)(F)F		-8.19	0.69	2.44	P	IN
QL03272	POLYFLGSID_880513	60164-51-4	FC(F)(F)C(F)(F)OC(F)(F)C(F)(F)C(F)(F)F		-6.92	0.68	1.89	P	IN
QL03273	alpha-Perfluoropropyl omega-perfluoroethyl poly(perfluoropropylene oxide)	52700-35-3	FC(F)(F)C(F)(F)OC(F)(F)C(F)(F)C(F)(F)OC(F)(F)C(F)(F)C(F)(F)F |lp:0:3,2:3,3:3,5:3,6:3,7:2,9:3,10:3,12:3,13:3,15:3,16:3,17:2,19:3,20:3,22:3,23:3,25:3,26:3,27:3,Sg:n:10,9,13,12,16,15,8,11,14,17::ht|		-8.18	0.68	2.50	P	IN
QL03274	Perfluoroethyl ether	358-21-4	FC(F)(F)C(F)(F)OC(F)(F)C(F)(F)F		-6.44	0.67	1.71	P	IN
QL03275	multiple ether-substituted Perfluoroalkyl (linear) sulfonic acids, n=8		FC(F)(F)C(F)(F)OC(F)(F)C(F)(F)OC(F)(F)C(F)(F)C(F)(F)C(F)(F)C(F)(F)C(F)(F)C(F)(F)C(F)(F)S(=O)(O)=O		-9.47	0.69	3.06	P	OUT
QL03276	multiple ether-substituted Perfluoroalkyl (linear) sulfonic acids, n=7		FC(F)(F)C(F)(F)OC(F)(F)C(F)(F)OC(F)(F)C(F)(F)C(F)(F)C(F)(F)C(F)(F)C(F)(F)C(F)(F)S(=O)(O)=O		-9.18	0.69	2.93	P	OUT
QL03277	multiple ether-substituted Perfluoroalkyl (linear) sulfonic acids, n=6		FC(F)(F)C(F)(F)OC(F)(F)C(F)(F)OC(F)(F)C(F)(F)C(F)(F)C(F)(F)C(F)(F)C(F)(F)S(=O)(O)=O		-9.06	0.69	2.88	P	OUT
QL03278	multiple ether-substituted Perfluoroalkyl (linear) sulfonic acids, n=5		FC(F)(F)C(F)(F)OC(F)(F)C(F)(F)OC(F)(F)C(F)(F)C(F)(F)C(F)(F)C(F)(F)S(=O)(O)=O		-8.73	0.68	2.73	P	IN
QL03279	multiple ether-substituted Perfluoroalkyl (linear) sulfonic acids, n=4		FC(F)(F)C(F)(F)OC(F)(F)C(F)(F)OC(F)(F)C(F)(F)C(F)(F)C(F)(F)S(=O)(O)=O		-8.48	0.68	2.63	P	IN
QL03280	multiple ether-substituted Perfluoroalkyl (linear) sulfonic acids, n=3		FC(F)(F)C(F)(F)OC(F)(F)C(F)(F)OC(F)(F)C(F)(F)C(F)(F)S(=O)(O)=O		-8.18	0.68	2.50	P	IN
QL03281	multiple ether-substituted Perfluoroalkyl (linear) sulfonic acids, n=2		FC(F)(F)C(F)(F)OC(F)(F)C(F)(F)OC(F)(F)C(F)(F)S(=O)(O)=O		-8.00	0.68	2.44	P	IN
QL03282	1,1,2,2-Tetrafluoro-2-(pentafluoroethoxy)ethane-1-sulfonyl fluoride	67990-78-7	FC(F)(F)C(F)(F)OC(F)(F)C(F)(F)S(F)(=O)=O		-6.92	0.68	1.89	P	IN
QL03283	2-(2-Bromo-1,1,2,2-tetrafluoroethoxy)-1,1,1,2,3,3-hexafluoro-3-(pentafluoroethoxy)propane	1482416-44-3	FC(F)(F)C(F)(F)OC(F)(F)C(F)(OC(F)(F)C(F)(F)Br)C(F)(F)F		-7.56	0.68	2.24	P	IN
QL03284	1,1,1,2,3,3-Hexafluoro-2,3-bis(pentafluoroethoxy)propane	95842-03-8	FC(F)(F)C(F)(F)OC(F)(F)C(F)(OC(F)(F)C(F)(F)F)C(F)(F)F		-7.56	0.68	2.24	P	IN
QL03285	1,1,2,2-Tetrafluoro-2-{[1,1,1,2,3,3-hexafluoro-3-(pentafluoroethoxy)propan-2-yl]oxy}ethane-1-sulfonyl fluoride	27744-59-8	FC(F)(F)C(F)(F)OC(F)(F)C(F)(OC(F)(F)C(F)(F)S(F)(=O)=O)C(F)(F)F		-8.00	0.68	2.43	P	IN
QL03286	1-Bromo-1,1,2,2,4,5,5,7,8,8,10,11,11,13,13,14,14,14-octadecafluoro-4,7,10-tris(trifluoromethyl)-3,6,9,12-tetraoxatetradecane	1482416-51-2	FC(F)(F)C(F)(F)OC(F)(F)C(F)(OC(F)(F)C(F)(OC(F)(F)C(F)(OC(F)(F)C(F)(F)Br)C(F)(F)F)C(F)(F)F)C(F)(F)F		-8.95	0.68	2.92	P	OUT

QL03287	1-Bromo-1,1,2,2,4,5,5,7,8,8,10,11,11,13,14,14,16,16,17,17,17-henicosafluoro-4,7,10,13-tetrakis(trifluoromethyl)-3,6,9,12,15-pentaoxaheptadecane	1482416-52-3	FC(F)(F)C(F)(F)OC(F)(F)C(F)(OC(F)(F)C(F)(OC(F)(F)C(F)(OC(F)(F)C(F)(OC(F)(F)C(F)(F)Br)C(F)(F)F)C(F)(F)F)C(F)(F)F)C(F)(F)F		-9.43	0.68	3.16	P	OUT
QL03288	1,2-Dichloro(perfluoro-1-(ethoxymethoxy)ethane)	369371-42-6	FC(F)(F)C(F)(F)OC(F)(F)OC(F)(Cl)C(F)(F)Cl		-7.02	0.67	2.09	P	IN
QL03289	2,2,3,5,6-Pentafluoro-5-(pentafluoroethoxy)-3,6-bis(trifluoromethyl)-1,4-dioxane	84041-66-7	FC(F)(F)C(F)(F)OC1(F)OC(F)(C(F)(F)F)C(F)(F)OC1(F)C(F)(F)F		-7.35	0.68	2.08	P	IN
QL03290	Pentafluoroethane-1-sulfonyl chloride	64773-40-6	FC(F)(F)C(F)(F)S(Cl)(=O)=O		-5.80	0.68	1.32	P	IN
QL03291	Heptafluoro-2-iodopropane	677-69-0	FC(F)(F)C(F)(I)C(F)(F)F		-5.80	0.68	1.32	P	IN
QL03292	1,1,1,2,3,3,3-Heptafluoro-2-(1,1,2,3,3,3-hexafluoro-2-iodopropoxy)propane	16005-45-1	FC(F)(F)C(F)(I)C(F)(F)OC(F)(C(F)(F)F)C(F)(F)F		-6.82	0.68	1.82	P	IN
QL03293	Morpholine, 2,2,3,3,5,5,6,6-octafluoro-4-[1,2,2,2-tetrafluoro-1-(trifluoromethyl)ethyl]-	1600-71-1	FC(F)(F)C(F)(N1C(F)(F)C(F)(F)OC(F)(F)C1(F)F)C(F)(F)F		-6.50	0.68	1.61	P	IN
QL03294	Heptafluoroisopropyl acrylate	13057-08-4	FC(F)(F)C(F)(OC(=O)C=C)C(F)(F)F		-5.33	0.63	1.69	P	IN
QL03295	1,1,1,2,3,3,3-Heptafluoropropan-2-yl benzoate	57024-21-2	FC(F)(F)C(F)(OC(=O)c1ccccc1)C(F)(F)F		-5.90	0.63	2.10	P	OUT
QL03296	2-(1,2-Dichloro-1,2,2-trifluoroethoxy)-1,1,1,2,3,3,3-heptafluoropropane	85720-81-6	FC(F)(F)C(F)(OC(F)(Cl)C(F)(F)Cl)C(F)(F)F		-6.32	0.68	1.61	P	IN
QL03297	2-(2-Chloro-1,1,2-trifluoro-2-iodoethoxy)-1,1,1,2,3,3,3-heptafluoropropane	16031-04-2	FC(F)(F)C(F)(OC(F)(F)C(F)(Cl)I)C(F)(F)F		-6.32	0.68	1.61	P	IN
QL03298	2-(2-Bromo-1,1,2,2-tetrafluoroethoxy)-1,1,1,2,3,3,3-heptafluoropropane	16005-49-5	FC(F)(F)C(F)(OC(F)(F)C(F)(F)Br)C(F)(F)F		-6.32	0.68	1.61	P	IN
QL03299	1,1,2,2,3,3,4,4,5,5,6,6,7,7,8,8-Hexadecafluoro-1-[(1,1,1,2,3,3,3-heptafluoropropan-2-yl)oxy]-8-iodooctane	25080-19-7	FC(F)(F)C(F)(OC(F)(F)C(F)(F)C(F)(F)C(F)(F)C(F)(F)C(F)(F)C(F)(F)C(F)(F)I)C(F)(F)F		-8.66	0.69	2.64	P	IN
QL03300	1,1,2,2,3,3,4,4,5,5,6,6-Dodecafluoro-1-[(1,1,1,2,3,3,3-heptafluoropropan-2-yl)oxy]-8-iodooctane	25080-25-5	FC(F)(F)C(F)(OC(F)(F)C(F)(F)C(F)(F)C(F)(F)C(F)(F)C(F)(F)CCI)C(F)(F)F		-8.17	0.67	2.67	P	IN
QL03301	1-Iodo-6-(heptafluoroisopropoxy)perfluorohexane	25080-18-6	FC(F)(F)C(F)(OC(F)(F)C(F)(F)C(F)(F)C(F)(F)C(F)(F)C(F)(F)I)C(F)(F)F		-8.10	0.69	2.39	P	IN
QL03302	1,1,2,2,3,3,4,4-Octafluoro-1-[(1,1,1,2,3,3,3-heptafluoropropan-2-yl)oxy]-6-iodohexane	25080-24-4	FC(F)(F)C(F)(OC(F)(F)C(F)(F)C(F)(F)C(F)(F)CCI)C(F)(F)F		-7.50	0.66	2.43	P	IN
QL03303	1,1,2,2,3,3,4,4-Octafluoro-1-[(1,1,1,2,3,3,3-heptafluoropropan-2-yl)oxy]-4-iodobutane	25080-17-5	FC(F)(F)C(F)(OC(F)(F)C(F)(F)C(F)(F)C(F)(F)I)C(F)(F)F		-7.40	0.68	2.08	P	IN
QL03304	1,1,2,2,3,3,4,4-Octafluoro-1,4-bis[(1,1,1,2,3,3,3-heptafluoropropan-2-yl)oxy]butane	23228-90-2	FC(F)(F)C(F)(OC(F)(F)C(F)(F)C(F)(F)C(F)(F)OC(F)(C(F)(F)F)C(F)(F)F)C(F)(F)F		-7.89	0.68	2.34	P	IN
QL03305	1-[2,3,3,3-Tetrafluoro-2-(heptafluoropropoxy)-1-(piperidin-1-yl)propyl]-1H-benzotriazole	133384-08-4	FC(F)(F)C(F)(OC(F)(F)C(F)(F)C(F)(F)F)C(N1CCCCC1)n1nnc2ccccc12		-6.68	0.63	2.47	P	OUT
QL03306	1,1,1,2,3,3,3-Heptafluoro-2-(1,1,2,2-tetrafluoro-2-iodoethoxy)propane	16005-38-2	FC(F)(F)C(F)(OC(F)(F)C(F)(F)I)C(F)(F)F		-6.32	0.68	1.61	P	IN
QL03307	Bis(perfluoro(1-[(3,6-dimethyl-1,4-dioxan-2-yl)oxy]ethyl)) ketone	61097-96-9	FC(F)(F)C(F)(OC1(F)OC(F)(C(F)(F)F)C(F)(F)OC1(F)C(F)(F)F)C(=O)C(F)(OC1(F)OC(F)(C(F)(F)F)C(F)(F)OC1(F)C(F)(F)F)C(F)(F)F		-8.75	0.68	2.79	P	IN
QL03308	Perfluoro-2,3-bis(2,5-dimethyl-1,4-dioxan-3-oxy)butane	61097-98-1	FC(F)(F)C(F)(OC1(F)OC(F)(C(F)(F)F)C(F)(F)OC1(F)C(F)(F)F)C(F)(OC1(F)OC(F)(C(F)(F)F)C(F)(F)OC1(F)C(F)(F)F)C(F)(F)F		-8.58	0.68	2.68	P	IN
QL03309	2-[(1,1,1,2,3,3,3-Heptafluoropropan-2-yl)oxy]-N-phenylacetamide	62656-67-1	FC(F)(F)C(F)(OCC(=O)NC1=CC=CC=C1)C(F)(F)F		-6.12	0.62	2.36	P	OUT
QL03310	Allyl perfluoroisopropyl ether	15242-17-8	FC(F)(F)C(F)(OCC=C)C(F)(F)F		-5.05	0.62	1.69	P	OUT
QL03311	Trichloro[3-[1,2,2,2-tetrafluoro-1-(trifluoromethyl)ethoxy]propyl]silane	15538-93-9	FC(F)(F)C(F)(OCCC[Si](Cl)(Cl)Cl)C(F)(F)F		-5.93	0.64	1.92	P	IN
QL03312	Dichloromethyl[3-[1,2,2,2-tetrafluoro-1-(trifluoromethyl)ethoxy]propyl]silane	20006-68-2	FC(F)(F)C(F)(OCCC[SiH2]C(Cl)Cl)C(F)(F)F		-5.54	0.62	2.06	P	OUT
QL03313	1-Bromo-4-[(1,1,1,2,3,3,3-heptafluoropropan-2-yl)oxy]butane	37027-49-9	FC(F)(F)C(F)(OCCCCBr)C(F)(F)F		-5.27	0.61	2.02	P	OUT
QL03314	1,1,1,2,3,3,5,5,7,7,7-Undecafluoro-6-iodo-2,4-bis(trifluoromethyl)heptane	922524-01-4	FC(F)(F)C(I)C(F)(F)C(C(F)(F)F)C(F)(F)C(F)(C(F)(F)F)C(F)(F)F		-6.99	0.67	2.04	P	IN
QL03315	1,1,1,2,3,3,5,5,5-Nonafluoro-4-iodo-2-(trifluoromethyl)pentane	922524-00-3	FC(F)(F)C(I)C(F)(F)C(F)(C(F)(F)F)C(F)(F)F		-6.41	0.67	1.71	P	IN
QL03316	1,1,1,2,2,3,3,4,4,5,5,6,6,7,7,9,9,9-Octadecafluoro-8-iodononane	922524-04-7	FC(F)(F)C(I)C(F)(F)C(F)(F)C(F)(F)C(F)(F)C(F)(F)C(F)(F)C(F)(F)F		-8.16	0.68	2.45	P	IN
QL03317	1,1,1,2,7,7,7-Heptafluoro-6-iodo-2,4-bis(trifluoromethyl)heptane	922523-99-7	FC(F)(F)C(I)CC(CC(F)(C(F)(F)F)C(F)(F)F)C(F)(F)F		-6.73	0.66	2.07	P	IN
QL03318	1,1,1,2,5,5,5-Heptafluoro-4-iodo-2-(trifluoromethyl)pentane	922523-98-6	FC(F)(F)C(I)CC(F)(C(F)(F)F)C(F)(F)F		-5.96	0.66	1.61	P	IN
QL03319	1,1,1,2,2,3,3,4,4,5,5,6,6,9,9,9-Hexadecafluoro-8-iodononane	922524-02-5	FC(F)(F)C(I)CC(F)(F)C(F)(F)C(F)(F)C(F)(F)C(F)(F)C(F)(F)C(F)(F)F		-7.98	0.68	2.45	P	IN

QL03320	1,1,1,2,2,3,3,4,4,7,7,7-Dodecafluoro-6-iodoheptane	935553-88-1	FC(F)(F)C(I)CC(F)(F)C(F)(F)C(F)(F)C(F)(F)F		-7.25	0.67	2.17	P	IN
QL03321	1,1,1,3,3,3-Hexafluoropropan-2-yl heptafluorobutanoate	922168-41-0	FC(F)(F)C(OC(=O)C(F)(F)C(F)(F)C(F)(F)F)C(F)(F)F		-6.85	0.66	2.06	P	IN
QL03322	Hexafluoroisopropyl acrylate	2160-89-6	FC(F)(F)C(OC(=O)C=C)C(F)(F)F		-4.76	0.61	1.69	P	OUT
QL03323	1,1,1,3,3,3-Hexafluoropropan-2-yl nonafluorobutane-1-sulfonate	118334-96-6	FC(F)(F)C(OS(=O)(=O)C(F)(F)C(F)(F)C(F)(F)C(F)(F)F)C(F)(F)F		-7.40	0.68	2.18	P	IN
QL03324	H-substituted perfluoroalkyl (linear) sulfonic acids_ii, n=0		FC(F)(F)C(S(=O)(O)=O)(F)[H]		-4.98	0.65	1.30	P	IN
QL03325	1,1,1,4,4,5,5,6,6,7,7,8,8,9,9,9-Hexadecafluoronon-2-ene	935477-01-3	FC(F)(F)C=CC(F)(F)C(F)(F)C(F)(F)C(F)(F)C(F)(F)C(F)(F)F		-7.90	0.67	2.44	P	IN
QL03326	1,1,1,4,4,5,5,6,6,7,7,8,8,8-Tetradecafluorooct-2-ene	935476-95-2	FC(F)(F)C=CC(F)(F)C(F)(F)C(F)(F)C(F)(F)C(F)(F)F		-7.39	0.67	2.23	P	IN
QL03327	1,1,1,4,4,5,5,6,6,7,7,7-Dodecafluorohept-2-ene	887111-62-8	FC(F)(F)C=CC(F)(F)C(F)(F)C(F)(F)C(F)(F)F		-7.14	0.67	2.17	P	IN
QL03328	(2E)-1,1,1,4,4,5,5,6,6,6-Decafluorohex-2-ene	83227-62-7	FC(F)(F)C=CC(F)(F)C(F)(F)C(F)(F)F		-6.45	0.66	1.91	P	IN
QL03329	Cyclohexane, decafluorobis(trifluoromethyl)- (6CI,7CI,8CI,9CI)	26637-68-3	FC(F)(F)C1(C(F)(F)F)C(F)(F)C(F)(F)C(F)(F)C(F)(F)C1(F)F		-6.56	0.69	1.52	P	IN
QL03330	Perfluorodimethylcyclobutane	28677-00-1	FC(F)(F)C1(C(F)(F)F)C(F)(F)C(F)(F)C1(F)F		-6.42	0.69	1.48	P	IN
QL03331	Perfluoro-1,3,5-trimethylcyclohexane	374-76-5	FC(F)(F)C1(F)C(F)(F)C(F)(C(F)(F)F)C(F)(F)C(F)(C(F)(F)F)C1(F)F		-6.22	0.69	1.35	P	IN
QL03332	Cyclobutane, 1,1,2,3,3,4-hexafluoro-2,4-bis(trifluoromethyl)-	13221-71-1	FC(F)(F)C1(F)C(F)(F)C(F)(C(F)(F)F)C1(F)F		-7.28	0.69	1.89	P	IN
QL03333	1,1,2,2,3,4,4,5,5,6-Decafluoro-3,6-bis(trifluoromethyl)cyclohexane	374-77-6	FC(F)(F)C1(F)C(F)(F)C(F)(F)C(F)(C(F)(F)F)C(F)(F)C1(F)F		-7.33	0.69	1.89	P	IN
QL03334	Perfluoro-1,3-dimethylcyclohexane	335-27-3	FC(F)(F)C1(F)C(F)(F)C(F)(F)C(F)(F)C(F)(C(F)(F)F)C1(F)F		-6.54	0.69	1.48	P	IN
QL03335	Perfluoro-5,5'-bis(trifluoromethyl)-1,1'-bicyclohexyl	105462-77-9	FC(F)(F)C1(F)C(F)(F)C(F)(F)C(F)(F)C(F)(C1(F)F)C1(F)C(F)(F)C(F)(F)C(F)(F)C(F)(C(F)(F)F)C1(F)F		-7.97	0.70	2.15	P	IN
QL03336	Perfluoro-1,2-dimethylcyclohexane	306-98-9	FC(F)(F)C1(F)C(F)(F)C(F)(F)C(F)(F)C(F)(F)C1(F)C(F)(F)F		-6.54	0.69	1.48	P	IN
QL03337	Perfluoromethylcyclohexane	355-02-2	FC(F)(F)C1(F)C(F)(F)C(F)(F)C(F)(F)C(F)(F)C1(F)F		-6.60	0.70	1.47	P	IN
QL03338	Cyclohexanesulfonyl fluoride, decafluoro(trifluoromethyl)-	68318-34-3	FC(F)(F)C1(F)C(F)(F)C(F)(F)C(F)(F)C(F)(F)C1(F)S(F)(=O)=O		-6.54	0.69	1.48	P	IN
QL03339	Perfluoromethylcyclopentane	1805-22-7	FC(F)(F)C1(F)C(F)(F)C(F)(F)C(F)(F)C1(F)F		-6.60	0.70	1.48	P	IN
QL03340	Perfluoromethyldecalin	51294-16-7	FC(F)(F)C1(F)C(F)(F)C(F)(F)C(F)(F)C2(F)C(F)(F)C(F)(F)C(F)(F)C(F)(F)C12F		-7.30	0.70	1.77	P	IN
QL03341	Perfluoro-1,2-dimethylcyclobutane	2994-71-0	FC(F)(F)C1(F)C(F)(F)C(F)(F)C1(F)C(F)(F)F		-6.22	0.69	1.35	P	IN
QL03342	Perfluoro-2-methyldecalin	306-95-6	FC(F)(F)C1(F)C(F)(F)C(F)(F)C2(F)C(F)(F)C(F)(F)C(F)(F)C(F)(F)C2(F)C1(F)F		-7.85	0.70	2.06	P	IN
QL03343	Perfluoro-3,6-dimethyl-1,4-dioxan-2-one	7309-84-4	FC(F)(F)C1(F)OC(F)(F)C(F)(OC1=O)C(F)(F)F		-6.96	0.67	1.98	P	IN
QL03344	Perfluoromethyloxirane	428-59-1	FC(F)(F)C1(F)OC1(F)F		-5.97	0.68	1.36	P	IN
QL03345	Perfluoroalkyl N-heterocycles, m+n+p=6		FC(F)(F)C1=C(OC(F)(F)F)N=C(C(F)(C(F)(F)F)C(F)(F)C(F)(F)F)[N-]1		-6.96	0.66	2.17	P	IN
QL03346	2,2,3,3,4,4,5,5,6,6,7,7,8,8,9,9,9-Heptadecafluoro-N-[3-(trifluoromethyl)phenyl]nonanamide	325989-96-6	FC(F)(F)c1cccc(NC(=O)C(F)(F)C(F)(F)C(F)(F)C(F)(F)C(F)(F)C(F)(F)C(F)(F)C(F)(F)F)c1		-8.65	0.67	2.95	P	IN
QL03347	2,2,3,3,4,4,4-Heptafluoro-N-[3-(trifluoromethyl)phenyl]butanamide	2059-15-6	FC(F)(F)c1cccc(NC(=O)C(F)(F)C(F)(F)C(F)(F)F)c1		-6.98	0.64	2.40	P	IN
QL03348	Dichlorobis(1,1,2,2,3,3,6,6,6-nonafluorohexyl)silane	156323-66-9	FC(F)(F)CCC(F)(F)C(F)(F)C(F)(F)[Si](Cl)(Cl)C(F)(F)C(F)(F)C(F)(F)CCC(F)(F)F		-8.44	0.67	2.80	P	IN
QL03349	Trichloro(1,1,2,2,3,3,6,6,6-nonafluorohexyl)silane	78560-47-1	FC(F)(F)CCC(F)(F)C(F)(F)C(F)(F)[Si](Cl)(Cl)Cl		-7.14	0.67	2.17	P	IN
QL03350	1-Bromo-4-(1,1,2,2,3,3,4,4,5,5,6,6,7,7,10,10,10-heptadecafluorodecyl)benzene	195324-88-0	FC(F)(F)CCC(F)(F)C(F)(F)C(F)(F)C(F)(F)C(F)(F)C(F)(F)C(F)(F)C1=CC=C(Br)C=C1		-8.35	0.66	2.88	P	IN
QL03351	1,1,1,2,2,3,3,4,4,7,7,7-Dodecafluoroheptane	503189-11-5	FC(F)(F)CCC(F)(F)C(F)(F)C(F)(F)C(F)(F)F		-7.14	0.67	2.17	P	IN
QL03352	1,1,1,2,2,3,3,6,6,6-Decafluorohexane	188189-52-8	FC(F)(F)CCC(F)(F)C(F)(F)C(F)(F)F		-6.45	0.66	1.91	P	IN
QL03353	Poly(difluoromethylene), .alpha.-[2-[(2-carboxyethyl)thio]ethyl]-.omega.-fluoro-, lithium salt (11)|Poly(difluoromethylene), .alpha.-[2-[(2-carboxyethyl)thio]ethyl]-.omega.-fluoro- (Zonyl 7950)	65530-69-0|65530-83-8	FC(F)(F)CCSCCC(O)=O		-4.29	0.57	2.12	P	OUT
QL03354	2,2,2-trifluoroethyl perfluorobutanimidate	4078-29-9	FC(F)(F)COC(=N)C(F)(F)C(F)(F)C(F)(F)F		-6.80	0.65	2.16	P	IN
QL03355	2,2,2-Trifluoroethyl 4,4,5,5,6,6,7,7,8,8,9,9,10,10,11,11,11-heptadecafluoroundecanoate	344580-04-7	FC(F)(F)COC(=O)CCC(F)(F)C(F)(F)C(F)(F)C(F)(F)C(F)(F)C(F)(F)C(F)(F)C(F)(F)F		-8.65	0.67	2.94	P	IN
QL03356	2,2,3,3,4,4,5,5,5-Nonafluoro-1-phenylpentyl bis(2,2,2-trifluoroethyl) phosphate	100664-15-1	FC(F)(F)COP(=O)(OCC(F)(F)F)OC(c1ccccc1)C(F)(F)C(F)(F)C(F)(F)C(F)(F)F		-6.87	0.64	2.43	P	IN
QL03357	2,2,2-Trifluoroethyl perfluorobutanesulfonate	79963-95-4	FC(F)(F)COS(=O)(=O)C(F)(F)C(F)(F)C(F)(F)C(F)(F)F		-7.39	0.67	2.23	P	IN
QL03358	Perfluoro(N-methylmorpholine)	382-28-5	FC(F)(F)N1C(F)(F)C(F)(F)OC(F)(F)C1(F)F		-5.42	0.68	1.13	P	IN
QL03359	1,1,1,3,3,3-Hexafluoro-2-(trifluoromethoxy)-2-(trifluoromethyl)propane	681-08-3	FC(F)(F)OC(C(F)(F)F)(C(F)(F)F)C(F)(F)F		-5.22	0.66	1.22	P	IN

QL03360	ether-substituted perfluoroalkyl (linear) caborxylic acids_i, n=1		FC(F)(F)OC(F)(C(F)(F)F)C(O)=O		-5.07	0.65	1.38	P	IN
QL03361	3,4,4,4-Tetrafluoro-3-(trifluoromethoxy)-1-butene	226575-14-0	FC(F)(F)OC(F)(C=C)C(F)(F)F		-4.87	0.64	1.42	P	IN
QL03362	[2,3,3,3-Tetrafluoro-2-(trifluoromethoxy)propyl]oxirane	243139-60-8	FC(F)(F)OC(F)(CC1CO1)C(F)(F)F		-5.74	0.64	1.83	P	IN
QL03363	1,2-Dibromo-1,1,2,3,3-pentafluoro-3-(trifluoromethoxy)propane	885275-98-9	FC(F)(F)OC(F)(F)C(F)(Br)C(F)(F)Br		-6.40	0.67	1.81	P	IN
QL03364	Perfluoro-3-methoxypropanoic anhydride	42566-65-4	FC(F)(F)OC(F)(F)C(F)(F)C(=O)OC(=O)C(F)(F)C(F)(F)OC(F)(F)F		-7.61	0.65	2.59	P	IN
QL03365	ether-substituted perfluoroalkyl (linear) caborxylic acids_ii, n=15		FC(F)(F)OC(F)(F)C(F)(F)C(F)(F)C(F)(F)C(F)(F)C(F)(F)C(F)(F)C(F)(F)C(F)(F)C(F)(F)C(F)(F)C(F)(F)C(F)(F)C(F)(F)C(F)(F)C(O)=O		-9.85	0.69	3.27	P	OUT
QL03366	ether-substituted perfluoroalkyl (linear) caborxylic acids_ii, n=14		FC(F)(F)OC(F)(F)C(F)(F)C(F)(F)C(F)(F)C(F)(F)C(F)(F)C(F)(F)C(F)(F)C(F)(F)C(F)(F)C(F)(F)C(F)(F)C(F)(F)C(F)(F)C(O)=O		-9.75	0.69	3.23	P	OUT
QL03367	ether-substituted perfluoroalkyl (linear) caborxylic acids_ii, n=13		FC(F)(F)OC(F)(F)C(F)(F)C(F)(F)C(F)(F)C(F)(F)C(F)(F)C(F)(F)C(F)(F)C(F)(F)C(F)(F)C(F)(F)C(F)(F)C(F)(F)C(O)=O		-9.50	0.68	3.11	P	OUT
QL03368	ether-substituted perfluoroalkyl (linear) caborxylic acids_ii, n=12		FC(F)(F)OC(F)(F)C(F)(F)C(F)(F)C(F)(F)C(F)(F)C(F)(F)C(F)(F)C(F)(F)C(F)(F)C(F)(F)C(F)(F)C(F)(F)C(O)=O		-9.38	0.68	3.07	P	OUT
QL03369	ether-substituted perfluoroalkyl (linear) caborxylic acids_ii, n=11		FC(F)(F)OC(F)(F)C(F)(F)C(F)(F)C(F)(F)C(F)(F)C(F)(F)C(F)(F)C(F)(F)C(F)(F)C(F)(F)C(F)(F)C(O)=O		-9.09	0.68	2.94	P	OUT
QL03370	ether-substituted perfluoroalkyl (linear) caborxylic acids_ii, n=10		FC(F)(F)OC(F)(F)C(F)(F)C(F)(F)C(F)(F)C(F)(F)C(F)(F)C(F)(F)C(F)(F)C(F)(F)C(F)(F)C(O)=O		-8.96	0.68	2.89	P	OUT
QL03371	ether-substituted perfluoroalkyl (linear) caborxylic acids_ii, n=9		FC(F)(F)OC(F)(F)C(F)(F)C(F)(F)C(F)(F)C(F)(F)C(F)(F)C(F)(F)C(F)(F)C(F)(F)C(O)=O		-8.62	0.68	2.74	P	IN
QL03372	ether-substituted perfluoroalkyl (linear) caborxylic acids_ii, n=8		FC(F)(F)OC(F)(F)C(F)(F)C(F)(F)C(F)(F)C(F)(F)C(F)(F)C(F)(F)C(F)(F)C(O)=O		-8.46	0.68	2.68	P	IN
QL03373	Heptane, 1,1,1,2,2,3,3,4,4,5,5,6,6,7,7-pentadecafluoro-7-(trifluoromethoxy)-	133881-46-6	FC(F)(F)OC(F)(F)C(F)(F)C(F)(F)C(F)(F)C(F)(F)C(F)(F)C(F)(F)F		-8.06	0.68	2.44	P	IN
QL03374	1-Chloro-1,1,2,2-tetrafluoro-2-(trifluoromethoxy)ethane	1561-50-8	FC(F)(F)OC(F)(F)C(F)(F)Cl		-5.48	0.66	1.46	P	IN
QL03375	1,1,2,2-Tetrafluoro-1-iodo-2-(trifluoromethoxy)ethane	1561-52-0	FC(F)(F)OC(F)(F)C(F)(F)I		-5.48	0.66	1.46	P	IN
QL03376	1,1,2,2-Tetrafluoro-2-(trifluoromethoxy)-N,N-bis(trifluoromethyl)ethanamine	160228-75-1	FC(F)(F)OC(F)(F)C(F)(F)N(C(F)(F)F)C(F)(F)F		-6.17	0.66	1.78	P	IN
QL03377	1-[bromo(difluoro)methoxy]-1,1,2,2-tetrafluoro-2-(trifluoromethoxy)ethane	87136-78-5	FC(F)(F)OC(F)(F)C(F)(F)OC(F)(F)Br		-5.99	0.65	1.81	P	IN
QL03378	2,2-difluoro-2-[1,1,2,2-tetrafluoro-2-(trifluoromethoxy)ethoxy]acetyl Chloride	261503-81-5	FC(F)(F)OC(F)(F)C(F)(F)OC(F)(F)C(Cl)=O		-6.75	0.65	2.19	P	IN
QL03379	1-[Bromo(difluoro)methoxy]-1,1,2,2-tetrafluoro-2-[1,1,2,2-tetrafluoro-2-(trifluoromethoxy)ethoxy]ethane	330562-45-3	FC(F)(F)OC(F)(F)C(F)(F)OC(F)(F)C(F)(F)OC(F)(F)Br		-7.23	0.66	2.33	P	IN
QL03380	1,1,1,3,3,4,4,6,6,7,7,9,9,10,10,12,12,13,13,15,15,15-Docosafluoro-2,5,8,11,14-pentaoxapentadecane	64028-06-4	FC(F)(F)OC(F)(F)C(F)(F)OC(F)(F)C(F)(F)OC(F)(F)C(F)(F)OC(F)(F)C(F)(F)OC(F)(F)F		-8.66	0.67	2.95	P	IN
QL03381	Perfluoro-2,5,8,11-tetraoxadodecane	64028-04-2	FC(F)(F)OC(F)(F)C(F)(F)OC(F)(F)C(F)(F)OC(F)(F)C(F)(F)OC(F)(F)F		-7.97	0.66	2.64	P	IN
QL03382	3-Ethenyl-1,1,2-trifluoro-2-(1,1,2,2-tetrafluoro-2-{1,1,2,2-tetrafluoro-2-[1,1,2,2-tetrafluoro-2-(trifluoromethoxy)ethoxy]ethoxy}ethoxy)cyclobutane	709620-35-9	FC(F)(F)OC(F)(F)C(F)(F)OC(F)(F)C(F)(F)OC(F)(F)C(F)(F)OC1(F)C(CC1(F)F)C=C		-8.50	0.66	2.94	P	IN
QL03383	1-[Chloro(difluoro)methoxy]-1,1,2,2-tetrafluoro-2-(trifluoromethoxy)ethane	87136-79-6	FC(F)(F)OC(F)(F)C(F)(F)OC(F)(F)Cl		-5.99	0.65	1.81	P	IN
QL03384	1-[Difluoro(iodo)methoxy]-1,1,2,2-tetrafluoro-2-(trifluoromethoxy)ethane	138370-72-6	FC(F)(F)OC(F)(F)C(F)(F)OC(F)(F)I		-5.99	0.65	1.81	P	IN
QL03385	1,2-Dichloro(perfluoro-1-[(2-methoxyethoxy)methoxy]ethane)	369371-43-7	FC(F)(F)OC(F)(F)C(F)(F)OC(F)(F)OC(F)(Cl)C(F)(F)Cl		-7.44	0.66	2.39	P	IN
QL03386	1,1,1,2,3,3-Hexafluoro-2,3-bis(trifluoromethoxy)propane	95842-02-7	FC(F)(F)OC(F)(F)C(F)(OC(F)(F)F)C(F)(F)F		-6.32	0.66	1.90	P	IN
QL03387	ether-substituted perfluoroalkyl (linear) caborxylic acids, n=0		FC(F)(F)OC(F)(F)C(O)=O		-4.90	0.63	1.49	P	IN
QL03388	1,2-dichloro(perfluoro-1-(methoxymethoxy)ethane)	874288-98-9	FC(F)(F)OC(F)(F)OC(F)(Cl)C(F)(F)Cl		-6.28	0.65	1.91	P	IN
QL03389	multiple ether-substituted perfluoroalkyl (linear) caborxylic acids, n=2	39492-89-2	FC(F)(F)OC(F)(F)OC(F)(F)C(O)=O		-5.85	0.64	1.93	P	IN
QL03390	multiple ether-substituted perfluoroalkyl (linear) caborxylic acids, n=3	39492-90-5	FC(F)(F)OC(F)(F)OC(F)(F)OC(F)(F)C(O)=O		-6.56	0.64	2.27	P	IN
QL03391	multiple ether-substituted perfluoroalkyl (linear) caborxylic acids, n=4	39492-91-6	FC(F)(F)OC(F)(F)OC(F)(F)OC(F)(F)OC(F)(F)C(O)=O		-7.13	0.64	2.54	P	IN

QL03392	1,1,1,2-Tetrafluoro-2-iodo-2-(trifluoromethoxy)ethane	139604-89-0	FC(F)(F)OC(F)(I)C(F)(F)F		-5.48	0.66	1.46	P	IN
QL03393	1,1,1,5,5,6,6,10,10,10-Decafluoro-3,3,8,8-tetrakis(trifluoromethyl)-2,4,7,9-tetraoxadecane	95841-80-8	FC(F)(F)OC(OC(F)(F)C(F)(F)OC(OC(F)(F)F)(C(F)(F)F)C(F)(F)F)(C(F)(F)F)C(F)(F)F		-7.33	0.67	2.29	P	IN
QL03394	Tetrakis(trifluoromethoxy)methane	92639-87-7	FC(F)(F)OC(OC(F)(F)F)(OC(F)(F)F)OC(F)(F)F		-5.08	0.63	1.58	P	IN
QL03395	Undecafluoro(trifluoromethoxy)cyclohexane	4943-06-0	FC(F)(F)OC1(F)C(F)(F)C(F)(F)C(F)(F)C(F)(F)C1(F)F		-7.18	0.69	1.92	P	IN
QL03396	1-Methyl-3-((perfluorobutyl)ethyl)-1H-imidazolium N,N-bis(perfluoromethansulfonyl)amide	872672-61-2	FC(F)(F)S(=O)(=O)[N-]S(=O)(=O)C(F)(F)F.C[NH+]1CN(CCC(F)(F)C(F)(F)C(F)(F)C(F)(F)F)C=C1		-8.16	0.66	2.81	P	IN
QL03397	1,8-Diiodoperfluorooctane	335-70-6	FC(F)(I)C(F)(F)C(F)(F)C(F)(F)C(F)(F)C(F)(F)C(F)(F)C(F)(F)I		-7.94	0.69	2.24	P	IN
QL03398	3,3,4,4,5,5,6,6-octafluoro-6-iodohex-1-ene	203929-12-8	FC(F)(I)C(F)(F)C(F)(F)C(F)(F)C=C		-6.40	0.67	1.81	P	IN
QL03399	3,3,4,4-Tetrafluoro-4-iodo-1-butene	33831-83-3	FC(F)(I)C(F)(F)C=C		-4.95	0.64	1.39	P	IN
QL03400	1,2-Diiodotetrafluoroethane	354-65-4	FC(F)(I)C(F)(F)I		-4.57	0.67	0.81	P	OUT
QL03401	5-Iodoperfluoro-3-oxapentanesulfonyl fluoride	66137-74-4	FC(F)(I)C(F)(F)OC(F)(F)C(F)(F)S(F)(=O)=O		-6.92	0.68	1.89	P	IN
QL03402	2-Allyloxyperfluoroethanesulfonyl fluoride	73606-13-0	FC(F)(OCC=C)C(F)(F)S(F)(=O)=O		-5.63	0.63	1.95	P	OUT
QL03403	10-10-phosphinic		FC(F)(P(C(F)(F)C(F)(F)C(F)(F)C(F)(F)C(F)(F)C(F)(F)C(F)(F)C(F)(C(F)(F)C(F)(F)F)F)(O)=O)C(F)(F)C(F)(F)C(F)(F)C(F)(F)C(F)(F)C(F)(F)C(F)(F)C(F)(F)C(F)(F)F		-10.59	0.70	3.51	P	OUT
QL03404	4-6-phosphinic		FC(F)(P(C(F)(F)C(F)(F)C(F)(F)C(F)(F)C(F)(F)C(F)(F)F)(O)=O)C(F)(F)C(F)(F)C(F)(F)F		-8.88	0.69	2.69	P	OUT
QL03405	Vinylidene fluoride	75-38-7	FC(F)=C		0.10	0.50	0.81	P	OUT
QL03406	Bromotrifluoroethylene	598-73-2	FC(F)=C(F)Br		-2.25	0.58	0.92	P	OUT
QL03407	1,1,2,4,4-Pentafluoro-3-(trifluoromethyl)buta-1,3-diene	384-04-3	FC(F)=C(F)C(=C(F)F)C(F)(F)F		-4.24	0.61	1.42	P	OUT
QL03408	1,1,2,3,4,4,4-Heptafluoro-3-(trifluoromethyl)but-1-ene	21581-82-8	FC(F)=C(F)C(F)(C(F)(F)F)C(F)(F)F		-5.27	0.65	1.40	P	IN
QL03409	1,1,2,3,3,4,5,5,5-Nonafluoro-4-(trifluoromethyl)pent-1-ene	287101-12-6	FC(F)=C(F)C(F)(F)C(F)(C(F)(F)F)C(F)(F)F		-6.14	0.66	1.75	P	IN
QL03410	1,1,2,3,3,4,4,5,5,6,6,7,7,8,8,9,9,10,10,11,11,12,12,13,13,13-Hexacosafluorotridec-1-ene	66325-97-1	FC(F)=C(F)C(F)(F)C(F)(F)C(F)(F)C(F)(F)C(F)(F)C(F)(F)C(F)(F)C(F)(F)C(F)(F)C(F)(F)C(F)(F)F		-9.03	0.68	2.89	P	OUT
QL03411	1,1,2,3,3,4,4,5,5,6,6,7,7,8,8,9,9,10,10,11,11,12,12,12-Tetracosafluorododec-1-ene	1828-40-6	FC(F)=C(F)C(F)(F)C(F)(F)C(F)(F)C(F)(F)C(F)(F)C(F)(F)C(F)(F)C(F)(F)C(F)(F)C(F)(F)F		-8.70	0.68	2.75	P	IN
QL03412	1,1,2,3,3,4,4,5,5,6,6,7,7,8,8,9,9,10,10,11,11,11-Docosafluoroundec-1-ene	66256-52-8	FC(F)=C(F)C(F)(F)C(F)(F)C(F)(F)C(F)(F)C(F)(F)C(F)(F)C(F)(F)C(F)(F)C(F)(F)F		-8.54	0.68	2.69	P	IN
QL03413	Perfluoro-1-decene	35328-43-9	FC(F)=C(F)C(F)(F)C(F)(F)C(F)(F)C(F)(F)C(F)(F)C(F)(F)C(F)(F)C(F)(F)F		-8.15	0.68	2.52	P	IN
QL03414	Perfluoronon-1-ene	376-22-7	FC(F)=C(F)C(F)(F)C(F)(F)C(F)(F)C(F)(F)C(F)(F)C(F)(F)C(F)(F)F		-7.96	0.68	2.45	P	IN
QL03415	Perfluorooct-1-ene	559-14-8	FC(F)=C(F)C(F)(F)C(F)(F)C(F)(F)C(F)(F)C(F)(F)C(F)(F)F		-7.47	0.67	2.24	P	IN
QL03416	Perfluorohept-1-ene	355-63-5	FC(F)=C(F)C(F)(F)C(F)(F)C(F)(F)C(F)(F)C(F)(F)F		-7.22	0.67	2.17	P	IN
QL03417	Perfluorohex-1-ene	755-25-9	FC(F)=C(F)C(F)(F)C(F)(F)C(F)(F)C(F)(F)F		-6.57	0.66	1.91	P	IN
QL03418	Perfluoropent-1-ene	376-87-4	FC(F)=C(F)C(F)(F)C(F)(F)C(F)(F)F		-6.21	0.66	1.83	P	IN
QL03419	Trifluoro(1,1,2,2,3,4,4-heptafluorobut-3-enyl)oxirane	15453-10-8	FC(F)=C(F)C(F)(F)C(F)(F)C1(F)OC1(F)F		-6.60	0.67	1.90	P	IN
QL03420	Octafluoro-1-butene	357-26-6	FC(F)=C(F)C(F)(F)C(F)(F)F		-5.25	0.65	1.48	P	IN
QL03421	4,5-Epoxy-1,1,2,3,3,4,5,5-octafluoropent-1-ene	15453-08-4	FC(F)=C(F)C(F)(F)C1(F)OC1(F)F		-6.26	0.66	1.84	P	IN
QL03422	Hexafluoro-1,3-butadiene	685-63-2	FC(F)=C(F)C(F)=C(F)F		-3.89	0.59	1.52	P	OUT
QL03423	Ethanesulfonic acid, 1,1,2,2-tetrafluoro-2-((trifluoroethenyl)oxy)-, polymer with tetrafluoroethene	111173-25-2	FC(F)=C(F)F.OS(=O)(=O)C(F)(F)C(F)(F)OC(F)=C(F)F		-6.77	0.63	2.49	P	OUT
QL03424	1,1,1,2,3,3,3-Heptafluoro-2-((trifluorovinyl)oxy)propane	10372-98-2	FC(F)=C(F)OC(F)(C(F)(F)F)C(F)(F)F		-5.60	0.65	1.65	P	IN
QL03425	Perfluoro(2-bromoethoxy)ethene	85737-06-0	FC(F)=C(F)OC(F)(F)C(F)(F)Br		-5.55	0.64	1.76	P	IN
QL03426	2,2,3,3,4,4,5,5,6,6-Decafluoro-6-[(trifluoroethenyl)oxy]hexanenitrile	120903-40-4	FC(F)=C(F)OC(F)(F)C(F)(F)C(F)(F)C(F)(F)C(F)(F)C#N		-7.30	0.65	2.44	P	IN
QL03427	1,1,2,2,3,3,4,4-Octafluoro-1-[(trifluoroethenyl)oxy]-4-(trifluoromethoxy)butane	609818-38-4	FC(F)=C(F)OC(F)(F)C(F)(F)C(F)(F)C(F)(F)OC(F)(F)F		-7.30	0.65	2.44	P	IN
QL03428	1-chloro-1,1,2,2,3,3-hexafluoro-3-[(trifluorovinyl)oxy]propane	80962-91-0	FC(F)=C(F)OC(F)(F)C(F)(F)C(F)(F)Cl		-6.19	0.65	1.92	P	IN
QL03429	1,1,2,2,3,3-Hexafluoro-1-[(trifluoroethenyl)oxy]-3-(trifluoromethoxy)propane	40573-09-9	FC(F)=C(F)OC(F)(F)C(F)(F)C(F)(F)OC(F)(F)F		-6.68	0.65	2.23	P	IN
QL03430	1,1,2,2,3,3-Hexafluoro-1,3-bis((trifluorovinyl)oxy)propane	13846-22-5	FC(F)=C(F)OC(F)(F)C(F)(F)C(F)(F)OC(F)=C(F)F		-6.75	0.63	2.42	P	OUT
QL03431	1,1,2,2,3,3-hexafluoro-1-[(trifluorovinyl)oxy]propane	84145-18-6	FC(F)=C(F)OC(F)(F)C(F)(F)C(F)F		-5.77	0.63	1.93	P	IN
QL03432	Trifluoro(1,1,2,2-tetrafluoro-2-iodoethoxy)ethylene	6037-91-8	FC(F)=C(F)OC(F)(F)C(F)(F)I		-5.55	0.64	1.76	P	IN

QL03433	1,1'-((1,1,2,2-Tetrafluoroethylene)bis(oxy))bis(1,2,2-trifluoroethylene)	1998-53-4	FC(F)=C(F)OC(F)(F)C(F)(F)OC(F)=C(F)F		-6.02	0.62	2.20	P	OUT
QL03434	Perfluoro(3-oxapent-4-ene)sulfonyl fluoride	29514-94-1	FC(F)=C(F)OC(F)(F)C(F)(F)S(F)(=O)=O		-6.19	0.65	1.92	P	IN
QL03435	Perfluoro(2-propoxypropyl vinyl ether)	1644-11-7	FC(F)=C(F)OC(F)(F)C(F)(OC(F)(F)C(F)(F)C(F)(F)F)C(F)(F)F		-7.53	0.66	2.44	P	IN
QL03436	Perfluoro(3-(1-(ethenyloxy)propan-2-yloxy)propanenitrile)	69804-19-9	FC(F)=C(F)OC(F)(F)C(F)(OC(F)(F)C(F)(F)C#N)C(F)(F)F		-7.09	0.65	2.42	P	IN
QL03437	Perfluoro(4-methyl-3,6-dioxaoct-7-ene)sulfonyl fluoride	16090-14-5	FC(F)=C(F)OC(F)(F)C(F)(OC(F)(F)C(F)(F)S(F)(=O)=O)C(F)(F)F		-7.53	0.66	2.44	P	IN
QL03438	1,1,1,2,2,3,3-Heptafluoro-3-{[1,1,1,2,3,3-hexafluoro-3-({1,1,1,2,3,3-hexafluoro-3-[(trifluoroethenyl)oxy]propan-2-yl}oxy)propan-2-yl]oxy}propane	2599-84-0	FC(F)=C(F)OC(F)(F)C(F)(OC(F)(F)C(F)(OC(F)(F)C(F)(F)C(F)(F)F)C(F)(F)F)C(F)(F)F		-8.15	0.67	2.67	P	IN
QL03439	1,1,2,3,3-pentafluoro-3-[(trifluorovinyl)oxy]propene	64080-43-9	FC(F)=C(F)OC(F)(F)C(F)=C(F)F		-5.21	0.61	1.95	P	OUT
QL03440	1-[Difluoro(pentafluoroethoxy)methoxy]-1,2,2-trifluoroethene	346662-93-9	FC(F)=C(F)OC(F)(F)OC(F)(F)C(F)(F)F		-6.33	0.64	2.13	P	IN
QL03441	Perfluoro[(2-methoxyethoxy)methoxy]ethene	369371-47-1	FC(F)=C(F)OC(F)(F)OC(F)(F)C(F)(F)OC(F)(F)F		-6.80	0.64	2.40	P	IN
QL03442	1-[Difluoro(trifluoromethoxy)methoxy]-1,2,2-trifluoroethene	700874-87-9	FC(F)=C(F)OC(F)(F)OC(F)(F)F		-5.32	0.62	1.92	P	OUT
QL03443	1,1'-[(difluoromethylene)bis(oxy)]bis[1,2,2-trifluoroethylene]	13845-92-6	FC(F)=C(F)OC(F)(F)OC(F)=C(F)F		-5.43	0.60	2.17	P	OUT
QL03444	2,2,3,5,6-Pentafluoro-3,6-bis(trifluoromethyl)-5-((trifluorovinyl)oxy)-1,4-dioxane	61097-79-8	FC(F)=C(F)OC1(F)OC(F)(C(F)(F)F)C(F)(F)OC1(F)C(F)(F)F		-6.91	0.66	2.11	P	IN
QL03445	2-(difluoromethylene)-4,4,5-trifluoro-5-(trifluoromethyl)-1,3-dioxolane	17256-52-9	FC(F)=C1OC(F)(F)C(F)(O1)C(F)(F)F		-6.10	0.65	1.83	P	IN
QL03446	1,1,3,4,4,4-Hexafluoro-3-(trifluoromethyl)but-1-ene	1840-17-1	FC(F)=CC(F)(C(F)(F)F)C(F)(F)F		-5.02	0.65	1.38	P	IN
QL03447	1,1,3,3,3-Pentafluoropropene	690-27-7	FC(F)=CC(F)(F)F		-3.43	0.59	1.30	P	OUT
QL03448	(Nonafluorobutyl)carbonimidoyl	424-32-8	FC(F)=NC(F)(F)C(F)(F)C(F)(F)C(F)(F)F		-6.37	0.66	1.90	P	IN
QL03449	1,1,2,2,3,3,4,4-Octafluorobutane-1-sulphonic anhydride	93894-55-4	FC(F)C(F)(F)C(F)(F)C(F)(F)[S](=O)(=O)O[S](=O)(=O)C(F)(F)C(F)(F)C(F)(F)C(F)F		-8.28	0.68	2.63	P	IN
QL03450	Trichloro(1,1,2,2,3,3,4,4-octafluorobutyl)silane	375-63-3	FC(F)C(F)(F)C(F)(F)C(F)(F)[Si](Cl)(Cl)Cl		-6.61	0.67	1.81	P	IN
QL03451	1-(6H-Perfluorohexyl)-1-(fluoren-2-yl)-N-(perfluorobutyl)sulfonyloxymethanimine	691358-66-4	FC(F)C(F)(F)C(F)(F)C(F)(F)C(F)(F)C(F)(F)C(=NOS(=O)(=O)C(F)(F)C(F)(F)C(F)(F)C(F)(F)F)C1=CC2=C(C=C1)C1=C(C2)C=CC=C1		-8.28	0.66	2.89	P	IN
QL03452	2,2,3,3,4,4,5,5,6,6,7,7,8,8-Tetradecafluoro-1-phenyloctan-1-one	1427-89-0	FC(F)C(F)(F)C(F)(F)C(F)(F)C(F)(F)C(F)(F)C(F)(F)C(=O)C1=CC=CC=C1		-7.75	0.65	2.68	P	IN
QL03453	1,1,1,2,2,3,3,4,4,5,5,6,6,7,7,8,8,9,9,10,10,11,11,12,12,13,13,14,14-Nonacosafluorotetradecane	66577-33-1	FC(F)C(F)(F)C(F)(F)C(F)(F)C(F)(F)C(F)(F)C(F)(F)C(F)(F)C(F)(F)C(F)(F)C(F)(F)C(F)(F)C(F)(F)C(F)(F)F		-9.31	0.69	2.95	P	OUT
QL03454	1,1,1,2,2,3,3,4,4,5,5,6,6,7,7,8,8,9,9,10,10,11,11,12,12,13,13-Heptacosafluorotridecane	423-66-5	FC(F)C(F)(F)C(F)(F)C(F)(F)C(F)(F)C(F)(F)C(F)(F)C(F)(F)C(F)(F)C(F)(F)C(F)(F)C(F)(F)C(F)(F)F		-9.18	0.69	2.89	P	OUT
QL03455	1,1,1,2,2,3,3,4,4,5,5,6,6,7,7,8,8,9,9,10,10,11,11,12,12-Pentacosafluorododecane	66563-68-6	FC(F)C(F)(F)C(F)(F)C(F)(F)C(F)(F)C(F)(F)C(F)(F)C(F)(F)C(F)(F)C(F)(F)C(F)(F)C(F)(F)F		-8.87	0.69	2.75	P	OUT
QL03456	1-H-Perflurodecane	375-97-3	FC(F)C(F)(F)C(F)(F)C(F)(F)C(F)(F)C(F)(F)C(F)(F)C(F)(F)C(F)(F)C(F)(F)F		-8.35	0.69	2.51	P	IN
QL03457	1H-Perfluorononane	375-94-0	FC(F)C(F)(F)C(F)(F)C(F)(F)C(F)(F)C(F)(F)C(F)(F)C(F)(F)C(F)(F)F		-8.18	0.68	2.45	P	IN
QL03458	2-{[(2,2,3,3,4,4,5,5,6,6,7,7,8,8,9,9-Hexadecafluorononyl)oxy]methyl}oxirane	125370-60-7	FC(F)C(F)(F)C(F)(F)C(F)(F)C(F)(F)C(F)(F)C(F)(F)C(F)(F)COCC1CO1		-8.13	0.66	2.74	P	IN
QL03459	8H-Perfluorooctane	335-65-9	FC(F)C(F)(F)C(F)(F)C(F)(F)C(F)(F)C(F)(F)C(F)(F)C(F)(F)F		-7.72	0.68	2.24	P	IN
QL03460	1H,8H-Perfluorooctane	307-99-3	FC(F)C(F)(F)C(F)(F)C(F)(F)C(F)(F)C(F)(F)C(F)(F)C(F)F		-7.47	0.67	2.23	P	IN
QL03461	1-Hydroperfluoroheptane	NOCAS_871634	FC(F)C(F)(F)C(F)(F)C(F)(F)C(F)(F)C(F)(F)C(F)(F)F		-7.50	0.68	2.16	P	IN
QL03462	(6H-Perfluorohexyl)methyl acrylate	2993-85-3	FC(F)C(F)(F)C(F)(F)C(F)(F)C(F)(F)C(F)(F)COC(=O)C=C		-7.26	0.65	2.51	P	IN
QL03463	1,1,2,2,3,3,4,4,5,5,6,6-Dodecafluoro-7-(trifluoromethoxy)heptane	1737-33-3	FC(F)C(F)(F)C(F)(F)C(F)(F)C(F)(F)C(F)(F)COC(F)(F)F		-7.67	0.67	2.45	P	IN
QL03464	1H-Perfluorohexane	355-37-3	FC(F)C(F)(F)C(F)(F)C(F)(F)C(F)(F)C(F)(F)F		-6.91	0.68	1.90	P	IN
QL03465	1H,6H-Perfluorohexane	336-07-2	FC(F)C(F)(F)C(F)(F)C(F)(F)C(F)(F)C(F)F		-6.57	0.67	1.89	P	IN
QL03466	1,1,1,2,2,3,3,4,4,5,5-Undecafluoropentane	375-61-1	FC(F)C(F)(F)C(F)(F)C(F)(F)C(F)(F)F		-6.61	0.67	1.81	P	IN
QL03467	5H-Perfluoropentanal	2648-47-7	FC(F)C(F)(F)C(F)(F)C(F)(F)C=O		-5.92	0.65	1.79	P	IN
QL03468	Butane, 1-chloro-1,1,2,2,3,3,4,4-octafluoro-	423-31-4	FC(F)C(F)(F)C(F)(F)C(F)(F)Cl		-5.76	0.67	1.46	P	IN
QL03469	1H,1H,5H-Perfluoropentyl acrylate	376-84-1	FC(F)C(F)(F)C(F)(F)C(F)(F)COC(=O)C=C		-6.29	0.63	2.26	P	OUT

QL03470	2,2,4,4,6,6-Hexakis[(2,2,3,3,4,4,5,5-octafluoropentyl)oxy]-1,3,5,2lambda~5~,4lambda~5~,6lambda~5~-triazatriphosphinine	16059-16-8	FC(F)C(F)(F)C(F)(F)C(F)(F)COP1(OCC(F)(F)C(F)(F)C(F)(F)C(F)F)=NP(OCC(F)(F)C(F)(F)C(F)(F)C(F)F)(OCC(F)(F)C(F)(F)C(F)(F)C(F)F)=NP(OCC(F)(F)C(F)(F)C(F)(F)C(F)F)(OCC(F)(F)C(F)(F)C(F)(F)C(F)F)=N1	-8.28	0.66	2.80	P	IN
QL03471	1H-Nonafluorobutane	375-17-7	FC(F)C(F)(F)C(F)(F)C(F)(F)F	-5.76	0.67	1.46	P	IN
QL03472	2,3,3,4,4,5,5-Heptafluoro-1-pentene	1547-26-8	FC(F)C(F)(F)C(F)(F)C(F)=C	-5.25	0.65	1.46	P	IN
QL03473	1H,4H-Octafluorobutane	377-36-6	FC(F)C(F)(F)C(F)(F)C(F)F	-5.25	0.65	1.46	P	IN
QL03474	1,1,2,2,3,3-Hexafluoro-1-(trifluoromethoxy)propane	67490-36-2	FC(F)C(F)(F)C(F)(F)OC(F)(F)F	-6.01	0.65	1.84	P	IN
QL03475	1,1,2,2-Tetrafluoro-3-iodopropane	679-87-8	FC(F)C(F)(F)CI	-4.03	0.61	1.41	P	OUT
QL03476	2,2,3,3-Tetrafluoropropyl acrylate	7383-71-3	FC(F)C(F)(F)COC(=O)C=C	-4.52	0.59	1.89	P	OUT
QL03477	(2H-Perfluoroethyl)(1H,1H,3H-perfluoropropyl)ether	16627-68-2	FC(F)C(F)(F)COC(F)(F)C(F)F	-5.57	0.63	1.84	P	IN
QL03478	Vinyl 1H,1H,3H-perfluoropropyl ether	29819-80-5	FC(F)C(F)(F)COC=C	-4.02	0.58	1.85	P	OUT
QL03479	((2,2,3,3-Tetrafluoropropoxy)methyl)oxirane	19932-26-4	FC(F)C(F)(F)COCC1CO1	-4.88	0.60	1.92	P	OUT
QL03480	2,2,3,3-Tetrafluoropropyl nonafluorobutane-1-sulfonate	66959-14-6	FC(F)C(F)(F)COS(=O)(=O)C(F)(F)C(F)(F)C(F)(F)C(F)(F)F	-7.79	0.67	2.45	P	IN
QL03481	1H,1H,3H-Perfluoropropyl triflate	6401-02-1	FC(F)C(F)(F)COS(=O)(=O)C(F)(F)F	-6.07	0.65	1.83	P	IN
QL03482	1,1,2,2-Tetrafluoro-2-(1,1,2,2-tetrafluoroethoxy)ethane-1-sulfonyl fluoride	104729-49-9	FC(F)C(F)(F)OC(F)(F)C(F)(F)S(F)(=O)=O	-6.56	0.66	1.90	P	IN
QL03483	1,1,2,2-Tetrafluoro-1-(trifluoromethoxy)ethane	2356-61-8	FC(F)C(F)(F)OC(F)(F)F	-4.90	0.63	1.49	P	IN
QL03484	1H,1H,5H-Perfluoropentyl-1,1,2,2-tetrafluoroethylether	16627-71-7	FC(F)C(F)(F)OCC(F)(F)C(F)(F)C(F)(F)C(F)F	-6.95	0.65	2.25	P	IN
QL03485	(1H,1H-Perfluoroethyl)(2H-perfluoroethyl)ether	406-78-0	FC(F)C(F)(F)OCC(F)(F)F	-5.35	0.63	1.78	P	OUT
QL03486	3-(1,1,2,2-tetrafluoroethoxy)propene	1428-33-7	FC(F)C(F)(F)OCC=C	-4.02	0.58	1.85	P	OUT
QL03487	(2H-Perfluoroethoxy)methyloxirane	85567-21-1	FC(F)C(F)(F)OCC1CO1	-4.95	0.61	1.82	P	OUT
QL03488	2,2,4,4,6-Pentakis[(2,2,3,3,4,4,5,5,6,6,7,7-dodecafluoroheptyl)oxy]-6-[(2,2,7,7-tetrafluoroheptyl)oxy]-1,3,5,2lambda~5~,4lambda~5~,6lambda~5~-triazatriphosphinine	3830-74-8	FC(F)CCCCC(F)(F)COP1(OCC(F)(F)C(F)(F)C(F)(F)C(F)(F)C(F)(F)C(F)F)=NP(OCC(F)(F)C(F)(F)C(F)(F)C(F)(F)C(F)(F)C(F)F)(OCC(F)(F)C(F)(F)C(F)(F)C(F)(F)C(F)(F)C(F)F)=NP(OCC(F)(F)C(F)(F)C(F)(F)C(F)(F)C(F)(F)C(F)F)(OCC(F)(F)C(F)(F)C(F)(F)C(F)(F)C(F)(F)C(F)F)=N1	-9.03	0.66	3.17	P	OUT
QL03489	Refrigerant 502	39432-81-0	FC(F)Cl.FC(F)(F)C(F)(F)Cl	-4.02	0.61	1.33	P	OUT
QL03490	2,2-Difluoroethyl nonafluorobutane-1-sulfonate	1036375-28-6	FC(F)COS(=O)(=O)C(F)(F)C(F)(F)C(F)(F)C(F)(F)F	-7.07	0.66	2.24	P	IN
QL03491	5-(Difluoromethoxy)-1,1,1,2,2,3,3,4,4-nonafluoropentane	1661005-79-3	FC(F)OCC(F)(F)C(F)(F)C(F)(F)C(F)(F)F	-6.77	0.65	2.19	P	IN
QL03492	4-(Difluoromethoxy)-1,1,1,2,2,3,3-heptafluorobutane	50428-19-8	FC(F)OCC(F)(F)C(F)(F)C(F)(F)F	-6.00	0.64	1.93	P	IN
QL03493	Perfluoro-2-methyl-12-iodododecane	3248-61-1	FC(I)(F)C(F)(F)C(F)(F)C(F)(F)C(F)(F)C(F)(F)C(F)(F)C(F)(F)C(F)(F)C(F)(F)C(F)(C(F)(F)F)C(F)(F)F	-8.93	0.69	2.72	P	OUT
QL03494	Perfluoro-14-iodo-2-(methyl)tetradecane	3248-63-3	FC(I)(F)C(F)(F)C(F)(F)C(F)(F)C(F)(F)C(F)(F)C(F)(F)C(F)(F)C(F)(F)C(F)(F)C(F)(F)C(F)(F)C(F)(C(F)(F)F)C(F)(F)F	-9.35	0.69	2.93	P	OUT
QL03495	Tripentacontafluoro-26-iodohexacosane	65975-15-7	FC(I)(F)C(F)(F)F	-11.12	0.70	3.78	P	OUT
QL03496	Nonatetracontafluoro-24-iodotetracosane	39823-55-7	FC(I)(F)C(F)(F)F	-10.90	0.70	3.67	P	OUT
QL03497	Pentatetracontafluoro-22-iodododocosane	29809-36-7	FC(I)(F)C(F)(F)F	-10.66	0.70	3.55	P	OUT
QL03498	Perfluorohexadecyl iodide	355-50-0	FC(I)(F)C(F)(F)C(F)(F)C(F)(F)C(F)(F)C(F)(F)C(F)(F)C(F)(F)C(F)(F)C(F)(F)C(F)(F)C(F)(F)C(F)(F)C(F)(F)C(F)(F)C(F)(F)F	-9.79	0.70	3.12	P	OUT
QL03499	Perfluoro-1-iodotetradecane	307-63-1	FC(I)(F)C(F)(F)C(F)(F)C(F)(F)C(F)(F)C(F)(F)C(F)(F)C(F)(F)C(F)(F)C(F)(F)C(F)(F)C(F)(F)C(F)(F)C(F)(F)F	-9.43	0.69	2.95	P	OUT
QL03500	Nonadecafluoro-9-Iodononane	558-97-4	FC(I)(F)C(F)(F)C(F)(F)C(F)(F)C(F)(F)C(F)(F)C(F)(F)C(F)(F)C(F)(F)F	-8.37	0.69	2.44	P	IN
QL03501	1,6-Diiodoperfluorohexane	375-80-4	FC(I)(F)C(F)(F)C(F)(F)C(F)(F)C(F)(F)C(F)(F)I	-7.19	0.69	1.90	P	IN
QL03502	1-Iodoperfluoropentane	638-79-9	FC(I)(F)C(F)(F)C(F)(F)C(F)(F)C(F)(F)F	-6.93	0.69	1.79	P	IN
QL03503	(1E)-1,2,3,4,4,4-Hexafluoro-1-iodo-3-(trifluoromethyl)but-1-ene	883882-12-0	FC(I)=C(/F)C(F)(C(F)(F)F)C(F)(F)F	-5.27	0.65	1.40	P	IN
QL03505	1,2,3,4,4,4-Hexafluoro-1-iodo-3-(trifluoromethyl)but-1-ene	167026-90-6	FC(I)=C(F)C(F)(C(F)(F)F)C(F)(F)F	-5.27	0.65	1.40	P	IN
QL03506	(1Z)-1,2,3,3,4,4,5,5,6,6,7,7,8,8,9,9,10,10,10-Nonadecafluoro-1-iododec-1-ene	56184-40-8	FC(I)=C(F)C(F)(F)C(F)(F)C(F)(F)C(F)(F)C(F)(F)C(F)(F)C(F)(F)C(F)(F)F	-8.15	0.68	2.52	P	IN
QL03507	Perfluorotributylamine	311-89-7	FC(N(C(C(C(C(F)(F)F)(F)F)(F)F)(F)F)C(C(C(C(F)(F)F)(F)F)(F)F)(F)F)(C(C(C(F)(F)F)(F)F)(F)F)F	-8.11	0.69	2.35	P	IN

QL03508	Perfluoromethoxy perfluoropentane sulfonic acid		FC(OC(C(C(C(C(S(=O)(=O)O)(F)F)(F)F)(F)F)(F)F)(F)F)(F)F		-7.58	0.68	2.23	P	IN
QL03509	1,1,2,2,4,4,4-Heptafluoro-3-(iodomethyl)-1-(1,2,2,2-tetrafluoroethoxy)butane	25080-20-0	FC(OC(F)(F)C(F)(F)C(Cl)C(F)(F)F)C(F)(F)F		-6.46	0.64	2.14	P	IN
QL03510	[1,2-Difluoro-2-(heptafluoropropoxy)ethenyl]benzene	854992-57-7	FC(OC(F)(F)C(F)(F)C(F)(F)F)=C(F)C1=CC=CC=C1		-6.61	0.63	2.45	P	OUT
QL03511	Perfluoro-3-(1H-perfluoroethoxy)propane	3330-15-2	FC(OC(F)(F)C(F)(F)C(F)(F)F)C(F)(F)F		-6.60	0.67	1.90	P	IN
QL03512	1-Ethenyl-3-[1,1,2-trifluoro-2-(heptafluoropropoxy)ethoxy]benzene	129051-93-0	FC(OC(F)(F)C(F)(F)C(F)(F)F)C(F)(F)Oc1cccc(C=C)c1		-7.15	0.63	2.69	P	OUT
QL03513	1-Ethenyl-2-[1,1,2-trifluoro-2-(heptafluoropropoxy)ethoxy]benzene	129051-94-1	FC(OC(F)(F)C(F)(F)C(F)(F)F)C(F)(F)Oc1ccccc1C=C		-6.69	0.63	2.47	P	OUT
QL03514	2H-Perfluoro-5-methyl-3,6-dioxanonane	3330-14-1	FC(OC(F)(F)C(F)(OC(F)(F)C(F)(F)C(F)(F)F)C(F)(F)F)C(F)(F)F		-7.79	0.67	2.43	P	IN
QL03515	2H-Perfluoro(5,8-dimethyl-3,6,9-trioxadodecane)	3330-16-3	FC(OC(F)(F)C(F)(OC(F)(F)C(F)(OC(F)(F)C(F)(F)C(F)(F)F)C(F)(F)F)C(F)(F)F)C(F)(F)F		-8.34	0.67	2.67	P	IN
QL03516	Fluoroether E4	26738-51-2	FC(OC(F)(F)C(F)(OC(F)(F)C(F)(OC(F)(F)C(F)(OC(F)(F)C(F)(F)C(F)(F)F)C(F)(F)F)C(F)(F)F)C(F)(F)F)C(F)(F)F		-9.04	0.68	3.01	P	OUT
QL03517	2H-Tricosafluoro-5,8,11,14-tetrakis(trifluoromethyl)-3,6,9,12,15-pentaoxaoctadecane	37486-69-4	FC(OC(F)(F)C(F)(OC(F)(F)C(F)(OC(F)(F)C(F)(OC(F)(F)C(F)(OC(F)(F)C(F)(F)C(F)(F)F)C(F)(F)F)C(F)(F)F)C(F)(F)F)C(F)(F)F)C(F)(F)F		-9.42	0.68	3.19	P	OUT
QL03518	Fluoroether E-6	55154-18-2	FC(OC(F)(F)C(F)(OC(F)(F)C(F)(OC(F)(F)C(F)(OC(F)(F)C(F)(OC(F)(F)C(F)(OC(F)(F)C(F)(F)C(F)(F)F)C(F)(F)F)C(F)(F)F)C(F)(F)F)C(F)(F)F)C(F)(F)F)C(F)(F)F		-9.92	0.68	3.44	P	OUT
QL03519	3,6,9,12,15,18,21-Heptaoxatetracosane, 1,1,1,2,4,4,5,7,7,8,10,10,11,13,13,14,16,16,17,19,19,20,22,22,23,23,24,24,24-nonacosafluoro-5,8,11,14,17,20-hexakis(trifluoromethyl)-	59852-65-2	FC(OC(F)(F)C(F)(OC(F)(F)C(F)(OC(F)(F)C(F)(OC(F)(F)C(F)(OC(F)(F)C(F)(OC(F)(F)C(F)(OC(F)(F)C(F)(F)C(F)(F)F)C(F)(F)F)C(F)(F)F)C(F)(F)F)C(F)(F)F)C(F)(F)F)C(F)(F)F)C(F)(F)F		-10.21	0.68	3.57	P	OUT
QL03520	3,6,9,12,15,18,21,24-Octaoxaheptacosane, 1,1,1,2,4,4,5,7,7,8,10,10,11,13,13,14,16,16,17,19,19,20,22,22,23,25,25,26,26,27,27,27-dotriacontafluoro-5,8,11,14,17,20,23-heptakis(trifluoromethyl)-	27120-63-4	FC(OC(F)(F)C(F)(OC(F)(F)C(F)(OC(F)(F)C(F)(OC(F)(F)C(F)(OC(F)(F)C(F)(OC(F)(F)C(F)(OC(F)(F)C(F)(OC(F)(F)C(F)(F)C(F)(F)F)C(F)(F)F)C(F)(F)F)C(F)(F)F)C(F)(F)F)C(F)(F)F)C(F)(F)F)C(F)(F)F		-10.59	0.68	3.77	P	OUT
QL03521	1,1,1,2,4,4,5,7,7,8,10,10,11,13,13,14,16,16,17,19,19,20,22,22,23,25,25,26,28,28,29,29,30,30,30-Pentatriacontafluoro-5,8,11,14,17,20,23,26-octakis(trifluoromethyl)-3,6,9,12,15,18,21,24,27-nonaoxatriacontane	58979-41-2	FC(OC(F)(F)C(F)(OC(F)(F)C(F)(OC(F)(F)C(F)(OC(F)(F)C(F)(OC(F)(F)C(F)(OC(F)(F)C(F)(OC(F)(F)C(F)(OC(F)(F)C(F)(OC(F)(F)C(F)(F)C(F)(F)F)C(F)(F)F)C(F)(F)F)C(F)(F)F)C(F)(F)F)C(F)(F)F)C(F)(F)F)C(F)(F)F)C(F)(F)F		-10.82	0.68	3.88	P	OUT
QL03522	(1Z)-1,2,3,3,3-Pentafluoro-1-(trifluoromethoxy)prop-1-ene	1620992-19-9	FC(OC(F)(F)F)=C(/F)C(F)(F)F		-5.40	0.63	1.78	P	OUT
QL03523	(1E)-1,2,3,3,3-Pentafluoro-1-(trifluoromethoxy)prop-1-ene	1621181-80-3	FC(OC(F)(F)F)=C(F)C(F)(F)F		-5.40	0.63	1.78	P	OUT
QL03524	4-((Heptadecafluorononenyl)oxy)benzenesulphonyl chloride	59536-15-1	FC(OC1=CC=C(C=C1)S(Cl)(=O)=O)=C(F)C(F)(F)C(F)(F)C(F)(F)C(F)(F)C(F)(F)C(F)(F)C(F)(F)F		-8.76	0.66	3.07	P	IN
QL03525	p-((Undecafluorohexenyl)oxy)benzenesulphonyl chloride	59493-82-2	FC(OC1=CC=C(C=C1)S(Cl)(=O)=O)=C(F)C(F)(F)C(F)(F)C(F)(F)C(F)(F)F		-7.77	0.65	2.74	P	IN
QL03526	(1Z)-1,2,3,3,4,4,5,5,6,6,7,7,8,8,8-Pentadecafluorooct-1-ene	1516885-18-9	FC=C(/F)C(F)(F)C(F)(F)C(F)(F)C(F)(F)C(F)(F)C(F)(F)F		-7.32	0.67	2.24	P	IN
QL03527	(1Z)-1,2,3,3,4,4,5,5,6,6,6-Undecafluorohex-1-ene	168332-72-7	FC=C(/F)C(F)(F)C(F)(F)C(F)(F)C(F)(F)F		-6.37	0.66	1.90	P	IN
QL03528	1,2,3,3,4,4,5,5,6,6,7,7,8,8,8-Pentadecafluorooct-1-ene	7621-51-4	FC=C(F)C(F)(F)C(F)(F)C(F)(F)C(F)(F)C(F)(F)C(F)(F)F		-7.32	0.67	2.24	P	IN
QL03529	1H-Perfluoro-1-heptene	75180-14-2	FC=C(F)C(F)(F)C(F)(F)C(F)(F)C(F)(F)C(F)(F)F		-7.06	0.66	2.17	P	IN
QL03530	1,2,3,3,4,4,5,5,6,6,6-Undecafluorohex-1-ene	66249-21-6	FC=C(F)C(F)(F)C(F)(F)C(F)(F)C(F)(F)F		-6.37	0.66	1.90	P	IN
QL03532	(Z)-1,2,3,3,3-pentafluoropropene	5528-43-8	FC=C(F)C(F)(F)F		-3.89	0.60	1.41	P	OUT
QL03533	(1Z)-1,3,4,4,4-Pentafluoro-3-(trifluoromethyl)but-1-ene	14318-61-7	FC=C/C(F)(C(F)(F)F)C(F)(F)F		-4.78	0.63	1.42	P	IN
QL03534	(1E)-1,3,4,4,4-Pentafluoro-3-(trifluoromethyl)but-1-ene	14149-41-8	FC=CC(F)(C(F)(F)F)C(F)(F)F		-4.78	0.63	1.42	P	IN
QL03535	Perfluoroglutaric anhydride	376-68-1	FC1(F)C(=O)OC(=O)C(F)(F)C1(F)F		-5.48	0.67	1.30	P	IN
QL03536	Perfluorosuccinic anhydride	699-30-9	FC1(F)C(=O)OC(=O)C1(F)F		-4.65	0.66	0.99	P	IN
QL03537	1,2-Dichloroperfluorocyclohexene	336-19-6	FC1(F)C(Cl)=C(Cl)C(F)(F)C(F)(F)C1(F)F		-5.16	0.68	0.95	P	IN
QL03538	Perfluorocyclohexanecarbonyl bromide	261761-76-6	FC1(F)C(F)(F)C(F)(F)C(F)(C(Br)=O)C(F)(F)C1(F)F		-6.29	0.69	1.44	P	IN
QL03539	Chloroperfluorocyclohexane	336-15-2	FC1(F)C(F)(F)C(F)(F)C(F)(Cl)C(F)(F)C1(F)F		-5.62	0.70	0.92	P	OUT
QL03540	(Perfluorocyclohexyl)methyl prop-2-enoate	40677-94-9	FC1(F)C(F)(F)C(F)(F)C(F)(COC(=O)C=C)C(F)(F)C1(F)F		-7.02	0.66	2.17	P	IN
QL03541	Perfluorocyclohexane	355-68-0	FC1(F)C(F)(F)C(F)(F)C(F)(F)C(F)(F)C1(F)F		-5.62	0.70	0.92	P	OUT
QL03542	Piperidine, 2,2,3,3,4,4,5,5,6,6-decafluoro-1-(pentafluoroethyl)-	564-11-4	FC1(F)C(F)(F)C(F)(F)C(F)(F)C(N1C(F)(F)C(F)(F)F)(F)F		-7.32	0.69	1.91	P	IN
QL03543	Decafluorocyclopentane	376-77-2	FC1(F)C(F)(F)C(F)(F)C(F)(F)C1(F)F		-5.62	0.70	0.92	P	OUT
QL03544	(Perfluorocyclopentyl)phenyldiazene	97388-28-8	FC1(F)C(F)(F)C(F)(F)C(F)(N=NC2=CC=CC=C2)C1(F)F		-6.55	0.65	2.13	P	IN

QL03545	Octafluorocyclobutane	115-25-3	FC1(F)C(F)(F)C(F)(F)C1(F)F		-5.62	0.70	0.92	P	OUT
QL03546	Tetracosafluorotetradecahydrophenanthrene	306-91-2	FC1(F)C(F)(F)C(F)(F)C2(F)C(F)(C1(F)F)C(F)(F)C(F)(F)C1(F)C(F)(F)C(F)(F)C(F)(F)C(F)(F)C21F		-8.00	0.70	2.08	P	IN
QL03547	Perflunafene	306-94-5	FC1(F)C(F)(F)C(F)(F)C2(F)C(F)(F)C(F)(F)C(F)(F)C(F)(F)C2(F)C1(F)F		-7.40	0.70	1.79	P	IN
QL03548	4,4,5,5,6,6-hexafluoro-1,3,2-dithiazinane 1,1,3,3-tetraoxide	84246-29-7	FC1(F)C(F)(F)S(=O)(=O)NS(=O)(=O)C1(F)F		-6.05	0.69	1.27	P	IN
QL03549	1H,2H-Hexafluorocyclopentene	1005-73-8	FC1(F)C=CC(F)(F)C1(F)F		-5.74	0.68	1.32	P	IN
QL03550	Perfluoroperhydrofluorene	307-08-4	FC1(F)C2(F)C(F)(C3(F)C1(F)C(F)(F)C(F)(F)C(F)(F)C3(F)F)C(F)(F)C(F)(F)C(F)(F)C2(F)F		-7.74	0.70	1.93	P	IN
QL03551	2,2,3,3-Tetrafluorooxetane	765-63-9	FC1(F)COC1(F)F		-4.49	0.67	0.81	P	IN
QL03552	Perfluoro(oxacyclopentane)	773-14-8	FC1(F)OC(F)(F)C(F)(F)C1(F)F		-5.38	0.69	0.96	P	OUT
QL03553	Perfluoro-15-crown-5-ether	97571-69-2	FC1(F)OC(F)(F)C(F)(F)OC(F)(F)C(F)(F)OC(F)(F)C(F)(F)OC(F)(F)C(F)(F)OC1(F)F		-5.16	0.68	0.99	P	IN
QL03554	6-Isocyanato-2,2,4,4-tetrefluoro-1,3-benzodioxene	26030-46-6	FC1(F)OC2=C(C=C(C=C2)N=C=O)C(F)(F)O1		-5.59	0.60	2.26	P	OUT
QL03555	Tetrafluoroethane beta-sultone	697-18-7	FC1(F)OS(=O)(=O)C1(F)F		-6.07	0.69	1.36	P	IN
QL03556	2,2,4,4-Tetrafluoro-1,3-dithietane	1717-50-6	FC1(F)SC(F)(F)S1		-5.26	0.65	1.50	P	IN
QL03557	2,2,3,4,4,5,5,6,6-Decafluoro-1-(trifluoromethyl)piperidine	359-71-7	FC1(N(C(C(F)(F)C(C1(F)F)(F)F)(F)F)C(F)(F)F)F		-6.40	0.69	1.50	P	IN
QL03558	1-Chloroheptafluorocyclopentene	1759-63-3	FC1=C(Cl)C(F)(F)C(F)(F)C1(F)F		-5.05	0.68	0.96	P	IN
QL03559	Perfluoro(4-isopropyltoluene)	20017-49-6	FC1=C(F)C(=C(F)C(F)=C1C(F)(F)F)C(F)(C(F)(F)F)C(F)(F)F		-6.80	0.65	2.16	P	IN
QL03560	Decafluorocyclohexene	355-75-9	FC1=C(F)C(F)(F)C(F)(F)C(F)(F)C1(F)F		-5.16	0.68	0.95	P	IN
QL03561	Octafluorocyclopentene	559-40-0	FC1=C(F)C(F)(F)C(F)(F)C1(F)F		-5.05	0.68	0.96	P	IN
QL03562	Hexafluorocyclobutene	697-11-0	FC1=C(F)C(F)(F)C1(F)F		-4.88	0.67	0.97	P	IN
QL03563	1,2,3,4,5-Pentafluoro-6-(heptadecafluorooctyl)benzene	54387-09-6	FC1=C(F)C(F)=C(C(F)=C1F)C(F)(F)C(F)(F)C(F)(F)C(F)(F)C(F)(F)C(F)(F)C(F)(F)C(F)(F)F		-8.42	0.67	2.73	P	IN
QL03564	Perfluorotetralin	2342-07-6	FC1=C(F)C2=C(C(F)=C1F)C(F)(F)C(F)(F)C(F)(F)C2(F)F		-6.40	0.67	1.79	P	IN
QL03565	Octafluoronaphthalene	313-72-4	FC1=C(F)C2=C(C(F)=C1F)C(F)=C(F)C(F)=C2F		-5.33	0.62	1.84	P	OUT
QL03566	Cyclopentene, 1,3,3,4,4,5,5-heptafluoro-	1892-03-1	FC1=CC(F)(F)C(F)(F)C1(F)F		-5.07	0.68	0.98	P	IN
QL03567	Undecafluorocyclohexane	308-24-7	FC1C(F)(F)C(F)(F)C(F)(F)C(F)(F)C1(F)F		-5.40	0.69	0.94	P	OUT
QL03568	1-Bromo-2,3,5,6-tetrafluoro-4-{1,1,2,3,3,3-hexafluoro-2-[1,1,2,3,3,3-hexafluoro-2-(heptafluoropropoxy)propoxy]propyl}benzene	60799-27-1	Fc1c(F)c(c(F)c(F)c1Br)C(F)(F)C(F)(OC(F)(F)C(F)(OC(F)(F)C(F)(F)C(F)(F)F)C(F)(F)F)C(F)(F)F		-8.40	0.67	2.80	P	IN
QL03569	2,3,3,3-Tetrafluoro-1-[2,3,5,6-tetrafluoro-4-(1,1,1,2,3,3,3-heptafluoropropan-2-yl)phenyl]-2-(trifluoromethyl)propan-1-one	19547-30-9	Fc1c(F)c(c(F)c(F)c1C(=O)C(F)(C(F)(F)F)C(F)(F)F)C(F)(C(F)(F)F)C(F)(F)F		-7.34	0.66	2.35	P	IN
QL03570	1,2,4,5-Tetrafluoro-3-(1,1,1,2,3,3,3-heptafluoropropan-2-yl)-6-(trichloromethyl)benzene	136264-73-8	Fc1c(F)c(c(F)c(F)c1C(Cl)(Cl)Cl)C(F)(C(F)(F)F)C(F)(F)F		-6.80	0.65	2.16	P	IN
QL03571	2,3,5,6-Tetrafluoro-4-(1,1,1,2,3,3,3-heptafluoropropan-2-yl)benzonitrile	20017-54-3	Fc1c(F)c(c(F)c(F)c1C#N)C(F)(C(F)(F)F)C(F)(F)F		-6.48	0.64	2.22	P	IN
QL03572	5,10,15,20-Tetrakis{2,3,5,6-tetrafluoro-4-[(3,3,4,4,5,5,6,6,7,7,8,8,9,9,10,10,11,11,12,12,12-henicosafluorododecyl)sulfanyl]phenyl}porphyrin	956790-67-3	Fc1c(F)c(c(F)c(F)c1SCCC(F)(F)C(F)(F)C(F)(F)C(F)(F)C(F)(F)C(F)(F)C(F)(F)C(F)(F)C(F)(F)C(F)(F)F)-<br>c1c2Nc(cc2)c(c2C=Cc(n2)c(c2Nc(cc2)c(c2C=Cc1n2)-<br>c1c(F)c(F)c(SCCC(F)(F)C(F)(F)C(F)(F)C(F)(F)C(F)(F)C(F)(F)C(F)(F)C(F)(F)C(F)(F)C(F)(F)F)c(F)c1F)-<br>c1c(F)c(F)c(SCCC(F)(F)C(F)(F)C(F)(F)C(F)(F)C(F)(F)C(F)(F)C(F)(F)C(F)(F)C(F)(F)C(F)(F)F)c(F)c1F)-<br>c1c(F)c(F)c(SCCC(F)(F)C(F)(F)C(F)(F)C(F)(F)C(F)(F)C(F)(F)C(F)(F)C(F)(F)C(F)(F)C(F)(F)F)c(F)c1F		-11.10	0.66	4.24	P	OUT
QL03573	3-(Pentadecafluoroheptyl)-5-(pentafluorophenyl)-1,2,4-oxadiazole	919489-99-9	Fc1c(F)c(F)c(-c2nc(no2)C(F)(F)C(F)(F)C(F)(F)C(F)(F)C(F)(F)C(F)(F)C(F)(F)F)c(F)c1F		-8.51	0.66	2.93	P	IN
QL03574	2,2,3,3,4,4,5,5,5-Nonafluoro-1-(pentafluorophenyl)pentan-1-one	1610931-27-5	Fc1c(F)c(F)c(C(=O)C(F)(F)C(F)(F)C(F)(F)C(F)(F)F)c(F)c1F		-7.35	0.66	2.41	P	IN
QL03575	2,2,3,3,4,4,4-Heptafluoro-1-(pentafluorophenyl)butan-1-one	19108-37-3	Fc1c(F)c(F)c(C(=O)C(F)(F)C(F)(F)C(F)(F)F)c(F)c1F		-6.81	0.65	2.21	P	IN
QL03576	2,3,3,3-Tetrafluoro-2-[1,1,2,3,3,3-hexafluoro-2-(heptafluoropropoxy)propoxy]-1-(pentafluorophenyl)propan-1-one	70627-97-3	Fc1c(F)c(F)c(C(=O)C(F)(OC(F)(F)C(F)(OC(F)(F)C(F)(F)C(F)(F)F)C(F)(F)F)C(F)(F)F)c(F)c1F		-8.31	0.66	2.82	P	IN
QL03577	1,2,3,4-Tetrafluoro-5-(1,1,1,2,3,3,3-heptafluoropropan-2-yl)-6-(trifluoromethyl)benzene	112754-12-8	Fc1c(F)c(F)c(c(c1F)C(F)(F)F)C(F)(C(F)(F)F)C(F)(F)F		-6.28	0.65	1.90	P	IN
QL03578	1,2,3,4,5-Pentafluoro-6-(1,1,1,2,3,3,3-heptafluoropropan-2-yl)benzene	17480-16-9	Fc1c(F)c(F)c(c(F)c1F)C(F)(C(F)(F)F)C(F)(F)F		-6.25	0.65	1.94	P	IN

QL03579	1,2,3,4,5-Pentafluoro-6-(nonafluorobutyl)benzene	19108-38-4	Fc1c(F)c(F)c(c(F)c1F)C(F)(F)C(F)(F)C(F)(F)C(F)(F)F		-7.04	0.66	2.22	P	IN
QL03580	1,2,3,4,5-Pentafluoro-6-(heptafluoropropyl)benzene	54326-26-0	Fc1c(F)c(F)c(c(F)c1F)C(F)(F)C(F)(F)C(F)(F)F		-6.67	0.65	2.11	P	IN
QL03581	2,3,3,3-Tetrafluoro-N-(pentafluorophenyl)-2-(trifluoromethyl)propanamide	105923-37-3	Fc1c(F)c(F)c(NC(=O)C(F)(C(F)(F)F)C(F)(F)F)c(F)c1F		-6.61	0.65	2.19	P	IN
QL03582	2,2,3,3,4,4,4-Heptafluoro-N-(pentafluorophenyl)butanamide	60839-98-7	Fc1c(F)c(F)c(NC(=O)C(F)(F)C(F)(F)C(F)(F)F)c(F)c1F		-7.03	0.65	2.37	P	IN
QL03583	1,2,3,4,5-Pentafluoro-6-(pentafluoroethoxy)benzene	159752-92-8	Fc1c(F)c(F)c(OC(F)(F)C(F)(F)F)c(F)c1F		-6.44	0.64	2.15	P	IN
QL03584	N-(2,2,3,3,4,4,5,5,6,6,7,7,8,8,8-Pentadecafluorooctyl)-P,P-bis(pentafluorophenyl)phosphinous amide	518358-34-4	Fc1c(F)c(F)c(P(NCC(F)(F)C(F)(F)C(F)(F)C(F)(F)C(F)(F)C(F)(F)C(F)(F)F)c2c(F)c(F)c(F)c(F)c2F)c(F)c1F		-8.14	0.65	2.84	P	IN
QL03585	2,3,4,5,8-Pentafluoro-8-(1,1,1,2,3,3,3-heptafluoropropan-2-yl)bicyclo[4.2.0]octa-1,3,5-trien-7-one	1419865-30-7	Fc1c2C(=O)C(F)(c2c(F)c(F)c1F)C(F)(C(F)(F)F)C(F)(F)F		-6.86	0.66	2.14	P	IN
QL03586	3,4,5,6,7-Pentafluoro-3-(1,1,1,2,3,3,3-heptafluoropropan-2-yl)-2-benzofuran-1(3H)-one	1419865-31-8	Fc1c2C(=O)OC(F)(c2c(F)c(F)c1F)C(F)(C(F)(F)F)C(F)(F)F		-6.76	0.65	2.15	P	IN
QL03587	2,3,4,5,7,7,8-Heptafluoro-8-(1,1,1,2,3,3,3-heptafluoropropan-2-yl)bicyclo[4.2.0]octa-1,3,5-triene	103634-36-2	Fc1c2c(c(F)c(F)c1F)C(F)(C(F)(C(F)(F)F)C(F)(F)F)C2(F)F		-7.02	0.66	2.13	P	IN
QL03588	4,5,6,7-Tetrafluoro-3-(nonafluorobutyl)-1H-indazole	1610931-28-6	Fc1c2Nnc(c2c(F)c(F)c1F)C(F)(F)C(F)(F)C(F)(F)C(F)(F)F		-7.33	0.66	2.42	P	IN
QL03589	4,5,6,7-Tetrafluoro-3-(heptafluoropropyl)-1H-indazole	1610931-26-4	Fc1c2Nnc(c2c(F)c(F)c1F)C(F)(F)C(F)(F)C(F)(F)F		-6.88	0.65	2.27	P	IN
QL03590	1H,1H,2H-Perfluorocyclopentane	15290-77-4	FC1CC(F)(F)C(F)(F)C1(F)F		-5.07	0.68	0.98	P	IN
QL03591	1,2,4,5-Tetrafluoro-3-(heptafluoropropyl)benzene	153817-18-6	Fc1cc(F)c(F)c(c1F)C(F)(F)C(F)(F)C(F)(F)F		-6.14	0.65	1.88	P	IN
QL03592	4,4,5,5,6,6,6-Heptafluoro-1-(4-fluorophenyl)hexane-1,3-dione	70862-62-3	Fc1ccc(cc1)C(=O)CC(=O)C(F)(F)C(F)(F)C(F)(F)F		-6.95	0.63	2.53	P	OUT
QL03593	1-Fluoro-4-(1,1,1,2,3,3,3-heptafluoropropan-2-yl)benzene	2396-25-0	Fc1ccc(cc1)C(F)(C(F)(F)F)C(F)(F)F		-5.86	0.64	1.90	P	IN
QL03594	1-Fluoro-4-(1,1,2,2,3,3,4,4,5,5,6,6,6-tridecafluorohexane-1-sulfonyl)benzene	149652-30-2	Fc1ccc(cc1)S(=O)(=O)C(F)(F)C(F)(F)C(F)(F)C(F)(F)C(F)(F)C(F)(F)F		-8.17	0.67	2.69	P	IN
QL03595	N-[4-(3,4-Difluorophenyl)-1,3-thiazol-2-yl]-2,2,3,3,4,4,4-heptafluorobutanamide	299198-90-6	Fc1ccc(cc1F)-c1csc(NC(=O)C(F)(F)C(F)(F)C(F)(F)F)n1		-7.34	0.63	2.75	P	OUT
QL03596	2,2,3,3,4,4,5,5,6,6,7,7,8,8,9,9,9-Heptadecafluoro-N-(4-fluorophenyl)nonanamide	344282-18-4	Fc1ccc(NC(=O)C(F)(F)C(F)(F)C(F)(F)C(F)(F)C(F)(F)C(F)(F)C(F)(F)C(F)(F)F)cc1		-8.57	0.66	2.95	P	IN
QL03597	2,2,3,3,4,4,4-Heptafluoro-N-(4-fluorophenyl)butanamide	550301-92-3	Fc1ccc(NC(=O)C(F)(F)C(F)(F)C(F)(F)F)cc1		-6.81	0.64	2.44	P	OUT
QL03598	N-(2,4-Difluorophenyl)-1,1,2,2,3,3,4,4,4-nonafluorobutane-1-sulfonamide	23375-29-3	Fc1ccc(NS(=O)(=O)C(F)(F)C(F)(F)C(F)(F)C(F)(F)F)c(F)c1		-7.50	0.65	2.52	P	IN
QL03599	2,2,3,3,4,4,5,5,6,6,7,7,8,8,9,9,9-Heptadecafluoro-N-(3-fluorophenyl)nonanamide	541514-85-6	Fc1cccc(NC(=O)C(F)(F)C(F)(F)C(F)(F)C(F)(F)C(F)(F)C(F)(F)C(F)(F)C(F)(F)F)c1		-8.45	0.66	2.90	P	IN
QL03600	2,2,3,3,4,4,4-Heptafluoro-N-(3-fluorophenyl)butanamide	332031-88-6	Fc1cccc(NC(=O)C(F)(F)C(F)(F)C(F)(F)F)c1		-6.38	0.63	2.24	P	OUT
QL03601	2,2,3,3,4,4,5,5,6,6,7,7,8,8,9,9,9-Heptadecafluoro-N-(2-fluorophenyl)nonanamide	344282-15-1	Fc1ccccc1NC(=O)C(F)(F)C(F)(F)C(F)(F)C(F)(F)C(F)(F)C(F)(F)C(F)(F)C(F)(F)F		-8.44	0.66	2.89	P	IN
QL03602	2,4-Difluoro-6-(1,1,1,2,3,3,3-heptafluoropropan-2-yl)-1,3,5-triazine	10271-35-9	Fc1nc(F)nc(n1)C(F)(C(F)(F)F)C(F)(F)F		-5.46	0.64	1.68	P	IN
QL03603	2-Fluoro-4,6-bis(1,1,1,2,3,3,3-heptafluoropropan-2-yl)-1,3,5-triazine	10271-36-0	Fc1nc(nc(n1)C(F)(C(F)(F)F)C(F)(F)F)C(F)(C(F)(F)F)C(F)(F)F		-6.64	0.66	1.98	P	IN
QL03604	2,2,3,3,4,4,5,5,6,6,7,7,8,8,9,9,10,10,11,11-Icosafluoroundecyl acrylate	4998-38-3	FCC(F)(F)C(F)(F)C(F)(F)C(F)(F)C(F)(F)C(F)(F)C(F)(F)C(F)(F)C(F)(F)C(F)OC(=O)C=C		-8.44	0.66	2.90	P	IN
QL03605	1,1,1,2,2,3,3,4-octafluorobutane	662-35-1	FCC(F)(F)C(F)(F)C(F)(F)F		-5.48	0.66	1.46	P	IN
QL03606	1,1,1,2,2,3,3,4,4,5,5,6,6,8-Tetradecafluorooctane	361532-17-4	FCCC(F)(F)C(F)(F)C(F)(F)C(F)(F)C(F)(F)C(F)(F)F		-7.16	0.66	2.23	P	IN
QL03607	Pentafluorosulfide Perfluoroalkyl (linear) sulfonic acids, n=9		FS(F)(F)(F)(F)C(F)(F)C(F)(F)C(F)(F)C(F)(F)C(F)(F)C(F)(F)C(F)(F)C(F)(F)C(F)(F)S(O)(=O)=O		-9.22	0.71	2.67	P	OUT
QL03608	Pentafluorosulfide Perfluoroalkyl (linear) sulfonic acids, n=8		FS(F)(F)(F)(F)C(F)(F)C(F)(F)C(F)(F)C(F)(F)C(F)(F)C(F)(F)C(F)(F)C(F)(F)S(O)(=O)=O		-8.91	0.71	2.50	P	OUT
QL03609	Pentafluorosulfide Perfluoroalkyl (linear) sulfonic acids, n=7		FS(F)(F)(F)(F)C(F)(F)C(F)(F)C(F)(F)C(F)(F)C(F)(F)C(F)(F)C(F)(F)S(O)(=O)=O		-8.77	0.71	2.42	P	IN
QL03610	Pentafluorosulfide Perfluoroalkyl (linear) sulfonic acids, n=6		FS(F)(F)(F)(F)C(F)(F)C(F)(F)C(F)(F)C(F)(F)C(F)(F)C(F)(F)S(O)(=O)=O		-8.40	0.71	2.22	P	IN
QL03611	Pentafluorosulfide Perfluoroalkyl (linear) sulfonic acids, n=5		FS(F)(F)(F)(F)C(F)(F)C(F)(F)C(F)(F)C(F)(F)C(F)(F)S(O)(=O)=O		-8.24	0.71	2.12	P	IN

QL03612	Pentafluorosulfide Perfluoroalkyl (linear) sulfonic acids, n=4		FS(F)(F)(F)(F)C(F)(F)C(F)(F)C(F)(F)C(F)(F)S(O)(=O)=O		-7.78	0.71	1.87	P	OUT
QL03613	Pentafluorosulfide Perfluoroalkyl (linear) sulfonic acids, n=3		FS(F)(F)(F)(F)C(F)(F)C(F)(F)C(F)(F)S(O)(=O)=O		-7.59	0.71	1.75	P	OUT
QL03614	Ammonium perfluorobutanoate	10495-86-0	N.[O-]C(=O)C(F)(F)C(F)(F)C(F)(F)F		-4.97	0.62	1.73	P	OUT
QL03615	perfluoropropanesulfonamido ethanol, n =3		N(CCO)S(=O)(=O)C(F)(F)C(F)(F)C(F)(F)F		-6.48	0.64	2.21	P	IN
QL03616	perfluoroethanesulfonamido ethanol, n =2		N(CCO)S(=O)(=O)C(F)(F)C(F)(F)F		-5.63	0.63	1.95	P	OUT
QL03617	4,4,5,5,6,6,6-Heptafluoro-L-norleucine	148043-94-1	N[C@@H](CC(F)(F)C(F)(F)C(F)(F)F)C(O)=O		-6.19	0.65	1.92	P	IN
QL03618	Isooctanesulfonamide, heptadecafluoro-	93894-56-5	N[S](=O)(=O)C(F)(F)C(F)(F)C(F)(F)C(F)(F)C(F)(F)C(F)(C(F)(F)F)C(F)(F)F		-7.82	0.69	2.20	P	IN
QL03619	(S)-Perfluorobutanesulfinamide	1303994-31-1	N[S@](=O)C(F)(F)C(F)(F)C(F)(F)C(F)(F)F		-6.61	0.67	1.81	P	IN
QL03620	3,3,4,4,5,5,6,6,6-Nonafluoro-1-nitrohex-1-en-2-amine	223260-92-2	NC(=C[N+]([O-])=O)C(F)(F)C(F)(F)C(F)(F)C(F)(F)F		-6.82	0.65	2.18	P	IN
QL03621	3-Amino-4,4,5,5,6,6,6-heptafluoro-1-phenylhex-2-en-1-one	91508-85-9	NC(=CC(=O)C1=CC=CC=C1)C(F)(F)C(F)(F)C(F)(F)F		-6.58	0.63	2.43	P	OUT
QL03622	Perfluorooctanamidine	307-31-3	NC(=N)C(F)(F)C(F)(F)C(F)(F)C(F)(F)C(F)(F)C(F)(F)C(F)(F)F		-7.72	0.68	2.24	P	IN
QL03623	2,2,3,3,4,4,5,5,6,6,7,7,7-Tridecafluoroheptanimidamide	25264-81-7	NC(=N)C(F)(F)C(F)(F)C(F)(F)C(F)(F)C(F)(F)C(F)(F)F		-7.50	0.68	2.16	P	IN
QL03624	2,2,3,3,4,4,5,5,6,6,6-Undecafluorohexanimidamide	464-21-1	NC(=N)C(F)(F)C(F)(F)C(F)(F)C(F)(F)C(F)(F)F		-6.91	0.68	1.90	P	IN
QL03625	Perfluorobutyrylamidine	375-19-9	NC(=N)C(F)(F)C(F)(F)C(F)(F)F		-5.76	0.67	1.46	P	IN
QL03626	2-(2,2,3,3,4,4,4-Heptafluorobutanoyl)hydrazine-1-carboximidamide	25978-98-7	NC(=N)NNC(=O)C(F)(F)C(F)(F)C(F)(F)F		-6.40	0.64	2.18	P	IN
QL03627	(1Z)-2,2,3,3,4,4,4-Heptafluoro-N'-(2,2,3,3,4,4,4-heptafluorobutanimidoyl)butanimidamide	648-13-5	NC(=N/C(=N)C(F)(F)C(F)(F)C(F)(F)F)C(F)(F)C(F)(F)C(F)(F)F		-7.72	0.67	2.38	P	IN
QL03628	(1r,4r)-4-(Nonafluorobutyl)cyclohexane-1-carboxamide	88951-21-7	NC(=O)[C@H]1CC[C@@H](CC1)C(F)(F)C(F)(F)C(F)(F)C(F)(F)F		-7.21	0.65	2.46	P	IN
QL03629	(1r,4r)-4-(Heptafluoropropyl)cyclohexane-1-carboxamide	88951-19-3	NC(=O)[C@H]1CC[C@@H](CC1)C(F)(F)C(F)(F)C(F)(F)F		-6.66	0.64	2.29	P	IN
QL03630	(1s,4s)-4-(Nonafluorobutyl)cyclohexane-1-carboxamide	88951-20-6	NC(=O)[C@H]1CC[C@H](CC1)C(F)(F)C(F)(F)C(F)(F)C(F)(F)F		-7.21	0.65	2.46	P	IN
QL03631	(1s,4s)-4-(Heptafluoropropyl)cyclohexane-1-carboxamide	88951-18-2	NC(=O)[C@H]1CC[C@H](CC1)C(F)(F)C(F)(F)C(F)(F)F		-6.66	0.64	2.29	P	IN
QL03632	2,3,3,3-Tetrafluoro-2-(trifluoromethyl)propanamide	662-20-4	NC(=O)C(F)(C(F)(F)F)C(F)(F)F		-5.20	0.66	1.24	P	IN
QL03633	nonadecafluorodecanamide	307-40-4	NC(=O)C(F)(F)C(F)(F)C(F)(F)C(F)(F)C(F)(F)C(F)(F)C(F)(F)C(F)(F)C(F)(F)F		-8.35	0.69	2.51	P	IN
QL03634	2,2,3,3,4,4,5,5,6,6,7,7,8,8,9,9,9-Heptadecafluorononanamide	82854-34-0	NC(=O)C(F)(F)C(F)(F)C(F)(F)C(F)(F)C(F)(F)C(F)(F)C(F)(F)C(F)(F)F		-8.18	0.68	2.45	P	IN
QL03635	Perfluorooctanamide	423-54-1	NC(=O)C(F)(F)C(F)(F)C(F)(F)C(F)(F)C(F)(F)C(F)(F)C(F)(F)F		-7.72	0.68	2.24	P	IN
QL03636	Perfluorooctanedioic diamide	3492-23-7	NC(=O)C(F)(F)C(F)(F)C(F)(F)C(F)(F)C(F)(F)C(F)(F)C(N)=O		-7.47	0.67	2.23	P	IN
QL03637	Perfluoroheptanamide	2358-22-7	NC(=O)C(F)(F)C(F)(F)C(F)(F)C(F)(F)C(F)(F)C(F)(F)F		-7.50	0.68	2.16	P	IN
QL03638	2,2,3,3,4,4,5,5,6,6,6-Undecafluorohexanamide	335-54-6	NC(=O)C(F)(F)C(F)(F)C(F)(F)C(F)(F)C(F)(F)F		-6.91	0.68	1.90	P	IN
QL03639	2,2,3,3,4,4,5,5,6,6-Decafluoro-6-[(1,1,1,2,3,3,3-heptafluoropropan-2-yl)oxy]hexanamide	29952-92-9	NC(=O)C(F)(F)C(F)(F)C(F)(F)C(F)(F)C(F)(F)OC(F)(C(F)(F)F)C(F)(F)F		-7.95	0.68	2.41	P	IN
QL03640	Octafluoroadipamide	355-66-8	NC(=O)C(F)(F)C(F)(F)C(F)(F)C(F)(F)C(N)=O		-6.57	0.67	1.89	P	IN
QL03641	Nonafluoropentanamide	13485-61-5	NC(=O)C(F)(F)C(F)(F)C(F)(F)C(F)(F)F		-6.61	0.67	1.81	P	IN
QL03642	Perfluoropentanamide	355-81-7	NC(=O)C(F)(F)C(F)(F)C(F)(F)C(F)F		-6.21	0.66	1.81	P	IN
QL03643	Heptafluorobutyramide	662-50-0	NC(=O)C(F)(F)C(F)(F)C(F)(F)F		-5.76	0.67	1.46	P	IN
QL03644	Pentafluoropropionamide	354-76-7	NC(=O)C(F)(F)C(F)(F)F		-5.29	0.66	1.36	P	IN
QL03645	Propanamide, 3-[1-[difluoro[(1,2,2-trifluoroethenyl)oxy]methyl]-1,2,2,2-tetrafluoroethoxy]-2,2,3,3-tetrafluoro-	69804-18-8	NC(=O)C(F)(F)C(F)(F)OC(F)(C(F)(F)F)C(F)(F)OC(F)=C(F)F		-7.28	0.65	2.43	P	IN
QL03646	2,2,3,3-Tetrafluoropropionamide	2069-86-5	NC(=O)C(F)(F)C(F)F		-4.61	0.63	1.39	P	OUT
QL03647	Perfluoro-2(1-propoxy)perfluoropropanamide	13140-35-7	NC(=O)C(F)(OC(F)(F)C(F)(F)C(F)(F)F)C(F)(F)F		-6.59	0.67	1.85	P	IN
QL03648	2,3,3,3-Tetrafluoro-2-[1,1,2,3,3,3-hexafluoro-2-(heptafluoropropoxy)propoxy]propanamide	60308-67-0	NC(=O)C(F)(OC(F)(F)C(F)(OC(F)(F)C(F)(F)C(F)(F)F)C(F)(F)F)C(F)(F)F		-7.79	0.67	2.40	P	IN
QL03649	2,3,3,3-Tetrafluoro-2-{1,1,2,3,3,3-hexafluoro-2-[1,1,2,3,3,3-hexafluoro-2-(heptafluoropropoxy)propoxy]propoxy}propanamide	51445-02-4	NC(=O)C(F)(OC(F)(F)C(F)(OC(F)(F)C(F)(OC(F)(F)C(F)(F)C(F)(F)F)C(F)(F)F)C(F)(F)F)C(F)(F)F		-8.36	0.68	2.67	P	IN
QL03650	2,3,3,3-Tetrafluoro-2-(trifluoromethoxy)propanamide	67710-48-9	NC(=O)C(F)(OC(F)(F)F)C(F)(F)F		-5.07	0.65	1.38	P	IN
QL03651	Flupoxam	119126-15-7	NC(=O)c1nc(-c2ccccc2)n(n1)-c1ccc(Cl)c(COCC(F)(F)C(F)(F)F)c1		-6.85	0.61	2.88	P	OUT

QL03652	1,1,1,2,3,3,4,4,5,5,6,6,7,7,8,8,8-Heptadecafluorooctan-2-yl carbamate	92681-65-7	NC(=O)OC(F)(C(F)(F)F)C(F)(F)C(F)(F)C(F)(F)C(F)(F)C(F)(F)C(F)(F)F		-7.86	0.67	2.43	P	IN
QL03653	2,2,3,3,4,4,4-Heptafluorobutanethioamide	375-20-2	NC(=S)C(F)(F)C(F)(F)C(F)(F)F		-5.76	0.67	1.46	P	IN
QL03654	2-(Perfluoropropyl)glycine	26208-02-6	NC(C(O)=O)C(F)(F)C(F)(F)C(F)(F)F		-6.21	0.66	1.83	P	IN
QL03655	4,4,5,5,6,6,6-Heptafluoronorleucine	15960-01-7	NC(CC(F)(F)C(F)(F)C(F)(F)F)C(O)=O		-6.19	0.65	1.92	P	IN
QL03656	3-Amino-4,4,5,5,6,6,6-heptafluorohexanoic acid	150618-42-1	NC(CC(O)=O)C(F)(F)C(F)(F)C(F)(F)F		-6.07	0.65	1.92	P	IN
QL03657	2-Amino-3-(perfluoropropyl)-1-phenyl-3-propanol	322392-46-1	NC(CC1=CC=CC=C1)C(O)C(F)(F)C(F)(F)C(F)(F)F		-6.60	0.63	2.45	P	OUT
QL03658	2-Amino-5,5,6,6,7,7,8,8,8-nonafluorooctanoic acid	125162-63-2	NC(CCC(F)(F)C(F)(F)C(F)(F)C(F)(F)F)C(O)=O		-6.93	0.65	2.26	P	IN
QL03659	2-Amino-5,5,6,6,7,7,7-heptafluoroheptanoic acid	10325-10-7	NC(CCC(F)(F)C(F)(F)C(F)(F)F)C(O)=O		-6.45	0.64	2.13	P	IN
QL03660	2-Amino-6,6,7,7,8,8,8-heptafluorooctanoic acid	10325-14-1	NC(CCCC(F)(F)C(F)(F)C(F)(F)F)C(O)=O		-6.38	0.64	2.21	P	IN
QL03661	Undecafluoropentan-1-amine	134380-80-6	NC(F)(F)C(F)(F)C(F)(F)C(F)(F)C(F)(F)F		-6.93	0.69	1.79	P	IN
QL03662	6-Bromo-8-(1,1,1,2,3,3,3-heptafluoropropan-2-yl)quinolin-5-amine	917923-88-7	NC1=C(Br)C=C(C2=C1C=CC=N2)C(F)(C(F)(F)F)C(F)(F)F		-5.65	0.63	1.86	P	IN
QL03663	3-(Tridecafluorohexyl)pyridin-4-amine	143094-26-2	NC1=C(C=NC=C1)C(F)(F)C(F)(F)C(F)(F)C(F)(F)C(F)(F)C(F)(F)F		-7.51	0.66	2.45	P	IN
QL03664	2-Fluoro-4-(nonafluorobutyl)aniline	821791-47-3	NC1=C(F)C=C(C=C1)C(F)(F)C(F)(F)C(F)(F)C(F)(F)F		-7.00	0.66	2.26	P	IN
QL03665	Phenol, 2-amino-4-(heptafluoropropyl)-	61324-30-9	NC1=C(O)C=CC(=C1)C(F)(F)C(F)(F)C(F)(F)F		-6.63	0.65	2.17	P	IN
QL03666	5-(1,1,2,2,3,3,3-heptafluoropropyl)benzene-1,3-diamine	5141-88-8	NC1=CC(=CC(N)=C1)C(F)(F)C(F)(F)C(F)(F)F		-6.01	0.64	1.91	P	IN
QL03667	2,5-Dichloro-4-(1,1,2,3,3,3-hexafluoropropoxy)aniline	103015-84-5	NC1=CC(Cl)=C(OC(F)(F)C(F)C(F)(F)F)C=C1Cl		-6.25	0.63	2.23	P	OUT
QL03668	4-(1,1,1,2,3,3,3-Heptafluoropropan-2-yl)benzene-1,3-diamine	58524-63-3	NC1=CC(N)=C(C=C1)C(F)(C(F)(F)F)C(F)(F)F		-5.97	0.64	1.93	P	IN
QL03669	4-[(Heptadecafluorooctyl)oxy]benzene-1,3-diamine	96573-73-8	NC1=CC(N)=C(OC(F)(F)C(F)(F)C(F)(F)C(F)(F)C(F)(F)C(F)(F)C(F)(F)C(F)(F)F)C=C1		-8.56	0.67	2.89	P	IN
QL03670	4-perfluoroisopropylaniline	2396-17-0	NC1=CC=C(C=C1)C(F)(C(F)(F)F)C(F)(F)F		-5.86	0.64	1.90	P	IN
QL03671	4-(Perfluorooctyl)aniline	83766-52-3	NC1=CC=C(C=C1)C(F)(F)C(F)(F)C(F)(F)C(F)(F)C(F)(F)C(F)(F)C(F)(F)C(F)(F)F		-8.35	0.67	2.74	P	IN
QL03672	4-(Tridecafluorohexyl)aniline	139613-90-4	NC1=CC=C(C=C1)C(F)(F)C(F)(F)C(F)(F)C(F)(F)C(F)(F)C(F)(F)F		-7.72	0.66	2.52	P	IN
QL03673	Benzenamine, 4-(nonafluorobutyl)-	114262-65-6	NC1=CC=C(C=C1)C(F)(F)C(F)(F)C(F)(F)C(F)(F)F		-6.90	0.65	2.26	P	IN
QL03674	4-(3,3,4,4,5,5,6,6,6-Nonafluorohexyl)aniline	919289-98-8	NC1=CC=C(CCC(F)(F)C(F)(F)C(F)(F)C(F)(F)F)C=C1		-7.09	0.64	2.49	P	IN
QL03675	4-(Nonafluorobutoxy)aniline	62315-18-8	NC1=CC=C(OC(F)(F)C(F)(F)C(F)(F)C(F)(F)F)C=C1		-7.08	0.65	2.43	P	IN
QL03676	3-(Heptadecafluorooctyl)aniline	119489-67-7	NC1=CC=CC(=C1)C(F)(F)C(F)(F)C(F)(F)C(F)(F)C(F)(F)C(F)(F)C(F)(F)C(F)(F)F		-8.22	0.67	2.69	P	IN
QL03677	ST50977988	299937-37-4	NC1=NN=C(S1)C(F)(F)C(F)(F)C(F)(F)F		-6.19	0.65	1.92	P	IN
QL03678	2-Bromo-6-(difluoromethoxy)-4-(1,1,1,2,3,3,3-heptafluoropropan-2-yl)aniline	1262665-49-5	Nc1c(Br)cc(cc1OC(F)F)C(F)(C(F)(F)F)C(F)(F)F		-6.12	0.63	2.10	P	IN
QL03679	2-Chloro-6-(difluoromethoxy)-4-(1,1,1,2,3,3,3-heptafluoropropan-2-yl)aniline	1262665-50-8	Nc1c(Cl)cc(cc1OC(F)F)C(F)(C(F)(F)F)C(F)(F)F		-6.12	0.63	2.10	P	IN
QL03680	4-(1,1,1,2,3,3,3-Heptafluoropropan-2-yl)-2-iodo-6-(trifluoromethyl)aniline	1207315-39-6	Nc1c(I)cc(cc1C(F)(F)F)C(F)(C(F)(F)F)C(F)(F)F		-6.05	0.65	1.79	P	IN
QL03681	N-[2-Amino-3-nitro-5-(trifluoromethyl)phenyl]-2,2,3,3,4,4,4-heptafluorobutanamide	57450-70-1	Nc1c(NC(=O)C(F)(F)C(F)(F)C(F)(F)F)cc(cc1[N+]([O-])=O)C(F)(F)F		-6.93	0.65	2.35	P	IN
QL03682	5-{[1,1,1,4,5,5,5-Heptafluoro-3-(1,1,1,2,3,3,3-heptafluoropropan-2-yl)-4-(trifluoromethyl)pent-2-en-2-yl]oxy}benzene-1,3-diamine	138781-42-7	Nc1cc(N)cc(OC(=C(C(F)(C(F)(F)F)C(F)(F)F)C(F)(C(F)(F)F)C(F)(F)F)C(F)(F)F)c1		-6.44	0.65	2.01	P	IN
QL03683	4-Amino-2-(1,1,1,2,3,3,3-heptafluoropropan-2-yl)benzonitrile	1463530-23-5	Nc1ccc(C#N)c(c1)C(F)(C(F)(F)F)C(F)(F)F		-5.14	0.63	1.68	P	OUT
QL03684	4-Amino-2-(undecafluoropentyl)benzonitrile	1463530-19-9	Nc1ccc(C#N)c(c1)C(F)(F)C(F)(F)C(F)(F)C(F)(F)C(F)(F)F		-6.77	0.65	2.24	P	IN
QL03685	4-[5-(Heptafluoropropyl)-1H-1,2,4-triazol-3-yl]aniline	1692915-43-7	Nc1ccc(cc1)-c1nNc(n1)C(F)(F)C(F)(F)C(F)(F)F		-6.99	0.63	2.56	P	OUT
QL03686	Butanamide, N-(4-amino-2-hydroxyphenyl)-2,2,3,3,4,4,4-heptafluoro-	847-51-8	Nc1ccc(NC(=O)C(F)(F)C(F)(F)C(F)(F)F)c(O)c1		-6.85	0.64	2.42	P	IN
QL03687	6-(Heptafluoropropyl)pyridin-3-amine	317810-57-4	Nc1ccc(nc1)C(F)(F)C(F)(F)C(F)(F)F		-6.51	0.64	2.18	P	IN
QL03688	4-(2,2,3,3,4,4,4-Heptafluorobutoxy)aniline	142706-74-9	Nc1ccc(OCC(F)(F)C(F)(F)C(F)(F)F)cc1		-6.72	0.63	2.44	P	OUT
QL03689	3-(1,1,1,2,3,3,3-Heptafluoropropan-2-yl)aniline	2396-16-9	Nc1cccc(c1)C(F)(C(F)(F)F)C(F)(F)F		-5.44	0.64	1.71	P	IN
QL03690	3-(Tridecafluorohexyl)aniline	119534-79-1	Nc1cccc(c1)C(F)(F)C(F)(F)C(F)(F)C(F)(F)C(F)(F)C(F)(F)F		-7.56	0.66	2.46	P	IN
QL03691	Heptadecafluorononanoic acid--aniline (1/1)	95682-67-0	Nc1ccccc1.OC(=O)C(F)(F)C(F)(F)C(F)(F)C(F)(F)C(F)(F)C(F)(F)C(F)(F)C(F)(F)F		-8.16	0.66	2.85	P	IN
QL03692	2-(1,1,1,2,3,3,3-Heptafluoropropan-2-yl)aniline	2396-15-8	Nc1ccccc1C(F)(C(F)(F)F)C(F)(F)F		-5.45	0.64	1.74	P	IN
QL03693	2-(Tridecafluorohexyl)aniline	139613-89-1	Nc1ccccc1C(F)(F)C(F)(F)C(F)(F)C(F)(F)C(F)(F)C(F)(F)F		-7.51	0.66	2.45	P	IN
QL03694	2-(Nonafluorobutyl)aniline	106877-28-5	Nc1ccccc1C(F)(F)C(F)(F)C(F)(F)C(F)(F)F		-6.64	0.65	2.17	P	IN
QL03695	3,5,6-Trifluoro-4-(1,1,1,2,3,3,3-heptafluoropropan-2-yl)pyridin-2-amine	55181-17-4	Nc1nc(F)c(F)c(c1F)C(F)(C(F)(F)F)C(F)(F)F		-5.65	0.65	1.68	P	IN

QL03696	6-[(2,2,3,3,4,4,5,5,6,6,7,7,7-Tridecafluoroheptyl)oxy]-1,3,5-triazine-2,4-diamine	57338-65-5	Nc1nc(N)nc(OCC(F)(F)C(F)(F)C(F)(F)C(F)(F)C(F)(F)C(F)(F)F)n1		-7.84	0.65	2.69	P	IN
QL03697	2-(Heptafluoropropyl)-9H-purin-6-amine	147916-77-6	Nc1nc(nc2Ncnc12)C(F)(F)C(F)(F)C(F)(F)F		-6.45	0.64	2.15	P	IN
QL03698	4-Amino-2-(heptafluoropropyl)pyrimidine-5-carbonitrile	647-78-9	Nc1nc(ncc1C#N)C(F)(F)C(F)(F)C(F)(F)F		-6.52	0.63	2.29	P	IN
QL03699	4,6-Bis[(2,2,3,3,4,4,5,5,6,6,7,7,7-tridecafluoroheptyl)oxy]-1,3,5-triazin-2-amine	57338-64-4	Nc1nc(OCC(F)(F)C(F)(F)C(F)(F)C(F)(F)C(F)(F)C(F)(F)F)nc(OCC(F)(F)C(F)(F)C(F)(F)C(F)(F)C(F)(F)C(F)(F)F)n1		-9.41	0.67	3.30	P	OUT
QL03700	8-(Heptafluoropropyl)adenosine	147916-81-2	Nc1ncnc2n([C@@H]3O[C@H](CO)[C@@H](O)[C@H]3O)c(nc12)C(F)(F)C(F)(F)C(F)(F)F		-6.56	0.63	2.38	P	OUT
QL03701	3-(Heptafluoropropyl)-1H-1,2,4-triazol-5-amine	25979-02-6	Nc1Nnc(n1)C(F)(F)C(F)(F)C(F)(F)F		-6.19	0.65	1.92	P	IN
QL03702	N-[5-Borono-2-(heptafluoropropyl)phenyl]glycinamide	174778-60-0	NCC(=O)Nc1cc(ccc1C(F)(F)C(F)(F)C(F)(F)F)B(O)O		-6.09	0.62	2.23	P	OUT
QL03703	N-[3-Borono-4-(heptafluoropropyl)phenyl]glycinamide	174778-59-7	NCC(=O)Nc1ccc(c(c1)B(O)O)C(F)(F)C(F)(F)C(F)(F)F		-6.73	0.62	2.57	P	OUT
QL03704	2,3,3,3-Tetrafluoro-2-(trifluoromethyl)propan-1-amine	182243-54-5	NCC(F)(C(F)(F)F)C(F)(F)F		-5.05	0.65	1.28	P	IN
QL03705	2,2,3,3,4,4,5,5,6,6,7,7,8,8,9,9,10,10,10-Nonadecafluorodecan-1-amine	307-36-8	NCC(F)(F)C(F)(F)C(F)(F)C(F)(F)C(F)(F)C(F)(F)C(F)(F)C(F)(F)C(F)(F)F		-8.24	0.68	2.51	P	IN
QL03706	1H,1H-Perfluorononylamine	355-47-5	NCC(F)(F)C(F)(F)C(F)(F)C(F)(F)C(F)(F)C(F)(F)C(F)(F)C(F)(F)F		-8.06	0.68	2.44	P	IN
QL03707	1H,1H-Perfluorooctylamine	307-29-9	NCC(F)(F)C(F)(F)C(F)(F)C(F)(F)C(F)(F)C(F)(F)C(F)(F)F		-7.58	0.68	2.23	P	IN
QL03708	1H,1H-Perfluoroheptylamine	423-49-4	NCC(F)(F)C(F)(F)C(F)(F)C(F)(F)C(F)(F)C(F)(F)F		-7.35	0.68	2.15	P	IN
QL03709	1H,1H-Perfluorohexylamine	355-34-0	NCC(F)(F)C(F)(F)C(F)(F)C(F)(F)C(F)(F)F		-6.72	0.67	1.89	P	IN
QL03710	1H,1H-Perfluoropentylamine	355-27-1	NCC(F)(F)C(F)(F)C(F)(F)C(F)(F)F		-6.40	0.67	1.81	P	IN
QL03711	1H,1H-Heptafluorobutylamine	374-99-2	NCC(F)(F)C(F)(F)C(F)(F)F		-5.48	0.66	1.46	P	IN
QL03712	1H,1H-Perfluoropropylamine	422-03-7	NCC(F)(F)C(F)(F)F		-4.95	0.64	1.39	P	IN
QL03713	1-Amino-3,3,4,4,5,5,6,6,6-nonafluorohexan-2-ol	135462-71-4	NCC(O)C(F)(F)C(F)(F)C(F)(F)C(F)(F)F		-6.37	0.66	1.90	P	IN
QL03714	1-Amino-3,3,4,4,5,5,5-heptafluoro-2-pentanol	336-53-8	NCC(O)C(F)(F)C(F)(F)C(F)(F)F		-5.98	0.65	1.84	P	IN
QL03715	1-Amino-4,4,5,5,6,6,7,7,8,9,9,9-dodecafluoro-8-(trifluoromethyl)nonan-2-ol	58935-89-0	NCC(O)CC(F)(F)C(F)(F)C(F)(F)C(F)(F)C(F)(C(F)(F)F)C(F)(F)F		-7.53	0.66	2.43	P	IN
QL03716	1-Amino-4,4,5,5,6,6,7,7,8,8,9,9,10,11,11,11-hexadecafluoro-10-(trifluoromethyl)undecan-2-ol	54735-87-4	NCC(O)CC(F)(F)C(F)(F)C(F)(F)C(F)(F)C(F)(F)C(F)(F)C(F)(C(F)(F)F)C(F)(F)F		-8.20	0.67	2.67	P	IN
QL03717	1-[4-(3,3,4,4,5,5,6,6,7,7,8,8,9,9,10,10,10-Heptadecafluorodecyl)phenyl]methanamine	609816-23-1	NCC1=CC=C(CCC(F)(F)C(F)(F)C(F)(F)C(F)(F)C(F)(F)C(F)(F)C(F)(F)C(F)(F)F)C=C1		-8.65	0.66	3.09	P	IN
QL03718	1H,1H,2H,2H-Perfluorodecylamine	30670-30-5	NCCC(F)(F)C(F)(F)C(F)(F)C(F)(F)C(F)(F)C(F)(F)C(F)(F)C(F)(F)F		-7.91	0.67	2.50	P	IN
QL03719	3,3,4,4,5,5,6,6,7,7,8,8,8-Tridecafluorooctylamine	30556-86-6	NCCC(F)(F)C(F)(F)C(F)(F)C(F)(F)C(F)(F)C(F)(F)F		-7.16	0.66	2.23	P	IN
QL03720	3,3,4,4,5,5,6,6,7,7,7-Undecafluoroheptan-1-amine	1346521-42-3	NCCC(F)(F)C(F)(F)C(F)(F)C(F)(F)C(F)(F)F		-6.87	0.66	2.17	P	IN
QL03721	3,3,4,4,5,5,5-Heptafluoropentan-1-amine	1000509-29-4	NCCC(F)(F)C(F)(F)C(F)(F)F		-5.71	0.64	1.85	P	IN
QL03722	3-(Perfluorooctyl)propylamine	139175-50-1	NCCCC(F)(F)C(F)(F)C(F)(F)C(F)(F)C(F)(F)C(F)(F)C(F)(F)C(F)(F)F		-8.10	0.66	2.68	P	IN
QL03723	N-(3-Aminopropyl)-2,2,3,3,4,4,5,5,6,6,7,7,8,8,8-pentadecafluorooctanamide	85938-56-3	NCCCNC(=O)C(F)(F)C(F)(F)C(F)(F)C(F)(F)C(F)(F)C(F)(F)C(F)(F)F		-7.88	0.65	2.75	P	IN
QL03724	2,2,3,3,4,4,4-Heptafluorobutanimidohydrazide	663-24-1	NNC(=N)C(F)(F)C(F)(F)C(F)(F)F		-5.98	0.65	1.84	P	IN
QL03725	Perfluorotetradecanedioic acid dihydrazide	5102-53-4	NNC(=O)C(F)(F)C(F)(F)C(F)(F)C(F)(F)C(F)(F)C(F)(F)C(F)(F)C(F)(F)C(F)(F)C(F)(F)C(F)(F)C(F)(F)C(=O)NN		-9.07	0.67	3.07	P	OUT
QL03726	2,2,3,3,4,4,5,5,6,6,7,7,7-Tridecafluoroheptanehydrazide	33843-69-5	NNC(=O)C(F)(F)C(F)(F)C(F)(F)C(F)(F)C(F)(F)C(F)(F)F		-7.32	0.67	2.24	P	IN
QL03727	2,2,3,3,4,4,4-heptafluorobutanehydrazide	1515-05-5	NNC(=O)C(F)(F)C(F)(F)C(F)(F)F		-5.98	0.65	1.84	P	IN
QL03728	perfluoropropane sulfonamide, n=3		NS(=O)(=O)C(F)(F)C(F)(F)C(F)(F)F		-6.18	0.68	1.45	P	IN
QL03729	perfluoroethane sulfonamide, n=2		NS(=O)(=O)C(F)(F)C(F)(F)F		-5.80	0.68	1.32	P	IN
QL03730	4-[(Heptadecafluorooctyl)oxy]benzene-1-sulfonamide	89932-76-3	NS(=O)(=O)C1=CC=C(OC(F)(F)C(F)(F)C(F)(F)C(F)(F)C(F)(F)C(F)(F)C(F)(F)C(F)(F)F)C=C1		-8.73	0.67	2.94	P	IN
QL03731	4-{2-[(1,1,2,2,3,3,4,4,4-Nonafluorobutane-1-sulfonyl)amino]ethyl}benzene-1-sulfonamide	316826-59-2	NS(=O)(=O)c1ccc(CCNS(=O)(=O)C(F)(F)C(F)(F)C(F)(F)C(F)(F)F)cc1		-7.99	0.65	2.83	P	IN
QL03732	2,2,3,3,4,4,5,5,6,6,7,7,8,8,9,9,9-Heptadecafluoro-N-[(4-sulfamoylphenyl)methyl]nonanamide	316827-33-5	NS(=O)(=O)c1ccc(CNC(=O)C(F)(F)C(F)(F)C(F)(F)C(F)(F)C(F)(F)C(F)(F)C(F)(F)C(F)(F)F)cc1		-8.93	0.66	3.12	P	OUT
QL03733	2,2,3,3,4,4,5,5,6,6,7,7,7-Tridecafluoro-N-[(4-sulfamoylphenyl)methyl]heptanamide	165618-99-5	NS(=O)(=O)c1ccc(CNC(=O)C(F)(F)C(F)(F)C(F)(F)C(F)(F)C(F)(F)C(F)(F)F)cc1		-8.38	0.66	2.94	P	IN
QL03734	2,2,3,3,4,4,5,5,5-Nonafluoro-N-[(4-sulfamoylphenyl)methyl]pentanamide	165618-98-4	NS(=O)(=O)c1ccc(CNC(=O)C(F)(F)C(F)(F)C(F)(F)C(F)(F)F)cc1		-7.68	0.65	2.71	P	IN
QL03735	2,2,3,3,4,4,4-Heptafluoro-N-[(4-sulfamoylphenyl)methyl]butanamide	165618-97-3	NS(=O)(=O)c1ccc(CNC(=O)C(F)(F)C(F)(F)C(F)(F)F)cc1		-7.39	0.64	2.66	P	IN
QL03736	3,3,4,4,5,5,6,6,7,7,8,8,8-Tridecafluorooctane-1-sulfonamide	29765-95-5	NS(=O)(=O)CCC(F)(F)C(F)(F)C(F)(F)C(F)(F)C(F)(F)C(F)(F)F		-7.90	0.67	2.44	P	IN

QL03737	5-(Perfluoropropyl)-2,2-dimethyl-3,5-pentanedione barium(2+) hydrate (211)	143457-82-3	O.[Ba++].CC(C)(C)C(=O)CC(=O)C(F)(F)C(F)(F)C(F)(F)F.CC(C)(C)C(=O)CC(=O)C(F)(F)C(F)(F)C(F)(F)F		-6.39	0.63	2.33	P	OUT
QL03738	2,2,3,3,4,4,5,5,5-Nonafluoropentanal hydrate (11)	907607-08-3	O.FC(F)(F)C(F)(F)C(F)(F)C(F)(F)C=O		-5.69	0.62	2.03	P	OUT
QL03739	2,2,3,3,4,4,4-Heptafluorobutanal--water (1/1)	907607-07-2	O.FC(F)(F)C(F)(F)C(F)(F)C=O		-4.63	0.61	1.74	P	OUT
QL03740	Potassium pentadecafluorooctanoate--water (1/1/2)	98065-31-7	O.O.[K+].[O-]C(=O)C(F)(F)C(F)(F)C(F)(F)C(F)(F)C(F)(F)C(F)(F)C(F)(F)F		-6.20	0.61	2.49	P	OUT
QL03741	Perfluorooctanoic acid-1,2-13C2	864071-08-9	O[13C](=O)[13C](F)(F)C(F)(F)C(F)(F)C(F)(F)C(F)(F)C(F)(F)C(F)(F)F		-7.72	0.68	2.24	P	IN
QL03742	Pentadecafluoro(1-13C)octanoic acid	864071-09-0	O[13C](=O)C(F)(F)C(F)(F)C(F)(F)C(F)(F)C(F)(F)C(F)(F)C(F)(F)F		-7.72	0.68	2.24	P	IN
QL03743	3,3,4,4,5,5,6,6,7,7,8,8,9,9,10,10,10-Heptadecafluorodecyl beta-D-glucopyranosiduronic acid	864551-34-8	O[C@@H]1[C@@H](O)[C@H](OCCC(F)(F)C(F)(F)C(F)(F)C(F)(F)C(F)(F)C(F)(F)C(F)(F)C(F)(F)F)O[C@@H]([C@H]1O)C(O)=O		-8.69	0.66	3.07	P	IN
QL03744	(3,3,4,4,5,5,6,6,7,7,8,8,8-Tridecafluorooctyl)silanetriol	185911-29-9	O[Si](O)(O)CCC(F)(F)C(F)(F)C(F)(F)C(F)(F)C(F)(F)C(F)(F)F		-7.90	0.67	2.44	P	IN
QL03745	double bond-perfluoroalkyl alcohols, n=0		O/C(F)=C(C(F)(F)F)F		-4.35	0.62	1.35	P	OUT
QL03746	Perfluorodecane sulfonic acid	335-77-3	O=[S]([O-])(=O)C(F)(F)C(F)(F)C(F)(F)C(F)(F)C(F)(F)C(F)(F)C(F)(F)C(F)(F)C(F)(F)C(F)(F)F.[NH4+]		-8.56	0.67	2.79	P	IN
QL03747	Cyclohexanesulfonic acid, nonafluorobis(trifluoromethyl)- , potassium salt (11)	68156-01-4	O=[S]([O-])(=O)C1(C(F)(C(F)(F)C(F)(F)C(F)(F)C1(F)F)C(F)(F)F)C(F)(F)F.[K+]		-6.03	0.67	1.62	P	IN
QL03748	Cyclohexanesulfonic acid, decafluoro(trifluoromethyl)-, potassium salt (TSCA, DSL, AICS)	68156-07-0	O=[S]([O-])(=O)C1(C(F)(F)C(F)(F)C(F)(F)C(F)(F)C1(F)F)C(F)(F)F.[K+]		-6.16	0.66	1.70	P	IN
QL03749	Cyclohexanesulfonic acid, undecafluoro-, potassium salt (TSCA, DSL, AICS)	3107-18-4	O=[S]([O-])(=O)C1(F)C(F)(F)C(F)(F)C(F)(F)C(F)(F)C1(F)F.[K+]		-6.12	0.66	1.67	P	IN
QL03750	1-Octanesulfinic acid, 1,1,2,2,3,3,4,4,5,5,6,6,7,7,8,8,8-heptadecafluoro-, sodium salt	68555-67-9	O=[S]([O-])C(F)(F)C(F)(F)C(F)(F)C(F)(F)C(F)(F)C(F)(F)C(F)(F)C(F)(F)F.[K+]		-7.79	0.66	2.58	P	IN
QL03751	1-Heptanesulfinic acid, 1,1,2,2,3,3,4,4,5,5,6,6,7,7,7-pentadecafluoro-, sodium salt	68555-66-8	O=[S]([O-])C(F)(F)C(F)(F)C(F)(F)C(F)(F)C(F)(F)C(F)(F)C(F)(F)F.[K+]		-7.29	0.66	2.39	P	IN
QL03752	Cyclohexanesulfonyl fluoride, decafluoro(1,1,2,2,2-pentafluoroethyl)-	68156-06-9	O=[S](=O)(C1(C(C(F)(F)C(C(C1(F)F)(F)F)(C(C(F)(F)F)(F)F)F)(F)F)F)F		-8.00	0.69	2.22	P	IN
QL03753	Perfluorododecanesulfonic acid (PFDOS)	79780-39-5	O=[S](=O)(O)C(F)(F)C(F)(F)C(F)(F)C(F)(F)C(F)(F)C(F)(F)C(F)(F)C(F)(F)C(F)(F)C(F)(F)C(F)(F)C(F)(F)F		-9.32	0.69	2.89	P	OUT
QL03757	Perfluorodecanesulphonyl fluoride	307-51-7	O=[S](C(C(C(C(C(C(C(C(C(C(F)(F)F)(F)F)(F)F)(F)F)(F)F)(F)F)(F)F)(F)F)(F)F)(F)F)(=O)F		-8.88	0.69	2.69	P	OUT
QL03758	Perfluorooctanesulfonyl fluoride	307-35-7	O=[S](C(C(C(C(C(C(C(C(F)(F)F)(F)F)(F)F)(F)F)(F)F)(F)F)(F)F)(F)F)(=O)F		-8.37	0.69	2.44	P	IN
QL03759	1,1,2,2,3,3,4,4,5,5,6,6,6-Tridecafluorohexane-1-sulphonyl chloride	55591-23-6	O=[S](C(C(C(C(C(C(F)(F)F)(F)F)(F)F)(F)F)(F)F)(F)F)(=O)Cl		-7.74	0.69	2.15	P	IN
QL03760	Perfluorohexanesulphonyl fluoride	423-50-7	O=[S](C(C(C(C(C(C(F)(F)F)(F)F)(F)F)(F)F)(F)F)(F)F)(=O)F		-7.74	0.69	2.15	P	IN
QL03761	Perfluorobutanesulfonyl fluoride	375-72-4	O=[S](C(C(C(C(F)(F)F)(F)F)(F)F)(F)F)(=O)F		-6.93	0.69	1.79	P	IN
QL03762	Cyclohexanesulfonyl fluoride, nonafluorobis(trifluoromethyl)-	68156-00-3	O=[S](C1(C(C(C(C(C1(F)F)(F)F)(F)F)(F)F)(C(F)(F)F)F)C(F)(F)F)(=O)F		-6.37	0.69	1.45	P	IN
QL03763	Perfluoroheptanesulphonyl fluoride	335-71-7	O=[S](F)(=O)C(F)(F)C(F)(F)C(F)(F)C(F)(F)C(F)(F)C(F)(F)C(F)(F)F		-7.94	0.69	2.24	P	IN
QL03764	Perfluoropentanesulfonyl fluoride	375-81-5	O=[S](F)(=O)C(F)(F)C(F)(F)C(F)(F)C(F)(F)C(F)(F)F		-7.19	0.69	1.90	P	IN
QL03765	Cyclohexanesulfonyl fluoride, undecafluoro-	355-03-3	O=[S](F)(=O)C1(F)C(F)(F)C(F)(F)C(F)(F)C(C1(F)F)(F)F		-6.60	0.70	1.47	P	IN
QL03766	1,1,2,2,3,3,4,4-Octafluorobutane-1-sulphonic acid	70259-86-8	O=[S](O)(=O)C(F)(F)C(F)(F)C(F)(F)C(F)F		-6.61	0.67	1.81	P	IN
QL03767	Octafluorotetrahydrothiophene 1,1-dioxide	42060-64-0	O=[S]1(C(C(C(C1(F)F)(F)F)(F)F)(F)F)=O		-5.62	0.70	0.92	P	OUT
QL03768	ammonium 8-chlorotetradecafluorooctanoate (EINECS)	16557-94-1	O=C([O-])C(F)(F)C(F)(F)C(F)(F)C(F)(F)C(F)(F)C(F)(F)C(Cl)(F)F.[NH4+]		-7.29	0.66	2.39	P	IN
QL03769	Perfluorooctadecanoic acid (PFODA)	16517-11-6	O=C(C(C(C(C(C(C(C(C(C(C(C(C(C(C(C(C(F)(F)F)(F)F)(F)F)(F)F)(F)F)(F)F)(F)F)(F)F)(F)F)(F)F)(F)F)(F)F)(F)F)(F)F)(F)F)(F)F)(F)F)O		-10.02	0.69	3.28	P	OUT
QL03770	Perfluorohexadecanoic acid (PFHxDA)	67905-19-5	O=C(C(C(C(C(C(C(C(C(C(C(C(C(C(C(F)(F)F)(F)F)(F)F)(F)F)(F)F)(F)F)(F)F)(F)F)(F)F)(F)F)(F)F)(F)F)(F)F)(F)F)(F)F)O		-9.68	0.69	3.12	P	OUT
QL03771	Hexacosafluoro-13-(trifluoromethyl)myristic acid	18024-09-4	O=C(C(C(C(C(C(C(C(C(C(C(C(C(C(F)(F)F)(F)C(F)(F)F)(F)F)(F)F)(F)F)(F)F)(F)F)(F)F)(F)F)(F)F)(F)F)(F)F)(F)F)O		-9.26	0.69	2.94	P	OUT
QL03772	Perfluorotetradecanoic acid (PFTeDA)	376-06-7	O=C(C(C(C(C(C(C(C(C(C(C(C(C(F)(F)F)(F)F)(F)F)(F)F)(F)F)(F)F)(F)F)(F)F)(F)F)(F)F)(F)F)(F)F)(F)F)O		-9.31	0.69	2.95	P	OUT
QL03773	Perfluoro-11-(trifluoromethyl)dodecanoic acid	16486-96-7	O=C(C(C(C(C(C(C(C(C(C(C(C(F)(F)F)(F)C(F)(F)F)(F)F)(F)F)(F)F)(F)F)(F)F)(F)F)(F)F)(F)F)(F)F)O		-8.81	0.69	2.73	P	OUT

QL03774	11-H-Perfluoroundecanoic acid	1765-48-6	O=C(C(C(C(C(C(C(C(C(C(C(F)F)(F)F)(F)F)(F)F)(F)F)(F)F)(F)F)(F)F)(F)F)(F)F)O	-8.54	0.68	2.68	P	IN
QL03775	Perfluoro-9-(trifluoromethyl)decanoic acid	16486-94-5	O=C(C(C(C(C(C(C(C(C(C(F)(F)F)(F)C(F)(F)F)(F)F)(F)F)(F)F)(F)F)(F)F)(F)F)(F)F)O	-8.29	0.68	2.50	P	IN
QL03776	2,2,3,3,4,4,5,5,6,6,7,7,8,8,9,9-Hexadecafluorononanoyl chloride	423-95-0	O=C(C(C(C(C(C(C(C(C(F)F)(F)F)(F)F)(F)F)(F)F)(F)F)(F)F)(F)F)Cl	-7.96	0.68	2.44	P	IN
QL03777	9-H-Perfluorononanoic acid	76-21-1	O=C(C(C(C(C(C(C(C(C(F)F)(F)F)(F)F)(F)F)(F)F)(F)F)(F)F)(F)F)O	-7.96	0.68	2.44	P	IN
QL03778	Ammonium 8-chlorohexadecafluoro-7-methyloctanoate	3658-57-9	O=C(C(C(C(C(C(C(C(F)(F)F)(C(Cl)(F)F)F)(F)F)(F)F)(F)F)(F)F)(F)F)O.N	-7.28	0.66	2.36	P	IN
QL03779	Perfluoro-6-methylheptanecarboxylic acid	15899-31-7	O=C(C(C(C(C(C(C(C(F)(F)F)(C(F)(F)F)F)(F)F)(F)F)(F)F)(F)F)(F)F)O	-7.65	0.68	2.22	P	IN
QL03780	Perfluorooctanoyl fluoride	335-66-0	O=C(C(C(C(C(C(C(C(F)(F)F)(F)F)(F)F)(F)F)(F)F)(F)F)(F)F)F	-7.72	0.68	2.24	P	IN
QL03781	6-Oxoperfluorooctanesulfonic acid		O=C(C(C(C(C(C(S(=O)(=O)O)(F)F)(F)F)(F)F)(F)F)(F)F)C(F)(F)F	-8.16	0.68	2.45	P	IN
QL03782	Perfluoro-5-(trifluoromethyl)hexanoic acid	15899-29-3	O=C(C(C(C(C(C(F)(F)F)(F)C(F)(F)F)(F)F)(F)F)(F)F)O	-6.83	0.68	1.87	P	IN
QL03783	Octafluoroadipic acid	336-08-3	O=C(C(C(C(C(O)=O)(F)F)(F)F)(F)F)(F)F)O	-6.57	0.67	1.89	P	IN
QL03784	Perfluorobutanoyl fluoride	335-42-2	O=C(C(C(C(F)(F)F)(F)F)(F)F)F	-5.76	0.67	1.46	P	IN
QL03785	Octafluoropentanoic acid	376-72-7	O=C(C(C(F)(F)C(F)(F)C(F)F)(F)F)O	-6.21	0.66	1.81	P	IN
QL03786	Hexafluoroacetone	684-16-2	O=C(C(F)(F)F)C(F)(F)F	-4.98	0.65	1.30	P	IN
QL03787	Perfluoro-2-methyl-3-oxahexanoic acid	13252-13-6	O=C(C(OC(C(C(F)(F)F)(F)F)(F)F)(C(F)(F)F)F)O	-6.59	0.67	1.85	P	IN
QL03788	6,6,7,7,8,8,8-heptafluoro-2-methylocta-3,5-dione	40002-62-8	O=C(CC(=O)C(F)(F)C(F)(F)C(F)(F)F)C(C)C	-6.60	0.65	2.17	P	IN
QL03789	Hexafluoroglutaryl difluoride	678-78-4	O=C(F)C(F)(F)C(F)(F)C(F)(F)C(=O)F	-6.21	0.66	1.81	P	IN
QL03790	Hexanoyl fluoride, 2,2,3,3,4,4,5,6,6,6-decafluoro-5-(trifluoromethyl)-	18017-31-7	O=C(F)C(F)(F)C(F)(F)C(F)(F)C(F)(C(F)(F)F)C(F)(F)F	-6.83	0.68	1.87	P	IN
QL03791	Octafluoroadipoyl difluoride	37881-62-2	O=C(F)C(F)(F)C(F)(F)C(F)(F)C(F)(F)C(=O)F	-6.57	0.67	1.89	P	IN
QL03792	Octanoyl fluoride, 2,2,3,3,4,4,5,5,6,6,7,8,8,8-tetradecafluoro-7-(trifluoromethyl)-	15742-62-8	O=C(F)C(F)(F)C(F)(F)C(F)(F)C(F)(F)C(F)(F)C(F)(C(F)(F)F)C(F)(F)F	-7.65	0.68	2.22	P	IN
QL03793	Perfluoro-9-methyldecanoyl fluoride	15720-98-6	O=C(F)C(F)(F)C(F)(F)C(F)(F)C(F)(F)C(F)(F)C(F)(F)C(F)(F)C(F)(C(F)(F)F)C(F)(F)F	-8.29	0.68	2.50	P	IN
QL03794	Perfluoro-11-methyldodecanoyl fluoride	15811-52-6	O=C(F)C(F)(F)C(F)(F)C(F)(F)C(F)(F)C(F)(F)C(F)(F)C(F)(F)C(F)(F)C(F)(F)C(F)(C(F)(F)F)C(F)(F)F	-8.81	0.69	2.73	P	OUT
QL03795	Perfluoro-13-(methyl)tetradecanoyl fluoride	68025-62-7	O=C(F)C(F)(F)C(F)(F)C(F)(F)C(F)(F)C(F)(F)C(F)(F)C(F)(F)C(F)(F)C(F)(F)C(F)(F)C(F)(F)C(F)(C(F)(F)F)C(F)(F)F	-9.26	0.69	2.94	P	OUT
QL03796	Perfluoroheptanoyl fluoride	375-84-8	O=C(F)C(F)(F)C(F)(F)C(F)(F)C(F)(F)C(F)(F)C(F)(F)F	-7.50	0.68	2.16	P	IN
QL03797	Perfluorohexanoyl fluoride	355-38-4	O=C(F)C(F)(F)C(F)(F)C(F)(F)C(F)(F)C(F)(F)F	-6.91	0.68	1.90	P	IN
QL03798	4-fluoro-5-oxo-2,4-bis(trifluoromethyl)-1,3-dioxolane-2-carbonyl fluoride	7345-49-5	O=C(F)C1(OC(=O)C(F)(O1)C(F)(F)F)C(F)(F)F	-6.14	0.66	1.75	P	IN
QL03799	4,4,5-trifluoro-2,5-bis(trifluoromethyl)-1,3-dioxolane-2-carbonyl fluoride	7385-65-1	O=C(F)C1(OC(F)(F)C(F)(O1)C(F)(F)F)C(F)(F)F	-6.39	0.67	1.75	P	IN
QL03800	Perfluoroeicosanoic acid	68310-12-3	O=C(O)C(F)(F)C(F)(F)C(F)(F)C(F)(F)C(F)(F)C(F)(F)C(F)(F)C(F)(F)C(F)(F)C(F)(F)C(F)(F)C(F)(F)C(F)(F)C(F)(F)C(F)(F)C(F)(F)C(F)(F)C(F)(F)C(F)(F)F	-10.31	0.69	3.42	P	OUT
QL03801	Perfluorotridecanoic acid (PFTrDA)	72629-94-8	O=C(O)C(F)(F)C(F)(F)C(F)(F)C(F)(F)C(F)(F)C(F)(F)C(F)(F)C(F)(F)C(F)(F)C(F)(F)C(F)(F)C(F)(F)F	-9.18	0.69	2.89	P	OUT
QL03808	2,2,3,3-tetrafluoro-3-(heptafluoropropoxy)propionic acid	378-03-0	O=C(O)C(F)(F)C(F)(F)OC(F)(F)C(F)(F)C(F)(F)F	-7.11	0.67	2.09	P	IN
QL03809	4,8-Dioxa-3H-perfluorononanoic acid	919005-14-4	O=C(O)C(F)(F)C(F)OC(F)(F)C(F)(F)C(F)(F)OC(F)(F)F	-7.44	0.66	2.47	P	IN
QL03810	Difluoro{[2,2,4,5-tetrafluoro-5-(trifluoromethoxy)-1,3-dioxolan-4-yl]oxy}acetic acid	1190931-41-9	O=C(O)C(F)(F)OC1(F)OC(F)(F)OC1(F)OC(F)(F)F	-6.69	0.65	2.17	P	IN
QL03811	4-oxo-4-[(4,4,5,5,6,6,7,7,8,8,9,9,10,10,11,11,11-heptadecafluoro-2-hydroxyundecyl)amino]isocrotonic acid	97259-81-9	O=C(O)C=CC(=O)NCC(O)CC(F)(F)C(F)(F)C(F)(F)C(F)(F)C(F)(F)C(F)(F)C(F)(F)C(F)(F)F	-8.62	0.65	3.13	P	IN
QL03812	n2 fluorotelomer thio proponic acids, n=6		O=C(O)CCSCCC(F)(F)C(F)(F)C(F)(F)C(F)(F)C(F)(F)C(F)(F)C(F)(F)C(F)(F)C(F)(F)C(F)(F)C(F)(F)C(F)(F)F	-9.31	0.67	3.27	P	OUT
QL03813	n2 fluorotelomer thio proponic acids, n=5		O=C(O)CCSCCC(F)(F)C(F)(F)C(F)(F)C(F)(F)C(F)(F)C(F)(F)C(F)(F)C(F)(F)C(F)(F)C(F)(F)F	-8.88	0.66	3.12	P	OUT
QL03814	n2 fluorotelomer thio proponic acids, n=4		O=C(O)CCSCCC(F)(F)C(F)(F)C(F)(F)C(F)(F)C(F)(F)C(F)(F)C(F)(F)C(F)(F)F	-8.36	0.66	2.95	P	IN
QL03815	n2 fluorotelomer thio proponic acids, n=2		O=C(O)CCSCCC(F)(F)C(F)(F)C(F)(F)C(F)(F)F	-6.79	0.63	2.51	P	OUT
QL03816	n2 fluorotelomer thio ethanoic acids, n=5		O=C(O)CSCCC(F)(F)C(F)(F)C(F)(F)C(F)(F)C(F)(F)C(F)(F)C(F)(F)C(F)(F)C(F)(F)C(F)(F)F	-8.91	0.67	3.07	P	OUT
QL03817	n2 fluorotelomer thio ethanoic acids, n=4		O=C(O)CSCCC(F)(F)C(F)(F)C(F)(F)C(F)(F)C(F)(F)C(F)(F)C(F)(F)C(F)(F)F	-8.40	0.66	2.90	P	IN
QL03818	n2 fluorotelomer thio ethanoic acids, n=3		O=C(O)CSCCC(F)(F)C(F)(F)C(F)(F)C(F)(F)C(F)(F)C(F)(F)F	-7.79	0.65	2.72	P	IN
QL03819	n2 fluorotelomer thio ethanoic acids, n=2		O=C(O)CSCCC(F)(F)C(F)(F)C(F)(F)C(F)(F)F	-6.85	0.64	2.44	P	IN

QL03820	Methyl 2,2,3,3,4,4-hexafluoro-4-[1,2,2-trifluoro-2-[(trifluorovinyl)oxy]-1-(trifluoromethyl)ethoxy]butyrate	62361-02-8	O=C(OC)C(F)(F)C(F)(F)C(F)(F)OC(F)(C(F)(F)F)C(F)(F)OC(F)=C(F)F	-7.42	0.65	2.55	P	IN
QL03821	2-[[[dimethyl(3,3,4,4,5,5,6,6,7,7,8,8,8-tridecafluorooctyl)silyl]oxy]methyl]-2-[[(1-oxoallyl)oxy]methyl]-1,3-propanediyl diacrylate	94237-11-3	O=C(OCC(COC(=O)C=C)(COC(=O)C=C)CO[Si](C)(C)CCC(F)(F)C(F)(F)C(F)(F)C(F)(F)C(F)(F)C(F)(F)F)C=C	-7.66	0.62	3.03	P	OUT
QL03822	2-[(acryloyloxy)methyl]-2-[[[bis[3-(acryloyloxy)-2,2-bis[(acryloyloxy)methyl]propoxy](3,3,4,4,5,5,6,6,7,7,8,8,8-tridecafluorooctyl)silyl]oxy]methyl]propane-1,3-diyl diacrylate	94237-13-5	O=C(OCC(COC(=O)C=C)(COC(=O)C=C)CO[Si](OCC(COC(=O)C=C)(COC(=O)C=C)COC(=O)C=C)(OCC(COC(=O)C=C)(COC(=O)C=C)COC(=O)C=C)CCC(F)(F)C(F)(F)C(F)(F)C(F)(F)C(F)(F)C(F)(F)F)C=C	-6.56	0.59	2.91	P	OUT
QL03823	2-[[[[3-(acryloyloxy)-2,2-bis[(acryloyloxy)methyl]propoxy]methyl(3,3,4,4,5,5,6,6,7,7,8,8,8-tridecafluorooctyl)silyl]oxy]methyl]-2-[(acryloyloxy)methyl]propane-1,3-diyl diacrylate	94237-12-4	O=C(OCC(COC(=O)C=C)(COC(=O)C=C)COC[SiH](OCC(COC(=O)C=C)(COC(=O)C=C)COC(=O)C=C)CCC(F)(F)C(F)(F)C(F)(F)C(F)(F)C(F)(F)C(F)(F)F)C=C	-7.00	0.60	3.06	P	OUT
QL03824	2-[[[[5-[[[4-[[(heptadecafluorooctyl)sulphonyl]methylamino]butoxy]carbonyl]amino]-2-methylphenyl]amino]carbonyl]oxy]propyl methacrylate	70900-34-4	O=C(OCCCCN(C)S(=O)(=O)C(F)(F)C(F)(F)C(F)(F)C(F)(F)C(F)(F)C(F)(F)C(F)(F)C(F)(F)F)NC1=CC=C(C(=C1)NC(=O)OC(C)COC(=O)C(=C)C)C	-9.51	0.63	3.84	P	OUT
QL03825	2-[[[[2-methyl-5-[[[4-[methyl[(pentadecafluoroheptyl)sulphonyl]amino]butoxy]carbonyl]amino]phenyl]amino]carbonyl]oxy]propyl methacrylate	70900-35-5	O=C(OCCCCN(C)S(=O)(=O)C(F)(F)C(F)(F)C(F)(F)C(F)(F)C(F)(F)C(F)(F)C(F)(F)F)NC1=CC=C(C(=C1)NC(=O)OC(C)COC(=O)C(=C)C)C	-9.36	0.63	3.81	P	OUT
QL03826	2-[[[[2-methyl-5-[[[4-[methyl[(tridecafluorohexyl)sulphonyl]amino]butoxy]carbonyl]amino]phenyl]amino]carbonyl]oxy]propyl methacrylate	70900-36-6	O=C(OCCCCN(C)S(=O)(=O)C(F)(F)C(F)(F)C(F)(F)C(F)(F)C(F)(F)C(F)(F)F)NC1=CC=C(C(=C1)NC(=O)OC(C)COC(=O)C(=C)C)C	-9.13	0.63	3.75	P	OUT
QL03827	2-[[[[2-methyl-5-[[[4-[methyl[(undecafluoropentyl)sulphonyl]amino]butoxy]carbonyl]amino]phenyl]amino]carbonyl]oxy]propyl methacrylate	70900-37-7	O=C(OCCCCN(C)S(=O)(=O)C(F)(F)C(F)(F)C(F)(F)C(F)(F)C(F)(F)F)NC1=CC=C(C(=C1)NC(=O)OC(C)COC(=O)C(=C)C)C	-8.96	0.62	3.72	P	OUT
QL03828	2-[[[[2-methyl-5-[[[4-[methyl[(nonafluorobutyl)sulphonyl]amino]butoxy]carbonyl]amino]phenyl]amino]carbonyl]oxy]propyl methacrylate	70900-38-8	O=C(OCCCCN(C)S(=O)(=O)C(F)(F)C(F)(F)C(F)(F)C(F)(F)F)NC1=CC=C(C(=C1)NC(=O)OC(C)COC(=O)C(=C)C)C	-8.67	0.62	3.65	P	OUT
QL03829	Perfluoroundecanal	63967-42-0	O=CC(F)(F)C(F)(F)C(F)(F)C(F)(F)C(F)(C(F)(C(F)(F)C(F)(F)C(F)(C(F)(F)F)F)F)F	-8.63	0.68	2.68	P	IN
QL03830	(Perfluorooctane)-1-carbaldehyde	63967-40-8	O=CC(F)(F)C(F)(F)C(F)(F)C(F)(F)C(F)(C(F)(C(F)(F)C(F)(F)F)F)F	-8.06	0.68	2.44	P	IN
QL03831	Perfluorooctanal	335-60-4	O=CC(F)(F)C(F)(F)C(F)(F)C(F)(F)C(F)(C(F)(C(F)(F)F)F)F	-7.58	0.68	2.23	P	IN
QL03832	Perfluoroheptanal	63967-41-9	O=CC(F)(F)C(F)(F)C(F)(F)C(F)(F)C(F)(C(F)(F)F)F	-7.35	0.68	2.15	P	IN
QL03833	122 Fluorotelomer aldehyde	67659-33-0	O=CCC(F)(F)C(F)(F)C(F)(F)C(F)(F)C(F)(F)C(F)(F)C(F)(F)C(F)(F)C(F)(F)C(F)(F)C(F)(F)C(F)(F)F	-8.99	0.68	2.93	P	OUT
QL03834	62 Fluorotelomer aldehyde	56734-81-7	O=CCC(F)(F)C(F)(F)C(F)(F)C(F)(F)C(F)(F)C(F)(F)F	-7.16	0.66	2.23	P	IN
QL03835	3,3,4,4,5,5,6,6,6-nonafluorohexyl dihydrogen phosphate		O=P(O)(O)OCCC(F)(F)C(F)(F)C(F)(F)C(F)(F)F	-7.06	0.66	2.25	P	IN
QL03836	Ethanol, 2,2'-iminobis-, compd. with .alpha.-fluoro-.omega.-[2-(phosphonooxy)ethyl]poly(difluoromethylene) (21)|Ethanol, 2,2'-iminobis-, compd. with .alpha.-fluoro-.omega.-[2-(phosphonooxy)ethyl]poly(difluoromethylene) (11)	65530-63-4|65530-74-7	O=P(O)(O)OCCC(F)(F)F	-4.70	0.61	1.69	P	OUT
QL03837	Fluorotelomer phosphate monoester (Perfluorodecyl phosphate)	576-16-9	O=P(O)(OCCC(F)(F)C(F)(F)C(F)(F)C(F)(F)C(F)(F)C(F)(F)C(F)(F)C(F)(F)F)O	-8.45	0.67	2.73	P	IN
QL03838	62 Fluorotelomer phosphate monoester		O=P(O)(OCCC(F)(F)C(F)(F)C(F)(F)C(F)(F)C(F)(F)C(F)(F)F)O	-7.84	0.67	2.51	P	IN
QL03839	N-ethyl-1,1,2,2,3,3,4,4,5,5,6,6,7,7,7-pentadecafluoro-N-[3-(trichlorosilyl)propyl]heptane-1-sulphonamide	67584-50-3	O=S(=O)(N(CC)CCC[Si](Cl)(Cl)Cl)C(F)(F)C(F)(F)C(F)(F)C(F)(F)C(F)(F)C(F)(F)C(F)(F)F	-8.44	0.66	2.89	P	IN
QL03840	N-ethyl-1,1,2,2,3,3,4,4,5,5,6,6,7,7,7-pentadecafluoro-N-[3-(trimethoxysilyl)propyl]heptane-1-sulphonamide	68239-75-8	O=S(=O)(N(CC)CCC[Si](OC)(OC)OC)C(F)(F)C(F)(F)C(F)(F)C(F)(F)C(F)(F)C(F)(F)C(F)(F)F	-8.07	0.65	2.94	P	IN
QL03841	p-[[4,4,5,5,5-pentafluoro-3-(pentafluoroethyl)-1,2,3-tris(trifluoromethyl)pent-1-enyl]oxy]-N-[3-(triethoxysilyl)propyl]benzenesulphonamide	52026-59-2	O=S(=O)(NCCC[Si](OCC)(OCC)OCC)C1=CC=C(OC(=C(C(F)(F)F)C(C(F)(F)F)(C(F)(F)C(F)(F)F)C(F)(F)C(F)(F)F)C(F)(F)F)C=C1	-7.65	0.62	3.05	P	OUT

QL03842	N-(2-aminoethyl)-2-[1-[difluoro[(trifluorovinyl)oxy]methyl]-1,2,2,2-tetrafluoroethoxy]-1,1,2,2-tetrafluoroethanesulphonamide	64346-90-3	O=S(=O)(NCCN)C(F)(F)C(F)(F)OC(F)(C(F)(F)F)C(F)(F)OC(F)=C(F)F		-7.22	0.64	2.60	P	IN
QL03843	102 Fluorotelomer sulfonic acid		O=S(CCC(F)(C(F)(C(F)(C(F)(C(F)(C(F)(C(F)(C(F)(C(F)(F)C(F)(F)F)F)F)F)F)F)F)F)F)(O)=O		-8.99	0.68	2.88	P	OUT
QL03845	multiple H-substituted perfluoroalkyl sulfates_i n=2		O=S(O)(=O)OC(F)(F)CC(F)(F)C[H]		-5.38	0.63	1.74	P	IN
QL03846	multiple H-substituted perfluoroalkyl sulfates_i n=3		O=S(O)(=O)OC(F)(F)CC(F)(F)CC(F)(F)C[H]		-6.03	0.64	2.03	P	IN
QL03847	multiple H-substituted perfluoroalkyl sulfates n=2		O=S(O)(=O)OCC(F)(F)CC(F)(F)[H]		-4.99	0.61	1.88	P	OUT
QL03848	multiple H-substituted perfluoroalkyl sulfates n=3		O=S(O)(=O)OCC(F)(F)CC(F)(F)CC(F)(F)[H]		-5.91	0.62	2.23	P	OUT
QL03849	p-perfluorous nonenoxybenzenesulfonate	271794-15-1	O=S(O)(C(C=C1)=CC=C1O/C(C(F)(F)F)=C(C(C(F)(F)F)(C(F)(F)F)F)C(C(F)(F)F)(C(F)(F)F)F)=O		-7.08	0.66	2.28	P	IN
QL03850	Perfluoro-2-butyltetrahydrofuran	335-36-4	O1C(C(C(C(F)(F)C(F)(F)F)(F)F)(F)F)(C(C(C1(F)F)(F)F)(F)F)F		-7.79	0.69	2.13	P	IN
QL03851	2,3,5-Trifluorotetrahydro-4-(pentafluoroethyl)-2,3,4,5-tetrakis(trifluoromethyl)furan	69661-30-9	O1C(F)(C(C(F)(C1(F)C(F)(F)F)C(F)(F)F)(C(F)(F)C(F)(F)F)C(F)(F)F)C(F)(F)F		-6.92	0.68	1.81	P	IN
QL03852	Perfluoro-2-methyloxane	356-47-8	O1C(F)(C(F)(F)C(F)(F)C(F)(F)C1(F)F)C(F)(F)F		-6.53	0.69	1.52	P	IN
QL03853	Perfluoro-2-nonylpentahydropyran	68155-54-4	O1C(F)(F)C(F)(F)C(C(C1(F)C(F)(F)C(F)(F)C(F)(F)C(F)(F)C(F)(F)C(F)(F)C(F)(F)C(F)(F)C(F)(F)F)(F)F)(F)F		-9.32	0.70	2.87	P	OUT
QL03854	Dodecafluorooxepane	788-41-0	O1C(F)(F)C(F)(F)C(F)(F)C(C(C1(F)F)(F)F)(F)F		-5.46	0.69	0.95	P	OUT
QL03855	B-(3,5-bis(Perfluoro-1-methylethyl)phenyl)boronic acid	1402929-36-5	OB(O)C1=CC(=CC(=C1)C(F)(C(F)(F)F)C(F)(F)F)C(F)(C(F)(F)F)C(F)(F)F		-6.34	0.66	1.87	P	IN
QL03856	B-(4-(Perfluoro-1-methylethyl)phenyl)boronic acid	1184850-50-7	OB(O)C1=CC=C(C=C1)C(F)(C(F)(F)F)C(F)(F)F		-6.20	0.64	2.10	P	IN
QL03857	multiple double bond-perfluoroalkyl (linear) carboxylic acids, n=0		OC(/C(F)=C(F)/C(F)=C(F)/F)=O		-4.65	0.60	1.86	P	OUT
QL03858	N-Allylperfluoro-2-propoxypropanamide	165663-65-0	OC(=NCC=C)C(F)(OC(F)(F)C(F)(F)C(F)(F)F)C(F)(F)F		-6.99	0.64	2.46	P	IN
QL03859	N-(2,2,3,3,4,4,4-Heptafluorobutanoyl)-D-phenylalanine	868275-40-5	OC(=O)[C@@H](Cc1ccccc1)NC(=O)C(F)(F)C(F)(F)C(F)(F)F		-6.58	0.62	2.52	P	OUT
QL03860	(1r,4r)-4-(Nonafluorobutyl)cyclohexane-1-carboxylic acid	88951-25-1	OC(=O)[C@H]1CC[C@@H](CC1)C(F)(F)C(F)(F)C(F)(F)C(F)(F)F		-7.21	0.65	2.46	P	IN
QL03861	(1r,4r)-4-(Heptafluoropropyl)cyclohexane-1-carboxylic acid	88951-23-9	OC(=O)[C@H]1CC[C@@H](CC1)C(F)(F)C(F)(F)C(F)(F)F		-6.66	0.64	2.29	P	IN
QL03862	(1s,4s)-4-(Nonafluorobutyl)cyclohexane-1-carboxylic acid	88951-24-0	OC(=O)[C@H]1CC[C@H](CC1)C(F)(F)C(F)(F)C(F)(F)C(F)(F)F		-7.21	0.65	2.46	P	IN
QL03863	(1s,4s)-4-(Heptafluoropropyl)cyclohexane-1-carboxylic acid	88951-22-8	OC(=O)[C@H]1CC[C@H](CC1)C(F)(F)C(F)(F)C(F)(F)F		-6.66	0.64	2.29	P	IN
QL03864	Monoperfluorooctyl itaconate	230295-05-3	OC(=O)C(=C)CC(=O)OC(F)(F)C(F)(F)C(F)(F)C(F)(F)C(F)(F)C(F)(F)C(F)(F)C(F)(F)F		-8.54	0.67	2.90	P	IN
QL03865	3,3,4,4,4-Pentafluoro-2,2-bis(pentafluoroethyl)butanoic acid	1882109-58-1	OC(=O)C(C(F)(F)C(F)(F)F)(C(F)(F)C(F)(F)F)C(F)(F)C(F)(F)F		-6.41	0.68	1.66	P	IN
QL03866	3,3,4,4,4-Pentafluoro-2-(1,1,1,2,3,3,3-heptafluoropropan-2-yl)-2-(trifluoromethyl)butanoic acid	1882109-59-2	OC(=O)C(C(F)(F)F)(C(F)(F)C(F)(F)F)C(F)(C(F)(F)F)C(F)(F)F		-6.32	0.67	1.66	P	IN
QL03867	3,3,4,4,5,5,5-Heptafluoro-2-(pentafluoroethyl)-2-(trifluoromethyl)pentanoic acid	1882109-63-8	OC(=O)C(C(F)(F)F)(C(F)(F)C(F)(F)F)C(F)(F)C(F)(F)C(F)(F)F		-6.68	0.68	1.80	P	IN
QL03868	4,4,4-Trifluoro-2,2,3,3-tetrakis(trifluoromethyl)butanoic acid	1882109-62-7	OC(=O)C(C(F)(F)F)(C(F)(F)F)C(C(F)(F)F)(C(F)(F)F)C(F)(F)F		-5.23	0.67	1.16	P	IN
QL03869	3,4,4,5,5,5-Hexafluoro-2,2,3-tris(trifluoromethyl)pentanoic acid	1882109-69-4	OC(=O)C(C(F)(F)F)(C(F)(F)F)C(F)(C(F)(F)F)C(F)(F)C(F)(F)F		-6.32	0.67	1.66	P	IN
QL03870	3,3,4,5,5,5-Hexafluoro-2,2,4-tris(trifluoromethyl)pentanoic acid	1882109-70-7	OC(=O)C(C(F)(F)F)(C(F)(F)F)C(F)(F)C(F)(C(F)(F)F)C(F)(F)F		-6.15	0.67	1.57	P	IN
QL03871	3,3,4,4,5,5,6,6,6-Nonafluoro-2,2-bis(trifluoromethyl)hexanoic acid	1812247-17-8	OC(=O)C(C(F)(F)F)(C(F)(F)F)C(F)(F)C(F)(F)C(F)(F)C(F)(F)F		-6.68	0.68	1.80	P	IN
QL03872	2H-Perfluoroisobutyric acid	564-10-3	OC(=O)C(C(F)(F)F)C(F)(F)F		-4.37	0.64	1.19	P	OUT
QL03873	(3,3,4,4,5,5,6,6,7,7,8,8,9,9,10,10,10-Heptadecafluorodecyl)propanedioic acid	36390-05-3	OC(=O)C(CCC(F)(F)C(F)(F)C(F)(F)C(F)(F)C(F)(F)C(F)(F)C(F)(F)C(F)(F)F)C(O)=O		-8.21	0.67	2.73	P	IN
QL03874	(3,3,4,4,5,5,6,6,7,7,8,8,8-Tridecafluorooctyl)propanedioic acid	26564-96-5	OC(=O)C(CCC(F)(F)C(F)(F)C(F)(F)C(F)(F)C(F)(F)C(F)(F)F)C(O)=O		-7.56	0.66	2.49	P	IN
QL03875	(3,3,4,4,5,5,6,6,6-Nonafluorohexyl)propanedioic acid	36390-03-1	OC(=O)C(CCC(F)(F)C(F)(F)C(F)(F)C(F)(F)F)C(O)=O		-6.71	0.65	2.22	P	IN
QL03876	2-(4,4,5,5,6,6,6-heptafluorohexyl)propanedioic Acid	244022-64-8	OC(=O)C(CCCC(F)(F)C(F)(F)C(F)(F)F)C(O)=O		-6.17	0.63	2.16	P	OUT
QL03877	2,3,4,4,4-Pentafluoro-2-(1,1,1,2,3,3,3-heptafluoropropan-2-yl)-3-(trifluoromethyl)butanoic acid	1882109-61-6	OC(=O)C(F)(C(F)(C(F)(F)F)C(F)(F)F)C(F)(C(F)(F)F)C(F)(F)F		-6.32	0.67	1.64	P	IN

QL03878	2,3,3,4,4,5,5,5-Octafluoro-2-(1,1,1,2,3,3,3-heptafluoropropan-2-yl)pentanoic acid	1882109-76-3	OC(=O)C(F)(C(F)(F)C(F)(F)C(F)(F)F)C(F)(C(F)(F)F)C(F)(F)F		-6.79	0.68	1.84	P	IN
QL03879	2,3,3,4,4,5,5,5-Octafluoro-2-(heptafluoropropyl)pentanoic acid	1882109-77-4	OC(=O)C(F)(C(F)(F)C(F)(F)C(F)(F)F)C(F)(F)C(F)(F)C(F)(F)F		-7.43	0.68	2.12	P	IN
QL03880	2,3,3,4,4,4-Hexafluoro-2-[1,1,1,3,3,3-hexafluoro-2-(trifluoromethyl)propan-2-yl]butanoic acid	1882109-60-5	OC(=O)C(F)(C(F)(F)C(F)(F)F)C(C(F)(F)F)(C(F)(F)F)C(F)(F)F		-6.28	0.67	1.64	P	IN
QL03881	2,3,4,4,5,5,5-Heptafluoro-2-(pentafluoroethyl)-3-(trifluoromethyl)pentanoic acid	1882109-64-9	OC(=O)C(F)(C(F)(F)C(F)(F)F)C(F)(C(F)(F)F)C(F)(F)C(F)(F)F		-6.71	0.68	1.80	P	IN
QL03882	2,3,3,4,5,5,5-Heptafluoro-2-(pentafluoroethyl)-4-(trifluoromethyl)pentanoic acid	1882109-65-0	OC(=O)C(F)(C(F)(F)C(F)(F)F)C(F)(F)C(F)(C(F)(F)F)C(F)(F)F		-6.79	0.68	1.84	P	IN
QL03883	2,3,3,4,4,5,5,6,6,6-Decafluoro-2-(pentafluoroethyl)hexanoic acid	35605-76-6	OC(=O)C(F)(C(F)(F)C(F)(F)F)C(F)(F)C(F)(F)C(F)(F)C(F)(F)F		-7.43	0.68	2.12	P	IN
QL03884	2,4,4,5,5,5-Hexafluoro-2,3,3-tris(trifluoromethyl)pentanoic acid	1882109-71-8	OC(=O)C(F)(C(F)(F)F)C(C(F)(F)F)(C(F)(F)F)C(F)(F)C(F)(F)F		-6.28	0.67	1.64	P	IN
QL03885	2,3,4,4,5,5,5-Heptafluoro-3-(pentafluoroethyl)-2-(trifluoromethyl)pentanoic acid	1882109-66-1	OC(=O)C(F)(C(F)(F)F)C(F)(C(F)(F)C(F)(F)F)C(F)(F)C(F)(F)F		-6.44	0.68	1.66	P	IN
QL03886	2,3,4,5,5,5-Hexafluoro-2,3,4-tris(trifluoromethyl)pentanoic acid	1882109-72-9	OC(=O)C(F)(C(F)(F)F)C(F)(C(F)(F)F)C(F)(C(F)(F)F)C(F)(F)F		-6.35	0.67	1.66	P	IN
QL03887	2,3,4,4,5,5,6,6,6-Nonafluoro-2,3-bis(trifluoromethyl)hexanoic acid	1812247-18-9	OC(=O)C(F)(C(F)(F)F)C(F)(C(F)(F)F)C(F)(F)C(F)(F)C(F)(F)F		-6.79	0.68	1.84	P	IN
QL03888	2,3,3,5,5,5-Hexafluoro-2,4,4-tris(trifluoromethyl)pentanoic acid	1882109-73-0	OC(=O)C(F)(C(F)(F)F)C(F)(F)C(C(F)(F)F)(C(F)(F)F)C(F)(F)F		-6.14	0.67	1.57	P	IN
QL03889	2,3,3,4,5,5,6,6,6-Nonafluoro-2,4-bis(trifluoromethyl)hexanoic acid	1812247-19-0	OC(=O)C(F)(C(F)(F)F)C(F)(F)C(F)(C(F)(F)F)C(F)(F)C(F)(F)F		-6.79	0.68	1.84	P	IN
QL03890	2,3,3,4,4,5,6,6,6-Nonafluoro-2,5-bis(trifluoromethyl)hexanoic acid	1882109-80-9	OC(=O)C(F)(C(F)(F)F)C(F)(F)C(F)(F)C(F)(C(F)(F)F)C(F)(F)F		-6.71	0.68	1.80	P	IN
QL03891	2,3,3,4,4,5,5,6,6,7,7,7-Dodecafluoro-2-(trifluoromethyl)heptanoic acid	207678-51-1	OC(=O)C(F)(C(F)(F)F)C(F)(F)C(F)(F)C(F)(F)C(F)(F)C(F)(F)F		-7.43	0.68	2.12	P	IN
QL03892	Perfluoroisobutyric acid	335-10-4	OC(=O)C(F)(C(F)(F)F)C(F)(F)F		-5.20	0.66	1.24	P	IN
QL03893	Nonanoic acid, 2,4,6,8,9-pentachloro-2,3,3,4,5,5,6,7,7,8,9,9-dodecafluoro-	754-87-0	OC(=O)C(F)(Cl)C(F)(F)C(F)(Cl)C(F)(F)C(F)(Cl)C(F)(F)C(F)(Cl)C(F)(F)Cl		-8.18	0.68	2.45	P	IN
QL03894	2,2,4,4,5,5,5-Heptafluoro-3-(pentafluoroethyl)-3-(trifluoromethyl)pentanoic acid	1882109-67-2	OC(=O)C(F)(F)C(C(F)(F)F)(C(F)(F)C(F)(F)F)C(F)(F)C(F)(F)F		-6.35	0.67	1.64	P	IN
QL03895	2,2,4,5,5,5-Hexafluoro-3,3,4-tris(trifluoromethyl)pentanoic acid	1882109-74-1	OC(=O)C(F)(F)C(C(F)(F)F)(C(F)(F)F)C(F)(C(F)(F)F)C(F)(F)F		-6.26	0.67	1.64	P	IN
QL03896	2,2,4,4,5,5,6,6,6-Nonafluoro-3,3-bis(trifluoromethyl)hexanoic acid	1812247-20-3	OC(=O)C(F)(F)C(C(F)(F)F)(C(F)(F)F)C(F)(F)C(F)(F)C(F)(F)F		-6.57	0.67	1.76	P	IN
QL03897	2,2,3,4,4,5,5,5-Octafluoro-3-(1,1,1,2,3,3,3-heptafluoropropan-2-yl)pentanoic acid	1882109-68-3	OC(=O)C(F)(F)C(F)(C(F)(F)C(F)(F)F)C(F)(C(F)(F)F)C(F)(F)F		-6.42	0.68	1.66	P	IN
QL03898	2,2,3,4,4,5,5,6,6,6-Decafluoro-3-(pentafluoroethyl)hexanoic acid	1882109-78-5	OC(=O)C(F)(F)C(F)(C(F)(F)C(F)(F)F)C(F)(F)C(F)(F)C(F)(F)F		-6.86	0.68	1.84	P	IN
QL03899	2,2,3,5,5,5-Hexafluoro-3,4,4-tris(trifluoromethyl)pentanoic acid	1882109-75-2	OC(=O)C(F)(F)C(F)(C(F)(F)F)C(C(F)(F)F)(C(F)(F)F)C(F)(F)F		-6.29	0.67	1.66	P	IN
QL03900	2,2,3,4,5,5,6,6,6-Nonafluoro-3,4-bis(trifluoromethyl)hexanoic acid	1882109-81-0	OC(=O)C(F)(F)C(F)(C(F)(F)F)C(F)(C(F)(F)F)C(F)(F)C(F)(F)F		-6.61	0.68	1.76	P	IN
QL03901	Perfluoro-3,5,5-trimethylhexanoic acid	238403-51-5	OC(=O)C(F)(F)C(F)(C(F)(F)F)C(F)(F)C(C(F)(F)F)(C(F)(F)F)C(F)(F)F		-6.66	0.67	1.80	P	IN
QL03902	2,2,3,4,4,5,6,6,6-Nonafluoro-3,5-bis(trifluoromethyl)hexanoic acid	1144512-35-5	OC(=O)C(F)(F)C(F)(C(F)(F)F)C(F)(F)C(F)(C(F)(F)F)C(F)(F)F		-6.77	0.68	1.84	P	IN
QL03903	Perfluoro-3,7-dimethyloctanoic acid	172155-07-6	OC(=O)C(F)(F)C(F)(C(F)(F)F)C(F)(F)C(F)(F)C(F)(F)C(F)(C(F)(F)F)C(F)(F)F		-7.59	0.68	2.19	P	IN
QL03904	2,2,3,4,4,5,5,6,6,7,7,7-Dodecafluoro-3-(trifluoromethyl)heptanoic acid	705240-04-6	OC(=O)C(F)(F)C(F)(C(F)(F)F)C(F)(F)C(F)(F)C(F)(F)C(F)(F)F		-7.36	0.68	2.10	P	IN
QL03905	3,5,7,9,11,11-Hexachloro-2,2,3,4,4,5,6,6,7,8,8,9,10,10,11-pentadecafluoroundecanoic acid	53281-37-1	OC(=O)C(F)(F)C(F)(Cl)C(F)(F)C(F)(Cl)C(F)(F)C(F)(Cl)C(F)(F)C(F)(Cl)C(F)(F)C(F)(Cl)Cl		-8.72	0.69	2.69	P	IN
QL03906	3,5,7,9,9-Pentachloro-2,2,3,4,4,5,6,6,7,8,8,9-dodecafluorononanoic acid	53281-25-7	OC(=O)C(F)(F)C(F)(Cl)C(F)(F)C(F)(Cl)C(F)(F)C(F)(Cl)C(F)(F)C(F)(Cl)Cl		-8.18	0.68	2.45	P	IN
QL03907	3,5,7,8-Tetrachloroperfluorooctanoic acid	2923-68-4	OC(=O)C(F)(F)C(F)(Cl)C(F)(F)C(F)(Cl)C(F)(F)C(F)(Cl)C(F)(F)Cl		-7.72	0.68	2.24	P	IN
QL03908	3,5,7,7-Tetrachloro-2,2,3,4,4,5,6,6,7-nonafluoroheptanoic acid	16069-78-6	OC(=O)C(F)(F)C(F)(Cl)C(F)(F)C(F)(Cl)C(F)(F)C(F)(Cl)Cl		-7.50	0.68	2.16	P	IN
QL03909	3,5,6-Trichloroperfluorohexanoic acid	2106-54-9	OC(=O)C(F)(F)C(F)(Cl)C(F)(F)C(F)(Cl)C(F)(F)Cl		-6.91	0.68	1.90	P	IN
QL03910	2,2,3,3,5,5,6,6,6-Nonafluoro-4,4-bis(trifluoromethyl)hexanoic acid	1192593-79-5	OC(=O)C(F)(F)C(F)(F)C(C(F)(F)F)(C(F)(F)F)C(F)(F)C(F)(F)F		-6.57	0.67	1.76	P	IN
QL03911	2,2,3,3,4,5,5,6,6,6-Decafluoro-4-(pentafluoroethyl)hexanoic acid	1882109-79-6	OC(=O)C(F)(F)C(F)(F)C(F)(C(F)(F)C(F)(F)F)C(F)(F)C(F)(F)F		-6.86	0.68	1.84	P	IN

QL03912	2,2,3,3,4,5,6,6,6-Nonafluoro-4,5-bis(trifluoromethyl)hexanoic acid	1144512-36-6	OC(=O)C(F)(F)C(F)(F)C(F)(C(F)(F)F)C(F)(C(F)(F)F)C(F)(F)F		-6.77	0.68	1.84	P	IN
QL03913	2,2,3,3,4,5,5,6,6,7,7,7-Dodecafluoro-4-(trifluoromethyl)heptanoic acid	1144512-18-4	OC(=O)C(F)(F)C(F)(F)C(F)(C(F)(F)F)C(F)(F)C(F)(F)C(F)(F)F		-7.36	0.68	2.10	P	IN
QL03914	2,2,3,3,4,4,6,6,6-Nonafluoro-5,5-bis(trifluoromethyl)hexanoic acid	1144512-34-4	OC(=O)C(F)(F)C(F)(F)C(F)(F)C(C(F)(F)F)(C(F)(F)F)C(F)(F)F		-6.65	0.67	1.80	P	IN
QL03915	2,2,3,3,4,4,5,6,6,7,7,7-Dodecafluoro-5-(trifluoromethyl)heptanoic acid	909009-42-3	OC(=O)C(F)(F)C(F)(F)C(F)(F)C(F)(C(F)(F)F)C(F)(F)C(F)(F)F		-7.36	0.68	2.10	P	IN
QL03916	Perfluoro-6-(trifluoromethyl)heptanoic acid	15166-06-0	OC(=O)C(F)(F)C(F)(F)C(F)(F)C(F)(F)C(F)(C(F)(F)F)C(F)(F)F		-7.41	0.68	2.12	P	IN
QL03917	TP_8230		OC(=O)C(F)(F)C(F)(F)C(F)(F)C(F)(F)C(F)(F)C(F)(F)C(F)(F)C(F)(F)C(F)(F)C(F)(F)C(F)(F)C(F)(F)C(F)(F)C(F)(F)C(F)(F)C(F)(F)C(F)(F)C(F)(F)F		-10.23	0.69	3.38	P	OUT
QL03918	Perfluorododecanoic acid (PFDoDA)	307-55-1	OC(=O)C(F)(F)C(F)(F)C(F)(F)C(F)(F)C(F)(F)C(F)(F)C(F)(F)C(F)(F)C(F)(F)C(F)(F)C(F)(F)F		-8.87	0.69	2.75	P	OUT
QL03919	Perfluoro-1,10-decanedicarboxylic acid	865-85-0	OC(=O)C(F)(F)C(F)(F)C(F)(F)C(F)(F)C(F)(F)C(F)(F)C(F)(F)C(F)(F)C(F)(F)C(F)(F)C(O)=O		-8.70	0.68	2.74	P	IN
QL03921	Perfluorosebacic acid	307-78-8	OC(=O)C(F)(F)C(F)(F)C(F)(F)C(F)(F)C(F)(F)C(F)(F)C(F)(F)C(F)(F)C(O)=O		-8.15	0.68	2.51	P	IN
QL03922	9-Chloro-perfluorononanoic acid	865-79-2	OC(=O)C(F)(F)C(F)(F)C(F)(F)C(F)(F)C(F)(F)C(F)(F)C(F)(F)C(F)(F)Cl		-8.18	0.68	2.45	P	IN
QL03923	Perfluoroazelaic acid	23453-64-7	OC(=O)C(F)(F)C(F)(F)C(F)(F)C(F)(F)C(F)(F)C(F)(F)C(F)(F)C(O)=O		-7.96	0.68	2.44	P	IN
QL03925	8-H-Perfluorooctanoic acid	13973-14-3	OC(=O)C(F)(F)C(F)(F)C(F)(F)C(F)(F)C(F)(F)C(F)(F)C(F)F		-7.47	0.67	2.23	P	IN
QL03926	Perfluorosuberic acid	678-45-5	OC(=O)C(F)(F)C(F)(F)C(F)(F)C(F)(F)C(F)(F)C(F)(F)C(O)=O		-7.47	0.67	2.23	P	IN
QL03927	2,2,3,3,4,4,5,5,6,6,7,7-Dodecafluoro-7-[(1,1,1,2,3,3,3-heptafluoropropan-2-yl)oxy]heptanoic acid	32347-41-4	OC(=O)C(F)(F)C(F)(F)C(F)(F)C(F)(F)C(F)(F)C(F)(F)OC(F)(C(F)(F)F)C(F)(F)F		-8.15	0.68	2.48	P	IN
QL03928	7-H-Perfluoroheptanoic acid	1546-95-8	OC(=O)C(F)(F)C(F)(F)C(F)(F)C(F)(F)C(F)(F)C(F)F		-7.22	0.67	2.15	P	IN
QL03929	6-H-Perfluorohexanoic acid	1726-50-7	OC(=O)C(F)(F)C(F)(F)C(F)(F)C(F)(F)C(F)F		-6.57	0.67	1.89	P	IN
QL03930	Hexafluoroglutaric acid	376-73-8	OC(=O)C(F)(F)C(F)(F)C(F)(F)C(O)=O		-6.21	0.66	1.81	P	IN
QL03931	Perfluoro-4-isopropoxybutanoic acid	801212-59-9	OC(=O)C(F)(F)C(F)(F)C(F)(F)OC(F)(C(F)(F)F)C(F)(F)F		-7.21	0.67	2.11	P	IN
QL03932	4-(1,2-Dichloro-1,2,2-trifluoroethoxy)-2,2,3,3,4,4-hexafluorobutanoic acid	86556-81-2	OC(=O)C(F)(F)C(F)(F)C(F)(F)OC(F)(Cl)C(F)(F)Cl		-7.26	0.67	2.17	P	IN
QL03933	Perfluoro(4-methoxybutanoic) acid	863090-89-5	OC(=O)C(F)(F)C(F)(F)C(F)(F)OC(F)(F)F		-6.39	0.66	1.90	P	IN
QL03934	Tetrafluorosuccinic acid	377-38-8	OC(=O)C(F)(F)C(F)(F)C(O)=O		-5.25	0.65	1.46	P	IN
QL03936	Perfluoro(3,3'-(piperazine-1,4-diyl)dipropanoic acid)	948014-40-2	OC(=O)C(F)(F)C(F)(F)N1C(F)(F)C(F)(F)N(C(F)(F)C(F)(F)C(O)=O)C(F)(F)C1(F)F		-7.90	0.68	2.43	P	IN
QL03937	Perfluoro-3-{[1-(ethenyloxy)propan-2-yl]oxy}propanoic acid	69087-46-3	OC(=O)C(F)(F)C(F)(F)OC(F)(C(F)(F)F)C(F)(F)OC(F)=C(F)F		-7.28	0.65	2.43	P	IN
QL03938	Perfluoro-3-ethoxypropanoic acid	377-76-4	OC(=O)C(F)(F)C(F)(F)OC(F)(F)C(F)(F)F		-6.60	0.67	1.90	P	IN
QL03939	Perfluoro-3-methoxypropanoic acid	377-73-1	OC(=O)C(F)(F)C(F)(F)OC(F)(F)F		-6.01	0.65	1.84	P	IN
QL03940	Flupropanate	756-09-2	OC(=O)C(F)(F)C(F)F		-4.61	0.63	1.39	P	OUT
QL03941	2,2-Difluoro-3-(nonafluorobutoxy)propanoic acid	824393-39-7	OC(=O)C(F)(F)COC(F)(F)C(F)(F)C(F)(F)C(F)(F)F		-7.26	0.67	2.24	P	IN
QL03942	[(1-Chloro-1,1,2,3,3,3-hexafluoropropan-2-yl)oxy](difluoro)acetic acid	903523-21-7	OC(=O)C(F)(F)OC(F)(C(F)(F)F)C(F)(F)Cl		-6.00	0.66	1.65	P	IN
QL03943	Perfluoro-3,6-dioxadecanoic acid	137780-69-9	OC(=O)C(F)(F)OC(F)(F)C(F)(F)OC(F)(F)C(F)(F)C(F)(F)C(F)(F)F		-7.95	0.67	2.51	P	IN
QL03944	Perfluoro-3,6-dioxaoctanoic acid	80153-82-8	OC(=O)C(F)(F)OC(F)(F)C(F)(F)OC(F)(F)C(F)(F)F		-7.07	0.66	2.17	P	IN
QL03945	Perfluoro-3,6,9-trioxatridecanoic acid	330562-41-9	OC(=O)C(F)(F)OC(F)(F)C(F)(F)OC(F)(F)C(F)(F)OC(F)(F)C(F)(F)C(F)(F)C(F)(F)F		-8.74	0.67	2.89	P	IN
QL03946	Perfluoro-3,6,9-trioxaundecane-1,11-dioic acid	55621-18-6	OC(=O)C(F)(F)OC(F)(F)C(F)(F)OC(F)(F)C(F)(F)OC(F)(F)C(O)=O		-7.73	0.66	2.59	P	IN
QL03947	Perfluoro-3,6,9-trioxadecanoic acid	151772-59-7	OC(=O)C(F)(F)OC(F)(F)C(F)(F)OC(F)(F)C(F)(F)OC(F)(F)F		-7.60	0.66	2.52	P	IN
QL03948	Perfluoro-3,6-dioxaoctane-1,8-dioic acid	55621-21-1	OC(=O)C(F)(F)OC(F)(F)C(F)(F)OC(F)(F)C(O)=O		-6.72	0.65	2.17	P	IN
QL03949	Perfluoro-3,6-dioxaheptanoic acid	151772-58-6	OC(=O)C(F)(F)OC(F)(F)C(F)(F)OC(F)(F)F		-6.75	0.65	2.19	P	IN
QL03950	3-Azido-2,3,3-trifluoro-2-(heptafluoropropoxy)propanoic acid	95906-13-1	OC(=O)C(F)(OC(F)(F)C(F)(F)C(F)(F)F)C(F)(F)N=[N+]=[N-]		-6.61	0.65	2.19	P	IN
QL03951	Perfluoro-2,5-dimethyl-3,6-dioxanonanoic acid	13252-14-7	OC(=O)C(F)(OC(F)(F)C(F)(OC(F)(F)C(F)(F)C(F)(F)F)C(F)(F)F)C(F)(F)F		-7.79	0.67	2.40	P	IN
QL03952	Perfluoro-(2,5,8-trimethyl-3,6,9-trioxadodecanoic)acid	65294-16-8	OC(=O)C(F)(OC(F)(F)C(F)(OC(F)(F)C(F)(OC(F)(F)C(F)(F)C(F)(F)F)C(F)(F)F)C(F)(F)F)C(F)(F)F		-8.36	0.68	2.67	P	IN
QL03953	2,4,4,5,7,7,8,10,10,11,13,13,14,14,15,15,15-Heptadecafluoro-2,5,8,11-tetrakis(trifluoromethyl)-3,6,9,12-tetraoxapentadecan-1-oic acid	65150-95-0	OC(=O)C(F)(OC(F)(F)C(F)(OC(F)(F)C(F)(OC(F)(F)C(F)(OC(F)(F)C(F)(F)C(F)(F)F)C(F)(F)F)C(F)(F)F)C(F)(F)F)C(F)(F)F		-9.06	0.68	3.01	P	OUT
QL03954	Perfluoro(2,5,8,11,14-pentamethyl-3,6,9,12,15-pentaoxaoctadecanoic) acid	52481-85-3	OC(=O)C(F)(OC(F)(F)C(F)(OC(F)(F)C(F)(OC(F)(F)C(F)(OC(F)(F)C(F)(OC(F)(F)C(F)(F)C(F)(F)F)C(F)(F)F)C(F)(F)F)C(F)(F)F)C(F)(F)F)C(F)(F)F		-9.44	0.68	3.19	P	OUT

QL03955	3-(Perfluoroisopropyl)-(2E)-difluoropropenoic acid	103229-89-6	OC(=O)C(F)=C(/F)C(F)(C(F)(F)F)C(F)(F)F		-5.77	0.65	1.71	P	IN
QL03956	2,3,4,4,4-Pentafluoro-3-(trifluoromethyl)butanoic acid	917951-62-3	OC(=O)C(F)C(F)(C(F)(F)F)C(F)(F)F		-5.27	0.65	1.40	P	IN
QL03957	(2Z)-4,4,5,5,6,6,6-Heptafluoro-3-phenylhex-2-enoic acid	154375-43-6	OC(=O)C=C(c1ccccc1)C(F)(F)C(F)(F)C(F)(F)F		-5.75	0.62	2.11	P	OUT
QL03959	(3,3,4,4,5,5,6,6,7,7,8,8,9,9,10,10,10-heptadecafluorodecyl) hydrogen maleate	54950-04-8	OC(=O)C=C/C(=O)OCCC(F)(F)C(F)(F)C(F)(F)C(F)(F)C(F)(F)C(F)(F)C(F)(F)C(F)(F)F		-8.61	0.65	3.09	P	IN
QL03960	4-[(Heptadecafluorooctyl)oxy]-4-oxobut-2-enoic acid	152682-85-4	OC(=O)C=CC(=O)OC(F)(F)C(F)(F)C(F)(F)C(F)(F)C(F)(F)C(F)(F)C(F)(F)C(F)(F)F		-8.45	0.66	2.90	P	IN
QL03961	(Perfluorooctyl)ethyl hydrogen fumarate	86130-60-1	OC(=O)C=CC(=O)OCCC(F)(F)C(F)(F)C(F)(F)C(F)(F)C(F)(F)C(F)(F)C(F)(F)C(F)(F)F		-8.61	0.65	3.09	P	IN
QL03962	4,4,5,5,6,6,6-Heptafluorohex-2-enoic acid	356-03-6	OC(=O)C=CC(F)(F)C(F)(F)C(F)(F)F		-6.00	0.64	1.93	P	IN
QL03964	5-[(Heptadecafluorononenyl)oxy]-1,3-benzenedicarboxylic acid	96210-37-6	OC(=O)C1=CC(=CC(OC(F)=C(F)C(F)(F)C(F)(F)C(F)(F)C(F)(F)C(F)(F)C(F)(F)C(F)(F)F)=C1)C(O)=O		-8.27	0.65	2.94	P	IN
QL03965	4-(Heptafluoroisopropyl)benzoic acid	2396-21-6	OC(=O)C1=CC=C(C=C1)C(F)(C(F)(F)F)C(F)(F)F		-6.20	0.64	2.10	P	IN
QL03966	4-(Heptadecafluorooctyl)benzoic acid	74701-33-0	OC(=O)C1=CC=C(C=C1)C(F)(F)C(F)(F)C(F)(F)C(F)(F)C(F)(F)C(F)(F)C(F)(F)C(F)(F)F		-8.58	0.67	2.90	P	IN
QL03967	4-(Nonafluorobutyl)benzoic acid	13835-88-6	OC(=O)C1=CC=C(C=C1)C(F)(F)C(F)(F)C(F)(F)C(F)(F)F		-7.21	0.65	2.46	P	IN
QL03968	4-(Heptafluoropropyl)benzoic acid	88950-94-1	OC(=O)C1=CC=C(C=C1)C(F)(F)C(F)(F)C(F)(F)F		-6.66	0.64	2.29	P	IN
QL03969	4-[(1,2,3,3,4,4,5,5,6,6,7,7,8,8,9,9,9-Heptadecafluoronon-1-en-1-yl)oxy]benzoic acid	58253-65-9	OC(=O)C1=CC=C(OC(F)=C(F)C(F)(F)C(F)(F)C(F)(F)C(F)(F)C(F)(F)C(F)(F)C(F)(F)F)C=C1		-8.60	0.65	3.08	P	IN
QL03970	2-[2,3,3,3-Tetrafluoro-2-(trifluoromethyl)propanoyl]benzoic acid	62656-73-9	OC(=O)C1=CC=CC=C1C(=O)C(F)(C(F)(F)F)C(F)(F)F		-5.48	0.63	1.82	P	OUT
QL03971	2-[(2,2,3,3,4,4,4-heptafluorobutanoyl)amino]benzoic acid	82422-33-1	OC(=O)C1=CC=CC=C1NC(=O)C(F)(F)C(F)(F)C(F)(F)F		-6.24	0.63	2.21	P	OUT
QL03972	2-(1,1,2,2-Tetrafluoroethoxy)benzoic acid	10008-97-6	OC(=O)C1=CC=CC=C1OC(F)(F)C(F)F		-4.76	0.60	1.88	P	OUT
QL03973	2,3,5,6-Tetrafluoro-4-(1,1,1,2,3,3,3-heptafluoropropan-2-yl)benzoic acid	20017-56-5	OC(=O)c1c(F)c(F)c(c(F)c1F)C(F)(C(F)(F)F)C(F)(F)F		-6.58	0.64	2.19	P	IN
QL03974	5-{[1,1,1,4,5,5,5-Heptafluoro-3-(1,1,1,2,3,3,3-heptafluoropropan-2-yl)-4-(trifluoromethyl)pent-2-en-2-yl]oxy}benzene-1,3-dicarboxylic acid	97220-62-7	OC(=O)c1cc(OC(=C(C(F)(C(F)(F)F)C(F)(F)F)C(F)(C(F)(F)F)C(F)(F)F)C(F)(F)F)cc(c1)C(O)=O		-6.62	0.65	2.15	P	IN
QL03975	5-[1,1,2-Trifluoro-2-(heptafluoropropoxy)ethoxy]benzene-1,3-dicarboxylic acid	1268707-97-6	OC(=O)c1cc(OC(F)(F)C(F)OC(F)(F)C(F)(F)C(F)(F)F)cc(c1)C(O)=O		-7.26	0.64	2.64	P	OUT
QL03976	4-(Pentadecafluoroheptyl)cyclohexane-1-carboxylic acid	88951-28-4	OC(=O)C1CCC(CC1)C(F)(F)C(F)(F)C(F)(F)C(F)(F)C(F)(F)C(F)(F)C(F)(F)F		-8.19	0.66	2.75	P	IN
QL03977	4-(Tridecafluorohexyl)cyclohexane-1-carboxylic acid	88951-27-3	OC(=O)C1CCC(CC1)C(F)(F)C(F)(F)C(F)(F)C(F)(F)C(F)(F)C(F)(F)F		-8.00	0.66	2.71	P	IN
QL03978	4-(Undecafluoropentyl)cyclohexane-1-carboxylic acid	88951-26-2	OC(=O)C1CCC(CC1)C(F)(F)C(F)(F)C(F)(F)C(F)(F)C(F)(F)F		-7.52	0.65	2.53	P	IN
QL03979	4-(Heptafluoropropyl)cyclohexane-1-carboxylic acid	88951-29-5	OC(=O)C1CCC(CC1)C(F)(F)C(F)(F)C(F)(F)F		-6.66	0.64	2.29	P	IN
QL03980	6-(Heptafluoropropyl)pyridine-3-carboxylic acid	1105981-44-9	OC(=O)c1ccc(nc1)C(F)(F)C(F)(F)C(F)(F)F		-6.66	0.64	2.29	P	IN
QL03981	4-[(2,2,3,3,4,4,5,5,6,6,7,7,8,8,8-Pentadecafluorooctyl)amino]benzoic acid	73782-58-8	OC(=O)c1ccc(NCC(F)(F)C(F)(F)C(F)(F)C(F)(F)C(F)(F)C(F)(F)C(F)(F)F)cc1		-8.40	0.66	2.96	P	IN
QL03982	4-{[1,1,1,4,5,5,5-Heptafluoro-3-(1,1,1,2,3,3,3-heptafluoropropan-2-yl)-4-(trifluoromethyl)pent-2-en-2-yl]oxy}benzoic acid	65439-50-1	OC(=O)c1ccc(OC(=C(C(F)(C(F)(F)F)C(F)(F)F)C(F)(C(F)(F)F)C(F)(F)F)C(F)(F)F)cc1		-6.95	0.65	2.30	P	IN
QL03983	4-[(Pentadecafluoroheptyl)oxy]benzoic acid	75895-47-5	OC(=O)c1ccc(OC(F)(F)C(F)(F)C(F)(F)C(F)(F)C(F)(F)C(F)(F)C(F)(F)F)cc1		-8.40	0.66	2.91	P	IN
QL03984	4-[(2,2,3,3,4,4,5,5,6,6,7,7,8,8,8-Pentadecafluorooctyl)oxy]benzoic acid	129560-87-8	OC(=O)c1ccc(OCC(F)(F)C(F)(F)C(F)(F)C(F)(F)C(F)(F)C(F)(F)C(F)(F)F)cc1		-8.40	0.66	2.96	P	IN
QL03985	4-(2,2,3,3,4,4,4-Heptafluorobutoxy)benzoic acid	129590-24-5	OC(=O)c1ccc(OCC(F)(F)C(F)(F)C(F)(F)F)cc1		-6.87	0.63	2.53	P	OUT
QL03986	4-[(3,3,4,4,5,5,6,6,7,7,8,8,9,9,10,10,10-Heptadecafluorodecyl)oxy]benzoic acid	188034-84-6	OC(=O)c1ccc(OCCC(F)(F)C(F)(F)C(F)(F)C(F)(F)C(F)(F)C(F)(F)C(F)(F)C(F)(F)F)cc1		-8.72	0.66	3.13	P	IN
QL03987	3-(1,1,1,2,3,3,3-Heptafluoropropan-2-yl)benzoic acid	2396-20-5	OC(=O)c1cccc(c1)C(F)(C(F)(F)F)C(F)(F)F		-5.65	0.64	1.85	P	IN
QL03988	2-(Nonafluorobutyl)benzoic acid	14670-68-9	OC(=O)c1ccccc1C(F)(F)C(F)(F)C(F)(F)C(F)(F)F		-6.49	0.64	2.16	P	IN
QL03989	2-(Heptafluoropropyl)pyridine-4-carboxylic acid	20897-65-8	OC(=O)c1ccnc(c1)C(F)(F)C(F)(F)C(F)(F)F		-6.36	0.64	2.16	P	IN
QL03990	3,4-Bis(2,2,3,3,4,4,4-heptafluorobutyl)-1H-pyrrole-2,5-dicarboxylic acid	83650-67-3	OC(=O)c1Nc(C(O)=O)c(CC(F)(F)C(F)(F)C(F)(F)F)c1CC(F)(F)C(F)(F)C(F)(F)F		-7.36	0.66	2.44	P	IN
QL03991	4,4,5,5,6,6,6-Heptafluoro-3-(nitromethyl)hexanoic acid	7079-86-9	OC(=O)CC(C[N+]([O-])=O)C(F)(F)C(F)(F)C(F)(F)F		-5.57	0.63	1.89	P	OUT
QL03992	2-(2,2,3,3,4,4,4-Heptafluorobutyl)butanedioic acid	121223-84-5	OC(=O)CC(CC(F)(F)C(F)(F)C(F)(F)F)C(O)=O		-6.12	0.63	2.10	P	IN

QL03993	3-(Heptafluoropropyl)pentanedioic acid	336-32-3	OC(=O)CC(CC(O)=O)C(F)(F)C(F)(F)C(F)(F)F		-5.57	0.63	1.89	P	OUT
QL03994	3,4,4,4-Tetrafluoro-3-(trifluoromethyl)butanoic acid	773869-22-0	OC(=O)CC(F)(C(F)(F)F)C(F)(F)F		-5.02	0.65	1.38	P	IN
QL03995	(Perfluoroheptyl)ethanoic acid	45291-33-6	OC(=O)CC(F)(F)C(F)(F)C(F)(F)C(F)(F)C(F)(F)C(F)(F)C(F)(F)F		-7.84	0.67	2.45	P	IN
QL03996	52 Fluorotelomer carboxylic acid	889944-77-8	OC(=O)CC(F)(F)C(F)(F)C(F)(F)C(F)(F)C(F)(F)F		-7.06	0.66	2.17	P	IN
QL03997	3,3,4,4,5,5,5-Heptafluoropentanoic acid	679-46-9	OC(=O)CC(F)(F)C(F)(F)C(F)(F)F		-5.99	0.65	1.84	P	IN
QL03998	3,3,4,4-Tetrafluoro-4-[perfluoro-2-(fluorosulfonyl)ethoxy]butanoic acid	363158-84-3	OC(=O)CC(F)(F)C(F)(F)OC(F)(F)C(F)(F)S(F)(=O)=O		-7.06	0.66	2.24	P	IN
QL03999	5,6,6,6-Tetrafluoro-4-oxo-5-(trifluoromethyl)hexanoic acid	62656-74-0	OC(=O)CCC(=O)C(F)(C(F)(F)F)C(F)(F)F		-5.57	0.64	1.81	P	IN
QL04000	5,5,6,6,7,7,7-heptafluoro-4,4-bis(trifluoromethyl)heptanoic Acid	129991-14-6	OC(=O)CCC(C(F)(F)F)(C(F)(F)F)C(F)(F)C(F)(F)C(F)(F)F		-6.68	0.66	2.05	P	IN
QL04001	4,4-bis(Trifluoromethyl)-4-fluoropropanoic acid	243139-62-0	OC(=O)CCC(F)(C(F)(F)F)C(F)(F)F		-5.36	0.64	1.64	P	IN
QL04002	5-Bromo-4,4,5,5-tetrafluoropentanoic acid	234443-22-2	OC(=O)CCC(F)(F)C(F)(F)Br		-5.31	0.63	1.78	P	OUT
QL04003	83 Fluorotelomer carboxylic acid	34598-33-9	OC(=O)CCC(F)(F)C(F)(F)C(F)(F)C(F)(F)C(F)(F)C(F)(F)C(F)(F)C(F)(F)F		-8.27	0.67	2.69	P	IN
QL04004	3-Perfluoroheptylpropanoic acid	812-70-4	OC(=O)CCC(F)(F)C(F)(F)C(F)(F)C(F)(F)C(F)(F)C(F)(F)C(F)(F)F		-7.84	0.67	2.51	P	IN
QL04005	33 Fluorotelomer carboxylic acid	356-02-5	OC(=O)CCC(F)(F)C(F)(F)C(F)(F)F		-6.00	0.64	1.93	P	IN
QL04006	4,4,5,5-Tetrafluoro-5-[(1,1,1,2,3,3,3-heptafluoropropan-2-yl)oxy]pentanoic acid	28793-36-4	OC(=O)CCC(F)(F)C(F)(F)OC(F)(C(F)(F)F)C(F)(F)F		-6.64	0.65	2.12	P	IN
QL04007	4,5,5,5-Tetrafluoro-4-(heptafluoropropoxy)pentanoic acid	243128-40-7	OC(=O)CCC(F)(OC(F)(F)C(F)(F)C(F)(F)F)C(F)(F)F		-6.87	0.65	2.22	P	IN
QL04008	5,5,6,6,7,7,8,8,8-Nonafluorooctanoic acid	27854-33-7	OC(=O)CCCC(F)(F)C(F)(F)C(F)(F)C(F)(F)F		-6.70	0.64	2.27	P	IN
QL04009	N-(Perfluoroheptanoyl)-3-aminopropanoic acid	160845-44-3	OC(=O)CCNC(=O)C(F)(F)C(F)(F)C(F)(F)C(F)(F)C(F)(F)C(F)(F)F		-7.85	0.65	2.71	P	IN
QL04010	3-(Nonafluorobutoxy)propanoic acid	919005-48-4	OC(=O)CCOC(F)(F)C(F)(F)C(F)(F)C(F)(F)F		-6.70	0.64	2.27	P	IN
QL04011	62 Fluorotelomer thioether propanoic acid	149339-57-1	OC(=O)CCSCCC(F)(F)C(F)(F)C(F)(F)C(F)(F)C(F)(F)C(F)(F)F		-7.74	0.65	2.77	P	IN
QL04012	NSC146413	424-55-5	OC(=O)CSC(CC(O)=O)C(F)(F)C(F)(F)C(F)(F)F		-5.95	0.62	2.15	P	OUT
QL04013	Perfluorotriethylcarbinol	6189-00-0	OC(C(C(F)(F)F)(F)F)(C(C(F)(F)F)(F)F)C(C(F)(F)F)(F)F		-6.75	0.69	1.69	P	IN
QL04014	Cl-substituted perfluoroalkyl (linear) carboyxlic acids_i, n=13		OC(C(F)(F)C(F)(F)C(F)(F)C(F)(F)C(F)(F)C(F)(F)C(F)(F)C(F)(F)C(F)(F)C(F)(F)C(F)(F)C(F)(F)C(F)(F)Cl)=O		-9.31	0.69	2.95	P	OUT
QL04015	Cl-substituted perfluoroalkyl (linear) carboyxlic acids_i, n=12		OC(C(F)(F)C(F)(F)C(F)(F)C(F)(F)C(F)(F)C(F)(F)C(F)(F)C(F)(F)C(F)(F)C(F)(F)C(F)(F)C(F)(F)Cl)=O		-9.18	0.69	2.89	P	OUT
QL04016	Cl-substituted perfluoroalkyl (linear) carboyxlic acids_i, n=11		OC(C(F)(F)C(F)(F)C(F)(F)C(F)(F)C(F)(F)C(F)(F)C(F)(F)C(F)(F)C(F)(F)C(F)(F)C(F)(F)Cl)=O		-8.87	0.69	2.75	P	OUT
QL04017	Cl-substituted perfluoroalkyl (linear) carboyxlic acids_i, n=10		OC(C(F)(F)C(F)(F)C(F)(F)C(F)(F)C(F)(F)C(F)(F)C(F)(F)C(F)(F)C(F)(F)C(F)(F)Cl)=O		-8.72	0.69	2.69	P	IN
QL04018	Cl-substituted perfluoroalkyl (linear) carboyxlic acids_i, n=9		OC(C(F)(F)C(F)(F)C(F)(F)C(F)(F)C(F)(F)C(F)(F)C(F)(F)C(F)(F)C(F)(F)Cl)=O		-8.35	0.69	2.51	P	IN
QL04019	Cl-substituted perfluoroalkyl (linear) carboyxlic acids_i, n=6		OC(C(F)(F)C(F)(F)C(F)(F)C(F)(F)C(F)(F)C(F)(F)Cl)=O		-7.50	0.68	2.16	P	IN
QL04020	Cl-substituted perfluoroalkyl (linear) carboyxlic acids_i, n=5		OC(C(F)(F)C(F)(F)C(F)(F)C(F)(F)C(F)(F)Cl)=O		-6.91	0.68	1.90	P	IN
QL04021	Cl-substituted perfluoroalkyl (linear) carboyxlic acids_i, n=4		OC(C(F)(F)C(F)(F)C(F)(F)C(F)(F)Cl)=O		-6.61	0.67	1.81	P	IN
QL04022	1,1,1,2,4,4,5,5,6,6,7,7-Dodecafluoro-2-(trifluoromethyl)heptan-3-ol	66566-87-8	OC(C(F)(F)C(F)(F)C(F)(F)C(F)F)C(F)(C(F)(F)F)C(F)(F)F		-7.23	0.67	2.12	P	IN
QL04023	Cl-substituted perfluoroalkyl (linear) carboyxlic acids_i, n=3		OC(C(F)(F)C(F)(F)C(F)(F)Cl)=O		-5.76	0.67	1.46	P	IN
QL04024	1,1,1,3,4,4,4-Heptafluoro-2,3-bis(trifluoromethyl)butan-2-ol	16500-37-1	OC(C(F)(F)F)(C(F)(F)F)C(F)(C(F)(F)F)C(F)(F)F		-5.80	0.68	1.30	P	IN
QL04025	1,1,1,3,3,4,4,4-Octafluoro-2-butanol	127256-73-9	OC(C(F)(F)F)C(F)(F)C(F)(F)F		-5.73	0.67	1.46	P	IN
QL04026	Perfluoro-2-(2-ethoxyethoxy)-1,1-diphenylethan-1-ol	217824-95-8	OC(C1=C(F)C(F)=C(F)C(F)=C1F)(C1=C(F)C(F)=C(F)C(F)=C1F)C(F)(F)OC(F)(F)C(F)(F)OC(F)(F)C(F)(F)F		-7.72	0.65	2.68	P	IN
QL04027	(Perfluoropropyl)(perfluorophenyl)methanol	19108-36-2	OC(C1=C(F)C(F)=C(F)C(F)=C1F)C(F)(F)C(F)(F)C(F)(F)F		-6.81	0.65	2.21	P	IN
QL04028	5-(Perfluorooctyl)-1,3-bis(bis(trifluoromethyl)hydroxymethyl)benzene	89780-02-9	OC(C1=CC(=CC(=C1)C(F)(F)C(F)(F)C(F)(F)C(F)(F)C(F)(F)C(F)(F)C(F)(F)C(F)(F)F)C(O)(C(F)(F)F)C(F)(F)F)(C(F)(F)F)C(F)(F)F		-8.68	0.68	2.74	P	IN
QL04029	(Perfluorobutyl)-4-bromophenylmethanol	1228786-78-4	OC(C1=CC=C(Br)C=C1)C(F)(F)C(F)(F)C(F)(F)C(F)(F)F		-7.21	0.65	2.46	P	IN
QL04030	[4-(3,3,4,4,5,5,6,6,7,7,8,8,9,9,10,10,10-Heptadecafluorodecyl)phenyl](diphenyl)methanol	649561-66-0	OC(C1=CC=CC=C1)(C1=CC=CC=C1)C1=CC=C(CCC(F)(F)C(F)(F)C(F)(F)C(F)(F)C(F)(F)C(F)(F)C(F)(F)C(F)(F)F)C=C1		-8.46	0.64	3.22	P	OUT
QL04031	(Perfluorooctyl)phenylmethanol	78960-85-7	OC(C1=CC=CC=C1)C(F)(F)C(F)(F)C(F)(F)C(F)(F)C(F)(F)C(F)(F)C(F)(F)C(F)(F)F		-8.19	0.66	2.75	P	IN
QL04032	(Perfluoropropyl)phenylmethanol	785-93-3	OC(C1=CC=CC=C1)C(F)(F)C(F)(F)C(F)(F)F		-6.23	0.63	2.17	P	IN
QL04033	1H-Pyrrol-2-yl(perfluoropropyl)methanol	159355-62-1	OC(C1=CC=CN1)C(F)(F)C(F)(F)C(F)(F)F		-5.95	0.64	1.92	P	IN
QL04034	3-(Bromomethyl)-1,1,1,2,2,4,4,5,5,6,6,6-dodecafluorohexan-3-ol	93417-72-2	OC(CBr)(C(F)(F)C(F)(F)F)C(F)(F)C(F)(F)C(F)(F)F		-6.78	0.67	1.88	P	IN

QL04035	122 Fluorotelomer carboxylic acid	70887-93-3	OC(CC(F)(F)C(F)(F)C(F)(F)C(F)(F)C(F)(F)C(F)(F)C(F)(F)C(F)(F)C(F)(F)C(F)(F)C(F)(C(F)(F)F)F)=O		-9.08	0.68	2.94	P	OUT
QL04036	2-Perfluorodecyl ethanoic acid	53826-13-4	OC(CC(F)(F)C(F)(F)C(F)(F)C(F)(F)C(F)(F)C(F)(F)C(F)(F)C(F)(F)C(F)(F)C(F)(F)F)=O		-8.61	0.68	2.74	P	IN
QL04037	(Perfluoropropyl)chloromethyl-2,4-difluorophenylmethanol	92737-03-6	OC(CCl)(C1=C(F)C=C(F)C=C1)C(F)(F)C(F)(F)C(F)(F)F		-6.53	0.64	2.22	P	IN
QL04038	3,3,4,4,5,5,6,6,7,7,8,8,8-Tridecafluoro-1-iodooctan-2-ol	89807-99-8	OC(CI)C(F)(F)C(F)(F)C(F)(F)C(F)(F)C(F)(F)C(F)(F)F		-7.32	0.67	2.24	P	IN
QL04039	alpha-(2,2,3,3,4,4,5,5,6,6,7,7,8,8,9,9,9-Heptadecafluorononyl)aziridine-1-ethanol	94159-85-0	OC(CN1CC1)CC(F)(F)C(F)(F)C(F)(F)C(F)(F)C(F)(F)C(F)(F)C(F)(F)C(F)(F)F		-8.35	0.67	2.74	P	IN
QL04040	Perfluoroheptane sulfonamidoacetic acid	1003194-00-0	OC(CNS(C(F)(F)C(F)(F)C(F)(F)C(F)(F)C(F)(F)C(C(F)(F)F)(F)F)(=O)=O)=O		-8.27	0.67	2.69	P	IN
QL04041	Perfluorohexane sulfonamidoacetic acid	1003193-99-4	OC(CNS(C(F)(F)C(F)(F)C(F)(F)C(F)(F)C(F)(F)C(F)(F)F)(=O)=O)=O		-7.84	0.67	2.51	P	IN
QL04042	Perfluoropentane sulfonamidoacetic acid	647-43-8	OC(CNS(C(F)(F)C(F)(F)C(F)(F)C(F)(F)C(F)(F)F)(=O)=O)=O		-7.62	0.66	2.45	P	IN
QL04043	(Perfluoro-3-methylbutyl)-2-hydroxypropyl acrylate	16083-76-4	OC(COC(=O)C=C)CC(F)(F)C(F)(F)C(F)(C(F)(F)F)C(F)(F)F		-6.97	0.64	2.48	P	IN
QL04044	3-(Perfluorooctyl)-2-hydroxypropyl acrylate	76962-34-0	OC(COC(=O)C=C)CC(F)(F)C(F)(F)C(F)(F)C(F)(F)C(F)(F)C(F)(F)C(F)(F)C(F)(F)F		-8.39	0.66	2.95	P	IN
QL04045	3-Perfluorohexyl-2-hydroxypropyl acrylate	127377-12-2	OC(COC(=O)C=C)CC(F)(F)C(F)(F)C(F)(F)C(F)(F)C(F)(F)C(F)(F)F		-7.78	0.65	2.76	P	IN
QL04046	4,4,5,5,6,6,7,7,7-Nonafluoro-2-hydroxyheptyl prop-2-enoate	98573-25-2	OC(COC(=O)C=C)CC(F)(F)C(F)(F)C(F)(F)C(F)(F)F		-6.93	0.63	2.53	P	OUT
QL04047	4,4,5,5,6,6,7,7,8,8,9,9,10,10,11,11,11-Heptadecafluoro-1-(vinyloxy)undecan-2-ol	94159-86-1	OC(COC=C)CC(F)(F)C(F)(F)C(F)(F)C(F)(F)C(F)(F)C(F)(F)C(F)(F)C(F)(F)F		-8.29	0.66	2.90	P	IN
QL04048	4,4,5,5,6,6,7,7,8,8,9,9,10,10,11,11,11-Heptadecafluoro-1-phenoxyundecan-2-ol	94159-91-8	OC(COC1=CC=CC=C1)CC(F)(F)C(F)(F)C(F)(F)C(F)(F)C(F)(F)C(F)(F)C(F)(F)C(F)(F)F		-8.54	0.65	3.09	P	IN
QL04049	1,3-Bis(2,2,3,3,4,4,4-heptafluorobutoxy)propan-2-ol	1598-17-0	OC(COCC(F)(F)C(F)(F)C(F)(F)F)COCC(F)(F)C(F)(F)C(F)(F)F		-7.80	0.65	2.76	P	IN
QL04050	4,5-Dihydro-2-phenyl-α-((perfluorohexyl)ethyloxy)methyl-1H-Imidazole-1-ethanol	1363391-48-3	OC(COCCC(F)(F)C(F)(F)C(F)(F)C(F)(F)C(F)(F)C(F)(F)F)CN1CCN=C1C1=CC=CC=C1		-8.07	0.63	3.18	P	OUT
QL04051	1,3-Bis[(3,3,4,4,5,5,6,6,6-nonafluorohexyl)oxy]propan-2-ol	384828-83-5	OC(COCCC(F)(F)C(F)(F)C(F)(F)C(F)(F)F)COCCC(F)(F)C(F)(F)C(F)(F)C(F)(F)F		-8.46	0.65	3.09	P	OUT
QL04052	Bis[(perfluoro-7-methyloctyl)-2-hydroxypropyl] hydrogen phosphate	63295-20-5	OC(COP(O)(=O)OCC(O)CC(F)(F)C(F)(F)C(F)(F)C(F)(F)C(F)(F)C(F)(F)C(F)(C(F)(F)F)C(F)(F)F)CC(F)(F)C(F)(F)C(F)(F)C(F)(F)C(F)(F)C(F)(F)C(F)(C(F)(F)F)C(F)(F)F		-10.14	0.67	3.59	P	OUT
QL04053	Bis(3-(perfluorooctyl)-2-hydroxypropyl) hydrogen phosphate	170004-70-3	OC(COP(O)(=O)OCC(O)CC(F)(F)C(F)(F)C(F)(F)C(F)(F)C(F)(F)C(F)(F)C(F)(F)C(F)(F)F)CC(F)(F)C(F)(F)C(F)(F)C(F)(F)C(F)(F)C(F)(F)C(F)(F)C(F)(F)F		-10.27	0.67	3.66	P	OUT
QL04054	3-(Perfluoro-5-methylhexyl)-1,2-propanediol 1-(dihydrogen phosphate)	105416-14-6	OC(COP(O)(O)=O)CC(F)(F)C(F)(F)C(F)(F)C(F)(F)C(F)(C(F)(F)F)C(F)(F)F		-7.98	0.66	2.63	P	IN
QL04055	3-(Perfluorononyl)-1,2-propanediol-1-phosphate	54009-74-4	OC(COP(O)(O)=O)CC(F)(F)C(F)(F)C(F)(F)C(F)(F)C(F)(F)C(F)(F)C(F)(F)C(F)(F)C(F)(F)F		-8.80	0.67	2.94	P	OUT
QL04056	(Perfluorooctyl)-2-hydroxypropyl dihydrogen phosphate	94158-69-7	OC(COP(O)(O)=O)CC(F)(F)C(F)(F)C(F)(F)C(F)(F)C(F)(F)C(F)(F)C(F)(F)C(F)(F)F		-8.65	0.67	2.89	P	IN
QL04057	double bond-perfluoroalkyl alcohols, n=1		OC(F)(F)/C(F)=C(C(F)(F)F)F		-5.18	0.65	1.46	P	IN
QL04058	double bond-perfluoroalkyl alcohols, n=2		OC(F)(F)C(F)(F)/C(F)=C(C(F)(F)F)F		-6.21	0.66	1.78	P	IN
QL04059	double bond-perfluoroalkyl alcohols, n=3		OC(F)(F)C(F)(F)C(F)(F)/C(F)=C(C(F)(F)F)F		-6.63	0.67	1.91	P	IN
QL04060	double bond-perfluoroalkyl alcohols, n=4		OC(F)(F)C(F)(F)C(F)(F)C(F)(F)/C(F)=C(C(F)(F)F)F		-7.27	0.67	2.17	P	IN
QL04061	double bond-perfluoroalkyl alcohols, n=5		OC(F)(F)C(F)(F)C(F)(F)C(F)(F)C(F)(F)/C(F)=C(C(F)(F)F)F		-7.52	0.68	2.24	P	IN
QL04062	double bond-perfluoroalkyl alcohols, n=6		OC(F)(F)C(F)(F)C(F)(F)C(F)(F)C(F)(F)C(F)(F)/C(F)=C(C(F)(F)F)F		-8.00	0.68	2.45	P	IN
QL04063	double bond-perfluoroalkyl alcohols, n=7		OC(F)(F)C(F)(F)C(F)(F)C(F)(F)C(F)(F)C(F)(F)C(F)(F)/C(F)=C(C(F)(F)F)F		-8.19	0.68	2.52	P	IN
QL04064	double bond-perfluoroalkyl alcohols, n=8		OC(F)(F)C(F)(F)C(F)(F)C(F)(F)C(F)(F)C(F)(F)C(F)(F)C(F)(F)/C(F)=C(C(F)(F)F)F		-8.58	0.68	2.69	P	IN
QL04065	double bond-perfluoroalkyl alcohols, n=9		OC(F)(F)C(F)(F)C(F)(F)C(F)(F)C(F)(F)C(F)(F)C(F)(F)C(F)(F)C(F)(F)/C(F)=C(C(F)(F)F)F		-8.73	0.68	2.75	P	IN
QL04066	double bond-perfluoroalkyl alcohols, n=10		OC(F)(F)C(F)(F)C(F)(F)C(F)(F)C(F)(F)C(F)(F)C(F)(F)C(F)(F)C(F)(F)C(F)(F)/C(F)=C(C(F)(F)F)F		-9.06	0.68	2.89	P	OUT
QL04067	double bond-perfluoroalkyl alcohols, n=11		OC(F)(F)C(F)(F)C(F)(F)C(F)(F)C(F)(F)C(F)(F)C(F)(F)C(F)(F)C(F)(F)C(F)(F)C(F)(F)/C(F)=C(C(F)(F)F)F		-9.19	0.69	2.95	P	OUT
QL04068	double bond-perfluoroalkyl alcohols, n=12		OC(F)(F)C(F)(F)C(F)(F)C(F)(F)C(F)(F)C(F)(F)C(F)(F)C(F)(F)C(F)(F)C(F)(F)C(F)(F)C(F)(F)/C(F)=C(C(F)(F)F)F		-9.47	0.69	3.07	P	OUT
QL04069	double bond-perfluoroalkyl alcohols, n=13		OC(F)(F)C(F)(F)C(F)(F)C(F)(F)C(F)(F)C(F)(F)C(F)(F)C(F)(F)C(F)(F)C(F)(F)C(F)(F)C(F)(F)C(F)(F)/C(F)=C(C(F)(F)F)F		-9.58	0.69	3.12	P	OUT

QL04070	1,1,2,2,3,3,4,4,5,5,6,6,7,7-Tetradecafluoroheptan-1-ol	7098-02-4	OC(F)(F)C(F)(F)C(F)(F)C(F)(F)C(F)(F)C(F)(F)C(F)F		-7.50	0.68	2.16	P	IN
QL04071	1,1,2,2,3,3,4,4,4-Nonafluoro-1-butanol	89807-87-4	OC(F)(F)C(F)(F)C(F)(F)C(F)(F)F		-6.18	0.68	1.45	P	IN
QL04072	perfluoroalkyl alcohols, n=3		OC(F)(F)C(F)(F)C(F)(F)F		-5.80	0.68	1.32	P	IN
QL04073	N,N'-bis(Perfluorooctanoyl)-1,2-cyclohexanediamine	305848-92-4	OC(NC1CCCCC1NC(O)C(F)(F)C(F)(F)C(F)(F)C(F)(F)C(F)(F)C(F)(F)C(F)(F)F)C(F)(F)C(F)(F)C(F)(F)C(F)(F)C(F)(F)C(F)(F)C(F)(F)F		-9.70	0.67	3.40	P	OUT
QL04074	1,1-Octanediol, 2,2,3,3,4,4,5,5,6,6,7,7,8,8,8-pentadecafluoro-	31185-69-0	OC(O)C(F)(F)C(F)(F)C(F)(F)C(F)(F)C(F)(F)C(F)(F)C(F)(F)F		-7.72	0.68	2.24	P	IN
QL04075	2,2,3,3,4,4,5,5,6,6,6-Undecafluoro-1,1-hexanediol	904037-24-7	OC(O)C(F)(F)C(F)(F)C(F)(F)C(F)(F)C(F)(F)F		-6.91	0.68	1.90	P	IN
QL04076	2,2,3,3,4,4,4-Heptafluorobutane-1,1-diol	375-21-3	OC(O)C(F)(F)C(F)(F)C(F)(F)F		-5.76	0.67	1.46	P	IN
QL04077	1H-Perfluoro-1,1-propanediol	422-63-9	OC(O)C(F)(F)C(F)(F)F		-5.29	0.66	1.36	P	IN
QL04078	5-(Tridecafluorohexyl)uridine	58671-32-2	OC[C@H]1O[C@H]([C@H](O)[C@@H]1O)N1C=C(C(=O)NC1=O)C(F)(F)C(F)(F)C(F)(F)C(F)(F)C(F)(F)C(F)(F)F		-8.10	0.65	2.88	P	IN
QL04079	5-(Heptafluoropropyl)uridine	144862-38-4	OC[C@H]1O[C@H]([C@H](O)[C@@H]1O)N1C=C(C(=O)NC1=O)C(F)(F)C(F)(F)C(F)(F)F		-6.91	0.63	2.50	P	OUT
QL04080	5-[1,2,2,2-Tetrafluoro-1-(heptafluoropropoxy)ethyl]uridine	144862-40-8	OC[C@H]1O[C@H]([C@H](O)[C@@H]1O)N1C=C(C(=O)NC1=O)C(F)(OC(F)(F)C(F)(F)C(F)(F)F)C(F)(F)F		-7.58	0.64	2.73	P	IN
QL04081	2'-Deoxy-5-(tridecafluorohexyl)uridine	144862-39-5	OC[C@H]1O[C@H](C[C@@H]1O)N1C=C(C(=O)NC1=O)C(F)(F)C(F)(F)C(F)(F)C(F)(F)C(F)(F)C(F)(F)F		-8.07	0.65	2.88	P	IN
QL04082	2'-Deoxy-5-(nonafluorobutyl)uridine	58671-35-5	OC[C@H]1O[C@H](C[C@@H]1O)N1C=C(C(=O)NC1=O)C(F)(F)C(F)(F)C(F)(F)C(F)(F)F		-7.36	0.64	2.66	P	OUT
QL04083	2'-Deoxy-5-(heptafluoropropyl)uridine	1033630-69-1	OC[C@H]1O[C@H](C[C@@H]1O)N1C=C(C(=O)NC1=O)C(F)(F)C(F)(F)C(F)(F)F		-6.88	0.63	2.52	P	OUT
QL04084	4,5,6,7-Tetrafluoro-3-(1,1,1,2,3,3,3-heptafluoropropan-2-yl)-3-hydroxy-2-benzofuran-1(3H)-one	1419865-32-9	OC1(OC(=O)c2c1c(F)c(F)c(F)c2F)C(F)(C(F)(F)F)C(F)(F)F		-6.76	0.65	2.15	P	IN
QL04085	2-(Tridecafluorohexyl)-5,6-dihydro-2H-pyran-2-ol	1401315-55-6	OC1(OCCC=C1)C(F)(F)C(F)(F)C(F)(F)C(F)(F)C(F)(F)C(F)(F)F		-7.67	0.67	2.45	P	IN
QL04086	Phenol, 4-(heptafluoropropyl)-2-nitro-	61324-29-6	OC1=C(C=C(C=C1)C(F)(F)C(F)(F)C(F)(F)F)[N+]([O-])=O		-6.44	0.64	2.15	P	IN
QL04087	1-(2,4-Dihydroxyphenyl)-2,2,3,3,4,4,4-heptafluorobutan-1-one	65240-03-1	OC1=CC(O)=C(C=C1)C(=O)C(F)(F)C(F)(F)C(F)(F)F		-6.63	0.64	2.25	P	IN
QL04088	4-(Nonafluorobutyl)phenol	123068-23-5	OC1=CC=C(C=C1)C(F)(F)C(F)(F)C(F)(F)C(F)(F)F		-6.90	0.65	2.26	P	IN
QL04089	Phenol, 4-(heptafluoropropyl)-	61324-28-5	OC1=CC=C(C=C1)C(F)(F)C(F)(F)C(F)(F)F		-6.51	0.64	2.18	P	IN
QL04090	4'-Hydroxy[1,1'-biphenyl]-4-yl 2-[perfluoro-4-morpholinylmethyl]-perfluoropropanoate	304658-31-9	OC1=CC=C(C=C1)C1=CC=C(OC(=O)C(F)(C(F)(F)F)C(F)(F)N2C(F)(F)C(F)(F)OC(F)(F)C2(F)F)C=C1		-8.17	0.65	2.99	P	OUT
QL04091	4-(1H,1H,2H,2H-Perfluorodecylthio)phenol	142623-70-9	OC1=CC=C(SCCC(F)(F)C(F)(F)C(F)(F)C(F)(F)C(F)(F)C(F)(F)C(F)(F)C(F)(F)F)C=C1		-8.70	0.66	3.09	P	IN
QL04092	4-[(3,3,4,4,5,5,6,6,7,7,8,8,8-Tridecafluorooctyl)sulfanyl]phenol	892155-19-0	OC1=CC=C(SCCC(F)(F)C(F)(F)C(F)(F)C(F)(F)C(F)(F)C(F)(F)F)C=C1		-8.05	0.65	2.88	P	IN
QL04093	4-[(3,3,4,4,5,5,6,6,6-Nonafluorohexyl)sulfanyl]phenol	892155-14-5	OC1=CC=C(SCCC(F)(F)C(F)(F)C(F)(F)C(F)(F)F)C=C1		-7.21	0.64	2.64	P	OUT
QL04095	Butanamide, 2,2,3,3,4,4,4-heptafluoro-N-(2-hydroxy-4-nitrophenyl)-	2712-83-6	Oc1cc(ccc1NC(=O)C(F)(F)C(F)(F)C(F)(F)F)N(=O)=O		-7.02	0.64	2.52	P	OUT
QL04096	3-(Nonafluorobutyl)-1-phenyl-1H-pyrazol-5-ol	96146-02-0	Oc1cc(nn1-c1ccccc1)C(F)(F)C(F)(F)C(F)(F)C(F)(F)F		-7.11	0.64	2.55	P	IN
QL04097	4-Bromo-2-[4,4,5,5,6,6,7,7,8,9,9,9-dodecafluoro-8-(trifluoromethyl)nonyl]phenol	610778-18-2	Oc1ccc(Br)cc1CCCC(F)(F)C(F)(F)C(F)(F)C(F)(F)C(F)(C(F)(F)F)C(F)(F)F		-7.81	0.65	2.71	P	IN
QL04098	2,2,3,3,4,4,5,5,6,6,7,7,8,8,8-Pentadecafluorooctyl 4-hydroxybenzoate	869308-35-0	Oc1ccc(cc1)C(=O)OCC(F)(F)C(F)(F)C(F)(F)C(F)(F)C(F)(F)C(F)(F)C(F)(F)F		-8.40	0.66	2.96	P	IN
QL04099	4-(1,1,1,2,3,3,3-Heptafluoropropan-2-yl)phenol	71360-30-0	Oc1ccc(cc1)C(F)(C(F)(F)F)C(F)(F)F		-5.86	0.64	1.90	P	IN
QL04100	4-(Tridecafluorohexyl)phenol	106877-15-0	Oc1ccc(cc1)C(F)(F)C(F)(F)C(F)(F)C(F)(F)C(F)(F)C(F)(F)F		-7.72	0.66	2.52	P	IN
QL04101	3-Fluoro-4-{(E)-[4'-(heptafluoropropyl)[1,1'-biphenyl]-4-yl]diazenyl}phenol	113448-90-1	Oc1ccc(N=Nc2ccc(cc2)-c2ccc(cc2)C(F)(F)C(F)(F)C(F)(F)F)c(F)c1		-7.88	0.62	3.23	P	OUT
QL04102	4-[(2,2,3,3,4,4,5,5,6,6,6-Undecafluorohexyl)oxy]phenol	129560-96-9	Oc1ccc(OCC(F)(F)C(F)(F)C(F)(F)C(F)(F)C(F)(F)F)cc1		-7.63	0.65	2.68	P	IN
QL04103	4-(2,2,3,3,4,4,4-Heptafluorobutoxy)phenol	129560-95-8	Oc1ccc(OCC(F)(F)C(F)(F)C(F)(F)F)cc1		-6.72	0.63	2.44	P	OUT
QL04104	4-[(3,3,4,4,5,5,6,6,6-Nonafluorohexyl)oxy]phenol	129560-98-1	Oc1ccc(OCCC(F)(F)C(F)(F)C(F)(F)C(F)(F)F)cc1		-7.21	0.64	2.64	P	OUT
QL04105	4-Hydroxyphenyl nonafluorobutane-1-sulfonate	1463426-93-8	Oc1ccc(OS(=O)(=O)C(F)(F)C(F)(F)C(F)(F)C(F)(F)F)cc1		-7.47	0.65	2.53	P	IN
QL04106	3-(6,6,7,7,8,8,9,9,10,10,11,11,11-Tridecafluoroundecyl)benzene-1,2-diol	131545-70-5	Oc1cccc(CCCCCC(F)(F)C(F)(F)C(F)(F)C(F)(F)C(F)(F)C(F)(F)F)c1O		-7.91	0.64	2.91	P	OUT
QL04107	3-(12,12,13,13,14,14,15,15,15-Nonafluoropentadecyl)benzene-1,2-diol	131545-71-6	Oc1cccc(CCCCCCCCCCCC(F)(F)C(F)(F)C(F)(F)C(F)(F)F)c1O		-7.36	0.60	3.16	P	OUT
QL04108	3-[(2,2,3,3,4,4,5,5,6,6,7,7,7-Tridecafluoroheptyl)amino]phenol	1441382-30-4	Oc1cccc(NCC(F)(F)C(F)(F)C(F)(F)C(F)(F)C(F)(F)C(F)(F)F)c1		-7.81	0.65	2.71	P	IN

QL04109	3-(Heptadecafluorooctyl)oxan-2-ol	169213-95-0	OC1OCCCC1C(F)(F)C(F)(F)C(F)(F)C(F)(F)C(F)(F)C(F)(F)C(F)(F)C(F)(F)F		-8.18	0.67	2.68	P	IN
QL04110	3-(Tridecafluorohexyl)oxan-2-ol	169213-94-9	OC1OCCCC1C(F)(F)C(F)(F)C(F)(F)C(F)(F)C(F)(F)C(F)(F)F		-7.51	0.66	2.45	P	IN
QL04111	2-(3,3,4,4,5,5,6,6,6-Nonafluorohexyl)propane-1,3-diol	152220-76-3	OCC(CO)CCC(F)(F)C(F)(F)C(F)(F)C(F)(F)F		-6.38	0.63	2.26	P	OUT
QL04112	1H,1H,3H-Perfluoro(2,4-dimethylpentan-1-ol)	25065-50-3	OCC(F)(C(F)C(F)(C(F)(F)F)C(F)(F)F)C(F)(F)F		-5.99	0.66	1.65	P	IN
QL04113	1H,1H-Perfluoro(3,5,5-trimethylhexan-1-ol)	232267-34-4	OCC(F)(F)C(F)(C(F)(F)F)C(F)(F)C(C(F)(F)F)(C(F)(F)F)C(F)(F)F		-6.57	0.67	1.82	P	IN
QL04114	2,2,3,4,4,5,5,6,6,7,8,8,8-Tridecafluoro-3,7-bis(trifluoromethyl)octan-1-ol	232587-50-7	OCC(F)(F)C(F)(C(F)(F)F)C(F)(F)C(F)(F)C(F)(F)C(F)(C(F)(F)F)C(F)(F)F		-7.50	0.68	2.20	P	IN
QL04115	2,2,3,4,4,4-Hexafluoro-3-(trifluoromethyl)butan-1-ol	782390-93-6	OCC(F)(F)C(F)(C(F)(F)F)C(F)(F)F		-5.51	0.66	1.43	P	IN
QL04116	1h,1h-perfluorooctadecan-1-ol	242142-82-1	OCC(F)(F)C(F)(F)C(F)(F)C(F)(F)C(F)(F)C(F)(F)C(F)(F)C(F)(F)C(F)(F)C(F)(F)C(F)(F)C(F)(F)C(F)(F)C(F)(F)C(F)(F)C(F)(F)C(F)(F)F		-9.96	0.69	3.27	P	OUT
QL04117	1H,1H-Perfluorohexadecan-1-ol	216144-94-4	OCC(F)(F)C(F)(F)C(F)(F)C(F)(F)C(F)(F)C(F)(F)C(F)(F)C(F)(F)C(F)(F)C(F)(F)C(F)(F)C(F)(F)C(F)(F)C(F)(F)C(F)(F)F		-9.61	0.69	3.11	P	OUT
QL04118	1H,1H-Perfluoro-1-tetradecanol	15622-57-8	OCC(F)(F)C(F)(F)C(F)(F)C(F)(F)C(F)(F)C(F)(F)C(F)(F)C(F)(F)C(F)(F)C(F)(F)C(F)(F)C(F)(F)C(F)(F)F		-9.23	0.69	2.94	P	OUT
QL04119	111 Fluorotelomer alcohol	423-65-4	OCC(F)(F)C(F)(F)C(F)(F)C(F)(F)C(F)(F)C(F)(F)C(F)(F)C(F)(F)C(F)(F)C(F)(F)C(F)(F)F		-8.78	0.69	2.74	P	IN
QL04120	101 Fluorotelomer alcohol	307-46-0	OCC(F)(F)C(F)(F)C(F)(F)C(F)(F)C(F)(F)C(F)(F)C(F)(F)C(F)(F)C(F)(F)C(F)(F)F		-8.63	0.68	2.68	P	IN
QL04121	91 Fluorotelomer alcohol	307-37-9	OCC(F)(F)C(F)(F)C(F)(F)C(F)(F)C(F)(F)C(F)(F)C(F)(F)C(F)(F)C(F)(F)F		-8.24	0.68	2.51	P	IN
QL04122	1H,1H,10H,10H-Perfluorodecane-1,10-diol	754-96-1	OCC(F)(F)C(F)(F)C(F)(F)C(F)(F)C(F)(F)C(F)(F)C(F)(F)C(F)(F)CO		-7.82	0.67	2.45	P	IN
QL04123	81 Fluorotelomer alcohol	423-56-3	OCC(F)(F)C(F)(F)C(F)(F)C(F)(F)C(F)(F)C(F)(F)C(F)(F)C(F)(F)F		-8.06	0.68	2.44	P	IN
QL04124	1H,1H,8H-Perfluoro-1-octanol	10331-08-5	OCC(F)(F)C(F)(F)C(F)(F)C(F)(F)C(F)(F)C(F)(F)C(F)F		-7.29	0.67	2.21	P	IN
QL04125	1H,1H,8H,8H-Perfluorooctane-1,8-diol	90177-96-1	OCC(F)(F)C(F)(F)C(F)(F)C(F)(F)C(F)(F)C(F)(F)CO		-7.05	0.66	2.16	P	IN
QL04126	1H,1H,6H,6H-Perfluoro-1,6-hexanediol	355-74-8	OCC(F)(F)C(F)(F)C(F)(F)C(F)(F)CO		-5.99	0.65	1.81	P	IN
QL04127	1H,1H-Perfluoro-1-pentanol	355-28-2	OCC(F)(F)C(F)(F)C(F)(F)C(F)(F)F		-6.40	0.67	1.81	P	IN
QL04128	Hexafluoroamylene glycol	376-90-9	OCC(F)(F)C(F)(F)C(F)(F)CO		-5.53	0.64	1.74	P	IN
QL04129	Heptafluorobutanol	375-01-9	OCC(F)(F)C(F)(F)C(F)(F)F		-5.48	0.66	1.46	P	IN
QL04130	21 Fluorotelomer alcohol	422-05-9	OCC(F)(F)C(F)(F)F		-4.95	0.64	1.39	P	IN
QL04131	1H,1H,3H-Perfluorobutanol	NOCAS_870523	OCC(F)(F)C(F)C(F)(F)F		-4.85	0.63	1.49	P	OUT
QL04132	2,2,3,3-Tetrafluoro-1-propanol	76-37-9	OCC(F)(F)C(F)F		-4.03	0.61	1.41	P	OUT
QL04133	2,2,3-Trifluoro-3-(heptafluoropropoxy)propan-1-ol	73320-57-7	OCC(F)(F)C(F)OC(F)(F)C(F)(F)C(F)(F)F		-6.66	0.65	2.11	P	IN
QL04134	1h,1h-perfluoro-3,6-dioxadecan-1-ol	152914-73-3	OCC(F)(F)OC(F)(F)C(F)(F)OC(F)(F)C(F)(F)C(F)(F)C(F)(F)F		-7.78	0.67	2.51	P	IN
QL04135	1h,1h-perfluoro-3,6,9-trioxatridecan-1-ol	317817-24-6	OCC(F)(F)OC(F)(F)C(F)(F)OC(F)(F)C(F)(F)OC(F)(F)C(F)(F)C(F)(F)C(F)(F)F		-8.61	0.67	2.89	P	IN
QL04136	1H,1H,11H,11H-Perfluorotetraethylene glycol	330562-44-2	OCC(F)(F)OC(F)(F)C(F)(F)OC(F)(F)C(F)(F)OC(F)(F)CO		-7.27	0.64	2.54	P	IN
QL04137	Fluorinated triethylene glycol monomethyl ether	147492-57-7	OCC(F)(F)OC(F)(F)C(F)(F)OC(F)(F)C(F)(F)OC(F)(F)F		-7.42	0.65	2.52	P	IN
QL04138	1H,1H-Nonafluoro-3,6-dioxaheptan-1-ol	330562-43-1	OCC(F)(F)OC(F)(F)C(F)(F)OC(F)(F)F		-6.49	0.64	2.21	P	IN
QL04139	2,3,3,3-Tetrafluoro-2-(perfluoropentoxy)propan-1-ol	78693-85-3	OCC(F)(OC(F)(F)C(F)(F)C(F)(F)C(F)(F)C(F)(F)F)C(F)(F)F		-7.40	0.67	2.22	P	IN
QL04140	2-(Perfluoropropoxy)-1H,1H-perfluoropropanol	26537-88-2	OCC(F)(OC(F)(F)C(F)(F)C(F)(F)F)C(F)(F)F		-6.48	0.66	1.87	P	IN
QL04141	Perfluoro(2-(2-propoxypropoxy)-1H,1H-propan-1-ol)	14548-74-4	OCC(F)(OC(F)(F)C(F)(OC(F)(F)C(F)(F)C(F)(F)F)C(F)(F)F)C(F)(F)F		-7.72	0.67	2.42	P	IN
QL04142	2,3,3,3-Tetrafluoro-2-{1,1,2,3,3,3-hexafluoro-2-[1,1,2,3,3,3-hexafluoro-2-(heptafluoropropoxy)propoxy]propoxy}propan-1-ol	14620-81-6	OCC(F)(OC(F)(F)C(F)(OC(F)(F)C(F)(OC(F)(F)C(F)(F)C(F)(F)F)C(F)(F)F)C(F)(F)F)C(F)(F)F		-8.30	0.67	2.67	P	IN
QL04143	2,4,4,5,7,7,8,10,10,11,13,13,14,14,15,15,15-Heptadecafluoro-2,5,8,11-tetrakis(trifluoromethyl)-3,6,9,12-tetraoxapentadecan-1-ol	141977-66-4	OCC(F)(OC(F)(F)C(F)(OC(F)(F)C(F)(OC(F)(F)C(F)(OC(F)(F)C(F)(F)C(F)(F)F)C(F)(F)F)C(F)(F)F)C(F)(F)F)C(F)(F)F		-9.01	0.67	3.01	P	OUT
QL04144	2,4,4,5,7,7,8,10,10,11,13,13,14,16,16,17,17,18,18,18-Icosafluoro-2,5,8,11,14-pentakis(trifluoromethyl)-3,6,9,12,15-pentaoxaoctadecan-1-ol	27617-34-1	OCC(F)(OC(F)(F)C(F)(OC(F)(F)C(F)(OC(F)(F)C(F)(OC(F)(F)C(F)(OC(F)(F)C(F)(F)C(F)(F)F)C(F)(F)F)C(F)(F)F)C(F)(F)F)C(F)(F)F)C(F)(F)F		-9.40	0.68	3.19	P	OUT
QL04145	2,4,4,5,7,7,8,10,10,11,13,13,14,16,16,17,19,19,20,20,21,21,21-Tricosafluoro-2,5,8,11,14,17-hexakis(trifluoromethyl)-3,6,9,12,15,18-hexaoxahenicosan-1-ol	167631-99-4	OCC(F)(OC(F)(F)C(F)(OC(F)(F)C(F)(OC(F)(F)C(F)(OC(F)(F)C(F)(OC(F)(F)C(F)(OC(F)(F)C(F)(F)C(F)(F)F)C(F)(F)F)C(F)(F)F)C(F)(F)F)C(F)(F)F)C(F)(F)F)C(F)(F)F		-9.90	0.68	3.44	P	OUT

QL04147	4,5,5,5-Tetrafluoro-2-iodo-4-(trifluoromethyl)pent-2-en-1-ol	261760-25-2	OCC(I)=CC(F)(C(F)(F)F)C(F)(F)F		-5.33	0.63	1.69	P	IN
QL04149	4,4,5,5,6,6,7,7,8,8,9,9,9-Tridecafluoro-2-iodonon-2-en-1-ol	261761-01-7	OCC(I)=CC(F)(F)C(F)(F)C(F)(F)C(F)(F)C(F)(F)C(F)(F)F		-7.54	0.66	2.45	P	IN
QL04150	4,4,5,5,6,6,6-heptafluoro-2-iodohex-2-en-1-ol	92835-82-0	OCC(I)=CC(F)(F)C(F)(F)C(F)(F)F		-5.87	0.64	1.93	P	IN
QL04152	4,5,5,5-Tetrafluoro-2-iodo-4-(trifluoromethyl)pentan-1-ol	114810-56-9	OCC(I)CC(F)(C(F)(F)F)C(F)(F)F		-5.33	0.63	1.69	P	IN
QL04153	4,4,5,5,6,6,7,7,8,9,9,9-dodecafluoro-2-iodo-8-(trifluoromethyl)nonan-1-ol	16083-62-8	OCC(I)CC(F)(F)C(F)(F)C(F)(F)C(F)(F)C(F)(C(F)(F)F)C(F)(F)F		-7.53	0.66	2.43	P	IN
QL04154	4,4,5,5,6,6,7,7,8,8,9,9,10,11,11,11-Hexadecafluoro-2-iodo-10-(trifluoromethyl)undecan-1-ol	65726-35-4	OCC(I)CC(F)(F)C(F)(F)C(F)(F)C(F)(F)C(F)(F)C(F)(F)C(F)(C(F)(F)F)C(F)(F)F		-8.20	0.67	2.67	P	IN
QL04155	4,4,5,5,6,6,7,7,8,8,9,9,10,10,11,11,12,12,13,13,14,14,15,15,15-Pentacosafluoro-2-iodopentadecan-1-ol	80459-24-1	OCC(I)CC(F)(F)C(F)(F)C(F)(F)C(F)(F)C(F)(F)C(F)(F)C(F)(F)C(F)(F)C(F)(F)C(F)(F)C(F)(F)C(F)(F)F		-9.20	0.68	3.07	P	OUT
QL04156	4,4,5,5,6,6,7,7,8,8,9,9,10,10,11,11,12,12,13,13,13-Henicosafluoro-2-iodotridecan-1-ol	38550-46-8	OCC(I)CC(F)(F)C(F)(F)C(F)(F)C(F)(F)C(F)(F)C(F)(F)C(F)(F)C(F)(F)C(F)(F)C(F)(F)F		-8.75	0.67	2.89	P	IN
QL04157	4,4,5,5,6,6,7,7,8,8,9,9,10,10,11,11,11-Heptadecafluoro-2-iodoundecan-1-ol	38550-45-7	OCC(I)CC(F)(F)C(F)(F)C(F)(F)C(F)(F)C(F)(F)C(F)(F)C(F)(F)C(F)(F)F		-8.21	0.67	2.69	P	IN
QL04158	2-Iodo-1h,1h,2h,3h,3h-perfluorodecan-1-ol	16083-64-0	OCC(I)CC(F)(F)C(F)(F)C(F)(F)C(F)(F)C(F)(F)C(F)(F)C(F)(F)F		-7.77	0.67	2.51	P	IN
QL04159	4,4,5,5,6,6,7,7,8,8,9,9,9-Tridecafluoro-2-iodononan-1-ol	38550-44-6	OCC(I)CC(F)(F)C(F)(F)C(F)(F)C(F)(F)C(F)(F)C(F)(F)F		-7.54	0.66	2.45	P	IN
QL04160	4,4,5,5,6,6,7,7,7-Nonafluoro-2-iodoheptan-1-ol	80233-96-1	OCC(I)CC(F)(F)C(F)(F)C(F)(F)C(F)(F)F		-6.67	0.65	2.19	P	IN
QL04161	4,4,5,5,6,6,6-Heptafluoro-2-iodohexan-1-ol	647-84-7	OCC(I)CC(F)(F)C(F)(F)C(F)(F)F		-5.87	0.64	1.93	P	IN
QL04162	3,3,4,4,5,5,6,6,6-Nonafluorohexane-1,2-diol	124995-00-2	OCC(O)C(F)(F)C(F)(F)C(F)(F)C(F)(F)F		-6.37	0.66	1.90	P	IN
QL04163	1-(Perfluorofluorooctyl)propane-2,3-diol	94159-84-9	OCC(O)CC(F)(F)C(F)(F)C(F)(F)C(F)(F)C(F)(F)C(F)(F)C(F)(F)C(F)(F)F		-8.21	0.67	2.69	P	IN
QL04164	4,4,5,5,6,6,7,7,8,8,9,9,9-tridecafluorononane-1,2-diol	107650-06-6	OCC(O)CC(F)(F)C(F)(F)C(F)(F)C(F)(F)C(F)(F)C(F)(F)F		-7.54	0.66	2.45	P	IN
QL04165	3-(Perfluoro-2-butyl)propane-1,2-diol	125070-38-4	OCC(O)CC(F)(F)C(F)(F)C(F)(F)C(F)(F)F		-6.67	0.65	2.19	P	IN
QL04166	3-(Perfluoro-1-propyl)-1,2-propanediol	1992-91-2	OCC(O)CC(F)(F)C(F)(F)C(F)(F)F		-5.87	0.64	1.93	P	IN
QL04167	1,2-Decanediol, 5,5,6,6,7,7,8,8,9,9,10,10,10-tridecafluoro-	147274-18-8	OCC(O)CCC(F)(F)C(F)(F)C(F)(F)C(F)(F)C(F)(F)C(F)(F)F		-7.55	0.66	2.52	P	IN
QL04168	3-(2,2,3,3,4,4,4-Heptafluorobutoxy)propane-1,2-diol	25385-72-2	OCC(O)COCC(F)(F)C(F)(F)C(F)(F)F		-6.09	0.62	2.23	P	OUT
QL04169	3-(2,2,3,3-Tetrafluoropropoxy)propane-1,2-diol	25385-68-6	OCC(O)COCC(F)(F)C(F)F		-4.81	0.58	2.13	P	OUT
QL04170	3-[(3,3,4,4,5,5,6,6,7,7,8,8,8-Tridecafluorooctyl)oxy]propane-1,2-diol	126814-93-5	OCC(O)COCCC(F)(F)C(F)(F)C(F)(F)C(F)(F)C(F)(F)C(F)(F)F		-7.72	0.64	2.77	P	IN
QL04171	3-[(3,3,4,4,5,5,6,6,6-Nonafluorohexyl)oxy]propane-1,2-diol	135548-22-0	OCC(O)COCCC(F)(F)C(F)(F)C(F)(F)C(F)(F)F		-6.77	0.63	2.51	P	OUT
QL04172	Bis(2-hydroxyethyl)ammonium bis(3,3,4,4,5,5,6,6,7,7,8,8,9,9,10,10,11,11,12,12,13,14,14,14-tetracosafluoro-13-(trifluoromethyl)tetradecyl) phosphate	94231-56-8	OCC[NH2+]CCO.[O-]P(=O)(OCCC(F)(F)C(F)(F)C(F)(F)C(F)(F)C(F)(F)C(F)(F)C(F)(F)C(F)(F)C(F)(F)C(F)(F)C(F)(C(F)(F)F)C(F)(F)F)OCCC(F)(F)C(F)(F)C(F)(F)C(F)(F)C(F)(F)C(F)(F)C(F)(F)C(F)(F)C(F)(F)C(F)(F)C(F)(C(F)(F)F)C(F)(F)F		-11.04	0.67	4.15	P	OUT
QL04173	Bis(2-hydroxyethyl)ammonium bis[3,3,4,4,5,5,6,6,7,7,8,8,9,9,10,10,11,11,12,12,13,13,14,14,15,16,16,16-octacosafluoro-15-(trifluoromethyl)hexadecyl] phosphate	93776-30-8	OCC[NH2+]CCO.[O-]P(=O)(OCCC(F)(F)C(F)(F)C(F)(F)C(F)(F)C(F)(F)C(F)(F)C(F)(F)C(F)(F)C(F)(F)C(F)(F)C(F)(F)C(F)(F)C(F)(C(F)(F)F)C(F)(F)F)OCCC(F)(F)C(F)(F)C(F)(F)C(F)(F)C(F)(F)C(F)(F)C(F)(F)C(F)(F)C(F)(F)C(F)(F)C(F)(F)C(F)(F)C(F)(C(F)(F)F)C(F)(F)F		-11.44	0.67	4.30	P	OUT
QL04174	Bis(2-hydroxyethyl)ammonium bis(3,3,4,4,5,5,6,6,7,7,8,8,9,9,10,10,11,11,12,12,13,13,14,14,15,15,16,16,16-nonacosafluorohexadecyl) phosphate	94291-75-5	OCC[NH2+]CCO.[O-]P(=O)(OCCC(F)(F)C(F)(F)C(F)(F)C(F)(F)C(F)(F)C(F)(F)C(F)(F)C(F)(F)C(F)(F)C(F)(F)C(F)(F)C(F)(F)C(F)(F)F)OCCC(F)(F)C(F)(F)C(F)(F)C(F)(F)C(F)(F)C(F)(F)C(F)(F)C(F)(F)C(F)(F)C(F)(F)C(F)(F)C(F)(F)C(F)(F)F		-11.52	0.67	4.35	P	OUT
QL04175	Bis(2-hydroxyethyl)ammonium bis[3,3,4,4,5,5,6,6,7,7,8,8,9,9,10,10,11,11,12,12,13,13,14,14,14-pentacosafluorotetradecyl] phosphate	57678-00-9	OCC[NH2+]CCO.[O-]P(=O)(OCCC(F)(F)C(F)(F)C(F)(F)C(F)(F)C(F)(F)C(F)(F)C(F)(F)C(F)(F)C(F)(F)C(F)(F)C(F)(F)C(F)(F)F)OCCC(F)(F)C(F)(F)C(F)(F)C(F)(F)C(F)(F)C(F)(F)C(F)(F)C(F)(F)C(F)(F)C(F)(F)C(F)(F)C(F)(F)F		-11.13	0.67	4.20	P	OUT
QL04176	Bis(2-hydroxyethyl)ammonium perfluorooctanesulfonic acid	70225-14-8	OCC[NH2+]CCO.[O-]S(=O)(=O)C(F)(F)C(F)(F)C(F)(F)C(F)(F)C(F)(F)C(F)(F)C(F)(F)C(F)(F)F		-7.84	0.65	2.83	P	IN
QL04177	Bis(2-hydroxyethyl)ammonium pentadecafluoroheptane-1-sulphonate	70225-15-9	OCC[NH2+]CCO.[O-]S(=O)(=O)C(F)(F)C(F)(F)C(F)(F)C(F)(F)C(F)(F)C(F)(F)C(F)(F)F		-7.27	0.64	2.60	P	IN
QL04178	Bis(2-hydroxyethyl)ammonium perfluorohexanesulfonate	70225-16-0	OCC[NH2+]CCO.[O-]S(=O)(=O)C(F)(F)C(F)(F)C(F)(F)C(F)(F)C(F)(F)C(F)(F)F		-6.70	0.63	2.40	P	OUT

QL04179	Bis(2-hydroxyethyl)ammonium 1,1,2,2,3,3,4,4,5,5,5-undecafluoropentane-1-sulphonate	70225-17-1	OCC[NH2+]CCO.[O-]S(=O)(=O)C(F)(F)C(F)(F)C(F)(F)C(F)(F)C(F)(F)F		-5.92	0.63	2.11	P	OUT
QL04180	Bis(2-hydroxyethyl)ammonium perfluorobutanesulfonate	70225-18-2	OCC[NH2+]CCO.[O-]S(=O)(=O)C(F)(F)C(F)(F)C(F)(F)C(F)(F)F		-5.18	0.62	1.86	P	OUT
QL04181	4,4,5,5,6,6,7,7,7-Nonafluorohept-2-yn-1-ol	81190-31-0	OCC#CC(F)(F)C(F)(F)C(F)(F)C(F)(F)F		-6.48	0.64	2.21	P	IN
QL04182	(2Z)-4,5,5,5-Tetrafluoro-4-(trifluoromethyl)pent-2-en-1-ol	83706-99-4	OCC=C/C(F)(C(F)(F)F)C(F)(F)F		-5.05	0.62	1.69	P	OUT
QL04183	(2Z)-4,4,5,5,6,6,7,7,8,8,9,9,9-Tridecafluoronon-2-en-1-ol	83707-01-1	OCC=C/C(F)(F)C(F)(F)C(F)(F)C(F)(F)C(F)(F)C(F)(F)F		-7.41	0.66	2.46	P	IN
QL04184	(2E)-4,5,5,5-Tetrafluoro-4-(trifluoromethyl)pent-2-en-1-ol	83706-95-0	OCC=CC(F)(C(F)(F)F)C(F)(F)F		-5.05	0.62	1.69	P	OUT
QL04185	4,4,5,5,6,6,7,7,8,8,9,9,10,10,11,11,11-heptadecafluoroundec-2-en-1-ol	2340-84-3	OCC=CC(F)(F)C(F)(F)C(F)(F)C(F)(F)C(F)(F)C(F)(F)C(F)(F)C(F)(F)F		-8.10	0.66	2.68	P	IN
QL04186	4,4,5,5,6,6,7,7,8,8,9,9,9-Tridecafluoro-2-nonen-1-ol	38550-47-9	OCC=CC(F)(F)C(F)(F)C(F)(F)C(F)(F)C(F)(F)C(F)(F)F		-7.41	0.66	2.46	P	IN
QL04189	4,4,5,5,6,6,7,7,7-Nonafluorohept-2-en-1-ol	72016-05-8	OCC=CC(F)(F)C(F)(F)C(F)(F)C(F)(F)F		-6.48	0.64	2.21	P	IN
QL04190	4,4,5,5,6,6,6-Heptafluorohex-2-en-1-ol	679-03-8	OCC=CC(F)(F)C(F)(F)C(F)(F)F		-5.63	0.63	1.95	P	OUT
QL04192	(Perfluorocyclohexyl)methanol	28788-68-3	OCC1(F)C(F)(F)C(F)(F)C(F)(F)C(F)(F)C1(F)F		-6.07	0.68	1.38	P	IN
QL04193	[4-(Heptadecafluorooctyl)phenyl]methanol	163114-33-8	OCC1=CC=C(C=C1)C(F)(F)C(F)(F)C(F)(F)C(F)(F)C(F)(F)C(F)(F)C(F)(F)C(F)(F)F		-8.46	0.66	2.90	P	IN
QL04194	[4-(3,3,4,4,5,5,6,6,7,7,8,8,9,9,10,10,10-Heptadecafluorodecyl)phenyl]methanol	356055-77-1	OCC1=CC=C(CCC(F)(F)C(F)(F)C(F)(F)C(F)(F)C(F)(F)C(F)(F)C(F)(F)C(F)(F)F)C=C1		-8.65	0.66	3.09	P	IN
QL04195	4-((Perfluorohexyl)ethyl)phenylmethanol	356055-76-0	OCC1=CC=C(CCC(F)(F)C(F)(F)C(F)(F)C(F)(F)C(F)(F)C(F)(F)F)C=C1		-7.99	0.65	2.88	P	IN
QL04196	[4-(3,3,4,4,5,5,6,6,6-Nonafluorohexyl)phenyl]methanol	892154-66-4	OCC1=CC=C(CCC(F)(F)C(F)(F)C(F)(F)C(F)(F)F)C=C1		-7.20	0.63	2.68	P	OUT
QL04197	{4-[(4,4,5,5,6,6,7,7,8,8,9,9,10,10,11,11,11-Heptadecafluoroundecyl)oxy]phenyl}methanol	892155-69-0	OCC1=CC=C(OCCCC(F)(F)C(F)(F)C(F)(F)C(F)(F)C(F)(F)C(F)(F)C(F)(F)C(F)(F)F)C=C1		-8.68	0.65	3.23	P	OUT
QL04198	{4-[(4,4,5,5,6,6,7,7,8,8,9,9,9-Tridecafluorononyl)oxy]phenyl}methanol	957206-65-4	OCC1=CC=C(OCCCC(F)(F)C(F)(F)C(F)(F)C(F)(F)C(F)(F)C(F)(F)F)C=C1		-8.04	0.64	3.04	P	OUT
QL04199	4-[3-(Perfluorobutyl)-1-propyloxy]benzylÂ alcohol	892154-51-7	OCC1=CC=C(OCCCC(F)(F)C(F)(F)C(F)(F)C(F)(F)F)C=C1		-7.28	0.62	2.87	P	OUT
QL04200	{3,4,5-Tris[1,1,2-trifluoro-2-(heptafluoropropoxy)ethoxy]phenyl}methanol	1233312-87-2	OCc1cc(OC(F)(F)C(F)OC(F)(F)C(F)(F)C(F)(F)F)c(OC(F)(F)C(F)OC(F)(F)C(F)(F)C(F)(F)F)c(OC(F)(F)C(F)OC(F)(F)C(F)(F)C(F)(F)F)c1		-8.82	0.66	3.17	P	OUT
QL04201	[4-(1,1,1,2,3,3,3-Heptafluoropropan-2-yl)phenyl]methanol	1137162-24-3	OCc1ccc(cc1)C(F)(C(F)(F)F)C(F)(F)F		-6.03	0.63	2.15	P	OUT
QL04202	3-(Heptafluoropropyl)pentane-1,5-diol	678-93-3	OCCC(CCO)C(F)(F)C(F)(F)C(F)(F)F		-5.03	0.61	1.92	P	OUT
QL04203	2-(Heptadecafluorooctyl)butane-1,4-diol	135440-42-5	OCCC(CO)C(F)(F)C(F)(F)C(F)(F)C(F)(F)C(F)(F)C(F)(F)C(F)(F)C(F)(F)F		-7.92	0.66	2.69	P	IN
QL04204	2(Perfluoro-2-propyl)ethanol	90999-87-4	OCCC(F)(C(F)(F)F)C(F)(F)F		-4.78	0.63	1.42	P	IN
QL04205	3,3,4,4,5,6,6,6-Octafluoro-5-(trifluoromethyl)hexan-1-ol	89076-11-9	OCCC(F)(F)C(F)(F)C(F)(C(F)(F)F)C(F)(F)F		-6.17	0.65	1.87	P	IN
QL04206	3,3,4,4,5,5,6,6,7,8,8,8-Dodecafluoro-7-(trifluoromethyl)octan-1-ol	20015-46-7	OCCC(F)(F)C(F)(F)C(F)(F)C(F)(F)C(F)(C(F)(F)F)C(F)(F)F		-7.17	0.66	2.22	P	IN
QL04207	2-(Perfluoro-7-methyloctyl)ethanol	31200-98-3	OCCC(F)(F)C(F)(F)C(F)(F)C(F)(F)C(F)(F)C(F)(F)C(F)(C(F)(F)F)C(F)(F)F		-7.90	0.67	2.50	P	IN
QL04208	3,3,4,4,5,5,6,6,7,7,8,8,9,9,10,10,11,12,12,12-Icosafluoro-11-(trifluoromethyl)dodecan-1-ol	194427-39-9	OCCC(F)(F)C(F)(F)C(F)(F)C(F)(F)C(F)(F)C(F)(F)C(F)(F)C(F)(F)C(F)(C(F)(F)F)C(F)(F)F		-8.49	0.68	2.73	P	IN
QL04209	3,3,4,4,5,5,6,6,7,7,8,8,9,9,10,10,11,11,12,12,13,13,14,14,15,15,15-Heptacosafluoropentadecan-1-ol	176676-70-3	OCCC(F)(F)C(F)(F)C(F)(F)C(F)(F)C(F)(F)C(F)(F)C(F)(F)C(F)(F)C(F)(F)C(F)(F)C(F)(F)C(F)(F)C(F)(F)F		-9.29	0.68	3.06	P	OUT
QL04210	3,3,4,4,5,5,6,6,7,7,8,8,9,9,10,10,11,11,12,12,13,13,13-Tricosafluorotridecan-1-ol	1545-59-1	OCCC(F)(F)C(F)(F)C(F)(F)C(F)(F)C(F)(F)C(F)(F)C(F)(F)C(F)(F)C(F)(F)C(F)(F)C(F)(F)F		-8.85	0.68	2.88	P	OUT
QL04212	3,3,4,4,5,5,6,6,7,7,8,8,9,9,10,10,11,11,11-Nonadecafluoroundecan-1-ol	87017-97-8	OCCC(F)(F)C(F)(F)C(F)(F)C(F)(F)C(F)(F)C(F)(F)C(F)(F)C(F)(F)C(F)(F)F		-8.33	0.67	2.68	P	IN
QL04213	3,3,4,4,5,5,6,6,7,7,8,8,9,9,9-Pentadecafluorononan-1-ol	755-02-2	OCCC(F)(F)C(F)(F)C(F)(F)C(F)(F)C(F)(F)C(F)(F)C(F)(F)F		-7.69	0.67	2.44	P	IN
QL04214	52 Fluorotelomer alcohol	185689-57-0	OCCC(F)(F)C(F)(F)C(F)(F)C(F)(F)C(F)(F)F		-6.87	0.66	2.17	P	IN
QL04216	32 Fluorotelomer alcohol	755-40-8	OCCC(F)(F)C(F)(F)C(F)(F)F		-5.71	0.64	1.85	P	IN
QL04217	22 fluorotelomer alcohol	54949-74-5	OCCC(F)(F)C(F)(F)F		-4.54	0.62	1.52	P	OUT

QL04218	Poly(difluoromethylene), .alpha.-fluoro-.omega.-(2-hydroxyethyl)-, dihydrogen 2-hydroxy-1,2,3-propanetricarboxylate|Poly(oxy-1,2-ethanediyl), alpha-hydro-omega-hydroxy-, ether with alpha-fluoro-omega-(2-hydroxyethyl)poly(difluoromethylene) (11)|Poly(difluoromethylene), .alpha.-fluoro-.omega.-(2-hydroxyethyl)-	65605-56-3|65545-80-4|65530-60-1	OCCC(F)(F)F		-2.78	0.56	1.38	P	OUT
QL04219	5,5,6,6,7,7,8,8,9,9,10,10,10-Tridecafluoro-3-iododecan-1-ol	16083-68-4	OCCC(I)CC(F)(F)C(F)(F)C(F)(F)C(F)(F)C(F)(F)C(F)(F)F		-7.42	0.65	2.52	P	IN
QL04220	5,5,6,6,7,7,8,8,8-Nonafluoro-3-iodooctan-1-ol	109574-81-4	OCCC(I)CC(F)(F)C(F)(F)C(F)(F)C(F)(F)F		-6.51	0.64	2.27	P	IN
QL04221	5,5,6,6,7,7,7-Heptafluoro-3-iodoheptan-1-ol	89621-93-2	OCCC(I)CC(F)(F)C(F)(F)C(F)(F)F		-5.95	0.62	2.15	P	OUT
QL04222	5,5,6,6,7,7,7-Heptafluoro-4,4-bis(trifluoromethyl)heptan-1-ol	110254-90-5	OCCCC(C(F)(F)F)(C(F)(F)F)C(F)(F)C(F)(F)C(F)(F)F		-6.43	0.65	2.04	P	IN
QL04223	3-(Perfluoroisopropyl)propanol	29819-73-6	OCCCC(F)(C(F)(F)F)C(F)(F)F		-5.05	0.62	1.69	P	OUT
QL04224	3-(Perfluorooctyl)propanol	1651-41-8	OCCCC(F)(F)C(F)(F)C(F)(F)C(F)(F)C(F)(F)C(F)(F)C(F)(F)C(F)(F)F		-8.10	0.66	2.68	P	IN
QL04225	73 fluorotelomer alcohol	25600-66-2	OCCCC(F)(F)C(F)(F)C(F)(F)C(F)(F)C(F)(F)C(F)(F)C(F)(F)F		-7.64	0.66	2.51	P	IN
QL04226	3-(Perfluorohexyl)propanol	80806-68-4	OCCCC(F)(F)C(F)(F)C(F)(F)C(F)(F)C(F)(F)C(F)(F)F		-7.41	0.66	2.46	P	IN
QL04227	4,4,5,5,6,6,7,7,8,8,8-Undecafluorooctan-1-ol	1404193-88-9	OCCCC(F)(F)C(F)(F)C(F)(F)C(F)(F)C(F)(F)F		-6.81	0.65	2.25	P	IN
QL04228	3-(Perfluorobutyl)-1-propanol	83310-97-8	OCCCC(F)(F)C(F)(F)C(F)(F)C(F)(F)F		-6.48	0.64	2.21	P	IN
QL04229	3-(Perfluoropropyl)propanol	679-02-7	OCCCC(F)(F)C(F)(F)C(F)(F)F		-5.63	0.63	1.95	P	OUT
QL04230	3-(Perfluoroethyl)propanol	NOCAS_869964	OCCCC(F)(F)C(F)(F)F		-4.83	0.61	1.82	P	OUT
QL04231	4,5,5,5-Tetrafluoro-4-(heptafluoropropoxy)pentan-1-ol	243128-41-8	OCCCC(F)(OC(F)(F)C(F)(F)C(F)(F)F)C(F)(F)F		-6.60	0.64	2.22	P	IN
QL04232	6,6,7,7,8,8,9,9,10,10,11,11,11-Tridecafluoro-4-iodoundecan-1-ol	132665-00-0	OCCCC(I)CC(F)(F)C(F)(F)C(F)(F)C(F)(F)C(F)(F)C(F)(F)F		-7.64	0.65	2.72	P	IN
QL04233	5,5,6,6,7,7,8,8,9,9,10,10,10-Tridecafluorodecan-1-ol	39239-79-7	OCCCCC(F)(F)C(F)(F)C(F)(F)C(F)(F)C(F)(F)C(F)(F)F		-7.35	0.65	2.52	P	IN
QL04234	44 Fluorotelomer alcohol	3792-02-7	OCCCCC(F)(F)C(F)(F)C(F)(F)C(F)(F)F		-6.41	0.63	2.28	P	OUT
QL04235	5,5,6,6,7,7,7-Heptafluoroheptan-1-ol	883543-64-4	OCCCCC(F)(F)C(F)(F)C(F)(F)F		-5.83	0.62	2.17	P	OUT
QL04236	(5E)-7,7,8,8,9,9,10,10,10-Nonafluoro-5-iododec-5-en-1-ol	137648-89-6	OCCCCC(I)=C/C(F)(F)C(F)(F)C(F)(F)C(F)(F)F		-6.69	0.62	2.55	P	OUT
QL04237	6,6,7,7,8,8,9,9,10,10,11,11,11-Tridecafluoroundecan-1-ol	134052-02-1	OCCCCCC(F)(F)C(F)(F)C(F)(F)C(F)(F)C(F)(F)C(F)(F)F		-7.58	0.64	2.72	P	IN
QL04238	6,6,7,7,8,8,9,9,9-Nonafluorononan-1-ol	219557-62-7	OCCCCCC(F)(F)C(F)(F)C(F)(F)C(F)(F)F		-6.57	0.63	2.46	P	OUT
QL04239	7,8,8,8-Tetrafluoro-7-(trifluoromethyl)octan-1-ol	220469-13-6	OCCCCCCC(F)(C(F)(F)F)C(F)(F)F		-5.26	0.60	2.11	P	OUT
QL04240	7,7,8,8,9,9,10,10,11,11,12,12,13,14,14,14-Hexadecafluoro-13-(trifluoromethyl)tetradecan-1-ol	390410-82-9	OCCCCCCC(F)(F)C(F)(F)C(F)(F)C(F)(F)C(F)(F)C(F)(F)C(F)(C(F)(F)F)C(F)(F)F		-8.20	0.65	2.92	P	IN
QL04241	7,7,8,8,9,9,10,10,11,11,12,12,13,13,14,14,14-heptadecafluorotetradecan-1-ol	129794-54-3	OCCCCCCC(F)(F)C(F)(F)C(F)(F)C(F)(F)C(F)(F)C(F)(F)C(F)(F)C(F)(F)F		-8.20	0.65	2.95	P	IN
QL04242	6-(Perfluorohexyl)hexanol	161981-35-7	OCCCCCCC(F)(F)C(F)(F)C(F)(F)C(F)(F)C(F)(F)C(F)(F)F		-7.54	0.64	2.78	P	OUT
QL04243	7,7,8,8,9,9,10,10,10-nonafluorodecan-1-ol	181042-39-7	OCCCCCCC(F)(F)C(F)(F)C(F)(F)C(F)(F)F		-6.52	0.62	2.52	P	OUT
QL04244	9,9,10,10,11,11,12,12,12-Nonafluoro-7-iodododecan-1-ol	172701-28-9	OCCCCCCC(I)CC(F)(F)C(F)(F)C(F)(F)C(F)(F)F		-6.80	0.61	2.75	P	OUT
QL04245	9,9,10,10,11,11,12,12,12-Nonafluorododecan-1-ol	133001-14-6	OCCCCCCCCC(F)(F)C(F)(F)C(F)(F)C(F)(F)F		-6.63	0.61	2.73	P	OUT
QL04246	11-(Perfluorooctyl)undec-10-enol	15364-19-9	OCCCCCCCCCC=CC(F)(F)C(F)(F)C(F)(F)C(F)(F)C(F)(F)C(F)(F)C(F)(F)C(F)(F)F		-8.46	0.63	3.37	P	OUT
QL04247	12,12,13,13,14,14,15,15,16,16,17,17,17-Tridecafluoroheptadec-10-en-1-ol	117543-54-1	OCCCCCCCCCC=CC(F)(F)C(F)(F)C(F)(F)C(F)(F)C(F)(F)C(F)(F)F		-7.78	0.62	3.21	P	OUT
QL04249	12,12,13,13,14,14,15,15,16,16,17,17,18,18,19,19,19-Heptadecafluorononadecan-1-ol	1512-02-3	OCCCCCCCCCCCC(F)(F)C(F)(F)C(F)(F)C(F)(F)C(F)(F)C(F)(F)C(F)(F)C(F)(F)F		-8.46	0.63	3.37	P	OUT
QL04250	12,12,13,13,14,14,15,15,16,16,17,17,17-Tridecafluoroheptadecan-1-ol	134052-01-0	OCCCCCCCCCCCC(F)(F)C(F)(F)C(F)(F)C(F)(F)C(F)(F)C(F)(F)F		-7.78	0.62	3.21	P	OUT
QL04251	12,12,13,13,14,14,15,15,15-Nonafluoropentadecan-1-ol	36096-97-6	OCCCCCCCCCCCC(F)(F)C(F)(F)C(F)(F)C(F)(F)F		-6.89	0.60	3.04	P	OUT
QL04252	9-[(4,4,5,5,5-Pentafluoropentyl)sulfanyl]nonan-1-ol	511545-94-1	OCCCCCCCCCSCCCC(F)(F)C(F)(F)F		-5.84	0.56	3.06	P	OUT
QL04253	6-(2,2,3,3,4,4,4-Heptafluorobutoxy)hexan-1-ol	1207481-10-4	OCCCCCCOCC(F)(F)C(F)(F)C(F)(F)F		-6.11	0.59	2.67	P	OUT
QL04254	3-(1,1,2,2,3,3,4,4,4-Nonafluorobutane-1-sulfonyl)propan-1-ol	762241-70-3	OCCCS(=O)(=O)C(F)(F)C(F)(F)C(F)(F)C(F)(F)F		-6.81	0.65	2.25	P	IN

QL04255	3-[(Nonafluorobutyl)sulfanyl]propan-1-ol	875628-33-4	OCCCSC(F)(F)C(F)(F)C(F)(F)C(F)(F)F		-6.41	0.63	2.28	P	OUT
QL04256	1-Propanol, 3-[(3,3,4,4,5,5,6,6,7,7,8,8,9,9,10,10,10-heptadecafluorodecyl)thio]-	36880-07-6	OCCCSCCC(F)(F)C(F)(F)C(F)(F)C(F)(F)C(F)(F)C(F)(F)C(F)(F)C(F)(F)F		-8.20	0.65	2.95	P	IN
QL04257	Isooctanesulfonamide, heptadecafluoro-N,N-bis(2-hydroxyethyl)-	93894-66-7	OCCN(CCO)[S](=O)(=O)C(F)(F)C(F)(F)C(F)(F)C(F)(F)C(F)(F)C(F)(C(F)(F)F)C(F)(F)F		-7.73	0.65	2.67	P	IN
QL04258	1-[Bis(2-hydroxyethyl)amino]-4,4,5,5,6,6,6-heptafluorohexan-2-ol	189160-58-5	OCCN(CCO)CC(O)CC(F)(F)C(F)(F)C(F)(F)F		-5.78	0.60	2.41	P	OUT
QL04259	2,2'-((3,3,4,4,5,5,6,6,7,7,8,8,9,9,10,10,10-Heptadecafluorodecyl)imino)bisethanol	27607-36-9	OCCN(CCO)CCC(F)(F)C(F)(F)C(F)(F)C(F)(F)C(F)(F)C(F)(F)C(F)(F)C(F)(F)F		-7.97	0.64	2.90	P	IN
QL04260	N-(3-(Bis(2-hydroxyethyl)amino)propyl)-perfluorooctanamide	41358-63-8	OCCN(CCO)CCCNC(=O)C(F)(F)C(F)(F)C(F)(F)C(F)(F)C(F)(F)C(F)(F)C(F)(F)F		-7.92	0.63	3.08	P	OUT
QL04261	1,1-Bis((perfluoro-1-nonyl)ethyloxy)-N-(2-hydroxyethyl)-2-oxa-3-aza-1-phosphapentan-5-ol 1-oxide	101896-22-4	OCCN(CCO)O[P](=O)(OCCC(F)(F)C(F)(F)C(F)(F)C(F)(F)C(F)(F)C(F)(F)C(F)(F)C(F)(F)C(F)(F)F)OCCC(F)(F)C(F)(F)C(F)(F)C(F)(F)C(F)(F)C(F)(F)C(F)(F)C(F)(F)C(F)(F)F		-10.05	0.66	3.71	P	OUT
QL04262	3,7-Bis(2-hydroxyethyl)-5-(((perfluorononyl)ethyl)oxy)-4,6-dioxa-3,7-diaza-5-phosphanonane-1,9-diol 5-oxide	101896-21-3	OCCN(CCO)OP(=O)(OCCC(F)(F)C(F)(F)C(F)(F)C(F)(F)C(F)(F)C(F)(F)C(F)(F)C(F)(F)C(F)(F)F)ON(CCO)CCO		-7.98	0.62	3.21	P	OUT
QL04263	1,1,2,3,3,3-Hexafluoro-N,N-bis(2-hydroxyethyl)-2-(trifluoromethyl)propane-1-sulphonamide	93762-12-0	OCCN(CCO)S(=O)(=O)C(F)(F)C(F)(C(F)(F)F)C(F)(F)F		-5.99	0.63	2.12	P	OUT
QL04264	N,N-Bis(2-hydroxyethyl)-4-(perfluoro(4-ethyl-3,4-dimethylhex-2-en-2-yl)oxy)benzenesulfonamide	93819-97-7	OCCN(CCO)S(=O)(=O)C1=CC=C(OC(=C(C(F)(F)F)C(C(F)(F)F)(C(F)(F)C(F)(F)F)C(F)(F)C(F)(F)F)C(F)(F)F)C=C1		-7.47	0.64	2.78	P	OUT
QL04265	2,2,3,3,4,4,5,5,6,6,7,7,8,8,9,9,9-Heptadecafluoro-N-(2-hydroxyethyl)nonanamide	6104-17-2	OCCNC(=O)C(F)(F)C(F)(F)C(F)(F)C(F)(F)C(F)(F)C(F)(F)C(F)(F)C(F)(F)F		-8.12	0.66	2.74	P	IN
QL04266	2,2,3,3,4,4,5,5,6,6,7,7,7-Tridecafluoro-N-(2-hydroxyethyl)heptanamide	58301-28-3	OCCNC(=O)C(F)(F)C(F)(F)C(F)(F)C(F)(F)C(F)(F)C(F)(F)F		-7.43	0.65	2.52	P	IN
QL04267	2,2,3,3,4,4,5,5,5-Nonafluoro-N-(2-hydroxyethyl)pentanamide	313535-73-8	OCCNC(=O)C(F)(F)C(F)(F)C(F)(F)C(F)(F)F		-6.53	0.64	2.27	P	IN
QL04268	N-(2-Hydroxyethyl)perfluorobutanamide	377-66-2	OCCNC(=O)C(F)(F)C(F)(F)C(F)(F)F		-6.07	0.63	2.20	P	OUT
QL04269	2,3,3,3-Tetrafluoro-2-[1,1,2,3,3,3-hexafluoro-2-(heptafluoropropoxy)propoxy]-N-(2-hydroxyethyl)propanamide	137506-19-5	OCCNC(=O)C(F)(OC(F)(F)C(F)(OC(F)(F)C(F)(F)C(F)(F)F)C(F)(F)F)C(F)(F)F		-7.76	0.65	2.68	P	IN
QL04270	2-((3,3,4,4,5,5,6,6,7,7,8,8,9,9,10,10,10-Heptadecafluorodecyl)amino)ethanol	27607-42-7	OCCNCCC(F)(F)C(F)(F)C(F)(F)C(F)(F)C(F)(F)C(F)(F)C(F)(F)C(F)(F)F		-8.24	0.65	2.91	P	IN
QL04271	2-({3-[3,4,4,4-Tetrafluoro-3-(trifluoromethyl)butane-1-sulfonyl]propyl}amino)ethan-1-ol	1353009-40-1	OCCNCCCS(=O)(=O)CCC(F)(C(F)(F)F)C(F)(F)F		-6.35	0.61	2.57	P	OUT
QL04272	Bis[3,3,4,4,5,5,6,6,7,7,8,8,9,10,10,10-hexadecafluoro-9-(trifluoromethyl)decyl] hydrogen phosphate, compound with 2,2'-iminodiethanol (11)	94291-77-7	OCCNCCO.OP(=O)(OCCC(F)(F)C(F)(F)C(F)(F)C(F)(F)C(F)(F)C(F)(F)C(F)(C(F)(F)F)C(F)(F)F)OCCC(F)(F)C(F)(F)C(F)(F)C(F)(F)C(F)(F)C(F)(F)C(F)(C(F)(F)F)C(F)(F)F		-10.03	0.66	3.79	P	OUT
QL04273	Bis[3,3,4,4,5,5,6,6,7,7,8,8,9,9,10,10,11,12,12,12-icosafluoro-11-(trifluoromethyl)dodecyl] hydrogen phosphate, compound with 2,2'-iminodiethanol (11)	94291-78-8	OCCNCCO.OP(=O)(OCCC(F)(F)C(F)(F)C(F)(F)C(F)(F)C(F)(F)C(F)(F)C(F)(F)C(F)(F)C(F)(C(F)(F)F)C(F)(F)F)OCCC(F)(F)C(F)(F)C(F)(F)C(F)(F)C(F)(F)C(F)(F)C(F)(F)C(F)(F)C(F)(C(F)(F)F)C(F)(F)F		-10.58	0.66	3.98	P	OUT
QL04274	Bis[3,3,4,4,5,5,6,6,7,7,8,8,9,9,10,10,11,11,12,12,13,13,14,14,15,15,16,16,17,18,18,18-dotriacontafluoro-17-(trifluoromethyl)octadecyl] hydrogen phosphate, compound with 2,2'-iminodiethanol (11)	93776-31-9	OCCNCCO.OP(=O)(OCCC(F)(F)C(F)(F)C(F)(F)C(F)(F)C(F)(F)C(F)(F)C(F)(F)C(F)(F)C(F)(F)C(F)(F)C(F)(F)C(F)(F)C(F)(F)C(F)(F)C(F)(C(F)(F)F)C(F)(F)F)OCCC(F)(F)C(F)(F)C(F)(F)C(F)(F)C(F)(F)C(F)(F)C(F)(F)C(F)(F)C(F)(F)C(F)(F)C(F)(F)C(F)(F)C(F)(F)C(F)(F)C(F)(C(F)(F)F)C(F)(F)F		-11.79	0.67	4.44	P	OUT
QL04275	Bis[3,3,4,4,5,5,6,6,7,7,8,8,9,9,10,10,11,11,12,12,13,13,14,14,15,15,16,16,17,17,18,18,18-tritriacontafluorooctadecyl] hydrogen phosphate, compound with 2,2'-iminodiethanol (11)	94291-76-6	OCCNCCO.OP(=O)(OCCC(F)(F)C(F)(F)C(F)(F)C(F)(F)C(F)(F)C(F)(F)C(F)(F)C(F)(F)C(F)(F)C(F)(F)C(F)(F)C(F)(F)C(F)(F)C(F)(F)C(F)(F)C(F)(F)F)OCCC(F)(F)C(F)(F)C(F)(F)C(F)(F)C(F)(F)C(F)(F)C(F)(F)C(F)(F)C(F)(F)C(F)(F)C(F)(F)C(F)(F)C(F)(F)C(F)(F)C(F)(F)C(F)(F)F		-11.87	0.67	4.48	P	OUT
QL04276	Bis[3,3,4,4,5,5,6,6,7,7,8,8,9,9,10,10,11,11,12,12,12-henicosafluorododecyl] hydrogen phosphate, compound with 2,2'-iminodiethanol (11)	57677-98-2	OCCNCCO.OP(=O)(OCCC(F)(F)C(F)(F)C(F)(F)C(F)(F)C(F)(F)C(F)(F)C(F)(F)C(F)(F)C(F)(F)C(F)(F)F)OCCC(F)(F)C(F)(F)C(F)(F)C(F)(F)C(F)(F)C(F)(F)C(F)(F)C(F)(F)C(F)(F)C(F)(F)F		-10.67	0.66	4.04	P	OUT
QL04277	Bis(3,3,4,4,5,5,6,6,7,7,8,8,9,9,10,10,10-heptadecafluorodecyl) hydrogen phosphate, compound with 2,2'-iminodiethanol (11)	57677-97-1	OCCNCCO.OP(=O)(OCCC(F)(F)C(F)(F)C(F)(F)C(F)(F)C(F)(F)C(F)(F)C(F)(F)C(F)(F)F)OCCC(F)(F)C(F)(F)C(F)(F)C(F)(F)C(F)(F)C(F)(F)C(F)(F)C(F)(F)F		-10.13	0.65	3.86	P	OUT
QL04278	2-(Perfluorohexyl)ethanol dihydrogen phosphate bis(2-hydroxyethyl)amine	57678-02-1	OCCNCCO.OP(O)(=O)OCCC(F)(F)C(F)(F)C(F)(F)C(F)(F)C(F)(F)C(F)(F)F		-7.57	0.62	3.01	P	OUT
QL04279	Phosphoric acid, .gamma.-.omega.-perfluoro-C8-16-alkyl esters, compds. with diethanolamine	74499-44-8	OCCNP(=O)(OC(F)(F)C(F)(F)C(F)(F)C(F)(F)C(F)(F)C(F)(F)C(F)(F)C(F)(F)F)OC(F)(F)C(F)(F)C(F)(F)C(F)(F)C(F)(F)C(F)(F)C(F)(F)C(F)(F)F		-9.74	0.68	3.36	P	OUT

QL04280	2-Hydroxyethyl heptadecafluorononanoate	92746-93-5	OCCOC(=O)C(F)(F)C(F)(F)C(F)(F)C(F)(F)C(F)(F)C(F)(F)C(F)(F)C(F)(F)F		-8.12	0.66	2.74	P	IN
QL04281	2-[(2,2,3,3,4,4,5,5,6,6,7,7,7-Tridecafluoroheptyl)oxy]ethan-1-ol	54336-60-6	OCCOCC(F)(F)C(F)(F)C(F)(F)C(F)(F)C(F)(F)C(F)(F)F		-7.35	0.65	2.52	P	IN
QL04282	2-[(2,2,3,3,4,4,5,5,6,6,6-Undecafluorohexyl)oxy]ethan-1-ol	169289-67-2	OCCOCC(F)(F)C(F)(F)C(F)(F)C(F)(F)C(F)(F)F		-7.08	0.64	2.49	P	IN
QL04283	2-(2,2,3,3,4,4,4-Heptafluorobutoxy)ethan-1-ol	2266-43-5	OCCOCC(F)(F)C(F)(F)C(F)(F)F		-5.83	0.62	2.17	P	OUT
QL04284	2-[(3,3,4,4,5,5,6,6,7,7,8,8,8-Tridecafluorooctyl)oxy]ethan-1-ol	55427-50-4	OCCOCCC(F)(F)C(F)(F)C(F)(F)C(F)(F)C(F)(F)C(F)(F)F		-7.58	0.64	2.72	P	IN
QL04285	2-[(3,3,4,4,5,5,6,6,6-Nonafluorohexyl)oxy]ethan-1-ol	54949-99-4	OCCOCCC(F)(F)C(F)(F)C(F)(F)C(F)(F)F		-6.57	0.63	2.46	P	OUT
QL04286	N-ethyl perfluorooctanesulfonamido polyethylene glycol, n=8, m=1		OCCOCCN(CC)S(=O)(=O)C(F)(F)C(F)(F)C(F)(F)C(F)(F)C(F)(F)C(F)(F)C(F)(F)C(F)(F)F		-8.43	0.65	3.08	P	OUT
QL04287	N-ethyl perfluoroheptanesulfonamido polyethylene glycol, n=7, m=1		OCCOCCN(CC)S(=O)(=O)C(F)(F)C(F)(F)C(F)(F)C(F)(F)C(F)(F)C(F)(F)C(F)(F)F		-8.04	0.64	2.95	P	OUT
QL04288	N-ethyl perfluorohexanesulfonamido polyethylene glycol, n=6, m=1		OCCOCCN(CC)S(=O)(=O)C(F)(F)C(F)(F)C(F)(F)C(F)(F)C(F)(F)C(F)(F)F		-7.83	0.64	2.91	P	OUT
QL04289	N-ethyl perfluoropentanesulfonamido polyethylene glycol, n=5, m=1		OCCOCCN(CC)S(=O)(=O)C(F)(F)C(F)(F)C(F)(F)C(F)(F)C(F)(F)F		-7.35	0.63	2.76	P	OUT
QL04290	2,2,3,3,4,4,5,5,6,6,7,7,8,8,8-Pentadecafluoro-N-[2-(2-hydroxyethoxy)ethyl]octanamide	110388-02-8	OCCOCCNC(=O)C(F)(F)C(F)(F)C(F)(F)C(F)(F)C(F)(F)C(F)(F)C(F)(F)F		-8.03	0.64	2.97	P	OUT
QL04291	2-[2-(2,2,3,3,4,4,4-Heptafluorobutoxy)ethoxy]ethan-1-ol	54336-56-0	OCCOCCOCC(F)(F)C(F)(F)C(F)(F)F		-5.93	0.60	2.50	P	OUT
QL04292	N-ethyl perfluorooctanesulfonamido polyethylene glycol, n=8, m=2		OCCOCCOCCN(CC)S(=O)(=O)C(F)(F)C(F)(F)C(F)(F)C(F)(F)C(F)(F)C(F)(F)C(F)(F)C(F)(F)F		-8.53	0.64	3.29	P	OUT
QL04293	N-ethyl perfluoroheptanesulfonamido polyethylene glycol, n=7, m=2		OCCOCCOCCN(CC)S(=O)(=O)C(F)(F)C(F)(F)C(F)(F)C(F)(F)C(F)(F)C(F)(F)C(F)(F)F		-8.32	0.63	3.25	P	OUT
QL04294	N-ethyl perfluorohexanesulfonamido polyethylene glycol, n=6, m=2		OCCOCCOCCN(CC)S(=O)(=O)C(F)(F)C(F)(F)C(F)(F)C(F)(F)C(F)(F)C(F)(F)F		-7.92	0.63	3.14	P	OUT
QL04295	N-ethyl perfluoropentanesulfonamido polyethylene glycol, n=5, m=2		OCCOCCOCCN(CC)S(=O)(=O)C(F)(F)C(F)(F)C(F)(F)C(F)(F)C(F)(F)F		-7.60	0.62	3.07	P	OUT
QL04296	N-ethyl perfluorooctanesulfonamido polyethylene glycol, n=8, m=3		OCCOCCOCCOCCN(CC)S(=O)(=O)C(F)(F)C(F)(F)C(F)(F)C(F)(F)C(F)(F)C(F)(F)C(F)(F)C(F)(F)F		-8.70	0.63	3.51	P	OUT
QL04297	N-ethyl perfluoroheptanesulfonamido polyethylene glycol, n=7, m=3		OCCOCCOCCOCCN(CC)S(=O)(=O)C(F)(F)C(F)(F)C(F)(F)C(F)(F)C(F)(F)C(F)(F)C(F)(F)F		-8.36	0.62	3.41	P	OUT
QL04298	N-ethyl perfluorohexanesulfonamido polyethylene glycol, n=6, m=3		OCCOCCOCCOCCN(CC)S(=O)(=O)C(F)(F)C(F)(F)C(F)(F)C(F)(F)C(F)(F)C(F)(F)F		-8.08	0.62	3.36	P	OUT
QL04299	N-ethyl perfluoropentanesulfonamido polyethylene glycol, n=5, m=3		OCCOCCOCCOCCN(CC)S(=O)(=O)C(F)(F)C(F)(F)C(F)(F)C(F)(F)C(F)(F)F		-7.67	0.61	3.25	P	OUT
QL04300	N-ethyl perfluorooctanesulfonamido polyethylene glycol, n=8, m=4		OCCOCCOCCOCCOCCN(CC)S(=O)(=O)C(F)(F)C(F)(F)C(F)(F)C(F)(F)C(F)(F)C(F)(F)C(F)(F)C(F)(F)F		-8.73	0.62	3.65	P	OUT
QL04301	N-ethyl perfluoroheptanesulfonamido polyethylene glycol, n=7, m=4		OCCOCCOCCOCCOCCN(CC)S(=O)(=O)C(F)(F)C(F)(F)C(F)(F)C(F)(F)C(F)(F)C(F)(F)C(F)(F)F		-8.50	0.61	3.60	P	OUT
QL04302	N-ethyl perfluorohexanesulfonamido polyethylene glycol, n=6, m=4		OCCOCCOCCOCCOCCN(CC)S(=O)(=O)C(F)(F)C(F)(F)C(F)(F)C(F)(F)C(F)(F)C(F)(F)F		-8.14	0.61	3.51	P	OUT
QL04303	N-ethyl perfluoropentanesulfonamido polyethylene glycol, n=5, m=4		OCCOCCOCCOCCOCCN(CC)S(=O)(=O)C(F)(F)C(F)(F)C(F)(F)C(F)(F)C(F)(F)F		-7.85	0.60	3.47	P	OUT
QL04304	16,16,17,17,18,18,19,19,20,20,21,21,22,22,22-Pentadecafluoro-3,6,9,12,15-pentaoxadocosan-1-ol	93704-25-7	OCCOCCOCCOCCOCCOC(F)(F)C(F)(F)C(F)(F)C(F)(F)C(F)(F)C(F)(F)C(F)(F)F		-8.21	0.61	3.51	P	OUT
QL04305	N-ethyl perfluorooctanesulfonamido polyethylene glycol, n=8, m=5		OCCOCCOCCOCCOCCOCCN(CC)S(=O)(=O)C(F)(F)C(F)(F)C(F)(F)C(F)(F)C(F)(F)C(F)(F)C(F)(F)C(F)(F)F		-8.85	0.61	3.81	P	OUT
QL04306	N-ethyl perfluoroheptanesulfonamido polyethylene glycol, n=7, m=5		OCCOCCOCCOCCOCCOCCN(CC)S(=O)(=O)C(F)(F)C(F)(F)C(F)(F)C(F)(F)C(F)(F)C(F)(F)C(F)(F)F		-8.54	0.61	3.73	P	OUT
QL04307	N-ethyl perfluorohexanesulfonamido polyethylene glycol, n=6, m=5		OCCOCCOCCOCCOCCOCCN(CC)S(=O)(=O)C(F)(F)C(F)(F)C(F)(F)C(F)(F)C(F)(F)C(F)(F)F		-8.29	0.60	3.70	P	OUT
QL04308	N-ethyl perfluoropentanesulfonamido polyethylene glycol, n=5, m=5		OCCOCCOCCOCCOCCOCCN(CC)S(=O)(=O)C(F)(F)C(F)(F)C(F)(F)C(F)(F)C(F)(F)F		-7.92	0.59	3.61	P	OUT
QL04309	N-ethyl perfluorooctanesulfonamido polyethylene glycol, n=8, m=6		OCCOCCOCCOCCOCCOCCOCCN(CC)S(=O)(=O)C(F)(F)C(F)(F)C(F)(F)C(F)(F)C(F)(F)C(F)(F)C(F)(F)C(F)(F)F		-8.90	0.61	3.92	P	OUT
QL04310	N-ethyl perfluoroheptanesulfonamido polyethylene glycol, n=7, m=6		OCCOCCOCCOCCOCCOCCOCCN(CC)S(=O)(=O)C(F)(F)C(F)(F)C(F)(F)C(F)(F)C(F)(F)C(F)(F)C(F)(F)F		-8.68	0.60	3.89	P	OUT
QL04311	N-ethyl perfluorohexanesulfonamido polyethylene glycol, n=6, m=6		OCCOCCOCCOCCOCCOCCOCCN(CC)S(=O)(=O)C(F)(F)C(F)(F)C(F)(F)C(F)(F)C(F)(F)C(F)(F)F		-8.35	0.59	3.82	P	OUT
QL04312	N-ethyl perfluoropentanesulfonamido polyethylene glycol, n=5, m=6		OCCOCCOCCOCCOCCOCCOCCN(CC)S(=O)(=O)C(F)(F)C(F)(F)C(F)(F)C(F)(F)C(F)(F)F		-8.09	0.59	3.79	P	OUT

QL04313	N-ethyl perfluorooctanesulfonamido polyethylene glycol, n=8, m=7		OCCOCCOCCOCCOCCOCCOCCN(CC)S(=O)(=O)C(F)(F)C(F)(F)C(F)(F)C(F)(F)C(F)(F)C(F)(F)C(F)(F)C(F)(F)F		-9.02	0.60	4.07	P	OUT
QL04314	N-ethyl perfluoroheptanesulfonamido polyethylene glycol, n=7, m=7		OCCOCCOCCOCCOCCOCCOCCN(CC)S(=O)(=O)C(F)(F)C(F)(F)C(F)(F)C(F)(F)C(F)(F)C(F)(F)C(F)(F)F		-8.73	0.59	4.00	P	OUT
QL04315	N-ethyl perfluorohexanesulfonamido polyethylene glycol, n=6, m=7		OCCOCCOCCOCCOCCOCCOCCN(CC)S(=O)(=O)C(F)(F)C(F)(F)C(F)(F)C(F)(F)C(F)(F)C(F)(F)F		-8.50	0.59	3.97	P	OUT
QL04316	N-ethyl perfluoropentanesulfonamido polyethylene glycol, n=5, m=7		OCCOCCOCCOCCOCCOCCOCCN(CC)S(=O)(=O)C(F)(F)C(F)(F)C(F)(F)C(F)(F)C(F)(F)F		-8.17	0.58	3.91	P	OUT
QL04317	N-ethyl perfluorooctanesulfonamido polyethylene glycol, n=8, m=8		OCCOCCOCCOCCOCCOCCOCCOCCN(CC)S(=O)(=O)C(F)(F)C(F)(F)C(F)(F)C(F)(F)C(F)(F)C(F)(F)C(F)(F)C(F)(F)F		-9.06	0.59	4.16	P	OUT
QL04318	N-ethyl perfluoroheptanesulfonamido polyethylene glycol, n=7, m=8		OCCOCCOCCOCCOCCOCCOCCOCCN(CC)S(=O)(=O)C(F)(F)C(F)(F)C(F)(F)C(F)(F)C(F)(F)C(F)(F)C(F)(F)F		-8.86	0.59	4.14	P	OUT
QL04319	N-ethyl perfluorohexanesulfonamido polyethylene glycol, n=6, m=8		OCCOCCOCCOCCOCCOCCOCCOCCN(CC)S(=O)(=O)C(F)(F)C(F)(F)C(F)(F)C(F)(F)C(F)(F)C(F)(F)F		-8.56	0.58	4.08	P	OUT
QL04320	N-ethyl perfluoropentanesulfonamido polyethylene glycol, n=5, m=8		OCCOCCOCCOCCOCCOCCOCCOCCN(CC)S(=O)(=O)C(F)(F)C(F)(F)C(F)(F)C(F)(F)C(F)(F)F		-8.32	0.58	4.06	P	OUT
QL04321	N-ethyl perfluorooctanesulfonamido polyethylene glycol, n=8, m=9		OCCOCCOCCOCCOCCOCCOCCOCCOCCN(CC)S(=O)(=O)C(F)(F)C(F)(F)C(F)(F)C(F)(F)C(F)(F)C(F)(F)C(F)(F)C(F)(F)F		-9.17	0.59	4.29	P	OUT
QL04322	N-ethyl perfluorooctanesulfonamido polyethylene glycol, n=8, m=10		OCCOCCOCCOCCOCCOCCOCCOCCOCCOCCN(CC)S(=O)(=O)C(F)(F)C(F)(F)C(F)(F)C(F)(F)C(F)(F)C(F)(F)C(F)(F)C(F)(F)F		-9.22	0.59	4.37	P	OUT
QL04323	N-ethyl perfluorooctanesulfonamido polyethylene glycol, n=8, m=11		OCCOCCOCCOCCOCCOCCOCCOCCOCCOCCOCCN(CC)S(=O)(=O)C(F)(F)C(F)(F)C(F)(F)C(F)(F)C(F)(F)C(F)(F)C(F)(F)C(F)(F)F		-9.33	0.58	4.48	P	OUT
QL04324	N-ethyl perfluorooctanesulfonamido polyethylene glycol, n=8, m=12		OCCOCCOCCOCCOCCOCCOCCOCCOCCOCCOCCOCCN(CC)S(=O)(=O)C(F)(F)C(F)(F)C(F)(F)C(F)(F)C(F)(F)C(F)(F)C(F)(F)C(F)(F)F		-9.38	0.58	4.55	P	OUT
QL04325	N-ethyl perfluorooctanesulfonamido polyethylene glycol, n=8, m=13		OCCOCCOCCOCCOCCOCCOCCOCCOCCOCCOCCOCCOCCN(CC)S(=O)(=O)C(F)(F)C(F)(F)C(F)(F)C(F)(F)C(F)(F)C(F)(F)C(F)(F)C(F)(F)F		-9.48	0.58	4.65	P	OUT
QL04326	N-ethyl perfluorooctanesulfonamido polyethylene glycol, n=8, m=14		OCCOCCOCCOCCOCCOCCOCCOCCOCCOCCOCCOCCOCCOCCN(CC)S(=O)(=O)C(F)(F)C(F)(F)C(F)(F)C(F)(F)C(F)(F)C(F)(F)C(F)(F)C(F)(F)F		-9.53	0.57	4.72	P	OUT
QL04327	2-(3,3,4,4,5,5,6,6,6-Nonafluorohexane-1-sulfinyl)ethan-1-ol	917966-97-3	OCCS(=O)CCC(F)(F)C(F)(F)C(F)(F)C(F)(F)F		-6.73	0.63	2.44	P	OUT
QL04328	2-[(3,3,4,4,5,5,6,6,6-Nonafluorohexyl)sulfanyl]ethan-1-ol	104568-28-7	OCCSCCC(F)(F)C(F)(F)C(F)(F)C(F)(F)F		-6.57	0.63	2.46	P	OUT
QL04329	4-Hydroxy-3-(hydroxyimino)-4-(nonafluorobutyl)-1,3,4,5-tetrahydro-2H-1,5-benzodiazepin-2-one	216307-12-9	ON=C1C(=O)NC2=CC=CC=C2NC1(O)C(F)(F)C(F)(F)C(F)(F)C(F)(F)F		-6.85	0.64	2.41	P	IN
QL04330	2,2,3,3,4,4,5,5,6,6,7,7,8,8,8-Pentadecafluoro-N-hydroxyoctanimidamide	4314-38-9	ONC(=N)C(F)(F)C(F)(F)C(F)(F)C(F)(F)C(F)(F)C(F)(F)C(F)(F)F		-7.83	0.67	2.45	P	IN
QL04331	2,2,3,3,4,4,4-Heptafluoro-N-hydroxybutanimidamide	4314-37-8	ONC(=N)C(F)(F)C(F)(F)C(F)(F)F		-5.98	0.65	1.84	P	IN
QL04332	2,2,3,3,4,4,5,5,6,6,7,7,8,8,8-Pentadecafluoro-N-hydroxyoctanamide	15435-88-8	ONC(=O)C(F)(F)C(F)(F)C(F)(F)C(F)(F)C(F)(F)C(F)(F)C(F)(F)F		-7.83	0.67	2.45	P	IN
QL04333	2,2,3,3,4,4,4-Heptafluoro-N-hydroxybutanamide	87050-96-2	ONC(=O)C(F)(F)C(F)(F)C(F)(F)F		-5.98	0.65	1.84	P	IN
QL04334	Perfluoro(pentadecaoxypropylene)oxyethylene	122998-72-5	OOOOOOOOOOOOOOOC(F)(F)F		-12.94	0.68	4.95	P	OUT
QL04335	Nonafluorobutane-1-sulfonoperoxoic acid	85963-79-7	OOS(=O)(=O)C(F)(F)C(F)(F)C(F)(F)C(F)(F)F		-6.72	0.67	1.89	P	IN
QL04336	2,2,3,3,4,4,4-Heptafluorobutyl hydrogen phosphate	425-67-2	OP([O-])(=O)OCC(F)(F)C(F)(F)C(F)(F)F		-6.45	0.66	1.91	P	IN
QL04337	Bis(pentacosafluorododecyl)phosphinic acid	63225-54-7	OP(=O)(C(F)(F)C(F)(F)C(F)(F)C(F)(F)C(F)(F)C(F)(F)C(F)(F)C(F)(F)C(F)(F)C(F)(F)C(F)(F)C(F)(F)F)C(F)(F)C(F)(F)C(F)(F)C(F)(F)C(F)(F)C(F)(F)C(F)(F)C(F)(F)C(F)(F)C(F)(F)C(F)(F)C(F)(F)F		-11.06	0.70	3.75	P	OUT
QL04338	2,2,3,3,4,4,4-Heptafluoro-1-phenylbutyl hydrogen phenylphosphonate	496032-46-3	OP(=O)(OC(c1ccccc1)C(F)(F)C(F)(F)C(F)(F)F)c1ccccc1		-6.24	0.62	2.37	P	OUT
QL04339	(3,3,4,4,5,5,6,6,7,7,8,8,9,9,10,10,11,11,12,12,12-Henicosafluorododecyl)phosphinic acid	731858-15-4	OP(=O)CCC(F)(F)C(F)(F)C(F)(F)C(F)(F)C(F)(F)C(F)(F)C(F)(F)C(F)(F)C(F)(F)C(F)(F)F		-8.80	0.68	2.89	P	OUT
QL04340	(3,3,4,4,5,5,6,6,7,7,8,8,9,9,10,10,10-Heptadecafluorodecyl)phosphinic acid	731858-13-2	OP(=O)CCC(F)(F)C(F)(F)C(F)(F)C(F)(F)C(F)(F)C(F)(F)C(F)(F)C(F)(F)F		-8.27	0.67	2.69	P	IN
QL04341	[[4-[(Heptadecafluorononenyl)oxy]phenyl]methyl]phosphonic acid	63513-12-2	OP(O)(=O)CC1=CC=C(OC(F)=C(F)C(F)(F)C(F)(F)C(F)(F)C(F)(F)C(F)(F)C(F)(F)C(F)(F)F)C=C1		-8.72	0.66	3.12	P	IN

QL04342	(3,3,4,4,5,5,6,6,7,7,8,8,9,9,10,10,11,11,12,12,13,13,14,14,14-Pentacosafluorotetradecyl)phosphonic acid	147290-12-8	OP(O)(=O)CCC(F)(F)C(F)(F)C(F)(F)C(F)(F)C(F)(F)C(F)(F)C(F)(F)C(F)(F)C(F)(F)C(F)(F)C(F)(F)C(F)(F)F		-9.41	0.69	3.06	P	OUT
QL04343	(3,3,4,4,5,5,6,6,7,7,8,8,9,9,10,10,11,11,12,12,12-Henicosafluorododecyl)phosphonic acid	252237-39-1	OP(O)(=O)CCC(F)(F)C(F)(F)C(F)(F)C(F)(F)C(F)(F)C(F)(F)C(F)(F)C(F)(F)C(F)(F)C(F)(F)F		-8.99	0.68	2.88	P	OUT
QL04344	((Perfluorooctyl)ethyl)phosphonic acid	80220-63-9	OP(O)(=O)CCC(F)(F)C(F)(F)C(F)(F)C(F)(F)C(F)(F)C(F)(F)C(F)(F)C(F)(F)F		-8.49	0.68	2.68	P	IN
QL04345	2-(Perfluorohexyl)ethylphosphonic acid	252237-40-4	OP(O)(=O)CCC(F)(F)C(F)(F)C(F)(F)C(F)(F)C(F)(F)C(F)(F)F		-7.90	0.67	2.44	P	IN
QL04346	(3,3,4,4,5,5,6,6,6-Nonafluorohexyl)phosphonic acid	503564-50-9	OP(O)(=O)CCC(F)(F)C(F)(F)C(F)(F)C(F)(F)F		-7.14	0.67	2.17	P	IN
QL04347	1,3-Propanediol, 2,2-bis[[(gamma-omega-perfluoro-C10-20-alkyl)thio]methyl] derivs., phosphates, ammonium salts	148240-89-5	OP(O)(=O)OCC(COP(O)(O)=O)(CSCCC(F)(F)C(F)(F)C(F)(F)C(F)(F)C(F)(F)C(F)(F)F)CSCCC(F)(F)C(F)(F)C(F)(F)C(F)(F)C(F)(F)C(F)(F)F		-9.46	0.66	3.46	P	OUT
QL04348	1,3-Propanediol, 2,2-bis[[(gamma-omega-perfluoro-C6-12-alkyl)thio]methyl] derivs., phosphates, ammonium salts	148240-87-3	OP(O)(=O)OCC(COP(O)(O)=O)(CSCCC(F)(F)C(F)(F)C(F)(F)C(F)(F)F)CSCCC(F)(F)C(F)(F)C(F)(F)C(F)(F)F		-8.61	0.65	3.17	P	OUT
QL04349	72 Fluorotelomer dihydrogen phosphate	37013-72-2	OP(O)(=O)OCCC(F)(F)C(F)(F)C(F)(F)C(F)(F)C(F)(F)C(F)(F)C(F)(F)F		-8.28	0.67	2.68	P	IN
QL04350	Heptadecafluorooctane-2-sulfonic acid	927670-12-0	OS(=O)(=O)C(F)(C(F)(F)F)C(F)(F)C(F)(F)C(F)(F)C(F)(F)C(F)(F)C(F)(F)F		-7.82	0.69	2.20	P	IN
QL04351	Perfluoro-2-(trifluoromethyl)propanesulphonic acid	93762-09-5	OS(=O)(=O)C(F)(F)C(F)(C(F)(F)F)C(F)(F)F		-6.00	0.68	1.38	P	IN
QL04354	Perfluoro(2-ethoxyethane)sulfonic acid	113507-82-7	OS(=O)(=O)C(F)(F)C(F)(F)OC(F)(F)C(F)(F)F		-6.92	0.68	1.89	P	IN
QL04355	1,1,2,2-Tetrafluoroethanesulfonic acid	464-14-2	OS(=O)(=O)C(F)(F)C(F)F		-5.29	0.66	1.36	P	IN
QL04356	4-[(Nonadecafluorodecenyl)oxy]benzenesulfonic acid, sodium salt	64910-98-1	OS(=O)(=O)C1=CC=C(OC(F)(F)C(F)(F)C(F)(F)C(F)(F)C(F)(F)C(F)(F)C(F)(F)C(F)(F)C(F)=C(F)F)C=C1		-8.87	0.66	3.11	P	OUT
QL04357	Potassium 3,3,4,4,5,5,6,6,7,7,8,8,9,9,10,10,11,11,12,12,12-henicosafluorododecyl sulfate|1H,1H,2H,2H-Perfluorododecanol sulfate ammonium salt	1262446-14-9|63225-58-1	OS(=O)(=O)OCCC(F)(F)C(F)(F)C(F)(F)C(F)(F)C(F)(F)C(F)(F)C(F)(F)C(F)(F)C(F)(F)C(F)(F)F		-8.95	0.68	2.93	P	OUT
QL04358	Potassium 1,1,2,2-tetrafluoro-2-{[1,1,1,2,3,3-hexafluoro-3-(pentafluoroethoxy)propan-2-yl]oxy}ethane-1-sulfonate|Ammonium 1,1,2,2-tetrafluoro-2-{[1,1,1,2,3,3-hexafluoro-3-(pentafluoroethoxy)propan-2-yl]oxy}ethane-1-sulfonate	70755-50-9|93644-65-6	OS(=O)(C(F)(C(F)(OC(C(F)(F)F)(C(F)(OC(F)(C(F)(F)F)F)F)F)F)F)=O		-8.00	0.68	2.43	P	IN
QL04359	Potassium perfluoro-p-ethylcyclohexanesulfonate	335-24-0	OS(=O)(C1(C(F)(C(F)(C(C(F)(C(F)(F)F)F)(C(F)(C1(F)F)F)F)F)F)F)=O		-8.00	0.69	2.22	P	IN
QL04360	4-(Perfluorononyl)oxybenzenesulfonate sodium	91998-13-9	OS(=O)(C1=CC=C(C=C1)OC(F)(C(F)(C(F)(C(F)(C(F)(C(F)(C(F)(C(F)(C(F)(F)F)F)F)F)F)F)F)F)F)=O		-9.06	0.67	3.07	P	OUT
QL04361	Sodium 5,5,6,6,7,7,8,8,8-nonafluorooctane-1-sulfonate|Potassium 5,5,6,6,7,7,8,8,8-nonafluorooctane-1-sulfonate|Lithium 5,5,6,6,7,7,8,8,8-nonafluorooctane-1-sulfonate	65345-91-7|1604816-60-5|1604816-75-2	OS(=O)(CCCCC(F)(C(F)(C(F)(C(F)(F)F)F)F)F)=O		-7.18	0.65	2.42	P	IN
QL04362	Potassium 3,3,4,4,5,5,6,6,7,7,8,8,9,9,10,10,10-heptadecafluorodecyl sulfate|1H,1H,2H,2H-Perfluorodecanol sulfate ammonium salt	1262446-13-8|63225-57-0	OS(=O)(OCCC(F)(C(F)(C(F)(C(F)(C(F)(C(F)(C(F)(C(F)(F)F)F)F)F)F)F)F)F)=O		-8.45	0.67	2.73	P	IN
QL04363	Tris(2,2,3,3,4,4,5,5-octafluoropentyl) phosphate	355-86-2	P(=O)(OCC(C(C(C(F)F)(F)F)(F)F)(F)F)(OCC(C(C(C(F)F)(F)F)(F)F)(F)F)OCC(C(C(C(F)F)(F)F)(F)F)(F)F		-8.19	0.66	2.76	P	IN
QL04364	Cl-substituted perfluoroalkyl (linear) sulfonic acids_i, n=6		S(=O)(O)(=O)C(F)(Cl)C(F)(F)C(F)(F)C(F)(F)C(F)(F)C(F)(F)C(F)(F)F		-7.94	0.69	2.24	P	IN
QL04365	Cl-substituted perfluoroalkyl (linear) sulfonic acids_i, n=4		S(=O)(O)(=O)C(F)(Cl)C(F)(F)C(F)(F)C(F)(F)C(F)(F)F		-7.19	0.69	1.90	P	IN
QL04366	Cl-substituted perfluoroalkyl (linear) sulfonic acids_i, n=3		S(=O)(O)(=O)C(F)(Cl)C(F)(F)C(F)(F)C(F)(F)F		-6.93	0.69	1.79	P	IN
QL04367	Cl-substituted perfluoroalkyl (linear) sulfonic acids_i, n=2		S(=O)(O)(=O)C(F)(Cl)C(F)(F)C(F)(F)F		-6.18	0.68	1.45	P	IN
QL04368	ether-substituted Perfluoroalkyl (linear) sulfonic acids, n=9		S(=O)(O)(=O)C(F)(F)C(F)(F)C(F)(F)C(F)(F)C(F)(F)C(F)(F)C(F)(F)C(F)(F)C(F)(F)OC(F)(F)F		-8.78	0.69	2.74	P	IN
QL04369	Cl-substituted perfluoroalkyl (linear) sulfonic acids, n=8		S(=O)(O)(=O)C(F)(F)C(F)(F)C(F)(F)C(F)(F)C(F)(F)C(F)(F)C(F)(F)C(F)(F)Cl		-8.37	0.69	2.44	P	IN
QL04370	ether-substituted Perfluoroalkyl (linear) sulfonic acids_i, n=8		S(=O)(O)(=O)C(F)(F)C(F)(F)C(F)(F)C(F)(F)C(F)(F)C(F)(F)C(F)(F)C(F)(F)OC(F)(F)C(C(F)(F)F)(F)C(F)(F)F		-9.10	0.69	2.85	P	OUT
QL04371	ether-substituted Perfluoroalkyl (linear) sulfonic acids, n=8		S(=O)(O)(=O)C(F)(F)C(F)(F)C(F)(F)C(F)(F)C(F)(F)C(F)(F)C(F)(F)C(F)(F)OC(F)(F)F		-8.63	0.68	2.68	P	IN
QL04372	Cl-substituted perfluoroalkyl (linear) sulfonic acids, n=7		S(=O)(O)(=O)C(F)(F)C(F)(F)C(F)(F)C(F)(F)C(F)(F)C(F)(F)C(F)(F)Cl		-7.94	0.69	2.24	P	IN

QL04373	ether-substituted Perfluoroalkyl (linear) sulfonic acids_i, n=7		S(=O)(O)(=O)C(F)(F)C(F)(F)C(F)(F)C(F)(F)C(F)(F)C(F)(F)C(F)(F)OC(F)(F)C(C(F)(F)F)(F)C(F)(F)F		-8.78	0.69	2.71	P	IN
QL04374	ether-substituted Perfluoroalkyl (linear) sulfonic acids_ii, n=7		S(=O)(O)(=O)C(F)(F)C(F)(F)C(F)(F)C(F)(F)C(F)(F)C(F)(F)C(F)(F)OC(F)(F)C(F)(F)F		-8.73	0.69	2.68	P	IN
QL04375	ether-substituted Perfluoroalkyl (linear) sulfonic acids, n=7		S(=O)(O)(=O)C(F)(F)C(F)(F)C(F)(F)C(F)(F)C(F)(F)C(F)(F)C(F)(F)OC(F)(F)F		-8.24	0.68	2.51	P	IN
QL04376	ether-substituted Perfluoroalkyl (linear) sulfonic acids_i, n=6		S(=O)(O)(=O)C(F)(F)C(F)(F)C(F)(F)C(F)(F)C(F)(F)C(F)(F)OC(F)(F)C(C(F)(F)F)(F)C(F)(F)F		-8.62	0.69	2.64	P	IN
QL04377	ether-substituted Perfluoroalkyl (linear) sulfonic acids_ii, n=6		S(=O)(O)(=O)C(F)(F)C(F)(F)C(F)(F)C(F)(F)C(F)(F)C(F)(F)OC(F)(F)C(F)(F)F		-8.36	0.69	2.51	P	IN
QL04378	ether-substituted Perfluoroalkyl (linear) sulfonic acids, n=6		S(=O)(O)(=O)C(F)(F)C(F)(F)C(F)(F)C(F)(F)C(F)(F)C(F)(F)OC(F)(F)F		-8.06	0.68	2.44	P	IN
QL04379	ether-substituted Perfluoroalkyl (linear) sulfonic acids_i, n=5		S(=O)(O)(=O)C(F)(F)C(F)(F)C(F)(F)C(F)(F)C(F)(F)OC(F)(F)C(C(F)(F)F)(F)C(F)(F)F		-8.25	0.69	2.47	P	IN
QL04380	ether-substituted Perfluoroalkyl (linear) sulfonic acids_ii, n=5		S(=O)(O)(=O)C(F)(F)C(F)(F)C(F)(F)C(F)(F)C(F)(F)OC(F)(F)C(F)(F)F		-8.19	0.69	2.44	P	IN
QL04381	ether-substituted Perfluoroalkyl (linear) sulfonic acids_i, n=4		S(=O)(O)(=O)C(F)(F)C(F)(F)C(F)(F)C(F)(F)OC(F)(F)C(C(F)(F)F)(F)C(F)(F)F		-8.06	0.68	2.39	P	IN
QL04382	ether-substituted Perfluoroalkyl (linear) sulfonic acids_ii, n=4		S(=O)(O)(=O)C(F)(F)C(F)(F)C(F)(F)C(F)(F)OC(F)(F)C(F)(F)F		-7.73	0.68	2.23	P	IN
QL04383	ether-substituted Perfluoroalkyl (linear) sulfonic acids, n=4		S(=O)(O)(=O)C(F)(F)C(F)(F)C(F)(F)C(F)(F)OC(F)(F)F		-7.35	0.68	2.15	P	IN
QL04384	ether-substituted Perfluoroalkyl (linear) sulfonic acids_i, n=3		S(=O)(O)(=O)C(F)(F)C(F)(F)C(F)(F)OC(F)(F)C(C(F)(F)F)(F)C(F)(F)F		-7.60	0.68	2.18	P	IN
QL04385	ether-substituted Perfluoroalkyl (linear) sulfonic acids, n=3		S(=O)(O)(=O)C(F)(F)C(F)(F)C(F)(F)OC(F)(F)F		-6.72	0.67	1.89	P	IN
QL04386	ether-substituted Perfluoroalkyl (linear) sulfonic acids_i, n=2		S(=O)(O)(=O)C(F)(F)C(F)(F)OC(F)(F)C(C(F)(F)F)(F)C(F)(F)F		-7.23	0.68	2.02	P	IN
QL04387	Cl-substituted,ether-substituted perfluoroalkyl (linear)sulfonic acids, n=12		S(=O)(O)(=O)C(F)(F)C(F)(F)OC(F)(F)C(F)(F)C(F)(F)C(F)(F)C(F)(F)C(F)(F)C(F)(F)C(F)(F)C(F)(F)C(F)(F)C(F)(F)C(F)(F)Cl		-9.69	0.69	3.11	P	OUT
QL04388	Cl-substituted,ether-substituted perfluoroalkyl (linear)sulfonic acids, n=10		S(=O)(O)(=O)C(F)(F)C(F)(F)OC(F)(F)C(F)(F)C(F)(F)C(F)(F)C(F)(F)C(F)(F)C(F)(F)C(F)(F)C(F)(F)C(F)(F)Cl		-9.31	0.69	2.94	P	OUT
QL04389	Cl-substituted,ether-substituted perfluoroalkyl (linear)sulfonic acids, n=7		S(=O)(O)(=O)C(F)(F)C(F)(F)OC(F)(F)C(F)(F)C(F)(F)C(F)(F)C(F)(F)C(F)(F)C(F)(F)Cl		-8.73	0.69	2.68	P	IN
QL04390	Cl-substituted,ether-substituted perfluoroalkyl (linear)sulfonic acids, n=5		S(=O)(O)(=O)C(F)(F)C(F)(F)OC(F)(F)C(F)(F)C(F)(F)C(F)(F)C(F)(F)Cl		-8.19	0.69	2.44	P	IN
QL04391	Cl-substituted,ether-substituted perfluoroalkyl (linear)sulfonic acids, n=4		S(=O)(O)(=O)C(F)(F)C(F)(F)OC(F)(F)C(F)(F)C(F)(F)C(F)(F)Cl		-7.73	0.68	2.23	P	IN
QL04392	ether-substituted Perfluoroalkyl (linear) sulfonic acids, n=2		S(=O)(O)(=O)C(F)(F)C(F)(F)OC(F)(F)F		-6.40	0.67	1.81	P	IN
QL04393	ether-substituted Perfluoroalkyl (linear) sulfonic acids_i, n=1		S(=O)(O)(=O)C(F)(F)OC(F)(F)C(C(F)(F)F)(F)C(F)(F)F		-6.76	0.68	1.82	P	IN
QL04394	N-methyl perfluorohexane sulfonamido acetic acid, n=6		S(N(C)CC(O)=O)(=O)(=O)C(F)(F)C(F)(F)C(F)(F)C(F)(F)C(F)(F)C(F)(F)F		-7.87	0.67	2.51	P	IN
QL04395	N-methyl perfluoropentane sulfonamido acetic acid, n=5		S(N(C)CC(O)=O)(=O)(=O)C(F)(F)C(F)(F)C(F)(F)C(F)(F)C(F)(F)F		-7.66	0.66	2.45	P	IN
QL04396	N-methyl perfluorobutane sulfonamido acetic acid, n=4	159381-10-9	S(N(C)CC(O)=O)(=O)(=O)C(F)(F)C(F)(F)C(F)(F)C(F)(F)F		-7.11	0.66	2.24	P	IN
QL04397	N-methyl perfluoropropane sulfonamido acetic acid, n=3		S(N(C)CC(O)=O)(=O)(=O)C(F)(F)C(F)(F)C(F)(F)F		-6.82	0.65	2.18	P	IN
QL04398	N-methyl perfluoroalkanesulfonamido quaternary ammonium, n=8		S(N(C)CCC[N+](C)(C)C)(=O)(=O)C(F)(F)C(F)(F)C(F)(F)C(F)(F)C(F)(F)C(F)(F)C(F)(F)C(F)(F)F		-8.81	0.67	2.94	P	OUT
QL04399	N-methyl perfluoroalkanesulfonamido quaternary ammonium, n=7		S(N(C)CCC[N+](C)(C)C)(=O)(=O)C(F)(F)C(F)(F)C(F)(F)C(F)(F)C(F)(F)C(F)(F)C(F)(F)F		-8.66	0.67	2.89	P	IN
QL04400	N-methyl perfluoroalkanesulfonamido quaternary ammonium, n=6		S(N(C)CCC[N+](C)(C)C)(=O)(=O)C(F)(F)C(F)(F)C(F)(F)C(F)(F)C(F)(F)C(F)(F)F		-8.28	0.67	2.74	P	IN
QL04401	N-methyl perfluoroalkanesulfonamido quaternary ammonium, n=5		S(N(C)CCC[N+](C)(C)C)(=O)(=O)C(F)(F)C(F)(F)C(F)(F)C(F)(F)C(F)(F)F		-8.10	0.66	2.70	P	IN
QL04402	N-methyl perfluoroalkanesulfonamido quaternary ammonium, n=4		S(N(C)CCC[N+](C)(C)C)(=O)(=O)C(F)(F)C(F)(F)C(F)(F)C(F)(F)F		-7.63	0.66	2.52	P	IN
QL04403	N-methyl perfluoroalkanesulfonamido quaternary ammonium, n=3		S(N(C)CCC[N+](C)(C)C)(=O)(=O)C(F)(F)C(F)(F)C(F)(F)F		-7.34	0.65	2.45	P	IN
QL04404	N-methyl perfluoroalkanesulfonamido quaternary ammonium, n=2		S(N(C)CCC[N+](C)(C)C)(=O)(=O)C(F)(F)C(F)(F)F		-6.72	0.65	2.23	P	IN
QL04405	perfluoroalkanesulfonamido sulfonate betaine D, n=6		S(N(C)CCC[N+](CCCS(=O)(O)=O)(C)C)(=O)(=O)C(F)(F)C(F)(F)C(F)(F)C(F)(F)C(F)(F)C(F)(F)F		-8.75	0.66	3.11	P	IN

QL04406	perfluoroalkanesulfonamido sulfonate betaine D, n=5	S(N(C)CCC[N+](CCCS(=O)(O)=O)(C)C)(=O)(=O)C(F)(F)C(F)(F)C(F)(F)C(F)(F)C(F)(F)F		-8.51	0.65	3.04	P	IN
QL04407	perfluoroalkanesulfonamido sulfonate betaine D, n=4	S(N(C)CCC[N+](CCCS(=O)(O)=O)(C)C)(=O)(=O)C(F)(F)C(F)(F)C(F)(F)C(F)(F)F		-8.12	0.65	2.90	P	IN
QL04408	perfluoroalkanesulfonamido sulfonate betaine D, n=3	S(N(C)CCC[N+](CCCS(=O)(O)=O)(C)C)(=O)(=O)C(F)(F)C(F)(F)C(F)(F)F		-7.85	0.64	2.84	P	IN
QL04409	perfluoroalkanesulfonamido quaternary ammonium dicarboxylate A, n=6	S(N(CC(O)=O)CCC[N+](CC(O)=O)(C)C)(=O)(=O)C(F)(F)C(F)(F)C(F)(F)C(F)(F)C(F)(F)C(F)(F)F		-8.17	0.65	2.94	P	IN
QL04410	perfluoroalkanesulfonamido quaternary ammonium dicarboxylate A, n=5	S(N(CC(O)=O)CCC[N+](CC(O)=O)(C)C)(=O)(=O)C(F)(F)C(F)(F)C(F)(F)C(F)(F)C(F)(F)F		-7.92	0.64	2.88	P	IN
QL04411	perfluoroalkanesulfonamido quaternary ammonium dicarboxylate A, n=4	S(N(CC(O)=O)CCC[N+](CC(O)=O)(C)C)(=O)(=O)C(F)(F)C(F)(F)C(F)(F)C(F)(F)F		-7.48	0.64	2.73	P	OUT
QL04412	perfluoroalkanesulfonamido quaternary ammonium dicarboxylate A, n=3	S(N(CC(O)=O)CCC[N+](CC(O)=O)(C)C)(=O)(=O)C(F)(F)C(F)(F)C(F)(F)F		-7.03	0.63	2.59	P	OUT
QL04413	perfluoroalkanesulfonamido quaternary ammonium sulfonate carboxylate B, n=6	S(N(CC(O)=O)CCC[N+](CC(O)CS(=O)(O)=O)(C)C)(=O)(=O)C(F)(F)C(F)(F)C(F)(F)C(F)(F)C(F)(F)C(F)(F)F		-8.51	0.65	3.10	P	IN
QL04414	perfluoroalkanesulfonamido quaternary ammonium sulfonate carboxylate B, n=5	S(N(CC(O)=O)CCC[N+](CC(O)CS(=O)(O)=O)(C)C)(=O)(=O)C(F)(F)C(F)(F)C(F)(F)C(F)(F)C(F)(F)F		-8.20	0.65	3.00	P	OUT
QL04415	perfluoroalkanesulfonamido quaternary ammonium sulfonate carboxylate B, n=4	S(N(CC(O)=O)CCC[N+](CC(O)CS(=O)(O)=O)(C)C)(=O)(=O)C(F)(F)C(F)(F)C(F)(F)C(F)(F)F		-7.83	0.64	2.87	P	OUT
QL04416	perfluoroalkanesulfonamido quaternary ammonium sulfonate carboxylate B, n=3	S(N(CC(O)=O)CCC[N+](CC(O)CS(=O)(O)=O)(C)C)(=O)(=O)C(F)(F)C(F)(F)C(F)(F)F		-7.54	0.64	2.80	P	OUT
QL04417	perfluoroalkanesulfonamido quaternary ammonium sulfonate carboxylate A, n=6	S(N(CC(O)=O)CCC[N+](CCCS(=O)(O)=O)(C)C)(=O)(=O)C(F)(F)C(F)(F)C(F)(F)C(F)(F)C(F)(F)C(F)(F)F		-8.50	0.65	3.10	P	IN
QL04418	perfluoroalkanesulfonamido quaternary ammonium sulfonate carboxylate A, n=5	S(N(CC(O)=O)CCC[N+](CCCS(=O)(O)=O)(C)C)(=O)(=O)C(F)(F)C(F)(F)C(F)(F)C(F)(F)C(F)(F)F		-8.19	0.65	3.00	P	OUT
QL04419	perfluoroalkanesulfonamido quaternary ammonium sulfonate carboxylate A, n=4	S(N(CC(O)=O)CCC[N+](CCCS(=O)(O)=O)(C)C)(=O)(=O)C(F)(F)C(F)(F)C(F)(F)C(F)(F)F		-7.81	0.64	2.87	P	OUT
QL04420	perfluoroalkanesulfonamido quaternary ammonium sulfonate carboxylate A, n=3	S(N(CC(O)=O)CCC[N+](CCCS(=O)(O)=O)(C)C)(=O)(=O)C(F)(F)C(F)(F)C(F)(F)F		-7.55	0.64	2.82	P	OUT
QL04421	perfluoroalkanesulfonamido quaternary ammonium disulfonate B, n=6	S(N(CC(O)CS(=O)(O)=O)CCC[N+](CC(O)CS(=O)(O)=O)(C)C)(=O)(=O)C(F)(F)C(F)(F)C(F)(F)C(F)(F)C(F)(F)C(F)(F)F		-8.51	0.65	3.09	P	IN
QL04422	perfluoroalkanesulfonamido quaternary ammonium disulfonate B, n=5	S(N(CC(O)CS(=O)(O)=O)CCC[N+](CC(O)CS(=O)(O)=O)(C)C)(=O)(=O)C(F)(F)C(F)(F)C(F)(F)C(F)(F)C(F)(F)F		-8.20	0.65	2.98	P	IN
QL04423	perfluoroalkanesulfonamido quaternary ammonium disulfonate B, n=4	S(N(CC(O)CS(=O)(O)=O)CCC[N+](CC(O)CS(=O)(O)=O)(C)C)(=O)(=O)C(F)(F)C(F)(F)C(F)(F)C(F)(F)F		-7.86	0.64	2.86	P	IN
QL04424	perfluoroalkanesulfonamido quaternary ammonium disulfonate B, n=3	S(N(CC(O)CS(=O)(O)=O)CCC[N+](CC(O)CS(=O)(O)=O)(C)C)(=O)(=O)C(F)(F)C(F)(F)C(F)(F)F		-7.52	0.64	2.75	P	OUT
QL04425	perfluoroalkanesulfonamido quaternary ammonium disulfonate B, n=2	S(N(CC(O)CS(=O)(O)=O)CCC[N+](CC(O)CS(=O)(O)=O)(C)C)(=O)(=O)C(F)(F)C(F)(F)F		-7.47	0.63	2.80	P	OUT
QL04426	perfluoroalkanesulfonamido quaternary diol sulfonic acid, n=6	S(N(CC(O)CS(O)(=O)=O)CCC[N+](CCO)(C)C)(=O)(=O)C(F)(F)C(F)(F)C(F)(F)C(F)(F)C(F)(F)C(F)(F)F		-8.02	0.65	2.90	P	IN
QL04427	perfluoroalkanesulfonamido quaternary diol sulfonic acid, n=5	S(N(CC(O)CS(O)(=O)=O)CCC[N+](CCO)(C)C)(=O)(=O)C(F)(F)C(F)(F)C(F)(F)C(F)(F)C(F)(F)F		-7.76	0.64	2.83	P	OUT
QL04428	perfluoroalkanesulfonamido quaternary diol sulfonic acid, n=4	S(N(CC(O)CS(O)(=O)=O)CCC[N+](CCO)(C)C)(=O)(=O)C(F)(F)C(F)(F)C(F)(F)C(F)(F)F		-7.37	0.64	2.70	P	OUT
QL04429	perfluoroalkanesulfonamido quaternary diol sulfonic acid, n=3	S(N(CC(O)CS(O)(=O)=O)CCC[N+](CCO)(C)C)(=O)(=O)C(F)(F)C(F)(F)C(F)(F)F		-6.96	0.63	2.58	P	OUT
QL04430	N-ethyl perfluorodecane sulfonamido acetic acid, n=10	S(N(CC)CC(O)=O)(=O)(=O)C(F)(F)C(F)(F)C(F)(F)C(F)(F)C(F)(F)C(F)(F)C(F)(F)C(F)(F)C(F)(F)C(F)(F)F		-8.78	0.67	2.95	P	IN
QL04431	N-ethyl perfluorononane sulfonamido acetic acid, n=9	S(N(CC)CC(O)=O)(=O)(=O)C(F)(F)C(F)(F)C(F)(F)C(F)(F)C(F)(F)C(F)(F)C(F)(F)C(F)(F)C(F)(F)F		-8.62	0.67	2.90	P	IN
QL04432	N-ethyl perfluoropropane sulfonamido acetic acid, n=3	S(N(CC)CC(O)=O)(=O)(=O)C(F)(F)C(F)(F)C(F)(F)F		-6.45	0.64	2.18	P	IN
QL04433	N-ethyl perfluoroethane sulfonamido acetic acid, n=2	S(N(CC)CC(O)=O)(=O)(=O)C(F)(F)C(F)(F)F		-5.65	0.63	1.92	P	OUT
QL04434	perfluoroalkanesulfonamido betaine C, n =6	S(N(CC)CCC[N+](CCC(O)=O)(C)C)(=O)(=O)C(F)(F)C(F)(F)C(F)(F)C(F)(F)C(F)(F)C(F)(F)F		-8.39	0.65	3.07	P	OUT
QL04435	perfluoroalkanesulfonamido betaine C, n =5	S(N(CC)CCC[N+](CCC(O)=O)(C)C)(=O)(=O)C(F)(F)C(F)(F)C(F)(F)C(F)(F)C(F)(F)F		-7.99	0.64	2.94	P	OUT
QL04436	perfluoroalkanesulfonamido quaternary ammonium carboxylic acid, n=6	S(N(CCC(=O)O)CCC[N+](C)(C)C)(=O)(=O)C(F)(F)C(F)(F)C(F)(F)C(F)(F)C(F)(F)C(F)(F)F		-7.90	0.65	2.75	P	IN
QL04437	perfluoroalkanesulfonamido quaternary ammonium carboxylic acid, n=5	S(N(CCC(=O)O)CCC[N+](C)(C)C)(=O)(=O)C(F)(F)C(F)(F)C(F)(F)C(F)(F)C(F)(F)F		-7.67	0.65	2.69	P	IN
QL04438	perfluoroalkanesulfonamido quaternary ammonium carboxylic acid, n=4	S(N(CCC(=O)O)CCC[N+](C)(C)C)(=O)(=O)C(F)(F)C(F)(F)C(F)(F)C(F)(F)F		-7.19	0.64	2.52	P	IN

QL04439	perfluoroalkanesulfonamido quaternary ammonium carboxylic acid, n=3	S(N(CCC(=O)O)CCC[N+](C)(C)C)(=O)(=O)C(F)(F)C(F)(F)C(F)(F)F		-6.84	0.64	2.43	P	IN
QL04440	perfluoroalkanesulfonamido quaternary ammonium dicarboxylate B, n=6	S(N(CCC(O)=O)CCC[N+](CC(O)=O)(C)C)(=O)(=O)C(F)(F)C(F)(F)C(F)(F)C(F)(F)C(F)(F)C(F)(F)F		-8.09	0.65	2.95	P	IN
QL04441	perfluoroalkanesulfonamido quaternary ammonium dicarboxylate B, n=5	S(N(CCC(O)=O)CCC[N+](CC(O)=O)(C)C)(=O)(=O)C(F)(F)C(F)(F)C(F)(F)C(F)(F)C(F)(F)F		-7.84	0.64	2.88	P	OUT
QL04442	perfluoroalkanesulfonamido quaternary ammonium dicarboxylate B, n=4	S(N(CCC(O)=O)CCC[N+](CC(O)=O)(C)C)(=O)(=O)C(F)(F)C(F)(F)C(F)(F)C(F)(F)F		-7.33	0.64	2.70	P	OUT
QL04443	perfluoroalkanesulfonamido quaternary ammonium dicarboxylate C, n=6	S(N(CCC(O)=O)CCC[N+](CCC(O)=O)(C)C)(=O)(=O)C(F)(F)C(F)(F)C(F)(F)C(F)(F)C(F)(F)C(F)(F)F		-8.26	0.64	3.06	P	OUT
QL04444	perfluoroalkanesulfonamido quaternary ammonium dicarboxylate C, n=5	S(N(CCC(O)=O)CCC[N+](CCC(O)=O)(C)C)(=O)(=O)C(F)(F)C(F)(F)C(F)(F)C(F)(F)C(F)(F)F		-7.81	0.64	2.90	P	OUT
QL04445	perfluoroalkanesulfonamido quaternary ammonium dicarboxylate C, n=4	S(N(CCC(O)=O)CCC[N+](CCC(O)=O)(C)C)(=O)(=O)C(F)(F)C(F)(F)C(F)(F)C(F)(F)F		-7.51	0.63	2.83	P	OUT
QL04446	perfluoroalkanesulfonamido quaternary ammonium dicarboxylate C, n=3	S(N(CCC(O)=O)CCC[N+](CCC(O)=O)(C)C)(=O)(=O)C(F)(F)C(F)(F)C(F)(F)F		-7.01	0.63	2.67	P	OUT
QL04447	perfluorooctanesulfonamido amine carboxylic acid B, n=8	S(N(CCC(O)=O)CCCN(C)C)(=O)(=O)C(F)(F)C(F)(F)C(F)(F)C(F)(F)C(F)(F)C(F)(F)C(F)(F)C(F)(F)F		-8.30	0.65	2.95	P	IN
QL04448	perfluoroheptanesulfonamido amine carboxylic acid B, n=7	S(N(CCC(O)=O)CCCN(C)C)(=O)(=O)C(F)(F)C(F)(F)C(F)(F)C(F)(F)C(F)(F)C(F)(F)C(F)(F)F		-8.10	0.65	2.90	P	IN
QL04449	perfluorohexanesulfonamido amine carboxylic acid B, n=6	S(N(CCC(O)=O)CCCN(C)C)(=O)(=O)C(F)(F)C(F)(F)C(F)(F)C(F)(F)C(F)(F)C(F)(F)F		-7.68	0.64	2.75	P	IN
QL04450	perfluoropentanesulfonamido amine carboxylic acid B, n=5	S(N(CCC(O)=O)CCCN(C)C)(=O)(=O)C(F)(F)C(F)(F)C(F)(F)C(F)(F)C(F)(F)F		-7.43	0.64	2.70	P	OUT
QL04451	perfluorobutanesulfonamido amine carboxylic acid B, n=4	S(N(CCC(O)=O)CCCN(C)C)(=O)(=O)C(F)(F)C(F)(F)C(F)(F)C(F)(F)F		-6.91	0.63	2.52	P	OUT
QL04452	perfluoropropanesulfonamido amine carboxylic acid B, n=3	S(N(CCC(O)=O)CCCN(C)C)(=O)(=O)C(F)(F)C(F)(F)C(F)(F)F		-6.53	0.63	2.44	P	OUT
QL04453	perfluorohexanesulfonamido amine sulfonic acid, n=6	S(N(CCCS(=O)(O)=O)C(CO)CCN(C)C)(=O)(=O)C(F)(F)C(F)(F)C(F)(F)C(F)(F)C(F)(F)C(F)(F)F		-7.62	0.64	2.73	P	IN
QL04454	perfluoropentanesulfonamido amine sulfonic acid, n=5	S(N(CCCS(=O)(O)=O)C(CO)CCN(C)C)(=O)(=O)C(F)(F)C(F)(F)C(F)(F)C(F)(F)C(F)(F)F		-7.36	0.64	2.67	P	OUT
QL04455	perfluoroalkanesulfonamido quaternary ammonium disulfonate A, n=8	S(N(CCCS(=O)(O)=O)CCC[N+](CCCS(=O)(O)=O)(C)C)(=O)(=O)C(F)(F)C(F)(F)C(F)(F)C(F)(F)C(F)(F)C(F)(F)C(F)(F)C(F)(F)F		-8.95	0.66	3.24	P	OUT
QL04456	perfluoroalkanesulfonamido quaternary ammonium disulfonate A, n=7	S(N(CCCS(=O)(O)=O)CCC[N+](CCCS(=O)(O)=O)(C)C)(=O)(=O)C(F)(F)C(F)(F)C(F)(F)C(F)(F)C(F)(F)C(F)(F)C(F)(F)F		-8.77	0.65	3.18	P	IN
QL04457	perfluoroalkanesulfonamido quaternary ammonium disulfonate A, n=6	S(N(CCCS(=O)(O)=O)CCC[N+](CCCS(=O)(O)=O)(C)C)(=O)(=O)C(F)(F)C(F)(F)C(F)(F)C(F)(F)C(F)(F)C(F)(F)F		-8.48	0.65	3.08	P	IN
QL04458	perfluoroalkanesulfonamido quaternary ammonium disulfonate A, n=5	S(N(CCCS(=O)(O)=O)CCC[N+](CCCS(=O)(O)=O)(C)C)(=O)(=O)C(F)(F)C(F)(F)C(F)(F)C(F)(F)C(F)(F)F		-8.22	0.65	3.00	P	OUT
QL04459	perfluoroalkanesulfonamido quaternary ammonium disulfonate A, n=4	S(N(CCCS(=O)(O)=O)CCC[N+](CCCS(=O)(O)=O)(C)C)(=O)(=O)C(F)(F)C(F)(F)C(F)(F)C(F)(F)F		-7.83	0.64	2.86	P	OUT
QL04460	perfluoroalkanesulfonamido quaternary ammonium disulfonate A, n=3	S(N(CCCS(=O)(O)=O)CCC[N+](CCCS(=O)(O)=O)(C)C)(=O)(=O)C(F)(F)C(F)(F)C(F)(F)F		-7.49	0.64	2.76	P	OUT
QL04461	perfluoroalkanesulfonamido quaternary ammonium triol sulfonic acid, n=6	S(N(CCCS(O)(=O)=O)C(CO)CC[N+](CC(CO)O)(C)C)(=O)(=O)C(F)(F)C(F)(F)C(F)(F)C(F)(F)C(F)(F)C(F)(F)F		-8.11	0.64	3.02	P	OUT
QL04462	perfluoroalkanesulfonamido quaternary ammonium triol sulfonic acid, n=5	S(N(CCCS(O)(=O)=O)C(CO)CC[N+](CC(CO)O)(C)C)(=O)(=O)C(F)(F)C(F)(F)C(F)(F)C(F)(F)C(F)(F)F		-7.79	0.64	2.92	P	OUT
QL04463	perfluoroalkanesulfonamido quaternary ammonium triol sulfonic acid, n=4	S(N(CCCS(O)(=O)=O)C(CO)CC[N+](CC(CO)O)(C)C)(=O)(=O)C(F)(F)C(F)(F)C(F)(F)C(F)(F)F		-7.49	0.63	2.84	P	OUT
QL04464	perfluoroalkanesulfonamido quaternary ammonium triol sulfonic acid, n=3	S(N(CCCS(O)(=O)=O)C(CO)CC[N+](CC(CO)O)(C)C)(=O)(=O)C(F)(F)C(F)(F)C(F)(F)F		-7.05	0.63	2.70	P	OUT
QL04465	perfluoroalkanesulfonamido quaternary ammonium triol sulfonic acid, n=2	S(N(CCCS(O)(=O)=O)C(CO)CC[N+](CC(CO)O)(C)C)(=O)(=O)C(F)(F)C(F)(F)F		-6.88	0.62	2.71	P	OUT
QL04466	perfluoroalkanesulfonamido quaternary ammonium alcohol sulfonic acid, n=8	S(N(CCCS(O)(=O)=O)CCC[N+](CCO)(C)C)(=O)(=O)C(F)(F)C(F)(F)C(F)(F)C(F)(F)C(F)(F)C(F)(F)C(F)(F)C(F)(F)F		-8.55	0.65	3.08	P	IN
QL04467	perfluoroalkanesulfonamido quaternary ammonium alcohol sulfonic acid, n=7	S(N(CCCS(O)(=O)=O)CCC[N+](CCO)(C)C)(=O)(=O)C(F)(F)C(F)(F)C(F)(F)C(F)(F)C(F)(F)C(F)(F)C(F)(F)F		-8.37	0.65	3.04	P	IN
QL04468	perfluoroalkanesulfonamido quaternary ammonium alcohol sulfonic acid, n=6	S(N(CCCS(O)(=O)=O)CCC[N+](CCO)(C)C)(=O)(=O)C(F)(F)C(F)(F)C(F)(F)C(F)(F)C(F)(F)C(F)(F)F		-8.00	0.65	2.90	P	IN
QL04469	perfluoroalkanesulfonamido quaternary ammonium alcohol sulfonic acid, n=5	S(N(CCCS(O)(=O)=O)CCC[N+](CCO)(C)C)(=O)(=O)C(F)(F)C(F)(F)C(F)(F)C(F)(F)C(F)(F)F		-7.74	0.64	2.83	P	OUT
QL04470	perfluoroalkanesulfonamido quaternary ammonium alcohol sulfonic acid, n=4	S(N(CCCS(O)(=O)=O)CCC[N+](CCO)(C)C)(=O)(=O)C(F)(F)C(F)(F)C(F)(F)C(F)(F)F		-7.33	0.64	2.70	P	OUT
QL04471	perfluoroalkanesulfonamido quaternary ammonium alcohol sulfonic acid, n=3	S(N(CCCS(O)(=O)=O)CCC[N+](CCO)(C)C)(=O)(=O)C(F)(F)C(F)(F)C(F)(F)F		-6.95	0.63	2.60	P	OUT

QL04472	perfluoroalkanesulfonamido quaternary ammonium alcohol sulfonic acid, n=2		S(N(CCCS(O)(=O)=O)CCC[N+](CCO)(C)C)(=O)(=O)C(F)(F)C(F)(F)F		-6.78	0.62	2.62	P	OUT
QL04473	perfluoroalkanesulfonamido sulfonate betaine E, n=6		S(N(CCO)CCC[N+](CC(O)CS(=O)(O)=O)(C)C)(=O)(=O)C(F)(F)C(F)(F)C(F)(F)C(F)(F)C(F)(F)C(F)(F)F		-8.41	0.65	3.11	P	OUT
QL04474	perfluoroalkanesulfonamido sulfonate betaine E, n=5		S(N(CCO)CCC[N+](CC(O)CS(=O)(O)=O)(C)C)(=O)(=O)C(F)(F)C(F)(F)C(F)(F)C(F)(F)C(F)(F)F		-8.11	0.64	3.02	P	OUT
QL04475	perfluoroalkanesulfonamido sulfonate betaine E, n=4		S(N(CCO)CCC[N+](CC(O)CS(=O)(O)=O)(C)C)(=O)(=O)C(F)(F)C(F)(F)C(F)(F)C(F)(F)F		-7.70	0.64	2.88	P	OUT
QL04476	perfluoroalkanesulfonamido sulfonate betaine E, n=3		S(N(CCO)CCC[N+](CC(O)CS(=O)(O)=O)(C)C)(=O)(=O)C(F)(F)C(F)(F)C(F)(F)F		-7.41	0.63	2.82	P	OUT
QL04477	perfluoroalkanesulfonamido quaternary ammonium diol B, n=8		S(N(CCO)CCC[N+](CCO)(C)C)(=O)(=O)C(F)(F)C(F)(F)C(F)(F)C(F)(F)C(F)(F)C(F)(F)C(F)(F)C(F)(F)F		-8.50	0.65	3.12	P	OUT
QL04478	perfluoroalkanesulfonamido quaternary ammonium diol B, n=7		S(N(CCO)CCC[N+](CCO)(C)C)(=O)(=O)C(F)(F)C(F)(F)C(F)(F)C(F)(F)C(F)(F)C(F)(F)C(F)(F)F		-8.32	0.64	3.08	P	OUT
QL04479	perfluoroalkanesulfonamido quaternary ammonium diol B, n=6		S(N(CCO)CCC[N+](CCO)(C)C)(=O)(=O)C(F)(F)C(F)(F)C(F)(F)C(F)(F)C(F)(F)C(F)(F)F		-7.93	0.64	2.95	P	OUT
QL04480	perfluoroalkanesulfonamido quaternary ammonium diol B, n=5		S(N(CCO)CCC[N+](CCO)(C)C)(=O)(=O)C(F)(F)C(F)(F)C(F)(F)C(F)(F)C(F)(F)F		-7.66	0.63	2.89	P	OUT
QL04481	perfluoroalkanesulfonamido quaternary ammonium diol B, n=4		S(N(CCO)CCC[N+](CCO)(C)C)(=O)(=O)C(F)(F)C(F)(F)C(F)(F)C(F)(F)F		-7.17	0.63	2.74	P	OUT
QL04482	perfluoroalkanesulfonamido quaternary ammonium diol B, n=3		S(N(CCO)CCC[N+](CCO)(C)C)(=O)(=O)C(F)(F)C(F)(F)C(F)(F)F		-6.72	0.62	2.62	P	OUT
QL04483	perfluoroalkanesulfonamido quaternary ammonium diol B, n=2		S(N(CCO)CCC[N+](CCO)(C)C)(=O)(=O)C(F)(F)C(F)(F)F		-6.06	0.61	2.44	P	OUT
QL04484	perfluorohexanesulfonamido amine carboxylic acid A, n=6		S(NC(C(O)=O)CCN(C)C)(=O)(=O)C(F)(F)C(F)(F)C(F)(F)C(F)(F)C(F)(F)C(F)(F)F		-7.96	0.66	2.74	P	IN
QL04485	perfluoropentanesulfonamido amine carboxylic acid A, n=5		S(NC(C(O)=O)CCN(C)C)(=O)(=O)C(F)(F)C(F)(F)C(F)(F)C(F)(F)C(F)(F)F		-7.74	0.65	2.69	P	IN
QL04486	perfluorobutanesulfonamido amine carboxylic acid A, n=4		S(NC(C(O)=O)CCN(C)C)(=O)(=O)C(F)(F)C(F)(F)C(F)(F)C(F)(F)F		-7.24	0.65	2.52	P	IN
QL04487	perfluoroalkanesulfonamido quaternary ammonium triol, n=6		S(NC(CO)CC[N+](CC(O)CO)(C)C)(=O)(=O)C(F)(F)C(F)(F)C(F)(F)C(F)(F)C(F)(F)C(F)(F)F		-8.31	0.65	3.05	P	OUT
QL04488	perfluoroalkanesulfonamido quaternary ammonium triol, n=5		S(NC(CO)CC[N+](CC(O)CO)(C)C)(=O)(=O)C(F)(F)C(F)(F)C(F)(F)C(F)(F)C(F)(F)F		-7.96	0.64	2.94	P	OUT
QL04489	perfluoroalkanesulfonamido quaternary ammonium triol, n=4		S(NC(CO)CC[N+](CC(O)CO)(C)C)(=O)(=O)C(F)(F)C(F)(F)C(F)(F)C(F)(F)F		-7.70	0.64	2.89	P	OUT
QL04490	perfluoroalkanesulfonamido quaternary ammonium triol, n=3		S(NC(CO)CC[N+](CC(O)CO)(C)C)(=O)(=O)C(F)(F)C(F)(F)C(F)(F)F		-7.20	0.63	2.73	P	OUT
QL04491	perfluoroalkanesulfonamido quaternary ammonium diol A, n=6		S(NCC(O)C[N+](CCO)(C)C)(=O)(=O)C(F)(F)C(F)(F)C(F)(F)C(F)(F)C(F)(F)C(F)(F)F		-8.31	0.65	2.95	P	IN
QL04492	perfluoroalkanesulfonamido quaternary ammonium diol A, n=5		S(NCC(O)C[N+](CCO)(C)C)(=O)(=O)C(F)(F)C(F)(F)C(F)(F)C(F)(F)C(F)(F)F		-8.02	0.65	2.87	P	IN
QL04493	perfluoroalkanesulfonamido quaternary ammonium diol A, n=4		S(NCC(O)C[N+](CCO)(C)C)(=O)(=O)C(F)(F)C(F)(F)C(F)(F)C(F)(F)F		-7.56	0.64	2.71	P	IN
QL04494	perfluoroalkanesulfonamido quaternary ammonium oxide, n=9		S(NCCC[N+]([O-])(C)C)(=O)(=O)C(F)(F)C(F)(F)C(F)(F)C(F)(F)C(F)(F)C(F)(F)C(F)(F)C(F)(F)C(F)(F)F		-9.09	0.67	3.07	P	OUT
QL04495	perfluoroalkanesulfonamido quaternary ammonium oxide, n=7		S(NCCC[N+]([O-])(C)C)(=O)(=O)C(F)(F)C(F)(F)C(F)(F)C(F)(F)C(F)(F)C(F)(F)C(F)(F)F		-8.61	0.67	2.89	P	IN
QL04496	perfluoroalkanesulfonamido quaternary ammonium oxide, n=6	30295-56-8	S(NCCC[N+]([O-])(C)C)(=O)(=O)C(F)(F)C(F)(F)C(F)(F)C(F)(F)C(F)(F)C(F)(F)F		-8.23	0.67	2.74	P	IN
QL04497	perfluoroalkanesulfonamido quaternary ammonium oxide, n=5		S(NCCC[N+]([O-])(C)C)(=O)(=O)C(F)(F)C(F)(F)C(F)(F)C(F)(F)C(F)(F)F		-8.04	0.66	2.70	P	IN
QL04498	perfluoroalkanesulfonamido quaternary ammonium oxide, n=4		S(NCCC[N+]([O-])(C)C)(=O)(=O)C(F)(F)C(F)(F)C(F)(F)C(F)(F)F		-7.56	0.66	2.52	P	IN
QL04499	perfluoroalkanesulfonamido quaternary ammonium, n=3		S(NCCC[N+](C)(C)C)(=O)(=O)C(F)(F)C(F)(F)C(F)(F)F		-7.18	0.65	2.42	P	IN
QL04500	perfluoroalkanesulfonamido betaine A, n =6		S(NCCC[N+](CC(O)=O)(C)C)(=O)(=O)C(F)(F)C(F)(F)C(F)(F)C(F)(F)C(F)(F)C(F)(F)F		-8.37	0.66	2.95	P	IN
QL04501	perfluoroalkanesulfonamido betaine A, n =5		S(NCCC[N+](CC(O)=O)(C)C)(=O)(=O)C(F)(F)C(F)(F)C(F)(F)C(F)(F)C(F)(F)F		-8.10	0.65	2.87	P	IN
QL04502	perfluoroalkanesulfonamido betaine A, n =4		S(NCCC[N+](CC(O)=O)(C)C)(=O)(=O)C(F)(F)C(F)(F)C(F)(F)C(F)(F)F		-7.64	0.65	2.71	P	IN
QL04503	perfluoroalkanesulfonamido betaine A, n =3		S(NCCC[N+](CC(O)=O)(C)C)(=O)(=O)C(F)(F)C(F)(F)C(F)(F)F		-7.28	0.64	2.62	P	IN
QL04504	perfluoroalkanesulfonamido sulfonate betaine B, n=9		S(NCCC[N+](CC(O)CS(=O)(O)=O)(C)C)(=O)(=O)C(F)(F)C(F)(F)C(F)(F)C(F)(F)C(F)(F)C(F)(F)C(F)(F)C(F)(F)C(F)(F)F		-9.47	0.67	3.38	P	OUT

QL04505	perfluoroalkanesulfonamido sulfonate betaine B, n=8	S(NCCC[N+](CC(O)CS(=O)(O)=O)(C)C)(=O)(=O)C(F)(F)C(F)(F)C(F)(F)C(F)(F)C(F)(F)C(F)(F)C(F)(F)C(F)(F)F		-9.19	0.66	3.27	P	OUT
QL04506	perfluoroalkanesulfonamido sulfonate betaine B, n=7	S(NCCC[N+](CC(O)CS(=O)(O)=O)(C)C)(=O)(=O)C(F)(F)C(F)(F)C(F)(F)C(F)(F)C(F)(F)C(F)(F)C(F)(F)F		-8.98	0.66	3.21	P	OUT
QL04507	perfluoroalkanesulfonamido sulfonate betaine B, n=6	S(NCCC[N+](CC(O)CS(=O)(O)=O)(C)C)(=O)(=O)C(F)(F)C(F)(F)C(F)(F)C(F)(F)C(F)(F)C(F)(F)F		-8.66	0.66	3.08	P	IN
QL04508	perfluoroalkanesulfonamido sulfonate betaine B, n=5	S(NCCC[N+](CC(O)CS(=O)(O)=O)(C)C)(=O)(=O)C(F)(F)C(F)(F)C(F)(F)C(F)(F)C(F)(F)F		-8.41	0.65	3.01	P	IN
QL04509	perfluoroalkanesulfonamido sulfonate betaine B, n=4	S(NCCC[N+](CC(O)CS(=O)(O)=O)(C)C)(=O)(=O)C(F)(F)C(F)(F)C(F)(F)C(F)(F)F		-8.00	0.65	2.87	P	IN
QL04510	perfluoroalkanesulfonamido sulfonate betaine B, n=3	S(NCCC[N+](CC(O)CS(=O)(O)=O)(C)C)(=O)(=O)C(F)(F)C(F)(F)C(F)(F)F		-7.79	0.64	2.84	P	OUT
QL04511	perfluoroalkanesulfonamido betaine B, n=6	S(NCCC[N+](CCC(O)=O)(C)C)(=O)(=O)C(F)(F)C(F)(F)C(F)(F)C(F)(F)C(F)(F)C(F)(F)F		-8.50	0.65	3.05	P	IN
QL04512	perfluoroalkanesulfonamido betaine B, n=5	S(NCCC[N+](CCC(O)=O)(C)C)(=O)(=O)C(F)(F)C(F)(F)C(F)(F)C(F)(F)C(F)(F)F		-8.10	0.65	2.92	P	IN
QL04513	perfluoroalkanesulfonamido betaine B, n=4	S(NCCC[N+](CCC(O)=O)(C)C)(=O)(=O)C(F)(F)C(F)(F)C(F)(F)C(F)(F)F		-7.80	0.64	2.84	P	OUT
QL04514	perfluoroalkanesulfonamido betaine B, n=3	S(NCCC[N+](CCC(O)=O)(C)C)(=O)(=O)C(F)(F)C(F)(F)C(F)(F)F		-7.29	0.64	2.68	P	OUT
QL04515	perfluoroalkanesulfonamido quaternary ammonium diol C, n=6	S(NCCC[N+](CCC(O)CO)(C)C)(=O)(=O)C(F)(F)C(F)(F)C(F)(F)C(F)(F)C(F)(F)C(F)(F)F		-8.45	0.65	3.10	P	OUT
QL04516	perfluoroalkanesulfonamido quaternary ammonium diol C, n=5	S(NCCC[N+](CCC(O)CO)(C)C)(=O)(=O)C(F)(F)C(F)(F)C(F)(F)C(F)(F)C(F)(F)F		-8.18	0.64	3.03	P	OUT
QL04517	perfluoroalkanesulfonamido quaternary ammonium diol C, n=4	S(NCCC[N+](CCC(O)CO)(C)C)(=O)(=O)C(F)(F)C(F)(F)C(F)(F)C(F)(F)F		-7.74	0.64	2.89	P	OUT
QL04518	perfluoroalkanesulfonamido sulfonate betaine A, n=8	S(NCCC[N+](CCCS(=O)(O)=O)(C)C)(=O)(=O)C(F)(F)C(F)(F)C(F)(F)C(F)(F)C(F)(F)C(F)(F)C(F)(F)C(F)(F)F		-9.19	0.66	3.27	P	OUT
QL04519	perfluoroalkanesulfonamido sulfonate betaine A, n=7	S(NCCC[N+](CCCS(=O)(O)=O)(C)C)(=O)(=O)C(F)(F)C(F)(F)C(F)(F)C(F)(F)C(F)(F)C(F)(F)C(F)(F)F		-8.99	0.66	3.21	P	OUT
QL04520	perfluoroalkanesulfonamido sulfonate betaine A, n=6	S(NCCC[N+](CCCS(=O)(O)=O)(C)C)(=O)(=O)C(F)(F)C(F)(F)C(F)(F)C(F)(F)C(F)(F)C(F)(F)F		-8.66	0.66	3.09	P	IN
QL04521	perfluoroalkanesulfonamido sulfonate betaine A, n=5	S(NCCC[N+](CCCS(=O)(O)=O)(C)C)(=O)(=O)C(F)(F)C(F)(F)C(F)(F)C(F)(F)C(F)(F)F		-8.40	0.65	3.01	P	IN
QL04522	perfluoroalkanesulfonamido sulfonate betaine A, n=4	S(NCCC[N+](CCCS(=O)(O)=O)(C)C)(=O)(=O)C(F)(F)C(F)(F)C(F)(F)C(F)(F)F		-8.00	0.65	2.87	P	IN
QL04523	perfluoroalkanesulfonamido sulfonate betaine A, n=3	S(NCCC[N+](CCCS(=O)(O)=O)(C)C)(=O)(=O)C(F)(F)C(F)(F)C(F)(F)F		-7.79	0.64	2.85	P	OUT
QL04524	perfluoroalkanesulfonamido sulfonate betaine C, n=6	S(NCCC[N+](CCCS(=O)(O)=O)(C)CCO)(=O)(=O)C(F)(F)C(F)(F)C(F)(F)C(F)(F)C(F)(F)C(F)(F)F		-8.31	0.64	3.08	P	OUT
QL04525	perfluoroalkanesulfonamido sulfonate betaine C, n=5	S(NCCC[N+](CCCS(=O)(O)=O)(C)CCO)(=O)(=O)C(F)(F)C(F)(F)C(F)(F)C(F)(F)C(F)(F)F		-8.03	0.64	3.01	P	OUT
QL04526	perfluoroalkanesulfonamido sulfonate betaine C, n=4	S(NCCC[N+](CCCS(=O)(O)=O)(C)CCO)(=O)(=O)C(F)(F)C(F)(F)C(F)(F)C(F)(F)F		-7.60	0.63	2.87	P	OUT
QL04527	perfluoroalkanesulfonamido quaternary ammonium alcohol, n=8	S(NCCC[N+](CCO)(C)C)(=O)(=O)C(F)(F)C(F)(F)C(F)(F)C(F)(F)C(F)(F)C(F)(F)C(F)(F)C(F)(F)F		-8.79	0.66	3.12	P	OUT
QL04528	perfluoroalkanesulfonamido quaternary ammonium alcohol, n=7	S(NCCC[N+](CCO)(C)C)(=O)(=O)C(F)(F)C(F)(F)C(F)(F)C(F)(F)C(F)(F)C(F)(F)C(F)(F)F		-8.63	0.66	3.09	P	IN
QL04529	perfluoroalkanesulfonamido quaternary ammonium alcohol, n=6	S(NCCC[N+](CCO)(C)C)(=O)(=O)C(F)(F)C(F)(F)C(F)(F)C(F)(F)C(F)(F)C(F)(F)F		-8.26	0.65	2.95	P	IN
QL04530	perfluoroalkanesulfonamido quaternary ammonium alcohol, n=5	S(NCCC[N+](CCO)(C)C)(=O)(=O)C(F)(F)C(F)(F)C(F)(F)C(F)(F)C(F)(F)F		-7.97	0.65	2.87	P	IN
QL04531	perfluoroalkanesulfonamido quaternary ammonium alcohol, n=4	S(NCCC[N+](CCO)(C)C)(=O)(=O)C(F)(F)C(F)(F)C(F)(F)C(F)(F)F		-7.49	0.64	2.72	P	OUT
QL04532	perfluoroalkanesulfonamido quaternary ammonium alcohol, n=3	S(NCCC[N+](CCO)(C)C)(=O)(=O)C(F)(F)C(F)(F)C(F)(F)F		-7.11	0.63	2.63	P	OUT
QL04533	perfluoroalkanesulfonamido quaternary ammonium alcohol, n=2	S(NCCC[N+](CCO)(C)C)(=O)(=O)C(F)(F)C(F)(F)F		-6.46	0.62	2.45	P	OUT
QL04534	perfluoropropanesulfonamido amine, n =3	S(NCCCN(C)C)(=O)(=O)C(F)(F)C(F)(F)C(F)(F)F		-6.85	0.64	2.44	P	IN
QL04535	perfluoropropanesulfonamido sulfonic acid, n=6	S(NCCCS(=O)(O)=O)(=O)(=O)C(F)(F)C(F)(F)C(F)(F)C(F)(F)C(F)(F)C(F)(F)F		-8.23	0.67	2.74	P	IN
QL04536	perfluoropropanesulfonamido sulfonic acid, n=5	S(NCCCS(=O)(O)=O)(=O)(=O)C(F)(F)C(F)(F)C(F)(F)C(F)(F)C(F)(F)F		-8.04	0.66	2.70	P	IN
QL04537	perfluoropropanesulfonamido sulfonic acid, n=4	S(NCCCS(=O)(O)=O)(=O)(=O)C(F)(F)C(F)(F)C(F)(F)C(F)(F)F		-7.56	0.66	2.52	P	IN
QL04538	perfluoropropanesulfonamido sulfonic acid, n=3	S(NCCCS(=O)(O)=O)(=O)(=O)C(F)(F)C(F)(F)C(F)(F)F		-7.18	0.65	2.42	P	IN
