# Supplementary material for: How the Structure of Per- and Polyfluoroalkyl Substances (PFAS) Influences Their Binding Potency to the Peroxisome Proliferator-Activated and Thyroid Hormone Receptors—An In Silico Screening Study
Source: Molecules. 2023 Jan 4;28(2):479. doi: 10.3390/molecules28020479 (PMC9866891; doi:10.3390/molecules28020479)
Supplement: Supplementary file 1 [file molecules-28-00479-s001.zip › Supplementary Materials 2.pdf]

Supplementary Materials 2

How the structure of Per- and Polyfluoroalkyl Substances (PFAS) influences their binding potency to the peroxisome proliferator-activated and thyroid hormone receptors – an *in silico* screening study.

Dominika Jurkiewicz[1+], Anita Sosnowska[1+*], Natalia Buławska[1], Maciej Stępnik[1], Harrie Besselink[2], Peter Behnisch[2], Tomasz Puzyn[1,3*]

1. QSAR Lab, ul. Trzy Lipy 3, Gdańsk, Poland
2. BioDetection Systems B.V., Science Park 406, 1098XH, Amsterdam, the Netherlands.
3. University of Gdansk, Faculty of Chemistry, Wita Stwosza 63, 80-308 Gdansk, Poland

* Corresponding authors.

E-mail address: t.puzyn@ug.edu.pl, t.puzyn@qsarlab.com, a.sosnowska@qsarlab.com

\+ equal contribution

Table of contents

Model's details 3

Peroxisome proliferator-activated receptor α 3

Fig. S1 Plot of calculated versus predicted values of binding scores PPARα (A). Williams plot: dash line indicates the critical leverage value, solid lines represent ±3 standard deviation units. (B). 4

Peroxisome proliferator-activated receptor β 4

Fig. S2 Plot of calculated versus predicted values of binding scores PPARβ (A). Williams plot: dash line indicates the critical leverage value, solid lines represent ±3 standard deviation units. (B). 5

Peroxisome proliferator-activated receptor γ 5

Fig. S3 Plot of calculated versus predicted values of binding scores PPARγ (A). Williams plot: dash line indicates the critical leverage value, solid lines represent ±3 standard deviation units. (B). 6

Thyroid Hormone Receptor α 6

Fig. S4 Plot of calculated versus predicted values of binding scores TRα (A). Williams plot: dash line indicates the critical leverage value, solid lines represent ±3 standard deviation units. (B). 7

Thyroid Hormone Receptor β 7

Fig. S5 Plot of calculated versus predicted values of binding scores TRβ (A). Williams plot: dash line indicates the critical leverage value, solid lines represent ±3 standard deviation units. (B). 8

QMRF prepared for developed models 9

Peroxisome proliferator-activated receptor α 9

Peroxisome proliferator-activated receptor β 16

Peroxisome proliferator-activated receptor γ 22

Thyroid hormone receptor α 28

Thyroid hormone receptor β 34

Bibliography 40

Model's details

Peroxisome proliferator-activated receptor α

The best QSAR model for predicting the PFAS-PPAR***α*** binding affinity utilized two molecular descriptors: radial centric information index (ICR) and path/walk 2 – Randic shape index (PW2), between which the correlation coefficient was small (r=0.31). ICR descriptor gives information about centricity in the molecules,[1] whereas the PW2 is defined as (P2/W2), the quotient of the path length of 2 (P2) and walk length of 2 (W2).[2] The visual representation of the observed vs. predicted values of binding scores for PPARα is presented in Fig. S1A. The results indicated a high correlation between calculated docking scores (using Endocrine Disruptome Tool)[3] and predicted by the developed QSAR model values. The values of R^2, $Q^2_{LOO,}$ and $Q^2_{F1,F2,F3}$ are close to 1, which confirms that the predictions were accurate, the model is stable, and has good predictive abilities. The model is characterized by relatively low values of the root mean square errors (Table 2) of prediction in the training and validation sets (respectively $RMSE_C$, $RMSE_{CV,}$ and $RMSE_{EXT}$). More details about statistical results can be found in Supplementary Materials 1. To verify the reliability of predictions (they should be located within the optimum prediction space) we have checked the applicability domain of the developed model using the Williams plot (Fig. S1B). This method allows to graphically present the standardized residuals (differences between observed and predicted values) versus the leverage value (indicates deviations of the structure of the compound from those used for the QSAR development). All in this work studied compounds were in the range of residuals differing by ±3 standard deviations from the mean value. The obtained leverage threshold for this model is h* = 0.310. One compound on the Williams plot: trifluoroacetic acid - TFA (43) has a higher leverage value than h*, but its activity has been predicted correctly.[4] To prove that the model is not a 'correlation-by-chance', Y^2_{SCR} has been calculated (Table 2). Regarding the mechanistic interpretation of the model, PFAS with the lowest binding scores - 10:2 FTUCA (2) and 4:2 diPAPs (3) exceeding -8.9 kcal/mol have also a high branching descriptor value

(ICR),[5] which confirms that binding affinity is higher when it comes to long-chain perfluoroalkyl compounds (with more than eight carbon atoms in a molecule).[6]

Fig. S1 Plot of calculated versus predicted values of binding scores PPARα (A). Williams plot: dash line indicates the critical leverage value, solid lines represent ±3 standard deviation units. (B).

A B

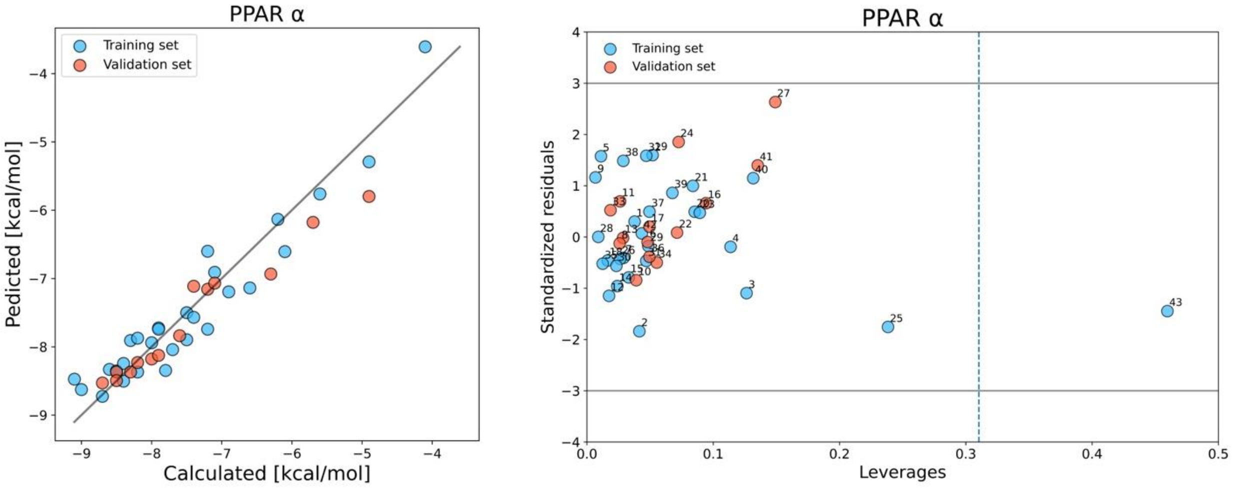

Peroxisome proliferator-activated receptor β

We have estimated the binding probability of PFAS to PPARβ based on two molecular descriptors 2D - radial centric information index (ICR) and the percentage of halogen atoms (X%). X% is a constitutional indices descriptor, which increases with the number of CF_2 groups in a molecule, whereas ICR is the topological indices descriptor that gives information about branching in structure.[1] The correlation coefficient of the descriptors was satisfactory at r=0.26. We have indicated high similarity between calculated and predicted values using a scatter plot representing observed vs. predicted values (Fig. S2A). The main reason for choosing a model based on this structure properties was the highest R^2, $Q^2_{LOO,}$ and $Q^2_{F1,F2,F3}$ (Table 2), but also low values of the root mean square errors ($RMSE_C$, $RMSEc_V$, $RMSE_{EXT}$). The Williams plot (Fig. S2B) indicates that all compounds were located within the optimum prediction space of the model. One point - TFA (43) has a leverage value higher than h*=0.310. However, attendance of this compound in the training set stabilizes the model. Nevertheless, the

predictions for compounds with h> h* are treated as the results of extrapolation, so they are less reliable.[7] Scrambling test (Y^2_{SCR}) confirms that the presented model is statistically significant. Only five compounds (10:2FTUCA, 4:2diPAPs, PFDA, PFNS, and PFUdA) from the entire dataset show a higher probability of binding to PPARβ (values are lower than -9.6). Compounds with the highest binding scores are characterized by X% and ICR on a high level (X% not lower than 40.91, and ICR higher than 2.51).

Fig. S2 Plot of calculated versus predicted values of binding scores PPARβ (A). Williams plot: dash line indicates the critical leverage value, solid lines represent ±3 standard deviation units. (B).

A B

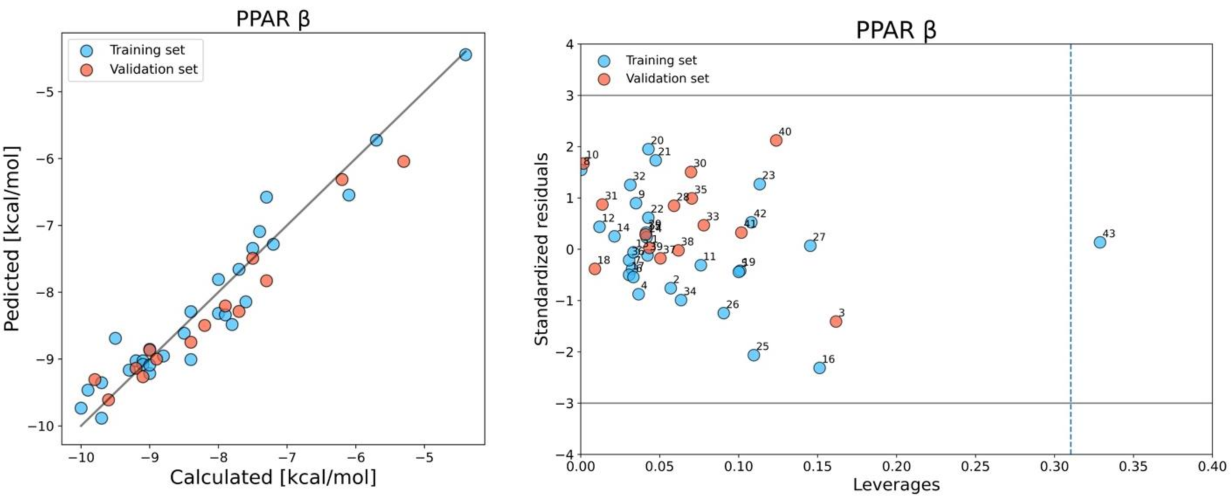

Peroxisome proliferator-activated receptor γ

To estimate PFAS binding probability to PPARγ we have developed a QSAR model based on the same descriptors as for PPARβ (X% and ICR), with a correlation at r=0.26. Fig. S3A presents a significant correlation between the observed vs. predicted values of binding scores for PPAR***γ***. Model is characterized by satisfactory goodness-of-fit, robustness, and predictive capabilities (R^2, $Q^2_{LOO,}$ and $Q^2_{F1,F2,F3}$), which proves the accuracy of predictions. Values of errors ($RMSE_C$, $RMSE_{CV}$, $RMSE_{EXT}$) are also acceptable (Table 2). A small Y^2_{SCR} value (Table 2) confirms that the model is not a 'correlation by chance'. Considering the applicability domain (Fig. S3B), it can be seen that TFA (43) structurally diverges from the rest

compounds but is situated in the range of residuals differing by ±3 standard deviations from the mean value. In this case, h*=0.273, which is lower than for PPARα and β, because the validation set includes a smaller number of compounds (different data split). The lowest binding score was observed for PFUdA (42), which had the highest value of percentage of halogen atoms (X%) and a relatively high value of ICR.

Fig. S3 Plot of calculated versus predicted values of binding scores PPAR*γ* (A). Williams plot: dash line indicates the critical leverage value, solid lines represent ±3 standard deviation units. (B).

A B

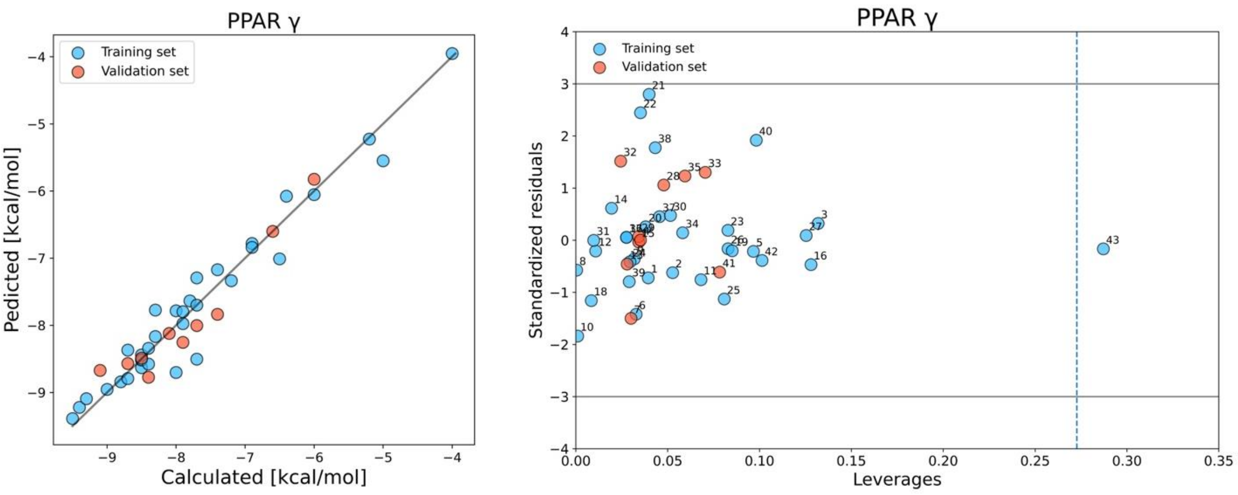

Thyroid Hormone Receptor α

We developed the QSAR model for predicting PFAS-TRα binding probability based on two molecular descriptors: percentage of halogen atoms in a molecule (X%) and radial centric information index (ICR). The correlation coefficient of the descriptors was low (r=0.16). A good correlation between observed (calculated using Endocrine Disruptome Tool)[3] and predicted values can be seen on a scatter plot (Fig. S4A). High values of R^2, $Q^2_{LOO,}$ and $Q^2_{F1,F2,F3}$ (all close to 0.9), confirm the accuracy of prediction and stability of the model with good predictive abilities. Additionally, the model is characterized by low values of the root mean square errors of prediction in the training and validation sets (respectively $RMSE_C$, $RMSE_{CV,}$ and $RMSE_{EXT}$) (Table 2). To verify the reliability of the predictions we have applied the

Williams plot (Fig. S4B). Compounds that were found between ±3 standard deviations from the mean value and do not exceed the value of h*=0.273 are inside the structural space of the model. In the case of TRα, only one compound – TFA (43) has been classified as an outlier due to the higher leverage value than h*. To prove that the model is not a 'correlation-by-chance', Y^2_{SCR} has been calculated (Table 2). 8:2 FTUCA with a binding energy of -9.8 kcal/mol, an ICR value of more than 2.5 and an X% of 53.33 was identified as the compound with the highest probability of binding to the TRα. Other compounds with a high probability of binding (binding score of less than -9.3 kcal/mol) had an X% value of no lower than 45 and an ICR of more than 2.24.

Fig. S4 Plot of calculated versus predicted values of binding scores TRα (A). Williams plot: dash line indicates the critical leverage value, solid lines represent ±3 standard deviation units. (B).

A B

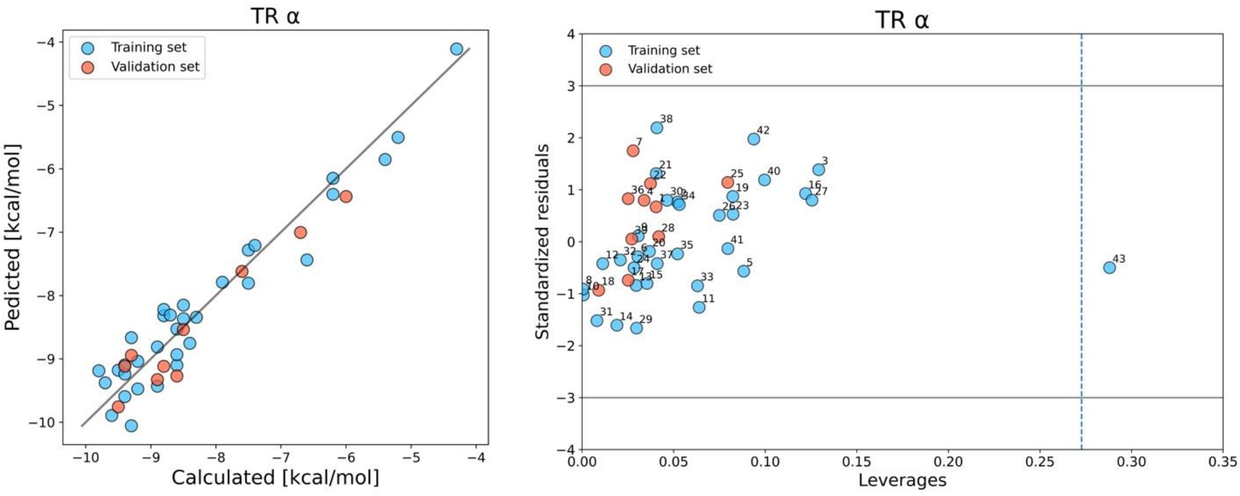

Thyroid Hormone Receptor β

The best QSAR model for predicting PFAS binding probability to TRβ is based on two relatively low correlated (r=0.47) molecular descriptors: the percentage of halogen atoms in a molecule (X%) and the total path count (TPC). TPC is the walk and path count descriptor, which describes the total number of paths (from order 0 to the maximum path length of the molecule). TPC reflects the size of the molecule as well as its complexity.[8] The plot of observed

vs. predicted values of binding scores for TRβ (Fig. S5A) confirms the good correlation between calculated and predicted data. We have selected the appropriate model based on robustness and ability of prediction confirmed by R^2, $Q^2_{LOO,}$ and $Q^2_{F1,F2,F3}$ values which are close to 0.95. A low value of scrambling validation (Y^2_{SCR}) confirms that the presented model is statistically significant. The analysis of the applicability domain using the Williams plot (Fig. S5B) shows that the same chemical as in the case of TRα - TFA (43) from the training set came as an outlier. It has a higher average hat value than h* = 0.273, however, does not have standard residuals greater than 3 standard deviation units (±3σ). Thus, it can be stated that TFA (43) is slightly structurally different from the rest in the set, and additionally has a positive effect on the extension of the model applicability domain. In the training set, two compounds: 10:2 FTOH (1) and 10:2 FTUCA (2) had the highest probability of binding to TRβ (binding score lower than -10.6 kcal/mol) with having one of the highest TPC values in the set (6.39), and an X% value exceeding 50.

Fig. S5 Plot of calculated versus predicted values of binding scores TRβ (A). Williams plot: dash line indicates the critical leverage value, solid lines represent ±3 standard deviation units. (B).

A

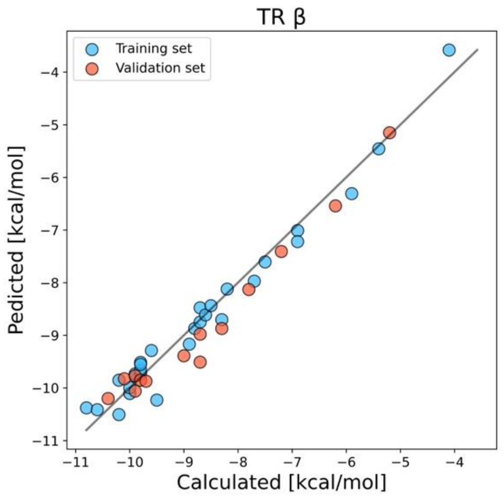

B

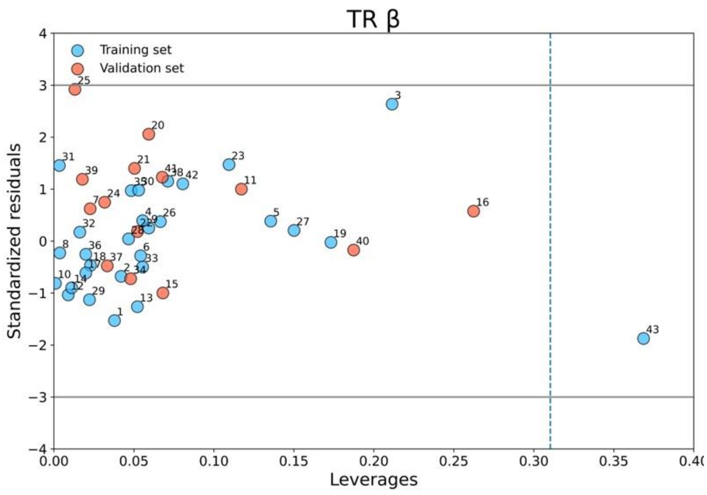

QMRF prepared for developed models

Peroxisome proliferator-activated receptor α

1. QSAR identifier

1.1. QSAR identifier (title):

QSAR model for predicting PFAS binding affinity PPARα

1.2. Other related models:

N/A

1.3. Software coding the model:

Python 3.8.8

2. General information

2.1. Date of QMRF:

May 2022

2.2. QMRF author(s) and contact details:

QSAR Lab Sp. z o.o.

Trzy Lipy 3 Street, Building B,

80-172 Gdansk, Poland

+48 795 160 760

contact@qsarlab.com https://www.qsarlab.com

2.3. Date of QMRF update(s):

N/A

2.4. QMRF update(s):

N/A

2.5. Model developer(s) and contact details:

1. D. Jurkiewicz, QSAR Lab Ltd, d.jurkiewicz@qsarlab.com
2. N. Buławska, QSAR Lab Ltd, n.bulawska@qsarlab.com

2.6. Date of model development and/or publication:

Published in 2022

2.7. Reference(s) to main scientific papers and/or software package:

1. alvaDesc
Mauri, A. (2020). alvaDesc: A Tool to Calculate and Analyze Molecular Descriptors and Fingerprints. In K. Roy (Ed.), Ecotoxicological QSARs (pp. 801–820). Humana Press Inc. https://doi.org/10.1007/978-1-0716-0150-1_32

2. Python 3.8.8

Python Software Foundation. Python Language Reference, version 3.8.8. Available at http://www.python.org

2.8. Availability of information about the model:

The set of 43 PFAS compounds encompassing perfluorinated carboxylic acids (PFCAs), fluorotelomer alcohols (FTOHs), perfluorinated sulfonic acids (PFSAs), and other related PFAS, were selected to model development. All compounds have a molecular weight lower than 600 g/mol. Model was obtained based on two theoretical molecular descriptors (PW2 and ICR). Structures, names, acronyms, CAS numbers, and SMILES of PFAS examined in this study are listed in a Supplementary Materials. For the modeling, the multiple linear regression (MLR) approach was applied.

2.9. Availability of another QMRF for exactly the same model:

N/A

3. Defining the endpoint – OECD Principle 1

3.1. Species:

N/A

3.2. Endpoint:

Binding potency

3.3. Comment on the endpoint:

Binding score obtained for peroxisome proliferator-activated receptor α

3.4. Endpoint units:

kcal/mol

3.5. Dependent variable:

PPARα BS

3.6. Experimental protocol:

Predictions were obtained according to Endocrine Disruptome Tool. Docking simulations are provided by Docking Interface for Target Systems (DOTS) platform. Molecular docking experiments were performed with Autodock Vina 1.1.2 using default settings.

3.7. Endpoint data quality and variability:

Docking was carried out on 103 different crystal structures. All structures and protocols are validated. The most important parameter is area under the curve (AUC) under receiver operating characteristic (ROC) curve. Models with higher AUC values should perform better. Endocrine Disruptome is intended to be used for screening

4. Defining the algorithm – OECD Principle 2

4.1. Type of model:

QSAR model -Multiple linear regression (MLR)

4.2. Explicit algorithm:

PPARαBS=-7.499(±0.067) - 0.947(±0.07) x ICR - 0.394(±0.07) x PW2

4.3. Descriptors in the model:

radial centric information index (ICR)

path/walk 2 – Randic shape index (PW2)

4.4. Descriptor selection:

Structural descriptors, which have an influence on the probability of binding to receptor were selected. Pairs of descriptors between which correlation did not exceed 0.6 were selected.

4.5. Algorithm and descriptor generation:

A total of 186 1D and 2D descriptors of different types were calculated using SMILES in alvaDesc software. They belong to constitutional indices, topological indices, walk and path counts, and molecular properties. The number of descriptors has been first reduced by constant values and by descriptors with no data for at least one compound.

4.6. Software name and version for descriptor generation:

alvaDesc software

4.7. Chemicals/ Descriptors ratio:

43 chemicals / 2 descriptors

5. Defining the applicability domain – OECD Principle 3

5.1. Description of the applicability domain of the model:

Applicability domain was verified based on a plot of the standardized residuals versus the leverage values, so-called Williams plot. All compounds used in the training and validation sets should be situated in the range of residuals differing by ±3 standard deviations from the mean value. They should not also exceed the h* value.

5.2. Method used to assess the applicability domain:

As it has been noted in section 5.1, the applicability domain of the model was assessed by the leverage approach, providing a cut-off hat value (h*=0.310). The response applicability domain was also verified by the standardized residuals.

5.3. Software name and version for applicability domain assessment:

Williams plot was generated in Python 3.8.8.

5.4. Limits of applicability:

One compounds (TFA) was classified as outlier, because its leverage value was higher than h*.

6. Defining goodness-of-fit and robustness – OECD Principle 4

6.1. Availability of the training set:

Yes

6.2. Availability information for the training set:

CAS RN:Yes

Chemical Name:Yes

Smiles:Yes

Formula:No

INChI:No

MOL file:No

6.3. Data for each descriptor variable for the training set:

All

6.4. Data for the dependent variable (response) for the training set:

All

6.5. Other information about the training set:

Dataset was split into training (T) and validation (V) sets by following the "1:Z algorithm". Training set consist of 29 compounds.

6.6. Pre-processing of data before modelling:

Standard scaler - Standardize features by removing the mean and scaling to unit variance.

6.7. Statistics for goodness-of-fit:

R^2= 0.908

$RMSE_C$= 0.360

MAE_C= 0.305

6.8. Robustness – Statistics obtained by leave-one-out cross validation:

Q^2_{LOO} = 0.863

$RMSE_{CV}$= 0.438

MAE_{CV}= 0.357

6.9. Robustness – Statistics obtained by leave-many-out cross validation:

N/A

6.10. Robustness – Statistics obtained by Y-scrambling:

R^2Y_{SCR}= 0.070

6.11. Robustness – Statistics obtained by bootstrap:

N/A

6.12. Robustness – Statistics obtained by other methods:

N/A

7. Defining predictivity – OECD Principle 4

7.1. Availability of the external validation set:

Yes

7.2. Availability information for the external validation set:

CAS RN:Yes

Chemical Name:Yes

Smiles:Yes

Formula:No

INChI:No

MOL file:No

7.3. Data for each descriptor variable for the external validation set:

Yes

7.4. Data for the dependent variable (response) for the external validation set:

Yes

7.5. Other information about the external validation set:

Validation set consist of 14 compounds.

7.6. Experimental design of test set:

Every 3th compound in a group of chemicals, sorted by experimental values in ascending order, was assigned to the validation set.

7.7. Predictivity – Statistics obtained by external validation:

Q^2_{F1}= 0.896

Q^2_{F2}= 0.896

Q^2_{F3}= 0.911

$RMSE_{EXT}$= 0.352

MAE_{EXT}=0.257

CCC_{EXT}= 0.937

7.8. Predictivity – Assessment of the external validation set:

Range of response for prediction set (n=14)

compounds:
PPARα BS (kcal/mol): -8.7/-4.9 (range of corresponding training set: -9.1/-4.1)
Range of modeling descriptors for prediction set (n=14)
compounds:
PW2: 0.66/0.69 (range of corresponding training set: 0.63/0.7)
ICR: 1.32/2.74 (range of corresponding training set: 0.86/2.96)

7.9. Comments on the external validation of the model:

N/A

8. Providing a mechanistic interpretation – OECD Principle 5

8.1. Mechanistic basis of the model:

The model was developed by statistical approach. No mechanistic was defined a priori.

8.2. A priori or a posteriori mechanistic interpretation:

The PFAS binding probability to PPARα depends on the presence of CF_2 groups, and of other structural properties (e.g chain length, functional groups). With increase of molecular descriptors (PW2 and. ICR) values, the binding score decrease.

8.3. Other information about the mechanistic interpretation:

N/A

9. Miscellaneous information

9.1. Comments:

N/A

9.2. Bibliography:

Katra Kolšek, Janez Mavri, Marija Sollner Dolenc, Stanislav Gobec, and Samo Turk

Journal of Chemical Information and Modeling 2014 54 (4), 1254-1267

DOI: 10.1021/ci400649p

9.3. Supporting information:

N/A

10. Summary for the JRC QSAR Model Database (compiled by JRC)

10.1. QMRF number:

10.2. Publication date:

10.3. Keywords:

10.4. Comments

Peroxisome proliferator-activated receptor β

1. QSAR identifier

1.1. QSAR identifier (title):

QSAR model for predicting PFAS binding affinity to PPARβ

1.2. Other related models:

N/A

1.3. Software coding the model:

Python 3.8.8

2. General information

2.1. Date of QMRF:

May 2022

2.2. QMRF author(s) and contact details:

QSAR Lab Sp. z o.o.

Trzy Lipy 3 Street, Building B,

80-172 Gdansk, Poland

+48 795 160 760

contact@qsarlab.com https://www.qsarlab.com

2.3. Date of QMRF update(s):

N/A

2.4. QMRF update(s):

N/A

2.5. Model developer(s) and contact details:

1. D. Jurkiewicz, QSAR Lab Ltd, d.jurkiewicz@qsarlab.com

2. N. Buławska, QSAR Lab Ltd, n.bulawska@qsarlab.com

2.6. Date of model development and/or publication:

Published in 2022

2.7. Reference(s) to main scientific papers and/or software package:

1. alvaDesc
Mauri, A. (2020). alvaDesc: A Tool to Calculate and Analyze Molecular Descriptors and Fingerprints. In K. Roy (Ed.), Ecotoxicological QSARs (pp. 801–820). Humana Press Inc. https://doi.org/10.1007/978-1-0716-0150-1_32

2. Python 3.8.8

Python Software Foundation. Python Language Reference, version 3.8.8. Available at http://www.python.org

2.8. Availability of information about the model:

The set of 43 PFAS compounds encompassing perfluorinated carboxylic acids (PFCAs), fluorotelomer alcohols (FTOHs), perfluorinated sulfonic acids (PFSAs), and other related PFAS, were selected to model development. All compounds have a molecular weight lower than 600 g/mol. Model was obtained based on two theoretical molecular descriptors (X% and ICR). Structures, names, acronyms, CAS numbers, and SMILES of PFAS examined in this study are listed in a Supplementary Materials. For the modeling, the multiple linear regression (MLR) approach was applied.

2.9. Availability of another QMRF for exactly the same model:

N/A

3. Defining the endpoint – OECD Principle 1

3.1. Species:

N/A

3.2. Endpoint:

Binding potency

3.3. Comment on the endpoint:

Binding score obtained for peroxisome proliferator-activated receptor β

3.4. Endpoint units:

kcal/mol

3.5. Dependent variable:

PPARβ BS

3.6. Experimental protocol:

Predictions were obtained according to Endocrine Disruptome Tool. Docking simulations are provided by Docking Interface for Target Systems (DOTS) platform. Molecular docking experiments were performed with Autodock Vina 1.1.2 using default settings.

3.7. Endpoint data quality and variability:

Docking was carried out on 103 different crystal structures. All structures and protocols are validated. The most important parameter is area under the curve (AUC) under receiver operating characteristic (ROC) curve. Models with higher AUC values should perform better. Endocrine Disruptome is intended to be used for screening.

4. Defining the algorithm – OECD Principle 2

4.1. Type of model:

QSAR model -Multiple linear regression (MLR)

4.2. Explicit algorithm:

PPARβ BS=- 8.248(±0.069) - 1.059(±0.071) x ICR -0.409(±0.071) x X%

4.3. Descriptors in the model:

radial centric information index (ICR)

percentage of halogen atoms (X%)

4.4. Descriptor selection:

Structural descriptors, which have an influence on the probability of binding to receptor were selected. Pairs of descriptors between which correlation did not exceed 0.6 were selected.

4.5. Algorithm and descriptor generation:

A total of 186 1D and 2D descriptors of different types were calculated using SMILES in alvaDesc software. They belong to constitutional indices, topological indices, walk and path counts, and molecular properties. The number of descriptors has been first reduced by constant values and by descriptors with no data for at least one compound.

4.6. Software name and version for descriptor generation:

alvaDesc software

4.7. Chemicals/ Descriptors ratio:

43 chemicals / 2 descriptors

5. Defining the applicability domain – OECD Principle 3

5.1. Description of the applicability domain of the model:

Applicability domain was verified based on a plot of the standardized residuals versus the leverage values, so-called Williams plot. All compounds used in the training and validation sets should be situated in the range of residuals differing by ±3 standard deviations from the mean value. They should not also exceed the h* value.

5.2. Method used to assess the applicability domain:

As it has been noted in section 5.1, the applicability domain of the model was assessed by the leverage approach, providing a cut-off hat value (h*=0.310). The response applicability domain was also verified by the standardized residuals.

5.3. Software name and version for applicability domain assessment:

Williams plot was generated in Python 3.8.8.

5.4. Limits of applicability:

One compounds (TFA) was classified as outlier, because its leverage value was higher than h*.

6. Defining goodness-of-fit and robustness – OECD Principle 4

6.1. Availability of the training set:

Yes

6.2. Availability information for the training set:

CAS RN:Yes

Chemical Name:Yes

Smiles:Yes

Formula:No

INChI:No

MOL file:No

6.3. Data for each descriptor variable for the training set:

All

6.4. Data for the dependent variable (response) for the training set:

All

6.5. Other information about the training set:

Dataset was split into training (T) and validation (V) sets by following the "1:Z algorithm". Training set consist of 29 compounds.

6.6. Pre-processing of data before modelling:

Standard scaler - Standardize features by removing the mean and scaling to unit variance.

6.7. Statistics for goodness-of-fit:

R^2= 0.924

$RMSE_C$= 0.352

MAE_C= 0.272

6.8. Robustness – Statistics obtained by leave-one-out cross validation:

Q^2_{LOO}= 0.903

$RMSE_{CV}$= 0.398

MAE_{CV}= 0.304

6.9. Robustness – Statistics obtained by leave-many-out cross validation:

N/A

6.10. Robustness – Statistics obtained by Y-scrambling:

R^2Y_{SCR}= 0.068

6.11. Robustness – Statistics obtained by bootstrap:

N/A

6.12. Robustness – Statistics obtained by other methods:

N/A

7. Defining predictivity – OECD Principle 4

7.1. Availability of the external validation set:

Yes

7.2. Availability information for the external validation set:

CAS RN:Yes

Chemical Name:Yes

Smiles:Yes

Formula:No

INChI:No

MOL file:No

7.3. Data for each descriptor variable for the external validation set:

Yes

7.4. Data for the dependent variable (response) for the external validation set:

Yes

7.5. Other information about the external validation set:

Validation set consist of 14 compounds.

7.6. Experimental design of test set:

Every 3th compound in a group of chemicals, sorted by experimental values in ascending order, was assigned to the validation set.

7.7. Predictivity – Statistics obtained by external validation:

Q^2_{F1}= 0.917

Q^2_{F2}= 0.916

Q^2_{F3}= 0.922

$RMSE_{EXT}$= 0.359

MAE_{EXT}= 0.279

CCC_{EXT}= 0.952

7.8. Predictivity – Assessment of the external validation set:

Range of response for prediction set (n=14) compounds:
PPARβ BS (kcal/mol): -9.8/-5.3(range of corresponding training set: -10/-4.4)
Range of modeling descriptors for prediction set (n=14) compounds:
X%: 40.91/58.62(range of corresponding training set: 37.5/60)

ICR: 1.36/2.96 (range of corresponding training set: 0.86/2.74)

7.9. Comments on the external validation of the model:

N/A

8. Providing a mechanistic interpretation – OECD Principle 5

8.1. Mechanistic basis of the model:

The model was developed by statistical approach. No mechanistic was defined a priori.

8.2. A priori or a posteriori mechanistic interpretation:

The PFAS binding probability to PPARβ depends on the presence of CF_2 groups, and of other structural properties (e.g chain length, functional groups). With increase of molecular descriptors (X% and ICR) values, the binding score decrease.

8.3. Other information about the mechanistic interpretation:

N/A

9. Miscellaneous information

9.1. Comments:

N/A

9.2. Bibliography:

Katra Kolšek, Janez Mavri, Marija Sollner Dolenc, Stanislav Gobec, and Samo Turk

Journal of Chemical Information and Modeling 2014 54 (4), 1254-1267

DOI: 10.1021/ci400649p

9.3. Supporting information:

N/A

10. Summary for the JRC QSAR Model Database (compiled by JRC)

10.1. QMRF number:

10.2. Publication date:

10.3. Keywords:

10.4. Comments:

Peroxisome proliferator-activated receptor γ

1. QSAR identifier

1.1. QSAR identifier (title):

QSAR model for predicting PFAS binding affinity to PPARγ

1.2. Other related models:

N/A

1.3. Software coding the model:

Python 3.8.8

2. General information

2.1. Date of QMRF:

May 2022

2.2. QMRF author(s) and contact details:

QSAR Lab Sp. z o.o.

Trzy Lipy 3 Street, Building B,

80-172 Gdansk, Poland

+48 795 160 760

contact@qsarlab.com https://www.qsarlab.com

2.3. Date of QMRF update(s):

N/A

2.4. QMRF update(s):

N/A

2.5. Model developer(s) and contact details:

1. D. Jurkiewicz, QSAR Lab Ltd, d.jurkiewicz@qsarlab.com

2. N. Buławska, QSAR Lab Ltd, n.bulawska@qsarlab.com

2.6. Date of model development and/or publication:

Published in 2022

2.7. Reference(s) to main scientific papers and/or software package:

1. alvaDesc
Mauri, A. (2020). alvaDesc: A Tool to Calculate and Analyze Molecular Descriptors and Fingerprints. In K. Roy (Ed.), Ecotoxicological QSARs (pp. 801–820). Humana Press Inc. https://doi.org/10.1007/978-1-0716-0150-1_32

2. Python 3.8.8

Python Software Foundation. Python Language Reference, version 3.8.8. Available at http://www.python.org

2.8. Availability of information about the model:

The set of 43 PFAS compounds encompassing perfluorinated carboxylic acids (PFCAs), fluorotelomer alcohols (FTOHs), perfluorinated sulfonic acids (PFSAs), and other related PFAS, were selected to model development. All compounds have a molecular weight lower than 600 g/mol. Model was obtained based on two theoretical molecular descriptors (X% and ICR). Structures, names, acronyms, CAS numbers, and SMILES of PFAS examined in this study are listed in a Supplementary Materials. For the modeling, the multiple linear regression (MLR) approach was applied.

2.9. Availability of another QMRF for exactly the same model:

N/A

3. Defining the endpoint – OECD Principle 1

3.1. Species:

N/A

3.2. Endpoint:

Binding potency

3.3. Comment on the endpoint:

Binding score obtained for peroxisome proliferator-activated receptor γ

3.4. Endpoint units:

kcal/mol

3.5. Dependent variable:

PPARγ BS

3.6. Experimental protocol:

Predictions were obtained according to Endocrine Disruptome Tool. Docking simulations are provided by Docking Interface for Target Systems (DOTS) platform. Molecular docking experiments were performed with Autodock Vina 1.1.2 using default settings.

3.7. Endpoint data quality and variability:

Docking was carried out on 103 different crystal structures. All structures and protocols are validated. The most important parameter is area under the curve (AUC) under receiver operating characteristic (ROC) curve. Models with higher AUC values should perform better. Endocrine Disruptome is intended to be used for screening.

4. Defining the algorithm – OECD Principle 2

4.1. Type of model:

QSAR model -Multiple linear regression (MLR)

4.2. Explicit algorithm:

PPARγ BS=-7.727(±0.052) - 1.099(±0.053) x ICR - 0.398(±0.053) x X%

4.3. Descriptors in the model:

radial centric information index (ICR)

percentage of halogen atoms (X%)

4.4. Descriptor selection:

Structural descriptors, which have an influence on the probability of binding to receptor were selected. Pairs of descriptors between which correlation did not exceed 0.6 were selected.

4.5. Algorithm and descriptor generation:

A total of 186 1D and 2D descriptors of different types were calculated using SMILES in alvaDesc software. They belong to constitutional indices, topological indices, walk and path counts, and molecular properties. The number of descriptors has been first reduced by constant values and by descriptors with no data for at least one compound.

4.6. Software name and version for descriptor generation:

alvaDesc software

4.7. Chemicals/ Descriptors ratio:

43 chemicals / 2 descriptors

5. Defining the applicability domain – OECD Principle 3

5.1. Description of the applicability domain of the model:

Applicability domain was verified based on a plot of the standardized residuals versus the leverage values, so-called Williams plot. All compounds used in the training and validation sets should be situated in the range of residuals differing by ±3 standard deviations from the mean value. They should not also exceed the h* value.

5.2. Method used to assess the applicability domain:

As it has been noted in section 5.1, the applicability domain of the model was assessed by the leverage approach, providing a cut-off hat value (h*=0.273). The response applicability domain was also verified by the standardized residuals.

5.3. Software name and version for applicability domain assessment:

Williams plot was generated in Python 3.8.8.

5.4. Limits of applicability:

One compounds (TFA) was classified as outlier, because its leverage value was higher than h*.

6. Defining goodness-of-fit and robustness – OECD Principle 4

6.1. Availability of the training set:

Yes

6.2. Availability information for the training set:

CAS RN:Yes

Chemical Name:Yes

Smiles:Yes

Formula:No

INChI:No

MOL file:No

6.3. Data for each descriptor variable for the training set:

All

6.4. Data for the dependent variable (response) for the training set:

All

6.5. Other information about the training set:

Dataset was split into training (T) and validation (V) sets by following the "1:Z algorithm". Training set consist of 10 compounds.

6.6. Pre-processing of data before modelling:

Standard scaler - Standardize features by removing the mean and scaling to unit variance.

6.7. Statistics for goodness-of-fit:

R^2=0.949

$RMSE_C$= 0.287

MAE_C= 0.201

6.8. Robustness – Statistics obtained by leave-one-out cross validation:

Q^2_{LOO} = 0.940

$RMSE_{CV}$= 0.310

MAE_{CV}= 0.219

6.9. Robustness – Statistics obtained by leave-many-out cross validation:

N/A

6.10. Robustness – Statistics obtained by Y-scrambling:

R^2Y_{SCR}= 0.062

6.11. Robustness – Statistics obtained by bootstrap:

N/A

6.12. Robustness – Statistics obtained by other methods:

N/A

7. Defining predictivity – OECD Principle 4

7.1. Availability of the external validation set:

Yes

7.2. Availability information for the external validation set:

CAS RN:Yes

Chemical Name:Yes

Smiles:Yes

Formula:No

INChI:No

MOL file:No

7.3. Data for each descriptor variable for the external validation set:

Yes

7.4. Data for the dependent variable (response) for the external validation set:

Yes

7.5. Other information about the external validation set:

Validation set consist of 10 compounds.

7.6. Experimental design of test set:

Every 4th compound in a group of chemicals, sorted by experimental values in ascending order, was assigned to the validation set.

7.7. Predictivity – Statistics obtained by external validation:

Q^2_{F1}= 0.907

Q^2_{F2}= 0.906

Q^2_{F3}= 0.952

$RMSE_{EXT}$ = 0.279

MAE_{EXT}= 0.223

CCC_{EXT} = 0.953

7.8. Predictivity – Assessment of the external validation set:

Range of response for prediction set (n=10)
compounds:
PPARγ BS (kcal/mol): -9.1/-6.0 (range of corresponding training set: -9.5/-4.0)

Range of modeling descriptors for prediction set (n=10) compounds:
ICR: 1.45/2.68 (range of corresponding training set: 0.86/2.96)
X%: 42.86/58.62 (range of corresponding training set: 37.5/60.0)

7.9. Comments on the external validation of the model:

N/A

8. Providing a mechanistic interpretation – OECD Principle 5

8.1. Mechanistic basis of the model:

The model was developed by statistical approach. No mechanistic was defined a priori.

8.2. A priori or a posteriori mechanistic interpretation:

The PFAS binding probability to PPARγ depends on the presence of CF_2 groups, and of other structural properties (e.g chain length, functional groups). With increase of molecular descriptors (X% and ICR) values, the binding score decrease.

8.3. Other information about the mechanistic interpretation:

N/A

9. Miscellaneous information

9.1. Comments:

N/A

9.2. Bibliography:

Katra Kolšek, Janez Mavri, Marija Sollner Dolenc, Stanislav Gobec, and Samo Turk

Journal of Chemical Information and Modeling 2014 54 (4), 1254-1267

DOI: 10.1021/ci400649p

9.3. Supporting information:

N/A

10. Summary for the JRC QSAR Model Database (compiled by JRC)

10.1. QMRF number:

10.2. Publication date:

10.3. Keywords:

10.4. Comments:

Thyroid hormone receptor α

1. QSAR identifier

1.1. QSAR identifier (title):

QSAR model for predicting PFAS binding affinity to TRα

1.2. Other related models:

N/A

1.3. Software coding the model:

Python 3.8.8

2. General information

2.1. Date of QMRF:

May 2022

2.2. QMRF author(s) and contact details:

QSAR Lab Sp. z o.o.

Trzy Lipy 3 Street, Building B,

80-172 Gdansk, Poland

+48 795 160 760

contact@qsarlab.com https://www.qsarlab.com

2.3. Date of QMRF update(s):

N/A

2.4. QMRF update(s):

N/A

2.5. Model developer(s) and contact details:

1. D. Jurkiewicz, QSAR Lab Ltd, d.jurkiewicz@qsarlab.com
2. N. Buławska, QSAR Lab Ltd, n.bulawska@qsarlab.com

2.6. Date of model development and/or publication:

Published in 2022

2.7. Reference(s) to main scientific papers and/or software package:

1. alvaDesc
Mauri, A. (2020). alvaDesc: A Tool to Calculate and Analyze Molecular Descriptors and Fingerprints. In K. Roy (Ed.), Ecotoxicological QSARs (pp. 801–820). Humana Press Inc. https://doi.org/10.1007/978-1-0716-0150-1_32

2. Python 3.8.8

Python Software Foundation. Python Language Reference, version 3.8.8. Available at http://www.python.org

2.8. Availability of information about the model:

The set of 43 PFAS compounds encompassing perfluorinated carboxylic acids (PFCAs), fluorotelomer alcohols (FTOHs), perfluorinated sulfonic acids (PFSAs), and other related PFAS, were selected to model development. All compounds have a molecular weight lower than 600 g/mol. Model was obtained based on two theoretical molecular descriptors (X% and ICR). Structures, names, acronyms, CAS numbers, and SMILES of PFAS examined in this study are listed in a Supplementary Materials. For the modeling, the multiple linear regression (MLR) approach was applied.

2.9. Availability of another QMRF for exactly the same model:

N/A

3. Defining the endpoint – OECD Principle 1

3.1. Species:

N/A

3.2. Endpoint:

Binding potency

3.3. Comment on the endpoint:

Binding score obtained for Thyroid hormone receptor alpha

3.4. Endpoint units:

kcal/mol

3.5. Dependent variable:

TRα BS

3.6. Experimental protocol:

Predictions were obtained according to Endocrine Disruptome Tool. Docking simulations are provided by Docking Interface for Target Systems (DOTS)33 platform. Molecular docking experiments were performed with Autodock Vina 1.1.2 using default settings.

3.7. Endpoint data quality and variability:

Docking was carried out on 103 different crystal structures. All structures and protocols are validated. The most important parameter is area under the curve (AUC) under receiver operating characteristic (ROC) curve. Models with higher AUC values should perform better. Endocrine Disruptome is intended to be used for screening.

4. Defining the algorithm – OECD Principle 2

4.1. Type of model:

QSAR model -Multiple linear regression (MLR)

4.2. Explicit algorithm:

TRα BS= -8.230 (±0.070) - 0.454 (±0.071) x X% - 1.189 (±0.071) x ICR

4.3. Descriptors in the model:

radial centric information index (ICR)

percentage of halogen atoms (X%)

4.4. Descriptor selection:

Structural descriptors, which have an influence on the probability of binding to receptor were selected. Pairs of descriptors between which correlation did not exceed 0.6 were selected.

4.5. Algorithm and descriptor generation:

A total of 186 1D and 2D descriptors of different types were calculated using SMILES in alvaDesc software. They belong to constitutional indices, topological indices, walk and path counts, and molecular properties. The number of descriptors has been first reduced by constant values and by descriptors with no data for at least one compound.

4.6. Software name and version for descriptor generation:

alvaDesc software

4.7. Chemicals/ Descriptors ratio:

43 chemicals / 2 descriptors

5. Defining the applicability domain – OECD Principle 3

5.1. Description of the applicability domain of the model:

Applicability domain was verified based on a plot of the standardized residuals versus the leverage values, so-called Williams plot. All compounds used in the training and validation sets should be situated in the range of residuals differing by ±3 standard deviations from the mean value. They should not also exceed the h* value.

5.2. Method used to assess the applicability domain:

As it has been noted in section 5.1, the applicability domain of the model was assessed by the leverage approach, providing a cut-off hat value (h*=0.273). The response applicability domain was also verified by the standardized residuals.

5.3. Software name and version for applicability domain assessment:

Williams plot was generated in Python 3.8.8.

5.4. Limits of applicability:

One compounds (TFA) was classified as outlier, because his leverage value was higher than h*.

6. Defining goodness-of-fit and robustness – OECD Principle 4

6.1. Availability of the training set:

Yes

6.2. Availability information for the training set:

CAS RN:Yes

Chemical Name:Yes

Smiles:Yes

Formula:No

INChI:No

MOL file:No

6.3. Data for each descriptor variable for the training set:

All

6.4. Data for the dependent variable (response) for the training set:

All

6.5. Other information about the training set:

Dataset was split into training (T) and validation (V) sets by following the "1:Z algorithm". Training set consist of 33 compounds.

6.6. Pre-processing of data before modelling:

Standard scaler - Standardize features by removing the mean and scaling to unit variance.

6.7. Statistics for goodness-of-fit:

R^2= 0.924

$RMSE_C$= 0.384

6.8. Robustness – Statistics obtained by leave-one-out cross validation:

Q^2_{LOO} = 0.908

$RMSE_{CV}$= 0.422

MAE_{CV}= 0.360

6.9. Robustness – Statistics obtained by leave-many-out cross validation:

N/A

6.10. Robustness – Statistics obtained by Y-scrambling:

R^2Y_{SCR}= 0.061

6.11. Robustness – Statistics obtained by bootstrap:

N/A

6.12. Robustness – Statistics obtained by other methods:

N/A

7. Defining predictivity – OECD Principle 4

7.1. Availability of the external validation set:

Yes

7.2. Availability information for the external validation set:

CAS RN:Yes

Chemical Name:Yes

Smiles:Yes

Formula:No

INChI:No

MOL file:No

7.3. Data for each descriptor variable for the external validation set:

Yes

7.4. Data for the dependent variable (response) for the external validation set:

Yes

7.5. Other information about the external validation set:

Validation set consist of 10 compounds.

7.6. Experimental design of test set:

Every 4th compound in a group of chemicals, sorted by experimental values in ascending order, was assigned to the validation set.

7.7. Predictivity – Statistics obtained by external validation:

Q^2_{F1}= 0.899

Q^2_{F2}= 0.899

Q^2_{F3}= 0.934

$RMSE_{EXT}$= 0.359

$RMSE_{EXT}$= 0.312

CCC_{EXT}= 0.946

7.8. Predictivity – Assessment of the external validation set:

Range of response for prediction set (n=10) compounds:
TRα BS (kcal/mol): -9.5/-6.0 (range of correspondig training set: -9.8/-4.3)
Range of modeling descriptors for prediction set (n=10) compounds:
X%: 42.86/56.52 (range of corrispondig training set: 37.5/60.0)
ICR: 1.47/2.74 (range of corresponding training set: 0.86/2.96)

7.9. Comments on the external validation of the model:

N/A

8. Providing a mechanistic interpretation – OECD Principle 5

8.1. Mechanistic basis of the model:

The model was developed by statistical approach. No mechanistic was defined a priori.

8.2. A priori or a posteriori mechanistic interpretation:

The PFAS binding probability to r TRα depends on the presence of CF_2 groups, and of other structural properties (e.g chain length, functional groups). With increase of molecular descriptors (X% and ICR) values, the binding score decrease.

8.3. Other information about the mechanistic interpretation:

N/A

9. Miscellaneous information

9.1. Comments:

N/A

9.2. Bibliography:

Katra Kolšek, Janez Mavri, Marija Sollner Dolenc, Stanislav Gobec, and Samo Turk

Journal of Chemical Information and Modeling 2014 54 (4), 1254-1267

DOI: 10.1021/ci400649p

9.3. Supporting information:

N/A

10. Summary for the JRC QSAR Model Database (compiled by JRC)

10.1. QMRF number:

10.2. Publication date:

10.3. Keywords:

10.4. Comments:

Thyroid hormone receptor β

1. QSAR identifier

1.1. QSAR identifier (title):

QSAR model for predicting PFAS binding affinity to TRβ

1.2. Other related models:

N/A

1.3. Software coding the model:

Python 3.8.8

2. General information

2.1. Date of QMRF:

May 2022

2.2. QMRF author(s) and contact details:

QSAR Lab Sp. z o.o.

Trzy Lipy 3 Street, Building B,

80-172 Gdansk, Poland

+48 795 160 760

contact@qsarlab.com https://www.qsarlab.com

2.3. Date of QMRF update(s):

N/A

2.4. QMRF update(s):

N/A

2.5. Model developer(s) and contact details:

1. D. Jurkiewicz, QSAR Lab Ltd, d.jurkiewicz@qsarlab.com
2. N. Buławska, QSAR Lab Ltd, n.bulawska@qsarlab.com

2.6. Date of model development and/or publication:

Published in 2022

2.7. Reference(s) to main scientific papers and/or software package:

1. alvaDesc
Mauri, A. (2020). alvaDesc: A Tool to Calculate and Analyze Molecular Descriptors and Fingerprints. In K. Roy (Ed.), Ecotoxicological QSARs (pp. 801–820). Humana Press Inc. https://doi.org/10.1007/978-1-0716-0150-1_32

2. Python 3.8.8

Python Software Foundation. Python Language Reference, version 3.8.8. Available at http://www.python.org

2.8. Availability of information about the model:

The set of 43 PFAS compounds encompassing perfluorinated carboxylic acids (PFCAs), fluorotelomer alcohols (FTOHs), perfluorinated sulfonic acids (PFSAs), and other related PFAS, were selected to model development. All compounds have a molecular weight lower than 600 g/mol. Model was obtained based on two theoretical molecular descriptors (X% and TPC). Structures, names, acronyms, CAS numbers, and SMILES of PFAS examined in this study are listed in a Supplementary Materials. For the modeling, the multiple linear regression (MLR) approach was applied.

2.9. Availability of another QMRF for exactly the same model:

N/A

3. Defining the endpoint – OECD Principle 1

3.1. Species:

N/A

3.2. Endpoint:

Binding potency

3.3. Comment on the endpoint:

Binding score obtained for thyroid hormone receptor beta

3.4. Endpoint units:

kcal/mol

3.5. Dependent variable:

TRβ BS

3.6. Experimental protocol:

Predictions were obtained according to Endocrine Disruptome Tool. Docking simulations are provided by Docking Interface for Target Systems (DOTS) platform. Molecular docking experiments were performed with Autodock Vina 1.1.2 using default settings.

3.7. Endpoint data quality and variability:

Docking was carried out on 103 different crystal structures. All structures and protocols are validated. The most important parameter is area under the curve (AUC) under receiver operating characteristic (ROC) curve. Models with higher AUC values should perform better. Endocrine Disruptome is intended to be used for screening.

4. Defining the algorithm – OECD Principle 2

4.1. Type of model:

QSAR model -Multiple linear regression (MLR)

4.2. Explicit algorithm:

TRβ BS= -8.724 (±0.054) - 0.137 (±0.061) x X% - 1.509 (±0.061) x TPC

4.3. Descriptors in the model:

percentage of halogen atoms (X%)

total path count (TPC)

4.4. Descriptor selection:

Structural descriptors, which have an influence on the probability of binding to receptor were selected. Pairs of descriptors between which correlation did not exceed 0.6 were selected.

4.5. Algorithm and descriptor generation:

A total of 186 1D and 2D descriptors of different types were calculated using SMILES in alvaDesc software. They belong to constitutional indices, topological indices, walk and path counts, and molecular properties. The number of descriptors has been first reduced by constant values and by descriptors with no data for at least one compound.

4.6. Software name and version for descriptor generation:

alvaDesc software

4.7. Chemicals/ Descriptors ratio:

43 chemicals / 2 descriptors

5. Defining the applicability domain – OECD Principle 3

5.1. Description of the applicability domain of the model:

Applicability domain was verified based on a plot of the standardized residuals versus the leverage values, so-called Williams plot. All compounds used in the training and validation sets should be situated in the range of residuals differing by ±3 standard deviations from the mean value. They should not also exceed the h* value.

5.2. Method used to assess the applicability domain:

As it has been noted in section 5.1, the applicability domain of the model was assessed by the leverage approach, providing a cut-off hat value (h*=0.273). The response applicability domain was also verified by the standardized residuals.

5.3. Software name and version for applicability domain assessment:

Williams plot was generated in Python 3.8.8.

5.4. Limits of applicability:

One compounds (TFA) was classified as outlier, because his leverage value was higher than h*.

6. Defining goodness-of-fit and robustness – OECD Principle 4

6.1. Availability of the training set:

Yes

6.2. Availability information for the training set:

CAS RN:Yes

Chemical Name:Yes

Smiles:Yes

Formula:No

INChI:No

MOL file:No

6.3. Data for each descriptor variable for the training set:

All

6.4. Data for the dependent variable (response) for the training set:

All

6.5. Other information about the training set:

Dataset was split into training (T) and validation (V) sets by following the “1:Z algorithm”. Training set consist of 29 compounds.

6.6. Pre-processing of data before modelling:

Standard scaler - Standardize features by removing the mean and scaling to unit variance.

6.7. Statistics for goodness-of-fit:

R^2= 0.970

$RMSE_C$= 0.276

6.8. Robustness – Statistics obtained by leave-one-out cross validation:

Q^2_{LOO} = 0.955

$RMSE_{CV}$= 0.340

MAE_{CV}= 0.256

6.9. Robustness – Statistics obtained by leave-many-out cross validation:

N/A

6.10. Robustness – Statistics obtained by Y-scrambling:

R^2Y_{SCR}= 0.070

6.11. Robustness – Statistics obtained by bootstrap:

N/A

6.12. Robustness – Statistics obtained by other methods:

N/A

7. Defining predictivity – OECD Principle 4

7.1. Availability of the external validation set:

Yes

7.2. Availability information for the external validation set:

CAS RN:Yes

Chemical Name:Yes

Smiles:Yes

Formula:No

INChI:No

MOL file:No

7.3. Data for each descriptor variable for the external validation set:

Yes

7.4. Data for the dependent variable (response) for the external validation set:

Yes

7.5. Other information about the external validation set:

Validation set consist of 14 compounds.

7.6. Experimental design of test set:

Every 3th compound in a group of chemicals, sorted by experimental values in ascending order, was assigned to the validation set.

7.7. Predictivity – Statistics obtained by external validation:

Q^2_{F1}= 0.948

Q^2_{F2}= 0.947

Q^2_{F3}= 0.954

$RMSE_{EXT}$= 0.344

MAE_{EXT}=0.280

CCC_{EXT}= 0.973

7.8. Predictivity – Assessment of the external validation set:

Range of response for prediction set (n=14) compounds:
TRβ BS (kcal/mol): -10.4/-7.2 (range of corresponding training set: -10.80/-4.10)
Range of modeling descriptors for prediction set (n=14) compounds:
X%: 38.64/57.58 (range of corresponding training set: 37.5/60.0)
TPC: 5.04/6.39 (range of corresponding training set: 3.37/6.45)

7.9. Comments on the external validation of the model:

N/A

8. Providing a mechanistic interpretation – OECD Principle 5

8.1. Mechanistic basis of the model:

The model was developed by statistical approach. No mechanistic was defined a priori.

8.2. A priori or a posteriori mechanistic interpretation:

The PFAS binding probability to r TRβ depends on the presence of CF_2 groups, and of other structural properties (e.g chain length, functional groups). With increase of molecular descriptors (X% and TPC) values, the binding score decrease.

8.3. Other information about the mechanistic interpretation:

N/A

9. Miscellaneous information

9.1. Comments:

N/A

9.2. Bibliography:

Katra Kolšek, Janez Mavri, Marija Sollner Dolenc, Stanislav Gobec, and Samo Turk

Journal of Chemical Information and Modeling 2014 54 (4), 1254-1267

DOI: 10.1021/ci400649p

9.3. Supporting information:

N/A

10. Summary for the JRC QSAR Model Database (compiled by JRC)

10.1. QMRF number:

10.2. Publication date:

10.3. Keywords:

10.4. Comments

Bibliography

1. Deshpande, S., Solomon, V. R., Katti, S. B. & Prabhakar, Y. S. Topological descriptors in modelling antimalarial activity: N(1)-(7-chloro-4-quinolyl)-1,4-bis(3-aminopropyl)piperazine as prototype. *J Enzym Inhib Med Ch* 24, 94–104 (2009).

2. Veras, L. da S., Arakawa, M., Funatsu, K. & Takahata, Y. 2D and 3D QSAR studies of the receptor binding affinity of progestins. *J Brazil Chem Soc* 21, 872–881 (2010).

3. Kolšek, K., Mavri, J., Dolenc, M. S., Gobec, S. & Turk, S. Endocrine Disruptome - An Open Source Prediction Tool for Assessing Endocrine Disruption Potential through Nuclear Receptor Binding. *J Chem Inf Model* 54, 1254–1267 (2014).

4. Netzeva, T. I. *et al.* Current Status of Methods for Defining the Applicability Domain of (Quantitative) Structure-Activity Relationships. *Altern Laboratory Animals* 33, 155–173 (2005).

5. Bonchev, D. (1983). Information theoretic indices for characterization of chemical structures (Vol. 5). Chichester (UK): Research Studies Press.

6. Li, F. *et al.* Quantitative characterization of short- and long-chain perfluorinated acids in solid matrices in Shanghai, China. *Sci Total Environ* 408, 617–23 (2009).

7. Gramatica, P. *et al.* QSAR Modeling is not "Push a Button and Find a Correlation": A Case Study of Toxicity of (Benzo-)triazoles on Algae. *Mol Inform* 31, 817–35 (2012).

8. Put, R. *et al.* Classification and regression tree analysis for molecular descriptor selection and retention prediction in chromatographic quantitative structure–retention relationship studies. *J Chromatogr A* 988, 261–276 (2003).
